# The distributions, mechanisms, and structures of metabolite-binding riboswitches

**DOI:** 10.1186/gb-2007-8-11-r239

**Published:** 2007-11-12

**Authors:** Jeffrey E Barrick, Ronald R Breaker

**Affiliations:** 1 Department of Molecular Biophysics and Biochemistry, Yale University, New Haven, Connecticut 06520-8103, USA; 2 Department of Microbiology and Molecular Genetics, Michigan State University, East Lansing, MI48824-4320, USA; 3 Howard Hughes Medical Institute, Yale University, New Haven, Connecticut 06520-8103, USA; 4 Department of Molecular, Cellular, and Developmental Biology, Yale University, New Haven, Connecticut 06520-8103, USA

## Abstract

**Background:**

Riboswitches are noncoding RNA structures that appropriately regulate genes in response to changing cellular conditions. The expression of many proteins involved in fundamental metabolic processes is controlled by riboswitches that sense relevant small molecule ligands. Metabolite-binding riboswitches that recognize adenosylcobalamin (AdoCbl), thiamin pyrophosphate (TPP), lysine, glycine, flavin mononucleotide (FMN), guanine, adenine, glucosamine-6-phosphate (GlcN6P), 7-aminoethyl 7-deazaguanine (preQ_1_), and *S*-adenosylmethionine (SAM) have been reported.

**Results:**

We have used covariance model searches to identify examples of ten widespread riboswitch classes in the genomes of organisms from all three domains of life. This data set rigorously defines the phylogenetic distributions of these riboswitch classes and reveals how their gene control mechanisms vary across different microbial groups. By examining the expanded aptamer sequence alignments resulting from these searches, we have also re-evaluated and refined their consensus secondary structures. Updated riboswitch structure models highlight additional RNA structure motifs, including an unusual double T-loop arrangement common to AdoCbl and FMN riboswitch aptamers, and incorporate new, sometimes noncanonical, base-base interactions predicted by a mutual information analysis.

**Conclusion:**

Riboswitches are vital components of many genomes. The additional riboswitch variants and updated aptamer structure models reported here will improve future efforts to annotate these widespread regulatory RNAs in genomic sequences and inform ongoing structural biology efforts. There remain significant questions about what physiological and evolutionary forces influence the distributions and mechanisms of riboswitches and about what forms of regulation substitute for riboswitches that appear to be missing in certain lineages.

## Abstract 2

Phylogenetic analyses revealed insights into the distribution of riboswitch classes in different microbial groups, and structural analyses led to updated aptamer structure models and insights into the mechanism of these non-coding RNA structures.

## Background

Riboswitches are autonomous noncoding RNA elements that monitor the cellular environment and control gene expression [1-4]. More than a dozen classes of riboswitches that respond to changes in the concentrations of specific small molecule ligands ranging from amino acids to coenzymes are currently known. These metabolite-binding riboswitches are classified according to the architectures of their conserved aptamer domains, which fold into complex three-dimensional structures to serve as precise receptors for their target molecules. Riboswitches have been identified in the genomes of archaea, fungi, and plants; but most examples have been found in bacteria.

Regulation by riboswitches does not require any macromolecular factors other than an organism's basal gene expression machinery. Metabolite binding to riboswitch aptamers typically causes an allosteric rearrangement in nearby mRNA structures that results in a gene control response. For example, bacterial riboswitches located in the 5' untranslated regions (UTRs) of messenger RNAs can influence the formation of an intrinsic terminator hairpin that prematurely ends transcription or the formation of an RNA structure that blocks ribosome binding. Most riboswitches inhibit the production of unnecessary biosynthetic enzymes or transporters when a compound is already present at sufficient levels. However, some riboswitches activate the expression of salvage or degradation pathways when their target molecules are present in excess. Certain riboswitches also employ more sophisticated mechanisms involving self-cleavage [5], cooperative ligand binding [6], or tandem aptamer arrangements [7].

Many aspects of riboswitch regulation have not yet been critically and quantitatively surveyed. To forward this goal, we have compiled a comparative genomics data set from systematic database searches for representatives of ten metabolite-binding riboswitch classes (Table 1). The results define the overall taxonomic distributions of each riboswitch class and outline trends in the mechanisms of riboswitch-mediated gene control preferred by different bacterial groups. The expanded riboswitch sequence alignments resulting from these searches include newly identified variants that provide valuable information about their conserved aptamer structures. Using this information, we have re-evaluated the consensus secondary structure models of these ten riboswitch classes. The updated structures reveal that certain riboswitch aptamers utilize previously unrecognized examples of common RNA structure motifs as components of their conserved architectures. They also highlight new base-base interactions predicted with a procedure that estimates the statistical significance of mutual information scores between alignment columns.

**Table 1:** Sources of riboswitch sequence alignments and molecular structures

		References		
		
Riboswitch class	Rfam accession	Seed alignment	Other alignments	Molecular structures
Thiamine pyrophosphate (TPP)	RF00059	[41]	[48]	[71-73]
Adenosylcobalamin (AdoCbl)	RF00174	[39]	[20]	
Lysine	RF00168	[37]	[21]	
Glycine	RF00504	[6]		
S-Adenosylmethionine class 1 (SAM-I)	RF00162	[94]	[9,52]	[78]
Flavin mononucleotide (FMN)	RF00050	[56]		
Guanine and adenine (purine)	RF00167	[22]		[95-97]
Glucosamine-6-phosphate (GlcN6P)	RF00234	[23]		[28,30]
7-Aminoethyl 7-deazaguanine (preQ_1_)	RF00522	[40]		
S-Adenosylmethionine class 2 (SAM-II)	RF00521	[18]		

Riboswitches are named for the metabolite that they sense with standard abbreviations in parentheses. Rfam database numbers are provided for each riboswitch along with references to the seed alignments we used to train covariance models for database searches in this study, other published multiple sequence alignments, and three-dimensional molecular structures.

## Results and discussion

### Riboswitch identification overview

Metabolite-binding riboswitch aptamers are typical of complex functional RNAs that must adopt precise three-dimensional shapes to perform their molecular functions. A conserved scaffold of base-paired helices organizes the overall fold of each aptamer. The identities of bases within most helices vary during evolution, but changes usually preserve base pairing to maintain the same architecture. In contrast, the base identities of nucleotides that directly contact the target molecule or stabilize tertiary interactions necessary to assemble a precise binding pocket are highly conserved even in distantly related organisms. Additionally, many riboswitches tolerate long nonconserved insertions at specific sites within their structures. These 'variable insertions' typically adopt stable RNA stem-loops that do not interfere with folding of the aptamer core.

Nearly all of the riboswitches discovered to date are *cis*-regulatory elements. For example, bacterial riboswitches are almost always located upstream of protein-coding genes related to the metabolism of their target molecules. Therefore, the genomic contexts of putative hits returned by an RNA homology search can be used to recognize legitimate riboswitches even when a search algorithm returns many false positives. Using this tactic, one can iteratively refine the description of a riboswitch aptamer by incorporating authentic low scoring hits into a new structure model and then re-searching the sequence database.

Several riboswitches were first identified as widespread RNA elements based on the presence of a highly conserved 'box' sequence within their structures. BLAST searches for the B12 box [8], S box [9], and THI box [10] sequences are effective for discovering many examples of the adenosylcobalamin (AdoCbl), *S*-adenosylmethionine (SAM)-I, and thiamin pyrophosphate (TPP) riboswitches, respectively. Other search techniques score how well a sequence matches a template of conserved bases and base-paired helices that the user manually devises from known examples of the riboswitch aptamer. The RNAmotif program performs this sort of generalized pattern matching [11]. A third strategy computationally defines and then searches for ungapped blocks of sequence conservation that are characteristic of a given riboswitch and spaced throughout its structure [12]. While these methods can be effective, they generally do not fully exploit the information contained in multiple sequence alignments of functional RNA families to efficiently identify highly diverged members.

Covariance models (CMs) are generalized probabilistic descriptions of RNA structures that offer several advantages over other homology search methods [13]. CMs can be directly trained on an input sequence alignment without time-consuming manual intervention. They also provide a more complete model of the sequence and structure conservation observed in functional RNA families that incorporates: first-order sequence consensus information; second-order covariation, where the probability of observing a base in one alignment column depends on the identity of the base in another column; insert states that allow variable-length insertions; and deletion states that allow omission of consensus nucleotides. This complexity comes at a computational cost, but several filtering techniques have recently been developed that make CM searches of large databases practical [14-16]. For example, CMs have been used to find divergent homologs of *Escherichia coli *6S RNA [17] and define a variety of regulatory RNA motifs in α-proteobacteria [18]. The Rfam database [19] maintains hundreds of covariance models for identifying a wide variety of functional RNAs, including riboswitches.

In the present study, we used covariance models to systematically search for ten classes of metabolite-binding riboswitches in microbial genomes, environmental sequences, and selected eukaryotic organisms. The riboswitch sequence alignments used to train these CMs were derived from a variety of published and unpublished sources (Table 1). The genomic contexts of prospective riboswitch hits were examined to confirm that each was appropriately positioned to function as a regulatory element. In general, CMs trained on the input alignments were able to discriminate valid riboswitch sequences from false positive hits on the basis of CM scores alone. The most common exceptions were spuriously high-scoring AU-rich matches to the smaller riboswitch models (for example, the purine riboswitch) and *bona fide *low-scoring hits with variable insertions at unusual positions in the more structurally complex riboswitch classes.

Prospective riboswitch matches were also examined to ensure that they conformed to known aptamer structure constraints. In certain cases, it was necessary to manually correct portions of the automated sequence alignments defined by the maximally scoring path of each hit through the states of the CM. For example, CMs model only hierarchically nested base pairs for algorithmic speed [13]. Consequently, the pseudoknotted helices and pairings present in several riboswitches were aligned by hand to achieve the desired accuracy. The automated CM alignments also tend to incorrectly shift nucleotides when deletions of consensus positions result in ambiguity concerning the optimal placement of remaining sequences. The alignments of new RNA structure motifs and base-base interactions described later that were not present in the seed alignments used to train the covariance models were also manually adjusted. Multiple sequence alignments of the resulting curated riboswitch hits are available as Additional data files 1 and 2.

### Riboswitch distributions

**Figure 1:**
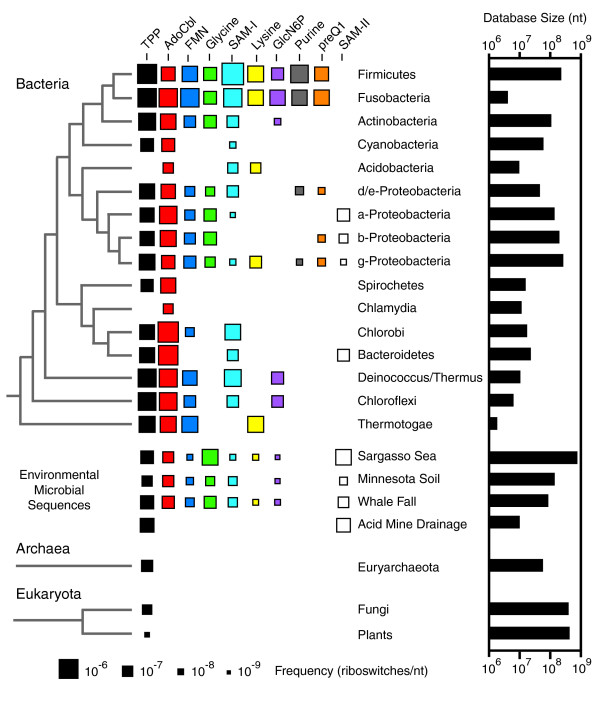
Riboswitch distributions. The dimensions of each square are proportional to the frequency with which a given riboswitch occurs in the corresponding taxonomic group. A phylogenetic tree with the standard accepted branching order for each group of organisms is shown on the left. For bacteria, this tree is adapted from [92] with the addition of Fusobacteria [93]. On the right is a graph depicting the total number of nucleotides from each taxonomic division in the sequence databases that were searched.

The phylogenetic distributions of the ten riboswitch classes were mapped from these search results (Figure 1). Members of the TPP riboswitch class are the only metabolite-binding RNAs known to occur outside of eubacteria. TPP riboswitch representatives are found in euryarchaeal, fungal, and plant species. The AdoCbl riboswitch is the most widespread class in bacteria, but TPP, flavin mononucleotide (FMN), and SAM-I riboswitches are also common in many groups. Glycine and lysine riboswitches have more fragmented distributions. They are widespread in certain bacterial groups, but appear to be missing from others. Finally, the glucosamine-6-phosphate (GlcN6P), purine, 7-aminoethyl 7-deazaguanine (preQ_1_), and SAM-II riboswitches were identified in only a few groups of bacteria. Interestingly, the SAM-I and SAM-II aptamer distributions overlap slightly. Examples of both SAM-sensing riboswitch classes were found in α-Proteobacteria, γ-Proteobacteria, and Bacteroidetes, but no single bacterial species was found to carry both SAM-I and SAM-II riboswitch classes.

It is possible that many of the relatively isolated examples where riboswitches occur only sporadically in certain clades (for example, SAM-I, SAM-II, purine, and preQ_1 _in γ-Proteobacteria) may be examples of horizontal DNA transfer. There is some evidence that this process has been important for the dispersal of riboswitches into new bacterial genomes. Entire transcriptional units containing AdoCbl riboswitches and their associated biosynthetic operons appear to have been transferred from *Bacillus*/*Clostridium *species to enterobacteria at some point [20]. In contrast, no evidence of recent horizontal transfer was observed in phylogenetic trees of lysine riboswitch aptamers, despite their disjointed distribution across different taxonomic groups [21].

Firmicutes (low G+C Gram-positive bacteria) appear to make the most extensive use of the riboswitch classes examined in this study. Every riboswitch except SAM-II is widespread in this clade, and most aptamer classes occur multiple times per genome. For example, *Bacillus subtilis *carries at least 29 riboswitches (5 TPP, 1 AdoCbl, 2 FMN, 1 glycine, 11 SAM-I, 2 lysine, 1 GlcN6P, 4 guanine, 1 adenine, and 1 preQ_1_) controlling approximately 73 genes. Experimental and computational efforts to identify riboswitches have been focused specifically on *B. subtilis *[22,23], so it is possible that the overrepresentation of these ten riboswitch classes in Firmicutes reflects a discovery bias. Indeed, new computational searches are beginning to identify riboswitch classes that are predominantly used by other groups of bacteria [18,24].

As a whole, γ-Proteobacteria employ a mixture of these ten riboswitch classes that is comparable to the diversity found in Firmicute species. However, individual species usually carry fewer riboswitch classes overall and fewer representatives of each class. For example, *E. coli *has six riboswitches (three TPP, one AdoCbl, one FMN, and one lysine) from the ten classes examined, which regulate a total of sixteen genes.

Deeply branched bacteria such as *Deinococcus*/*Thermus *and *Thermotoga *species also appear to utilize a variety of riboswitches. However, no riboswitch sequences have yet been identified in *Aquifex *species, and riboswitches also seem to occur only rarely in *Chlamydia *species, Cyanobacteria, and Spirochetes. However, the sequence database sizes for many of these bacterial groups are relatively small so the observed frequencies will probably need to be revised as more genomic sequences become available.

As expected, representatives of almost all ten riboswitch classes are found in sequences from shotgun cloning projects that target environments supporting diverse bacterial communities. These sources of additional sequences have been helpful in some cases for defining consensus structure models and adding statistical merit to mutual information calculations (see below). It is notable that glycine and SAM-II riboswitches are unusually common in Sargasso Sea metagenomic sequences [25]. This data set appears to be contaminated with some non-native *Shewanella *and *Burkholderia *sequences [26], but the large number of SAM-II matches probably accurately reflects the abundance of α-Proteobacteria in this environment.

### Riboswitch mechanism overview

GlcN6P riboswitches are ribozymes that harness a self-cleavage event to repress expression of downstream *glmS *genes [5]. Members of this class are unique compared to other riboswitches because they adopt a preformed binding pocket for glucosamine-6-phosphate [27,28] and use the metabolite target as a cofactor to accelerate RNA cleavage [28-30]. The nine other riboswitch classes studied here utilize ligand-induced changes in 'expression platform' sequences to control a variety of gene expression processes [1]. The architectures of riboswitch expression platforms can be used to predict their gene control mechanisms on a genomic scale, as described below.

Riboswitches typically contain disordered regions in their conserved aptamer cores that become structured upon metabolite binding. These changes may trigger rearrangements in additional expression platform structures located outside of the aptamer, such that two alternative conformations with mutually exclusive base-paired architectures exist for the entire riboswitch. Some riboswitches operate at thermodynamic equilibrium [31]. They are able to interconvert between these ligand-bound and ligand-free structures in the context of the full-length RNA. Regulation by other riboswitches is kinetically controlled [32-35]. The relative speeds of transcription and co-transcriptional ligand binding dominate a one-time decision as to which folding pathway to follow. The active and inactive conformations of these riboswitches are trapped in the final RNA molecule and do not readily interconvert on a time scale that is relevant to the gene control system.

In most riboswitches, bases from the aptamer's outermost P1 'switching' helix, which is enforced in the ligand-bound conformation, pair to expression platform sequences to form an alternative structure in the absence of ligand, for example, [36,37]. However, some riboswitches harness shape changes elsewhere in their aptamers to regulate gene expression. AdoCbl riboswitches usually rely on the ligand-dependent formation of a pseudoknot between a specific C-rich loop and sequences outside the aptamer core to exert gene control [20,38,39]. SAM-II aptamers enforce a distal pseudoknot to interface with their expression platforms [18], and preQ_1 _riboswitches sequester conserved 3' tail sequences upon metabolite binding [40].

Riboswitches can use ligand-induced structure changes to control gene expression in a variety of contexts. For example, the TPP riboswitches found in eukaryotes reside in introns located near the 5' ends of fungal pre-mRNAs [41-43] or in the 3' UTRs of plant pre-mRNAs [41]. Ligand binding modulates splicing of these introns, generating alternative-processed mRNAs that are expressed at different levels. In each example studied, a portion of the P4-P5 stem region pairs near a 5' splice-site, and this pairing is displaced when TPP is bound [43] (A Wachter, M Tunc-Ozdemir, BC Grove, PJ Green, DK Shintani, RRB, unpublished data). In contrast, almost all bacterial riboswitches occur in the 5' UTRs of mRNAs. Metabolite binding to these riboswitches generally regulates either transcription or translation of the encoded genes.

Bacterial riboswitches that regulate transcription usually control the formation of intrinsic terminator stems located within the same 5' UTR. Intrinsic terminators are stable GC-rich stem-loops followed by polyuridine tracts that cause RNA polymerase to stall and release the nascent RNA with some probability [44,45]. Certain glycine [6] adenine [46], and lysine [21] riboswitches with ON genetic logic use structural rearrangements triggered by metabolite binding to bury pieces of terminator stems in alternative pairing interactions. However, most riboswitches controlling transcription are OFF switches that add an extra folding element to reverse this logic. Metabolite binding to these riboswitches disrupts an antiterminator, which normally sequesters bases required to form the terminator stem, allowing the terminator to form and repress gene expression. Similar antiterminator/terminator trade-offs occur in bacterial RNAs regulated by protein- or ribosome-mediated transcription attenuation mechanisms [47].

Bacterial riboswitches that regulate translation typically use ligand-induced structure changes to block translation initiation. Unlike riboswitches with transcription control mechanisms, which require very specific terminator structures in their expression platforms, the RNA structures that prevent translation initiation may be more varied. Sometimes, they rely on simple hairpins that sequester the ribosome binding site (RBS) of the downstream gene in a base-paired helix. In these cases, a riboswitch with OFF genetic logic can harness metabolite binding to disrupt a mutually exclusive antisequestor pairing, allowing the sequestor hairpin to form and attenuate translation. More convoluted base-pairing trade-offs and shape changes may operate in other expression platforms to alter the efficiency of translation initiation in response to ligand binding.

Two variants of these mechanisms that dispense with or combine the elements of a typical bacterial riboswitch expression platform are worth noting. Some riboswitches bury the RBS of the downstream gene within their conserved aptamer cores [48,49]. Thus, ligand binding directly attenuates translation without the involvement of any additional expression platform sequences. Other riboswitches regulate the formation of a transcription terminator located so close to the adjacent open reading frame that its RBS resides within the 3' side of the terminator hairpin [48]. Riboswitches with these dual expression platforms could attenuate transcription and, if termination does not occur, could also inhibit translation.

Metabolite-dependent inhibition of ribosome binding has been proven *in vitro *for the *E. coli *AdoCbl riboswitch located upstream of the *btuB *gene [50]. In addition, *in vivo *expression assays using translational fusions between AdoCbl riboswitches and reporter genes indicate that control of translation is occurring [38]. However, other co- or post-transcription mechanisms might also contribute to the observed gene expression changes. For example, AdoCbl riboswitches from *E. coli *and *B. subtilis *can be cleaved by RNase P [51]. Such findings raise the interesting possibility that differential RNA processing or degradation caused by ligand-induced conformational changes might be the primary mechanism by which some riboswitches regulate gene expression.

There is one interesting instance where a *Clostridium acetobutylicum *SAM-I riboswitch appears to regulate protein expression through an antisense RNA intermediate [52]. This riboswitch is located immediately downstream, and in the opposite orientation from, an operon encoding a putative salvage pathway for converting methionine to cysteine. It has an expression platform, consisting of a typical terminator/antiterminator arrangement, with OFF genetic logic. Presumably, when SAM (and consequently methionine) pools are low, transcription of the full-length antisense RNA causes inhibition and degradation of the sense mRNA as is observed in some bacterial regulatory systems that employ small RNAs [53]. When SAM levels are high, the SAM-I riboswitch will prematurely terminate the antisense transcript, allowing expression of this operon to recycle excess methionine.

In some instances, riboswitches or their components are found in tandem arrangements. Almost all glycine riboswitches consist of two aptamers that regulate a single downstream expression platform [6]. In the genomic sequences searched here, 88% of the mRNA leaders containing one glycine aptamer also carry a second aptamer. Cooperative binding of two ligand molecules by these glycine riboswitches yields a genetic switch that is more 'digital', that is, more responsive to smaller changes in ligand concentration, than a single aptamer.

Far less common are tandem arrangements of other riboswitch classes such as TPP [7,54,55] or AdoCbl [55]. Fewer than 1% of the UTRs regulated by these riboswitch classes contain multiple aptamers. In these cases, each aptamer appears to function as an independent riboswitch that regulates its own expression platform to yield a more digital, compound genetic switch [7]. Also rare are tandem arrangements wherein representatives of two different riboswitches are in the same UTR. In the *metE *mRNA leader from *Bacillus clausii*, a SAM-I and an AdoCbl riboswitch independently control transcription termination to combinatorially regulate expression of this gene in response to two different metabolite inputs [55].

### Riboswitch mechanisms

**Figure 2:**
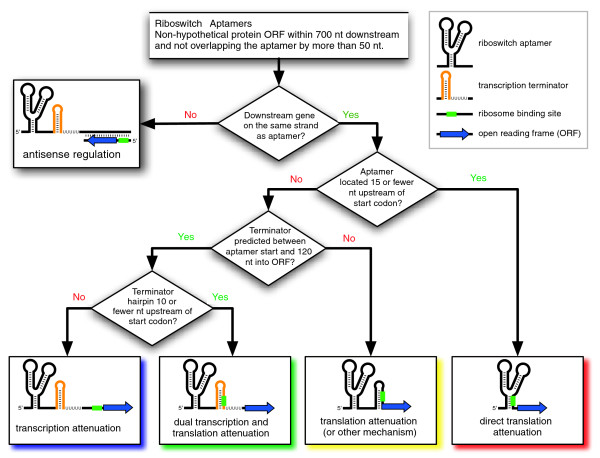
Riboswitch mechanism prediction scheme. The decision tree used to classify riboswitch mechanisms into five categories is shown. Depicted are OFF switches in their ligand-bound state where a P1 switching helix has formed. See the main text and Materials and methods for additional details.

A decision tree was established for computationally classifying the gene control mechanisms of microbial riboswitches (Figure 2). The five categories assigned are: transcription attenuation; dual transcription and translation attenuation; translation attenuation; direct translation attenuation; and antisense regulation. The same mechanisms have been predicted for TPP [48], AdoCbl [20], FMN [56], and lysine [21] riboswitches in previous comparative studies. The use of the term attenuation here does not imply that a switch operates with OFF genetic logic, that is, gene expression may be attenuated in the ligand-free state and relieved by metabolite binding. Overall, computational assignments by this procedure have an accuracy of 88% when compared to expert predictions of TPP riboswitch mechanisms [48].

It is important to note that the decision tree does not explicitly predict RBS-hiding structures in expression platforms. Rather, it assumes that control of translation initiation is the most likely mechanism for riboswitches not classified into the other categories. It is possible that these riboswitches could operate by mechanisms other than the five assigned by this procedure (as described above). Another caveat is that this prediction scheme considers only intrinsic terminator structures consisting of RNA stem-loops followed by polyuridine tails. These are currently the only structures that riboswitches with transcription attenuation mechanisms are known to regulate. However, some bacteria appear to be able to utilize other structures that may lack a canonical U-tail or consist of tandem hairpins to terminate transcription [57].

**Figure 3:**
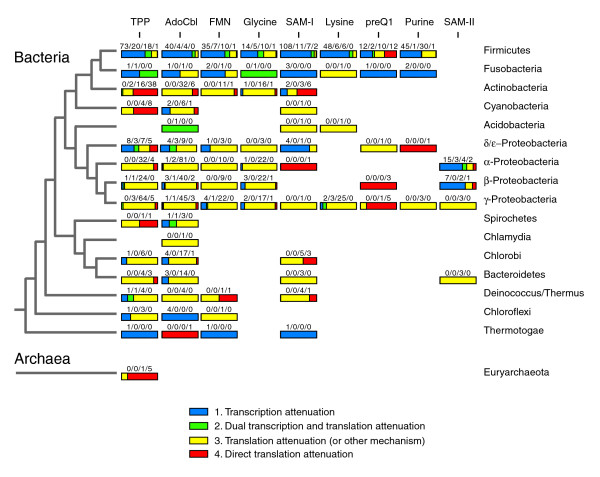
Riboswitch mechanisms. The mechanisms that riboswitches from different taxonomic groups use to regulate gene expression were classified on the basis of expression platform features (Figure 2). The fractions of riboswitch expression platforms in each category are displayed visually as shaded bars with the actual numbers observed written above in the order given in the legend. The phylogenetic tree on the left is described in the legend to Figure 1.

Mapping riboswitch mechanism predictions onto a phylogenetic tree (Figure 3) reveals that transcription attenuation dominates in Firmicutes and that translation attenuation is most common in other bacterial groups. The phylogenetic distribution of SAM-II riboswitch mechanisms is an exception. It is the only riboswitch aptamer that appears to be most often associated with regulatory transcription terminators in α- and β-Proteobacteria, although the mechanisms by which SAM-II aptamers control gene expression have not yet been experimentally established [18]. Transcription attenuation mechanisms may also be generally overrepresented in Fusobacteria, δ/ε-Proteobacteria, Thermatogae, and Chloroflexi species, although smaller sample sizes make these conclusions less certain.

Mechanisms that rely on sequestering the RBS within the conserved aptamer core are most common for the TPP, preQ_1_, and SAM-I riboswitches. In the first two cases, purine-rich conserved regions near the 3' ends of the riboswitch substitute for RBS sequences. In SAM-I riboswitches, the RBS is incorporated into the 3' side of the P1 stem. Other riboswitch classes also have purine-rich conserved regions near their 3' ends with consensus sequences close to ribosome binding sites. It is not clear why direct regulation of translation attenuation is not more common in these other classes. Perhaps access to the RBS-like sequences in these aptamers is not modulated by ligand binding. Riboswitch regulation by direct translation attenuation appears to be most frequent in Actinobacteria and Cyanobacteria, except for the preQ_1 _riboswitch where this mechanism is unusually prevalent, even in Firmicutes and Proteobacteria.

There do not appear to be any additional examples of riboswitches positioned for antisense regulation in this data set. An antisense arrangement may be rare because it inverts the gene control logic of the riboswitch and requires the evolutionary maintenance of a second promoter. A handful of high-scoring hits were found that appear to be functional aptamers even though they are not located upstream of genes related to the cognate metabolite. It is possible that these riboswitches affect their target genes by regulating the production or function of *trans*-acting antisense RNAs or that they have been recently orphaned by genomic rearrangements and are now pseudo-regulatory sequences.

### Evaluating structure models

Constructing an RNA secondary structure model using phylogenetic sequence data requires identifying possible base-paired stems and adjusting a sequence alignment to determine whether each proposed stem appears reasonable for all representatives. This recursive refinement process has been used to create detailed comparative models of many functional RNA structures that accurately reflect later genetic, biochemical and biophysical data. However, the presence of stretches of unvarying nucleotides within an RNA structure, the tolerance of stems to some non-canonical base pairs or mismatches, and the non-negligible frequency of sequencing errors in biological databases can introduce enough uncertainty that multiple structures may seem to agree with a sequence alignment and incorrect base-paired elements may be proposed. This problem is compounded if the multiple sequence alignment is incomplete and does not yet capture all of the variation that truly exists at each nucleotide position.

Inconsistencies and ambiguities in some riboswitch aptamer models motivated us to evaluate the statistical support for base pairs in their proposed structures. We chose to use mutual information (MI) scores [58] to mathematically formalize the interdependence between sequence alignment columns that is indicative of base interactions. MI is a normalized version of covariance that represents the amount of information (in bits) gained about what base occurs at a given position from knowing the identity of a base at another position. The prediction of RNA secondary structures and tertiary interactions from covariation in sequence alignments has a long history, and the nuances of calculating and interpreting MI scores have been comprehensively covered elsewhere [59,60].

Fundamentally, columns of interacting bases must be correctly aligned and there must be variation within each column (that is, it cannot be completely conserved) in order to detect mutual information. Even when these preconditions are met, there are two difficulties with directly comparing MI scores to determine which columns in a sequence alignment truly covary. First, sequence conservation derived from the shared evolutionary histories of sequence subsets in an alignment may result in a high residual background MI score between many columns whether or not they are functionally linked. Second, alignments with fewer sequences will have more column pairs with elevated MI scores simply by chance. Simulations addressing the expected magnitudes of these two sources of error in different data sets have been explored recently in the context of protein sequence alignments [61].

**Figure 4:**
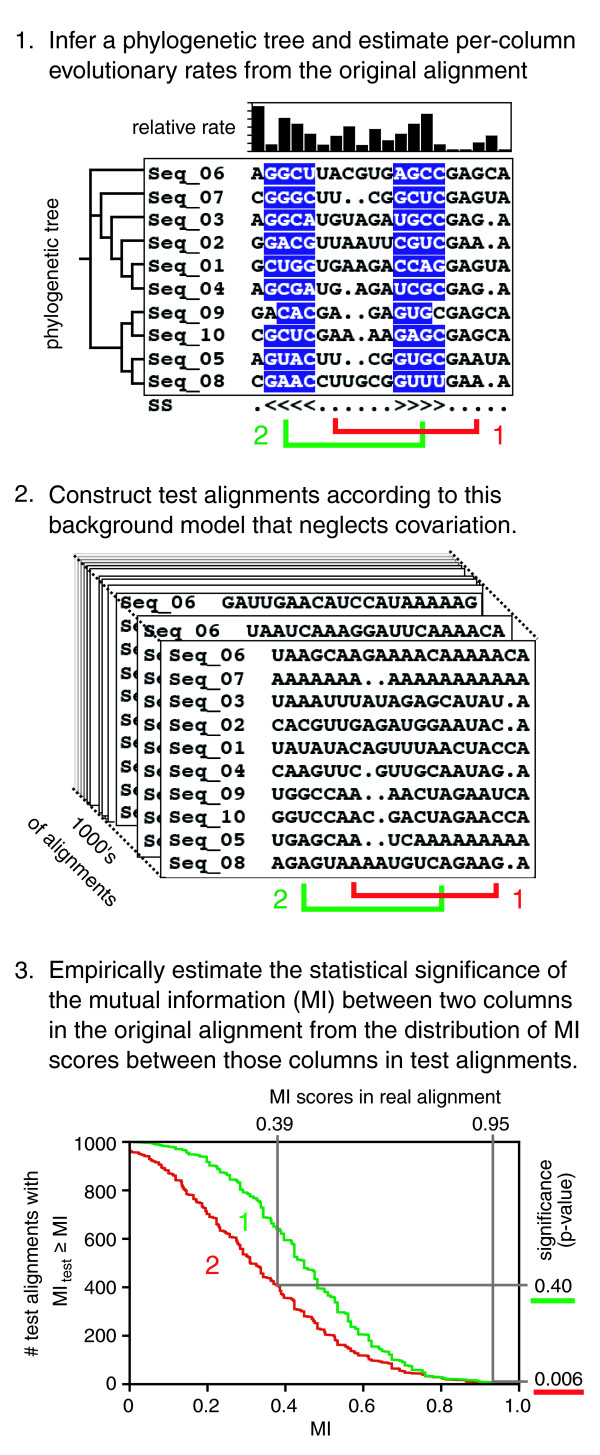
Procedure for estimating MI significance between alignment columns. See the main text and Materials and methods for a complete description of the procedure used to estimate the statistical significance of MI scores between columns in a multiple sequence alignment in order to evaluate riboswitch secondary structures and predict new base-base interactions.

In order to better gauge whether MI scores support proposed base interactions in an RNA alignment, we developed a procedure for empirically estimating their statistical significance (Figure 4). First, a phylogenetic tree is inferred from the observed RNA sequence alignment according to a model that assumes independent evolution at each position and allows for varying per-column mutation rates. Then, resampled alignments with the same topology, branch lengths, and evolutionary rates are generated. MI scores between columns in these test alignments reflect the null hypothesis that there is no covariation between positions. They implicitly correct for the evolutionary history and sample size of the real sequence alignment. Therefore, the *p *value significance for an observed MI score in the real alignment is the fraction of *test *alignments with higher MI scores between these two columns.

### Riboswitch structures

**Figure 5:**
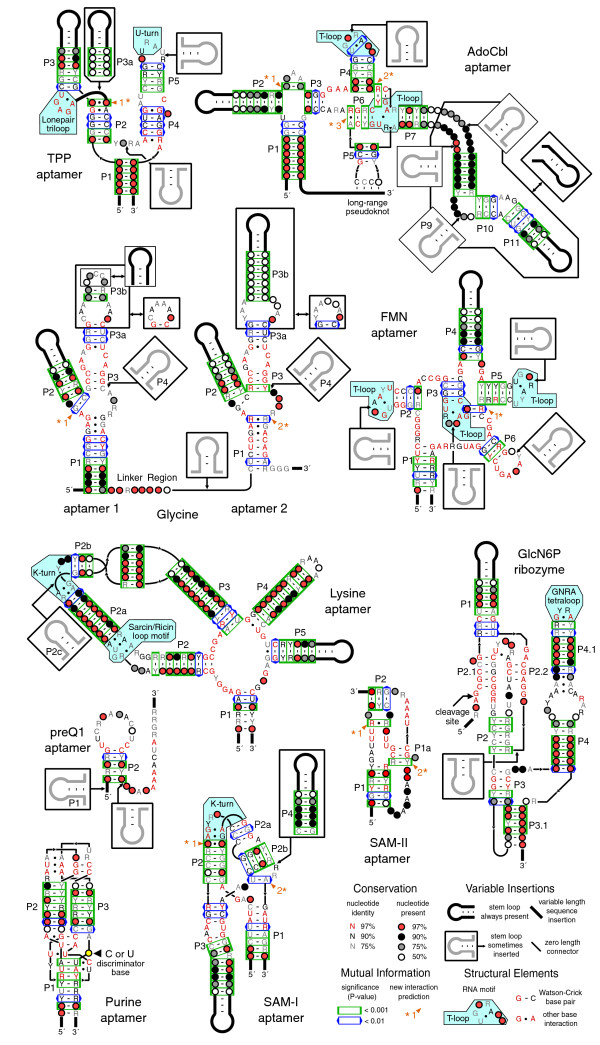
Riboswitch aptamer structures. The consensus secondary structure models based on expanded riboswitch sequence alignments are depicted according to the symbols defined in the inset. Each structure is further annotated with RNA structure motifs and the statistical significances (*p *values) of the mutual information scores between base-paired alignment columns. New predictions of interacting bases from the MI analysis are numbered and indicated by asterisks. More detailed descriptions of these predictions are provided in Figure 7.

The consensus secondary structure models of the ten riboswitch classes (Figure 5) have been updated to reflect information from newly identified aptamer variants. The purine, TPP, SAM-I, and GlcN6P riboswitch consensus structures have been drawn in accordance with their molecular structures (references in Table 1). Other riboswitch structures have been revised to be consistent with the new predictions of structure motifs and base-base interactions explained below. In all cases, previous numbering schemes for the paired helical elements (designated P1, P2, P3, and so on, beginning at the 5' end of each the aptamer) have been maintained, even when these stems do not occur in a majority of the sequences in the updated alignment. Newly discovered paired elements that do not appear in most examples of a riboswitch aptamer have not been assigned numbers.

The results of the mutual information analysis are shown superimposed on the consensus riboswitch structures. Most base-paired helices are supported by at least one contiguous base pair with a highly significant MI (*p *< 0.001), and almost all contain a base pair with at least a marginal MI significance (*p *< 0.01). No significant MI scores are present within the P2.1 and P2.2 stems observed in the crystal structures of the GlcN6P-dependent ribozyme [28,30]. However, most of the predicted base pairs in the P2.1 and P2.2 helices are between highly conserved bases that may not vary enough to produce significant covariation with their pairing partners. The MI analysis also does not support an alternative P1.1 pseudoknot (not shown) proposed on the basis of biochemical experiments where the register of the regions involved in making the P2.1 pairing is slightly shifted [29,62,63].

**Figure 6:**
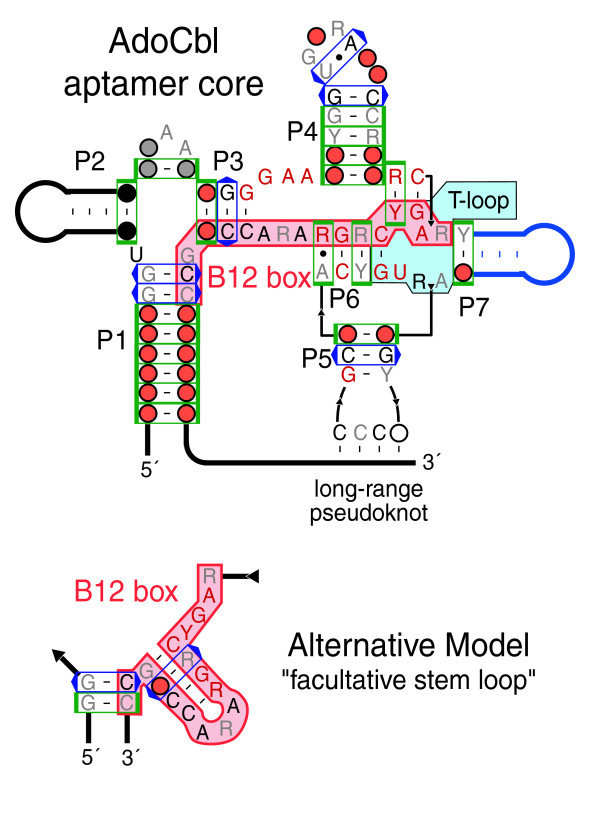
Comparison of B12 box structure models. In addition to the model of the AdoCbl riboswitch aptamer structure presented here [39], an alternative model that folds the highly conserved B12 box sequence (highlighted in red) into a 'facultative stem-loop' has been proposed [20]. The core of the AdoCbl riboswitch aptamer is shown with abbreviated peripheral helices and without the optional P8-P10-P11 domain for comparison with the alternative secondary structure model. The upper model is supported by multiple base pairs with significant MI scores between B12 box bases and remote positions. In it, a portion of the B12 box also forms part of an internal T-loop motif between P6 and P7. Each diagram uses the symbols described in the legend to Figure 5.

MI significance scores do resolve a conflict between two pairing models that have been proposed for the highly conserved B12 box of the AdoCbl riboswitch (Figure 6). One model posits that a 'facultative stem loop' forms by pairing nucleotides within the B12 box [20]. The other model proposes long-range pairings between portions of the B12 box and nucleotides more distant in RNA sequence [39]. There is only a single, marginally significant MI score that supports the formation of the 'facultative stem loop', even though this region was correctly aligned to optimally discover such interactions. The MI analysis strongly supports several base pairs in the alternative proposed structure wherein portions of the conserved B12 box form the 3' sides of the short P3 and P6 helical stems.

### RNA structure motifs

Several riboswitches contain common RNA structure motifs that are recognizable from their consensus features. A GNRA tetraloop [64] that favors a pyrimidine at its second position caps P4a of most GlcN6P ribozymes. A K-turn [65,66] between P2 and P2a is conserved in SAM-I riboswitch aptamers [66]. The asymmetric bulge between helices P2a and P2b in the lysine riboswitch also fits a K-turn consensus in most sequences [67], but a number of variants appear to lack this motif. A sarcin-ricin motif [68] (a specific type of loop E motif) in the asymmetric bulge between the P2 and P2a helices of the lysine riboswitch is more highly conserved [37,67].

We also find examples of other RNA structure motifs that have not previously been reported in these riboswitch classes. The consensus features of the three terminal loops capping P2, P3, and P5 in the FMN riboswitch and the P4 loop and P6-P7 bulge in the AdoCbl riboswitch are remarkably similar. Each has two closing G-C base pairs with a strand bias, a possible U-A pair separated from the helical stem by two bulged nucleotides on the 3' side, and a terminal GNR triloop sequence that is sometimes interrupted at a specific position by an intervening base-paired helix. These characteristics strongly suggest that they adopt T-loop structures (named for the T-loop of tRNA) where the U-A forms a key *trans *Watson-Crick/Hoogsteen pair [69].

Sequence conservation in the UNR loop that closes the P5 stem in the TPP aptamer suggests that it forms a conserved U-turn [70]. As expected, there is a sharp reversal of backbone direction following this uridine, subsequent bases stack on the 3' side of the loop, and the uracil base can hydrogen bond with the phosphate group 3' of the third U-turn nucleotide in the X-ray crystal structures of *E. coli *[71,72] and *Arabidopsis thaliana *[73] riboswitches. Also, in the TPP aptamer, the conserved UGAGA sequence 3' of the P3 helix fits the UGNRA consensus for a type R1 lonepair triloop [74]. The crystal structures confirm that this motif is present with the characteristic *trans *Watson-Crick/Hoogsteen U-A closing pair around the triloop. Commonly, a tertiary interaction between the triloop G base and an outside A leads to a composite GNRA tetraloop structure. However, in this case, the pyrimidine ring from the TPP ligand intercalates into the triloop at an equivalent position.

### New base-base interaction predictions

**Figure 7:**
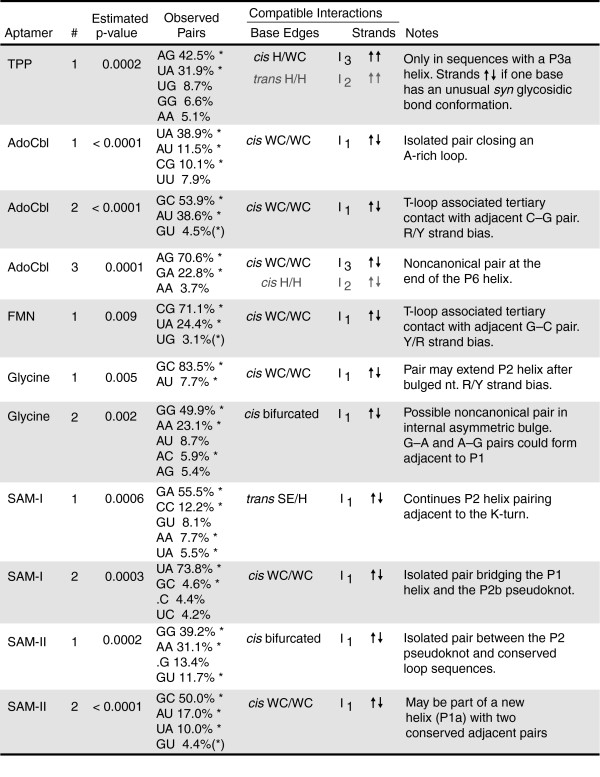
New base-base interaction predictions. For each numbered and asterisked prediction in Figure 5 the statistical significance (*p *value) of the mutual information between the two alignment columns is shown, followed by the relative frequencies with which specific combinations of bases are observed in those columns. Base pair geometries and isostericity groups compatible with the asterisked pairs are described in more detail elsewhere [75]. These descriptions include the relative orientations of the glycosidic bonds across the pair (*cis *or *trans*), the edges of each base that interact (WC, Watson-Crick; H, Hoogsteen; SE, sugar edge; bifurcated, intermediate between two edges), and the relative backbone strand geometry (parallel or anti-parallel) assuming both glycosidic bonds are in default *anti *conformations.

In addition to supporting almost all of the helical elements in the riboswitch structure models, the MI analysis predicts eleven additional base-pairing interactions (Figures 5 and 7). Significant MI scores between two alignment columns should be interpreted with caution. They represent a statistical correlation and do not necessarily imply hydrogen bonding between nucleobases. Correlations between adjacent nucleotides that probably represent favored base stacking patterns in helices and column pairs with many gaps where MI scores can be dominated by the presence and absence of nucleotides rather than their base identities have been ignored. It is also possible to observe high mutual information between two bases that do not interact if several separate structure motifs with their own specific sequence requirements can substitute for each other in a functional RNA, as is seen for GNRA, UNCG, and CUUG tetraloops in 16S rRNA [59].

Furthermore, the estimates of MI significance rely on a phylogenetic tree reconstruction method that may not adequately capture the evolution of these RNA sequences, especially for the shorter riboswitch alignments. Even assuming that the estimated *p *values are completely accurate, there are 4,950 possible combinations of columns in an alignment with 100 columns, and that would imply that, on average, 5 pairs with a MI significance of ≤0.01 will be observed by chance. Some columns that are known to be base paired do not have MI scores this significant. In light of this noisy background, we manually screened MI predictions and concentrated on interacting columns that seem to have structural relevance.

The identities of interacting bases in a functional RNA are constrained during evolution. They can mutate only to other base pairs that preserve the local geometry of the sugar-phosphate backbone and any hydrogen bonds that are important for maintaining structure and function. Generally, only one of the three planar edges of a nucleobase participates in any given interaction: the Watson-Crick face (WC), Hoogsteen face (H), or sugar edge (SE). A systematic study of RNA structures has produced isostericity matrices [75] that tabulate which of the possible 16 base pairs should be interchangeable (in terms of C1'-C1' distances) when two nucleobases are interacting between different combinations of these three base edges and when the glycosidic bonds on both sides of the pair are *cis *or *trans *with respect to each other. The pairs of bases conserved at some of the new correlated positions in riboswitches suggest unusual non-Watson-Crick interactions, and this isostericity framework can be used to tentatively assign possible geometries to the newly predicted base pairs (Figure 7).

In the TPP riboswitch, there is significant MI between the two bases directly 5' of P3 and 3' of P3a that could bridge this helical junction. This correlation was highly significant (*p *= 0.0002) in an alignment of all TPP riboswitch sequences. However, re-examination of the alignment showed that the predominant A-G and U-A pairs mainly occurred in the 552 sequences that have the optional P3a stem-loop. In fact, there is no correlation between these columns in the remaining 355 sequences that lack P3a. Exchange of U-A and A-G pairs is most consistent with a *cis *H/WC edge interaction between these two bases. These pairs are also isosteric in a *trans *H/H geometry, but this configuration involves only a single hydrogen bond, and there are four other isosteric nucleobase combinations that are not observed. Both pair geometries imply that either the sugar-phosphate backbones of the interacting bases are in a parallel orientation or that they are anti-parallel, with one of the bases adopting a rare *syn *glycosidic bond rotation. It may be necessary for these bases to assume an unusual geometry to accommodate the P3a helix at this location.

The molecular resolution structures of TPP riboswitches do not impinge on this prediction, as each of these constructs lacks P3a [71-73]. On the basis of the consensus structure, it is possible to further predict that when the P3a helix is present it will coaxially stack on the P2 helix as part of a type C three-way helical junction [76] wherein P3a, P2, and P3 are assigned P1, P2, and P3 roles, respectively. The molecular structures show a diagnostic feature of this configuration even in the absence of P3a: the J13 motif sequence (corresponding to the conserved UGAGA) forms a pseudohairpin that makes adenine base contacts to the minor groove of the motif's P1 helix (P2 of the riboswitch). Furthermore, there is space in the crystal structure to accommodate P3a cohelically stacking on P2, and this would place P3a parallel to and offset from P3, as is expected for this common three-way junction geometry.

Three new base interactions are predicted in AdoCbl riboswitch aptamers. A lone WC base pair (*p *< 0.0001) seems to enclose the conserved A-rich sequence between the P2 and P3 helices. A highly significant MI score (*p *< 0.0001) also supports a WC pair with purine/pyrimidine strand bias between the nucleotide directly 3' of the P4 helix and a position within the two nucleotide 3' bulge of the P6-P7 T-loop motif. The adjacent nucleotides in this strand and the T-loop bulge could form a highly conserved, cohelical C-G base pair. Similar long-range Watson-Crick base-pairing interactions to these two bulged nucleotides are common with 'type-II' T-loops [69]. The final new prediction in the AdoCbl riboswitch is a non-canonical G-A or A-G pair (*p *= 0.0001) that probably assumes a *cis *WC/WC geometry to continue base stacking with the P6 helix. These pairs are also isosteric in a *cis *H/H geometry, but this geometry seems less likely to be conserved because it involves only a single hydrogen bond.

The FMN riboswitch may contain a strikingly similar T-loop interaction. The nucleotide directly 3' of its P5 helix can form a Watson-Crick pair (*p *= 0.009) with a pyrimidine/purine strand bias to the 3' bulge of the T-loop motif that caps P3. An adjacent G-C base pair is also possible here between highly conserved nucleotides in the strand and T-loop bulge. In both the AdoCbl and FMN riboswitches, the stem-loops adjacent to this predicted interaction have exactly five paired nucleotides and are capped by a second T-loop motif. Although the second T-loop does not seem to be directly relevant to this predicted pairing interaction, the double T-loop substructure that these riboswitches have in common suggests that significant similarity exists between their overall tertiary folds even though they recognize very different ligand molecules.

The MI analysis suggests two new base-base interactions in the glycine riboswitch. The first is a WC pair (*p *= 0.005) with purine/pyrimidine strand bias at the base of the P2 stem of the first aptamer. If this pair cohelically stacks with the P2 stem, then it would often require a bulged nucleotide on the 5' side of the composite helix. The second interaction is a predicted G-G or A-A homopurine pair (*p *= 0.002) that might adopt a *cis *bifurcated geometry within the central bulge of the second aptamer. Bifurcated pairs hydrogen bond between an exocyclic functional group on one base and the edge of the other base, and they are consequently intermediate between two edge geometries (possibly *cis *WC/WC and *trans *WC/H in this case). If this pair forms, it suggests that the two bases on each strand between it and the P1 stem may form G-A and A-G pairs. Both of these putative interactions are maintained in the opposite aptamer of the glycine riboswitch. However, the nucleotides at the corresponding positions are less variable, which may explain why they were not detected a second time by the MI analysis.

Two new base-pairing contacts are predicted for SAM-I riboswitches. The first occurs at the end of the P2 helix adjacent to the conserved G-A and A-G pairs of the K-turn motif. This pair has a highly significant MI score (*p *= 0.0006) and mainly varies from G-A to C-C, which is most compatible with a *trans *SE/H base interaction within this cohelical stacking context. Noncanonical pairs with this configuration are known to occur frequently adjacent to K-turns in other functional RNA structures [77]. The second predicted interaction (*p *= 0.0003) is an unexpected long-range *cis *WC/WC base pair between the base directly upstream of the 5' side of the P2b pseudoknot and the base directly upstream of the P1 3' strand. After originally discovering these new interactions from sequence analysis, we were able to verify that both interactions occur with the predicted configurations in the X-ray crystal structure of a minimized version of the *Thermoanaerobacter tengcongensis metF *SAM-I riboswitch [78].

The MI analysis predicts two new base-base interactions in the SAM-II riboswitch. A homopurine G-G or A-A pair (*p *= 0.0002) could form between two positions in the bulge between P1 and the 5' strand of the P2 pseudoknot. This pair may adopt a *cis *bifurcated geometry. A Watson-Crick base pair (*p *< 0.0001) may also exist between the last nucleotide in the central loop that is contained within the P1 stem and a downstream position. This pair could be extended into a short helical element (P1a) if the adjacent, conserved C-G and G-C base pairs also form canonical WC pairs and an intervening base is bulged out.

## Conclusion

The ten metabolite-sensing riboswitch classes surveyed here are widespread and versatile gene control elements. The conserved secondary structure models of these riboswitch aptamers have been revised to include information from additional sequence variants. These models incorporate newly recognized RNA structure motifs, including a double T-loop substructure that is conserved in AdoCbl and FMN aptamers, and specify new sites where the insertion of unconserved RNA domains is possible. Furthermore, an analysis of mutual information scores using an evolutionarily informed background model has enabled the prediction of new base-base interactions in several riboswitch aptamers. These refinements should improve the accuracy of future computational searches for riboswitches as the automated annotation of functional RNAs in genomic sequences becomes more routine [19]. They will also inform and validate ongoing efforts to determine the molecular resolution structures of riboswitch aptamers.

It is believed that some metabolite-binding riboswitch classes may be descended from the RNA World [79] and that others may be more recent evolutionary innovations [80], but the exact provenance of each riboswitch class is unclear. Significant uncertainty also remains about what physiological and evolutionary forces affect riboswitch use by modern organisms. Particularly, there are unexplained differences in the distributions and preferred regulatory mechanisms of riboswitches across contemporary bacteria. Riboswitches found in Firmicutes (low G+C Gram-positive bacteria) predominantly regulate transcription attenuation, whereas translation attenuation mechanisms are most prevalent in other groups. Overall, riboswitches also appear to be more common in Firmicutes than other bacterial groups.

One of the more interesting aspects of the riboswitch phylogenetic profile is that it outlines gaps and holes in the known distributions of riboswitch classes. Some of these apparently vacant regulatory niches may be occupied by regulatory proteins that fulfill the same role or by extreme structural variants of these riboswitch classes that are not detectable with current RNA homology search techniques. Other gaps could harbor new aptamer classes that recognize the same metabolite as a known riboswitch class. The discovery of SAM-II riboswitches in α-Proteobacteria [18], which are almost devoid of SAM-I riboswitches, sets a precedent for this latter scenario. The existence of a third SAM riboswitch in some lactic acid bacteria species [81], a subdivision of the Firmicutes, suggests that new riboswitch classes may occupy empty regulatory niches that exist at an even finer taxonomic resolution.

## Materials and methods

### Computational analysis

In-house Perl scripts were used to organize the execution of other software tools, compute various statistics, and maintain local relational databases of genome and gene information. Many of these scripts rely on Bioperl [82], and the Bio::Graphics module was particularly useful for visualizing the genomic contexts of riboswitch matches.

### Riboswitch identification

Covariance models were trained on sequence alignments adapted from various sources (Table 1) using the Infernal software package (version 0.55) [83]. Heuristic filtering techniques [16] were used to accelerate CM searches of microbial sequences in the RefSeq database (version 12) [84] and environmental shotgun sequences from an acid mine drainage community [85], the Sargasso Sea [25], and Minnesota soil and whale fall sites [86]. CM searches for TPP riboswitches were also conducted against the plant and fungal portions of the RefSeq database (version 13).

The regulatory potentials of putative riboswitch aptamers were assessed by examining their genomic contexts. To uniformly predict gene functions, protein domains were assigned to COGs (orthologous gene clusters) [87] using RPS-BLAST and scoring matrices from the Conserved Domain Database (CDD) [88]. The plausibility of putative aptamer structures was assessed by computationally aligning hits to the original CM with Infernal and manually examining divergent RNA structures. Using these two complementary criteria, we established trusted CM score cutoffs. All hits in the microbial RefSeq database above these thresholds were judged to be functional riboswitches. Since gene context information is not available for most environmental sequences, hits from these data sets were included only if they had CM scores above the trusted threshold. Additional low-scoring sequences from the RefSeq database were also included when their genomic contexts and alignments strongly indicated that they were functional riboswitches.

To verify that this approach efficiently recovers known riboswitches, the final results were compared to a list of TPP riboswitches compiled in a comparative genomics analysis of thiamin metabolic genes and this regulatory RNA element [48]. The new searches successfully found all TPP riboswitches that had been previously identified in the set of complete microbial genomes analyzed in both studies. They also discovered a small number of TPP riboswitches upstream of thiamin-related genes (for example, a *pnuC *homolog in *Helicobacter pylori *and *thiM *in *Lactococcus lactis*) in genomes examined by the former study that had not yet been reported.

For the glycine riboswitch, a single aptamer covariance model and a tandem model containing both the first and second aptamers were used to separately identify matches. Every aptamer that is part of a tandem configuration was found by the single aptamer CM search, and cases of lone aptamers were noted. For consensus structure and MI calculations only the tandem glycine aptamer alignment was considered, but the complete set of lone and tandem aptamer glycine riboswitches were included in the expression platform analysis. Expression platform counts for other riboswitch classes that rarely occur in tandem were not corrected.

### Mechanism classification

Expression platforms were classified according to the scheme in Figure 2 for a subset of the riboswitch matches found in complete and unfinished microbial genomes. Aptamer sequences with more than 95% pairwise identity at reference columns (positions where ≥50% of the weighted sequences in the alignment do not contain a gap) were omitted to avoid biasing statistics with duplicate sequences. Riboswitches with suspect gene annotations where >60 nucleotides (nt) of an open reading frame (ORF) on the same strand overlapped the aptamer or >700 nt separated the aptamer and the nearest downstream ORF were also screened out. Most of these cases appear to result from incorrect start codon choices, overpredictions of hypothetical ORFs, or missing annotation of real genes. The remaining sequences constituted the expression platform data set, and sequences beginning at the 5' end of each aptamer and continuing through the first 120 nt of the downstream ORF were extracted for further analysis.

Riboswitches where the downstream gene was on the opposite strand were examined as candidates for antisense regulation. Other riboswitches were classified as directly regulating translation initiation when the downstream gene's start codon was within 15 nt of the end of the conserved aptamer core structure (usually the P1 paired element). The remaining expression platforms were scanned with the local RNA secondary structure prediction program Rnall (version 1.1) [89] for intrinsic transcription terminators with a scanning window of 50 nt, a U-tail weight threshold of 4.0, a U-tail pairing stability cutoff of -8.3 kcal/mol, and default settings for other parameters. Riboswitches with a terminator predicted in their expression platform sequence were assigned transcription attenuation mechanisms. These riboswitches were classified as also regulating translation if the distance between the terminator hairpin and the gene's start codon is no more than 10 nt. Expression platforms that did not match any of the above criteria are assumed to employ translation attenuation mechanisms.

Rnall and distance parameters were calibrated by comparing expression platform predictions to expert predictions for a large and phylogenetically diverse collection of TPP riboswitches [48]. Rnall correctly predicts 46 out of 52 terminators in this data set with only 3 predictions of terminators in sequences not manually evaluated as containing a terminator (a sensitivity of 88% and an accuracy of 94%). The three false positives resemble terminators and may be functional, whereas the terminators that Rnall misses usually have large hairpins with poor thermodynamic stabilities. Overall, the decision tree classifies 159 out of 180 TPP riboswitch expression platforms (88%) correctly into the category assigned in the control set.

### Consensus secondary structures

We manually adjusted the covariance model alignments of riboswitch aptamers while refining their consensus secondary structures. In particular, bases taking part in pseudoknotted pairings that cannot be represented by CMs were shifted to accurately represent these interactions. Bases flanking gapped consensus columns, which are sometimes ambiguously spread out across many possible positions by the alignment algorithm, were also systematically condensed into a minimum number of overall consensus columns. As new structure motifs and base-base interactions became evident, the alignments were adjusted to reflect these new constraints. Riboswitch sequences in the final alignments were weighted using Infernal's internal implementation of the GSC algorithm [90] to reduce biases from duplicate and similar sequences before calculating consensus structure statistics.

### Mutual information significance

Duplicate sequences were purged and columns with >50% gaps were removed from riboswitch alignments prior to the MI analysis, and, if necessary, alignments were further pruned to the 300 most diverse sequences (as judged by pairwise base differences). A customized version of the program Rate4Site (version 2.01) [91] with modified output options was used to simultaneously estimate distances and per-column rates of evolution according to a gamma distributed model with at least 16 rate categories and a phylogenetic tree created with Jukes-Cantor distances that treated gaps as missing information. The resulting trees, rates, and distances were used to simulate 10,000 resampled alignments starting from an arbitrary ancestral sequence. Then, gaps and sequence weights were re-inserted into each of these derivative alignments at the same positions that they occupied in the original alignment.

Mutual information was calculated between column pairs for all alignments according to standard formulas [60], taking into account sequence weights and treating gaps as a fifth character state. The resampled alignments were used to estimate what the MI score distribution would have been if the bases present in each column had evolved independently, without covariation constraints. The *p *value significance of the actual MI between two columns is the fraction of the resampled alignments that have a greater MI score than the value observed between those two columns in the real alignment.

## Abbreviations

AdoCbl, adenosylcobalamin; CM, covariance model; FMN, flavin mononucleotide; GlcN6P, glucosamine-6-phosphate; H, Hoogsteen face; MI, mutual information; nt, nucleotides; ORF, open reading frame; preQ_1_, 7-aminoethyl 7-deazaguanine; RBS, ribosome binding site; SAM, *S*-adenosylmethionine; SE, sugar edge; TPP, thiamin pyrophosphate; UTR, untranslated region; WC, Watson-Crick face.

## Authors' contributions

JEB designed the computational analyses, carried out the comparative studies, and created the figures. JEB and RRB interpreted the results and wrote the manuscript.

## Additional data files

The following additional data files are available with the online version of this article. Additional data file 1 contains sequence alignments of the riboswitch aptamer data sets annotated with new base-base interactions in Stockholm format. Additional data file 2 contains sequence alignments of the riboswitch aptamer data sets annotated with new base-base interactions in HTML format.

## Supplementary Material

Additional data file 1 Sequence alignments of the riboswitch aptamer data sets annotated with new base-base interactions in Stockholm format. Click here for file

Additional data file 2 Sequence alignments of the riboswitch aptamer data sets annotated with new base-base interactions in HTML format. Click here for file
