# Supplementary material for: The distributions, mechanisms, and structures of metabolite-binding riboswitches
Source: Genome Biol. 2007 Nov 12;8(11):R239. doi: 10.1186/gb-2007-8-11-r239 (PMC2258182; doi:10.1186/gb-2007-8-11-r239)
Supplement: Additional data file 2 — Sequence alignments of the riboswitch aptamer data sets annotated with new base-base interactions in HTML format. [file gb-2007-8-11-r239-S2.zip › HTML/AdoCbl.html]

|  |  |  |  |  |
| --- | --- | --- | --- | --- |
|  |  | **Accession/Start-End** |  | **Sequence** |
|  |  | NC\_002678.2/6749128-6749334  | CUACGGUGGGU**G****C****G****U****G**..**A**.**U**..**G**.**G**.U...**C****C**.**C**.**C****G****C****G**CC.................................GAAA..........GG**C****A****A****G****G**.**G****G****U**..G....AA..**A****A**..**G**..**G**.G..AA.**C**..**A**.**C**.**G****G**.**U**GAGACCUCAAAAGGUC.......................................................................................................................................................G**A**GA.....**C****C****G****U**.**G****G****C****U****G****C**C..C.CC..**G****C****A****A****C****U****G****U**AA..**G****C****G****G**.....................................................AGAGCA**A****G****A****U****C****C**GACAU..........................................................................................**G****C****C**A...**C**.**U****G****G****C****C**.................GGCAA................................................................................**G****G****C****U****G****G**G.A.A.**G****G****C**..A**G****G****A**..**U****U****G**CGCUGAGAC-.......................................................................................................**C****C****G****C**...G.**A**.....**G**.**C**....**C****A****G**....**G**AG..A**C**.**C**..**U**.....G...**C****C**.**A**..**U**.**C****A****C****U****G**AGUUGACCGGA | |
|  |  | NC\_006177.1/2078690-2078498  | AAUACGGGCAC**A****G****C****G****G**..**C**.**A**..**G**.**G**.UG..**C****U**.**C**.**C****C****G****G**C..................................CCCG........CGCU**C****C****G****G****G**.**A****G****U**..U....AA..**A****A**..**G**..**G**.G..AA.**G**..**C**.**C**.**G****G**.**U**GC.....................................................................................................................................................................A**A**GU.....**C****C****G****G**.**C****A****C****G****G****U**-..C.CC..**G****C****C****A****C****U****G****U**GA..**C****G****G**-.....................................................GGAGCC**G****U****C****C****C****C**ACGAUGC........................................................................................**G****C****C**A...**C**.**U****G****G****C****C**.................GGAA-................................................................................**G****G****C****U****G****G**G.A.A.**G****G****C**...**G****G****G**..**G****G****A**AGGCGAUGAU.......................................................................................................-**C****C****G**...A.**A**.....**G**.**U**....**C****A****G**....**G**AG..A**C**.**C**..**U**.....G...**C****C**.**U**..**G**.**U****C****G****C****U**GCAACGUCACU | |
|  |  | NC\_006526.1/1014953-1015154  | GCAAUGAGGAA**G****G****A****U****U**..**A**.**A**..**G**.**G**.UU..**C****U**.**U**.**U****G****U****C**...................................-AUU............**G****G****C****A****A**.**A****G****C**..U....AA..**G****A**..**G**..**G**.G..AA.**A**..**C**.**U**.**G****G**.**U**GCGAAAGAAUUUUC.........................................................................................................................................................A**A**AG.....**C****C****A****G**.**U****G****C****U****G****C**C..C.CC..**G****C****A****A****C****U****G****U**AA..**A****C****G****G**.....................................................CGAGCA**A****A****G****A****U****C**AAAAU..........................................................................................**G****C****C**A...**C**.**U****G****A****U****A**.................UUAU-................................................................................**U****A****U****C****G****G**G.A.A.**G****G****C**..U**G****A****U**..**C****G****G**ACGCGGUGAC.......................................................................................................**C****C****G****U**..CA.**A**.....**G**.**U**....**C****A****G**....**G**AG..A**C**.**C**..**U**.....G...**C****C**.**U**..**U**.**A****A****A****C****C**AAGUCAUCCAC | |
|  |  | NZ\_AAAG02000002.1/239969-239773  | UAGCCUAUGUU**G****U****G****U****U**..**A**.**C**..**G**.**G**.UU..**C****C**.**C**.**C****G****G****A**CACC...............................ACAA..........UA**U****C****C****G****G**.**G****G****C**..U....AA..**G****A**..**G**..**G**.G..AA.**G**..**C**.**C**.**G****G**.**U**GC.....................................................................................................................................................................G**A**UG.....**C****C****G****G**.**C****G****C****U****G****C**C..C.CC..**G****C****A****A****C****U****G****U**CA..**G****C****G****G**C....................................................GAGCCU**U****C****A****U****C****C**AUUCAU.........................................................................................**G****C****C**A...**C**.**U****G****G****G****G**.................AAAU-................................................................................**U****C****C****C****G****G**G.A.A.**G****G****C**...**G****G****A**..**C****G****A**AGGCGACGAC.......................................................................................................**C****C****G****U**...G.**A**.....**G**.**C**....**C****A****G**....**G**AG..A**C**.**C**..**U**.....G...**C****C**.**G**..**U**.**G****A****C****C****C**UUGAAACGUCC | |
|  |  | NC\_006177.1/2074430-2074237  | AUAGAGCAAAC**A****G****C****G****G**..**C**.**A**..**G**.**G**.UG..**C****U**.**C**.**C****C****G****A**C..................................CCUG.........CGG**U****C****G****G****G**.**A****G****U**..U....AA..**A****A**..**G**..**G**.G..AA.**G**..**C**.**C**.**G****G**.**U**GC.....................................................................................................................................................................A**A**GU.....**C****C****G****G**.**C****A****C****G****G****U**-..C.CC..**G****C****C****A****C****U****G****U**GA..**C****G****G****G**.....................................................GAGUCG**C****C****C****C****U****C**GGGAUGU........................................................................................**G****C****C**A...**C**.**U****G****G****C****C**.................CGAA-................................................................................**G****G****C****C****G****G**G.A.A.**G****G****C**..G**G****A****G**..**G****G****G**CGGCGAGGA-.......................................................................................................**U****C****C****G**...G.**A**.....**G**.**U**....**C****A****G**....**G**AA..A**C**.**C**..**U**.....G...**C****C**.**U**..**G**.**C****C****G****C****G**CUGGCGCUGCU | |
|  |  | NC\_002678.2/4957343-4957143  | UGAUUGUGCGC**A****U****G****U****C**..**G**.**U**..**G**.**G**.UU..**C****U**.**C**.**C****G****C****G**CGGCACU............................GCCG...........U**A****G****C****G****G**.**A****G****C**..U....AA..**G****A**..**G**..**G**.G..AA.**G**..**C**.**C**.**G****G**.**U**GC.....................................................................................................................................................................G**A**UG.....**C****C****G****G**.**C****G****C****U****G****C**C..C.CC..**G****C****A****A****C****U****G****U**UA..**G****C****G****G**.....................................................CGAGCC**A****A****G****C****C****C**AUUGGU.........................................................................................**G****U****C**A...**C**.**U****G****A****G****G**C................GAACG...............................................................................G**C****C****U****C****G****G**G.A.A.**G****A****C**...**G****G****G**..**C****A****G**AGGCUUUGAC.......................................................................................................**C****C****G****C**...G.**A**.....**G**.**C**....**C****A****G**....**G**AG..A**C**.**C**..**U**.....G...**C****C**.**A**..**C**.**G****A****C****G****A**ACAACGUCCAC | |
|  |  | NZ\_AABQ07000001.1/543893-544098  | CCCAUCCGGCC**C****G****U****U****C**..**C**.**A**..**G**.**G**.UG..**C****C**.**U**.**C****C****U****G**CC.................................GCCG..........CG**C****A****G****G****A**.**G****G****U**..G....AA..**A****C**..**G**..**G**.G..AA.**G**..**C**.**C**.**G****G**.**U**GCGUCACUUCAGUGAU.......................................................................................................................................................C**A**GU.....**C****C****G****G**.**C****G****C****U****G****C**C..C.CC..**G****C****A****A****C****G****G****U**AA..**G****C****G****A**.....................................................-GCGAA**A****U****C****C****U****C**UUCAG..........................................................................................**G****C****C**A...**C**.**U****G****U****G****C**.................UCCA-................................................................................**G****C****A****U****G****G**G.A.A.**G****G****C**...**G****A****G**..**G****A****U**UUCACGACCC.......................................................................................................**U****C****G****C**...A.**A**.....**G**.**C**....**C****C****G**....**G**AG..A**C**.**C**..**G**.....G...**C****C**.**U**..**G**.**C****A****A****C****G**CCCUGUUGGCA | |
|  |  | NC\_005296.1/2715829-2716025  | CAUAGUGAUCG**C****C****G****U****C**..**G**.**A**..**G**.**G**.UU..**C****U**.**U**.**C****G****G****G**CACG...............................AUCG...........U**C****C****C****G****A**.**A****G****C**..U....AA..**G****A**..**G**..**G**.G..AA.**G**..**C**.**C**.**G****G**.**U**GC.....................................................................................................................................................................A**A**GG.....**C****C****G****G**.**C****G****C****U****G****C**C..C.CC..**G****C****A****A****C****U****G****U**UA..**G****C****G****G**U....................................................GAGCCG**A****C****G****U****U****C**AGCAAA.........................................................................................**G****C****C**A...**C**.**U****G****G****G****A**.................GCGA-................................................................................**U****C****C****C****G****G**G.A.A.**G****G****U**..C**G****A****A**..**C****A****A**AGGCGAUGAC.......................................................................................................**C****C****G****C**...G.**A**.....**G**.**C**....**C****A****G**....**G**AG..A**C**.**C**..**U**.....G...**C****C**.**U**..**C**.**G****U****C****G****A**ACGAAACGUCC | |
|  |  | NZ\_AAIG01000016.1/8169-7981  | GAACAGGGUCA**C****G****G****C****G**..**A**.**U**..**G**.**G**.U...**G****C**.**C**.**C****U****U**-...................................CUUG............-**G****A****G****G**.**G****C****C**..U....AA..**G****A**..**G**..**G**.G..AA.**C**..**G**.**C**.**G****G**.**U**GA.....................................................................................................................................................................A**A**CC.....**C****C****G****C**.**G****G****C****U****G****C**C..C.CC..**G****C****A****A****C****U****G****U**AA..**G****C****G****G**.....................................................UGAGCG**G****A****C****C****U****G**CGUAU..........................................................................................**G****C****C**A...**C**.**U****G****A****G****C**CG...............ACAA-................................................................................**G****C****U****C****G****G**G.A.A.**G****G****C**..G**C****A****G**..**G****C****C**CCGCCCUGAC.......................................................................................................**C****C****G****C**...A.**A**.....**G**.**C**....**C****A****G**....**G**AG..A**C**.**C**..**U**.....G...**C****C**.**A**..**U**.**C****A****C****C****U**GUGAAACGCAU | |
|  |  | NC\_002696.2/1924997-1925186  | AGCUCUAGCUU**C****G****C****G****U**..**C**.**A**..**G**.**G**.U...**U****C**.**C**.**U****C**--...................................GAAA............--**G****A****G**.**G****A****U**..G....AA..**A****A**..**G**..**G**.G..AA.**C**..**G**.**A**.**G****G**.**U**UG.....................................................................................................................................................................A**A**GA.....**C****C****U****C**.**G****G****C****U****G****C**C..C.CC..**G****C****A****A****C****U****G****U**AA..**G****C****G****G**C....................................................GAGCUU**C****G****C****G****U****C**ACAU-..........................................................................................**G****C****C**A...**C**.**U****G****G****G****C**CC...............AAAAG................................................................................**G****C****C****U****G****G**G.A.A.**G****G****C**...**G****A****C**..**G****C****C**CAGAAGCAUU....................................................................................................GAC**C****C****G****U**...G.**A**.....**G**.**C**....**C****A****G**....**G**AG..A**C**.**C**..**U**.....G...**C****C**.**C**..**G**.**G****C****G****C****A**GUCGUUCAUCG | |
|  |  | NC\_002947.3/2768973-2768765  | AGAUGCGCGCC**A****G****U****U****U**..**C**.**A**..**G**.**G**.UG..**C****C**.**C**.**U****G****C****G**CC.................................GCCG..........CG**C****G****C****A****G**.**G****G****U**..G....AA..**A****C**..**G**..**G**.G..AA.**A**..**C**.**C**.**G****G**.**U**GCGUCGUGUUGCCCACGAC....................................................................................................................................................A**A**GU.....**C****C****G****G**.**U****G****C****U****G****C**C..C.CC..**G****C****A****A****C****G****G****U**AA..**G****C****G****A**.....................................................-GCGAA**C****C****C****U****U****C**GAGAU..........................................................................................**A****C****C**A...**C**.**U****G****U****G****C**.................UCAA-................................................................................**G****C****A****U****G****G**G.A.A.**G****G****U**...**G****A****A**..**G****G****U**UUCAUGCCCC.......................................................................................................**U****C****G****C**...A.**A**.....**G**.**C**....**C****C****G**....**G**AG..A**C**.**C**..**G**.....G...**C****C**.**U**..**G**.**G****A****G****C****U**UCACUUGGCAA | |
|  |  | NC\_002516.1/3265543-3265748  | CCCAUCCGGCC**C****G****U****U****C**..**C**.**A**..**G**.**G**.UG..**C****C**.**U**.**C****C****U****G**CC.................................GCCG..........CG**C****A****G****G****A**.**G****G****U**..G....AA..**A****C**..**G**..**G**.G..AA.**G**..**C**.**C**.**G****G**.**U**GCGUCACUUCGGUGAU.......................................................................................................................................................C**A**GU.....**C****C****G****G**.**C****G****C****U****G****C**C..C.CC..**G****C****A****A****C****G****G****U**AA..**G****C****G****A**.....................................................-GCGAA**A****U****C****C****U****C**UUCAG..........................................................................................**G****C****C**A...**C**.**U****G****U****G****C**.................UCCG-................................................................................**G****C****A****U****G****G**G.A.A.**G****G****C**...**G****A****G**..**G****A****U**UUCACGACCC.......................................................................................................**U****C****G****C**...A.**A**.....**G**.**C**....**C****C****G**....**G**AG..A**C**.**C**..**G**.....G...**C****C**.**U**..**G**.**C****A****A****C****G**CCCUGUUGGCA | |
|  |  | NZ\_AAED02000003.1/507119-506864  | CAAAUCUGCAC**A****C****G****U****C**..**G**.**U**..**G**.**G**.U...**C****C**.**C**.**C****G****G****C**GUCU...............................UUCA..........GC**G****C****C****G****G**.**G****G****C**..U....AA..**G****A**..**G**..**G**.G..AA.**U**..**U**.**C**.**G****G**.**U**GCGCCCGUCAGCGCUAUUGCGCUUUCCGAAAGCUGGUUUUGGUUUUCGGGGUUGAUGUAUCGGGU......................................................................................................A**A**UG.....**C****C****G****A**.**A****G****C****U****G****C**C..C.CC..**G****C****A****A****C****U****G****U**GA..**G****C****G****G**C....................................................GAGCUC**U****U****C****U****C****C**AACAU..........................................................................................**G****C****C**A...**C**.**U****G****G****A**-.................-GACA................................................................................-**C****C****C****G****G**G.A.A.**G****G****C**...**G****G****A**..**G****A****G**AGGCUGUGAC.......................................................................................................**C****C****G****C**...G.**A**.....**G**.**C**....**C****A****G**....**G**AG..A**C**.**C**..**U**.....G...**C****C**.**A**..**U**.**G****A****C****G****A**GAACGUCCACG | |
|  |  | NC\_003902.1/3631190-3630967  | UACCAUGCGCG**C****C****C****C****U**..**G**.**A**..**G**.**G**.UG..**A****C**.**U**.**G****C****C****G**G..................................AAUU...........C**C****G****G****U****G**.**G****U****U**..U....AA..**A****C**..**G**..**G**.G..AA.**U**..**C**.**C**.**G****G**.**U**GCGCGCAUCGCCUUGGCGAGACGC...............................................................................................................................................A**A**GU.....**C****C****G****G**.**A****G****C****U****G****C**C..C.CC..**G****C****A****A****C****G****G****U**GG..**G****C****G****A**G....................................................GUCAGG**U****G****C****C****G****C**AACAG..........................................................................................**G****C****C**A...**C**.**U****G****U****G****C**.................ACAC-................................................................................**G****C****A****U****G****G**G.A.A.**G****G****C**...**G****C****G**..**G****U****A**CCGGAAGCGC.............................................................................................AGGCUUCCAC**U****C****G****C**...G.**A**.....**G**.**C**....**C****C****G**....**G**AG..A**C**.**C**..**G**.....G...**C****C**.**U**..**G**.**A****G****G****G****A**UUGACCCGGCA | |
|  |  | NC\_007086.1/1316898-1317121  | UACCAUGCGCG**C****C****C****C****U**..**G**.**A**..**G**.**G**.UG..**A****C**.**U**.**G****C****C****G**G..................................AAUU...........C**C****G****G****U****G**.**G****U****U**..U....AA..**A****C**..**G**..**G**.G..AA.**U**..**C**.**C**.**G****G**.**U**GCGCGCAUCGCCUUGGCGAGACGC...............................................................................................................................................A**A**GU.....**C****C****G****G**.**A****G****C****U****G****C**C..C.CC..**G****C****A****A****C****G****G****U**GG..**G****C****G****A**G....................................................GUCAGG**U****G****C****C****G****C**AACAG..........................................................................................**G****C****C**A...**C**.**U****G****U****G****C**.................ACAC-................................................................................**G****C****A****U****G****G**G.A.A.**G****G****C**...**G****C****G**..**G****U****A**CCGGAAGCGC.............................................................................................AGGCUUCCAC**U****C****G****C**...G.**A**.....**G**.**C**....**C****C****G**....**G**AG..A**C**.**C**..**G**.....G...**C****C**.**U**..**G**.**A****G****G****G****A**UUGACCCGGCA | |
|  |  | NZ\_AAIT01000014.1/65427-65222  | GUAACAUAUGC**G****U****G****U****A**..**U**.**C**..**G**.**G**.UG..**C****C**.**C**.**C**---...................................UUUC............---**G****G**.**G****G****C**..C....AA..**A****A**..**G**..**G**.G..AA.**U**..**G**.**C**.**G****G**.**U**GCCGUCUCCGGCGUCUUGGGGACC...............................................................................................................................................A**A**AU.....**C****C****G****C**.**A****G****C****C****G****C**C..C.CC..**G****C****G****A****C****U****G****U**AG..**G****C****G****G**A....................................................GAGCGG**A****C****G****C****C****C**AUCGU..........................................................................................**G****C****C**A...**C**.**U****G****G****C****C**.................AUCC-................................................................................**G****G****C****C****G****G**G.A.A.**G****G****C**...**G****G****G**..**C****G****U**CAGCCGUGAC.......................................................................................................**C****C****G****C**...G.**A**.....**G**.**C**....**C****A****G**....**G**AG..A**C**.**C**..**U**.....G...**C****C**.**G**..**A**.**U****G****C****G****U**GAAACCGCAAU | |
|  |  | NZ\_AAIS01000004.1/502007-501803  | CCAUAGGUUGG**U****U****G****U****C**..**G**.**A**..**G**.**G**.UU..**C****U**.**C**.**C****G****G****G**UA.................................UUCA..........UG**C****U****U****G****G**.**A****G****C**..U....AA..**G****A**..**G**..**G**.G..AA.**G**..**C**.**C**.**G****G**.**U**GCGCUCUUGAGC...........................................................................................................................................................A**A**GA.....**C****C****G****G**.**C****G****C****U****G****C**C..C.CC..**G****C****A****A****C****U****G****U**AA..**G****C****G****G**U....................................................GAGCCG**U****G****G****U****C****C**AUCAU..........................................................................................**G****C****C**A...**C**.**U****G****G****G****U**.................GUUG-................................................................................**U****C****C****C****G****G**G.A.A.**G****G****C**..C**G****G****A**..**C****C****A**AAGCUGAGAC.......................................................................................................**C****C****G****C**...G.**A**.....**G**.**C**....**C****A****G**....**G**AG..A**C**.**C**..**U**.....G...**C****C**.**C**..**C**.**G****G****C****A****A**CAUAGUCGUCC | |
|  |  | NZ\_AAIT01000004.1/82476-82280  | ACUACACGGUC**G****G****G****U****G**..**A**.**C**..**G**.**G**.U...**U****U**.**C**.**C****G**--...................................AUUU............--**C****G****G**.**A****A****U**..G....AA..**A****A**..**G**..**G**.G..AA.**C**..**G**.**C**.**A****G**.**U**GCGGACCAGCCGGUCC.......................................................................................................................................................C**A**UG.....**C****U****G****C**.**G****G****C****U****G****C**C..C.CC..**G****C****A****A****C****U****G****U**AG..**G****C****G****G**.....................................................AUAGCC**U****U****U****G****C****C**AACG-..........................................................................................**G****C****C**A...**C**.**U****G****G****G****U**.................GCGA-................................................................................**C****C****C****C****G****G**G.A.A.**G****G****C**...**G****G****C**..**G****A****C**AGGCGACGAC.......................................................................................................**C****C****G****C**...G.**A**.....**G**.**C**....**C****A****G**....**G**AG..A**C**.**C**..**U**.....G...**C****C**.**G**..**U**.**C****A****G****C****C**GUUGUCACGCG | |
|  |  | NC\_003063.1/1153059-1152850  | ACAUUGGUUAG**C****C****A****U****C**..**G**.**U**..**G**.**G**.UU..**C****U**.**G**.**C****G****G****A**C..................................GAAG...........G**U****C****C****G****G**.**A****G****C**..U....AA..**G****A**..**G**..**G**.G..AA.**U**..**U**.**C**.**G****G**.**U**GAGGGCUUUAAUCACAGCCU...................................................................................................................................................G**A**AU.....**C****C****G****A**.**A****G****C****U****G****C**C..C.CC..**G****C****A****A****C****U****G****U**AA..**G****C****G****A**C....................................................GAGCGA**A****A****G****U****C****C**AUCAU..........................................................................................**G****U****C**A...**C**.**U****G****A****G****G**.................-CCGG................................................................................**C****C****U****C****G****G**G.A.A.**G****A****C**...**G****G****A**..**C****C****A**AAGCUAUGAC.......................................................................................................**C****C****G****C**...A.**A**.....**G**.**C**....**C****A****G**....**G**AG..A**C**.**C**..**U**.....G...**C****C**.**G**..**C**.**G****A****U****A****G**AUAACGUCCAC | |
|  |  | NC\_003305.1/921697-921906  | ACAUUGGUUAG**C****C****A****U****C**..**G**.**U**..**G**.**G**.UU..**C****U**.**G**.**C****G****G****A**C..................................GAAG...........G**U****C****C****G****G**.**A****G****C**..U....AA..**G****A**..**G**..**G**.G..AA.**U**..**U**.**C**.**G****G**.**U**GAGGGCUUUAAUCACAGCCU...................................................................................................................................................G**A**AU.....**C****C****G****A**.**A****G****C****U****G****C**C..C.CC..**G****C****A****A****C****U****G****U**AA..**G****C****G****A**C....................................................GAGCGA**A****A****G****U****C****C**AUCAU..........................................................................................**G****U****C**A...**C**.**U****G****A****G****G**.................-CCGG................................................................................**C****C****U****C****G****G**G.A.A.**G****A****C**...**G****G****A**..**C****C****A**AAGCUAUGAC.......................................................................................................**C****C****G****C**...A.**A**.....**G**.**C**....**C****A****G**....**G**AG..A**C**.**C**..**U**.....G...**C****C**.**G**..**C**.**G****A****U****A****G**AUAACGUCCAC | |
|  |  | NC\_004463.1/5279689-5279475  | CGUAGAUUGAU**C****G****G****U****G**..**A**.**C**..**G**.**G**.U...**U****C**.**U**.**C****C**--...................................GCAC............--**G****G****A**.**G****A****U**..C....AA..**A****A**..**G**..**G**.G..AA.**C**..**G**.**U**.**G****G**.**U**GCGAGAUUGUCCCAAUGCCGGGAUUGUCCC.........................................................................................................................................A**A**CG.....**C****C****A****C**.**G****G****C****U****G****C**C..C.CC..**G****C****A****A****C****U****G****U**AA..**G****C****G****G**U....................................................GAAUCU**U****U****C****G****U****C**AUAU-..........................................................................................**G****C****C**A...**C**.**U****G****G****G****A**A................UCUCG...............................................................................G**U****C****C****U****G****G**G.A.A.**G****G****C**...**G****A****C**..**G****U****A**AGGUAACGAC.......................................................................................................**C****C****G****C**...G.**A**.....**G**.**C**....**C****A****G**....**G**AG..A**C**.**C**..**U**.....G...**C****C**.**G**..**U**.**C****A****G****C****C**GUGGUCACACG | |
|  |  | NZ\_AAIS01000001.1/880810-880613  | UUCUAAAUGAC**C****G****G****C****G**..**A**.**C**..**G**.**G**.U...**U****C**.**U**.**C****C**--...................................CUCG............--**G****G****A**.**G****A****U**..C....AA..**A****A**..**G**..**G**.G..AA.**C**..**G**.**U**.**G****G**.**U**GCGGGAUCAUCCU..........................................................................................................................................................A**A**CG.....**C****C****A****C**.**G****G****C****U****G****C**C..C.CC..**G****C****A****A****C****U****G****U**AA..**G****C****G****G**.....................................................CGAAUC**U****G****C****G****U****C**ACAG-..........................................................................................**G****C****C**A...**C**.**U****G****G****G****A**CA...............UCUCG...............................................................................G**C****C****C****C****G****G**G.A.A.**G****G****C**...**G****A****C**..**G****C****C**AGGUAACGAC.......................................................................................................**C****C****G****C**...G.**A**.....**G**.**C**....**C****A****G**....**G**AG..A**C**.**C**..**U**.....G...**C****C**.**G**..**U**.**C****A****A****C****C**GUGGUCACACG | |
|  |  | NC\_005296.1/449890-449694  | AAUACCGUGAC**C****A****G****C****G**..**A**.**C**..**G**.**G**.U...**U****C**.**C**.**C**---...................................GAAA............---**G****G**.**G****A****U**..C....AA..**U****A**..**G**..**G**.G..AA.**C**..**G**.**C**.**G****G**.**U**GCGGGCUUUAGGGCUC.......................................................................................................................................................U**A**UU.....**C****C****G****C**.**G****G****C****U****G****C**C..C.CC..**G****C****A****A****C****U****G****U**AA..**G****C****G****G**C....................................................GAGCCA**U****U****C****G****C****C**ACAC-..........................................................................................**G****C****C**A...**C**.**U****G****G****G****C**.................UUUCG................................................................................**U****C****C****U****G****G**G.A.A.**G****G****C**...**G****G****U**..**G****A****A**CGGCAACGAC.......................................................................................................**C****C****G****C**...G.**A**.....**G**.**C**....**C****A****G**....**G**AG..A**C**.**C**..**U**.....G...**C****C**.**G**..**U**.**C****A****G****U****C**GUGGUCACACG | |
|  |  | NZ\_AAIG01000002.1/116167-115953  | UGAAAUCAAAC**U****G****G****U****G**..**A**.**U**..**G**.**G**.UG..**U****U**.**U**.**C****G****A****C**AUCCAA.............................GACG..........AU**G****U****C****G****G**.**A****C****U**..G....AA..**A****A**..**G**..**G**.G..AA.**U**..**A**.**C**.**G****G**.**U**GAGGACGCGCCUUAGGGCACC..................................................................................................................................................A**A**AU.....**C****C****G****U**.**A****A****C****U****G****C**C..C.CC..**G****C****A****A****C****U****G****U**AA..**G****C****G****G**.....................................................UAAGCG**A****C****G****C****C****A**AAUAU..........................................................................................**G****C****C**A...**C**.**U****G****G****G****A**.................CAGC-................................................................................**U****C****C****C****G****G**G.A.A.**G****G****U**...**U****G****G**..**C****C****A**AGCGUUGAU-.......................................................................................................**C****C****G****C**...A.**A**.....**G**.**U**....**C****A****G**....**G**AG..A**C**.**C**..**U**.....G...**C****C**.**A**..**U**.**C****A****G****G****U**GUUGAAACAUU | |
|  |  | NC\_003919.1/3758448-3758225  | UACCAUGCGCG**C****C****C****C****U**..**G**.**A**..**G**.**G**.UG..**A****C**.**U**.**G****C****C****G**G..................................UUGG...........C**C****G****G****U****G**.**G****U****U**..U....AA..**A****C**..**G**..**G**.G..AA.**U**..**C**.**C**.**G****G**.**U**GCGCGGAUCGCCUUGGCGAGCUGC...............................................................................................................................................A**A**UU.....**C****C****G****G**.**A****G****C****U****G****C**C..C.CC..**G****C****A****A****C****G****G****U**GG..**G****C****G****A**G....................................................GUCAGA**U****G****C****C****G****C**ACUAC..........................................................................................**G****C****C**A...**C**.**U****G****U****G****C**.................AGUC-................................................................................**G****C****A****U****G****G**G.A.A.**G****G****C**...**G****C****G**..**G****C****A**UCGGAAGCGC.............................................................................................CAGCUUCCAC**U****C****G****C**...A.**A**.....**G**.**C**....**C****C****G**....**G**AG..A**C**.**C**..**G**.....G...**C****C**.**U**..**G**.**A****G****G****G****A**UUGACCCGGCA | |
|  |  | NC\_003318.1/559738-559970  | CCGUAAUACCG**U****C****A****U****G**..**A**.**C**..**G**.**G**.U...**U****C**.**C**.**C****C****G****A**CC.................................GAGA.........GCG**A****A****G****G****G**.**G****A****U**..U....AA..**U****A**..**G**..**G**.G..AA.**C**..**A**.**C**.**G****G**.**U**GAGGACGACCCAUCAAGGGGCC.................................................................................................................................................G**A**GA.....**C****C****G****U**.**G****G****C****U****G****C**C..C.CC..**G****C****A****A****C****U****G****U**AA..**G****C****G****G**A....................................................UUGCCG**U****U****C****A****U****C**CUCGUGACGCCGAAAGCGUCAU.........................................................................**G****C****C**A...**C**.**U****G****U****G****C**.................CCACG................................................................................**G****C****A****C****G****G**G.A.A.**G****G****C**..A**G****A****U**..**G****G****A**CGGCGAUUAU.......................................................................................................**C****C****G****C**...A.**A**.....**G**.**C**....**C****A****G**....**G**AG..A**C**.**C**..**U**.....G...**C****C**.**G**..**U**.**C****U****U****A****C**GUAGUCCAUUG | |
|  |  | NC\_004311.2/738657-738425  | CCGUAAUACCG**U****C****A****U****G**..**A**.**C**..**G**.**G**.U...**U****C**.**C**.**C****C****G****A**CC.................................GAGA.........GCG**A****A****G****G****G**.**G****A****U**..U....AA..**U****A**..**G**..**G**.G..AA.**C**..**A**.**C**.**G****G**.**U**GAGGACGACCCAUCAAGGGGCC.................................................................................................................................................G**A**GA.....**C****C****G****U**.**G****G****C****U****G****C**C..C.CC..**G****C****A****A****C****U****G****U**AA..**G****C****G****G**A....................................................UUGCCG**U****U****C****A****U****C**CUCGUGACGCCGAAAGCGUCAU.........................................................................**G****C****C**A...**C**.**U****G****U****G****C**.................CCACG................................................................................**G****C****A****C****G****G**G.A.A.**G****G****C**..A**G****A****U**..**G****G****A**CGGCGAUUAU.......................................................................................................**C****C****G****C**...A.**A**.....**G**.**C**....**C****A****G**....**G**AG..A**C**.**C**..**U**.....G...**C****C**.**G**..**U**.**C****U****U****A****C**GUAGUCCAUUG | |
|  |  | NC\_006933.1/478516-478748  | CCGUAAUACCG**U****C****A****U****G**..**A**.**C**..**G**.**G**.U...**U****C**.**C**.**C****C****G****A**CC.................................GAGA.........GCG**A****A****G****G****G**.**G****A****U**..U....AA..**U****A**..**G**..**G**.G..AA.**C**..**A**.**C**.**G****G**.**U**GAGGACGACCCAUCAAGGGGCC.................................................................................................................................................G**A**GA.....**C****C****G****U**.**G****G****C****U****G****C**C..C.CC..**G****C****A****A****C****U****G****U**AA..**G****C****G****G**A....................................................UUGCCG**U****U****C****A****U****C**CUCGUGACGCCGAAAGCGUCAU.........................................................................**G****C****C**A...**C**.**U****G****U****G****C**.................CCACG................................................................................**G****C****A****C****G****G**G.A.A.**G****G****C**..A**G****A****U**..**G****G****A**CGGCGAUUAU.......................................................................................................**C****C****G****C**...A.**A**.....**G**.**C**....**C****A****G**....**G**AG..A**C**.**C**..**U**.....G...**C****C**.**G**..**U**.**C****U****U****A****C**GUAGUCCAUUG | |
|  |  | NC\_004463.1/3634102-3634304  | AUCCUAGAUGC**U****C****G****C****G**..**A**.**C**..**G**.**G**.UU..**U****C**.**C**.**C****C****C**-...................................GAGA............--**G****G****G**.**G****A****U**..G....AA..**A****A**..**G**..**G**.G..AA.**U**..**G**.**C**.**G****G**.**U**GCGGGGAUGUUUCCCC.......................................................................................................................................................A**A**UG.....**C****C****G****C**.**G****G****C****U****G****C**C..C.CC..**G****C****A****A****C****U****G****U**AA..**G****C****G****G**A....................................................UAAUCC**U****U****C****G****U****C**AGAA-..........................................................................................**G****C****C**A...**C**.**U****G****G****G****U**C................CUCGG................................................................................**U****C****C****C****G****G**G.A.A.**G****G****C**...**G****A****C**..**G****A****A**GUGGUGACGA......................................................................................................C**C****C****G****C**...G.**A**.....**G**.**C**....**C****A****G**....**G**AG..A**C**.**C**..**U**.....G...**C****C**.**G**..**U**.**C****A****G****C****C**GUGGUCACACG | |
|  |  | NC\_003911.11/3757898-3758110  | CGCUACCGCUG**G****G****U****C****G**..**A**.**U**..**G**.**G**.U...**U****C**.**C**.**C****G****G****U**CGU................................GCAU..........GC**G****C****C****G****G**.**G****A****U**..G....AA..**A****A**..**G**..**G**.G..AA.**C**..**A**.**C**.**G****G**.**U**GCGGCCCUCUGGCGACAGAGGACC...............................................................................................................................................A**A**UG.....**C****C****G****A**.**G****A****C****U****G****C**C..C.CC..**G****C****A****A****C****U****G****U**AA..**G****C****G****G**.....................................................UGAGCG**A****U****G****C****C****G**AAAU-..........................................................................................**G****C****C**A...**C**.**U****G****G****C****C**.................-CCCG................................................................................**G****G****C****C****G****G**G.A.A.**G****G****C**...**C****G****G**..**C****A****A**AGCCCGGAC-.......................................................................................................**C****C****G****C**...A.**A**.....**G**.**U**....**C****A****G**....**G**AG..A**C**.**C**..**U**.....G...**C****C**.**A**..**U**.**C****G****C****A****A**CCGUAACUCGA | |
|  |  | NC\_005296.1/784348-784546  | GCUAGCGUGCC**A****A****G****C****G**..**A**.**U**..**G**.**G**.U...**C****C**.**U**.**U****C****C**-...................................GUCA............-**G****G****A****A**.**G****G****U**..G....AA..**A****A**..**G**..**G**.G..AA.**G**..**C**.**C**.**G****G**.**U**GGGAGGCGCGGUGCGUCUC....................................................................................................................................................G**A**UC.....**C****C****G****G**.**A****G****C****U****G****C**C..C.CC..**G****C****A****A****C****U****G****U**AA..**G****C****G****A**.....................................................CGAGCC**A****U****U****G****C****C**GAG--..........................................................................................**G****C****C**A...**C**.**U****G****G****G**-.................-AAUU................................................................................-**C****C****U****G****G**G.A.A.**G****G****C**...**G****G****C**..**G****A****A**UGGCGGCGAC.......................................................................................................**U****C****G****C**...G.**A**.....**G**.**C**....**C****A****G**....**G**AG..A**C**.**C**..**U**.....G...**C****C**.**A**..**U**.**C****G****C****G****U**AUUGUGCUUUG | |
|  |  | NZ\_AAIT01000019.1/32709-32517  | GCUAACUGCAA**C****G****G****U****C**..**A**.**U**..**G**.**G**.UUG.**U****C**.**C**.**C****G****G****G**G..................................CCGA...........C**U****C****G****G****G**.**G****A****U**..G....AA..**A****A**..**G**..**G**.G..AA.**G**..**C**.**C**.**G****G**.**U**GA.....................................................................................................................................................................A**A**AG.....**C****C****G****G**.**C****A****C****U****G****C**C..C.CC..**G****C****A****G****C****U****G****U**AA..**G****C****G****G**A....................................................GAGCGA**C****G****C****C****G****G**AUCA-..........................................................................................-**C****C**A...**C**.**U****G****G****G****G**CC...............ACGCG................................................................................**C****C****C****C****G****G**G.A.A.**G****G**-...**C****C****G**..**G****C****C**AAGCCGAGAC.......................................................................................................**C****C****G****C**...A.**A**.....**G**.**U**....**C****A****G**....**A**AG..A**C**.**C**..**U**.....G...**C****C**.**A**..**U**.**G****A****C****G****C**GAAAGGACGAA | |
|  |  | NC\_003078.1/580383-580598  | CAUACCAGAUC**A****U****G****U****G**..**A**.**U**..**G**.**G**.U...**U****C**.**C**.**G****C****C****C**GACU...............................GAAG..........AA**C****G****G****C****G**.**G****A****U**..G....AA..**A****A**..**G**..**G**.G..AA.**C**..**A**.**C**.**G****G**.**U**GAGGACGACCCAUCAGGGGCU..................................................................................................................................................A**A**AA.....**C****C****G****U**.**G****G****C****U****G****C**C..C.CC..**G****C****A****A****C****U****G****U**GA..**G****C****G****G**.....................................................CGAGCA**A****A****G****U****C****C**AAGGAU.........................................................................................**G****C****C**A...**U**.**U****G****G****C****C**A................UGAAU...............................................................................C**G****G****C****U****G****A**U.A.A.**G****G****C**...**G****G****A**..**C****A****A**AGCUACGAC-.......................................................................................................**C****C****G****C**...A.**A**.....**G**.**C**....**C****A****G**....**G**AG..A**C**.**C**..**U**.....G...**C****C**.**A**..**U**.**C****A****C****C****U**UGGGCGACACG | |
|  |  | NC\_002678.2/1149122-1149327  | CCUAAAUCCGC**U****C****C****A****G**..**A**.**C**..**G**.**G**.U...**C****C**.**C**.**U****U****G****C**CC.................................GCAA..........CG**G****C****A****G****G**.**G****G****C**..U....AA..**G****A**..**G**..**G**.G..AA.**U**..**G**.**C**.**G****G**.**U**GCGGGAUUUCGAUCUC.......................................................................................................................................................A**A**AU.....**C****C****G****C**.**G****G****C****U****G****U**C..C.CC..**G****C****A****A****C****U****G****U**AA..**G****C****G****A**.....................................................AGAGCC**A****A****G****G****C****C**GAAA-..........................................................................................**G****C****C**A...**C**.**U****G****G****G**-.................ACGUU................................................................................-**C****C****C****G****G**G.A.A.**G****G****C**...**G****G****C**AC**C****C****A**AGGCGAUGAC.......................................................................................................**C****C****G****C**...G.**A**.....**G**.**C**....**C****A****G**....**G**AG..A**C**.**C**..**U**.....G...**C****C**.**G**..**U**.**C****U****G****C****G**ACAAAAGAAUC | |
|  |  | NC\_003062.1/2809985-2809768  | UAAGGGUAAGG**G****A****C****U****G**..**A**.**C**..**G**.**G**.U...**C****U**.**U**.**U****U****C****C**CG.................................GCAA..........CG**G****G****A****A****A**.**A****G****C**..U....AA..**G****A**..**G**..**G**.G..AA.**C**..**A**.**C**.**G****G**.**U**UCCGCCCCGAGAAAGGGU.....................................................................................................................................................C**A**UU.....**C****C****G****U**.**G****G****C****U****G****C**C..C.CC..**G****C****A****A****C****U****G****U**AA..**G****C****G****G**.....................................................UAAGCC**C****G****C****A****C****C**GUAAA..........................................................................................**G****C****C**A...**C**.**U****G****A****A****C**C................UUUAU...........................................................................GAUCG**G****U****U****C****G****G**G.A.A.**G****G****C**...**G****G****U**..**G****A****C**AGGGUGUUGA....................................................................................................UAG**C****C****G****C**...A.**A**.....**G**.**C**....**C****A****G**....**G**AG..A**C**.**C**..**U**.....G...**C****C**.**G**..**U**.**U****U****C****A****G**GAAAAAGCGUC | |
|  |  | NC\_003304.1/2810096-2809879  | UAAGGGUAAGG**G****A****C****U****G**..**A**.**C**..**G**.**G**.U...**C****U**.**U**.**U****U****C****C**CG.................................GCAA..........CG**G****G****A****A****A**.**A****G****C**..U....AA..**G****A**..**G**..**G**.G..AA.**C**..**A**.**C**.**G****G**.**U**UCCGCCCCGAGAAAGGGU.....................................................................................................................................................C**A**UU.....**C****C****G****U**.**G****G****C****U****G****C**C..C.CC..**G****C****A****A****C****U****G****U**AA..**G****C****G****G**.....................................................UAAGCC**C****G****C****A****C****C**GUAAA..........................................................................................**G****C****C**A...**C**.**U****G****A****A****C**C................UUUAU...........................................................................GAUCG**G****U****U****C****G****G**G.A.A.**G****G****C**...**G****G****U**..**G****A****C**AGGGUGUUGA....................................................................................................UAG**C****C****G****C**...A.**A**.....**G**.**C**....**C****A****G**....**G**AG..A**C**.**C**..**U**.....G...**C****C**.**G**..**U**.**U****U****C****A****G**GAAAAAGCGUC | |
|  |  | NZ\_AAAP01002228.1/195-394  | CCUAGCGUGGC**C****G****U****C****G**..**A**.**C**..**G**.**G**.U...**U****C**.**C**.**C**---...................................GAAA............---**G****G**.**G****A****U**..C....AA..**A****A**..**G**..**G**.G..AA.**C**..**G**.**C**.**G****G**.**U**GAAGGGCCUUUGCCC........................................................................................................................................................G**A**UC.....**C****C****G****C**.**G****G****C****U****G****C**C..C.CC..**G****C****A****A****C****U****G****U**GA..**G****C****G****G**A....................................................GAGCCC**U****C****C****A****U****C**ACCAC..........................................................................................**G****U****C**A...**C**.**U****G****G****G****C**G................AGCCU..............................................................................UC**G****C****C****U****G****G**G.A.A.**G****A****C**...**G****A****U**..**G****G****A**GGACAACGAC.......................................................................................................**C****C****G****C**...G.**A**.....**G**.**C**....**C****A****G**....**G**AG..A**C**.**C**..**U**.....G...**C****C**.**G**..**U**.**C****A****G****C****C**GUGGUCACACG | |
|  |  | NC\_005296.1/2339917-2340118  | UUAACCCGAGG**U****U****G****U****A**..**C**.**C**..**G**.**G**.UG..**C****C**.**U**.**C****U****C**-...................................GCCG............**A****G****A****G****A**.**G****G****U**..G....AA..**A****C**..**G**..**G**.G..AA.**U**..**G**.**C**.**G****G**.**U**GCGGGGCGAUGCCCC........................................................................................................................................................A**A**UG.....**C****C****G****C**.**A****G****C****U****G****C**C..C.UC..**G****C****A****A****C****U****G****U**GG..**G****C****G****G**.....................................................AUCGGA**G****C****G****U****C****C**UCGCAU.........................................................................................**G****C****C**A...**C**.**U****G****A****C****C**.................AGAUC................................................................................**G****G****U****C****G****G**G.A.A.**G****G****C**...**G****G****A**..**C****G****C**GCCGGAUAU-.......................................................................................................**C****C****G****C**...G.**A**.....**G**.**C**....**C****A****G**....**G**AG..A**C**.**C**..**G**.....G...**C****C**.**G**..**G**.**U****A****C****A****A**GGUGUGCAACU | |
|  |  | NZ\_AAFG02000003.1/65310-65503  | UCAAUAUCGUG**U****U****G****C****U**..**U**.**U**..**G**.**G**.UU..**C****U**.**U**.**U****C****G****A**G..................................GCGA...........C**U****C****G****C****A**.**A****G****C**..G....AA..**G****A**..**G**..**G**.G..AA.**A**..**C**.**U**.**G****G**.**U**GA.....................................................................................................................................................................A**A**AU.....**C****C****G****G**.**U****G****C****U****G****C**C..C.CC..**G****C****A****A****C****U****G****U**AA..**G****C****G****G**C....................................................--AAGC**C****C****U****G****C****C**CGU--..........................................................................................**G****C****C**A...**C**.**U****G****A****C****G**CA...............UCGCU...............................................................................G**C****G****U****U****G****G**G.A.A.**G****G****C**...**G****G****G**..**A****G****G**GGGCGUUUGA......................................................................................................G**C****C****G****C**..GA.**A**.....**G**.**C**....**C****A****G**....**G**AG..A**C**.**C**..**U**.....G...**C****C**.**A**..**G**.**A****G****C****A****C**GACACACGACG | |
|  |  | NC\_003062.1/70220-70444  | ACUAUAUGUGG**U****G****U****U****C**..**A**.**A**..**G**.**G**.UU..**C****U**.**U**.**C****C****G****A**UUC................................GCUA.........GGG**U****U****G****G****G**.**A****G****C**..U....AA..**G****A**C.**G**..**G**.G..AA.**U**..**U**.**C**.**G****G**.**U**GCGUAACGCCAUCACGGCGGAGC................................................................................................................................................A**A**GG.....**C****C****G****A**.**A****A****C****U****G****C**C..C.CC..**G****C****A****A****C****U****G****U**GA..**G****C****G****G**C....................................................GAGCAU**C****G****U****U****C****C**GAUUUGA........................................................................................**G****C****C**A...**C**.**U****G****G****A****G**CC...............AAAAG................................................................................**C****U****C****C****G****G**G.A.A.**G****G****C**..U**G****G****A**..**A****U****A**GAUGUUGUGA......................................................................................................C**C****C****G****C**...A.**A**.....**G**.**U**....**C****A****G**....**G**AG..A**C**.**C**..**U**.....G...**C****C**.**U**..**U**.**G****A****G****C****G**CAAAUGUCCAC | |
|  |  | NC\_003304.1/70421-70645  | ACUAUAUGUGG**U****G****U****U****C**..**A**.**A**..**G**.**G**.UU..**C****U**.**U**.**C****C****G****A**UUC................................GCUA.........GGG**U****U****G****G****G**.**A****G****C**..U....AA..**G****A**C.**G**..**G**.G..AA.**U**..**U**.**C**.**G****G**.**U**GCGUAACGCCAUCACGGCGGAGC................................................................................................................................................A**A**GG.....**C****C****G****A**.**A****A****C****U****G****C**C..C.CC..**G****C****A****A****C****U****G****U**GA..**G****C****G****G**C....................................................GAGCAU**C****G****U****U****C****C**GAUUUGA........................................................................................**G****C****C**A...**C**.**U****G****G****A****G**CC...............AAAAG................................................................................**C****U****C****C****G****G**G.A.A.**G****G****C**..U**G****G****A**..**A****U****A**GAUGUUGUGA......................................................................................................C**C****C****G****C**...A.**A**.....**G**.**U**....**C****A****G**....**G**AG..A**C**.**C**..**U**.....G...**C****C**.**U**..**U**.**G****A****G****C****G**CAAAUGUCCAC | |
|  |  | NZ\_AAAE01000113.1/27784-27584  | AUAUUGAGGAA**C****G****G****C****U**..**U**.**C**..**G**.**G**.U...**U****C**.**C**.**G****G****C****G**...................................UGAU............**G****G****C****C****G**.**G****A****U**..G....AA..**A****A**..**G**..**G**.G..AA.**C**..**G**.**C**.**G****G**.**U**GCGGGGAGAUCUCCCC.......................................................................................................................................................A**A**CU.....**C****C****G****C**.**G****G****C****U****G****C**C..C.CC..**G****C****A****A****C****U****G****U**AG..**G****C****G****G**C....................................................GAGCGA**C****G****A****C****C****G**ACGGC..........................................................................................**G****C****C**A...**C**.**U****G****G****G**-.................AUGU-................................................................................-**C****C****C****G****G**G.A.A.**G****G****C**...**C****G****G**..**U****C****C**GAGCUGCGAC.......................................................................................................**C****C****G****C**...A.**A**.....**G**.**U**....**C****A****G**....**G**AG..A**C**.**C**..**U**.....G...**C****C**.**G**..**G**.**A****G****C****G****A**UCACCCUUUCC | |
|  |  | NC\_002516.1/3305800-3305609  | GUAGCCUUGCC**G****G****U****U****C**..**G**.**A**..**G**.**G**.UU..**C****C**.**U**.**C****G****C****C**G..................................GCGA...........C**G****G****C****G****G**.**G****G****C**..U....AA..**G****A**..**G**..**G**.G..AA.**C**..**G**.**C**.**G****G**.**U**C-.....................................................................................................................................................................G**A**UG.....**C****C****G****C**.**G****G****C****U****G****C**C..C.CC..**G****C****A****A****C****U****G****U**GA..**A****C****G****G**C....................................................-GAUCG**U****U****C****C****C****C**AAU--..........................................................................................**G****C****C**A...**C**.**U****G****C****G**-.................-UGA-................................................................................-**C****G****C****G****G**G.A.A.**G****G****C**...**G****G****G**..**G****A****A**CCGGCGGAGA................................................................................................CGCCAGA**C****C****G****U**...G.**A**.....**G**.**C**....**C****A****G**....**G**AG..A**C**.**C**..**U**.....G...**C****C**.**U**..**C**.**G****U****C****G****A**UCCCGUGGCGC | |
|  |  | NZ\_AAAP01003520.1/1076-875  | GGCCAAGAUCA**U****C****C****C****G**..**A**.**C**..**G**.**G**.U...**U****C**.**C**.**C**---...................................GCAA............---**G****G**.**G****A****U**..C....AA..**A****A**..**G**..**G**.G..AA.**C**..**G**.**C**.**G****G**.**U**GAGGACCGUUCGGUCC.......................................................................................................................................................G**A**UG.....**C****C****G****C**.**G****G****C****U****G****C**C..C.CC..**G****C****A****A****C****U****G****U**GA..**G****C****G****G**CG...................................................AGCCUU**U****U****C****G****C****C**ACCGAC.........................................................................................**G****U****C**A...**C**.**U****G****G****G****C**C................ACCUC................................................................................**G****C****C****C****G****G**G.A.A.**G****A****C**...**G****G****U**..**G****A****G**AAGGCGACGA......................................................................................................C**C****C****G****C**...G.**A**.....**G**.**C**....**C****A****G**....**G**AG..A**C**.**C**..**U**.....G...**C****C**.**G**..**U**.**C****A****G****C****C**GUGGUCACACG | |
|  |  | NC\_002570.2/870579-870768  | AAGCACGCUCA**A****G****C****A****U**..**U**.**A**..**G**.**G**.U...**G****G**.**U**.**U****C****A****A**ACA................................AUCG...........G**U****U****G****A****A**.**U****C****U**..G....AA..**A****A**..**G**..**G**.G..AA.**G**..**C**.**U**.**G****G**.**U**GA.....................................................................................................................................................................A**A**GU.....**C****C****A****G**.**C****A****C****G****G****U**-..C.GC..**G****C****C****A****C****U****G****U**AAU.**A****A****G**-.....................................................GAGCUA**C****A****U****G****U****G**AGGAA..........................................................................................**A****C****C**A...**C**.**U****G****U****C****C**.................-AAA-................................................................................**G****G****A****U****G****G**G.A.A.**G****G****U**..A**C****A****C**..**A****U****G**GAGUGUUGAU.......................................................................................................-**C****U****U**...A.**A**.....**G**.**U**....**C****A****G**....**G**AG..A**C**.**C**..**U**.....G...**C****C**.**U**..**A**.**A****U****G****U****A**UGCACUUGCAC | |
|  |  | NC\_002570.2/466924-466726  | UUUCAUCGUUU**G****G****G****A****A**..**C**.**A**..**G**.**G**.U...**A****C**.**G**.**U****U****A****A**GUCACAU............................GAUA.........GAC**U****U****A****A****U**.**G****U****U**..U....AA..**A****A**..**G**..**G**.G..AA.**U**..**C**.**C**.**G****G**.**U**GC.....................................................................................................................................................................A**A**AU.....**C****C****G****G**.**A****G****C****G****G****U**-..C.CC..**G****C****C****A****C****U****G****U**CA..**U****A****G****C**.....................................................UGAGUU**G****U****A****A****C****G**AUAUU..........................................................................................**G****U****C**A...**C**.**U****G****A****C****C**G................UUCAU...............................................................................U**G****G****U****U****G****G**G.A.A.**G****A****C**...**U****G****U**..**U****G****C**AAUGUUGAC-.......................................................................................................**G****C****U****A**...G.**A**.....**G**.**C**....**C****A****G**....**G**AG..A**C**.**C**..**U**.....G...**C****C**.**U**..**G**.**U****U****C****U****A**ACAGCACUGCU | |
|  |  | NC\_005085.1/1690702-1690517  | AUUCUUGCCAU**U****C****U****U****G**..**A**.**C**..**G**.**G**.UGC.**C****C**.**C**.----...................................AAAA............----**G**.**G****G****U**..G....AA..**A****C**..**G**..**G**.G..AA.**C**..**G**.**C**.**G****G**.**U**GG.....................................................................................................................................................................A**A**UG.....**C****C****G****C**.**G****G****C****U****G****C**C..C.CC..**G****C****A****A****C****U****G****U**AA..**G****C****G****C**.....................................................AGAGUC**C****A****C****G****C****C**UGAC-..........................................................................................**G****C****C**A...**C**.**U****G****G****C****C**CU...............UAGCA...............................................................................A**G****G****U****C****G****G**G.A.A.**G****G****C**...**G****G****C**..**G****C****C**CGGACGGCGA......................................................................................................C**G****C****G****C**...G.**A**.....**G**.**C**....**C****A****G**....**G**AG..A**C**.**C**..**G**.....G...**C****C**.**G**..**U**.**C****A****A****A****C**GUGUCGACGCA | |
|  |  | NZ\_AAAE01000144.1/17615-17822  | ACGGAAGGUGC**G****G****G****C****G**..**A**.**C**..**G**.**G**.UU..**C****C**.**C**.**G****G****A****G**...................................GAAG............**C****U****C****C****G**.**G****G****C**..G....AA..**G****A**..**G**..**G**.G..AA.**U**..**G**.**C**.**G****G**.**U**GCGGGCCUGACAGGCCC......................................................................................................................................................C**A**AG.....**C****C****G****C**.**G****A****C****U****G****C**C..C.CC..**G****C****A****A****C****U****G****U**AG..**G****C****G****G**.....................................................CGAGCG**C****G****U****C****C****C**AUCAUG.........................................................................................**C****C****C**A...**C**.**U****G****G****C****C**CA...............GGACA................................................................................**G****G****C****C****G****G**G.A.A.**G****G****G**..C**G****G****G**..**A****C****A**AGCCCAGAC-.......................................................................................................**C****C****G****C**...G.**A**.....**G**.**U**....**C****A****G**....**G**AG..A**C**.**C**..**U**.....G...**C****C**.**G**..**U**.**C****G****A****C****G**GACCUGUAACA | |
|  |  | NZ\_AAIT01000002.1/172004-172217  | CCUAUAUGUAG**G****C****A****U****C**..**A**.**C**..**G**.**G**.UU..**C****U**.**U**.**U****C****G****G**G..................................UCGA......UCCGAC**C****C****G****A****A**.**A****G****C**..G....AA..**G****A**..**G**..**G**.G..AA.**G**..**C**.**C**.**G****G**.**U**GCGCCUUUCGGGGC.........................................................................................................................................................A**A**GG.....**C****C****G****G**.**C****G****C****U****G****C**C..C.CC..**G****C****A****A****C****U****G****U**AA..**G****C****A****G**C....................................................GAGCCU**G****C****G****C****C****G**GAUCAC.........................................................................................**G****C****C**A...**C**.**U****G****G****U****G**CC...............GCACG...............................................................................G**C****A****U****U****G****G**G.A.A.**G****G****C**...**C****G****G**..**C****A****A**GGGCAGCGAC.......................................................................................................**C****U****G****C**...G.**A**.....**G**.**C**....**C****A****G**....**G**AG..A**C**.**C**..**U**.....G...**C****C**.**G**..**U**.**G****A****U****G****A**UGACGAUGAAC | |
|  |  | NZ\_AAED02000003.1/511627-511424  | CUAAAUAUAGC**C****C****A****U****C**..**A**.**C**..**G**.**G**.U...**G****C**.**U**.**G****U****C****G**A..................................GUCG.....UUCGGGU**C****G****A****G****A**.**G****C****U**..U....AA..**G****A**..**G**..**G**.G..AA.**G**..**C**.**C**.**G****G**.**U**GU.....................................................................................................................................................................G**A**GG.....**C****C****G****G**.**C****G****C****U****G****C**C..C.CC..**G****C****A****A****C****U****G****U**AA..**G****C****A****G**C....................................................GAGCUU**G****U****G****U****C****G**CGUAUU.........................................................................................**G****C****C**A...**C**.**U****G****G****U****G**CUCC.............UAGAG................................................................................**C****G****C****U****G****G**G.A.A.**G****G****C**..C**U****G****A**..**C****A****G**AAGCGAUGAC.......................................................................................................**C****U****G****C**...A.**A**.....**G**.**C**....**C****A****G**....**G**AG..A**C**.**C**..**U**.....G...**C****C**.**G**..**U**.**G****A****U****G****A**GCAUUGAACGU | |
|  |  | NZ\_AAAP01002012.1/63-265  | GCACAUCCUGG**A****A****G****U****G**..**A**.**C**..**G**.**G**.U...**U****C**.**C**.**C**---...................................UUCG............---**G****G**.**G****A****U**..C....AA..**A****A**..**G**..**G**.G..AA.**C**..**G**.**C**.**G****G**.**U**GAGGACUUUCGGGUCC.......................................................................................................................................................G**A**UG.....**C****C****G****C**.**G****G****C****U****G****C**C..C.CC..**G****C****A****A****C****U****G****U**AA..**G****C****G****G**CG...................................................AGCCUU**U****U****C****A****C****C**ACCGAU.........................................................................................**G****U****C**A...**C**.**U****G****G****A****U**G................AGCCU...............................................................................C**A****U****C****C****G****G**G.A.A.**G****A****C**...**G****G****U**..**G****A****G**ACGGCGACGA......................................................................................................C**C****C****G****C**...G.**A**.....**G**.**C**....**C****A****G**....**G**AG..A**C**.**C**..**U**.....G...**C****C**.**G**..**U**.**C****A****G****C****C**GUGGUCACACG | |
|  |  | NC\_003318.1/973126-972913  | ACCAUAUCUUG**U****G****U****U****C**..**G**.**A**..**G**.**G**.UU..**C****U**.**U**.**U****C****G****A**UUC................................GACG..........AG**U****C****G****G****G**.**A****G****C**..U....AA..**G****A**C.**G**..**G**.G..AA.**U**..**C**.**C**.**G****G**.**U**GCGCUUGCCCAUGGUGGGCGGGC................................................................................................................................................A**A**UG.....**C****C****G****G**.**A****G****C****U****G****C**C..C.CC..**G****C****A****A****C****U****G****U**AA..**G****C****G****G**CG...................................................AGCUUU**G****C****G****C****C****C**CAU--..........................................................................................**G****C****C**A...**C**.**U****G****G****C**-.................GAAA-................................................................................-**G****C****C****G****G**G.A.A.**G****G****C**...**G****G****G**..**U****G****G**AAGCGUUGAG.......................................................................................................**C****C****G****U**...G.**A**.....**G**.**C**....**C****A****G**....**G**AG..A**C**.**C**..**U**.....G...**C****C**.**U**..**U**.**G****A****G****C****G**UGAACGUCCAC | |
|  |  | NC\_004311.2/297512-297725  | ACCAUAUCUUG**U****G****U****U****C**..**G**.**A**..**G**.**G**.UU..**C****U**.**U**.**U****C****G****A**UUC................................GACG..........AG**U****C****G****G****G**.**A****G****C**..U....AA..**G****A**C.**G**..**G**.G..AA.**U**..**C**.**C**.**G****G**.**U**GCGCUUGCCCAUGGUGGGCGGGC................................................................................................................................................A**A**UG.....**C****C****G****G**.**A****G****C****U****G****C**C..C.CC..**G****C****A****A****C****U****G****U**AA..**G****C****G****G**CG...................................................AGCUUU**G****C****G****C****C****C**CAU--..........................................................................................**G****C****C**A...**C**.**U****G****G****C**-.................GAAA-................................................................................-**G****C****C****G****G**G.A.A.**G****G****C**...**G****G****G**..**U****G****G**AAGCGUUGAG.......................................................................................................**C****C****G****U**...G.**A**.....**G**.**C**....**C****A****G**....**G**AG..A**C**.**C**..**U**.....G...**C****C**.**U**..**U**.**G****A****G****C****G**UGAACGUCCAC | |
|  |  | NC\_006933.1/874132-873919  | ACCAUAUCUUG**U****G****U****U****C**..**G**.**A**..**G**.**G**.UU..**C****U**.**U**.**U****C****G****A**UUC................................GACG..........AG**U****C****G****G****G**.**A****G****C**..U....AA..**G****A**C.**G**..**G**.G..AA.**U**..**C**.**C**.**G****G**.**U**GCGCUUGCCCAUGGUGGGCGGGC................................................................................................................................................A**A**UG.....**C****C****G****G**.**A****G****C****U****G****C**C..C.CC..**G****C****A****A****C****U****G****U**AA..**G****C****G****G**CG...................................................AGCUUU**G****C****G****C****C****C**CAU--..........................................................................................**G****C****C**A...**C**.**U****G****G****C**-.................GAAA-................................................................................-**G****C****C****G****G**G.A.A.**G****G****C**...**G****G****G**..**U****G****G**AAGCGUUGAG.......................................................................................................**C****C****G****U**...G.**A**.....**G**.**C**....**C****A****G**....**G**AG..A**C**.**C**..**U**.....G...**C****C**.**U**..**U**.**G****A****G****C****G**UGAACGUCCAC | |
|  |  | NC\_002570.2/1661058-1661239  | UCGUAUAUCGC**G****C****U****G****A**..**A**.**G**..**G**.**G**.U...**C****G**.**U**.**U****C****A****A**...................................-UGU............**U****U****G****A****G**.**C****G****U**..G....AA..**A****A**..**G**..**G**.G..AA.**G**..**U**.**C**.**G****G**.**U**GA.....................................................................................................................................................................A**A**AU.....**C****C****G****A**.**C****A****C****G****G****U**-..C.CC..**G****C****C****A****C****U****G****U**AA..**A****U****G**-.....................................................GGAGAG**G****C****U****U****G****C**AAGA-..........................................................................................**U****C****C**A...**C**.**U****G****U****C**-.................UAGC-................................................................................-**G****A****C****G****G**G.A.A.**G****G****G**..G**G****C****A**..**A****G****U**ACUCGAUGAA.......................................................................................................-**C****A****U**...A.**A**.....**G**.**U**....**C****A****G**....**G**AG..A**C**.**C**..**U**.....G...**C****C**.**U**..**U**.**U****C****A****G****U**UUGAGUGUGUA | |
|  |  | NZ\_AADT03000018.1/23328-23505  | CAAAUUUAAUA**A****A****C****A****A**..**C**.**A**..**G**.**G**.UG..**C****C**.**U**.----...................................GCA-............----**G**.**G****G****U**..U....AA..**A****A**..**G**..**G**.G..AA.**U**..**C**.**A**.**G****G**.**U**GU.....................................................................................................................................................................A**A**AU.....**C****C****U****G**.**A****G****C****G****G****U**-..C.GC..**G****C****C****A****C****U****G****U**AA..**C****C****G****G**G....................................................GAGUUU**U****U****C****C****U****C**AGAG-..........................................................................................**G****C****C**A...**C**.**U****G****G****U**-.................GAUCU................................................................................-**A****C****U****G****G**G.A.A.**G****G****C**...**G****A****G**..**G****A****A**AAGUAAUGAG.......................................................................................................**C****C****G****G**...G.**A**.....**G**.**C**....**C****A****G**....**G**AA..A**C**.**C**..**U**.....G...**C****C**.**U**..**G**.**U****U****G****G****U**UCAACCCUCGC | |
|  |  | NZ\_AAAW03000009.1/14789-14611  | CAACCGAAUAG**U****A****U****U****C**..**A**.**A**..**G**.**G**.U...**U****C**.**C**.**U****C**--...................................UUUA............--**G****A****G**.**G****A****A**..G....AA..**A****A**..**G**..**G**.G..AA.**G**..**C**.**C**.**G****G**.**U**GA.....................................................................................................................................................................A**A**AU.....**C****C****G****G**.**C****G****C****G****G****U**-..C.CC..**G****C****C****A****C****U****G****U**GAA.**C****C****A**-.....................................................CGAGUG**C****C****U****C****U****U**AAGG-..........................................................................................**C****C****C**A...**C**.**U****G****G****G**-.................AUGAG................................................................................-**C****C****U****G****G**G.A.A.**G****G****G**...**A****A****G**..**A****G****G**CAUAAAGAC-.......................................................................................................-**U****G****G**...A.**A**.....**G**.**C**....**C****A****G**....**G**AG..A**C**.**C**..**U**.....G...**C****C**.**U**..**U**.**G****A****A****C****A**UUGCGCUACGU | |
|  |  | NZ\_AABQ07000001.1/584146-583954  | GUAGCCUUGCC**G****G****U****U****C**..**G**.**A**..**G**.**G**.UU..**C****C**.**U**.**C****G****C****C**G..................................GCGA...........C**G****G****C****G****G**.**G****G****C**..U....AA..**G****A**..**G**..**G**.G..AA.**C**..**G**.**C**.**G****G**.**U**C-.....................................................................................................................................................................G**A**UG.....**C****C****G****C**.**G****G****C****U****G****C**C..C.CC..**G****C****A****A****C****U****G****U**GA..**A****C****G****G**C....................................................-GAUCG**U****U****C****C****C****C**AAU--..........................................................................................**G****C****C**A...**C**.**U****G****C****G**-.................-UGA-................................................................................-**C****G****C****G****G**G.A.A.**G****G****C**...**G****G****G**..**G****A****A**CCCGGCGGAG...............................................................................................ACGCCAGA**C****C****G****U**...G.**A**.....**G**.**C**....**C****A****G**....**G**AG..A**C**.**C**..**U**.....G...**C****C**.**U**..**C**.**G****U****C****G****A**UCCCGUGGCGC | |
|  |  | NZ\_AADT03000003.1/96733-96933  | AUAUGAAAAAA**A****U****G****G****A**..**C**.**A**..**G**.**G**.UG..**C****C**.**C**.**C****C**--...................................GGGA............--**G****G****G**.**G****G****A**..G....AA..**U****A**..**G**..**G**.G..AA.**G**U.**C**.**C**.**G****G**.**U**AACCGCCUUUUCAGGCGG.....................................................................................................................................................C**G**AU.....**C****C****G****G**.**C****G****C****G****G****U**-..C.CC..**G****C****C****A****C****U****G****U**GA..**G****C****G****U**.....................................................GGAGUU**G****G****C****G****A****U**AUAAC..........................................................................................**G****C****C**A...**C**.**U****G****G****G****A**.................UAGU-................................................................................**U****C****C****C****G****G**G.A.A.**G****G****C**...**A****U****C**..**G****C****U**AAACAAGGAA.......................................................................................................**G****C****G****C**...G.**A**.....**G**.**C**....**C****A****G**....**G**AG..A**C**.**C**..**U**.....G...**C****C**.**U**..**G**.**U****C****C****A****U**AUGACCUCUCA | |
|  |  | NZ\_AAAG02000003.1/62343-62564  | GUAAGCUUUGA**G****G****A****U****C**..**G**.**U**..**G**.**G**.UU..**C****U**.**U**.**U****G****G****C**ACC................................GUUU....UGUCGGGU**G****C****C****G****G**.**A****G****C**..U....AA..**G****A**..**G**..**G**.G..AA.**U**..**C**.**C**.**G****G**.**U**GAGCCGCGUUUUGGC........................................................................................................................................................G**A**UG.....**C****C****G****G**.**A****G****C****U****G****C**C..C.CC..**G****C****A****A****C****U****G****U**AA..**G****C****G****G**.....................................................CGAGCC**G****C****U****G****U****C**AAGACGU........................................................................................**G****C****C**A...**C**.**U****G****C****C****C**CCG..............GCAAG..............................................................................GG**G****G****G****U****G****G**G.A.A.**G****G****C**.CA**G****A****C**..**A****G****C**GGCGACGAC-.......................................................................................................**C****C****G****U**...A.**A**.....**G**.**C**....**C****A****G**....**G**AG..A**C**.**C**..**U**.....G...**C****C**.**G**..**C**.**G****A****U****G****U**CAUAACCUCCC | |
|  |  | NC\_005296.1/798739-798524  | UUUGAUCGCGC**C****C****G****U****U**..**C**.**A**..**G**.**G**.U...**G****U**.**G**.**C****C****G****G**GA.................................AUCG...........U**C****C****G****G****C**.**A****C**-..-....--..-**A**..**G**..**G**.G..AA.**G**..**C**.**C**.**G****G**.**U**GCGGGCCCAAGCUUAUGGCCGCAAACCC...........................................................................................................................................A**A**AU.....**C****C****G****G**.**C****G****C****U****G****C**G..C.CC..**G****C****A****A****C****U****G****U**GA..**G****C****G****G**U....................................................GAGCGA**U****C****C****U****U****C**AAUCG..........................................................................................**G****C****C**A...**C**.**U****G****G****G****C**A................GCACU...............................................................................U**G****C****C****C****G****G**G.A.A.**G****G****C**...**G****A****A**..**G****G****A**UUGCGACGAC.......................................................................................................**C****C****G****U**...G.**A**.....**G**.**C**....**C****A****G**....**G**AG..A**C**.**C**..**G**.....G...**C****C**.**U**..**G**.**A****G****U****A****C**GUCAUCUUCCA | |
|  |  | NZ\_AAED02000002.1/352623-352411  | UAUUCAAUGCA**U****G****U****C****U**..**G**.**U**..**G**.**G**.UU..**C****U**.**C**.**C****G****G****G**GCG................................GUUU......UGCCGU**U****C****C****G****G**.**A****G****C**..U....AA..**G****A**..**G**..**G**.G..AA.**G**..**C**.**C**.**G****G**.**U**GCGCGAUGUUGC...........................................................................................................................................................A**A**AG.....**C****C****G****G**.**C****G****C****U****G****C**C..C.CC..**G****C****A****A****C****U****G****U**AA..**A****C****G****G**C....................................................GAGCCG**A****A****A****U****C****C**ACCGGU.........................................................................................**G****U****C**A...**C**.**U****G****A****G****G**UG...............AUAAG................................................................................**C****C****U****U****G****G**G.A.A.**G****A****C**...**G****G****A**..**U****U****G**AAGCGAAGAC.......................................................................................................**C****C****G****C**...G.**A**.....**G**.**C**....**C****A****G**....**G**AG..A**C**.**C**..**U**.....G...**C****C**.**A**..**C**.**G****G****C****A****A**GAAGAAACGUC | |
|  |  | NZ\_AAIT01000007.1/203053-203263  | CCAUGAGGGCC**G****A****G****C****U**..**A**.**C**..**G**.**G**.U...**U****C**.**C**.**G****G****C****G**G..................................CGUG...........C**C****U****C****C****G**.**G****A****U**..U....AA..**A****A**..**G**..**G**.G..AA.**U**C.**C**.**C**.**G****G**.**U**GCGCCUGACCUGUUCAGGC....................................................................................................................................................A**A**UG.....**C****C****G****G**.**A****G****C****U****G****C**C..C.CC..**G****C****A****A****C****U****G****U**AU..**G****C****G****G**U....................................................GAGUGA**C****G****G****U****C****G**AUCAU..........................................................................................**G****C****C**A...**C**.**U****G****G****C****G**C................GAAUG................................................................................**C****G****C****C****G****G**G.A.A.**G****G****C**...**C****G****G**..**C****C****C**GAACGAUGAC.......................................................................................................**C****C****G****C**...A.**A**.....**G**.**C**....**C****A****G**....**G**AG..A**C**.**C**..**U**.....G...**C****C**.**G**..**C**.**A****G****C****A****A**UCACCCAUUUC | |
|  |  | NZ\_AAIT01000007.1/220883-220683  | AUUUUACCGUC**A****G****A****U****C**..**A**.**A**..**G**.**G**.UG..**C****C**.**G**.**G****G****G**-...................................AAAU............-**C****U****C****C**.**G****G****G**..U....AA..**A****A**..**G**..**G**.G..AA.**U**..**U**.**C**.**G****G**.**U**GAGGCUGUCGCC...........................................................................................................................................................A**A**AU.....**C****C****G****A**.**A****G****C****U****G****C**C..C.CC..**G****C****A****A****C****U****G****U**AG..**G****C****G****G**.....................................................AGAGCC**U****G****U****C****C****G**ACAU-..........................................................................................**G****C****C**A...**C**.**U****G****G****C****G**CGGA.............UUUCG................................................................................**C****G****C****C****G****G**G.A.A.**A****G****C**...**U****G****G**..**A****C****C**AGGUAAAGAU.......................................................................................................**C****C****G****C**...A.**A**.....**G**.**U**....**C****A****G**....**G**AA..A**C**.**C**..**U**.....G...**C****C**.**U**..**G**.**G****A****U****C****A**AUCACCUGUUC | |
|  |  | NZ\_AAFG02000004.1/314534-314749  | CCGCAUGAGAG**G****G****G****C****G**..**A**.**U**..**G**.**G**.U...**U****C**.**C**.**C****C****G****A**CGCCU..............................AAGG..........CC**G****G****U****G****G**.**G****A****U**..G....AA..**A****A**..**G**..**G**.G..AA.**U**..**U**.**C**.**G****G**.**U**GCGGAGGAGCCAAAGGGCGCC..................................................................................................................................................A**A**AU.....**C****C****G****A**.**A****A****C****U****G****C**C..C.CC..**G****C****A****A****C****U****G****U**AA..**G****C****G****G**.....................................................CGAGCG**C****C****C****C****G****U**CACAG..........................................................................................**C****C****C**A...**C**.**U****G****A****U****C**C................UCAUG................................................................................**G****A****U****C****G****G**G.A.A.**G****G****G**...**A****G****G**..**G****A****C**GCGCAAUAAC.......................................................................................................**C****C****G****C**...A.**A**.....**G**.**U**....**C****A****G**....**G**AG..A**C**.**C**..**U**.....G...**C****C**.**A**..**U**.**C****G****C****G****A**CUUUUUAACCA | |
|  |  | NC\_003078.1/66246-66476  | ACAUUAACUGG**G****A****C****C****G**..**A**.**C**..**G**.**G**.U...**U****C**.**C**.**C****C****U****A**CCC................................GUGA........GGUG**G****A****G****G****G**.**G****A****U**..U....AA..**U****A**..**G**..**G**.G..AA.**C**..**A**.**C**.**G****G**.**U**GCGGACGACCCAAGAGGGACC..................................................................................................................................................A**A**AA.....**C****C****G****U**.**G****G****C****U****G****C**C..C.CC..**G****C****A****A****C****U****G****U**AA..**G****C****G****G**A....................................................UCGUCG**U****U****C****A****U****C**CUUGUGGCGCCAAGGCGCCA...........................................................................**G****C****C**A...**C**.**U****G****C****G****C**.................GCGUU................................................................................**G****C****G****C****G****G**G.A.A.**G****G****C**..A**G****A****U**..**G****A****G**CGACUCUGU-.......................................................................................................**C****C****G****U**...G.**A**.....**G**.**C**....**C****A****G**....**G**AG..A**C**.**C**..**U**.....G...**C****C**.**G**..**U**.**C****A****A****A****U**CGAUCCAACGU | |
|  |  | NZ\_AAEI01000008.1/333950-333757  | UUACCCUUGCG**C****G****C****U****G**..**A**.**U**..**G**.**G**.U...**U****C**.**C**.**C****G**--...................................GAAA............--**C****G****G**.**G****A****U**..C....AA..**A****A**..**G**..**G**.G..AA.**C**..**G**.**C**.**A****G**.**G**AGAGGCAGCGCCUC.........................................................................................................................................................G**A**AU.....**C****U****G****C**.**G****G****C****U****G****C**C..C.CC..**G****C****A****A****C****U****G****U**GA..**G****C****G****G**.....................................................CGAAUC**C****G****U****G****C****C**ACUGCAU........................................................................................**G****C****C**A...**C**.**U****G****G**--.................GAAA-................................................................................--**C****C****G****G**G.A.A.**G****G****C**..C**G****G****C**..**A****C****G**AAUCAGGAC-.......................................................................................................**C****C****G****C**...G.**A**.....**G**.**C**....**C****A****G**....**G**AG..A**C**.**C**..**U**.....G...**C****C**.**A**..**U**.**C****G****A****C****C**CGAGGUCCAAG | |
|  |  | NC\_003869.1/396055-396295  | GAAUAUAAAAA**G****C****C****U****U**..**A**.**U**..**G**.**G**.U...**C****C**.**C**.----...................................GUGA...........U----**G**.**G****G****U**..U....AA..**A****A**..**G**..**G**.G..AA.**G**A.**C**.**G**.**G****G**.**U**GA.....................................................................................................................................................................G**A**AU.....**C****C****C****G**.**C****G****C****A****G****C**C..C.CC..**G****C****U****A****C****U****G****U**GA..**G****G****G****A**.....................................................GGACGA**A****G****C****C****C****U**AGUAA..........................................................................................**G****C****C**A...**C**.**U****G****U****C****C**GGCAC............UCAAC..........................UGAGCGCGUUAGUAAGGAGAAAAGAGGGAGAGAAAUUGCGUUCAGUUGAGUGCC**G****G****A****U****G****G**G.A.A.**G****G****C**...**A****G****G**..**G****U****G**GAGGAUGAG-.......................................................................................................**U****C****C****C**...G.**A**.....**G**.**C**....**C****A****G**....**G**AG..A**C**.**C**..**U**.....G...**C****C**.**A**..**U**.**A****A****G****G****U**UUUUAAAAGUU | |
|  |  | NC\_003317.1/679010-679238  | UCGCAAUUUUC**A****G****G****A****G**..**A**.**C**..**G**.**G**.U...**U****C**.**C**.**G****C****C**-...................................AUUG...........C-**G****G****C****G**.**G****A****U**..G....AA..**A****A**..**G**..**G**.G..AA.**C**..**A**.**C**.**G****G**.**U**GAAGCCAUAGGGCU.........................................................................................................................................................G**A**AA.....**C****C****G****A**.**G****A****C****U****G****C**C..C.CC..**G****C****A****A****C****U****G****U**AA..**C****C****G****G**.....................................................AGAGCU**A****U****C****C****U****C**CACAGGCCGCGCAAGCGGCCAAA........................................................................**G****C****C**A...**C**.**U****G****A****A****A**GCAGCA...........AUAUG............................................................................CUGC**A****A****U****C****G****G**G.A.A.**G****G****C**..G**G****A****G**..**G****C****A**AAGCGAAGAC.......................................................................................................**C****C****G****G**...A.**A**.....**G**.**U**....**C****A****G**....**G**AG..A**C**.**C**..**U**.....G...**C****C**.**G**..**U**.**A****U****C****C****G**GUCACCCAUGC | |
|  |  | NC\_004310.3/1309296-1309068  | UCGCAAUUUUC**A****G****G****A****G**..**A**.**C**..**G**.**G**.U...**U****C**.**C**.**G****C****C**-...................................AUUG...........C-**G****G****C****G**.**G****A****U**..G....AA..**A****A**..**G**..**G**.G..AA.**C**..**A**.**C**.**G****G**.**U**GAAGCCAUAGGGCU.........................................................................................................................................................G**A**AA.....**C****C****G****A**.**G****A****C****U****G****C**C..C.CC..**G****C****A****A****C****U****G****U**AA..**C****C****G****G**.....................................................AGAGCU**A****U****C****C****U****C**CACAGGCCGCGCAAGCGGCCAAA........................................................................**G****C****C**A...**C**.**U****G****A****A****A**GCAGCA...........AUAUG............................................................................CUGC**A****A****U****C****G****G**G.A.A.**G****G****C**..G**G****A****G**..**G****C****A**AAGCGAAGAC.......................................................................................................**C****C****G****G**...A.**A**.....**G**.**U**....**C****A****G**....**G**AG..A**C**.**C**..**U**.....G...**C****C**.**G**..**U**.**A****U****C****C****G**GUCACCCAUGC | |
|  |  | NC\_006932.1/1327262-1327034  | UCGCAAUUUUC**A****G****G****A****G**..**A**.**C**..**G**.**G**.U...**U****C**.**C**.**G****C****C**-...................................AUUG...........C-**G****G****C****G**.**G****A****U**..G....AA..**A****A**..**G**..**G**.G..AA.**C**..**A**.**C**.**G****G**.**U**GAAGCCAUAGGGCU.........................................................................................................................................................G**A**AA.....**C****C****G****A**.**G****A****C****U****G****C**C..C.CC..**G****C****A****A****C****U****G****U**AA..**C****C****G****G**.....................................................AGAGCU**A****U****C****C****U****C**CACAGGCCGCGCAAGCGGCCAAA........................................................................**G****C****C**A...**C**.**U****G****A****A****A**GCAGCA...........AUAUG............................................................................CUGC**A****A****U****C****G****G**G.A.A.**G****G****C**..G**G****A****G**..**G****C****A**AAGCGAAGAC.......................................................................................................**C****C****G****G**...A.**A**.....**G**.**U**....**C****A****G**....**G**AG..A**C**.**C**..**U**.....G...**C****C**.**G**..**U**.**A****U****C****C****G**GUCACCCAUGC | |
|  |  | NZ\_AAAP01003877.1/22278-22051  | AUUAUAGUGCC**G****G****G****U****G**..**A**.**U**..**G**.**G**.UU..**C****C**.**C**.**C****G****C****G**UCC................................GCUU........AGGG**C****A****A****G****G**.**G****G****U**..G....AA..**A****A**U.**G**..**G**.G..AA.**C**..**G**.**U**.**G****G**.**U**GCGGCGCCCGAAAAGGGGGCC..................................................................................................................................................A**A**UU.....**C****C****G****C**.**G****A****C****U****G****C**C..C.CC..**G****C****A****A****C****U****G****U**AG..**G****C****G****G**.....................................................AGAGGU**U****C****C****G****G****C**CGUUAGAGAC.....................................................................................**G****C****C**A...**C**.**U****G****G****C****C**C................UGAAA.............................................................................UGG**G****G****C****U****G****G**G.A.A.**G****G****C**UAU**G****C****C**..**G****G****A**CCCAACGAC-.......................................................................................................**C****C****G****C**...G.**A**.....**G**.**U**....**C****A****G**....**G**AG..A**C**.**C**..**U**.....G...**C****C**.**A**..**U**.**C****A****U****C****G**AGAGUCGCUUG | |
|  |  | NZ\_AAGG01000005.1/2185-2371  | CGGCUGCUUCA**C****G****U****C****A**..**C**.**G**..**G**.**G**.UU..**G****C**.**C**.**G****C**--...................................GUAA..........CA--**C****C****G**.**G****C****C**..G....AA..**G****A**..**G**..**G**.G..AA.**G**..**G**.**G**.**A****G**.**U**GC.....................................................................................................................................................................G**A**UG.....**C****U****C****C**.**C****G****C****U****G****C**C..C.CC..**G****C****A****A****C****U****G****U**GA..**C****C****G****G**G....................................................GAGCGA**C****C****C****U****G****C**CAAGG..........................................................................................**G****C****C**A...**C**.**U****G****G****U**-.................-UUCG................................................................................-**A****C****C****G****G**G.A.A.**G****G****C**..G**G****C****G**..**G****G****G**AAGCGACGAU.......................................................................................................**C****C****G****G**...G.**A**.....**G**.**C**....**C****A****G**....**G**AG..A**C**.**C**..**U**.....G...**C****C**.**C**..**G**.**A****G****A****C****C**GUCGCUUGAGC | |
|  |  | NC\_003062.1/877424-877652  | GGAUAACAUGU**C****C****G****U****G**..**A**.**U**..**G**.**G**.UU..**C****C**.**U**.**U****C****C****G**G..................................GCGU..........UC**C****G****G****A****A**.**G****G****U**..G....AA..**A****A**..**G**..**G**.G..AA.**C**..**A**.**C**.**G****A**.**U**AGGGACAAAUCC...........................................................................................................................................................U**C**AU.....**U****C****G****U**.**G****G****C****U****G****C**C..C.CC..**G****C****A****A****C****U****G****U**GA..**G****C****G****G**.....................................................AGAGCC**U****G****A****A****A****C**GAAAU..........................................................................................**G****C****C**A...**C**.**U****G****G****C****A**AGCCAUCUCGCCUCCA.UCAAG.....................................................................GGGGAAGGCAA**U****G****C****C****G****G**G.A.A.**G****G****U**...**G****U****U**..**U****C****A**GGUUUUGAC-.......................................................................................................**C****C****G****U**...A.**A**.....**G**.**C**....**C****A****G**....**G**AG..A**C**.**C**..**U**.....G...**C****C**.**A**..**U**.**C****A****C****G****G**AAAUAUCCAUG | |
|  |  | NC\_003304.1/877625-877853  | GGAUAACAUGU**C****C****G****U****G**..**A**.**U**..**G**.**G**.UU..**C****C**.**U**.**U****C****C****G**G..................................GCGU..........UC**C****G****G****A****A**.**G****G****U**..G....AA..**A****A**..**G**..**G**.G..AA.**C**..**A**.**C**.**G****A**.**U**AGGGACAAAUCC...........................................................................................................................................................U**C**AU.....**U****C****G****U**.**G****G****C****U****G****C**C..C.CC..**G****C****A****A****C****U****G****U**GA..**G****C****G****G**.....................................................AGAGCC**U****G****A****A****A****C**GAAAU..........................................................................................**G****C****C**A...**C**.**U****G****G****C****A**AGCCAUCUCGCCUCCA.UCAAG.....................................................................GGGGAAGGCAA**U****G****C****C****G****G**G.A.A.**G****G****U**...**G****U****U**..**U****C****A**GGUUUUGAC-.......................................................................................................**C****C****G****U**...A.**A**.....**G**.**C**....**C****A****G**....**G**AG..A**C**.**C**..**U**.....G...**C****C**.**A**..**U**.**C****A****C****G****G**AAAUAUCCAUG | |
|  |  | NZ\_AAAW03000045.1/21996-22179  | UUAAAUAUUCA**G****U****U****U****U**..**U**.**A**..**G**.**G**.UG..**C****C**.**U**.**C****U****G****A**...................................UUUA............**U****C****G****G****A**.**G****G****A**..U....AA..**U****A**..**G**..**G**.G..AA.**C**..**C**.**A**.**G****G**.**U**GA.....................................................................................................................................................................A**A**UU.....**C****C****U****G**.**G****A****C****G****G****U**-..C.GC..**G****C****C****A****C****U****G****U**AU..**G****C****G****G**.....................................................AGAGCU**U****C****U****U****U****U**AUAA-..........................................................................................-**C****C**A...**C**.**U****G****G****U**-.................UUUA-................................................................................-**U****C****C****G****G**G.A.A.**G****G**-..A**G****A****A**..**A****G****A**AAGCAAAGAC.......................................................................................................**C****C****G****C**...A.**A**.....**G**.**U**....**C****A****G**....**G**AA..A**C**.**C**..**U**.....G...**C****C**.**U**..**A**.**A****U****A****C****U**GGUUGUCUACG | |
|  |  | NZ\_AAAG02000002.1/607022-606806  | GGCUAGCCUGC**G****G****C****U****G**..**A**.**C**..**G**.**G**.U...**U****C**.**C**.**C****C****C****C**AAGC...............................GCAA.........GCC**G****A****A****G****G**.**G****A****U**..U....AA..**A****A**..**G**..**G**.G..AA.**C**..**A**.**C**.**G****G**.**U**GCAGGCGAAGGAUUUGUCC....................................................................................................................................................G**A**AA.....**C****C****G****U**.**G****G****C****U****G****C**C..C.CC..**G****C****A****A****C****U****G****U**GA..**G****C****G****G**C....................................................GAGCCU**U****C****C****U****C****C**GAUGAAA........................................................................................**A****C****C**A...**C**.**U****G****G****G****G**C................GCGUG................................................................................**C****C****U****U****G****G**G.A.A.**G****G****G**...**G****G****A**..**U****G****A**AGGCGAUGAC.......................................................................................................**C****C****G****U**...G.**A**.....**G**.**C**....**C****A****G**....**G**AG..A**C**.**C**..**U**.....G...**C****C**.**G**..**U**.**C****C****G****A****C**CCUAAUCCAUG | |
|  |  | NZ\_AAAE01000116.1/15838-16017  | GCUUUGUGGCA**G****G****G****G****U**..**C**.**A**..**G**.**G**.UG..**C****C**.**G**.**C****C**--...................................UUCG............--**G****G****C**.**G****G****A**..G....AA..**U****C**..**G**..**G**.G..AA.**G**..**C**.**C**.**G****G**.**U**GG.....................................................................................................................................................................A**A**AU.....**C****C****G****G**.**C****G****C****G****G****G**-..C.CC..**G****C****C****G****C****U****G****U**GA..**C****G****G****G**.....................................................GAUGCU**C****C****G****G****G****C**AAGAG..........................................................................................**G****C****C**A...**C**.**C****G****G**--.................-UUCG................................................................................--**C****C****G****G**G.A.A.**G****G****C**...**G****C****C**..**C****G****G**CGGCAGAUGA.......................................................................................................**A****C****C****G**...G.**A**.....**G**.**C**....**C****A****G**....**A**AG..A**C**.**C**..**G**.....G...**C****C**.**U**..**G**.**A****C****G****C****A**GAGGUUCCCGC | |
|  |  | NC\_006510.1/935015-935213  | AUAUCGUUGCG**C****C****A****A****G**..**C**.**A**AU**G**.**G**.U...**G****U**.**C**.**C**---...................................AUCA............---**G****G**.**A****C****U**..U....AA..**U****A**..**G**..**G**.G..AA.**U**..**C**.**C**.**G****G**.**U**GU.....................................................................................................................................................................G**A**AU.....**C****C****G****G**.**A****A****C****U****G****C**C..C.CC..**G****C****A****A****C****U****G****U**AU..**G****U****G****C**.....................................................GGACGA**A****A****U****G****G****G**AUG--..........................................................................................**G****C****C**A...**C**.**U****G****G****C****G**GC...............AAGAG...................................................................CCAUUCGGCGCGC**C****G****C****U****G****G**G.A.A.**G****G****C**...**C****C****C**..**A****A****A**GUAGGACGAU.......................................................................................................**G****C****A****C**...G.**A**.....**G**.**U**....**C****A****G**....**G**AG..A**C**.**C**..**U**.....G...**C****C**U**U**..**G**.**C****U****U****G****G**AACGUUUCAUG | |
|  |  | NC\_003366.1/1431311-1431132  | UAAUAUUUUAU**A****U****U****U****U**..**U**.**A**..**G**.**G**.U...**U****U**.**G**.----...................................AUUU............----**U**.**A****A****U**..U....AA..**A****A**..**G**..**G**.G..AA.**A**..**G**.**U**.**G****G**.**U**UA.....................................................................................................................................................................A**A**GU.....**C****C****A****C**.**U****A****C****A****G****C**C..C.CC..**G****C****U****A****C****U****G****U**GA..**U****A****G****G**.....................................................AUACAA**G****U****U****U****C****U**AUUUG..........................................................................................**A****C****C**A...**C**.**U****G****A****U****U**.................AUAUA................................................................................**A****A****U****U****G****G**G.A.A.**G****G****G**...**A****G****A**..**A****A****U**GAGGAUAAG-.......................................................................................................**C****C****U****U**...A.**A**.....**G**.**U**....**C****A****G**....**G**AU..A**C**.**C**..**U**.....G...**C****C**.**U**..**A**.**A****A****G****A****U**CAUGAACUAAG | |
|  |  | NC\_003869.1/395133-395373  | UGAAUAUUAAA**G****C****C****U****U**..**A**.**U**..**G**.**G**.U...**C****C**.**C**.----...................................AUGA...........U----**G**.**G****G****U**..U....AA..**A****A**..**G**..**G**.G..AA.**G**A.**C**.**G**.**G****G**.**U**GA.....................................................................................................................................................................G**A**AU.....**C****C****C****G**.**C****G****C****A****G****C**C..C.CC..**G****C****U****A****C****U****G****U**GA..**G****G****G****A**.....................................................GGACGA**A****G****C****C****C****U**AGUAA..........................................................................................**G****C****C**A...**C**.**U****G****U****C****C**GGCAC............UCAAC..........................UGAGCGCGUUAGUAAGGAGAAAAGAGGGAGAGAAAUUGCGUUCAGUUGAGUGCC**G****G****G****U****G****G**G.A.A.**G****G****C**...**A****G****G**..**G****U****G**GAGGAUGAG-.......................................................................................................**U****C****C****C**...G.**A**.....**G**.**C**....**C****A****G**....**G**AG..A**C**.**C**..**U**.....G...**C****C**.**A**..**U**.**A****A****G****G****U**UUUAGAAGUUC | |
|  |  | NC\_002947.3/3857543-3857766  | CCUUAUGCCUC**G****C****G****U****U**..**C**.**A**..**G**.**G**.UG..**C****C**.**C**.**C**---...................................UCAG............---**G****G**.**G****G****U**..G....AA..**A****C**..**G**..**G**.G..AA.**A**..**C**.**C**.**G****G**.**U**GCGUCCCAGGCCCUUCAGCAGGGCCGGAC..........................................................................................................................................A**A**UG.....**C****C****G****G**.**U****G****C****U****G****C**C..C.CC..**G****C****A****A****C****G****G****U**AA..**G****C****G****A**.....................................................--GUGA**A****G****C****G****U****C**UGU--..........................................................................................**A****C****C**A...**C**.**U****G****U****G****C**CUCGUA...........GUACG................................................................................**G****C****A****U****G****G**G.A.A.**G****G****U**...**G****A****C**..**G****C****G**UUCCAGGAGC............................................................................................CCAGCUCUUCC**U****C****G****C**...A.**A**.....**G**.**C**....**C****C****G**....**G**AG..A**C**.**C**..**G**.....G...**C****C**.**U**..**G**.**G****C****G****U****U**CAUGAACACCC | |
|  |  | NC\_005296.1/2352000-2352211  | UGUGGUGCCGC**U****C****G****C****U**..**U**.**C**..**G**.**G**.UG..**C****C**.**C**.**U****U****C**-...................................GUUC............-**G****A****A****G**.**G****G****U**..G....AA..**A****C**..**G**..**G**.G..AA.**U**..**G**.**C**.**G****G**.**U**GCGGCGAGCAAUCGCC.......................................................................................................................................................A**A**GU.....**C****C****G****C**.**G****G****C****U****G****C**C..C.CC..**G****C****A****A****C****U****G****U**AA..**G****C****G****G**A....................................................UCGUCU**C****C****G****G****U****C**ACUCC..........................................................................................**G****C****C**A...**C**.**U****G****A****G****U**UCGGCAUCC........UAGAG..............................................................................CC**G****C****U****C****G****G**G.A.A.**G****G****C**...**G****A****C**..**C****G****G**GACAAUGU--.......................................................................................................**C****C****G****C**...G.**A**.....**G**.**C**....**C****A****G**....**G**AG..A**C**.**C**..**G**.....G...**C****C**.**G**..**A**.**A****G****U****C****C**GCAACCAAGCC | |
|  |  | NZ\_AAAW03000049.1/19726-19545  | AAUAAGAAACU**A****U****U****G****A**..**C**.**A**..**G**.**G**.U...**U****U**.**A**.**C**---...................................GCAA............---**G****U**.**A****A****U**..G....AA..**A****A**..**G**..**G**.G..AA.**U**..**C**.**A**.**G****G**.**U**GC.....................................................................................................................................................................A**A**AU.....**C****C****U****G**.**A****G****C****A****A****C**C..C.CC..**G****U****U****A****C****U****G****U**AA..**G****C****G****C**CGUUAAA..............................................GAUUUA**U****U****U****A****U****C**UUCAU..........................................................................................**G****C****C**A...**C**.**U****G****G****C**-.................-GAAG................................................................................-**A****C****U****G****G**G.A.A.**G****G****C**...**G****A****U**..**A****A****A**UAAAG-----.......................................................................................................**G****C****G****C**...G.**A**.....**G**.**C**....**C****A****G**....**G**AG..A**C**.**C**..**U**.....G...**C****C**.**U**..**G**.**U****U****A****A****U**AAAACUACAAU | |
|  |  | NZ\_AAAV02000001.1/1405662-1405860  | UGGCGCGCCGA**C****G****C****C****A**..**G**.**A**..**G**.**G**.UG..**C****C**.**C**.**G****G**--...................................UAAC............--**A****C****G**.**G****G****C**..U....AA..**G****A**..**G**..**G**.G..AA.**G**..**C**.**C**.**G****G**.**U**UC.....................................................................................................................................................................G**A**UU.....**C****C****G****G**.**C****G****C****U****G****C**C..C.CC..**G****C****A****A****C****U****G****U**AA..**C****C****G****G**A....................................................UAGCGC**G****U****C****G****C****C**CAUUCAU........................................................................................**G****C****C**A...**C**.**U****G****G****U****G**UCGG.............CUUCG...........................................................................GCCAG**C****A****C****C****G****G**G.A.A.**G****G****C**..G**G****G****C**..**G****A****C**GAGCCGUGAC.......................................................................................................**C****C****G****G**...A.**A**.....**G**.**C**....**C****A****G**....**G**AA..A**C**.**C**..**U**.....G...**C****C**.**C**..**U**.**U****G****G****U****U**GUCGUUCCGCG | |
|  |  | NZ\_AADX02000005.1/115090-115301  | AGAAUCCACCU**C****A****C****U****U**..**A**.**C**..**G**.**G**.UU..**U****U**.**C**.**C****C****C****U**GC.................................AUCA.........UCA**C****G****G****G****G**.**A****A****A**..U....AA..**U****A**..**G**..**G**.G..AA.**U**..**C**.**C**.**G****G**.**U**GAAGACAAGAUGUUCG.......................................................................................................................................................U**A**AU.....**C****C****G****G**.**A****A****C****U****G****C**C..C.CC..**G****C****A****A****C****U****G****U**AA..**U****C****G****G**U....................................................GAGUCU**G****U****U****U****G****C**CACUCUU........................................................................................**G****C****C**A...**C**.**U****G****G****A**-.................CUUGA................................................................................-**U****C****C****G****G**G.A.A.**G****G****C**..C**G****C****A**..**A****G****C**AAGACGAUGA......................................................................................................C**C****C****G****A**...G.**A**.....**G**.**U**....**C****A****G**....**G**AG..A**C**.**C**..**U**.....G...**C****C**.**G**..**C**.**A****A****G****U****G**AGCUAUGUUGU | |
|  |  | NZ\_AAEH02000007.1/165025-164832  | UUACGCUUGCG**C****A****C****C****G**..**A**.**U**..**G**.**G**.U...**U****C**.**C**.**C****G**--...................................GAGA............--**C****G****G**.**G****A****U**..C....AA..**A****A**..**G**..**G**.G..AA.**C**..**G**.**C**.**A****G**.**C**AGAGGCCGAGCCUC.........................................................................................................................................................C**A**AU.....**C****U****G****C**.**G****G****C****U****G****C**C..C.CC..**G****C****A****A****C****U****G****U**AA..**G****C****G****G**.....................................................CGAAUC**C****G****C****G****C****C**ACCACGC........................................................................................**G****C****C**A...**C**.**U****G****G**--.................GAAA-................................................................................--**C****C****G****G**G.A.A.**G****G****C**..C**G****G****C**..**G****C****G**GAUGACGAC-.......................................................................................................**C****C****G****C**...A.**A**.....**G**.**C**....**C****A****G**....**G**AG..A**C**.**C**..**U**.....G...**C****C**.**A**..**U**.**C****G****A****C****C**CGAGGUCCAGC | |
|  |  | NZ\_AAAW03000208.1/1274-1493  | UACACUGCGCC**C****G****C****C****A**..**C**.**A**..**G**.**G**.UG..**U****C**.**C**.**U****G****C****C**GUCCGC.............................GCAU.....GCGGGCG**G****G****C****A****G**.**G****G****U**..G....AA..**A****C**..**G**..**G**.G..AA.**G**..**C**.**C**.**G****G**.**U**GCGGACGCGAGUCC.........................................................................................................................................................C**A**UC.....**C****C****G****G**.**C****A****C****U****G****C**C..C.CC..**G****C****A****A****C****G****G****U**AG..**G****C****G****A**.....................................................-GAAAA**A****C****C****G****G****C**AUCG-..........................................................................................**G****C****C**A...**C**.**U****G****C****G****C**.................GAAC-................................................................................**G****C****G****C****G****G**G.A.A.**G****G****C**...**G****C****C**..**G****G****C**GGGACGCGAC...............................................................................................AGCGUCGC**U****C****G****C**...G.**A**.....**G**.**U**....**C****C****G**....**G**AG..A**C**.**C**..**G**.....G...**C****C**.**U**..**G**.**U****G****G****C****G**GACAUCGCCGA | |
|  |  | NZ\_AAIE01000029.1/3124-2926  | CAAUGGCAUCA**U****G****A****G****C**..**G**.**G**..**G**.**G**.UG..**C****C**.**C**.**C****G****G**-...................................GGGA............-**C****C****G****G**.**G****G****A**..G....AA..**U****C**..**G**..**G**.G..AA.**G**..**C**.**C**.**G****G**.**U**GA.....................................................................................................................................................................A**A**GU.....**C****C****G****G**.**C****A****C****G****G****G**-..C.CC..**G****C****C****G****C****G****G****U**AA..**C****C****G****G**.....................................................GAAGUC**G****C****C****G****U****C**CAUGCGCGUCGCGCA................................................................................**G****C****C**A...**C**.**U****G****G****G****C**C................CUCGG................................................................................**G****U****C****C****G****G**G.A.A.**G****G****C**..G**G****A****C**..**G****G****C**CGGCGUUGAU.......................................................................................................**C****C****G****G**...A.**A**.....**G**.**U**....**C****C****G**....**A**AG..A**C**.**C**..**G**.....G...**C****C**.**C**..**C**.**G****C****A****U****C**GACGUCGCCCG | |
|  |  | NC\_004757.1/696804-696577  | UCUGCGCCCCU**U****G****U****U****U**..**G**.**A**..**G**.**G**.UG..**U****C**.**C**.**C****A****G****A**UGC................................AUUU.........GCA**C****G****A****G****G**.**G****A****U**..G....AA..**A****C**..**G**..**G**.G..AA.**G**..**C**.**C**.**G****G**.**U**GCGCGCUGGAAUCAGCCAGCGC.................................................................................................................................................A**A**UG.....**C****C****G****G**.**C****A****C****U****G****C**C..C.CC..**G****C****A****A****C****G****G****U**AA..**A****U****G****A**G....................................................UCAAUG**A****U****C****U****G****C**ACAC-..........................................................................................**G****C****C**A...**C**.**U****G****U****G****C**U................GUAUG................................................................................**G****C****A****C****G****G**G.A.A.**G****G****C**...**G****C****A**..**G****A****A**UCAGGAAAAC............................................................................................ACAGCUUCCGC**U****C****A****U**...A.**A**.....**G**.**U**....**C****C****G**....**G**AG..A**C**.**C**..**G**.....G...**C****C**.**U**..**G**.**A****A****G****C****A**AUAUCAUGUUA | |
|  |  | NC\_004578.1/1878136-1878350  | CCUUCGCGGCU**U****G****U****U****U**..**C**.**A**..**G**.**G**.UG..**C****U**.**C**.**U****G****C****A**AUCCUC.............................GAUU............**G****G****C****A****G**.**G****G****U**..G....AA..**A****C**A.**G**..**G**.G..AA.**G**..**C**.**C**.**G****G**.**U**GUGGAUCGACCGAUCC.......................................................................................................................................................G**A**UC.....**C****C****G****G**.**C****G****C****U****G****C**C..C.CC..**G****C****A****A****C****G****G****U**AA..**A****U****G****A**G....................................................UCAAGG**C****U****G****U****G****C**AUCGU..........................................................................................**G****C****C**A...**C**.**U****G****U****G****U**.................UUCG-................................................................................**A****C****A****C****G****G**G.A.A.**G****G****C**...**G****C****G**..**C****A****G**CCGGGGUAAC...................................................................................................CCGC**U****C****A****U**...G.**A**.....**G**.**C**....**C****C****G**....**G**AG..A**C**.**C**..**G**.....G...**C****C**.**U**..**G**.**A****A****C****C****A**CUCAAUGGCAU | |
|  |  | NC\_003047.1/954760-954963  | UUAGAUGAGGA**C****A****C****U****C**..**A**.**A**..**G**.**G**.UG..**C****C**.**G**.**C****C****U****C**G..................................AAGG...........G**A****G****G****G****C**.**G****G****A**..G....AA..**U****U**..**G**..**G**.G..AA.**G**..**C**.**C**.**G****G**.**U**CA.....................................................................................................................................................................A**A**UC.....**C****C****G****G**.**C****G****C****U****G****C**C..C.CC..**G****C****A****A****C****G****G****U**GG..**U****G****G****A**GCG..................................................AACAGC**C****A****C****G****G****C**AGAAG..........................................................................................**G****C****C**A...**C**.**U****G****G****A****C**.................ACCGC................................................................................**G****U****C****C****G****G**G.A.A.**G****G****C**...**G****C****C**..**G****G****G**CAGGUCCCUU..............................................................................................GCGGACGGC**U****C****C****A**...G.**A**.....**G**.**C**....**C****C****G**....**G**AA..A**C**.**C**..**A**.....G...**C****C**.**U**..**U**.**G****A****A****G****C**AGAAAUAGACC | |
|  |  | NZ\_AAIT01000019.1/24385-24170  | GCAAUGCGGGG**C****C****G****C****U**..**A**.**C**..**G**.**G**.U...**U****C**.**C**.**G****G****C**-...................................GCGA.........UCU-**G****C****C****G**.**G****A****U**..G....AA..**A****A**..**G**..**G**.G..AA.**C**A.**C**.**C**.**G****G**.**C**GCGCCCAUCGCCGCGAUGCGGACGGGC............................................................................................................................................A**A**GG.....**C****C****G****G**.**G****G****C****U****G****C**C..C.CC..**G****C****A****A****C****U****G****U**AG..**G****C****G****G**U....................................................GAGCGA**A****G****G****C****C****G**AAGGC..........................................................................................**G****C****C**A...**C**.**U****G****G****G****A**.................AGAU-................................................................................**C****C****C****C****G****G**G.A.A.**G****G****C**...**C****G****G**..**C****C****C**GAGCGAUGAU.......................................................................................................**C****C****G****C**...C.**A**.....**G**.**C**....**C****A****G**....**G**AG..A**C**.**C**..**U**.....G...**C****C**.**G**..**C**.**A****G****C****G****A**UCACCCAUUUC | |
|  |  | NC\_006085.1/2294565-2294765  | AUAGUGACGAA**U****G****C****A****C**..**G**.**U**..**G**.**G**.UU..**C****C**.**C**.**G****U****G**-...................................UUCG............-**C****A****C****G**.**G****G****C**..C....AA..**G****A**..**G**..**G**.G..AA.**U**..**C**.**C**.**G****G**.**U**GU.....................................................................................................................................................................G**A**AU.....**C****C****G****G**.**A****G****C****U****G****C**-..C.CC..**G****C****A****G****C****G****G****U**AU..**A****U****G****A**GA...................................................ACGACC**G****C****C****G****U****C**AUGAC..........................................................................................-**G****C**A...**C**.**U****G****U****G****C**.................UUUG-................................................................................**G****C****A****U****G****G**G.A.A.**G****C**-...**G****A****C**..**G****G****C**UAGUAGGAGU........................................................................................CCGAUCAGGACGAGC**U****C****A****U**...G.**A**.....**G**.**C**....**C****C****G**....**A**AG..A**C**.**C**..**U**.....G...**C****C**.**A**..**G**.**G****U****G****U****G**GUCCCGCAACC | |
|  |  | NC\_003296.1/765144-764965  | AUCCCGCCUCA**C****G****A****U****G**..**A**.**U**..**G**.**G**.UG..**C****C**.**C**.**U**---...................................-UCC............---**A****G**.**G****G****U**..G....AA..**A****C**..**G**..**G**.G..AA.**C**..**G**.**C**.**G****G**.**U**GC.....................................................................................................................................................................G**A**UG.....**C****C****G****C**.**G****G****C****U****G****C**C..C.CC..**G****C****A****A****C****U****G****U**AA..**G****C****G****A**.....................................................CGAGUC**U****G****C****G****C****C**AACCA..........................................................................................**G****C****C**A...**C**.**C****G****C****A**-.................-CGAU................................................................................-**G****C****C****G****G**G.A.A.**G****G****C**...**G****G****C**..**G****C****C**AGGCGAUGAC.......................................................................................................**G****C****G****C**...G.**A**.....**G**.**C**....**C****A****G**....**G**AG..A**C**.**C**..**G**.....G...**C****C**.**A**..**U**.**C****U****C****C****U**UCUGUCGACGU | |
|  |  | NC\_003911.11/3073055-3073267  | GGCUAAAUGAA**G****G****G****G****U**..**C**.**U**..**G**.**G**.UC..**C****U**.**G**.**C****C****G****G**A..................................GCAA...........U**C****C****G****G****A**.**U****G****C**..U....AA..**G****A**..**G**..**G**.G..AA.**U**..**U**.**U**.**G****G**.**U**GCGACCGCCCGUCGGUU......................................................................................................................................................C**A**GG.....**C****C****A****A**.**A****G****C****C****G****C**C..C.CC..**G****C****G****A****C****U****G****U**AA..**G****C****G****G**U....................................................GAGCCU**C****U****G****U****U****C**AACCA..........................................................................................**G****C****C**A...**C**.**U****C****C****C****G**G................GUUCG............................................................................CCCC**C****G****G****G****G****G**G.A.A.**G****G****C**...**G****A****A**..**C****A****G**AGGCACUGAC.......................................................................................................**C****C****G****C**...G.**A**.....**G**.**C**....**C****A****G**....**G**AG..A**C**.**C**..**U**.....G...**C****C**.**A**..**G**.**A****C****A****G****G**CACUGUAACAC | |
|  |  | NC\_005296.1/5422528-5422731  | ACUUCUAAUGG**C****G****G****U****G**..**A**.**C**..**G**.**G**.U...**U****C**.**C**.**C**---...................................GAGA............---**G****G**.**G****A****U**..G....AA..**A****A**..**G**..**G**.G..AA.**U**..**A**.**C**.**G****G**.**U**GCGGACGCAGACACUUUCGCGUUC...............................................................................................................................................U**A**GG.....**C****C****G****U**.**A****G****C****U****G****U**U..C.CC..**G****C****A****A****C****U****G****U**AA..**G****C****G****G**.....................................................AUCGUC**U****U****U****C****G****U**CGGAU..........................................................................................**G****C****C**A...**C**.**U****G****G****G****A**A................CCUCG...............................................................................G**U****C****C****U****G****G**G.A.A.**G****G****C**..G**A****C****G**..**G****A****A**GAUCAA----.......................................................................................................**C****C****G****C**...G.**A**.....**G**.**C**....**C****A****G**....**G**AG..A**C**.**C**..**U**.....G...**C****C**.**G**..**U**.**C****A****U****U****C**GUGGUCACACG | |
|  |  | NZ\_AAFG02000009.1/29707-29921  | GCAUUGUCGAC**G****G****C****A****U**..**A**.**C**..**G**.**G**.U...**G****U**.**C**.**C****C****A****G**AGC................................GCAA........AGCG**C****C****G****G****G**.**A****C****U**..G....AA..**A****C**..**G**..**G**.G..AA.**U**..**G**.**A**.**G****G**.**A**ACGGCGGACCCAAUCGCGGCGCC................................................................................................................................................A**A**AG.....**C****C****U****C**.**A****A****C****C****G****C**C..C.CC..**G****C****G****A****C****U****G****U**GA..**G****C****G****G**.....................................................UGAGCG**A****C****A****U****U****C**AGCA-..........................................................................................**U****C****C**A...**C**.**U****G****G****C****C**.................GCUG-................................................................................**G****G****C****C****G****G**G.A.A.**G****G****A**...**G****A****A**..**U****G****A**UCGCCGCGAC.......................................................................................................**C****C****G****C**...G.**A**.....**G**.**U**....**C****A****G**....**G**AG..A**C**.**C**..**G**.....G...**C****C**.**G**..**U**.**A****U****G****A****A**ACGAGGACAGU | |
|  |  | NZ\_AAIS01000004.1/579994-579789  | CUUGUUUGGGA**G****A****U****C****A**..**U**.**C**..**G**.**G**.UG..**C****C**.**U**.**C****C****C****G**...................................----............**C****G****G****G****A**.**G****G****U**..G....AA..**A****C**..**G**..**G**.G..AA.**U**..**G**.**C**.**G****G**.**U**GCGGGGAUACUGCCCCC......................................................................................................................................................A**A**UA.....**C****C****G****C**.**G****G****C****U****G****C**C..C.CC..**G****C****A****A****C****U****G****U**AA..**G****C****G****G**.....................................................CCAUCC**G****U****A****U****C****C**AUUCG..........................................................................................**G****C****C**A...**C**.**U****G****A****C****C**AUGCC............AUGCA...............................................................................U**G****U****G****C****G****G**G.A.A.**G****G****C**...**G****G****A**..**U****C****C**GGAUAACGG-.......................................................................................................**C****C****G****C**...G.**A**.....**G**.**C**....**C****A****G**....**G**AG..A**C**.**C**..**G**.....G...**C****C**.**G**..**G**.**U****G****A****C****G**CAUUCUUAGGC | |
|  |  | NC\_007005.1/4368222-4368008  | CCUUCGCGGUU**U****G****U****U****U**..**C**.**A**..**G**.**G**.UG..**C****U**.**C**.**U****G****C****A**AUCCUC.............................GAUU............**G****G****C****A****G**.**A****G****U**..G....AA..**A****C**A.**G**..**G**.G..AA.**G**..**C**.**C**.**G****G**.**U**GUGGGUCGACAGAUCC.......................................................................................................................................................G**A**UC.....**C****C****G****G**.**C****G****C****U****G****C**C..C.CC..**G****C****A****A****C****G****G****U**AA..**A****U****G****A**G....................................................UCAAGG**C****U****G****U****G****C**CUGAC..........................................................................................**G****C****C**A...**C**.**U****G****U****G****U**.................UUCG-................................................................................**A****C****A****U****G****G**G.A.A.**G****G****C**...**G****C****G**..**C****A****G**CCGGGGUGAC...................................................................................................CCGC**U****C****A****U**...G.**A**.....**G**.**C**....**C****C****G**....**G**AG..A**C**.**C**..**G**.....G...**C****C**.**U**..**G**.**A****A****U****C****A**CUCAAUGACAU | |
|  |  | NZ\_AAHI01000004.1/50301-50108  | UUACCCUUGCG**C****G****C****U****G**..**A**.**U**..**G**.**G**.U...**U****C**.**C**.**C****G**--...................................GAGA............--**C****G****G**.**G****A****U**..C....AA..**A****A**..**G**..**G**.G..AA.**C**..**G**.**C**.**A****G**G**U**GAGGCAACGCCUC..........................................................................................................................................................G**A**AG.....**C****U****G****C**.**G****G****C****U****G****C**C..C.CC..**G****C****A****A****C****U****G****U**GA..**G****C****G****G**.....................................................CGAAUC**C****G****U****G****C****C**ACCGCAU........................................................................................**G****C****C**A...**C**.**U****G****G**--.................GAAA-................................................................................--**C****C****G****G**G.A.A.**G****G****C**..C**C****G****C**..**A****C****G**GACGACGAC-.......................................................................................................**C****C****G****C**...G.**A**.....**G**.**C**....**C****A****G**....**G**AG..A**C**.**C**..**U**.....G...**C****C**.**A**..**U**.**C****G****A****C****C**CGAGGUCCAGC | |
|  |  | NZ\_AAHL01000020.1/75570-75763  | UUACCCUUGCG**C****G****C****U****G**..**A**.**U**..**G**.**G**.U...**U****C**.**C**.**C****G**--...................................GAGA............--**C****G****G**.**G****A****U**..C....AA..**A****A**..**G**..**G**.G..AA.**C**..**G**.**C**.**A****G**G**U**GAGGCAACGCCUC..........................................................................................................................................................G**A**AG.....**C****U****G****C**.**G****G****C****U****G****C**C..C.CC..**G****C****A****A****C****U****G****U**GA..**G****C****G****G**.....................................................CGAAUC**C****G****U****G****C****C**ACCGCAU........................................................................................**G****C****C**A...**C**.**U****G****G**--.................GAAA-................................................................................--**C****C****G****G**G.A.A.**G****G****C**..C**C****G****C**..**A****C****G**GACGACGAC-.......................................................................................................**C****C****G****C**...G.**A**.....**G**.**C**....**C****A****G**....**G**AG..A**C**.**C**..**U**.....G...**C****C**.**A**..**U**.**C****G****A****C****C**CGAGGUCCAGC | |
|  |  | NC\_004557.1/1511755-1511938  | CAUAUUAUGAA**U****U****U****U****U**..**A**.**U**..**G**.**G**.U...**G****U**.**A**.**A****U**--...................................UUCA...........C--**A****U****U**.**A****C****G**..U....AA..**A****A**..**G**..**G**.G..AA.**G**..**C**.**U**U**G****G**.**U**GU.....................................................................................................................................................................A**A**AU.....**C****C****A****G**.**C****A****C****G****G****U**-..C.CC..**G****C****C****A****C****U****G****U**AA..**G****A****G****A**.....................................................GAGUAU**A****U****C****A****U****U**AUAU-..........................................................................................**G****C****C**A...**C**.**U****G****U****U****G**.................UUUAU................................................................................**C****A****A****U****G****G**G.A.A.**G****G****C**...**A****A****U**..**G****A****U**GUACUAUGA-.......................................................................................................**U****C****U****C**...A.**A**.....**G**.**U**....**C****A****G**....**G**AG..A**C**.**C**..**U**.....A...**C****C**.**A**..**U**.**A****A****A****A****A**CUUAUACAAUU | |
|  |  | NZ\_AAIS01000004.1/502910-503132  | ACUAGAUAUGG**U****G****G****U****C**..**A**.**C**..**G**.**G**.U...**C****U**.**C**.**U****C****G****A**GUC................................GCAA..........AA**U****C****G****A****G**.**A****G****C**..U....AA..**G****A**..**G**..**G**.G..AA.**G**..**C**.**C**.**G****G**.**U**GCGCCCGAGUCUUAAUGGCGGGC................................................................................................................................................A**A**UG.....**C****C****G****G**.**C****G****C****U****G****C**C..C.CC..**G****C****A****A****C****U****G****U**AA..**G****C****G****G**C....................................................GAGCUG**A****A****G****U****C****C**AUUAUUGU.......................................................................................**G****U****C**A...**C**.**U****G****G****G****A**CC...............GCAAG...............................................................................G**C****C****C****U****G****G**G.A.A.**G****A****C**..C**G****G****A**..**C****C****G**ACGCCUAGAC.......................................................................................................**C****C****G****C**...A.**A**.....**G**.**C**....**C****A****G**....**U**AG..A**U**.**C**..**U**.....G...**C****C**.**G**..**C**.**G****A****C****A****A**CAAAAACGUCC | |
|  |  | NZ\_AAAP01003421.1/1751-1551  | GGUUAGAAGGU**C****A****G****C****G**..**A**.**C**..**G**.**G**.U...**U****C**.**C**.**C**---...................................UUCG............---**G****G**.**G****A****U**..C....AA..**A****C**..**G**..**G**.G..AA.**C**..**G**.**C**.**G****G**.**U**GAGGGUUCGCCCUC.........................................................................................................................................................G**A**UG.....**C****C****G****C**.**G****G****C****U****G****C**C..C.CC..**G****C****A****A****C****U****G****U**GA..**G****C****G****G**CG...................................................AGCCUU**U****U****C****A****C****C**ACCGAU.........................................................................................**G****U****C**A...**C**.**U****G****G****A****U**GGG..............CCUC-................................................................................**A****U****C****C****G****G**G.A.A.**G****A****C**...**G****G****U**..**G****A****G**AGGGCGACGA......................................................................................................C**C****C****G****C**...G.**A**.....**G**.**C**....**C****A****G**....**G**AG..A**C**.**C**..**U**.....G...**C****C**.**G**..**U**.**C****A****G****C****C**GUGGUCACACG | |
|  |  | NZ\_AAAG02000002.1/445655-445880  | UACCGUAUUUA**G****C****G****U****U**..**A**.**C**..**G**.**G**.UU..**C****C**.**U**.**U****G****G****G**UAU................................GCAA..........UA**U****C****U****A****G**.**G****G****U**..G....AA..**G****A**..**G**..**G**.G..AA.**G**..**A**.**C**.**G****G**.**U**GCGCCCUUGAAGGGC........................................................................................................................................................A**A**UG.....**C****C****G****G**.**C****G****C****U****G****C**C..C.CC..**G****C****A****A****C****U****G****U**GA..**G****C****G****G**C....................................................GAGCCC**G****C****G****U****C****C**AAGACU.........................................................................................**G****C****C**A...**C**.**U****G****G****U****G**CGCCGG...........UCCUU.......................................................................CGGAACCGG**C****C****C****C****G****G**G.A.A.**G****G****C**..C**G****G****A**..**C****G****G**GGGCUGUGAC.......................................................................................................**C****C****G****C**...A.**A**.....**G**.**C**....**C****A****G**....**G**AG..A**C**.**C**..**U**.....G...**C****C**.**G**..**U**.**A****A****U****G****G**CCGCAACGUCC | |
|  |  | NZ\_AAAP01003109.1/772-976  | CAAAGGUCGUG**C****G****G****C****C**..**A**.**A**..**G**.**G**.UU..**C****C**.**U**.**G****C****G****G**A..................................CUCU.........UCC**C****C****G****U****G**.**G****G****C**..U....AA..**G****A**..**G**..**G**.G..AA.**U**..**C**.**C**.**G****G**.**U**CGGUUCGUCC.............................................................................................................................................................G**A**UG.....**C****C****G****G**.**A****G****C****U****G****C**C..C.CC..**G****C****A****A****C****U****G****U**GA..**G****C****G****G**.....................................................CGAGCU**C****C****C****G****C****C**GCGAGAG........................................................................................**G****U****C**A...**C**.**U****G****G****U****G**.................CCCCG................................................................................**C****A****C****C****G****G**G.A.A.**G****G****C**.CG**G****G****C**..**G****G****G**GGCGCUGAC-.......................................................................................................**C****C****G****U**...G.**A**.....**G**.**C**....**C****A****G**....**G**AG..A**C**.**C**..**U**.....G...**C****C**.**U**..**C**.**G****G****C****A****C**GACGAUAUUCC | |
|  |  | NC\_002570.2/528874-529071  | AGUGUUUGUGG**A****C****G****G****U**..**A**.**A**..**G**.**G**.U...**G****C**.**C**.**C**---...................................GAAG............---**C****G**.**G****C****U**..U....AA..**A****A**..**G**..**G**.G..AA.**U**..**C**.**U**.**G****G**.**U**GC.....................................................................................................................................................................A**A**AU.....**C****C****G****G**.**A****G****C****U****G****U**C..C.CC..**G****C****A****A****C****U****G****U**GA..**G****U****G****C**.....................................................UACGAA**C****G****G****A****A****C**GAUUU..........................................................................................**G****C****C**A...**C**.**U****G****U****A****C**AUCCUCUACUUC.....UUGAG............................................................................AAAU**G****U****A****U****G****G**G.A.A.**G****G****C**...-**U****U**..**C****U****A**AGUAGGUAAA.......................................................................................................**G****C****A****C**...G.**A**.....**G**.**U**....**C****A****G**....**G**AG..A**C**.**C**..**U**.....G...**C****C**.**U**..**U**.**A****C****U****U****C**CACAAGUUUCG | |
|  |  | NC\_002939.4/3303201-3302976  | GAAUAUUUCAA**G****C****G****U****U**..**C**.**A**..**G**.**G**.UG..**C****U**.**U**.**C****C****A****C**U..................................CCCA..........CG**G****U****G****G****A**.**A****G****G**..U....AA..**A****A**..**G**..**G**.G..AA.**A**..**A**.**G**.**G****G**.**U**GA.....................................................................................................................................................................G**A**AU.....**C****C****C****U**.**U****G****C****U****G****U**-..C.CC..**G****C****A****A****C****U****G****U**GA..**A****C****G****G**U....................................................GAUGAA**A****G****C****C****G****C**AGCGAU.........................................................................................**G****C****C**A...**C**.**U****G****G****A****G**AGUCCUUG.........UUGAU.........................................................GUGAAGGCGUGAAAUACGCCUUC**U****U****C****C****G****G**G.A.A.**G****G****C**...**G****C****G**..**G****C****G**AGUAAAGUGA......................................................................................................U**C****C****G****U**...G.**A**.....**G**.**C**....**C****A****G**....**G**AA..A**C**.**C**..**U**.....G...**C****C**.**C**..**G**.**A****A****C****G****C**AUGAGCAGCGG | |
|  |  | NC\_006834.1/1368227-1368452  | UACCAUGCGCG**C****C****C****C****U**..**G**.**A**..**G**.**G**.UG..**A****C**.**U**.**G****U****C****G**G..................................CUUG...........C**C****G****G****C****G**.**G****U****U**..U....AA..**A****C**..**G**..**G**.G..AA.**U**..**C**.**C**.**G****G**.**U**GCGCAGAUCGCCUUGGCGUGGUGC...............................................................................................................................................A**A**UU.....**C****C****G****G**.**A****G****C****U****G****C**C..C.CC..**G****C****A****A****C****G****G****U**GG..**G****C****G****A**G....................................................ACAAGG**G****U****C****U****G****C**AUGUAAC........................................................................................**A****C****C**A...**C**.**U****G****U****G****C**.................-GGU-................................................................................**G****C****A****C****G****G**G.A.A.**G****G****C**...**G****C****A**..**G****A****U**CCGGGAAGCG............................................................................................AUUGCUUCCGC**U****C****G****C**...A.**A**.....**G**.**C**....**C****C****G**....**G**AG..G**C**.**C**..**G**.....G...**C****C**.**U**..**G**.**A****A****G****G****G**AUUGACCCGGC | |
|  |  | NZ\_AAAE01000118.1/30848-30635  | GUCACGCCGGG**C****U****A****U****G**..**A**.**C**..**G**.**G**.U...**U****C**.**C**.**C****G****A****A**CGCC...............................GCAA.........GGU**C****A****A****G****G**.**G****A****U**..G....AA..**A****A**..**G**..**G**.G..AA.**C**..**G**.**C**.**G****G**.**U**GAGGGGUGCUGACCCC.......................................................................................................................................................A**G**UU.....**C****C****G****C**.**G****A****C****U****G****C**C..C.CC..**G****C****A****A****C****U****G****U**GA..**G****C****G****G**C....................................................GAGCCC**G****C****C****C****C****G**AAGAC..........................................................................................**G****C****C**A...**C**.**U****G****G****A****C**C................GAAAG...............................................................................G**G****C****C****C****G****G**G.A.A.**G****G****U**..U**C****G****G**..**G****G****A**AGGCCGCGAC.......................................................................................................**C****C****G****C**...G.**A**.....**G**.**U**....**C****A****G**....**G**AG..A**C**.**C**..**U**.....G...**C****C**.**G**..**U**.**C****G****A****G****C**GCGCAAGCGCC | |
|  |  | NC\_006510.1/2300644-2300445  | UAAAUCAACUC**G****C****A****G****C**..**A**.**A**..**G**.**G**.C...**G****C**.**C**.----...................................GAAA...........A----**G**.**G****C****U**..U....AA..**U****A**..**G**..**G**.G..AA.**U**..**C**.**C**.**G****G**.**U**GG.....................................................................................................................................................................G**A**AU.....**C****C****G****G**.**A****G****C****U****G****U**C..C.CC..**G****C****A****A****C****U****G****U**CA..**A****U****G****C**.....................................................GGACGA**A****A****U****G****A****A**AUC--..........................................................................................**G****C****C**A...**C**.**U****G****U****A****C**GGACGGAUGGC......UUGCG.......................................................................CUGUUUUCC**G****U****A****C****G****G**G.A.A.**G****G****C**...**U****U****C**..**A****G****A**GUAGGAUGAA.......................................................................................................**G****C****A****U**...G.**A**.....**G**.**C**....**C****A****G**....**U**AG..A**C**.**C**..**U**.....G...**C****C**.**U**..**U**.**G****C****U****U****G**CCGCAGUUUCC | |
|  |  | NC\_002678.2/4044596-4044395  | UUAGAUCAUGU**C****A****U****C****U**..**C**.**A**..**G**.**G**.UG..**C****C**.**G**.**C****U****U****C**GU.................................GACG..........AC**G****G****G****G****C**.**G****G****A**..G....AA..**U****U**..**G**..**G**.G..AA.**G**..**C**.**C**.**G****G**.**U**CA.....................................................................................................................................................................A**A**GU.....**C****C****G****G**.**C****G****C****U****G****C**C..C.CC..**G****C****A****A****C****G****G****U**GG..**U****G****G****A**GU...................................................UCAAGU**C****G****C****A****A****C**GGGAG..........................................................................................**A****C****C**A...**C**.**U****G****G****G****C**.................AAAA-................................................................................**G****C****C****U****G****G**G.A.A.**G****G****U**...**G****U****C**..**G****C****G**ACCGUCCGCA................................................................................................AGGACAC**U****C****C****A**...G.**A**.....**G**.**C**....**C****C****G**....**G**AA..A**C**.**C**..**A**.....G...**C****C**.**C**..**G**.**A****G****A****U****U**UUUGAACUCGA | |
|  |  | NC\_003909.8/3791525-3791713  | CCUUUCAAAAG**G****A****A****A****A**..**U**.**G**..**G**.**G**.U...**A****C**.**A**.**C****G****A****A**CA.................................GUUU..........GU**U****U****C****G****U**.**G****U****U**..U....AA..**A****A**..**G**..**G**.G..AA.**G**C.**U**.**U**.**G****G**.**U**GA.....................................................................................................................................................................A**A**CU.....**C****C****A****A**.**C****A****C****G****G****U**-..C.CC..**G****C****C****A****C****U****G****U**AA..**A****U****G****C**.....................................................UGAGAU**U****U****C****U****U****U**UUAAU..........................................................................................**G****C****C**A...**C**.**U****G****U**--.................GAAA-................................................................................--**A****C****G****G**G.A.A.**G****G****C**..G**A****A****A**..**G****A****A**AUCAUAUGAA.......................................................................................................**G****C****A****U**...A.**A**.....**G**.**U**....**C****A****G**....**G**AG..A**C**.**C**..**U**.....G...**C****C**.**U**..**G**.**U****U****U****U****A**ACAACACUGAU | |
|  |  | NC\_004557.1/745345-745529  | UGAAUAUGAAA**A****U****A****U****U**..**C**.**A**..**G**.**G**.U...**G****C**.**C**.**A**---...................................UUUU............---**A****G**.**G****U****U**..U....AA..**A****A**..**G**..**G**.G..AA.**U**..**G**.**U**.**G****G**.**U**UU.....................................................................................................................................................................A**A**UU.....**C****C****A****C**.**A****G****C****A****G****C**C..C.CC..**G****C****U****A****C****U****G****U**AA..**U****U****G****A**.....................................................GGACGA**A****U****C****U****U****U**CACAA..........................................................................................**A****C****C**A...**C**.**U****C****U****U****U**C................AAAAA..............................................................................GG**G****A****A****G****G****G**G.A.A.**G****G****G**...**A****A****A**..**G****A****C**AAGGGUGAA-.......................................................................................................**U****C****A****U**...G.**A**.....**G**.**C**....**C****A****G**....**G**AG..A**C**.**C**..**U**.....G...**C****C**.**U**..**G**.**U****A****U****A****U**GAAAGAUAAAC | |
|  |  | NZ\_AAHJ01000001.1/57754-57537  | AUAAGUAAUAA**C****A****G****U****U**..**A**.**C**..**G**.**G**.U...**U****U**.**C**.**C****G****G****C**GCCUG..............................GAAG.........GGC**G****C****C****G****G**.**A****A****U**..G....AA..**A****A**..**G**..**G**.G..AA.**C**..**C**.**C**.**G****G**.**U**GA.....................................................................................................................................................................A**A**AU.....**C****C****G****G**.**G****A****C****A****G****U**G..C.CC..**G****C****U****G****C****U****G****U**GA..**U****C****C****C**CCCGUCGGCAUCUGCCGGCGGC...............................GCGGUG**C****U****U****C****C****G**AAAAG..........................................................................................**G****C****C**A...**C**.**U****G****G****U****C**CGCGC............CUGCG................................................................................**G****A****C****C****G****G**G.A.A.**G****G****C**...**C****G****G**..**A****A****G**CAUG------.......................................................................................................**G****G****G****A**...G.**A**.....**G**.**U**....**C****A****G**....**A**AG..A**C**.**C**..**U**.....G...**C****C**.**G**..**U**.**A****A****C****G****A**AGUAAUGCUUC | |
|  |  | NC\_003030.1/2557883-2558061  | AAAUAAUACCA**U****A****U****U****U**..**U**.**A**..**G**.**G**.C...**A****C**.**C**.**U****A**--...................................AUCU............--**U****A****G**.**G****U****U**..U....AA..**U****A**..**G**..**G**.G..AA.**A**..**U**.**U**.**G****G**.**U**GA.....................................................................................................................................................................A**A**AU.....**C****C****A****A**.**U****G****C****A****A****C**C..C.CC..**G****U****U****A****C****U****G****U**AU..**A****C****A**-.....................................................GUUACA**A****A****A****C****C****A**AUG--..........................................................................................**U****C****C**A...**C**.**U****G****G****A****G**.................UUUU-................................................................................**C****U****C****U****G****G**G.A.A.**G****G****A**...**U****G****G**..**U****U****G**AGGCUAAAC-.......................................................................................................-**U****G****U**...G.**A**.....**G**.**C**....**C****A****G**....**G**AG..A**C**.**C**..**U**.....A...**C****C**.**U**..**A**.**A****A****A****U****A**UUAUGGAACUU | |
|  |  | NC\_006526.1/994341-994519  | GGAAAUUUUUU**U****G****C****A****U**..**A**.**G**..**G**.**G**.U...**U****U**.**C**.**C****U****U****C**...................................GAGU............**G****A****A****G****G**.**A****A**-..-....AA..**U****U**..**G**..**G**.G..AA.**C**..**A**.**A**.**G****G**.**U**GC.....................................................................................................................................................................A**A**AA.....**C****C****U****U**.**G****G****C****U****G****C**C..C.CU..**G****C****A****A****C****U****G****U**AA..**A****C****A****G**.....................................................--UUGA**A****A****C****G****C****C**AAAAA..........................................................................................**G****C****C**A...**C**.**U****G****A****A**-.................UCUA-................................................................................-**U****U****C****G****G**G.A.A.**G****G****C**...**G****G****U**..**U****G****U**UUCGAUG---.......................................................................................................**C****U****G****U**...G.**A**.....**G**.**C**....**C****A****G**....**G**AG..A**C**.**C**..**G**.....A...**C****C**.**C**..**U**.**A****U****G****U****A**AUCGUUCCACG | |
|  |  | NC\_002678.2/6170874-6170694  | AGGUCGCCGCC**A****C****U****G****C**..**C**.**U**..**G**.**G**.UG..**C****C**.**C**.**G****C****C**-...................................GCAA............--**G****C****G**.**G****G****A**..G....AA..**U****C**..**G**..**G**.G..AA.**C**..**A**.**C**.**G****G**.**U**UG.....................................................................................................................................................................A**A**CU.....**C****C****G****U**.**G****G****C****G****U****G**-..C.CC..**A****A****C****G****C****U****G****U**AA..**G****G****G****G**.....................................................GACCGC**G****C****C****G****G****U**AAAU-..........................................................................................**G****C****C**A...**C**.**U****G****U****C**-.................-GAU-................................................................................-**G****A****C****G****G**G.A.A.**G****G****C**...**A****C****C**..**G****G****A**CGCGGGUUGA.......................................................................................................**U****C****C****C**...G.**A**.....**G**.**C**....**C****A****G**....**A**AG..A**C**.**C**..**G**.....G...**C****C**.**U**..**G**.**G****C****A****G****G**CAUCGUCAUCC | |
|  |  | NC\_006270.2/1417626-1417440  | UUUCAUCGCCG**G****G****A****A****C**..**A**.**U**..**G**.**G**.U...**A****A**.**U**.**C****U****A****A**C..................................GAGG............**U****U****A****G****A**.**U****U****U**..U....AA..**A****A**..**G**..**G**.G..AA.**G**U.**U**.**U**.**G****G**.**U**GA.....................................................................................................................................................................A**A**AU.....**C****C****A****A**.**C****G****C****G****G****U**-..C.CC..**G****C****C****A****C****U****G****U**GA..**A****U****G****A**.....................................................GGAGGU**U****A****U****U****U****C**AUAAAA.........................................................................................**C****C****C**A...**C**.**U****G****U****U**-.................UCUA-................................................................................-**U****A****U****G****G**G.A.A.**G****G****G**..G**G****A****A**..**A****U****A**ACCGUCGAU-.......................................................................................................**U****C****A****U**...G.**A**.....**G**.**C**....**C****A****G**....**G**AG..A**C**.**C**..**U**.....G...**C****C**.**U**..-.**G****U****U****C****U**GACGCACCAUA | |
|  |  | NC\_006322.1/1418488-1418302  | UUUCAUCGCCG**G****G****A****A****C**..**A**.**U**..**G**.**G**.U...**A****A**.**U**.**C****U****A****A**C..................................GAGG............**U****U****A****G****A**.**U****U****U**..U....AA..**A****A**..**G**..**G**.G..AA.**G**U.**U**.**U**.**G****G**.**U**GA.....................................................................................................................................................................A**A**AU.....**C****C****A****A**.**C****G****C****G****G****U**-..C.CC..**G****C****C****A****C****U****G****U**GA..**A****U****G****A**.....................................................GGAGGU**U****A****U****U****U****C**AUAAAA.........................................................................................**C****C****C**A...**C**.**U****G****U****U**-.................UCUA-................................................................................-**U****A****U****G****G**G.A.A.**G****G****G**..G**G****A****A**..**A****U****A**ACCGUCGAU-.......................................................................................................**U****C****A****U**...G.**A**.....**G**.**C**....**C****A****G**....**G**AG..A**C**.**C**..**U**.....G...**C****C**.**U**..-.**G****U****U****C****U**GACGCACCAUA | |
|  |  | NZ\_AADT03000015.1/25415-25245  | GCAUAUGGUUA**C****U****U****U****U**..**C**.**A**..**G**.**G**.UG..**C****C**.**C**.----...................................GCAA............----**G**.**G****G****A**..G....AA..**U****A**..**G**..**G**.G..AA.**C**..**C**.**G**.**G****G**.**U**GC.....................................................................................................................................................................G**A**AU.....**C****C****C****G**.**G****A****C****G****G****A**-..C.CC..**G****C****C****A****C****U****G****U**GAA.**G****G****U**-.....................................................-GAGCA**C****U****U****G****C****C**AGA--..........................................................................................-**C****C**A...**C**.**U****C****C**--.................UUAAU................................................................................--**G****G****G****G**G.A.A.**G****G**-...**G****G****C**..**A****A****A**GGUGUGAUGA.......................................................................................................-**U****C****C**...G.**A**.....**G**.**U**....**C****A****G**....**G**AG..A**C**.**C**..**U**.....G...**C****C**.**U**..**G**.**A****A****A****A****G**UUUAUGCCGCC | |
|  |  | NZ\_AADF01000001.1/901367-901574  | AAAAUGGCGGC**C****G****U****C****U**..**C**.**A**..**G**.**G**.UG..**C****U**.**G**.**C****G****U****A**CG.................................GAUU.......AUCCA**A****G****C****G****C**.**A****G****U**..U....AA..**A****C**..**G**..**G**.G..AA.**A**..**G**.**C**.**G****G**.**U**GCGUCUCCAACGAGAC.......................................................................................................................................................C**A**UG.....**C****C****G****C**.**U****G****C****U****G****C**C..C.CC..**G****C****A****A****C****G****G****U**AA..**G****C****A****A**G....................................................UGCGGA**C****G****U****A****U****C**ACAAG..........................................................................................**G****C****C**A...**C**.**U****G****G****G****U**.................GACC-................................................................................**A****C****C****U****G****G**G.A.A.**G****G****C**...**G****A****U**..**A****C****G**UCAAGA----......................................................................................................C**U****U****G****U**...G.**A**.....**G**.**C**....**C****C****G**....**G**AU..A**C**.**C**..**G**.....G...**C****C**.**U**..**G**.**G****A****A****C****A**CCCAUUGGAAG | |
|  |  | NZ\_AAFG02000003.1/428172-428406  | CGUAGCAUCCA**G****A****C****A****G**..**A**.**U**..**G**.**G**.U...**U****C**.**C**.**U****G****C****C**UCU................................GUUU..........GG**G****G****U****G****G**.**G****A****U**..G....AA..**A****A**..**G**..**G**.G..AA.**U**..**A**.**C**.**G****G**.**U**GAGGCUUUGGACCAUUGGGUCCCGCC.............................................................................................................................................A**A**GU.....**C****C****G****U**.**G****A****C****U****G****C**C..C.CC..**G****C****A****A****C****U****G****U**GA..**G****C****G****G**C....................................................GAGUUC**C****C****C****G****G****C**AAAG-..........................................................................................-**C****C**A...**C**.**U****G****A****C****C**CCAACGUCGUAACCGGCAAAAA..............................................................................AG**G****G****U****C****G****G**G.A.A.**G****G**-...**G****C****C**..**G****G****U**UCGCGAUGAC.......................................................................................................**C****C****G****C**...A.**A**.....**G**.**U**....**C****A****G**....**G**AG..A**C**.**C**..**U**.....G...**C****C**.**A**..**U**.**C****G****G****C****A**AUGUAAACCUG | |
|  |  | NZ\_AADF01000001.1/232251-232043  | ACAAUCCGCCA**U****G****U****U****U**..**U**.**G**..**G**.**G**.UG..**C****C**.**U**.**U****G****C****C**UC.................................GCGU.......UCGCG**G****G****G****C****G**.**G****G****U**..G....AA..**A****C**..**G**..**G**.G..AA.**G**..**C**.**C**.**G****G**.**U**GACUCUGUGCAAGCAGA......................................................................................................................................................C**A**UU.....**C****C****G****G**.**C****G****C****A****G****C**C..C.CC..**G****C****U****G****C****U****G****U**AA..**G****C****C**-.....................................................UGACGA**C****U****C****C****A****C**CCCAC..........................................................................................**G****C****C**A...**C**.**U****G****U****G****C**.................-AUU-................................................................................**G****C****A****U****G****G**G.A.A.**G****G****U**.UG**G****U****G**..**G****A****G**AAGAACGAA-.......................................................................................................-**G****G****C**...G.**A**.....**G**.**C**....**C****A****G**....**A**AG..A**C**.**C**..**G**.....G...**C****C**.**C**..**A**.**A****A****U****C****A**ACGAAGUACCA | |
|  |  | NC\_004557.1/1488022-1487834  | UAAUAUAAUAA**G****A****C****A****A**..**C**.**A**..**G**.**G**.U...**U****U**.**G**.**A****A****A****U**A..................................AUAU.......UUAUU**A****U****U****U****C**.**A****A****U**..U....AA..**A****A**..**G**..**G**.G..AA.**A**..**C**.**A**.**G****G**.**U**GU.....................................................................................................................................................................A**A**CU.....**C****C****U****G**.**U****A****C****G****G****U**-..C.CC..**G****C****C****G****C****U****G****U**AA..**U****A****A****A**.....................................................AGAGUA**G****U****G****C****A****G**AAUAU..........................................................................................**G****C****C**A...**C**.**U****G****U**--.................-UAAU................................................................................--**A****U****G****G**G.A.A.**G****G****C**..U**U****U****G**..**U****A****C**UAUGUUGAU-.......................................................................................................**U****U****U****U**...A.**A**.....**G**.**U**....**C****A****G**....**A**AG..A**C**.**C**..**U**.....G...**C****C**.**U**..**G**.**U****U****G****U****U**AUAGAUCAUAC | |
|  |  | NC\_006905.1/2135812-2135637  | CCAUAACGUAA**A****C****C****A****A**..**C**.**A**..**G**.**G**.UUU.**G****C**.**C**.**A****C**--...................................AUUU............--**G****U****G**.**G****U**-..-....--..-**A**..**G**..**G**.G..AA.**G**..**G**.**G**.**G****G**.**U**GA.....................................................................................................................................................................A**A**AU.....**C****C****C****C**.**C****G****C****A****G****C**C..C.CC..**G****C****U****G****C****U****G****U**GA..**U****G****C**-.....................................................UGACGA**C****C****C****C****G****U**AAAGA..........................................................................................-**C****C**A...**C**.**U****G****A****U****C**.................GCAA-................................................................................**G****A****U****U****G****G**G.A.A.**G****G**-...**A****C****G**..**G****G****C**GAGGAGGAC-.......................................................................................................-**G****C****U**...A.**A**.....**G**.**C**....**C****A****G**....**A**AG..A**C**.**C**..**U**.....G...**C****C**.**U**..**G**.**U****U****G****G****U**GAUAACCAACA | |
|  |  | NC\_006177.1/361924-361720  | UAUAUUGGGGU**U****G****U****G****A**..**C**.**C**..**G**.**G**.U...**G****C**.**A**.**C****C****U**-...................................AUC-............-**G****G****G****U**.**G****C****U**..G....AA..**A****A**..**G**..**G**.G..AA.**C**..**C**.**C**.**U****G**.**U**GC.....................................................................................................................................................................G**A**AU.....**C****A****G****G**.**G****A****C****U****G****C**-..C.CC..**G****C****A****G****C****G****G****U**GA..**G****C****A****G**G....................................................AACGAC**C****G****C****C****A****C**AGCAA..........................................................................................-**G****C**A...**C**.**U****G****G****G****C**.................UCCG-................................................................................**G****C****C****U****G****G**G.A.A.**G****C**-...**G****U****G**..**G****C****C**AGUAGGACAG.................................................................................GCGGCAGAUGGUGCCGCCCCGC**C****U****G****C**...A.**A**.....**G**.**U**....**C****C****G**....**A**AG..A**C**.**C**..**U**.....G...**C****C**.**G**..**G**.**U****C****A****U****C**CGCUUUCAGGG | |
|  |  | NC\_005957.1/3839045-3839233  | CCUUUCAAAAG**G****A****A****A****A**..**U**.**A**..**G**.**G**.U...**A****C**.**A**.**C****G****A****A**C..................................AUUU.........CGU**U****U****C****G****U**.**G****U****U**..U....AA..**A****A**..**G**..**G**.G..AA.**G**C.**U**.**U**.**G****G**.**U**GA.....................................................................................................................................................................A**A**CU.....**C****C****A****A**.**C****A****C****G****G****U**-..C.CC..**G****C****C****A****C****U****G****U**AA..**A****U****G****C**.....................................................UGAGAU**U****U****C****U****U****U**GUAGU..........................................................................................**G****C****C**A...**C**.**U****G****U**--.................GAAA-................................................................................--**A****C****G****G**G.A.A.**G****G****U**..A**A****A****A**..**G****A****A**AUUAUAUGAA.......................................................................................................**G****C****A****U**...A.**A**.....**G**.**U**....**C****A****G**....**G**AG..A**C**.**C**..**U**.....G...**C****C**.**U**..**G**.**U****U****U****U****A**ACAACACUGAU | |
|  |  | NC\_006138.1/230113-229918  | UUCUGCCAAGG**U****G****U****U****U**..**C**.**A**..**G**.**G**.U...**G****C**.**U**.**U****U****G****C**CUGUG..............................GUAA.........AAG**G****U****U****U****A**.**G****C****U**..U....AA..**U****A**..**G**..**G**.G..AA.**U**..**C**.**C**.**U****G**.**U**GG.....................................................................................................................................................................A**A**UU.....**C****A****G****G**.**A****A****C****G****G****G**-..C.CC..**G****C****C****G****C****U****G****U**AA..**C****U****G****G**G....................................................GACAAC**U****U****C****U****G****C**AUUAU..........................................................................................**G****U****C**A...**C**.**U****G****A****A****C**.................GUUU-................................................................................**G****U****U****U****G****G**G.A.A.**G****G****C**...**G****C****A**..**G****A****A**GAAGGAUGAU.......................................................................................................**C****C****A****G**...A.**A**.....**G**.**U**....**C****A****G**....**A**AG..A**C**.**C**..**U**.....G...**C****C**.**U**..**G**.**C****A****A****C****A**AGUUUUGGUAU | |
|  |  | NC\_003997.3/3859224-3859412  | CCUUUCAAAAG**G****A****A****A****A**..**U**.**A**..**G**.**G**.U...**A****C**.**A**.**C****G****A****A**C..................................AUUU.........CGU**U****U****C****G****U**.**G****U****U**..U....AA..**A****A**..**G**..**G**.G..AA.**G**C.**U**.**U**.**G****G**.**U**GA.....................................................................................................................................................................A**A**CU.....**C****C****A****A**.**C****A****C****G****G****U**-..C.CC..**G****C****C****A****C****U****G****U**AA..**A****U****G****C**.....................................................UGAGAU**U****U****C****U****U****U**UUGAU..........................................................................................**A****C****C**A...**C**.**U****G****U**--.................GAAA-................................................................................--**A****C****G****G**G.A.A.**G****G****U**..A**A****A****A**..**G****A****A**AUUAUAUGAA.......................................................................................................**G****C****A****U**...A.**A**.....**G**.**U**....**C****A****G**....**G**AG..A**C**.**C**..**U**.....G...**C****C**.**U**..**G**.**U****U****U****U****A**ACAACACUGAU | |
|  |  | NC\_005945.1/3859724-3859912  | CCUUUCAAAAG**G****A****A****A****A**..**U**.**A**..**G**.**G**.U...**A****C**.**A**.**C****G****A****A**C..................................AUUU.........CGU**U****U****C****G****U**.**G****U****U**..U....AA..**A****A**..**G**..**G**.G..AA.**G**C.**U**.**U**.**G****G**.**U**GA.....................................................................................................................................................................A**A**CU.....**C****C****A****A**.**C****A****C****G****G****U**-..C.CC..**G****C****C****A****C****U****G****U**AA..**A****U****G****C**.....................................................UGAGAU**U****U****C****U****U****U**UUGAU..........................................................................................**A****C****C**A...**C**.**U****G****U**--.................GAAA-................................................................................--**A****C****G****G**G.A.A.**G****G****U**..A**A****A****A**..**G****A****A**AUUAUAUGAA.......................................................................................................**G****C****A****U**...A.**A**.....**G**.**U**....**C****A****G**....**G**AG..A**C**.**C**..**U**.....G...**C****C**.**U**..**G**.**U****U****U****U****A**ACAACACUGAU | |
|  |  | NC\_006274.1/3895737-3895925  | CCUUUCAAAAG**G****A****A****A****A**..**U**.**A**..**G**.**G**.U...**A****C**.**A**.**C****G****A****A**C..................................AUUU.........UGU**U****U****C****G****U**.**G****U****U**..U....AA..**A****A**..**G**..**G**.G..AA.**G**C.**U**.**U**.**G****G**.**U**GA.....................................................................................................................................................................A**A**CU.....**C****C****A****A**.**C****A****C****G****G****U**-..C.CC..**G****C****C****A****C****U****G****U**AA..**A****U****G****C**.....................................................UGAGAU**U****U****C****U****U****U**UUGAU..........................................................................................**A****C****C**A...**C**.**U****G****U**--.................GAAA-................................................................................--**A****C****G****G**G.A.A.**G****G****U**..A**A****A****A**..**G****A****A**AUUAUAUGAA.......................................................................................................**G****C****A****U**...A.**A**.....**G**.**U**....**C****A****G**....**G**AG..A**C**.**C**..**U**.....G...**C****C**.**U**..**G**.**U****U****U****U****A**ACAACACUGAU | |
|  |  | NC\_007530.2/3859351-3859539  | CCUUUCAAAAG**G****A****A****A****A**..**U**.**A**..**G**.**G**.U...**A****C**.**A**.**C****G****A****A**C..................................AUUU.........CGU**U****U****C****G****U**.**G****U****U**..U....AA..**A****A**..**G**..**G**.G..AA.**G**C.**U**.**U**.**G****G**.**U**GA.....................................................................................................................................................................A**A**CU.....**C****C****A****A**.**C****A****C****G****G****U**-..C.CC..**G****C****C****A****C****U****G****U**AA..**A****U****G****C**.....................................................UGAGAU**U****U****C****U****U****U**UUGAU..........................................................................................**A****C****C**A...**C**.**U****G****U**--.................GAAA-................................................................................--**A****C****G****G**G.A.A.**G****G****U**..A**A****A****A**..**G****A****A**AUUAUAUGAA.......................................................................................................**G****C****A****U**...A.**A**.....**G**.**U**....**C****A****G**....**G**AG..A**C**.**C**..**U**.....G...**C****C**.**U**..**G**.**U****U****U****U****A**ACAACACUGAU | |
|  |  | NZ\_AAAC02000001.1/4308800-4308988  | CCUUUCAAAAG**G****A****A****A****A**..**U**.**A**..**G**.**G**.U...**A****C**.**A**.**C****G****A****A**C..................................AUUU.........CGU**U****U****C****G****U**.**G****U****U**..U....AA..**A****A**..**G**..**G**.G..AA.**G**C.**U**.**U**.**G****G**.**U**GA.....................................................................................................................................................................A**A**CU.....**C****C****A****A**.**C****A****C****G****G****U**-..C.CC..**G****C****C****A****C****U****G****U**AA..**A****U****G****C**.....................................................UGAGAU**U****U****C****U****U****U**UUGAU..........................................................................................**A****C****C**A...**C**.**U****G****U**--.................GAAA-................................................................................--**A****C****G****G**G.A.A.**G****G****U**..A**A****A****A**..**G****A****A**AUUAUAUGAA.......................................................................................................**G****C****A****U**...A.**A**.....**G**.**U**....**C****A****G**....**G**AG..A**C**.**C**..**U**.....G...**C****C**.**U**..**G**.**U****U****U****U****A**ACAACACUGAU | |
|  |  | NZ\_AAEN01000013.1/113715-113903  | CCUUUCAAAAG**G****A****A****A****A**..**U**.**A**..**G**.**G**.U...**A****C**.**A**.**C****G****A****A**C..................................AUUU.........CGU**U****U****C****G****U**.**G****U****U**..U....AA..**A****A**..**G**..**G**.G..AA.**G**C.**U**.**U**.**G****G**.**U**GA.....................................................................................................................................................................A**A**CU.....**C****C****A****A**.**C****A****C****G****G****U**-..C.CC..**G****C****C****A****C****U****G****U**AA..**A****U****G****C**.....................................................UGAGAU**U****U****C****U****U****U**UUGAU..........................................................................................**A****C****C**A...**C**.**U****G****U**--.................GAAA-................................................................................--**A****C****G****G**G.A.A.**G****G****U**..A**A****A****A**..**G****A****A**AUUAUAUGAA.......................................................................................................**G****C****A****U**...A.**A**.....**G**.**U**....**C****A****G**....**G**AG..A**C**.**C**..**U**.....G...**C****C**.**U**..**G**.**U****U****U****U****A**ACAACACUGAU | |
|  |  | NZ\_AAEO01000019.1/200840-201028  | CCUUUCAAAAG**G****A****A****A****A**..**U**.**A**..**G**.**G**.U...**A****C**.**A**.**C****G****A****A**C..................................AUUU.........CGU**U****U****C****G****U**.**G****U****U**..U....AA..**A****A**..**G**..**G**.G..AA.**G**C.**U**.**U**.**G****G**.**U**GA.....................................................................................................................................................................A**A**CU.....**C****C****A****A**.**C****A****C****G****G****U**-..C.CC..**G****C****C****A****C****U****G****U**AA..**A****U****G****C**.....................................................UGAGAU**U****U****C****U****U****U**UUGAU..........................................................................................**A****C****C**A...**C**.**U****G****U**--.................GAAA-................................................................................--**A****C****G****G**G.A.A.**G****G****U**..A**A****A****A**..**G****A****A**AUUAUAUGAA.......................................................................................................**G****C****A****U**...A.**A**.....**G**.**U**....**C****A****G**....**G**AG..A**C**.**C**..**U**.....G...**C****C**.**U**..**G**.**U****U****U****U****A**ACAACACUGAU | |
|  |  | NZ\_AAEP01000031.1/246254-246066  | CCUUUCAAAAG**G****A****A****A****A**..**U**.**A**..**G**.**G**.U...**A****C**.**A**.**C****G****A****A**C..................................AUUU.........CGU**U****U****C****G****U**.**G****U****U**..U....AA..**A****A**..**G**..**G**.G..AA.**G**C.**U**.**U**.**G****G**.**U**GA.....................................................................................................................................................................A**A**CU.....**C****C****A****A**.**C****A****C****G****G****U**-..C.CC..**G****C****C****A****C****U****G****U**AA..**A****U****G****C**.....................................................UGAGAU**U****U****C****U****U****U**UUGAU..........................................................................................**A****C****C**A...**C**.**U****G****U**--.................GAAA-................................................................................--**A****C****G****G**G.A.A.**G****G****U**..A**A****A****A**..**G****A****A**AUUAUAUGAA.......................................................................................................**G****C****A****U**...A.**A**.....**G**.**U**....**C****A****G**....**G**AG..A**C**.**C**..**U**.....G...**C****C**.**U**..**G**.**U****U****U****U****A**ACAACACUGAU | |
|  |  | NZ\_AAEQ01000038.1/135120-135308  | CCUUUCAAAAG**G****A****A****A****A**..**U**.**A**..**G**.**G**.U...**A****C**.**A**.**C****G****A****A**C..................................AUUU.........CGU**U****U****C****G****U**.**G****U****U**..U....AA..**A****A**..**G**..**G**.G..AA.**G**C.**U**.**U**.**G****G**.**U**GA.....................................................................................................................................................................A**A**CU.....**C****C****A****A**.**C****A****C****G****G****U**-..C.CC..**G****C****C****A****C****U****G****U**AA..**A****U****G****C**.....................................................UGAGAU**U****U****C****U****U****U**UUGAU..........................................................................................**A****C****C**A...**C**.**U****G****U**--.................GAAA-................................................................................--**A****C****G****G**G.A.A.**G****G****U**..A**A****A****A**..**G****A****A**AUUAUAUGAA.......................................................................................................**G****C****A****U**...A.**A**.....**G**.**U**....**C****A****G**....**G**AG..A**C**.**C**..**U**.....G...**C****C**.**U**..**G**.**U****U****U****U****A**ACAACACUGAU | |
|  |  | NZ\_AAER01000035.1/372074-372262  | CCUUUCAAAAG**G****A****A****A****A**..**U**.**A**..**G**.**G**.U...**A****C**.**A**.**C****G****A****A**C..................................AUUU.........CGU**U****U****C****G****U**.**G****U****U**..U....AA..**A****A**..**G**..**G**.G..AA.**G**C.**U**.**U**.**G****G**.**U**GA.....................................................................................................................................................................A**A**CU.....**C****C****A****A**.**C****A****C****G****G****U**-..C.CC..**G****C****C****A****C****U****G****U**AA..**A****U****G****C**.....................................................UGAGAU**U****U****C****U****U****U**UUGAU..........................................................................................**A****C****C**A...**C**.**U****G****U**--.................GAAA-................................................................................--**A****C****G****G**G.A.A.**G****G****U**..A**A****A****A**..**G****A****A**AUUAUAUGAA.......................................................................................................**G****C****A****U**...A.**A**.....**G**.**U**....**C****A****G**....**G**AG..A**C**.**C**..**U**.....G...**C****C**.**U**..**G**.**U****U****U****U****A**ACAACACUGAU | |
|  |  | NZ\_AAES01000024.1/111737-111925  | CCUUUCAAAAG**G****A****A****A****A**..**U**.**A**..**G**.**G**.U...**A****C**.**A**.**C****G****A****A**C..................................AUUU.........CGU**U****U****C****G****U**.**G****U****U**..U....AA..**A****A**..**G**..**G**.G..AA.**G**C.**U**.**U**.**G****G**.**U**GA.....................................................................................................................................................................A**A**CU.....**C****C****A****A**.**C****A****C****G****G****U**-..C.CC..**G****C****C****A****C****U****G****U**AA..**A****U****G****C**.....................................................UGAGAU**U****U****C****U****U****U**UUGAU..........................................................................................**A****C****C**A...**C**.**U****G****U**--.................GAAA-................................................................................--**A****C****G****G**G.A.A.**G****G****U**..A**A****A****A**..**G****A****A**AUUAUAUGAA.......................................................................................................**G****C****A****U**...A.**A**.....**G**.**U**....**C****A****G**....**G**AG..A**C**.**C**..**U**.....G...**C****C**.**U**..**G**.**U****U****U****U****A**ACAACACUGAU | |
|  |  | NZ\_AADF01000001.1/1229779-1229583  | CCGGCCUUUAU**G****U****C****U****C**..**U**.**C**..**G**.**G**.UG..**C****C**.**C**.**G****G****C****C**G..................................AUUU..........AU**C****G****G****C****G**.**G****G****A**..G....AA..**U****C**..**G**..**G**.G..AA.**G**..**G**.**C**.**G****G**.**U**GC.....................................................................................................................................................................A**A**AU.....**C****C****G****C**.**C****G****C****G****U****G**-..C.CC..**A****A****C****G****C****U****G****U**AA..**G****G****A****G**.....................................................GACGGG**C****G****A****U****G****C**GAAAA..........................................................................................**G****C****C**A...**C**.**U****G****G****U****C**AGG..............GAAAA.............................................................................CCC**G****G****C****C****G****G**G.A.A.**G****G****C**...**G****C****A**..**U****C****G**GCCGGGUGA-.......................................................................................................**A****U****C****C**...G.**A**.....**G**.**U**....**C****A****G**....**A**AG..A**C**.**C**..**G**.....G...**C****C**.**G**..**G**.**G****A****G****A****C**GAAACCGGGCU | |
|  |  | NC\_004463.1/2232793-2232995  | GAUAAUCCAAG**U****C****G****U****C**..**G**.**A**..**G**.**G**.UU..**C****U**.**C**.**C****G****G****U**UCCC...............................AUUG...........A**U****C****C****G****G**.**A****G****C**..U....AA..**G****A**..**G**..**G**.G..AA.**G**..**C**.**C**.**G****G**.**U**GC.....................................................................................................................................................................A**A**AUG....**C****C****G****G**.**C****U****C****U****G****C**C..C.CC..**G****C****A****A****C****U****G****U**GA..**G****C****G****G**C....................................................GAGCCG**C****U****G****U****C****C**GACGAU.........................................................................................**G****U****C**G...**C**.**U****G****A****A****G**CCU..............GCACG...............................................................................G**C****U****U****C****G****G**G.A.A.**G****G****C**..C**G****G****A**..**C****A****G**CAGCGAUGAC.......................................................................................................**C****A****G****C**...A.**A**.....**G**.**C**....**C****A****G**....**G**AG..A**C**.**C**..**G**.....G...**C****C**.**C**..**C**.**G****A****C****A****A**UAUAUUGGUCC | |
|  |  | NZ\_AADF01000001.1/898855-899061  | AGAAUGCGCGC**C****G****U****G****U**..**C**.**A**..**G**.**G**.UG..**C****U**.**G**.**C****G****U****A**CG.................................GAUU.......CUCCA**A****G****C****G****C**.**A****G****U**..U....AA..**A****C**..**G**..**G**.G..AA.**A**..**G**.**C**.**G****G**.**U**GCGUCUCCAACGAGAC.......................................................................................................................................................C**A**UG.....**C****C****G****C**.**U****G****C****U****G****C**C..C.CC..**G****C****A****A****C****G****G****U**AA..**G****C****A****A**G....................................................UGCGGG**C****G****U****A****U****C**ACACA..........................................................................................**G****C****C**A...**C**.**U****G****G****G**-.................AAAUA................................................................................-**C****C****U****G****G**G.A.A.**G****G****C**...**G****A****U**..**A****C****G**UCAAAA----......................................................................................................C**U****U****G****C**...G.**A**.....**G**.**C**....**C****C****G**....**G**AU..A**C**.**C**..**G**.....G...**C****C**.**U**..**G**.**A****G****A****C****A**AAAAGUGGAAG | |
|  |  | NC\_006677.1/1111874-1111665  | CGCUUCAUGAC**A****C****C****U****G**..**A**.**C**..**G**.**G**.UG..**C****C**.**C**.**G****G**--...................................CCUG............--**C****C****G**.**G****G****U**..G....AA..**A****A**..**G**..**G**.G..AA.**C**..**G**.**C**.**A****G**.**U**GC.....................................................................................................................................................................A**A**AA.....**C****U****G****C**.**G****G****C****U****G****C**C..C.CC..**G****C****A****A****C****U****G****U**AA..**G****U****G****C**UGACUGUCGCUCCGUAACGGGCGAACAGU........................ACGGCU**U****C****A****G****G****A**GAGAA..........................................................................................**C****C****C**A...**C**.**U****G****G****C**-.................CCGGU................................................................................-**G****C****C****G****G**G.A.A.**G****G****G**...**G****C****C**..**U****G****A**GACCGGA---.......................................................................................................**G****C****G****C**...G.**A**.....**G**.**C**....**C****A****G**....**G**AG..A**C**.**C**..**U**.....G...**C****C**.**G**..**U**.**C****A****G****C****A**UGAGUUGUCCG | |
|  |  | NC\_002696.2/502948-503176  | GUCUGUUGCCG**U****U****G****U****C**..**G**.**U**..**G**.**G**.U...**C****U**.**G**.**C****G****G****A**CG.................................UUCG..........CG**U****C****C****G****G**.**A****G****C**..U....AA..**G****A**..**G**..**G**.G..AA.**G**..**U**.**C**.**G****G**.**U**GAGGGCGUGAAACCCU.......................................................................................................................................................G**A**AU.....**C****C****G****G**.**C****G****C****U****G****C**C..C.CC..**G****C****A****A****C****U****G****U**GA..**G****C****G****G**C....................................................GAGCCG**C****U****G****U****C****C**GUUUCGU........................................................................................**G****U****C**A...**C**.**U****G****A****C****G**CGC..............CGAAG..................................................................CUGGUUCGGGGAUG**C****G****U****C****G****G**G.A.A.**G****G****C**.CA**G****G****G**..**C****A****G**GGGUGACGAC.......................................................................................................**C****C****G****U**...G.**A**.....**G**.**C**....**C****A****G**....**G**AG..A**C**.**C**..**U**.....G...**C****C**.**U**..**C**.**G****A****C****A****G**AUAACGUCCUC | |
|  |  | NZ\_AAAV02000001.1/132997-133225  | CCCUUCAAUGA**U****C****G****C****G**..**C**.**C**..**G**.**G**.U...**G****C**.**C**.**C****C**--...................................GCAA............--**G****G****G**.**G****C****U**..U....AA..**U****C**..**G**..**G**.G..AA.**U**..**G**.**C**.**G****G**.**U**GCGGGAGCGUGCUCCC.......................................................................................................................................................A**A**AU.....**C****C****G****U**.**G****G****C****U****G****U**C..C.CU..**G****C****A****A****C****U****G****U**AA..**G****C****G****G**AU...................................................AGCGCG**A****U****G****C****A****C**AUGCUCCUGAAUUCAGGAGCA..........................................................................**G****C****C**A...**C**.**U****G****G****G****C**AGCGC............GCAAG..........................................................................CGCCAA**G****U****C****U****G****G**G.A.A.**G****G****C**...**G****U****G**..**C****A****U**CAAGCUGUGA......................................................................................................C**C****C****G****C**...G.**A**.....**G**.**C**....**C****A****G**....**G**AG..A**C**.**C**..**U**.....G...**C****C**.**G**..**G**.**C****G****C****A****C**CGGUCGCUCUU | |
|  |  | NZ\_AAAG02000006.1/272070-271838  | AGAGCGAAGGG**G****A****C****U****G**..**C**.**C**..**G**.**G**.U...**U****C**.**C**.**A****G****G****C**CGGGC..............................AUAA......CGCCCC**G****G****C****U****G**.**G****A****U**..U....AA..**A****A**C.**G**..**G**.G..AA.**C**..**A**.**C**.**G****G**.**U**GCGGCCCCUUGCGGGCC......................................................................................................................................................C**A**AU.....**C****C****G****U**.**G****A****C****U****G****C**C..C.CC..**G****C****A****A****C****U****G****U**GA..**G****U****G****G**U....................................................GAGCCC**C****U****C****U****C****C**AUUCG..........................................................................................**G****C****C**A...**C**.**U****A****G****G****A**CCAGCGC..........UCGCG.........................................................................CGCGUCG**U****C****C****C****G****G**G.A.A.**G****G****C**...**G****G****A**..**G****A****U**CAGGGUCAGG.....................................................................................................AC**C****C****A****C**...G.**A**.....**G**.**U**....**C****A****G**....**G**AG..A**C**.**C**..**U**.....G...**C****C**.**G**..**G**.**C****U****G****G****U**CGUCACCUAAC | |
|  |  | NC\_005296.1/1097952-1098152  | UCUGGUUUUGA**C****G****U****C****U**..**U**.**C**..**G**.**G**.UG..**C****C**.**U**.**C****G**--...................................CGUG............--**C****G****A**.**G****G****U**..G....AA..**A****C**U.**G**..**G**.G..AA.**U**..**A**.**C**.**G****G**.**U**GCGGUGUUUUUCACC........................................................................................................................................................U**A**AU.....**C****C****G****U**.**A****G****C****U****G****C**C..C.CC..**G****C****A****A****C****U****G****U**AG..**G****C****G****G**A....................................................UCUGUC**C****G****G****A****U****C**AUGUA..........................................................................................**G****C****C**A...**C**.**U****G****A****C****G**U................CCUCG...............................................................................G**C****G****U****C****G****G**G.A.A.**G****G****C**...**G****G****U**..**C****C****A**GGCGAUAU--.......................................................................................................**C****C****G****U**...G.**A**.....**G**.**C**....**C****A****G**....**G**AG..A**C**.**C**..**G**.....G...**C****C**.**G**..**A**.**A****G****A****C****G**GGAAGCAUCUA | |
|  |  | NC\_003062.1/1640470-1640287  | CCAUAGCUUCU**C****C****G****G****U**..**C**.**A**..**G**.**G**.UG..**C****C**.**C**.**G****C****C****C**...................................-UUG............**C****G****G****C****G**.**G****G****A**..G....AA..**U****C**..**G**..**G**.G..AA.**U**..**C**.**C**.**G****G**.**U**GA.....................................................................................................................................................................A**A**GA.....**C****C****G****G**.**A****A****C****G****U****G**-..C.CC..**A****A****C****G****C****U****G****U**AA..**G****G****C****G**.....................................................GAUGCU**C****U****U****U****U****U**CUCAU..........................................................................................**G****C****C**A...**C**.**U****G****A****A**-.................GCAA-................................................................................-**U****U****C****G****G**G.A.A.**G****G****C**...**G****A****A**..**A****G****G**GGCGGAUGA-.......................................................................................................**A****G****C****U**...U.**A**.....**G**.**U**....**C****A****G**....**A**AG..A**C**.**C**..**G**.....G...**C****C**.**U**..**G**.**G****C****A****G****G**AUAGACCGAAC | |
|  |  | NC\_003304.1/1640583-1640400  | CCAUAGCUUCU**C****C****G****G****U**..**C**.**A**..**G**.**G**.UG..**C****C**.**C**.**G****C****C****C**...................................-UUG............**C****G****G****C****G**.**G****G****A**..G....AA..**U****C**..**G**..**G**.G..AA.**U**..**C**.**C**.**G****G**.**U**GA.....................................................................................................................................................................A**A**GA.....**C****C****G****G**.**A****A****C****G****U****G**-..C.CC..**A****A****C****G****C****U****G****U**AA..**G****G****C****G**.....................................................GAUGCU**C****U****U****U****U****U**CUCAU..........................................................................................**G****C****C**A...**C**.**U****G****A****A**-.................GCAA-................................................................................-**U****U****C****G****G**G.A.A.**G****G****C**...**G****A****A**..**A****G****G**GGCGGAUGA-.......................................................................................................**A****G****C****U**...U.**A**.....**G**.**U**....**C****A****G**....**A**AG..A**C**.**C**..**G**.....G...**C****C**.**U**..**G**.**G****C****A****G****G**AUAGACCGAAC | |
|  |  | NC\_004722.1/3977076-3977264  | CCUUUCAAAAG**G****A****A****A****A**..**U**.**A**..**G**.**G**.U...**A****C**.**A**.**C****G****A****A**A..................................GUUU.........UGU**U****U****C****G****U**.**G****U****U**..U....AA..**A****A**..**G**..**G**.G..AA.**G**C.**U**.**U**.**G****G**.**U**GA.....................................................................................................................................................................A**A**CU.....**C****C****A****A**.**C****A****C****G****G****U**-..C.CC..**G****C****C****A****C****U****G****U**AA..**A****U****G****C**.....................................................UGAGAU**U****U****C****U****U****U**UUGGU..........................................................................................**G****C****C**A...**C**.**U****G****U**--.................GAAA-................................................................................--**A****C****G****G**G.A.A.**G****G****U**..A**A****A****A**..**G****A****A**AUUAUAUGAA.......................................................................................................**G****C****A****U**...A.**A**.....**G**.**U**....**C****A****G**....**G**AG..A**C**.**C**..**U**.....G...**C****C**.**U**..**G**.**U****U****U****U****A**ACAACACUGAU | |
|  |  | NZ\_AABG04000115.1/6-191  | UGAAUAUAGUG**G****U****U****U****G**..**C**.**A**..**G**.**G**.U...**U****C**.**G**U**G****C****A****U**A..................................UUUU...........U**A****U****G****C****C**.**G****A****U**..U....AA..**A****A**..**G**..**G**.G..AA.**G**..**C**.**A**.**G****G**.**U**GA.....................................................................................................................................................................G**A**UU.....**C****C****U****G**.**C****A****C****G****G****U**-..C.CC..**G****C****C****G****C****U****G****U**GAU.**G****G****G**-.....................................................GAGACU**C****U****U****C****A****G**AUUAUU.........................................................................................**G****C****C**A...**C**.**U****G****G**--.................GAGA-................................................................................--**C****U****G****G**G.A.A.**G****G****C**...**A****U****G**..**A****A****G**AGUUGAUGAA.......................................................................................................-**C****C****U**...A.**A**.....**G**.**U**....**C****A****G**....**A**AG..A**C**.**C**..**U**.....G...**C****C**.**U**..**G**.**C****A****G****A****C**AACACCGGAAA | |
|  |  | NC\_004557.1/793388-793200  | AAUAGUAAAUG**A****A****A****U****U**..**U**.**A**..**G**.**G**.UG..**U****C**.**C**.**A****C****U****U**U..................................GUAA.........AAA**U****G****A****U****G**.**G****A****U**..G....AA..**A****A**..**G**..**G**.G..AA.**U**..**G**.**U**.**G****G**.**U**UC.....................................................................................................................................................................A**A**UU.....**C****C****A****C**.**A****G****C****A****G****C**C..C.CC..**G****C****U****A****C****U****G****U**AA..**U****A****G****A**.....................................................UGACAA**G****C****C****U****U****U**AGUA-..........................................................................................**A****C****C**A...**C**.**U****C****U****U**-.................UAAA-................................................................................-**A****G****G****A****G**G.A.A.**G****G****U**...**A****A****A**..**G****G****U**UAGAAUGAU-.......................................................................................................**U****C****U****U**...A.**A**.....**G**.**C**....**C****A****G**....**G**AG..A**C**.**C**..**U**.....G...**C****C**.**U**..**A**.**G****A****U****U****G**UUAAAAUCCUC | |
|  |  | NC\_002947.3/1866934-1867155  | CCUUCGCGGCG**U****G****U****U****U**..**C**.**A**..**G**.**G**.UG..**C****C**.**C**.**U****G****C****C**AGCCCC.............................GACU...........G**G****G****C****A****G**.**G****G****U**..G....AA..**A****C**U.**G**..**G**.G..AA.**G**..**C**.**C**.**G****G**.**U**GGGCGCCAGCAUGGCGC......................................................................................................................................................G**A**UU.....**C****C****G****G**.**C****G****C****U****G****C**C..C.CC..**G****C****A****A****C****G****G****U**GG..**A****U****G****A**GU...................................................AAAAGG**C****C****G****C****G****C**AGG--..........................................................................................**G****C****C**A...**C**.**U****G****G****A****U**G................-CCAG...............................................................................C**A****U****C****C****G****G**G.A.A.**G****G****C**...**G****C****G**..**C****A****G**GCCAGGGCCA...............................................................................................AGGCCCAC**U****C****A****C**...A.**A**.....**G**.**C**....**C****C****G**....**G**AG..A**C**.**C**..**G**.....G...**C****C**.**U**..**G**.**A****U****A****C****U**GCCAACGGCAU | |
|  |  | NC\_006510.1/1836535-1836343  | AGGCGAGAAAC**A****A****A****A****C**..**A**.**A**..**G**.**G**.UG..**C****C**.**U**.**A****G****C****C**C..................................GCUU.........AGG**G****C****U****U****G**.**G****G****U**..G....AA..**A****A**..**G**..**G**.G..AA.**G**..**C**U**C**.**G****G**.**U**GA.....................................................................................................................................................................A**A**AU.....**C****C****G****G**.**C****A****C****G****G****U**G..C.CC..**G****C****C****A****C****U****G****U**GAU.**G****G****G**-.....................................................GAGCUU**U****G****C****G****G****C**AGCAA..........................................................................................**G****U****C**A...**C**.**U****G****A****A****G**.................-GAUG................................................................................**C****U****U****C****G****G**G.A.A.**G****A****C**...**G****C****C**..**G****C****C**AAGCGAUGAU.......................................................................................................-**C****C****U**...A.**A**.....**G**.**U**....**C****A****G**....**G**AG..A**C**.**C**..**U**.....G...**C****C**.**U**..**U**.**G****U****U****U****G**GAUCGGACGGA | |
|  |  | NZ\_AAAT03000008.1/14478-14252  | CCUUCGCGGCU**U****G****U****U****U**..**C**.**A**..**G**.**G**.UG..**C****U**.**C**.**U****G****U****G**GC.................................GCAU.........GUC**G****A****C****A****G**.**A****G****U**..G....AA..**A****C**A.**G**..**G**.G..AA.**G**..**C**.**C**.**G****G**.**U**GAGGCGGGUCUUCGAGCGAAGAACCCUCAC.........................................................................................................................................G**A**UC.....**C****C****G****G**.**C****G****C****U****G****C**C..C.CC..**G****C****A****A****C****G****G****U**AA..**A****U****G****A**G....................................................UAAAAG**C****U****G****C****G****U**CUGAU..........................................................................................**G****C****C**A...**C**.**U****G****C****U**-.................UAAC-................................................................................-**A****G****C****G****G**G.A.A.**G****G****C**...**G****C****G**..**C****A****G**CCAGGCACCA..................................................................................................GCCGC**U****C****A****U**...G.**A**.....**G**.**C**....**C****C****G**....**G**AG..A**C**.**C**..**G**.....G...**C****C**.**U**..**G**.**A****U****C****C****A**UCCAGCGGCAU | |
|  |  | NC\_002947.3/3982004-3981798  | GUAGCCUUGCC**A****C****U****U****C**..**G**.**A**..**G**.**G**.UU..**C****U**.**U**.**C****G****G****C**...................................-CUG............**G****C****C****G****A**.**A****G****C**..U....AA..**G****A**C.**G**..**G**.G..AA.**C**..**G**.**C**.**G****G**.**U**AC.....................................................................................................................................................................-**A**AG.....**C****C****G****C**.**G****G****C****U****G****C**C..C.CC..**G****C****A****A****C****U****G****U**AA..**G****C****A****C**CGA..................................................CAACGG**A****U****C****G****A****C**ACA--..........................................................................................**G****C****C**A...**C**.**U****G****C****G****C**.................CAAC-................................................................................**G****C****G****C****G****G**G.A.A.**G****G****C**...**G****U****C**..**A****U****C**CCGCCAGCCC.....................................................................................GAACGGGGACAUGGAACG**G****U****G****C**...A.**A**.....**G**.**C**....**C****A****G**....**G**AG..A**C**.**C**..**U**.....G...**C****C**.**U**..**C**.**G****U****C****A****C**GUUUUCGACUU | |
|  |  | NZ\_AAFG02000005.1/329268-329471  | UACAACUCUGC**C****C****U****A****C**..**G**.**A**..**G**.**G**.UG..**C****U**.**G**.**U****C****C****G**G..................................GCUU..........UC**C****G****G****A****C**.**G****G****A**..G....AA..**U****U**..**G**..**G**.G..AA.**G**..**C**.**C**.**G****G**.**U**GC.....................................................................................................................................................................G**A**UU.....**C****C****G****G**.**C****G****C****U****G****C**C..C.CC..**G****C****A****A****C****G****G****U**CA..**U****G****G****G**AUU..................................................CGCGGG**C****G****U****A****A****C**AUCU-..........................................................................................**G****C****C**A...**C**.**U****G****G****G****A**.................GGAGG................................................................................**A****C****C****C****G****G**G.A.A.**G****G****C**...**G****U****U**..**G****C****G**CUCCGACCAG..............................................................................................CAGGUCACC**C****C****C****A**...G.**A**.....**G**.**C**....**C****C****G**....**G**AA..A**C**.**C**..**A**.....G...**C****C**.**U**..**C**.**G****G****C****A****G**AUAUCGUCAAC | |
|  |  | NZ\_AADX02000005.1/101890-102110  | AUAGCGACCAA**G****U****U****U****U**..**G**.**A**..**G**.**G**.UGU.**C****C**.**U**.**U****G****G****C**CUGUUC.............................AUGG...........C**G****C****A****A****G**.**G****G****U**..G....AA..**A****C**U.**G**..**G**.G..AA.**A**..**C**.**A**.**G****G**.**U**GCGUGAGAUCCAAUGCGAUCCAC................................................................................................................................................A**A**UG.....**C****C****U****G**.**U****G****C****U****G****C**C..C.CC..**G****C****A****A****C****G****G****U**AA..**G****C****A****A**.....................................................-GCCGA**U****A****U****G****C****C**AACAU..........................................................................................**G****C****C**A...**C**.**U****G****U****G****A**A................AGACC...............................................................................U**U****C****A****U****G****G**G.A.A.**G****G****C**...**G****G****C**..**A****U****A**UUCUUAAAUC.......................................................................................................**U****U****G****C**...A.**A**.....**G**.**C**....**C****C****G**....**G**AG..A**C**.**C**..**G**.....G...**C****C**.**U**..**G**.**A****A****A****A****C**GAUCAAGGAAG | |
|  |  | NC\_003197.1/2114073-2113898  | CCAUAACGUAA**A****C****C****A****A**..**C**.**A**..**G**.**G**.UUU.**G****C**.**C**.**A****C**--...................................AUUU............--**G****U****G**.**G****U**-..-....--..-**A**..**G**..**G**.G..AA.**G**..**G**.**G**.**G****G**.**U**GA.....................................................................................................................................................................A**A**AU.....**C****C****C****C**.**C****G****C****A****G****C**C..C.CC..**G****C****U****G****C****U****G****U**GA..**U****G****C**-.....................................................UGACGA**C****C****C****C****G****U**AAAGA..........................................................................................-**C****C**A...**C**.**U****G****A****U****C**.................GCAA-................................................................................**G****A****U****U****G****G**G.A.A.**G****G**-...**A****C****G**..**G****G****C**GAGGAGGAC-.......................................................................................................-**G****C****U**...A.**A**.....**G**.**C**....**C****A****G**....**A**AG..A**C**.**C**..**U**.....G...**C****C**.**U**..**G**.**U****C****G****G****U**GAUAACCAACA | |
|  |  | NC\_003198.1/2070647-2070472  | CCAUAACGUAA**A****C****C****A****A**..**C**.**A**..**G**.**G**.UUU.**G****C**.**C**.**A****C**--...................................AUUU............--**G****U****G**.**G****U**-..-....--..-**A**..**G**..**G**.G..AA.**G**..**G**.**G**.**G****G**.**U**GA.....................................................................................................................................................................A**A**AU.....**C****C****C****C**.**C****G****C****A****G****C**C..C.CC..**G****C****U****G****C****U****G****U**GA..**U****G****C**-.....................................................UGACGA**C****C****C****C****G****U**AAAGA..........................................................................................-**C****C**A...**C**.**U****G****A****U****C**.................GCAA-................................................................................**G****A****U****U****G****G**G.A.A.**G****G**-...**A****C****G**..**G****G****C**GAGGAGGAC-.......................................................................................................-**G****C****U**...A.**A**.....**G**.**C**....**C****A****G**....**A**AG..A**C**.**C**..**U**.....G...**C****C**.**U**..**G**.**U****C****G****G****U**GAUAACCAACA | |
|  |  | NC\_004631.1/933281-933456  | CCAUAACGUAA**A****C****C****A****A**..**C**.**A**..**G**.**G**.UUU.**G****C**.**C**.**A****C**--...................................AUUU............--**G****U****G**.**G****U**-..-....--..-**A**..**G**..**G**.G..AA.**G**..**G**.**G**.**G****G**.**U**GA.....................................................................................................................................................................A**A**AU.....**C****C****C****C**.**C****G****C****A****G****C**C..C.CC..**G****C****U****G****C****U****G****U**GA..**U****G****C**-.....................................................UGACGA**C****C****C****C****G****U**AAAGA..........................................................................................-**C****C**A...**C**.**U****G****A****U****C**.................GCAA-................................................................................**G****A****U****U****G****G**G.A.A.**G****G**-...**A****C****G**..**G****G****C**GAGGAGGAC-.......................................................................................................-**G****C****U**...A.**A**.....**G**.**C**....**C****A****G**....**A**AG..A**C**.**C**..**U**.....G...**C****C**.**U**..**G**.**U****C****G****G****U**GAUAACCAACA | |
|  |  | NC\_006511.1/931753-931928  | CCAUAACGUAA**A****C****C****A****A**..**C**.**A**..**G**.**G**.UUU.**G****C**.**C**.**A****C**--...................................AUUU............--**G****U****G**.**G****U**-..-....--..-**A**..**G**..**G**.G..AA.**G**..**G**.**G**.**G****G**.**U**GA.....................................................................................................................................................................A**A**AU.....**C****C****C****C**.**C****G****C****A****G****C**C..C.CC..**G****C****U****G****C****U****G****U**GA..**U****G****C**-.....................................................UGACGA**C****C****C****C****G****U**AAAGA..........................................................................................-**C****C**A...**C**.**U****G****A****U****C**.................GCAA-................................................................................**G****A****U****U****G****G**G.A.A.**G****G**-...**A****C****G**..**G****G****C**GAGGAGGAC-.......................................................................................................-**G****C****U**...A.**A**.....**G**.**C**....**C****A****G**....**A**AG..A**C**.**C**..**U**.....G...**C****C**.**U**..**G**.**U****C****G****G****U**GAUAACCAACA | |
|  |  | NZ\_AAEK01000007.1/65192-65004  | CCUUUCAAAAG**G****A****A****A****A**..**U**.**A**..**G**.**G**.U...**A****C**.**A**.**C****G****A****A**A..................................AUUU.........CGC**U****U****C****G****U**.**G****U****U**..U....AA..**A****A**..**G**..**G**.G..AA.**G**C.**U**.**U**.**G****G**.**U**GA.....................................................................................................................................................................A**A**CU.....**C****C****A****A**.**C****A****C****G****G****U**-..C.CC..**G****C****C****A****C****U****G****U**AA..**A****U****G****C**.....................................................UGAGAU**U****U****C****U****U****U**UCUAU..........................................................................................**G****C****C**A...**C**.**U****G****U**--.................GAAA-................................................................................--**A****C****G****G**G.A.A.**G****G****U**..A**A****A****A**..**G****A****A**AUUAUAUGAA.......................................................................................................**G****C****A****U**...A.**A**.....**G**.**U**....**C****A****G**....**G**AG..A**C**.**C**..**U**.....G...**C****C**.**U**..**G**.**U****U****U****U****A**ACAACACUGAU | |
|  |  | NC\_004557.1/823404-823586  | UUAAAUAUUUA**U****U****U****A****U**..**A**.**A**..**A**.**G**.U...**U****U**.**C**.**U****U****G**-...................................UAAA............-**A****G****A****G**.**A****A****U**..U....AA..**A****A**..**G**..**G**.G..AA.**G**..**G**.**A**.**G****G**.**C**GA.....................................................................................................................................................................A**A**AU.....**C****C****U****C**.**C****A****C****G****G****U**-..C.CG..**G****C****C****A****C****U****G****U**AA..**U****U****G****G**.....................................................UGAGUU**U****A****U****U****C****C**AUAA-..........................................................................................**G****C****C**A...**U**.**U****G****A****G**-.................AUAUU................................................................................-**C****U****U****G****A**G.A.A.**G****G****C**...**G****G****A**..**A****A****U**AAAUGAUGAA.......................................................................................................**C****C****A****U**...A.**A**.....**G**.**U**....**C****A****G**....**G**AG..A**C**.**C**..**U**.....G...**C****U**.**U**..**U**.**A****U****A****A****G**AAUAAAUAUAC | |
|  |  | NC\_002516.1/1381500-1381708  | GCUGCGCGCCU**U****G****C****G****A**..**C**.**A**..**G**.**G**.UG..**C****C**.**C**.**C**---...................................GCAA............---**G****G**.**G****G****U**..G....AA..**A****C**A.**G**..**G**.G..AA.**G**..**C**.**U**.**G****G**.**U**GCGUUCCGUCGGAAC........................................................................................................................................................C**A**GG.....**C****C****A****G**.**C****G****C****U****G****C**C..C.CC..**G****C****A****A****C****G****G****U**AG..**G****C****G****A**A....................................................UCAGAC**A****G****C****C****G****C**UCGAUG.........................................................................................**A****C****C**A...**C**.**U****G****U****G****C**.................UCCG-................................................................................**G****C****A****U****G****G**G.A.A.**G****G****C**...**G****C****G**..**G****C****U**GGAAGCGUCC.............................................................................................AGCGCUUCGC**U****C****G****C**...G.**A**.....**G**.**C**....**C****C****G**....**G**AG..A**C**.**C**..**G**.....G...**C****C**.**U**..**G**.**A****C****G****C****A**CCCACGGCAUC | |
|  |  | NZ\_AABQ07000002.1/700068-700276  | GCUGCGCGCCU**U****G****C****G****A**..**C**.**A**..**G**.**G**.UG..**C****C**.**C**.**C**---...................................GCAA............---**G****G**.**G****G****U**..G....AA..**A****C**A.**G**..**G**.G..AA.**G**..**C**.**U**.**G****G**.**U**GCGUUCCGUCGGAAC........................................................................................................................................................C**A**GG.....**C****C****A****G**.**C****G****C****U****G****C**C..C.CC..**G****C****A****A****C****G****G****U**AG..**G****C****G****A**A....................................................UCAGAC**A****G****C****C****G****C**UCGAUG.........................................................................................**A****C****C**A...**C**.**U****G****U****G****C**.................UCCG-................................................................................**G****C****A****U****G****G**G.A.A.**G****G****C**...**G****C****G**..**G****C****U**GGAAGCGUCC.............................................................................................AGCGCUUCGC**U****C****G****C**...G.**A**.....**G**.**C**....**C****C****G**....**G**AG..A**C**.**C**..**G**.....G...**C****C**.**U**..**G**.**A****C****G****C****A**CCCACGGCAUC | |
|  |  | NC\_003210.1/1179809-1179999  | UUAAAUAGGUC**U****U****A****U****G**..**U**.**U**..**G**.**G**.U...**G****G**.**A**.**A****U****G****U**...................................AUGU............**A****C****A****U****U**.**U****C****U**..G....AA..**A****G**..**A**..**G**.G..AA.**U**..**U**.**C**.**G****G**.**U**GC.....................................................................................................................................................................G**A**UG.....**C****C****G****A**.**A****A****C****U****G****C**C..C.CC..**G****C****A****A****C****U****G****U**AA..**G****G****U**-.....................................................GGACAA**G****A****A****U****C****G**AGAUA..........................................................................................**A****C****C**A...**C**.**U****G****U****A****C**GUU..............UUUAG...............................................................................C**G****U****A****U****G****G**G.A.A.**G****G****U**..U**C****G****A**..**U****U****G**UUGGAUGAA-.......................................................................................................-**G****C****C**...A.**A**.....**G**.**U**....**C****A****G**....**G**AU..A**C**.**U**..**C**.....G...**C****C**.**A**..**A**.**A****U****A****A****G**ACGGAAGCAAC | |
|  |  | NZ\_AADQ01000003.1/74724-74534  | UUAAAUAGGUC**U****U****A****U****G**..**U**.**U**..**G**.**G**.U...**G****G**.**A**.**A****U****G****U**...................................AUGU............**A****C****A****U****U**.**U****C****U**..G....AA..**A****G**..**A**..**G**.G..AA.**U**..**U**.**C**.**G****G**.**U**GC.....................................................................................................................................................................G**A**UG.....**C****C****G****A**.**A****A****C****U****G****C**C..C.CC..**G****C****A****A****C****U****G****U**AA..**G****G****U**-.....................................................GGACAA**G****A****A****U****C****G**AGAUA..........................................................................................**A****C****C**A...**C**.**U****G****U****A****C**GUU..............UUUAG...............................................................................C**G****U****A****U****G****G**G.A.A.**G****G****U**..U**C****G****A**..**U****U****G**UUGGAUGAA-.......................................................................................................-**G****C****C**...A.**A**.....**G**.**U**....**C****A****G**....**G**AU..A**C**.**U**..**C**.....G...**C****C**.**A**..**A**.**A****U****A****A****G**ACGGAAGCAAC | |
|  |  | NC\_005296.1/2349339-2349135  | CAAACAUCGCG**C****G****C****C****G**..**A**.**C**..**G**.**G**.UG..**U****C**.**C**.**U****G****U****C**...................................UUCG............**G****A****C****A****G**.**G****A****C**..G....AA..**G****A**..**G**..**G**.G..AA.**U**A.**U**.**C**.**G****G**.**A**AGAUCGCUGCCUGCAGCGGU...................................................................................................................................................C**G**CG.....**C****C****G****A**.**A****G****C****U****G****C**C..C.CC..**G****C****A****A****C****U****G****U**AA..**A****C****G****G**.....................................................UGAGCC**G****A****C****G****C****A**AUAC-..........................................................................................**G****C****C**A...**C**.**U****G****G****A**-.................UCAUA................................................................................-**U****C****C****G****G**G.A.A.**G****G****C**...**C****G****C**..**G****U****C**GGCGACGAC-.......................................................................................................**C****C****G****U**...G.**A**.....**G**.**C**....**C****A****G**....**G**AG..A**C**.**C**..**U**.....G...**C****C**.**G**..**U**.**C****G****C****C****U**GCUAUGUUGAC | |
|  |  | NC\_004663.1/2460405-2460213  | AUUUUGCAGUC**G****G****G****A****A**..**A**.**U**..**G**.**G**.U...**U****U**.**C**.**G****G****G****A**G..................................GUUU.......ACGCC**U****U****C****C****G**.**A****A****U**..G....AA..**A****A**..**G**..**G**.G..AA.**C**..**C**.**C**.**G****G**.**U**GA.....................................................................................................................................................................A**A**AU.....**C****C****G****G**.**G****A****C****A****G****U**A..C.CC..**G****C****U****G****C****U****G****U**GA..**G****U****C****C**ACAA.................................................AAACGG**A****C****A****U****C****G**AACUUUU........................................................................................**G****C****C**A...**C**.**U****G****G****U**-.................GUAAU................................................................................-**A****C****C****G****G**G.A.A.**G****G****C**..A**C****G****A**..**U****G****A**CCG-------.......................................................................................................**G****G****A****C**...G.**A**.....**G**.**U**....**C****A****G**....**A**AG..A**C**.**C**..**U**.....G...**C****C**.**A**..**U**.**G****A****C****C****G**GAAUGAGAUUU | |
|  |  | NC\_006347.1/3437566-3437374  | AUUUUGCAGUC**G****G****G****A****A**..**A**.**U**..**G**.**G**.U...**U****U**.**C**.**G****G****G****A**G..................................GUUU.......ACGCC**U****U****C****C****G**.**A****A****U**..G....AA..**A****A**..**G**..**G**.G..AA.**C**..**C**.**C**.**G****G**.**U**GA.....................................................................................................................................................................A**A**AU.....**C****C****G****G**.**G****A****C****A****G****U**A..C.CC..**G****C****U****G****C****U****G****U**GA..**G****U****C****C**ACAA.................................................AAACGG**A****C****A****U****C****G**AACUUUU........................................................................................**G****C****C**A...**C**.**U****G****G****U**-.................GUAAU................................................................................-**A****C****C****G****G**G.A.A.**G****G****C**..A**C****G****A**..**U****G****A**CCG-------.......................................................................................................**G****G****A****C**...G.**A**.....**G**.**U**....**C****A****G**....**A**AG..A**C**.**C**..**U**.....G...**C****C**.**A**..**U**.**G****A****C****C****G**GAAUGAGAUUU | |
|  |  | NC\_002678.2/1101095-1100897  | UAUAGUCAUGC**A****G****U****C****G**..**U**.**C**..**G**.**G**.U...**U****C**.**C**.----...................................GUUU...........U----**G**.**G****A****G**..CC...AA..**G****A**..**G**..**G**.G..AA.**U**..**G**.**C**.**G****G**.**U**GCGGGCGAAAUUCUUGCCC....................................................................................................................................................A**A**UG.....**C****C****G****U**.**G****G****C****U****G****C**C..C.CC..**G****C****A****A****C****U****G****U**GU..**G****C****G****G**.....................................................-UAGUC**C****U****C****U****C****C**AUAU-..........................................................................................**G****C****C**A...**C**.**U****G****A****A****G**A................UUCGU................................................................................**C****U****U****C****G****G**G.A.A.**G****G****U**...**G****G****G**..**G****A****A**GGGCGCUGAU.......................................................................................................**C****C****G****U**...G.**A**.....**G**.**C**....**C****A****G**....**G**AG..A**C**.**C**..**U**.....G...**C****C**.**G**..**A**.**C****G****A****C****G**GCAAAACUGAC | |
|  |  | NZ\_AAED02000001.1/687690-687893  | AGUCUCGGCCG**U****G****C****C****U**..**G**.**A**..**G**.**G**.UG..**C****C**.**C**.**C****G****U****C**...................................CGCA............**A****A****C****G****G**.**G****G****U**..U....AA..**A****A**..**G**..**G**.G..AA.**U**..**A**.**U**.**G****G**.**A**AGGGCAUUUUCCGCC........................................................................................................................................................C**G**AG.....**C****C****A****U**.**A****G****C****U****G****C**U..C.CC..**G****C****A****A****C****U****G****U**CA..**G****C****G****C**.....................................................UGAGGC**U****U****C****C****U****C**AACAU..........................................................................................**G****C****C**A...**C**.**U****G****G****G****C**CC...............UUUCG................................................................................**G****C****C****U****G****G**G.A.A.**G****G****C**...**G****A****G**..**G****A****C**GCCGUCGAA-.......................................................................................................**G****C****G****C**...A.**A**.....**G**.**C**....**C****A****G**....**G**AA..A**C**.**C**..**U**.....G...**C****C**.**U**..**U**.**G****G****G****U****C**GUCACCGUCCG | |
|  |  | NZ\_AAIJ01000009.1/58867-58653  | UUUAUAUAACC**A****C****C****U****U**..**G**.**U**..**G**.**G**.U...**G****C**.**C**.**A****G****U****U**GCUG...............................GAAU...........C**A****G****C****U****G**.**G****C****U**..U....AA..**A****A**..**G**..**G**.G..AA.**U**..**C**.**C**.**G****G**.**U**GA.....................................................................................................................................................................A**A**AU.....**C****C****G****G**.**A****A****C****A****G****U**A..C.CC..**G****C****U****G****C****U****G****U**AA..**U****C****C****U**CAGCCGGCAUAUUUGCCGGCAU...............................AUAUGU**U****C****U****G****U****C**AAUGAA.........................................................................................**G****C****C**A...**C**.**U****G****A****A****C**CGGG.............CCACC...............................................................................G**G****A****U****C****G****G**G.A.A.**G****G****C**...**G****A****C**..**A****G****A**CAG-------.......................................................................................................**G****G****G****A**...G.**A**.....**G**.**U**....**C****A****G**....**A**AG..A**C**.**C**..**U**.....G...**C****C**.**A**..**C**.**A****A****U****G****U**CUUGAGCUUCG | |
|  |  | NZ\_AAAW03000002.1/28224-28042  | AACAAUUUAAG**G****A****U****U****U**..**C**.**A**..**G**.**G**.UG..**C****C**.**C**.**U****U****C**-...................................GCCA............**A****G****A****A****G**.**G****G****A**..G....AA..**U****A**..**G**..**G**.G..AA.**C**..**C**.**G**.**G****G**.**U**GC.....................................................................................................................................................................A**A**UU.....**C****C****C****G**.**G****A****C****G****G****A**-..C.CC..**G****C****C****A****C****U****G****U**AA..**A****G****A****G**G....................................................AGUCUC**A****G****C****G****C****U**GAAU-..........................................................................................**G****C****C**A...**C**.**U****G****G**--.................GUAA-................................................................................--**C****U****G****G**G.A.A.**G****G****C**...**A****G****C**..**A****A****G**GAGACAAUGA.......................................................................................................**C****U****C****G**...A.**A**.....**G**.**U**....**C****A****G**....**G**AG..A**C**.**C**..**U**.....G...**C****C**.**U**..**G**.**G****A****U****C****C**GGGGACACAUG | |
|  |  | NC\_003047.1/1999767-1999554  | GGCCAUAUGCC**G****C****C****G****U**..**C**.**A**..**G**.**G**.UG..**C****C**.**C**.**G****C**--...................................GAAA...........U--**G****C****G**.**G****G****G**..G....AA..**U****C**..**G**..**G**.G..AA.**G**..**C**.**C**.**G****G**.**U**GC.....................................................................................................................................................................A**G**UU.....**C****C****G****G**.**C****A****C****G****U****G**-..C.CC..**A****A****C****G****C****U****G****U**GA..**A****G****G****G**.....................................................GACGUU**C****U****C****G****C****C**AAAAAGGGCUCUGAAUCUUUUCAGAGCUUU.................................................................**G****C****C**A...**C**.**U****G****A****A****U**A................UUGAA.............................................................................GCU**A****U****U****C****G****G**G.A.A.**G****G****C**...**G****G****C**..**G****C****G**AACGGAUGA-.......................................................................................................**U****C****C****G**...A.**A**.....**G**.**U**....**C****A****G**....**A**AG..A**C**.**C**..**G**.....G...**C****C**.**U**..**G**.**G****C****G****A****G**AUAGACCGGCC | |
|  |  | NC\_002932.3/443749-443971  | AAUAAAUAAUU**C****A****G****U****U**..**A**.**C**..**G**.**G**.U...**U****U**.**C**.**C****G****G****U**GCCCG..............................GUGG.........GGC**G****C****C****G****G**.**A****A****U**..G....AA..**A****A**..**G**..**G**.G..AA.**C**..**C**.**C**.**G****G**.**U**GA.....................................................................................................................................................................A**A**AU.....**C****C****G****G**.**G****A****C****A****G****U**G..C.CC..**G****C****U****G****C****U****G****U**GA..**U****C****C****U**CCCGUCGGCCACAAUCGGGUCGGCGGA..........................CGAUCG**C****U****U****C****C****G**AUGAG..........................................................................................**G****C****C**A...**C**.**U****G****G****U****U**CGC..............GCCCG..............................................................................CG**A****A****C****C****G****G**G.A.A.**G****G****C**...**C****G****G**..**A****A****G**CGAG------.......................................................................................................**G****G****G****A**...G.**A**.....**G**.**U**....**C****A****G**....**A**AG..A**C**.**C**..**U**.....G...**C****C**.**G**..**U**.**A****A****U****G****C**AGUAAAUGCUC | |
|  |  | NZ\_AAAW03000026.1/20946-20762  | UUAAUAAGGAA**U****C****U****C****A**..**U**.**A**..**G**.**G**.UG..**A****C**.**U**.**G****U**--...................................ACAA..........CC--**A****C****A**.**G****U****U**..G....AA..**A****A**..**G**..**G**.G..AA.**G**..**C**.**C**.**G****G**.**U**UA.....................................................................................................................................................................G**A**GG.....**C****C****G****G**.**C****A****C****G****G****U**-..C.CC..**G****C****C****G****C****U****G****U**AA..**G****G****G****A**.....................................................AAUAUA**C****U****U****C****U****U**GGCAUU.........................................................................................**A****C****C**A...**C**.**U****G****A****A**-.................AGGGU................................................................................-**U****U****C****G****G**G.A.A.**G****G****U**...**A****A****G**..**A****A****G**UAUAGAUGA-.......................................................................................................**U****C****C****U**...A.**A**.....**G**.**U**....**C****A****G**....**A**AG..A**C**.**C**..**U**.....G...**C****C**.**U**..**A**.**U****G****U****G****U**AUACCACCAAC | |
|  |  | NC\_007005.1/3709306-3709505  | UUUACCAUGCC**G****C****G****C****G**..**U**.**C**..**G**.**G**.U...**U****U**.**C**.**C**---...................................GAGA............---**G****G**.**A****A****C**..U....AA..**C****A**..**G**..**G**.G..AA.**U**..**U**.**C**.**G**-.---CACGCCUUUCUCACCAGGCGA................................................................................................................................................A**A**AA.....-**C****G****A**.**A****A****C****U****G****C**C..C.CC..**G****C****A****A****C****U****G****U**AG..**G****C****A****C**.....................................................CGAGCC**U****G****C****U****C****C**AUGACU.........................................................................................**G****C****C**A...**C**.**U****G****G****A****U**.................-CGUG................................................................................**A****U****C****C****G****G**G.A.A.**G****G****C**..C**G****G****A**..**G****C****C**UGGCGAUGAC.......................................................................................................**G****U****G****U**...C.**A**.....**G**.**U**....**C****A****G**....**G**AG..A**C**.**C**..**U**.....G...**C****C**.**G**..**A**.**C****C****C****G****C**UUCAACCCACU | |
|  |  | NC\_002973.5/1158709-1158899  | UUAAAUAGGUC**U****U****A****U****G**..**U**.**U**..**G**.**G**.U...**G****G**.**A**.**A****U****G****U**...................................AUGU............**A****C****A****U****U**.**U****C****U**..G....AA..**A****G**..**A**..**G**.G..AA.**U**..**U**.**C**.**G****G**.**U**GC.....................................................................................................................................................................G**A**UG.....**C****C****G****A**.**A****A****C****U****G****C**C..C.CC..**G****C****A****A****C****U****G****U**AA..**G****G****U**-.....................................................GGACAA**G****A****A****U****C****G**AGAUA..........................................................................................**G****C****C**A...**C**.**U****G****U****A****C**GUU..............UUUAG...............................................................................C**G****U****A****U****G****G**G.A.A.**G****G****U**..U**C****G****A**..**U****U****G**UUGGAUGAA-.......................................................................................................-**G****C****C**...A.**A**.....**G**.**U**....**C****A****G**....**G**AU..A**C**.**U**..**C**.....G...**C****C**.**A**..**A**.**A****U****A****A****G**CCGGAAGCAAC | |
|  |  | NZ\_AADR01000004.1/72171-71981  | UUAAAUAGGUC**U****U****A****U****G**..**U**.**U**..**G**.**G**.U...**G****G**.**A**.**A****U****G****U**...................................AUGU............**A****C****A****U****U**.**U****C****U**..G....AA..**A****G**..**A**..**G**.G..AA.**U**..**U**.**C**.**G****G**.**U**GC.....................................................................................................................................................................G**A**UG.....**C****C****G****A**.**A****A****C****U****G****C**C..C.CC..**G****C****A****A****C****U****G****U**AA..**G****G****U**-.....................................................GGACAA**G****A****A****U****C****G**AGAUA..........................................................................................**G****C****C**A...**C**.**U****G****U****A****C**GUU..............UUUAG...............................................................................C**G****U****A****U****G****G**G.A.A.**G****G****U**..U**C****G****A**..**U****U****G**UUGGAUGAA-.......................................................................................................-**G****C****C**...A.**A**.....**G**.**U**....**C****A****G**....**G**AU..A**C**.**U**..**C**.....G...**C****C**.**A**..**A**.**A****U****A****A****G**CCGGAAGCAAC | |
|  |  | NC\_002505.1/145123-145326  | UACUAUCAGCG**C****C****A****A****G**..**C**.**U**..**G**.**G**.U...**G****C**.**U**.**A****U****U****U**AGAUGCCUG..........................GAUG.......GCUAA**A****A****A****U****G**.**G****C****U**..G....AA..**A****A**..**G**..**G**.G..AA.**U**..**C**.**C**.**G****G**.**U**GU.....................................................................................................................................................................A**A**CU.....**C****C****G****G**.**A****A****C****U****G****A**-..C.GC..**G****C****A****G****C****G****G****U**AA..**G****A****G****A**.....................................................GAACGA**A****C****G****C****U****C**AAAC-..........................................................................................**G****A****C**A...**C**.**U****G****C****U**-.................UUUCG................................................................................-**A****G****U****G****G**G.A.A.**G****U****C**...**G****A****G**..**C****C****A**GUAGGCCAAC..................................................................................................AGUGC**U****C****U****C**...A.**A**.....**G**.**U**....**C****C****G**....**A**AG..A**C**.**C**..**U**.....G...**C****C**.**A**..**G**.**C****A****A****C****U**GAGUUAUGCAG | |
|  |  | NC\_004557.1/1481204-1481027  | AUUUAUAGAAU**A****A****A****U****U**..**U**.**A**..**G**.**G**.U...**G****C**.**U**.----...................................-UAU............----**A**.**G****C****U**..U....AA..**U****A**..**G**..**G**.G..AA.**G**..**C**.**A**.**G****G**.**U**GA.....................................................................................................................................................................A**A**AU.....**C****C****U****G**.**A****A****C****G****G****U**-..C.CC..**G****C****C****G****C****U****G****U**GAU.**G****G****G**-.....................................................GAGUUU**U****U****U****C****A****A**AGUAA..........................................................................................**A****C****C**A...**C**.**U****G****G****A****U**.................AAUAU................................................................................**A****U****C****U****G****G**G.A.A.**G****G****U**...**A****U****G**..**A****A****A**AAAUAAUGAA.......................................................................................................-**C****C****U**...A.**A**.....**G**.**U**....**C****A****G**....**A**AU..A**C**.**C**..**U**.....A...**C****C**.**U**..**A**.**A****A****U****U****A**AAUACACUAUA | |
|  |  | NZ\_AAAU03000001.1/683746-683967  | CCUGCGCGCCU**C****G****C****U****U**..**C**.**A**..**G**.**G**.UG..**C****C**.**C**.----...................................-CAA............----**G**.**G****G****U**..G....AA..**A****C**A.**G**..**G**.G..AA.**G**..**C**.**C**.**G****G**.**U**GCGUGCCGCGACCAUUCGCGGCGC...............................................................................................................................................A**A**GG.....**C****C****G****G**.**C****G****C****U****G****C**C..C.CC..**G****C****A****A****C****G****G****U**AG..**A****C****G****A**G....................................................UCGCGA**A****A****C****C****G****C**AAUA-..........................................................................................**G****C****C**A...**C**.**U****G****U****G****U**U................GCUCG...............................................................................G**A****C****A****C****G****G**G.A.A.**G****G****C**...**G****C****G**..**G****U****U**UCGGAAGCCG.......................................................................................CCGCAUGGCGCUUCGC**U****C****G****U**...G.**A**.....**G**.**C**....**C****C****G**....**G**AG..A**C**.**C**..**G**.....G...**C****C**.**U**..**G**.**U****G****G****C****G**AUCCAUGGCGG | |
|  |  | NZ\_AAAS03000001.1/133514-133719  | AAAUACAUUUU**A****C****G****U****U**..**C**.**A**..**G**.**G**.UG..**C****U**.**U**.**U****U****G****C**C..................................UCAU...........G**G****C****A****G****A**.**A****G****G**..U....AA..**A****A**..**G**..**G**.G..AA.**A**..**A**.**G**.**G****G**.**U**GC.....................................................................................................................................................................G**A**CU.....**C****C****C****U**.**U****G****C****U****G****U**-..C.CC..**G****C****A****A****C****U****G****U**GA..**A****C****G****G**U....................................................GAUGAA**A****G****C****C****G****C**AACGAU.........................................................................................**G****C****C**A...**C**.**U****G****A****U****C**UGGU.............UUCCA........................................................................UGAUGAAA**C****C****C****C****G****G**G.A.A.**G****G****C**...**G****C****G**..**G****C****G**AGUAAAGUGA......................................................................................................U**C****C****G****U**...G.**A**.....**G**.**C**....**C****A****G**....**G**AA..A**C**.**C**..**U**.....G...**C****C**.**U**..**G**.**A****C****C****G****U**CAGCUUCACAG | |
|  |  | NC\_004459.1/1165704-1165902  | UAGUAUGCGCU**U****C****A****A****G**..**C**.**U**..**G**.**G**.U...**G****C**.**U**.**A****U****C****U**G..................................GAAG.........UAU**A****G****A****U****G**.**G****C****U**..G....AA..**A****A**..**G**..**G**.G..AA.**U**..**C**.**C**.**G****G**.**U**GU.....................................................................................................................................................................G**A**AU.....**C****C****G****G**.**A****A****C****U****G****A**-..C.GC..**G****C****A****G****C****G****G****U**AA..**U****A****G****A**.....................................................GAACGA**A****A****G****C****U****U**AAUCA..........................................................................................**G****A****C**A...**C**.**U****G****C****A****C**GA...............UGGAA..............................................................................UC**G****U****G****U****G****G**G.A.A.**G****U****C**...**A****G****G**..**C****A****A**GUAGGUUAAC....................................................................................................AGC**U****C****U****U**...G.**A**.....**G**.**U**....**C****C****G**....**A**AU..A**C**.**C**..**U**.....G...**C****C**.**A**..**G**.**C****A****A****C****U**GAGCAAACACU | |
|  |  | NZ\_AAEW01000042.1/12423-12605  | UCUGCGCUGAA**A****G****U****U****U**..**C**.**A**..**G**.**G**.U...**G****C**.**U**.**U****G**--...................................-UUG............--**C****A****A**.**G****C****A**..C....AA..**U****A**..**G**..**G**.G..AA.**U**..**U**.**C**.**C****G**.**U**GA.....................................................................................................................................................................A**A**UU.....**C****G****G****G**.**A****G****U****G****G****G**-..C.CC..**G****C****C****G****C****U****G****U**AA..**C****C****A****G**G....................................................GACGAU**C****A****A****C****A****C**AUUUG..........................................................................................**G****C****C**A...**C**.**U****G****A****C**-.................UUAGA................................................................................-**G****U****C****G****G**G.A.A.**G****G****C**...**G****U****G**..**U****U****G**AGACGGAAGA......................................................................................................U**C****U****G****G**...A.**A**.....**G**.**C**....**C****A****G**....**A**AG..A**C**.**C**..**U**.....G...**C****C**.**U**..**G**.**A****A****A****C****G**CAAUGGUUGCA | |
|  |  | NC\_004463.1/3630657-3630877  | GGCACACAGGA**C****G****G****G****C**..**A**.**U**..**G**.**G**.U...**G****C**.**U**.**C****G****A****G**GUGGC..............................GCAA........AGCG**C****C****G****G****A**.**G****C****A**..U....AA..**U****C**..**G**..**G**.G..AA.**U**..**G**.**G**.**G****G**.**A**UGGGCGGACCCAGUUGCGGCGCCC...............................................................................................................................................A**A**AA.....**C****C****C****C**.**A****G****C****C****G****C**C..C.CC..**G****C****G****A****C****U****G****U**AA..**G****C****G****G**.....................................................-UGAGG**G****G****C****U****C****C**GAACC..........................................................................................**G****C****C**A...**C**.**U****G****G****G****C**C................GCAAG................................................................................**G****U****C****C****G****G**G.A.A.**G****G****C**..C**G****G****A**..**G****A****A**CCCCAGUGAA.......................................................................................................**C****C****G****C**...G.**A**.....**G**.**C**....**C****A****G**....**G**AG..A**C**.**C**..**G**.....G...**C****C**.**G**..**U**.**G****C****A****U****G**UUUUGAGGCCA | |
|  |  | NZ\_AABG04000041.1/12329-12128  | GAUAUUGAUAA**A****G****A****U****A**..**U**.**G**..**G**.**G**.UG..**C****C**.**U**.**G****G****G****A**A..................................GUUG.........CCG**U****C****U****C****A**.**G****G****U**..U....AA..**A****A**..**G**..**G**.G..AA.**G**..**C**.**A**.**G****G**.**U**GA.....................................................................................................................................................................G**A**AU.....**C****C****U****G**.**C****A****C****G****G****U**-..C.CC..**G****C****C****G****C****U****G****U**AU..**U****G****A**-.....................................................GGAGAU**G****U****U****U****C****A**AAUUUGU........................................................................................**G****C****C**A...**C**.**U****G****A****A****U**UAA..............UUGCA..........................................................................GAAUUA**A****U****U****U****G****G**G.A.A.**G****G****C**..U**U****G****A**..**A****A****C**GUCGAUGAU-.......................................................................................................-**U****C****U**...A.**A**.....**G**.**U**....**C****A****G**....**A**AA..A**C**.**C**..**U**.....G...**C****C**.**U**..**G**.**U****A****U****C****U**GAGAAACACCG | |
|  |  | NC\_004578.1/3542594-3542808  | CCUGUGCACCU**U****G****U****U****U**..**C**.**G**..**G**.**G**.UG..**C****C**.**C**.**C**---...................................UCAC............---**G****G**.**G****G****U**..G....AA..**A****C**..**G**..**G**.G..AA.**A**..**C**.**C**.**G****G**.**U**GCGCUCAACCUGUUGAGC.....................................................................................................................................................A**A**GU.....**C****C****G****G**.**U****G****C****U****G****C**C..C.CC..**G****C****A****A****C****G****G****U**AA..**G****C****G****A**.....................................................---GAG**A****A****G****G****U****C**UGA--..........................................................................................**U****C****C**A...**C**.**U****G****U****G****C**.................UCUG-................................................................................**G****C****A****U****G****G**G.A.A.**G****G****U**...**G****A****C**..**C****U****U**GAAGGUCUGA..................................................................................ACGCACACGUGUUCAAGCCCC**U****C****G****C**...G.**A**.....**G**.**C**....**C****C****G**....**G**AG..A**C**.**C**..**G**.....G...**C****C**.**C**..**G**.**A****C****A****U****U**UUUCCAAUGAC | |
|  |  | NZ\_AAEW01000001.1/38071-37865  | AAAUACGUUCA**C****C****A****C****G**..**A**.**C**..**A**.**G**.UGU.**U****C**.**A**.**G****C****C****A**UAGGAGCCACCAA......................GCCU.........CCG**C****G****G****C****U**.**G****C****U**..G....AA..**A****A**..**G**..**G**.G..AA.**U**..**C**.**C**.**C****G**.**U**GA.....................................................................................................................................................................A**A**AU.....**C****G****G****G**.**A****A****C****G****G****A**-..C.CC..**A****C****C****G****C****U****G****U**AA..**C****C****G****G**G....................................................GACAAC**A****A****U****C****G****C**AUAAC..........................................................................................**G****C****C**A...**C**.**U****G****A****A****G**.................AAUGA................................................................................**C****U****U****C****G****G**G.A.A.**G****G****C**...**G****C****G**..**A****U****U**GGAGGAUGAU.......................................................................................................**C****C****G****G**...A.**A**.....**G**.**U**....**C****A****G**....**A**AG..A**C**.**C**..**U**.....G...**C****U**.**A**..**U**.**C****G****U****G****G**UUUAAUGUCAA | |
|  |  | NC\_007005.1/3613772-3613987  | CCUGUGCGCCU**U****G****U****U****U**..**C**.**G**..**G**.**G**.UG..**C****C**.**C**.**C**---...................................UCAC............---**G****G**.**G****G****U**..G....AA..**A****C**..**G**..**G**.G..AA.**A**..**C**.**C**.**G****G**.**U**GCGCUCGCGUUGUCGAGC.....................................................................................................................................................A**A**GU.....**C****C****G****G**.**U****G****C****U****G****C**C..C.CC..**G****C****A****A****C****G****G****U**AA..**G****C****G****A**.....................................................----GA**G****U****A****G****G****U**CUGA-..........................................................................................**U****C****C**A...**C**.**U****G****U****G****C**.................CGCUG................................................................................**G****C****A****U****G****G**G.A.A.**G****G****U**..G**A****C****C**..**U****U****G**CAGGCUUUGA...................................................................................CGCAUUGACGUCCUGGCCCC**U****C****G****C**...G.**A**.....**G**.**C**....**C****C****G**....**G**AG..A**C**.**C**..**G**.....G...**C****C**.**C**..**G**.**A****U****A****U****G**CUUGCAAUGAC | |
|  |  | NZ\_AAAQ02000005.1/122974-122779  | CGACGGCGGAA**C****C****G****C****C**..**G**.**A**..**G**.**G**.UG..**C****C**.**C**.**C****A**--...................................AUCC...........A--**C****G****G**.**G****G****A**..U....AA..**U****C**..**G**..**G**.G..AA.**G**..**C**.**C**.**G****G**.**U**GC.....................................................................................................................................................................G**A**AU.....**C****C****G****G**.**C****A****C****A****G****G**-..C.CC..**G****C****U****G****C****G****G****U**GA..**C****C****U****G**.....................................................GGAGCC**G****C****C****G****A****U**CACGCGCAUCGCGCA................................................................................**G****C****C**A...**C**.**U****G****G****A****C**.................GGCA-................................................................................**G****U****C****U****G****G**G.A.A.**G****G****C**..G**A****U****C**..**G****G****A**CGGUGUUGAU.......................................................................................................**C****A****G****G**...A.**A**.....**G**.**U**....**C****C****G**....**A**AG..A**C**.**C**..**G**.....G...**C****C**.**U**..**C**.**G****G****C****A****U**GGCUGCCGUUU | |
|  |  | NC\_004578.1/3679925-3680123  | UUUACCAUGCC**G****G****C****C****G**..**U**.**C**..**G**.**G**.U...**U****U**.**C**.**C**---...................................GAGA............---**G****G**.**A****A****C**..U....AA..**C****A**..**G**..**G**.G..AA.**U**..**U**.**C**.**G**-.---CCAGCUUUGUUUCAAAGGCC.................................................................................................................................................A**A**AA.....-**C****G****A**.**A****A****C****U****G****C**C..C.CC..**G****C****A****A****C****U****G****U**AG..**G****C****A****U**.....................................................CGAGCC**U****G****C****U****C****C**AAGACU.........................................................................................**G****C****C**A...**C**.**U****G****G****A****U**.................UCAG-................................................................................**A****U****C****C****G****G**G.A.A.**G****G****C**..C**G****G****A**..**G****C****A**UGGUGAUGAC.......................................................................................................**A****U****G****C**...C.**A**.....**G**.**U**....**C****A****G**....**G**AG..A**C**.**C**..**U**.....G...**C****C**.**G**..**A**.**C****C****C****G****A**UUCAACCAACU | |
|  |  | NC\_004578.1/3542535-3542311  | GUAGCCUUGCC**G****G****U****U****C**..**G**.**A**..**G**.**G**.UU..**C****U**.**U**.**C****A****U****G**U..................................GUAA..AGCGUUACGA**C****A****U****G****A**.**A****G****C**..U....AA..**G****A**C.**G**..**G**.G..AA.**U**..**G**.**C**.**G****G**.**U**AC.....................................................................................................................................................................-**A**UG.....**C****C****G****C**.**A****G****C****U****G****C**C..C.CC..**G****C****A****A****C****U****G****U**AA..**A****C****G****G**U....................................................CAUGUU**C****A****U****U****G****C**ACA--..........................................................................................**G****C****C**A...**C**.**U****G****C**--.................-UGCG................................................................................--**G****C****G****G**G.A.A.**G****G****C**...**G****C****G**..**A****U****G**AAUGUGUGUC.........................................................................GGCAAGGUGUUCAACGCCCGCCACACGCUG**C****C****G****U**...G.**A**.....**G**.**C**....**C****A****G**....**G**AG..A**C**.**C**..**U**.....G...**C****C**.**U**..**C**.**G****A****A****C****C**GGGCUGACAAA | |
|  |  | NC\_003888.3/1037849-1038073  | AGGCUGGCCCG**U****G****C****A****G**..**C**.**U**..**G**.**G**.UU..**C****G**.**C**.**C****C****C****G**UCC................................GCCA..........GG**C****G****G****G****A**.**U****G****C**..GUCGCAA..**G****A**..**G**..**G**.G..AA.**C**..**C**.**C**.**G****G**.**U**GG.....................................................................................................................................................................G**A**AU.....**C****C****G****G**.**G****A****C****U****G****C**-..C.CC..**G****C****A****G****C****G****G****U**GA..**G****C****G****G**GA...................................................ACGACC**G****C****C****G****U****C**AUAC-..........................................................................................-**G****C**A...**C**.**U****G****G****G****C**CCGACG...........UACCG...............................................................................G**G****C****C****C****G****G**G.A.A.**G****C**-...**G****A****C**..**G****G****C**CAGUAGGUGU..................................................................................CCUCCGGACAGGAGGGUGGGC**C****C****G****C**...G.**A**.....**G**.**U**....**C****C****G**....**A**AG..A**C**.**C**..**U**.....G...**C****C**.**A**..**C**.**C****U****G****C****C**CGCGCGCGGAC | |
|  |  | NC\_003888.3/1045879-1046125  | ACGCUGAUGCC**C****G****C****A****G**..**U**.**U**..**G**.**G**.U...**U****C**.**G**.**C****G****C****C**UCCUGUCCGAUCAG.....................GUCU........CGGC**G****G****C****G****C**.**G****A****C**..GC...AA..**G****A**..**G**..**G**.G..AA.**C**..**C**.**C**.**G****G**.**U**GG.....................................................................................................................................................................G**A**AU.....**C****C****G****G**.**G****A****C****U****G****U**-..C.CC..**G****C****A****G****C****G****G****U**GA..**G****U****G****G**GA...................................................ACGAAA**G****C****C****G****U****C**AACA-..........................................................................................-**G****C**A...**C**.**U****G****G****G****C**CCCAG............AUGAG...........................................................................UUGGA**G****C****C****C****G****G**G.A.A.**G****C**-...**G****A****C**..**G****G****C**CGGUAGGUGC.......................................................................CCGCCGGUGAUCCGUGUCCCCGGUGAGCGCGC**C****C****A****C**...G.**A**.....**G**.**U**....**C****C****G**....**A**AG..A**C**.**C**..**U**.....G...**C****C**.**A**..-.**C****U****G****C****G**CCCGUACGCGA | |
|  |  | NZ\_AAIT01000002.1/180320-180125  | CUACCAAGUCG**C****U****G****C****A**..**U**.**C**..**G**.**G**.U...**U****U**.**C**.**C**---...................................GCAA............---**G****G**.**A****A****U**..G....AA..**A****A**..**G**..**G**.G..AA.**U**..**C**.**C**.**A****G**.**C**GCGCCCGAUGGGCGC........................................................................................................................................................G**A**AA.....**C**-**G****G**.**A****A****C****U****G****C**C..C.CC..**G****C****A****A****C****U****G****U**AG..**G****C****G****G**.....................................................CGAGCG**C****G****A****U****C****U**GGCAG..........................................................................................**G****C****C**A...**C**.**U****G****G****G****C**.................ACGAU................................................................................**G****U****C****C****G****G**G.A.A.**G****G****C**..A**G****G****A**..**U****C****C**GCGCAACGAC.......................................................................................................**C****C****G****C**...C.**A**.....**G**.**U**....**C****A****G**....**G**AG..A**C**.**C**..**U**.....G...**C****C**.**G**..**G**.**C****G****C****G****A**AUUCACCAGAC | |
|  |  | NC\_003030.1/1509950-1510136  | UGCUACUAAAA**U****U****U****G****U**..**A**.-..**G**.**G**.U...**U****C**.**A**.**A****C****U****G**AG.................................GAGU..........CU**U****A****G****U****U**.**G****A****U**..U....AA..**A****A**..**A**..**G**.G..AA.**U**..**C**.**A**.**G****G**.**U**GA.....................................................................................................................................................................A**A**AG.....**C****C****U****G**.**A****G****C****G****G****U**-..C.CC..**G****C****C****A****C****U****G****U**AAU.**A****A****A****G**.....................................................GAGUUU**A****A****G****U****A****C**AAUAU..........................................................................................**G****U****C**A...**C**.**U****G****G**--.................GAAA-................................................................................--**C****U****G****G**G.A.A.**G****G****C**...**G****U****A**..**C****U****U**AAGCAAUGAU.......................................................................................................**U****U****U****U**...G.**A**.....**G**.**C**....**C****A****G**....**G**AU..A**C**.**U**..**U**.....G...**C****C**.**A**..**U**.**A****U****U****C****U**AGUAUGUUUUU | |
|  |  | NC\_000853.1/84307-84124  | AGCCUCCCUCA**C****C****G****U****G**..**C**.-..**G**.**G**.U...**A****C**.**C**.**C**---...................................UUCG............---**G****G**.**G****U****U**..C....AA..**A****G**..**G**..**G**.G..AA.**G**..**C**.**C**.**G****G**.**U**GA.....................................................................................................................................................................A**A**AU.....**C****C****G****G**.**C****G****C****G****G****G**-..G.CC..**G****C****C****A****C****C****G****U**GA..**C****C****G****G**G....................................................GACGAA**A****C****C****C****G****C**AGAAC..........................................................................................**G****C****C**A...**C**.**U****G****G****G****G**CG...............AUCA-................................................................................**C****C****C****U****G****G**G.A.A.**G****G****C**...**G****C****G**..**G****G****G**AGUAGGAUGA......................................................................................................U**C****C****G****G**...A.**A**.....**G**.**C**....**C****G****G**....**G**AA..A**C**.**C**..**C**.....G...**C****C**.**C**..**G**.**C****G****G****U****G**AAGGGGAACCA | |
|  |  | NC\_004557.1/2416636-2416459  | AUUUAAUAUUA**G****A****U****U****U**..**U**.**A**..**G**.**G**.U...**G****U**.**G**.**A****U****C**-...................................UUAU............-**A****A****U****U**.**A****C****U**..U....AA..**A****A**..**G**..**A**.G..AA.**A**..**G**.**U**.**G****G**.**U**GA.....................................................................................................................................................................A**A**AU.....**C****C****A****C**.**U****A****C****A****G****C**C..C.CC..**G****C****U****A****C****U****G****U**AA..**U****A****G**-.....................................................UGGACG**A****U****U****C****C****U**AUUA-..........................................................................................**U****C****C**A...**C**.**C****A****G**--.................UUAA-................................................................................--**C****U****G****G**G.A.A.**G****G****A**...**A****G****G**..**A****U****A**AGGAAGAAA-.......................................................................................................-**C****U****A**...A.**A**.....**G**.**U**....**C****A****G**....**G**AG..A**A**.**C**..**U**.....G...**C****C**.**U**..**A**.**A****A****A****U****A**UAUUACUUCGG | |
|  |  | NC\_003063.1/1633405-1633640  | CUUAUGUGAGA**A****A****G****C****G**..**A**.**C**..**G**.**G**.U...**U****C**.**C**.**U****A****C****A**GCC................................GAAA.........GGC**G****A****A****G****G**.**G****A****U**..U....AA..**U****A**..**G**..**G**.G..AA.**C**..**A**.**U**.**G****G**.**U**GCGGGCGAUCUUUUUCGUCC...................................................................................................................................................A**A**UG.....**C****C****U****U**.**G****G****C****U****G****C**C..C.CC..**G****C****A****A****C****U****G****U**AA..**G****C****G****G**A....................................................UUGUUG**U****U****C****A****U****C**CCAGUGACGCUUGAAGGCGUCAU........................................................................**G****C****C**A...**C**.**U****G****U****U****U**U................UUUCG..............................................................................GA**A****U****G****C****G****G**G.A.A.**G****G****C**..A**G****A****U**..**G****A****G**GGACGCAAAU.......................................................................................................**C****C****G****U**...G.**A**.....**G**.**C**....**C****A****G**....**G**AG..A**C**.**C**..**U**.....G...**C****C**.**G**..**U**.**C****A****A****A****A**UGGAAACCAUC | |
|  |  | NC\_003305.1/441351-441116  | CUUAUGUGAGA**A****A****G****C****G**..**A**.**C**..**G**.**G**.U...**U****C**.**C**.**U****A****C****A**GCC................................GAAA.........GGC**G****A****A****G****G**.**G****A****U**..U....AA..**U****A**..**G**..**G**.G..AA.**C**..**A**.**U**.**G****G**.**U**GCGGGCGAUCUUUUUCGUCC...................................................................................................................................................A**A**UG.....**C****C****U****U**.**G****G****C****U****G****C**C..C.CC..**G****C****A****A****C****U****G****U**AA..**G****C****G****G**A....................................................UUGUUG**U****U****C****A****U****C**CCAGUGACGCUUGAAGGCGUCAU........................................................................**G****C****C**A...**C**.**U****G****U****U****U**U................UUUCG..............................................................................GA**A****U****G****C****G****G**G.A.A.**G****G****C**..A**G****A****U**..**G****A****G**GGACGCAAAU.......................................................................................................**C****C****G****U**...G.**A**.....**G**.**C**....**C****A****G**....**G**AG..A**C**.**C**..**U**.....G...**C****C**.**G**..**U**.**C****A****A****A****A**UGGAAACCAUC | |
|  |  | NC\_003366.1/248249-248449  | AUUAAAUAUUU**A****G****A****A****A**..**U**.**A**..**G**.**G**.U...**U****A**.**A**.**A****U****A****G**UUAC...............................AUUU........GUAA**C****U****A****U****A**.**U****A****U**..U....AA..**A****A**..**G**..**G**.G..AA.**G**U.**U**.**G**.**G****G**.**U**UU.....................................................................................................................................................................A**A**AU.....**C****C****C****A**.**C****G****C****G****G****U**-..C.CC..**G****C****C****G****C****U****G****U**AA..**U****A****G****A**.....................................................GGAGCU**U****U****U****U****G****U**ACUUUAA........................................................................................**G****C****C**A...**C**.**U****G****G****A****A**U................AUAAU...............................................................................A**U****U****U****U****G****G**G.A.A.**G****G****C**..C**A****C****A**..**A****A****A**AGUGAUGAU-.......................................................................................................**A****C****U****U**...G.**A**.....**G**.**C**....**C****A****G**....**A**AG..A**C**.**C**..**U**.....G...**C****C**.**U**..**A**.**U****U****U****U****U**AAAACAUCAAG | |
|  |  | NC\_002977.5/2496905-2496675  | GUGUGCGCUCU**G****C****U****U****U**..**C**.**G**..**G**.**G**.UG..**C****U**.**C**.**G****G****C****C**G..................................GCAU.....UCGCCGC**G****U****C****C****G**.**G****G****U**..U....AA..**A****C**..**G**..**G**.G..AA.**G**..**C**.**C**.**G****G**.**U**GCGGCGGAGGGGAGCCUCCGGC.................................................................................................................................................A**A**GG.....**C****C****G****G**.**C****G****C****U****G****C**C..C.CC..**G****C****A****A****C****G****G****U**AA..**G****C****G****A**G....................................................UCAAAC**C****C****C****A****U****C**ACGAC..........................................................................................**G****C****C**A...**C**.**U****G****C****G****A**GG...............GUUGG..............................................................................CC**U****U****G****C****G****G**G.A.A.**G****G****C**...**G****A****U**..**G****G****G**CCGGCACGGC...............................................................................................CGUGUCGC**U****C****G****C**...A.**A**.....**G**.**U**....**C****C****G**....**G**AG..A**C**.**C**..**G**.....G...**C****C**.**C**..**G**.**U****G****G****C****A**GUGGUGCUGAC | |
|  |  | NC\_007005.1/3613713-3613489  | GUAGCCUUGCC**G****G****U****U****C**..**G**.**A**..**G**.**G**.UU..**C****U**.**C**.**C****C****C****G**G..................................GUAA..AACAUCACCC**A****G****U****G****G**.**A****G****C**..U....AA..**G****A**C.**G**..**G**.G..AA.**U**..**G**.**C**.**G****G**.**U**AC.....................................................................................................................................................................-**A**AG.....**C****C****G****C**.**A****G****C****U****G****C**C..C.CC..**G****C****A****A****C****U****G****U**AA..**A****C****G****G**U....................................................CAUGUU**C****A****U****U****G****C**ACA--..........................................................................................**G****C****C**A...**C**.**U****G****C**--.................-UGCG................................................................................--**G****C****G****G**G.A.A.**G****G****C**...**G****C****G**..**A****U****G**AAUGCGUGUC.........................................................................GGCACAUUAUUGAUUGCCUGCCGCACGCCG**C****C****G****U**...G.**A**.....**G**.**C**....**C****A****G**....**G**AG..A**C**.**C**..**U**.....G...**C****C**.**U**..**C**.**G****A****A****C****G**GGCCACACAUG | |
|  |  | NC\_002977.5/2108769-2109006  | UAAUCCGCGCC**C****U****U****C****A**..**C**.**A**..**G**.**G**.UG..**U****C**.**G**.**A****C****C****G**GCG................................GCCU.........GGC**C****G****G****U****C**.**G****G****U**..G....AA..**A****C**..**G**..**G**.G..AA.**G**..**C**.**C**.**G****G**.**U**GCGGAUACCUUGUCC........................................................................................................................................................C**A**AU.....**C****C****G****G**.**C****G****C****U****G****C**C..C.CC..**G****C****A****A****C****G****G****U**GA..**U****U****G****A**GCCGAAACGUACCAAACGUUC................................CGAGGC**G****G****A****U****U****C**CUCAA..........................................................................................**G****C****C**A...**C**.**U****G****C****G****C**.................GCCU-................................................................................**G****C****G****C****G****G**G.A.A.**G****G****C**..G**G****A****A**..**C****A****C**CGCCCGGCGU...............................................................................................CAUGCCGC**U****C****A****U**...C.**A**.....**G**.**C**....**C****C****G**....**G**AG..A**C**.**C**..**G**.....G...**C****C**.**U**..**G**.**C****G****A****U****G**ACAUGAAGACC | |
|  |  | NC\_002967.9/2086789-2086586  | AAGGUUAUAGA**C****A****A****A****U**..**A**.**A**..**G**.**G**.U...**U****C**.**U**.**U****A****A****A**U..................................UAAU...........U**U****U****U****A****A**.**G****A****U**..U....AA..**A****A**..**G**..**G**.G..AA.**A**..**C**.**C**.**G****G**.**U**GA.....................................................................................................................................................................A**A**AA.....**C****C****G****G**.**C****A****C****A****G****C**C..C.CC..**G****C****U****A****C****U****G****U**AA..**U****U****G**-.....................................................AGUUUG**U****U****U****A****U****C**AUAAUGCAUCUAUGUAUUGGAAGCAUAA...................................................................**G****C****C**A...**C**.**U****G****U**--.................-UAAU................................................................................--**A****U****G****G**G.A.A.**G****G****C**...**G****A****U**..**A****A****A**CUUUAAAU--.......................................................................................................-**C****A****U**...A.**A**.....**G**.**U**....**C****A****G**....**G**AG..A**C**.**C**..**U**.....G...**C****C**.**U**..-.**A****U****U****U****G**UAUUACGAAAU | |
|  |  | NZ\_AAIC01000019.1/13368-13557  | CGAUUUUUGAU**A****A****A****G****C**..**A**.**G**..**G**.**A**.U...**A****C**.**C**.**C****C****U**-...................................UGAC............-**A****G****G****G**.**G****U****U**..U....AA..**A****A**..**G**..**G**.G..AA.**U**..**C**.**A**.**C****G**.**U**GA.....................................................................................................................................................................G**A**AU.....**C****G****U****G**.**A****G****C****U****G****A**-..C.GC..**G****C****A****A****C****U****G****U**AA..**G****C****C****C**ACCGAG...............................................AUGUUU**U****G****C****G****G****C**AAAGU..........................................................................................**G****C****C**A...**C**.**U****G****U****C****C**CG...............AUGAA..............................................................................CG**G****G****A****U****G****G**G.A.A.**G****G****C**...**G****U****C**..**G****U****A**AA--------.....................................................................................................AC**G****G****G****U**...A.**A**.....**G**.**C**....**C****A****G**....**G**AG..A**C**.**C**..**U**.....G...**U****C**.**U**..**U**.**G****U****U****U****U**UUAUCCGGAAG | |
|  |  | NZ\_AAEW01000022.1/39264-39081  | CGACAACAAAC**G****A****U****U****U**..**C**.**A**..**G**.**A**.U...**G****C**.**U**.**C**---...................................UCAC............---**G****A**.**G****C****U**..U....AA..**U****A**..**G**..**G**.G..AA.**C**..**C**.**C**.**U****G**.**U**GU.....................................................................................................................................................................A**A**UU.....**C****A****G****G**.**G****A****C****G****G****G**-..C.CC..**G****C****C****G****C****U****G****U**AA..**C****C****G****A**G....................................................GACGAA**A****G****C****C****G****C**UUGAU..........................................................................................**G****U****C**A...**C**.**U****G****C****C****G**.................AAAUU................................................................................**C****G****G****U****G****G**G.A.A.**G****A****C**...**G****U****G**..**G****C****C**GGUAGGAUGA......................................................................................................U**C****C****G****G**...A.**A**.....**G**.**U**....**C****A****G**....**A**AG..A**C**.**C**..**U**.....G...**U****C**.**U**..**G**.**A****A****A****U****U**UGGUAAUGCGC | |
|  |  | NZ\_AAIP01000003.1/46080-46299  | CCCCGCGCUGA**U****C****G****C****G**..**C**.**C**..**G**.**G**.U...**G****C**.**C**.**C**---...................................GAAA............---**G****G**.**G****C****U**..U....AA..**U****A**..**G**..**G**.G..AA.**C**..**G**.**C**.**G****G**.**U**GGGCGCGCCGGAAGGCGCUCC..................................................................................................................................................G**C**AA.....**C****C****G****A**.**G****G****C****U****G****U**C..C.CU..**G****C****A****A****C****U****G****U**AA..**G****C****G****G**C....................................................GAGCGC**G****A****U****G****C****A**CCGUCCGGGCCCAAGGCCCGGACA.......................................................................**G****C****C**A...**C**.**U****G****G****G**-.................GCAA-................................................................................-**C****C****C****G****G**G.A.A.**G****G****U**..G**U****G****C**..**A****U****C**AAGCCCUGAC.......................................................................................................**C****C****G****C**...G.**A**.....**G**.**C**....**C****A****G**....**G**AG..A**C**.**C**..**U**.....G...**C****C**.**G**..**G**.**C****G****U****G****G**GUCGCUCAAUG | |
|  |  | NZ\_AAAT03000001.1/1640163-1640384  | CCUACACAACU**U****G****U****U****A**..**C**.**G**..**G**.**G**.UG..**C****C**.**C**.----...................................CUCA..........CA----**G**.**G****G****U**..G....AA..**A****C**..**G**..**G**.G..AA.**A**..**C**.**C**.**G****G**.**U**GAAUCAUGUGCUUUACUCUAAAGCCAUGU..........................................................................................................................................C**A**GU.....**C****C****G****G**.**U****G****C****U****G****C**C..C.CC..**G****C****A****A****C****G****G****U**AA..**G****C****G****A**.....................................................--GCGA**A****G****C****G****U****C**AGA--..........................................................................................**U****C****C**A...**C**.**U****G****U****G****C**C................-CACG...............................................................................G**G****C****A****U****G****G**G.A.A.**G****G****U**...**G****A****C**..**G****C****U**UGCAGGUCGG.........................................................................................CAGACAGCCACCCC**U****C****G****U**...G.**A**.....**G**.**C**....**C****C****G**....**G**AG..A**C**.**C**..**G**.....G...**C****C**.**C**..**G**.**C****A****A****C****A**CACAGUGCGCA | |
|  |  | NZ\_AAAT03000001.1/1639264-1639054  | GUAGCCUUGCG**C****U****U****U****C**..**G**.**A**..**G**.**G**.UU..**C****U**.**U**.**C****G****G****C**...................................CCAC............**G****C****C****G****A**.**A****G****C**..U....AA..**G****A**A.**G**..**G**.G..AA.**C**..**G**.**C**.**G****G**.**U**CC.....................................................................................................................................................................-**A**AG.....**C****C****G****C**.**G****G****C****U****G****C**C..C.CC..**G****C****A****A****C****U****G****U**GA..**A****C****G****G**U....................................................GCUGUU**C****G****C****U****G****C**CAC--..........................................................................................**G****C****C**A...**C**.**U****G****C****C****A**AC...............UCAAU.........................................................................CCAGAGC**C****A****G****C****G****G**G.A.A.**G****G****C**...**G****C****A**..**G****C****G**AAUACCGGGC..........................................................................................CAAGGCCCGAACA**C****C****G****U**...C.**A**.....**G**.**C**....**C****A****G**....**G**AG..A**C**.**C**..**U**.....G...**C****C**.**U**..**C**.**G****U****C****A****C**AGAUUCUCACU | |
|  |  | NZ\_AADX02000001.1/76303-76080  | AUAGUCCACAC**C****A****U****C****U**..**A**.**C**..**G**.**G**.UG..**U****C**.**C**.**G****G****C****G**A..................................UUGU.....UCGAUCC**A****G****C****C****G**.**G****A**-..-....AA..**C****A**..**G**..**G**.G..AA.**U**..**G**.**C**.**G****G**.**U**CAAGGGAAUAUCCUGU.......................................................................................................................................................C**A**AG.....**C****C****G****C**.**A****G****C****U****G****C**C..C.CC..**G****C****A****A****C****U****G****U**GA..**C****C****A****G**U....................................................GAGCGG**C****A****A****G****C****C**AAAUAGAAG......................................................................................**G****U****C**A...**C**.**U****G****G****G****C**CUGGAAGU.........UUGAG................................................................................**G****C****C****C****G****G**G.A.A.**G****A****C**..A**G****G****C**..**U****G****U**CCGCGUGGAC.......................................................................................................**C****U****G****G**...G.**A**.....**G**.**C**....**C****A****G**....**G**AA..A**C**.**C**..**U**.....G...**C****C**.**G**..**U**.**A****G****A****U****C**AUUUUCAUAAC | |
|  |  | NC\_002932.3/409822-409610  | UACUUCAUCCG**A****U****U****A****U**..**G**.**U**..**G**.**G**.U...**G****C**.**C**.**C****G****C****C**AUG................................GAAA..........CA**U****A****C****G****G**.**G****C****U**..U....AA..**A****A**..**G**..**G**.G..AA.**U**..**C**.**C**.**G****G**.**U**GA.....................................................................................................................................................................G**A**GU.....**C****C****G****G**.**A****A****C****A****G****U**A..C.CC..**G****C****U****G****C****U****G****U**AAU.**U****C****C****G**CGCUGGCCGCAAGGCUGGCGA................................CAAGGU**U****U****G****C****C****G**ACAAU..........................................................................................**G****C****C**A...**C**.**U****G****U****C****C**CG...............UUCAG................................................................................**G****G****A****U****G****G**G.A.A.**G****G****C**...**C****G****G**..**C****A****G**AAUCC-----.......................................................................................................**G****G****G****A**...A.**A**.....**G**.**U**....**C****A****G**....**A**AG..A**C**.**C**..**U**.....G...**C****C**.**U**..**C**.**A****U****A****U****U**UUUUGGCUUCG | |
|  |  | NC\_002939.4/3297128-3296929  | GAUAAGGUGUA**G****G****G****C****U**..**C**.**A**..**G**.**G**.CA..**G****U**.**U**.**U****C****A****A**UCUGAA.............................GAAG.......CAUCC**U****U****C****C****G**.**G****C****G**..U....A-..-**A**..**G**..**G**.G..AA.**A**..**C**.**C**.**G****G**.**U**GC.....................................................................................................................................................................A**A**UA.....**C****C****G****G**.**U****G****C****U****G****C**-..C.CC..**G****C****A****A****C****U****G****U**GA..**G****G****G****G**G....................................................GACCGA**U****G****C****C****G****C**AGUGAU.........................................................................................**G****C****C**A...**C**.**U****G****U****C****C**.................-GAAG................................................................................**G****G****A****C****G****G**G.A.A.**G****G****C**...**G****C****G**..**G****C****A**GGCGGGAUGA......................................................................................................U**C****C****C****C**...G.**A**.....**G**.**C**....**C****A****G**....**G**AG..A**C**.**C**..**U**.....G...**G****C**.**U**..**G**.**G****A****A****G****G**CGACAACACAC | |
|  |  | NC\_002947.3/2765223-2765025  | UCUACCAUGCG**G****G****C****C****G**..**C**.**C**..**G**.**G**.U...**U****U**.**C**.**C**---...................................ACCA...........C---**G****G**.**A****A****C**..U....AA..**C****A**..**G**..**G**.G..AA.**U**..**C**.**C**.**C****A**.**G**GCCUGCCAAUACAGG........................................................................................................................................................C**C**AA.....**U****C****G****G**.**A****A****C****U****G****C**C..C.CC..**G****C****A****A****C****U****G****U**AG..**G****U****G****C**.....................................................CGAGCC**U****G****C****U****C****C**AUCGAU.........................................................................................**G****C****C**A...**C**.**U****G****G****G****C**.................CUGCC................................................................................**G****C****C****C****G****G**G.A.A.**G****G****C**..C**G****G****A**..**G****C****C**GGGCCGUGAC.......................................................................................................**G****C****A****C**...C.**A**.....**G**.**U**....**C****A****G**....**G**AG..A**C**.**C**..**U**.....G...**C****C**.**G**..**G**.**C****C****U****A****C**AUUCACCAACC | |
|  |  | NZ\_AAAJ03000004.1/222188-222008  | AGGCAUAAUAC**A****G****C****C****G**..**C**.**C**..**G**.**G**.UU..**G****C**.**C**.**C**---...................................UUCG............---**G****G**.**G****C****C**..G....AA..**G****A**..**G**..**G**.G..AA.**C**..**A**.**C**.**A****G**.**U**GC.....................................................................................................................................................................C**A**--.....**C****U****G****U**.**G****G****C****U****G****C**C..C.CC..**G****C****A****A****C****U****G****U**AU..**G****C****A****G**CG...................................................AGUCCG**C****C****U****G****C****G**CUUCAC.........................................................................................**G****C****C**A...**C**.**U****G****G****U**-.................-UCG-................................................................................-**A****C****C****G****G**G.A.A.**G****G****C**...**C****G****C**..**A****G****C**GGACCAUGAC.......................................................................................................**C****U****G****C**...C.**A**.....**G**.**C**....**C****A****G**....**G**AG..A**C**.**C**..**U**.....G...**C****C**.**G**..**G**.**C****G****A****G****A**CUGUUCGUGCC | |
|  |  | NZ\_AAAE01000010.1/2515-2315  | GGCUACUCAAU**C****C****U****C****G**..**U**.**C**..**G**.**G**.U...**U****U**.**C**.**C**---...................................GUAA............---**G****G**.**A****A****U**..U....GA..**A****A**..**G**..**G**.G..AA.**U**..**C**.**C**.**G****C**.**C**GCCGGACCUGUCCGGC.......................................................................................................................................................G**A**A-.....**C****C****G****G**.**A****A****C****U****G****C**C..C.CC..**G****C****A****A****C****U****G****U**AG..**G****C****G****G**C....................................................GAGGAG**C****U****C****G****C****G**AUCAAA.........................................................................................**G****C****C**A...**C**.**U****G****U****G****G**CC...............UCAAG................................................................................**C****C****A****U****G****G**G.A.A.**G****G****C**...**C****G****C**..**G****C****C**AGCCCUGUGA......................................................................................................C**C****C****G****C**...C.**A**.....**G**.**U**....**C****A****G**....**G**AG..A**C**.**C**..**U**.....G...**C****C**.**G**..**G**.**C****G****U****U****C**GAUCUCAACGC | |
|  |  | NZ\_AAAW03000016.1/17753-17574  | GAAUAUCAGGC**G****G****A****A****U**..**A**.**G**..**G**.**G**.U...**U****G**.**C**.----...................................UUAA..........AU----**G**.**C****A****U**..U....AA..**U****A**..**G**..**G**.G..AA.**C**U.**C**.**C**.**G****G**.**U**GU.....................................................................................................................................................................G**A**AG.....**C****C****G****G**.**G****A****C****A****G****C**-..C.CC..**G****C****U****A****C****U****G****U**AA..**G****A****A**-.....................................................GGACGA**G****U****C****C****C****U**UAGAGGA........................................................................................**U****C****C**A...**C**.**U****G****G****U**-.................GAAA-................................................................................-**A****C****C****G****G**G.A.A.**G****G****U**..A**A****G****G**..**G****A****U**GAGGAUGAG-.......................................................................................................-**U****U****C**...A.**A**.....**G**.**U**....**C****A****G**....**G**AU..A**C**.**C**..**U**.....G...**C****C**.**C**..**C**.**A****U****U****C****C**GGAAACACAAG | |
|  |  | NZ\_AABD03000009.1/120765-120953  | ACCUUUGUUCC**C****G****U****U****A**..**A**.**U**..**G**.**G**.U...**U****U**.**A**.**U****U****U****C**A..................................ACGU..........UU**G****A****A****A****U**.**A****A****U**..U....AA..**A****A**..**G**..**G**.G..AA.**G**..**C**.**C**.**G****G**.**U**GU.....................................................................................................................................................................A**A**AU.....**C****C****G****G**.**A****A****C****A****G****U**U..C.CC..**G****C****U****G****C****U****G****U**AA..**G****C****U****C**AUCAUU...............................................ACGUUA**U****A****U****U****U****C**AUUUA..........................................................................................**G****C****C**A...**C**.**U****G****A****A**-.................UUUA-................................................................................-**U****U****U****G****G**G.A.A.**G****G****C**..G**G****A****U**..**A****U****A**AC--------......................................................................................................A**G****A****G****U**...A.**A**.....**G**.**U**....**C****A****G**....**A**AG..A**C**.**C**..**U**.....G...**C****C**.**A**..**C**.**U****A****A****C****A**CACUAUUCGAA | |
|  |  | NC\_004342.1/1347834-1348014  | UCUUGGAACGG**A****A****A****A****C**..**U**.**U**..**G**.**U**.U...**U****A**.**U**.**U****C****U****C**...................................GUGA............-**U****G****A****G**.**G****A**-..-....--..-**A**..**G**..**G**.G..AA.**U**..**C**.**C**.**G****G**.**U**UC.....................................................................................................................................................................A**A**AU.....**C****C****G****G**.**A****G****C****U****G****A**A..C.CC..**G****C****A****G****C****U****G****U**AA..**U****C****G****C**CGAA.................................................UGAGAU**U****U****C****G****C****A**AUCAU..........................................................................................**G****C****C**A...**C**.**U****G****C****G****U**.................UAAAU................................................................................**A****C****G****C****G****G**G.A.A.**G****G****C**...**U****G****C**..**G****A****A**AUCG------.......................................................................................................**G****C****G****A**...A.**A**.....**G**.**C**....**C****A****G**....**A**AG..A**C**.**C**..**U**.....A...**A****C**.**A**..**A**.**G****U****A****A****A**AAAACAAACUA | |
|  |  | NC\_005823.1/2878773-2878593  | UCUUGGAACGG**A****A****A****A****C**..**U**.**U**..**G**.**U**.U...**U****A**.**U**.**U****C****U****C**...................................GUGA............-**U****G****A****G**.**G****A**-..-....--..-**A**..**G**..**G**.G..AA.**U**..**C**.**C**.**G****G**.**U**UC.....................................................................................................................................................................A**A**AU.....**C****C****G****G**.**A****G****C****U****G****A**A..C.CC..**G****C****A****G****C****U****G****U**AA..**U****C****G****C**CGAA.................................................UGAGAU**U****U****C****G****C****A**AUCAU..........................................................................................**G****C****C**A...**C**.**U****G****C****G****U**.................UAAAU................................................................................**A****C****G****C****G****G**G.A.A.**G****G****C**...**U****G****C**..**G****A****A**AUCG------.......................................................................................................**G****C****G****A**...A.**A**.....**G**.**C**....**C****A****G**....**A**AG..A**C**.**C**..**U**.....A...**A****C**.**A**..**A**.**G****U****A****A****A**AAAACAAACUA | |
|  |  | NZ\_AAIT01000007.1/212219-212415  | CGCUAUGGCGG**A****G****U****U****G**..**U**.**C**..**G**.**G**.U...**U****U**.**C**.**C**---...................................GCAA............---**G****G**.**A****A****U**..U....GA..**A****A**..**G**..**G**.G..AA.**U**..**C**.**C**.**G**-.---CCGGGGCAUCAGCCCC.....................................................................................................................................................G**A**AC.....-**C****G****G**.**A****A****C****U****G****C**C..C.CC..**G****C****A****A****C****U****G****U**AG..**G****C****G****G**.....................................................CGAGCA**G****G****G****U****G****C**GAAACA.........................................................................................**G****C****C**A...**C**.**U****G****U****G****G**C................CCAUG................................................................................**C****C****A****U****G****G**G.A.A.**G****G****C**..C**G****C****A**..**U****C****C**CUGCCGUGAC.......................................................................................................**C****C****G****U**...C.**A**.....**G**.**U**....**C****A****G**....**G**AG..A**C**.**C**..**U**.....G...**C****C**.**G**..**A**.**C****G****C****G****A**UGACCAACCCG | |
|  |  | NZ\_AAIK01000006.1/75708-75514  | UUUUUUACUGA**U****G****A****A****G**..**C**.**A**..**G**.**G**AU...**A****C**.**C**.**C****C**--...................................AUUA.........CAA--**G****G****G**.**G****U****U**..U....AA..**U****A**..**G**..**G**.G..AA.**U**..**C**.**A**.**C****G**.**U**GA.....................................................................................................................................................................A**A**AU.....**C****G****U****G**.**A****G****C****U****G****A**-..C.GC..**G****C****A****A****C****U****G****U**AAA.**G****C****C****G**AUCAC................................................GUGUUG**U****G****C****A****G****C**GUUAU..........................................................................................**A****C****C**A...**C**.**U****G****U****U****C**CGU..............UUGAU.............................................................................GCG**G****A****A****U****G****G**G.A.A.**G****G****U**...**G****C****U**..**G****C****A**CA--------.....................................................................................................AC**C****G****G****C**...A.**A**.....**G**.**C**....**C****A****G**....**G**AG..A**C**.**C**..**U**.....G..U**C****C**.**C**..**G**.**C****U****U****C****U**UUUCUUGUAAC | |
|  |  | NZ\_AAIJ01000009.1/44053-43832  | UGAUUUUGUAA**A****C****C****U****U**..**G**.**C**..**G**.**G**.U...**G****C**.**C**.**C****U****G****G**CUG................................GUCA.......GUCCU**G****C****C****G****G**.**G****C****U**..U....AA..**A****A**..**G**..**G**.G..AA.**U**..**C**.**C**.**G****G**.**U**GA.....................................................................................................................................................................A**A**AU.....**C****C****G****G**.**A****A****C****A****G****U**U..C.CC..**G****C****U****G****C****U****G****U**AA..**U****C****C****C**CUUCCGGUCUUCGGACCGGAACAU.............................CACAGU**C****U****G****C****A****G**AGAGA..........................................................................................**G****C****C**A...**C**.**U****G****U****C****G**C................GCACC...........................................................................CCGCG**C****A****A****C****G****G**G.A.A.**G****G****C**...**C****U****G**..**C****A****G**ACCGG-----.......................................................................................................**G****G****G****A**...G.**A**.....**G**.**U**....**C****A****G**....**A**AG..A**C**.**C**..**U**.....G...**C****C**.**G**..**U**.**A****A****U****G****G**AGAAUGCUUCG | |
|  |  | NC\_004603.1/3143153-3142951  | UAAUAUUUGCC**U****C****A****A****G**..**C**.**U**..**G**.**G**.U...**G****U**.**C**.**A****U****C****U**A..................................GCAA.........UCU**A****G****A****U****G**.**G****C****U**..G....AA..**A****U**..**G**..**G**.G..AA.**U**..**C**.**U**.**G****G**.**U**GA.....................................................................................................................................................................A**A**AU.....**C****C****A****G**.**A****A****C****U****G****A**-..C.GC..**G****C****A****G****C****G****G****U**AA..**A****A****G****A**.....................................................GAACGA**A****C****G****C****U****C**AUCAC..........................................................................................**G****A****C**A...**C**.**U****G****C****A****U**U................AGUUU................................................................................**A****U****G****U****G****G**G.A.A.**G****U****C**...**G****A****G**..**C****A****A**CUAGGUGGUG.............................................................................................UAAACCUCGC**U****C****U****U**...C.**A**.....**G**.**U**....**C****C****G**....**A**AU..A**C**.**C**..**U**.....G...**C****C**.**A**..**G**.**C****A****A****C****U**GAGCCACAGCA | |
|  |  | NC\_002937.3/721811-721630  | CCUAUGCCGCA**C****G****A****G****U**..**C**.**A**..**G**.**G**.U...**G****C**.**C**.----...................................CUGA..........GC----**G**.**G****C****U**..U....GA..**U****A**..**G**..**G**.G..AA.**U**..**C**.**C**.**C****G**.**U**GU.....................................................................................................................................................................G**A**AU.....**C****G****G****G**.**A****G****C****G****G****A**-..C.CC..**G****C****C****G****C****C****G****U**AA..**G****C****C****C**AUAA.................................................AAGACC**U****U****C****U****C****C**CCCGUGU........................................................................................**G****C****C**A...**C**.**U****G****G****A****C**.................GACC-................................................................................**G****U****C****C****G****G**G.A.A.**G****G****C**..C**G****G****A**..**G****C****A**GG--------.....................................................................................................UC**G****G****G****U**...G.**A**.....**G**.**C**....**C****G****G**....**A**AG..A**C**.**C**..**U**.....G...**C****C**.**U**..**G**.**A****C****A****C****G**UCCGAUGCCAU | |
|  |  | NZ\_AAAW03000045.1/22264-22444  | CCGAAUACAUA**G****C****U****U**-..**A**.**A**..**G**.**G**.UG..**C****C**.**C**.----...................................-AUA............----**G**.**G****G****A**..G....AA..**U****A**..**G**..**G**.G..AA.**A**..**C**.**C**.**G****G**.**U**AU.....................................................................................................................................................................A**A**GU.....**C****C****G****G**.**U****G****C****G****G****A**-..U.CC..**G****C****C****G****C****U****G****U**AA..**U****C****G****G**.....................................................AGACGA**A****U****C****C****C****C**AGAAU..........................................................................................**G****U****C**A...**C**.**U****G****U****C****U**UU...............UUUAG................................................................................**A****G****A****U****G****G**G.A.A.**G****G****C**...**G****U****G**..**G****G****A**GGAGGAUGAC.......................................................................................................**A****C****G****A**...G.**A**.....**G**.**C**....**C****A****G**....**A**AG..A**C**.**C**..**U**.....G...**C****C**.**U**..**U**.**U****U****A****G****A**AAGCUGAUUAA | |
|  |  | NC\_004557.1/768610-768791  | AAUAAUAUGAA**U****A****A****A****A**..**A**.**A**..**G**.**G**.U...**G****A**.**A**.**A****U**--...................................AGUU............--**A****U****U**.**U****C****U**..U....AA..**A****A**..**G**..**G**.G..AA.**G**C.**A**.**U**.**G****G**.**U**GA.....................................................................................................................................................................A**A**AU.....**C****C****A****U**.**C****A****C****G****G****U**-..C.CC..**G****C****C****A****C****U****G****U**AA..**U****C****A****G**G....................................................GAGUUA**G****G****U****U****U****U**A----..........................................................................................-**C****C**A...**C**.**U****G****U****U**-.................-AAUU................................................................................-**A****A****C****G****G**G.A.A.**G****G**-...**A****G****A**..**A****U****C**UAUAAAAACU..................................................................................................AUGAA**C****U****G****U**...A.**A**.....**G**.**U**....**C****A****G**....**G**AG..A**C**.**C**..**U**.....G...**C****C**.**U**..**U**.**U****U****U****U****U**AAUUAAACAUU | |
|  |  | NZ\_AAIJ01000008.1/51823-52037  | CGAUAUCAGAA**A****U****A****C****U**..**G**.**U**..**G**.**G**.U...**G****C**.**C**.**G****G****U****C**AU.................................CUCU.........CCU**G****A****C****C****G**.**G****U****U**..U....AA..**A****A**..**G**..**G**.G..AA.**U**..**C**.**C**.**G****G**.**U**GA.....................................................................................................................................................................A**A**AU.....**C****C****G****G**.**A****A****C****A****G****U**A..C.CC..**G****C****U****G****C****U****G****U**AA..**U****C****C****U**GUACCGGUGUUAAACCGGUGAG...............................CUAUGU**U****C****A****U****C****A**AAGAU..........................................................................................**G****U****C**A...**C**.**U****G****A****C****C**CGAGC............CCUCG................................................................................**G****G****U****C****G****G**G.A.A.**G****G****C**...**U****G****A**..**U****G****A**AA--------.....................................................................................................UC**A****G****G****A**...A.**A**.....**G**.**U**....**C****A****G**....**A**AG..A**C**.**C**..**U**.....G...**C****C**.**C**..**C**.**G****G****U****A****U**UAUUUCGCUUC | |
|  |  | NZ\_AAFH01000002.1/242976-242770  | UUAUAAUGCGC**G****G****C****U****U**..**C**.**A**..**G**.**G**.UG..**C****C**.**G**.**A****C****G****C**G..................................GCUU......UCCGCC**G****C****C****G****A**.**G****G****A**..U....AA..**A****C**..**G**..**G**.G..AA.**A**..**C**.**A**.**G****G**.**U**GCGUGGCUGCAAGCCAU......................................................................................................................................................C**A**UG.....**C****C****U****G**.**U****G****C****U****G****C**C..C.CC..**G****C****A****A****C****G****G****U**AA..**G****C****G****C**U....................................................AAACGG**U****C****C****A****U****C**ACACC..........................................................................................**G****C****C**A...**C**.**U****G****G****G**-.................-UUCG................................................................................-**C****C****C****G****G**G.A.A.**G****G****C**...**G****A****U**..**G****G****U**CUUACAA---.......................................................................................................**G****C****G****C**...G.**A**.....**G**.**C**....**C****C****G**....**G**AU..A**C**.**C**..**G**.....G...**C****C**.**U**..**G**.**G****G****C****C****C**UGUCGUGAAUA | |
|  |  | NZ\_AABI03000008.1/109312-109092  | UCAUGCGCCCG**C****U****U****C****A**..**U**.**A**..**G**.**G**.UG..**C****G**.**C**.**U****U****G****U**...................................GUAG............**A****C****A****A****G**.**U****G****U**..U....AA..**A****C**..**G**..**G**.G..AA.**G**..**U**.**A**.**G****G**.**U**GUCAUUGUGGCCACUAGCCAUAAUAUU............................................................................................................................................C**A**GU.....**C****C****U****A**.**C****G****C****U****G****C**C..C.CC..**G****C****A****A****C****G****G****U**GA..**U****U****G****G**U....................................................---UAA**A****G****C****U****A****U**UGCAU..........................................................................................**G****C****C**A...**C**.**U****G****C****G****A**.................UUUU-................................................................................**U****C****G****U****G****G**G.A.A.**G****G****U**.AA**A****U****A**..**G****C****C**CGCGCGUAUA................................................................................................GCGCUCG**C****C****A****U**...A.**A**.....**G**.**C**....**C****C****G**....**G**AU..A**C**.**C**..**G**.....G...**C****C**.**U**..**A**.**A****G****A****A****G**GUUAUUCGAGG | |
|  |  | NZ\_AAEW01000085.1/12025-11841  | GGAUCACGGUG**C****C****C****A****A**..**C**.**G**..**G**.**G**.A...**G****C**.**U**.**U****A****A****A**U..................................GUCU...........C**G****U****G****A****G**.**G****C****U**..U....GA..**U****A**..**G**..**G**.G..AA.**G**A.**G**.**G**.**G****G**.**U**GC.....................................................................................................................................................................A**A**CU.....**C****C****C****C**.**C****G****C****G****G****A**-..C.CC..**G****C****C****G****C****U****G****U**AA..**A****C****G**-.....................................................UGGACA**A****C****A****G****G****C**UCCAU..........................................................................................**G****C****C**A...**C**.**U****G****G****U**-.................-UUCG................................................................................-**A****C****C****G****G**G.A.A.**G****G****C**...**G****C****C**..**U****G****A**AGGAUGAAG-.......................................................................................................-**C****G****U**...A.**A**.....**G**.**C**....**C****A****G**....**A**AG..A**C**.**C**..**U**.....G...**C****C**.**U**..**G**.**A****U****C****C****C**AACACUCAAUG | |
|  |  | NC\_002516.1/3261435-3261184  | CAUAAUAGCGC**G****U****U****C****G**..**U**.**C**..**G**.**G**.U...**G****C**.**C**.**C****G****G****C**CCUUUCGC...........................GUUAGCGCGGGGCCAA**C****G****A****G****G**.**G****C****C**..G....AA..**G****A**..**G**..**G**.G..AA.**C**..**A**.**C**.**G****G**.**A**GCCGCGGUCUUUUCGAAGCCCGGGC..............................................................................................................................................C**U**AG.....**C****C****G****U**.**G****G****C****U****G****C**C..C.CC..**G****C****A****A****C****U****G****U**AU..**G****C****A****G**CC...................................................UGUAUU**C****G****C****G****C****C**AUUC-..........................................................................................**G****C****C**A...**C**.**U****G****G**--.................AUUA-................................................................................--**C****C****G****G**G.A.A.**G****G****C**...**G****G****C**..**G****C****G**AAGCGGAGGU...............................................................................UCCUCCCCCGGGUGGAACGCCGGG**C****U****G****C**...G.**A**.....**G**.**C**....**C****A****G**....**G**AG..A**C**.**C**..**U**.....G...**C****C**.**G**..**C**.**C****G****A****A****A**CCAGUCGCGAG | |
|  |  | NC\_003212.1/1132425-1132616  | UUAAAUAGGUC**U****U****A****U****G**..**U**.**U**..**G**.**G**.U...**G****G**.**A**.**A****U****G****U**...................................GUUU............**G****C****A****U****U**.**U****C****U**..G....AA..**A****G**..**A**..**G**.G..AA.**U**..**U**.**C**.**G****G**.**U**AU.....................................................................................................................................................................G**A**AG.....**C****C****G****G**.**A****A****C****U****G****C**C..C.CC..**G****C****A****A****C****U****G****U**AA..**G****G****U****G**.....................................................GACAAG**A****A****U****U****G****A**AAAUA..........................................................................................**A****C****C**A...**C**.**U****G****U****G****C**GU...............UUUUG..............................................................................AC**G****U****A****U****G****G**G.A.A.**G****G****U**...**U****C****A**..**A****U****U**GUUGAAUGA-.......................................................................................................**A****G****C****C**...A.**A**.....**G**.**U**....**C****A****G**....**G**AU..A**C**.**U**..**C**.....G...**C****C**.**A**..**A**.**A****U****A****A****G**ACGGAAGCAAC | |
|  |  | NC\_006347.1/3073837-3073630  | UAUAUUUGUCG**G****C****A****G****A**..**U**.**U**..**G**.**G**.U...**U****C**.**G**.**C****C****G****A**A..................................GCUU....UCCUGCUU**U****G****G****G****C**.**G****A****U**..U....AA..**A****A**..**G**..**G**.G..AA.**U**..**C**.**G**.**G****G**.**U**GU.....................................................................................................................................................................A**A**CU.....**C****C****C****G**.**G****A****C****A****G****U**-..C.CC..**G****C****U****G****C****U****G****U**GA..**A****G****C****U**UUAUUAU..............................................AAACAU**U****C****C****G****A****U**AGGAUACUCUUUG..................................................................................**G****C****C**A...**C**.**U****G****A****C****U**.................CGUGU................................................................................**A****G****U****C****G****G**G.A.A.**G****G****C**..G**U****U****C**..**G****G****A**AUGGA-----.......................................................................................................**A****G****C****U**...A.**A**.....**G**.**U**....**C****A****G**....**A**AG..A**C**.**C**..**U**.....G...**C****C**.**A**..**C**.**U****C****U****C****G**CUGAUAAGUCU | |
|  |  | NC\_002967.9/799355-799174  | CAAUUUUCAAA**C****C****A****U****A**..**C**.**A**..**G**.**G**.UG..**C****C**.**G**.----...................................AGAU...........U----**C**.**G****G****U**..U....AA..**A****A**..**G**..**G**.G..AA.**G**C.**A**.**C**.**G****G**.**U**GU.....................................................................................................................................................................A**A**UU.....**C****C****G****U**.**C****A****C****G****G****U**-..C.CC..**G****C****C****G****C****U****G****U**AA..**G****A****G****A**A....................................................UAGUCU**U****U****U****U****A****A**AAUAU..........................................................................................**G****U****C**A...**C**.**U****C****G****G**-.................GAAA-................................................................................-**U****C****G****G****G**G.A.A.**G****G****C**...**U****U****A**..**A****A****A**AGAUGAUGAA.......................................................................................................**G****C****U****C**..GA.**A**.....**G**.**U**....**C****A****G**....**A**AU..A**C**.**C**..**U**.....G...**C****C**.**U**..**G**.**U****A****A****A****G**GACUAAGCCUU | |
|  |  | NC\_002936.3/122538-122730  | AAUUGAAUAUG**G****U****C****U****G**..**C**.**C**..**G**.**G**.U...**G****C**.**U**.**U****U**--...................................GUAA..........CA--**A****A****A**.**G****C****U**..U....AA..**A****A**..**G**..**G**.G..AA.**G**..**C**.**C**.**U****G**.**U**UA.....................................................................................................................................................................G**A**AU.....**C****A****G****G**.**U****G****C****G****G****U**-..C.CC..**G****C****C****G****C****U****G****U**AU..**A****C****G****G**G....................................................GACGAA**A****A****C****U****G****G**AAUAC..........................................................................................**G****C****C**A...**C**.**U****G****U****U****U**CA...............UUAAA.............................................................................AUG**A****A****G****C****G****G**G.A.A.**G****G****C**...**C****C****G**..**G****U****G**AGUAGGUUGA......................................................................................................G**C****C****G****U**...A.**A**.....**G**.**C**....**C****A****G**....**A**AU..A**U**.**C**..**U**.....G...**C****C**.**G**..**G**.**U****A****U****A****G**AGUUUCGCGGA | |
|  |  | NZ\_AAAV02000001.1/129339-129573  | CCUUUCAUUGA**U****C****G****C****G**..**C**.**C**..**G**.**G**.U...**G****C**.**C**.**C****C****C**-...................................GAAA............--**G****G****G**.**G****C****U**..U....AA..**U****C**..**G**..**G**.G..AA.**U**..**G**.**C**.**G****G**.**U**GCGGAAGCUUGCUUCC.......................................................................................................................................................A**A**AU.....**C****C****G****C**.**G****G****C****U****G****U**C..U.CU..**G****C****A****A****C****U****G****U**AA..**G****C****G****G**AU...................................................AGCGCG**A****U****G****U****A****C**AUCGCUCCUCUUGGAAGGGGCA.........................................................................**G****C****C**A...**C**.**U****G****G****G****C**CGGACGC..........UAAAU........................................................................CAUGCGAG**G****U****C****U****G****G**G.A.A.**G****G****C**...**G****U****G**..**C****A****U**CAAGCUGUGA......................................................................................................C**C****C****G****U**...G.**A**.....**G**.**C**....**C****A****G**....**G**AG..A**C**.**C**..**U**.....G...**C****C**.**G**..**G**.**C****G****U****C****U**GGUCGCUCUUG | |
|  |  | NZ\_AABQ07000001.1/539786-539535  | CAUAAUAGCGC**G****U****U****C****G**..**U**.**C**..**G**.**G**.U...**G****C**.**C**.**C****G****G****C**CCUUUCGC...........................GUUAGCGCGGGGCCAA**C****G****A****G****G**.**G****C****C**..G....AA..**G****A**..**G**..**G**.G..AA.**C**..**A**.**C**.**G****G**.**A**GCCGCGGUCUUUUCGAAGCCCGGGC..............................................................................................................................................C**U**AG.....**C****C****G****U**.**G****G****C****U****G****C**C..C.CC..**G****C****A****A****C****U****G****U**AU..**G****C****A****G**CC...................................................UGUAUU**C****G****C****G****C****C**AUUC-..........................................................................................**G****C****C**A...**C**.**U****G****G**--.................AUUA-................................................................................--**C****C****G****G**G.A.A.**G****G****C**...**G****G****C**..**G****U****G**AAGCGGAGGU...............................................................................UCCUCCCCCGGGUGGAACGCCGGG**C****U****G****C**...G.**A**.....**G**.**C**....**C****A****G**....**G**AG..A**C**.**C**..**U**.....G...**C****C**.**G**..**C**.**C****G****A****A****A**CCAGUCGCGAG | |
|  |  | NZ\_AAAE01000151.1/45452-45646  | CACCACUUCCG**G****C****G****C****G**..**C**.**U**..**G**.**G**.UU..**C****C**.**U**.**G****U****C****C**...................................GACC............**G****G****A****C****A**.**G****G****C**..G....AA..**G****A**..**G**..**G**.G..AA.**U**..**G**.**C**.**C**-.--C.....................................................................................................................................................................C**A**GA.....-**G****G****C**.**A****G****C****C****G****C**C..C.CC..**G****C****G****A****C****C****G****U**GA..**C****C****G****G**.....................................................-AGAGG**U****U****G****C****C****C**AA---..........................................................................................**G****C****C**A...**C**.**U****G****G****C****C**.................GCAA-................................................................................**G****G****C****C****G****G**G.A.A.**G****G****C**...**G****G****G**..**C****C****G**CCGCGGAGCC.........................................................................................GCAAGGCCCCACAU**C****C****G****C**...A.**A**.....**G**.**C**....**C****G****G**....**G**AG..A**C**.**C**..**U**.....G...**C****C**.**A**..**G**.**C****G****C****U****G**AGACUUACGGG | |
|  |  | NC\_003155.3/2496008-2496226  | AGGCUGGGUGG**U****G****C****A****G**..**C**.**U**..**G**.**G**.U...**U****C**.**G**.**C****C****C****C**GUCC...............................GCCA.......GGCGG**G****A****G****G****C**.**G****U****C**..GU...AA..**G****A**..**G**..**G**.G..AA.**C**..**C**.**C**.**G****G**.**C**GG.....................................................................................................................................................................G**A**AU.....**C****C****G****G**.**G****A****C****U****G****C**-..C.CC..**G****C****A****G****C****G****G****U**GA..**G****U****G****G**GA...................................................ACGAAC**G****C****U****G****U****C**AUAC-..........................................................................................-**G****C**A...**C**.**U****G****G****G****C**CC...............AAAAG................................................................................**G****C****C****C****G****G**G.A.A.**G****C**-...**G****A****C**..**G****G****C**CAGUAGGUGU...................................................................................CCUCCGUACGGAGGGCGUGC**U****C****G****C**...G.**A**.....**G**.**U**....**C****C****G**....**A**AG..A**C**.**C**..**U**.....G...**C****C**.**C**..**G**.**C****U****G****C****C**CGUGCGCGACC | |
|  |  | NC\_003228.3/3136661-3136454  | UAUAUUUGUCG**G****C****A****G****A**..**U**.**U**..**G**.**G**.U...**U****C**.**G**.**C****C****G****A**A..................................GCUU....UCCUGCUU**U****G****G****G****C**.**G****A****U**..U....AA..**A****A**..**G**..**G**.G..AA.**U**..**C**.**G**.**G****G**.**U**GU.....................................................................................................................................................................A**A**CU.....**C****C****C****G**.**G****A****C****A****G****U**-..C.CC..**G****C****U****G****C****U****G****U**GA..**A****G****U****U**UUAUUAU..............................................AAACAU**U****C****C****G****A****C**AGGAUACUCUUUG..................................................................................**G****C****C**A...**C**.**U****G****A****C****U**.................CGUGU................................................................................**A****G****U****C****G****G**G.A.A.**G****G****C**..G**U****U****C**..**G****G****A**AUGGA-----.......................................................................................................**A****G****C****U**...A.**A**.....**G**.**U**....**C****A****G**....**A**AG..A**C**.**C**..**U**.....G...**C****C**.**A**..**C**.**U****C****U****C****G**CUGAUAAGUCU | |
|  |  | NC\_006513.1/2366730-2366982  | CGGGUGCGACG**U****G****U****U****U**..**C**.**A**..**G**.**G**.UG..**C****C**.**C**.**G****A****A****G**GCGA...............................ACCU.........CGC**C****G****G****A****A**.**G****G****U**..U....AA..**A****C**..**G**..**G**.G..AA.**A**..**C**.**A**.**G****G**.**U**GCGCGAACGGUCGUCCGACAGGGCCUUCGC.........................................................................................................................................C**A**UG.....**C****C****U****G**.**U****G****C****U****G****C**C..C.CC..**G****C****A****A****C****G****G****U**AA..**G****U****G****G**AC...................................................GCAAGG**C****G****C****A****U****C**CAACC..........................................................................................**G****C****C**A...**C**.**U****G****G****A****C**.................GACCA................................................................................**G****U****C****C****G****G**G.A.A.**G****G****C**...**G****A****U**..**G****C****G**ACCGGUCCGG.............................................................................CAUUCCCCGACGGGACGCCGGUCAGC**C****C****A****C**...G.**A**.....**G**.**C**....**C****C****G**....**G**AU..A**C**.**C**..**G**.....G...**C****C**.**C**..**G**.**A****A****C****G****C**GCUAUGGCUGA | |
|  |  | NC\_004663.1/2628653-2628440  | GUAUCUUUGCA**C****C****U****G****C**..**A**.**A**..**G**.**G**.U...**U****C**.**G**.**U****A****U****U**UGGAGUU............................GUCU....AACCUUCC**A****A****A****A****C**.**G****A****U**..G....AA..**A****A**..**G**..**G**.G..AA.**U**..**C**.**C**.**G****G**.**U**GU.....................................................................................................................................................................A**A**AU.....**C****C****G****G**.**G****A****C****A****G****U**A..C.CU..**G****C****U****G****C****U****G****U**AA..**G****U****C****C**UCUCUUUCGGGAGA.......................................UGAGUU**U****A****U****C****A****C**AUUCUUU........................................................................................**G****C****C**A...**C**.**U****G****G****U****G**.................ACAU-................................................................................**C****A****C****U****G****G**G.A.A.**G****G****C**...**G****U****G**..**A****U****A**AAUGG-----.......................................................................................................**G****G****A****C**...G.**A**.....**G**.**U**....**C****A****G**....**A**AG..A**C**.**C**..**U**.....G...**C****C**.**U**..**U**.**G****A****C****G****C**UGCAGAUUUAU | |
|  |  | NZ\_AAIJ01000009.1/40902-40711  | UUCUUAUUGGU**G****A****A****G****C**..**U**.**U**..**G**.**A**.U...**A****C**.**C**.**C****U****G**-...................................UAAA............-**A****A****G****G**.**G****U****U**..U....AA..**U****A**..**G**..**G**.G..AA.**U**..**C**.**A**.**C****G**.**U**GC.....................................................................................................................................................................A**A**AU.....**C****G****U****G**.**A****G****C****U****G****A**-..C.GC..**G****C****A****A****C****U****G****U**AAA.**G****C****C****G**AUUGA................................................AUGUUG**U****G****C****A****G****C**GUUAU..........................................................................................**G****C****C**A...**C**.**U****G****U****U****U**CGCC.............UCUUG..............................................................................CG**G****A****A****C****G****G**G.A.A.**G****G****U**...**G****C****U**..**G****C****A**GA--------.....................................................................................................AC**C****G****G****C**...A.**A**.....**G**.**C**....**C****A****G**....**G**AG..A**C**.**C**..**U**.....G...**U****C**.**C**..**U**.**G****C****U****U****C**UUUUUCUCUAA | |
|  |  | NC\_003888.3/1051400-1051583  | CAGACCGUAGU**A****U****C****A****G**..**C**.**G**..**G**.**G**.U...**C****A**.**U**.**C****G**--...................................-CCG............--**C****G****A**.**C****G****G**..G....AG..**A****C**..**A**..**G**.G..AA.**G**..**C**.**C**.**G****G**.**U**GU.....................................................................................................................................................................G**A**AU.....**C****C****G****G**.**C****A****C****G****G****U**-..C.CC..**G****C****C****A****C****U****G****U**GA..**C****C****G****G**.....................................................GGAGUG**C****A****C****C****C****U**UCGACAC........................................................................................**G****C****C**A...**C**.**U****G****C****G****C**.................-GCC-................................................................................**G****C****G****C****G****G**G.A.A.**G****G****C**..C**A****G****G**..**G****A****G**GAGCGUCGAU.......................................................................................................**C****C****G****G**...G.**A**.....**G**.**U**....**C****A****G**....**G**AC..A**C**.**U**..**G**.....G...**C****C**.**U**..**G**.**U****C****G****C****G**GGCCCGUUCCG | |
|  |  | NC\_004557.1/1024579-1024760  | AAAUGUUUUAU**G****A****A****A****A**..**U**.**A**..**G**.**G**.U...**U****U**.**A**.----...................................AUAA.........CUU----**U**.**A****A****U**..U....AA..**A****A**..**G**..**G**.G..AA.**G**U.**U**.**G**.**G****G**.**U**GA.....................................................................................................................................................................A**A**AU.....**C****C****C****A**.**C****G****C****G****G****U**-..A.CC..**G****C****C****G****C****U****G****U**AA..**G****A****G****A**G....................................................AAGUUU**U****C****U****U****C****U**AUUAU..........................................................................................**G****C****C**A...**C**.**U****G****U****U**-.................UUUU-................................................................................-**A****A****U****G****G**G.A.A.**G****G****C**...**A****G****A**..**A****G****A**AAAUUAUGAU.......................................................................................................**A****C****U****C**...G.**A**.....**G**.**C**....**C****A****G**....**A**AU..A**U**.**C**..**U**.....G...**C****C**.**U**..**A**.**U****U****U****U****A**UUCACUAUAGC | |
|  |  | NZ\_AABN02000002.1/14526-14344  | AUAACCCGAAU**C****G****A****U****U**..**C**.**A**..**G**.**G**.U...**G****C**.**C**.**C**---...................................UGCG............---**G****G**.**G****C****U**..U....GA..**U****A**..**G**..**G**.G..AA.**U**..**C**.**C**.**C****G**.**U**UA.....................................................................................................................................................................A**A**GU.....**C****G****G****G**.**A****G****C****G****G****A**-..C.CC..**G****C****C****G****C****C****G****U**AA..**G****U****C****C**ACGA.................................................UACGCC**G****C****A****U****C****C**ACACCGU........................................................................................**G****C****C**A...**C**.**U****G****G****G****A**.................AAAAU................................................................................**U****C****C****C****G****G**G.A.A.**G****G****C**..C**G****G****A**..**U****G****C**CGCG------.......................................................................................................**G****G****A****C**...A.**A**.....**G**.**C**....**C****G****G**....**A**AG..A**C**.**C**..**U**.....G...**C****C**.**U**..**G**.**A****G****U****G****U**ACAGGAUGACU | |
|  |  | NZ\_AABN02000134.1/188-6  | AUAACCCGAAU**C****G****A****U****U**..**C**.**A**..**G**.**G**.U...**G****C**.**C**.**C**---...................................UGCG............---**G****G**.**G****C****U**..U....GA..**U****A**..**G**..**G**.G..AA.**U**..**C**.**C**.**C****G**.**U**UA.....................................................................................................................................................................A**A**GU.....**C****G****G****G**.**A****G****C****G****G****A**-..C.CC..**G****C****C****G****C****C****G****U**AA..**G****U****C****C**ACGA.................................................UACGCC**G****C****A****U****C****C**ACACCGU........................................................................................**G****C****C**A...**C**.**U****G****G****G****A**.................AAAAU................................................................................**U****C****C****C****G****G**G.A.A.**G****G****C**..C**G****G****A**..**U****G****C**CGCG------.......................................................................................................**G****G****A****C**...A.**A**.....**G**.**C**....**C****G****G**....**A**AG..A**C**.**C**..**U**.....G...**C****C**.**U**..**G**.**A****G****U****G****U**ACAGGAUGACU | |
|  |  | NZ\_AAIT01000014.1/62009-61808  | GCACAGACAUG**G****G****G****C****G**..**C**.**U**..**G**.**G**.UU..**C****C**.**U**.**G****U****C**-...................................CUAU............-**G****A****C****A**.**G****G****C**..U....AA..**G****A**..**G**..**G**.G..AA.**U**..**G**.**C**.**G**-.---ACAGGCAACCGCCU.......................................................................................................................................................G**A**AA.....--**G****C**.**A****G****C****C****G****C**C..C.CC..**G****C****G****A****C****C****G****U**GA..**C****C****G****G**.....................................................AAAGGU**C****G****C****C****C****C**UCU--..........................................................................................**G****C****C**A...**C**.**U****G****G****C**-.................GAAA-................................................................................-**G****C****C****G****G**G.A.A.**G****G****C**...**G****G****G**..**G****C****G**GCCGUGGGCC............................................................................................CAGGCCCGAAU**C****C****G****C**...A.**A**.....**G**.**C**....**C****G****G**....**G**AG..A**C**.**C**..**U**.....G...**C****C**.**G**..**G**.**U****G****C****A****G**AAGGAUUGACC | |
|  |  | NC\_004557.1/793516-793697  | GAAUAUUAAAA**U****U****U****A****U**..**A**.**G**..**A**.**G**.UU..**A****C**.**C**.**C****A**--...................................UUUU............--**U****G****G**.**G****G****U**..U....AA..**A****A**..**G**..**G**.G..AA.**G**..**G**.**G**.**G****G**.**U**UA.....................................................................................................................................................................G**A**AU.....**C****C****U****C**.**C****A****C****G****G****U**-..C.CG..**G****C****C****A****C****U****G****U**AA..**U****U****G****G**.....................................................UAAGUU**U****A****U****U****C****C**AUAA-..........................................................................................**G****C****C**A...**U**.**U****G****A****G**-.................AUAUU................................................................................-**C****U****C****G****A**G.A.A.**G****G****U**...**G****G****A**..**A****A****U**AAAUGAUGAA.......................................................................................................**C****C****A****U**...A.**A**.....**G**.**U**....**C****A****G**....**G**AG..A**C**.**C**..**U**.....G...**C****U**.**U**..**U**.**A****U****A****U****U**UAAGAUGCAGC | |
|  |  | NZ\_AAHI01000024.1/65571-65820  | CUAAACUGGCG**C****C****G****C****C**..**U**.**C**..**G**.**G**.U...**G****C**.**U**.**C****G****C****G**C..................................GCAG..........GA**C****G****C****G****C**.**G****G****U**..U....AA..**A****C**..**G**..**G**.G..AA.**G**..**C**.**A**.**G****G**G**U**GCGGGCUCCACGAGCGUG.....................................................................................................................................................C**C**AA.....**C****C****U****G**.**C****G****C****U****G****C**C..C.CC..**G****C****A****A****C****G****G****U**AA..**G****C****G****G**CCGCGUCGUGUGAC.......................................GCAGGC**C****G****G****C****U****C**GGGCGCGUUUCGCGUGCAGGUCGCAGGCGGGCGCGG...........................................................**G****C****C**A...**C**.**U****G****C****G****C**G................UCAUC................................................................................**G****C****G****C****G****G**G.A.A.**G****G****C**...**G****A****G**..**C****C****G**GACCG-----.......................................................................................................**C****C****G****C**...C.**A**.....**G**.**C**....**C****C****G**....**G**AU..A**C**.**C**..**G**.....G...**C****C**.**G**..**A**.**G****A****C****G****G**GGAGCCCGCAC | |
|  |  | NZ\_AAHL01000002.1/24164-24413  | CUAAACUGGCG**C****C****G****C****C**..**U**.**C**..**G**.**G**.U...**G****C**.**U**.**C****G****C****G**C..................................GCAG..........GA**C****G****C****G****C**.**G****G****U**..U....AA..**A****C**..**G**..**G**.G..AA.**G**..**C**.**A**.**G****G**G**U**GCGGGCUCCACGAGCGUG.....................................................................................................................................................C**C**AA.....**C****C****U****G**.**C****G****C****U****G****C**C..C.CC..**G****C****A****A****C****G****G****U**AA..**G****C****G****G**CCGCGUCGUGUGAC.......................................GCAGGC**C****G****G****C****U****C**GGGCGCGUUUCGCGUGCAGGUCGCAGGCGGGCGCGG...........................................................**G****C****C**A...**C**.**U****G****C****G****C**G................UCAUC................................................................................**G****C****G****C****G****G**G.A.A.**G****G****C**...**G****A****G**..**C****C****G**GACCG-----.......................................................................................................**C****C****G****C**...C.**A**.....**G**.**C**....**C****C****G**....**G**AU..A**C**.**C**..**G**.....G...**C****C**.**G**..**A**.**G****A****C****G****G**GGAGCCCGCAC | |
|  |  | NC\_003911.11/3058882-3059105  | CCGCAUGUCCG**C****C****C****U****G**..**U**.**U**..**G**.**G**.UG..**U****C**.**C**.**G****G**--...................................GCAA............--**C****C****G**.**G****A****U**..G....AA..**A****A**..**G**..**G**.G..AA.**U**..**G**.**C**.**G**-.---CCAGCGGUUCCGGGACAUGAUCCCGGGCAGCCGU...................................................................................................................................C**A**AG.....-**C****G****C**.**A****G****C****C****G****C**C..C.CC..**G****C****G****A****C****C****G****U**GA..**C****C****G****G**.....................................................-AGAGG**G****U****G****C****C****C**AA---..........................................................................................**G****C****C**A...**C**.**U****G****G****C****C**.................AAAU-................................................................................**G****G****C****C****G****G**G.A.A.**G****G****C**..G**G****G****A**..**C****A****U**CCGCCAGAGC..........................................................................................AGCGCUCUGACAU**C****C****G****C**...A.**A**.....**G**.**C**....**C****G****G**....**G**AG..A**C**.**C**..**U**.....G...**C****C**.**A**..**G**.**C****A****C****C****C**GAACUGCAACC | |
|  |  | NC\_006513.1/2373817-2374002  | AGUAUUUAACC**G****U****A****U****C**..**G**.**A**..**G**.**G**.UG..**C****C**.**C**.**G****C****G**-...................................CUCG............-**C****G****C****G**.**G****G****A**..G....AA..**U****C**..**G**..**G**.G..AA.**G**..**G**.**C**.**G****G**.**U**GC.....................................................................................................................................................................G**A**UU.....**C****C****G****C**.**C****A****C****G****U****G**-..C.CC..**A****G****C****G****C****C****G****U**GAU.**G****A****G**-.....................................................GAUCGU**C****C****G****G****G****C**AGGCGUCA.......................................................................................**G****C****C**A...**C**.**U****G****G****C**-.................GAAA-................................................................................-**G****C****C****G****G**G.A.A.**G****G****C**...**G****C****C**..**C****G****G**ACGAGUCGAG.......................................................................................................-**C****C****U**...C.**A**.....**G**.**U**....**C****G****G**....**A**AG..A**C**.**C**..**G**.....G...**C****C**.**U**..**C**.**G****A****G****A****C**AUAUUGGCAGC | |
|  |  | NC\_002967.9/1025870-1025682  | AAUAACGGUUU**C****G****G****U****C**..**U**.**U**..**G**.**G**.UG..**C****U**.**C**.**C****U****U**-...................................UUAG..........CU-**A****A****G****G**.**A****G****U**..G....AA..**A****A**..**G**..**G**.G..AA.**U**..**C**.**A**.**G****G**.**U**GA.....................................................................................................................................................................A**A**GU.....**C****C****U****G**.**A****G****C****A****G****U**-..C.CG..**G****C****U****G****A****C****G****U**AAG.**U****G****A****G**A....................................................GAGUUG**U****U****U****U****U****C**AAAAU..........................................................................................**G****C****C**A...**C**.**U****G****G****U**-.................UUAU-................................................................................-**U****C****C****G****G**G.A.A.**G****G****C**...**G****A****A**..**A****A****U**CAAUGAUGAC.......................................................................................................**C****U****C****C**...G.**A**.....**G**.**C**....**C****G****U**....**A**AG..A**C**.**C**..**U**.....G...**C****C**.**A**..**A**U**G****A****C****U****A**UAAGGGUAAAA | |
|  |  | NZ\_AAHI01000043.1/6705-6896  | UACGGCCGUUC**G****U****U****C****G**..**C**.**C**..**G**.**G**.U...**G****C**.**C**.**C**---...................................UUCG............---**G****G**.**G****C****C**..G....AA..**G****A**..**G**..**G**.G..AA.**C**..**A**.**C**.**A****G**.**G**GCGCCCCGCGC............................................................................................................................................................G**C**CG.....**C****U****G****U**.**G****G****C****U****G****C**C..C.CC..**G****C****A****A****C****U****G****U**AG..**A****C****A****G**CG...................................................AGCCGA**G****C****U****C****C****C**CCUCAU.........................................................................................**G****C****C**A...**C**.**U****G****G****U**-.................-UUC-................................................................................-**A****C****C****G****G**G.A.A.**G****G****C**..C**G****G****G**..**A****G****C**CGGCGCCGAC.......................................................................................................**C****U****G****U**...C.**A**.....**G**.**C**....**C****A****G**....**G**AG..A**C**.**C**..**U**.....G...**C****C**.**G**..**G**.**C****C****A****G****A**AAGUGCGUGCG | |
|  |  | NZ\_AAHL01000324.1/162-353  | UACGGCCGUUC**G****U****U****C****G**..**C**.**C**..**G**.**G**.U...**G****C**.**C**.**C**---...................................UUCG............---**G****G**.**G****C****C**..G....AA..**G****A**..**G**..**G**.G..AA.**C**..**A**.**C**.**A****G**.**G**GCGCCCCGCGC............................................................................................................................................................G**C**CG.....**C****U****G****U**.**G****G****C****U****G****C**C..C.CC..**G****C****A****A****C****U****G****U**AG..**A****C****A****G**CG...................................................AGCCGA**G****C****U****C****C****C**CCUCAU.........................................................................................**G****C****C**A...**C**.**U****G****G****U**-.................-UUC-................................................................................-**A****C****C****G****G**G.A.A.**G****G****C**..C**G****G****G**..**A****G****C**CGGCGCCGAC.......................................................................................................**C****U****G****U**...C.**A**.....**G**.**C**....**C****A****G**....**G**AG..A**C**.**C**..**U**.....G...**C****C**.**G**..**G**.**C****C****A****G****A**AAGUGCGUGCG | |
|  |  | NZ\_AAEI01000013.1/158190-158441  | CUAAACUGGCG**C****C****G****C****C**..**U**.**C**..**G**.**G**.U...**G****C**.**U**.**C****G****C****G**C..................................GUGG..........AA**C****G****C****G****C**.**G****G****U**..U....AA..**A****C**..**G**..**G**.G..AA.**G**..**C**.**A**.**G****G**.**G**CGCGGGCGCCGGAAGCCCG....................................................................................................................................................C**C**AA.....**C****C****U****G**.**C****G****C****U****G****C**C..C.CC..**G****C****A****A****C****G****G****U**AA..**G****C****G****G**CCGCGUCCGAUGAC.......................................GCAGGC**C****G****G****C****U****C**GGGCGCGUUUCACGUGCAGGUUCUGCAUGCGGACGCGG.........................................................**G****C****C**A...**C**.**U****G****C****G****C**G................UCAUC................................................................................**G****C****G****C****G****G**G.A.A.**G****G****C**...**G****A****G**..**C****C****G**GACCG-----.......................................................................................................**C****C****G****C**...C.**A**.....**G**.**C**....**C****C****G**....**G**AU..A**C**.**C**..**G**.....G...**C****C**.**G**..**A**.**G****A****C****G****G**GGAGCCCGCAC | |
|  |  | NC\_006840.1/2743827-2743609  | UACUAUUUCGC**C****C****A****U****G**..**U**.**U**..**G**.**G**.UG..**C****G**.**C**.**U****A****U****U**U..................................UUAA...........U**U****A****U****A****G**.**C****C****U**..G....AA..**U****A**..**G**..**G**.G..AA.**U**..**C**.**U**.**G****G**.**U**GA.....................................................................................................................................................................A**A**AU.....**C****C****A****G**.**A****A****C****U****G****A**-..C.GC..**G****C****A****G****C****G****G****U**AA..**G****A****G****A**.....................................................GAACGA**A****G****G****U****A****C**AAUUUUU........................................................................................**C****A****C**A...**C**.**U****G****U****U****A**AUUUUAGAUGU......UUAAG....................................................................GAUAUCGACGAA**U****A****A****U****G****G**G.A.A.**G****U****G**..A**G****U****A**..**C****U****C**UAGGUUAACU.....................................................................................................GC**U****C****U****C**...A.**A**.....**G**.**U**....**C****C****G**....**A**AG..A**C**.**C**..**U**.....G...**C****C**.**A**..**G**.**C****A****A****U****A**AGUAAUACAUU | |
|  |  | NC\_000913.2/4161407-4161597  | CUGUAGCAUCC**A****C****U****U****G**..**C**.**C**..**G**.**G**.UC..**C****U**.-.----...................................GUG-............-----.**A****G****U**..U....AA..**U****A**..**G**..**G**.G..AA.**U**..**C**.**C**.**A****G**.**U**GC.....................................................................................................................................................................G**A**AU.....**C****U****G****G**.**A****G****C****U****G****A**-..C.GC..**G****C****A****G****C****G****G****U**AA..**G****G****A****A**.....................................................AGGUGC**G****A****U****G****A****U**UGCGUUAUGCG....................................................................................**G****A****C**A...**C**.**U****G****C****C**-.................AUUC-................................................................................-**G****G****U****G****G**G.A.A.**G****U****C**...**A****U****C**..**A****U****C**UCUUAGUAUC.............................................................................................UUAGAUACCC**C****U****C****C**...A.**A**.....**G**.**C**....**C****C****G**....**A**AG..A**C**.**C**..**U**.....G...**C****C**.**G**..**G**.**C****C****A****A****C**GUCGCAUCUGG | |
|  |  | NC\_002655.2/5041415-5041605  | CUGUAGCAUCC**A****C****U****U****G**..**C**.**C**..**G**.**G**.UC..**C****U**.-.----...................................GUG-............-----.**A****G****U**..U....AA..**U****A**..**G**..**G**.G..AA.**U**..**C**.**C**.**A****G**.**U**GC.....................................................................................................................................................................G**A**AU.....**C****U****G****G**.**A****G****C****U****G****A**-..C.GC..**G****C****A****G****C****G****G****U**AA..**G****G****A****A**.....................................................AGGUGC**G****A****U****G****A****U**UGCGUUAUGCG....................................................................................**G****A****C**A...**C**.**U****G****C****C**-.................AUUC-................................................................................-**G****G****U****G****G**G.A.A.**G****U****C**...**A****U****C**..**A****U****C**UCUUAGUAUC.............................................................................................UUAGAUACCC**C****U****C****C**...A.**A**.....**G**.**C**....**C****C****G**....**A**AG..A**C**.**C**..**U**.....G...**C****C**.**G**..**G**.**C****C****A****A****C**GUCGCAUCUGG | |
|  |  | NC\_002695.1/4972649-4972839  | CUGUAGCAUCC**A****C****U****U****G**..**C**.**C**..**G**.**G**.UC..**C****U**.-.----...................................GUG-............-----.**A****G****U**..U....AA..**U****A**..**G**..**G**.G..AA.**U**..**C**.**C**.**A****G**.**U**GC.....................................................................................................................................................................G**A**AU.....**C****U****G****G**.**A****G****C****U****G****A**-..C.GC..**G****C****A****G****C****G****G****U**AA..**G****G****A****A**.....................................................AGGUGC**G****A****U****G****A****U**UGCGUUAUGCG....................................................................................**G****A****C**A...**C**.**U****G****C****C**-.................AUUC-................................................................................-**G****G****U****G****G**G.A.A.**G****U****C**...**A****U****C**..**A****U****C**UCUUAGUAUC.............................................................................................UUAGAUACCC**C****U****C****C**...A.**A**.....**G**.**C**....**C****C****G**....**A**AG..A**C**.**C**..**U**.....G...**C****C**.**G**..**G**.**C****C****A****A****C**GUCGCAUCUGG | |
|  |  | NC\_004431.1/4695788-4695978  | CUGUAGCAUCC**A****C****U****U****G**..**C**.**C**..**G**.**G**.UC..**C****U**.-.----...................................GUG-............-----.**A****G****U**..U....AA..**U****A**..**G**..**G**.G..AA.**U**..**C**.**C**.**A****G**.**U**GC.....................................................................................................................................................................G**A**AU.....**C****U****G****G**.**A****G****C****U****G****A**-..C.GC..**G****C****A****G****C****G****G****U**AA..**G****G****A****A**.....................................................AGGUGC**G****A****U****G****A****U**UGCGUUAUGCG....................................................................................**G****A****C**A...**C**.**U****G****C****C**-.................AUCC-................................................................................-**G****G****U****G****G**G.A.A.**G****U****C**...**A****U****C**..**A****U****C**UCUUAGUAUC.............................................................................................UUAGAUACCC**C****U****C****C**...A.**A**.....**G**.**C**....**C****C****G**....**A**AG..A**C**.**C**..**U**.....G...**C****C**.**G**..**G**.**C****C****A****A****C**GUCGCAUCUGG | |
|  |  | NZ\_AAHJ01000023.1/44618-44425  | UUUUUUACUGA**U****G****A****A****G**..**C**.**U**..**G**.**G**AU...**A****C**.**C**.**C****C**--...................................AUAA..........AA--**A****G****G**.**G****U****U**..U....AA..**U****A**..**G**..**G**.G..AA.**U**..**C**.**A**.**C****G**.**U**UA.....................................................................................................................................................................A**A**AU.....**C****G****U****G**.**A****G****C****U****G****A**-..C.GC..**G****C****A****A****C****U****G****U**AAA.**G****C****C****G**AUCGA................................................GUGUUG**U****G****C****A****G****C**GUUAU..........................................................................................**A****C****C**A...**C**.**U****G****U****U****C**CGAACG...........AUACG................................................................................**G****A****A****U****G****G**G.A.A.**G****G****U**...**G****C****U**..**G****C****A**GA--------.....................................................................................................AC**C****G****G****C**...A.**A**.....**G**.**C**....**C****A****G**....**G**AG..A**C**.**C**..**U**.....G..U**C****C**.**C**..**G**.**C****U****U****C****U**UUUCUUGUAAC | |
|  |  | NC\_006347.1/1825866-1825658  | UUAUCUUUGCA**G****U****G****C****A**..**U**.**U**..**G**.**G**.U...**U****U**.**G**.**U****A****U****U**CUCC...............................GUGU......UCCGGG**G****A****U****G****C**.**A****A****U**..U....AA..**A****A**..**G**..**G**.G..AA.**U**..**C**.**A**.**G****G**.**U**GC.....................................................................................................................................................................A**A**AU.....**C****C****U****G**.**A****A****C****A****G****U**-..C.CC..**G****C****U****G****C****U****G****U**AA..**G****U****U****U**CACAUCAG.............................................AUUGUU**G****U****A****A****G****C**AACCACCACUU....................................................................................**G****C****C**A...**C**.**U****G****G****G****A**A................ACAUU................................................................................**U****C****C****U****G****G**G.A.A.**G****G****C**...**G****C****U**..**U****A****U**CAACAG----.......................................................................................................**A****A****A****C**...G.**A**.....**G**.**U**....**C****A****G**....**A**AG..A**C**.**C**..**U**.....G...**C****C**.**U**..**G**.**U****G****C****A****U**CUUUUUCAUUG | |
|  |  | NC\_003454.1/934497-934678  | UUAAUAUCAUG**U****C****A****A****U**..**U**.**A**..**U**.**G**.UU..**C****C**.**U**.**U****A**--...................................UUUU...........U--**U****A****A**.**G****G****C**..U....AA..**G****A**..**G**..**G**.G..AA.**U**..**U**.**U**.**G****G**.**U**GA.....................................................................................................................................................................G**A**UA.....**C****C****A****A**.**A****A****C****G****A****G**-..C.CC..**G****U****C****G****C****U****G****U**AA..**U****U****G****A**.....................................................--GUUU**U****U****U****C****U****U**GUUUUAU........................................................................................**A****C****C**A...**C**.**U****G****G****A****U**.................UUUU-................................................................................**A****U****U****U****G****G**G.A.A.**G****G****U**..A**A****A****G**..**A****A****A**UAUAAA----.......................................................................................................**U****C****A****U**...A.**A**.....**G**.**U**....**C****A****G**....**A**AG..A**C**.**C**..**U**.....G...**C****A**.**U**..**A**.**A****U****U****G****A**AUUACUCUAUC | |
|  |  | NZ\_AAAW03000112.1/5600-5773  | AAAAAAAGUCC**A****U****U****C****U**..**U**.**U**..**G**.**G**.UG..**C****C**.**C**.**C**---...................................-CAA............---**G****G**.**G****G****A**..G....AA..**U****A**..**G**..**G**.G..AA.**A**..**C**.**C**.**G****G**.**U**GC.....................................................................................................................................................................G**A**UU.....**C****C****G****G**.**U****G****C****G****G****A**-..C.CC..**A****C****C****A****C****U****G****U**GUA.**G****G****A**-.....................................................UGAGCC**U****G****A****A****C****C**AUAA-..........................................................................................-**C****C**A...**C**.**C****G****G**--.................CCAGA................................................................................--**C****C****G****G**G.A.A.**G****G**-...**G****G****U**..**A****G****C**GGCGUUUGAA.......................................................................................................-**U****C****C**...A.**A**.....**G**.**C**....**C****A****G**....**G**AG..A**C**.**C**..**U**.....G...**C****C**.**A**..**G**.**G****A****A****U****G**AAGAAAGCCUU | |
|  |  | NC\_004463.1/3617290-3617510  | AAAUGGUGGCC**C****G****G****C****G**..**U**.**U**..**G**.**G**.UU..**C****C**.**U**.**G****U****C**-...................................CUAU............-**G****A****C****A**.**G****G****C**..G....AA..**G****A**..**G**..**G**.G..AA.**U**..**G**.**C**.**G****A**.**U**AGGGUCCGAAUCGGCAAGAUUUGGGUCC...........................................................................................................................................A**A**AA.....**U**-**G****C**.**A****G****C****C****G****C**C..C.CC..**G****C****G****A****C****C****G****U**GA..**C****C****G****G**.....................................................--AGAG**A****U****G****C****C****C**GAG--..........................................................................................**G****C****C**A...**C**.**U****G****A****U****C**CC...............UGACG...............................................................................G**G****A****U****C****G****G**G.A.A.**G****G****C**...**G****G****G**..**G****A****U**CGAAGGGCAA..............................................................................................AACCCUGCU**C****C****G****C**...A.**A**.....**G**.**C**....**C****G****G**....**G**AG..A**C**.**C**..**U**.....G...**C****C**.**A**..**G**.**C****G****C****G****G**ACGAUUUUGGA | |
|  |  | NZ\_AAEH02000023.1/76913-77102  | UACGGCCGUUC**G****U****U****C****G**..**C**.**C**..**G**.**G**.U...**G****C**.**C**.**C**---...................................UUCG............---**G****G**.**G****C****C**..G....AA..**G****A**..**G**..**G**.G..AA.**C**..**A**.**C**.**A****G**.**G**GCGCGCCGC..............................................................................................................................................................G**C**CG.....**C****U****G****U**.**G****G****C****U****G****C**C..C.CC..**G****C****A****A****C****U****G****U**AG..**A****C****A****G**C....................................................GAGCCG**C****G****C****U****C****C**GCCACAU........................................................................................**G****C****C**A...**C**.**U****G****G****U**-.................-UUC-................................................................................-**A****C****C****G****G**G.A.A.**G****G****C**.CG**G****G****A**..**G****C****C**GGCGCCGAC-.......................................................................................................**C****U****G****U**...C.**A**.....**G**.**C**....**C****A****G**....**G**AG..A**C**.**C**..**U**.....G...**C****C**.**G**..**G**.**C****C****A****G****A**AAGUGCGUGCG | |
|  |  | NC\_003228.3/1843997-1843789  | UUAUCUUUGCA**G****U****G****C****A**..**U**.**U**..**G**.**G**.U...**U****U**.**G**.**U****A****U****U**CUCC...............................GUGU......UCCGGG**G****A****U****G****C**.**A****A****U**..U....AA..**A****A**..**G**..**G**.G..AA.**U**..**C**.**A**.**G****G**.**U**GC.....................................................................................................................................................................A**A**AU.....**C****C****U****G**.**A****A****C****A****G****U**-..C.CC..**G****C****U****G****C****U****G****U**AA..**G****U****U****U**CACAUCAG.............................................AUUGUU**G****U****A****A****G****C**AACCACCACUU....................................................................................**G****C****C**A...**C**.**U****G****G****G****A**A................ACGUU................................................................................**U****C****C****U****G****G**G.A.A.**G****G****C**...**G****C****U**..**U****A****U**CAACAG----.......................................................................................................**A****A****A****C**...G.**A**.....**G**.**U**....**C****A****G**....**A**AG..A**C**.**C**..**U**.....G...**C****C**.**U**..**G**.**U****G****C****A****U**CUUUUUCAUUG | |
|  |  | NC\_002950.2/1495987-1495774  | UUAUCUUUGUG**C****G****G****A****C**..**U**.**U**..**U**.**G**.U...**U****C**.**A**.**G****A****G****C**UGCCUUUUUCCUAUUG...................GGAU.......CGGUG**G****U****G****A****U**.**G****A****U**..U....AA..**A****A**..**G**..**G**.G..AA.**U**..**C**.**G**.**G****G**.**U**GA.....................................................................................................................................................................A**A**AU.....**C****C****C****G**.**A****G****C****A****G****U**-..C.CC..**G****C****U****G****C****U****G****U**GA..**A****C****C****U**UGU..................................................UAAGCU**U****U****C****G****G****C**AUUUAUA........................................................................................**U****C****C**A...**C**.**U****G****U****C****C**G................UUCUG.............................................................................UGC**G****G****A****U****G****G**G.A.A.**G****G****A**...**G****U****C**..**G****G****A**AGUAUG----.......................................................................................................**G****G****G****U**...G.**A**.....**G**.**C**....**C****A****G**....**A**AG..A**C**.**C**..**U**.....G...**C****A**.**A**..**A**.**G****U****C****U****U**UGUCUGCGGCU | |
|  |  | NZ\_AAEB02000018.1/9405-9223  | GUUAUCCUGCC**C****G****G****C****G**..**U**.**A**..**G**.**G**.C...**G****G**.**G**.**C**---...................................GAGA............---**G****G**.**U****C**-..-....--..-**C**..**A**..**G**.G..AA.**G**..**C**.**C**.**G****G**.**U**GA.....................................................................................................................................................................G**A**GU.....**C****C****G****G**.**C****A****C****G****G****U**-..C.CC..**G****C****C****A****C****U****G****U**GA..**C****C****G****G**G....................................................GAGCGA**C****C****C****C****C****G**CACGCAGG.......................................................................................**G****C****C**A...**C**.**U****G****U****C****G**CC...............GCGGG................................................................................**C****G****A****U****G****G**G.A.A.**G****G****C**...**C****G****G**..**G****G****G**AAGCGGAGAU.......................................................................................................**C****C****G****G**...G.**A**.....**G**.**U**....**C****A****G**....**G**AU..A**C**.**U**..**G**.....G...**C****C**.**U**..**C**.**U****C****G****C****C**GGAGAAACCCC | |
|  |  | NZ\_AAEI01000028.1/56670-56861  | UACGGCCGUUC**G****U****U****C****G**..**C**.**C**..**G**.**G**.U...**G****C**.**C**.**C**---...................................CUCG............---**G****G**.**G****C****C**..G....AA..**G****A**..**G**..**G**.G..AA.**C**..**A**.**C**.**A****G**.**G**GCGCGCCACGC............................................................................................................................................................G**C**CG.....**C****U****G****U**.**G****G****C****U****G****C**C..C.CC..**G****C****A****A****C****U****G****U**AG..**A****C****A****G**CGA..................................................GCCGAU**C****U****C****C****C****C**CUCAU..........................................................................................**G****C****C**A...**C**.**U****G****G****U**-.................-UCC-................................................................................-**A****C****C****G****G**G.A.A.**G****G****C**...**C****G****G**..**G****A****G**CCGGCGCCGA......................................................................................................C**C****U****G****U**...C.**A**.....**G**.**C**....**C****A****G**....**G**AG..A**C**.**C**..**U**.....G...**C****C**.**G**..**G**.**C****C****U****G****A**AAGUGCGUGCG | |
|  |  | NZ\_AAIJ01000007.1/29492-29684  | UUUUGUAUUGG**G****G****A****A****G**..**C**.**U**..**G**.**G**AU...**A****C**.**C**.**C****U****G**-...................................UAAA............-**A****G****G****G**.**G****U****U**..U....AA..**U****A**..**G**..**G**.G..AA.**U**..**C**.**A**.**C****G**.**U**UC.....................................................................................................................................................................A**A**AU.....**C****G****U****G**.**A****G****C****U****G****A**-..C.GC..**G****C****A****A****C****U****G****U**AA..**G****U****C****C**CAGGA................................................UAUUUC**U****G****C****A****G****C**AUUGU..........................................................................................**G****C****C**A...**C**.**U****G****U****G****C**CG...............UUAUA.............................................................................CCG**G****C****G****C****G****G**G.A.A.**G****G****C**...**G****C****U**..**G****C****A**GAAG------.......................................................................................................**G****G****A****U**..GA.**A**.....**G**.**C**....**C****A****G**....**G**AG..A**C**.**C**..**U**.....G..U**C****C**.**U**..**G**.**U****U****U****U****C**UUUGUUUUCCA | |
|  |  | NC\_003197.1/4347851-4348044  | CGGUAGCAUCC**G****U****G****G****G**..**C**.**C**..**G**.**G**.UC..**C****U**.-.----...................................GUG-............-----.**A****G****U**..U....AA..**U****A**..**G**..**G**.G..AA.**U**..**C**.**C**.**A****G**.**U**GA.....................................................................................................................................................................A**A**AU.....**C****U****G****G**.**A****G****C****U****G****A**-..C.GC..**G****C****A****G****C****G****G****U**AA..**G****G****A**-.....................................................AAGGUG**A****G****A****U****G****A**GAGCGUAAGCA....................................................................................**G****A****C**A...**C**.**U****G****C****C**-.................-UCC-................................................................................-**G****G****C****G****G**G.A.A.**G****U****C**..A**U****C****A**..**U****U****U**CUGCUAUCCA........................................................................................GCCAACGGAUAACCC-**U****C****C**...A.**A**.....**G**.**C**....**C****C****G**....**A**AG..A**C**.**C**..**U**.....G...**C****C**.**G**..**G**.**C****U****A****A****C**GUCGCAUCUGG | |
|  |  | NC\_003198.1/3603350-3603157  | CGGUAGCAUCC**G****U****G****G****G**..**C**.**C**..**G**.**G**.UC..**C****U**.-.----...................................GUG-............-----.**A****G****U**..U....AA..**U****A**..**G**..**G**.G..AA.**U**..**C**.**C**.**A****G**.**U**GA.....................................................................................................................................................................A**A**AU.....**C****U****G****G**.**A****G****C****U****G****A**-..C.GC..**G****C****A****G****C****G****G****U**AA..**G****G****A**-.....................................................AAGGUG**A****G****A****U****G****A**GAGCGUAAGCA....................................................................................**G****A****C**A...**C**.**U****G****C****C**-.................-UCC-................................................................................-**G****G****C****G****G**G.A.A.**G****U****C**..A**U****C****A**..**U****U****U**CUGCUAUCCA........................................................................................GUCAACGGAUAACCC-**U****C****C**...A.**A**.....**G**.**C**....**C****C****G**....**A**AG..A**C**.**C**..**U**.....G...**C****C**.**G**..**G**.**C****U****A****A****C**GUCGCAUCUGG | |
|  |  | NC\_004631.1/3589010-3588817  | CGGUAGCAUCC**G****U****G****G****G**..**C**.**C**..**G**.**G**.UC..**C****U**.-.----...................................GUG-............-----.**A****G****U**..U....AA..**U****A**..**G**..**G**.G..AA.**U**..**C**.**C**.**A****G**.**U**GA.....................................................................................................................................................................A**A**AU.....**C****U****G****G**.**A****G****C****U****G****A**-..C.GC..**G****C****A****G****C****G****G****U**AA..**G****G****A**-.....................................................AAGGUG**A****G****A****U****G****A**GAGCGUAAGCA....................................................................................**G****A****C**A...**C**.**U****G****C****C**-.................-UCC-................................................................................-**G****G****C****G****G**G.A.A.**G****U****C**..A**U****C****A**..**U****U****U**CUGCUAUCCA........................................................................................GUCAACGGAUAACCC-**U****C****C**...A.**A**.....**G**.**C**....**C****C****G**....**A**AG..A**C**.**C**..**U**.....G...**C****C**.**G**..**G**.**C****U****A****A****C**GUCGCAUCUGG | |
|  |  | NC\_006511.1/4121601-4121794  | CGGUAGCAUCC**G****U****G****G****G**..**C**.**C**..**G**.**G**.UC..**C****U**.-.----...................................GUG-............-----.**A****G****U**..U....AA..**U****A**..**G**..**G**.G..AA.**U**..**C**.**C**.**A****G**.**U**GA.....................................................................................................................................................................A**A**AU.....**C****U****G****G**.**A****G****C****U****G****A**-..C.GC..**G****C****A****G****C****G****G****U**AA..**G****G****A**-.....................................................AAGGUG**A****G****A****U****G****A**GAGCGUAAGCA....................................................................................**G****A****C**A...**C**.**U****G****C****C**-.................-UCC-................................................................................-**G****G****C****G****G**G.A.A.**G****U****C**..A**U****C****A**..**U****U****U**CUGCUAUCCA........................................................................................GUCAACGGAUAACCC-**U****C****C**...A.**A**.....**G**.**C**....**C****C****G**....**A**AG..A**C**.**C**..**U**.....G...**C****C**.**G**..**G**.**C****U****A****A****C**GUCGCAUCUGG | |
|  |  | NC\_006905.1/4282220-4282413  | CGGUAGCAUCC**G****U****G****G****G**..**C**.**C**..**G**.**G**.UC..**C****U**.-.----...................................GUG-............-----.**A****G****U**..U....AA..**U****A**..**G**..**G**.G..AA.**U**..**C**.**C**.**A****G**.**U**GA.....................................................................................................................................................................A**A**AU.....**C****U****G****G**.**A****G****C****U****G****A**-..C.GC..**G****C****A****G****C****G****G****U**AA..**G****G****A**-.....................................................AAGGUG**A****G****A****U****G****A**GAGCGUAAGCA....................................................................................**G****A****C**A...**C**.**U****G****C****C**-.................-UCC-................................................................................-**G****G****C****G****G**G.A.A.**G****U****C**..A**U****C****A**..**U****U****U**CUGCUAUCCA........................................................................................GUCAACGGAUAACCC-**U****C****C**...A.**A**.....**G**.**C**....**C****C****G**....**A**AG..A**C**.**C**..**U**.....G...**C****C**.**G**..**G**.**C****U****A****A****C**GUCGCAUCUGG | |
|  |  | NC\_005966.1/624557-624347  | UUUACACAAUU**C****G****U****A****A**..**C**.**A**..**A**.**G**.UUA.**A****A**.**A**.**G****C**--...................................AUUC............--**G****C****U**.**U****U**-..-....--..-**A**..**G**..**G**.G..AA.**A**..**C**.**U**.**G****G**.**U**GC.....................................................................................................................................................................A**A**AU.....**C****C****A****G**.**U****G****C****U****G****C**C..C.CC..**G****C****A****A****C****G****G****U**AA..**A****A****A****U**.....................................................GUAAAC**C****A****U****A****U****U**AAAAAAGUCAUUUAGACUUAC..........................................................................**G****C****C**A...**C**.**U****G****C****A****U**G................CAUAG................................................................................**A****U****G****U****G****G**G.A.A.**G****G****U**..G**A****A****U**..**A****U****G**CUUGUCUCUU............................................................................................UUUGAGAUGCC**A****U****U****U**...G.**A**.....**G**.**U**....**C****C****G**....**G**AG..A**C**.**C**..**U**.....G...**C****U**.**U**..**G**.**U****U****A****C****A**UCUAUCCACUC | |
|  |  | NC\_003228.3/3157593-3157815  | UAUCUUUGCUC**C****C****U****G****A**..**U**.**C**..**G**.**G**.U...**U****C**.**C**.**G****A****A****U**A..................................GUCA.....UUCCUCU**A****U****C****C****G**.**G****A****U**..U....AA..**A****A**..**G**..**G**.G..AA.**U**..**C**.**G**.**G****G**.**U**GU.....................................................................................................................................................................A**A**AU.....**C****C****C****G**.**G****A****C****A****G****U**-..C.CC..**G****C****U****G****C****U****G****U**GAA.**G****C****U****C**CGUC.................................................UGAAUU**U****C****C****G****A****U**AACAACUGUU.....................................................................................**G****C****C**A...**C**.**U****G****G****G****A**UACCUUUUUG.......UAAAU.....................................................................AGAUAAGGAGU**C****A****C****C****G****G**G.A.A.**G****G****C**...**G****U****C**..**G****G****A**AACAAG----.......................................................................................................**G****A****G****U**...C.**A**.....**G**.**U**....**C****A****G**....**A**AG..A**C**.**C**..**U**.....G...**C****C**.**G**..**C**.**U****U****A****U****C**AAAGGCUGUUU | |
|  |  | NC\_006347.1/3096302-3096524  | UAUCUUUGCUC**C****C****U****G****A**..**U**.**C**..**G**.**G**.U...**U****C**.**C**.**G****A****A****U**A..................................GUCA.....UUCCUCU**A****U****C****C****G**.**G****A****U**..U....AA..**A****A**..**G**..**G**.G..AA.**U**..**C**.**G**.**G****G**.**U**GU.....................................................................................................................................................................A**A**AU.....**C****C****C****G**.**G****A****C****A****G****U**-..C.CC..**G****C****U****G****C****U****G****U**GAA.**G****C****U****C**CGUC.................................................UGAAUU**U****C****C****G****A****U**AACAACUGUU.....................................................................................**G****C****C**A...**C**.**U****G****G****G****A**UACCUUUUUG.......UAAAU.....................................................................AGAUAAGGAGU**C****A****C****C****G****G**G.A.A.**G****G****C**...**G****U****C**..**G****G****A**AACAAG----.......................................................................................................**G****A****G****U**...C.**A**.....**G**.**U**....**C****A****G**....**A**AG..A**C**.**C**..**U**.....G...**C****C**.**G**..**C**.**U****U****A****U****C**AAAGGCUGUUU | |
|  |  | NC\_002944.2/2995815-2996105  | AAGCUGCGCUA**C****G****C****A****G**..**C**.**U**..**G**.**G**.UC..**U****G**.**C**.**U****C****G****C**GUCGCAACCGCGCCGUGAGCAUCGAGCGAAGCAACGUUA.........GUA**A****C****G****C****U**.**U****C****G**..U....AA..**G****A**..**G**..**G**.G..AA.**C**..**C**.**C**.**G****G**.**U**GA.....................................................................................................................................................................G**A**AU.....**C****C****G****G**.**G****A****C****U****G****U**-..C.CC..**G****C****A****A****C****G****G****U**AU..**G****C****A****G**GA...................................................ACGACC**G****C****C****G****U****C**ACCA-..........................................................................................-**G****C**A...**C**.**U****G****G****U****C**.................GCAA-................................................................................**G****A****C****U****G****G**G.A.A.**G****C**-...**G****A****C**..**G****G****C**CACUAGGAAC.....................................UGCCCGGCUCCUCGGCGGCCCUUCGGGACCGCAUCGUCGGCCGGGCACGCACCACCCGGUGCGCGC**C****U****G****C**...G.**A**.....**G**.**U**....**C****C****G**....**G**AG..A**C**.**C**..**U**.....G...**C****C**.**A**..**G**.**C****U****G****U****G**CCGGGCGCGCC | |
|  |  | NC\_002950.2/749595-749392  | UAUCUUUGCCG**U****G****U****C****A**..**A**.**U**..**G**.**G**.UU..**U****U**.**C**.**U****A****C****A**CCGGUUC............................AUGG..........AG**U****G****U****A****G**.**A****A****U**..G....AA..**A****A**..**G**..**G**.G..AA.**C**..**C**.**C**.**A****G**.**U**GA.....................................................................................................................................................................A**A**UU.....**C****U****G****G**.**G****A****C****U****G****U**A..C.CC..**U****C****A****G****C****U****G****U**AA..**G****U****U****C**AGAUGU...............................................AAGGGU**U****U****U****G****C****C**ACGAGAAA.......................................................................................**G****C****C**A...**C**.**U****A****U****A****C**AG...............AAUC-................................................................................**G****U****A****U****G****G**G.A.A.**G****G****C**...**A****G****C**..**G****A****A**GCCUU-----.......................................................................................................**G****A****A****U**...A.**A**.....**G**.**U**....**C****A****G**....**A**AG..A**C**.**C**..**U**.....G...**C****C**.**A**..**U**.**U****A****C****A****A**GCGUUCUUUCU | |
|  |  | NC\_003366.1/1241729-1241907  | GUUGAUUAACU**A****A****U****A****A**..**U**.**U**..**G**.**G**.U...**G****U**.**G**.----...................................AUUU...........U----**C**.**G****C****U**..U....AA..**U****A**..**G**..**G**.G..AA.**U**..**G**.**A**.**A****G**.**U**UA.....................................................................................................................................................................A**A**GU.....**C****U****U****C**.**A****A****C****U****A****C**C..U.CA..**G****U****A****A****C****C****G****U**GA..**A****G****C**-.....................................................AGACAA**A****A****U****C****U****C**AAUAU..........................................................................................**G****U****C**A...**C**.**U****G****C****A****U**.................UUUUU................................................................................**G****U****G****U****G****G**G.A.A.**G****A****C**...**G****A****G**..**A****U****G**GAGGAAGAA-.......................................................................................................-**G****C****A**...A.**A**.....**G**.**U**....**C****G****G**....**G**AU..A**C**.**C**..**U**.....G...**C****C**.**U**..**U**.**U****U****A****U****U**UAAGUACUAUU | |
|  |  | NC\_006350.1/1133342-1133609  | CUAAACUGGCG**C****C****G****C****C**..**U**.**C**..**G**.**G**.U...**G****C**.**U**.**C****G****C****G**C..................................GCUU..........CG**C****G****C****G****C**.**G****G****U**..U....AA..**A****C**..**G**..**G**.G..AA.**G**..**C**.**A**.**G****G**.**G**CGCGACGUCCGCCAGGGCCGAG.................................................................................................................................................C**C**AA.....**C****C****U****G**.**C****G****C****U****G****C**C..C.CC..**G****C****A****A****C****G****G****U**AA..**G****C****G****A**UCGCAUCGGAGCCGAUCCGAUGCC.............................AGCCGC**A****C****U****C****U****C**GAUGCGCCGGCACGGCCGCGAUGGCCGGCCGCCGGCGCGGU......................................................**G****C****C**A...**C**.**U****G****C****G****C**.................UUCGC................................................................................**G****C****G****C****G****G**G.A.A.**G****G****C**...**G****A****G**..**G****C****G**GUGACG----.......................................................................................................**U****C****G****C**...G.**A**.....**G**.**C**....**C****C****G**....**G**AU..A**C**.**C**..**G**.....G...**C****C**.**G**..**A**.**G****G****C****G****G**GGAGGCGGACG | |
|  |  | NZ\_AAHM01000002.1/1394105-1393838  | CUAAACUGGCG**C****C****G****C****C**..**U**.**C**..**G**.**G**.U...**G****C**.**U**.**C****G****C****G**C..................................GCUU..........CG**C****G****C****G****C**.**G****G****U**..U....AA..**A****C**..**G**..**G**.G..AA.**G**..**C**.**A**.**G****G**.**G**CGCGACGUCCGCCAGGGCCGAG.................................................................................................................................................C**C**AA.....**C****C****U****G**.**C****G****C****U****G****C**C..C.CC..**G****C****A****A****C****G****G****U**AA..**G****C****G****A**UCGCAUCGGAGCCGAUCCGAUGCC.............................AGCCGC**A****C****U****C****U****C**GAUGCGCCGGCACGGCCGCGAUGGCCGGCCGCCGGCGCGGU......................................................**G****C****C**A...**C**.**U****G****C****G****C**.................UUCGC................................................................................**G****C****G****C****G****G**G.A.A.**G****G****C**...**G****A****G**..**G****C****G**GUGACG----.......................................................................................................**U****C****G****C**...G.**A**.....**G**.**C**....**C****C****G**....**G**AU..A**C**.**C**..**G**.....G...**C****C**.**G**..**A**.**G****G****C****G****G**GGAGGCGGACG | |
|  |  | NZ\_AAHP01000071.1/7990-8257  | CUAAACUGGCG**C****C****G****C****C**..**U**.**C**..**G**.**G**.U...**G****C**.**U**.**C****G****C****G**C..................................GCUU..........CG**C****G****C****G****C**.**G****G****U**..U....AA..**A****C**..**G**..**G**.G..AA.**G**..**C**.**A**.**G****G**.**G**CGCGACGUCCGCCAGGGCCGAG.................................................................................................................................................C**C**AA.....**C****C****U****G**.**C****G****C****U****G****C**C..C.CC..**G****C****A****A****C****G****G****U**AA..**G****C****G****A**UCGCAUCGGAGCCGAUCCGAUGCC.............................AGCCGC**A****C****U****C****U****C**GAUGCGCCGGCACGGCCGCGAUGGCCGGCCGCCGGCGCGGU......................................................**G****C****C**A...**C**.**U****G****C****G****C**.................UUCGC................................................................................**G****C****G****C****G****G**G.A.A.**G****G****C**...**G****A****G**..**G****C****G**GUGACG----.......................................................................................................**U****C****G****C**...G.**A**.....**G**.**C**....**C****C****G**....**G**AU..A**C**.**C**..**G**.....G...**C****C**.**G**..**A**.**G****G****C****G****G**GGAGGCGGACG | |
|  |  | NZ\_AAHR01000031.1/79565-79298  | CUAAACUGGCG**C****C****G****C****C**..**U**.**C**..**G**.**G**.U...**G****C**.**U**.**C****G****C****G**C..................................GCUU..........CG**C****G****C****G****C**.**G****G****U**..U....AA..**A****C**..**G**..**G**.G..AA.**G**..**C**.**A**.**G****G**.**G**CGCGACGUCCGCCAGGGCCGAG.................................................................................................................................................C**C**AA.....**C****C****U****G**.**C****G****C****U****G****C**C..C.CC..**G****C****A****A****C****G****G****U**AA..**G****C****G****A**UCGCAUCGGAGCCGAUCCGAUGCC.............................AGCCGC**A****C****U****C****U****C**GAUGCGCCGGCACGGCCGCGAUGGCCGGCCGCCGGCGCGGU......................................................**G****C****C**A...**C**.**U****G****C****G****C**.................UUCGC................................................................................**G****C****G****C****G****G**G.A.A.**G****G****C**...**G****A****G**..**G****C****G**GUGACG----.......................................................................................................**U****C****G****C**...G.**A**.....**G**.**C**....**C****C****G**....**G**AU..A**C**.**C**..**G**.....G...**C****C**.**G**..**A**.**G****G****C****G****G**GGAGGCGGACG | |
|  |  | NZ\_AAHS01000036.1/778-511  | CUAAACUGGCG**C****C****G****C****C**..**U**.**C**..**G**.**G**.U...**G****C**.**U**.**C****G****C****G**C..................................GCUU..........CG**C****G****C****G****C**.**G****G****U**..U....AA..**A****C**..**G**..**G**.G..AA.**G**..**C**.**A**.**G****G**.**G**CGCGACGUCCGCCAGGGCCGAG.................................................................................................................................................C**C**AA.....**C****C****U****G**.**C****G****C****U****G****C**C..C.CC..**G****C****A****A****C****G****G****U**AA..**G****C****G****A**UCGCAUCGGAGCCGAUCCGAUGCC.............................AGCCGC**A****C****U****C****U****C**GAUGCGCCGGCACGGCCGCGAUGGCCGGCCGCCGGCGCGGU......................................................**G****C****C**A...**C**.**U****G****C****G****C**.................UUCGC................................................................................**G****C****G****C****G****G**G.A.A.**G****G****C**...**G****A****G**..**G****C****G**GUGACG----.......................................................................................................**U****C****G****C**...G.**A**.....**G**.**C**....**C****C****G**....**G**AU..A**C**.**C**..**G**.....G...**C****C**.**G**..**A**.**G****G****C****G****G**GGAGGCGGACG | |
|  |  | NZ\_AAHT01000001.1/1230426-1230693  | CUAAACUGGCG**C****C****G****C****C**..**U**.**C**..**G**.**G**.U...**G****C**.**U**.**C****G****C****G**C..................................GCUU..........CG**C****G****C****G****C**.**G****G****U**..U....AA..**A****C**..**G**..**G**.G..AA.**G**..**C**.**A**.**G****G**.**G**CGCGACGUCCGCCAGGGCCGAG.................................................................................................................................................C**C**AA.....**C****C****U****G**.**C****G****C****U****G****C**C..C.CC..**G****C****A****A****C****G****G****U**AA..**G****C****G****A**UCGCAUCGGAGCCGAUCCGAUGCC.............................AGCCGC**A****C****U****C****U****C**GAUGCGCCGGCACGGCCGCGAUGGCCGGCCGCCGGCGCGGU......................................................**G****C****C**A...**C**.**U****G****C****G****C**.................UUCGC................................................................................**G****C****G****C****G****G**G.A.A.**G****G****C**...**G****A****G**..**G****C****G**GUGACG----.......................................................................................................**U****C****G****C**...G.**A**.....**G**.**C**....**C****C****G**....**G**AU..A**C**.**C**..**G**.....G...**C****C**.**G**..**A**.**G****G****C****G****G**GGAGGCGGACG | |
|  |  | NZ\_AAHU01000019.1/122767-123034  | CUAAACUGGCG**C****C****G****C****C**..**U**.**C**..**G**.**G**.U...**G****C**.**U**.**C****G****C****G**C..................................GCUU..........CG**C****G****C****G****C**.**G****G****U**..U....AA..**A****C**..**G**..**G**.G..AA.**G**..**C**.**A**.**G****G**.**G**CGCGACGUCCGCCAGGGCCGAG.................................................................................................................................................C**C**AA.....**C****C****U****G**.**C****G****C****U****G****C**C..C.CC..**G****C****A****A****C****G****G****U**AA..**G****C****G****A**UCGCAUCGGAGCCGAUCCGAUGCC.............................AGCCGC**A****C****U****C****U****C**GAUGCGCCGGCACGGCCGCGAUGGCCGGCCGCCGGCGCGGU......................................................**G****C****C**A...**C**.**U****G****C****G****C**.................UUCGC................................................................................**G****C****G****C****G****G**G.A.A.**G****G****C**...**G****A****G**..**G****C****G**GUGACG----.......................................................................................................**U****C****G****C**...G.**A**.....**G**.**C**....**C****C****G**....**G**AU..A**C**.**C**..**G**.....G...**C****C**.**G**..**A**.**G****G****C****G****G**GGAGGCGGACG | |
|  |  | NZ\_AAHV01000029.1/13724-13457  | CUAAACUGGCG**C****C****G****C****C**..**U**.**C**..**G**.**G**.U...**G****C**.**U**.**C****G****C****G**C..................................GCUU..........CG**C****G****C****G****C**.**G****G****U**..U....AA..**A****C**..**G**..**G**.G..AA.**G**..**C**.**A**.**G****G**.**G**CGCGACGUCCGCCAGGGCCGAG.................................................................................................................................................C**C**AA.....**C****C****U****G**.**C****G****C****U****G****C**C..C.CC..**G****C****A****A****C****G****G****U**AA..**G****C****G****A**UCGCAUCGGAGCCGAUCCGAUGCC.............................AGCCGC**A****C****U****C****U****C**GAUGCGCCGGCACGGCCGCGAUGGCCGGCCGCCGGCGCGGU......................................................**G****C****C**A...**C**.**U****G****C****G****C**.................UUCGC................................................................................**G****C****G****C****G****G**G.A.A.**G****G****C**...**G****A****G**..**G****C****G**GUGACG----.......................................................................................................**U****C****G****C**...G.**A**.....**G**.**C**....**C****C****G**....**G**AU..A**C**.**C**..**G**.....G...**C****C**.**G**..**A**.**G****G****C****G****G**GGAGGCGGACG | |
|  |  | NZ\_AAHW01000088.1/10515-10248  | CUAAACUGGCG**C****C****G****C****C**..**U**.**C**..**G**.**G**.U...**G****C**.**U**.**C****G****C****G**C..................................GCUU..........CG**C****G****C****G****C**.**G****G****U**..U....AA..**A****C**..**G**..**G**.G..AA.**G**..**C**.**A**.**G****G**.**G**CGCGACGUCCGCCAGGGCCGAG.................................................................................................................................................C**C**AA.....**C****C****U****G**.**C****G****C****U****G****C**C..C.CC..**G****C****A****A****C****G****G****U**AA..**G****C****G****A**UCGCAUCGGAGCCGAUCCGAUGCC.............................AGCCGC**A****C****U****C****U****C**GAUGCGCCGGCACGGCCGCGAUGGCCGGCCGCCGGCGCGGU......................................................**G****C****C**A...**C**.**U****G****C****G****C**.................UUCGC................................................................................**G****C****G****C****G****G**G.A.A.**G****G****C**...**G****A****G**..**G****C****G**GUGACG----.......................................................................................................**U****C****G****C**...G.**A**.....**G**.**C**....**C****C****G**....**G**AU..A**C**.**C**..**G**.....G...**C****C**.**G**..**A**.**G****G****C****G****G**GGAGGCGGACG | |
|  |  | NC\_006348.1/707548-707816  | CUAAACUGGCG**C****C****G****C****C**..**U**.**C**..**G**.**G**.U...**G****C**.**U**.**C****G****C****G**C..................................GCUU..........CG**C****G****C****G****C**.**G****G****U**..U....AA..**A****C**..**G**..**G**.G..AA.**G**..**C**.**A**.**G****G**.**G**CGCGACGUCCGCCAGGGCCGAG.................................................................................................................................................C**C**AA.....**C****C****U****G**.**C****G****C****U****G****C**C..C.CC..**G****C****A****A****C****G****G****U**AA..**G****C****G****A**UCGCAUCGGAGCCGAUCCGAUGCC.............................AGCCGC**A****C****U****C****U****C**GAUGCGCCGGCACGGCCGCGAUGGCCGUGCCGCCGGCGCGGU.....................................................**G****C****C**A...**C**.**U****G****C****G****C**.................UUCGC................................................................................**G****C****G****C****G****G**G.A.A.**G****G****C**...**G****A****G**..**G****C****G**GUGACG----.......................................................................................................**U****C****G****C**...G.**A**.....**G**.**C**....**C****C****G**....**G**AU..A**C**.**C**..**G**.....G...**C****C**.**G**..**A**.**G****G****C****G****G**GGAGGCGGACG | |
|  |  | NZ\_AAHN01000031.1/48621-48889  | CUAAACUGGCG**C****C****G****C****C**..**U**.**C**..**G**.**G**.U...**G****C**.**U**.**C****G****C****G**C..................................GCUU..........CG**C****G****C****G****C**.**G****G****U**..U....AA..**A****C**..**G**..**G**.G..AA.**G**..**C**.**A**.**G****G**.**G**CGCGACGUCCGCCAGGGCCGAG.................................................................................................................................................C**C**AA.....**C****C****U****G**.**C****G****C****U****G****C**C..C.CC..**G****C****A****A****C****G****G****U**AA..**G****C****G****A**UCGCAUCGGAGCCGAUCCGAUGCC.............................AGCCGC**A****C****U****C****U****C**GAUGCGCCGGCACGGCCGCGAUGGCCGUGCCGCCGGCGCGGU.....................................................**G****C****C**A...**C**.**U****G****C****G****C**.................UUCGC................................................................................**G****C****G****C****G****G**G.A.A.**G****G****C**...**G****A****G**..**G****C****G**GUGACG----.......................................................................................................**U****C****G****C**...G.**A**.....**G**.**C**....**C****C****G**....**G**AU..A**C**.**C**..**G**.....G...**C****C**.**G**..**A**.**G****G****C****G****G**GGAGGCGGACG | |
|  |  | NZ\_AAHO01000084.1/8522-8790  | CUAAACUGGCG**C****C****G****C****C**..**U**.**C**..**G**.**G**.U...**G****C**.**U**.**C****G****C****G**C..................................GCUU..........CG**C****G****C****G****C**.**G****G****U**..U....AA..**A****C**..**G**..**G**.G..AA.**G**..**C**.**A**.**G****G**.**G**CGCGACGUCCGCCAGGGCCGAG.................................................................................................................................................C**C**AA.....**C****C****U****G**.**C****G****C****U****G****C**C..C.CC..**G****C****A****A****C****G****G****U**AA..**G****C****G****A**UCGCAUCGGAGCCGAUCCGAUGCC.............................AGCCGC**A****C****U****C****U****C**GAUGCGCCGGCACGGCCGCGAUGGCCGUGCCGCCGGCGCGGU.....................................................**G****C****C**A...**C**.**U****G****C****G****C**.................UUCGC................................................................................**G****C****G****C****G****G**G.A.A.**G****G****C**...**G****A****G**..**G****C****G**GUGACG----.......................................................................................................**U****C****G****C**...G.**A**.....**G**.**C**....**C****C****G**....**G**AU..A**C**.**C**..**G**.....G...**C****C**.**G**..**A**.**G****G****C****G****G**GGAGGCGGACG | |
|  |  | NZ\_AAHQ01000002.1/571122-571390  | CUAAACUGGCG**C****C****G****C****C**..**U**.**C**..**G**.**G**.U...**G****C**.**U**.**C****G****C****G**C..................................GCUU..........CG**C****G****C****G****C**.**G****G****U**..U....AA..**A****C**..**G**..**G**.G..AA.**G**..**C**.**A**.**G****G**.**G**CGCGACGUCCGCCAGGGCCGAG.................................................................................................................................................C**C**AA.....**C****C****U****G**.**C****G****C****U****G****C**C..C.CC..**G****C****A****A****C****G****G****U**AA..**G****C****G****A**UCGCAUCGGAGCCGAUCCGAUGCC.............................AGCCGC**A****C****U****C****U****C**GAUGCGCCGGCACGGCCGCGAUGGCCGUGCCGCCGGCGCGGU.....................................................**G****C****C**A...**C**.**U****G****C****G****C**.................UUCGC................................................................................**G****C****G****C****G****G**G.A.A.**G****G****C**...**G****A****G**..**G****C****G**GUGACG----.......................................................................................................**U****C****G****C**...G.**A**.....**G**.**C**....**C****C****G**....**G**AU..A**C**.**C**..**G**.....G...**C****C**.**G**..**A**.**G****G****C****G****G**GGAGGCGGACG | |
|  |  | NC\_005126.1/3500805-3500618  | GGGUAUAAACC**G****C****C****G****U**..**U**.**A**..**G**.**G**.UU..**A****A**.**U**.**A****C****C****A**CAC................................CUUA...........A**U****G****G****U****G**.**U****G****G**..U....--..-**U**..**G**..**G**.G..AA.**G**..**G**.**A**.**G****G**.**U**GA.....................................................................................................................................................................A**A**GU.....**C****C****U****C**.**C****G****C****A****G****C**C..C.CC..**G****C****U****G****C****U****G****U**GA..**U****G****C**-.....................................................UGACAA**C****U****C****C****G****C**UGAU-..........................................................................................**G****C****C**A...**C**.**U****G****G****U****C**.................GGAAA................................................................................**G****A****C****U****G****G**G.A.A.**G****G****U**..U**G****C****G**..**G****G****G**AAGGGUGAC-.......................................................................................................-**G****C****U**...A.**A**.....**G**.**C**....**C****A****G**....**A**AG..A**C**.**C**..**G**.....A...**C****C**.**U**..**G**.**A****C****A****G****G**CACGAGACAUU | |
|  |  | NZ\_AAHJ01000001.1/91154-90913  | UUCUUUCUCGU**C****A****U****A****A**..**C**.**A**..**G**.**G**.UG..**C****C**.**G**.**G****C****U**-...................................UGAA............-**A****G****C****C**.**G****G****A**..G....AA..**U****A**..**G**..**G**.G..AA.**G**..**U**.**A**.**C****G**.**U**GA.....................................................................................................................................................................G**A**UU.....**C****G****U****A**.**C****A****C****U****G****U**A..C.CC..**G****C****A****A****C****U****G****U**ACA.**A****C****G****G**UAA..................................................ACCGCC**G****A****U****C****A****G**AGCACAUGACCUUGUGUGCACGAAAAGCGGUCUUUUCAG........................................................**G****U****C**A...**C**.**U****G****C****G****C**UGUU.............UUCCU......................................................................CCCAGGAAAA**G****C****G****C****G****G**G.A.A.**G****G****U**...**C****U****G**..**A****A****A**AGGAUGUAAA.......................................................................................................**C****C****G****U**.UAA.**A**.....**G**.**U**....**C****A****G**....**G**AG..A**C**.**C**..**U**.....G...**C****C**.**G**..**G**.**U****U****A****U****G**CUAUUGCAAUC | |
[truncated: 489,811 more chars]
