# Supplementary material for: The distributions, mechanisms, and structures of metabolite-binding riboswitches
Source: Genome Biol. 2007 Nov 12;8(11):R239. doi: 10.1186/gb-2007-8-11-r239 (PMC2258182; doi:10.1186/gb-2007-8-11-r239)
Supplement: Additional data file 2 — Sequence alignments of the riboswitch aptamer data sets annotated with new base-base interactions in HTML format. [file gb-2007-8-11-r239-S2.zip › HTML/FMN.html]

|  |  |  |  |  |
| --- | --- | --- | --- | --- |
|  |  | **Accession/Start-End** |  | **Sequence** |
|  |  | NZ\_AACK01000020.1/31945-31820  | **A****A****U****A****A****U****C****U****U****C**.A..GGG.C**A****G****G****G****U**GA...............A**A**UU**C****C****C****G**.AUC.G**G****C****G****G****U**A-..........................................................................................................................A**A****G****U**.**C****C****G****C**GA.**G****C****C****G****A****A****C**GA.......................................................................................................AAAA.................................G**G****U****U****U**....**G****G****C**AGGA....**A**.**C****C****G****G****U**G.A.......G.......**A**..UU.........................**C****C****G****G****U****A****C**.C.GA**C****A****G**.UA..U.A.GU................................**C****U****G**.G.AUGGA.A.**G**..**A**.**A****G****A****U****G****A****A****A** | |
|  |  | NZ\_AAGO01000045.1/7697-7813  | **A****U****A****A****A****U****C****U****U****C**.A..GGG.C**A****G****G****G****U**GA...............A**A**UU**C****C****C****U**.ACC.G**G****C****G****G****U**A-..........................................................................................................................U**A****G****C**.**C****C****G****C**GA.**G****C****U****G****C****U**-.........................................................................................................----..................................-**A****G****C**....**A****G****C**AUGA....**U**.**U****C****G****G****U**G.A.......A.......**A**..CU.........................**C****C****G****A****G****G****C**.C.GA**C****A****G**.UA..U.A.GU................................**C****U****G**.G.AUGAA.A.**G**..**A**.**A****G****A****U****A****A****A****A** | |
|  |  | NC\_000964.2/2410071-2409937  | **A****U****C****A****A****U****C****U****U****C**.G..GGG.C**A****G****G****G****U**GA...............A**A**UU**C****C****C****U**.ACC.G**G****C****G****G****U**GAUGAGCCAAUGGCUCU...........................................................................................................A**A****G****C**.**C****C****G****C**GA.**G****C****U****G****U**--.........................................................................................................CUUU..................................--**A****C**....**A****G****C**AGGA....**U**.**U****C****G****G****U**G.A.......G.......**A**..UU.........................**C****C****G****G****A****G****C**.C.GA**C****A****G**.UA..C.A.GU................................**C****U****G**.G.AUGGG.A.**G**..**A**.**A****G****A****U****G****G****A****G** | |
|  |  | NC\_003028.1/171686-171572  | **A****A****C****U****A****U****C****U****U****C**.A..GGG.C**A****G****G****G****U**GA...............A**A**UU**C****C****C****U**.ACC.G**G****U****G****G****U**A-..........................................................................................................................U**A****G****C**.**C****C****A****C**GA.**G****C****C**----.........................................................................................................GAAA..................................----....**G****G****C**AUGA....**U**.**U****U****G****G****U**G.A.......A.......**A**..UU.........................**C****C****A****A****A****G****C**.C.GA**C****A****G**.UA..U.A.GU................................**C****U****G**.G.AUGAA.A.**G**..**A**.**A****G****A****U****A****A****A****A** | |
|  |  | NZ\_AAGY01000091.1/5945-5831  | **A****A****C****U****A****U****C****U****U****C**.A..GGG.C**A****G****G****G****U**GA...............A**A**UU**C****C****C****U**.ACC.G**G****U****G****G****U**A-..........................................................................................................................U**A****G****C**.**C****C****A****C**GA.**G****C****C**----.........................................................................................................GAAA..................................----....**G****G****C**AUGA....**U**.**U****U****G****G****U**G.A.......A.......**A**..UU.........................**C****C****A****A****A****G****C**.C.GA**C****A****G**.UA..U.A.GU................................**C****U****G**.G.AUGAA.A.**G**..**A**.**A****G****A****U****A****A****A****A** | |
|  |  | NC\_003366.1/704095-704210  | **A****A****A****A****U****U****C****U****U****C**.A..GGG.C**A****G****G****G****U**GA...............A**A**UU**C****C****C****U**.ACC.G**G****C****G****G****U**A-..........................................................................................................................A**A****G****C**.**C****C****G****C**GA.**G****C****U****A**---.........................................................................................................GUU-..................................---**U**....**A****G****C**AUGA....**U**.**U****C****G****G****U**G.A.......A.......**A**..UU.........................**C****C****G****A****G****G****C**.C.GA**C****A****G**.UA..A.A.GU................................**C****U****G**.G.AUGAA.A.**G**..**A**.**A****G****A****A****U****A****U****C** | |
|  |  | NC\_003030.1/2971922-2971806  | **A****A****U****G****A****U****C****U****U****C**.A..GGG.C**A****G****G****G****U**GA...............A**A**UU**C****C****C****U**.ACC.G**G****C****G****G****U**A-..........................................................................................................................U**A****G****C**.**C****C****G****C**GA.**G****C****C****U**---.........................................................................................................UUUU..................................---**A**....**G****G****U**AUGA....**U**.**C****C****G****G****U**U.U.......G.......**A**..UU.........................**C****C****G****G****A****G****C**.C.GA**C****A****G**.UA..A.A.GU................................**C****U****G**.G.AUGAA.A.**G**..**A**.**A****G****A****U****A****U****A****U** | |
|  |  | NC\_003098.1/174006-173892  | **A****A****C****U****A****U****C****U****U****C**.A..GGG.C**A****G****G****G****U**GC...............A**A**UU**C****C****C****U**.ACC.G**G****U****G****G****U**A-..........................................................................................................................U**A****G****C**.**C****C****A****C**GA.**G****C****C**----.........................................................................................................GAAA..................................----....**G****G****C**AUGA....**U**.**U****U****G****G****U**G.A.......A.......**A**..UU.........................**C****C****A****A****A****G****C**.C.GA**C****A****G**.UA..U.A.GU................................**C****U****G**.G.AUGAA.A.**G**..**A**.**A****G****A****U****A****A****A****A** | |
|  |  | NC\_003869.1/541530-541649  | **U****A****A****A****A****U****C****U****U****C**.G..GGG.C**A****G****G****G****U**GG...............A**A**UU**C****C****C****G**.ACC.G**G****C****A****G****U**GA..........................................................................................................................A**A****G****C**.**C****U****G****C**GAG**C****C****G****C**---.........................................................................................................AUAU..................................---**G**....**C****G****G**UUGA....**C**.**U****C****G****G****U**G.A.......A.......**A**..UU.........................**C****C****G****A****G****G****C**.C.GA**C****G****G**UAA..A.A.GU................................**C****C****G**.G.AUGGG.A.**G**..**A**.**A****G****A****U****U****U****A****C** | |
|  |  | NC\_002662.1/1021729-1021845  | **A****U****A****A****A****U****C****U****U****C**.A..GGG.C**A****G****G****G****U**GU...............A**A**UU**C****C****C****U**.ACC.G**G****C****G****G****U**A-..........................................................................................................................U**A****G****C**.**C****C****G****C**GA.**G****C****U****G****C**--.........................................................................................................--UU..................................--**G****C**....**A****G****C**AUGA....**U**.**U****C****G****G****U**G.A.......A.......**A**..CU.........................**C****C****G****A****G****G****C**.C.GA**C****A****G**.UA..U.A.GU................................**C****U****G**.G.AUGAA.A.**G**..**A**.**A****G****A****U****A****A****U****A** | |
|  |  | NC\_004557.1/318413-318527  | **A****A****A****U****A****U****C****U****U****C**.A..GGG.C**G****G****G****G****U**GU...............A**A**UU**C****C****C****C**.ACC.G**G****C****G****G****U**A-..........................................................................................................................U**A****G****C**.**C****C****G****C**GA.**G****C****C**----.........................................................................................................GCAA..................................----....**G****G****C**AUGA....**U**.**U****C****G****G****U**G.U.......A.......**A**..CU.........................**C****C****G****A****A****G****C**.C.GA**C****A****G**.UA..A.A.GU................................**C****U****G**.G.AUGAA.A.**G**..**A**.**A****G****G****U****A****U****A****A** | |
|  |  | NC\_006270.2/2381339-2381206  | **A****U****C****A****A****U****C****U****U****C**.G..GGG.C**A****G****G****G****U**GA...............A**A**UU**C****C****C****U**.ACC.G**G****C****G****G****U**GAUGAGCAAGCGCUCU............................................................................................................A**A****G****C**.**C****C****G****C**GA.**G****C****C****U****U**--.........................................................................................................UUUA..................................--**U****G**....**G****G****C**AGGA....**U**.**U****C****G****G****U**G.C.......G.......**A**..UU.........................**C****C****G****A****A****G****C**.C.GA**C****A****G**.UA..U.A.GU................................**C****U****G**.G.AUGGG.A.**G**..**A**.**A****G****A****U****G****G****A****G** | |
|  |  | NC\_006322.1/2382202-2382069  | **A****U****C****A****A****U****C****U****U****C**.G..GGG.C**A****G****G****G****U**GA...............A**A**UU**C****C****C****U**.ACC.G**G****C****G****G****U**GAUGAGCAAGCGCUCU............................................................................................................A**A****G****C**.**C****C****G****C**GA.**G****C****C****U****U**--.........................................................................................................UUUA..................................--**U****G**....**G****G****C**AGGA....**U**.**U****C****G****G****U**G.C.......G.......**A**..UU.........................**C****C****G****A****A****G****C**.C.GA**C****A****G**.UA..U.A.GU................................**C****U****G**.G.AUGGG.A.**G**..**A**.**A****G****A****U****G****G****A****G** | |
|  |  | NC\_002678.2/6017355-6017207  | **U****A****A****A****G****U****U****C****U****C**.A..GGG.C**G****G****G****G****U**GA...............A**A**GU**C****C****C****C**.ACC.G**G****C****G****G****U**AAGGGCCUCAAACCC.............................................................................................................A**A****G****C**.**C****C****G****C**GA.**G****C****G****C****U****U****U**CCAAGA...................................................................................................CAAU...............................UGG**A****A****A****G**.GUC**A****G****C**A-GA....**U**.**C****C****G****G****U**G.U.......G.......**A**..UU.........................**C****C****G****G****A****G****C**.C.GA**C****G****G**UUA..G.A.GU................................**C****C****G**.G.AUGAA.A.**G**..**A**.**G****G****A****C****G****A****A****A** | |
|  |  | NC\_000853.1/1804292-1804171  | **A****A****A****C****G****C****U****C****U****C**.G..GGG.C**A****G****G****G****U**GG...............A**A**UU**C****C****C****G**.ACC.G**G****C****G****G****U**GA..........................................................................................................................A**A****G****C**.**C****C****G****C**GA.**G****C****C****C****U****C**-.........................................................................................................UCAG..................................-**G****A****G**....**G****G****U**U-GA....**C**.**C****C****G****G****U**G.G.......A.......**A**..UU.........................**C****C****G****G****G****G****C**.C.GA**C****G****G**UGA..A.A.GU................................**C****C****G**.G.AUGGG.A.**G**..**A**.**G****A****G****C****G****U****G****A** | |
|  |  | NC\_006510.1/1793427-1793295  | **A****U****U****C****A****U****C****U****U****C**.G..GGG.C**A****G****G****G****U**GA...............A**A**UU**C****C****C****G**.ACC.G**G****C****G****G****U**GAUGAGGCGCUUGGCCUCU.........................................................................................................C**A****G****C**.**C****C****G****C**GA.**G****C****C**----.........................................................................................................GCAA..................................----....**G****G****C**AGGA....**U**.**C****C****G****G****U**G.C.......G.......**A**..GU.........................**C****C****G****G****A****G****C**.C.GA**C****A****G**.UA..U.A.GU................................**C****U****G**.G.AUGGG.A.**G**..**A**.**A****G****A****U****G****A****A****G** | |
|  |  | NC\_003062.1/1871994-1872144  | **G****G****U****U****G****U****U****C****U****C**.A..GGG.C**G****G****G****G****U**GC...............A**A**UU**C****C****C****C**.ACC.G**G****C****G****G****U**AUCGGGAUUUGUCCCG............................................................................................................G**A****G****C**.**C****C****G****C**GA.**G****C****G****C****U****U****C**CGGUCU...................................................................................................CCAA.............................AGCCG**G****A****A****G**.GUC**A****G****C**A-GA....**U**.**C****C****G****G****U**G.A.......G.......**A**..GG.........................**C****C****G****G****A****G****C**.C.GA**C****G****G**.UA..U.A.GU................................**C****C****G**.G.AUGGA.A.**G**..**A**.**G****G****A****C****A****A****G****G** | |
|  |  | NC\_003304.1/1872108-1872258  | **G****G****U****U****G****U****U****C****U****C**.A..GGG.C**G****G****G****G****U**GC...............A**A**UU**C****C****C****C**.ACC.G**G****C****G****G****U**AUCGGGAUUUGUCCCG............................................................................................................G**A****G****C**.**C****C****G****C**GA.**G****C****G****C****U****U****C**CGGUCU...................................................................................................CCAA.............................AGCCG**G****A****A****G**.GUC**A****G****C**A-GA....**U**.**C****C****G****G****U**G.A.......G.......**A**..GG.........................**C****C****G****G****A****G****C**.C.GA**C****G****G**.UA..U.A.GU................................**C****C****G**.G.AUGGA.A.**G**..**A**.**G****G****A****C****A****A****G****G** | |
|  |  | NZ\_AABO02000010.1/2370-2514  | **A****C****G****C****A****U****U****C****U****C**.A..GGG.C**A****G****G****G****U**GA...............A**A**UU**C****C****C****U**.ACC.G**G****U****G****G****U**AAAUAUUUCGAUAU..............................................................................................................A**A****G****C**.**C****C****A****C**GA.**G****C****G****C****U****U****A**CUUAU....................................................................................................CUUA.................................G**U****A****A****G**.GUC**A****G****C**A-GA....**U**.**U****U****G****G****U**G.A.......A.......**A**..AU.........................**C****C****A****A****A****G****C**.C.GA**C****A****G**UGA..U.A.GU................................**C****U****G**.G.AUGAA.A.**G**..**A**.**G****A****A****U****A****G****A****G** | |
|  |  | NZ\_AAFG02000006.1/142668-142537  | **U****U****C****C****A****U****U****C****U****C**.A..GGG.C**G****G****G****G****U**GA...............A**A**UU**C****C****C****C**.ACC.G**G****C****G****G****U**UA..........................................................................................................................U**A****G****C**.**C****C****G****C**GA.**G****C****G****C****C****A****C**CU.......................................................................................................CUAC...............................GGG**G****U****G****G**.GUC**A****G****C**A-GA....**U**.**C****C****G****G****U**G.G.......A.......**A**..UU.........................**C****C****G****G****G****G****C**.C.GA**C****G****G**UCA..U.A.GU................................**C****C****G**.G.AUGGG.A.**G**..**A**.**G****A****A****U****G****U****A****C** | |
|  |  | NC\_006570.1/1740360-1740509  | **A****A****U****A****A****U****C****U****U****C**.A..GGG.C**A****G****G****G****C**GA...............A**A**UU**C****C****C****U**.ACC.G**G****C****G****G****U**AACUGCUUAUAGCAG.............................................................................................................A**A****G****C**.**C****C****G****C**GA.**G****C****G****C****U****U****U**CUA......................................................................................................AUAA...........................AAAUAGA**G****A****G****G**.UCA**A****G****C**A-GA....**U**.**U****U****G****G****U**G.U.......A.......**A**..UU.........................**C****C****A****A****A****G****C**.C.GA**C****A****G**UUA..A.A.GU................................**C****U****G**.G.AUGAA.A.**G**..**A**.**A****G****A****U****U****G****A****G** | |
|  |  | NC\_004605.1/1323634-1323774  | **A****A****U****U****G****U****U****C****U****C**.A..GGG.C**G****G****G****G****C**GA...............A**A**UU**C****C****C****C**.ACC.G**G****C****G****G****U**AUACUUUUUCAAGU..............................................................................................................G**A****G****C**.**C****C****G****C**GA.**G****C****G****C****U****C****G**A........................................................................................................UUCG.................................U**C****G****A****G**.GUC**A****G****C**A-GA....**U**.**C****U****G****G****U**G.A.......G.......**A**..UG.........................**C****C****A****G****A****G****C**.C.GA**C****G****G**UUA..U.A.GU................................**C****C****G**.G.AUGAA.A.**G**..**A**.**G****A****A****U****A****A****G****A** | |
|  |  | NC\_003366.1/2484221-2484104  | **A****A****A****A****G****U****C****U****U****C**.A..GGG.C**G****G****G****G****U**GU...............G**A**GU**C****C****C****C**.ACC.G**G****C****G****G****U**A-..........................................................................................................................A**A****G****C**.**C****C****G****C**GA.**G****C****U****A****G**--.........................................................................................................-UUU..................................--**U****U**....**A****G****C**AUGA....**U**.**U****C****G****G****U**G.A.......A.......**A**..UU.........................**C****C****G****A****G****G****C**.C.GA**C****A****G**.UA..U.A.GU................................**C****U****G**.G.AUGAA.A.**G**..**A**.**A****G****A****U****G****A****G****U** | |
|  |  | NC\_004369.1/86557-86391  | **G****G****G****U****G****U****U****C****U****C**.A..GGG.C**G****G****G****G****U**GU...............A**A**UU**C****C****C****C**.ACC.G**G****C****G****G****U**GAGAGUGUCCUCCAGAUGGGGAGGCUC.................................................................................................A**A****G****C**.**C****C****G****C**GA.**G****C****G****C****C****A****C**C........................................................................................................GUAU....................UCCGCUUAUGGAGG**G****U****G****G**.GUC**A****G****C**A-GA....**U**.**C****C****G****G****U**G.U.......G.......**A**..UU.........................**C****C****G****G****A****G****C**.C.GA**C****G****G**UCA..U.A.GU................................**C****C****G**.G.AUGGA.A.**G**..**A**.**G****A****A****C****C****G****U****G** | |
|  |  | NZ\_AAEW01000017.1/10067-9921  | **C****C****C****U****A****U****U****C****U****C**.A..GGG.C**G****G****G****G****U**GA...............A**A**UU**C****C****C****C**.ACC.G**G****C****G****G****U**GACUCCUUGUGAG...............................................................................................................A**A****G****C**.**C****C****G****C**GA.**G****C****G****C****C****U****U**UA.......................................................................................................UUAU...........................UGGGAUA**A****A****G****G**.GUC**A****G****C**A-GA....**U**.**C****U****G****G****U**G.A.......G.......**A**..UU.........................**C****C****A****G****A****G****C**.C.GA**C****G****G**UUA..U.A.GU................................**C****C****G**.G.AUGAA.A.**G**..**A**.**G****G****A****U****A****U****U****C** | |
|  |  | NC\_003997.3/1422565-1422704  | **G****U****C****U****A****U****C****U****U****C**.G..GGG.C**A****G****G****G****U**GA...............A**A**AU**C****C****C****G**.ACC.G**G****C****G****G****U**GAUGAACUAUUUAUGAUUUUGUUCU...................................................................................................A**A****G****C**.**C****C****G****C**GA.**G****C****C****G**---.........................................................................................................-UUA..................................---**A**....**G****G****C**AGGA....**U**.**U****U****G****G****U**G.U.......G.......**A**..UU.........................**C****C****A****A****A****G****C**.C.GA**C****A****G**.UA..U.A.GU................................**C****U****G**.G.AUGGG.A.**G**..**A**.**A****G****A****U****G****G****A****G** | |
|  |  | NC\_004722.1/1439581-1439720  | **G****U****C****U****A****U****C****U****U****C**.G..GGG.C**A****G****G****G****U**GA...............A**A**AU**C****C****C****G**.ACC.G**G****C****G****G****U**GAUGAACUAUUUAUGAUUUUGUUCU...................................................................................................A**A****G****C**.**C****C****G****C**GA.**G****C****C****G**---.........................................................................................................-UUA..................................---**A**....**G****G****C**AGGA....**U**.**U****U****G****G****U**G.U.......G.......**A**..UU.........................**C****C****A****A****A****G****C**.C.GA**C****A****G**.UA..U.A.GU................................**C****U****G**.G.AUGGG.A.**G**..**A**.**A****G****A****U****G****G****A****G** | |
|  |  | NC\_005945.1/1422634-1422773  | **G****U****C****U****A****U****C****U****U****C**.G..GGG.C**A****G****G****G****U**GA...............A**A**AU**C****C****C****G**.ACC.G**G****C****G****G****U**GAUGAACUAUUUAUGAUUUUGUUCU...................................................................................................A**A****G****C**.**C****C****G****C**GA.**G****C****C****G**---.........................................................................................................-UUA..................................---**A**....**G****G****C**AGGA....**U**.**U****U****G****G****U**G.U.......G.......**A**..UU.........................**C****C****A****A****A****G****C**.C.GA**C****A****G**.UA..U.A.GU................................**C****U****G**.G.AUGGG.A.**G**..**A**.**A****G****A****U****G****G****A****G** | |
|  |  | NC\_007530.2/1422688-1422827  | **G****U****C****U****A****U****C****U****U****C**.G..GGG.C**A****G****G****G****U**GA...............A**A**AU**C****C****C****G**.ACC.G**G****C****G****G****U**GAUGAACUAUUUAUGAUUUUGUUCU...................................................................................................A**A****G****C**.**C****C****G****C**GA.**G****C****C****G**---.........................................................................................................-UUA..................................---**A**....**G****G****C**AGGA....**U**.**U****U****G****G****U**G.U.......G.......**A**..UU.........................**C****C****A****A****A****G****C**.C.GA**C****A****G**.UA..U.A.GU................................**C****U****G**.G.AUGGG.A.**G**..**A**.**A****G****A****U****G****G****A****G** | |
|  |  | NZ\_AAEN01000008.1/1395-1256  | **G****U****C****U****A****U****C****U****U****C**.G..GGG.C**A****G****G****G****U**GA...............A**A**AU**C****C****C****G**.ACC.G**G****C****G****G****U**GAUGAACUAUUUAUGAUUUUGUUCU...................................................................................................A**A****G****C**.**C****C****G****C**GA.**G****C****C****G**---.........................................................................................................-UUA..................................---**A**....**G****G****C**AGGA....**U**.**U****U****G****G****U**G.U.......G.......**A**..UU.........................**C****C****A****A****A****G****C**.C.GA**C****A****G**.UA..U.A.GU................................**C****U****G**.G.AUGGG.A.**G**..**A**.**A****G****A****U****G****G****A****G** | |
|  |  | NZ\_AAEO01000017.1/49710-49849  | **G****U****C****U****A****U****C****U****U****C**.G..GGG.C**A****G****G****G****U**GA...............A**A**AU**C****C****C****G**.ACC.G**G****C****G****G****U**GAUGAACUAUUUAUGAUUUUGUUCU...................................................................................................A**A****G****C**.**C****C****G****C**GA.**G****C****C****G**---.........................................................................................................-UUA..................................---**A**....**G****G****C**AGGA....**U**.**U****U****G****G****U**G.U.......G.......**A**..UU.........................**C****C****A****A****A****G****C**.C.GA**C****A****G**.UA..U.A.GU................................**C****U****G**.G.AUGGG.A.**G**..**A**.**A****G****A****U****G****G****A****G** | |
|  |  | NZ\_AAEP01000030.1/228594-228733  | **G****U****C****U****A****U****C****U****U****C**.G..GGG.C**A****G****G****G****U**GA...............A**A**AU**C****C****C****G**.ACC.G**G****C****G****G****U**GAUGAACUAUUUAUGAUUUUGUUCU...................................................................................................A**A****G****C**.**C****C****G****C**GA.**G****C****C****G**---.........................................................................................................-UUA..................................---**A**....**G****G****C**AGGA....**U**.**U****U****G****G****U**G.U.......G.......**A**..UU.........................**C****C****A****A****A****G****C**.C.GA**C****A****G**.UA..U.A.GU................................**C****U****G**.G.AUGGG.A.**G**..**A**.**A****G****A****U****G****G****A****G** | |
|  |  | NZ\_AAEQ01000023.1/228910-229049  | **G****U****C****U****A****U****C****U****U****C**.G..GGG.C**A****G****G****G****U**GA...............A**A**AU**C****C****C****G**.ACC.G**G****C****G****G****U**GAUGAACUAUUUAUGAUUUUGUUCU...................................................................................................A**A****G****C**.**C****C****G****C**GA.**G****C****C****G**---.........................................................................................................-UUA..................................---**A**....**G****G****C**AGGA....**U**.**U****U****G****G****U**G.U.......G.......**A**..UU.........................**C****C****A****A****A****G****C**.C.GA**C****A****G**.UA..U.A.GU................................**C****U****G**.G.AUGGG.A.**G**..**A**.**A****G****A****U****G****G****A****G** | |
|  |  | NZ\_AAER01000019.1/1243-1104  | **G****U****C****U****A****U****C****U****U****C**.G..GGG.C**A****G****G****G****U**GA...............A**A**AU**C****C****C****G**.ACC.G**G****C****G****G****U**GAUGAACUAUUUAUGAUUUUGUUCU...................................................................................................A**A****G****C**.**C****C****G****C**GA.**G****C****C****G**---.........................................................................................................-UUA..................................---**A**....**G****G****C**AGGA....**U**.**U****U****G****G****U**G.U.......G.......**A**..UU.........................**C****C****A****A****A****G****C**.C.GA**C****A****G**.UA..U.A.GU................................**C****U****G**.G.AUGGG.A.**G**..**A**.**A****G****A****U****G****G****A****G** | |
|  |  | NZ\_AAES01000029.1/228826-228965  | **G****U****C****U****A****U****C****U****U****C**.G..GGG.C**A****G****G****G****U**GA...............A**A**AU**C****C****C****G**.ACC.G**G****C****G****G****U**GAUGAACUAUUUAUGAUUUUGUUCU...................................................................................................A**A****G****C**.**C****C****G****C**GA.**G****C****C****G**---.........................................................................................................-UUA..................................---**A**....**G****G****C**AGGA....**U**.**U****U****G****G****U**G.U.......G.......**A**..UU.........................**C****C****A****A****A****G****C**.C.GA**C****A****G**.UA..U.A.GU................................**C****U****G**.G.AUGGG.A.**G**..**A**.**A****G****A****U****G****G****A****G** | |
|  |  | NC\_002745.2/1523932-1523798  | **A****U****U****C****A****U****C****U****U****C**.G..GGG.U**C****G****G****G****U**GU...............A**A**UU**C****C****C****A**.ACC.G**G****C****A****G****U**AAAUA.......................................................................................................................A**A****G****C**.**C****U****G****C**GA.**C****C****U****G****C****U****A**GUAU.....................................................................................................GUAU.............................CAUAU**U****A****G****U**....-**G****G**CUGA....**U**.**C****U****A****G****U**G.A.......G.......**A**..UU.........................**C****U****A****G****A****G****C**.C.GA**C****A****G**.UA..U.A.GU................................**C****U****G**.G.AUGGG.A.**G**..**A**.**A****G****A****U****G****G****A****G** | |
|  |  | NC\_002758.2/1600344-1600210  | **A****U****U****C****A****U****C****U****U****C**.G..GGG.U**C****G****G****G****U**GU...............A**A**UU**C****C****C****A**.ACC.G**G****C****A****G****U**AAAUA.......................................................................................................................A**A****G****C**.**C****U****G****C**GA.**C****C****U****G****C****U****A**GUAU.....................................................................................................GUAU.............................CAUAU**U****A****G****U**....-**G****G**CUGA....**U**.**C****U****A****G****U**G.A.......G.......**A**..UU.........................**C****U****A****G****A****G****C**.C.GA**C****A****G**.UA..U.A.GU................................**C****U****G**.G.AUGGG.A.**G**..**A**.**A****G****A****U****G****G****A****G** | |
|  |  | NC\_002951.2/1565491-1565357  | **A****U****U****C****A****U****C****U****U****C**.G..GGG.U**C****G****G****G****U**GU...............A**A**UU**C****C****C****A**.ACC.G**G****C****A****G****U**AAAUA.......................................................................................................................A**A****G****C**.**C****U****G****C**GA.**C****C****U****G****C****U****A**GUAU.....................................................................................................GUAU.............................CAUAU**U****A****G****U**....-**G****G**CUGA....**U**.**C****U****A****G****U**G.A.......G.......**A**..UU.........................**C****U****A****G****A****G****C**.C.GA**C****A****G**.UA..U.A.GU................................**C****U****G**.G.AUGGG.A.**G**..**A**.**A****G****A****U****G****G****A****G** | |
|  |  | NC\_002952.2/1589008-1588874  | **A****U****U****C****A****U****C****U****U****C**.G..GGG.U**C****G****G****G****U**GU...............A**A**UU**C****C****C****A**.ACC.G**G****C****A****G****U**AAAUA.......................................................................................................................A**A****G****C**.**C****U****G****C**GA.**C****C****U****G****C****U****A**GUAU.....................................................................................................GUAU.............................CAUAU**U****A****G****U**....-**G****G**CUGA....**U**.**C****U****A****G****U**G.A.......G.......**A**..UU.........................**C****U****A****G****A****G****C**.C.GA**C****A****G**.UA..U.A.GU................................**C****U****G**.G.AUGGG.A.**G**..**A**.**A****G****A****U****G****G****A****G** | |
|  |  | NC\_002953.3/1551439-1551305  | **A****U****U****C****A****U****C****U****U****C**.G..GGG.U**C****G****G****G****U**GU...............A**A**UU**C****C****C****A**.ACC.G**G****C****A****G****U**AAAUU.......................................................................................................................A**A****G****C**.**C****U****G****C**GA.**C****C****U****G****C****U****A**GUAU.....................................................................................................GUAU.............................CAUAU**U****A****G****U**....-**G****G**CUGA....**U**.**C****U****A****G****U**G.A.......G.......**A**..UU.........................**C****U****A****G****A****G****C**.C.GA**C****A****G**.UA..U.A.GU................................**C****U****G**.G.AUGGG.A.**G**..**A**.**A****G****A****U****G****G****A****G** | |
|  |  | NC\_003923.1/1525791-1525657  | **A****U****U****C****A****U****C****U****U****C**.G..GGG.U**C****G****G****G****U**GU...............A**A**UU**C****C****C****A**.ACC.G**G****C****A****G****U**AAAUU.......................................................................................................................A**A****G****C**.**C****U****G****C**GA.**C****C****U****G****C****U****A**GUAU.....................................................................................................GUAU.............................CAUAU**U****A****G****U**....-**G****G**CUGA....**U**.**C****U****A****G****U**G.A.......G.......**A**..UU.........................**C****U****A****G****A****G****C**.C.GA**C****A****G**.UA..U.A.GU................................**C****U****G**.G.AUGGG.A.**G**..**A**.**A****G****A****U****G****G****A****G** | |
|  |  | NZ\_AAIT01000004.1/236781-236653  | **G****C****G****U****G****U****U****C****U****C**.G..GGG.C**G****G****G****G****U**GC...............A**A**UU**C****C****C****C**.ACC.G**G****C****G****G****U**UA..........................................................................................................................C**A****G****C**.**C****C****G****C**GA.**G****C****G****C****C****U****G**CA.......................................................................................................AUCC.................................G**C****A****G****G**.GUC**A****G****C**A-GA....**U**.**C****C****G****G****U**G.A.......A.......**A**..CU.........................**C****C****G****G****G****G****C**.C.GA**C****G****G**.UA..U.A.GU................................**C****C****G**.G.AUGAA.A.**G**..**A**.**G****A****A****U****G****G****A****C** | |
|  |  | NZ\_AACJ01000024.1/8138-8282  | **A****U****G****C****A****U****U****C****U****C**.A..GGG.C**A****G****G****G****U**GA...............A**A**CU**C****C****C****U**.ACC.G**G****U****G****G****U**AAAUAUUUCGAUAU..............................................................................................................A**A****G****C**.**C****C****A****C**GA.**G****C****G****C****U****U****A**CUUAU....................................................................................................CUUA.................................G**U****A****A****G**.GUC**A****G****C**A-GA....**U**.**U****U****G****G****U**G.A.......A.......**A**..AU.........................**C****C****A****A****A****G****C**.C.GA**C****A****G**UGA..U.A.GU................................**C****U****G**.G.AUGAA.A.**G**..**A**.**G****A****A****U****A****G****A****G** | |
|  |  | NC\_004460.1/1572137-1571997  | **U****U****U****U****A****U****U****C****U****C**.A..GGG.C**G****G****G****G****C**GA...............A**A**UU**C****C****C****C**.ACC.G**G****C****G****G****U**AUACCGAAUGAGGU..............................................................................................................G**A****G****C**.**C****C****G****C**GA.**G****C****G****C****U****C****G**A........................................................................................................UUCG.................................U**C****G****A****G**.GUC**A****G****C**A-GA....**U**.**C****U****G****G****U**G.A.......G.......**A**..GG.........................**C****C****A****G****A****G****C**.C.GA**C****G****G**UUA..U.A.GU................................**C****C****G**.G.AUGAG.A.**G**..**A**.**G****A****A****U****G****A****A****A** | |
|  |  | NC\_005140.1/298514-298374  | **U****U****U****U****A****U****U****C****U****C**.A..GGG.C**G****G****G****G****C**GA...............A**A**UU**C****C****C****C**.ACC.G**G****C****G****G****U**AUACCGAAUGAGGU..............................................................................................................G**A****G****C**.**C****C****G****C**GA.**G****C****G****C****U****C****G**A........................................................................................................UUCG.................................U**C****G****A****G**.GUC**A****G****C**A-GA....**U**.**C****U****G****G****U**G.A.......G.......**A**..GG.........................**C****C****A****G****A****G****C**.C.GA**C****G****G**UUA..U.A.GU................................**C****C****G**.G.AUGAG.A.**G**..**A**.**G****A****A****U****G****A****A****A** | |
|  |  | NC\_006461.1/1011571-1011450  | **C****A****C****C****U****C****C****U****U****C**.G..GGG.C**G****G****G****G****U**GG...............A**A**GU**C****C****C****C**.ACC.G**G****C****G****G****U**GA..........................................................................................................................A**A****G****C**.**C****C****G****C**GAA**G****C****C****C****C**--.........................................................................................................CAAA..................................--**G****G**....**G****G****C**CCGA....**C**.**C****C****G****G****U**G.G.......A.......**A**..UU.........................**C****C****G****G****G****G****C**.C.GA**C****G****G**UGA..A.A.GU................................**C****C****G**.G.AUGGG.A.**G**..**A**.**A****G****G****A****G****G****G****C** | |
|  |  | NC\_003296.1/1513911-1513755  | **G****U****A****C****G****U****C****U****U****C**.A..GGG.C**G****G****G****G****U**GG...............A**A**UU**C****C****C****C**.ACC.G**G****C****G****G****U**AUGCGGGCCAUCGGUCUGC.........................................................................................................G**A****G****C**.**C****C****G****C**GA.**G****C****G****C****C****C****G**GCAUGCGCAGC..............................................................................................GCAU................................GC**C****G****G****G**.GUC**A****G****C**A-GA....**U**.**C****C****G****G****U**G.A.......G.......**A**..UG.........................**C****C****G****G****G****G****C**.C.GA**C****G****G**UCA..G.A.GU................................**C****C****G**.G.AUGGA.A.**G**..**A**.**A****G****A****U****G****U****G****C** | |
|  |  | NZ\_AABF02000010.1/25196-25311  | **A****A****U****A****A****U****C****U****U****C**.G..GGG.C**A****G****G****G****U**GA...............A**A**UU**C****C****C****G**.ACC.G**G****U****G****G****U**A-..........................................................................................................................U**A****G****U**.**C****C****A****C**GA.**A****A****G****C**---.........................................................................................................UUAU..................................---**G**....**C****U****U**U-GA....**U**.**U****U****G****G****U**G.A.......A.......**A**..UU.........................**C****C****A****A****A****A****C**.C.GA**C****A****G**.UA..G.A.GU................................**C****U****G**.G.AUGAG.A.**G**..**A**.**A****G****A****A****A****A****G****A** | |
|  |  | NC\_005835.1/684544-684423  | **C****A****C****C****U****C****C****U****U****C**.G..GGG.C**G****G****G****G****U**GG...............A**A**GU**C****C****C****C**.ACC.G**G****C****G****G****U**GA..........................................................................................................................A**A****G****C**.**C****C****G****C**GAA**G****C****C****C****C**--.........................................................................................................UAAA..................................--**G****G**....**G****G****C**CCGA....**C**.**C****C****G****G****U**G.G.......A.......**A**..UU.........................**C****C****G****G****G****G****C**.C.GA**C****G****G**UGA..A.A.GU................................**C****C****G**.G.AUGGG.A.**G**..**A**.**A****G****G****A****G****G****G****C** | |
|  |  | NZ\_AAEI01000030.1/41625-41469  | **G****A****A****C****G****U****U****C****U****C**.G..GGG.C**G****G****G****G****U**GA...............A**A**UU**C****C****C****C**.ACC.G**G****C****G****G****U**AAUCGCGCGAUUGCGCG...........................................................................................................U**A****G****C**.**C****C****G****C**GA.**G****C****G****C****U****U****G**CCGGCAC..................................................................................................GCAU..........................GUGCCCGG**C****A****A****G**.GUC**A****G****C**A-GA....**C**.**C****C****G****G****U**U.G.......G.......**A**..UU.........................**C****C****G****G****G****G****C**.C.GA**C****G****G**UCA..A.A.GU................................**C****C****G**.G.AUGAG.A.**G**..**A**.**G****A****G****C****G****G****G****G** | |
|  |  | NC\_000913.2/3182740-3182592  | **G****C****U****U****A****U****U****C****U****C**.A..GGG.C**G****G****G****G****C**GA...............A**A**UU**C****C****C****C**.ACC.G**G****C****G****G****U**AAAUCAACUCAGUUGA............................................................................................................A**A****G****C**.**C****C****G****C**GA.**G****C****G****C****U****U****U**GGGUGC...................................................................................................GAAC................................UC**A****A****A****G**.GAC**A****G****C**A-GA....**U**.**C****C****G****G****U**G.U.......A.......**A**..UU.........................**C****C****G****G****G****G****C**.C.GA**C****G****G**UUA..G.A.GU................................**C****C****G**.G.AUGGG.A.**G**..**A**.**G****A****G****U****A****A****C****G** | |
|  |  | NC\_002655.2/3998127-3997979  | **G****C****U****U****A****U****U****C****U****C**.A..GGG.C**G****G****G****G****C**GA...............A**A**UU**C****C****C****C**.ACC.G**G****C****G****G****U**AAAUCAACUCAGUUGA............................................................................................................A**A****G****C**.**C****C****G****C**GA.**G****C****G****C****U****U****U**GGGUGC...................................................................................................GAAC................................UC**A****A****A****G**.GAC**A****G****C**A-GA....**U**.**C****C****G****G****U**G.U.......A.......**A**..UU.........................**C****C****G****G****G****G****C**.C.GA**C****G****G**UUA..G.A.GU................................**C****C****G**.G.AUGGG.A.**G**..**A**.**G****A****G****U****A****A****C****G** | |
|  |  | NC\_002695.1/3930812-3930664  | **G****C****U****U****A****U****U****C****U****C**.A..GGG.C**G****G****G****G****C**GA...............A**A**UU**C****C****C****C**.ACC.G**G****C****G****G****U**AAAUCAACUCAGUUGA............................................................................................................A**A****G****C**.**C****C****G****C**GA.**G****C****G****C****U****U****U**GGGUGC...................................................................................................GAAC................................UC**A****A****A****G**.GAC**A****G****C**A-GA....**U**.**C****C****G****G****U**G.U.......A.......**A**..UU.........................**C****C****G****G****G****G****C**.C.GA**C****G****G**UUA..G.A.GU................................**C****C****G**.G.AUGGG.A.**G**..**A**.**G****A****G****U****A****A****C****G** | |
|  |  | NC\_004337.1/3177853-3177705  | **G****C****U****U****A****U****U****C****U****C**.A..GGG.C**G****G****G****G****C**GA...............A**A**UU**C****C****C****C**.ACC.G**G****C****G****G****U**AAAUCAACUCAGUUGA............................................................................................................A**A****G****C**.**C****C****G****C**GA.**G****C****G****C****U****U****U**GGGUGC...................................................................................................GAAC................................UC**A****A****A****G**.GAC**A****G****C**A-GA....**U**.**C****C****G****G****U**G.U.......A.......**A**..UU.........................**C****C****G****G****G****G****C**.C.GA**C****G****G**UUA..G.A.GU................................**C****C****G**.G.AUGGG.A.**G**..**A**.**G****A****G****U****A****A****C****G** | |
|  |  | NC\_004431.1/3630511-3630363  | **G****C****U****U****A****U****U****C****U****C**.A..GGG.C**G****G****G****G****C**GA...............A**A**UU**C****C****C****C**.ACC.G**G****C****G****G****U**AAAUCAACUCAGUUGA............................................................................................................A**A****G****C**.**C****C****G****C**GA.**G****C****G****C****U****U****U**GGGUGC...................................................................................................GAAC................................UC**A****A****A****G**.GAC**A****G****C**A-GA....**U**.**C****C****G****G****U**G.U.......A.......**A**..UU.........................**C****C****G****G****G****G****C**.C.GA**C****G****G**UUA..G.A.GU................................**C****C****G**.G.AUGGG.A.**G**..**A**.**G****A****G****U****A****A****C****G** | |
|  |  | NC\_004741.1/3166516-3166368  | **G****C****U****U****A****U****U****C****U****C**.A..GGG.C**G****G****G****G****C**GA...............A**A**UU**C****C****C****C**.ACC.G**G****C****G****G****U**AAAUCAACUCAGUUGA............................................................................................................A**A****G****C**.**C****C****G****C**GA.**G****C****G****C****U****U****U**GGGUGC...................................................................................................GAAC................................UC**A****A****A****G**.GAC**A****G****C**A-GA....**U**.**C****C****G****G****U**G.U.......A.......**A**..UU.........................**C****C****G****G****G****G****C**.C.GA**C****G****G**UUA..G.A.GU................................**C****C****G**.G.AUGGG.A.**G**..**A**.**G****A****G****U****A****A****C****G** | |
|  |  | NC\_003909.8/1576743-1576882  | **G****U****C****U****A****U****C****U****U****C**.G..GGG.C**A****G****G****G****U**GA...............A**A**AU**C****C****C****G**.ACC.G**G****C****G****G****U**GAUGAACUAUAUGUGAUUUUGUUCU...................................................................................................A**A****G****C**.**C****C****G****C**GA.**G****C****C****G**---.........................................................................................................-UUA..................................---**A**....**G****G****C**AGGA....**U**.**U****U****G****G****U**G.U.......G.......**A**..UU.........................**C****C****A****A****A****G****C**.C.GA**C****A****G**.UA..C.A.GU................................**C****U****G**.G.AUGGG.A.**G**..**A**.**A****G****A****U****G****G****A****G** | |
|  |  | NC\_005957.1/1447059-1447198  | **G****U****C****U****A****U****C****U****U****C**.G..GGG.C**A****G****G****G****U**GA...............A**A**AU**C****C****C****G**.ACC.G**G****C****G****G****U**GAUGAACUAUAUGUGAUUUUGUUCU...................................................................................................A**A****G****C**.**C****C****G****C**GA.**G****C****C****G**---.........................................................................................................-UUA..................................---**A**....**G****G****C**AGGA....**U**.**U****U****G****G****U**G.U.......G.......**A**..UU.........................**C****C****A****A****A****G****C**.C.GA**C****A****G**.UA..C.A.GU................................**C****U****G**.G.AUGGG.A.**G**..**A**.**A****G****A****U****G****G****A****G** | |
|  |  | NC\_006274.1/1457677-1457816  | **G****U****C****U****A****U****C****U****U****C**.G..GGG.C**A****G****G****G****U**GA...............A**A**AU**C****C****C****G**.ACC.G**G****C****G****G****U**GAUGAACUAUAUGUGAUUUUGUUCU...................................................................................................A**A****G****C**.**C****C****G****C**GA.**G****C****C****G**---.........................................................................................................-UUA..................................---**A**....**G****G****C**AGGA....**U**.**U****U****G****G****U**G.U.......G.......**A**..UU.........................**C****C****A****A****A****G****C**.C.GA**C****A****G**.UA..C.A.GU................................**C****U****G**.G.AUGGG.A.**G**..**A**.**A****G****A****U****G****G****A****G** | |
|  |  | NZ\_AAEK01000041.1/42669-42808  | **G****U****C****U****A****U****C****U****U****C**.G..GGG.C**A****G****G****G****U**GA...............A**A**AU**C****C****C****G**.ACC.G**G****C****G****G****U**GAUGAACUAUAUGUGAUGUUGUUCU...................................................................................................A**A****G****C**.**C****C****G****C**GA.**G****C****C****G**---.........................................................................................................-UUA..................................---**A**....**G****G****C**AGGA....**U**.**U****U****G****G****U**G.U.......G.......**A**..UU.........................**C****C****A****A****A****G****C**.C.GA**C****A****G**.UA..C.A.GU................................**C****U****G**.G.AUGGG.A.**G**..**A**.**A****G****A****U****G****G****A****G** | |
|  |  | NC\_002745.2/1828580-1828445  | **U****A****A****U****U****C****U****U****U****C**.G..GGG.C**A****G****G****G****U**GA...............A**A**UU**C****C****C****A**.ACC.G**G****C****A****G****U**AAAUA.......................................................................................................................A**A****G****C**.**C****U****G****C**GA.**C****C****U****G****C****U****A**AUAU.....................................................................................................GUUU.............................CAUAU**U****A****G****U**....-**G****G**CUGA....**U**.**C****U****A****G****U**G.A.......G.......**A**..UU.........................**C****U****A****G****A****G****C**.C.GA**C****A****G**UUA..A.A.GU................................**C****U****G**.G.AUGGG.A.**G**..**A**.**A****A****G****A****A****U****G****U** | |
|  |  | NC\_002758.2/1906373-1906238  | **U****A****A****U****U****C****U****U****U****C**.G..GGG.C**A****G****G****G****U**GA...............A**A**UU**C****C****C****A**.ACC.G**G****C****A****G****U**AAAUA.......................................................................................................................A**A****G****C**.**C****U****G****C**GA.**C****C****U****G****C****U****A**AUAU.....................................................................................................GUUU.............................CAUAU**U****A****G****U**....-**G****G**CUGA....**U**.**C****U****A****G****U**G.A.......G.......**A**..UU.........................**C****U****A****G****A****G****C**.C.GA**C****A****G**UUA..A.A.GU................................**C****U****G**.G.AUGGG.A.**G**..**A**.**A****A****G****A****A****U****G****U** | |
|  |  | NC\_002951.2/1876222-1876087  | **U****A****A****U****U****C****U****U****U****C**.G..GGG.C**A****G****G****G****U**GA...............A**A**UU**C****C****C****A**.ACC.G**G****C****A****G****U**AAAUA.......................................................................................................................A**A****G****C**.**C****U****G****C**GA.**C****C****U****G****C****U****A**AUAU.....................................................................................................GUUU.............................CAUAU**U****A****G****U**....-**G****G**CUGA....**U**.**C****U****A****G****U**G.A.......G.......**A**..UU.........................**C****U****A****G****A****G****C**.C.GA**C****A****G**UUA..A.A.GU................................**C****U****G**.G.AUGGG.A.**G**..**A**.**A****A****G****A****A****U****G****U** | |
|  |  | NC\_002953.3/1845723-1845588  | **U****A****A****U****U****C****U****U****U****C**.G..GGG.C**A****G****G****G****U**GA...............A**A**UU**C****C****C****A**.ACC.G**G****C****A****G****U**AAAUA.......................................................................................................................A**A****G****C**.**C****U****G****C**GA.**C****C****U****G****C****U****A**AUAU.....................................................................................................GUUU.............................CAUAU**U****A****G****U**....-**G****G**CUGA....**U**.**C****U****A****G****U**G.A.......G.......**A**..UU.........................**C****U****A****G****A****G****C**.C.GA**C****A****G**UUA..A.A.GU................................**C****U****G**.G.AUGGG.A.**G**..**A**.**A****A****G****A****A****U****G****U** | |
|  |  | NC\_003923.1/1866387-1866252  | **U****A****A****U****U****C****U****U****U****C**.G..GGG.C**A****G****G****G****U**GA...............A**A**UU**C****C****C****A**.ACC.G**G****C****A****G****U**AAAUA.......................................................................................................................A**A****G****C**.**C****U****G****C**GA.**C****C****U****G****C****U****A**AUAU.....................................................................................................GUUU.............................CAUAU**U****A****G****U**....-**G****G**CUGA....**U**.**C****U****A****G****U**G.A.......G.......**A**..UU.........................**C****U****A****G****A****G****C**.C.GA**C****A****G**UUA..A.A.GU................................**C****U****G**.G.AUGGG.A.**G**..**A**.**A****A****G****A****A****U****G****U** | |
|  |  | NC\_003454.1/10346-10461  | **U****A****A****A****G****U****C****U****U****C**.A..GGG.C**A****G****G****G****U**GA...............A**A**UU**C****C****C****G**.ACC.G**G****U****G****G****U**A-..........................................................................................................................C**A****G****U**.**C****C****A****C**GA.**A****A****G****C**---.........................................................................................................AUUU..................................---**G**....**C****U****U**U-GA....**U**.**U****U****G****G****U**G.A.......A.......**A**..UU.........................**C****C****A****A****A****A****C**.C.GA**C****A****G**.UA..G.A.GU................................**C****U****G**.G.AUGGG.A.**G**..**A**.**A****G****A****A****U****U****A****G** | |
|  |  | NC\_003047.1/2398173-2398337  | **A****A****G****C****G****U****U****C****U****C**.A..GGG.C**G****G****G****G****U**GA...............A**A**UU**C****C****C****C**.ACC.G**G****C****G****G****U**AGCGAACACAGGAUCGGGAAUGGUCGGUGGUCG...........................................................................................G**A****G****C**.**C****C****G****C**GA.**G****C****G****C****U****U****C**GC.......................................................................................................AUUU.............................CGUGC**G****A****A****G**.GUC**A****G****C**A-GA....**U**.**C****C****G****G****U**C.G.......A.......**A**..UU.........................**C****C****G****G****A****G****C**.C.GA**C****G****G**UUA..U.A.GU................................**C****C****G**.G.AUGGA.A.**G**..**A**.**G****A****G****C****A****A****G****C** | |
|  |  | NC\_002663.1/1582210-1582055  | **G****C****G****C****A****U****U****C****U****C**.A..GGG.C**A****G****G****G****U**GA...............A**A**UU**C****C****C****U**.ACC.G**G****U****G****G****U**AAAUGUAUAACAU...............................................................................................................A**A****G****C**.**C****C****A****C**GA.**G****C****G****U****U****U****A**CUAUU....................................................................................................UCAU....................GUCACAAGAGAUAG**U****A****A****A**.GUC**A****G****C**A-GA....**U**.**U****U****G****G****U**G.A.......G.......**A**..AU.........................**C****C****A****A****A****G****C**.C.GA**C****A****G**.UA..U.A.GU................................**C****U****G**.G.AUGAA.A.**G**..**A**.**G****A****A****U****A****A****G****C** | |
|  |  | NC\_002927.3/5289498-5289651  | **G****U****A****C****G****U****C****U****U****C**.A..GGG.C**G****G****G****G****U**GC...............A**A**UU**C****C****C****C**.ACC.G**G****C****G****G****U**AUGCCACGCAAUGUGGC...........................................................................................................G**A****G****C**.**C****C****G****C**GA.**G****C****G****C****C****C****U**GCGUUG...................................................................................................GCAA............................CAGCGC**A****G****G****G**.GUC**A****G****C**A-GA....**C**.**C****U****G****G****U**G.A.......G.......**A**..UG.........................**C****C****A****G****G****G****C**.C.GA**C****G****G**UCA..U.A.GU................................**C****C****G**.G.AUGAG.A.**G**..**A**.**A****G****A****U****G****U****G****C** | |
|  |  | NC\_002928.3/4727823-4727976  | **G****U****A****C****G****U****C****U****U****C**.A..GGG.C**G****G****G****G****U**GC...............A**A**UU**C****C****C****C**.ACC.G**G****C****G****G****U**AUGCCACGCAAUGUGGC...........................................................................................................G**A****G****C**.**C****C****G****C**GA.**G****C****G****C****C****C****U**GCGUUG...................................................................................................GCAA............................CAGCGC**A****G****G****G**.GUC**A****G****C**A-GA....**C**.**C****U****G****G****U**G.A.......G.......**A**..UG.........................**C****C****A****G****G****G****C**.C.GA**C****G****G**UCA..U.A.GU................................**C****C****G**.G.AUGAG.A.**G**..**A**.**A****G****A****U****G****U****G****C** | |
|  |  | NC\_002929.2/481881-482034  | **G****U****A****C****G****U****C****U****U****C**.A..GGG.C**G****G****G****G****U**GC...............A**A**UU**C****C****C****C**.ACC.G**G****C****G****G****U**AUGCCACGCAAUGUGGC...........................................................................................................G**A****G****C**.**C****C****G****C**GA.**G****C****G****C****C****C****U**GCGUUG...................................................................................................GCAA............................CAGCGC**A****G****G****G**.GUC**A****G****C**A-GA....**C**.**C****U****G****G****U**G.A.......G.......**A**..UG.........................**C****C****A****G****G****G****C**.C.GA**C****G****G**UCA..U.A.GU................................**C****C****G**.G.AUGAG.A.**G**..**A**.**A****G****A****U****G****U****G****C** | |
|  |  | NC\_003450.3/66442-66279  | **U****U****U****U****G****U****U****C****U****C**.A..GGG.C**G****G****G****G****C**GA...............A**A**UU**C****C****C****C**.ACC.G**G****C****G****G****U**AAGACGAAUCAUCAGAUAUGACUAGUU.................................................................................................G**A****G****C**.**C****C****G****C**GA.**G****C****G****C****C****A****C**CAACUA...................................................................................................GCAA............................UGGUUU**G****U****G****G**.GUC**A****G****C**A-GA....**U**.**C****C****G****G****U**U.A.......A.......**A**..UU.........................**C****C****G****G****A****G****C**.C.GA**C****G****G**UCA..U.A.GU................................**C****C****G**.G.AUGCA.A.**G**..**A**.**G****A****A****C****C****G****U****G** | |
|  |  | NC\_006958.1/66442-66279  | **U****U****U****U****G****U****U****C****U****C**.A..GGG.C**G****G****G****G****C**GA...............A**A**UU**C****C****C****C**.ACC.G**G****C****G****G****U**AAGACGAAUCAUCAGAUAUGACUAGUU.................................................................................................G**A****G****C**.**C****C****G****C**GA.**G****C****G****C****C****A****C**CAACUA...................................................................................................GCAA............................UGGUUU**G****U****G****G**.GUC**A****G****C**A-GA....**U**.**C****C****G****G****U**U.A.......A.......**A**..UU.........................**C****C****G****G****A****G****C**.C.GA**C****G****G**UCA..U.A.GU................................**C****C****G**.G.AUGCA.A.**G**..**A**.**G****A****A****C****C****G****U****G** | |
|  |  | NC\_006510.1/2329952-2329820  | **U****G****C****A****U****C****C****U****U****C**.G..GGG.C**A****G****G****G****U**GA...............A**A**UU**C****C****C****G**.ACC.G**G****C****G****G****U**GAUGGGGCUUUCCGCCCCU.........................................................................................................C**A****G****C**.**C****C****G****C**GA.**G****C****C**----.........................................................................................................GAAA..................................----....**G****G****C**AGGA....**U**.**C****C****G****G****U**G.C.......G.......**A**..UU.........................**C****C****G****G****A****G****C**.C.GA**C****A****G**.UA..U.A.GU................................**C****U****G**.G.AUGGG.A.**G**..**A**.**A****G****G****A****U****G****C****C** | |
|  |  | NC\_004116.1/1411878-1411733  | **A****G****G****U****G****U****C****U****U****C**.A..GGG.C**A****G****G****G****U**GU...............A**A**UU**C****C****C****G**.ACC.G**G****C****G****G****U**AAUCGCUUCCAACUUAUUUGUCAGUUGUGAAGCAU.........................................................................................C**A****G****U**.**C****C****G****C**GA.**G****C**-----.........................................................................................................GAAA..................................----....-**G****C**U-GA....**U**.**G****U****G****G****U**G.A.......G.......**A**..UU.........................**C****C****A****C****A****A****C**.C.GA**C****A****G**.UA..A.A.GU................................**C****U****G**.G.AUGGG.A.**G**..**A**.**A****G****A****C****A****A****A****G** | |
|  |  | NC\_004368.1/1523027-1522882  | **A****G****G****U****G****U****C****U****U****C**.A..GGG.C**A****G****G****G****U**GU...............A**A**UU**C****C****C****G**.ACC.G**G****C****G****G****U**AAUCGCUUCCAACUUAUUUGUCAGUUGUGAAGCAU.........................................................................................C**A****G****U**.**C****C****G****C**GA.**G****C**-----.........................................................................................................GAAA..................................----....-**G****C**U-GA....**U**.**G****U****G****G****U**G.A.......G.......**A**..UU.........................**C****C****A****C****A****A****C**.C.GA**C****A****G**.UA..A.A.GU................................**C****U****G**.G.AUGGG.A.**G**..**A**.**A****G****A****C****A****A****A****G** | |
|  |  | NC\_003318.1/623747-623584  | **G****C****U****U****G****U****U****C****U****C**.G..GGG.C**G****G****G****G****U**GA...............A**A**CU**C****C****C****C**.ACC.G**G****C****G****G****U**AUGAAAAGCAAUUUUC............................................................................................................A**A****G****C**.**C****C****G****C**GA.**G****C****G****C****C****U****G**AAAUGGAAGCCGAUUCGCAUG....................................................................................CCAU................................UU**C****A****G****G**.GUC**A****G****C**A-GA....**U**.**C****C****G****G****U**G.A.......G.......**A**..UG.........................**C****C****G****G****A****G****C**.C.GA**C****G****G**UUA..A.A.GU................................**C****C****G**.G.AUGGA.A.**G**..**A**.**G****A****G****C****G****A****A****U** | |
|  |  | NC\_004311.2/674086-674249  | **G****C****U****U****G****U****U****C****U****C**.G..GGG.C**G****G****G****G****U**GA...............A**A**CU**C****C****C****C**.ACC.G**G****C****G****G****U**AUGAAAAGCAAUUUUC............................................................................................................A**A****G****C**.**C****C****G****C**GA.**G****C****G****C****C****U****G**AAAUGGAAGCCGAUUCGCAUG....................................................................................CCAU................................UU**C****A****G****G**.GUC**A****G****C**A-GA....**U**.**C****C****G****G****U**G.A.......G.......**A**..UG.........................**C****C****G****G****A****G****C**.C.GA**C****G****G**UUA..A.A.GU................................**C****C****G**.G.AUGGA.A.**G**..**A**.**G****A****G****C****G****A****A****U** | |
|  |  | NC\_006933.1/543091-542928  | **G****C****U****U****G****U****U****C****U****C**.G..GGG.C**G****G****G****G****U**GA...............A**A**CU**C****C****C****C**.ACC.G**G****C****G****G****U**AUGAAAAGCAAUUUUC............................................................................................................A**A****G****C**.**C****C****G****C**GA.**G****C****G****C****C****U****G**AAAUGGAAGCCGAUUCGCAUG....................................................................................CCAU................................UU**C****A****G****G**.GUC**A****G****C**A-GA....**U**.**C****C****G****G****U**G.A.......G.......**A**..UG.........................**C****C****G****G****A****G****C**.C.GA**C****G****G**UUA..A.A.GU................................**C****C****G**.G.AUGGA.A.**G**..**A**.**G****A****G****C****G****A****A****U** | |
|  |  | NC\_002976.3/1099570-1099435  | **A****U****U****C****A****U****C****U****U****C**.G..GGG.U**A****G****G****G****U**GU...............A**A**UU**C****C****C****A**.ACC.G**G****C****A****G****U**AAUCU.......................................................................................................................A**A****G****C**.**C****U****G****C**GAC**G****A****C****A****A****C****G**UA.......................................................................................................UUAA............................UUUUUG**C****U****U****U**....**G****U****C**UUGA....**U**.**U****C****A****G****U**G.A.......G.......**A**..GU.........................**C****U****G****A****A****G****C**.C.GA**C****A****G**.UA..U.A.GU................................**C****U****G**.G.AUGGG.A.**G**..**A**.**A****G****A****U****G****G****A****G** | |
|  |  | NC\_004461.1/1211514-1211379  | **A****U****U****C****A****U****C****U****U****C**.G..GGG.U**A****G****G****G****U**GU...............A**A**UU**C****C****C****A**.ACC.G**G****C****A****G****U**AAUCU.......................................................................................................................A**A****G****C**.**C****U****G****C**GAC**G****A****C****A****A****C****G**UA.......................................................................................................UUAA............................UUUUUG**C****U****U****U**....**G****U****C**UUGA....**U**.**U****C****A****G****U**G.A.......G.......**A**..GU.........................**C****U****G****A****A****G****C**.C.GA**C****A****G**.UA..U.A.GU................................**C****U****G**.G.AUGGG.A.**G**..**A**.**A****G****A****U****G****G****A****G** | |
|  |  | NC\_004193.1/2380854-2380731  | **U****C****C****A****A****U****C****U****U****C**.G..GGG.C**A****G****G****G****U**GC...............A**A**UU**C****C****C****G**.ACC.G**G****C****G****G****U**A-..........................................................................................................................A**A****G****U**.**C****C****G****C**GA.**G****C****U****G****C****A****U**A........................................................................................................-CAU.................................A**U****U****G****U**....**G****G****U**U-GA....**U**.**C****U****G****G****U**U.A.......A.......**A**..UU.........................**C****C****A****G****U****A****C**.C.GA**C****A****G**UUA..U.A.GU................................**C****U****G**.G.AUGGG.A.**G**..**A**.**A****G****A****U****G****U****C****G** | |
|  |  | NZ\_AAAH01000998.2/2872-2740  | **G****A****A****G****A****C****C****U****U****C**.G..GGG.C**A****A****G****G****U**GA...............A**A**UU**C****C****U****G**.AUC.G**G****C****G****G****U**AAUGGUGGCGUGCCCACAC.........................................................................................................A**A****G****C**.**C****C****G****C**GA.**G****C****C**----.........................................................................................................GUAA..................................----....**G****G****C**AGGA....**C**.**C****C****G****G****U**G.U.......G.......**A**..UU.........................**C****C****G****G****G****G****C**.C.GA**C****G****G**.UA..U.A.GU................................**C****C****G**.G.AUGGG.A.**G**..**A**.**A****G****G****U****C****G****G****C** | |
|  |  | NC\_002952.2/1943822-1943687  | **U****A****A****U****U****C****U****U****U****C**.G..GGG.C**A****G****G****G****U**GA...............A**A**UU**C****C****C****A**.ACC.G**G****C****A****G****U**AAAUU.......................................................................................................................A**A****G****C**.**C****U****G****C**GA.**C****C****U****G****C****U****A**AUAU.....................................................................................................GUUU.............................CGUAU**U****A****G****U**....-**G****G**CUGA....**U**.**C****U****A****G****U**G.A.......G.......**A**..CU.........................**C****U****A****G****A****G****C**.C.GA**C****A****G**UUA..A.A.GU................................**C****U****G**.G.AUGGG.A.**G**..**A**.**A****A****G****A****A****U****G****U** | |
|  |  | NZ\_AAAI03000003.1/244059-244220  | **U****U****A****C****G****U****C****U****U****C**.A..GGG.C**G****G****G****G****U**GC...............A**A**UU**C****C****C****C**.ACC.G**G****C****G****G****U**AUGUGGCCGGCGCCAAUGGCGCAUUGUCAC..............................................................................................G**A****G****C**.**C****C****G****C**GA.**G****C****G****C****C****C****A**CG.......................................................................................................CUCU.............................CCGCG**U****G****G****G**.GUC**A****G****C**A-GA....**U**.**C****U****G****G****U**G.A.......G.......**A**..GG.........................**C****C****A****G****G****G****C**.C.GA**C****G****G**UUA..A.A.GU................................**C****C****G**.G.AUGAA.A.**G**..**A**.**A****G****A****U****G****G****G****C** | |
|  |  | NC\_004193.1/3348439-3348301  | **U****G****U****A****U****U****C****U****U****C**.G..GGG.C**A****G****G****G****U**GU...............A**A**UU**C****C****C****G**.ACC.G**G****C****G****G****U**GACAAGUCAUCAGAACUUG.........................................................................................................A**A****G****U**.**C****C****G****U**GAC**C****C****G****C****U**--.........................................................................................................UUUA..................................--**U****G**....**C****G****G**UGGA....**U**.**C****U****A****G****U**G.A.......G.......**A**..AU.........................**C****U****A****G****A****G****C**.C.GA**C****A****G**UAA..U.A.GU................................**C****U****G**.G.AUGGG.A.**G**..**A**.**A****G****A****A****U****A****G****C** | |
|  |  | NC\_003197.1/3358808-3358610  | **G****C****U****U****A****U****U****C****U****C**.A..GGG.C**G****G****G****G****C**GA...............A**A**UU**C****C****C****C**.ACC.G**G****C****G****G****U**AAAUCAGCGAUGUUUAACGCAUUUCUCAUCACGGCGCGCCGUGACGGAACGCGGAGAGCAGGUUGA..........................................................A**A****G****C**.**C****C****G****C**GA.**G****C****G****C****U****U****U**UUG......................................................................................................CCAU.............................AGCGA**A****A****A****G**.GUC**A****G****C**A-GA....**U**.**C****C****G****G****U**G.U.......A.......**A**..UU.........................**C****C****G****G****G****G****C**.C.GA**C****G****G**UUA..A.A.GU................................**C****C****G**.G.AUGGG.A.**G**..**A**.**G****G****G****U****A****A****C****G** | |
|  |  | NC\_003198.1/3216311-3216113  | **G****C****U****U****A****U****U****C****U****C**.A..GGG.C**G****G****G****G****C**GA...............A**A**UU**C****C****C****C**.ACC.G**G****C****G****G****U**AAAUCAGCGAUGUUUAACGCAUUUCUCAUCACGGUGCGCCGUGACGGAACGCGGAGAGCAGGUUGA..........................................................A**A****G****C**.**C****C****G****C**GA.**G****C****G****C****U****U****U**UUG......................................................................................................CCAU.............................AGCGA**A****A****A****G**.GUC**A****G****C**A-GA....**U**.**C****C****G****G****U**G.U.......A.......**A**..UU.........................**C****C****G****G****G****G****C**.C.GA**C****G****G**UUA..A.A.GU................................**C****C****G**.G.AUGGG.A.**G**..**A**.**G****G****G****U****A****A****C****G** | |
|  |  | NC\_004631.1/3201806-3201608  | **G****C****U****U****A****U****U****C****U****C**.A..GGG.C**G****G****G****G****C**GA...............A**A**UU**C****C****C****C**.ACC.G**G****C****G****G****U**AAAUCAGCGAUGUUUAACGCAUUUCUCAUCACGGUGCGCCGUGACGGAACGCGGAGAGCAGGUUGA..........................................................A**A****G****C**.**C****C****G****C**GA.**G****C****G****C****U****U****U**UUG......................................................................................................CCAU.............................AGCGA**A****A****A****G**.GUC**A****G****C**A-GA....**U**.**C****C****G****G****U**G.U.......A.......**A**..UU.........................**C****C****G****G****G****G****C**.C.GA**C****G****G**UUA..A.A.GU................................**C****C****G**.G.AUGGG.A.**G**..**A**.**G****G****G****U****A****A****C****G** | |
|  |  | NC\_006511.1/3169269-3169071  | **G****C****U****U****A****U****U****C****U****C**.A..GGG.C**G****G****G****G****C**GA...............A**A**UU**C****C****C****C**.ACC.G**G****C****G****G****U**AAAUCAGCGAUGUUUAACGCAUUUCUCAUCACGGUGCGCCGUGACGGAACGCGGAGAGCAGGUUGA..........................................................A**A****G****C**.**C****C****G****C**GA.**G****C****G****C****U****U****U**UUG......................................................................................................CCAU.............................AGCGA**A****A****A****G**.GUC**A****G****C**A-GA....**U**.**C****C****G****G****U**G.U.......A.......**A**..UU.........................**C****C****G****G****G****G****C**.C.GA**C****G****G**UUA..A.A.GU................................**C****C****G**.G.AUGGG.A.**G**..**A**.**G****G****G****U****A****A****C****G** | |
|  |  | NC\_006905.1/3320886-3320688  | **G****C****U****U****A****U****U****C****U****C**.A..GGG.C**G****G****G****G****C**GA...............A**A**UU**C****C****C****C**.ACC.G**G****C****G****G****U**AAAUCGGCGAUGUUUAACGCAUUUCUCAUCACGGCGCGCCGUGACGGAACGCGGAGAGCAGGUUGA..........................................................A**A****G****C**.**C****C****G****C**GA.**G****C****G****C****U****U****U**UUG......................................................................................................CCAU.............................AGCGA**A****A****A****G**.GUC**A****G****C**A-GA....**U**.**C****C****G****G****U**G.U.......A.......**A**..UU.........................**C****C****G****G****G****G****C**.C.GA**C****G****G**UUA..A.A.GU................................**C****C****G**.G.AUGGG.A.**G**..**A**.**G****G****G****U****A****A****C****G** | |
|  |  | NC\_003454.1/2491-2606  | **A****A****U****A****A****U****C****U****U****C**.G..GGG.C**A****G****G****G****U**GA...............A**A**UU**C****C****C****G**.ACC.G**G****U****G****G****U**A-..........................................................................................................................U**A****G****U**.**C****C****A****C**GA.**A****A****G****U**---.........................................................................................................AUUU..................................---**G**....**C****U****U**U-GA....**U**.**U****U****G****G****U**G.A.......A.......**A**..UU.........................**C****C****A****A****A****A****C**.C.GA**C****A****G**.UA..G.A.GU................................**C****U****G**.G.AUGAG.A.**G**..**A**.**A****G****A****A****A****A****G****A** | |
|  |  | NC\_002973.5/2008026-2007904  | **G****U****U****C****A****U****C****U****U****C**.G..GGG.C**A****G****G****G****U**GC...............A**A**UU**C****C****C****G**.ACC.G**G****U****G****G****U**UA..........................................................................................................................A**A****G****U**.**C****C****A****C**GAU**C****U****G****C****U**--.........................................................................................................-UUU..................................**U****U****A****G**....**C****A****G**UUGA....**C**U**C****U****G****G****U**G.U.......A.......**A**..UU.........................**C****C****A****G****G****A****C**.C.GA**C****A****G**.UA..U.A.GU................................**C****U****G**.G.AUGGG.A.**G**..**A**.**A****G****A****U****G****U****U****G** | |
|  |  | NC\_003210.1/2020609-2020487  | **G****U****U****C****A****U****C****U****U****C**.G..GGG.C**A****G****G****G****U**GC...............A**A**UU**C****C****C****G**.ACC.G**G****U****G****G****U**UA..........................................................................................................................A**A****G****U**.**C****C****A****C**GAU**C****U****G****C****U**--.........................................................................................................-UUU..................................**U****U****A****G**....**C****A****G**UUGA....**C**U**C****U****G****G****U**G.U.......A.......**A**..UU.........................**C****C****A****G****G****A****C**.C.GA**C****A****G**.UA..U.A.GU................................**C****U****G**.G.AUGGG.A.**G**..**A**.**A****G****A****U****G****U****U****G** | |
|  |  | NZ\_AADQ01000046.1/7762-7640  | **G****U****U****C****A****U****C****U****U****C**.G..GGG.C**A****G****G****G****U**GC...............A**A**UU**C****C****C****G**.ACC.G**G****U****G****G****U**UA..........................................................................................................................A**A****G****U**.**C****C****A****C**GAU**C****U****G****C****U**--.........................................................................................................-UUU..................................**U****U****A****G**....**C****A****G**UUGA....**C**U**C****U****G****G****U**G.U.......A.......**A**..UU.........................**C****C****A****G****G****A****C**.C.GA**C****A****G**.UA..U.A.GU................................**C****U****G**.G.AUGGG.A.**G**..**A**.**A****G****A****U****G****U****U****G** | |
|  |  | NZ\_AADR01000031.1/13879-13757  | **G****U****U****C****A****U****C****U****U****C**.G..GGG.C**A****G****G****G****U**GC...............A**A**UU**C****C****C****G**.ACC.G**G****U****G****G****U**UA..........................................................................................................................A**A****G****U**.**C****C****A****C**GAU**C****U****G****C****U**--.........................................................................................................-UUU..................................**U****U****A****G**....**C****A****G**UUGA....**C**U**C****U****G****G****U**G.U.......A.......**A**..UU.........................**C****C****A****G****G****A****C**.C.GA**C****A****G**.UA..U.A.GU................................**C****U****G**.G.AUGGG.A.**G**..**A**.**A****G****A****U****G****U****U****G** | |
|  |  | NZ\_AABJ03000004.1/85924-86055  | **U****A****A****A****U****U****C****U****U****C**.G..GGG.C**A****G****G****G****U**GU...............G**A**UU**C****C****C****A**.ACC.G**A****C****G****G****U**AACAACAUUUUGUUG.............................................................................................................A**A****G****U**.**C****C****G****U**GAC**C****C****G****C**---.........................................................................................................GCAA..................................---**G**....**C****G****G**UUGA....**C**.**C****C****A****G****U**G.A.......A.......**A**..CU.........................**C****U****G****G****G****A****C**.C.GA**C****A****G**.UA..U.A.GU................................**C****U****G**.G.AUGGG.A.**G**..**A**.**A****G****A****A****A****U****A****G** | |
|  |  | NZ\_AAGR01000090.1/4475-4593  | **G****U****U****C****G****U****C****U****U****C**.A..GGG.C**A****G****G****G****U**GC...............A**A**UU**C****C****C****G**.ACC.G**G****C****G****G****U**A-..........................................................................................................................U**A****G****U**.**C****C****G****C**GAC**C****C****G****C**---.........................................................................................................UUAU..................................---**G**....**C****G****G**UGGA....**A**.**C****C****G****G****U**G.U.......G.......**A**..CU.........................**C****C****G****G****U****A****C**.C.GA**U****A****G**UGA..U.A.GU................................**C****U****A**.G.AUGGA.A.**G**..**A**.**A****G****A****U****G****G****G****G** | |
|  |  | NZ\_AAAU03000007.1/63133-63289  | **C****G****A****C****G****U****U****C****U****C**.A..GGG.C**G****G****G****G****U**GG...............A**A**UU**C****C****C****C**.ACC.G**G****C****G****G****U**GAUGACGCCCAGGCGUC...........................................................................................................C**A****G****C**.**C****C****G****C**GA.**G****C****G****C****U****U****G**CCACGGG..................................................................................................GCAA..........................CCCCCGCG**C****A****A****G**.GUC**A****G****C**A-GA....**U**.**C****C****G****G****U**G.C.......G.......**A**..UU.........................**C****C****G****G****A****G****C**.C.GA**C****G****G**UCA..G.A.GU................................**C****C****G**.G.AUGAG.A.**G**..**A**.**G****A****G****C****G****G****G****A** | |
|  |  | NC\_005085.1/4551002-4551150  | **U****U****A****C****G****U****C****U****U****C**.G..GGG.C**G****G****G****G****U**GC...............A**A**CU**C****C****C****C**.ACC.G**G****C****G****G****U**AUGGCGCGCAAGCGCC............................................................................................................G**A****G****C**.**C****C****G****C**GA.**G****C****G****C****C****U****U**U........................................................................................................GCCU...........................GACGGCA**A****A****G****G**.GUC**A****G****C**A-GA....**U**.**C****U****G****G****U**G.A.......A.......**A**..CG.........................**C****C****A****G****A****G****C**.C.GA**C****G****G**UAA..U.A.GU................................**C****C****G**.G.AUGAG.A.**G**..**A**.**A****G****A****C****G****A****C****G** | |
|  |  | NZ\_AADW02000042.1/4241-4101  | **U****U****C****C****A****U****C****U****U****C**.A..GGG.C**A****G****G****G****U**GA...............A**A**UU**C****C****C****G**.ACC.G**G****C****G****G****U**AAACAGGCGAUUCGCCUG..........................................................................................................A**A****G****C**.**C****C****G****C**GA.**G****C****G****A****A****U****A**G........................................................................................................GGCG.................................C**U****A****U****U**....**U****G****C**U-GA....**U**.**U****C****U****G****U**G.U.......A.......**A**..CU.........................**C****A****G****G****A****G****C**.C.GA**C****A****G**.UA..U.A.GU................................**C****U****G**.G.AUGGG.A.**G**..**A**.**A****G****A****U****G****G****C****G** | |
|  |  | NC\_003212.1/2074996-2074874  | **G****U****U****C****A****U****C****U****U****C**.G..GGG.C**A****G****G****G****U**GC...............A**A**UU**C****C****C****G**.ACC.G**G****U****G****G****U**UA..........................................................................................................................A**A****G****U**.**C****C****A****C**GAU**C****U****G****C****U**--.........................................................................................................-UUC..................................**U****U****A****G**....**C****A****G**UUGA....**C**U**C****U****G****G****U**G.U.......A.......**A**..UU.........................**C****C****A****G****G****A****C**.C.GA**C****A****G**.UA..U.A.GU................................**C****U****G**.G.AUGGG.A.**G**..**A**.**A****G****A****U****G****U****U****G** | |
|  |  | NC\_003902.1/837066-837234  | **G****A****A****C****G****U****C****U****U****C**.A..GGG.C**G****G****G****G****U**GC...............G**A**UU**C****C****C****C**.ACC.G**G****C****G****G****U**AUGCAUGCGCAAGCGUGU..........................................................................................................G**A****G****C**.**C****C****G****C**GA.**G****C****G****C****U****U****C**GCUGCCGAU................................................................................................CUCU................CGCUGAGAGCACGGCGGU**G****A****A****G**.GUC**A****G****C**A-GA....**U**.**C****C****G****G****U**G.C.......G.......**A**..UG.........................**C****C****G****G****G****G****C**.C.GA**C****G****G**.UA..U.A.GU................................**C****C****G**.G.AUGAA.A.**G**..**A**.**A****G****A****C****G****G****C****C** | |
|  |  | NC\_007086.1/4203454-4203286  | **G****A****A****C****G****U****C****U****U****C**.A..GGG.C**G****G****G****G****U**GC...............G**A**UU**C****C****C****C**.ACC.G**G****C****G****G****U**AUGCAUGCGCAAGCGUGU..........................................................................................................G**A****G****C**.**C****C****G****C**GA.**G****C****G****C****U****U****C**GCUGCCGAU................................................................................................CUCU................CGCUGAGAGCACGGCGGU**G****A****A****G**.GUC**A****G****C**A-GA....**U**.**C****C****G****G****U**G.C.......G.......**A**..UG.........................**C****C****G****G****G****G****C**.C.GA**C****G****G**.UA..U.A.GU................................**C****C****G**.G.AUGAA.A.**G**..**A**.**A****G****A****C****G****G****C****C** | |
|  |  | NZ\_AADY01000001.1/76356-76519  | **U****U****A****C****G****U****C****U****U****C**.A..GGG.C**G****G****G****G****U**GA...............A**A**CU**C****C****C****C**.ACC.G**G****C****G****G****U**AUGCCGCAGCGUGCGUUUCCGUACGAUGCAGC............................................................................................G**A****G****C**.**C****C****G****C**GA.**G****C****G****C****C****U****G**C........................................................................................................ACAU............................GCCGUG**C****A****G****G**.GUC**A****G****C**A-GA....**C**.**C****U****G****G****U**G.C.......G.......**A**..UG.........................**C****C****A****G****G****G****C**.C.GA**C****G****G**UCA..U.A.GU................................**C****C****G**.G.AUGAA.A.**G**..**A**.**A****G****A****U****G****G****G****C** | |
|  |  | NC\_004547.2/1001970-1002170  | **G****C****U****U****A****U****U****C****U****C**.A..GGG.C**G****G****G****G****U**GU...............A**A**UU**C****C****C****C**.ACC.G**G****C****G****G****U**AAACCAGUGAACGUAUGAACGAAUAAGGUUCUGCGCACUGG...................................................................................A**A****G****C**.**C****C****G****C**GA.**G****C****G****C****U****C****A**GGCUGUGUUUUCCGAUAUCAAUUGUCGGUUUUC........................................................................GCAA................................CA**U****G****A****G**.GUC**A****G****C**A-GA....**C**.**C****C****G****G****U**G.U.......A.......**A**..UU.........................**C****C****G****G****G****G****C**.C.GA**C****G****G**UUA..A.A.GU................................**C****C****G**.G.AUGGG.A.**G**..**A**.**G****A****G****U****A****A****C****G** | |
|  |  | NC\_005966.1/2847072-2847219  | **A****C****A****U****C****G****C****U****U****C**.A..GGG.C**G****G****G****G****C**GU...............A**A**UU**C****C****C****C**.ACC.G**G****C****G****G****U**AAGCUCAACACGAGC.............................................................................................................A**A****G****C**.**C****C****G****C**GA.**G****C****A****A****U****U****G**GA.......................................................................................................CCAU........................UUAAUGGCGU**C****A****A****U**....**C****G****C**A-GA....**U**.**C****U****G****G****U**G.U.......A.......**A**..AU.........................**C****C****A****G****A****G****C**.C.GA**C****G****G**.UA..U.A.GU................................**C****C****G**.G.AUGAA.A.**G**..**A**.**A****G****A****C****G****A****C****G** | |
|  |  | NC\_002947.3/616521-616380  | **G****U****C****G****G****U****C****U****U****C**.A..GGG.C**G****G****G****G****U**GU...............A**A**GU**C****C****C****C**.ACC.G**G****C****G****G****U**AAAUCGAAAGAU................................................................................................................G**A****G****C**.**C****C****G****C**GA.**G****C****G****C****C****C****C**GA.......................................................................................................CCAU..............................GUCG**G****G****G****G**..UC**A****G****C**A-GA....**U**.**C****U****G****G****U**G.C.......A.......**A**..CU.........................**C****C****A****G****A****G****C**.C.GA**C****G****G**UCA..U.A.GU................................**C****C****G**.G.AUGAA.A.**G**..**A**.**A****G****G****C****G****U****C****A** | |
|  |  | NC\_002737.1/319186-319309  | **A****A****G****U****G****U****C****U****U****C**.A..GGG.C**A****G****G****G****U**GU...............G**A**UU**C****C****C****G**.ACC.G**G****C****G****G****U**GACUAGUAACUAG...............................................................................................................A**A****G****U**.**C****C****G****C**GA.**G****C**-----.........................................................................................................GCAA..................................----....-**G****C**U-GA....**U**.**G****U****G****G****U**G.U.......A.......**A**..CU.........................**C****C****A****C****A****A****C**.C.GA**C****A****G**.UA..U.A.GU................................**C****U****G**.G.AUGAG.A.**G**..**A**.**A****G****A****C****C****G****G****G** | |
|  |  | NC\_003485.1/351348-351471  | **A****A****G****U****G****U****C****U****U****C**.A..GGG.C**A****G****G****G****U**GU...............G**A**UU**C****C****C****G**.ACC.G**G****C****G****G****U**GACUAGUAACUAG...............................................................................................................A**A****G****U**.**C****C****G****C**GA.**G****C**-----.........................................................................................................GCAA..................................----....-**G****C**U-GA....**U**.**G****U****G****G****U**G.U.......A.......**A**..CU.........................**C****C****A****C****A****A****C**.C.GA**C****A****G**.UA..U.A.GU................................**C****U****G**.G.AUGAG.A.**G**..**A**.**A****G****A****C****C****G****G****G** | |
|  |  | NC\_006086.1/349226-349349  | **A****A****G****U****G****U****C****U****U****C**.A..GGG.C**A****G****G****G****U**GU...............G**A**UU**C****C****C****G**.ACC.G**G****C****G****G****U**GACUAGUAACUAG...............................................................................................................A**A****G****U**.**C****C****G****C**GA.**G****C**-----.........................................................................................................GCAA..................................----....-**G****C**U-GA....**U**.**G****U****G****G****U**G.U.......A.......**A**..CU.........................**C****C****A****C****A****A****C**.C.GA**C****A****G**.UA..U.A.GU................................**C****U****G**.G.AUGAG.A.**G**..**A**.**A****G****A****C****C****G****G****G** | |
|  |  | NC\_004070.1/309667-309791  | **A****A****G****U****G****U****C****U****U****C**.A..GGG.C**A****G****G****G****U**GU...............G**A**UU**C****C****C****G**.ACC.G**G****C****G****G****U**GACUAGGUAACUAG..............................................................................................................A**A****G****U**.**C****C****G****C**GA.**G****C**-----.........................................................................................................GCAA..................................----....-**G****C**U-GA....**U**.**G****U****G****G****U**G.U.......A.......**A**..CU.........................**C****C****A****C****A****A****C**.C.GA**C****A****G**.UA..U.A.GU................................**C****U****G**.G.AUGAG.A.**G**..**A**.**A****G****A****C****C****G****G****G** | |
|  |  | NC\_004606.1/1587078-1586954  | **A****A****G****U****G****U****C****U****U****C**.A..GGG.C**A****G****G****G****U**GU...............G**A**UU**C****C****C****G**.ACC.G**G****C****G****G****U**GACUAGGUAACUAG..............................................................................................................A**A****G****U**.**C****C****G****C**GA.**G****C**-----.........................................................................................................GCAA..................................----....-**G****C**U-GA....**U**.**G****U****G****G****U**G.U.......A.......**A**..CU.........................**C****C****A****C****A****A****C**.C.GA**C****A****G**.UA..U.A.GU................................**C****U****G**.G.AUGAG.A.**G**..**A**.**A****G****A****C****C****G****G****G** | |
|  |  | NC\_007005.1/4239088-4239253  | **C****A****A****C****G****U****U****C****U****C**.A..GGG.C**G****G****G****G****U**GC...............A**A**UU**C****C****C****C**.ACC.G**G****C****G****G****U**AAUGACGCGCAAUGCGUC..........................................................................................................U**A****G****C**.**C****C****G****C**GA.**G****C****G****C****U****U****G**GGGGUCGUGGCUUUGGCUG......................................................................................CUAU...............................CUG**C****A****A****G**.GUC**A****G****C**A-GA....**C**.**C****C****G****G****U**G.U.......G.......**A**..UU.........................**C****C****G****G****G****G****C**.C.GA**C****G****G**UCA..U.A.GU................................**C****C****G**.G.AUGAAGA.**G**..**A**.**G****A****G****C****G****G****G****A** | |
|  |  | NC\_002506.1/1011593-1011455  | **C****A****A****U****A****U****U****C****U****C**.A..GGG.C**G****G****G****G****C**GA...............A**A**UU**C****C****C****C**.ACC.G**G****U****G****G****U**AUGCCGCAAGGC................................................................................................................G**A****G****C**.**C****C****A****C**GA.**G****C****G****C****U****C****G**A........................................................................................................UUCG.................................U**C****G****A****G**.GUC**A****G****C**A-GA....**U**.**C****U****G****G****U**G.A.......G.......**A**..AG.........................**C****C****A****G****G****G****C**.C.GA**C****G****G**UUA..C.A.GU................................**C****C****G**.G.AUGAG.A.**G**..**A**.**G****A****A****U****G****A****C****A** | |
|  |  | NC\_003143.1/717671-717860  | **G****C****U****U****A****U****U****C****U****C**.A..GGG.C**G****G****G****G****U**GA...............A**A**GU**C****C****C****C**.ACC.G**G****C****G****G****U**AAAUUGUAUUGCGACGAUAUAGUCACGUCGGUGGUGCAA.....................................................................................A**A****G****C**.**C****C****G****C**GA.**G****C****G****C****U****C****A**UAUUGUUUCUCUUAU..........................................................................................CCAA.......................GAGAGCAAGGU**A****G****A****G**.GUC**A****G****C**A-GA....**C**.**C****C****G****G****U**G.U.......A.......**A**..UU.........................**C****C****G****G****G****G****C**.C.GA**C****G****G**UUA..U.A.GU................................**C****C****G**.G.AUGGG.A.**G**..**A**.**G****A****G****U****A****A****C****G** | |
|  |  | NC\_004088.1/3904827-3904638  | **G****C****U****U****A****U****U****C****U****C**.A..GGG.C**G****G****G****G****U**GA...............A**A**GU**C****C****C****C**.ACC.G**G****C****G****G****U**AAAUUGUAUUGCGACGAUAUAGUCACGUCGGUGGUGCAA.....................................................................................A**A****G****C**.**C****C****G****C**GA.**G****C****G****C****U****C****A**UAUUGUUUCUCUUAU..........................................................................................CCAA.......................GAGAGCAAGGU**A****G****A****G**.GUC**A****G****C**A-GA....**C**.**C****C****G****G****U**G.U.......A.......**A**..UU.........................**C****C****G****G****G****G****C**.C.GA**C****G****G**UUA..U.A.GU................................**C****C****G**.G.AUGGG.A.**G**..**A**.**G****A****G****U****A****A****C****G** | |
|  |  | NC\_005810.1/3309736-3309925  | **G****C****U****U****A****U****U****C****U****C**.A..GGG.C**G****G****G****G****U**GA...............A**A**GU**C****C****C****C**.ACC.G**G****C****G****G****U**AAAUUGUAUUGCGACGAUAUAGUCACGUCGGUGGUGCAA.....................................................................................A**A****G****C**.**C****C****G****C**GA.**G****C****G****C****U****C****A**UAUUGUUUCUCUUAU..........................................................................................CCAA.......................GAGAGCAAGGU**A****G****A****G**.GUC**A****G****C**A-GA....**C**.**C****C****G****G****U**G.U.......A.......**A**..UU.........................**C****C****G****G****G****G****C**.C.GA**C****G****G**UUA..U.A.GU................................**C****C****G**.G.AUGGG.A.**G**..**A**.**G****A****G****U****A****A****C****G** | |
|  |  | NC\_006155.1/4043394-4043205  | **G****C****U****U****A****U****U****C****U****C**.A..GGG.C**G****G****G****G****U**GA...............A**A**GU**C****C****C****C**.ACC.G**G****C****G****G****U**AAAUUGUAUUGCGACGAUAUAGUCACGUCGGUGGUGCAA.....................................................................................A**A****G****C**.**C****C****G****C**GA.**G****C****G****C****U****C****A**UAUUGUUUCUCUUAU..........................................................................................CCAA.......................GAGAGCAAGGU**A****G****A****G**.GUC**A****G****C**A-GA....**C**.**C****C****G****G****U**G.U.......A.......**A**..UU.........................**C****C****G****G****G****G****C**.C.GA**C****G****G**UUA..U.A.GU................................**C****C****G**.G.AUGGG.A.**G**..**A**.**G****A****G****U****A****A****C****G** | |
|  |  | NZ\_AAAT03000005.1/289914-290085  | **A****A****A****C****G****U****U****C****U****C**.A..GGG.C**G****G****G****G****U**GC...............A**A**UU**C****C****C****C**.ACC.G**G****C****G****G****U**AAUUGCGCGCAAUGUGCA..........................................................................................................U**A****G****C**.**C****C****G****C**GA.**G****C****G****C****U****U****G**GUGACGGACACG.............................................................................................GCAU..................AAGCCGUGAGCGGCAG**C****A****A****G**.GUC**A****G****C**A-GA....**C**.**C****C****G****G****U**G.U.......G.......**A**..UU.........................**C****C****G****G****G****G****C**.C.GA**C****G****G**UCA..C.A.GU................................**C****C****G**.G.AUGAAGA.**G**..**A**.**G****A****A****C****G****G****G****A** | |
|  |  | NC\_002971.2/594013-594149  | **A****A****G****C****G****C****C****U****U****C**.A..GGG.C**G****G****G****G****U**GG...............A**A**GU**C****C****C****C**.ACC.G**G****C****G****G****U**AAGUUUCUUUGAAAU.............................................................................................................A**A****G****C**.**C****C****G****C**GA.**G****U****C****U****C****U****A**.........................................................................................................UUUU..................................**A****A****G****A**....**G****A****C**A-GA....**U**.**C****U****G****G****U**U.A.......A.......**A**..UU.........................**C****C****A****G****A****G****C**.C.GA**C****G****G**UCA..G.A.GU................................**C****C****G**.G.AUGAG.A.**G**..**A**.**A****G****G****U****A****U****G****C** | |
|  |  | NZ\_AADP01000001.1/239232-239409  | **U****C****G****C****A****U****U****C****U****C**.A..GGG.C**A****G****G****G****U**GA...............A**A**UU**C****C****C****U**.ACC.G**G****U****G****G****U**A-..........................................................................................................................A**A****G****C**.**C****C****A****C**GA.**G****C****G****U****U****U****A**AAAGUGCGGUCAAUUUUUGGCAAAUUUUUCUUG........................................................................CUAA..............AUUGUCCUGAUUUUCACUUU**U****A****A****A**.GUC**A****G****C**A-GA....**U**.**U****U****G****G****U**G.A.......A.......**A**..UU.........................**C****C****A****A****A****G****C**.C.GA**C****A****G**.UA..A.A.GU................................**C****U****G**.G.AUGAA.A.**G**..**A**.**G****A****A****U****A****A****A****A** | |
|  |  | NZ\_AABN02000048.1/2926-3089  | **A****C****U****U****A****U****U****C****U****C**.A..GGG.C**G****G****G****G****U**GC...............A**A**UU**C****C****C****C**.ACC.G**G****C****G****G****U**CAGCCGGCCCGCGGUGAUCAGCUCCGCGCCGGCA..........................................................................................C**A****G****C**.**C****C****G****C**GA.**G****C****G****C****C****U****G**C........................................................................................................CGUG..............................GCGG**C****A****G****G**.GUC**A****G****C**A-GA....**U**.**C****C****G****G****U**G.C.......A.......**A**..UU.........................**C****C****G****G****A****G****C**.C.GA**C****G****G**UCA..U.A.GU................................**C****C****G**.G.AUGAA.A.**G**..**A**.**G****G****A****U****A****A****G****G** | |
|  |  | NC\_006300.1/145278-145429  | **G****G****G****C****A****U****U****C****U****C**.A..GGG.C**A****G****G****G****U**GA...............A**A**UU**C****C****C****U**.ACU.G**G****U****G****G****U**AUAUGUUUACAU................................................................................................................A**A****G****C**.**C****C****A****C**GA.**G****C****G****C****C****U****A**UUU......................................................................................................UUAU......................GUGAUGUAGAAU**U****A****G****G**.GUC**A****G****C**A-GA....**U**.**U****U****G****G****U**G.A.......G.......**A**..UU.........................**C****C****A****A****A****G****C**.C.AA**C****A****G**UUA..A.A.GU................................**C****U****G**.G.AUGAA.A.**G**..**A**.**G****A****A****U****A****A****A****A** | |
|  |  | NC\_000907.1/827580-827757  | **U****C****G****C****A****U****U****C****U****C**.A..GGG.C**A****G****G****G****U**GA...............A**A**UU**C****C****C****U**.ACC.G**G****U****G****G****U**A-..........................................................................................................................A**A****G****C**.**C****C****A****C**GA.**G****C****G****U****U****U****A**AAAGUGCGGUCAAUUUUUG......................................................................................GCAAAUUUUUCCUGCGAAAUUGUCCUGAUUUUCACUUU**U****A****A****A**.GUC**A****G****C**A-GA....**U**.**U****U****G****G****U**G.A.......A.......**A**..UU.........................**C****C****A****A****A****G****C**.C.GA**C****A****G**.UA..A.A.GU................................**C****U****G**.G.AUGAA.A.**G**..**A**.**G****A****A****U****A****A****A****A** | |
|  |  | NC\_007146.1/891908-892085  | **U****C****G****C****A****U****U****C****U****C**.A..GGG.C**A****G****G****G****U**GA...............A**A**UU**C****C****C****U**.ACC.G**G****U****G****G****U**A-..........................................................................................................................A**A****G****C**.**C****C****A****C**GA.**G****C****G****U****U****U****A**AAAGUGCGGUCAAUUUUUG......................................................................................GCAAAUUUUUCCUGCGAAAUUGUCCUGAUUUUCACUUU**U****A****A****A**.GUC**A****G****C**A-GA....**U**.**U****U****G****G****U**G.A.......A.......**A**..UU.........................**C****C****A****A****A****G****C**.C.GA**C****A****G**.UA..A.A.GU................................**C****U****G**.G.AUGAA.A.**G**..**A**.**G****A****A****U****A****A****A****A** | |
|  |  | NZ\_AADO01000001.1/389304-389127  | **U****C****G****C****A****U****U****C****U****C**.A..GGG.C**A****G****G****G****U**GA...............A**A**UU**C****C****C****U**.ACC.G**G****U****G****G****U**A-..........................................................................................................................A**A****G****C**.**C****C****A****C**GA.**G****C****G****U****U****U****A**AAAGUGCGGUCAAUUUUUG......................................................................................GCAAAUUUUUCCUGCGAAAUUGUCCUGAUUUUCACUUU**U****A****A****A**.GUC**A****G****C**A-GA....**U**.**U****U****G****G****U**G.A.......A.......**A**..UU.........................**C****C****A****A****A****G****C**.C.GA**C****A****G**.UA..A.A.GU................................**C****U****G**.G.AUGAA.A.**G**..**A**.**G****A****A****U****A****A****A****A** | |
|  |  | NZ\_AAAG02000007.1/77843-78000  | **G****C****U****U****G****U****U****C****U****C**.A..GGG.C**A****G****G****G****U**GA...............A**A**CU**C****C****C****G**.ACC.G**G****C****G****G****U**AAUCCUUCCCUUUCACGGGGGGGA....................................................................................................C**A****G****C**.**C****C****G****C**GA.**G****C****G****C****C****C****U**UU.......................................................................................................GCCG...........................GUGGUGA**A****G****G****G**GUCC**A****G****C**A-GA....**U**.**C****C****G****G****U**G.C.......G.......**A**..AG.........................**C****C****G****G****A****G****C**.C.GA**C****G****G**.UA..U.A.GU................................**C****C****G**.G.AUGGA.A.**G**..**A**.**G****A****A****C****G****A****G****G** | |
|  |  | NC\_006677.1/1075963-1076120  | **A****G****A****C****G****U****U****C****U****C**.A..GGG.C**G****G****G****G****U**GA...............A**A**UU**C****U****C****C**.ACC.G**G****C****G****G****U**AUUGGCGGAUUUCUCCGCCU........................................................................................................C**A****G****C**.**C****C****G****C**GA.**G****C****G****C****C****U****U**CCGG.....................................................................................................AUUU..........................CGGUUCGG**A****A****G****G**GACA**A****G****C**A-GA....**U**.**C****C****G****G****U**G.A.......G.......**A**..CU.........................**C****C****G****G****A****G****C**.C.GA**C****G****G**UUA..G.A.GU................................**C****C****G**.G.AUGGA.A.**G**..**A**.**G****A****A****C****G****C****A****G** | |
|  |  | NC\_004463.1/8190549-8190375  | **A****U****A****A****G****U****U****C****U****C**.A..GGG.C**G****G****G****G****U**GA...............A**A**GU**C****C****C****C**.ACC.G**G****C****G****G****U**AAGGGCCGAAAGGCCU............................................................................................................A**A****G****C**.**C****C****G****C**GA.**G****C****G****C****C****U****U**CU.......................................................................................................CCUU..GAUAGGGAUUCCCUCGGGGACCCCUCGGAGGG**A****A****G****G**.GUC**A****G****C**A-GA....**U**.**U****C****G****G****U**G.C.......A.......**A**..CU.........................**C****C****G****A****A****G****C**.C.GA**C****G****G**UUA..A.A.GU................................**C****C****G**.G.AUGAA.A.**G**..**A**.**G****A****A****C****G****G****U****C** | |
|  |  | NZ\_AAEK01000025.1/41573-41430  | **A****G****C****A****U****C****C****U****U****C**.G..GGG.U**C****G****G****G****U**GA...............A**A**UU**C****C****C****A**.ACC.G**G****C****G****G****U**GAUGAAGUGCAAACUUCU..........................................................................................................A**A****G****U**.**C****C****G****U**GAC**C****C****G****U****U****U****U**C........................................................................................................AAAU................................CG**A****A****A****A**....**C****G****G**UGGA....**U**.**C****U****A****G****U**G.A.......A.......**A**..UU.........................**C****U****A****G****G****G****C**.C.GA**C****A****G**.UA..U.A.GU................................**C****U****G**.G.AUGGG.A.**G**..**A**.**A****G****G****A****U****A****U****G** | |
|  |  | NC\_006448.1/268469-268591  | **A****A****G****G****G****U****C****U****U****C**.G..GGG.C**A****G****G****G****U**GA...............A**A**CU**C****C****C****G**.ACC.G**G****C****G****G****U**GAUUCUUUGGAU................................................................................................................A**A****G****U**.**C****C****G****C**GA.**G****C**-----.........................................................................................................GCAA..................................----....-**G****C**U-GA....**U**.**G****A****G****G****U**G.U.......A.......**A**..UU.........................**C****C****U****C****A****A****C**.C.GA**C****A****G**.UA..U.A.GU................................**C****U****G**.G.AUGGA.A.**G**..**A**.**A****G****A****C****C****A****G****U** | |
|  |  | NZ\_AAGS01000063.1/7272-7150  | **A****A****G****G****G****U****C****U****U****C**.G..GGG.C**A****G****G****G****U**GA...............A**A**CU**C****C****C****G**.ACC.G**G****C****G****G****U**GAUUCUUUGGAU................................................................................................................A**A****G****U**.**C****C****G****C**GA.**G****C**-----.........................................................................................................GCAA..................................----....-**G****C**U-GA....**U**.**G****A****G****G****U**G.U.......A.......**A**..UU.........................**C****C****U****C****A****A****C**.C.GA**C****A****G**.UA..U.A.GU................................**C****U****G**.G.AUGGA.A.**G**..**A**.**A****G****A****C****C****A****G****U** | |
|  |  | NC\_006138.1/773582-773727  | **A****U****U****A****A****U****U****C****U****C**.A..GGG.C**G****G****G****G****U**GG...............A**A**UU**C****C****C****C**.ACC.G**G****C****G****G****U**GAUUCCCUGUGGGGA.............................................................................................................C**A****G****C**.**C****C****G****C**GA.**G****C****G****C****C****U****G**UCA......................................................................................................UUAU................................GG**C****A****G****G**GUCC**A****G****C**U-GA....**U**.**C****U****G****G****U**G.A.......G.......**A**..UU.........................**C****C****A****G****A****G****C**.C.GA**C****G****G**UUA..G.A.GU................................**C****C****G**.G.AAGGA.A.**G**..**A**.**G****A****U****A****A****G****G****C** | |
|  |  | NZ\_AAEI01000006.1/90987-90839  | **G****U****G****C****G****U****C****U****U****C**.A..GGG.C**G****G****G****G****C**GA...............A**A**UU**C****C****C****C**.ACC.G**G****C****G****G****U**AGGCUGGCGUAAGCCGGC..........................................................................................................G**A****G****C**.**C****C****G****C**GA.**G****C****G****C****C****C****G**C........................................................................................................GCAU.............................CCGCG**C****G****G****G**.GUC**A****G****C**A-GA....**U**.**C****U****G****G****U**C.G.......A.......**A**..UG.........................**C****C****A****G****A****G****C**.C.GA**C****G****G**UCA..U.A.GU................................**C****C****G**.G.AUGAG.A.**G**..**A**.**A****G****A****U****G****U****G****C** | |
|  |  | NC\_004350.1/1615325-1615112  | **A****A****G****U****G****U****C****U****U****C**.A..GGG.C**A****G****G****G****U**GA...............G**A**UU**C****C****C****G**.ACC.G**G****C****G****G****U**GACAAAGGCGAUGAUUUUUCAACGAGAUUAGAUAGUAAGUCGAAGAUGAUAUUAGACUAUAUCAAGAUGAGCUAAUGAUGCGUCAGAGAAAAGAUUCCUUUUG.....................A**A****G****U**.**C****C****G****C**GA.**G****C**-----.........................................................................................................GCAA..................................----....-**G****C**U-GA....**U**.**G****U****G****G****U**G.A.......A.......**A**..UU.........................**C****C****A****C****A****A****C**.C.GA**C****A****G**.UA..A.A.GU................................**C****U****G**.G.AUGGG.A.**G**..**A**.**A****G****A****C****U****G****A****G** | |
|  |  | NC\_006350.1/772307-772458  | **G****U****G****C****G****U****C****U****U****C**.A..GGG.C**G****G****G****G****C**GA...............A**A**UU**C****C****C****C**.ACC.G**G****C****G****G****U**AGGCCGGCAUGUUGCCGGC.........................................................................................................G**A****G****C**.**C****C****G****C**GA.**G****C****G****C****C****C****G**CGC......................................................................................................GAUU.............................GCGCG**C****G****G****G**.GUC**A****G****C**A-GA....**U**.**C****U****G****G****U**C.C.......G.......**A**..UG.........................**C****C****A****G****A****G****C**.C.GA**C****G****G**UCA..U.A.GU................................**C****C****G**.G.AUGAA.A.**G**..**A**.**A****G****A****U****G****U****G****C** | |
|  |  | NZ\_AAHR01000045.1/20893-20742  | **G****U****G****C****G****U****C****U****U****C**.A..GGG.C**G****G****G****G****C**GA...............A**A**UU**C****C****C****C**.ACC.G**G****C****G****G****U**AGGCCGGCAUGUUGCCGGC.........................................................................................................G**A****G****C**.**C****C****G****C**GA.**G****C****G****C****C****C****G**CGC......................................................................................................GAUU.............................GCGCG**C****G****G****G**.GUC**A****G****C**A-GA....**U**.**C****U****G****G****U**C.C.......G.......**A**..UG.........................**C****C****A****G****A****G****C**.C.GA**C****G****G**UCA..U.A.GU................................**C****C****G**.G.AUGAA.A.**G**..**A**.**A****G****A****U****G****U****G****C** | |
|  |  | NZ\_AAHS01000021.1/20898-20747  | **G****U****G****C****G****U****C****U****U****C**.A..GGG.C**G****G****G****G****C**GA...............A**A**UU**C****C****C****C**.ACC.G**G****C****G****G****U**AGGCCGGCAUGUUGCCGGC.........................................................................................................G**A****G****C**.**C****C****G****C**GA.**G****C****G****C****C****C****G**CGC......................................................................................................GAUU.............................GCGCG**C****G****G****G**.GUC**A****G****C**A-GA....**U**.**C****U****G****G****U**C.C.......G.......**A**..UG.........................**C****C****A****G****A****G****C**.C.GA**C****G****G**UCA..U.A.GU................................**C****C****G**.G.AUGAA.A.**G**..**A**.**A****G****A****U****G****U****G****C** | |
|  |  | NZ\_AAHT01000001.1/929904-930055  | **G****U****G****C****G****U****C****U****U****C**.A..GGG.C**G****G****G****G****C**GA...............A**A**UU**C****C****C****C**.ACC.G**G****C****G****G****U**AGGCCGGCAUGUUGCCGGC.........................................................................................................G**A****G****C**.**C****C****G****C**GA.**G****C****G****C****C****C****G**CGC......................................................................................................GAUU.............................GCGCG**C****G****G****G**.GUC**A****G****C**A-GA....**U**.**C****U****G****G****U**C.C.......G.......**A**..UG.........................**C****C****A****G****A****G****C**.C.GA**C****G****G**UCA..U.A.GU................................**C****C****G**.G.AUGAA.A.**G**..**A**.**A****G****A****U****G****U****G****C** | |
|  |  | NZ\_AAHU01000005.1/126143-126294  | **G****U****G****C****G****U****C****U****U****C**.A..GGG.C**G****G****G****G****C**GA...............A**A**UU**C****C****C****C**.ACC.G**G****C****G****G****U**AGGCCGGCAUGUUGCCGGC.........................................................................................................G**A****G****C**.**C****C****G****C**GA.**G****C****G****C****C****C****G**CGC......................................................................................................GAUU.............................GCGCG**C****G****G****G**.GUC**A****G****C**A-GA....**U**.**C****U****G****G****U**C.C.......G.......**A**..UG.........................**C****C****A****G****A****G****C**.C.GA**C****G****G**UCA..U.A.GU................................**C****C****G**.G.AUGAA.A.**G**..**A**.**A****G****A****U****G****U****G****C** | |
|  |  | NZ\_AAHV01000077.1/20883-20732  | **G****U****G****C****G****U****C****U****U****C**.A..GGG.C**G****G****G****G****C**GA...............A**A**UU**C****C****C****C**.ACC.G**G****C****G****G****U**AGGCCGGCAUGUUGCCGGC.........................................................................................................G**A****G****C**.**C****C****G****C**GA.**G****C****G****C****C****C****G**CGC......................................................................................................GAUU.............................GCGCG**C****G****G****G**.GUC**A****G****C**A-GA....**U**.**C****U****G****G****U**C.C.......G.......**A**..UG.........................**C****C****A****G****A****G****C**.C.GA**C****G****G**UCA..U.A.GU................................**C****C****G**.G.AUGAA.A.**G**..**A**.**A****G****A****U****G****U****G****C** | |
|  |  | NZ\_AAHW01000035.1/21295-21144  | **G****U****G****C****G****U****C****U****U****C**.A..GGG.C**G****G****G****G****C**GA...............A**A**UU**C****C****C****C**.ACC.G**G****C****G****G****U**AGGCCGGCAUGUUGCCGGC.........................................................................................................G**A****G****C**.**C****C****G****C**GA.**G****C****G****C****C****C****G**CGC......................................................................................................GAUU.............................GCGCG**C****G****G****G**.GUC**A****G****C**A-GA....**U**.**C****U****G****G****U**C.C.......G.......**A**..UG.........................**C****C****A****G****A****G****C**.C.GA**C****G****G**UCA..U.A.GU................................**C****C****G**.G.AUGAA.A.**G**..**A**.**A****G****A****U****G****U****G****C** | |
|  |  | NC\_003028.1/467151-467385  | **A****A****G****A****G****U****C****U****U****C**.A..GGG.C**A****G****G****G****U**GA...............A**A**UU**C****C****C****G**.ACC.G**G****C****G****G****U**GACUUUAACUAGGAAAUGAUCUUUUCCUUUUAUACUUUGUUGACAAGCUUUGCCUAACCAGAAGUUAUGCCUACAGCUUGUCGCCUAGUCUAAAAGAAAAAUCUCUAUUUCCUUCUCUUUAAAGA**A****G****U**.**C****C****G****U**GA.**G****C**-----.........................................................................................................GCAA..................................----....-**G****C**U-GA....**U**.**G****U****G****G****U**G.A.......G.......**A**..UU.........................**C****C****A****C****A****A****C**.C.GA**C****A****G**.UA..U.A.GU................................**C****U****G**.G.AUGGG.A.**G**..**A**.**A****G****A****C****G****A****A****A** | |
|  |  | NZ\_AAGY01000002.1/45062-44828  | **A****A****G****A****G****U****C****U****U****C**.A..GGG.C**A****G****G****G****U**GA...............A**A**UU**C****C****C****G**.ACC.G**G****C****G****G****U**GACUUUAACUAGGAAAUGAUCUUUUCCUUUUAUACUUUGUUGACAAGCUUUGCCUAACCAGAAGUUAUGCCUACAGCUUGUCGCCUAGUCUAAAAGAAAAAUCUCUAUUUCCUUCUCUUUAAAGA**A****G****U**.**C****C****G****U**GA.**G****C**-----.........................................................................................................GCAA..................................----....-**G****C**U-GA....**U**.**G****U****G****G****U**G.A.......G.......**A**..UU.........................**C****C****A****C****A****A****C**.C.GA**C****A****G**.UA..U.A.GU................................**C****U****G**.G.AUGGG.A.**G**..**A**.**A****G****A****C****G****A****A****A** | |
|  |  | NC\_003030.1/682355-682469  | **G****A****U****G****U****U****C****U****U****C**.A..GGG.A**U****G****G****G****U**GA...............A**A**UU**C****C****C****A**.AUC.G**G****C****G****G****U**A-..........................................................................................................................A**A****G****C**.**C****C****G****C**AA.**G****C****C**----.........................................................................................................AUUU..................................----....**G****G****C**A-GA....**U**.**C****C****G****G****U**U.A.......A.......**A**..CU.........................**C****C****G****G****G****G****C**.C.GA**C****A****G**UUA..A.A.GU................................**C****U****G**.G.AUGAA.A.**G**..**A**.**A****G****A****A****A****U****A****G** | |
|  |  | NC\_002516.1/4536838-4536676  | **U****A****A****C****G****U****U****C****U****C**.A..GGG.C**G****G****G****G****U**GA...............A**A**GU**C****C****C****C**.ACC.G**G****C****G****G****U**AAUGGCGCGCAAGGCGCC..........................................................................................................U**A****G****C**.**C****C****G****C**GA.**G****C****G****C****U****U****G**CCGGACCG.................................................................................................GCCA.......................CCGCCGGACGA**C****A****A****G**.GUC**A****G****C**A-GA....**C**.**C****C****G****G****U**G.C.......G.......**A**..UU.........................**C****C****G****G****G****G****C**.C.GA**C****G****G**UCA..U.A.GU................................**C****C****G**.G.AUAAAGA.**G**..**A**.**G****A****A****C****G****G****G****A** | |
|  |  | NZ\_AABQ07000001.1/1858082-1857920  | **U****A****A****C****G****U****U****C****U****C**.A..GGG.C**G****G****G****G****U**GA...............A**A**GU**C****C****C****C**.ACC.G**G****C****G****G****U**AAUGGCGCGCAAGGUGCC..........................................................................................................U**A****G****C**.**C****C****G****C**GA.**G****C****G****C****U****U****G**CCGGACCG.................................................................................................GCCA.......................CCGCCGGACGA**C****A****A****G**.GUC**A****G****C**A-GA....**C**.**C****C****G****G****U**G.C.......G.......**A**..UU.........................**C****C****G****G****G****G****C**.C.GA**C****G****G**UCA..U.A.GU................................**C****C****G**.G.AUAAAGA.**G**..**A**.**G****A****A****C****G****G****G****A** | |
|  |  | NC\_002940.2/943036-943169  | **U****U****U****A****A****U****C****U****U****C**.A..GGG.C**A****G****G****G****U**GA...............A**A**UU**C****C****C****G**.AUC.G**G****U****G****G****U**A-..........................................................................................................................A**A****G****U**.**C****C****G****C**GA.**G****C****C****G****A****A****C**UUCGA....................................................................................................UUAA............................CAUCAA**G****U****U****U**....**A****G****C**AGGA....**A**.**C****U****A****G****U**G.A.......A.......**A**..UU.........................**C****U****A****G****U****A****C**.C.GA**C****A****G**.UA..U.A.GU................................**C****U****G**.G.AUGGA.A.**G**..**A**.**A****G****A****G****C****A****G****A** | |
|  |  | NC\_004461.1/1494108-1493970  | **U****A****U****U****U****C****U****U****U****C**.G..GGG.C**A****G****G****G****U**GA...............A**A**UU**C****C****C****A**.ACC.G**G****C****A****G****U**AAAUU.......................................................................................................................G**A****G****C**.**C****U****G****C**GAC**C****C****A****C****U****A****A**UAUU.....................................................................................................GUUU............................UUGAUG**U****U****A****G**....**U****G****G**CUGA....**U**.**C****U****A****G****U**G.U.......G.......**A**..AU.........................**C****U****A****G****A****G****C**.C.GA**C****A****G**UUA..G.A.GU................................**C****U****G**.G.AUGGG.A.**G**..**A**.**A****A****G****A****A****U****U****U** | |
|  |  | NC\_003919.1/888098-888276  | **G****A****A****C****G****U****C****U****U****C**.A..GGG.C**G****G****G****G****U**GC...............G**A**UU**C****C****C****C**.ACC.G**G****C****G****G****U**AGGCAUGCGCAAGCAUGC..........................................................................................................G**A****G****C**.**C****C****G****C**GA.**G****C****G****C****U****U****G**CACUCCAGGGUUGAGCGGA......................................................................................CUAA................CGCCAAACCCCUGCUGCG**C****A****A****G**.GUC**A****G****C**A-GA....**U**.**C****C****G****G****U**C.C.......G.......**A**..UG.........................**C****C****G****G****A****G****C**.C.GA**C****G****G**.UA..U.A.GU................................**C****C****G**.G.AUGAA.A.**G**..**A**.**A****G****A****C****G****G****C****C** | |
|  |  | NC\_002976.3/1390073-1389935  | **U****A****A****U****U****C****U****U****U****C**.G..GGG.C**A****G****G****G****U**GA...............A**A**UU**C****C****C****A**.ACC.G**G****C****A****G****U**AAAUU.......................................................................................................................G**A****G****C**.**C****U****G****C**GAC**C****C****A****C****U****A****A**UAUU.....................................................................................................GUUU............................UUGAUG**U****U****A****G**....**U****G****G**CUGA....**U**.**C****U****A****G****U**G.U.......G.......**A**..AU.........................**C****U****A****G****A****G****C**.C.GA**C****A****G**UUA..G.A.GU................................**C****U****G**.G.AUGGG.A.**G**..**A**.**A****A****G****A****A****G****U****U** | |
|  |  | NC\_004578.1/2009078-2008912  | **U****A****A****C****G****U****U****C****U****C**.A..GGG.C**G****G****G****G****U**GC...............A**A**CU**C****C****C****C**.ACC.G**G****C****G****G****U**AAUGGCGCGCAAUGCGUC..........................................................................................................U**A****G****C**.**C****C****G****C**GA.**G****C****G****C****U****U****G**GGGGUUUCAG...............................................................................................CUUU.....................GGCUGACGACCUG**C****A****A****G**.GUC**A****G****C**A-GA....**C**.**C****C****G****G****U**G.U.......G.......**A**..UU.........................**C****C****G****G****G****G****C**.C.GA**C****G****G**UCA..U.A.GU................................**C****C****G**.G.AUGAAGA.**G**..**A**.**G****A****G****C****G****G****G****A** | |
|  |  | NC\_002937.3/1840778-1840631  | **G****C****C****A****A****U****U****C****U****C**.A..GGG.C**G****G****G****G****U**GC...............A**A**UU**C****C****C****C**.ACC.G**G****U****G****G****U**AUCCGGCGUAUGCCGGU...........................................................................................................A**A****G****C**.**C****C****A****C**GA.**G****C****G****C****C****U****C**U........................................................................................................CCAC.............................GGGGA**G****A****G****G**.GUC**A****G****C**A-GA....**U**.**C****U****G****G****U**G.A.......G.......**A**..GA.........................**C****C****A****G****A****G****C**.C.GA**C****G****G**UGA..C.A.GU................................**C****C****G**.G.AUGAA.A.**G**..**A**.**G****A****A****U****G****C****G****G** | |
|  |  | NC\_004567.1/1315753-1315883  | **C****G****A****U****U****U****C****U****U****C**.G..GGG.C**A****G****G****G****U**GC...............A**A**UU**C****C****C****G**.ACC.G**A****C****G****G****U**AACAACGUAAGUUG..............................................................................................................A**A****G****U**.**C****C****G****U**GAC**C****C****G****C**---.........................................................................................................GUGA..................................---**G**....**C****G****G**UGGA....**C**.**C****C****A****G****U**G.C.......A.......**A**..GU.........................**C****U****G****G****G****A****C**.C.GA**C****A****G**.UA..U.A.GU................................**C****U****G**.G.AUGGG.A.**G**..**A**.**A****G****A****A****A****A****U****U** | |
|  |  | NZ\_AAEH02000062.1/30589-30441  | **G****U****G****C****G****U****C****U****U****C**.A..GGG.C**G****G****G****G****C**GA...............A**A**UU**C****C****C****C**.ACC.G**G****C****G****G****U**AGGCUGGCGAAAGCCGGC..........................................................................................................G**A****G****C**.**C****C****G****C**GA.**G****C****G****C****C****C****G**C........................................................................................................GCAG.............................CUGCG**C****G****G****G**.GUC**A****G****C**A-GA....**U**.**C****U****G****G****U**C.G.......A.......**A**..UG.........................**C****C****A****G****A****G****C**.C.GA**C****G****G**UCA..U.A.GU................................**C****C****G**.G.AUGAG.A.**G**..**A**.**A****G****A****U****G****U****G****C** | |
|  |  | NZ\_AAHI01000063.1/9021-8873  | **G****U****G****C****G****U****C****U****U****C**.A..GGG.C**G****G****G****G****C**GA...............A**A**UU**C****C****C****C**.ACC.G**G****C****G****G****U**AGGCUGGCGUAAGCCGGC..........................................................................................................G**A****G****C**.**C****C****G****C**GA.**G****C****G****C****C****C****G**C........................................................................................................GCAU.............................CCGCG**C****G****G****G**.GUC**A****G****C**A-GA....**U**.**C****U****G****G****U**C.G.......A.......**A**..UG.........................**C****C****A****G****A****G****C**.C.GA**C****G****G**UCA..C.A.GU................................**C****C****G**.G.AUGAG.A.**G**..**A**.**A****G****A****U****G****U****G****C** | |
|  |  | NZ\_AAHL01000015.1/94812-94664  | **G****U****G****C****G****U****C****U****U****C**.A..GGG.C**G****G****G****G****C**GA...............A**A**UU**C****C****C****C**.ACC.G**G****C****G****G****U**AGGCUGGCGUAAGCCGGC..........................................................................................................G**A****G****C**.**C****C****G****C**GA.**G****C****G****C****C****C****G**C........................................................................................................GCAU.............................CCGCG**C****G****G****G**.GUC**A****G****C**A-GA....**U**.**C****U****G****G****U**C.G.......A.......**A**..UG.........................**C****C****A****G****A****G****C**.C.GA**C****G****G**UCA..C.A.GU................................**C****C****G**.G.AUGAG.A.**G**..**A**.**A****G****A****U****G****U****G****C** | |
|  |  | NC\_003098.1/433445-433679  | **A****A****G****A****G****U****C****U****U****C**.A..GGG.C**A****G****G****G****U**GU...............G**A**UU**C****C****C****G**.ACC.G**G****C****G****G****U**GACUUUAACUAGGAAAUGAUCUUUUCCUUUUAUACUUUGUUGACAAGCUUUGCCUAACCAGAAGUUAUGCCUACAGCUUGUCGCCUAGUCUAAAAGAAAAAUCUCUAUUUCCUUCUCUUUAAAGA**A****G****U**.**C****C****G****C**GA.**G****C**-----.........................................................................................................GCAA..................................----....-**G****C**U-GA....**U**.**G****U****G****G****U**G.A.......G.......**A**..UU.........................**C****C****A****C****A****A****C**.C.GA**C****A****G**.UA..U.A.GU................................**C****U****G**.G.AUGGG.A.**G**..**A**.**A****G****A****C****G****A****A****A** | |
|  |  | NC\_004116.1/739286-739400  | **G****A****A****U****U****U****C****U****U****C**.G..GGG.U**C****A****G****G****U**GA...............A**A**AU**C****C****U****A**.ACC.G**G****C****G****G****U**A-..........................................................................................................................U**A****G****U**.**C****C****G****C**GA.**G****C****U****U**---.........................................................................................................-UC-..................................---**G**....**A****G****C**AUGA....**A**.**C****U****G****G****U**G.U.......G.......**A**..UU.........................**C****C****A****G****U****A****C**.C.GA**C****A****G**.UA..A.A.GU................................**C****U****G**.G.AUGAG.A.**G**..**A**.**A****G****A****A****U****U****C****A** | |
|  |  | NC\_004368.1/784995-785109  | **G****A****A****U****U****U****C****U****U****C**.G..GGG.U**C****A****G****G****U**GA...............A**A**AU**C****C****U****A**.ACC.G**G****C****G****G****U**A-..........................................................................................................................U**A****G****U**.**C****C****G****C**GA.**G****C****U****U**---.........................................................................................................-UC-..................................---**G**....**A****G****C**AUGA....**A**.**C****U****G****G****U**G.U.......G.......**A**..UU.........................**C****C****A****G****U****A****C**.C.GA**C****A****G**.UA..A.A.GU................................**C****U****G**.G.AUGAG.A.**G**..**A**.**A****G****A****A****U****U****C****A** | |
|  |  | NC\_005126.1/4649612-4649435  | **G****C****U****U****A****U**-**C****U****C**.A..GGG.C**G****G****G****G****U**GA...............A**A**GU**C****C****C****C**.ACC.G**G****C****G****G****U**AAACAUCAGUCCAUUUUUUGAGUGGCAUGGUG............................................................................................A**A****G****C**.**C****C****G****C**GA.**G****C****G****C****U****C****C**GUUUGUGGUUAU.............................................................................................CUUA........................AUGAUAAAGC**G****G****A****G**.GUC**A****G****C**A-GA....**U**.**C****C****A****G****U**G.U.......A.......**A**..UU.........................**C****U****G****G****A****G****C**.C.GA**C****G****G**UGA..U.A.GU................................**C****C****G**.G.AUGGG.A.**G**..**A**.**G****A****A****U****A****A****C****G** | |
|  |  | NZ\_AAEV01000003.1/30598-30730  | **A****G****A****U****U****U****C****U****U****C**.G..GGG.C**A****G****G****G****U**GU...............A**A**UU**C****C****C****G**.ACC.G**A****C****G****G****U**AACAACCAAGGGUUG.............................................................................................................A**A****G****U**.**C****C****G****U**GAC**C****C****G****C****G****A**-.........................................................................................................----..................................**G****U****A****G**....**C****G****G**UGGA....**C**.**C****U****A****G****U**G.A.......G.......**A**..GU.........................**C****U****A****G****G****A****C**.C.GA**C****A****G**.UA..U.A.GU................................**C****U****G**.G.AUGGG.A.**G**..**A**.**A****G****A****A****A****C****G****U** | |
|  |  | NC\_003909.8/3897823-3897966  | **A****G****C****A****U****C****C****U****U****C**.G..GGG.U**C****G****G****G****U**GA...............A**A**UU**C****C****C****A**.ACC.G**G****C****G****G****U**GAUGAAGUGCAAACUUCU..........................................................................................................A**A****G****U**.**C****C****G****U**GAC**C****C****G****U****U****U****U**C........................................................................................................AACU................................CG**A****A****A****A**....**C****G****G**UGGA....**U**.**C****U****A****G****U**G.A.......A.......**A**..CU.........................**C****U****A****G****G****G****C**.C.GA**C****A****G**.UA..U.A.GU................................**C****U****G**.G.AUGGG.A.**G**..**A**.**A****G****G****A****U****A****U****G** | |
|  |  | NC\_003997.3/3953886-3954029  | **A****G****C****A****U****C****C****U****U****C**.G..GGG.U**C****G****G****G****U**GA...............A**A**UU**C****C****C****A**.ACC.G**G****C****G****G****U**GAUGAAGUGCAAACUUCU..........................................................................................................A**A****G****U**.**C****C****G****U**GAC**C****C****G****U****U****U****U**C........................................................................................................AACU................................CG**A****A****A****A**....**C****G****G**UGGA....**U**.**C****U****A****G****U**G.A.......A.......**A**..CU.........................**C****U****A****G****G****G****C**.C.GA**C****A****G**.UA..U.A.GU................................**C****U****G**.G.AUGGG.A.**G**..**A**.**A****G****G****A****U****A****U****G** | |
|  |  | NC\_004722.1/4072882-4073025  | **A****G****C****A****U****C****C****U****U****C**.G..GGG.U**C****G****G****G****U**GA...............A**A**UU**C****C****C****A**.ACC.G**G****C****G****G****U**GAUGAAGUGAAAACUUCU..........................................................................................................A**A****G****U**.**C****C****G****U**GAC**C****C****G****U****U****U****U**C........................................................................................................AACU................................CG**A****A****A****A**....**C****G****G**UGGA....**U**.**C****U****A****G****U**G.A.......A.......**A**..CU.........................**C****U****A****G****G****G****C**.C.GA**C****A****G**.UA..U.A.GU................................**C****U****G**.G.AUGGG.A.**G**..**A**.**A****G****G****A****U****A****U****G** | |
|  |  | NC\_005945.1/3954385-3954528  | **A****G****C****A****U****C****C****U****U****C**.G..GGG.U**C****G****G****G****U**GA...............A**A**UU**C****C****C****A**.ACC.G**G****C****G****G****U**GAUGAAGUGCAAACUUCU..........................................................................................................A**A****G****U**.**C****C****G****U**GAC**C****C****G****U****U****U****U**C........................................................................................................AACU................................CG**A****A****A****A**....**C****G****G**UGGA....**U**.**C****U****A****G****U**G.A.......A.......**A**..CU.........................**C****U****A****G****G****G****C**.C.GA**C****A****G**.UA..U.A.GU................................**C****U****G**.G.AUGGG.A.**G**..**A**.**A****G****G****A****U****A****U****G** | |
|  |  | NC\_005957.1/3935950-3936093  | **A****G****C****A****U****C****C****U****U****C**.G..GGG.U**C****G****G****G****U**GA...............A**A**UU**C****C****C****A**.ACC.G**G****C****G****G****U**GAUGAAGUGCAAACUUCU..........................................................................................................A**A****G****U**.**C****C****G****U**GAC**C****C****G****U****U****U****U**C........................................................................................................AACU................................CG**A****A****A****A**....**C****G****G**UGGA....**U**.**C****U****A****G****U**G.A.......A.......**A**..CU.........................**C****U****A****G****G****G****C**.C.GA**C****A****G**.UA..U.A.GU................................**C****U****G**.G.AUGGG.A.**G**..**A**.**A****G****G****A****U****A****U****G** | |
|  |  | NC\_006274.1/3991406-3991549  | **A****G****C****A****U****C****C****U****U****C**.G..GGG.U**C****G****G****G****U**GA...............A**A**UU**C****C****C****A**.ACC.G**G****C****G****G****U**GAUGAAGUGCAAACUUCU..........................................................................................................A**A****G****U**.**C****C****G****U**GAC**C****C****G****U****U****U****U**C........................................................................................................AACU................................CG**A****A****A****A**....**C****G****G**UGGA....**U**.**C****U****A****G****U**G.A.......A.......**A**..CU.........................**C****U****A****G****G****G****C**.C.GA**C****A****G**.UA..U.A.GU................................**C****U****G**.G.AUGGG.A.**G**..**A**.**A****G****G****A****U****A****U****G** | |
|  |  | NC\_007530.2/3954013-3954156  | **A****G****C****A****U****C****C****U****U****C**.G..GGG.U**C****G****G****G****U**GA...............A**A**UU**C****C****C****A**.ACC.G**G****C****G****G****U**GAUGAAGUGCAAACUUCU..........................................................................................................A**A****G****U**.**C****C****G****U**GAC**C****C****G****U****U****U****U**C........................................................................................................AACU................................CG**A****A****A****A**....**C****G****G**UGGA....**U**.**C****U****A****G****U**G.A.......A.......**A**..CU.........................**C****U****A****G****G****G****C**.C.GA**C****A****G**.UA..U.A.GU................................**C****U****G**.G.AUGGG.A.**G**..**A**.**A****G****G****A****U****A****U****G** | |
|  |  | NZ\_AAAC02000001.1/4403563-4403706  | **A****G****C****A****U****C****C****U****U****C**.G..GGG.U**C****G****G****G****U**GA...............A**A**UU**C****C****C****A**.ACC.G**G****C****G****G****U**GAUGAAGUGCAAACUUCU..........................................................................................................A**A****G****U**.**C****C****G****U**GAC**C****C****G****U****U****U****U**C........................................................................................................AACU................................CG**A****A****A****A**....**C****G****G**UGGA....**U**.**C****U****A****G****U**G.A.......A.......**A**..CU.........................**C****U****A****G****G****G****C**.C.GA**C****A****G**.UA..U.A.GU................................**C****U****G**.G.AUGGG.A.**G**..**A**.**A****G****G****A****U****A****U****G** | |
|  |  | NZ\_AAEN01000013.1/208396-208539  | **A****G****C****A****U****C****C****U****U****C**.G..GGG.U**C****G****G****G****U**GA...............A**A**UU**C****C****C****A**.ACC.G**G****C****G****G****U**GAUGAAGUGCAAACUUCU..........................................................................................................A**A****G****U**.**C****C****G****U**GAC**C****C****G****U****U****U****U**C........................................................................................................AACU................................CG**A****A****A****A**....**C****G****G**UGGA....**U**.**C****U****A****G****U**G.A.......A.......**A**..CU.........................**C****U****A****G****G****G****C**.C.GA**C****A****G**.UA..U.A.GU................................**C****U****G**.G.AUGGG.A.**G**..**A**.**A****G****G****A****U****A****U****G** | |
|  |  | NZ\_AAEO01000019.1/295488-295631  | **A****G****C****A****U****C****C****U****U****C**.G..GGG.U**C****G****G****G****U**GA...............A**A**UU**C****C****C****A**.ACC.G**G****C****G****G****U**GAUGAAGUGCAAACUUCU..........................................................................................................A**A****G****U**.**C****C****G****U**GAC**C****C****G****U****U****U****U**C........................................................................................................AACU................................CG**A****A****A****A**....**C****G****G**UGGA....**U**.**C****U****A****G****U**G.A.......A.......**A**..CU.........................**C****U****A****G****G****G****C**.C.GA**C****A****G**.UA..U.A.GU................................**C****U****G**.G.AUGGG.A.**G**..**A**.**A****G****G****A****U****A****U****G** | |
|  |  | NZ\_AAEP01000031.1/151592-151449  | **A****G****C****A****U****C****C****U****U****C**.G..GGG.U**C****G****G****G****U**GA...............A**A**UU**C****C****C****A**.ACC.G**G****C****G****G****U**GAUGAAGUGCAAACUUCU..........................................................................................................A**A****G****U**.**C****C****G****U**GAC**C****C****G****U****U****U****U**C........................................................................................................AACU................................CG**A****A****A****A**....**C****G****G**UGGA....**U**.**C****U****A****G****U**G.A.......A.......**A**..CU.........................**C****U****A****G****G****G****C**.C.GA**C****A****G**.UA..U.A.GU................................**C****U****G**.G.AUGGG.A.**G**..**A**.**A****G****G****A****U****A****U****G** | |
|  |  | NZ\_AAEQ01000038.1/229779-229922  | **A****G****C****A****U****C****C****U****U****C**.G..GGG.U**C****G****G****G****U**GA...............A**A**UU**C****C****C****A**.ACC.G**G****C****G****G****U**GAUGAAGUGCAAACUUCU..........................................................................................................A**A****G****U**.**C****C****G****U**GAC**C****C****G****U****U****U****U**C........................................................................................................AACU................................CG**A****A****A****A**....**C****G****G**UGGA....**U**.**C****U****A****G****U**G.A.......A.......**A**..CU.........................**C****U****A****G****G****G****C**.C.GA**C****A****G**.UA..U.A.GU................................**C****U****G**.G.AUGGG.A.**G**..**A**.**A****G****G****A****U****A****U****G** | |
|  |  | NZ\_AAER01000035.1/466732-466875  | **A****G****C****A****U****C****C****U****U****C**.G..GGG.U**C****G****G****G****U**GA...............A**A**UU**C****C****C****A**.ACC.G**G****C****G****G****U**GAUGAAGUGCAAACUUCU..........................................................................................................A**A****G****U**.**C****C****G****U**GAC**C****C****G****U****U****U****U**C........................................................................................................AACU................................CG**A****A****A****A**....**C****G****G**UGGA....**U**.**C****U****A****G****U**G.A.......A.......**A**..CU.........................**C****U****A****G****G****G****C**.C.GA**C****A****G**.UA..U.A.GU................................**C****U****G**.G.AUGGG.A.**G**..**A**.**A****G****G****A****U****A****U****G** | |
|  |  | NZ\_AAES01000024.1/206398-206541  | **A****G****C****A****U****C****C****U****U****C**.G..GGG.U**C****G****G****G****U**GA...............A**A**UU**C****C****C****A**.ACC.G**G****C****G****G****U**GAUGAAGUGCAAACUUCU..........................................................................................................A**A****G****U**.**C****C****G****U**GAC**C****C****G****U****U****U****U**C........................................................................................................AACU................................CG**A****A****A****A**....**C****G****G**UGGA....**U**.**C****U****A****G****U**G.A.......A.......**A**..CU.........................**C****U****A****G****G****G****C**.C.GA**C****A****G**.UA..U.A.GU................................**C****U****G**.G.AUGGG.A.**G**..**A**.**A****G****G****A****U****A****U****G** | |
|  |  | NC\_006348.1/247640-247791  | **G****U****G****C****G****U****C****U****U****C**.A..GGG.C**G****G****G****G****C**GA...............A**A**AU**C****C****C****C**.ACC.G**G****C****G****G****U**AGGCCGGCAUGUUGCCGGC.........................................................................................................G**A****G****C**.**C****C****G****C**GA.**G****C****G****C****C****C****G**CGC......................................................................................................GAUU.............................GCGCG**C****G****G****G**.GUC**A****G****C**A-GA....**U**.**C****U****G****G****U**C.C.......G.......**A**..UG.........................**C****C****A****G****A****G****C**.C.GA**C****G****G**UCA..U.A.GU................................**C****C****G**.G.AUGAA.A.**G**..**A**.**A****G****A****U****G****U****G****C** | |
|  |  | NZ\_AAHM01000003.1/610218-610369  | **G****U****G****C****G****U****C****U****U****C**.A..GGG.C**G****G****G****G****C**GA...............A**A**AU**C****C****C****C**.ACC.G**G****C****G****G****U**AGGCCGGCAUGUUGCCGGC.........................................................................................................G**A****G****C**.**C****C****G****C**GA.**G****C****G****C****C****C****G**CGC......................................................................................................GAUU.............................GCGCG**C****G****G****G**.GUC**A****G****C**A-GA....**U**.**C****U****G****G****U**C.C.......G.......**A**..UG.........................**C****C****A****G****A****G****C**.C.GA**C****G****G**UCA..U.A.GU................................**C****C****G**.G.AUGAA.A.**G**..**A**.**A****G****A****U****G****U****G****C** | |
|  |  | NZ\_AAHN01000010.1/20880-20729  | **G****U****G****C****G****U****C****U****U****C**.A..GGG.C**G****G****G****G****C**GA...............A**A**AU**C****C****C****C**.ACC.G**G****C****G****G****U**AGGCCGGCAUGUUGCCGGC.........................................................................................................G**A****G****C**.**C****C****G****C**GA.**G****C****G****C****C****C****G**CGC......................................................................................................GAUU.............................GCGCG**C****G****G****G**.GUC**A****G****C**A-GA....**U**.**C****U****G****G****U**C.C.......G.......**A**..UG.........................**C****C****A****G****A****G****C**.C.GA**C****G****G**UCA..U.A.GU................................**C****C****G**.G.AUGAA.A.**G**..**A**.**A****G****A****U****G****U****G****C** | |
|  |  | NZ\_AAHO01000031.1/36416-36567  | **G****U****G****C****G****U****C****U****U****C**.A..GGG.C**G****G****G****G****C**GA...............A**A**AU**C****C****C****C**.ACC.G**G****C****G****G****U**AGGCCGGCAUGUUGCCGGC.........................................................................................................G**A****G****C**.**C****C****G****C**GA.**G****C****G****C****C****C****G**CGC......................................................................................................GAUU.............................GCGCG**C****G****G****G**.GUC**A****G****C**A-GA....**U**.**C****U****G****G****U**C.C.......G.......**A**..UG.........................**C****C****A****G****A****G****C**.C.GA**C****G****G**UCA..U.A.GU................................**C****C****G**.G.AUGAA.A.**G**..**A**.**A****G****A****U****G****U****G****C** | |
|  |  | NZ\_AAHP01000016.1/67372-67523  | **G****U****G****C****G****U****C****U****U****C**.A..GGG.C**G****G****G****G****C**GA...............A**A**AU**C****C****C****C**.ACC.G**G****C****G****G****U**AGGCCGGCAUGUUGCCGGC.........................................................................................................G**A****G****C**.**C****C****G****C**GA.**G****C****G****C****C****C****G**CGC......................................................................................................GAUU.............................GCGCG**C****G****G****G**.GUC**A****G****C**A-GA....**U**.**C****U****G****G****U**C.C.......G.......**A**..UG.........................**C****C****A****G****A****G****C**.C.GA**C****G****G**UCA..U.A.GU................................**C****C****G**.G.AUGAA.A.**G**..**A**.**A****G****A****U****G****U****G****C** | |
|  |  | NZ\_AAHQ01000002.1/208831-208982  | **G****U****G****C****G****U****C****U****U****C**.A..GGG.C**G****G****G****G****C**GA...............A**A**AU**C****C****C****C**.ACC.G**G****C****G****G****U**AGGCCGGCAUGUUGCCGGC.........................................................................................................G**A****G****C**.**C****C****G****C**GA.**G****C****G****C****C****C****G**CGC......................................................................................................GAUU.............................GCGCG**C****G****G****G**.GUC**A****G****C**A-GA....**U**.**C****U****G****G****U**C.C.......G.......**A**..UG.........................**C****C****A****G****A****G****C**.C.GA**C****G****G**UCA..U.A.GU................................**C****C****G**.G.AUGAA.A.**G**..**A**.**A****G****A****U****G****U****G****C** | |
|  |  | NZ\_AAIQ01001356.1/805-654  | **G****U****G****C****G****U****C****U****U****C**.A..GGG.C**G****G****G****G****C**GA...............A**A**AU**C****C****C****C**.ACC.G**G****C****G****G****U**AGGCCGGCAUGUUGCCGGC.........................................................................................................G**A****G****C**.**C****C****G****C**GA.**G****C****G****C****C****C****G**CGC......................................................................................................GAUU.............................GCGCG**C****G****G****G**.GUC**A****G****C**A-GA....**U**.**C****U****G****G****U**C.C.......G.......**A**..UG.........................**C****C****A****G****A****G****C**.C.GA**C****G****G**UCA..U.A.GU................................**C****C****G**.G.AUGAA.A.**G**..**A**.**A****G****A****U****G****U****G****C** | |
|  |  | NC\_006834.1/4115343-4115168  | **G****A****A****C****G****U****C****U****U****C**.A..GGG.C**G****G****G****G****U**GC...............G**A**UU**C****C****C****C**.ACC.G**G****C****G****G****U**AGGCAUGCGCACGCAUGU..........................................................................................................G**A****G****C**.**C****C****G****C**GA.**G****C****G****C****U****U****C**GUUGCGACGCUCGGCGCU.......................................................................................GCAU..................GUGCCGGGUACCGCGC**G****A****A****G**.GUC**A****G****C**A-GA....**U**.**C****C****G****G****U**C.C.......G.......**A**..CG.........................**C****C****G****G****A****G****C**.C.GA**C****G****G**.UA..U.A.GU................................**C****C****G**.G.AUGAA.A.**G**..**A**.**A****G****A****C****G****G****C****C** | |
|  |  | NZ\_AAET01000196.1/2326-2503  | **U****C****G****C****A****U****U****C****U****C**.A..GGG.C**A****G****G****G****U**GA...............A**A**UU**C****C****C****U**.ACC.G**G****G****G****G****U**A-..........................................................................................................................A**A****G****C**.**C****C****A****C**GA.**G****C****G****U****U****U****A**AAAGUGCGGUCAAUUUUUG......................................................................................CCAAAUUUUUCCUGCGAAAUUGUCCUGAUUUUCACUUU**U****A****A****A**.GUC**A****G****C**A-GA....**U**.**U****U****G****G****U**G.A.......A.......**A**..UU.........................**C****C****A****A****A****G****C**.C.GA**C****A****G**.UA..A.A.GU................................**C****U****G**.G.AUGAA.A.**G**..**A**.**G****A****A****U****A****U****A****A** | |
|  |  | NC\_003911.11/286629-286477  | **U****G****U****C****G****U****U****C****U****C**.A..GGG.C**G****G****G****G****C**GA...............A**A**UU**C****C****C****C**.ACC.G**G****C****G****G****U**AUGCGGGGCUUGAUCCCGC.........................................................................................................C**A****G****C**.**C****C****G****C**GA.**G****C****G****G****C****C****C**CGG......................................................................................................CCCA............................CGUCCG**G****G****G****U**.GUC**A****G****C**A-GA....**U**.**C****U****G****G****U**G.A.......G.......**A**..GA.........................**C****C****A****G****A****G****C**.C.GA**C****G****G**UUA..C.A.GU................................**C****C****G**.G.AUGAA.A.**G**..**A**.**G****A****A****C****G****C****A****C** | |
|  |  | NC\_006449.1/268509-268631  | **A****A****G****G****G****U****C****U****U****C**.G..GGG.C**A****A****G****G****U**GA...............A**A**CU**C****C****C****G**.ACC.G**G****C****G****G****U**GAUUCUUUGGAU................................................................................................................A**A****G****U**.**C****C****G****C**GA.**G****C**-----.........................................................................................................GCAA..................................----....-**G****C**U-GA....**U**.**G****A****G****G****U**G.U.......A.......**A**..UU.........................**C****C****U****C****A****A****C**.C.GA**C****A****G**.UA..U.A.GU................................**C****U****G**.G.AUGGA.A.**G**..**A**.**A****G****A****C****C****A****G****U** | |
|  |  | NZ\_AADW02000002.1/254821-254677  | **C****C****A****G****U****U****C****U****U****C**.G..GGG.U**A****G****G****G****U**GA...............A**A**AU**C****C****C****A**.ACC.G**G****C****G****G****U**GAUGCGAUUUUUAAAUCGAC........................................................................................................G**A****G****C**.**C****C****G****C**GA.**C****C****C****A****U****A****G**U........................................................................................................GUAC...............................GCA**C****U****A****U**....**G****G****C**U-GA....**C**.**U****U****G****G****U**G.U.......G.......**A**..UU.........................**C****C****A****G****G****G****C**.C.GA**C****G****G**.UA..A.A.GU................................**C****C****G**.G.AUGGG.A.**G**..**A**.**A****G****A****A****C****G****A****A** | |
|  |  | NC\_006270.2/2408405-2408255  | **U****G****U****A****U****C****C****U****U****C**.G..GGG.C**U****G****G****G****U**GA...............A**A**AU**C****C****C****G**.ACC.G**G****C****G****G****U**AAUAAAGCGAGAGACAGCUUUA......................................................................................................G**A****G****C**.**C****C****G****U**GAC**C****C****G****C****U****G****U**G........................................................................................................CUUC..............................GUGC**A****A****A****G**....**C****G****G**UGGA....**U**.**U****C****A****G****U**G.A.......A.......**A**..AG.........................**C****U****G****A****A****G****C**.C.GA**C****A****G**UGA..A.A.GU................................**C****U****G**.G.AUGGG.A.**G**..**A**.**A****G****G****A****U****G****A****A** | |
|  |  | NC\_006322.1/2409268-2409118  | **U****G****U****A****U****C****C****U****U****C**.G..GGG.C**U****G****G****G****U**GA...............A**A**AU**C****C****C****G**.ACC.G**G****C****G****G****U**AAUAAAGCGAGAGACAGCUUUA......................................................................................................G**A****G****C**.**C****C****G****U**GAC**C****C****G****C****U****G****U**G........................................................................................................CUUC..............................GUGC**A****A****A****G**....**C****G****G**UGGA....**U**.**U****C****A****G****U**G.A.......A.......**A**..AG.........................**C****U****G****A****A****G****C**.C.GA**C****A****G**UGA..A.A.GU................................**C****U****G**.G.AUGGG.A.**G**..**A**.**A****G****G****A****U****G****A****A** | |
|  |  | NC\_004557.1/688281-688395  | **U****A****C****A****U****C****C****U****U****C**.A..GGG.U**U****U****G****G****U**GA...............G**A**UU**C****C****A****U**.ACC.G**G****U****G****G****U**A-..........................................................................................................................A**A****G****C**.**C****C****A****C**GA.**G****C****C**----.........................................................................................................GCAA..................................----....**G****G****C**A-GA....**U**.**C****U****G****G****U**G.A.......A.......**A**..UU.........................**C****C****A****G****G****G****C**.C.GA**C****A****G**UUA..A.A.GU................................**C****U****G**.G.AUGAA.A.**G**..**A**.**A****G****G****A****A****A****U****U** | |
|  |  | NC\_004307.2/55585-55434  | **U****G****U****C****A****G****U****U****U****C**.A..GGG.-**A****A****G****G****U**GC...............A**A**UU**C****C****U****U**.ACU.G**G****C****G****G****U**AACCUGCACCCCGGGUUUCAUAACGGGGAUGGCAGG........................................................................................A**A****G****C**.**C****C****G****C**GAC**C****C****G****C**---.........................................................................................................GCAA..................................---**G**....**C****G****G**CUGA....**U**.**C****C****G****G****U**G.A.......G.......**A**..UU.........................**C****C****G****G****A****G****C**.C.AA**C****G****G**.UA..U.A.GU................................**C****C****G**.G.AUGGA.A.**G**..**A**.**A****A****C****G****A****G****G****G** | |
|  |  | NZ\_AABM02000010.1/77749-77900  | **U****G****U****C****A****G****U****U****U****C**.A..GGG.-**A****A****G****G****U**GC...............A**A**UU**C****C****U****U**.ACU.G**G****C****G****G****U**AACCUGCACCCCGGGUUUCAUAACGGGGAUGGCAGG........................................................................................A**A****G****C**.**C****C****G****C**GAC**C****C****G****C**---.........................................................................................................GCAA..................................---**G**....**C****G****G**CUGA....**U**.**C****C****G****G****U**G.A.......G.......**A**..UU.........................**C****C****G****G****A****G****C**.C.AA**C****G****G**.UA..U.A.GU................................**C****C****G**.G.AUGGA.A.**G**..**A**.**A****A****C****G****A****G****G****G** | |
|  |  | NZ\_AADT03000004.1/77156-77271  | **A****A****C****G****U****C****C****U****U****C**.G..GGG.U**U****A****G****G****U**GU...............A**A**UU**C****C****U****U**.ACC.G**G****C****G****G****U**A-..........................................................................................................................A**A****G****C**.**C****C****G****C**GA.**G****C****C****G**---.........................................................................................................-UUA..................................---**U**....**G****G****C**A-GA....**C**.**C****C****G****G****U**G.A.......A.......**A**..UU.........................**C****C****G****G****G****G****C**.C.GA**C****A****G**UAA..A.A.GU................................**C****U****G**.G.AAGGG.A.**G**..**A**.**A****G****G****A****G****G****U****U** | |
|  |  | NZ\_AAFG02000008.1/74962-74828  | **C****G****A****U****A****U****U****C****U****C**.A..GGG.C**A****G****G****G****U**GA...............A**A**GU**C****C****C****U**.ACC.G**G****U****G****G****U**CA..........................................................................................................................C**A****G****C**.**C****C****A****C**GA.**G****C****G****G****C****C****C**UUG......................................................................................................GUUU.............................GACAA**G****G****G****U**.GAC**A****G****C**A-GA....**C**.**C****C****G****G****C**G.C.......A.......**A**..UU.........................**C****C****G****G****G****G****C**.C.GA**C****G****G**UUA..C.A.GU................................**C****C****G**.G.AUGAA.A.**G**..**A**.**G****A****A****U****G****G****A****G** | |
|  |  | NC\_006055.1/670666-670782  | **U****A****A****C****A****U****U****U****U****C**.G..GGG.C**A****A****G****G****C**GA...............A**A**UU**C****C****U****U**.ACC.G**A****U****G****G****U**AA..........................................................................................................................U**A****G****U**.**C****C****A****U**GA.**A****C****C****U**---.........................................................................................................UAAU..................................---**U**....**G****G****U**U-GA....**U**.**U****C****A****G****U**G.A.......A.......**A**..UU.........................**C****U****G****A****A****A****C**.C.GA**C****G****G**.UA..U.A.GU................................**C****C****G**.G.AUGGU.A.**G**..**A**.**A****A****A****U****G****U****C****A** | |
|  |  | NZ\_AAAK03000067.1/10080-9955  | **G****U****U****C****G****U****C****U****U****C**.A..GGGGC**A****G****G****G****U**GA...............A**A**UU**C****C****C****G**.ACC.G**G****U****G****G****U**UA..........................................................................................................................U**A****G****U**.**C****C****A****C**GA.**C****C****U****A****U****U****U**U........................................................................................................GCAA................................AA**A****A****A****U**....**A****G****C**U-GA....**A**.**U****C****G****G****U**G.A.......A.......**A**..UU.........................**C****C****G****A****U****A****C**.C.GA**C****A****G**.UA..U.A.GU................................**C****U****G**.G.AU-AA.A.**G**..**A**.**A****G****A****U****A****G****A****G** | |
|  |  | NC\_006582.1/3181483-3181616  | **A****G****G****U****A****U****C****U****U****C**.G..GGG.C**A**-**G****G****U**GA...............A**A**CU**C****C****U****G**.GCC.G**G****C****G****G****U**AA..........................................................................................................................A**A****G****C**.**C****C****G****C**GAC**U****C****U****C****C****U****U**UU.......................................................................................................CUUU...........................UAUCAAU**A****A****G****G**....**G****G****A**CUGA....**U**.**U****U****G****G****U**G.G.......A.......**A**..UU.........................**C****C****A****A****A****G****C**.C.GA**C****G****G**UUA..A.A.GU................................**C****C****G**.G.AUGGG.A.**G**..**A**.**A****G****A****U****C****A****A****C** | |
|  |  | NZ\_AAHE01000001.1/276274-276122  | **C****G****C****U****U****C****C****U****U****C**.G..GGG.C**G****G****G****G****U**GA...............A**A**UU**C****C****C****C**.ACC.G**G****C****G****G****U**GAUCCGGCUCUGGCCGGU..........................................................................................................C**A****G****C**.**C****C****G****C**GAA**G****C****C****C****G****C****G**CAAACUGCA................................................................................................CCAC.................................G**C****G****C****G**...A**G****G****C**CCGA....**C**.**U****C****C****G****U**G.C.......G.......**A**..UU.........................**C****G****G****A****G****G****C**.C.GA**C****G****G**UCA..C.A.GU................................**C****C****G**.G.AUGAG.A.**G**..**A**.**A****G****G****A****G****G****A****A** | |
|  |  | NC\_002570.2/1634615-1634762  | **U****U****U****A****U****C****C****U****U****C**.G..GGG.C**U****G****G****G****U**GG...............A**A**AU**C****C****C****G**.ACC.G**G****C****G****G****U**GAUGAAGCGAAUGCUUCU..........................................................................................................U**A****G****U**.**C****C****G****U**GAC**C****C****G****G****U****U****G**CUG......................................................................................................AUAU...............................CAG**U****A****A****G**....**C****G****G**UGGA....**C**.**C****U****G****G****U**G.A.......A.......**A**..AU.........................**C****C****G****G****G****A****C**.C.GA**C****A****G**UGA..A.A.GU................................**C****U****G**.G.AUGGG.A.**G**..**A**.**A****G****G****A****A****A****C****G** | |
|  |  | NC\_002662.1/1307881-1307745  | **A****A****A****U****A****U****C****U****U****C**.A..GGG.C**A****C****C****G****U**GU...............A**A**UU**C****G****G****G**.ACC.G**G****C****G****G****U**AAAUAGGGCUUUGACCUUAU........................................................................................................G**A****C****U**.**C****C****G****C**GAU**U****C****G****C**---.........................................................................................................UACG..................................---**G**....**C****G****A**UUGA....**A**.**G****C****A****G****U**G.A.......G.......**A**..AU.........................**C****U****G****C****U****A****G**.C.GA**C****A****G**.UA..A.A.GU................................**C****U****G**.G.AUGGA.A.**G**..**A**.**A****G****A****U****G****A****A****C** | |
|  |  | NZ\_AAGO01000042.1/12116-11980  | **A****A****A****U****A****U****C****U****U****C**.A..GGG.C**A****C****C****G****U**GU...............A**A**UU**C****G****G****G**.ACC.G**G****C****G****G****U**AAAUAAGGCUUUGACCUUAU........................................................................................................G**A****C****U**.**C****C****G****C**GAU**U****C****G****C**---.........................................................................................................UACG..................................---**G**....**C****G****A**UUGA....**A**.**G****C****A****G****U**G.A.......A.......**A**..CU.........................**C****U****G****C****U****A****G**.C.GA**C****A****G**.UA..A.A.GU................................**C****U****G**.G.AUGGA.A.**G**..**A**.**A****G****A****U****G****A****A****C** | |
|  |  | NC\_006582.1/1930361-1930511  | **A****C****U****C****A****A****C****U****U****C**.G..GGG.C**A****G****G****G****U**GU...............A**A**AU**C****C****C****G**.ACC.G**G****C****G****G****U**GAUAAGGCAAUCGCCUUU..........................................................................................................U**A****G****U**.**C****C****G****U**GAC**C****C****G****C****A****U****A**A........................................................................................................GCAU..........................UACUCGUG**C****G****U****G**....**C****G****G**UGGA....**U**.**C****U****G****G****U**G.C.......A.......**A**..AU.........................**C****C****A****G****A****G****C**.C.GA**C****A****G**UAA..U.A.GU................................**C****U****G**.G.AUGGG.A.**G**..**A**.**A****G****U****U****C****G****A****G** | |
|  |  | NZ\_AAHJ01000001.1/15775-15623  | **C****U****U****G****U****U****U****C****U****C**.A..GGG.C**G****G****G****G****U**GC...............A**A**GU**C****C****C****C**.ACC.G**G****C****G****G****U**AUUCCGGGUACGUAUCCGGA........................................................................................................G**A****G****C**.**C****C****G****C**GA.**G****C****G****C****U****U****U**CGCU.....................................................................................................GCAA..............................GGCG**G****A****A****G**.GUC**A****G****C**A-GA....**U**.**U****U****G****G****U**G.C.......A.......**A**..AG.........................**C****C****A****A****A****G****C**.C.GA**C****G****G**UCA..U.A.GU................................**C****C****G**.G.ACGAA.A.**G**..**A**.**G****A****A****U****G****A****G****A** | |
|  |  | NC\_004668.1/1495313-1495432  | **G****U****U****C****G****U****C****U****U****C**.A..GGGGC**A****G****G****G****U**GU...............A**A**UU**C****C****C****G**.ACC.G**G****U****G****G****U**UA..........................................................................................................................U**A****G****U**.**C****C****A****C**GAC**U****C****G****U****U**--.........................................................................................................-UUU..................................--**A****A**....**C****G****A**UUGA....**A**.**U****U****G****G****U**G.U.......A.......**A**..UU.........................**C****C****A****A****U****A****C**.C.GA**C****A****G**.UA..U.A.GU................................**C****U****G**.G.AU-AA.A.**G**..**A**.**A****G****A****U****A****G****G****G** | |
|  |  | NC\_000964.2/2430811-2430667  | **U****U****G****U****A****U****C****U****U****C**.G..GGG.C**A****G****G****G****U**GG...............A**A**AU**C****C****C****G**.ACC.G**G****C****G****G****U**AGUAAAGCACAUUUGCUUUA........................................................................................................G**A****G****C**.**C****C****G****U**GAC**C****C****G****U****G****U****G**C........................................................................................................AUAA.................................G**C****A****C****G**....**C****G****G**UGGA....**U**.**U****C****A****G****U**U.U.......A.......**A**..-G.........................**C****U****G****A****A****G****C**.C.GA**C****A****G**UGA..A.A.GU................................**C****U****G**.G.AUGGG.A.**G**..**A**.**A****G****G****A****U****G****A****U** | |
|  |  | NC\_001263.1/153631-153779  | **G****A****C****C****U****C****U****U****U****C**.G..GGG.C**G****G****G****G****C**GA...............A**A**UU**C****C****C****C**.ACC.G**G****C****G****G****U**AAGUUCUCCCGAAC..............................................................................................................A**A****G****C**.**C****C****G****C**GAA**G****C****C****C****G****C****G**CAAACC...................................................................................................GCAC................................CA**C****G****C****G**.CCG**G****G****C**CCGA....**U**.**G****C****C****G****C**G.C.......A.......**A**..CU.........................**C****G****G****C****A****G****C**.C.GA**C****G****G**UCA..C.A.GU................................**C****C****G**.G.ACGAA.A.**G**..**A**.**A****G****G****A****G****G****A****G** | |
|  |  | NZ\_AAAW03000004.1/12009-11860  | **A****C****G****A****A****C****C****U****U****C**.G..AGG.U**A****G****G****G****U**GA...............A**A**UU**C****C****C****G**.ACC.G**G****C****G****G****U**AAUGAUUGCCCGUGCAAUU.........................................................................................................C**A****G****C**.**C****C****G****C**AA.**C****U****C****A****G****U****G**UAGC.....................................................................................................GCCA............................GUGCUG**C****A****C****C**....**G****A****C**U-GA....**C**.**U****U****G****G****U**G.A.......G.......**A**..CU.........................**C****C****A****A****G****G****C**.C.GA**C****G****G**.UA..U.A.GU................................**C****C****G**.G.AUGGG.A.**G**..**A**.**A****G****G****U****A****C****A****A** | |
|  |  | NZ\_AAEB02000032.1/17972-17823  | **G****G****U****A****A****C****U****U****U****C**.G..GGG.G**C****G****G****G****U**GG...............A**A**UU**C****C****C****G**CACC.G**G****C****G****G****U**GAGCCCGGGUCCAGCGCGCCCGGG....................................................................................................G**A****G****C**.**C****C****G****C**GAC**C****C****C****C****C****G****C**C........................................................................................................GCA-.................................G**G****C****G****G**....**G****G****G**UGGA....**G**.**C****C****G****G****U**G.A.......G.......**A**..GU.........................**C****C****G****G****C****G****C**.C.GA**C****G****G**UGA..U.A.GU................................**C****C****G**.G.AUGGG.A.**G**..**A**.**A****A****G****G****A****A****G****C** | |
|  |  | NC\_003888.3/1539404-1539276  | **C****A****G****C****G****C****A****C****U****C**.C..GGG.G**U****C****G****G****U**GA...............A**A**GU**C****C****G****A**.ACC.G**G****C****G****G****U**UA..........................................................................................................................C**A****G****U**.**C****C****G****C**GAC**C****C****G****G****U****C****G**CC.......................................................................................................UCCA.................................G**C****G****A****C**....**C****G****G**UUGA....**C**.**C****A****G****G****U**G.A.......A.......**A**..UU.........................**C****C****U****G****G****A****C**.C.GA**C****G****G**UUA..A.A.GU................................**C****C****G**.G.AUGGG.A.**G**..**G**.**C****A****G****U****G****C****G****C** | |
|  |  | NZ\_AAEV01000002.1/120578-120702  | **G****U****U****C****A****U****C****U****U****C**.AAGGGG.C**A****G****G****G****U**GC...............G**A**UU**C****C****C****G**.ACC.G**G****U****G****G****U**A-..........................................................................................................................A**A****G****U**.**C****C****A****U**GA.**G****C****U****G****U****C****A**.........................................................................................................UUCG..................................**U****G****A****U**....**G****G****C**U-GA....**U**.**U****C****G****G****U**G.A.......A.......**A**..UU.........................**C****C****G****A****A****A****C**.C.GA**U****A****G**UUA..A.A.GU................................**C****U****A**.G.AUAAA.G.**G**..**A**.**A****G****A****U****U****G****A****A** | |
|  |  | NZ\_AAIE01000060.1/930-1059  | **A****C****G****C****G****C****G****C****U****C**.C..GGG.G**U****C****G****G****U**GA...............A**A**UU**C****C****G****A**.ACC.G**G****C****G****G****U**GA..........................................................................................................................C**A****G****U**.**C****C****G****C**GA.**C****C****C****G****U****C****C**GCAG.....................................................................................................GUAC................................UG**G****G****A****C**....**G****G****C**U-GA....**C**.**C****C****G****G****U**G.G.......A.......**A**..UU.........................**C****C****G****G****G****A****C**.C.GA**C****G****G**UGA..A.A.GU................................**C****C****G**.G.AUGGG.A.**G**..**G**.**A****C****G****C****G****C****G****C** | |
|  |  | NZ\_AAGP01000003.1/53843-53971  | **C****A****C****A****U****G****U****U****C****C**.G..GGG.-**A****C****G****G****U**GA...............A**A**AU**C****C****G****U**.ACC.G**G****C****G****G****U**GA..........................................................................................................................U**A****G****U**.**C****C****G****C**GAG**C****C****G****G****C****U****G**C........................................................................................................AUUC...............................GUG**C****A****G****A**....**U****G****G**UUGA....**C**.**U****U****G****G****U**G.A.......A.......**A**..UU.........................**C****C****G****A****G****A****C**.C.GA**C****A****G**UCA..G.A.GU................................**C****U****G**.G.AUGGG.A.**G**..**G**.**A****G****C****A****U****G****G****C** | |
|  |  | NC\_003155.3/8236206-8236333  | **C****A****G****C****G****C****A****C****U****C**.C..GGG.G**U****C****G****G****U**GA...............A**A**GU**C****C****G****A**.ACC.G**G****C****G****G****U**UA..........................................................................................................................U**A****G****U**.**C****C****G****C**GAC**C****C****G****C****U****C****G**C........................................................................................................CUCG.................................G**C****G****A****U**....**C****G****G**UUGA....**C**.**C****A****G****G****U**G.G.......A.......**A**..UU.........................**C****C****U****G****G****A****C**.C.GA**C****G****G**UUA..A.A.GU................................**C****C****G**.G.AUGGG.A.**G**..**G**.**C****A****G****U****G****C****G****C** | |
|  |  | NC\_006371.1/1039643-1039458  | **C****U****G****A****A****U****U****C****U****C**.A..GGG.C**G****G****G****G****C**GC...............A**A**UU**C****C****C****C**.ACC.G**G****C****G****G****U**AAUUCAGAGCACUCAAUAGUGAGUUAACUGAA............................................................................................A**A****G****C**.**C****C****G****C**GA.**G****C****G****C****U****U****A**U........................................................................................................CUUU........................UUUAUAAUGA**U****A****A****G**.GUC**A****G****C**A-GA....**U**.**C****U****G****G****U**G.U.......A.......**A**..U-ACUUCCUGUCAGUAUGAGU......**C****C****A****G****A****G****C**.C.GA**C****G****G**UUA..U.A.GU................................**C****C****G**.G.AUGAA.A.**G**..**A**.**G****G****A****U****C****A****G****U** | |
|  |  | NZ\_AAAW03000198.1/2512-2629  | **U****G****A****A****U****A****C****C****U****C**UG..GGG.A**U****U****G****G****U**GA...............A**A**UU**C****C****A****U**.ACCCG**G****C****G****G****U**C-..........................................................................................................................A**A****G****C**.**C****C****G****C**GA.**G****C****C****A**---.........................................................................................................-UUA..................................---**U**....**G****G****C**A-GA....**U**.**C****U****G****G****U**G.U.......A.......**A**..UU.........................**C****C****A****G****A****G****C**.C.GA**C****A****G**.UA..A.A.GU................................**C****U****G**.G.AUGAG.AA**G**..**A**.**G****G****A****A****A****U****U****A** | |
|  |  | NC\_004567.1/1706025-1705904  | **G****U****U****C****A****U****C****U****U****C**.A..GGG.C**A****G****G****G****U**GU...............G**A**UU**C****C****C****G**.ACC.G**G****C****G****G****U**AA..........................................................................................................................U**A****A****C**.**C****C****G****C**GAC**C****U****A****U****G****C**-.........................................................................................................GUGU..................................-**G****C****A**....**U****A****G**U-GA....**U**.**U****U****G****G****U**G.C.......A.......**A**..UU.........................**C****C****A****A****A****G****U**.C.GA**C****A****G**.UA..A.A.GU................................**C****U****G**.GUAU-AA.A.**G**..**A**.**A****G****A****U****C****G****A****G** | |
|  |  | NZ\_AAEF02000025.1/29234-29360  | **A****C****G****C****G****U****G****C****U****C**.C..GGG.G**U****C****G****G****U**GC...............A**A**UU**C****C****G****A**.ACC.G**G****C****G****G****U**GA..........................................................................................................................C**A****G****U**.**C****C****G****C**GA.**G****C****C****C****C****C****G**C........................................................................................................UUCG.................................G**C****G****G****G**...U**G****G****U**U-GA....**U**.**C****C****G****G****U**G.G.......A.......**A**..CU.........................**C****C****G****G****G****A****C**.C.GA**C****G****G**UGA..G.A.GU................................**C****C****G**.G.AUGGG.A.**G**..**G**.**C****A****G****C****G****C****G****C** | |
|  |  | NZ\_AAHG01000012.1/83108-82954  | **C****U****A****C****G****U****G****C****U****C**.C..GGG.G**U****C****G****G****U**GU...............A**A**GU**C****C****G****A**.ACC.G**G****C****G****G****U**GA..........................................................................................................................C**A****G****U**.**C****C****G****C**GAC**C****C****G****C****G****A****G**CCGGCACAGAUC.............................................................................................CUCU......................CCAGGAACUGUU**C****C****G****G**CGGA**C****G****G**UUGA....**A**.**C****C****G****G****U**G.A.......A.......**A**..UU.........................**C****C****G****G****U****A****C**.C.GA**C****A****G**UUA..A.A.GU................................**C****U****G**.G.AUGAG.A.**G**..**A**A**G****C****A****C****G****U****A****C** | |
|  |  | NC\_006085.1/1913787-1913653  | **A****C****A****C****G****U****G****C****U****C**.C..GGG.G**U****C****G****G****U**GA...............A**A**GU**C****C****G****A**.ACC.G**G****C****G****G****U**GA..........................................................................................................................U**A****G****U**.**C****C****G****C**GAC**C****C****G****A****G****C****C**G........................................................................................................UCCU............................CGUGGC**G****G****U****G**....**A****G****G**UUGA....**A**.**U****C****G****G****U**G.A.......A.......**A**..UU.........................**C****C****G****G****U****A****C**.C.GA**C****G****G**UAA..A.A.GU................................**C****C****G**.G.AUGGG.A.**G**GU**A**.**G****C****A****C****G****G****A****A** | |
|  |  | NZ\_AAAQ02000011.1/30579-30709  | **A****C****G****C****G****U****G****C****U****C**.C..GGG.G**U****C****G****G****U**GA...............A**A**GU**C****C****G****A**.ACC.G**G****C****G****G****U**GA..........................................................................................................................C**A****G****U**.**C****C****G****C**GAC**C****C****A****G****U****C****G**AA.......................................................................................................GCCA.................................U**C****G****G****C**....**U****G****G**UGGA....**A**.**C****C****G****G****U**G.A.......A.......**A**..CU.........................**C****C****G****G****U****A****C**.C.GA**C****G****G**UGA..A.A.GU................................**C****C****G**.G.AUGGG.A.**G**GU**A**.**G****U****A****C****G****U****G****G** | |
|  |  | NZ\_AABH02000065.1/4916-5052  | **U****A****A****U****A****A****C****U****U****C**.A..GGG.U**C****G****G****G****U**GU...............A**A**GU**C****C****C****A**.ACC.G**G****C****G****G****U**GAGACAUGAAUUUUCAUGGU........................................................................................................A**A****G****C**.**C****C****G****C**GA.**C****C****U****G****C**--.........................................................................................................UUUU..................................--**G****C**....**A****G****C**U-GA....**U**.**C****U****G****G****U**C.A.......A.......**A**..AU.........................**C****C****A****G****A****G****C**.C.GA**C****C****G**.UA..A.A.GU................................**C****G****G**.GUAU-AA.A.**G**..**A**.**A****G****U****C****U****A****A****A** | |
|  |  | NZ\_AAAJ03000006.1/451137-450859  | **G****U****G****C****G****U****C****U****U****C**.A..GGG.C**G****G****G****G****U**GA...............A**A**UU**C****C****C****C**.ACC.G**G****C****G****G****U**AUGCCGGCAACGCGAGAGCGUGAGCCGGU...............................................................................................G**A****G****C**.**C****C****G****C**GA.**G****C****G****C****C****C****G**CGUCGUCAAGGUGUUUGGACCUGUGCAACUGCGUUGCACGGGACCCGAGUCAGUGCUUUGCAUGGGACCGGAGUAAUGCGCCAAAGCGCGAACUCCGGCCAACGGCUAA..............AGCGCCAACUCUGGUCGACA**U****G****G****G**.GUC**A****G****C**A-GA....**U**.**C****U****G****G****U**G.A.......G.......**A**..AG.........................**C****C****A****G****A****G****C**.C.GA**C****G****G**UUA..G.A.GU................................**C****C****G**.G.AUGGA.A.**G**..**A**.**A****G****A****U****G****U****G****C** | |
|  |  | NC\_005362.1/991108-991219  | **U****G****U****U****G****U****C****U****U****C**.A..GGG.C**A****G****G****G****C**GA...............A**A**UU**C****C****C****G**.ACC.G**G****C****G****G****U**UA..........................................................................................................................G**A****G****U**.**C****C****G****C**GA.**G****C****U****A****C**--.........................................................................................................ACAU..................................--**G****U**....**A****G****U**U-GA....**A**.**C****C**----.-.......-.......-..-U.........................**C****A****G****G****U****A****C**.C.GA**U****A****G**UUA..U.A.GU................................**C****U****A**.G.AUGGA.A.**G**..**A**.**A****G****A****C****A****G****A****C** | |
|  |  | NZ\_AAAO02000007.1/2328-2218  | **U****G****U****U****G****U****C****U****U****C**.A..GGG.C**A****G****G****G****U**GC...............A**A**UU**C****C****C****G**.ACC.G**G****C****G****G****U**UA..........................................................................................................................G**A****G****U**.**C****C****G****C**GA.**G****C****U****A****C**--.........................................................................................................-GAU..................................--**G****U**....**A****G****U**U-GA....**A**.**C****C**----.-.......-.......-..-U.........................**C****A****G****G****U****A****C**.C.GA**U****A****G**UUA..A.A.GU................................**C****U****A**.G.AUGGA.A.**G**..**A**.**A****G****A****C****A****G****A****C** | |
|  |  | NZ\_AAII01000114.1/6313-6183  | **A****C****G****C****G****C****G****C****U****C**.C..GGG.G**U****C****G****G****U**GA...............A**A**CU**C****C****G****A**.GCC.G**G****C****G****G****U**GA..........................................................................................................................U**A****G****U**.**C****C****G****C**GAC**C****C****G****U****C****C****G**C........................................................................................................CCUU..............................GUGG**C****G****G****C**....**C****G****G**UUGA....**C**.**C****U****G****G****U**G.G.......A.......**A**..UU.........................**C****C****G****G****G****A****C**.C.GA**C****G****G**UGA..A.A.GU................................**C****C****G**.G.AUGGG.A.**G**..**G**.**A****C****G****C****G****C****G****C** | |
|  |  | NC\_006814.1/939724-939836  | **U****G****U****U****G****U****C****U****U****C**.A..GGG.C**A****G****G****G****U**GA...............A**A**AU**C****C****C****G**.ACC.G**G****C****G****G****U**UA..........................................................................................................................G**A****G****U**.**C****C****G****C**GAC**C****C****A****C**---.........................................................................................................GUAA..................................---**G**....**U****G****G**UUGA....**A**.**U****C****G**---.-.......-.......-..UU.........................-**A****G****A****U****A****C**.C.GA**U****G****G**UUA..U.A.GU................................**C****U****A**.G.AUGGA.A.**G**..**A**.**A****G****A****C****A****G****A****C** | |
|  |  | NC\_006908.1/318798-318911  | **U****G****A****G****U****C****U****U****U****C**.A..GGG.A**C****A****U****G****U**UU...............A**A**UU**C****A****G****U**.ACC.G**G****U****A****G****U**AA..........................................................................................................................A**A****G****U**.**C****U****A****C**GA.**G****C****A**----.........................................................................................................-AAA..................................----....**A****G****C**U-GA....**U**.**U****C****U****G****U**G.U.......A.......**A**..UU.........................**C****A****G****A****A****A****C**.C.GA**C****G****G**.UA..A.A.GU................................**C****C****G**.G.AUGAG.A.**A**..**A**.**A****A****G****A****A****A****G****A** | |
|  |  | NZ\_AAGQ01000020.1/2199-2084  | **A****G****U****U****G****U****C****U****U****C**.G..GGG.C**A****G****G****G****U**GA...............A**A**AU**C****C****C****G**.ACC.G**G****C****G****G****U**GC..........................................................................................................................A**A****G****U**.**C****C****G****C**GA.**G****C****C****A****C**--.........................................................................................................GCGA..................................--**G****U**....**G****G****U**U-GA....**A**.**U****G****C**-**U**G.U.......A.......**A**..AG.........................---**C****U****A****C**.C.GA**U****A****G**UUA..U.A.GU................................**C****U****A**.G.AUGGG.A.**G**..**A**.**A****G****G****C****A****G****A****C** | |
|  |  | NZ\_AAIV01000010.1/89114-89283  | **U****A****U****A****A****U****U****C****U****C**.A..GGG.C**G****G****G****G****C**GC...............A**A**UU**C****C****C****C**.ACC.G**G****C****G****G****U**AAACUUGAAAAAGU..............................................................................................................G**A****G****C**.**C****C****G****C**GA.**G****C****G****C****U****C****G**A........................................................................................................UUCG.................................U**C****G****A****G**.GUC**A****G****C**A-GA....**U**.**C****U****G****G****U**G.ACUUA...G.......**G**..UUUUACUCAUGAGUAAUGCCUAACAGU**C****C****A****G****A****G****C**.C.GA**C****G****G**UUA..C.A.GU................................**C****C****G**.G.AUGAG.A.**G**..**A**.**G****A****A****U****A****G****A****G** | |
|  |  | NZ\_AAAO02000005.1/92035-91922  | **A****A****U****G****U****U****C****U****U****C**.G..AGA.C**A****G****G****G****U**GA...............A**A**UU**C****C****C****G**.ACC.G**G****C****G****G****U**A-..........................................................................................................................A**A****G****U**.**C****C****G****C**AAC**C****C****G****C**---.........................................................................................................GCAA..................................---**G**....**C****G****G**UUGA....**A**.**C****C****C**-**U**U.A.......A.......-..-U.........................--**G****G****U****A****C**.C.GA**U****A****G**UUA..U.A.GU................................**C****U****A**.G.AUGGA.A.**G**..**A**.**A****G****G****A****U****A****A****U** | |
|  |  | NC\_006177.1/2924773-2924949  | **G****A****G****C****G****C****C****U****U****C**.A..GGG.C**A****G****G****G****U**GAGGCGGCACUGCCGCCA**A**UU**C****C****C****G**.ACC.G**G****C****G****G****U**GAUGCUCCCCGCGGGGCGGAGGAGAC..................................................................................................G**A****G****C**G**C****C****G****C**GA.**G****C**-**C****C****C****G**UGCGGC...................................................................................................CUCA..........................GGGCGGCA**C****G****G****G**....**U****G****C**A-GA....**C**U**C****C****G****G****U**G.A.......G.......**A**..UC.........................**C****C****G****G****G****G****C**.C.GA**C****G****G**.UA..C.A.GU................................**C****C****G**.G.AUGGG.A.**G**..**A**.**A****G****G****C****G****A****G****A** | |
|  |  | NZ\_AABF02000227.1/9-120  | **U****A****G****G****C****G****A****A****U****C**.G..AGG.C**A****G****G**-**U**GA...............A**A**UC-**C****C****G**.ACC.G**G****U****G****G****U**--..........................................................................................................................C**A****G**-.**C****C****A****C**GA.**A****A****G****C**---.........................................................................................................AUUU..................................---**G**....**C****U****U**U-GA....**U**.**U****U****G****G****U**G.A.......A.......**A**..UU.........................**C****C****A****A****A****A****C**.C.GA**C****A****G**.UA..G.A.GU................................**C****U****G**.G.AUGGG.A.**G**..**A**.**A****G****A****A****U****U****A****G** | |
|  |  | NZ\_AABG04000003.1/43741-43574  | **A****C****U****U****A****U****C****U****U****C**.A..GGG.A**U****A****G****G****U**GA...............A**A**UU**C****C****U****U**.ACC.G**G****C****G****G****U**AGACAGCAUGGUAUUUGCUGU.......................................................................................................G**A****G****C**.**C****C****G****C**GA.**G****C****C**----.........................................................................................................UUUU..................................---**U**....**G****G****C**A-GA....**U**.**C****C****G****G****U**C.A.......A.......**A**..GU.........................**C****C****G****G****A****G****C**.C.GA**C****A****G**UGA..A.AUGUAUUAAUAUGUUUUGUUAUUAAUACAUUGAGU.**C****U****G**.G.AUGGG.A.**G**..**A**.**A****G****A****U****A****A****A****G** | |
|  |  | NZ\_AAIN01000001.1/58663-58491  | **U****U****A****C****A****U****U****C****U****C**.A..GGG.C**G****G****G****G****U**GA...............A**A**UU**C****C****C****C**.ACC.G**G****C****G****G****U**GAAGAGGCAUCUCUGGAUGCCACA....................................................................................................A**A****G****C**.**C****C****G****C**GA.**G****C****G****C****C****U****G**CGA......................................................................................................GCAU.........................GGUUUUGCG**C****A****G****G**.GUC**A****G****C**A-GA....**U**.**C****C****G****G****U**G.AUCCCGUU-AACGGGU**A**..UU.........................**C****C****G****G****A****G****C**.C.GA**C****G****G**.UG..U.U.GU................................**C****C****U**.G.GUGCA.G.**G**..**A**.**A****U****U****C****A****U****C****C** | |
|  |  | NC\_004347.1/151287-151051  | **A****A****C****A****A****U****U****C****U****C**.A..GGG.C**G****G****G****G****U**GA...............A**A**CU**C****C****C****C**.ACC.G**G****C****G****G****U**AAAUAGUGAGGUAAAAGAGAUUUUCCCACGCUA...........................................................................................A**A****G****C**.**C****C****G****C**GA.**G****C****G****C****C****C****G**AACGCAAUUUCGGGGUCAGCAGAUCUGGUGCCCUAGCAUGUCUCGCCAUGGUUAACGCCAAACGAGACCGCG.................................CUAU..............................UAUU**C****C****A****G**....**A****G****C**C-GACGGU**A**.**A****U****G****G****A**C.U.......G.......**U**..UUG........................**C****C****A****C****U****A****G**.C.GA**C****A****G**.UACUG.A.GU................................**C****C****G**.G.AUGGA.A.**G**..**A**.**G****A****A****U****G****U****A****A** | |
|  |  | NZ\_AAIO01000007.1/50592-50821  | **G****C****A****A****A****U****U****C****U****C**.A..GGG.C**G****G****G****G****U**GA...............A**A**CU**C****C****C****C**.ACC.G**G****C****G****G****U**AAAUGGGUAACGCUAAGUGAUUAUUUAACUGAGCAUCCCCA...................................................................................A**A****G****C**.**C****C****G****C**GA.**G****C****G****C****C****C****G**AAAUCACUUUCGGGGUCAGCAGAUCUGGUGCCUUAGAUUUUGCCUAGUGCAAAACUGA...............................................CUAU.................................U**C****C****A****G**....**A****G****C**C-GA....**C**G**G****U****A****A****U**G.G.......A.......**C**..UGUUUGCU...................**U****U****A****A****U****G****G**.C.GA**C****A****G**.UACUG.A.GU................................**C****C****G**.G.AUGGA.A.**G**..**A**.**G****A****A****U****G****U****A****A** | |
|  |  | NC\_006087.1/440484-440331  | **A****U****A****C****G****U****G****C****U****C**.C..GGG.G**U****C****G****G****U**GA...............G**A**GU**C****C****G****A**.ACC.G**G****C****G****G****U**GA..........................................................................................................................C**A****G****U**.**C****C****G****C**GA.**G****C****G****G****A****G****G**GU.......................................................................................................GAGA................................GC**C****C****G****C**....**U****G****C**U-GA....**A**.**C****C****G****G****U**G.G.......A.......**A**..UU.........................**C****C****G****G****U****G****C**.C.GA**C****G****G**.UA..A.U.G-CGUCCAGGUUCCGCCCUGGCGCUCAGU.....**C****C****G**.G.AUGGG.A.**G**..**G**C**G****C****A****C****G****U****C****G** | |
|  |  | AACY01245701.1/598-749  | **G****C****U****U****A****U****U****C****U****C**.A..GGG.C**G****G****G****G****C**GA...............A**A**UU**C****C****C****C**.ACC.G**G****C****G****G****U**AAAUCAACCAUAGCGUUGA.........................................................................................................A**A****G****C**.**C****C****G****C**GA.**G****C****G****C****U****U****U**CUGU.....................................................................................................GAAA..............................GCAG**A****A****A****G**.GUC**A****G****C**A-GA....**U**.**C****C****G****G****U**G.U.......A.......**A**..UU.........................**C****C****G****G****G****G****C**.C.GA**C****G****G**UUA..A.A.GU................................**C****C****G**.G.AUGGG.A.**G**..**A**.**G****A****G****U****A****A****C****G** | |
|  |  | AACY01025006.1/156-305  | **G****C****U****U****A****U****U****C****U****C**.A..GGG.C**G****G****G****G****C**GA...............A**A**UU**C****C****C****C**.ACC.G**G****C****G****G****U**AAAUCAACUGAUGUUGA...........................................................................................................A**A****G****C**.**C****C****G****C**GA.**G****C****G****C****U****U****U**UU.......................................................................................................GUUA............................CGGCAA**A****A****A****G**.GUC**A****G****C**A-GA....**U**.**C****C****G****G****U**G.U.......A.......**A**..UU.........................**C****C****G****G****G****G****C**.C.GA**C****G****G**UUA..G.A.GU................................**C****C****G**.G.AUGGG.A.**G**..**A**.**G****A****G****U****A****A****C****G** | |
|  |  | AACY01026190.1/815-972  | **G****C****U****U****A****U****U****C****U****C**.A..GGG.C**G****G****G****G****U**GA...............A**A**UU**C****C****C****C**.ACC.G**G****C****G****G****U**AUCCCGAUGUGACUCGGG..........................................................................................................G**A****G****C**.**C****C****G****C**GA.**G****C****G****C****C****C****G**ACCCG....................................................................................................GUAU........................UGCACCGGGA**C****G****G****G**.GUC**A****G****C**A-GA....**U**.**C****C****G****G****U**G.A.......G.......**A**..UG.........................**C****C****G****G****A****G****C**.C.GA**C****G****G**UCA..U.A.GU................................**C****C****G**.G.AUGAA.A.**G**..**A**.**G****G****A****U****A****A****G****A** | |
|  |  | AACY01174362.1/107-258  | **G****C****U****U****A****U****U****C****U****C**.A..GGG.C**G****G****G****G****C**GA...............A**A**UU**C****C****C****C**.ACC.G**G****C****G****G****U**AAAUCAACCAUAGCGUUGA.........................................................................................................A**A****G****C**.**C****C****G****C**GA.**G****C****G****C****U****U****U**CUGC.....................................................................................................GAAU..............................GCAG**A****A****A****G**.GUC**A****G****C**A-GA....**U**.**C****C****G****G****U**G.U.......A.......**A**..UU.........................**C****C****G****G****G****G****C**.C.GA**C****G****G**UUA..G.A.GU................................**C****C****G**.G.AUGGG.A.**G**..**A**.**G****A****G****U****A****A****C****G** | |
|  |  | AAFY01011024.1/855-707  | **U****G****G****A****A****U****U****C****U****C**.A..GGG.C**G****G****G****G****U**GA...............A**A**UU**C****C****C****C**.ACC.G**G****C****G****G****U**GAUUUUGAUGCAGUCAAAU.........................................................................................................A**A****G****C**.**C****C****G****C**GA.**G****C****G****C****C****U****G**UUG......................................................................................................CUUU................................AA**C****A****G****G**.GUC**A****G****C**A-GA....**U**.**C****U****G****G****U**G.A.......A.......**A**..CU.........................**C****C****A****G****A****G****C**.C.GA**C****G****G**UGA..A.A.GU................................**C****C****G**.G.AUGGG.A.**G**..**A**.**G****A****A****C****A****A****C****A** | |
|  |  | AACY01217512.1/622-467  | **G****C****C****G****G****U****U****C****U****C**.A..GGG.C**G****G****G****G****U**GC...............A**A**UU**C****C****C****C**.ACC.G**G****C****G****G****U**AAGCGCGAAAGCGU..............................................................................................................A**A****G****C**.**C****C****G****C**GA.**G****C****G****C****C****U****U**UGCGG....................................................................................................CCAA......................GUCAUUGGAGCA**A****A****G****G**.GUC**A****G****C**A-GA....**U**.**C****U****G****G****U**G.C.......G.......**A**..CU.........................**C****C****A****G****A****G****C**.C.GA**C****G****G**UCA..U.A.GU................................**C****C****G**.G.AUGGA.A.**G**..**A**.**G****A****A****C****G****A****C****A** | |
|  |  | AACY01057788.1/168568-168716  | **G****U****G****C****G****U****C****U****U****C**.A..GGG.C**G****G****G****G****U**GA...............A**A**UU**C****C****C****C**.ACC.G**G****C****G****G****U**AGGCUGGCGUAAGCCGGC..........................................................................................................G**A****G****C**.**C****C****G****C**GA.**G****C****G****C****C****C****G**C........................................................................................................GCAU.............................CCGCG**C****G****G****G**.GUC**A****G****C**A-GA....**U**.**C****U****G****G****U**C.G.......A.......**A**..UG.........................**C****C****A****G****A****G****C**.C.GA**C****G****G**UCA..C.A.GU................................**C****C****G**.G.AUGAG.A.**G**..**A**.**A****G****A****U****G****U****G****C** | |
|  |  | AAGA01005017.1/469-316  | **A****C****G****U****A****U****U****C****U****C**.A..GGG.C**G****G****G****G****U**GC...............A**A**UU**C****C****C****C**.ACC.G**G****C****G****G****U**AUGUGGCCUUGCGCCAC...........................................................................................................C**A****G****C**.**C****C****G****C**GA.**G****C****G****C****A****A****C**CACC.....................................................................................................CCAA..........................ACAGGGCG**G****U****U****G**.GUC**A****G****C**A-GA....**U**.**C****U****G****G****U**A.A.......G.......**A**..UG.........................**C****C****A****G****A****G****C**.C.GA**C****G****G**UCA..U.A.GU................................**C****C****G**.G.AUGGA.A.**G**..**A**.**G****A****A****U****G****C****G****U** | |
|  |  | AACY01312162.1/559-405  | **U****G****C****U****A****A****C****U****U****C**.A..GGG.C**G****G****G****G****U**GA...............A**A**CU**C****C****C****C**.ACC.G**G****C****G****G****U**GAUUGUGAACUUAACCAUUCGCAC....................................................................................................A**A****G****C**.**C****C****G****C**GA.**G****C****G****C****U****G****U**UA.......................................................................................................GAAA..............................CCUA**A****C****A****G**.GUC**A****G****C**A-GA....**U**.**C****U****G****G****U**G.U.......A.......**A**..CU.........................**C****C****A****G****A****G****C**.C.GA**C****G****G**UUA..U.A.GU................................**C****C****G**.G.AUGCA.A.**G**..**A**.**A****G****U****A****U****U****A****A** | |
|  |  | AAFX01111890.1/551-403  | **A****C****U****U****A****U****U****C****U****C**.A..GGU.C**G****G****G****G****U**GA...............A**A**AU**C****C****C****C**.ACC.G**G****C****G****G****U**AUCCCAGGUUAUACUGGG..........................................................................................................G**A****G****C**.**C****C****G****C**GA.**G****C****G****C****U****C****C**UUC......................................................................................................GAUU...............................GAU**G****G****A****G**.GUC**A****G****C**A-GA....**U**.**C****U****G****G****U**G.A.......A.......**A**..AA.........................**C****C****A****G****A****G****C**.C.GA**C****G****G**UCA..C.A.GU................................**C****C****G**.G.AUUGA.A.**G**..**A**.**G****G****A****U****A****A****G****G** | |
|  |  | AACY01591319.1/56-198  | **C****A****U****A****G****U****C****C****U****C**.G..GGG.U**U****G****G****G****U**GA...............A**A**AU**C****C****C****U**.ACC.G**G****C****G****G****U**GACCUGUUAAUAACAG............................................................................................................C**A****G****C**.**C****C****G****C**GA.**C****C**-**C****U****U****G**U........................................................................................................GAAA............................AUUGAU**C****A****A****G**....-**G****U**U-GA....**U**.**C****A****G****G****U**G.A.......A.......**A**..UU.........................**C****C****U****G****A****G****C**.C.GA**C****G****G**UAA..G.A.GU................................**C****C****G**.G.AUGGG.A.**G**..**A**.**G****G****A****U****G****U****A****A** | |
|  |  | AACY01172042.1/156-331  | **C****G****G****C****A****U****C****U****U****C**.A..GGG.C**A****G****A****G****U**GG...............A**A**UU**C****U****C****U**.ACC.G**G****U****G****G****U**GAUUUGCACUUAUUUAAAUUAAAAUAAUAUUAAAGAAGAGGCAAU...............................................................................C**A****G****C**.**C****C****A****C**GA.**G****C****G****C****U****A****A**CA.......................................................................................................AUAU..............................CAUG**U****U****A****G**.GUC**A****G****C**A-GA....**U**.**C****U****G****G****U**G.U.......C.......**A**..AU.........................**C****C****A****G****A****G****C**.C.GA**C****G****G**UAA..C.A.GU................................**C****C****G**.G.AUGAA.A.**G**..**A**.**A****G****A****U****C****A****A****C** | |
|  |  | AAFZ01027387.1/37-164  | **A****U****C****C****A****U****U****C****U****C**.A..GGN.C**G****G****G****G****U**GA...............A**A**U-**C****C****C****C**.ACC.G**G****C****G****G****U**U-..........................................................................................................................C**A****G****C**.**C****C****G****C**GA.**G****C****G****C****C****A****U**C........................................................................................................UUCG.................................G**G****U****G****G**.GUC**A****G****C**A-GA....**C**A**C****U****G****G****U**G.G.......A.......**A**..CU.........................**C****C****A****G****G****G****C**.C.GA**C****G****G**UUA..G.A.GU................................**C****C****G**.G.AUGGU.A.**G**..**A**.**G****A****A****U****G****G****G****U** | |
|  |  | AAFX01084096.1/141-288  | **C****C****G****C****G****U****U****C****U****C**.A..GGG.C**G****G****G****G****U**GA...............A**A**GU**C****C****C****C**.ACC.G**G****C****G****G****U**AAUGCCUGCGCAGGCAGGAC........................................................................................................G**A****G****C**.**C****C****G****C**GG.**G****C****G****C****C****C****C**U........................................................................................................CUCC..............................GGAG**G****G****G****G**..AC**A****G****C**A-GA....**C**A**C****C****G****G****U**G.C.......G.......**A**..UC.........................**C****C****G****G****G****G****C**.C.GA**C****G****G**UUA..G.A.GU................................**C****C****G**.G.UU--A.U.**G**..**A**.**G****A****A****C****C****G****C****U** | |
|  |  | AAFY01009023.1/227-397  | **U****U****U****A****A****U****U****C****U****C**.A..GGG.C**G****G****G****G****C**GC...............A**A**UU**C****C****C****C**.ACC.G**G****C****G****G****U**AUACUUGAAAAAGU..............................................................................................................G**A****G****C**.**C****C****G****C**GA.**G****C****G****C****U****C****G**A........................................................................................................UUCG.................................U**C****G****A****G**.GUC**A****G****C**A-GA....**U**.**C****U****G****G****U**G.ACU.....-UAGGU..**A**..UUUACUCAUGAGUAAUGUCUAACAGU.**C****C****A****G****A****G****C**.C.GA**C****G****G**UUA..U.A.GU................................**C****C****G**.G.AUGAG.A.**G**..**A**.**G****A****A****U****A****G****A****G** | |
|  |  | AAFX01032056.1/755-882  | **U****U****G****C****G****U****A****C****U****C**.C..GGG.G**U****C****G****G****U**GA...............A**A**UU**C****C****G****A**.ACC.G**G****C****G****G****U**GA..........................................................................................................................C**A****G****U**.**C****C****G****C**GAC**C****C****G****G****U****C****A**CA.......................................................................................................GCCA.................................G**U****G****G****C**....**C****G****G**UGGA....**G**.**C****C****G****G****U**G.A.......A.......**A**..CU.........................-**C****G****G****C****A****C**.C.GA**C****G****G**UGA..A.A.GU................................**C****C****G**.G.AUGGG.A.**G**..**G**.**C****G****G****U****A****C****G****C** | |
|  |  | AAFY01022492.1/757-922  | **C****A****U****C****G****U****U****C****U****C**.G..GGG.C**G****G****G****G****U**GA...............A**A**UU**C****C****C****C**.ACCCG**G****C****G****G****U**ACAGCGUUUAUUAACCCUG.........................................................................................................A**A****G****C**.**C****C****G****C**CA.**G****C****G****C****U****U****G**CCGGCCG..................................................................................................GUUU........................AACUGCCCAG**C****A****A****G**.GUC**A****G****C**A-GA....**U**C**C****C****G****G****U**G.U.......A.......**A**ACUU.........................**C****C****G****G****A****G****C**.CCAA**C****G****G**.UAU.U.A.GU...............................C**C****C****G**.G.AU-AA.A.**A**..**A**.**G****A****A****G****A****G****C****C** | |
|  |  | AAFX01125378.1/416-589  | **A****A****U****A****A****C****C****U****U****C**.G..GGG.C**A****G****G****G****C**GC...............A**A**UU**C****C****C****G**.AUC.G**G****U****G****G****U**A-..........................................................................................................................U**A****G****C**.**C****C****A****C**GAA**C****A****C****A****U****G****G**ACUCAGGAAGCUUGCUUCCGUUAUUACGCCC..........................................................................GCAA.....................GCUGGCUGAACUC**C****A****A****A**....**G****C****A**UGGA....**U**.**C****G****G****G****U**GGU.......A.......**A**..UUC........................**C****C****A****G****A****G****C**CC.GA**C****A****C**GUA..ACA.GU................................**C****U****G**GG.AUAGA.A.**G**..**A**.**A****G****G****G****A****U****A****U** | |
|  |  | AACY01230681.1/464-691  | **G****C****A****A****A****U****U****C****U****C**.A..GGG.C**G****G****G****G****U**GA...............A**A**CU**C****C****C****C**.ACC.G**G****C****G****G****U**AAAUGAGUAACGGCUAAGUGAUGGUGACUUAGUGUGGCUCA...................................................................................A**A****G****C**.**C****C****G****C**GA.**G****C****G****C****C****C****G**AAAUCAUGUUCGGGGUCAGCAGAUCUGGUGCCUCAGU....................................................................GUUU................................GC**C****G****A****G**....**U****G****C**A-AA....**U**.**C****U****G****A****U**-.-.......-.......**A**..UU.........................**C****C****A****G****A****G****C**.C.GA**C****G****G**.UA..A.U.G-GACUGUGACCUUAAUGGCAACAGUGCUAAGU.**C****C****G**.G.AUGGA.A.**G**..**A**.**G****A****A****U****G****U****A****A** | |
|  |  | SS\_cons |  | <<<<<<<<<<.........<<<<[..................]..>>>>......<<<<[.............................................................................................................................]AA.>>>>...<<<<<<<...............................................................................................................................................>>>>....>>>........<.<<<<[..................].............................>>>>>aa.....<<<...........................................>>>...........>..>.>>>>>>>> |
|  |  | SS\_label |  | ====P1====.........-P2-......................-P2-......=P3=..................................................................................................................................=P3=...---P4--...............................................................................................................................................----P4-----........==P5==.................................................=P5==.......-P6...........................................P6-...........=====P1====== |
|  |  | pair\_prediction\_1 |  | ...........................................................................................................................................................................................<.............................................................................................................................................................................................................................................>............................................................................... |
|  |  | RF |  | gaucaUcUUC.A..GGG.CgGGGUGa...............AAUUCCCc.ACC.GGCGGUAA..........................................................................................................................AAGC.CCGCGA.gCgcuug.........................................................................................................cuau..................................caag....aGcaUGA....u.ccGGUG.A.......G.......A..UU.........................CCggaGC.C.GACaG.UA..u.A.GU................................CuG.G.AUGGA.A.G..A.AgAugaag |
|  |  | SS\_align |  | ((((((((((.,..,,,.,<<<<\_\_\_...............\_\_\_\_>>>>.,,,.,<<<<\_\_\_..........................................................................................................................\_\_\_\_.>>>>,,.<<<<<<<.........................................................................................................\_\_\_\_..................................>>>>....>>>,,,,....<.<<<<\_\_.\_.......\_.......\_..\_\_.........................>>>>>,,.,.,,<<<.\_\_..\_.\_.\_\_................................>>>.,.,,,,,.,.)..).)))))))) |
