# Supplementary material for: The distributions, mechanisms, and structures of metabolite-binding riboswitches
Source: Genome Biol. 2007 Nov 12;8(11):R239. doi: 10.1186/gb-2007-8-11-r239 (PMC2258182; doi:10.1186/gb-2007-8-11-r239)
Supplement: Additional data file 2 — Sequence alignments of the riboswitch aptamer data sets annotated with new base-base interactions in HTML format. [file gb-2007-8-11-r239-S2.zip › HTML/GlcN6P.html]

|  |  |  |  |  |
| --- | --- | --- | --- | --- |
|  |  | **Accession/Start-End** |  | **Sequence** |
|  |  | NZ\_AAEK01000074.1/10289-10125  | UGUAAAUUAUAGAAG**C****G****C****C**A**G****A****A****C****U****A****C****A****.**..................AGUAG...........................................................................................................**.****U****G****U****A****G****U****U**GA**C****G****A**GGU............................................**G****G****G****G****U**.UUA**U****C****G****A**.GAUU.**U****C****G****G****C****G**GAUG.**A****C****U****C****C****C****G****G****U****U****G**......U**U****C****A****U****C****A**..**C****A****A****C****C****G**CAA..**G****C****U****U****U****U****A**C.UUAAAU.**C****A****U****U****A****A****G**GUGA.....**C****U****U****A****G****U****G**GACAAAG.G**U****A****A****A****A****G****U**G..**U****G****A****U****G****A**GAGAAAAGGA | |
|  |  | NC\_003997.3/157525-157689  | UGUAAAUUAUAGAAG**C****G****C****C**A**G****A****A****C****U****A****C****A****.**..................AGUAG...........................................................................................................**.****U****G****U****A****G****U****U**GA**C****G****A**GGU............................................**G****G****G****G****U**.UUA**U****C****G****A**.GAUU.**U****C****G****G****C****G**GAUG.**A****C****U****C****C****C****G****G****U****U****G**......U**U****C****A****U****C****A**..**C****A****A****C****C****G**CAA..**G****C****U****U****U****U****A**C.UUAAAU.**C****A****U****U****A****A****G**GUGA.....**C****U****U****A****G****U****G**GACAAAG.G**U****G****A****A****A****G****U**G..**U****G****A****U****G****A**GAGAAAAGGA | |
|  |  | NC\_005945.1/157526-157690  | UGUAAAUUAUAGAAG**C****G****C****C**A**G****A****A****C****U****A****C****A****.**..................AGUAG...........................................................................................................**.****U****G****U****A****G****U****U**GA**C****G****A**GGU............................................**G****G****G****G****U**.UUA**U****C****G****A**.GAUU.**U****C****G****G****C****G**GAUG.**A****C****U****C****C****C****G****G****U****U****G**......U**U****C****A****U****C****A**..**C****A****A****C****C****G**CAA..**G****C****U****U****U****U****A**C.UUAAAU.**C****A****U****U****A****A****G**GUGA.....**C****U****U****A****G****U****G**GACAAAG.G**U****G****A****A****A****G****U**G..**U****G****A****U****G****A**GAGAAAAGGA | |
|  |  | NC\_005957.1/163247-163411  | UGUAAAUUAUAGAAG**C****G****C****C**A**G****A****A****C****U****A****C****A****.**..................AGUAG...........................................................................................................**.****U****G****U****A****G****U****U**GA**C****G****A**GGU............................................**G****G****G****G****U**.UUA**U****C****G****A**.GAUU.**U****C****G****G****C****G**GAUG.**A****C****U****C****C****C****G****G****U****U****G**......U**U****C****A****U****C****A**..**C****A****A****C****C****G**CAA..**G****C****U****U****U****U****A**C.UUAAAU.**C****A****U****U****A****A****G**GUGA.....**C****U****U****A****G****U****G**GACAAAG.G**U****G****A****A****A****G****U**G..**U****G****A****U****G****A**GAGAAAAGGA | |
|  |  | NC\_006274.1/157515-157679  | UGUAAAUUAUAGAAG**C****G****C****C**A**G****A****A****C****U****A****C****A****.**..................AGUAG...........................................................................................................**.****U****G****U****A****G****U****U**GA**C****G****A**GGU............................................**G****G****G****G****U**.UUA**U****C****G****A**.GAUU.**U****C****G****G****C****G**GAUG.**A****C****U****C****C****C****G****G****U****U****G**......U**U****C****A****U****C****A**..**C****A****A****C****C****G**CAA..**G****C****U****U****U****U****A**C.UUAAAU.**C****A****U****U****A****A****G**GUGA.....**C****U****U****A****G****U****G**GACAAAG.G**U****G****A****A****A****G****U**G..**U****G****A****U****G****A**GAGAAAAGGA | |
|  |  | NC\_007530.2/157525-157689  | UGUAAAUUAUAGAAG**C****G****C****C**A**G****A****A****C****U****A****C****A****.**..................AGUAG...........................................................................................................**.****U****G****U****A****G****U****U**GA**C****G****A**GGU............................................**G****G****G****G****U**.UUA**U****C****G****A**.GAUU.**U****C****G****G****C****G**GAUG.**A****C****U****C****C****C****G****G****U****U****G**......U**U****C****A****U****C****A**..**C****A****A****C****C****G**CAA..**G****C****U****U****U****U****A**C.UUAAAU.**C****A****U****U****A****A****G**GUGA.....**C****U****U****A****G****U****G**GACAAAG.G**U****G****A****A****A****G****U**G..**U****G****A****U****G****A**GAGAAAAGGA | |
|  |  | NZ\_AAAC02000001.1/697152-697316  | UGUAAAUUAUAGAAG**C****G****C****C**A**G****A****A****C****U****A****C****A****.**..................AGUAG...........................................................................................................**.****U****G****U****A****G****U****U**GA**C****G****A**GGU............................................**G****G****G****G****U**.UUA**U****C****G****A**.GAUU.**U****C****G****G****C****G**GAUG.**A****C****U****C****C****C****G****G****U****U****G**......U**U****C****A****U****C****A**..**C****A****A****C****C****G**CAA..**G****C****U****U****U****U****A**C.UUAAAU.**C****A****U****U****A****A****G**GUGA.....**C****U****U****A****G****U****G**GACAAAG.G**U****G****A****A****A****G****U**G..**U****G****A****U****G****A**GAGAAAAGGA | |
|  |  | NZ\_AAEN01000019.1/9340-9504  | UGUAAAUUAUAGAAG**C****G****C****C**A**G****A****A****C****U****A****C****A****.**..................AGUAG...........................................................................................................**.****U****G****U****A****G****U****U**GA**C****G****A**GGU............................................**G****G****G****G****U**.UUA**U****C****G****A**.GAUU.**U****C****G****G****C****G**GAUG.**A****C****U****C****C****C****G****G****U****U****G**......U**U****C****A****U****C****A**..**C****A****A****C****C****G**CAA..**G****C****U****U****U****U****A**C.UUAAAU.**C****A****U****U****A****A****G**GUGA.....**C****U****U****A****G****U****G**GACAAAG.G**U****G****A****A****A****G****U**G..**U****G****A****U****G****A**GAGAAAAGGA | |
|  |  | NZ\_AAEO01000023.1/88432-88268  | UGUAAAUUAUAGAAG**C****G****C****C**A**G****A****A****C****U****A****C****A****.**..................AGUAG...........................................................................................................**.****U****G****U****A****G****U****U**GA**C****G****A**GGU............................................**G****G****G****G****U**.UUA**U****C****G****A**.GAUU.**U****C****G****G****C****G**GAUG.**A****C****U****C****C****C****G****G****U****U****G**......U**U****C****A****U****C****A**..**C****A****A****C****C****G**CAA..**G****C****U****U****U****U****A**C.UUAAAU.**C****A****U****U****A****A****G**GUGA.....**C****U****U****A****G****U****G**GACAAAG.G**U****G****A****A****A****G****U**G..**U****G****A****U****G****A**GAGAAAAGGA | |
|  |  | NZ\_AAEP01000033.1/6922-7086  | UGUAAAUUAUAGAAG**C****G****C****C**A**G****A****A****C****U****A****C****A****.**..................AGUAG...........................................................................................................**.****U****G****U****A****G****U****U**GA**C****G****A**GGU............................................**G****G****G****G****U**.UUA**U****C****G****A**.GAUU.**U****C****G****G****C****G**GAUG.**A****C****U****C****C****C****G****G****U****U****G**......U**U****C****A****U****C****A**..**C****A****A****C****C****G**CAA..**G****C****U****U****U****U****A**C.UUAAAU.**C****A****U****U****A****A****G**GUGA.....**C****U****U****A****G****U****G**GACAAAG.G**U****G****A****A****A****G****U**G..**U****G****A****U****G****A**GAGAAAAGGA | |
|  |  | NZ\_AAEQ01000020.1/87337-87173  | UGUAAAUUAUAGAAG**C****G****C****C**A**G****A****A****C****U****A****C****A****.**..................AGUAG...........................................................................................................**.****U****G****U****A****G****U****U**GA**C****G****A**GGU............................................**G****G****G****G****U**.UUA**U****C****G****A**.GAUU.**U****C****G****G****C****G**GAUG.**A****C****U****C****C****C****G****G****U****U****G**......U**U****C****A****U****C****A**..**C****A****A****C****C****G**CAA..**G****C****U****U****U****U****A**C.UUAAAU.**C****A****U****U****A****A****G**GUGA.....**C****U****U****A****G****U****G**GACAAAG.G**U****G****A****A****A****G****U**G..**U****G****A****U****G****A**GAGAAAAGGA | |
|  |  | NZ\_AAER01000024.1/88435-88271  | UGUAAAUUAUAGAAG**C****G****C****C**A**G****A****A****C****U****A****C****A****.**..................AGUAG...........................................................................................................**.****U****G****U****A****G****U****U**GA**C****G****A**GGU............................................**G****G****G****G****U**.UUA**U****C****G****A**.GAUU.**U****C****G****G****C****G**GAUG.**A****C****U****C****C****C****G****G****U****U****G**......U**U****C****A****U****C****A**..**C****A****A****C****C****G**CAA..**G****C****U****U****U****U****A**C.UUAAAU.**C****A****U****U****A****A****G**GUGA.....**C****U****U****A****G****U****G**GACAAAG.G**U****G****A****A****A****G****U**G..**U****G****A****U****G****A**GAGAAAAGGA | |
|  |  | NZ\_AAES01000019.1/7927-8091  | UGUAAAUUAUAGAAG**C****G****C****C**A**G****A****A****C****U****A****C****A****.**..................AGUAG...........................................................................................................**.****U****G****U****A****G****U****U**GA**C****G****A**GGU............................................**G****G****G****G****U**.UUA**U****C****G****A**.GAUU.**U****C****G****G****C****G**GAUG.**A****C****U****C****C****C****G****G****U****U****G**......U**U****C****A****U****C****A**..**C****A****A****C****C****G**CAA..**G****C****U****U****U****U****A**C.UUAAAU.**C****A****U****U****A****A****G**GUGA.....**C****U****U****A****G****U****G**GACAAAG.G**U****G****A****A****A****G****U**G..**U****G****A****U****G****A**GAGAAAAGGA | |
|  |  | NC\_004722.1/163174-163338  | UGUAAAUUAUAAAAG**C****G****C****C**A**G****A****A****C****U****A****C****A****.**..................AAUAG...........................................................................................................**.****U****G****U****A****G****U****U**GA**C****G****A**GGU............................................**G****G****G****G****U**.UUA**U****C****G****A**.GAUU.**U****C****G****G****C****G**GAUG.**A****C****U****C****C****C****G****G****U****U****G**......U**U****C****A****U****C****A**..**C****A****A****C****C****G**CAA..**G****C****U****U****U****U****A**C.UUAAAU.**C****A****U****U****A****A****G**GUGA.....**C****U****U****A****G****U****G**GACAAAG.G**U****G****A****A****A****G****U**G..**U****G****A****U****G****A**GAGAAGAGGC | |
|  |  | NC\_002570.2/288764-288941  | CUAUGACAGUUGAAG**C****G****C****C**A**G****G****A****C****U****A****U****U****C**CACG..............GACGG......................................................................................................UAACG**G****A****A****U****A****G****U****U**GA**C****G****A**GGA............................................**G****A****G****G****G**UUUA**U****C****G****A**.AGGU.**U****C****G****G****C****G**GAUG.**C****C****C****U****C****C****G****G****U****U****G**......C**A****C****A****U****G****A**..**C****A****G****C****C****G**CAA..**G****C****U****U****U****U****G**U.AAAAAA.**C****A****G****A****G****G****G**GCGA.....**C****C****U****U****C****U****G**GACAAAG.G**C****A****A****A****A****G****C**GU.**U****C****A****U****G****C**UAACAAAAAA | |
|  |  | NC\_003909.8/157527-157691  | UGUAAAUUAUAGAAG**C****G****C****C**A**G****A****A****C****U****A****C****A****.**..................AGUAG...........................................................................................................**.****U****G****U****A****G****U****U**GA**C****G****A**GGU............................................**G****G****G****G****U**.UUA**U****C****G****A**.GAUU.**U****C****G****G****C****G**GAUG.**G****C****U****C****C****C****G****G****U****U****G**......U**U****C****A****U****C****A**..**C****A****A****C****C****G**CAA..**G****C****U****U****U****U****A**C.UUAAAU.**C****A****U****U****A****A****G**GUGA.....**C****U****U****A****G****U****G**GACAAAG.G**U****G****A****A****A****G****U**G..**U****G****A****U****G****A**GAGAAAAGGA | |
|  |  | NC\_004193.1/258874-259052  | GACUAUAAUCAAAAG**C****G****C****C**A**G****G****A****C****U****A****U****U****C**UUCGGACG..........GAUAG........................................................................................................GAA**G****G****A****U****A****G****U****U**GA**C****G****A**GGUA...........................................**G****G****A****G****G**.UUA**U****C****G****A**.AUUU.**U****C****G****G****C****G**GGUG.**C****C****U****C****C****C****G****G****C****U****G**.......**U****C****A****U****C****A**..**C****A****G****U****C****G**AAA..**C****U****U****U****A****U****C**A.CUAAAA.**C****A****G****U****A****G****G**GUGA.....**C****C****U****A****U****U****G**CACAAAA.G**G****A****U****A****A****A****G**AA.**A****G****A****U****G****U**CUUAUCAAAU | |
|  |  | NC\_003030.1/179913-180082  | AAAUUAUAGUUAAAG**C****G****C****C**C**G****A****A****C****U****U****G****A****G**UGA...............UAUAG.......................................................................................................UUCA**U****U****U****A****A****G****U****U**GA**C****G****A**GGA............................................**U****G****G****G****G**AGAA**U****C****G****A**.AUCU.**U****C****G****G****C****G**GAUG.**C****C****C****C****A****C****G****G****U****A****.**.......**C****C****G****C****A****C**..**.****U****A****C****C****G**UUAGC**G****G****U****U****G****G****.**...UAAAA.**G****C****A****G****A****A****A**GUGA.....**U****U****U****C****U****G****U**CACAAAG..**.****C****C****A****A****U****C**UG.**G****U****G****U****U****A**AAACCUAUUU | |
|  |  | NC\_003366.1/2679186-2678959  | AAUAAUAGAUUAAAG**C****G****C****C**A**G****A****A****C****U****U****A****A****A**GUUUUAUU..........UAUAG...................................................ACUAAUAAGUAAUUUAACUUUAGUAGAUUAAAAUGCUUAUUAAGAUUCUUAAUAAA**U****U****U****A****A****G****U****U**GA**C****G****A**GGU............................................**U****G****G****G****G**AGUA**U****C****G****A**.AUUU.**U****C****G****G****C****G**GAUG.**C****C****C****C****A****C****G****G****U****A****.**.......**A****A****G****C****A****C**..**.****U****A****C****C****G**UAAAA**G****A****U****U****G****G****.**..UUAAAA.**C****U****U****A****A****A****A**GUGA.....**U****U****U****U****A****A****G**GACAAAG..**.****C****C****A****A****U****U**GG.**G****U****G****U****U****U**AAAACCUUCU | |
|  |  | NC\_002976.3/1799230-1799002  | UAUUAAUAAUUAAAG**C****G****C****C**U**G****U****G****C****A****A****A****A****U**ACC...............UAAAU.....................................................AGUUAUCAAGUAUUAAAAGUUAAAAUAGUAGAACUUUUAUAGAUAACGAUUUGG**A****U****U****U****U****G****U****A**GA**C****G****A**GGA............................................**G****G****A****U****A**GUGA**U****C****G****A**AUCAGA**U****C****G****G****C****G**GAUGC**U****A****U****C****C****C****G****G****A****U****G**.......**.****.****U****G****G****C**A.**C****A****U****U****C****G**UUAG.**C****U****U****A****U****U****A**A.GUAAAU.**C****A****U****U****A****A****G**GUGA.....**C****U****U****A****G****U****G**GACAAAG.U**U****A****A****U****A****A****G**AUC**G****C****C****A****A****A**CUAAAUAAAU | |
|  |  | NC\_004461.1/1804202-1803974  | UAUUAAUAAUUAAAG**C****G****C****C**U**G****U****G****C****A****A****A****A****U**ACC...............UAAAU.....................................................AGUUAUCAAGUAUUAAAAGUUAAAAUAGUAGAACUUUUAUAGAUAACGAUUUGG**A****U****U****U****U****G****U****A**GA**C****G****A**GGA............................................**G****G****A****U****A**GUGA**U****C****G****A**AUCAGA**U****C****G****G****C****G**GAUGC**U****A****U****C****C****C****G****G****A****U****G**.......**.****.****U****G****G****C**A.**C****A****U****U****C****G**UUAG.**C****U****U****A****U****U****A**A.GUAAAU.**C****A****U****U****A****A****G**GUGA.....**C****U****U****A****G****U****G**GACAAAG.U**U****A****A****U****A****A****G**AUC**G****C****C****A****A****A**CUAAAUAAAU | |
|  |  | NZ\_AAAW03000067.1/17266-17083  | GGCAAGUUUAUACAG**C****G****C****C**U**G****G****A****C****C****G****U****A****A**UCGAGUUACGAACUCG..AAUAU........................................................................................................CGA**U****U****A****G****G****G****U****U**GA**C****G****A**GGA............................................**G****G****A****G****A**GUUA**U****C****G****A**AAGAC.**U****C****G****G****C****G**GAUGC**U****C****U****C****C****C****G****G****U****G****.**.......**U****C****U****U****G****A**..**.****C****A****C****C****G**UAA..**G****C****U****U****A****U****U**..ACAAAA.**C****C****G****U****G****A****A**GUAA.....**U****U****U****G****C****G****G**GACAAAA..**G****G****U****A****A****G****C**C..**U****C****A****A****G****G**AACGGGAGUA | |
|  |  | NC\_002745.2/2212308-2212081  | GCAGUACAGUUAAAG**C****G****C****C**U**G****U****G****C****A****A****A****U****A**..................AAUAU..................................................UUGUAUUUGAAGAUUAAAGGUUAAUAUAUGAGUGGCCUUUAUAGAGUGCAAUAUAUG**U****A****U****U****U****G****U****A**GA**C****G****A**GGA............................................**G****G****A****U****A**GUGA**U****C****G****A**.AUAGA**U****C****G****G****C****G**GAUGC**U****A****U****C****C****C****G****G****A****U****G**.......**.****.****U****G****G****C**U.**C****A****U****U****C****G**UUAG.**C****U****U****A****U****U****A**A.GUAAAA.**C****A****U****U****A****G****G**GUGA.....**C****U****U****A****A****U****G**GACAAAG.U**U****A****A****U****A****A****G**AUC**G****C****C****A****G****A**AAUUGAAUAU | |
|  |  | NC\_002758.2/2282028-2281801  | GCAGUACAGUUAAAG**C****G****C****C**U**G****U****G****C****A****A****A****U****A**..................AAUAU..................................................UUGUAUUUGAAGAUUAAAGGUUAAUAUAUGAGUGGCCUUUAUAGAGUGCAAUAUAUG**U****A****U****U****U****G****U****A**GA**C****G****A**GGA............................................**G****G****A****U****A**GUGA**U****C****G****A**.AUAGA**U****C****G****G****C****G**GAUGC**U****A****U****C****C****C****G****G****A****U****G**.......**.****.****U****G****G****C**U.**C****A****U****U****C****G**UUAG.**C****U****U****A****U****U****A**A.GUAAAA.**C****A****U****U****A****G****G**GUGA.....**C****U****U****A****A****U****G**GACAAAG.U**U****A****A****U****A****A****G**AUC**G****C****C****A****G****A**AAUUGAAUAU | |
|  |  | NC\_002951.2/2208552-2208325  | GCAGUACAGUUAAAG**C****G****C****C**U**G****U****G****C****A****A****A****U****A**..................AAUAU..................................................UUGUAUUUGAAGAUUAAAGGUUAAUAUAUGAGUGGCCUUUAUAGAGUGCAAUAUAUG**U****A****U****U****U****G****U****A**GA**C****G****A**GGA............................................**G****G****A****U****A**GUGA**U****C****G****A**.AUAGA**U****C****G****G****C****G**GAUGC**U****A****U****C****C****C****G****G****A****U****G**.......**.****.****U****G****G****C**U.**C****A****U****U****C****G**UUAG.**C****U****U****A****U****U****A**A.GUAAAA.**C****A****U****U****A****G****G**GUGA.....**C****U****U****A****A****U****G**GACAAAG.U**U****A****A****U****A****A****G**AUC**G****C****C****A****G****A**AAUUGAAUAU | |
|  |  | NC\_002952.2/2311875-2311648  | GCAGUACAGUUAAAG**C****G****C****C**U**G****U****G****C****A****A****A****U****A**..................AAUAU..................................................UUGUAUUUGAAGAUUAAAGGUUAAUAUAUGAGUGGCCUUUAUAGAGUGCAAAAUAUG**U****A****U****U****U****G****U****A**GA**C****G****A**GGA............................................**G****G****A****U****A**GUGA**U****C****G****A**.AUAGA**U****C****G****G****C****G**GAUGC**U****A****U****C****C****C****G****G****A****U****G**.......**.****.****U****G****G****C**U.**C****A****U****U****C****G**UUAG.**C****U****U****A****U****U****A**A.GUAAAA.**C****A****U****U****A****G****G**GUGA.....**C****U****U****A****A****U****G**GACAAAG.U**U****A****A****U****A****A****G**AUC**G****C****C****A****G****A**AAUUGAAUAU | |
|  |  | NC\_002953.3/2211350-2211123  | GCAGUACAGUUAAAG**C****G****C****C**U**G****U****G****C****A****A****A****U****A**..................AAUAU..................................................UUGUAUUUGAAGAUUAAAGGUUAAUAUAUGAGUGGCCUUUAUAGAGUGCAAUAUAUG**U****A****U****U****U****G****U****A**GA**C****G****A**GGA............................................**G****G****A****U****A**GUGA**U****C****G****A**.AUAGA**U****C****G****G****C****G**GAUGC**U****A****U****C****C****C****G****G****A****U****G**.......**.****.****U****G****G****C**U.**C****A****U****U****C****G**UUAG.**C****U****U****A****U****U****A**A.GUAAAA.**C****A****U****U****A****G****G**GUGA.....**C****U****U****A****A****U****G**GACAAAG.U**U****A****A****U****A****A****G**AUC**G****C****C****A****G****A**AAUUGAAUAU | |
|  |  | NC\_003923.1/2232245-2232018  | GCAGUACAGUUAAAG**C****G****C****C**U**G****U****G****C****A****A****A****U****A**..................AAUAU..................................................UUGUAUUUGAAGAUUAAAGGUUAAUAUAUGAGUGGCCUUUAUAGAGUGCAAUAUAUG**U****A****U****U****U****G****U****A**GA**C****G****A**GGA............................................**G****G****A****U****A**GUGA**U****C****G****A**.AUAGA**U****C****G****G****C****G**GAUGC**U****A****U****C****C****C****G****G****A****U****G**.......**.****.****U****G****G****C**U.**C****A****U****U****C****G**UUAG.**C****U****U****A****U****U****A**A.GUAAAA.**C****A****U****U****A****G****G**GUGA.....**C****U****U****A****A****U****G**GACAAAG.U**U****A****A****U****A****A****G**AUC**G****C****C****A****G****A**AAUUGAAUAU | |
|  |  | NC\_006270.2/183342-183526  | UUUUUAUAGUUAUAG**C****G****C****C**C**G****G****A****C****U****A****A****G****C**GUACG.............GAUGA.......................................................................................................AACA**G****C****U****U****A****G****U****U**GA**C****G****A**GGAU...........................................**G****G****A****G****G**UUUA**U****C****G****A**.AUUU.**U****C****G****G****C****G**GAUG.**C****C****U****C****C****C****G****G****C****U****G**ACCUCAA**A****G****A****U****C****A**..**C****A****G****C****C****G**CAAG.**G****A****U****U****U****C****C**..UCAAAC.**C****A****A****A****G****A****G**GCGA.....**C****U****C****U****U****U****G**AACAAAG..**G****G****A****A****A****U****U**GCA**U****G****A****U****C****U**UCCAUAAAAA | |
|  |  | NC\_006322.1/183150-183334  | UUUUUAUAGUUAUAG**C****G****C****C**C**G****G****A****C****U****A****A****G****C**GUACG.............GAUGA.......................................................................................................AACA**G****C****U****U****A****G****U****U**GA**C****G****A**GGAU...........................................**G****G****A****G****G**UUUA**U****C****G****A**.AUUU.**U****C****G****G****C****G**GAUG.**C****C****U****C****C****C****G****G****C****U****G**ACCUCAA**A****G****A****U****C****A**..**C****A****G****C****C****G**CAAG.**G****A****U****U****U****C****C**..UCAAAC.**C****A****A****A****G****A****G**GCGA.....**C****U****C****U****U****U****G**AACAAAG..**G****G****A****A****A****U****U**GCA**U****G****A****U****C****U**UCCAUAAAAA | |
|  |  | NC\_004557.1/2702477-2702194  | UAAAAUAUAAUAAAG**C****G****C****C**A**G****G****A****C****U****U****A****G****G**UGAUGAAACUCAGAC...UAUGGUCUGAGUAAGUUCGACUAACCAAAUCACAGAUUUGGAGUAAGAUUAAUGAAACUCAGACUAUGGUCUGAGUAAGUUCCACCAACCAAAUCACAGAUUUGGGGUGUCA**C****U****U****A****A****G****U****U**GA**C****G****A**GGA............................................**U****G****G****G****G**AGUA**U****C****G****A**.AUCU.**U****C****G****G****C****G**GGUG.**C****C****C****C****A****C****G****G****U****A****.**.......**C****U****G****C****A****C**..**.****U****A****C****C****G**UUAAA**G****A****U****U****G****A****.**...CAAAA.**C****C****A****A****G****G****A**GUAA.....**U****U****U****U****U****G****G**UACAAA...**.****U****C****A****A****U****C**AG.**G****U****G****U****U****A**AAACCUUAAA | |
|  |  | NC\_003869.1/2101607-2101440  | GCUUUAAUUUAGAAG**C****G****C****C**U**G****G****A****C****U****U****A****A****A**GCC...............UUAAG.........................................................................................................GC**U****U****U****A****A****G****U****U**GA**C****G****A**GGG............................................**C****A****G****G****G**UUUA**U****C****G****A**.GACA.**U****C****G****G****C****G**GGUG.**C****C****C****U****G****C****G****G****U****C****U**.......**U****C****C****U****G****C**..**.****G****A****C****C****G**UUAGA**G****G****A****C****U****G****.**..GUAAAA.**.****C****C****A****C****A****G**GCGA.....**C****U****G****U****G****G****.**CAUAGAG..**.****C****A****G****U****C****C**GG.**G****C****A****G****G****A**AAAUUUUAUA | |
|  |  | NC\_002973.5/771065-771271  | ACAAUUAAAUAGAAG**C****G****C****C**A**G****A****A****C****U****G****A****U****U**GGGACG............AAAAU.............................................................................ACUUGAAGGUGAAAUCCCUGAAAAGUAAAC**A****G****U****C****A****G****U****U**GA**C****G****A**GGA............................................**G****G****A****G****A**UUAA**U****C****G****A**.AGUU.**U****C****G****G****C****G**GGAG.**U****C****U****C****C****C****G****G****C****U****G**.......**U****G****C****A****U****G**..**C****A****G****U****C****G**UUAAG**U****C****U****U****A****C****U**U.ACAAAU.**C****A****C****U****U****G****G**GUGA.....**C****C****A****A****G****U****G**GACAGAG.U**A****G****U****A****A****U****G**AAA**C****A****U****G****U****U**UUUUACCCUC | |
|  |  | NZ\_AADR01000002.1/45417-45211  | ACAAUUAAAUAGAAG**C****G****C****C**A**G****A****A****C****U****G****A****U****U**GGAACG............AAAAU.............................................................................ACUUGAAGGUGAAAUCCCUGAAAAGUAAAC**A****G****U****C****A****G****U****U**GA**C****G****A**GGA............................................**G****G****A****G****A**UUAA**U****C****G****A**.AGUU.**U****C****G****G****C****G**GGAG.**U****C****U****C****C****C****G****G****C****U****G**.......**U****G****C****A****U****G**..**C****A****G****U****C****G**UUAAG**U****C****U****U****A****C****U**U.ACAAAU.**C****A****C****U****U****G****G**GUGA.....**C****C****A****A****G****U****G**GACAGAG.U**A****G****U****A****A****U****G**AAA**C****A****U****G****U****U**UUUUACCCUC | |
|  |  | NZ\_AADQ01000005.1/54176-53971  | ACAAUUGAAUAGAAG**C****G****C****C**A**G****A****A****C****U****G****A****U****U**GGGACG............AAAAU..............................................................................GCUUGAAGGUGAAAUCCCUGAAAAGUAUC**G****A****U****C****A****G****U****U**GA**C****G****A**GGA............................................**G****G****A****G****A**UUAA**U****C****G****A**.AGUU.**U****C****G****G****C****G**GGAG.**U****C****U****C****C****C****G****G****C****U****G**.......**U****G****C****A****U****G**..**C****A****G****U****C****G**UUAAG**U****C****U****U****A****C****U**U.ACAAAU.**C****A****U****U****U****G****G**GUGA.....**C****C****A****A****G****U****G**GACAGAG.U**A****G****U****A****A****U****G**AAA**C****A****U****G****C****U**UUUUACCCUC | |
|  |  | NC\_003210.1/756456-756661  | ACAAUUGAAUAGAAG**C****G****C****C**A**G****A****A****C****U****G****A****U****U**GGGACG............AAAAU..............................................................................GCUUGAAGGUGAAAUCCCUGAAAAGUAUC**G****A****U****C****A****G****U****U**GA**C****G****A**GGA............................................**G****G****A****G****A**UUAA**U****C****G****A**.AGUU.**U****C****G****G****C****G**GGAG.**U****C****U****C****C****C****G****G****C****U****G**.......**U****G****C****A****U****G**..**C****A****G****U****C****G**UUAAG**U****C****U****U****A****C****U**U.ACAAAU.**C****A****U****U****U****G****G**GUGA.....**C****C****A****A****G****U****G**GACAGAG.U**A****G****U****A****A****U****G**AAA**U****A****U****G****C****U**UUUUACCCUC | |
|  |  | NC\_003212.1/755732-755938  | ACAACUGAAUAGAAG**C****G****C****C**A**G****A****A****C****U****G****A****U****U**GGGACG............AAAAU.............................................................................GCUUAAAGGUGAAAUUCCUGGAAAGUAAAC**A****A****U****C****A****G****U****U**GA**C****G****A**GGA............................................**G****G****A****G****A**UUAA**U****C****G****A**.AAUU.**U****C****G****G****C****G**GGAG.**U****C****U****C****C****C****G****G****C****U****G**.......**U****G****C****A****U****G**..**C****A****G****U****C****G**UUAAG**U****C****U****U****A****C****U**U.ACAAAU.**C****A****U****U****U****G****G**GUGA.....**C****C****A****A****G****U****G**GACAGAG.U**A****G****U****A****A****U****G**AAA**C****A****U****G****U****G**CAAGAACCCU | |
|  |  | NC\_006582.1/270891-271055  | UUUCAUACACCAAAG**C****G****C****C**A**G****G****A****C****U****G****G****C****A**....................UGA...........................................................................................................**C****G****C****C****A****G****U****U**GA**C****G****A**GGA............................................**G****A****G****G****G**CUAA**U****C****G****A**.ACUU.**U****C****G****G****C****G**GGUU.**C****C****C****U****C****C****G****G****C****U****G**......C**G****U****G****U****G****A**..**C****A****G****C****C****G**CAA..**G****C****U****U****U****U****G**U..CAAAA.**C****A****A****U****G****G****G**GUGA.....**C****C****U****G****U****U****G**CACAAAA.A**C****A****A****A****A****G****C**A..**G****C****A****C****G****C**AACAAAUUCA | |
|  |  | NC\_006510.1/166976-167156  | UUGACGAGACAAAAG**C****G****C****C**U**G****G****A****C****U****A****A****G****C**G..................UUGG.................................................................................................ACGGAAGAAC**G****C****U****U****A****G****U****U**GA**C****G****A**GGA............................................**G****G****A****G****G**UUUA**U****C****G****A**GGUUU.**U****C****G****G****C****G**GAUG.**C****C****U****C****C****C****G****G****C****U****G**....GAG**C****G****A****U****G****A**..**C****A****G****C****C****G**CAA..**G****U****C****C****U****C****U**C.UUAAAA.**C****A****A****A****G****G****G**GUGA.....**C****C****C****U****U****U****G**CACAAAG.G**G****G****A****G****G****A****U**G..**U****C****A****U****C****G**CGCGAAACGA | |
|  |  | NZ\_AABF02000219.1/891-1045  | UUUAUGUAAAAGAAG**C****G****C****C**A**G****A****A****C****U****C****U****.****.**...................UUUU...........................................................................................................**.****.****A****G****A****G****U****U**GA**C****G****A**GGA............................................**U****U****G****G****A**AUUA**U****C****G****A**AGUUU.**U****C****G****G****C****G**GAUGU**U****C****C****A****A****A****G****G****U****G****.**.......**.****G****U****U****A****C**..**.****A****A****C****C****.**.....**A****U****U****A****U****C****A**..ACAAAA.**A****C****A****C****A****G****A**GUAA.....**U****U****U****G****U****G****U**AACAAAG..**G****G****A****U****A****A****U**A..**G****U****A****A****C****A**GUCUCCUGUU | |
|  |  | NC\_003454.1/1095654-1095808  | UUUAUGUAAAAGAAG**C****G****C****C**A**G****A****A****C****U****C****U****.****.**...................UUUU...........................................................................................................**.****.****A****G****A****G****U****U**GA**C****G****A**GGA............................................**U****U****G****G****A**AUUA**U****C****G****A**AGUUU.**U****C****G****G****C****G**GAUGU**U****C****C****A****A****A****G****G****U****G****.**.......**.****G****U****U****A****C**..**.****A****A****C****C****.**.....**A****U****U****A****U****C****A**..ACAAAA.**A****C****A****C****A****G****A**GCAA.....**U****U****U****G****U****G****U**AACAAAG..**A****G****A****U****A****A****U**A..**G****U****A****A****C****A**AUCUCCUAUU | |
|  |  | NC\_004668.1/2055462-2055306  | ACCAAUAUCGGAUAG**C****G****C****C**A**G****A****C****C****U****G****A****.****.**...................ACGU...........................................................................................................**.****.****U****C****A****G****G****U**GA**C****G****A**GGAG...........................................**A****G****A****G****C**.UUA**U****C****G****A**.AGAU.**U****C****G****G****C****G**GGUG.**G****C****U****C****U****A****G****G****G****.****.**.......**A****C****U****G****C****A**..**.****.****C****U****C****U**ACA..**G****A****U****A****A****C****A**A.AGAAAA.**A****C****U****A****A****U****U**GUGA.....**A****G****U****U****A****G****A**.ACAAAG.C**G****G****U****U****A****U****C**A..**C****G****C****A****G****G**UAGAAACAUA | |
|  |  | NC\_004567.1/761447-761607  | GUUAAAGCGGAAUAG**C****G****C****C**A**G****G****A****C****U****U****U****A****G**....................AAU...........................................................................................................**C****U****A****A****A****G****U****U**GA**C****G****A**GGAU...........................................**G****A****C****G****U**.UUA**U****C****G****A**.UAA..**U****C****G****A****C****G**GGUG.**A****C****G****U****C****A****G****G****G****.****.**.......**A****C****U****G****C****A**..**.****.****C****U****C****U**ACA..**G****G****U****C****A****A****U**U.ACAAAA.**A****C****C****G****A****C****U**GUGA.....**G****G****U****U****G****G****U**GACAGAUAU**A****U****U****G****A****C****C**A..**C****G****C****A****G****C**UAGAAACAAU | |
|  |  | NC\_000964.2/200004-200182  | UACCUAUAAUUAUAG**C****G****C****C**C**G****A****A****C****U****A****A****G****C**GCCCGGA...........AAAAG...........................................................................................................**G****C****U****U****A****G****U****U**GA**C****G****A**GGAU...........................................**G****G****A****G****G**.UUA**U****C****G****A**.AUU..**U****C****G****.****C****G**GAU..**C****C****U****C****C****C****G****G****C****U****G**AGUGUGC**A****G****A****U****C****A**..**C****A****G****C****C****G**UAAG.**A****A****U****U****U****C****U**..UCAAAC.**C****A****A****G****G****G****G**GUGA.....**C****U****C****C****U****U****G**AACAAAG..**A****G****A****A****A****U****C**ACA**U****G****A****U****C****U**UCCAAAAAAC | |
|  |  | NZ\_AABG04000024.1/10700-10512  | AAUUUAUAGAAAAAG**C****G****C****C**A**G****C****A****C****G****U****G****U****.**..................UUUAA...........................................................................................................**.****C****C****A****C****G****U****G**GA**C****G****A**GGAGAAGGAGGUCACCGGUAAUUUUUCAAUC................**G****G****U****G****A**GUUA**U****C****G****A**.GUUU.**U****C****G****G****C****G**GAAA.**C****C****U****U****C****C****G****G****U****U****.**.......**.****G****U****C****A****C**..**.****G****A****C****C****G**UCA..**G****G****G****U****C****C****U**..ACAAAGA**C****U****C****G****C****A****G**GCAA.....**C****U****G****C****G****.****G**UACAAAA..**G****G****G****A****U****C****U**GA.**G****U****G****A****A****U**UUUCCUGUCU | |
|  |  | NZ\_AAEB02000024.1/15360-15189  | AAGAAAAAGAGCAAG**C****G****C****C**A**G****G****A****C****U****C****C****G****G**GCC...............UCCCG.........................................................................................................GC**C****C****G****G****A****G****U****U**GA**C****G****A**GGU............................................**A****G****A****C****G**UUCA**U****C****G****A**.GCUU.**U****C****G****G****C****G**GGUG.**C****G****U****C****U****C****G****G****C****C****.**......C**U****C****G****G****G****C**..**.****G****G****U****C****G**UCA..**G****G****G****C****G****G****C**G.ACAAAU.**C****C****C****G****G****C****G**GCGA.....**C****G****C****C****G****G****G**GACAAAA.C**G****C****C****G****U****C****C**G.G**G****C****C****C****G****A**AGCGGGGCGC | |
|  |  | NZ\_AADT03000001.1/73229-73028  | AAAAACUAACGCAAG**C****G****C****C**G**G****G****A****C****C****A****G****C****C**AGCACUGACCGCUGUCCAGAUGG........................................................................................GUUCCCACUGGUUAGUGCU**G****G****U****U****G****G****U****U**GA**C****G****A**GGC............................................**G****G****G****G****G**UUAA**U****C****G****A**.GGCA.**U****C****G****G****C****G**GGUG.**C****C****C****C****C****C****G****G****U****U****.**......U**G****G****U****U****A****C**..**.****G****A****C****C****G**ACAA.**A****G****G****C****G****U****U**..ACAAAA.**C****C****C****G****G****G****A**GCAA.....**U****C****C****C****G****G****G**GACAAAG..**G****G****C****G****C****C****G**G..**G****U****A****A****C****C**CUUUACCUCU | |
|  |  | NZ\_AADW02000013.1/50941-50774  | GGACAGAAGUGAAAG**C****G****C****C**A**G****G****G****C****U****G****A****.****.**...CUAUGU.........GAUAG...........................................................................................................**.****.****G****C****A****G****C****U**GA**C****G****A**GGU............................................**G****G****G****G****G**UUUA**U****C****G****A**AUCAU.**U****C****G****G****C****G**GAUG.**C****C****U****C****C****C****G****G****U****A****C**......C**A****U****G****U****C****A**..**G****U****A****C****C****G**UAAAC**U****C****A****U****C****U****G**....AAAU.**C****G****U****U****U****A****A**GUGA.....**U****U****A****G****A****C****G**GACAG.G..**C****A****G****A****U****G****C**G..**U****G****A****U****G****A**CAACUCUCGA | |
|  |  | NZ\_AAAK03000020.1/30101-29944  | UACCUUUUAGGAUAG**C****G****C****C**A**G****G****U****C****U****G****U****.****.**...................GUAA...........................................................................................................**.****.****A****C****A****G****A****U**GA**C****G****A**GGAG...........................................**A****G****G****G****U**.UUA**U****C****G****A**AAGAU.**U****C****G****G****C****G**GAUG.**G****C****C****C****U****A****G****G****G****.****.**.......**A****C****U****G****C****A**..**.****.****C****U****C****U**ACA..**G****A****A****A****A****U****C**C.ACAAAA.**A****A****U****G****U****C****U**GCAA.....**A****G****A****U****A****.****A**AACAAAA.G**G****A****U****U****U****U****C**C..**U****G****C****A****G****C**UGGAAGCAUU | |
|  |  | NC\_006177.1/222192-222364  | UUCAGGAUCCGCAAG**C****G****C****C**A**G****G****A****C****U****U****G****C****G**GCC...............GGCGG........................................................................................................GGC**C****G****C****A****A****G****U****U**GA**C****G****A**GGU............................................**G****G****G****G****G**.AUC**U****C****G****G**AGGAU.**U****C****G****G****C****G**GGUG.**A****C****C****C****C****C****G****G****U****U****.**.......**G****C****U****C****A****C**..**.****G****A****C****C****G**UAA..**G****C****G****G****C****U****C**U.ACAAAG.**G****C****C****G****G****G****A**GCGA.....**U****C****C****C****G****G****A**GACAAAGGG**G****C****C****A****G****G****U**GA.**G****G****C****C****G****A**GGGACGGGCA | |
|  |  | NZ\_AAAH01000890.2/3528-3738  | CAAUCUGCUUUGAAG**C****G****C****A**U**G****G****A****C****U****U****C****G****C**..................GAGGA...........................................................................................................**G****C****G****A****A****G****U****U**GA**C****G****A**GGAGGGAGGUUCCCGGCGCGAGCCGGAGCUAACGGGUCUGAACGACC**C****G****G****A****A**C..A**U****C****G****A**.GAGA.**U****C****A****G****C****G**GAAGC**C****U****C****C****C****C****G****G****U****G****.**.....CA**G****G****G****C****A****C**..**.****C****A****C****C****G**AUA..**A****C****A****G****A****C****A**AACCAAUA.**C****C****U****G****C****C****G**GUGA.....**C****G****G****U****G****A****G**GGCAACG.U**U****G****U****C****U****G****U**A..**G****U****G****A****C****C**CACCACUAUC | |
|  |  | NC\_001263.1/302515-302702  | GACUUCUUCGGGCAG**C****G****C****A**A**G****G****C****C****C****C****G****G****C**GACACGU...........GAUGU......................................................................................................CACAA**G****C****C****G****G****G****G****A**GA**C****G****A**GGU............................................**G****G****A****G****G**.UCA**G****C****G****A**.CUUU.**U****C****U****G****C****G**GAUG.**C****C****U****C****C****A****G****G****C****C****C**.......**C****G****G****U****G****A**AC**G****G****G****C****C****U**ACCCG**G****C****G****C****G****U****G**C.UUUGCC.**G****C****U****C****U****G****A**GUCAAAGAC**U****C****C****G****G****C****A**GGCAGAA.C**C****A****C****G****C****G****C**AA.**G****C****C****C****G****G**CGAUAAGCCC | |
|  |  | NZ\_AAHE01000006.1/74295-74120  | CACACAAACGGGAAG**C****G****C****A**A**G****G****U****C****U****G****U****C****U**C.................AACAG................................................................................................GUCGUCCUGGA**A****G****G****C****A****G****A****A**GA**C****G****A**GGU............................................**G****G****A****G****G**.UGA**G****C****G****A**.GGGU.**U****C****U****G****C****G**GAUG.**C****C****U****C****C****A****G****G****U****C****C**.......**.****G****G****C****A****C**..**G****G****G****C****C****U**CCCC.**G****A****C****U****U****C****C**..CCGA...**.****C****U****U****G****A****G**GGGA.....**A****G****G****A****A****G****.**CGCGGCGAU**A****A****C****C****C****C****C**ACG**G****U****G****A****C****G**UGGACAUAAG | |
|  |  | AAFZ01014893.1/406-220  | AUUUUCUCUACGAAG**C****G****C****C**A**G****G****A****C****U****G****G****U****U**GUG...............AUUCG.............................................................................................GGUACUAUCCCGAU**G****A****C****C****A****G****U****U**GA**C****G****A**GGA............................................**G****A****G****G****G**CUUA**U****C****G****A**AGCAU.**U****C****G****G****C****G**GAUGG**C****U****C****U****C****C****G****G****C****C****U**.......**.****G****A****C****C****G**..**G****G****G****U****C****G**UAA..**C****A****G****C****A****C****C**G.ACAAAG.**U****C****U****G****C****G****G**GUGA.....**C****C****G****U****A****G****A**GACAAAG.C**G****G****U****G****U****C****G**AAU**C****G****G****U****A****U**CUCAUCACUC | |
|  |  | AACY01510269.1/91-248  | AACGAGCAAAUUAAG**C****G****C****C**A**G****G****C****C****U****C****G****.****.**....................AA............................................................................................................**.****.****A****G****A****G****G****U**GA**C****G****A**GGA............................................**A****G****U****A****U**GUUA**U****C****G****A**AAGAU.**U****C****G****G****C****G**GAUGC**A****U****A****C****U****C****G****G****U****U****.**.......**.****.****C****U****G****C**..**.****G****G****U****C****G**UAA..**U****U****A****A****U****U****G**U.CUAAAA.**A****C****A****G****A****A****A**GUAA.....**U****U****U****C****U****G****A**.ACAAAG.A**A****A****A****U****U****A****A**AA.**G****C****A****G****U****C**UUAUUUCAAC | |
|  |  | AACY01005023.1/1163-1006  | ACGAGCAAAUAUUAG**C****G****C****C**A**G****G****C****C****U****U****U****.****.**....................AA............................................................................................................**.****.****U****U****A****G****G****U**GA**C****G****A**GGA............................................**A****G****U****G****U**GUUA**U****C****G****A**AACAU.**U****C****G****G****C****G**GAUGC**A****C****A****C****U****C****G****G****C****U****.**.......**.****.****C****U****G****C**..**.****G****G****U****C****G**UAA..**U****A****A****A****U****U****G**U.UUAAAA.**A****C****A****G****G****A****A**GUAA.....**U****U****U****C****U****G****A**.ACAGAA.G**C****A****A****U****U****U****A**AA.**G****C****A****G****U****C**UUAUUUCAAC | |
|  |  | AACY01011387.1/1763-1920  | UAACGAGCAAUAUAG**C****G****C****C**U**G****G****C****C****U****U****U****.****.**....................AA............................................................................................................**.****.****C****G****A****G****G****U**GA**C****G****A**GGA............................................**A****G****U****A****U**GUUA**U****C****G****A**AAAAU.**U****C****G****G****C****G**GAUGC**A****U****A****C****U****C****G****G****C****U****.**.......**.****.****C****U****G****C**..**.****A****G****U****C****G**AUA..**U****A****A****A****U****U****G**U.UUAAAA.**A****C****A****G****A****G****A**GAAA.....**U****U****U****C****U****G****A**.ACAGAG.A**C****A****A****U****U****U****A**AA.**G****C****A****G****U****C**UUUAUUUUAA | |
|  |  | AACY01059580.1/1171-1014  | UUAACGAGCAAUUAG**C****G****C****C**A**G****G****C****C****U****U****G****.****.**....................AA............................................................................................................**.****.****A****A****A****G****G****U**GA**C****G****A**GGA............................................**A****G****U****A****U**GUUA**U****C****G****A**AACAU.**U****C****G****G****C****G**GAUGC**A****U****A****C****U****C****G****G****C****U****.**.......**.****.****C****U****G****C**..**.****A****G****U****C****G**UAA..**U****A****A****A****U****U****G**U.UUAAAA.**A****C****A****U****A****G****A**GAAA.....**U****C****U****G****U****G****A**.ACAGAG.A**C****A****A****U****U****U****A**CA.**G****C****A****G****U****C**UUUAUUUUAA | |
|  |  | AAFX01006262.1/185-18  | UGUCCGUACACAUAG**C****G****C****C**A**G****G****A****C****C****C****G****G****A**UCG.................AGG.........................................................................................................CU**U****U****C****G****G****G****U****U**GA**C****G****A**GGU............................................**G****G****G****G****A**UUUA**U****C****G****A**AUCAU.**U****C****G****G****C****G**GAUGA**U****C****C****C****C****C****G****G****C****.****.**.......**.****C****G****C****A****C**A.**.****.****G****U****C****G**UGAU.**G****A****U****G****C****G****C**..UCAAAC.**C****G****A****U****U****U****G**GCAA.....**C****A****G****A****U****C****G**.ACAAAU..**G****C****U****C****G****U****C**AGC**G****U****G****C****A****C**AUGGUUCAAG | |
|  |  | SS\_cons |  | ...............AAAA.A<<<<<<<<..................................................................................................................................>>>>>>>>..BBB...............................................<<<<<....bbb[......]aaaaa.....>>>>><<<<<<.......CCCCCC..>>>>>>.....<<<<<<<.........<<<<<<<.........>>>>>>>.........>>>>>>>...cccccc.......... |
|  |  | SS\_label |  | ...............=P2.1=---P1---..................................................................................................................................---P1---..P2.2..............................................==P2=....P2.2.......P2.1=.....=P2==--P3-.................-P3--.....===P4==.........--P4.1-.........--P4.1-.........==P4===................... |
|  |  | RF |  | gauauauaauaAAAGCGCCAGaaCuaaacgcc...............aauag...........................................................................................................guuuaGuuGACGAGGA............................................ggaGg.UUAuCGA.auuU.UCGgCGGAUG.cCucccGguu........agguca...aacCgUuAa.gcuuuuu..ACAAAA.caaaaagGUGA.....cuuuuuggACAAAg..gaaaagcAa.ggaaaguaaaaaaAua |
|  |  | SS\_align |  | :::::::::.::.::::::::<<<<<<<<\_\_\_...............\_\_\_\_\_...........................................................................................................>>>>>>>>,,,,,,,,............................................<<<<<.---<<<<.\_\_\_\_.>>>>------.>>>>><<<<<........\_\_\_\_\_\_...>>>>>,,,,.<<<<<<<..------.<<<<<<<\_\_\_\_.....>>>>>>>-------..>>>>>>>::.:::::::::::::::: |
