# Supplementary material for: The distributions, mechanisms, and structures of metabolite-binding riboswitches
Source: Genome Biol. 2007 Nov 12;8(11):R239. doi: 10.1186/gb-2007-8-11-r239 (PMC2258182; doi:10.1186/gb-2007-8-11-r239)
Supplement: Additional data file 2 — Sequence alignments of the riboswitch aptamer data sets annotated with new base-base interactions in HTML format. [file gb-2007-8-11-r239-S2.zip › HTML/Glycine.html]

|  |  |  |  |  |
| --- | --- | --- | --- | --- |
|  |  | **Accession/Start-End** |  | **Sequence** |
|  |  | NC\_002937.3/808242-808071  | AUGAC...**G****A**.**C**.**A**.**G**.**C****A****G****G****A****G**A**G**.A**C****C****U****C****C**CC.........CAGA.........................................G**G****G****A****G****G**..**C****G**.**C****C**GAC**G****A****A****G**UA...A..--................----........................--.-AU**C****U****U****U**CA**G****G****C**..........................................................................G.A.A.A.**G**.**G****A**.**C****U****G**..**C**..**U****G**.**U**.**C**.....CGACGAG-............................................................**C****C****U****C****U****G****G****A****G**A**G**A.**C**.**C****C****C**--...............................GCAA.......................................---**G****G****G**..**G****C****A****C****C**GAA**G****G****A****G**CAA..**G****C****C**..**G**.**C****C****C**-..........CAUG..........................................................................................-**U****G****G**..**C**.**G****G****U**.G.AAA.**C****U****C**.**U**CA**G****G****U**..............AAAA**G**.**G****A****C****A****G****A****G****G**GAGGGCC | |
|  |  | NC\_003366.1/1674771-1674599  | AUGAA...**G****G**.**U**.**A**.**A**.**U****G****G****G****A****G**A**G**.U**G****A****U****A****U**U..........UUAA.........................................-**A****U****A****U****C**..**C****A**.**C****C**GAA**G****A****G****G**GA...A..--................----........................--.-AU**C****U****U****U**CA**G****G****U**..........................................................................A.U.U.A.**G**.**G****A**.**C****C****G**..**U**..**U****A**.**C**.**U**.....GGACGAG-............................................................**C****C****U****C****U****G****G****A****G**A**G**A.**C**.**U****C****U****U****U**...............................UAAA.......................................-**A****A****A****G****A**..**G****C****A****C****C**GAA**G****G****A****G**CAA..**G****G****U**..**C**.**A****A****A**-..........UUU-..........................................................................................-**U****U****U**..**G**.**A****C****U**.G.AAA.**C****U****C**.**U**CA**G****G****U**..............AAAA**G**.**G****A****C****A****G****A****G****G**AUAAGGU | |
|  |  | NZ\_AABN02000002.1/274017-273819  | AUGAC...**G****A**.**C**.**A**.**G**.**C****A****G****G****A****G**A**G**.A**U****U****U****C****C**GCCGU......UCCACACCCCCGGA...............................C**G****G****A****A****A**..**C****G**.**C****C**GAA**G****A****A****G**UA...A..--................----........................--.-AU**C****U****U****U**CA**G****G****C**..........................................................................A.A.A.A.**G**.**G****A**.**C****U****G**..**C**..**U****G**.**U**.**U**.....UGACGAG-............................................................**C****C****U****C****U****G****G****A****G**A**G**A.**C**.**U****C****C****C**-...............................CCUGU......................................--**G****G****G****A**..**G****C****A****C****C**GAA**G****G****A****G**CAA..**G****C****C**..**G**.**G****U****C****G**U.........AGAU..................................................................................CUCCUCCA**C****G****A****C**..**C**.**G****G****U**.G.AAA.**C****U****C**.**U**CA**G****G****U**..............AAAA**G**.**G****A****C****A****G****A****G****G**GCCCUUU | |
|  |  | NC\_004557.1/2100681-2100496  | AUGAA...**G****G**.**U**.**A**.**A**.**U****A****G****G****A****G**A**G**.A**G****G****U****C**-...........UUUU.........................................--**G****A****C****C**..**C****A**.**C****C**GAA**G****A****U****G**CA...A..--................----........................--AAAU**C****U****U****U**CA**G****G****U**...........................................................ACCAUUGUUUAUGGC-.-.G.A.**G**.**G****A**.**C****U****G**..**U**..**U****A**.**U**.**U**.....GGACGAAA............................................................-**C****U****C****U****G****G****A****G**A**G**A.**C**.**U****C****U****U****U**U..............................UUAU.......................................A**A****A****A****G****A**..**G****C****A****C****C**GAA**G****G****A****G**CAA..**G****U****U**..**G**.**G****G**--..........UAAA..........................................................................................--**A****C**..**C**.**A****A****U**.G.AAA.**C****U****C**.**U**CA**G****G****U**..............AAAA**G**.**G****A****C****A****G****A****G****C**GUAGAAG | |
|  |  | NC\_002927.3/2944593-2944399  | ACGUG...**C****A**.**G**.**U**.**U**.**C****G****G****G****A****G**A**G**.A**C****C****G****U****C**C..........AGCG.........................................-**G****A****C****G****G**..**C****G**.**C****C**GAC**G****G****A****G**CA...A..**C****C**A...............CCCC........................**G****G**.AAA**C****U****C****U**CA**G****G****C**..........................................................................A.A.A.A.**G**.**G****A**.**C****C****G**..**A**..**C****C**.**U**.-.....GCUGGAA-............................................................**C****A****U****C****U****G****G****A****G**A**G**U.**G**.**G****C****G****C****G**CG.............................GUAC.......................................-**G****G****C****G****C**..**C****C****A****C****C**GAA**G****G****G****G**AU-..**C****C****C**..**U**.**G****G****C****G**CG........UUUG....................................................................................CAGCGG**C****G****C****A**..**C**.**G****G****G**UG.AAG.**C****U****C**.**U**CA**G****G****U**..............AAAU**G**.**G****A****C****A****G****A****U****G**GGGUUGC | |
|  |  | NC\_002928.3/2916058-2916252  | ACGUG...**C****A**.**G**.**U**.**U**.**C****G****G****G****A****G**A**G**.A**C****C****G****U****C**C..........AGCG.........................................-**G****A****C****G****G**..**C****G**.**C****C**GAC**G****G****A****G**CA...A..**C****C**A...............CCCC........................**G****G**.AAA**C****U****C****U**CA**G****G****C**..........................................................................A.A.A.A.**G**.**G****A**.**C****C****G**..**A**..**C****C**.**U**.-.....GCUGGAA-............................................................**C****A****U****C****U****G****G****A****G**A**G**U.**G**.**G****C****G****C****G**CG.............................GUAC.......................................-**G****G****C****G****C**..**C****C****A****C****C**GAA**G****G****G****G**AU-..**C****C****C**..**U**.**G****G****C****G**CG........UUUG....................................................................................CAGCGG**C****G****C****A**..**C**.**G****G****G**UG.AAG.**C****U****C**.**U**CA**G****G****U**..............AAAU**G**.**G****A****C****A****G****A****U****G**GGGUUGC | |
|  |  | NC\_002696.2/3682615-3682420  | AGGCC...**U****C**.**U**.**C**.**G**.**C****G****G****G****A****G**A**G**.A**U****C****G****G****G**C..........CUUG.........................................-**C****C****C****G****G**..**C****G**.**C****U**GAA**G****G****C****G**AA...A..**C****C**G...............CCCC........................**G****G**.AAA**C****G****C****U**CA**A****G****C**..........................................................................A.G.A.A.**G**.**G****A**.**C****C****G**..**C**..**G****C**.**G**.**A**GA...CGUUGAA-............................................................-**C****G****C****U****G****G****A****A**A**G**C.**A**.**G****A****G****C****G**...............................CGCG.......................................C**C****G****C****U****C**..**U****C****G****C****C**GAA**G****G****A****G**CAAG.**G****C****C**..**U**.**G****C****C****G**AC........UGUC......................................................................................UGGU**C****G****G****C**..**C**.**G****C****U**.G.AAU.**C****U****C**.**U**CA**G****G****C**.............GCCAA**G**.**G****A****C****A****G****C****G****G**GGGCAGA | |
|  |  | NZ\_AAIT01000002.1/370480-370654  | ACGGU...**C****U**.**G**.**G**.**A**.**C****A****G****G****A****G**A**G**.U**C****G****G****G****U**U..........CCC-.........................................-**A****C****C****C****G**..**C****G**.**C****C**GAC**G****G****A****G**CA...A..**C****G**................CCCC........................**G****G**.AAU**C****U****C****U**CA**G****G****C**..........................................................................A.C.C.A.**G**.**G****A**.**C****U****G**..-..-**C**.**C**.**G**.....GACCGCCA............................................................-**C****U****C****U****G****G****A****G**A**G**A.**G**.**A****U****G****C**-...............................GACA.......................................--**G****C****A****U**..**C****C****G****C****C**GAA**G****G****A****G**AAA..**G****C****C**..**G**.**C****G**--..........AAG-..........................................................................................--**C****G**..**C**.**G****G****C**.G.AUA.**C****U****C**.**U**CA**G****G****C**..............AAAA**G**.**G****A****C****A****G****A****G****G**GGGGAUG | |
|  |  | NC\_002505.1/1520656-1520426  | UUGAA...**G****A**.**C**.**U**.**G**.**C****A****G****G****A****G**A**G**.U**G****G****U****U****G**UUAACCAGAU.UUUAACAUCUGAGCCAA............................A**U****A****A****C****C**..**C****G**.**C****C**GAA**G****A****A****G**UA...A..--................----........................--.-AU**C****U****U****U**CA**G****G****U**......................................GCAUUAUUCUUAGCCAUAUAUUGGCAACGAAUAAGC-.-.G.A.**G**.**G****A**.**C****U****G**..**U**..**A****G**.**U**.**U**.....GGAGGAA-............................................................**C****C****U****C****U****G****G****A****G**A**G**A.**A**.**C****C****G****U**-...............................UUAA.......................................--**U****C****G****G**..**U****C****G****C****C**GAA**G****G****A****G**CAA..**G****C****U**..**C**.**U****G****C****G**..........CAUA..........................................................................................**U****G****C****A**..**G**.**A****G****U**.G.AAA.**C****U****C**.**U**CA**G****G****C**..............AAAA**G**.**G****A****C****A****G****A****G****G**AGUGAAA | |
|  |  | NC\_006840.1/1610602-1610817  | AUGAA...**G****A**.**C**.**U**.**G**.**C****A****G****G****A****G**A**G**.U**G****G****C****A****A**AAACCG.....UCAAGGU......................................U**A****C****G****C****C**..**C****G**.**C****C**GAA**G****A****A****G**UA...A..--................----........................--.-AU**C****U****U****U**CA**G****G****U**.............................................GCCGAGUUCAUUUUUUAAGUGAAUAAGGU-.-.G.A.**G**.**G****A**.**C****U****G**..**U**..**A****G**.**U**.**U**.....GGAGGAAU............................................................-**C****U****C****U****G****G****A****G**A**G**A.**G**.**C****C****G****U**-...............................UAAA.......................................--**U****C****G****G**..**C****C****G****C****C**GAA**G****G****A****G**CAA..**G****C****C**..**C**.**U****U****A****U**CUGAA.....AAAU.........................................................................................G**A****U****A****A**..**A**.**G****G****U**.G.AAA.**C****U****C**.**U**CA**G****G****C**..............AAAA**G**.**G****A****C****A****G****A****G****G**AGAAAGU | |
|  |  | NC\_002696.2/3603520-3603314  | UCGAC...**C****C**.**U**.**C**.**G**.**C****G****G****G****A****G**A**C**.A**U****C****G****G****G**A..........UUCGA........................................U**C****C****C****G****A**..**G****G**.**C****C**GAA**G****G****C****G**CA...A..**C****C**G...............CCCC........................**G****G**.AAA**C****G****C****U**CA**G****G****C**..........................................................................A.A.A.A.**G**.**G****A**.**C****C****G**..**C**..**G****C**.**G**.**G**G....UUUAGGAA............................................................-**C****G****C****U****G****G****A****A**A**G**C.**A**.**G****U****C****U****C**...............................UCCACCGG...................................A**G****G****G****G****C**..**U****C****G****C****C**GAA**G****G****A****G**CAA..**G****G****C**..**C**.**A****A****A****C**C.........CGUC................................................................................CGGCAGAGGG**G****C****G****A**..**G**.**G****C****C**GG.AAU.**C****U****C**.**U**CA**G****G****C**.............CCAAG**G**.**G****A****C****A****G****C****G****G**GGGCGAC | |
|  |  | NZ\_AAEH02000016.1/88178-88371  | UGAAA...**C****A**.**A**.**C**.**A**.**C****G****G****G****A****G**A**G**.A**C****U****G****U****C**AUCCG......AAUUGGAG.....................................U**G****A****C****A****G**..**C****G**.**C****C**GAC**G****G****A****G**CA...A..**C****C**G...............CCCC........................**G****G**.AAA**C****U****C****U**CA**G****G****C**..........................................................................A.A.A.A.**G**.**G****A**.**C****C****G**..**U**..**G****G**.**U**.**G**.....UCA-GAA-............................................................**C****A****U****C****U****G****G****A****G**A**G**A.**G**.**G****C****G****C**-...............................AUUG.......................................C-**G****C****G****C**..**A****C****A****C****C**GAA**G****G****G****G**AU-..**U****U****C**..**C**.**U****G****C****A**C.........AGGC..........................................................................................**U****G****C****A**..**G**.**G****A****G**AG.AAA.**C****U****C**.**U**CA**G****G****U**..............ACAU**G**.**G****A****C****A****G****A****U****G**GGGCAUC | |
|  |  | NC\_003454.1/964072-963902  | AUGAA...**G****A**.**U**.**A**.**U**.**G****A****G****G****A****G**A**G**.A**U****U****U****C****A**U..........UUUA.........................................A**U****G****A****A****A**..**C****A**.**C****C**GAA**G****A****A****G**UA...A..--................----........................--.-AU**C****U****U****U**CA**G****G****U**.........................................................................AA.A.A.A.**G**.**G****A**.**C****U****C**..**A**..**U****A**.**U**.**U**.....GGACGAA-............................................................**C****C****U****C****U****G****G****A****G**A**G**C.**U**.**U****A****U****C****U**...............................-AAG.......................................-**A****G****A****U****A**..**A****C****A****C****C**GAA**G****G****A****G**CAA..**A****G****C**..**U**.**A**---..........AUUU..........................................................................................---**U**..**A**.**G****C****C**.U.AAA.**C****U****C**.**U**CA**G****G****U**..............AAAA**G**.**G****A****C****G****G****A****G****U**AAUUGUG | |
|  |  | NC\_004459.1/2743819-2744048  | AUGAA...**G****G**.**C**.**U**.**A**.**C****A****G****G****A****G**A**G**.U**G****G****U****A****A**UUAACCAUAU.UUUAACAUUUGGUUAGU............................C**A****U****A****C****C**..**C****G**.**C****C**GAA**G****A****A****G**UA...A..--................----........................--.-AU**C****U****U****U**CA**G****G****U**.......................................GCAAUAUUCUUAUUGGUUAUAUCAAGAGAAUAUGC-.-.G.A.**G**.**G****A**.**C****U****G**..**U**..**A****G**.**U**.**U**.....GGAGGAA-............................................................**C****C****U****C****U****G****G****A****G**A**G**A.**A**.**C****C****G****U**-...............................UAAA.......................................--**U****C****G****G**..**U****C****G****C****C**GAA**G****G****A****G**CAA..**G****U****C**..**C**.**U****G****C****C**..........CAUG..........................................................................................**U****G****C****A**..**G**.**G****G****U**.G.AAA.**C****U****C**.**U**CA**G****G****C**..............AAAA**G**.**G****A****C****A****G****A****G****G**AGUGGAA | |
|  |  | NC\_005139.1/1639478-1639249  | AUGAA...**G****G**.**C**.**U**.**A**.**C****A****G****G****A****G**A**G**.U**G****G****U****A****A**UUAACCAUAU.UUUAACAUUUGGUUAGU............................C**A****U****A****C****C**..**C****G**.**C****C**GAA**G****A****A****G**UA...A..--................----........................--.-AU**C****U****U****U**CA**G****G****U**.......................................GCAAUAUUCUUAUUGGUUAUAUCAAAAGAAUAUGC-.-.G.A.**G**.**G****A**.**C****U****G**..**U**..**A****G**.**U**.**U**.....GGAGGAA-............................................................**C****C****U****C****U****G****G****A****G**A**G**A.**A**.**C****C****G****U**-...............................UAAA.......................................--**U****C****G****G**..**U****C****G****C****C**GAA**G****G****A****G**CAA..**G****U****C**..**C**.**U****G****C****C**..........CAUG..........................................................................................**U****G****C****A**..**G**.**G****G****U**.G.AAA.**C****U****C**.**U**CA**G****G****C**..............AAAA**G**.**G****A****C****A****G****A****G****G**AGUGGAA | |
|  |  | NC\_003155.3/8344959-8345170  | UGACC...**C****C**.**G**.**U**.**G**.**C****G****G****G****A****G**A**G**.U**C****C****U****C****C**GG.........AAGUCGU......................................C**G****G****A****G****G**..**C****G**.**C****C**GAA**G****G****A****G**CA...AAU**C****C**U...............CCCC........................**G****G**.AAU**C****U****C****U**CA**G****G****C**..........................................................................A.C.A.C.**G**.**U****A**.**C****C****G**..**C**..**A****C**.**G**.**G**A....CGAGGUCA............................................................-**C****U****C****U****G****G****A****A**A**G**C.**A**.**G****G****G****C****G**GAUGU..........................CUAUGGCUU..................................C**C****G****C****U****C**..**U****C****A****C****C**GAC**G****G****U****G**AAA..**G****C****C**..**G**.**G****A****G****C**GCCC......UCGG........................................................................................GC**G****G****A****C**..**C**.**G****G****C**.G.AAG.**C****U****C**.**U**CA**G****G****U**.............UGAGA**U**.**G****A****C****A****G****A****G****G**GGGAGGC | |
|  |  | NC\_003888.3/1457939-1457725  | UGACC...**C****C**.**G**.**C**.**G**.**C****G****G****G****A****G**A**G**.U**C****C****U****C****C**GGAC.......AUCAC........................................C**G****G****A****G****G**..**C****G**.**C****C**GAA**G****G****A****G**CA...AAU**C****C**U...............CCCC........................**G****G**.AAU**C****U****C****U**CA**G****G****C**..........................................................................U.C.A.C.**G**.**U****A**.**C****C****G**..**C**..**A****C**.**G**.**G**A....CGAGGUCA............................................................-**C****U****C****U****G****G****A****A**A**G**C.**A**.**G****G****G****C****G**GGUGUC.........................GACGGCUU...................................C**C****G****C****U****C**..**U****C****A****C****C**GAC**G****G****U****G**AAA..**G****C****C**..**G**.**G****G****C****A**G.........AGCU..................................................................................CCAGGGCU**C****G****C****C**..**C**.**G****G****U**.G.AAG.**C****U****C**.**U**CA**G****G****U**.............UGAGA**U**.**G****A****C****A****G****A****G****G**GGGAGGC | |
|  |  | NC\_004603.1/1855938-1856148  | AUGAA...**G****A**.**C**.**U**.**G**.**C****A****G****G****A****G**A**G**.U**G****G****U****U****A**UUAACCAAAC.UUUAACAUUUGGUUAGA............................U**U****A****A****C****U**..**C****G**.**C****C**GAA**G****A****A****U**UA...A..--................----........................--.-CU**A****U****U****U**CA**G****G****U**............................................................GCUACCUUGGUAGC-.-.G.G.**G**.**G****A**.**C****U****G**..**U**..**A****G**.**U**.**U**.....GGAGGAA-............................................................**C****C****U****C****U****G****G****A****G**A**G**A.**A**.**C****C****G****U**-...............................UAAA.......................................--**U****C****G****G**..**U****C****G****C****C**GAA**G****G****A****G**CAA..**G****U****C**..**C**.**U****G****C****A**C.........AUGU.........................................................................................G**U****G****C****G**..**G**.**G****G****U**.G.AAA.**C****U****C**.**U**CA**G****G****C**..............AAAA**G**.**G****A****C****A****G****A****G****G**AGUGGAA | |
|  |  | NC\_002663.1/115965-116172  | AUGAA...**G****G**.**U**.**A**.**G**.**C****U****G****G****A****G**A**G**.-**U****A****G****G****G**AA.........UUGA.........................................-**C****C****C****U****A**..**C****G**.**C****C**GAC**G****A****G****G**UA...A..--................----........................--.ACU**C****U****U****U**CA**G****G****C**.............................................................GCUGAUUAUAAGC-.-.A.G.**G**.**G****A**.**C****U****G**..**U**..**U****A**.**C**.**U**.....GGACGAA-............................................................**C****C****C****U****U****G****G****A****G**A**G**A.**G**.**C****C****G****U****U**AUA............................UUAAAAAGAGAUA..............................A**A****A****C****G****G**..**C****C****G****C****C**GAA**G****G****C****G**CAA..**A****A****A**..**G**.**A****G****C****G**GUU.......AAUU......................................................................................UUUC**C****G****U****U**..**U**.**U****U****U**.C.AAA.**C****G****C**.**U**CA**G****G****C**..............AAAA**G**.**G****A****C****A****G****G****G****G**CAACAAG | |
|  |  | NC\_005085.1/3728361-3728158  | CCGGG...**U****C**.**A**.**U**.**A**.**C****A****G****G****A****G**A**G**.A**G****C****G****G****C**U..........UUCU.........................................G**G****C****C****G****C**..**C****G**.**C****C**GAA**G****G****C****G**CA...A..**G****C**G...............CACC.......................C**G****C**.AAU**C****G****C****U**CA**G****G****C**..........................................................................A.A.A.A.**G**.**G****A**.**C****U****G**..**U**..**A****U**.**C**.**A**.....UCGGGCCGGCGGCAGCCGGUUCGCGCA.........................................-**A****U****C****U****G****G****A****G**A**G**C.**G**.**G****C****G****U****C**...............................CGCG.......................................C**G****A****C****G****C**..**C****C****A****C****C**GAA**G****G****G****G**CUAA.**C****G****G**..**C**.**U**---..........UAUC..........................................................................................**C**--**G**..**G**.**C****C****G**.G.AAA.**A****U****C**.**U**CA**G****G****U**..............GCAG**G**.**G****A****C****A****G****A****G****G**GGUGUGG | |
|  |  | NZ\_AAAP01003811.1/3058-2840  | AUGAU...**C****C**.**C**.**C**.**G**.**U****G****G****G****A****G**A**G**.A**C****C****G****G****C**CUGA.......AAUUCA.......................................G**G****U****U****G****G**..**C****G**.**C****C**GAA**G****G****A****G**CA...A..**C****C**G...............CCCC.......................U**G****G**.AAA**C****U****C****U**CA**G****G****C**..........................................................................A.A.A.A.**G**.**G****A**.**C****C****G**..**C**..**G****G**.**G**.**G**.....GGAUGAAA............................................................-**C****U****C****U****G****G****A****A**A**G**C.**G**U**G****C****C****G****C**AGGACCGGC......................UAAGGUCG...................................G**G****C****G****G****C**..**C****C****A****C****C**GAC**G****A****U****G**AAA..**G****C****C**..**G**.**C****C****U****U**GGCCUCUC..UUUU.......................................................................................GGC**C****G****U****G**..**C**.**G****G****U**.G.AAU.**C****U****U**.**U**CA**G****G****U**..............UCCC**A**.**G****A****C****A****G****A****G****G**GGGGCAA | |
|  |  | NZ\_AAAJ03000004.1/332455-332239  | AUGCA...--.**G**.**C**.**G**.**C****G****G****G****A****G**A**G**.A**U****C****G****U****C**AGA........UGUUCG.......................................U**G****A****C****G****A**..**C****G**.**C****C**GAC**G****G****A****G**CA...A..**C****C**G...............CCCC........................**G****G**.AAA**C****U****C****U**CA**G****G****C**..........................................................................A.A.A.A.**G**.**G****A**.**C****C****G**..**U**..**G****C**.**A**.-.....GCAGGAA-............................................................**C****A****U****C****U****G****G****A****G**A**G**C.**G**.**G****C****G****C****A**UCGG...........................CACAGCUU...................................G**U****G****C****G****C**..**C****C****A****C****C**GAA**G****G****G****G**--A..**U****U****C**..**C**.**C****C****G****C**AC........ACGG......................................................................CUCGCAUCGGGCCGGCCGCC**G****C****G****G**..**A**.**G****A****A**.G.AAA.**C****U****C**.**U**CA**G****G****U**..............ACAU**G**.**G****A****C****A****G****A****U****G**GGGCAUC | |
|  |  | NC\_003888.3/5959095-5958886  | UGAAU...**C****C**.**G**.**C**.**G**.**C****G****G****G****A****G**A**G**.U**C****C****C****C****G**GC.........CGCG.........................................C**C****G****G****G****G**..**C****G**.**C****C**GAA**G****G****A****G**CA...AG.**U****C**CC..............UCCC......................UU**G****A**.-AU**C****U****C****U**CA**G****G****C**..........................................................................A.C.C.G.**U**.**U****A**.**C****C****G**..**C**..**G****C**.**G**.**G**G....CGAGGCA-............................................................**C****A****U****C****U****G****A****A****A**A**G**C.**G**.**G****A****C****C****G**CCCCC..........................GACG.......................................G**C****G****G****U****C**..**C****C****A****C****C**CAA**G****G****U****G**CAA..**G****C****C**..**C**.**U****G****A****U**CGCCG.....UACU....................................................................................CCGGUG**G****C****C****G**..**U**.**G****G****C**.G.AAC.**C****U****C**.**U**CA**G****G****U**.............UCCGA**U**.**G****A****C****A****G****A****U****G**GGGAGGA | |
|  |  | NC\_003030.1/1622059-1621882  | AUGAA...**G****U**.**U**.**A**.**G**.**C****G****G****G****A****G**A**G**.C**U****U****U****G****G**C..........UUUU.........................................G**C****C****A****U****A**..**C****A**.**C****C**GAA**G****A****A****G**UA...A..--................----........................--.-AU**C****U****U****U**CA**G****G****U**...........................................................AUCUAUUUAAUUAGA-.-.G.A.**U**.**G****A**.**C****C****G**..**C**..**U****A**.**U**.**U**.....GGAUGAA-............................................................**C****C****C****U****U****G****G****A****G**A**G**A.**C**.**U****C****U**--...............................-UAA.......................................---**A****G****A**..**G****C****A****C****C**GAA**G****G****A****G**AAA..**G****C****A**..**U**.**A**---..........AAA-..........................................................................................---**A**..**A**.**A****G****C**.G.AAA.**C****U****C**.**U**CA**G****G****U**..............AAAA**G**.**G****A****C****A****G****G****G****G**ACAGAUA | |
|  |  | NC\_007146.1/992923-992732  | AUGAA...**G****G**.**U**.**A**.**G**.**C****U****G****G****A****G**A**G**.-**C****G****G****G****G**AA.........UUGA.........................................-**C****C****C****C****A**..**C****A**.**C****C**GAC**G****A****U****G**UA...A..--................----........................--.AAU**C****U****U****U**CA**G****G****U**..................................................................GCGAUGGC-.-.A.G.**G**.**G****A**.**C****U****G**..**U**..**U****A**.**C**.**U**.....GGACGAA-............................................................**C****C****C****U****U****G****G****A****G**A**G**A.**U**.**C****C****A****U****U**...............................UUAG.......................................A**A****A****U****G****G**..**A****C****G****C****C**GAA**G****G****C****G**CAA..**A****A****G**..**A**.**G****C****G****G**UU........AAUU....................................................................................UUUCAA**U****C****G****U**..**U**.**U****U****U**.C.AAA.**C****G****C**.**U**CA**G****G****C**..............AAAA**G**.**G****A****C****A****G****G****G****G**CAAAAGA | |
|  |  | NZ\_AADO01000001.1/277409-277600  | AUGAA...**G****G**.**U**.**A**.**G**.**C****U****G****G****A****G**A**G**.-**C****G****G****G****G**AA.........UUGA.........................................-**C****C****C****C****A**..**C****A**.**C****C**GAC**G****A****U****G**UA...A..--................----........................--.AAU**C****U****U****U**CA**G****G****U**..................................................................GCGAUGGC-.-.A.G.**G**.**G****A**.**C****U****G**..**U**..**U****A**.**C**.**U**.....GGACGAA-............................................................**C****C****C****U****U****G****G****A****G**A**G**A.**U**.**C****C****A****U****U**...............................UUAG.......................................A**A****A****U****G****G**..**A****C****G****C****C**GAA**G****G****C****G**CAA..**A****A****G**..**A**.**G****C****G****G**UU........AAUU....................................................................................UUUCAA**U****C****G****U**..**U**.**U****U****U**.C.AAA.**C****G****C**.**U**CA**G****G****C**..............AAAA**G**.**G****A****C****A****G****G****G****G**CAAAAGA | |
|  |  | NZ\_AAET01000260.1/481-672  | AUGAA...**G****G**.**U**.**A**.**G**.**C****U****G****G****A****G**A**G**.-**C****G****G****G****G**AA.........UUGA.........................................-**C****C****C****C****A**..**C****A**.**C****C**GAC**G****A****U****G**UA...A..--................----........................--.AAU**C****U****U****U**CA**G****G****U**..................................................................GCGAUGGC-.-.A.G.**G**.**G****A**.**C****U****G**..**U**..**U****A**.**C**.**U**.....GGACGAA-............................................................**C****C****C****U****U****G****G****A****G**A**G**A.**U**.**C****C****A****U****U**...............................UUAG.......................................A**A****A****U****G****G**..**A****C****G****C****C**GAA**G****G****C****G**CAA..**A****A****G**..**A**.**G****C****G****G**UU........AAUU....................................................................................UUUCAA**U****C****G****U**..**U**.**U****U****U**.C.AAA.**C****G****C**.**U**CA**G****G****C**..............AAAA**G**.**G****A****C****A****G****G****G****G**CAAAAGA | |
|  |  | NC\_002678.2/707689-707879  | UACCU...**G****U**.**U**.**U**.**G**.**C****G****G****G****A****G**A**G**.A**G****C****A****G****C**G..........AGA-.........................................-**G****C****U****G****C**..**C****G**.**C****C**GAA**G****G****G****G**AA...A..**U****C**................GCCC........................**G****A**.AAU**C****U****C****U**CA**G****G****C**..........................................................................A.A.A.A.**G**.**A****A**.**C****C****G**..**U**..**A****G**.**A**.**C**GG...GAAAGACA............................................................-**C****U****C****U****G****G****A****A**A**G**U.**C**.**G****G****G****G**-...............................CUUG.......................................C-**C****C****C****C**..**G****C****G****C****C**GAA**G****G****U****G**UAA..**G****C****G**..**C**.**C****G****C****U**GACAG.....AGUU........................................................................................CC**G****G****U****U**..**G**.**C****G****C**.G.AGU.**C****U****C**.**U**CA**G****G****C**..............UUCA-.**G****A****C****A****G****A****G****G**GGCACGG | |
|  |  | NC\_002927.3/901485-901718  | ACGCA...**U****G**.**U**.**C**.**G**.**C****G****G****G****A****G**A**G**.A**G****C****G****G****C**CG.........AUUGC........................................G**G****C****U****G****C**..**C****G**.**C****C**GAA**G****G****C****G**CA...AU.**U****C**................GCCC........................**G****G**.AAU**C****G****C****U**CA**G****G****U**..........................................................................A.ACC.C.**A**.**U****A**.**C****C****G**..**C**..**G****A**.**C**.**U**.....GCAUCGAGUAGCGCCUGGCGCGCUCGAAACAGCA..................................-**C****U****C****U****G****G****A****G**A**G**A.**C**.**C****U****G****G****C**GCGGCCACCUUCGCGGUGCG...........CAAGCA.....................................G**C****C****C****A****G**..**G****C****G****C****C**GAA**G****G****U****G**CAAA.**C****C****C**..**G**.**C**---..........UCGC..........................................................................................---**G**..**C**.**G****G****G**.G.CAA.**C****U****C**.**U**CA**G****G****C**..............AAAA**G**.**G****A****C****A****G****A****G****G**GGCGGAA | |
|  |  | NC\_002928.3/817544-817777  | ACGCA...**U****G**.**U**.**C**.**G**.**C****G****G****G****A****G**A**G**.A**G****C****G****G****C**CG.........AUUGC........................................G**G****C****U****G****C**..**C****G**.**C****C**GAA**G****G****C****G**CA...AU.**U****C**................GCCC........................**G****G**.AAU**C****G****C****U**CA**G****G****U**..........................................................................A.ACC.C.**A**.**U****A**.**C****C****G**..**C**..**G****A**.**C**.**U**.....GCAUCGAGUAGCGCCUGGCGCGCUCGAAACAGCA..................................-**C****U****C****U****G****G****A****G**A**G**A.**C**.**C****U****G****G****C**GUGGCCACCUUCGCGGUGCG...........CAAGCA.....................................G**C****C****C****A****G**..**G****C****G****C****C**GAA**G****G****U****G**CAAA.**C****C****C**..**G**.**C**---..........UCGC..........................................................................................---**G**..**C**.**G****G****G**.G.CAA.**C****U****C**.**U**CA**G****G****C**..............AAAA**G**.**G****A****C****A****G****A****G****G**GGCGGAA | |
|  |  | NC\_002929.2/197088-197321  | ACGCA...**U****G**.**U**.**C**.**G**.**C****G****G****G****A****G**A**G**.A**G****C****G****G****C**CG.........AUUGC........................................G**G****C****U****G****C**..**C****G**.**C****C**GAA**G****G****C****G**CA...AU.**U****C**................GCCC........................**G****G**.AAU**C****G****C****U**CA**G****G****U**..........................................................................A.ACC.C.**A**.**U****A**.**C****C****G**..**C**..**G****A**.**C**.**U**.....GCAUCGAGUAGCGCCUGGCGCGCUCGAAACAGCA..................................-**C****U****C****U****G****G****A****G**A**G**A.**C**.**C****U****G****G****C**GCGGCCACCUUCGCGGUGCG...........CAAGCA.....................................G**C****C****C****A****G**..**G****C****G****C****C**GAA**G****G****U****G**CAAA.**C****C****C**..**G**.**C**---..........UCGC..........................................................................................---**G**..**C**.**G****G****G**.G.CAA.**C****U****C**.**U**CA**G****G****C**..............AAAA**G**.**G****A****C****A****G****A****G****G**GGCGGAA | |
|  |  | NZ\_AAAJ03000016.1/157954-157765  | AUGAA...**U****C**.**G**.**C**.**G**.**C****G****G****A****A****G**A**G**.C**C****C****A****G****C**A..........UCCCGGA......................................U**G****C****U****G****G**..**C****G**.**C****C**GAC**G****G****A****G**CA...A..**C****C**G...............CCCC........................**G****G**.AAA**C****U****C****U**CA**G****G****C**..........................................................................A.ACC.A.**G**.**G****A**.**C****C****G**..**C**..**G****C**.**G**.**A**.....UCAGGACA............................................................-**A****U****C****U****G****G****A****G**A**G**A.**G**.**G****C****G****C**-...............................-GAG.......................................--**G****C****G****C**..**C****C****A****C****C**GAA**G****G****G****G**AA-..**A****C****C**..**G**.**G****C****U****C**A.........UGCA........................................................................................CG**C****C****G****C**..**C**.**G****G****U**.C.AAG.**C****U****C**.**U**CA**G****G****U**..............ACCC**G**.**U****A****C****A****G****A****U****G**GGGUGUC | |
|  |  | NC\_005085.1/2193587-2193399  | UCCGG...**U****U**.**C**.**U**.**G**.**C****G****G****G****A****G**A**G**.A**G****G****G****G****C**...........CAC-.........................................-**G****C****C****C****C**..**C****G**.**C****C**GAA**G****A****C****G**CA...A..**G****C**................UCCC........................**A****U**.AAU**C****G****C****U**CA**G****G****C**..........................................................................A.A.C.C.**G**.**U****A**.**C****C****G**..**C**..**A****G**.**C**.**G**CC...GUAUAGAAUCAAGC......................................................**C****G****A****U****U****G****G****A****G**A**G**A.**G**.**G****C****C****G****C**...............................CCCG.......................................C**G****C****G****G****C**..**C****C****A****C****C**GAA**G****G****G****G**CAA..**G****U****G**..**G**.**C****C**--..........UAA-..........................................................................................--**G****G**..**C**.**C****G****C**.G.CAA.**C****U****C**.**U**CA**G****G****U**.............AAAAA**G**.**G****A****C****A****A****G****G****G**GAGAGGC | |
|  |  | NC\_003155.3/3402031-3402235  | UGAAU...**C****C**.**G**.**C**.**G**.**C****G****G****G****A****G**A**G**.U**U****C****C****G****G**G..........UACGUGUG.....................................C**C****C****G****G****A**..**C****G**.**C****C**GAA**G****G****A****G**CA...AG.**U****C**CC..............UCCC......................UU**G****A**.-AU**C****U****C****U**CA**G****G****C**..........................................................................C.C.C.G.**U**.**U****A**.**C****C****G**..**C**..**G****C**.**G**.**G**G....CGAGGCA-............................................................**C****A****U****C****U****G****A****A****A**A**G**C.**G**.**G****G****C****C****G**CUGU...........................CCAG.......................................-**U****G****G****C****U**..**C****C****A****C****C**CAA**G****G****U****G**CAA..**G****C****C**..**A**G**U****G****A****C**CCG.......UGAC.........................................................................................G**G****U****C****A**..**U**.**G****G****C**.G.AAC.**C****U****C**.**U**CA**G****G****U**.............UCCGA**U**.**G****A****C****A****G****A****U****G**GGGAGGA | |
|  |  | NC\_005966.1/1990167-1989965  | AUGAU...**U****A**.**U**.**U**.**G**.**C****A****G****G****A****G**A**G**.A**U****A****U****U****C**U..........UGCAA........................................A**G****G****A****U****A**..**C****G**.**C****C**GAA**G****G****A****G**CA...A..**C****G**A...............CCCC........................**G****G**.AAA**C****U****C****U**CA**G****G****C**..........................................................................A.G.A.A.**G**.**G****A**.**C****U****G**..**U**..**A****A**.**U**.**A**AUC..GAUCUAUA............................................................-**A****U****C****U****G****G****A****G**A**G**A.**A**.**G****U****A****U****U**U..............................CAAA.......................................-**A****A****U****A****C**..**U****C****A****C****C**GAA**G****G****G****G**AU-..**G****G****U**..**A**.**C****G****U****A**G.........AUUU...............................................................................AUGGAAAUCUA**U****A****G****C**..**U**.**A****C****C**.G.AAG.**C****U****C**.**U**CA**G****G****U**..............ACCC**U**.**G****A****C****A****G****A****U****G**GGGCAAC | |
|  |  | NZ\_AAHG01000002.1/247427-247224  | UGAUC...**U****G**.**C**.**G**.**G**.**C****G****G****G****A****G**A**G**.U**C****C****U****G****C**CGGUACG....UUCA.........................................-**G****C****A****G****G**..**C****G**.**C****C**GUA**G****G****A****G**CA...AAU**C****C**U...............CCCC.......................A**G****G**.AAU**C****U****C****U**CA**G****G****C**..........................................................................C.C.A.C.**G**.**U****A**.**C****C****G**..**C**..**C****G**.**C**.**G**G....CGAGGCAA............................................................-**C****U****C****U****G****G****A****A**A**G**U.**A**.**G****U****C****C****G**...............................GUCC.......................................C**C****G****G****G****C**..**U****C****A****C****C**GAC**G****G****U****G**CAA..**G****C****G**..**G**.**C****A****C****G**CUG.......UAAC......................................................................................AGCA**G****G****G****U**..**C**.**C****G****C**GG.AAA.**C****U****C**.**U**CA**G****G****U**..............CAAA**U**.-**A****C****A****G****A****G****C**GGGGAGG | |
|  |  | NZ\_AAFH01000002.1/542640-542422  | GUUCC...**C****G**.**U**.**U**.**G**.**C****G****G****G****A****G**A**G**.C**G****C****A****G**-...........CCCAG........................................C-**C****U****G****C**..**C****G**.**C****C**GAA**G****G****C****G**UA...A..**U****C**................ACCC........................**G****G**.AAU**C****G****C****U**CA**G****G****C**..........................................................................A.A.A.A.**G**.**G****A**.**C****C****G**..**C**..**A****A**.**C**.**A**C....CCAGGACAAUCCUGGGCACACA..............................................-**C****U****C****U****G****G****A****G**A**G**C.**G**.**C****A****G****U****C**GCCUUCUU.......................GAAGCGC....................................G**A****A****C****U****G**..**C****C****A****C****C**GAA**G****G****G****G**CAG..**C****G****G**..**C**.**G****C****C****G**G.........UUGA....................................................................................UCAACC**U****G****G****C**..**A**.**C****C****G**.U.AAU.**C****U****C**.**U**CA**G****G****U**.............ACCAA**G**.**G****A****C****A****G****A****G****G**GGUUCAC | |
|  |  | NZ\_AADP01000001.1/356993-356802  | AUGAA...**G****G**.**U**.**A**.**G**.**C****U****G****G****A****G**A**G**.-**C****G****G****G****A**AA.........UUGA.........................................-**C****C****C****C****A**..**C****A**.**C****C**GAC**G****A****U****G**UA...A..--................----........................--.AAU**C****U****U****U**CA**G****G****U**..................................................................GCGAUGGC-.-.A.G.**G**.**G****A**.**C****U****G**..**U**..**U****A**.**C**.**U**.....GGACGAA-............................................................**C****C****C****U****U****G****G****A****G**A**G**A.**U**.**C****C****A****U****U**...............................UUAG.......................................A**A****A****U****G****G**..**A****C****G****C****C**GAA**G****G****C****G**CAA..**A****A****G**..**A**.**G****C****G****G**UU........AAUU....................................................................................UUUCAA**U****C****G****U**..**U**.**U****U****U**.C.AAA.**C****G****C**.**U**CA**G****G****C**..............AAAA**G**.**G****A****C****A****G****G****G****G**CAAAAGA | |
|  |  | NC\_004757.1/660005-660207  | UUCAU...**C****C**.**A**.**G**.**G**.**C****A****G****G****A****G**A**G**.U**G****C****G****C****U**GU.........AUCAAAAUA....................................C**A****G****U****G****U**..**C****A**.**C****C**GAA**G****G****C****G**UA...A..**C****C**................CCCC........................**G****G**.AAU**C****G****C****U**CA**G****G****U**...........................................................GUGGCCAUAAGGCUU-.-.G.A.**G**.**U****A**A**C****U****G**..**C**..**U****U**.**G**.**U**.....GAUUGGCA............................................................-**A****U****C****U****G****G****A****G**A**G**U.**G**.**C****U****G****A****A**...............................ACAG.......................................C**U****U****C****A****G**..**C****C****A****C****C**GAA**G****G****G****G**CA-..**U****G****C**..**G**.**G****A****A**-..........AGUU..........................................................................................-**G****A****C**..**C**.**G****U****A**.-.AAA.**C****U****C**.**U**CA**G****G****U**..............AAAA**G**.**G****A****C****A****G****A****G****G**GGUAAGU | |
|  |  | NC\_003318.1/584390-584575  | UGUCU...**G****U**.**U**.**U**.**G**.**C****G****G****G****A****G**A**G**.A**G****C****C**--G..........UUAA.........................................---**G****G****C**..**C****G**.**C****C**GAA**G****G****G****G**AA...A..**A****C**................GCCC........................**G****A**.AAU**C****U****C****U**CA**G****G****U**..........................................................................A.C.A.A.**G**.**G****A**A**C****C****G**..**C**..**A****G**.**G**.**C**GGG..UAAGACAA............................................................-**C****U****C****U****G****G****A****A**A**G**U.**C**.**G****G****G****G**-...............................GCAA.......................................--**C****U****C****C**..**G****C****G****C****C**GAA**G****G****U****G**UAA..**G****U****A**..**U**.**G****G****C****U**U.........UAUA.......................................................................................UAU**A****G****C****C**..**A**.**U****G****C**.G.AGU.**C****U****C**.**U**CA**G****G****C**..............-CUG**A**.**G****A****C****A****G****A****G****G**GGCACGA | |
|  |  | NC\_004311.2/713403-713218  | UGUCU...**G****U**.**U**.**U**.**G**.**C****G****G****G****A****G**A**G**.A**G****C****C**--G..........UUAA.........................................---**G****G****C**..**C****G**.**C****C**GAA**G****G****G****G**AA...A..**A****C**................GCCC........................**G****A**.AAU**C****U****C****U**CA**G****G****U**..........................................................................A.C.A.A.**G**.**G****A**A**C****C****G**..**C**..**A****G**.**G**.**C**GGG..UAAGACAA............................................................-**C****U****C****U****G****G****A****A**A**G**U.**C**.**G****G****G****G**-...............................GCAA.......................................--**C****U****C****C**..**G****C****G****C****C**GAA**G****G****U****G**UAA..**G****U****A**..**U**.**G****G****C****U**U.........UAUA.......................................................................................UAU**A****G****C****C**..**A**.**U****G****C**.G.AGU.**C****U****C**.**U**CA**G****G****C**..............-CUG**A**.**G****A****C****A****G****A****G****G**GGCACGA | |
|  |  | NC\_006933.1/503740-503925  | UGUCU...**G****U**.**U**.**U**.**G**.**C****G****G****G****A****G**A**G**.A**G****C****C**--G..........UUAA.........................................---**G****G****C**..**C****G**.**C****C**GAA**G****G****G****G**AA...A..**A****C**................GCCC........................**G****A**.AAU**C****U****C****U**CA**G****G****U**..........................................................................A.C.A.A.**G**.**G****A**A**C****C****G**..**C**..**A****G**.**G**.**C**GGG..UAAGACAA............................................................-**C****U****C****U****G****G****A****A**A**G**U.**C**.**G****G****G****G**-...............................GCAA.......................................--**C****U****C****C**..**G****C****G****C****C**GAA**G****G****U****G**UAA..**G****U****A**..**U**.**G****G****C****U**U.........UAUA.......................................................................................UAU**A****G****C****C**..**A**.**U****G****C**.G.AGU.**C****U****C**.**U**CA**G****G****C**..............-CUG**A**.**G****A****C****A****G****A****G****G**GGCACGA | |
|  |  | NC\_004347.1/880823-881061  | AUGAA...**G****G**.**U**.**A**.**G**.**C****A****G****G****A****G**A**G**.-**U****G****G****G****G**A..........AUUA.........................................A**C****C****C****C****A**..**C****A**.**C****C**GAC**G****A****G****G**CA...A..**C****U**UUGUUGUUGGAAGCAUUCCA.................UAAACAA**A****G**CACU**C****U****U****U**CA**G****G****U**................................................................GCCGCAAGGC-.-.G.U.**G**.**G****A**.**C****U****G**..**U**..**U****A**.**C**.**U**.....GGACGAG-............................................................**C****C****U****C****U****G****G****A****G**A**G**A.**C**.**U****A****C****C****G**AUUGCAUUAACUUGCAA..............UAAU.......................................-**A****G****G****U****G**..**G****C****G****C****C**GAA**G****G****C****G**AAA..**G****U****G**..**U**.**C****G****C****U**GUG.......UUGU........................................................................................UA**A****G****C****G**..**A**.**C****G****C**.G.AAA.**C****G****C**.**U**CA**G****G****C**..............AAAA**G**.**G****A****C****A****G****A****G****G**AGAGGAU | |
|  |  | NC\_003869.1/1826408-1826220  | AUGAA...**G****A**.**A**.**U**.**G**.**C****G****G****G****A****G**A**G**.A**C****C****C**--...........UAAC.........................................C--**G****G****G**..**C****G**.**C****C**GAA**G****G****A****G**CA...A..**G****C**GGGUAUAUG.......GCCU................GUAUACUC**G****U**GAAA**C****U****C****U**CA**G****G****C**..........................................................................A.A.A.A.**G**.**G****A**.**C****C****G**..**C**..**A****U**.**U**.**C**.....GGACCAUA............................................................-**U****C****C****C****G****G****A****A**A**G**-.**C**.**C****U****C**--...............................UAAA.......................................---**G****A****G**..**G****C****A****C****C**GAA**G****G****A****G**CAA..**U****U****C**..**U**.**U****C**--..........UAU-..........................................................................................--**A****A**..**A**.**G****A****A**.G.AAU.**C****U****C**.**U**CA**G****G****U**..............AAAC**A**.**G****A****C****G****G****G****G****G**AAUAAAA | |
|  |  | NZ\_AAGG01000003.1/199574-199772  | ACGG-...**A****C**.**C**.**A**.**G**.**C****G****G****G****A****G**A**G**.U**U****C****G****C****G**C..........UUCGCG.......................................G**C****G****C****G****A**..**C****G**.**C****C**GAA**G****G****A****G**CA...A..**C****C**G...............CCCC........................**G****G**.AAU**C****U****C****U**CA**G****G****C**..........................................................................C.C.A.C.**G**.**G****A**.**C****C****G**..**C**..**U****G**.**G**.**A**CC...GGCUGACA............................................................-**C****U****C****U****G****G****A****A**A**G**C.**G**.**C****A****G****C****G**GCUUU..........................UGCGG......................................C**C****A****A****U****G**..**C****C****A****C****C**GAA**G****G****G****G**GAA..**G****U****G**..**C**.**G****G****A****U**C.........ACCC.........................................................................................G**G****C****C****C**..**G**.**C****A****C**.G.AUG.**C****U****C**.**U**CA**G****G****U**..............UUCC**C**.**G****A****C****A****G****A****G****G**GGGCGGA | |
|  |  | NZ\_AACJ01000012.1/32264-32068  | AUGAA...**G****G**.**U**.**A**.**G**.**C****U****G****G****A****G**A**A**.-**U****G****G****G****G**AA.........UUGA.........................................-**C****C****C****C****A**..**A****A**.**C****C**GAC**G****A****G****G**UA...A..--................----........................--.AAU**C****U****U****U**CA**G****G****U**..................................................................GCGAUGGC-.-.G.A.**G**.**G****A**.**C****U****G**..**U**..**U****A**.**C**.**U**.....GGACGAA-............................................................**C****C****C****U****U****G****G****A****G**A**G**A.**U**.**C****C****A****A****U**GUG............................CAAUGCACA..................................A**A****A****U****G****G**..**A****C****G****C****C**GAA**G****G****C****G**CAA..**A****A****G**..**A**.**G****C****G****G**UG........UUUU.......................................................................................UUA**C****U****G****C**..**U**.**U****U****U**.C.AAA.**C****G****C**.**U**CA**G****G****C**..............AAAA**G**.**G****A****C****A****G****G****G****G**CAAAAGA | |
|  |  | NZ\_AACK01000035.1/11103-11291  | AUGAA...**G****G**.**U**.**A**.**G**.**C****U****G****G****A****G**A**A**.-**G****A****G****C**-G..........AUAA.........................................--**G****C****U****C**..**U****A**.**C****C**GAC**G****A****G****G**UA...A..--................----........................--.AAU**C****U****U****U**CA**G****G****U**..............................................................GCUAGCAAUAGC-.-.G.A.**G**.**G****A**.**C****A****G**..**U**..**U****A**.**C**.**U**.....GGACGAA-............................................................**C****C****C****U****U****G****G****A****G**A**G**A.**U**.**C****C****A****A****U**CCAUGUAG.......................UUAAUGGA...................................A**A****A****U****G****G**..**A****C****G****C****C**GAA**G****G****C****G**CAA..**G****U****G**..**C**.----..........GUAA..........................................................................................----..**G**.**C****A****U**.G.AAA.**C****G****C**.**U**CA**G****G****C**..............AAAA**G**.**G****A****C****A****G****G****G****G**AGAAAAG | |
|  |  | NZ\_AABO02000001.1/34418-34221  | AUGAA...**G****G**.**U**.**A**.**G**.**C****U****G****G****A****G**A**A**.-**U****G****G****G****G**AA.........UUGA.........................................-**C****C****C****C****A**..**A****A**.**C****C**GAC**G****A****G****G**UA...A..--................----........................--.AAU**C****U****U****U**CA**G****G****U**..................................................................GCGAUGGC-.-.G.A.**G**.**G****A**.**C****U****G**..**U**..**U****A**.**C**.**U**.....GGACGAA-............................................................**C****C****C****U****U****G****G****A****G**A**G**A.**U**.**C****C****A****A****U**GUG............................CAAUGCACA..................................A**A****A****U****G****G**..**A****C****G****C****C**GAA**G****G****C****G**CAA..**A****A****G**..**A**.**G****C****G****G**UG........UUUU......................................................................................UUUA**C****U****G****C**..**U**.**U****U****U**.C.AAA.**C****G****C**.**U**CA**G****G****C**..............AAAA**G**.**G****A****C****A****G****G****G****G**CAAAAGA | |
|  |  | NC\_003902.1/1299978-1299786  | GGUAA...**C****A**.**C**.**G**.**G**.**U****G****G****G****A****G**A**A**.-**G****C****G****G****C**...........ACU-.........................................-**G****C****C****G****C**..**U****G**.**C****C**GAA**G****G****C****G**CA...-..**A****C**A...............GCCC........................**G****U**.AAU**C****G****C****U**CA**G****G****C**..........................................................................-.C.C.G.**A**.**U****A**.**C****C****A**..**U**..**C****U**.**U**.-.....CAACACAA............................................................-**C****U****C****U****G****G****A****G**A**G**A.**C**.**C****G****G**--...............................UUCAC......................................U--**C****C****G**..**G****C****G****C****C**GAA**G****G****G****G**CAC..**G****G****A**..**A**.**C****G****C****A**GGC.......AGGC..............................................................................CACAGGCCAGGC**C****G****C****G**..**U**.**U****C****U**.U.AAA.**C****U****C**.**U**CA**G****G****C**..............AAAA**G**.**G****A****C****A****G****A****G****G**GGCGCGA | |
|  |  | NC\_007086.1/3745218-3745410  | GGUAA...**C****A**.**C**.**G**.**G**.**U****G****G****G****A****G**A**A**.-**G****C****G****G****C**...........ACU-.........................................-**G****C****C****G****C**..**U****G**.**C****C**GAA**G****G****C****G**CA...-..**A****C**A...............GCCC........................**G****U**.AAU**C****G****C****U**CA**G****G****C**..........................................................................-.C.C.G.**A**.**U****A**.**C****C****A**..**U**..**C****U**.**U**.-.....CAACACAA............................................................-**C****U****C****U****G****G****A****G**A**G**A.**C**.**C****G****G**--...............................UUCAC......................................U--**C****C****G**..**G****C****G****C****C**GAA**G****G****G****G**CAC..**G****G****A**..**A**.**C****G****C****A**GGC.......AGGC..............................................................................CACAGGCCAGGC**C****G****C****G**..**U**.**U****C****U**.U.AAA.**C****U****C**.**U**CA**G****G****C**..............AAAA**G**.**G****A****C****A****G****A****G****G**GGCGCGA | |
|  |  | NZ\_AAAI03000005.1/395625-395413  | CCGCG...**U****G**.**U**.**A**.**U**.**C****G****G****G****A****G**A**G**.U**C****U****G****U****G**C..........CAGCCGCCCGGCCG...............................A**C****A****C****G****G**..**C****G**.**C****C**GAA**G****G****A****G**CA...A..**C****C**G...............CCCC........................**G****G**.AAA**C****U****C****U**CA**G****G****C**..........................................................................A.A.A.A.**G**.**G****A**.**C****C****G**..**G**..**U****A**.**C**.**C**CG...CCAUUGAA............................................................-**C****U****C****U****G****A****A****G**A**A**C.**C**.**C****C****G****G****C**UUG............................CACGUC.....................................A**G****C****C****G****G**CG**G****U****A****C****C**GAA**G****G****A****G**CAA..**G****C****G**..**G**.**C****A****C****G**CG........UAUU.....................................................................................GUUCG**U****G****U****G**..**C**.**C****G****C**.G.AAU.**C****U****C**.**U**CA**G****G****U**..............-CAA**G**.**G****A****C****A****G****A****G****G**GGGCGUC | |
|  |  | NZ\_AAEF02000068.1/19841-20029  | UUCCC...**C****C**.**C**.**G**.**G**.**C****G****G****G****A****G**A**G**.C**C****C****C****A****C**CGG........CCCGCC.......................................G**G****U****G****G****G**..**C****A**.**C****C**GAA**G****G****A****G**CA...A..**C****C**A...............CCCC........................**G****G**.AAC**C****U****C****U**CA**G****G****U**..........................................................................C.C.A.C.**G**.**U****A**.**C****C****G**..**C**..**C****A**.**C**.**G**G....GCAGGCCG............................................................-**C****U****C****U****G****G****A****A**A**G**C.**A**.**G****G****A****C****C**...............................CCAC.......................................-**G****G****U****C****C**..**U****C****A****C****C**GAC**G****G****G****G**AAA..**G****C****C**..**U**.**C****G****C**-..........UGC-..........................................................................................-**G****C****G**..**G**.**G****G****U**.G.AAG.**C****U****C**.**U**CA**G****G****U**..............CCCG**C**.**G****A****C****A****G****A****G****G**GGGUCCA | |
|  |  | NZ\_AAED02000005.1/441705-441894  | UGCCC...**G****U**.**C**.**C**.**G**.**U****G****G****G****A****G**A**G**.A**G****C****A****G****C**...........CCG-.........................................-**G****C****U****G****C**..**C****G**.**C****C**GAA**G****G****G****G**AA...A..**U****C**................GCCC........................**G****A**.AAU**C****U****C****U**CA**G****G****C**..........................................................................A.A.A.A.**G**.**A****A**.**C****C****G**..**C**..**G****A**.**A**.**U**GG...GUUAGACA............................................................-**C****U****C****U****G****G****A****A**A**G**U.**C**.**G****G****G****G****C**...............................UUUU.......................................-**G****C****C****C****C**..**G****C****G****C****C**GAA**G****G****U****G**UAA..**G****C****G**..**U**.**G****G****C****C**G.........ACAG.....................................................................................GGUUC**C****G****G****C**..**G**.**C****G****C**.G.AGU.**C****U****C**.**U**CA**G****G****C**..............UUCA-.**G****A****C****A****G****A****G****G**GGCACGG | |
|  |  | NC\_006834.1/3817062-3817254  | GGCAA...**C****A**.**C**.**G**.**G**.**U****G****G****G****A****G**A**A**.-**G****C****G****G****C**...........AUU-.........................................-**G****C****C****G****C**..**U****G**.**C****C**GAA**G****G****C****G**CA...-..**A****C**A...............GCCC........................**G****U**.AAU**C****G****C****U**CA**G****G****C**..........................................................................-.C.C.G.**A**.**U****A**.**C****C****A**..**U**..**C****C**.**G**.-.....CAGGACAA............................................................-**C****U****C****U****G****G****A****G**A**G**A.**C**.**C****G****G****C**-...............................CGAU.......................................--**G****C****C****G**..**G****C****G****C****C**GAA**G****G****G****G**CAC..**G****A****A**..**A**.**C****G****C****A**GGCGGGCC..ACGC...................................................................................GCCUUGA**C****G****C****G**..**U**.**U****U****U**.U.AAA.**C****U****C**.**U**CA**G****G****C**..............AAAA**G**.**G****A****C****A****G****A****G****G**GGCGCGA | |
|  |  | NC\_006513.1/3631246-3631038  | GCCCG...**U****C**.**G**.**C**.**G**.**C****G****G****G****A****G**A**G**.C**A****C****U****C****U**C..........CCCG.........................................-**A****G****A****G****U**..**C****G**.**C****C**GAA**G****G****C****G**UA...A..**C****C**C...............CCCA........................**G****G**.AAU**C****G****C****U**CA**G****G****C**..........................................................................A.A.A.A.**G**.**G****A**.**C****C****G**..**C**..-**C**.**G**.**A**CA...GAACACAA............................................................-**U****U****C****U****G****G****A****G**A**G**C.**G**.**A****U****G****C****G**GGCCCGGGAAACGUC................GAAGCGUUCCCGACCG...........................G**C****G****C****A****U**..**C****C****A****C****C**GAA**G****G****G****G**CAC..**C****C****G**..**G**.**C**---..........AUCG..........................................................................................---**G**..**C**.**C****G****G**.-.AAU.**C****U****C**.**U**CA**G****G****U**.............ACAAG**G**.**G****A****C****A****G****A****U****G**GGGCGAC | |
|  |  | NC\_002944.2/1693363-1693585  | CGCAU...**U****C**.**G**.**A**.**G**.**C****G****G****G****A****G**A**G**.U**U****C****C****G****U**G..........ACGGCCAGU....................................C**A****C****G****G****A**..**C****G**.**C****C**GAA**G****G****A****G**CA...AC.**A****C**C...............UCUC.......................C**G****U**CAAC**C****U****C****U**CA**G****G****C**..........................................................................A.C.C.C.**G**.**G****A**.**C****C****G**..**C**..**G****C**.**G**.**A**A....ACAUGAUG............................................................**C****C****U****C****U****G****G****A****A**A**G**C.**G**.**G****U****G****C****C**GGCC...........................CUCGGGC....................................C**C****G****C****A****C**..**C****C****G****C****C**GAU**G****G****G****G**AAA..**G****G****C**..**G**.**G****C****C****G**CGA.......CAGC........................................................................................AU**C****G****G****C**..**C**.**G****C****C**.G.AAU.**C****U****C**.**U**CA**G****G****C**.GCCCGGCGAACGGGUGA**A**.**G****A****C****A****G****A****G****G**AAGAGGA | |
|  |  | NC\_006138.1/1448610-1448793  | AUGAA...**C****A**.**U**.**A**.**G**.**C****A****G****G****A****G**A**G**.A**U****U****U****U****U**CC.........CAUGGCAGUACGGAUGG............................G**G****A****A****G****G**..**C****A**.**C****C**GAA**G****A****A****G**UA...A..--................----........................--.-AU**C****U****U****U**CA**G****G****U**..........................................................................C.A.A.A.**G**.**G****A**.**C****U****G**..**C**..**U****A**.**U**.**G**.....GGACGAG-............................................................**C****C****U****C****U****G****G****A****G**A**G**U.**C**.**U****C****A**--...............................-UUA.......................................---**U****G****G**..**G****C****A****C****C**GAA**G****G****A****G**CAA..**G****C****C**..**A**.**G****C****G**-..........UGUA..........................................................................................--**G****C**..**U**.**G****G****U**.G.AAC.**C****U****C**.**U**CA**G****G****U**.............ACAAG**G**.**G****A****C****A****G****A****G****U**GUAGCAG | |
|  |  | NC\_006361.1/2650089-2650305  | UCGCG...**C****C**.**G**.**U**.**G**.**C****G****G****G****A****G**A**G**.U**C****C****C****G****G**U..........CUCAGCCGAG...................................U**C****C****G****G****G**..**C****G**.**C****C**GAA**G****G****A****G**CA...GC.**A****C**C...............UCCC.......................C**G****U**CAAU**C****U****C****U**CA**G****G****C**..........................................................................A.A.C.C.**G**.**G****A**.**C****C****G**..**U**..**G****C**.**G**.**G**C....UUUCGACG............................................................**C****C****U****C****U****G****G****A****A**A**G**C.**G**.**G****U****G**--...............................GUCCGCGCGCUGACGCGGGU.......................G--**C****G****C**..**C****C****G****C****C**CAC**G****G****G****G**AAA..**G****G****G**..-.**G****G****C****C**GG........CAGC.....................................................................................GCGUC**G****G****C****A**..**U**.**C****C****C**.G.AAU.**C****U****C**.**U**CA**G****G****C**..............AUCA**C**.**G****A****C****A****G****A****G****G**GGGAGGG | |
|  |  | NC\_002679.1/179373-179552  | AAGGC...**U****G**.**G**.**A**.-.**C****G****G****G****A****G**A**G**.A**U****C****G****G****C**...........UACU.........................................-**G****C****C****G****A**..**C****G**.**C****C**GAC**G****G****A****G**CA...A..**C****C**C...............CCCA.......................A**G****G**.AAA**C****U****C****U**CA**G****G****C**..........................................................................A.A.A.A.**G**U**G****A**.**C****C****G**..**C**..**A****C**.**C**.-.....GCCGAAC-............................................................**G****A****U****C****U****G****G****A****G**A**G**A.**G**.**A****C****G****C**-...............................CUCG.......................................A-**G****C****G****U**..**C****C****A****C****C**GAA**G****G****G****G**AAA..**G****C****C**..**G**.**G****C**--..........AGG-..........................................................................................--**C****C**..**C**.**G****G****U**.U.AAG.**C****U****C**.**U**CA**G****G****U**.............AGCCG**A**.**G****A****C****A****G****A****U****U**UGGGGAU | |
|  |  | NC\_002940.2/362943-363144  | AUGAA...**G****G**.**U**.**G**.**G**.**C****U****G****G****A****G**A**A**.-**G****A****G**--...........UUUAG........................................G--**C****U****C**..**U****A**.**C****C**GAC**G****A****G****G**UA...A..--................----........................--.AAU**C****U****U****U**CA**G****G****U**........................................................GCAAUAGUUUAACUAUGU-.-.G.A.**G**.**G****A**.**C****A****G**..**U**..**U****A**.**C**.**U**.....GGACGAA-............................................................**C****C****C****U****U****G****G****A****G**A**G**A.**U**.**C****C****A****C****U**CUGUACUAUAG....................UACAGA.....................................A**A****A****U****G****G**..**A****C****G****C****C**GAA**G****G****C****G**CAA..**A****U****A**..**U**.**A****A****A****G**..........CAA-..........................................................................................**A****U****U****U**..**A**.**U****G****U**.G.AAA.**C****G****C**.**U**CA**G****G****C**..............AAAA**G**.**G****A****C****A****G****G****G****G**AGAAAAG | |
|  |  | NC\_003919.1/1392548-1392356  | CGCAA...**C****A**.**C**.**G**.**G**.**U****G****G****G****A****G**A**A**.-**G****C****G****G****C**...........ACU-.........................................-**G****C****C****G****C**..**U****G**.**C****C**GAA**G****G****C****G**CA...-..**A****C**A...............GCCC........................**G****U**.AAU**C****G****C****U**CA**G****G****C**..........................................................................-.C.C.G.**A**.**U****A**.**C****C****A**..**U**..**C****C**.**G**.-.....CAGUACAA............................................................-**C****U****C****U****G****G****A****G**A**G**A.**C**.**C****G****G****C**-...............................CGAU.......................................--**G****C****C****G**..**G****C****G****C****C**GAA**G****G****G****G**CAC..**G****A****A**..**A**.**C****G****C****A**GGC.......AGGC..............................................................................CACGCGCCAGGC**C****G****C****G**..**U**.**U****U****U**.U.AAA.**C****U****C**.**U**CA**G****G****C**..............AAAA**G**.**G****A****C****A****G****A****G****G**GGCGCGA | |
|  |  | NC\_003062.1/1462264-1462091  | GCAUC...**A****U**.**C**.**G**.**U**.**U****G****G****G****A****G**A**A**.A**C****C****G****C**-...........UUCAU........................................U-**G****C****G****G**..**U****G**.**C****C**GAA**G****G****A****G**CA...A..**C****C**G...............CCCC........................**G****G**.AAA**C****U****C****U**CA**G****G****C**..........................................................................A.A.A.A.**G**.**G****A**.**C****C****A**..**G**..**C****G**.**A**.**U**GA...CGACGGAA............................................................-**C****U****C****U****G****G****A****G**A**G**A.**A**.**G****C****C****A****C**CUUGAC.........................UAAAG......................................G**A****C****G****G****C**..**U****C****G****C****C**GAA**G****G****G****A**UAA..---..-.----..........----..........................................................................................----..-.---.-.-CA.**A****U****C**.**U**CA**G****G****C**.............GACAA**G**.**G****A****C****A****G****A****G****G**GGGCUCU | |
|  |  | NC\_003304.1/1462378-1462205  | GCAUC...**A****U**.**C**.**G**.**U**.**U****G****G****G****A****G**A**A**.A**C****C****G****C**-...........UUCAU........................................U-**G****C****G****G**..**U****G**.**C****C**GAA**G****G****A****G**CA...A..**C****C**G...............CCCC........................**G****G**.AAA**C****U****C****U**CA**G****G****C**..........................................................................A.A.A.A.**G**.**G****A**.**C****C****A**..**G**..**C****G**.**A**.**U**GA...CGACGGAA............................................................-**C****U****C****U****G****G****A****G**A**G**A.**A**.**G****C****C****A****C**CUUGAC.........................UAAAG......................................G**A****C****G****G****C**..**U****C****G****C****C**GAA**G****G****G****A**UAA..---..-.----..........----..........................................................................................----..-.---.-.-CA.**A****U****C**.**U**CA**G****G****C**.............GACAA**G**.**G****A****C****A****G****A****G****G**GGGCUCU | |
|  |  | NC\_006300.1/332021-331815  | AUGAA...**G****G**.**U**.**A**.**G**.**C****U****G****G****A****G**A**G**.U**G****G****G****G****A**...........AUUA.........................................-**A****C****C****C****U**.A**C****G**.**C****C**GAC**G****A****U****G**AA...A..--................----........................--.AAU**C****U****U****U**CA**G****G****C**............................................................GCAAAGUAAUUUGC-.-.A.A.**G**.**G****A**.**C****U****G**..**U**..**U****A**.**C**.**U**.....GGACGAA-............................................................**C****C****C****U****U****G****G****A****G**A**G**A.**U**.**C****C****A****A****U**GUGC...........................UUAGCACA...................................A**A****A****U****G****G**..**A****C****G****C****C**GAA**G****G****C****G**CAA..**A****A****G**..**U**.**G****C****G****G**UC........AAUU...................................................................................UUGACCG**A****U****U****U**..**U**.**C****U****U**.G.AAA.**C****G****C**.**U**CA**G****G****C**..............AAAA**G**.**G****A****C****A****G****G****G****G**CAAGAGA | |
|  |  | NC\_003047.1/1674991-1674829  | UCGAC...**C****U**.**C**.**G**.**U**.**U****G****G****G****A****G**A**A**.A**C****C****G****G**-...........UUCGA........................................U-**C****C****G****G**..**U****G**.**C****C**GAA**G****G****A****G**CA...A..**C****C**G...............CCCC........................**G****G**.AAA**C****U****C****U**CA**G****G****C**..........................................................................C.A.A.A.**G**.**G****A**.**C****C****A**..**G**..**C****A**.**A**.**G**G....UGCCGGUAGGA.........................................................-**C****U****C****U****G****G****A****G**A**G**A.**A**.**G****C****G**--...............................UUCG.......................................---**C****G****C**..**U****C****G****C****C**GAA**G****G****G****A**UAA..---..-.----..........----..........................................................................................----..-.---.-.-CA.**A****U****C**.**U**CA**G****G****C**..............AAAG**G**.**G****A****C****A****G****A****G****G**GGGCUCG | |
|  |  | NZ\_AAIN01000002.1/336503-336264  | AUGAA...**G****G**.**U**.**A**.**G**.**C****A****G****G****A****G**A**G**.U**U****G****G****G****G**...........CGUG.........................................-**C****C****C****C****A**..**C****A**.**C****C**GAC**G****A****G****G**CC...A..**A****C**U...............UCGA.............UGCAAGUCGAA**G****G**.ACU**C****U****U****U**CA**G****G****U**........................................................GCCAGGAAUUGACCUGGU-.-.G.U.**G**.**G****A**.**C****U****G**..**C**..**U****A**.**C**.**U**.....GGACGAG-............................................................**C****C****U****C****U****G****G****A****G**A**G**A.**U**.**C****C****G****G****U**CAGUUACGUUUGGCAUCACAGAUGCCUGCG.GUAAUCGG...................................C**A****C****C****G****G**..**G****C****G****C****C**GAA**G****G****C****G**AAA..**G****C****C**..**C**.**C**---..........UUU-..........................................................................................---**G**..**G**.**G****G****U**.G.AAA.**C****G****C**.**U**CA**G****G****C**..............AAAA**G**.**G****A****C****A****G****A****G****G**AGAGGAU | |
|  |  | NZ\_AADF01000007.1/209500-209700  | AAAAC...**A****U**.**C**.**U**.**G**.**C****A****G****G****A****G**A**G**.A**G****A****G****U****C**GUGGA......UUUAC........................................A**C****A****C****U****C**..**C****G**.**C****C**GAA**G****G****C****G**CA...A..**A****C**................UCCC........................**A****U**.AAU**C****G****C****U**CA**G****G****C**..........................................................................A.U.A.C.**C**.**G****A**A**C****U****G**..**C**..**A****G**.**A**.**A**A....UGAUUCGC............................................................**C****G****G****C****U****G****G****A****G**A**G**A.**A**.**G****U****U****G****A**GAGCGUCUUGGCG..................CUCG.......................................-**G****C****A****A****C**..**U****C****A****C****C**GAA**G****G****G****G**CA-..**G****G****C**..**U**.**C**---U.........UGUU..........................................................................................---**G**..**A**.**G****C****C**.G.AAA.**C****U****C**.**U**CA**G****G****U**..............AAAA**G**.**G****A****C****A****G****G****G****G**GAGAGGC | |
|  |  | NC\_003997.3/2221716-2221496  | AUGAA...**C****C**.**A**.**U**.**U**.**C****A****G****G****A****G**A**A**.-**G****G****U****C**-...........UAUU.........................................--**G****A****U****C**..**U****A**.**C****C**GAC**G****G****G****G**CA...A..**A****A**AGUUGUUAA.......CCGA......................CU**U****U**GAAA**C****U****C****U**CA**G****G****U**................................................................CUUGUUUACAA.G.U.A.**G**.**A****A**.**C****U****G**..**C**..**A****U**.**G**.**G**.....GGACGAA-............................................................**U****C****U****C****U****G****G****A****G**A**G**A.**C**.**U****C****C****C****U**CUCGCU.........................UUAAAUAGCGUAGAGGA..........................A**A****A****C****G****A**..**G****C****A****C****C**GAA**G****G****A****G**CAA..**A****U****C**..**C**.**G****C****U****A**..........CUA-..........................................................................................**U****A****G****C**..**G**.**G****A****U**.-.AAU.**C****U****C**.**U**CA**G****G****U**..............AAAA**G**.**G****A****C****A****G****A****G****A**CAAGCGA | |
|  |  | NC\_005945.1/2221822-2221602  | AUGAA...**C****C**.**A**.**U**.**U**.**C****A****G****G****A****G**A**A**.-**G****G****U****C**-...........UAUU.........................................--**G****A****U****C**..**U****A**.**C****C**GAC**G****G****G****G**CA...A..**A****A**AGUUGUUAA.......CCGA......................CU**U****U**GAAA**C****U****C****U**CA**G****G****U**................................................................CUUGUUUACAA.G.U.A.**G**.**A****A**.**C****U****G**..**C**..**A****U**.**G**.**G**.....GGACGAA-............................................................**U****C****U****C****U****G****G****A****G**A**G**A.**C**.**U****C****C****C****U**CUCGCU.........................UUAAAUAGCGUAGAGGA..........................A**A****A****C****G****A**..**G****C****A****C****C**GAA**G****G****A****G**CAA..**A****U****C**..**C**.**G****C****U****A**..........CUA-..........................................................................................**U****A****G****C**..**G**.**G****A****U**.-.AAU.**C****U****C**.**U**CA**G****G****U**..............AAAA**G**.**G****A****C****A****G****A****G****A**CAAGCGA | |
|  |  | NC\_005957.1/2242962-2242742  | AUGAA...**C****C**.**A**.**U**.**U**.**C****A****G****G****A****G**A**A**.-**G****G****U****C**-...........UAUU.........................................--**G****A****U****C**..**U****A**.**C****C**GAC**G****G****G****G**CA...A..**A****A**AGUUGUUAA.......CCGA......................CU**U****U**GAAA**C****U****C****U**CA**G****G****U**................................................................CUUGUUUACAA.G.U.A.**G**.**A****A**.**C****U****G**..**C**..**A****U**.**G**.**G**.....GGACGAA-............................................................**U****C****U****C****U****G****G****A****G**A**G**A.**C**.**U****C****C****C****U**CUCGCU.........................UUAAAUAGCGUAGAGGA..........................A**A****A****C****G****A**..**G****C****A****C****C**GAA**G****G****A****G**CAA..**A****U****C**..**C**.**G****C****U****A**..........CUA-..........................................................................................**U****A****G****C**..**G**.**G****A****U**.-.AAU.**C****U****C**.**U**CA**G****G****U**..............AAAA**G**.**G****A****C****A****G****A****G****A**CAAGCGA | |
|  |  | NC\_007530.2/2221840-2221620  | AUGAA...**C****C**.**A**.**U**.**U**.**C****A****G****G****A****G**A**A**.-**G****G****U****C**-...........UAUU.........................................--**G****A****U****C**..**U****A**.**C****C**GAC**G****G****G****G**CA...A..**A****A**AGUUGUUAA.......CCGA......................CU**U****U**GAAA**C****U****C****U**CA**G****G****U**................................................................CUUGUUUACAA.G.U.A.**G**.**A****A**.**C****U****G**..**C**..**A****U**.**G**.**G**.....GGACGAA-............................................................**U****C****U****C****U****G****G****A****G**A**G**A.**C**.**U****C****C****C****U**CUCGCU.........................UUAAAUAGCGUAGAGGA..........................A**A****A****C****G****A**..**G****C****A****C****C**GAA**G****G****A****G**CAA..**A****U****C**..**C**.**G****C****U****A**..........CUA-..........................................................................................**U****A****G****C**..**G**.**G****A****U**.-.AAU.**C****U****C**.**U**CA**G****G****U**..............AAAA**G**.**G****A****C****A****G****A****G****A**CAAGCGA | |
|  |  | NZ\_AAAC02000001.1/2701219-2700999  | AUGAA...**C****C**.**A**.**U**.**U**.**C****A****G****G****A****G**A**A**.-**G****G****U****C**-...........UAUU.........................................--**G****A****U****C**..**U****A**.**C****C**GAC**G****G****G****G**CA...A..**A****A**AGUUGUUAA.......CCGA......................CU**U****U**GAAA**C****U****C****U**CA**G****G****U**................................................................CUUGUUUACAA.G.U.A.**G**.**A****A**.**C****U****G**..**C**..**A****U**.**G**.**G**.....GGACGAA-............................................................**U****C****U****C****U****G****G****A****G**A**G**A.**C**.**U****C****C****C****U**CUCGCU.........................UUAAAUAGCGUAGAGGA..........................A**A****A****C****G****A**..**G****C****A****C****C**GAA**G****G****A****G**CAA..**A****U****C**..**C**.**G****C****U****A**..........CUA-..........................................................................................**U****A****G****C**..**G**.**G****A****U**.-.AAU.**C****U****C**.**U**CA**G****G****U**..............AAAA**G**.**G****A****C****A****G****A****G****A**CAAGCGA | |
|  |  | NZ\_AAEN01000016.1/520616-520396  | AUGAA...**C****C**.**A**.**U**.**U**.**C****A****G****G****A****G**A**A**.-**G****G****U****C**-...........UAUU.........................................--**G****A****U****C**..**U****A**.**C****C**GAC**G****G****G****G**CA...A..**A****A**AGUUGUUAA.......CCGA......................CU**U****U**GAAA**C****U****C****U**CA**G****G****U**................................................................CUUGUUUACAA.G.U.A.**G**.**A****A**.**C****U****G**..**C**..**A****U**.**G**.**G**.....GGACGAA-............................................................**U****C****U****C****U****G****G****A****G**A**G**A.**C**.**U****C****C****C****U**CUCGCU.........................UUAAAUAGCGUAGAGGA..........................A**A****A****C****G****A**..**G****C****A****C****C**GAA**G****G****A****G**CAA..**A****U****C**..**C**.**G****C****U****A**..........CUA-..........................................................................................**U****A****G****C**..**G**.**G****A****U**.-.AAU.**C****U****C**.**U**CA**G****G****U**..............AAAA**G**.**G****A****C****A****G****A****G****A**CAAGCGA | |
|  |  | NZ\_AAEO01000022.1/179736-179516  | AUGAA...**C****C**.**A**.**U**.**U**.**C****A****G****G****A****G**A**A**.-**G****G****U****C**-...........UAUU.........................................--**G****A****U****C**..**U****A**.**C****C**GAC**G****G****G****G**CA...A..**A****A**AGUUGUUAA.......CCGA......................CU**U****U**GAAA**C****U****C****U**CA**G****G****U**................................................................CUUGUUUACAA.G.U.A.**G**.**A****A**.**C****U****G**..**C**..**A****U**.**G**.**G**.....GGACGAA-............................................................**U****C****U****C****U****G****G****A****G**A**G**A.**C**.**U****C****C****C****U**CUCGCU.........................UUAAAUAGCGUAGAGGA..........................A**A****A****C****G****A**..**G****C****A****C****C**GAA**G****G****A****G**CAA..**A****U****C**..**C**.**G****C****U****A**..........CUA-..........................................................................................**U****A****G****C**..**G**.**G****A****U**.-.AAU.**C****U****C**.**U**CA**G****G****U**..............AAAA**G**.**G****A****C****A****G****A****G****A**CAAGCGA | |
|  |  | NZ\_AAEP01000025.1/319762-319542  | AUGAA...**C****C**.**A**.**U**.**U**.**C****A****G****G****A****G**A**A**.-**G****G****U****C**-...........UAUU.........................................--**G****A****U****C**..**U****A**.**C****C**GAC**G****G****G****G**CA...A..**A****A**AGUUGUUAA.......CCGA......................CU**U****U**GAAA**C****U****C****U**CA**G****G****U**................................................................CUUGUUUACAA.G.U.A.**G**.**A****A**.**C****U****G**..**C**..**A****U**.**G**.**G**.....GGACGAA-............................................................**U****C****U****C****U****G****G****A****G**A**G**A.**C**.**U****C****C****C****U**CUCGCU.........................UUAAAUAGCGUAGAGGA..........................A**A****A****C****G****A**..**G****C****A****C****C**GAA**G****G****A****G**CAA..**A****U****C**..**C**.**G****C****U****A**..........CUA-..........................................................................................**U****A****G****C**..**G**.**G****A****U**.-.AAU.**C****U****C**.**U**CA**G****G****U**..............AAAA**G**.**G****A****C****A****G****A****G****A**CAAGCGA | |
|  |  | NZ\_AAEQ01000035.1/1010669-1010889  | AUGAA...**C****C**.**A**.**U**.**U**.**C****A****G****G****A****G**A**A**.-**G****G****U****C**-...........UAUU.........................................--**G****A****U****C**..**U****A**.**C****C**GAC**G****G****G****G**CA...A..**A****A**AGUUGUUAA.......CCGA......................CU**U****U**GAAA**C****U****C****U**CA**G****G****U**................................................................CUUGUUUACAA.G.U.A.**G**.**A****A**.**C****U****G**..**C**..**A****U**.**G**.**G**.....GGACGAA-............................................................**U****C****U****C****U****G****G****A****G**A**G**A.**C**.**U****C****C****C****U**CUCGCU.........................UUAAAUAGCGUAGAGGA..........................A**A****A****C****G****A**..**G****C****A****C****C**GAA**G****G****A****G**CAA..**A****U****C**..**C**.**G****C****U****A**..........CUA-..........................................................................................**U****A****G****C**..**G**.**G****A****U**.-.AAU.**C****U****C**.**U**CA**G****G****U**..............AAAA**G**.**G****A****C****A****G****A****G****A**CAAGCGA | |
|  |  | NZ\_AAER01000039.1/319460-319240  | AUGAA...**C****C**.**A**.**U**.**U**.**C****A****G****G****A****G**A**A**.-**G****G****U****C**-...........UAUU.........................................--**G****A****U****C**..**U****A**.**C****C**GAC**G****G****G****G**CA...A..**A****A**AGUUGUUAA.......CCGA......................CU**U****U**GAAA**C****U****C****U**CA**G****G****U**................................................................CUUGUUUACAA.G.U.A.**G**.**A****A**.**C****U****G**..**C**..**A****U**.**G**.**G**.....GGACGAA-............................................................**U****C****U****C****U****G****G****A****G**A**G**A.**C**.**U****C****C****C****U**CUCGCU.........................UUAAAUAGCGUAGAGGA..........................A**A****A****C****G****A**..**G****C****A****C****C**GAA**G****G****A****G**CAA..**A****U****C**..**C**.**G****C****U****A**..........CUA-..........................................................................................**U****A****G****C**..**G**.**G****A****U**.-.AAU.**C****U****C**.**U**CA**G****G****U**..............AAAA**G**.**G****A****C****A****G****A****G****A**CAAGCGA | |
|  |  | NZ\_AAES01000035.1/122207-121987  | AUGAA...**C****C**.**A**.**U**.**U**.**C****A****G****G****A****G**A**A**.-**G****G****U****C**-...........UAUU.........................................--**G****A****U****C**..**U****A**.**C****C**GAC**G****G****G****G**CA...A..**A****A**AGUUGUUAA.......CCGA......................CU**U****U**GAAA**C****U****C****U**CA**G****G****U**................................................................CUUGUUUACAA.G.U.A.**G**.**A****A**.**C****U****G**..**C**..**A****U**.**G**.**G**.....GGACGAA-............................................................**U****C****U****C****U****G****G****A****G**A**G**A.**C**.**U****C****C****C****U**CUCGCU.........................UUAAAUAGCGUAGAGGA..........................A**A****A****C****G****A**..**G****C****A****C****C**GAA**G****G****A****G**CAA..**A****U****C**..**C**.**G****C****U****A**..........CUA-..........................................................................................**U****A****G****C**..**G**.**G****A****U**.-.AAU.**C****U****C**.**U**CA**G****G****U**..............AAAA**G**.**G****A****C****A****G****A****G****A**CAAGCGA | |
|  |  | NC\_006677.1/1200182-1200348  | CUGAC...**C****G**.**A**.**C**.**A**.**C****G****G****G****A****G**A**G**.A**C****C****A****G****C**CG.........UCAG.........................................-**G****C****U****G****G**..**C****G**.**C****C**GAA**G****G****A****G**CA...A..**C****C**G...............CCCC........................**G****G**.AAA**C****U****C****U**CA**G****G****C**..........................................................................C.A.A.A.**G**.**G****A**.**C****C****G**..**A**..**G****G**.**C**.**G**G....UCAGGACA............................................................-**U****U****C****U****G****G****A****G**A**G**A.**G**.**G****U****G****C****G**...............................CUGG.......................................A**C****G****C****A****U**..**C****C****G****C****C**GAC**G****G****G****A**UAG..---..-.----..........----..........................................................................................----..-.---.-.-CG.**A****U****C**.**U**CA**G****G****C**..............GAAA**G**.**G****A****C****A****G****A****A****G**GGGCGAC | |
|  |  | NC\_003869.1/313165-312961  | AUGAA...**G****A**.**U**.**A**.**G**.**C****G****A****G****A****G**A**U**.U**A****U****C****U****U**CCA........UAAAUG.......................................G**A****A****G****A****U**..**A****G**.**C****C**GAA**G****G****G****G**AA...A..**U****A**CAAAG...........GCCC............GCCAAGCCUUUG**U****A**GAAG**C****U****C****U**CA**G****G****C**..........................................................................G.G.C.A.**G**.**G****A**.**U****C****G**..**C**..**U****A**.**U**.**C**.....GGAUAGG-............................................................**C****C****U****C****U****G****G****A****A**A**G**U.**C**.**U****C**---...............................GUAA.......................................A---**G****A**..**G****C****A****C****C**GAA**G****G****A****G**CAA..**U****A****C**..**A**.**U****A****U****G**G.........AAGG.........................................................................................C**C****A****U****A**..**U**.**G****U****A**.G.AAG.**C****U****C**.**U**CA**G****G****U**..............AGAA**A**.**A****A****C****A****G****A****G****G**AGUUGUG | |
|  |  | NC\_000907.1/936415-936225  | AUGAA...**G****G**.**U**.**A**.**G**.**C****U****G****G****A****G**A**G**.-**C****G****G****G****A**AA.........UUGA.........................................-**C****C****C****C****A**..**C****A**.**C****C**GAC**G****A****U****G**UA...A..--................----........................--.AAU**C****U****U****U**CA**G****G****U**..................................................................GCGAUGGC-.-.A.G.**G**.**G****A**.**C****U****G**..**U**..**U****A**.**C**.**U**.....GGACGAA-............................................................**C****C****C****U****U****G****G****A****G**A**G**A.**U**.**C****C****A****U****U**...............................UUAG.......................................A**A****A****U****G****G**..**A****C****G****C****C**GAA-**G****C****G**CAA..**A****A****G**..**A**.**G****C****G****G**UU........AAUU....................................................................................UUUCAA**U****C****G****U**..**U**.**U****U****U**.C.AAA.**C****G****C**.**U**CA**G****G****C**..............AAAA**G**.**G****A****C****A****G****G****G****G**CAAAAGA | |
|  |  | NC\_004722.1/2266553-2266331  | AUGAA...**C****C**.**A**.**U**.**U**.**C****A****G****G****A****G**A**A**.-**G****G****U****C**-...........UAUU.........................................--**G****A****U****C**..**U****A**.**C****C**GAC**G****G****G****G**CA...A..**A****A**AGUUGUUA........UACC....................AGCU**U****U**GAAA**C****U****C****U**CA**G****G****U**................................................................CUUGGUUACAA.G.U.A.**G**.**A****A**.**C****U****G**..**C**..**A****U**.**G**.**G**.....GGACGAA-............................................................**U****C****U****C****U****G****G****A****G**A**G**A.**C**.**U****C****C****C****U**...............................CUCGCUUUCAUAGAGCGCGGAGGA...................A**A****A****C****G****A**..**G****C****A****C****C**GAA**G****G****A****G**CAA..**A****U****C**..**C**.**G****C****U****A**..........CUU-..........................................................................................**U****A****G****C**..**G**.**G****A****U**.-.AAU.**C****U****C**.**U**CA**G****G****U**..............AAAA**G**.**G****A****C****A****G****A****G****A**CAAGCGA | |
|  |  | NC\_003909.8/2304914-2304694  | AUGAA...**C****C**.**A**.**U**.**U**.**C****A****G****G****A****G**A**A**.-**G****G****U****C**-...........UAUU.........................................--**G****A****U****C**..**U****A**.**C****C**GAC**G****G****G****G**CA...A..**A****A**AGUUGUUAA.......CCGA......................CU**U****U**GAAA**C****U****C****U**CA**G****G****U**................................................................CUUGUUUACAA.G.U.A.**G**.**A****A**.**C****U****G**..**C**..**A****U**.**G**.**G**.....GGACGAA-............................................................**U****C****U****C****U****G****G****A****G**A**G**A.**C**.**U****C****C****C****U**...............................CUCGCUUUAUAUAGCGUAGAGGA....................A**A****A****C****G****A**..**G****C****A****C****C**GAA**G****G****A****G**CAA..**A****U****C**..**C**.**G****C****U****A**..........CUA-..........................................................................................**U****A****G****C**..**G**.**G****A****U**.-.AAU.**C****U****C**.**U**CA**G****G****U**..............AAAA**G**.**G****A****C****A****G****A****G****A**CAAGCGA | |
|  |  | NC\_006274.1/2270010-2269790  | AUGAA...**C****C**.**A**.**U**.**U**.**C****A****G****G****A****G**A**A**.-**G****G****U****C**-...........UAUU.........................................--**G****A****U****C**..**U****A**.**C****C**GAC**G****G****G****G**CA...A..**A****A**AGUUUUUAA.......CCGA......................CU**U****U**GAAA**C****U****C****U**CA**G****G****U**................................................................CUUGUUUACAA.G.U.A.**G**.**A****A**.**C****U****G**..**C**..**A****U**.**G**.**G**.....GGACGAA-............................................................**U****C****U****C****U****G****G****A****G**A**G**A.**C**.**U****C****C****C****U**...............................CUCGCUUUAUAUAGCGCAGAGGA....................A**A****A****C****G****A**..**G****C****A****C****C**GAA**G****G****A****G**CAA..**A****U****C**..**C**.**G****C****U****A**..........CUA-..........................................................................................**U****A****G****C**..**G**.**G****A****U**.-.AAU.**C****U****C**.**U**CA**G****G****U**..............AAAA**G**.**G****A****C****A****G****A****G****A**CAAGCGA | |
|  |  | NZ\_AAED02000016.1/54004-53806  | UCAAC...**U****G**G**U**.**U**.**G**.**C****G****G****G****A****G**A**G**.A**G****C****A****G****C**G..........CACG.........................................-**G****C****U****G****C**..**C****G**.**C****C**GAA**G****G****G****G**-A...A..**U****A**C...............GCC-........................**U****A**AAAU**C****U****C****U**CA**G****G****C**..........................................................................A.A.A.A.**G**.**A****A**.**C****C****G**..**U**..**A****A**G**C**.**A**.....GGGCGACA............................................................-**C****U****C****U****G****G****A****A**A**G**A.**C**.**G****G****G****G****C**CUGU...........................CAAA.......................................G**A****U****C****C****C**..**G****C****G****C****C**GAA**G****G****U****G**UAA..**G****U****G**..**C**.**U****A****U****C**GGAGCCC...UAUC.........................................................................................C**G****G****C****A**..**G**.**C****G****C**.G.AGU.**C****U****C**.**U**CA**G****G****C**..............UUCA-.**G****A****C****A****G****A****G****G**GGCACGG | |
|  |  | NZ\_AAEK01000006.1/35074-35294  | AUGAA...**C****C**.**A**.**U**.**U**.**C****A****G****G****A****G**A**A**.-**G****G****U****C**-...........UAUU.........................................--**G****A****U****C**..**U****A**.**C****C**GAC**G****G****G****G**CA...A..**A****A**AGUUGUUA........ACCG.....................GCU**U****U**GAAA**C****U****C****U**CA**G****G****U**................................................................CUUGUUUACAA.G.U.A.**G**.**A****A**.**C****U****G**..**C**..**A****U**.**G**.**G**.....GGACGAA-............................................................**U****C****U****C****U****G****G****A****G**A**G**A.**C**.**U****C****C****C****U**...............................CUCGCUUUAUAUAGCGUAGAGGA....................A**A****A****C****G****A**..**G****C****A****C****C**GAA**G****G****A****G**CAA..**A****U****C**..**C**.**G****C****U****A**..........CUA-..........................................................................................**U****A****G****C**..**G**.**G****A****U**.-.AAU.**C****U****C**.**U**CA**G****G****U**..............AAAA**G**.**G****A****C****A****G****A****G****A**CAAGCGA | |
|  |  | NC\_000962.2/79294-79095  | UGACG...**C****G**.**A**.**U**.**G**.**U****G****G****G****A****G**A**A**.-**C****C****U****C****C**A..........UGU-.........................................-**C****G****A****G****G**..**C****G**.**C****C**GUA**G****G****A****G**CA...A..**U****C**UCC.............UCCC.......................C**G****A**GAAU**C****U****C****U**CA**G****G****C**..........................................................................C.C.A.A.**G**.**C****A**.**C****C****A**..**C**..**A****C**.**C**.**G**C....CGAGGCAA............................................................-**C****U****C****U****G****G****A****G**A**C**A.**G**.**G****G****A****C****G**...............................GUCGCACCGAC................................C**G****U****G****C****C**..**U****G****A****C****C**GAA**G****G****U****G**UAGA.**G****C****G**..**G**.**C****G****C****C**A.........UGAU........................................................................................GC**G****A****C****G**..**C**.**C****G****C**.-.AGA.**C****U****C**.**U**CA**G****G****U**..............UUCA**G**.**G****A****C****A****G****A****G****C**GGGGAGG | |
|  |  | NC\_002755.2/79275-79076  | UGACG...**C****G**.**A**.**U**.**G**.**U****G****G****G****A****G**A**A**.-**C****C****U****C****C**A..........UGU-.........................................-**C****G****A****G****G**..**C****G**.**C****C**GUA**G****G****A****G**CA...A..**U****C**UCC.............UCCC.......................C**G****A**GAAU**C****U****C****U**CA**G****G****C**..........................................................................C.C.A.A.**G**.**C****A**.**C****C****A**..**C**..**A****C**.**C**.**G**C....CGAGGCAA............................................................-**C****U****C****U****G****G****A****G**A**C**A.**G**.**G****G****A****C****G**...............................GUCGCACCGAC................................C**G****U****G****C****C**..**U****G****A****C****C**GAA**G****G****U****G**UAGA.**G****C****G**..**G**.**C****G****C****C**A.........UGAU........................................................................................GC**G****A****C****G**..**C**.**C****G****C**.-.AGA.**C****U****C**.**U**CA**G****G****U**..............UUCA**G**.**G****A****C****A****G****A****G****C**GGGGAGG | |
|  |  | NC\_002945.3/79321-79122  | UGACG...**C****G**.**A**.**U**.**G**.**U****G****G****G****A****G**A**A**.-**C****C****U****C****C**A..........UGU-.........................................-**C****G****A****G****G**..**C****G**.**C****C**GUA**G****G****A****G**CA...A..**U****C**UCC.............UCCC.......................C**G****A**GAAU**C****U****C****U**CA**G****G****C**..........................................................................C.C.A.A.**G**.**C****A**.**C****C****A**..**C**..**A****C**.**C**.**G**C....CGAGGCAA............................................................-**C****U****C****U****G****G****A****G**A**C**A.**G**.**G****G****A****C****G**...............................GUCGCACCGAC................................C**G****U****G****C****C**..**U****G****A****C****C**GAA**G****G****U****G**UAGA.**G****C****G**..**G**.**C****G****C****C**A.........UGAU........................................................................................GC**G****A****C****G**..**C**.**C****G****C**.-.AGA.**C****U****C**.**U**CA**G****G****U**..............UUCA**G**.**G****A****C****A****G****A****G****C**GGGGAGG | |
|  |  | NC\_007164.1/259290-259496  | UUAGC...**U****G**.**U**.**G**.**G**.**U****G****G****G****A****G**A**G**GC**U****C****G****G****C**G..........CUAGCCACG....................................C**G****C****C****G****G**.C**C****G**.**C****C**GAC**G****G****A****G**CA...AUU**C****C**U...............CCCC.......................A**G****G**.ACA**C****U****C****U**CA**G****G****C**.......................................................................CCC-.-.A.A.**G**.**U****A**.**C****C****G**..**U**..**C****A**.**C**.**G**G....UGAGGCAA............................................................-**C****U****C****U****G****G****A****A**A**G**A.**C**.**C****U****G****C****G**...............................CGCAGUCGUGUGC..............................A**C****G****C****G****G**..**G****C****G****C****C**GAA**G****G****A****G**AAAA.**G****C****C**..**G**.**G****G**--..........UUGA..........................................................................................--**C****C**..**G**.**G****G****U**.G.AAG.**C****U****C**.**U**CA**G****G****C**..............ACAA**G**C**G****A****C****A****G****A****G****U**GGGGAGG | |
|  |  | NC\_006510.1/2456344-2456129  | AUGAC...**G****G**.**C**.**A**.**A**.**G****G****G****G****A****G**A**G**.A**C****U****A****C**-GG.........AAU-.........................................--**G****U****A****G**..**C****G**.**C****C**GAA**G****G****A****G**CA...A..**G****C**G...............GAAA.......................C**G****C**GAAU**C****U****C****U**CA**G****G****C**..........................................................................A.A.A.A.**A**.**G****A**.**C****U****C**..**U**..**U****G**.**C**.**U**.....UGACGCA-............................................................**G****C****U****C****U****G****G****A****G**A**G**C.**G**.**C****C****U****G****C**G..............................UUCA.......................................C**G****C****A****G****G**..**C****C****A****C****C**CAA**G****A****G****G**AAA..**C****C****G**AU**C**.**G****G****C****C**CC........AUGG...................................................................ACCGCGAACGGACGCGCCAAACG**G****G****C****C**..**G**G**C****G****G**.G.AAA.**C****U****U**.**U**CA**G****G****U**..............GCCA**G**.**G****A****C****A****G****A****G****C**CCCCUUA | |
|  |  | NZ\_AAIP01000001.1/176731-176523  | ACCGG...**C****C**.**G**.**C**.**G**.**C****G****G****G****A****G**A**G**.C**G****U****G****A****G**GGCAGGGC...AACCGGCG.....................................C**C****U****C****A****C**..**C****A**.**C****C**GAA**G****G****A****G**CA...A..**C****C**G...............CCCC........................**G****G**.AAA**C****U****C****U**CA**G****G****U**.........................................................................CA.A.A.G.**G**.**G****A**.**C****C****G**..**C**..**G****C**.**A**.**G**GCUG.GAACGGAC............................................................**A****C****U****C****U****G****G****A****A**A**G**C.**G**.**U****U****C****C****G**GCGGG..........................UUGU.......................................-**C****G****G****A****A**..**C****C****A****C****C**GAA**G****G****G****G**UAA..**C****U****C**..**C**.**G****U****C**-..........AACC..........................................................................................-**G****A****U**..**G**.**G****A****G**.GAAAA.**C****U****C**.**U**CA**G****G****U**.............UCCCG**U**.**G****A****C****A****G****A****G****G**GGGCAUG | |
|  |  | NZ\_AADY01000006.1/276487-276291  | UGCGA...**C****C**.**U**.**C**.**G**.**C****A****G****G****A****G**A**G**.A**C****C****G****G****A**ACGCCGC....CAGGCGC......................................G**U****C****C****G****G**..**C****G**.**C****C**GAC**G****G****A****G**CA...A..**C****C**G...............CCCC........................**G****G**.AAA**C****U****C****U**CA**G****G****C**..........................................................................ACA.C.A.**G**.**G****A**.**C****U****G**..**U**..**G****G**.-.-.....CGUCGCAAGUACAG......................................................-**A****U****C****U****G****G****A****G**A**G**A.**G**.**G****C****G****C**-...............................-UUU.......................................--**G****C****G****C**..**C****C****A****C****C**GAA**G****G****G****G**AC-..**G****G****C**..**A**.**U****G****G****C**..........CUG-..........................................................................................**G****C****C****A**..**U**.**G****U****C**.C.AAG.**C****U****C**.**U**CA**G****G****U**.............ACCGC**G**.**G****A****C****A****G****A****U****G**GGGCAGG | |
|  |  | NZ\_AAFQ02000005.1/234008-234225  | AGCGU...**G****G**.**U**.**A**.**U**.**C****G****G****G****A****G**A**G**.U**C****U****G****C****C**G..........UCCAGCAUUGCCUGG..............................C**G****G****C****G****G**..**C****G**.**C****C**GAA**G****G****A****G**CA...A..**C****C**G...............CCCC........................**G****G**.AAA**C****U****C****U**CA**G****G****C**..........................................................................A.A.A.U.**G**.**A****A**.**C****C****G**..**G**..**U****A**.**C**.**C**AC...CCAUGCA-............................................................-**C****U****C****U****G****A****A****G**A**G**C.**U**.**G****C****C****G****G**GUUUU..........................UUCGUCA....................................C**C****C****G****G****C**..**G****C****A****C****C**GAA**G****G****A****G**CAA..**G****C****G**..**G**.**A****U****U****C**GCGCG.....UCAC.....................................................................................AGCGC**C****G****G****U**..**U**.**C****G****U**.G.AAU.**C****U****C**.**U**CA**G****G****U**..............CCAG**C**.**A****A****C****A****G****A****G****G**GGGCGUC | |
|  |  | NC\_000964.2/2548838-2548639  | AUGAC...**A****G**.**C**.**A**.**A**.**G****G****G****G****A****G**A**G**.A**C****C****U****G****A**CCG........AAAACCUCGGGA.................................U**A****C****A****G****G**..**C****G**.**C****C**GAA**G****G****A****G**CA...A..**A****C**U...............GCGG.......................A**G****U**GAAU**C****U****C****U**CA**G****G****C**..........................................................................A.A.A.A.**G**.**A****A**.**C****U****C**..**U**..**U****G**.**C**.**U**.....CGACGCAA............................................................-**C****U****C****U****G****G****A****G**A**G**U.**G**.**U****U****U****G****U**GC.............................GGAUG......................................C**G****C****A****A****A**..**C****C****A****C****C**UUU**G****G****G****G**ACG..**U****C****U**..**U**.**U****G****C**-..........GUAU..........................................................................................-**G****C****A**..**A**.**A****G****U**.-.AAA.**C****U****U**.**U**CA**G****G****U**..............GCCA**G**.**G****A****C****A****G****A****G****A**ACCUUCA | |
|  |  | NZ\_AAAW03000073.1/5078-5276  | AUGAU...**U****C**.**C**.**A**.**G**.**U****A****G****G****A****G**A**G**.A**C****U****C****G****A**C..........AUC-.........................................-**C****U****G****A****G**..**C****G**.**C****C**GAA**G****G****A****G**AA...A..**C****U**UCCUGAAUGUC.....GCCU..............GUUCAGAAAG**G****G**.AAA**C****U****C****U**CA**G****G****C**..........................................................................A.A.A.A.**G**.**U****A**.**C****U****G**..**C**..**U****G**.**G**.**A**.....AGACGAAA............................................................-**C****U****C****U****G****G****A****G**A**G**C.**U**.**U****C****U****C**-...............................AGCG.......................................--**G****A****G****A**..**A****C****A****C****C**AAA**G****G****G****G**UA-..**G****C****C**..**C**.**G**---C.........AAGC........................................................................................AA---**A**..**G**.**G****G****U**.A.AAU.**C****U****C**.**U**CA**G****G****U**..............CAAA**G**.**G****A****C****A****G****A****G****G**UAGCGCG | |
|  |  | NZ\_AADF01000003.1/360820-360999  | UCCCG...**U****U**.**C**.**C**.**G**.**U****G****G****G****A****G**A**G**.C**G****C****A****G****A**...........UCCG.........................................-**U****C****U****G****C**..**C****G**.**C****C**GAA**G****G****C****G**CA...A..**U****U**U...............CACC.......................C**G****A**.AAA**C****G****C****U**CA**G****G****C**..........................................................................A.A.A.A.**G**.**G****A**.**C****C****G**..**C**..**G****G**.**A**.**A**.....CCAAAAUGGCUGGCCAUUUUGAAA............................................-**C****U****C****U****G****G****A****G**A**G**C.**G**.**G****C****G**--...............................UUAACA.....................................G--**C****G****C**..**C****C****A****C****C**GAU**G****G****G****G**CAA..---..-.----..........----..........................................................................................----..-.---.-.-AU.**C****U****C**.**U**CA**G****G****U**.............AUCGA**G**.**G****A****C****A****G****A****G****G**GGUGAAA | |
|  |  | NC\_002946.2/1372605-1372352  | GGGAA...**A****U**.**G**.**U**.**G**.**C****A****G****G****A****G**A**G**.U**G****U****U****A****C**ACCCAAC....UACAA........................................U**G****U****A****A****C**..**C****A**.**C****C**GAA**G****G****C****G**CA...G..**A****C**................ACCC........................**U****U**AAAU**C****G****C****U**CA**G****G****U**..........................................................................A.U.C.A.**G**.**G****G**A**C****U****G**..**C**..**A****C**.**A**.**U**.....UGAAACAAACA.........................................................-**A****U****C****U****G****G****A****G**A**G**C.**G**.**G****C****G****U****U**A..............................GAAU.......................................-**A****A****C****G****U**..**C****C****A****C****C**GAA**G****G****G****G**AGAA.**G****G****C**..**C**.**G****U****C****U**G.........AACC.....................................ACCAUUCAGACAACCGCGCAAAGCAGUGAGCAGACUGGUUUGCCAUCAUGCGG**A****U****A****C**..**G**.**G****C****C**.G.AAA.**A****U****C**.**U**CA**G****G****U**..............UCAA**G**.**G****A****C****A****G****A****U****A**GGGUCAU | |
|  |  | NC\_003116.1/748896-749149  | GGGAA...**A****U**.**G**.**U**.**G**.**C****A****G****G****A****G**A**G**.U**G****U****U****A****C**ACCCAAC....UACAA........................................U**G****U****A****A****C**..**C****A**.**C****C**GAA**G****G****C****G**CA...G..**A****C**................ACCC........................**U****U**AAAU**C****G****C****U**CA**G****G****U**..........................................................................A.U.C.A.**G**.**G****G**A**C****U****G**..**C**..**A****C**.**A**.**U**.....UGAAACAAACA.........................................................-**A****U****C****U****G****G****A****G**A**G**C.**G**.**G****C****G****U****U**G..............................GAAU.......................................-**A****A****C****G****U**..**C****C****A****C****C**GAA**G****G****G****G**AGAA.**G****G****C**..**C**.**G****U****C****U**G.........AACC.....................................ACCAUUCAGACAACCGCGCAAAGCAGUGAGCAGACUGGUUUGCCAUCAUGCGG**A****U****A****C**..**G**.**G****C****C**.G.AAA.**A****U****C**.**U**CA**G****G****U**..............UCAA**G**.**G****A****C****A****G****A****U****A**GGGUCAU | |
|  |  | NZ\_AAII01000028.1/17332-17550  | CACAC...**C****C**.**G**.**U**.**G**.**C****G****G****G****A****G**A**G**.C**U****C****C****G****G**GC.........CGUGG........................................C**C****C****G****G****G**.G**C****G**.**C****C**GAA**G****G****A****G**CA...A..**G****A**AC..............UCCC.......................C**G****C**.AAA**C****U****C****U**CA**G****G****C**..........................................................................-.C.C.A.**A**.**G****A**.**C****C****G**..**C**..**C****G**.**G**.**G**.....UCAGGCGA............................................................-**C****U****C****U****G****A****A****A**A**G**C.**G**.**G****A****C****G****C**...............................GCAA.......................................-**G****C****G****U****C**..**C****C****G****C****C**GAC**G****G****U****G**CAA..**G****C****C**..**G**.**G****C****C****G**CCGCG.....UCUC..................................................................UCGGGAGGCUCCGGGUGGCAUGGC**A****G****G****C**..**C**.**G****G****C**.G.AAG.**C****U****C**.**U**CA**G****G****C**..............-CCA**U**.**G****A****C****A****G****A****G****G**GGGAGGC | |
|  |  | NC\_000962.2/2075613-2075844  | CGCUC...**U****A**.**G**.**U**.**G**.**C****G****G****G****A****G**A**G**.U**U****C****U****G****U**GG.........CUGCCAGC.....................................U**A****C****G****G****A**..**C****G**.**C****C**GAA**G****G****A****G**CA...AU.**A****C**C...............UCUC.......................C**G****U**CAAC**C****U****C****U**CA**G****G****C**..........................................................................A.C.C.C.**G**.**G****A**.**C****C****G**..**C**..**G****C**.**G**.**A**GA...CUACGAUG............................................................**C****C****U****C****U****G****G****A****A**A**G**C.**G**.**G****U****G****G****C**...............................GACCCCUGGCGGU..............................C**C****U****C****A****C**..**C****C****G****C****C**GAU**G****G****G****G**AAA..**G****G****C**..**G**.**A****U****U****C**ACC.......UGAC..................................................................................GGUGGACA**G****A****G****U**..**C**.**G****C****C**.G.AAU.**C****U****C**.**U**CA**G****G****C**.GCCUGGCGUGCAGGUGA**A**.**G****A****C****A****G****A****G****G**GAGAGGG | |
|  |  | NC\_002755.2/2072838-2073069  | CGCUC...**U****A**.**G**.**U**.**G**.**C****G****G****G****A****G**A**G**.U**U****C****U****G****U**GG.........CUGCCAGC.....................................U**A****C****G****G****A**..**C****G**.**C****C**GAA**G****G****A****G**CA...AU.**A****C**C...............UCUC.......................C**G****U**CAAC**C****U****C****U**CA**G****G****C**..........................................................................A.C.C.C.**G**.**G****A**.**C****C****G**..**C**..**G****C**.**G**.**A**GA...CUACGAUG............................................................**C****C****U****C****U****G****G****A****A**A**G**C.**G**.**G****U****G****G****C**...............................GACCCCUGGCGGU..............................C**C****U****C****A****C**..**C****C****G****C****C**GAU**G****G****G****G**AAA..**G****G****C**..**G**.**A****U****U****C**ACC.......UGAC..................................................................................GGUGGACA**G****A****G****U**..**C**.**G****C****C**.G.AAU.**C****U****C**.**U**CA**G****G****C**.GCCUGGCGUGCAGGUGA**A**.**G****A****C****A****G****A****G****G**GAGAGGG | |
|  |  | NC\_002945.3/2065965-2066196  | CGCUC...**U****A**.**G**.**U**.**G**.**C****G****G****G****A****G**A**G**.U**U****C****U****G****U**GG.........CUGCCAGC.....................................U**A****C****G****G****A**..**C****G**.**C****C**GAA**G****G****A****G**CA...AU.**A****C**C...............UCUC.......................C**G****U**CAAC**C****U****C****U**CA**G****G****C**..........................................................................A.C.C.C.**G**.**G****A**.**C****C****G**..**C**..**G****C**.**G**.**A**GA...CUACGAUG............................................................**C****C****U****C****U****G****G****A****A**A**G**C.**G**.**G****U****G****G****C**...............................GACCCCUGGCGGU..............................C**C****U****C****A****C**..**C****C****G****C****C**GAU**G****G****G****G**AAA..**G****G****C**..**G**.**A****U****U****C**ACC.......UGAC..................................................................................GGUGGACA**G****A****G****U**..**C**.**G****C****C**.G.AAU.**C****U****C**.**U**CA**G****G****C**.GCCUGGCGUGCAGGUGA**A**.**G****A****C****A****G****A****G****G**GAGAGGG | |
|  |  | NC\_006270.2/2546561-2546349  | AUGAC...**G****G**.**C**.**A**.**A**.**G****G****G****G****A****G**A**G**.A**C****C****G****G****C**AGAGGGAAACCUAAA.........................................-**A****G****C****G****G**..**C****G**.**C****C**GAA**G****G****A****G**CA...A..**G****C**UGGG............GAAA......................CA**G****U**GAAU**C****U****C****U**CA**G****G****C**..........................................................................A.A.A.A.**G**.**A****A**.**C****U****C**..**U**..**U****G**.**C**.**U**.....UGACGCAA............................................................-**C****U****C****U****G****G****A****G**A**G**A.**G**.**U****U****U****G****U**AUCAUA.........................UUAUGUUU...................................U**G****C****A****A****A**..**C****C****A****C****C**AAA**G****G****G****G**ACAU.**C****U****U**..**U**.**G****C****U****U**..........UUG-..........................................................................................**A****U****G****C**..**A**.**A****A****G**.U.AAA.**C****U****U**.**U**CA**G****G****U**..............GCCA**G**.**G****A****C****A****G****A****G****A**GCCUCCA | |
|  |  | NC\_006322.1/2547422-2547210  | AUGAC...**G****G**.**C**.**A**.**A**.**G****G****G****G****A****G**A**G**.A**C****C****G****G****C**AGAGGGAAACCUAAA.........................................-**A****G****C****G****G**..**C****G**.**C****C**GAA**G****G****A****G**CA...A..**G****C**UGGG............GAAA......................CA**G****U**GAAU**C****U****C****U**CA**G****G****C**..........................................................................A.A.A.A.**G**.**A****A**.**C****U****C**..**U**..**U****G**.**C**.**U**.....UGACGCAA............................................................-**C****U****C****U****G****G****A****G**A**G**A.**G**.**U****U****U****G****U**AUCAUA.........................UUAUGUUU...................................U**G****C****A****A****A**..**C****C****A****C****C**AAA**G****G****G****G**ACAU.**C****U****U**..**U**.**G****C****U****U**..........UUG-..........................................................................................**A****U****G****C**..**A**.**A****A****G**.U.AAA.**C****U****U**.**U**CA**G****G****U**..............GCCA**G**.**G****A****C****A****G****A****G****A**GCCUCCA | |
|  |  | NC\_002570.2/2941604-2941411  | AUGAA...**A****G**.**C**.**A**.**A**.**G****G****G****G****A****G**A**G**.A**C****U****G****C****A**...........AGGA.........................................-**U****G****C****A****G**..**C****G**.**C****C**GAA**G****G****A****G**CA...A..**A****C**A...............CAAU..................UAGGGU**G****U**GAAU**C****U****C****U**CA**G****G****C**..........................................................................A.A.A.A.**A**.**G****A**.**C****U****C**..**U**..**U****G**.**C**.**U**.....CGACGCA-............................................................**G****C****U****C****U****G****G****A****G**A**G**C.**G**.**U****C****U****A**-...............................ACAC.......................................--**U****A****G****A**..**C****C****A****C****C**UAC**G****A****A****G**ACA..**U****U****U**..**C**.**C****U****U****U**U.........UACG.......................................................................................AUA**A****G****G****G**..**G**.**A****A****A**.G.AAA.**C****U****U**.**U**CU**G****G****U**..............AACC**G**.**G****A****C****A****G****A****G****C**UUUACAC | |
|  |  | NC\_003112.1/601762-602015  | GGGAA...**A****U**.**G**.**U**.**G**.**C****A****G****G****A****G**A**G**.U**G****U****U****A****C**ACCCAAC....UACAA........................................U**G****U****A****A****C**..**C****A**.**C****C**GAA**G****G****C****G**CA...G..**A****C**................ACCC........................**U****U**AAAU**C****G****C****U**CA**G****G****U**..........................................................................A.U.C.A.**G**.**G****G**A**C****U****G**..**C**..**A****C**.**A**.**U**.....UGAAACAAACA.........................................................-**A****U****C****U****G****G****A****G**A**G**C.**G**.**G****C****G****U****U**G..............................GAAU.......................................-**A****A****C****G****U**..**C****C****A****C****C**GAA**G****G****G****G**AGAA.**G****G****C**..**C**.**G****U****C****U**G.........AACC.....................................ACCAUUCAGACAACCGCGCAAAGCAGUGAGCAGACUGGUUUGCCAUCAUGCGG**A****U****A****C**..**A**.**G****C****C**.G.AAA.**A****U****C**.**U**CA**G****G****U**..............UCAA**G**.**G****A****C****A****G****A****U****A**GGGUCAU | |
|  |  | NZ\_AAAE01000060.1/7723-7559  | AGCCA...**C****A**.**G**.**G**.**A**U**C****G****G****G****A****G**A**A**.U**C****C****G****G****G**GG.........CGAC.........................................-**C****C****C****G****G**..**U****G**.**C****C**GAA**G****G****A****G**CA...A..**C****C**G...............CCCC........................**G****G**UAAA**C****U****C****U**CA**G****G****C**..........................................................................G.C.A.A.**G**.**G****A**.**C****C****G**..**U**..**C****C**.**U**.**G**.....UUUCGAAG............................................................**A****C****U****C****U****G****G****A****G**A**G**A.**G**.**G****C****C**--...............................GGAC.......................................---**G****G****C**..**C****C****G****C****C**GAA**G****G****G****A**UAA..---..-.----..........----..........................................................................................----..-.---.-.-CG.**A****U****C**.**U**CA**G****G****C**.............GCAAG**G**.**G****A****C****A****G****A****G****G**GGGCACU | |
|  |  | NC\_004463.1/6318642-6318826  | ACGGC...**C****A**.**C**.**G**.**U**.**C****G****G****G****A****G**A**G**.A**C****C****G****G****C**...........UUUAGGC......................................A**G****C****C****G****G**..**C****G**.**C****C**GAA**G****G****A****G**CA...A..**C****C**G...............CCCC........................**G****G**.AAA**C****U****C****U**CA**G****G****C**..........................................................................A.A.A.A.**G**.**G****A**.**C****C****G**..-..**C****G**.**U**.**G**GC...UUUGACAG............................................................**C****A****U****C****U****G****G****A****A**A**G**A.**G**.**G****C****G****C****C**GACGAGCU.......................UGAGGCUCGGA................................C**A****G****C****G****U**..**C****C****G****C****C**GAC**G****G****G****A**UAA..---..-.----..........----..........................................................................................----..-.---.-.-UA.**C****U****C**.**U**CA**G****G****C**..............ACAG**C**.**G****A****C****A****G****A****U****G**GGGCUUC | |
|  |  | NC\_002737.1/1046361-1046528  | UGAUG...**U****C**.**A**.**U**.**G**.**C****A****G****G****A****G**A**A**.-**G****A****A**--...........UUUU.........................................---**U****U****U**..**C****G**.**C****C**GAA**G****G****A****G**UU...A..--................----........................--.-UA**C****U****C****U**CA**G****G****U**..............................................................GUUCAGUUUUUGA.A.C.G.**G**.**G****A**.**C****U****G**..**U**..**U****U**.**G**.**A**U....GGACGGA-............................................................**C****U****U****C****U****G****G****A****G**A**G**A.**C**.**C****U**---...............................UAUU.......................................----**A****G**..**G****C****G****C****C**GAA**G****G****G****G**CAA..**G****G****C**..**A**.----..........UAC-..........................................................................................----..**U**.**G****C****U**.C.AAU.**C****U****C**.**U**CA**G****G****C**..............AAAA**G**.**G****A****C****A****G****A****A****G**GUAAAAU | |
|  |  | NC\_004070.1/958264-958431  | UGAUG...**U****C**.**A**.**U**.**G**.**C****A****G****G****A****G**A**A**.-**G****A****A**--...........UUUU.........................................---**U****U****U**..**C****G**.**C****C**GAA**G****G****A****G**UU...A..--................----........................--.-UA**C****U****C****U**CA**G****G****U**..............................................................GUUCAGUUUUUGA.A.C.G.**G**.**G****A**.**C****U****G**..**U**..**U****U**.**G**.**A**U....GGACGGA-............................................................**C****U****U****C****U****G****G****A****G**A**G**A.**C**.**C****U**---...............................UAUU.......................................----**A****G**..**G****C****G****C****C**GAA**G****G****G****G**CAA..**G****G****C**..**A**.----..........UAC-..........................................................................................----..**U**.**G****C****U**.C.AAU.**C****U****C**.**U**CA**G****G****C**..............AAAA**G**.**G****A****C****A****G****A****A****G**GUAAAAU | |
|  |  | NC\_004606.1/1088784-1088951  | UGAUG...**U****C**.**A**.**U**.**G**.**C****A****G****G****A****G**A**A**.-**G****A****A**--...........UUUU.........................................---**U****U****U**..**C****G**.**C****C**GAA**G****G****A****G**UU...A..--................----........................--.-UA**C****U****C****U**CA**G****G****U**..............................................................GUUCAGUUUUUGA.A.C.G.**G**.**G****A**.**C****U****G**..**U**..**U****U**.**G**.**A**U....GGACGGA-............................................................**C****U****U****C****U****G****G****A****G**A**G**A.**C**.**C****U**---...............................UAUU.......................................----**A****G**..**G****C****G****C****C**GAA**G****G****G****G**CAA..**G****G****C**..**A**.----..........UAC-..........................................................................................----..**U**.**G****C****U**.C.AAU.**C****U****C**.**U**CA**G****G****C**..............AAAA**G**.**G****A****C****A****G****A****A****G**GUAAAAU | |
|  |  | NC\_006086.1/969359-969526  | UGAUG...**U****C**.**A**.**U**.**G**.**C****A****G****G****A****G**A**A**.-**G****A****A**--...........UUUU.........................................---**U****U****U**..**C****G**.**C****C**GAA**G****G****A****G**UU...A..--................----........................--.-UA**C****U****C****U**CA**G****G****U**..............................................................GUUCAGUUUUUGA.A.C.G.**G**.**G****A**.**C****U****G**..**U**..**U****U**.**G**.**A**U....GGACGGA-............................................................**C****U****U****C****U****G****G****A****G**A**G**A.**C**.**C****U**---...............................UAUU.......................................----**A****G**..**G****C****G****C****C**GAA**G****G****G****G**CAA..**G****G****C**..**A**.----..........UAC-..........................................................................................----..**U**.**G****C****U**.C.AAU.**C****U****C**.**U**CA**G****G****C**..............AAAA**G**.**G****A****C****A****G****A****A****G**GUAAAAU | |
|  |  | NC\_003485.1/1022142-1022310  | UGAUG...**U****C**.**A**.**U**.**G**.**C****A****G****G****A****G**A**A**.-**G****A****A**--...........UUUA.........................................U--**U****U****U**..**C****G**.**C****C**GAA**G****G****A****G**UU...A..--................----........................--.-UA**C****U****C****U**CA**G****G****U**..............................................................GUUCAGUUUUUGA.A.C.G.**G**.**G****A**.**C****U****G**..**U**..**U****U**.**G**.**A**U....GGACGGA-............................................................**C****U****U****C****U****G****G****A****G**A**G**A.**C**.**C****U**---...............................UAUU.......................................----**A****G**..**G****C****G****C****C**GAA**G****G****G****G**CAA..**G****G****C**..**A**.----..........UAC-..........................................................................................----..**U**.**G****C****U**.C.AAU.**C****U****C**.**U**CA**G****G****C**..............AAAA**G**.**G****A****C****A****G****A****A****G**GUAAAAU | |
|  |  | NZ\_AAAV02000002.1/98990-98767  | UGAUC...**G**-.-.**C**.**G**.**C****G****G****G****A****G**A**G**.U**U****C****U****C****U**CGAUUCG....UUCAGGAUC....................................G**A****G****A****G****A**..**C****G**.**C****C**GAA**G****G****A****G**CA...A..**C****C**G...............CCCC........................**G****G**.AAU**C****U****C****U**CA**G****G****C**..........................................................................A.A.A.U.**G**.**G****A**.**C****C****G**..**C**..**G**-.**U**.**U**.....GGUCGAUAGCGACG......................................................-**C****U****C****U****G****G****A****A**A**G**U.**G**.**C****C****A****C****U**CCCA...........................CAAAGG.....................................G**A****A****G****G****G**..**C****C****G****C****C**GAA**G****G****G****G**UAA..**C****C****C**..**C**.**A****U****C****C**A.........AACC.............................................................................CGUCUGGAGAGGC**G****G****G****C**..**G**.**G****G****G**.G.AAAG**C****U****C**.**U**CA**G****G****U**.............UUCCG**U**.**G****A****C****A****G****A****G****G**GGGCGCC | |
|  |  | NC\_006370.1/2453318-2453611  | AUGAA...**G****A**.**U**.**A**.**G**.**C****A****G****G****A****G**A**G**.U**G****G****C****A****U**AU.........UAUUCGCUAGCAGAAUCAUUCUGAUACGAGUA.............U**A****A****G****C****C**..**C****G**.**C****C**GAA**G****A****A****G**UA...A..**G****C**GUUCUUAA........ACCGUUUGUUACUGUUCAAACACAAGAC**G****U**UAAU**C****U****U****U**CA**G****G****C**........................................ACUGGAUUACAUACCUUUUAACAAGGUAUCCGGU-.-.A.U.**G**.**G****A**.**C****U****G**..**U**..**U****A**.**U**.**U**.....GGAGGAG-............................................................**C****C****U****C****U****G****G****A****G**A**G**A.**C**.**C****C****G****U**-...............................UAAA.......................................--**U****C****G****G**..**G****C****G****C****C**GAA**G****G****A****G**CAA..**G****C****U**..**U**.**U****C****C****U**UUUUGCUG..UUUU..................................................................................AACAUGCA**U****G****G****A**..**A**.**A****G****U**.G.AAA.**C****U****C**.**U**CA**G****G****C**..............AAAA**G**.**G****A****C****A****G****A****G****G**AGAUAAG | |
|  |  | NC\_006582.1/2619955-2619769  | AUGAU...**G****G**.**U**.**A**.**A**.**A****G****G****G****A****G**A**G**.A**C****U****G****G****G**CA.........UUUAC........................................G**C****C****C****A****G**..**C****G**.**C****C**GAA**G****G****A****G**CA...A..**G****C**AUUUU...........----........................**G****C**GAAU**C****U****C****U**CA**G****G****C**..........................................................................A.A.AGA.**A**.**G****A**.**C****U****U**..**U**..**U****A**.**C**.**U**.....UGACGCA-............................................................**U****C****U****C****U****G****G****A****G**A**G**C.**G**.**C****U****U**--...............................UUUAU......................................---**A****A****G**..**C****C****A****C****C**UAC**G****A****A****G**ACA..**A****U****U**..**C**.**G****G****C****U**..........UUU-..........................................................................................**G****G****C****C**..**G**.**A****G****G**.G.AAA.**C****U****U**.**U**CU**G****G****U**..............UCAA**G**.**G****A****C****A****G****G****G****C**AUACACA | |
|  |  | NZ\_AAAW03000005.1/66004-66178  | AUGAG...**A****U**.**U**.**U**.**A**.**C****G****G****G****A****G**A**A**.G**G****G****C**--...........ACG-.........................................---**G****C****C**..**C****G**.**C****C**GAA**G****A****A****G**UU...G..--................----........................--.AAA**C****U****U****U**CA**G****G****U**............................................................GACUGGAAUAAGUC-.-.A.G.**G**.**G****A**.**C****C****G**..**U**..**A****A**.**A**.**U**.....UGACAGCA............................................................-**C****U****C****U****G****G****A****G**A**G**A.**C**.**U****C**---...............................GAUU.......................................A---**G****A**..**G****C****G****C****C**GAA**G****G****G****G**CAA..**U****A****C**..**A**.**A****U****G**-..........ACGU..........................................................................................--**G****U**..**U**.**G****U****U**.A.AAU.**C****U****C**.**U**CA**G****G****C**..............AAAA**G**.**G****A****C****A****G****A****G****A**AGGAACA | |
|  |  | NC\_006177.1/3096183-3095966  | GGGCC...**G****G**.**C**.**G**.**G**.**C****G****G****G****A****G**A**G**.U**C****U****U****C****C**...........UUCAG........................................G**G****G****A****A****G**..**C****G**.**C****C**GAA**G****G****A****G**CA...A..**G****C**GGGGAGGGGGUCGGACUCUC.......................C**G****U**GAAU**C****U****C****U**CA**G****G****C**..........................................................................A.C.C.A.**G**.**G****A**.**C****C****G**..**U**..**C****G**.**C**.**C**.....UGGGCGUGCACAGCGCGCCCCCCCCCCGC.......................................**C****C****U****C****U****G****G****A****G**A**G**-.**C**.**C****G****C**--...............................CCUG.......................................C--**G****C****G**..**G****C****A****C****C**GAA**G****G****A****G**CAA..**G****G****C**..**C**.**U****C**--..........CCGC..........................................................................................--**A****C**..**G**.**G****C****C**.-.AAA.**C****U****C**.**U**CA**G****G****U**.............GGAGG**A**.**G****A****C****A****G****A****G****G**AACGACC | |
|  |  | NZ\_AAHG01000005.1/320753-320507  | UGAUC...**U****G**.**C**.**G**.**G**.**C****G****G****G****A****G**A**G**.U**U****C****U****G****C**A..........AGUAGGUACA...................................A**G****C****A****G****G**..**C****G**.**C****C**GUA**G****G****A****G**CA...AA.**C****C**CU..............CCCC.......................A**G****G**.AAU**C****U****C****U**CA**G****G****C**..........................................................................C.C.A.U.**G**.**U****A**.**C****C****G**..**C**..**C****G**.**C**.**G**G....CAAGGCAA............................................................-**C****U****C****U****G****G****A****A**A**G**C.**A**.**G****C****A****U****G**CUGUCCGUUUGUGUCCCAACCCAGUUGGGGGCAAACCAGGACACG.............................C**C****A****U****G****C**..**U****C****A****C****C**GAC**G****G****U****G**CAA..**G****C****G**..**G**.**A****G****C****U**GUGC......GAGA......................................................................................GCAC**G****G****U****C**..**C**.**U****G****C**GG.AAA.**C****U****C**.**U**CA**G****G****U**..............CCAA**U**.-**A****C****A****G****A****G****C**GGGGAGG | |
|  |  | NZ\_AAAG02000001.1/778571-778342  | CGAAC...**C****G**.**U**.**U**.**A**.**C****G****G****G****A****G**A**G**.A**U****C****G****G****U**CGCCCGUGCC.AUCAGGCGCGGGGCAG.............................A**G****C****C****G****G**..**C****G**.**C****C**GAA**G****G****A****G**CA...A..**C****C**G...............CCCC.......................C**G****G**.AAA**C****U****C****U**CA**G****G****C**..........................................................................A.A.A.U.**G**.**G****A**.**C****C****G**..**U**..**A****A**.**C**.**G**.....GCAUGGGU............................................................**U****C****U****C****U****G****G****A****A**A**G**C.**A**.**G****A****A****C****A**GGCGGCGGGAU....................GACGUCCCCGGG...............................C**C****G****U****U****C**..**U****C****A****C****C**GAA**G****A****U****G**GAA..**G****C****C**..**G**.**G****C****G****G**..........UCCG..........................................................................................**A****C****C****C**..**C**.**G****G****U**.G.ACU.**C****U****U**.**U**CA**G****G****U**..............UUCG**A**.**G****A****C****A****G****A****G****G**GGGGCGU | |
|  |  | NC\_006087.1/1714518-1714292  | GAGGC...**C****G**.**A**.**U**.**G**.**C****G****G****G****A****G**A**G**CU**G****C****G****G****G**GG.........AGAACCC......................................G**C****C****C****G****C**GG**C****A**.**C****C**GAA**G****G****A****G**CA...A..**U****C**C...............UCCC.......................C**G****A**CAAU**C****U****C****U**CA**G****G****U**..........................................................................A.C.G.C.**G**.**U****A**.**C****C****G**..**C**..**A****U**.**C**.**G**G....ACUGGCCA............................................................-**C****U****C****U****G****A****A****A**A**G**C.**A**.**G****G****G****C****A**GCGUCGACCCUCCGGGUCGAUCCCGCGG...GACAUG.....................................C**U****G****G****C****C**..**U****C****G****C****C**CAC**G****G****U****G**AAA..**G****C****C**..**G**.**C****G****A****A**..........CGC-..........................................................................................**U****U****C****G**..**C**.**G****G****U**.G.AAA.**C****U****C**.**U**CA**G****G****C**..............CGGA**U**.**G****A****C****A****G****A****G****G**GGGAGUU | |
|  |  | NC\_004193.1/1936325-1936128  | AAGAU...**U****A**.**C**.**A**.**A**.**G****G****G****G****A****G**A**G**.U**U****U****A****C****A**ACG........AAU-.........................................-**A****G****U****A****A**..**C****G**.**C****C**GAA**G****G****A****G**CA...A..**G****U**G...............AAGA........................**G****C**GAAU**C****U****C****U**CA**G****G****C**.........................................................................CA.A.A.A.**A**.**G****A**.**C****U****C**..**U**..**U****G**.**U**.**A**.....UGACGCAA............................................................-**C****U****C****U****G****G****A****G**A**G**U.**G**.**U****U****U****A****C**...............................GAAG.......................................-**G****U****A****A****A**..**C****C****A****C****C**CAC**G****A****A****G**CAA..**A****U****A**..**U**.**U****U****G****U**UC........UUUU...................................................................................UUGAAGA**A****U****G****A**..**A**.**U****A****U**.G.CAA.**C****U****U**.**U**CU**G****G****U**..............AUAA**G**.**G****A****C****A****G****A****G****A**UUUCUUC | |
|  |  | NZ\_AADY01000004.1/128460-128694  | CGCAU...**C****C**.**G**.**A**.**C**.**C****G****G****G****A****G**A**G**.C**G****C****G****C****A**CAG........UCU-.........................................-**U****G****C****G****C**..**C****G**.**C****C**GAA**G****G****G****G**UA...-..**U****C**................ACCC........................**G****A**.AAA**C****U****C****U**CA**G****G****C**..........................................................................A.C.C.AC**G**.**G****A**.**C****C****G**..**U**..**U****C**.**G**.**G**AUCG.GCAUGCAACAUGCACACGAUGCAUGCCGCA......................................-**C****U****C****U****G****G****A****G**A**G**C.**G**.**G****C****A****C****C**CGCCA..........................UCAAUGGCGA.................................G**C****G****U****G****C**..**C****C****A****C****C**GAA**G****G****G****G**CGC..**G****C****G**..**A**.**G****G****A****U**CG........GAUC.................................................................................CAACGGAUC**G****G****C****C**..**U**.**C****G****U**.-.AAU.**C****U****C**.**U**CA**G****G****U**.............AUCGA**G**.**G****A****C****A****G****A****G****G**GGUCAUC | |
|  |  | NZ\_AAIT01000008.1/195925-195747  | GGUCG...**G****A**.**A**.**A**.**U**.**C****G****G****G****A****G**A**C**.A**U****U****G****G****C**G..........CAA-.........................................-**G****C****C****A****G**..**G****G**.**C****C**GAA**G****G****A****G**CA...A..**C****C**G...............CCCC.......................G**G****G**.AAU**C****U****C****U**CA**G****G****C**..........................................................................A.A.A.G.**G**.**G****A**.**C****C****G**..**U**..**U****U**.**U**.**C**.....CGCCGAAC............................................................**G****C****U****C****U****G****G****A****G**A**G**A.**G**.**G****C****G****G****C**...............................UUGC.......................................-**G****C****C****G****C**..**C****C****G****C****C**GAA**G****G****G****A**UAA..---..-.----..........----..........................................................................................----..-.---.-.-CG.**A****U****C**.**U**CA**G****G****C**GCCCCCGUUUCGGGGUAG**G**.**G****A****C****A****G****A****G****G**GGGCAUC | |
|  |  | NZ\_AABG04000005.1/22395-22216  | AUGAU...**U****G**.**U**.**C**.**A**.**U****G****G****G****A****G**A**G**.U**C****A****U****U****G**GAG........AUGUU........................................C**C****A****A****U****G**..**C****A**.**C****C**GAA**G****G****A****G**UG...A..--................----........................--.-UA**C****U****U****U**CA**G****G****U**..........................................................................U.U.U.G.**U**.**G****A**.**C****C****A**..**U**..**G****G**.**C**.**C**.....GGACGCA-............................................................**C****C****U****C****U****G****G****A****G**A**G**A.**C**.**C****G**---...............................UAAA.......................................-----**G**..**G****C****G****C****C**GAA**G****G****G****G**CAA..**A****U****G**..**A**.**A****C****A****G**U.........UUUU....................................................................................GGGGAA**C****U****G****U**..**U**.**C****A****G**.A.AAU.**C****U****C**.**U**CA**G****G****C**..............AAAA**G**.**G****A****C****A****G****A****G****A**AGGAAUU | |
|  |  | NC\_005296.1/4348220-4348399  | CUAAG...**U****C**.**G**.**C**.**G**U**C****G****G****G****A****G**A**G**.A**U****C****G****G****C**CG.........CAAG.........................................-**G****C****C****G****G**..**C****G**.**C****C**GAA**G****G****A****G**CA...A..**C****C**G...............CCCC........................**G****G**.AAA**C****U****C****U**CA**G****G****C**..........................................................................A.A.A.C.**G**.**G****A**.**C****C****G**..**C**..**G****C**.**G**.**G**C....CGACUGA-............................................................**C****A****U****C****U****G****G****A****A**A**G**A.**G**.**A****C****C****C****U**...............................GCCGGGCGAUCCCGGG...........................A**C****G****G****G****U**..**C****C****G****C****C**GAC**G****G****G****A**UAA..---..-.----..........----..........................................................................................----..-.---.-.-UG.**C****U****C**.**U**CA**G****G****C**..............ACAG**C**.**G****A****C****A****G****A****U****G**GGGCUCG | |
|  |  | NC\_003295.1/3545575-3545809  | GCCAU...**C****C**.**A**.**U**.**C**.**C****G****G****G****A****G**A**G**.C**G****C****G****U****C**GGCCG......CAUACGGCC....................................A**G****U****C****G****C**..**C****G**.**C****C**GAA**G****G****G****G**UA...-..**G****C**................ACCC........................**G****A**.AAA**C****U****C****U**CA**G****G****C**..........................................................................AUC.C.A.**G**.**G****A**.**C****C****G**..**G**..**G****U**.**G**.**G**AU...CGGCUGGGCAUGCGUCCGGCCGCA............................................-**C****U****C****U****G****G****A****G**A**G**C.**G**.**G****C****G****C****C**GC.............................GUCACCAUCGC................................G**G****G****C****G****C**..**C****C****A****C****C**GAA**G****G****G****G**CUCA.**C****G****G**..**A**.**A****C****C****G**UCGCACGCG.UUGU........................................................................................GU**C****G****C****U**..**U**.**C****C****C**.-.AAU.**C****U****C**.**U**CA**G****G****U**.............ACCAA**G**.**G****A****C****A****G****A****G****G**GGCCACC | |
|  |  | NZ\_AAIO01000044.1/20448-20176  | AUGAA...**G****G**.**U**.**A**.**G**.**C****A****G****G****A****G**A**G**.-**U****G****G****G****G**AA.........UUGA.........................................-**C****C****C****C****A**..**C****A**.**C****C**GAC**G****A****G****G**CA...A..--................----........................--.---**C****U****U****U**--**G****G****U**UUUAUUUGAAGCGACGGUUAUAUUGAAUCGAAUCUUCCCAUAAAAACAAAGCACUCUUUCAGGUGCCGAUAGGC-.-.G.U.**G**.**G****A**.**C****U****G**..**U**..**U****A**.**C**.**U**.....GGACGAG-............................................................**C****C****U****C****U****G****G****A****G**A**G**A.**C**.**U****G****C****C****A**AUUGCACUUAAU...................UUAAAGUGUGAU...............................C**A****G****G****U****G**..**G****C****G****C****C**GAA**G****G****C****G**AAA..**G****U****G**..**U**.**C****G****U****G**GUGU......UAUU......................................................................................GAAC**C****G****C****G**..**A**.**C****G****C**.G.AAA.**C****G****C**.**U**CA**G****G****C**..............AAAA**G**.**G****A****C****A****G****A****G****G**AGAGGAU | |
|  |  | NC\_004116.1/1147464-1147676  | UGACG...**U****C**.**A**.**U**.**U**.**C****A****G****G****A****G**A**A**.G**A****A****A****G****U**...........UAG-.........................................-**A****C****U****U****U**..**C****G**.**C****C**GAA**G****G****A****A**UU...A..--................----........................--.-CA**C****U****C****U**CA**G****G****U**.................................................................GUCUUAAGA-.-.C.A.**G**.**G****A**.**C****U****G**..**A**..**U****U**.**G**.**A**C....AGACGGA-............................................................**C****U****U****C****U****G****G****A****G**A**G**A.**C**.**C****U****A****U****A**AGUAGCAACAUCUUUGUAUUGACAC......CAAGAUGUG..................................C**U****C****U****A****G**..**G****C****G****C****C**GAA**G****G****G****G**CAA..**G****A****A**..**G**.**A****G****U****A**..........AAAC..........................................................................................**A****A****C****U**..**C**.**C****U****C**.C.AAU.**C****U****C**.**U**CA**G****G****C**..............AAAA**G**.**G****A****C****A****G****A****A****G**CUAAAAG | |
|  |  | NC\_004368.1/1250560-1250772  | UGACG...**U****C**.**A**.**U**.**U**.**C****A****G****G****A****G**A**A**.G**A****A****A****G****U**...........UAG-.........................................-**A****C****U****U****U**..**C****G**.**C****C**GAA**G****G****A****A**UU...A..--................----........................--.-CA**C****U****C****U**CA**G****G****U**.................................................................GUCUUAAGA-.-.C.A.**G**.**G****A**.**C****U****G**..**A**..**U****U**.**G**.**A**C....AGACGGA-............................................................**C****U****U****C****U****G****G****A****G**A**G**A.**C**.**C****U****A****U****A**AGUAGCAACAUCUUUGUAUUGACAC......CAAGAUGUG..................................C**U****C****U****A****G**..**G****C****G****C****C**GAA**G****G****G****G**CAA..**G****A****A**..**G**.**A****G****U****A**..........AAAC..........................................................................................**A****A****C****U**..**C**.**C****U****C**.C.AAU.**C****U****C**.**U**CA**G****G****C**..............AAAA**G**.**G****A****C****A****G****A****A****G**CUAAAAG | |
|  |  | NZ\_AAFG02000008.1/195700-195882  | CCGCA...**G****C**.**A**.**C**.**A**.**U****G****G****G****A****G**A**A**.-**C****C****G****A****C**C..........AAGAGGCUUGGCGCCUCGCU.........................G**A****U****C****G****G**..**U****G**.**C****C**GAA**G****G****A****G**CA...A..**C****C**G...............CCCC........................**G****G**.AAA**C****U****C****U**CA**G****G****C**.........................................................................CA.C.A.A.**G**.**G****A**.**C****C****G**..**U**..**G****U**.**G**.**C**G....CGUAACUA............................................................-**C****U****C****U****G****G****A****G**A**G**A.**G**.**C****C****C****G****A**...............................CCAG.......................................A**U****C****G****G****G**..**C****C****G****C****C**GAA**G****G****G****A**UAA..---..-.----..........----..........................................................................................----..-.---.-.-CG.**A****U****C**.**U**CA**G****G****C**..............GAAG**G**.**G****A****C****A****G****A****G****G**GGGCAUC | |
|  |  | NZ\_AAIG01000003.1/211451-211270  | ACGCA...**A****C**.**C**.**G**.**U**.**A****G****G****G****A****G**A**C**.U**C****U****U****G****G**GCGCG......CAAACCACGCGCA................................C**C****C****C****A****G**..**G****G**.**C****C**GAA**G****G****A****G**CA...A..**C****C**G...............CCCC........................**G****G**UAAA**C****U****C****U**CA**G****G****C**..........................................................................A.A.A.C.**G**.**G****A**.**C****C****U**..**G**..**U****G**.**G**.**U**UGU..GGACAUAA............................................................-**C****U****C****U****G****G****A****G**A**G**U.**G**.**A****G****G****G****C**...............................AGUG.......................................-**C****C****C****C****U**..**C****C****G****C****C**GAA**G****G****G****A**UAA..---..-.----..........----..........................................................................................----..-.---.-.-CG.**A****U****C**.**U**CA**G****G****C**..............CGAA**G**.**G****A****C****A****G****A****G****G**GGGCAUC | |
|  |  | NZ\_AAIS01000002.1/1019986-1020178  | UUAAG...**U****C**.**G**.**C**.**G**U**C****G****G****G****A****G**A**G**.A**U****C****G****G****C**CGU........UUCUGAAUGGAAACA..............................G**G****C****C****G****G**..**C****G**.**C****C**GAA**G****G****A****G**CA...A..**C****C**G...............CCCC........................**G****G**.AAA**C****U****C****U**CA**G****G****C**..........................................................................A.A.A.C.**G**.**G****A**.**C****C****G**..**C**..**G****C**.**G**.**A**.....CUUGUUA-............................................................**C****A****U****C****U****G****G****A****A**A**G**A.**G**.**A****C****C****C****U**GCGGGCC........................GACAGCCCGG.................................G**A****G****G****G****U**..**C****C****G****C****C**GAC**G****G****G****A**UAA..---..-.----..........----..........................................................................................----..-.---.-.-UA.**C****U****C**.**U**CA**G****G****C**..............ACAG**C**.**G****A****C****A****G****A****U****G**GGGUCUC | |
|  |  | NZ\_AAEB02000107.1/3157-3329  | CAGAG...**G****G**.**C**.**C**.**G**.**C****G****G****G****A****G**A**G**.A**G****C****C****G****C**CG.........CGC-.........................................-**G****C****G****G****C**..**C****G**.**C****C**GAA**G****G****U****G**AA...A..**G****G**U...............CCCC..............GGUUUCGGGG**C****C**GAAU**C****U****C****U**CA**G****G****C**..........................................................................A.A.A.A.**G**.**G****A**.**C****C****G**..**C**..**G****G**.**C**.**C**.....GGAGGGC-............................................................**G****U****C****C****U****G****G****A****G**A**G**U.**C**.**C****G****G****C**-...............................AGGG.......................................--**G****C****C****G**..**G****C****G****C****C**GAC**G****G****G****G**UAA..---..-.----..........----..........................................................................................----..-.---.-.-CA.**C****U****C**.**U**CA**G****G****U**..............UCCG**G**.**G****A****C****A****G****G****A****C**GACAAAG | |
|  |  | NZ\_AAAI03000001.1/792054-792282  | CGCAU...**C****C**.**G**.**G**.**C**.**C****G****G****G****A****G**A**G**.C**G****C****G****C****A**G..........CGAU.........................................-**U****G****C****G****C**..**C****G**.**C****C**GAA**G****G****G****G**UA...-..**U****C**................ACCC........................**G****A**.AAA**C****U****C****U**CA**G****G****C**..........................................................................A.C.C.AU**G**.**G****A**.**C****U****G**..**G**..**C****U**.**G**.**G**AUCG.GCAUGCAACAUGCACACAAUGCAUGCCGCA......................................-**C****U****C****U****G****G****A****G**A**G**C.**G**.**A****C****A****U****C**CGCUAUUG.......................CAAGGCAAUGGUGA.............................G**C****A****U****G****U**..**C****C****A****C****C**GAA**G****G****G****G**CGC..**A****C****G**..**A**.**A****G****C****A**..........CCAC..........................................................................................-**G****C****U**..**U**.**C****G****U**.-.AAU.**C****U****C**.**U**CA**G****G****U**.............AUCGA**G**.**G****A****C****A****G****A****G****G**GGUCAUC | |
|  |  | NZ\_AAIE01000001.1/414702-414480  | ACGC-...**C****C**.**G**.**U**.**G**.**C****G****G****G****A****G**A**G**.U**C****C****C****G****G**UCG........CUCGCCG......................................U**U****C****G****G****G**.A**C****G**.**C****C**GAA**G****G****A****G**CA...A..**G****C**UC..............UCCC.......................C**A****U**.AAA**C****U****C****U**CA**G****G****C**..........................................................................-.C.C.C.**C**.**A****A**.**C****C****G**..**C**..**C****G**.**G**.**G**.....UCAGGCAA............................................................-**C****U****C****U****G****G****A****A**A**G**U.**G**.**G****A****C****G****C**...............................CGUGG......................................G**G****U****G****U****C**..**C****C****G****C****C**GAC**G****G****U****G**AAA..**G****C****C**..-.**G****C****G****U**UCCCCGCCCGCAGC......................................................................UCCCCGGACUGCCGGCGGCG**G****C****G****C**..**U**.**G****G****U**.G.AAG.**C****U****C**.**U**CA**G****G****U**..............-CAA**U**.**G****A****C****A****G****A****G****G**GGGAGGC | |
|  |  | NZ\_AADT03000002.1/77628-77372  | AUGAG...**G****G**.**G**.**A**.**G**.**U****G****G****A****A****G**A**G**.A**C****C****C****U**-...........UUAA.........................................--**A****G****G****G**..**C****G**.**C****C**GAA**G****G****A****G**CA...A..**U****U**UU..............CCCC.......GGCAGGUGACGGGGGAA**G****G**.AAC**C****U****C****U**CA**G****G****C**..........................................................................A.A.A.A.**G**.**U****A**.**C****C****G**..**C**..**U****C**.**C**.**C**.....GGACCCAA............................................................-**C****U****C****U****G****G****A****G**A**G**C.**U**.**U****C****C****U****U**AAAGCCGGUUUCAACACCCGACC........UUAGGGUCGGCGGGUAGCCGCUUGGCCCGGAAACAGCCGGCUUC**A****G****G****G****G**..**A****C****A****C****C**AAA**G****G****G****G**AA-..**G****G****C**..**A**.**G**---..........GCAA..........................................................................................---**C**..**U**.**G****C****C**.U.AAU.**C****U****C**.**U**CA**G****G****U**..............ACCA**G**.**G****A****C****A****G****A****G****G**CCAUCAC | |
|  |  | NC\_005956.1/1435974-1435783  | AUUC-...**U****U**.**U**.**U**.**G**.**C****G****G****G****A****G**A**G**.U**G****C****A****G****G**CUGCAAG....UUUUUGG......................................G**U****C****U****G****C**..**C****G**.**C****C**GAA**G****G****G****G**AA...A..**A****U**A...............GCCC........................**G****U**.AAU**C****U****C****U**CA**G****G****C**.......................................................................UCU-.-.A.A.**G**.**G****A**.**C****C****G**..**U**..**A****A**.**A**.**A**GAAG.AGAGGCAG............................................................-**A****U****C****U****G****G****A****A**A**G**U.**C**.**G****G****G**--...............................AGGG.......................................---**G****C****C**..**G****C****G****C****C**GAA**G****G****U****G**UAA..**G****C****G**..**A**.**A****A****A**-..........CUU-..........................................................................................-**U****U****U**..**U**.**C****G****C**.G.AGU.**C****U****C**.**U**CA**G****G****U**..............-UUG**U**.**A****A****C****A****G****A****G****G**GGUGCAG | |
|  |  | NZ\_AAEM01000001.1/146378-146606  | UCGUU...**A****C**.**C**.**G**.**U**.**C****A****G****G****A****G**A**G**.C**G****U****U****G****C**CGUCAG.....CACACCGAC....................................G**G****C****A****G****C**..**C****G**.**C****C**GAA**G****G****C****G**CA...G..**A****G**GACA............CACC.......................U**C****G**.-AA**C****G****C****U**CA**G****G****C**..........................................................................A.A.A.A.**G**.**G****A**.**C****U****G**..**G**..**C****A**.**A**.**A**.....CGCCUCCCAGCGGAGGCGUCCU..............................................-**C****A****C****U****G****G****A****G**A**G**A.**G**.**G****C****U****G****C**A..............................CCCGG......................................G**G****C****A****G****C**..**C****C****A****C****C**GAA**G****G****G****G**CAA..**G****C****G**..**G**.**G****U****C****G**AGGUGCG...ACAC......................................................................................ACCG**G****A****A****C**..**C**.**G****G****C**.C.AAU.**C****U****C**.**U**CA**G****G****U**.............AAAGC**G**.**G****A****C****A****G****C****G****A**GGGCAGG | |
|  |  | NZ\_AAFQ02000006.1/211115-211350  | UUGAG...**A****G**.**G**.**U**.-.**C****A****G****G****A****G**A**G**.A**G****U****U****U****U**C..........AUCGC........................................G**A****A****A****A****C**..**C****G**.**C****C**GAA**G****G****C****G**CACGAA..**C****A**CCAUGAGGGCA.....CCCC.............UCGUGGUGUAC**U****G**.-AA**C****G****C****U**CA**G****G****C**..........................................................................A.A.A.A.**G**.**G****A**.**C****U****G**GC**A**..**A****C**.**C**.**G**UU...UUCCGGCGCCUGGCGCCGGAAAACGUUCC.......................................**C****U****A****C****U****G****G****A****G**A**G**A.**G**.**A****C****C****G****C**U..............................UUCGA......................................G**G****C****G****G****C**..**C****C****A****C****C**GAA**G****G****A****G**CAA..**G****C****C**..**U**.**G****C**--..........AUCG..........................................................................................--**C****C**..**A**.**G****G****U**.G.AAU.**C****U****C**.**U**CA**G****G****U**.............AAAGU**G**.**G****A****C****A****G****C****A****G**GGGCAGC | |
|  |  | NC\_006348.1/3101409-3101131  | CCGCA...**C****G**.**C**.**G**.**U**.**C****G****G****G****A****G**A**G**.C**G****C****G****C****G**GCCGCCGUUCGUUGAUCGAAAGGCAAG.............................C**C****G****C****G****C**..**C****G**.**C****C**GAA**G****G****G****G**CA...-..**C****A**................CCC-........................-**G**CAAA**C****U****C****U**CA**G****G****C**..........................................................................A.A.A.A.**G**.**G****A**.**C****C****G**..**A**.C**C****G**.**C**.**G**U....CGAAGAAUCCGUCUGACGACAUGCGUCGGACGGCGUUUUUCGCA........................-**C****U****C****U****G****G****A****G**A**G**C.**G**.**G****C****A****G****U**AGCCGCGUGCGCGCAACGCGACG........CACGGGCA...................................G**G****C****U****G****C**..**C****C****A****C****C**GAA**G****G****G****G**CGC..**G****C****G**..**C**.**G****A****G****C**CGGAUCG...AAAC..........................................................................................**C****G****U****C**..**G**.**C****G****C**.-.AAU.**C****U****C**.**U**CA**G****G****U**.............AUCGA**G**.**G****A****C****A****G****A****G****G**GGCAUGC | |
|  |  | NC\_006350.1/3984000-3984278  | CCGCA...**C****G**.**C**.**G**.**U**.**C****G****G****G****A****G**A**G**.C**G****C****G****C****G**GCCGCCGUUCGUUGAUCGAAAGGCAAG.............................C**C****G****C****G****C**..**C****G**.**C****C**GAA**G****G****G****G**CA...-..**C****A**................CCC-........................-**G**CAAA**C****U****C****U**CA**G****G****C**..........................................................................A.A.A.A.**G**.**G****A**.**C****C****G**..**A**.C**C****G**.**C**.**G**U....CGAAGAAUCCGUCUGACGACAUGCGUCGGACGGCGUUUUUCGCA........................-**C****U****C****U****G****G****A****G**A**G**C.**G**.**G****C****A****G****U**AGCCGCGUGCGCGCAACGCGACG........CACGGGCA...................................G**G****C****U****G****C**..**C****C****A****C****C**GAA**G****G****G****G**CGC..**G****C****G**..**C**.**G****A****G****C**CGGAUCG...AAAC..........................................................................................**C****G****U****C**..**G**.**C****G****C**.-.AAU.**C****U****C**.**U**CA**G****G****U**.............AUCGA**G**.**G****A****C****A****G****A****G****G**GGCAUGC | |
|  |  | NZ\_AAHM01000006.1/19061-18783  | CCGCA...**C****G**.**C**.**G**.**U**.**C****G****G****G****A****G**A**G**.C**G****C****G****C****G**GCCGCCGUUCGUUGAUCGAAAGGCAAG.............................C**C****G****C****G****C**..**C****G**.**C****C**GAA**G****G****G****G**CA...-..**C****A**................CCC-........................-**G**CAAA**C****U****C****U**CA**G****G****C**..........................................................................A.A.A.A.**G**.**G****A**.**C****C****G**..**A**.C**C****G**.**C**.**G**U....CGAAGAAUCCGUCUGACGACAUGCGUCGGACGGCGUUUUUCGCA........................-**C****U****C****U****G****G****A****G**A**G**C.**G**.**G****C****A****G****U**AGCCGCGUGCGCGCAACGCGACG........CACGGGCA...................................G**G****C****U****G****C**..**C****C****A****C****C**GAA**G****G****G****G**CGC..**G****C****G**..**C**.**G****A****G****C**CGGAUCG...AAAC..........................................................................................**C****G****U****C**..**G**.**C****G****C**.-.AAU.**C****U****C**.**U**CA**G****G****U**.............AUCGA**G**.**G****A****C****A****G****A****G****G**GGCAUGC | |
|  |  | NZ\_AAHM01000270.1/307-585  | CCGCA...**C****G**.**C**.**G**.**U**.**C****G****G****G****A****G**A**G**.C**G****C****G****C****G**GCCGCCGUUCGUUGAUCGAAAGGCAAG.............................C**C****G****C****G****C**..**C****G**.**C****C**GAA**G****G****G****G**CA...-..**C****A**................CCC-........................-**G**CAAA**C****U****C****U**CA**G****G****C**..........................................................................A.A.A.A.**G**.**G****A**.**C****C****G**..**A**.C**C****G**.**C**.**G**U....CGAAGAAUCCGUCUGACGACAUGCGUCGGACGGCGUUUUUCGCA........................-**C****U****C****U****G****G****A****G**A**G**C.**G**.**G****C****A****G****U**AGCCGCGUGCGCGCAACGCGACG........CACGGGCA...................................G**G****C****U****G****C**..**C****C****A****C****C**GAA**G****G****G****G**CGC..**G****C****G**..**C**.**G****A****G****C**CGGAUCG...AAAC..........................................................................................**C****G****U****C**..**G**.**C****G****C**.-.AAU.**C****U****C**.**U**CA**G****G****U**.............AUCGA**G**.**G****A****C****A****G****A****G****G**GGCAUGC | |
|  |  | NZ\_AAHN01000006.1/48673-48951  | CCGCA...**C****G**.**C**.**G**.**U**.**C****G****G****G****A****G**A**G**.C**G****C****G****C****G**GCCGCCGUUCGUUGAUCGAAAGGCAAG.............................C**C****G****C****G****C**..**C****G**.**C****C**GAA**G****G****G****G**CA...-..**C****A**................CCC-........................-**G**CAAA**C****U****C****U**CA**G****G****C**..........................................................................A.A.A.A.**G**.**G****A**.**C****C****G**..**A**.C**C****G**.**C**.**G**U....CGAAGAAUCCGUCUGACGACAUGCGUCGGACGGCGUUUUUCGCA........................-**C****U****C****U****G****G****A****G**A**G**C.**G**.**G****C****A****G****U**AGCCGCGUGCGCGCAACGCGACG........CACGGGCA...................................G**G****C****U****G****C**..**C****C****A****C****C**GAA**G****G****G****G**CGC..**G****C****G**..**C**.**G****A****G****C**CGGAUCG...AAAC..........................................................................................**C****G****U****C**..**G**.**C****G****C**.-.AAU.**C****U****C**.**U**CA**G****G****U**.............AUCGA**G**.**G****A****C****A****G****A****G****G**GGCAUGC | |
|  |  | NZ\_AAHO01000007.1/114046-113768  | CCGCA...**C****G**.**C**.**G**.**U**.**C****G****G****G****A****G**A**G**.C**G****C****G****C****G**GCCGCCGUUCGUUGAUCGAAAGGCAAG.............................C**C****G****C****G****C**..**C****G**.**C****C**GAA**G****G****G****G**CA...-..**C****A**................CCC-........................-**G**CAAA**C****U****C****U**CA**G****G****C**..........................................................................A.A.A.A.**G**.**G****A**.**C****C****G**..**A**.C**C****G**.**C**.**G**U....CGAAGAAUCCGUCUGACGACAUGCGUCGGACGGCGUUUUUCGCA........................-**C****U****C****U****G****G****A****G**A**G**C.**G**.**G****C****A****G****U**AGCCGCGUGCGCGCAACGCGACG........CACGGGCA...................................G**G****C****U****G****C**..**C****C****A****C****C**GAA**G****G****G****G**CGC..**G****C****G**..**C**.**G****A****G****C**CGGAUCG...AAAC..........................................................................................**C****G****U****C**..**G**.**C****G****C**.-.AAU.**C****U****C**.**U**CA**G****G****U**.............AUCGA**G**.**G****A****C****A****G****A****G****G**GGCAUGC | |
|  |  | NZ\_AAHP01000033.1/48619-48897  | CCGCA...**C****G**.**C**.**G**.**U**.**C****G****G****G****A****G**A**G**.C**G****C****G****C****G**GCCGCCGUUCGUUGAUCGAAAGGCAAG.............................C**C****G****C****G****C**..**C****G**.**C****C**GAA**G****G****G****G**CA...-..**C****A**................CCC-........................-**G**CAAA**C****U****C****U**CA**G****G****C**..........................................................................A.A.A.A.**G**.**G****A**.**C****C****G**..**A**.C**C****G**.**C**.**G**U....CGAAGAAUCCGUCUGACGACAUGCGUCGGACGGCGUUUUUCGCA........................-**C****U****C****U****G****G****A****G**A**G**C.**G**.**G****C****A****G****U**AGCCGCGUGCGCGCAACGCGACG........CACGGGCA...................................G**G****C****U****G****C**..**C****C****A****C****C**GAA**G****G****G****G**CGC..**G****C****G**..**C**.**G****A****G****C**CGGAUCG...AAAC..........................................................................................**C****G****U****C**..**G**.**C****G****C**.-.AAU.**C****U****C**.**U**CA**G****G****U**.............AUCGA**G**.**G****A****C****A****G****A****G****G**GGCAUGC | |
|  |  | NZ\_AAHP01000156.1/562-284  | CCGCA...**C****G**.**C**.**G**.**U**.**C****G****G****G****A****G**A**G**.C**G****C****G****C****G**GCCGCCGUUCGUUGAUCGAAAGGCAAG.............................C**C****G****C****G****C**..**C****G**.**C****C**GAA**G****G****G****G**CA...-..**C****A**................CCC-........................-**G**CAAA**C****U****C****U**CA**G****G****C**..........................................................................A.A.A.A.**G**.**G****A**.**C****C****G**..**A**.C**C****G**.**C**.**G**U....CGAAGAAUCCGUCUGACGACAUGCGUCGGACGGCGUUUUUCGCA........................-**C****U****C****U****G****G****A****G**A**G**C.**G**.**G****C****A****G****U**AGCCGCGUGCGCGCAACGCGACG........CACGGGCA...................................G**G****C****U****G****C**..**C****C****A****C****C**GAA**G****G****G****G**CGC..**G****C****G**..**C**.**G****A****G****C**CGGAUCG...AAAC..........................................................................................**C****G****U****C**..**G**.**C****G****C**.-.AAU.**C****U****C**.**U**CA**G****G****U**.............AUCGA**G**.**G****A****C****A****G****A****G****G**GGCAUGC | |
|  |  | NZ\_AAHQ01000008.1/227607-227329  | CCGCA...**C****G**.**C**.**G**.**U**.**C****G****G****G****A****G**A**G**.C**G****C****G****C****G**GCCGCCGUUCGUUGAUCGAAAGGCAAG.............................C**C****G****C****G****C**..**C****G**.**C****C**GAA**G****G****G****G**CA...-..**C****A**................CCC-........................-**G**CAAA**C****U****C****U**CA**G****G****C**..........................................................................A.A.A.A.**G**.**G****A**.**C****C****G**..**A**.C**C****G**.**C**.**G**U....CGAAGAAUCCGUCUGACGACAUGCGUCGGACGGCGUUUUUCGCA........................-**C****U****C****U****G****G****A****G**A**G**C.**G**.**G****C****A****G****U**AGCCGCGUGCGCGCAACGCGACG........CACGGGCA...................................G**G****C****U****G****C**..**C****C****A****C****C**GAA**G****G****G****G**CGC..**G****C****G**..**C**.**G****A****G****C**CGGAUCG...AAAC..........................................................................................**C****G****U****C**..**G**.**C****G****C**.-.AAU.**C****U****C**.**U**CA**G****G****U**.............AUCGA**G**.**G****A****C****A****G****A****G****G**GGCAUGC | |
|  |  | NZ\_AAHR01000003.1/87112-87390  | CCGCA...**C****G**.**C**.**G**.**U**.**C****G****G****G****A****G**A**G**.C**G****C****G****C****G**GCCGCCGUUCGUUGAUCGAAAGGCAAG.............................C**C****G****C****G****C**..**C****G**.**C****C**GAA**G****G****G****G**CA...-..**C****A**................CCC-........................-**G**CAAA**C****U****C****U**CA**G****G****C**..........................................................................A.A.A.A.**G**.**G****A**.**C****C****G**..**A**.C**C****G**.**C**.**G**U....CGAAGAAUCCGUCUGACGACAUGCGUCGGACGGCGUUUUUCGCA........................-**C****U****C****U****G****G****A****G**A**G**C.**G**.**G****C****A****G****U**AGCCGCGUGCGCGCAACGCGACG........CACGGGCA...................................G**G****C****U****G****C**..**C****C****A****C****C**GAA**G****G****G****G**CGC..**G****C****G**..**C**.**G****A****G****C**CGGAUCG...AAAC..........................................................................................**C****G****U****C**..**G**.**C****G****C**.-.AAU.**C****U****C**.**U**CA**G****G****U**.............AUCGA**G**.**G****A****C****A****G****A****G****G**GGCAUGC | |
|  |  | NZ\_AAHS01000024.1/55906-56184  | CCGCA...**C****G**.**C**.**G**.**U**.**C****G****G****G****A****G**A**G**.C**G****C****G****C****G**GCCGCCGUUCGUUGAUCGAAAGGCAAG.............................C**C****G****C****G****C**..**C****G**.**C****C**GAA**G****G****G****G**CA...-..**C****A**................CCC-........................-**G**CAAA**C****U****C****U**CA**G****G****C**..........................................................................A.A.A.A.**G**.**G****A**.**C****C****G**..**A**.C**C****G**.**C**.**G**U....CGAAGAAUCCGUCUGACGACAUGCGUCGGACGGCGUUUUUCGCA........................-**C****U****C****U****G****G****A****G**A**G**C.**G**.**G****C****A****G****U**AGCCGCGUGCGCGCAACGCGACG........CACGGGCA...................................G**G****C****U****G****C**..**C****C****A****C****C**GAA**G****G****G****G**CGC..**G****C****G**..**C**.**G****A****G****C**CGGAUCG...AAAC..........................................................................................**C****G****U****C**..**G**.**C****G****C**.-.AAU.**C****U****C**.**U**CA**G****G****U**.............AUCGA**G**.**G****A****C****A****G****A****G****G**GGCAUGC | |
|  |  | NZ\_AAHT01000001.1/132430-132708  | CCGCA...**C****G**.**C**.**G**.**U**.**C****G****G****G****A****G**A**G**.C**G****C****G****C****G**GCCGCCGUUCGUUGAUCGAAAGGCAAG.............................C**C****G****C****G****C**..**C****G**.**C****C**GAA**G****G****G****G**CA...-..**C****A**................CCC-........................-**G**CAAA**C****U****C****U**CA**G****G****C**..........................................................................A.A.A.A.**G**.**G****A**.**C****C****G**..**A**.C**C****G**.**C**.**G**U....CGAAGAAUCCGUCUGACGACAUGCGUCGGACGGCGUUUUUCGCA........................-**C****U****C****U****G****G****A****G**A**G**C.**G**.**G****C****A****G****U**AGCCGCGUGCGCGCAACGCGACG........CACGGGCA...................................G**G****C****U****G****C**..**C****C****A****C****C**GAA**G****G****G****G**CGC..**G****C****G**..**C**.**G****A****G****C**CGGAUCG...AAAC..........................................................................................**C****G****U****C**..**G**.**C****G****C**.-.AAU.**C****U****C**.**U**CA**G****G****U**.............AUCGA**G**.**G****A****C****A****G****A****G****G**GGCAUGC | |
|  |  | NZ\_AAHU01000007.1/110804-111082  | CCGCA...**C****G**.**C**.**G**.**U**.**C****G****G****G****A****G**A**G**.C**G****C****G****C****G**GCCGCCGUUCGUUGAUCGAAAGGCAAG.............................C**C****G****C****G****C**..**C****G**.**C****C**GAA**G****G****G****G**CA...-..**C****A**................CCC-........................-**G**CAAA**C****U****C****U**CA**G****G****C**..........................................................................A.A.A.A.**G**.**G****A**.**C****C****G**..**A**.C**C****G**.**C**.**G**U....CGAAGAAUCCGUCUGACGACAUGCGUCGGACGGCGUUUUUCGCA........................-**C****U****C****U****G****G****A****G**A**G**C.**G**.**G****C****A****G****U**AGCCGCGUGCGCGCAACGCGACG........CACGGGCA...................................G**G****C****U****G****C**..**C****C****A****C****C**GAA**G****G****G****G**CGC..**G****C****G**..**C**.**G****A****G****C**CGGAUCG...AAAC..........................................................................................**C****G****U****C**..**G**.**C****G****C**.-.AAU.**C****U****C**.**U**CA**G****G****U**.............AUCGA**G**.**G****A****C****A****G****A****G****G**GGCAUGC | |
|  |  | NZ\_AAHV01000016.1/97514-97792  | CCGCA...**C****G**.**C**.**G**.**U**.**C****G****G****G****A****G**A**G**.C**G****C****G****C****G**GCCGCCGUUCGUUGAUCGAAAGGCAAG.............................C**C****G****C****G****C**..**C****G**.**C****C**GAA**G****G****G****G**CA...-..**C****A**................CCC-........................-**G**CAAA**C****U****C****U**CA**G****G****C**..........................................................................A.A.A.A.**G**.**G****A**.**C****C****G**..**A**.C**C****G**.**C**.**G**U....CGAAGAAUCCGUCUGACGACAUGCGUCGGACGGCGUUUUUCGCA........................-**C****U****C****U****G****G****A****G**A**G**C.**G**.**G****C****A****G****U**AGCCGCGUGCGCGCAACGCGACG........CACGGGCA...................................G**G****C****U****G****C**..**C****C****A****C****C**GAA**G****G****G****G**CGC..**G****C****G**..**C**.**G****A****G****C**CGGAUCG...AAAC..........................................................................................**C****G****U****C**..**G**.**C****G****C**.-.AAU.**C****U****C**.**U**CA**G****G****U**.............AUCGA**G**.**G****A****C****A****G****A****G****G**GGCAUGC | |
|  |  | NZ\_AAHW01000022.1/53137-52859  | CCGCA...**C****G**.**C**.**G**.**U**.**C****G****G****G****A****G**A**G**.C**G****C****G****C****G**GCCGCCGUUCGUUGAUCGAAAGGCAAG.............................C**C****G****C****G****C**..**C****G**.**C****C**GAA**G****G****G****G**CA...-..**C****A**................CCC-........................-**G**CAAA**C****U****C****U**CA**G****G****C**..........................................................................A.A.A.A.**G**.**G****A**.**C****C****G**..**A**.C**C****G**.**C**.**G**U....CGAAGAAUCCGUCUGACGACAUGCGUCGGACGGCGUUUUUCGCA........................-**C****U****C****U****G****G****A****G**A**G**C.**G**.**G****C****A****G****U**AGCCGCGUGCGCGCAACGCGACG........CACGGGCA...................................G**G****C****U****G****C**..**C****C****A****C****C**GAA**G****G****G****G**CGC..**G****C****G**..**C**.**G****A****G****C**CGGAUCG...AAAC..........................................................................................**C****G****U****C**..**G**.**C****G****C**.-.AAU.**C****U****C**.**U**CA**G****G****U**.............AUCGA**G**.**G****A****C****A****G****A****G****G**GGCAUGC | |
|  |  | NZ\_AAIR01000024.1/368-646  | CCGCA...**C****G**.**C**.**G**.**U**.**C****G****G****G****A****G**A**G**.C**G****C****G****C****G**GCCGCCGUUCGUUGAUCGAAAGGCAAG.............................C**C****G****C****G****C**..**C****G**.**C****C**GAA**G****G****G****G**CA...-..**C****A**................CCC-........................-**G**CAAA**C****U****C****U**CA**G****G****C**..........................................................................A.A.A.A.**G**.**G****A**.**C****C****G**..**A**.C**C****G**.**C**.**G**U....CGAAGAAUCCGUCUGACGACAUGCGUCGGACGGCGUUUUUCGCA........................-**C****U****C****U****G****G****A****G**A**G**C.**G**.**G****C****A****G****U**AGCCGCGUGCGCGCAACGCGACG........CACGGGCA...................................G**G****C****U****G****C**..**C****C****A****C****C**GAA**G****G****G****G**CGC..**G****C****G**..**C**.**G****A****G****C**CGGAUCG...AAAC..........................................................................................**C****G****U****C**..**G**.**C****G****C**.-.AAU.**C****U****C**.**U**CA**G****G****U**.............AUCGA**G**.**G****A****C****A****G****A****G****G**GGCAUGC | |
|  |  | NZ\_AAIQ01000042.1/3373-3095  | UCCGC...**C****G**.**C**.**G**.**U**.**C****G****G****G****A****G**A**G**.C**G****C****G****C****G**GCCGCCGUUCGUUGAUCGAAAGGCAAG.............................C**C****G****C****G****C**..**C****G**.**C****C**GAA**G****G****G****G**CA...-..**C****A**................CCC-........................-**G**CAAA**C****U****C****U**CA**G****G****C**..........................................................................A.A.A.A.**G**.**G****A**.**C****C****G**..**A**.C**C****G**.**C**.**G**U....CGAAGAAUCCGUCUGACGACAUGCGUCGGACGGCGUUUUUCGCA........................-**C****U****C****U****G****G****A****G**A**G**C.**G**.**G****C****A****G****U**AGCCGCGUGCGCGCAACGCGACG........CACGGGCA...................................G**G****C****U****G****C**..**C****C****A****C****C**GAA**G****G****G****G**CGC..**G****C****G**..**C**.**G****A****G****C**CGGAUCG...AAAC..........................................................................................**C****G****U****C**..**G**.**C****G****C**.-.AAU.**C****U****C**.**U**CA**G****G****U**.............AUCGA**G**.**G****A****C****A****G****A****G****G**GGCAUGC | |
|  |  | NC\_004547.2/2645260-2645034  | CCGCU...**C****C**.**U**.**C**.**G**.**C****A****G****G****A****G**A**G**.A**G****G****G****C****G**UG.........UACUCCAGCAUACUUCAAGCUACAUGUACGUUGGCAUAAAGUAUAA**C****G****C****U****C**..**C****G**.**C****C**GAA**G****G****C****G**CA...A..**A****C**................UCCC........................**A****U**.AAU**C****G****C****U**CA**G****G****C**..........................................................................U.A.C.C.**C**.**U****A**A**C****U****G**..**C**..**G****A**.**A**.**U**UCCAUUGAAGCCA............................................................--**A****C****U****G****G****A****G**A**G**A.**G**.**G****U****U****G****C**...............................-GAU.......................................-**G****C****A****A****C**..**C****C****A****C****C**GAA**G****G****G****G**CAA..**G****C****A**..**G**.**C**---..........UCU-..........................................................................................---**G**..**C**.**U****G****C**GU.AAA.**C****U****C**.**U**CA**G****G****U**.............AAAGC**G**.**G****A****C****A****G****A****G****G**GAGUGGC | |
|  |  | NZ\_AAEH02000035.1/33498-33230  | CCGCA...**C****G**.**C**.**G**.**U**.**C****G****G****G****A****G**A**G**.C**G****C****G****U****G**ACCG.......CAUAGUCGAUACGCCGG............................U**C****G****C****G****C**..**C****G**.**C****C**GAA**G****G****G****G**CA...-..**C****A**................CCC-........................-**G**CAAA**C****U****C****U**CA**G****G****C**..........................................................................A.A.A.A.**G**.**G****A**.**C****C****G**..**A**.C**C****G**.**C**.**G**.....UCGGGGAAUCCCGUUCCGCGCAUUGCGCGGGCCGCCUUCCCCGCA.......................-**C****U****C****U****G****G****A****G**A**G**C.**G**.**G****C****A****G****U**AGCCCGCUGCGCACAUGCUGCGCGG......GCAG.......................................-**G****C****U****G****C**..**C****C****A****C****C**GAA**G****G****G****G**CG-..**C****G****C**..**G**.**U****U****U****G**CCGGC.....UUGG.........................................................................................U**C****C****G****C**..**A**.**G****C****G**.C.AAU.**C****U****C**.**U**CA**G****G****U**.............AUCGA**G**.**G****A****C****A****G****A****G****G**GGCAUGU | |
|  |  | NZ\_AAEI01000018.1/146053-145783  | CCGCA...**C****G**.**C**.**G**.**U**.**C****G****G****G****A****G**A**G**.C**G****U****G****U****A**ACCAGGCUGU.UUCAGCACCGG..................................U**U****G****C****G****C**..**C****G**.**C****C**GAA**G****G****G****G**CA...-..**C****A**................CCC-........................-**G**CAAA**C****U****C****U**CA**G****G****C**..........................................................................A.A.A.A.**G**.**G****A**.**C****C****G**..**A**.C**C****G**.**C**.**G**.....UCGGGGAAUCCCGUCCGCGCAUUGCGCGGGCCACACUCUCCCCGCA......................-**C****U****C****U****G****G****A****G**A**G**C.**G**.**G****C****A****G****U**AGCCCGCAGCGCAUCCGCUGCGCGG......GCAA.......................................-**G****C****U****G****C**..**C****C****A****C****C**GAA**G****G****G****G**CG-..**C****G****C**..**G**.**U****U****C****G**CC........AGGC.....................................................................................UCGGC**C****G****G****C**..**A**.**G****C****G**.C.AAU.**C****U****C**.**U**CA**G****G****U**.............AUCGA**G**.**G****A****C****A****G****A****G****G**GGCAUGU | |
|  |  | NZ\_AAAJ03000020.1/95680-95394  | CCGUA...**C****G**.**C**.**G**.**U**.**C****G****G****G****A****G**A**G**.C**G****U****G****C****U**GGCCGCAG...AAAACCGUAGG..................................C**C****G****C****G****C**..**C****G**.**C****C**GAA**G****G****G****G**CA...-..**C****A**................CCC-........................-**G**CAAA**C****U****C****U**CA**G****G****C**..........................................................................A.A.A.A.**G**.**G****A**.**C****C****G**..**A**..**C**-.**C**.**G**.....CGUCGAAAAACCCGCGUUGAGGCAACAUCAAGGCACCGGUUUUUCGCA....................-**C****U****C****U****G****G****A****G**A**G**C.**G**.**G****C****A****G****U**AGCCGUCUGCAUCGU................UCAAUCGAGCAGAUUGGCA........................G**G****C****U****G****C**..**C****C****A****C****C**GAA**G****G****G****G**CGC..**G****C****G**..**U**.**U****U****C****A**CCG.......UGGC..........................................................................GGACUCACGUUCUCAC**G****G****C****A**..**A**.**C****G****C**.-.AAU.**C****U****C**.**U**CA**G****G****U**.............AUCGA**G**.**G****A****C****A****G****A****G****G**GGCCAUG | |
|  |  | NZ\_AAHI01000010.1/146395-146123  | CCGCA...**C****G**.**C**.**G**.**U**.**C****G****G****G****A****G**A**G**.C**G****U****G****U****G**ACCGGGUCG..UUCCGACACCGG.................................U**U****G****C****G****C**..**C****G**.**C****C**GAA**G****G****G****G**CA...-..**C****A**................CCC-........................-**G**CAAA**C****U****C****U**CA**G****G****C**..........................................................................A.A.A.A.**G**.**G****A**.**C****C****G**..**A**.C**C****G**.**C**.**G**.....UCGGGGAAUCGCGUCCGCGCAUUGCGCGGGCACCUUACUCUCCCCGCA....................-**C****U****C****U****G****G****A****G**A**G**C.**G**.**G****C****A****G****U**AGCCCGCAGCGCAUUGGCUGCGCGG......GCAA.......................................-**G****C****U****G****C**..**C****C****A****C****C**GAA**G****G****G****G**CGC..**G****C****G**..**U**.**U****C****G****C**CGGGC.....UUUG........................................................................................CC**C****G****C****A**..**G**.**C****G****C**.-.AAU.**C****U****C**.**U**CA**G****G****U**.............AUCGA**G**.**G****A****C****A****G****A****G****G**GGCAUGU | |
|  |  | NZ\_AAHL01000003.1/133931-134203  | CCGCA...**C****G**.**C**.**G**.**U**.**C****G****G****G****A****G**A**G**.C**G****U****G****U****G**ACCGGGUCG..UUCCGACACCGG.................................U**U****G****C****G****C**..**C****G**.**C****C**GAA**G****G****G****G**CA...-..**C****A**................CCC-........................-**G**CAAA**C****U****C****U**CA**G****G****C**..........................................................................A.A.A.A.**G**.**G****A**.**C****C****G**..**A**.C**C****G**.**C**.**G**.....UCGGGGAAUCGCGUCCGCGCAUUGCGCGGGCACCUUACUCUCCCCGCA....................-**C****U****C****U****G****G****A****G**A**G**C.**G**.**G****C****A****G****U**AGCCCGCAGCGCAUUGGCUGCGCGG......GCAA.......................................-**G****C****U****G****C**..**C****C****A****C****C**GAA**G****G****G****G**CGC..**G****C****G**..**U**.**U****C****G****C**CGGGC.....UUUG........................................................................................CC**C****G****C****A**..**G**.**C****G****C**.-.AAU.**C****U****C**.**U**CA**G****G****U**.............AUCGA**G**.**G****A****C****A****G****A****G****G**GGCAUGU | |
|  |  | NZ\_AADI01000003.1/76990-77313  | AAGCG...**U****U**.**U**.**U**.**U**.**C****A****G****G****A****G**A**G**.A**G****U****C****U****U**UAGCC......UAAAGUUAGUGAUACUCAUAUUAAUGAGCUGACAGCCGCUA....U**A****U****G****A****C**..**C****A**.**C****C**GAA**G****G****C****G**CA...U..**C****C**A...............CCC-.......................U**G****C**UAAU**C****G****C****U**CA**G****G****U**..........................................................................A.A.A.A.**G**.**G****A**.**C****U****G**..**A**..**A****A**.**A**.**A**C....ACAUGUCACCAUGGUUUAUUUGUAUCGCGCUGCUAAGCGUAUUGCGCUCUAAACCUUAGGCAUAACAA-**A****U****C****U****G****G****A****G**A**G**C.**G**.**G****U****A****A****G**...............................GACGGACGACG................................C**C****U****U****A****U**..**C****C****A****C****C**GAA**G****G****G****G**AGAA.**A****A****C**..**G**.**C****G****A****U**A.........UUAU..................................................................AGGCUUGUUAAAAAAGACAAUAAU**A****U****C****G**..**U**.**G****U****U**.G.AAA.**A****U****C**.**U**CA**G****G****U**..............UACA**G**.**G****A****C****A****G****A****U****A**GGGCUUU | |
|  |  | AAFX01016906.1/855-672  | UGGAC...**C****C**.**U**.**C**.**G**.**C****G****G****G****A****G**A**G**.A**C****C****G****G****G**UGCG.......AUU-.........................................-**C****C****C****G****G**..**C****G**.**C****C**GAA**G****G****C****G**CA...A..**C****C**G...............CCCC........................**G****G**.AAA**C****G****C****U**CA**G****G****C**..........................................................................A.A.A.C.**G**.**G****A**.**C****C****G**..**C**..**G****A**.**A**.**G**GC...UGAUGAA-............................................................-**C****G****C****U****G****G****A****A**A**G**U.**G**.**A****U****C****C**-...............................GAAA.......................................--**G****G****G****U**..**C****C****G****C****C**GAA**G****G****A****G**CAA..**G****G****A**..**A**.**G****G**--..........GACC..........................................................................................--**C****C**..**U**.**U****C****C**.G.AAAU**C****U****C**.**U**CA**G****G****U**..............UCCA**G**.**G****A****C****A****G****C****G****G**GGGCGCC | |
|  |  | AAFZ01024364.1/420-227  | ACGAA...**C****C**.**C**.**U**.**G**.**C****G****G****G****A****G**A**G**.-**A****C****G****G****A**U..........UCCGCAACG....................................G**A****C****C****G****A**..**C****G**.**C****C**GAA**G****G****A****G**CA...A..**C****C**G...............CCCC........................**G****G**.AAA**C****U****C****U**CA**G****G****C**..........................................................................A.A.A.U.**G**.**G****A**.**C****C****G**..**C**..**A****G**.**G**.**G**.....GGAUGUUA............................................................-**C****U****C****U****G****G****A****A**A**G**C.**A**.**G****G****C****G****G**...............................CACGUU.....................................G**C****C****G****U****C**..**U****C****A****C****C**GAA**G****G****C****G**AAA..**A****C****C**..**C**.**G****U****A****U**..........CACG..........................................................................................**G****C****G****C**..**G**.**G****G****C**.G.AAU.**C****U****C**.**U**CA**G****G****U**..............UACC**G**.**G****A****C****A****G****A****G****G**GGGCACG | |
|  |  | AAFX01086649.1/566-373  | ACGAU...**C****C**.**C**.**U**.**G**.**U****G****G****G****A****G**A**G**.A**C****C****G****C****C**UCG........UUCAAG.......................................G**G****G****C****G****G**..**C****G**.**C****C**GAA**G****G****A****G**CA...A..**C****C**G...............GCCC.......................C**G****G**.AAA**C****U****C****U**CA**G****G****C**..........................................................................A.A.A.C.**G**.**G****A**.**C****C****G**..**C**..**A****G**.**G**.**A**.....GGAUGGGU............................................................-**C****U****C****U****G****G****A****A**A**G**U.**G**.**A****C****G****G****G**CACC...........................CGAACGGGA..................................G**C****G****C****G****U**..**C****C****A****C****C**GAC**G****A****A****G**UAA..**G****C****C**..**G**.----..........GCAA..........................................................................................----..**C**.**G****G****C**.G.AAU.**C****U****U**.**U**CA**G****G****U**..............CUCC**G**.**G****A****C****A****G****A****G****G**GGGUCGC | |
|  |  | AACY01040028.1/764-959  | AUGAA...**G****G**.**U**.**A**.**G**.**C****A****G****G****A****G**A**G**.C**G****A****U****G****G**G..........CAA-.........................................-**C****C****A****U****C**..**C****A**.**C****C**GAA**G****A****A****G**UG...A..--................----........................--.-AU**C****U****U****U**CA**G****G****U**............................................................ACCGGGCAACCGGU-.-.G.U.**G**.**G****A**.**C****U****G**..**C**..**U****A**.**C**.**U**.....GGACGAUA............................................................-**C****U****C****U****G****G****A****G**A**G**A.**C**.**C****C****G****U**-...............................UGAA.......................................--**U****C****G****G**..**G****C****G****C****C**GAA**G****G****A****G**CAA..**G****G****C**..**A**.**C****C****G****U**G.........AUUG.................................................................................CAUCGCAAU**C****U****G****G**..**C**.**G****C****C**GG.AAA.**C****U****C**.**U**CA**G****G****C**..............AAAA**G**.**G****A****C****A****G****A****G****G**GGAUGGG | |
|  |  | AACY01063243.1/3316-3109  | AUGCA...--.**G**.**U**.**G**.**C****G****G****G****A****G**A**G**.A**U****C****G****U****C**AGAGGC.....UUGU.........................................-**G****A****C****G****A**..**C****G**.**C****C**GAC**G****G****A****G**CA...A..**C****C**G...............CCCC........................**G****G**.AAA**C****U****C****U**CA**G****G****C**..........................................................................A.A.A.A.**G**.**G****A**.**C****C****G**..**C**..**G****C**.**A**.-.....GCAGGAA-............................................................**C****A****U****C****U****G****G****A****G**A**G**C.**G**.**G****C****G****C**-...............................CGUG.......................................C-**G****C****G****C**..**C****C****A****C****C**GAA**G****G****G****G**--A..**U****U****C**..**C**.**C****G****G****C**AUGGCGCC..UAGA...........................................................................UCGCGCUCCUUUGCU**C****G****C****G**..**G**.**G****A****A**.G.AAA.**C****U****C**.**U**CA**G****G****U**..............ACAU**G**.**G****A****C****A****G****A****U****G**GGGCAUC | |
|  |  | AACY01457116.1/72-267  | AUGAU...**C****C**.**U**.**U**.**A**.**A****A****G****A****A****G**A**G**.A**G****C****G****A****C**G..........AGCAUCCGUU...................................U**A****U****C****G****C**..**C****G**.**C****C**GAA**G****G****A****G**CA...A..**C****C**G...............CCCC........................**G****G**.AAA**C****U****C****U**CA**G****G****C**..........................................................................A.A.A.A.**G**.**G****A**.**C****U****U**..**U**..**A****A**.**G**.**G**.....UGAGGAG-............................................................**C****C****U****C****U****G****G****A****A**A**G**C.**A**.**G****G****G****A**-...............................UAAGAU.....................................U-**U****C****C****C**..**U****C****A****C****C**GAA**G****G****U****G**UAA..**G****C****C**..**A**.**G****U****C****U**UG........AAGU.........................................................................................C**A****G****G****C**..**U**.**G****G****U**.C.AAU.**C****U****C**.**U**CA**G****G****U**..............-UUU**C**.**G****A****C****A****G****A****G****G**GGGCUUU | |
|  |  | AACY01111469.1/1238-1417  | UUAAU...**U****U**.**U**.**A**.**G**.**U****G****G****G****A****G**A**G**.U**C****U****A****A****G**UA.........UUUA.........................................-**C****U****U****A****G**..**C****G**.**C****C**GAA**G****G****A****G**UA...A..**C****C**G...............CCUC........................**G****G**.AAC**C****U****C****U**CA**G****G****C**..........................................................................A.A.A.C.**G**.**G****A**.**C****C****G**..**C**..**U****A**.**A**.**A**.....AUUUGACA............................................................-**C****U****C****U****G****G****A****A**A**G**U.**G**.**G****C**---...............................AAUA.......................................----**G****C**..**C****C****A****C****C**CAA**G****G****U****G**AAA..**G****U****U**..**A**.**G****A****U****A**..........UUU-..........................................................................................**U****A****U****C**..**U**.**A****A****U**.U.AAU.**C****U****C**.**U**CA**G****G****U**..............ACCA**A**.**G****A****C****A****G****A****G****G**GGGCACA | |
|  |  | AACY01687253.1/189-368  | UUAAU...**U****U**.**U**.**A**.**G**.**U****G****G****G****A****G**A**G**.U**C****U****A****A****G**UA.........UUUA.........................................-**C****U****U****A****G**..**C****G**.**C****C**GAA**G****G****A****G**UA...A..**C****C**G...............CCUC........................**G****G**.AAC**C****U****C****U**CA**G****G****C**..........................................................................A.A.A.C.**G**.**G****A**.**C****C****G**..**C**..**U****A**.**A**.**A**.....AUUUGACA............................................................-**C****U****C****U****G****G****A****A**A**G**U.**G**.**G****C**---...............................AAUA.......................................----**G****C**..**C****C****A****C****C**CAA**G****G****U****G**AAA..**G****U****U**..**A**.**G****A****U****A**..........UUU-..........................................................................................**U****A****U****C**..**U**.**A****A****U**.U.AAU.**C****U****C**.**U**CA**G****G****U**..............ACCA**A**.**G****A****C****A****G****A****G****G**GGGCACA | |
|  |  | AACY01713268.1/419-598  | UUAAU...**U****U**.**U**.**A**.**G**.**U****G****G****G****A****G**A**G**.U**C****U****A****A****G**UA.........UUUA.........................................-**C****U****U****A****G**..**C****G**.**C****C**GAA**G****G****A****G**UA...A..**C****C**G...............CCUC........................**G****G**.AAC**C****U****C****U**CA**G****G****C**..........................................................................A.A.A.C.**G**.**G****A**.**C****C****G**..**C**..**U****A**.**A**.**A**.....AUUUGACA............................................................-**C****U****C****U****G****G****A****A**A**G**U.**G**.**G****C**---...............................AAUA.......................................----**G****C**..**C****C****A****C****C**CAA**G****G****U****G**AAA..**G****U****U**..**A**.**G****A****U****A**..........UUU-..........................................................................................**U****A****U****C**..**U**.**A****A****U**.U.AAU.**C****U****C**.**U**CA**G****G****U**..............ACCA**A**.**G****A****C****A****G****A****G****G**GGGCACA | |
|  |  | AACY01282805.1/339-160  | UUAAU...**U****U**.**U**.**A**.**G**.**U****G****G****G****A****G**A**G**.C**C****U****A****A****G**UA.........UUUA.........................................-**C****U****U****A****G**..**C****G**.**C****C**GAA**G****G****A****G**UA...A..**C****C**G...............CCUC........................**G****G**.AAC**C****U****C****U**CA**G****G****C**..........................................................................A.A.A.C.**G**.**G****A**.**C****C****G**..**C**..**U****A**.**A**.**A**.....AUUUGACA............................................................-**C****U****C****U****G****G****A****A**A**G**U.**G**.**G****C**---...............................AAUA.......................................----**G****C**..**C****C****A****C****C**CAA**G****G****U****G**AAA..**G****U****U**..**A**.**G****A****U****A**..........UUU-..........................................................................................**U****A****U****C**..**U**.**A****A****U**.U.AAU.**C****U****C**.**U**CA**G****G****U**..............ACCA**A**.**G****A****C****A****G****A****G****G**GGGCACA | |
|  |  | AACY01378226.1/46-219  | AGUCU...**U****U**.**U**.**U**.**A**.**U****G****U****G****A****G**A**G**.U**G****U****U****U**-...........UUU-.........................................--**A****A****A****C**..**C****G**.**C****C**GAA**G****G****A****G**CA...A..**C****C**G...............CCCC........................**G****G**.AAA**C****U****C****U**CA**G****G****C**..........................................................................A.A.A.U.**G**.**G****A**.**A****C****A**..**U**..**A****A**.**A**.**A**.....AGUUGAUA............................................................-**A****U****C****U****G****G****A****G**A**G**U.**G**.**U****C****G****U****U**...............................UUCU.......................................-**A****A****C****G****A**..**C****C****A****C****C**GAA**G****G****G****G**UAA..**G****C****C**..**A**.----..........CAAU..........................................................................................----..**U**.**G****G****U**.G.AAU.**C****U****U**.**U**CA**G****G****U**..............UCCG**U**.**G****A****C****A****G****A****U****G**GGGUAAA | |
|  |  | AAFX01042973.1/245-47  | AAACG...**C****C**.**A**.**A**.**G**.**C****G****G****G****A****G**A**G**.A**U****C****G****G****G**GG.........AAGC.........................................-**C****C****C****G****G**..**C****G**.**C****C**GAA**G****G****A****G**CA...A..**C****C**G...............CCCC........................**G****G**.AAA**C****U****C****U**CA**G****G****C**..........................................................................A.A.A.A.**G**.**G****A**.**C****C****G**..**C**..**C****G**.**C**.**G**.....UUUUGGAA............................................................-**C****U****C****U****G****G****A****G**A**G**C.**A**.**G****G****G****G****U**C..............................UUAC.......................................-**A****C****C****C****C**..**U****C****A****C****C**GUA**G****G****A****G**CAA..**G****U****C**..**U**.**G****G****C****C**G.........UGUC.................................................................................GGAGUGACG**G****U****U****C**..**G**.**G****G****C**.GAAAA.**C****U****C**.**U**CA**G****G****U**..............UCUC**U**.**G****A****C****A****G****A****G****G**GGGCAGG | |
|  |  | AAFZ01025915.1/381-178  | UAUCU...**C****C**.**U**.**C**.**G**.**C****G****G****G****A****G**A**G**.A**C****C****G****G****C**G..........AAUA.........................................U**G****C****C****G****G**..**C****G**.**C****C**GAA**G****G****C****G**CA...A..**C****C**G...............CCCC........................**G****G**.AAA**C****G****C****U**CA**G****G****C**..........................................................................A.G.A.C.**G**.**G****A**.**C****C****G**..**C**..**G****A**.**G**.**A**.....GAGACAAA............................................................-**C****G****C****U****G****G****A****A**A**G**U.**G**.**A****G****U**--...............................GAGGCCGCCAGGCUUC...........................A--**A****C****U**..**C****C****A****C****C**GAC**G****G****A****G**CAA..**G****C****G**..**U**.**G****U****C****U**CGC.......AAGG......................................................................................GCGG**G****U****U****C**..**G**.**C****G****C**GG.AAU.**C****U****C**.**U**CA**G****G****U**..............CACG**G**.**G****A****C****A****G****C****G****G**GGGCGAC | |
|  |  | AAFZ01027930.1/602-399  | UAUCU...**C****C**.**U**.**C**.**G**.**C****G****G****G****A****G**A**G**.A**C****C****G****G****C**G..........AACA.........................................G**G****C****C****G****G**..**C****G**.**C****C**GAA**G****G****C****G**CA...A..**C****C**G...............CCCC........................**G****G**.AAA**C****G****C****U**CA**G****G****C**..........................................................................A.G.A.C.**G**.**G****A**.**C****C****G**..**C**..**G****A**.**G**.**A**G....AGACGAA-............................................................-**C****G****C****U****G****G****A****A**A**G**U.**G**.**A****G****U****G****A**GGC............................CAAAAGGCU..................................U**C****A****A****C****U**..**C****C****A****C****C**GAC**G****G****A****G**CAA..**G****C****G**..**U**.**G****U****C****U**CGC.......AAGG......................................................................................GCGG**G****U****U****C**..**G**.**C****G****C**GG.AAU.**C****U****C**.**U**CA**G****G****U**..............CACG**G**.**G****A****C****A****G****C****G****G**GGGCGAC | |
|  |  | AACY01118838.1/22330-22093  | AUGAA...**G****G**.**U**.**A**.**G**.**C****A****G****G****A****G**A**G**.-**U****G****G****G****G**A..........AUUA.........................................A**C****C****C****C****A**..**C****A**.**C****C**GAC**G****A****G****G**CA...A..**C****U**UUGUUGUUGGAAGCAUUCCA.................UAAACAA**A****G**CACU**C****U****U****U**CA**G****G****U**................................................................GCCGCAAGGC-.-.G.U.**G**.**G****A**.**C****U****G**..**U**..**U****A**.**C**.**U**.....GGACGAG-............................................................**C****C****U****C****U****G****G****A****G**A**G**A.**C**.**U****A****C****C****G**AUUGCAUUAACUUGCAA..............UAAU.......................................-**A****G****G****U****G**..**G****C****G****C****C**GAA**G****G****C****G**AAA..**G****U****G**..**U**.**C****G****C****A**GUG.......UUUU.........................................................................................A**A****G****C****G**..**A**.**C****G****C**.G.AAA.**C****G****C**.**U**CA**G****G****C**..............AAAA**G**.**G****A****C****A****G****A****G****G**AGAGGAU | |
|  |  | AACY01554302.1/839-686  | CUAAU...**U****A**.**U**.**A**.**A**.**C****G****G****G****A****G**A**G**.A**C****C****A**--...........AUUU.........................................---**U****G****G**..**C****G**.**C****C**GAA**G****G****A****G**CA...A..**C****C**A...............CCCC........................**G****G**.AAA**C****U****C****U**CA**G****G****C**..........................................................................A.A.A.A.**G**.**G****A**.**C****C****G**..**U**..**U****A**.**U**.**C**.....GUAAUAA-............................................................-**C****U****C****U****G****G****A****A**A**G**C.**A**.**G****G****U**--...............................UAAA.......................................---**G****C****C**..**U****C****A****C****C**GAA**G****G****A****G**UAA..---..-.----..........----..........................................................................................----..-.---.-.-AU.**C****U****C**.**U**CA**G****G****U**..............ACUU**G**.**G****A****C****A****G****A****G****G**GGGACGA | |
|  |  | AACY01425731.1/653-810  | AUGA-...**C****A**.**C**.**G**.**A**.**U****G****G****G****A****G**A**A**.-**C****U****G****C**-...........AUU-.........................................--**G****C****A****G**..**U****G**.**C****C**GAA**G****G****A****G**CA...A..**C****C**G...............CCCC.......................C**G****G**.AAA**C****U****C****U**CA**G****G****C**..........................................................................A.A.A.A.**G**.**G****A**.**C****C****G**..**U**..**C****G**.**U**.**G**A....CGAAUAA-............................................................-**C****U****C****U****G****G****A****G**A**G**U.**G**.**G****U****G****C**-...............................AAAA.......................................C-**G****C****A****C**..**C****C****G****C****C**GAA**G****G****G****A**UAA..---..-.----..........----..........................................................................................----..-.---.-.-CG.**A****U****C**.**U**CA**G****G****C**..............AAAA**G**.**G****A****C****A****G****A****G****G**GGGCAUC | |
|  |  | AACY01094464.1/3446-3620  | UAGAU...**A****U**.**U**.**U**.**G**.**C****G****G****G****A****G**A**G**.A**C****U**---...........UUUA.........................................U---**A****G**..**C****G**.**C****C**GAA**G****G****A****G**CA...A..**C****C**G...............CCCU.......................C**G****G**.AAA**C****U****C****U**CA**G****G****C**..........................................................................A.A.A.A.**G**.**G****A**.**C****C****G**..**C**..**A****A**.**A**.**U**A....UCAUAAAA............................................................-**C****U****C****U****G****G****A****A**A**G**U.**A**.**A****G****U**--...............................UUAAU......................................---**A****C****U**..**U****C****U****C****C**GAC**G****G****G****G**UAA..**G****C****A**..**A**.**C**---..........UUAG..........................................................................................---**G**..**U**.**U****G****U**CA.AAU.**C****U****C**.**U**CA**G****G****G**.............UCCAA**U**.**G****A****C****A****G****A****G****G**AGGGCAG | |
|  |  | AACY01203478.1/609-432  | AGUCU...**U****U**.**U**.**U**.**G**.**U****G****U****G****A****G**A**G**.C**G****U****U****U**-...........UUUU.........................................--**A****A****A****C**..**C****A**.**C****C**GAA**G****G****A****G**CA...A..**C****C**A...............CCCC........................**G****G**.AAA**C****U****C****U**CA**G****G****U**..........................................................................A.A.C.C.**G**.**A****A**.**A****C****A**..**U**..**A****A**.**A**.**A**.....AGUUGAUA............................................................-**A****U****C****U****G****G****A****G**A**G**C.**G**.**A****U****G****A****U**...............................UUUU.......................................-**U****U****C****A****U**..**C****C****A****C****C**GAA**G****G****G****G**UAA..**G****C****C**..**A**.**A**---U.........UUUU..........................................................................................---**U**..**U**.**G****G****U**.C.AAU.**C****U****U**.**U**CA**G****G****U**..............UCCG**U**.**G****A****C****A****G****A****U****G**GGGUAAA | |
|  |  | AACY01620299.1/610-456  | AUUAC...**U****U**.**U**.**U**.**A**.**A****G****G****G****A****G**A**G**.A**C****U****A****C**-...........UUUU.........................................--**G****U****A****G**..**C****G**.**C****C**GAA**G****G****A****G**CA...A..**C****C**A...............CCCC........................**G****G**.AAA**C****U****C****U**CA**G****G****C**..........................................................................A.A.A.A.**G**.**G****A**.**C****C****U**..**U**..**A****A**.**A**.**C**.....GUAUUAA-............................................................-**C****U****C****U****G****G****A****A**A**G**A.**A**.**G****A**---...............................CUUG.......................................A---**U****C**..**U****C****A****C****C**GAA**G****G****A****G**CAA..---..-.----..........----..........................................................................................----..-.---.-.-AU.**C****U****C**.**U**CA**G****G****U**..............AAAA**A**.**G****A****C****A****G****A****U****G**GGGUAAA | |
|  |  | AACY01595506.1/306-133  | UGUCU...**U****U**.**U**.**U**.**G**.**U****G****U****G****A****G**A**G**.A**G****U****U****A**-...........UUUA.........................................--**U****A****A****C**..**C****G**.**C****C**GAA**G****G****A****G**CA...A..**C****C**G...............CCCC........................**G****G**.AAA**C****U****C****U**CA**G****G****C**..........................................................................A.C.C.C.**G**.**A****A**.**A****C****A**..**C**..**A****G**.**A**.**A**.....AGUUGAUA............................................................-**A****U****C****U****G****G****A****G**A**G**U.**G**.**A****U****G****A****U**...............................-UUU.......................................-**A****U****C****A****U**..**C****C****A****C****C**GAA**G****G****G****G**UAA..**G****C****C**..**A**.----..........UUUU..........................................................................................----..**U**.**G****G****U**.C.AGU.**C****U****U**.**U**CA**G****G****U**..............UCCG**U**.**G****A****C****A****G****A****U****G**GGGUAGU | |
|  |  | AACY01113489.1/622-469  | AAGAA...**C****C**.**U**.**U**.**A**.**C****G****G****G****A****G**A**G**.A**C****C****A****C****A**...........UUAA.........................................-**U****G****U****G****G**..**C****G**.**C****C**GAA**G****G****A****G**CA...A..**C****C**A...............CCCC........................**G****G**.AAA**C****U****C****U**CA**G****G****C**..........................................................................A.A.A.A.**G**.**G****A**.**C****C****G**..**U**..**A****A**.-.-.....AUAUUAA-............................................................-**C****U****C****U****G****G****A****A**A**G**-.**A**.**G****A****A**--...............................-AAA.......................................---**U****U****C**..**U****C****G****C****C**GAA**G****G****A****G**CAA..---..-.----..........----..........................................................................................----..-.---.-.-AU.**C****U****C**.**U**CA**G****G****C**..............ACAU**A**.**G****A****C****A****G****A****G****G**GGGCAAA | |
|  |  | AACY01416717.1/684-857  | AGUCU...**U****U**.**U**.**U**.**G**.**U****G****U****G****A****G**A**G**.U**G****U****U****A**-...........UUUA.........................................--**U****A****A****C**..**C****G**.**C****C**GAA**G****G****A****G**CA...A..**C****C**A...............CCCC........................**G****G**.AAA**C****U****C****U**CA**G****G****C**..........................................................................A.C.C.C.**G**.**A****A**.**A****C****A**..**C**..**A****A**.**A**.**A**.....AGUUGAUA............................................................-**A****U****C****U****G****G****A****G**A**G**U.**G**.**A****U****G****A****U**...............................-CUG.......................................-**A****U****C****A****U**..**C****C****A****C****C**GAA**G****G****G****G**UAA..**G****C****C**..**A**.----..........GUUU..........................................................................................----..**U**.**G****G****U**.C.AGU.**C****U****U**.**U**CA**G****G****U**..............UCCG**U**.**G****A****C****A****G****A****U****G**GGGUGUU | |
|  |  | AACY01343298.1/549-706  | AAUCC...**C****A**.**U**.**A**.**A**.**C****G****G****G****A****G**A**G**.A**C****U****A****C**-...........UUUAA........................................U-**G****U****A****G**..**C****G**.**C****C**GAA**G****G****A****G**CA...A..**C****C**A...............CCCC........................**G****G**.AAA**C****U****C****U**CA**G****G****C**..........................................................................A.A.A.A.**G**.**G****A**.**C****C****G**..**U**..**U****A**.**U**.**C**.....GUAAUAA-............................................................-**C****U****C****U****G****G****A****A**A**G**C.**A**.**G****G****C**--...............................UAAA.......................................---**G****C****C**..**U****C****A****C****C**GAA**G****G****A****G**CAA..---..-.----..........----..........................................................................................----..-.---.-.-AU.**C****U****C**.**U**CA**G****G****U**..............UCUU**A**.**G****A****C****A****G****A****U****G**GGGAAAG | |
|  |  | AACY01090750.1/270-426  | AAACC...**U****A**.**A**.**U**.**A**.**C****G****G****G****A****G**A**G**.A**C****C****A****A****U**...........UUA-.........................................-**A****U****U****G****G**..**C****G**.**C****C**GAA**G****G****A****G**CA...A..**C****C**A...............CCCC........................**G****G**.AAA**C****U****C****U**CA**G****G****C**..........................................................................A.A.A.A.**G**.**A****A**.**C****C****G**..**U**..**A****G**.**U**.**A**.....AAUAAUAA............................................................-**C****U****C****U****G****G****A****A**A**G**A.**A**.**G****G****C**--...............................-UUG.......................................---**G****C****C**..**U****C****A****C****C**GAA**G****G****A****G**UAA..---..-.----..........----..........................................................................................----..-.---.-.-AU.**C****U****C**.**U**CA**G****G****U**..............AACU**A**.**G****A****C****A****G****A****G****G**GGGUUGA | |
|  |  | AACY01742622.1/543-386  | AAAAG...**C****A**.**U**.**A**.**A**.**C****G****G****G****A****G**A**G**.A**C****U****A****U**-...........UUAAA........................................U-**A****U****A****G**..**C****G**.**C****C**GAA**G****G****A****G**CA...A..**C****C**U...............CCC-.......................U**G****G**.AAA**C****U****C****U**CA**G****G****C**..........................................................................A.A.A.A.**G**.**G****A**.**C****C****G**..**U**..**U****A**.**U**.**U**.....GUAAUAA-............................................................-**C****U****C****U****G****G****A****A**A**G**C.**A**.**G****G****C**--...............................UUAA.......................................---**G****C****C**..**U****C****A****C****C**GAA**G****G****A****G**CAA..---..-.----..........----..........................................................................................----..-.---.-.-AU.**C****U****C**.**U**CA**G****G****U**..............UCUU**A**.**G****A****C****A****G****A****U****G**GGGAAAA | |
|  |  | AACY01049317.1/140331-140569  | AUGAA...**G****G**.**U**.**A**.**G**.**C****A****G****G****A****G**A**G**.-**U****G****G****G****G**A..........AUUA.........................................A**C****C****C****C****A**..**C****A**.**C****C**GAC**G****A****G****G**CA...A..**C****U**UUGUUUGGGAUCUU..UCCC.................GAUACAA**A****G**CACU**C****U****U****U**CA**G****G****U**................................................................GCCGCAAGGC-.-.G.U.**G**.**G****A**.**C****U****G**..**U**..**U****A**.**C**.**U**.....GGACGAG-............................................................**C****C****U****C****U****G****G****A****G**A**G**A.**C**.**U****A****C****C****G**AUUGCGUUAAGUCGCGA..............UAAU.......................................-**A****G****G****U****G**..**G****C****G****C****C**GAA**G****G****C****G**AAA..**G****U****A**..**G**G**U****G****C****G**UCG.......AUGG.........................................................................................A**U****G****C****A**UU**C**.**U****G****C**.G.AAA.**C****G****C**.**U**CA**G****G****C**..............AAAA**G**.**G****A****C****A****G****A****G****G**AGAGGAU | |
|  |  | AAFZ01013783.1/486-737  | UGGGG...**C****G**.**U**.**U**.**G**.**C****G****G****G****A****G**A**G**.U**G****U****U****U****U**UCUAUCCC...AAUAGACCG....................................A**A****A****A****G****C**..**C****G**.**C****C**GAA**G****G****A****G**CA...A..**C****C**G...............CCCC........................**G****G**.AAA**C****U****C****U**CA**G****G****C**..........................................................................A.A.A.C.**G**.**G****A**.**C****C****G**..**C**..**A****A**.**C**.**C**G....UCUCACAAGCG.........................................................-**C****U****C****U****G****G****A****A**A**G**A.**G**.**U****U****C****G****C**CGAAGCU........................UUAGCGAC...................................G**G****C****G****G****G**..**C****C****A****C****C**GAA**G****G****G****G**UAA..**A****U****C**..**C**.**U****U****G****U**CGC.......UUUU........................................................GUGCCGGUGGGCUUGUCCGCCGAAGCCUUGGCGA**A****G****G****A**..**G**.**G****A****U**.G.AAA.**C****U****C**.**U**CA**G****G****U**..............UCCG**U**.**G****A****C****A****G****A****G****G**GGGCAGU | |
|  |  | AACY01026978.1/654-811  | AAAAU...**C****A**.**U**.**A**.**A**.**C****G****G****G****A****G**A**G**.A**C****U****A****U**-...........UUUAA........................................U-**A****U****A****G**..**C****G**.**C****C**GAA**G****G****A****G**CA...A..**C****C**U...............CCC-.......................U**G****G**.AAA**C****U****C****U**CA**G****G****C**..........................................................................A.A.A.A.**G**.**G****A**.**C****C****G**..**U**..**U****A**.**U**.**U**.....GUAAUAA-............................................................-**C****U****C****U****G****G****A****A**A**G**C.**A**.**G****G****C**--...............................UUAG.......................................---**G****C****C**..**U****C****A****C****C**GAA**G****G****A****G**CAA..---..-.----..........----..........................................................................................----..-.---.-.-AU.**C****U****C**.**U**CA**G****G****U**..............UCUU**A**.**G****A****C****A****G****A****U****G**GGGAAUA | |
|  |  | AAFX01074356.1/709-462  | CCGUA...**G****C**.**U**.**G**.**G**.**U****G****G****G****A****G**A**A**.-**G****C****G****A****C**CG.........CAUGCG.......................................C**G****U****C****G****C**..**U****G**.**C****C**GAA**G****G****C****G**CA...A..**C****C**................GCCC........................**G****G**.AAU**C****G****C****U**CA**G****G****C**..........................................................................-.C.C.G.**A**.**U****A**.**C****C****A**..**C**..**C****G**.**C**.**C**.....GCAAGGGAUGGUCGUUACCCACCGGUAGCAGCCGCCCGACAA..........................-**C****U****C****U****G****G****A****G**A**G**A.**U**.**C****G****G****C****U**...............................GCCGCCGUGUCCGCGGCG.........................A**G****G****C****C****G**..**G****C****G****C****C**GAA**G****G****G****G**CAA..**G****A****A**..**G**.**C****G****C****G**GGGCG.....ACCU...................................................................................CCCCACC**C****G****C****G**..**C**.**C****U****C**.C.AAA.**C****U****C**.**U**CA**G****G****C**..............GAAA**G**.**G****A****C****A****G****A****G****G**GGCCCGG | |
|  |  | AACY01118123.1/1235-996  | AUGAA...**G****G**.**U**.**A**.**G**.**C****A****G****G****A****G**A**G**.-**U****G****G****G****G**A..........AUUA.........................................A**C****C****C****C****A**..**C****A**.**C****C**GAC**G****A****G****G**CA...A..**C****U**UUGUUUUGGGAUGUG.UCCC.................GAACCAA**A****G**CACU**C****U****U****U**CA**G****G****U**................................................................GCCGCAAGGC-.-.G.U.**G**.**G****A**.**C****U****G**..**U**..**U****A**.**C**.**U**.....GGACGAG-............................................................**C****C****U****C****U****G****G****A****G**A**G**A.**C**.**U****A****C****C****G**AUUGCGUUAAGUCGCGA..............UAAU.......................................-**A****G****G****U****G**..**G****C****G****C****C**GAA**G****G****C****G**AAA..**G****U****A**..**G**G**U****G****C****G**UCG.......AUGG.........................................................................................A**U****G****C****A**UU**C**.**U****G****C**.G.AAA.**C****G****C**.**U**CA**G****G****C**..............AAAA**G**.**G****A****C****A****G****A****G****G**AGAGGAU | |
|  |  | AACY01677127.1/98-255  | AAAAG...**C****A**.**U**.**A**.**A**.**C****G****G****G****A****G**A**G**.A**C****U****A****U****U**...........UAGA.........................................-**U****A****U****A****G**..**C****G**.**C****C**GAA**G****G****A****G**CA...A..**C****C**U...............CCC-.......................U**G****G**.AAA**C****U****C****U**CA**G****G****C**..........................................................................A.A.A.A.**G**.**G****A**.**C****C****G**..**U**..**U****A**.**U**.**U**.....GUAAUAA-............................................................-**C****U****C****U****G****G****A****A**A**G**C.**A**.**G****G****C**--...............................UUAG.......................................---**G****C****C**..**U****C****A****C****C**GAA**G****G****A****G**CAA..---..-.----..........----..........................................................................................----..-.---.-.-AU.**C****U****C**.**U**CA**G****G****U**..............UCUU**A**.**G****A****C****A****G****A****U****G**GGGAAAA | |
|  |  | AACY01054830.1/1232-1058  | UAGAU...**A****U**.**U**.**C**.**G**.**C****G****G****G****A****G**A**G**.A**C****U**---...........UUUA.........................................U---**A****G**..**C****G**.**C****C**GAA**G****G****A****G**CA...A..**C****C**G...............CCCU.......................C**G****G**.AAA**C****U****C****U**CA**G****G****C**..........................................................................A.A.A.A.**G**.**G****A**.**C****C****G**..**C**..**A****A**.**A**.**U**A....UCUUCAAA............................................................-**C****U****C****U****G****G****A****A**A**G**U.**A**.**A****G****U**--...............................UAAAU......................................---**A****C****U**..**U****C****U****C****C**GAC**G****G****G****G**UAA..**G****C****A**..**A**.**C**---..........UUUG..........................................................................................---**G**..**U**.**U****G****U**CA.AAU.**C****U****C**.**U**CA**G****G****G**.............UCCAA**U**.**G****A****C****A****G****A****G****G**AGGGCAG | |
|  |  | AACY01191722.1/559-731  | UGAUU...**G****A**.**C**.**U**.**A**.**C****G****G****G****A****G**A**G**.U**U****C****A****A****C**...........UUA-.........................................-**G****U****U****G****A**..**C****G**.**C****C**GAA**G****G****A****G**CA...A..**G****G**G...............UCCC.......................C**C****C**GAAA**C****U****C****U**CA**G****G****C**..........................................................................A.A.A.A.**G**.**U****A**.**C****C****G**..**U**UU**A****G**.**A**.**U**.....CGACCAA-............................................................--**U****C****U****G****G****A****A**A**G**-.**A**.**A****U****G****U**-...............................UCAG.......................................--**U****C****A****U**..**U****C****A****C****C**GAA**G****G****G****G**AAA..**G****G****C**..-.----..........AAAA..........................................................................................----..-.**G****C****C**.G.AAG.**C****U****C**.**U**CA**G****G****U**..............AAUA**A**.**G****A****C****A****G****A****G****G**GGGACAU | |
|  |  | AACY01555112.1/863-706  | GGAAG...**C****A**.**U**.**A**.**A**.**C****G****G****G****A****G**A**G**.A**C****U****A****U**-...........UUAAA........................................U-**A****U****A****G**..**C****G**.**C****C**GAA**G****G****A****G**CA...A..**C****C**U...............CCC-.......................U**G****G**.AAA**C****U****C****U**CA**G****G****C**..........................................................................A.A.A.A.**G**.**G****A**.**C****C****G**..**U**..**U****A**.**U**.**U**.....GUAAUAA-............................................................-**C****U****C****U****G****G****A****A**A**G**C.**A**.**G****G****C**--...............................UUAG.......................................---**G****C****C**..**U****C****A****C****C**GAA**G****G****A****G**UAA..---..-.----..........----..........................................................................................----..-.---.-.-AU.**C****U****C**.**U**CA**G****G****U**..............UCUU**A**.**G****A****C****A****G****A****U****G**GGGAAAA | |
|  |  | AACY01312344.1/358-198  | AAUAA...**C****U**.**U**.**A**.**A**.**C****G****G****G****A****G**A**G**.A**C****U****A****C****U**U..........AUUAA........................................A**U****G****U****A****G**..**C****G**.**C****C**GAA**G****G****A****G**CA...A..**C****C**A...............CCCC........................**G****G**.AAA**C****U****C****U**CA**G****G****C**..........................................................................A.A.A.A.**G**.**G****A**.**C****C****G**..**U**..**U****A**.**U**.**C**.....GUAAUAA-............................................................-**C****U****C****U****G****G****A****A**A**G**C.**A**.**G****G****C**--...............................UUUA.......................................---**G****C****C**..**U****C****A****C****C**GAA**G****G****A****G**CAA..---..-.----..........----..........................................................................................----..-.---.-.-AU.**C****U****C**.**U**CA**G****G****U**..............UCUU**A**.**G****A****C****A****G****A****U****G**GGGAUGG | |
|  |  | AACY01057751.1/2452-2299  | AAAUU...**U****C**.**U**.**U**.**A**.**C****G****G****G****A****G**A**G**.A**C****U****A****C****U**...........UAAG.........................................-**A****G****U****A****G**..**C****G**.**C****C**GAA**G****G****A****G**CA...A..**C****C**A...............CCCC........................**G****G**.AAA**C****U****C****U**CA**G****G****C**..........................................................................A.A.A.A.**G**.**G****A**.**C****C****G**..**U**..**A****C**.-.-.....AUAUUAA-............................................................-**C****U****C****U****G****G****A****A**A**G**A.**G**.**A****A**---...............................UUAU.......................................----**U****C**..**U****C****G****C****C**GAA**G****G****A****G**CAA..---..-.----..........----..........................................................................................----..-.---.-.-AU.**C****U****C**.**U**CA**G****G****C**..............AAAA**A**.**G****A****C****A****G****A****G****G**GGGUUAA | |
|  |  | AAFZ01014837.1/525-722  | AAGAA...**C****G**.**C**.**A**.-.**C****A****G****A****A****G**A**G**.A**C****C****C****C****G**A..........UUCGA........................................A**C****G****G****G****G**..**C****G**.**C****C**GAA**G****G****C****G**CA...A..**C****U**C...............GCCC........................**G****G**.AAA**C****G****C****U**CA**G****G****C**..........................................................................A.A.A.U.**G**.**G****A**.**C****U****G**..**U**.G**U****G**.**C**.**G**C....UGAUUAG-............................................................-**C****U****C****U****G****G****A****G**A**G**C.**G**.**G****U****C**--...............................GAAGUUG....................................C--**G****G****C**..**C****C****A****C****C**GAA**G****G****G****G**AAU..**G****U****G**..**G**.**C****C****A****G**A.........GCUC...................................................................................CCGGAGC**C****C****G****G**..**U**.**C****A****U**.U.GAU.**C****U****C**.**U**CA**G****G****U**............ACAAGA**G**.**G****A****C****A****G****A****G****G**GGCGGCA | |
|  |  | AACY01036988.1/13-169  | AGAAG...**A****A**.**U**.**A**.**A**.**C****G****G****G****A****G**A**G**.A**C****U****A****U**-...........UUAAA........................................U-**A****U****A****G**..**C****G**.**C****C**GAA**G****G****A****G**CA...A..**C****C**U...............CCC-.......................U**G****G**.AAA**C****U****C****U**CA**G****G****C**..........................................................................A.A.A.A.**G**.**G****A**.**C****C****G**..**U**..**U****A**.**U**.**U**.....GUAAUAA-............................................................-**C****U****C****U****G****G****A****A**A**G**C.**A**.**G****G****C**--...............................-AUA.......................................---**G****C****C**..**U****C****A****C****C**GAA**G****G****A****G**UAA..---..-.----..........----..........................................................................................----..-.---.-.-AU.**C****U****C**.**U**CA**G****G****U**..............UCUU**A**.**G****A****C****A****G****A****U****G**GGGAAAA | |
|  |  | AACY01208374.1/679-523  | AAUUG...**U****A**.**U**.**A**.**A**.**C****G****G****G****A****G**A**G**.A**C****U****A****C**-...........UUAAA........................................U-**G****U****A****G**..**C****G**.**C****C**GAA**G****G****A****G**CA...A..**C****C**................CCCC........................**G****G**.AAA**C****U****C****U**CA**G****G****C**..........................................................................A.A.A.A.**G**.**G****A**.**C****C****G**..**U**..**U****A**.**U**.**U**.....GUAAUAA-............................................................-**C****U****C****U****G****G****A****A**A**G**C.**A**.**G****G****C**--...............................UUUA.......................................---**G****C****C**..**U****C****A****C****C**GAA**G****G****A****G**CAA..---..-.----..........----..........................................................................................----..-.---.-.-AU.**C****U****C**.**U**CA**G****G****U**..............UCUU**A**.**G****A****C****A****G****A****U****G**GGGAAUG | |
|  |  | AACY01172300.1/554-707  | AUCAU...**U****A**.**A**.**U**.**A**.**C****G****G****G****A****G**A**G**.A**C****C****A****A**-...........UUCA.........................................A-**U****U****G****G**..**C****G**.**C****C**GAA**G****G****A****G**CA...A..**A****U**A...............CCCC........................**G****G**.AAA**C****U****C****U**CA**G****G****C**..........................................................................A.A.A.A.**G**.**G****A**.**C****C****G**..**U**..**A****C**.-.-.....AUAAUAA-............................................................-**C****U****C****U****G****G****A****A**A**G**A.**A**.**G****G****C**--...............................-UUA.......................................---**G****C****C**..**U****C****A****C****C**GAA**G****G****A****G**UAA..---..-.----..........----..........................................................................................----..-.---.-.-AU.**C****U****C**.**U**CA**G****G****U**..............ACCA**A**.**G****A****C****A****G****A****G****G**GGGAAGA | |
|  |  | AACY01022034.1/1959-2110  | AGAAU...**U****U**.**A**.**U**.**A**.**C****G****G****G****A****G**A**G**.A**C****U****A****C**-...........UUUU.........................................--**G****U****A****G**..**C****G**.**C****C**GAA**G****G****A****G**CA...A..**C****C**A...............CCCC........................**G****G**.AAA**C****U****C****U**CA**G****G****C**..........................................................................A.A.A.U.**G**.**G****A**.**C****C****G**..**U**..**A****C**.**A**.-.....U-AUUAA-............................................................-**C****U****C****U****G****G****A****A**A**G**A.**G**.**A****A**---...............................UUAU.......................................----**U****C**..**U****C****G****C****C**GAA**G****G****A****G**CAA..---..-.----..........----..........................................................................................----..-.---.-.-AU.**C****U****C**.**U**CA**G****G****C**..............AAAA**A**.**G****A****C****A****G****A****G****G**GGGCAUA | |
|  |  | AACY01556926.1/216-43  | AAUUU...**A****U**.**U**.**U**.**G**.**C****G****G****G****A****G**A**G**.A**C****U**---...........UUAU.........................................----**A****G**..**C****G**.**C****C**GAA**G****G****A****G**CA...A..**C****C**G...............CCCU.......................C**G****G**.AAA**C****U****C****U**CA**G****G****C**..........................................................................A.A.A.A.**G**.**G****A**.**C****C****G**..**C**..**A****A**.**A**.**U**A....AAUAUAAA............................................................-**C****U****C****U****G****G****A****A**A**G**U.**A**.**A****G****U**--...............................UUAAU......................................---**A****C****U**..**U****C****U****C****C**GAC**G****G****G****G**UAA..**G****C****A**..**A**.**C**---..........UUAA..........................................................................................---**G**..**U**.**U****G****U**CA.AAU.**C****U****C**.**U**CA**G****G****G**.............UCCAA**U**.**G****A****C****A****G****A****G****G**AGGGCAG | |
|  |  | AACY01770991.1/518-675  | AUGA-...**C****A**.**C**.**G**.**A**.**U****G****G****G****A****G**A**A**.-**C****U****G****C**-...........GUU-.........................................--**G****C****A****G**..**U****G**.**C****C**GAA**G****G****A****G**CA...A..**C****C**G...............CCCC.......................C**G****G**.AAA**C****U****C****U**CA**G****G****C**..........................................................................A.A.A.A.**G**.**G****A**.**C****C****G**..**U**..**C****G**.**U**.**G**A....CGAAUAG-............................................................-**C****U****C****U****G****G****A****G**A**G**U.**G**.**A****U****A****C**-...............................UAAAU......................................--**G****U****A****U**..**C****C****G****C****C**GAA**G****G****G****A**UAA..---..-.----..........----..........................................................................................----..-.---.-.-CG.**A****U****C**.**U**CA**G****G****C**..............AAAA**G**.**G****A****C****A****G****A****G****G**GGGCAUC | |
|  |  | AACY01030181.1/493-341  | CGCGA...**U****G**.**U**.**A**.**A**.**C****G****G****G****A****G**A**G**.A**C****C****A**--...........UUA-.........................................---**U****G****G**..**C****G**.**C****C**GAA**G****G****A****G**CA...A..**C****C**A...............CCCC........................**G****G**.AAA**C****U****C****U**CA**G****G****C**..........................................................................A.A.A.A.**G**.**G****A**.**C****C****G**..**U**..**U****A**.**C**.**C**.....GUAAUAA-............................................................-**C****U****C****U****G****G****A****A**A**G**C.**A**.**G****G****C**--...............................UUAG.......................................---**G****C****C**..**U****C****A****C****C**GAA**G****G****A****U**UAA..---..-.----..........----..........................................................................................----..-.---.-.-AU.**C****U****C**.**U**CA**G****G****U**..............ACAU**A**.**G****A****C****A****G****A****U****G**GGGUUAG | |
|  |  | AACY01436834.1/264-112  | CACAA...**U****G**.**U**.**A**.**A**.**C****G****G****G****A****G**A**G**.A**C****C****A**--...........UUU-.........................................---**U****G****G**..**C****G**.**C****C**GAA**G****G****A****G**CA...A..**C****C**A...............CCCC........................**G****G**.AAA**C****U****C****U**CA**G****G****C**..........................................................................A.A.A.A.**G**.**G****A**.**C****C****G**..**U**..**U****A**.**C**.**C**.....GUAAUAA-............................................................-**C****U****C****U****G****G****A****A**A**G**C.**A**.**G****G****C**--...............................GGAA.......................................---**G****C****C**..**U****C****A****C****C**GAA**G****G****A****U**UAA..---..-.----..........----..........................................................................................----..-.---.-.-AU.**C****U****C**.**U**CA**G****G****U**..............ACAU**A**.**G****A****C****A****G****A****U****G**GGGUUAG | |
|  |  | AACY01062053.1/3687-3535  | AAACU...**U****A**.**A**.**U**.**A**.**C****G****G****G****A****G**A**G**.A**C****C****A****A****U**...........UUA-.........................................-**A****U****U****G****G**..**C****G**.**C****C**GAA**G****G****A****G**CA...A..**C****C**A...............CCCC........................**G****G**.AAA**C****U****C****U**CA**G****G****C**..........................................................................A.A.A.A.**G**.**G****A**.**C****C****G**..**U**..**A****C**.-.-.....AUAUUAA-............................................................-**C****U****C****U****G****G****A****A**A**G**A.**G**.**A****A**---...............................UUAU.......................................----**U****C**..**U****C****G****C****C**GAA**G****G****A****G**CAA..---..-.----..........----..........................................................................................----..-.---.-.-AU.**C****U****C**.**U**CA**G****G****C**..............ACAA**A**.**G****A****C****A****G****A****G****G**GGGCACA | |
|  |  | AACY01566166.1/473-631  | AUUGA...**C****A**.**C**.**G**.**A**.**U****G****G****G****A****G**A**A**.-**C****U****G****C**-...........GUU-.........................................--**G****C****A****G**..**U****G**.**C****C**GAA**G****G****A****G**CA...A..**C****C**G...............CCCC.......................C**G****G**.AAA**C****U****C****U**CA**G****G****C**..........................................................................A.A.A.A.**G**.**G****A**.**C****C****G**..**U**..**C****G**.**U**.**G**A....CGAAUAG-............................................................-**C****U****C****U****G****G****A****G**A**G**U.**G**.**A****U****G****C**-...............................UAAAU......................................--**G****U****A****U**..**C****C****G****C****C**GAA**G****G****G****A**UAA..---..-.----..........----..........................................................................................----..-.---.-.-CG.**A****U****C**.**U**CA**G****G****C**..............AAAA**G**.**G****A****C****A****G****A****G****G**GGGCAUC | |
|  |  | AACY01038366.1/1358-1557  | CAACA...**C****C**.**C**.**A**.**G**.**U****G****G****G****A****G**A**G**.U**U****C****A****U****C**CAGCAAGU...AACACAGCU....................................G**G****C****U****G****G**..**C****G**.**C****C**GAA**G****G****A****G**CA...A..**C****C**G...............CCCC.......................G**G****G**.AAA**C****U****C****U**CA**G****G****C**..........................................................................C.C.C.A.**G**.**G****A**.**C****C****G**..**C**..**A****G**.**G**.**G**A....AGAAGCAA............................................................-**C****G****C****U****G****G****A****G**A**G**C.**G**.**A****G****U****U****G**UC.............................CUCGG......................................G**C****G****G****C****U**..**C****C****A****C****C**GAA**G****G****G****G**AAA..--**A**..**C**.**U****G****C****A**..........AAUC..........................................................................................-**G****C****G**..**G**.**C****C**-.-.AAU.**C****U****C**.**U**CA**G****G****U**..............UCCA**C**.**G****A****C****A****G****C****G****C**GGGGCGU | |
|  |  | AACY01214094.1/342-189  | AAACC...**U****A**.**A**.**U**.**A**.**C****G****G****G****A****G**A**G**.A**C****C****A****A****U**...........UUA-.........................................-**A****U****U****G****G**..**C****G**.**C****C**GAA**G****G****A****G**CA...A..**C****C**A...............CCCC........................**G****G**.AAA**C****U****C****U**CA**G****G****C**..........................................................................A.A.A.A.**G**.**G****A**.**C****C****G**..**U**..**A****C**.-.-.....AUAUUAA-............................................................-**C****U****C****U****G****G****A****A**A**G**A.**G**.**A****A**---...............................UUAU.......................................----**U****C**..**U****C****G****C****C**GAA**G****G****A****G**CAA..---..-.----..........----..........................................................................................----..-.---.-.-AU.**C****U****C**.**U**CA**G****G****C**.............AAAAA**A**.**G****A****C****A****G****A****G****G**GGGUUAA | |
|  |  | AACY01069903.1/1929-1776  | UUAAA...**C****C**.**U**.**C**.**A**.**C****G****G****G****A****G**A**G**.A**C****U****A****U****U**U..........UAU-.........................................-**A****A****U****A****G**..**C****G**.**C****C**GAA**G****G****A****G**CA...A..**C****C**A...............CCCC........................**G****G**.AAA**C****U****C****U**CA**G****G****C**..........................................................................A.A.A.A.**G**.**G****A**.**C****C****G**..**U**..**A****C**.-.-.....AUAUUAA-............................................................-**C****U****C****U****G****G****A****A**A**G**A.**G**.**A****A**---...............................UUAU.......................................----**U****C**..**U****C****G****C****C**GAA**G****G****A****G**CAA..---..-.----..........----..........................................................................................----..-.---.-.-AU.**C****U****C**.**U**CA**G****G****C**..............ACAU**A**.**G****A****C****A****G****A****G****G**GGGUCGA | |
|  |  | AACY01536227.1/768-616  | UGCCA...**C****G**.**U**.**A**.**A**.**C****G****G****G****A****G**A**G**.A**C****C****A**--...........UUU-.........................................---**A****G****G**..**C****G**.**C****C**GAA**G****G****A****G**CA...A..**C****C**A...............CCCC........................**G****G**.AAA**C****U****C****U**CA**G****G****C**..........................................................................A.A.A.A.**G**.**G****A**.**C****C****G**..**U**..**U****A**.**C**.**A**.....GUAAUAA-............................................................-**C****U****C****U****G****G****A****A**A**G**C.**A**.**G****G****C**--...............................GAGA.......................................---**G****C****C**..**U****C****A****C****C**GAA**G****G****A****U**UAA..---..-.----..........----..........................................................................................----..-.---.-.-AU.**C****U****C**.**U**CA**G****G****U**..............ACAU**A**.**G****A****C****A****G****A****U****G**GGGUUAG | |
|  |  | AACY01098174.1/1075-1234  | AUUUA...**U****A**.**U**.**A**.**U**.**C****G****G****G****A****G**A**G**.A**C****U****G****A****U**AAG........CUU-.........................................-**A****U****U****A****G**..**C****G**.**C****C**GAA**G****G****A****G**CA...A..**C****C**A...............CCCC........................**G****G**.AAA**C****U****C****U**CA**G****G****C**..........................................................................A.A.A.A.**G**.**G****A**.**C****C****G**..**U**..**U****A**.**A**.**A**C....GCAUAACA............................................................-**C****U****C****U****G****G****A****A**A**G**A.**G**.**A****U**---...............................UAAU.......................................----**U****U**..**C****C****G****C****C**GAA**G****G****A****G**CAA..---..-.----..........----..........................................................................................----..-.---.-.-AA.**C****U****C**.**U**CA**G****G****C**..............AAAA**A**.**U****A****C****A****G****A****U****G**GGGUAAA | |
|  |  | AACY01097204.1/366-521  | AUGA-...**C****A**.**U**.**A**.**A**.**C****G****G****G****A****G**A**G**.A**C****U****A****C**-...........UUGAA........................................U-**G****U****A****G**..**C****G**.**C****C**GAA**G****G****A****G**CA...A..**C****C**U...............CCCC........................**G****G**.AAA**C****U****C****U**CA**G****G****C**..........................................................................A.A.A.A.**G**.**G****A**.**C****C****G**..**U**..**U****A**.**U**.**U**.....GUAAUAA-............................................................-**C****U****C****U****G****G****A****A**A**G**C.**A**.**G****G****C**--...............................-UUU.......................................---**G****C****C**..**U****C****A****C****C**GAA**G****G****A****G**UAA..---..-.----..........----..........................................................................................----..-.---.-.-AU.**C****U****C**.**U**CA**G****G****U**..............UCUU**A**.**G****A****C****A****G****A****U****G**GGGAUGA | |
|  |  | AACY01292673.1/323-167  | AAAAA...**C****A**.**U**.**A**.**A**.**C****G****G****G****A****G**A**G**.A**C****U****A****U**-...........UUAAA........................................U-**A****U****A****G**..**C****G**.**C****C**GAA**G****G****A****G**CA...A..**C****C**U...............CCC-.......................U**G****G**.AAA**C****U****C****U**CA**G****G****C**..........................................................................A.A.A.A.**G**.**G****A**.**C****C****G**..**U**..**U****A**.**U**.**U**.....GUAAUAA-............................................................-**C****U****C****U****G****G****A****A**A**G**C.**A**.**G****G****C**--...............................-AUA.......................................---**G****C****C**..**U****C****A****C****C**GAA**G****G****A****G**UAA..---..-.----..........----..........................................................................................----..-.---.-.-AU.**C****U****C**.**U**CA**G****G****U**..............UCUU**A**.**G****A****C****A****G****A****U****G**GGGAAAA | |
|  |  | AAGA01003599.1/263-457  | UAAGC...**U****G**.**C**.**G**.**A**.**C****A****G****G****A****G**A**G**.A**G****U****C****A**-...........UUCAA........................................U-**U****G****G****C**..**C****A**.**C****C**GAA**G****A****C****G**CA...A..**C****C**................GCCC........................**G****G**.AAA**C****G****C****U**CA**G****G****U**.........................................................................AA.A.A.A.**G**.**G****A**.**C****U****G**..**C**..**U****G**.**C**.**A**GU...UGUCUAA-............................................................-**C****U****C****U****G****G****A****G**A**G**U.**G**.**G****U****A****G****U**...............................GUCUG......................................A**A****C****U****A****U**..**C****C****A****C****C**GAA**G****G****U****G**AAA..**G****G****U**..**G**.**A****C****C****G**AGUUU.....AAGU........................................................................................GC**U****C****G****U**..**C**.**G****C****C**.C.GAU.**C****U****C**.**U**CA**G****G****U**..............AACC**G**.**G****A****C****A****G****A****G****G**GGUUUUU | |
|  |  | AACY01057775.1/1210-1360  | AUUUA...**U**-.**U**.**U**.**A**.**C****G****G****G****A****G**A**G**.A**C****U****A****C**-...........UUUU.........................................--**G****U****A****G**..**C****G**.**C****C**GAA**G****G****A****G**CA...A..**C****C**A...............CCCC........................**G****G**.AAA**C****U****C****U**CA**G****G****C**..........................................................................A.A.A.A.**G**.**G****A**.**C****C****G**..**U**..**A****C**.-.-.....AUAUUAA-............................................................-**C****U****C****U****G****G****A****A**A**G**A.**G**.**A****A**---...............................UUAU.......................................----**U****C**..**U****C****G****C****C**GAA**G****G****A****G**CAA..---..-.----..........----..........................................................................................----..-.---.-.-AU.**C****U****C**.**U**CA**G****G****C**..............ACCA**A**.**G****A****C****A****G****A****G****G**GGGCAAA | |
|  |  | AACY01692717.1/872-719  | AAGAC...**U****U**.**U**.**U**.**A**.**C****G****G****G****A****G**A**G**.A**C****U****A****C**-...........UCAA.........................................U-**G****U****A****G**..**C****G**.**C****C**GAA**G****G****A****G**CA...A..**A****U**A...............CCCA........................**A****U**.AAU**C****U****C****U**CA**G****G****C**..........................................................................A.A.A.A.**G**.**G****A**.**C****C****G**..**U**..**A****C**.-.-.....GUAUUAA-............................................................-**C****U****C****U****G****G****A****A**A**G**-.**A**.**G****A****U**--...............................UAAG.......................................---**U****U****C**..**U****C****G****C****C**GAA**G****G****A****G**CAA..---..-.----..........----..........................................................................................----..-.---.-.-AA.**C****U****C**.**U**CA**G****G****C**..............AACA**A**.**G****A****C****A****G****A****G****G**GGAUUAA | |
|  |  | AACY01764368.1/88-243  | AAAAA...**U****C**.**G**.**U**.**A**.**C****G****G****G****A****G**A**G**.A**C****U****A****C**-...........UUGAU........................................U-**G****U****A****G**..**C****G**.**C****C**GAA**G****G****A****G**CA...A..**C****C**A...............CCCC........................**G****G**.AAA**C****U****C****U**CA**G****G****C**..........................................................................A.A.A.A.**G**.**G****A**.**C****C****G**..**U**..**A****C**.-.-.....AUAUUAA-............................................................-**C****U****C****U****G****G****A****A**A**G**-.**A**.**G****A****A**--...............................-AAU.......................................---**U****U****C**..**U****C****G****C****C**GAA**G****G****A****G**CUAA.---..-.----..........----..........................................................................................----..-.---.-.AAU.**C****U****C**.**U**CA**G****G****C**..............ACCA**C**.**G****A****C****A****G****A****G****G**GGGUUAG | |
|  |  | AACY01607003.1/442-595  | UUAAU...**C****C**.**U**.**C**.**A**.**C****G****G****G****A****G**A**G**.A**C****U****A****U****U**U..........UAU-.........................................-**A****A****U****A****G**..**C****G**.**C****C**GAA**G****G****A****G**CA...A..**C****C**A...............CCCC........................**G****G**.AAA**C****U****C****U**CA**G****G****C**..........................................................................A.A.A.A.**G**.**G****A**.**C****C****G**..**U**..**A****C**.-.-.....AUAUUAA-............................................................-**C****U****C****U****G****G****A****A**A**G**A.**G**.**A****A**---...............................UUAU.......................................----**U****C**..**U****C****G****C****C**GAA**G****G****A****G**CAA..---..-.----..........----..........................................................................................----..-.---.-.-AU.**C****U****C**.**U**CA**G****G****C**..............ACAU**A**.**G****A****C****A****G****A****G****G**GGGUUGA | |
|  |  | AACY01477438.1/702-551  | AACUU...**A****C**.**U**.**U**.**A**.**C****G****G****G****A****G**A**G**.A**C****U****A****C**-...........UUUU.........................................--**G****U****A****G**..**C****G**.**C****C**GAA**G****G****A****G**CA...A..**C****C**A...............CCCC........................**G****G**.AAA**C****U****C****U**CA**G****G****C**..........................................................................A.A.A.A.**G**.**G****A**.**C****C****G**..**U**..**A****C**.-.-.....AUAUUAA-............................................................-**C****U****C****U****G****G****A****A**A**G**A.**G**.**A****A**---...............................UUAU.......................................----**U****C**..**U****C****G****C****C**GAA**G****G****A****G**CAA..---..-.----..........----..........................................................................................----..-.---.-.-AU.**C****U****C**.**U**CA**G****G****C**..............ACCA**A**.**G****A****C****A****G****A****G****G**GGGCAAA | |
|  |  | AACY01073923.1/767-617  | AUUUA...**U**-.**A**.**U**.**A**.**C****G****G****G****A****G**A**G**.A**C****U****A****C**-...........UUUU.........................................--**G****U****A****G**..**C****G**.**C****C**GAA**G****G****A****G**CA...A..**C****C**A...............CCCC........................**G****G**.AAA**C****U****C****U**CA**G****G****C**..........................................................................A.A.A.A.**G**.**G****A**.**C****C****G**..**U**..**A****C**.-.-.....AUAUUAA-............................................................-**C****U****C****U****G****G****A****A**A**G**A.**G**.**A****A**---...............................UUAU.......................................----**U****C**..**U****C****G****C****C**GAA**G****G****A****G**CAA..---..-.----..........----..........................................................................................----..-.---.-.-AU.**C****U****C**.**U**CA**G****G****C**..............ACCA**A**.**G****A****C****A****G****A****G****G**GGGCAAA | |
|  |  | AACY01089582.1/102-259  | CUAUA...**U****G**.**U**.**A**.**A**.**C****G****G****G****A****G**A**G**.A**U****C****A****C****G**...........UUUA.........................................-**U****G****U****G****G**..**C****G**.**C****C**GAA**G****G****A****G**CA...A..**C****U**A...............CCC-.......................U**A****G**.AAA**C****U****C****U**CA**G****G****C**..........................................................................A.A.A.A.**G**.**G****A**.**C****C****G**..**U**..**U****G**.**C**.**U**.....GUAAUAA-............................................................-**C****U****C****U****G****G****A****A**A**G**C.**A**.**G****G****C**--...............................GUAA.......................................---**G****C****C**..**U****C****A****C****C**GAA**G****G****A****U**UAA..---..-.----..........----..........................................................................................----..-.---.-.-AU.**C****U****C**.**U**CA**G****G****U**..............ACAC**G**.**G****A****C****A****G****A****U****A**GGGAGUG | |
|  |  | AACY01227047.1/394-244  | AUUUA...**U**-.**A**.**U**.**A**.**C****G****G****G****A****G**A**G**.A**C****U****A****C**-...........UUUU.........................................--**G****U****A****G**..**C****G**.**C****C**GAA**G****G****A****G**CA...A..**C****C**A...............CCCC........................**G****G**.AAA**C****U****C****U**CA**G****G****C**..........................................................................A.A.A.A.**G**.**G****A**.**C****C****G**..**U**..**A****C**.-.-.....AUAUUAA-............................................................-**C****U****C****U****G****G****A****A**A**G**A.**G**.**A****A**---...............................UUAU.......................................----**U****C**..**U****C****G****C****C**GAA**G****G****A****G**CAA..---..-.----..........----..........................................................................................----..-.---.-.-AU.**C****U****C**.**U**CA**G****G****C**..............ACCA**A**.**G****A****C****A****G****A****G****G**GGGCAAA | |
|  |  | AACY01637419.1/809-659  | AUUUA...**U**-.**A**.**U**.**A**.**C****G****G****G****A****G**A**G**.A**C****U****A****C**-...........UUUU.........................................--**G****U****A****G**..**C****G**.**C****C**GAA**G****G****A****G**CA...A..**C****C**A...............CCCC........................**G****G**.AAA**C****U****C****U**CA**G****G****C**..........................................................................A.A.A.A.**G**.**G****A**.**C****C****G**..**U**..**A****C**.-.-.....AUAUUAA-............................................................-**C****U****C****U****G****G****A****A**A**G**A.**G**.**A****A**---...............................UUAU.......................................----**U****C**..**U****C****G****C****C**GAA**G****G****A****G**CAA..---..-.----..........----..........................................................................................----..-.---.-.-AU.**C****U****C**.**U**CA**G****G****C**..............ACCA**A**.**G****A****C****A****G****A****G****G**GGGCAAA | |
|  |  | AACY01458105.1/145-300  | UUUAU...**U****U**.**U**.**U**.**A**.**A****G****G****A****A****G**A**G**.A**C****U****A****C****A**...........UUU-.........................................-**U****G****U****A****G**..**C****G**.**C****C**GAA**G****A****A****G**CA...A..**C****C**G...............CCCC........................**G****G**.AAA**C****U****U****U**CA**G****G****C**..........................................................................A.A.A.A.**G**.**G****A**.**C****C****U**..**U**..**A****A**.**A**.**C**.....AUAUUAA-............................................................-**C****U****C****U****G****G****A****A**A**G**A.**A**.**G****A****U**--...............................-UAA.......................................---**A****U****C**..**U****C****A****C****C**GAA**G****G****A****G**UAA..---..-.----..........----..........................................................................................----..-.---.-.-AU.**C****U****C**.**U**CA**G****G****U**..............AAAA**A**.**G****A****C****A****G****A****U****G**GGGUAAG | |
|  |  | AACY01006032.1/1891-1737  | AUUAU...**U****U**.**A**.**U**.**A**.**C****G****G****G****A****G**A**G**.A**C****U****A****C****U**U..........AUGA.........................................-**U****G****U****A****G**..**C****G**.**C****C**GAA**G****G****A****G**CA...A..**C****C**A...............CCCC........................**G****G**.AAA**C****U****C****U**CA**G****G****C**..........................................................................A.A.A.A.**G**.**G****A**.**C****C****G**..**U**..**A****C**.**A**.-.....U-AUUAA-............................................................-**C****U****C****U****G****G****A****A**A**G**A.**G**.**A****A**---...............................UAAU.......................................----**U****C**..**U****C****G****C****C**GAA**G****G****A****G**CAA..---..-.----..........----..........................................................................................----..-.---.-.-AU.**C****U****C**.**U**CA**G****G****C**..............ACAU**A**.**G****A****C****A****G****A****G****G**GGGUUGA | |
|  |  | AACY01290605.1/170-13  | AAACA...**C****U**.**G**.**U**.**A**.**C****G****G****G****A****G**A**G**.U**C****U****G**--...........UUUAG........................................U--**C****A****G**..**C****A**.**C****C**GAA**G****G****A****G**CA...A..**A****G**................CCCA........................**G****G**.AAA**C****U****C****U**CA**G****G****U**..........................................................................A.A.A.U.**G**.**G****A**.**C****C****G**..**U**..**A****C**.**G**.**U**.....AAAAUAAA............................................................-**C****U****C****U****G****G****A****A**A**G**C.**G**.**G****A****C**--...............................UAAA.......................................---**G****U****C**..**C****C****A****C****C**GAA**G****G****A****G**CUAA.---..-.----..........----..........................................................................................----..-.---.-.AAU.**C****U****C**.**U**CA**G****G****U**..............AAAC**C**.**G****A****C****A****G****A****A****G**GGCAUAA | |
|  |  | AACY01240424.1/517-361  | AAAAG...**U****A**.**U**.**A**.**A**.**C****G****G****G****A****G**A**G**.A**C****U****A****U**-...........UUAAA........................................U-**A****U****A****G**..**C****G**.**C****C**GAA**G****G****A****G**CA...A..**C****C**U...............CCC-.......................U**G****G**.AAA**C****U****C****U**CA**G****G****C**..........................................................................A.A.A.A.**G**.**G****A**.**C****C****G**..**U**..**U****A**.**U**.**U**.....GUAAUAA-............................................................-**C****U****C****U****G****G****A****A**A**G**C.**A**.**G****G****C**--...............................-AUA.......................................---**G****C****C**..**U****C****A****C****C**GAA**G****G****A****G**UAA..---..-.----..........----..........................................................................................----..-.---.-.-AU.**C****U****C**.**U**CA**G****G****U**..............UCUU**A**.**G****A****C****A****G****A****U****G**GGGAAAA | |
|  |  | AACY01301178.1/197-41  | AAAAG...**C****A**.**U**.**A**.**A**.**C****G****G****G****A****G**A**G**.A**C****U****A****U**-...........UUAAA........................................U-**A****U****A****G**..**C****G**.**C****C**GAA**G****G****A****G**CA...A..**C****C**U...............CCC-.......................U**G****G**.AAA**C****U****C****U**CA**G****G****C**..........................................................................A.A.A.A.**G**.**G****A**.**C****C****G**..**U**..**U****A**.**U**.**U**.....GUAAUAA-............................................................-**C****U****C****U****G****G****A****A**A**G**C.**A**.**G****G****C**--...............................-AUA.......................................---**G****C****C**..**U****C****A****C****C**GAA**G****G****A****G**UAA..---..-.----..........----..........................................................................................----..-.---.-.-AU.**C****U****C**.**U**CA**G****G****U**..............UCUU**A**.**G****A****C****A****G****A****U****G**GGGAAAA | |
|  |  | AACY01073416.1/341-189  | AAAUU...**U****C**.**A**.**U**.**A**.**C****G****G****G****A****G**A**G**.A**C****U****A****C**-...........UUAA.........................................U-**G****U****A****G**..**C****G**.**C****C**GAA**G****G****A****G**CA...A..**C****C**A...............CCCC........................**G****G**.AAA**C****U****C****U**CA**G****G****C**..........................................................................A.A.A.A.**G**.**G****A**.**C****C****G**..**U**..**A****C**.-.-.....AUAUUAA-............................................................-**C****U****C****U****G****G****A****A**A**G**A.**G**.**A****A**---...............................UUAU.......................................----**U****C**..**U****C****G****C****C**GAA**G****G****A****G**CAA..---..-.----..........----..........................................................................................----..-.---.-.-AU.**C****U****C**.**U**CA**G****G****C**..............ACCA**A**.**G****A****C****A****G****A****G****G**GGGCUAG | |
|  |  | AACY01156350.1/205-47  | AGUCC...**A****U**.**U**.**A**.**U**.**C****G****G****G****A****G**A**G**.A**C****U****A****A****A**A..........ACUU.........................................-**U****U****U****A****G**..**C****G**.**C****C**GAA**G****G****A****G**CA...A..**C****C**A...............CCCC........................**G****G**.AAA**C****U****C****U**CA**G****G****C**..........................................................................A.A.A.A.**G**.**G****A**.**C****C****G**..**A**..**U****G**.**U**.**A**G....GCAUAACA............................................................-**C****U****C****U****G****G****A****A**A**G**A.**G**.**A****U**---...............................UAAA.......................................----**U****U**..**C****C****G****C****C**GAA**G****G****A****G**CAA..---..-.----..........----..........................................................................................----..-.---.-.-AA.**C****U****C**.**U**CA**G****G****C**..............AAAA**A**.**U****A****C****A****G****A****U****G**GGGUCAC | |
|  |  | AACY01747919.1/167-319  | AGUUU...**U****U**.**U**.**A**.**A**.**C****G****G****G****A****G**A**G**.A**C****U****A****C**-...........UUAG.........................................U-**G****U****A****G**..**C****G**.**C****C**GAA**G****G****A****G**CA...A..**C****C**A...............CCCC........................**G****G**.AAA**C****U****C****U**CA**G****G****C**..........................................................................A.A.A.A.**G**.**G****A**.**C****C****G**..**U**..**A****C**.**A**.-.....U-AUUAA-............................................................-**C****U****C****U****G****G****A****A**A**G**A.**G**.**A****A**---...............................UUAU.......................................----**U****C**..**U****C****G****C****C**GAA**G****G****A****G**CAA..---..-.----..........----..........................................................................................----..-.---.-.-AU.**C****U****C**.**U**CA**G****G****C**..............ACAA**G**.**G****A****C****A****G****A****G****G**GGGUUAA | |
|  |  | AACY01307493.1/187-32  | UAAAG...**U****U**.**C**.**U**.**A**.**C****G****G****G****A****G**A**G**.A**C****U****A****C**-...........UUUAA........................................U-**G****U****A****G**..**C****G**.**C****C**GAA**G****G****A****G**CA...A..**C****C**A...............CCCC........................**G****G**.AAA**C****U****C****U**CA**G****G****C**..........................................................................A.A.A.A.**G**.**G****A**.**C****C****G**..**U**..**U****C**.**A**.-.....U-AUUAA-............................................................-**C****U****C****U****G****G****A****A**A**G**-.**A**.**G****A****A**--...............................UAAA.......................................---**U****U****C**..**U****C****G****C****C**GAA**G****G****A****G**CAA..---..-.----..........----..........................................................................................----..-.---.-.-AU.**C****U****C**.**U**CA**G****G****C**.............ACAAA**A**.**G****A****C****A****G****A****G****G**GGGCAAU | |
|  |  | AACY01082871.1/1142-990  | AAUUU...**U****C**.**U**.**U**.**A**.**C****G****G****G****A****G**A**G**.A**C****U****A****C**-U..........UAUU.........................................--**G****U****A****G**..**C****G**.**C****C**GAA**G****G****A****G**CA...A..**C****C**A...............CCCC........................**G****G**.AAA**C****U****C****U**CA**G****G****C**..........................................................................A.A.A.A.**G**.**G****A**.**C****C****G**..**U**..**A****C**.-.-.....AUAUUAA-............................................................-**C****U****C****U****G****G****A****A**A**G**A.**G**.**A****A**---...............................UUAU.......................................----**U****C**..**U****C****G****C****C**GAA**G****G****A****G**CAA..---..-.----..........----..........................................................................................----..-.---.-.-AU.**C****U****C**.**U**CA**G****G****C**..............AACA**A**.**G****A****C****A****G****A****G****G**GGGCUAA | |
|  |  | AACY01645358.1/717-566  | ACUAA...**U****U**.**A**.**U**.**A**.**C****G****G****G****A****G**A**G**.A**C****U****A****C**-...........UUUU.........................................--**G****U****A****G**..**C****G**.**C****C**GAA**G****G****A****G**CA...A..**C****C**A...............CCCC........................**G****G**.AAA**C****U****C****U**CA**G****G****C**..........................................................................A.A.A.U.**G**.**G****A**.**C****C****G**..**U**..**A****C**.**A**.-.....U-AUUAA-............................................................-**C****U****C****U****G****G****A****A**A**G**A.**G**.**A****A**---...............................UUAU.......................................----**U****C**..**U****C****G****C****C**GAA**G****G****A****G**CAA..---..-.----..........----..........................................................................................----..-.---.-.-AU.**C****U****C**.**U**CA**G****G****C**..............ACCA**A**.**G****A****C****A****G****A****G****G**GGGCUAA | |
|  |  | AACY01009815.1/2077-1920  | AUUUU...**A****C**.**U**.**A**.**U**.**C****G****G****G****A****G**A**G**.A**C****U****A****U**-...........UAGA.........................................U-**U****U****A****G**..**C****A**.**C****C**GAA**G****G****A****G**CA...A..**C****C**A...............CCCC........................**G****G**.AAA**C****U****C****U**CA**G****G****U**.........................................................................AA.A.A.A.**G**.**G****A**.**C****C****G**..**A**..**U****A**.**G**.**U**U....GCAUAACA............................................................-**C****U****C****U****G****G****A****A**A**G**A.**G**.**A****A**---...............................UUAA.......................................----**U****U**..**C****C****G****C****C**GAA**G****G****A****G**CAA..---..-.----..........----..........................................................................................----..-.---.-.-AA.**C****U****C**.**U**CA**G****G****C**..............AAAA**A**.**U****A****C****A****G****A****U****G**GGGUAAU | |
|  |  | AACY01108908.1/2722-2566  | AAGAG...**A****A**.**U**.**A**.**G**.**U****G****G****G****A****G**A**A**.-**C****U****G****G**-...........UGUA.........................................--**U****C****A****G**..**U****G**.**C****C**GAA**G****G****A****G**CA...A..**C****C**G...............CCCC........................**G****G**.AAA**C****U****C****U**CA**G****G****C**..........................................................................A.A.A.C.**G**.**G****A**.**C****C****A**..**C**..**U****G**.**U**.**U**.....UUAUAAAA............................................................--**U****C****U****G****G****A****G**A**G**A.**G**.**A****U****G****C**-...............................GAGA.......................................--**G****C****A****U**..**C****C****G****C****C**GAA**G****G****G****A**UAA..---..-.----..........----..........................................................................................----..-.---.-.-CG.**A****U****C**.**U**CA**G****G****C**..............AAAG**G**.**G****A****C****A****G****A****A****A**GGGGCAU | |
|  |  | AACY01052317.1/253-403  | AUUUA...**U**-.**A**.**U**.**A**.**C****G****G****G****A****G**A**G**.A**C****U****A****C**-...........UUUU.........................................--**G****U****A****G**..**C****G**.**C****C**GAA**G****G****A****G**CA...A..**C****C**A...............CCCC........................**G****G**.AAA**C****U****C****U**CA**G****G****C**..........................................................................A.A.A.A.**G**.**G****A**.**C****C****G**..**U**..**A****C**.-.-.....AUAUUAA-............................................................-**C****U****C****U****G****G****A****A**A**G**A.**G**.**A****A**---...............................UUAU.......................................----**U****C**..**U****C****G****C****C**GAA**G****G****A****G**CAA..---..-.----..........----..........................................................................................----..-.---.-.-AU.**C****U****C**.**U**CA**G****G****C**..............ACCA**A**.**G****A****C****A****G****A****G****G**GGGCACA | |
|  |  | AACY01178142.1/345-495  | AUUUA...**U**-.**A**.**U**.**A**.**C****G****G****G****A****G**A**G**.A**C****U****A****C**-...........UUUU.........................................--**G****U****A****G**..**C****G**.**C****C**GAA**G****G****A****G**CA...A..**C****C**A...............CCCC........................**G****G**.AAA**C****U****C****U**CA**G****G****C**..........................................................................A.A.A.A.**G**.**G****A**.**C****C****G**..**U**..**A****C**.-.-.....AUAUUAA-............................................................-**C****U****C****U****G****G****A****A**A**G**A.**G**.**A****A**---...............................UUAU.......................................----**U****C**..**U****C****G****C****C**GAA**G****G****A****G**CAA..---..-.----..........----..........................................................................................----..-.---.-.-AU.**C****U****C**.**U**CA**G****G****C**..............ACCA**A**.**G****A****C****A****G****A****G****G**GGGCACA | |
|  |  | AACY01457821.1/592-742  | AUUUA...**U**-.**A**.**U**.**A**.**C****G****G****G****A****G**A**G**.A**C****U****A****C**-...........UUUU.........................................--**G****U****A****G**..**C****G**.**C****C**GAA**G****G****A****G**CA...A..**C****C**A...............CCCC........................**G****G**.AAA**C****U****C****U**CA**G****G****C**..........................................................................A.A.A.A.**G**.**G****A**.**C****C****G**..**U**..**A****C**.-.-.....AUAUUAA-............................................................-**C****U****C****U****G****G****A****A**A**G**A.**G**.**A****A**---...............................UUAU.......................................----**U****C**..**U****C****G****C****C**GAA**G****G****A****G**CAA..---..-.----..........----..........................................................................................----..-.---.-.-AU.**C****U****C**.**U**CA**G****G****C**..............ACCA**A**.**G****A****C****A****G****A****G****G**GGGCACA | |
|  |  | AACY01720423.1/172-322  | AUUUA...**U**-.**A**.**U**.**A**.**C****G****G****G****A****G**A**G**.A**C****U****A****C**-...........UUUU.........................................--**G****U****A****G**..**C****G**.**C****C**GAA**G****G****A****G**CA...A..**C****C**A...............CCCC........................**G****G**.AAA**C****U****C****U**CA**G****G****C**..........................................................................A.A.A.A.**G**.**G****A**.**C****C****G**..**U**..**A****C**.-.-.....AUAUUAA-............................................................-**C****U****C****U****G****G****A****A**A**G**A.**G**.**A****A**---...............................UUAU.......................................----**U****C**..**U****C****G****C****C**GAA**G****G****A****G**CAA..---..-.----..........----..........................................................................................----..-.---.-.-AU.**C****U****C**.**U**CA**G****G****C**..............ACCA**A**.**G****A****C****A****G****A****G****G**GGGCACA | |
|  |  | AACY01004991.1/6376-6224  | CUUUG...**C****G**.**U**.**A**.**A**.**C****G****G****G****A****G**A**G**.A**C****C****A**--...........UUU-.........................................---**A****G****G**..**C****G**.**C****C**GAA**G****G****A****G**CA...A..**C****C**A...............CCCC........................**G****G**.AAA**C****U****C****U**CA**G****G****C**..........................................................................A.A.A.A.**G**.**G****A**.**C****C****G**..**U**..**U****A**.**C**.**C**.....GUAAUAA-............................................................-**C****U****C****U****G****G****A****A**A**G**C.**A**.**G****G****C**--...............................GAAA.......................................---**G****C****C**..**U****C****A****C****C**GAA**G****G****A****U**UAA..---..-.----..........----..........................................................................................----..-.---.-.-AU.**C****U****C**.**U**CA**G****G****U**..............ACAU**A**.**G****A****C****A****G****A****U****G**GGGUUAG | |
|  |  | AACY01539255.1/462-618  | AAGAG...**U****G**.**U**.**A**.**A**.**C****G****G****G****A****G**A**G**.A**C****U****A****A**-...........UUUA.........................................U-**U****U****A****G**..**C****G**.**C****C**GAA**G****G****A****G**CA...A..**C****U**A...............CCC-.......................U**A****G**.AAA**C****U****C****U**CA**G****G****C**..........................................................................A.A.A.A.**G**.**G****A**.**C****C****G**..**U**..**U****A**.**C**.**C**.....GUAAUAA-............................................................-**C****U****C****U****G****G****A****A**A**G**U.**A**.**G****G****C**--...............................AUUA.......................................---**G****C****C**..**U****C****A****C****C**GAA**G****G****A****U**UAA..---..-.----..........----..........................................................................................----..-.---.-.-AU.**C****U****C**.**U**CA**G****G****U**..............ACAU**G**.**G****A****C****A****G****A****U****A**GGGAUUG | |
|  |  | AACY01564010.1/396-551  | AGUAU...**U****U**.**A**.**A**.**A**.**C****G****G****G****A****G**A**A**.U**G****G****U****U****U**GA.........AAUA.........................................-**A****A****A****C****C**..**U****G**.**C****C**GAA**G****G****A****G**CA...A..**C****C**A...............CCCC........................**G****G**.AAA**C****U****C****U**CA**G****G****C**..........................................................................A.A.A.U.**G**.**G****A**.**C****C****G**..**U**..**A****C**.**A**.-.....U-AUUAA-............................................................-**C****U****C****U****G****G****A****A**A**G**A.**G**.**A****A**---...............................UUAU.......................................----**U****C**..**U****C****G****C****C**GAA**G****G****A****G**CAA..---..-.----..........----..........................................................................................----..-.---.-.-AU.**C****U****C**.**U**CA**G****G****C**..............AAAA**A**.**G****A****C****A****G****A****G****G**GGGCAUA | |
|  |  | AACY01349110.1/404-247  | UUUAA...**C**-.**A**.**A**.**G**.**C****G****G****G****A****G**A**G**.A**C****U****G****C****A**G..........AAAGA........................................U**A****G****C****A****G**..**A****G**.**C****C**GAA**G****G****A****G**CA...A..**A****C**A...............CCCC........................**G****G**.AAA**C****U****C****U**CA**G****G****C**..........................................................................A.A.A.A.**G**.**G****A**.**C****C****G**..**C**..**A****U**.-.-.....GUAAUAA-............................................................-**C****U****C****U****G****G****A****A**A**G**C.**A**.**G****G****C**--...............................ACAG.......................................---**G****C****C**..**U****C****A****C****C**GAA**G****G****A****G**UAA..---..-.----..........----..........................................................................................----..-.---.-.-AU.**C****U****C**.**U**CA**G****G****U**..............ACCC**A**.**G****A****C****A****G****A****G****G**GGGCUGA | |
|  |  | AAFZ01019250.1/545-308  | CGUAC...**C****G**.**U**.**G**.**A**.**C****G****G****G****A****G**A**G**.U**U****C****G****A****C**C..........AUUGCCGU.....................................G**G****U****C****G****G**..**C****G**.**C****C**GAA**G****G****A****G**CA...A..**U****C**G...............CCCC.......................A**G****A**.AAA**C****U****C****U**CA**G****G****C**..........................................................................A.G.A.U.**G**.**G****A**.**C****C****G**..**U**..**C****G**.**C**.**G**.....GAUUGAGG............................................................-**C****U****C****U****G****G****A****G**A**G**A.**G**.**A****A****A****A****A**...............................GAAAGUCGCAUCUAACAUAUCAGGGCGAUGUU...........C**G****G****A****U****U**..**C****C****A****C****C**GAA**G****G****A****U**GAA..**A****U****C**..**C**.**G****G****U****U**GGCAG.....AAAC..............................................................................AUUGUUUUUGCA**A****A****C****A**..**G**.**G****G****G**.U.ACA.**A****U****C**.**U**CA**G****G****U**..............CAAG**G**.**G****A****C****A****G****A****G****G**GGGCGAG | |
|  |  | AACY01051039.1/2512-2359  | UAAGA...**U****C**.**U**.**U**.**A**.**C****G****G****G****A****G**A**G**.A**C****U****A****C****U**...........CAA-.........................................-**A****G****U****A****G**..**C****G**.**C****C**GAA**G****G****A****G**CA...A..**A****U**A...............CCCA........................**A****U**.AAU**C****U****C****U**CA**G****G****C**..........................................................................A.A.A.A.**G**.**G****A**.**C****C****G**..**U**..**A****C**.-.-.....GUAUUAA-............................................................-**C****U****C****U****G****G****A****A**A**G**-.**A**.**G****A****U**--...............................UAAG.......................................---**U****U****C**..**U****C****G****C****C**GAA**G****G****A****G**CAA..---..-.----..........----..........................................................................................----..-.---.-.-AA.**C****U****C**.**U**CA**G****G****C**..............AACA**A**.**G****A****C****A****G****A****G****G**GGGUUUA | |
|  |  | AACY01162000.1/699-879  | UACCU...**C****U**.**U**.**U**.**U**.**U****G****U****G****A****G**A**G**.C**G****U****U****U**-...........UAAA.........................................--**U****A****A****C**..**C****G**.**C****C**GAA**G****G****A****G**CA...A..**C****U**A...............CCC-.......................U**A****G**.AAA**C****U****C****U**CA**G****G****U**..........................................................................A.A.A.A.**G**.**U****A**.**U****C****A**..**U**..**A****A**.**A**.**G**.....AGUUGACA............................................................-**A****U****C****U****G****G****A****G**A**G**A.**G**.**A****C****A****U****A**UU.............................UUUU.......................................-**U****A****U****G****U**..**C****C****A****C****C**GAA**G****G****A****G**UAA..**G****C****U**..**C**.**A****U**--..........ACA-..........................................................................................--**A****A**..**G**.**A****G****U**.U.AAU.**C****U****C**.**U**CA**G****G****U**.............ACACA**U**.**G****A****C****A****G****A****U****U**AGGGUUU | |
|  |  | AACY01627527.1/773-616  | AACCU...**A****C**.**U**.**G**.**U**.**C****G****G****G****A****G**A**G**.A**C****U****A****U**-...........UAGA.........................................U-**U****U****A****G**..**C****A**.**C****C**GAA**G****G****A****G**CA...A..**C****C**A...............CCCC........................**G****G**.AAA**C****U****C****U**CA**G****G****U**.........................................................................AA.A.A.A.**G**.**G****A**.**C****C****G**..**A**..**U****A**.**G**.**U**U....GCAUAACA............................................................-**C****U****C****U****G****G****A****A**A**G**A.**G**.**A****A**---...............................UUAA.......................................----**U****U**..**C****C****G****C****C**GAA**G****G****A****G**CAA..---..-.----..........----..........................................................................................----..-.---.-.-AA.**C****U****C**.**U**CA**G****G****C**..............AAAA**A**.**U****A****C****A****G****A****U****G**GGGGUAA | |
|  |  | AACY01133166.1/464-314  | AUUUA...**C**-.**A**.**U**.**A**.**C****G****G****G****A****G**A**G**.A**C****U****A****C**-...........UUUU.........................................--**G****U****A****G**..**C****G**.**C****C**GAA**G****G****A****G**CA...A..**C****C**A...............CCCC........................**G****G**.AAA**C****U****C****U**CA**G****G****C**..........................................................................A.A.A.A.**G**.**G****A**.**C****C****G**..**U**..**A****C**.-.-.....AUAUUAA-............................................................-**C****U****C****U****G****G****A****A**A**G**A.**G**.**A****A**---...............................UUAU.......................................----**U****C**..**U****C****G****C****C**GAA**G****G****A****G**CAA..---..-.----..........----..........................................................................................----..-.---.-.-AU.**C****U****C**.**U**CA**G****G****C**..............ACCA**A**.**G****A****C****A****G****A****G****G**GGGCACA | |
|  |  | AACY01063905.1/213-363  | GUUCU...**G****C**.**A**.**A**.**A**.**C****G****G****G****A****G**A**G**.A**C****U****A****A**-...........UUU-.........................................--**U****U****A****G**..**C****G**.**C****C**GAA**G****G****A****G**CA...A..**C****C**A...............CCCC........................**G****G**.AAA**C****U****C****U**CA**G****G****C**..........................................................................A.A.A.A.**G**.**G****A**.**C****C****G**..**U**..**U****C**.**A**.**U**.....A---UUAA............................................................-**C****U****C****U****G****G****A****A**A**G**A.**G**.**A****A**---...............................UUAU.......................................----**U****C**..**U****C****G****C****C**GAA**G****G****A****G**CAA..---..-.----..........----..........................................................................................----..-.---.-.-AU.**C****U****C**.**U**CA**G****G****C**..............ACAA**A**.**G****A****C****A****G****A****G****G**GGGUCAA | |
|  |  | AACY01036273.1/264-414  | AUUUA...**U**-.**U**.**U**.**A**.**C****G****G****G****A****G**A**G**.A**C****U****A****C**-...........UUUU.........................................--**G****U****A****G**..**C****G**.**C****C**GAA**G****G****A****G**CA...A..**C****C**A...............CCCC........................**G****G**.AAA**C****U****C****U**CA**G****G****C**..........................................................................A.A.A.A.**G**.**G****A**.**C****C****G**..**U**..**A****C**.-.-.....AUAUUAA-............................................................-**C****U****C****U****G****G****A****A**A**G**A.**G**.**A****A**---...............................UUAU.......................................----**U****C**..**U****C****G****C****C**GAA**G****G****A****G**CAA..---..-.----..........----..........................................................................................----..-.---.-.-AU.**C****U****C**.**U**CA**G****G****C**..............ACCA**A**.**G****A****C****A****G****A****G****G**GGGCACU | |
|  |  | AACY01009687.1/849-1007  | AACUC...**A****U**.**U**.**A**.**U**.**C****G****G****G****A****G**A**G**.A**C****U****A****A****A**C..........ACUU.........................................-**U****U****U****A****G**..**C****G**.**C****C**GAA**G****G****A****G**CA...A..**C****C**A...............CCCC........................**G****G**.AAA**C****U****C****U**CA**G****G****C**..........................................................................A.A.A.C.**G**.**G****A**.**C****C****G**..**A**..**U****A**.**C**.**A**G....GCAUAACA............................................................-**C****U****C****U****G****G****A****A**A**G**A.**G**.**A****U**---...............................UAAA.......................................----**U****U**..**C****C****G****C****C**GAA**G****G****A****G**CAA..---..-.----..........----..........................................................................................----..-.---.-.-AA.**C****U****C**.**U**CA**G****G****C**..............AAAA**A**.**U****A****C****A****G****A****U****G**GGGUCUU | |
|  |  | AACY01437467.1/168-322  | GUUUU...**A****C**.**U**.**A**.**U**.**C****G****G****G****A****G**A**G**.A**C****U****A**--...........UUUU.........................................U--**U****A****G**..**C****G**.**C****C**GAA**G****G****A****G**CA...A..**C****C**A...............CCCC........................**G****G**.AAA**C****U****C****U**CA**G****G****C**..........................................................................A.A.A.A.**G**.**G****A**.**C****C****G**..**A**..**U****C**.**G**.**A**A....GCAUAACA............................................................-**C****U****C****U****G****G****A****A**A**G**A.**G**.**A****U**---...............................UUAU.......................................----**U****U**..**C****C****G****C****C**GAA**G****G****A****G**CAA..---..-.----..........----..........................................................................................----..-.---.-.-AA.**C****U****C**.**U**CA**G****G****C**..............AAAA**A**.**U****A****C****A****G****A****U****G**GGGUGUG | |
|  |  | AACY01462169.1/663-506  | AUUUU...**A****C**.**U**.**G**.**U**.**C****G****G****G****A****G**A**G**.A**C****U****A****U**-...........UAAA.........................................U-**U****U****A****G**..**C****A**.**C****C**GAA**G****G****A****G**CA...A..**C****C**A...............CCCC........................**G****G**.AAA**C****U****C****U**CA**G****G****U**.........................................................................AA.A.A.A.**G**.**G****A**.**C****C****G**..**A**..**U****A**.**G**.**U**U....GCAUAACA............................................................-**C****U****C****U****G****G****A****A**A**G**A.**G**.**A****A**---...............................UUAA.......................................----**U****U**..**C****C****G****C****C**GAA**G****G****A****G**CAA..---..-.----..........----..........................................................................................----..-.---.-.-AA.**C****U****C**.**U**CA**G****G****C**..............AAAA**A**.**U****A****C****A****G****A****U****G**GGGUAAU | |
|  |  | AACY01687076.1/388-238  | AUUCA...**U**-.**A**.**U**.**A**.**C****G****G****G****A****G**A**G**.A**C****U****A****C**-...........UUUU.........................................--**G****U****A****G**..**C****G**.**C****C**GAA**G****G****A****G**CA...A..**C****C**A...............CCCC........................**G****G**.AAA**C****U****C****U**CA**G****G****C**..........................................................................A.A.A.A.**G**.**G****A**.**C****C****G**..**U**..**A****C**.-.-.....AUAUUAA-............................................................-**C****U****C****U****G****G****A****A**A**G**A.**G**.**A****A**---...............................UUAU.......................................----**U****C**..**U****C****G****C****C**GAC**G****G****A****G**CAA..---..-.----..........----..........................................................................................----..-.---.-.-AU.**C****U****C**.**U**CA**G****G****C**..............ACCA**A**.**G****A****C****A****G****A****G****G**GGGCAAA | |
|  |  | AACY01804208.1/346-195  | AUUUU...**U**-.**U**.**U**.**A**.**C****G****G****G****A****G**A**G**.A**C****U****A****C**-...........UUGA.........................................U-**G****U****A****G**..**C****G**.**C****C**GAA**G****G****A****G**CA...A..**C****C**A...............CCCC........................**G****G**.AAA**C****U****C****U**CA**G****G****C**..........................................................................A.A.A.A.**G**.**G****A**.**C****C****G**..**U**..**A****C**.-.-.....AUAUUAA-............................................................-**C****U****C****U****G****G****A****A**A**G**A.**G**.**A****A**---...............................UUAU.......................................----**U****C**..**U****C****G****C****C**GAA**G****G****A****G**CAA..---..-.----..........----..........................................................................................----..-.---.-.-AU.**C****U****C**.**U**CA**G****G****C**..............AAAU**A**.**G****A****C****A****G****A****G****G**GGGCAAA | |
|  |  | AACY01656328.1/626-470  | AUCCA...**U****G**.-.**A**.**U**.**C****G****G****G****A****G**A**G**.A**C****U****A****A****G**C..........AUU-.........................................-**U****U****U****A****G**..**C****G**.**C****C**GAA**G****G****A****G**CA...A..**C****C**A...............CCCC........................**G****G**.AAA**C****U****C****U**CA**G****G****C**..........................................................................A.A.A.A.**G**.**G****A**.**C****C****G**..**A**..**U****G**.**U**.**A**G....GCAUAACA............................................................-**C****U****C****U****G****G****A****A**A**G**A.**G**.**A****U**---...............................UAAA.......................................----**U****U**..**C****C****G****C****C**GAA**G****G****A****G**CAA..---..-.----..........----..........................................................................................----..-.---.-.-AA.**C****U****C**.**U**CA**G****G****C**..............AAAA**A**.**U****A****C****A****G****A****U****G**GGGUCAG | |
|  |  | AACY01427220.1/515-358  | AUUCG...**U****G**.**A**.**U**.-.**C****G****G****G****A****G**A**G**.A**C****U****A****A****A**C..........AAUU.........................................-**U****U****U****A****G**..**C****G**.**C****C**GAA**G****G****A****G**CA...A..**C****C**A...............CCCC........................**G****G**.AAA**C****U****C****U**CA**G****G****C**..........................................................................A.A.A.A.**G**.**G****A**.**C****C****G**..**A**..**U****U**.**U**.**A**G....GCAUAACA............................................................-**C****U****C****U****G****G****A****A**A**G**A.**G**.**A****U**---...............................UAAA.......................................----**U****U**..**C****C****G****C****C**GAA**G****G****A****G**CAA..---..-.----..........----..........................................................................................----..-.---.-.-AA.**C****U****C**.**U**CA**G****G****C**..............AAAA**A**.**U****A****C****A****G****A****U****G**GGGUCAU | |
|  |  | AACY01054414.1/999-840  | UUAAA...**A****A**.-.-.**A**.**C****G****G****G****A****G**A**G**.U**C****U****A****U****U**...........UAA-.........................................-**A****U****U****A****G**..**C****G**.**C****C**GAA**G****G****A****G**CA...A..**C****G**................ACCC........................**G****G**.AAA**C****U****C****U**CA**G****G****C**..........................................................................A.A.A.A.**G**.**G****A**.**C****C****G**..-..--.-.-.....UAAUUAA-............................................................**C****A****U****C****U****G****G****A****A**A--.**A**.**G****A****G****U****U**...............................-UGA.......................................-**A****A****C****U****C**..**U**-**A****C****C**GAA**G****G****G****G**CAA..**G****G****C**..**U**.----..........---U..........................................................................................----..**A**.**G****C****U**.-.AAA.**C****U****C**.**U**CA**G****G****U**..............ACAA**U**.**G****A****C****A****G****A****G****G**GGAAAAG | |
|  |  | AACY01563612.1/74-229  | CCAUU...**U****A**.**A**.**U**.**A**.**C****G****G****G****A****G**A**G**.A**C****C****A****A****U**...........UUA-.........................................-**A****U****U****G****G**..**C****G**.**C****C**GAA**G****G****A****G**CA...A..**C****C**A...............CCCC........................**G****G**.AAA**C****U****C****U**CA**G****G****C**..........................................................................A.A.A.A.**G**.**G****A**.**C****C****G**..**U**..**A****G**.**A**.**C**.....AUAAUAA-............................................................-**C****U****C****U****G****G****A****A**A**G**A.**A**.**G****G****C**--...............................-UUA.......................................---**G****C****C**..**U****C****A****C****C**GAC**G****G****A****G**UAA..---..-.----..........----..........................................................................................----..-.---.-.-AU.**C****U****C**.**U**CA**G****G****U**..............AACU**A**.**G****A****C****A****G****A****G****G**GGGUUUA | |
|  |  | AACY01791867.1/375-525  | AUUUA...**U**-.**A**.**U**.**A**.**C****G****G****G****A****G**A**G**.A**C****U****A****C**-...........UUUU.........................................--**G****U****A****G**..**C****G**.**C****C**GAA**G****G****A****G**CA...A..**C****C**A...............CCCC........................**G****G**.AAA**C****U****C****U**CA**G****G****C**..........................................................................A.A.A.A.**G**.**G****A**.**C****C****G**..**U**..**A****C**.-.-.....AUAUUAA-............................................................-**C****U****C****U****G****G****A****A**A**G**A.**G**.**A****A**---...............................UUUU.......................................----**U****C**..**U****C****G****C****C**GAA**G****G****A****G**CAA..---..-.----..........----..........................................................................................----..-.---.-.-AU.**C****U****C**.**U**CA**G****G****C**..............ACCA**A**.**G****A****C****A****G****A****G****G**GGGCACA | |
|  |  | AACY01768664.1/736-581  | CCGAU...**U****G**.**U**.**A**.**A**.**C****G****G****G****A****G**A**G**.U**C****U****A****U**-...........AAAA.........................................U-**A****U****A****G**..**C****G**.**C****C**GAA**G****G****A****G**CA...A..**C****C**A...............CCCC........................**G****G**.AAA**C****U****C****U**CA**G****G****C**..........................................................................A.A.A.A.**G**.**G****A**.**C****C****G**..**U**..**U****G**.**C**.**C**.....GUAAUAA-............................................................-**C****U****C****U****G****G****A****A**A**G**C.**A**.**G****G****C**--...............................-UUU.......................................---**G****C****C**..**U****C****A****C****C**GAA**G****G****A****G**UAA..---..-.----..........----..........................................................................................----..-.---.-.-AU.**C****U****C**.**U**CA**G****G****U**..............UCCA**A**.**A****A****C****A****G****A****U****G**GGGUACA | |
|  |  | AACY01054200.1/433-280  | ACCUA...**A****A**.**U**.**A**.**U**.**C****G****G****G****A****G**A**G**.A**C****U****A**--...........AUAA.........................................---**U****A****G**..**C****G**.**C****C**GAA**G****G****A****G**CA...A..**C****C**A...............CCCC........................**G****G**.AAA**C****U****C****U**CA**G****G****C**..........................................................................A.A.A.A.**G**.**G****A**.**C****C****G**..**A**..**U****U**.**A**.**A**A....GCAUAACA............................................................-**C****U****C****U****G****G****A****A**A**G**A.**G**.**A****U**---...............................UAAU.......................................----**U****U**..**C****C****G****C****C**GAA**G****G****A****G**UAA..---..-.----..........----..........................................................................................----..-.---.-.-AA.**C****U****C**.**U**CA**G****G****C**..............AAAA**A**.**U****A****C****A****G****A****U****G**GGGGUGA | |
|  |  | AACY01682843.1/246-399  | AUUAA...**U****A**.**A**.**A**.**A**.**C****G****G****A****A****G**A**G**.A**C****U****A****C**-...........UUAAA........................................U-**G****U****G****G**..**C****G**.**C****C**GAA**G****G****A****G**CA...A..**C****C**A...............CCCC........................**G****G**.AAA**C****U****C****U**CA**G****G****C**..........................................................................A.A.A.A.**G**.**G****A**.**C****C****G**..**U**..**A****C**.-.-.....AUAUUAA-............................................................-**C****U****C****U****G****G****A****A**A**G**A.**G**.**A****A**---...............................UUAU.......................................----**U****C**..**U****C****G****C****C**GAA**G****G****A****G**CAA..---..-.----..........----..........................................................................................----..-.---.-.-AU.**C****U****C**.**U**CA**G****G****C**..............AAAA**A**.**G****A****C****A****G****A****G****G**GGGAAAG | |
|  |  | AACY01596656.1/750-597  | UUAUU...**U****U**.**A**.**A**.**A**.**C****G****G****G****A****G**A**G**.A**C****U****A****C****U**...........CAA-.........................................-**A****G****U****A****G**..**C****G**.**C****C**GAA**G****G****A****G**CA...A..**A****U**A...............CCCA........................**A****U**.AAU**C****U****C****U**CA**G****G****C**..........................................................................A.A.A.A.**G**.**G****A**.**C****C****G**..**U**..**A****C**.-.-.....GUAUUAA-............................................................-**C****U****C****U****G****G****A****A**A**G**-.**A**.**G****A****U**--...............................UAAG.......................................---**U****U****C**..**U****C****G****C****C**GAA**G****G****A****G**CAA..---..-.----..........----..........................................................................................----..-.---.-.-AA.**C****U****C**.**U**CA**G****G****C**..............AACA**A**.**G****A****C****A****G****A****G****G**GGUUUAA | |
|  |  | AACY01621280.1/508-662  | ACCAA...**U**-.**A**.**U**.**A**.**C****G****G****G****A****G**A**G**.A**C****U****A****C****G**...........UAUU.........................................-**U****G****U****A****G**..**C****G**.**C****C**GAA**G****G****A****G**CA...A..**C****C**A...............CCCC........................**G****G**.AAA**C****U****C****U**CA**G****G****C**..........................................................................A.A.A.A.**G**.**G****A**.**C****C****G**..**U**..**A****C**.-.-.....AUAUUAA-............................................................-**C****U****C****U****G****G****A****A**A**G**-.**A**.**G****A**---...............................UAAAU......................................----**U****C**..**U****C****G****C****C**GAA**G****G****A****G**CCAA.---..-.----..........----..........................................................................................----..-.---.-.AAU.**C****U****C**.**U**CA**G****G****C**..............ACAC**C**.**G****A****C****A****G****A****G****G**GGGUUAA | |
|  |  | AACY01477794.1/131-284  | AGUCC...**A****U**.**U**.**A**.**U**.**C****G****G****G****A****G**A**G**.A**C****U****A**--...........AUUA.........................................---**U****A****G**..**C****G**.**C****C**GAA**G****G****A****G**CA...A..**C****C**A...............CCCC........................**G****G**.AAA**C****U****C****U**CA**G****G****C**..........................................................................A.A.A.A.**G**.**G****A**.**C****C****G**..**A**..**U****U**.**A**.**A**A....GCAUAACA............................................................-**C****U****C****U****G****G****A****A**A**G**A.**G**.**A****U**---...............................UAAU.......................................----**U****U**..**C****C****G****C****C**GAA**G****G****A****G**UAA..---..-.----..........----..........................................................................................----..-.---.-.-AA.**C****U****C**.**U**CA**G****G****C**..............AAAA**A**.**U****A****C****A****G****A****U****G**GGGUACG | |
|  |  | AACY01114623.1/638-788  | AUAUU...**U**-.**A**.**U**.**A**.**C****G****G****G****A****G**A**G**.A**C****U****A****C**-...........UAUU.........................................--**G****U****A****G**..**A****G**.**C****C**GAA**G****G****A****G**CA...A..**C****C**A...............CCCC........................**G****G**.AAA**C****U****C****U**CA**G****G****C**..........................................................................A.A.A.A.**G**.**G****A**.**C****C****G**..**U**..**A****C**.-.-.....AUAUUAA-............................................................-**C****U****C****U****G****G****A****A**A**G**A.**G**.**A****A**---...............................UUAU.......................................----**U****C**..**U****C****G****C****C**GAA**G****G****A****G**CAA..---..-.----..........----..........................................................................................----..-.---.-.-AU.**C****U****C**.**U**CA**G****G****C**..............ACCA**A**.**G****A****C****A****G****A****G****G**GGGCAAA | |
|  |  | AACY01640817.1/384-541  | CUAAU...**U****G**.**C**.**A**.**A**.**C****G****G****G****A****G**A**G**.A**C****U****A****A**-...........UUUAU........................................U-**U****U****G****G**..**C****G**.**C****C**GAA**G****G****A****G**CA...A..**C****U**A...............CCC-.......................U**A****G**.AAA**C****U****C****U**CA**G****G****C**..........................................................................A.A.A.A.**G**.**G****A**.**C****C****G**..**U**..**U****G**.**U**.**A**.....GUAAUAA-............................................................-**C****U****C****U****G****G****A****A**A**G**A.**A**.**G****G****C**--...............................GAUU.......................................---**G****C****C**..**U****C****A****C****C**GAA**G****G****A****U**UAA..---..-.----..........----..........................................................................................----..-.---.-.-AU.**C****U****C**.**U**CA**G****G****U**..............ACAU**G**.**G****A****C****A****G****A****U****A**GGGAGUU | |
|  |  | AACY01347600.1/353-201  | UUGUU...**U****U**.**U**.**A**.-.-**G****G****G****A****G**A**G**.A**C****U****A****C**-...........UUAA.........................................--**G****U****A****G**..**C****G**.**C****C**GAA**G****G****A****G**CA...A..**C****C**G...............ACCC........................**G****G**.AAA**C****U****C****U**CA**G****G****C**..........................................................................A.A.A.A.**G**.**G****A**.**C****C**-..**U**..**U****G**.**A**.**A**.....CAUAUUAA............................................................-**C****U****C****U****G****G****A****A**A**G**A.**A**.**G****A****C**--...............................-UAA.......................................---**G****U****C**..**U****C****A****C****C**GAA**G****G****G****G**UAA..---..-.----..........----..........................................................................................----..-.---.-.-AU.**C****U****C**.**U**CA**G****G****U**..............AAAA**A**.**G****A****C****A****G****A****U****U**GGGUCAA | |
|  |  | AACY01341716.1/458-611  | AAUUA...**A****U**.**C**.**A**.**U**.**C****G****G****G****A****G**A**G**.A**C****U****A**--...........UUUA.........................................---**U****A****G**..**C****G**.**C****C**GAA**G****G****A****G**CA...A..**C****C**A...............CCCC........................**G****G**.AAA**C****U****C****U**CA**G****G****C**..........................................................................A.A.A.A.**G**.**G****A**.**C****C****G**..**A**..**U****U**.**A**.**A**A....GCAUAACA............................................................-**C****U****C****U****G****G****A****A**A**G**A.**G**.**A****U**---...............................UAAU.......................................----**U****U**..**C****C****G****C****C**GAA**G****G****A****G**UAA..---..-.----..........----..........................................................................................----..-.---.-.-AA.**C****U****C**.**U**CA**G****G****C**..............AAAA**A**.**U****A****C****A****G****A****U****G**GGGUACG | |
|  |  | AACY01147930.1/180-336  | AUAAU...**U****U**U**A**.**U**.**A**.**C****G****G****G****A****G**A**G**.A**C****U****A****C**-...........UUGAA........................................U-**G****U****A****G**..**C****G**.**C****C**GAA**G****G****A****G**CA...A..**C****C**A...............CCCC........................**G****G**.AAA**C****U****C****U**CA**G****G****C**..........................................................................A.A.A.A.**G**.**G****A**.**C****C****G**..**U**..**A****C**.-.-.....AUAUUAA-............................................................-**C****U****C****U****G****G****A****A**A**G**A.**G**.**A****A**---...............................UUAU.......................................----**U****C**..**U****C****G****C****C**GAA**G****G****A****G**CUAA.---..-.----..........----..........................................................................................----..-.---.-.AAU.**C****U****C**.**U**CA**G****G****C**..............ACAC**C**.**G****A****C****A****G****A****G****G**GGGUUAA | |
|  |  | AACY01195812.1/301-455  | CAAAA...**U****G**.**U**.**A**.**A**.**C****G****G****G****A****G**A**G**.U**C****U****A****U**-...........UUUU.........................................--**A****U****A****G**..**C****G**.**C****C**GAA**G****G****A****G**CA...A..**C****C**A...............CCCC........................**G****G**.AAA**C****U****C****U**CA**G****G****C**..........................................................................A.A.A.A.**G**.**G****A**.**C****C****G**..**U**..**U****A**.**C**.**C**.....AUAAUAA-............................................................-**C****U****C****U****G****G****A****A**A**G**C.**A**.**G****G****C**--...............................-UUU.......................................---**G****C****C**..**U****C****A****C****C**GAA**G****G****A****G**UAA..---..-.----..........----..........................................................................................----..-.---.-.-AU.**C****U****C**.**U**CA**G****G****U**..............UUCA**A**.**A****A****C****A****G****A****U****G**GGGUACA | |
|  |  | AACY01481166.1/639-462  | AGGAA...**U****A**.**U**.**A**.**G**.**C****G****G****A****A****G**A**G**.A**G****U****G****G****C**AGG........UUAAUCAAAUAGCACAUGAUUA.......................U**G****U****C****A****C**..**C****G**.**C****C**GAA**G****G****A****G**CA...A..**C****C**A...............CCUC........................**G****G**.AAA**C****U****C****U**CA**G****G****C**..........................................................................A.A.A.A.**G**.**G****A**.**C****C****G**..**U**..**A****C**.-.-.....AUAAUAA-............................................................-**C****U****C****U****G****G****A****A**A**G**C.**A**.**G****G****C**--...............................GUAG.......................................---**G****C****C**..**U****C****A****C****C**GAA**G****G****A****G**UAA..---..-.----..........----..........................................................................................----..-.---.-.-AU.**C****U****C**.**U**CA**G****G****U**..............AACA**A**.**G****A****C****A****G****A****G****G**GGGUAAG | |
|  |  | AACY01646969.1/699-549  | AUUUA...**C**-.**A**.**U**.**A**.**C****G****G****G****A****G**A**G**.A**C****U****A****C**-...........UUUU.........................................--**G****U****A****G**..**C****G**.**C****C**GAA**G****G****A****G**CA...A..**C****C**A...............CCCC........................**G****G**.AAA**C****U****C****U**CA**G****G****C**..........................................................................A.A.A.A.**G**.**G****A**.**C****C****G**..**U**..**A****C**.-.-.....AUAUUAA-............................................................-**C****U****C****U****G****G****A****A**A**G**A.**G**.**A****A**---...............................CUAU.......................................----**U****C**..**U****C****G****C****C**GAC**G****G****A****G**CAA..---..-.----..........----..........................................................................................----..-.---.-.-AU.**C****U****C**.**U**CA**G****G****C**..............ACCA**A**.**G****A****C****A****G****A****G****G**GGGCACA | |
|  |  | AACY01185575.1/801-649  | AUUUU...**U****U**.**U**.**A**.**A**.**C****G****G****G****A****G**A**G**.A**C****U****A****C**-...........AUUA.........................................A-**G****U****A****G**..**C****G**.**C****C**GAA**G****G****A****G**CA...A..**C****U**A...............CCCC........................**G****G**.AAA**C****U****C****U**CA**G****G****C**..........................................................................A.A.A.A.**G**.**G****A**.**C****C****G**..**U**..**A****C**.**A**.-.....U-AUUAA-............................................................-**C****U****C****U****G****G****A****A**A**G**A.**G**.**A****A**---...............................UUAU.......................................----**U****C**..**U****C****G****C****C**GAA**G****G****A****G**CAA..---..-.----..........----..........................................................................................----..-.---.-.-AU.**C****U****C**.**U**CA**G****G****C**..............AAAU**A**.**G****A****C****A****G****A****G****G**GGGCAAA | |
|  |  | AACY01714267.1/431-589  | AGCAA...**A****G**.**U**.**A**.**U**.**C****G****G****G****A****G**A**A**.A**C****U****G****A**-...........UUUC.........................................--**U****C****A****G**..**U****G**.**C****C**GAA**G****G****A****G**CA...A..**C****C**A...............CCCC........................**G****G**.AAA**C****U****C****U**CA**G****G****A**..........................................................................A.A.A.A.**G**.**G****A**.**C****C****G**..**U**..**U****A**.**C**.**U**.....UUUUUUUA............................................................-**C****U****C****U****G****G****A****G**A**G**A.**G**.**G****C****C****A**-...............................GAUA.......................................--**U****G****G****U**..**C****C****G****C****C**GAA**G****G****G****A**UAA..---..-.----..........----..........................................................................................----..-.---.-.-UG.**A****U****C**.**U**CA**G****G****C**..............AAAU**G**.**A****A****C****A****G****A****G****G**GGGUGUC | |
|  |  | AACY01075025.1/210-364  | UAAUC...**U****G**.**A**.**U**.**A**.**C****G****G****G****A****G**A**G**.A**C****U****A****C**-...........AUUA.........................................A-**G****U****A****G**..**C****G**.**C****C**GAA**G****G****A****G**CA...A..**C****C**A...............CCCC........................**G****G**.AAA**C****U****C****U**CA**G****G****C**..........................................................................A.A.A.A.**G**.**G****A**.**C****C****G**..**U**..**A****C**.-.-.....AUAUUAA-............................................................-**C****U****C****U****G****G****A****A**A**G**-.**A**.**G****A**---...............................CAAAU......................................----**U****C**..**U****C****G****C****C**GAA**G****G****A****G**CUAA.---..-.----..........----..........................................................................................----..-.---.-.AAU.**C****U****C**.**U**CA**G****G****C**..............ACCA**C**.**G****A****C****A****G****A****G****G**GGGUUAG | |
|  |  | AACY01315701.1/491-339  | AUCUA...**U****C**.**A**.**U**.-.**C****G****G****G****A****G**A**G**.A**C****U****A**--...........UGUU.........................................---**U****A****G**..**C****G**.**C****C**GAA**G****G****A****G**CA...A..**C****C**A...............CCCC........................**G****G**.AAA**C****U****C****U**CA**G****G****C**..........................................................................A.A.A.A.**G**.**G****A**.**C****C****G**..**A**..**A****A**.**A**.**A**A....GCAUAACA............................................................-**C****U****C****U****G****G****A****A**A**G**A.**G**.**A****U**---...............................UAAU.......................................----**U****U**..**C****C****G****C****C**GAA**G****G****A****G**CAA..---..-.----..........----..........................................................................................----..-.---.-.-AA.**C****U****C**.**U**CA**G****G****C**..............AAAA**A**.**U****A****C****A****G****A****U****G**GGGAUAC | |
|  |  | AACY01112401.1/822-974  | AUUAA...**U****U**.**A**.**U**.-.**C****G****G****G****A****G**A**G**.A**C****U****A**--...........UUUA.........................................---**U****A****G**..**C****G**.**C****C**GAA**G****G****A****G**CA...A..**C****C**A...............CCCC........................**G****G**.AAA**C****U****C****U**CA**G****G****C**..........................................................................A.A.A.A.**G**.**G****A**.**C****C****G**..**A**..**U****U**.**A**.**A**A....GCAUAACA............................................................-**C****U****C****U****G****G****A****A**A**G**A.**G**.**A****U**---...............................UGAU.......................................----**U****U**..**C****C****G****C****C**GAA**G****G****A****G**UAA..---..-.----..........----..........................................................................................----..-.---.-.-AG.**C****U****C**.**U**CA**G****G****C**..............AAAA**A**.**U****A****C****A****G****A****U****G**GGGUACG | |
|  |  | AACY01098920.1/189-341  | AUUCA...**U****U**.**A**.**U**.-.**C****G****G****G****A****G**A**G**.A**C****U****A**--...........UUAA.........................................---**U****A****G**..**C****G**.**C****C**GAA**G****G****A****G**CA...A..**C****C**A...............CCCC........................**G****G**.AAA**C****U****C****U**CA**G****G****C**..........................................................................A.A.A.A.**G**.**G****A**.**C****C****G**..**A**..**U****U**.**A**.**A**A....GCAUAACA............................................................-**C****U****C****U****G****G****A****A**A**G**A.**G**.**A****U**---...............................UAAU.......................................----**U****U**..**C****C****G****C****C**GAA**G****G****A****G**UAA..---..-.----..........----..........................................................................................----..-.---.-.-AG.**C****U****C**.**U**CA**G****G****C**..............AAAA**A**.**U****A****C****A****G****A****U****G**GGGUAAG | |
|  |  | AACY01364955.1/524-683  | AAAUC...**C****A**.**U**G**A**.**U**.**C****G****G****G****A****G**A**G**.A**C****U****A****A****G**C..........AUUU.........................................-**U****U****U****A****G**..**C****G**.**C****C**GAA**G****G****A****G**CA...A..**C****A**A...............CCCC........................**G****G**.AAA**C****U****C****U**CA**G****G****C**..........................................................................A.A.A.A.**G**.**G****A**.**C****C****G**..**A**..**U****G**.**U**.**G**G....GCAUAACA............................................................-**C****U****C****U****G****G****A****A**A**G**A.**G**.**A****U**---...............................UAAA.......................................----**U****U**..**C****C****G****C****C**GAA**G****G****A****G**CAA..---..-.----..........----..........................................................................................----..-.---.-.-AA.**C****U****C**.**U**CA**G****G****C**..............AAAA**A**.**U****A****C****A****G****A****U****G**GGGUUAU | |
|  |  | AACY01568116.1/70-224  | GUUUU...**A****U**.**U**.**A**.**U**.**C****G****G****G****A****G**A**G**.A**C****U****A**--U..........CUUU.........................................---**U****A****G**..**C****G**.**C****C**GAA**G****G****A****G**CA...A..**C****C**A...............CCCC........................**G****G**.AAA**C****U****C****U**CA**G****G****C**..........................................................................A.A.A.A.**G**.**G****A**.**C****C****G**..**A**..**U****C**.**G**.**A**A....GCAUAACA............................................................-**C****U****C****U****G****G****A****A**A**G**A.**G**.**A****U**---...............................UAAU.......................................----**U****U**..**C****C****G****C****C**GAA**G****G****A****G**CAA..---..-.----..........----..........................................................................................----..-.---.-.-AA.**C****U****C**.**U**CA**G****G****C**..............AAAA**A**.**U****A****C****A****G****A****U****G**GGGUACG | |
|  |  | AACY01065101.1/1031-1183  | AUCCA...**U****U**.**A**.**U**.-.**C****G****G****G****A****G**A**G**.A**C****U****A**--...........AUUA.........................................---**U****A****G**..**C****G**.**C****C**GAA**G****G****A****G**CA...A..**C****C**A...............CCCC........................**G****G**.AAA**C****U****C****U**CA**G****G****C**..........................................................................A.A.A.A.**G**.**G****A**.**C****C****G**..**A**..**U****U**.**A**.**A**A....GCAUAACA............................................................-**C****U****C****U****G****G****A****A**A**G**A.**G**.**A****U**---...............................UGAU.......................................----**U****U**..**C****C****G****C****C**GAA**G****G****A****G**UAA..---..-.----..........----..........................................................................................----..-.---.-.-AG.**C****U****C**.**U**CA**G****G****C**..............AAAA**A**.**U****A****C****A****G****A****U****G**GGGUACG | |
|  |  | AACY01327614.1/773-615  | UAACC...**A****A**.**U**.**A**.**U**.**C****G****G****G****A****G**A**A**.-**A****C****U****A**-...........UUAA.........................................--**U****A****G****U**..**U****G**.**C****C**GAA**G****G****A****G**CA...A..**C****C**G...............CCCC........................**G****G**.AAA**C****U****C****U**CA**G****G****C**..........................................................................U.A.A.A.**G**.**G****A**.**C****C****G**..**G**..**U****A**.**U**.**A**.....GACUGAAC............................................................**A****C****U****C****U****G****G****A****G**A**G**C.**G**.**A****C****U****U**-...............................UAAA.......................................--**U****A****G****U**..**C****C****G****C****C**GAA**G****G****G****A**UAA..---..-.----..........----..........................................................................................----..-.---.-.-CG.**A****U****C**.**U**CA**G****G****C**..............AAAU**G**.**A****A****C****A****G****A****G****G**GGGCAUU | |
|  |  | AACY01176347.1/846-695  | AUAAA...--.-.-.-.**C****G****G****G****A****G**A**G**.A**C****U****A****C****A**...........AUU-.........................................-**U****G****U****A****G**..**C****G**.**C****C**GAA**G****G****A****G**CA...A..**C****C**A...............CCCC........................**G****G**.AAA**C****U****C****U**CA**G****G****C**..........................................................................A.A.A.A.**G**.**G****A**.**C****C****G**..**U**..--.**A**.**C**.....AUAUUAA-............................................................-**C****U****C****U****G****G****A****A**A**G**A.**G**.**G****A**---...............................CAAG.......................................A---**U****C**..**U****C****G****C****C**GAA**G****G****A****G**CUAA.---..-.----..........----..........................................................................................----..-.---.-.AAU.**C****U****C**.**U**CA**G****G****C**.............ACCAA**A**.**G****A****C****A****G****A****G****G**GGGCGAU | |
|  |  | AACY01122287.1/757-909  | AUAAA...**U**-.-.**A**.**A**.**C****G****G****G****A****G**A**U**.A**C****U****G****A****U**...........AAA-.........................................-**A****U****C****A****G**..**A****G**.**C****C**GAA**G****G****A****G**CA...A..**C****G**................ACCC........................**G****G**.AAA**C****U****C****U**CA**G****G****C**..........................................................................A.A.A.U.**G**.**G****A**.**C****C****G**..**U**..**U**-.**U**.-.....UAUUAAAA............................................................-**C****U****C****U****G****G****A****A**A--.**A**.**G****A****G****C**-...............................UGUG.......................................--**G****C****U****C**..**U**-**A****C****C**GAA**G****G****U****G**UAA..---..-.----..........----..........................................................................................----..-.---.-.AAA.**C****U****C**.**U**CA**G****G****U**..............AAAA**A**.**G****A****C****A****G****A****G****G**GGUAGCU | |
|  |  | AACY01117553.1/743-895  | AUCA-...**A****U**.**C**.**A**.**U**.**C****G****G****G****A****G**A**G**.A**C****U****A**--...........UUUA.........................................---**U****A****G**..**C****G**.**C****C**GAA**G****G****A****G**CA...A..**C****C**A...............CCCC........................**G****G**.AAA**C****U****C****U**CA**G****G****C**..........................................................................A.A.A.A.**G**.**G****A**.**C****C****G**..**A**..**U****U**.**A**.**A**A....GCAUAACA............................................................-**C****U****C****U****G****G****A****A**A**G**U.**G**.**A****U**---...............................UAAU.......................................----**U****U**..**C****C****G****C****C**GAA**G****G****A****G**UAA..---..-.----..........----..........................................................................................----..-.---.-.-AG.**C****U****C**.**U**CA**G****G****C**..............AAAA**A**.**U****A****C****A****G****A****U****G**GGGUACG | |
|  |  | AACY01739989.1/249-410  | ACUUA...**U****A**.**U**.**A**.**U**.**C****G****G****G****A****G**A**C**.A**A****U****G****C**-...........AUAC.........................................--**G****C****A****C**..**G****G**.**C****C**GAA**G****G****A****G**CA...A..**C****U**G...............CCUC........................**G****G**.AAA**C****U****C****U**CA**G****G****C**..........................................................................A.A.A.A.**G**.**G****A**.**C****C****G**..**G**..**U****A**.**U**.**A**AAA..UUACAAA-............................................................-**C****U****C****U****G****G****A****G**A**G**A.**U**.**G****U****C**--...............................UUAUAU.....................................G--**G****A****C**..**A****C****G****C****C**GAA**G****G****G****A**UAA..---..-.----..........----..........................................................................................----..-.---.-.-CA.**A****U****C**.**U**CA**G****G****C**..............AAAU**G**.**A****A****C****A****G****A****G****G**GGGCGUU | |
|  |  | AACY01062054.1/247-95  | UAACC...**U****A**.**A**.**U**.**A**.**C****G****G****G****A****G**A**G**.A**C****C****A****A****U**...........UUA-.........................................-**A****U****U****G****G**..**A****G**.**C****C**GAA**G****G****A****G**AA...A..**C****C**A...............CCCC........................**G****G**.AAA**C****U****C****U**CA**G****G****C**..........................................................................A.A.A.A.**G**.**G****A**.**C****C****G**..**U**..**A****C**.-.-.....AUAUUAA-............................................................-**C****U****C****U****G****G****A****A**A**G**A.**G**.**A****A**---...............................UUAU.......................................----**U****C**..**U****C****G****C****C**GAA**G****G****A****G**CAA..---..-.----..........----..........................................................................................----..-.---.-.-AU.**C****U****C**.**U**CA**G****G****C**..............ACCA**A**.**G****A****C****A****G****A****G****G**GGGCACA | |
|  |  | AACY01058057.1/446-598  | AUUCA...**U****U**.**A**.**U**.-.**C****G****G****G****A****G**A**G**.A**C****U****A**--...........UUUA.........................................---**U****A****G**..**C****G**.**C****C**GAA**G****G****A****G**CA...A..**C****C**A...............CCCC........................**G****G**.AAA**C****U****C****U**CA**G****G****C**..........................................................................A.A.A.A.**G**.**G****A**.**C****C****G**..**A**..**U****U**.**A**.**A**A....GCAUAACA............................................................-**C****U****C****U****G****G****A****A**A**G**A.**G**.**A****U**---...............................UGAU.......................................----**U****U**..**C****C****G****C****C**GAA**G****G****A****G**UAA..---..-.----..........----..........................................................................................----..-.---.-.-AG.**C****U****C**.**U**CA**G****G****C**..............AAAA**A**.**U****A****C****A****G****A****U****G**GGGUACG | |
|  |  | AACY01077499.1/425-577  | AUUCA...**U****U**.**A**.**U**.-.**C****G****G****G****A****G**A**G**.A**C****U****A**--...........UUUA.........................................---**U****A****G**..**C****G**.**C****C**GAA**G****G****A****G**CA...A..**C****C**A...............CCCC........................**G****G**.AAA**C****U****C****U**CA**G****G****C**..........................................................................A.A.A.A.**G**.**G****A**.**C****C****G**..**A**..**U****U**.**A**.**A**A....GCAUAACA............................................................-**C****U****C****U****G****G****A****A**A**G**A.**G**.**A****U**---...............................UGAU.......................................----**U****U**..**C****C****G****C****C**GAA**G****G****A****G**UAA..---..-.----..........----..........................................................................................----..-.---.-.-AG.**C****U****C**.**U**CA**G****G****C**..............AAAA**A**.**U****A****C****A****G****A****U****G**GGGUACG | |
|  |  | AACY01552849.1/475-627  | AUUCA...**U****U**.**A**.**U**.-.**C****G****G****G****A****G**A**G**.A**C****U****A**--...........UUUA.........................................---**U****A****G**..**C****G**.**C****C**GAA**G****G****A****G**CA...A..**C****C**A...............CCCC........................**G****G**.AAA**C****U****C****U**CA**G****G****C**..........................................................................A.A.A.A.**G**.**G****A**.**C****C****G**..**A**..**U****U**.**A**.**A**A....GCAUAACA............................................................-**C****U****C****U****G****G****A****A**A**G**A.**G**.**A****U**---...............................UGAU.......................................----**U****U**..**C****C****G****C****C**GAA**G****G****A****G**UAA..---..-.----..........----..........................................................................................----..-.---.-.-AG.**C****U****C**.**U**CA**G****G****C**..............AAAA**A**.**U****A****C****A****G****A****U****G**GGGUACG | |
|  |  | AACY01709626.1/266-418  | AUUCA...**U****U**.**A**.**U**.-.**C****G****G****G****A****G**A**G**.A**C****U****A**--...........UUUA.........................................---**U****A****G**..**C****G**.**C****C**GAA**G****G****A****G**CA...A..**C****C**A...............CCCC........................**G****G**.AAA**C****U****C****U**CA**G****G****C**..........................................................................A.A.A.A.**G**.**G****A**.**C****C****G**..**A**..**U****U**.**A**.**A**A....GCAUAACA............................................................-**C****U****C****U****G****G****A****A**A**G**A.**G**.**A****U**---...............................UGAU.......................................----**U****U**..**C****C****G****C****C**GAA**G****G****A****G**UAA..---..-.----..........----..........................................................................................----..-.---.-.-AG.**C****U****C**.**U**CA**G****G****C**..............AAAA**A**.**U****A****C****A****G****A****U****G**GGGUACG | |
|  |  | AACY01719516.1/275-432  | AUUCA...**U****G**.-.**A**.**U**.**C****G****G****G****A****G**A**G**.A**C****U****A****A****G**C..........AAUU.........................................-**U****U****U****A****G**..**C****G**.**C****C**GAA**G****G****A****G**CA...A..**C****C**A...............CCCC........................**G****G**.AAA**C****U****C****U**CA**G****G****C**..........................................................................A.A.A.A.**G**.**G****A**.**C****C****G**..**A**..**U****A**.**U**.**A**G....GCAUAACA............................................................-**C****U****C****U****G****G****A****A**A**G**A.**G**.**A****U**---...............................UAAA.......................................----**U****U**..**C****C****G****C****C**GAA**G****G****A****G**CAA..---..-.----..........----..........................................................................................----..-.---.-.-AA.**C****U****C**.**U**CA**G****G****C**..............AAAU**A**.**U****A****C****A****G****A****U****G**GGGUCAC | |
|  |  | AACY01025341.1/474-317  | ACAAA...**U****G**.**U**.**A**.**A**.**C****G****G****A****A****G**A**G**.A**C****U****G****U**-...........UUUAA........................................U-**U****C****A****G**..**C****G**.**C****C**GAA**G****G****A****G**CA...A..**C****U**U...............CCC-.......................U**A****G**.AAC**C****U****C****U**CA**G****G****C**..........................................................................A.A.A.A.**G**.**G****A**.**C****C****G**..**U**..**U****G**.**C**.**A**.....AUAAUAA-............................................................-**C****U****C****U****G****G****A****A**A**G**U.**A**.**G****G****C**--...............................GAUA.......................................---**G****C****C**..**U****C****A****C****C**GAA**G****G****A****U**UAA..---..-.----..........----..........................................................................................----..-.---.-.-AU.**C****U****C**.**U**CA**G****G****U**..............ACAU**G**.**G****A****C****A****G****A****U****A**GGGGUUG | |
|  |  | AACY01044810.1/303-147  | CUAAA...**U****G**.**U**.**A**.**A**.**C****G****G****G****A****G**A**G**.A**C****U****A****A**-...........UUUA.........................................U-**U****U****A****G**..**C****G**.**C****C**GAA**G****G****A****G**CA...A..**C****U**A...............CCC-.......................U**A****G**.AAA**C****U****C****U**CA**G****G****C**..........................................................................A.A.A.A.**G**.**G****A**.**C****C****G**..**U**..**U****G**.**C**.**C**.....GUAAUAA-............................................................-**C****U****C****U****G****G****A****A**A**G**U.**A**.**G****G****C**--...............................UUAA.......................................---**G****C****C**..**U****C****A****C****C**GAA**G****G****A****U**UAA..---..-.----..........----..........................................................................................----..-.---.-.-AU.**C****U****C**.**U**CA**G****G****U**..............ACAU**A**.**G****A****C****A****G****G****U****A**GGGUUAG | |
|  |  | AACY01686769.1/656-505  | UUCAA...**U**-.-.**A**.**U**.**C****G****G****G****A****G**A**G**.A**C****U****A**--...........UUUA.........................................---**U****A****G**..**C****G**.**C****C**GAA**G****G****A****G**CA...A..**C****C**A...............CCCC........................**G****G**.AAA**C****U****C****U**CA**G****G****C**..........................................................................A.A.A.A.**G**.**G****A**.**C****C****G**..**A**..**U****U**.**A**G**A**.....GCAUAACA............................................................-**C****U****C****U****G****G****A****A**A**G**A.**G**.**A****U**---...............................UGAU.......................................----**U****U**..**C****C****G****C****C**GAA**G****G****A****G**UAA..---..-.----..........----..........................................................................................----..-.---.-.-AA.**C****U****C**.**U**CA**G****G****C**..............AAAA**A**.**U****A****C****A****G****A****U****G**GGGUACG | |
|  |  | AACY01008861.1/426-271  | AAAAG...**U****A**.**A**.**U**.**A**.**C****G****G****G****A****G**A**G**.A**C****U****A****C**-...........UUAGC........................................U-**G****U****A****G**..**C****G**.**C****C**GAA**G****G****A****G**CA...A..**C****U**A...............CCCC........................**G****G**.AAA**C****U****C****U**CA**G****G****C**..........................................................................A.A.A.A.**G**.**G****A**.**C****C****G**..**U**..**A****C**.-.-.....AUAUUAA-............................................................-**C****U****C****U****G****G****A****A**A**G**-.**A**.**G****A**---...............................UAAAU......................................----**U****C**..**U****C****G****C****C**GAA**G****G****A****G**CCAA.---..-.----..........----..........................................................................................----..-.---.-.AAU.**C****U****C**.**U**CA**G****G****C**..............ACAC**C**.**G****A****C****A****G****A****G****G**GGGUUGA | |
|  |  | AACY01469098.1/24-179  | CCUCU...**U****G**.**U**.**A**.**A**.**C****G****G****G****A****G**A**G**.U**C****U****A****U****U**...........UAA-.........................................-**A****A****U****A****G**..**C****G**.**C****C**GAA**G****G****A****G**CA...A..**C****C**A...............CCCC........................**G****G**.AAA**C****U****C****U**CA**G****G****C**..........................................................................A.A.A.A.**G**.**G****A**.**C****C****G**..**U**..**U****G**.**C**.**C**.....GUAAUAA-............................................................-**C****U****C****U****G****G****A****A**A**G**C.**A**.**G****G****C**--...............................-UUU.......................................---**G****C****C**..**U****C****A****C****C**GAA**G****G****A****G**UAA..---..-.----..........----..........................................................................................----..-.---.-.-AU.**C****U****C**.**U**CA**G****G****U**..............UUCA**A**.**A****A****C****A****G****A****U****G**GGGUACA | |
|  |  | AACY01537465.1/152-1  | AUAAAUUU**A****U**.**U**.**U**.**A**.**C****G****G****G****A****G**A**G**.A**C****U****A****C**-...........UUUU.........................................--**G****U****A****G**..**C****G**.**C****C**GAA**G****G****A****G**CA...A..**C****C**A...............CCCC........................**G****G**.AAA**C****U****C****U**CA**G****G****C**..........................................................................A.A.A.A.**G**.**G****A**.**C****C****G**..**U**..**A****C**.**A**.**U**.....A---UUAA............................................................-**C****U****C****U****G****G****A****A**A**G**A.**G**.**A****A**---...............................UUAU.......................................----**U****C**..**U****C****G****C****C**GAA**G****G****A****G**CAA..---..-.----..........----..........................................................................................----..-.---.-.-AU.**C****U****C**.**U**CA**G****G****C**..............ACCA**A**.**G****A****C****A****G****A****G****G**GGGC--- | |
|  |  | AACY01110200.1/46-202  | AUUAU...**U****C**.**A**.**A**.**A**.**C****G****G****G****A****G**A**G**.A**C****U****A****U****U**...........UAGA.........................................-**U****A****U****A****G**..**C****G**.**C****C**GAA**G****G****A****G**CA...A..**C****C**A...............CCCC........................**G****G**.AAA**C****U****C****U**CA**G****G****C**..........................................................................A.A.A.A.**G**.**G****A**.**C****C****G**..**U**..**A****C**.**A**.**A**.....AUAAAAUU............................................................-**C****U****C****U****G****G****A****A**A**G**-.**A**.**G****A****U**--...............................-AAG.......................................---**A****U****C**..**U****C****A****C****C**GAA**G****G****A****G**UAA..---..-.----..........----..........................................................................................----..-.---.-.-AU.**C****U****C**.**U**CA**G****G****U**..............ACCA**A**.**U****A****C****A****G****A****U****G**GGGUAAA | |
|  |  | AACY01105425.1/860-703  | AUGGU...**C****G**.**U**.**A**.**A**.**C****G****G****A****A****G**A**G**.A**C****U****G****U**-...........UUUAA........................................U-**G****C****A****G**..**C****G**.**C****C**GAA**G****G****A****G**CA...A..**C****U**U...............CCC-.......................U**A****G**.AAA**C****U****C****U**CA**G****G****C**..........................................................................A.A.A.A.**G**.**G****A**.**C****C****G**..**U**..**U****A**.**C**.**A**.....AUAAUAA-............................................................-**C****U****C****U****G****G****A****A**A**G**U.**A**.**G****G****C**--...............................AAUA.......................................---**G****C****C**..**U****C****A****C****C**GAA**G****G****A****U**UAA..---..-.----..........----..........................................................................................----..-.---.-.-AU.**C****U****C**.**U**CA**G****G****U**..............ACAU**G**.**G****A****C****A****G****A****U****A**GGGGUUG | |
|  |  | AACY01163770.1/692-533  | ACUGA...**U****A**.**U**.**A**.**U**.**C****G****G****G****A****G**A**A**.-**A****C****U**--...........AGCAA........................................U--**A****G****U**..**U****G**.**C****C**GAA**G****G****A****G**CA...A..**C****C**G...............CCCC........................**G****G**.AAA**C****U****C****U**CA**G****G****C**.........................................................................AA.A.A.A.**G**.**G****A**.**C****C****G**..**G**..**U****A**.**U**.**A**.....GACUGAAC............................................................**A****C****U****C****U****G****G****A****G**A**G**C.**G**.**A****C****U****U**-...............................UAAA.......................................--**U****A****G****U**..**C****C****G****C****C**GAA**G****G****G****A**UAA..---..-.----..........----..........................................................................................----..-.---.-.-CG.**A****U****C**.**U**CA**G****G****C**..............AAAG**G**.**A****A****C****A****G****A****G****G**GGGUAUU | |
|  |  | AACY01056859.1/1927-2080  | AAUUA...**A****U**.**C**.**A**.**U**.**C****G****G****G****A****G**A**G**.A**C****U****A**--...........UUUA.........................................---**U****A****G**..**C****G**.**C****C**GAA**G****G****A****G**CA...A..**C****C**A...............CCCC........................**G****G**.AAA**C****U****C****U**CA**G****G****C**..........................................................................A.A.A.A.**G**.**G****A**.**C****C****G**..**A**..**U****U**.**A**.**A**A....GCAUAACA............................................................-**C****U****C****U****G****G****A****A**A**G**A.**G**.**A****U**---...............................UAAU.......................................----**U****U**..**C****C****G****C****C**GAA**G****G****A****G**UAA..---..-.----..........----..........................................................................................----..-.---.-.-AG.**C****U****C**.**U**CA**G****G****C**..............AAAU**A**.**U****A****C****A****G****A****U****G**GGGUACG | |
|  |  | AACY01072900.1/236-80  | AUUAA...**U****U**.**C**.**U**.**G**.**C****G****G****A****A****G**A**G**.A**C****U****A****C**-...........UUCAA........................................U-**G****U****A****G**..**A****G**.**C****C**GAA**G****G****A****G**CA...A..**C****C**A...............CCCC........................**G****G**.AAU**C****U****C****U**CA**G****G****C**..........................................................................A.A.A.A.**G**.**G****A**.**C****C****G**..**C**..**G****C**.**A**.-.....U-AUUAA-............................................................-**C****U****C****U****G****G****A****A**A-A.**A**.**G****A****A****C**-...............................AGAA.......................................--**G****U****U****C**..**U****U****G****C****C**GAA**G****G****A****G**CAA..---..-.----..........----..........................................................................................----..-.---.-.-AA.**C****U****C**.**U**CA**G****G****C**..............CAAU**A**.**G****A****C****A****G****A****G****G**GGGCGAA | |
|  |  | AACY01409873.1/765-608  | AGAUU...**G****U**.**U**.**U**A**A**.**A****G****G****G****A****G**A**G**.A**C****U****A****C**-...........UUUA.........................................G-**G****U****G****G**..**C****A**.**C****C**GAA**G****G****A****G**CA...A..**C****C**G...............ACCC........................**G****G**.AAA**C****U****C****U**CA**G****G****U**..........................................................................A.A.A.A.**G**.**G****A**.**C****C****U**..**U**..**A****A**.**A**.**C**.....AUAUUAA-............................................................-**C****U****C****U****G****G****A****A**A**G**A.**A**.**G****A****C**--...............................-UGG.......................................---**G****U****C**..**U****C****A****C****C**GAA**G****G****G****G**UAA..---..-.----..........----..........................................................................................----..-.---.-.-AU.**C****U****C**.**U**CA**G****G****U**.............AAAAA**A**.**G****A****C****A****G****A****U****A**GGGUCAA | |
|  |  | AACY01518951.1/288-442  | AUUAU...**C****U**.**A**.**U**.**A**.**C****G****G****A****A****G**A**G**.A**C****U****A****C****U**U..........AAGA.........................................-**U****G****U****A****G**..**C****G**.**C****C**GAA**G****G****A****G**CA...A..**C****C**A...............CCCC........................**G****G**.AAA**C****U****C****U**CA**G****G****C**..........................................................................A.A.A.A.**G**.**G****A**.**C****C****G**..**U**..**A****C**.**A**.-.....U-AUUAA-............................................................-**C****U****C****U****G****G****A****A**A**G**A.**G**.**A****A**---...............................UAAU.......................................----**U****C**..**U****C****G****C****C**GAA**G****G****A****G**CAA..---..-.----..........----..........................................................................................----..-.---.-.-AU.**C****U****C**.**U**CA**G****G****C**..............ACAU**A**.**A****A****C****A****G****A****G****G**GGGUUAG | |
|  |  | AACY01030086.1/717-871  | AAAUA...**G****C**.**U**.**A**.**A**.**C****G****G****G****A****G**A**U**.A**C****U****G****A**-...........AAUA.........................................--**U****C****A****G**..**A****G**.**C****C**GAA**G****G****A****G**CA...A..**C****G**................ACCC........................**G****G**.AAA**C****U****C****U**CA**G****G****C**..........................................................................A.A.A.AU**G**.**G****A**.**C****C****G**..**U**..**U****A**.-.-.....AAAAAAAA............................................................-**A****U****C****U****G****G****A****A**A--.**A**.**G****A****G****C**-...............................AUUA.......................................--**G****C****U****C**..**U**-**A****C****C**GAA**G****G****U****G**UAA..---..-.----..........----..........................................................................................----..-.---.-.AAA.**C****U****C**.**U**CA**G****G****U**..............AAAA**A**.**G****A****C****A****G****A****G****G**GGUAACA | |
|  |  | AACY01068347.1/1238-1390  | UUCUU...**U****G**.**A**.**U**.**A**.**C****G****G****G****A****G**A**G**.A**C****U****A****C**-...........AUA-.........................................--**G****U****A****G**..**C****G**.**C****C**GAA**G****G****A****G**CA...A..**A****A**A...............CCCC........................**A****G**.AAA**C****U****C****U**CA**G****G****C**..........................................................................A.A.A.A.**G**.**G****A**.**C****C****G**..**U**..**A****C**.-.-.....GUAUUAA-............................................................-**C****U****C****U****G****G****A****A**A**G**-.**A**.**G****A**---...............................CAAAU......................................----**U****C**..**U****C****G****C****C**GAA**G****G****A****G**CUAA.---..-.----..........----..........................................................................................----..-.---.-.AAU.**C****U****C**.**U**CA**G****G****C**..............ACCA**C**.**G****A****C****A****G****A****G****G**GGGUUAG | |
|  |  | AACY01428461.1/130-307  | UUGAU...**U****G**.**U**.**G**.**U**.**C****A****G****G****A****G**A**G**.A**G****U****C**--...........GAAA.........................................---**G****A****C**..**C****G**.**C****C**GAA**G****G****C****U**GA...A..--................----........................--.---**C****G****C****U**CA**G****G****C**..........................................................................A.A.A.A.**G**.**U****A**.**C****U****G**..**A**..**C****A**.**U**.**U**.....UUAUAAUU............................................................**C****U****G****U****U****G****G****A****G**A**G**U.**G**.**A****C****G****A****C**C..............................CAAAAGG....................................U**G****G****C****G****U**..**C****C****A****C****C**GAA**G****G****G****G**CAA..**A****C****C**..**C**.**C****G****A****G**CCG.......AUGC..........................................................................................**U****C****A****C**..**G**.**G****G****U**.G.AAU.**C****U****C**.**U**CA**G****G****U**..............CCAA**G**.**G****A****C****A****A****C****A****G**GGUUCCC | |
|  |  | AACY01551906.1/519-363  | AUUAA...**A****G**.**A**.**U**.**G**.**C****G****G****A****A****G**A**G**.A**C****U****A****C**-...........UUUAA........................................U-**G****U****A****G**..**A****G**.**C****C**GAA**G****G****A****G**CA...A..**C****C**A...............CCCC........................**G****G**.AAU**C****U****C****U**CA**G****G****C**..........................................................................A.A.A.A.**G**.**G****A**.**C****C****G**..**C**..**G****C**.**A**.**U**.....A---UUAA............................................................-**C****U****C****U****G****G****A****A**A-A.**A**.**G****A****A****C**-...............................GUUU.......................................--**G****U****U****C**..**U****U****G****C****C**GAA**G****G****A****G**CAA..---..-.----..........----..........................................................................................----..-.---.-.-AA.**C****U****C**.**U**CA**G****G****C**..............CAAU**A**.**G****A****C****A****G****A****G****G**GGGCAUA | |
|  |  | AACY01062287.1/1234-1392  | UAUUC...**U****G**.**C**.**U**.**A**.**C****G****G****G****A****G**A**G**.A**C****U****A****C****A**G..........AAUA.........................................U**C****G****U****A****G**..**C****G**.**C****C**GAA**G****G****A****G**CA...A..**C****C**A...............CCCA........................**G****G**.AAU**C****U****C****U**CA**G****G****C**.........................................................................AA.A.A.A.**G**.**G****A**.**C****C****G**..**U**..**A****A**.**C**.-.....AUAUUAA-............................................................-**C****U****C****U****G****G****A****A**A**G**-.**A**.**G****A****U**--...............................UUAA.......................................---**U****U****C**..**U****C****G****C****C**GAA**G****G****A****G**CAA..---..-.----..........----..........................................................................................----..-.---.-.-AA.**C****U****C**.**U**CA**G****G****C**..............AAAU**A**.**U****A****C****A****G****A****U****G**GGUACAA | |
|  |  | AACY01119960.1/1161-1005  | UUGAC...--.**U**.**U**.**A**.**C****G****G****G****A****G**A**G**.A**C****U****A****C****A**G..........AUUA.........................................U**C****G****U****A****G**..**C****G**.**C****C**GAA**G****G****A****G**CA...A..**C****C**A...............CCCA........................**G****G**.AAU**C****U****C****U**CA**G****G****C**.........................................................................AA.A.A.A.**G**.**G****A**.**C****C****G**..**U**..**A****A**.**C**.-.....AUAUUAA-............................................................-**C****U****C****U****G****G****A****A**A**G**-.**A**.**G****A****U**--...............................UAAA.......................................---**U****U****C**..**U****C****G****C****C**GAA**G****G****A****G**CAA..---..-.----..........----..........................................................................................----..-.---.-.-AA.**C****U****C**.**U**CA**G****G****C**..............AAAU**A**.**U****A****C****A****G****A****U****G**GGUAUUA | |
|  |  | AACY01034051.1/741-891  | AGAUA...**U****U**G**A**.**A**.**A**.**C****G****G****G****A****G**A**G**.A**C****U****A****C**-...........UAAA.........................................U-**G****U****A****G**..**C****G**.**C****C**GAA**G****G****A****G**CA...A..**C****C**A...............CCCC........................**G****G**.AAA**C****U****C****U**CA**G****G****C**..........................................................................A.A.A.A.**G**.**G****A**.**C****C****G**..**U**..**A****U**.-.-.....AUAUUAA-............................................................-**C****U****C****U****G****G****A****A**A**G**A.**A**.-**C**---...............................UUUC.......................................------..**U****C****G****C****C**GAA**G****G****A****G**UAA..---..-.----..........----..........................................................................................----..-.---.-.-AU.**C****U****C**.**U**CA**G****G****C**..............UCAU**A**.**G****A****C****A****G****A****G****G**GGGUAGU | |
|  |  | AACY01191796.1/303-147  | UUCAA...--.**U**.**U**.**A**.**C****G****G****G****A****G**A**G**.A**C****U****A****C****A**G..........AAUA.........................................U**C****G****U****A****G**..**C****G**.**C****C**GAA**G****G****A****G**CA...A..**C****C**A...............CCCA........................**G****G**.AAU**C****U****C****U**CA**G****G****C**.........................................................................AA.A.A.A.**G**.**G****A**.**C****C****G**..**U**..**A****A**.**C**.-.....AUAUUAA-............................................................-**C****U****C****U****G****G****A****A**A**G**-.**A**.**G****A****U**--...............................UUAG.......................................---**U****U****C**..**U****C****G****C****C**GAA**G****G****A****G**CAA..---..-.----..........----..........................................................................................----..-.---.-.-AA.**C****U****C**.**U**CA**G****G****C**..............AAAU**A**.**U****A****C****A****G****A****U****G**GGUACAA | |
|  |  | AACY01655759.1/420-578  | UAUCU...**C****G**.**U**.**A**.**A**.**C****G****G****G****A****G**A**G**.A**C****U****A****C****A**G..........AAUA.........................................U**C****G****U****A****G**..**C****G**.**C****C**GAA**G****G****A****G**CA...A..**C****C**A...............CCCA........................**G****G**.AAU**C****U****C****U**CA**G****G****C**.........................................................................AA.A.A.A.**G**.**G****A**.**C****C****G**..**U**..**A****A**.**C**.-.....AUAUUAA-............................................................-**C****U****C****U****G****G****A****A**A**G**-.**A**.**G****A****U**--...............................UUAA.......................................---**U****U****C**..**U****C****G****C****C**GAA**G****G****A****G**CAA..---..-.----..........----..........................................................................................----..-.---.-.-AA.**C****U****C**.**U**CA**G****G****C**..............AAAU**A**.**U****A****C****A****G****A****U****G**GGUACAA | |
|  |  | AACY01096262.1/913-757  | AUCUA...**U**-.**U**.**C**.**A**.**C****G****G****G****A****G**A**G**.A**C****U****A****C****A**A..........AUUA.........................................U**U****G****U****A****G**..**C****G**.**C****C**GAA**G****G****A****G**CA...A..**C****C**A...............CCCA........................**G****G**.AAU**C****U****C****U**CA**G****G****C**.........................................................................AA.A.A.A.**G**.**G****A**.**C****C****G**..**U**..**A****C**.-.-.....GUAUUAA-............................................................-**C****U****C****U****G****G****A****A**A**G**-.**A**.**G****A****U**--...............................UAAA.......................................---**U****U****C**..**U****C****G****C****C**GAA**G****G****A****G**CAA..---..-.----..........----..........................................................................................----..-.---.-.-AA.**C****U****C**.**U**CA**G****G****C**..............AAAU**A**.**U****A****C****A****G****A****U****G**GGUAUAA | |
|  |  | AACY01063370.1/600-755  | AAAAA...**G****C**.**A**.**U**.**G**.**C****G****G****A****A****G**A**G**.A**C****U****A****C****U**...........UAGA.........................................-**U****G****U****A****G**..**A****G**.**C****C**GAA**G****G****A****G**CA...A..**C****C**A...............CCCC........................**G****G**.AAU**C****U****C****U**CA**G****G****C**..........................................................................A.A.A.A.**G**.**G****A**.**C****C****G**..**C**..**G****C**.**A**.**U**.....A---UUAA............................................................-**C****U****C****U****G****G****A****A**A-A.**A**.**G****A****A****C**-...............................-UUU.......................................--**G****U****U****C**..**U****U****G****C****C**GAA**G****G****A****G**CAA..---..-.----..........----..........................................................................................----..-.---.-.-AA.**C****U****C**.**U**CA**G****G****C**..............CAAU**A**.**G****A****C****A****G****A****G****G**GGGCAAA | |
|  |  | AACY01091526.1/911-754  | AUUUA...**C**-.**U**.**U**.**A**.**C****G****G****G****A****G**A**G**.A**C****U****A****C****A**G..........AUUA.........................................U**C****G****U****A****G**..**C****G**.**C****C**GAA**G****G****A****G**CA...A..**C****C**G...............CCCA........................**G****G**.AAU**C****U****C****U**CA**G****G****C**.........................................................................AA.A.A.A.**G**.**G****A**.**C****C****G**..**U**..**A****A**.**C**.-.....AUAUUAA-............................................................-**C****U****C****U****G****G****A****A**A**G**-.**A**.**G****A****U**--...............................UUAA.......................................---**U****U****C**..**U****C****G****C****C**GAA**G****G****A****G**CAA..---..-.----..........----..........................................................................................----..-.---.-.-AA.**C****U****C**.**U**CA**G****G****C**..............AAAU**A**.**U****A****C****A****G****A****U****G**GGUAUAA | |
|  |  | AACY01100816.1/95-249  | AAAUA...**A****U**.**U**.**A**.**A**.**C****G****G****G****A****G**A**U**.A**C****U****G****A**-...........AAUU.........................................--**U****C****A****G**..**A****G**.**C****C**GAA**G****G****A****G**CA...A..**C****G**................ACCC........................**G****G**.AAA**C****U****C****U**CA**G****G****C**..........................................................................A.C.A.AU**G**.**G****A**.**C****C****G**..**U**..**U****A**.**A**.-.....AAAAAAA-............................................................-**C****U****C****U****G****G****A****A**A--.**A**.**G****A****G****C****A**...............................UUUA.......................................--**G****C****U****C**..**U**-**A****C****C**GAA**G****G****U****G**AAA..---..-.----..........----..........................................................................................----..-.---.-.-AG.**C****U****C**.**U**CA**G****G****U**..............AAAA**A**.**G****A****C****A****G****A****G****G**GGUAGCU | |
|  |  | AACY01739450.1/320-474  | AAAUA...**A****C**.**U**.**A**.**A**.**C****G****G****G****A****G**A**U**.A**C****U****G****A**-...........AAUU.........................................--**U****C****A****G**..**A****G**.**C****C**GAA**G****G****A****G**CA...A..**C****G**................ACCC........................**G****G**.AAA**C****U****C****U**CA**G****G****C**..........................................................................A.C.A.AU**G**.**G****A**.**C****C****G**..**U**..**U****A**.-.-.....AAAAAAAA............................................................-**C****U****C****U****G****G****A****A**A--.**A**.**G****A****G****C**-...............................AUGU.......................................A-**G****C****U****C**..**U**-**A****C****C**GAA**G****G****U****G**AAA..---..-.----..........----..........................................................................................----..-.---.-.-AG.**C****U****C**.**U**CA**G****G****U**..............AAAA**A**.**G****A****C****A****G****A****G****G**GGUAGCU | |
|  |  | AACY01028155.1/378-220  | UAUUG...**U****U**.**U**.**U**.**A**.**C****G****G****G****A****G**A**G**.A**C****U****A****C****A**G..........AAUA.........................................U**C****G****U****A****G**..**C****A**.**C****C**GAA**G****G****A****G**CA...A..**C****C**A...............CCCA........................**G****G**.AAU**C****U****C****U**CA**G****G****U**.........................................................................AA.A.A.A.**G**.**G****A**.**C****C****G**..**U**..**A****A**.**C**.-.....AUAUUAA-............................................................-**C****U****C****U****G****G****A****A**A**G**-.**A**.**G****A****U**--...............................UUAA.......................................---**U****U****C**..**U****C****G****C****C**GAA**G****G****A****G**CAA..---..-.----..........----..........................................................................................----..-.---.-.-AA.**C****U****C**.**U**CA**G****G****C**..............AAAU**A**.**U****A****C****A****G****A****U****G**GGUACAA | |
|  |  | AACY01039409.1/354-199  | GUUAA...**A****U**.**U**.**U**.**A**.**C****G****G****G****A****G**A**G**.A**C****U****G****U****A**A..........UAG-.........................................-**U****A****C****A****G**..**C****G**.**C****C**GAA**G****G****A****G**CA...A..**C****C**A...............CCCC........................**G****G**.AAA**C****U****C****U**CA**G****G****C**..........................................................................A.A.A.A.**G**.**G****A**.**C****C****G**..**U**..**G****U**.**G**.**U**.....UAAUAUA-............................................................-**U****U****C****U****G****G****A****A**A**G**A.**G**.**A****A**---...............................UGUU.......................................----**U****C**..**U****C****A****C****C**GAA**G****G****A****G**UAA..---..-.----..........----..........................................................................................----..-.---.-.-AU.**C****U****C**.**U**CA**G****G****U**..............GCCA**A**.**U****A****C****A****G****A****A****G**GGGUUUU | |
|  |  | AACY01033800.1/2654-2497  | UAUUU...**C****U**.**U**.**A**.**A**.**C****G****G****G****A****G**A**G**.A**U****C****A****C****A**A..........UUUA.........................................A**C****G****U****G****A**..**C****A**.**C****C**GAA**G****G****A****G**CA...A..**C****C**A...............CCCA........................**G****G**.AAA**C****U****C****U**CA**G****G****U**..........................................................................A.A.A.A.**G**.**G****A**.**C****C****G**..**U**..**A****A**.**C**.-.....AUAUUAA-............................................................-**C****U****C****U****G****G****A****A**A**G**-.**A**.**G****A****U**--...............................UAGA.......................................---**U****U****C**..**U****C****G****C****C**GAA**G****G****A****G**UAA..---..-.----..........----..........................................................................................----..-.---.-.-AA.**C****U****C**.**U**CA**G****G****C**..............AAAU**A**.**U****A****C****A****G****A****U****G**GGUACGA | |
|  |  | AACY01328518.1/764-916  | AUAUU...**U****C**C**U**.**U**.**A**.**C****G****G****G****A****G**A**G**.A**C****U****A****U****U**U..........CUU-.........................................-**A****A****U****A****G**..**C****G**.**C****C**GAA**G****G****A****G**CA...A..**C****C**A...............CCCC........................**G****G**.AAA**C****U****C****U**CA**G****G****C**..........................................................................A.A.A.A.**G**.**G****A**.**C****C****G**..**U**..**A****C**.-.-.....AUAUUAA-............................................................-**C****U****C****U****G****G****A****A**A**G**-.**A**.**G****A**---...............................AUAU.......................................----**U****C**..**U****C****G****C****C**GAA**G****G****A****G**CAA..---..-.----..........----..........................................................................................----..-.---.-.-AU.**C****U****C**.**U**CA**G****G****C**..............-AAU**A**.**G****A****C****A****G****A****A****G**GGGUUUG | |
|  |  | AACY01164513.1/245-89  | UUGAC...--.**U**.**U**.**A**.**C****G****G****G****A****G**A**G**.A**C****U****A****C****A**G..........AUUA.........................................U**C****G****U****A****G**..**C****G**.**C****C**GAA**G****G****A****G**CA...A..**C****U**A...............CCCA........................**G****G**.AAU**C****U****C****U**CA**G****G****C**.........................................................................AA.A.A.A.**G**.**G****A**.**C****C****G**..**U**..**A****A**.**C**.-.....AUAUUAA-............................................................-**C****U****C****U****G****G****A****A**A**G**-.**A**.**G****A****U**--...............................UUAA.......................................---**U****U****C**..**U****C****G****C****C**GAA**G****G****A****G**CAA..---..-.----..........----..........................................................................................----..-.---.-.-AA.**C****U****C**.**U**CA**G****G****C**..............AAAU**A**.**U****A****C****A****G****A****U****G**GGUAUAA | |
|  |  | AACY01628554.1/246-403  | AUUCA...**C**-.**U**.**U**.**A**.**C****G****G****G****A****G**A**G**.A**C****U****A****C****A**G..........AAAA.........................................U**C****G****U****A****G**..**C****G**.**C****C**GAA**G****G****A****G**CA...A..**C****C**A...............CCCA........................**G****G**.AAU**C****U****C****U**CA**G****G****C**.........................................................................AA.A.A.A.**G**.**G****A**.**C****C****G**..**U**..**A****A**.**C**.-.....AUAUUAA-............................................................-**C****U****C****U****G****G****A****A**A**G**-.**A**.**G****A****U**--...............................UUAA.......................................---**U****U****C**..**U****C****G****C****C**GAA**G****G****A****G**CAA..---..-.----..........----..........................................................................................----..-.---.-.-AA.**C****U****C**.**U**CA**G****G****C**..............AAAU**A**.**U****A****C****A****G****A****U****G**GGUACAA | |
|  |  | AACY01755171.1/732-575  | AUACA...**U**-.**U**.**A**.**A**.**C****G****G****G****A****G**A**G**.A**C****U****A****C****A**AA.........CUUA.........................................-**U****G****U****A****G**..**C****G**.**C****C**GAA**G****G****A****G**UA...A..**C****U**A...............CCCA........................**G****G**.AAU**C****U****C****U**CA**G****G****C**.........................................................................AA.A.A.A.**G**.**G****A**.**C****C****G**..**U**..**A****A**.**U**.-.....AUAUUAA-............................................................-**C****U****C****U****G****G****A****A**A**G**-.**A**.**G****A****U**--...............................UAAA.......................................---**U****U****C**..**U****C****G****C****C**GAA**G****G****A****G**CAA..---..-.----..........----..........................................................................................----..-.---.-.-AA.**C****U****C**.**U**CA**G****G****C**..............AAAU**A**.**U****A****C****A****G****A****U****G**GGUAGGA | |
|  |  | AACY01335752.1/433-590  | AUUAA...-**G**.**U**.**A**.**A**.**C****G****G****G****A****G**A**G**.A**C****U****A****C****A**G..........AUUA.........................................U**C****G****U****A****G**..**C****G**.**C****C**GAA**G****G****A****G**CA...A..**C****U**A...............CCCA........................**G****G**.AAU**C****U****C****U**CA**G****G****C**.........................................................................AA.A.A.A.**G**.**G****A**.**C****C****G**..**U**..**A****A**.**C**.-.....AUAUUAA-............................................................-**C****U****C****U****G****G****A****A**A**G**-.**A**.**G****A****U**--...............................UUAA.......................................---**U****U****C**..**U****C****G****C****C**GAA**G****G****A****G**CAA..---..-.----..........----..........................................................................................----..-.---.-.-AA.**C****U****C**.**U**CA**G****G****C**..............AAAU**A**.**U****A****C****A****G****A****U****G**GGUACGA | |
|  |  | AACY01132952.1/41-194  | UUGAA...--.-.**U**.**A**.**C****G****G****G****A****G**A**G**.A**U****C****A****C****A**A..........UUAA.........................................-**U****G****U****G****G**..**C****A**.**C****C**GAA**G****G****A****G**CA...A..**C****U**A...............CCCA........................**G****G**.AAA**C****U****C****U**CA**G****G****U**..........................................................................A.A.A.A.**G**.**G****A**.**C****C****G**..**U**..**A**-.**A**.**C**.....AUAUUAA-............................................................-**C****U****C****U****G****G****A****A**A**G**-.**A**.**G****A****U**--...............................UAAA.......................................---**U****U****C**..**U****C****G****C****C**GAA**G****G****A****G**UAA..---..-.----..........----..........................................................................................----..-.---.-.-AA.**C****U****C**.**U**CA**G****G****C**..............AAAU**A**.**U****A****C****A****G****A****U****G**GGUACAA | |
|  |  | AACY01064603.1/688-439  | UCGCC...**C****G**.**G**.**U**.-.**C****A****G****G****A****G**A**G**.A**G****U****G****C****U**CCG........UCUUGUCCCCAGGACACG...........................C**A****G****C****A****C**..**C****G**.**C****C**GAA**G****G****C****G**CA...G..**G****C**A...............GCA-........................**G****C**CGAA**C****G****C****U**CA**G****G****C**..........................................................................A.A.A.A.**G**.**G****A**.**C****U****G**GC**A**..**A****C**.**C**.**G**CC...CGCAGCGAUGCGGGCGUUCCCU..............................................-**U****G****C****U****G****G****A****G**A**G**A.**G**.**G****U****G****G****C**CCCACAGAAAA....................GAAACUGUGUGCA..............................G**G****C****C****A****U**..**C****C****A****C****C**GAA**G****G****A****G**CAA..**A****C****C**..**G**.**C****A****C****G**GC........CAGC....................................................................................CAGGGC**G****C****U****G**..**C**.**G****G****U**.G.AAU.**C****U****C**.**U**CA**G****G****U**.............AAAGC**G**.**G****A****C****A****G****C****A****G**GGCAAAU | |
|  |  | AACY01090297.1/398-241  | AUUAG...**U**-.**U**.**A**.**A**.**C****G****G****G****A****G**A**G**.A**C****U****A****C****A**G..........AAUA.........................................U**C****G****U****A****G**..**C****G**.**C****C**GAA**G****G****A****G**CA...A..**C****C**A...............CCCA........................**G****G**.AAU**C****U****C****U**CA**G****G****C**.........................................................................AA.A.A.A.**G**.**G****A**.**C****C****G**..**U**..**A****A**.**C**.-.....AUAUUAA-............................................................-**C****U****C****U****G****G****A****A**A**G**-.**A**.**G****A****U**--...............................UAAA.......................................---**U****U****C**..**U****C****G****C****C**GAA**G****G****A****G**CAA..---..-.----..........----..........................................................................................----..-.---.-.-AA.**C****U****C**.**U**CA**G****G****C**..............AAAU**A**.**U****A****C****A****G****A****U****G**GGUAUAA | |
|  |  | AAGA01022452.1/685-381  | UGAGG...**C****G**.**U**.**U**.**G**.**C****G****G****G****A****G**A**G**.C**G****U****U****U****U**GCUGU......CUCACAGGACUG.................................U**A****A****G****G****C**..**C****G**.**C****C**GAA**G****G****A****G**CA...A..**C****C**G...............CCCC........................**G****G**.AAA**C****U****C****U**CA**G****G****C**..........................................................................C.A.A.C.**G**.**G****A**.**C****C****G**..**C**..**A****A**.**C**.**G**.....GCUGGCAAGCA.........................................................-**C****U****C****U****G****G****A****A**A**G**A.**G**.**A****G****C****G****G**AUGGU..........................CAAGUGACC..................................G**G****C****G****U****U**..**C****C****A****C****C**GAA**G****G****G****G**UAA..**A****U****G**..**G**.**C****G****U****C**GG........AUUUGAUCCGGCAUGAACGCUCGCCGUUCAUCCUGAGCUUGUUGAAGGAUGGUUGGCAAGCGUGCUUCGACAAGCUCGGCGCGAGCGGAAGUGA**A****A****A****G**..**C**.**C****A****U**.G.AAA.**C****U****C**.**U**CA**G****G****U**..............UCCG**U**.**G****A****C****A****G****A****G****G**GGGCAGU | |
|  |  | AACY01088449.1/960-1114  | UUAAA...**A****A**.**A**.**C**.**A**.**C****G****G****G****A****G**A**G**.A**C****U****A****C**-...........UUAAG........................................U-**G****U****A****G**..**C****G**.**C****C**GAA**G****G****A****G**CA...A..**C****C**A...............CCCC........................**G****G**.AAA**C****U****C****U**CA**G****G****C**..........................................................................A.A.A.A.**G**.**G****A**.**C****C****G**..**U**..**A****A**.-.-.....AAACUUAA............................................................-**U****U****C****U****G****G****A****A**A**G**-.**A**.**G****A**---...............................CAUGU......................................----**U****C**..**U****C****A****C****C**GAA**G****G****A****G**UAA..---..-.----..........----..........................................................................................----..-.---.-.-AU.**C****U****C**.**U**CA**G****G****U**..............ACCG**A**.**U****A****C****A****G****A****U****G**GGGUAAA | |
|  |  | AACY01390167.1/804-648  | UUGAU...--.**U**.**U**.**A**.**C****G****G****G****A****G**A**G**.A**C****U****A****C****A**G..........AUGA.........................................U**C****G****U****A****G**..**C****G**.**C****C**GAA**G****G****A****G**CA...A..**C****U**A...............CCCA........................**G****G**.AAU**C****U****C****U**CA**G****G****C**.........................................................................AA.A.A.A.**G**.**G****A**.**C****C****G**..**U**..**A****A**.**C**.-.....AUAUUAA-............................................................-**C****U****C****U****G****G****A****A**A**G**-.**A**.**G****A****U**--...............................UUAA.......................................---**U****U****C**..**U****C****G****C****C**GAA**G****G****A****G**CAA..---..-.----..........----..........................................................................................----..-.---.-.-AA.**C****U****C**.**U**CA**G****G****C**..............AAAU**A**.**U****A****C****A****G****A****U****G**GGUAUAA | |
|  |  | AACY01545924.1/310-155  | UUGAU...**U**-.-.**A**.**A**.**C****G****G****G****A****G**A**G**.A**C****U****A****C****A**U..........UAAA.........................................U**U****G****U****A****G**..**C****G**.**C****C**GAA**G****G****A****G**CA...A..**C****U**A...............CCCA........................**G****G**.AAU**C****U****C****U**CA**G****G****C**.........................................................................AA.A.A.A.**G**.**G****A**.**C****C****G**..**U**..**A**-.**C**.-.....AUAUUAA-............................................................-**C****U****C****U****G****G****A****A**A**G**-.**A**.**G****A****U**--...............................UAAA.......................................---**U****U****C**..**U****C****G****C****C**GAA**G****G****A****G**CAA..---..-.----..........----..........................................................................................----..-.---.-.-AA.**C****U****C**.**U**CA**G****G****C**..............AAAC**G**.**U****A****C****A****G****A****U****G**GGUUAAG | |
|  |  | AACY01049336.1/5837-5682  | AUUUA...**U**-.**A**.**U**.**A**.**C****G****G****G****A****G**A**G**.A**U****C****A****C****A**A..........UUAA.........................................-**U****G****U****G****G**..**C****A**.**C****C**GAA**G****G****A****G**CA...A..**C****U**A...............CCCA........................**G****G**.AAA**C****U****C****U**CA**G****G****U**..........................................................................A.A.A.A.**G**.**G****A**.**C****C****G**..**U**..**A**-.-.**A**.....CAUAUUAA............................................................-**C****U****C****U****G****G****A****A**A**G**-.**A**.**G****A****U**--...............................UAAA.......................................---**U****U****C**..**U****C****G****C****C**GAA**G****G****A****G**UAA..---..-.----..........----..........................................................................................----..-.---.-.-AA.**C****U****C**.**U**CA**G****G****C**..............AAAU**A**.**U****A****C****A****G****A****U****G**GGUACAA | |
|  |  | AACY01755755.1/740-586  | AUACU...**U****A**G**A**.**U**.**A**.**C****G****G****G****A****G**A**G**.A**C****U****A****C**-...........AUA-.........................................--**G****U****A****G**..**C****G**.**C****C**GAA**G****G****A****G**CA...A..**A****A**AA..............CCCC........................**A****G**.AAA**C****U****C****U**CA**G****G****C**..........................................................................A.A.A.A.**G**.**G****A**.**C****C****G**..**U**..**A****C**.-.-.....AUAUUAA-............................................................-**C****U****C****U****G****G****A****A**A**G**-.**A**.**G****A**---...............................CAAAU......................................----**U****C**..**U****C****G****C****C**GAA**G****G****A****G**CUAA.---..-.----..........----..........................................................................................----..-.---.-.AAU.**C****U****C**.**U**CA**G****G****C**..............ACCA**C**.**G****A****C****A****G****A****G****G**GGGUUAG | |
|  |  | AACY01415625.1/1-150  | A----...**G**-.-.-.-.**C****G****G****G****A****G**A**G**.A**C****U****G****C****A**A..........AAAGA........................................A**A****G****C****G****G**..**C****G**.**C****C**GAA**G****G****A****G**CA...A..**C****C**A...............CCCC........................**G****G**.AAA**C****U****C****U**CA**G****G****C**..........................................................................A.A.A.A.**G**.**G****A**.**C****C****G**..-..--.-.**U**.....GCAUAAUA............................................................**A****C****U****C****U****G****G****A****A**A**G**A.**A**.**G****G****C**--...............................-GUA.......................................---**G****C****C**..**U****C****A****C****C**GAA**G****G****A****G**UAA..---..-.----..........----..........................................................................................----..-.---.-.-AU.**C****U****C**.**U**CA**G****G****U**..............ACUC**A**.**G****A****C****A****G****A****G****G**GGGCGGG | |
|  |  | AACY01086237.1/242-419  | UAGAA...**A****G**.**G**.**G**.**U**.**C****A****G****G****A****G**A**G**.A**G****U****C**--...........GAAA.........................................---**G****A****C**..**C****G**.**C****C**GAA**G****G****C****U**GA...A..--................----........................--.---**C****G****C****U**CA**G****G****C**..........................................................................A.A.A.A.**G**.**U****A**.**C****U****G**..**A**..**C****A**.**U**.**U**.....UUAUAAUU............................................................**C****U****G****U****U****G****G****A****G**A**G**U.**G**.**A****C****A****A****C**GCA............................GAAGG......................................C**G****G****C****G****U**..**C****C****A****C****C**GAA**G****G****G****G**CAA..**A****C****C**..**C**.**C****G****A****G**CCG.......AUGC..........................................................................................**U****C****A****C**..**G**.**G****G****U**.G.AAU.**C****U****C**.**U**CA**G****G****U**..............CCAA**G**.**G****A****C****A****A****C****A****G**GGUUCCC | |
|  |  | AACY01208123.1/397-243  | AUUUC...**U****A**.**G**.**A**.**U**.**C****G****G****G****A****G**A**G**.A**C****U****A****U**-...........UUAAA........................................U-**A****U****A****G**..**C****G**.**C****C**GAA**G****G****A****G**CA...A..**C****C**A...............CCCC........................**G****G**.AAA**C****U****C****U**CA**G****G****C**..........................................................................A.A.A.A.**G**.**G****A**.**C****C****G**..-..-**C**.**A**.**A**.....AAAAUAAA............................................................-**U****U****C****U****G****G****A****A**A**G**-.**A**.**G****A****U**--...............................-AAU.......................................---**A****U****C**..**U****C****A****C****C**GAA**G****G****A****G**UAA..---..-.----..........----..........................................................................................----..-.---.-.-UU.**C****U****C**.**U**CA**G****G****U**..............ACCA**A**.**U****A****C****A****G****A****G****G**GGUUUAU | |
|  |  | AACY01026566.1/2296-2140  | UAACU...**U****U**.**C**.**A**.**A**.**C****G****G****G****A****G**A**U**.A**C****U****G****U****U**...........AAUA.........................................-**U****A****C****A****G**..**A****G**.**C****C**GAA**G****G****A****G**CA...A..**C****G**................ACCC........................**G****G**.AAA**C****U****C****U**CA**G****G****C**.........................................................................AA.A.A.A.**G**.**G****A**.**C****C****G**..**U**..**U****A**.**A**.**A**.....AUAAAAA-............................................................--**U****C****U****G****G****A****A**A--.**A**.**G****A****G****A**-...............................UUAU.......................................--**U****C****U****C**..**U**-**A****C****C**GAA**G****G****A****G**UAA..---..-.----..........----..........................................................................................----..-.---.-.AAA.**C****U****C**.**U**CA**G****G****U**..............AAAA**A**.**U****A****C****A****G****A****G****G**GCUUAGA | |
|  |  | AACY01434567.1/722-575  | -----...--.**U**.**A**.**A**.**C****G****G****G****A****G**A**G**.A**C****C****A****A**-...........UGU-.........................................--**U****U****G****G**..**C****G**.**C****C**GAA**G****G****A****G**CA...A..**C****C**A...............CCCC........................**G****G**.AAA**C****U****C****U**CA**G****G****C**..........................................................................A.A.A.A.**G**.**G****A**.**C****C****G**..**U**..**U****A**.-.-.....CCAUAAUA............................................................**A****C****U****C****U****G****G****A****A**A**G**C.**A**.**G****G****C**--...............................UUUA.......................................---**G****C****C**..**U****C****A****C****C**GAA**G****G****A****U**UAA..---..-.----..........----..........................................................................................----..-.---.-.-AU.**C****U****C**.**U**CA**G****G****U**..............ACAU**A**.**G****A****C****A****G****A****U****G**GGGUUGA | |
|  |  | AACY01073053.1/446-293  | AGAUU...**U****U**.**C**.**A**.**A**.**C****G****G****A****A****G**A**G**.A**C****U****A****C**-...........UUAAU........................................U-**G****U****A****G**..**C****G**.**C****C**GAA**G****G****A****G**CA...A..**C****C**A...............CCCC........................**G****G**.AAA**C****U****C****U**CA**G****G****C**..........................................................................A.A.A.A.**G**.**G****A**.**C****C****G**..**U**..**A****A**.**A**.-.....U-AUUAA-............................................................-**C****U****C****U****G****G****A****A**A**G**-.**A**.**G****A****A**--...............................-AGU.......................................---**U****U****C**..**U****C****G****C****C**GAC**G****G****A****G**UAA..---..-.----..........----..........................................................................................----..-.---.-.-AU.**C****U****C**.**U**CA**G****G****C**..............UCAU**A**.**G****A****C****A****G****A****G****G**GGGUAAG | |
|  |  | AACY01068117.1/1162-1004  | UAUAU...**C****U**.**C**.**U**.**A**.**C****G****G****G****A****G**A**G**.A**C****U****A****C****A**A..........AAUA.........................................U**C****G****U****A****G**..**C****G**.**C****C**GAA**G****G****A****G**CA...A..**C****C**A...............CCCA........................**G****G**.AAU**C****U****C****U**CA**G****G****C**.........................................................................AA.A.A.A.**G**.**G****A**.**C****C****G**..**U**..**A****A**.**C**.-.....AUAUUAA-............................................................-**C****U****C****U****G****G****A****A**A**G**-.**A**.**G****A****U**--...............................UUAA.......................................---**U****U****C**..**U****C****G****C****C**GAA**G****G****A****G**UAA..---..-.----..........----..........................................................................................----..-.---.-.-UA.**C****U****C**.**U**CA**G****G****C**..............AAAU**A**.**U****A****C****A****G****A****U****G**GGUACAA | |
|  |  | AACY01264455.1/670-517  | UUAAA...**A****A**.**A**.-.**A**.**C****G****G****G****A****G**A**G**.A**C****U****G****C**-...........UUAAA........................................U-**G****U****G****G**..**C****G**.**C****C**GAA**G****G****A****G**CA...A..**C****C**A...............CCC-.......................U**G****G**.AAA**C****U****C****U**CA**G****G****C**..........................................................................A.A.A.A.**G**.**G****A**.**C****C****G**..-..-**U**.**G**.**A**.....AAGUAAAA............................................................-**U****U****C****U****G****G****A****A**A**G**-.**A**.**G****A**---...............................CAAGU......................................----**U****C**..**U****C****A****C****C**GAA**G****G****A****G**UAA..---..-.----..........----..........................................................................................----..-.---.-.-AU.**C****U****C**.**U**CA**G****G****U**..............ACCG**A**.**U****A****C****A****G****A****U****G**GGGUAAG | |
|  |  | AACY01026567.1/413-256  | AUAUU...**U****A**.**U**.**A**.**A**A**C****G****G****G****A****G**A**U**.A**C****U****G****U****A**...........AUUA.........................................-**U****G****C****A****G**..**A****G**.**C****C**GAA**G****G****A****G**CA...A..**C****G**................ACCC........................**G****G**.AAA**C****U****C****U**CA**G****G****C**.........................................................................AA.A.A.A.**G**.**G****A**.**C****C****G**..**U**..**U****A**.**U**.**A**.....AUAUAAA-............................................................--**U****C****U****G****G****A****A**A--.**A**.**G****A****G****A**-...............................UAAA.......................................--**U****C****U****C**..**U**-**A****C****C**GAA**G****G****A****G**UAA..---..-.----..........----..........................................................................................----..-.---.-.AAA.**C****U****C**.**U**CA**G****G****U**..............ACAA**A**.**U****A****C****A****G****A****G****A**GGGCUUA | |
|  |  | AACY01072949.1/406-251  | UUAAA...--.-.**A**.**A**.**C****G****G****G****A****G**A**G**.A**C****U****A****C****A**G..........ACUA.........................................U**C****G****U****A****G**..**C****G**.**C****C**GAA**G****G****A****G**CA...A..**C****C**A...............CCCA........................**G****G**.AAU**C****U****C****U**CA**G****G****C**..........................................................................A.A.A.AU**G**.**G****A**.**C****C****G**..**U**..**A**-.**G**.**C**.....AUAUUAA-............................................................-**C****U****C****U****G****G****A****A**A**G**-.**A**.**G****A****U**--...............................UUAA.......................................---**U****U****C**..**U****C****G****C****C**GAA**G****G****A****G**CAA..---..-.----..........----..........................................................................................----..-.---.-.-AA.**C****U****C**.**U**CA**G****G****C**..............AAAU**A**.**U****A****C****A****G****A****U****G**GGUACAA | |
|  |  | AACY01600566.1/354-195  | AUUGA...**U****U**.**U**.**A**.**A**.**C****G****G****G****A****G**A**G**.A**C****U****A****C****A**GG.........AUAU.........................................-**U****G****U****A****G**..**C****G**U**C****C**GAA**G****G****A****G**CA...A..**C****C**A...............CCCA........................**G****G**.AAU**C****U****C****U**CA**G****G****C**..........................................................................A.A.A.G.**G**.**G****G**A**C****C****G**..**U**..**A****A**.**C**.-.....AUAUUAA-............................................................-**C****U****C****U****G****G****A****A**A**G**-.**A**.**G****A****U**--...............................UAAA.......................................---**U****U****C**..**U****C****G****C****C**GAA**G****G****A****G**CAA..---..-.----..........----..........................................................................................----..-.---.-.-AA.**C****U****C**.**U**CA**G****G****C**..............AAAU**A**.**U****A****C****A****G****A****U****G**GGGAUUA | |
|  |  | AACY01581218.1/668-818  | AUGU-...**U****U**.**U**.**A**.**A**.**U****G****G****G****A****G**A**G**.A**C****U****A**--...........UUU-.........................................---**U****A****G**..**C****G**.**C****C**GAA**G****G****A****G**CA...A..**C****U**U...............CCCC........................**G****G**.AAA**C****U****C****U**CA**G****G****C**..........................................................................A.A.A.A.**G**.**G****A**.**C****C****A**..**U**..**U****C**.**U**.**U**.....ACAUAAUA............................................................-**A****U****C****U****G****G****A****A**A**G**A.**G**.**A****A**---...............................UAAA.......................................----**U****C**..**U****C****A****A****C**GAC**G****G****A****G**UAA..---..-.----..........----..........................................................................................----..-.---.-.-AG.**C****U****C**.**U**CA**G****G****U**..............UAAU**C**.**A****A****C****A****G****A****U****G**GGGUUAA | |
|  |  | AACY01168808.1/826-680  | -----...**U**-.-.**A**.**U**.**C****G****G****G****A****G**A**G**.A**C****U****U**--...........UUUU.........................................---**U****A****G**..**C****G**.**C****C**GAA**G****G****A****G**CA...A..**C****A**A...............CCCC........................**G****G**.AAA**C****U****C****U**CA**G****G****C**..........................................................................A.A.A.A.**G**.**G****A**.**C****C****G**..**A**..**U****C**.**G**.**A**G....GCAUAACA............................................................-**C****U****C****U****G****G****A****A**A**G**U.**G**.**A****U**---...............................UAAU.......................................----**U****U**..**C****C****G****C****C**GAA**G****G****A****G**CAA..---..-.----..........----..........................................................................................----..-.---.-.-AA.**C****U****C**.**U**CA**G****G****C**..............AAAA**A**.**U****A****C****A****G****A****U****G**GGGUAGU | |
|  |  | AACY01323037.1/413-569  | AUUCA...**U**-.**U**.**U**.**A**.**C****G****G****G****A****G**A**G**.A**C****U****A****C****A**G..........AUUA.........................................U**C****G****U****A****G**..**C****G**.**C****C**GAA**G****G****A****G**CA...A..**C****C**A...............CCCA........................**G****G**.AAU**C****U****C****U**CA**G****G****C**.........................................................................AA.A.A.A.**G**.**G****A**.**C****C****G**..**U**..**G****C**.-.-.....AUAUUAA-............................................................-**C****U****C****U****G****G****A****A**A**G**-.**A**.**G****A****U**--...............................UAGA.......................................---**U****U****C**..**U****C****G****C****C**GAA**G****G****A****G**UAA..---..-.----..........----..........................................................................................----..-.---.-.-UA.**C****U****C**.**U**CA**G****G****C**..............AAAU**A**.**U****A****C****A****G****A****U****G**GGUAAAU | |
|  |  | AACY01474099.1/857-680  | -----...--.-.-.-.**C****G****G****G****A****G**A**G**.U**U****C****A****A**-...........UUCG.........................................U-**U****U****G****A**..**C****G**.**C****C**GAA**A****G****A****G**CA...A..**U****U**U...............CCCC........................**G****A**.AAA**C****U****C****U**CA**G****G****C**..........................................................................A.C.AUA.**G**.**G****A**.**C****C****G**..**C**..**A****A**.**A**.**A**.....GGAUGAAA............................................................-**C****U****C****U****G****G****A****A**A**G**U.**U**.**G****A****U****U****A**...............................GCAAUUUUUUG................................U**U****A****A****U****C**..**U****C****A****C****C**GAA**G****G****U****G**CAG..**A****G****C**..**A**.**U**---..........AUAU..........................................................................................---**A**..**U**.**G****C****U**.A.AAU.**C****U****C**U**U**CA**G****G****U**..............UCCA**C**.**G****A****C****A****G****A****G****G**GGGAGUA | |
|  |  | AACY01007479.1/155553-155285  | CCGCA...**C****G**.**C**.**G**.**U**.**C****G****G****G****A****G**A**G**.C**G****U****G****U****G**ACCGGGCUGU.UUCAGCCGG....................................U**U****G****C****G****C**..**C****G**.**C****C**GAA**G****G****G****G**CA...-..**C****A**................CCC-........................-**G**CAAA**C****U****C****U**CA**G****G****C**..........................................................................A.A.A.A.**G**.**G****A**.**C****C****G**..**A**.C**C****G**.**C**.**G**.....UCGGGGAAUCCCGUCCGCGCAUUGCGCGGGCCACACUCUCCCCGCA......................-**C****U****C****U****G****G****A****G**A**G**C.**G**.**G****C****A****G****U**AGCCCGCAGCGCAUCCGCUGCGCGG......GCAA.......................................-**G****C****U****G****C**..**C****C****A****C****C**GAA**G****G****G****G**CGC..**G****C****G**..**U**.**U****C****G****C**C.........AGGC....................................................................................CUGGCC**G****G****C****A**..**A**.**C****G****C**.-.AAU.**C****U****C**.**U**CA**G****G****U**.............AUCGA**G**.**G****A****C****A****G****A****G****G**GGCAUGU | |
|  |  | AACY01171926.1/805-962  | AUUGA...**U****U**.**U**.**U**.-.**C****G****G****G****A****G**A**G**.A**C****U****A****C****A**G..........AUUA.........................................U**C****G****U****A****G**..**C****G**.**C****C**GAA**A****G****A****G**CA...A..**C****U**A...............CCCA........................**G****G**.AAU**C****U****C****U**CA**G****G****C**.........................................................................AA.A.A.A.**G**.**G****A**.**C****C****G**..**U**..**A****A**.**C**.-.....AUAUUAA-............................................................-**C****U****C****U****G****G****A****A**A**G**-.**A**.**G****A****U**--...............................UUAA.......................................---**U****U****C**..**U****C****G****C****C**GAG**G****G****A****G**CAA..---..-.----..........----..........................................................................................----..-.---.-.-AA.**C****U****C**.**U**CA**G****G****C**..............AAAU**A**.**U****A****C****A****G****A****U****G**GGUAAAA | |
|  |  | AAFZ01013399.1/651-435  | ACGUG...**U****U**.**A**.**A**.**C**.**C****G****G****G****A****G**A**G**.C**C****U****G****A****A**UCG........CACACCUGUGU..................................G**A****U****C****G****G**..**C****C**.**C****C**GAA**G****G****A****G**CG...A..**A****C**U...............GCCC.......................C**G****G**.AAA**C****U****A****U**CA**G****C****U**..........................................................................A.G.A.C.**G**.**G****A**.**C****C****G**..**C**..**U****C**.**A**.**C**A....UGACGACU............................................................-**U****U****C****C****G****U****A****G**A**G**C.**U**.**G****U****C****G****A**CCCAUGGCG......................UUAGU......................................-**C****A****G****G****C**..**A****C****A****C****C**GAA**G****G****A****G**CAA..**C****U****G**..**C**.**U****G****G****U**ACA.......UAUU.....................................................................................UGAAA**C****G****G****C**..**G**.**C****G****U**.G.AAU.**C****U****C**.**U**CA**G****G****U**.............CCCAG**U**.**G****A****C****G****G****A****A****G**GGGUGCU | |
|  |  | AACY01092553.1/2821-2526  | CCGCA...**C****G**.**C**.**G**.**U**.**C****G****G****G****A****G**A**G**.C**G****U****G****C****U**GGCCGCAGA..AAUUCGCAGG...................................C**C****G****C****G****C**..**C****G**.**C****C**GAA**G****G****G****G**CA...C..--A...............CCCA.......................C--.AAA**C****U****C****U**CA**G****G****C**..........................................................................A.A.A.A.**G**.**G****A**.**C****C****G**..**G**..**C**-.**C**.**G**.....CGUCGAAAAACCCGCGUCAAUGCAGCAUCGAGACAUCGGUUUUUCGCA....................-**C****U****C****U****G****G****A****G**A**G**C.**G**.**G****C****A****G****U**AGCCGUCUGCAUCGU................UUAUAGCGGUGCAGAUUCCGGCA....................A**G****C****U****G****C**..**C****C****A****C****C**GAA**G****G****G****G**CGC..**G****C****G**..**U**.**U****U****C****A**CCG.......UAGC.....................................................................GAUCACGGCUUCACAUCGCAC**G****G****C****A**..**G**.**C****G****C**.-.AAU.**C****U****C**.**U**CA**G****G****U**.............AUCGA**G**.**G****A****C****A****G****A****G****G**GGUCAUG | |
|  |  | AACY01278240.1/8-245  | CGCCC...**U****C**.**C**.**U**.**C**.**C****G****G****G****A****G**A**G**.C**G****U****G****C****C**GGCCA......UACACGGCA....................................A**G****G****C****G****C**..**C****G**.**C****C**GAA**A****G****G****G**UA...-..**G****C**................ACCC........................**G****A**.AAA**C****U****C****U**CA**A****G****C**..........................................................................AUC.C.A.**G**.**G****A**.**C****C****G**..**G**..**G****C**.**G**.**G**AU...CGGCGGACAUGCGUCCGACCGCA.............................................-**C****U****C****U****G****G****A****G**A**G**CU**G**.**G****C****G****C****C**G..............................CACGACCACUGC...............................G**G****G****C****G****C**..**C****C****A****C****C**GAA**A****G****G****G**CUC..**A****C****G**..**A**.**A****C****G****G**CGCGCACCGCUAUG.....................................................................................CGCCC**C****G****C****U**..**U**.**C****G****C**.-.AAU.**C****U****C**.**U**CA**G****G****U**.............ACCAA**G**.**G****A****C****A****G****A****G****G**GGCCAUC | |
[truncated: 5,213 more chars]
