# Supplementary material for: The distributions, mechanisms, and structures of metabolite-binding riboswitches
Source: Genome Biol. 2007 Nov 12;8(11):R239. doi: 10.1186/gb-2007-8-11-r239 (PMC2258182; doi:10.1186/gb-2007-8-11-r239)
Supplement: Additional data file 2 — Sequence alignments of the riboswitch aptamer data sets annotated with new base-base interactions in HTML format. [file gb-2007-8-11-r239-S2.zip › HTML/Lysine.html]

|  |  |  |  |  |
| --- | --- | --- | --- | --- |
|  |  | **Accession/Start-End** |  | **Sequence** |
|  |  | NC\_003997.3/855442-855261  | CGGUG**A****G****G****U****A****G**AGGUU**G****C****A****G****U****C**.**A****U****U**AAG**A**G**U****A****U****C****A****U**.**U****U****C**U............**G****G****A****G****A****U****G****.****.****U****A****G****.****U****G****G**.**C****A****U****U**.GAU**G****A****A**....**G****G****A****A****U**.**G****A****G****A****A**AGG**A****A****U**.**G****A**.**U****U****G****C**CGA**A****G****U****A****A****G****U****U**.**G****U****G****U**....**C****C****A****C****C****A****U****.****.**.**G****C****A****C**..-**A****C****U**.**U****G**..**C****U****G****G****G****U****C****U****G**.**C****A****U****U**..UAAUA**A****G****U****G**.**C****A****G****A****A****C****U**G**U****C****A****C****A****A****A****C**G................UUUC........................................-**G****U****U****U****G****U****G****G**.AGAG**C****U****A****U****C****G**AGAGG | |
|  |  | NC\_005945.1/855337-855156  | CGGUG**A****G****G****U****A****G**AGGUU**G****C****A****G****U****C**.**A****U****U**AAG**A**G**U****A****U****C****A****U**.**U****U****C**U............**G****G****A****G****A****U****G****.****.****U****A****G****.****U****G****G**.**C****A****U****U**.GAU**G****A****A**....**G****G****A****A****U**.**G****A****G****A****A**AGG**A****A****U**.**G****A**.**U****U****G****C**CGA**A****G****U****A****A****G****U****U**.**G****U****G****U**....**C****C****A****C****C****A****U****.****.**.**G****C****A****C**..-**A****C****U**.**U****G**..**C****U****G****G****G****U****C****U****G**.**C****A****U****U**..UAAUA**A****G****U****G**.**C****A****G****A****A****C****U**G**U****C****A****C****A****A****A****C**G................UUUC........................................-**G****U****U****U****G****U****G****G**.AGAG**C****U****A****U****C****G**AGAGG | |
|  |  | NC\_005957.1/854453-854272  | CGGUG**A****G****G****U****A****G**AGGUU**G****C****A****G****U****C**.**A****U****U**AAG**A**G**U****A****U****C****A****U**.**U****U****C**U............**G****G****A****G****A****U****G****.****.****U****A****G****.****U****G****G**.**C****A****U****U**.GAU**G****A****A**....**G****G****A****A****U**.**G****A****G****A****A**AGG**A****A****U**.**G****A**.**U****U****G****C**CGA**A****G****U****A****A****G****U****U**.**G****U****G****U**....**C****C****A****C****C****A****U****.****.**.**G****C****A****C**..-**A****C****U**.**U****G**..**C****U****G****G****G****U****C****U****G**.**C****A****U****U**..UAAUA**A****G****U****G**.**C****A****G****A****A****C****U**G**U****C****A****C****A****A****A****C**G................UUUC........................................-**G****U****U****U****G****U****G****G**.AGAG**C****U****A****U****C****G**AGAGG | |
|  |  | NC\_006274.1/852008-851827  | CGGUG**A****G****G****U****A****G**AGGUU**G****C****A****G****U****C**.**A****U****U**AAG**A**G**U****A****U****C****A****U**.**U****U****C**U............**G****G****A****G****A****U****G****.****.****U****A****G****.****U****G****G**.**C****A****U****U**.GAU**G****A****A**....**G****G****A****A****U**.**G****A****G****A****A**AGG**A****A****U**.**G****A**.**U****U****G****C**CGA**A****G****U****A****A****G****U****U**.**G****U****G****U**....**C****C****A****C****C****A****U****.****.**.**G****C****A****C**..-**A****C****U**.**U****G**..**C****U****G****G****G****U****C****U****G**.**C****A****U****U**..UAAUA**A****G****U****G**.**C****A****G****A****A****C****U**G**U****C****A****C****A****A****A****C**G................UUUC........................................-**G****U****U****U****G****U****G****G**.AGAG**C****U****A****U****C****G**AGAGG | |
|  |  | NC\_007530.2/855443-855262  | CGGUG**A****G****G****U****A****G**AGGUU**G****C****A****G****U****C**.**A****U****U**AAG**A**G**U****A****U****C****A****U**.**U****U****C**U............**G****G****A****G****A****U****G****.****.****U****A****G****.****U****G****G**.**C****A****U****U**.GAU**G****A****A**....**G****G****A****A****U**.**G****A****G****A****A**AGG**A****A****U**.**G****A**.**U****U****G****C**CGA**A****G****U****A****A****G****U****U**.**G****U****G****U**....**C****C****A****C****C****A****U****.****.**.**G****C****A****C**..-**A****C****U**.**U****G**..**C****U****G****G****G****U****C****U****G**.**C****A****U****U**..UAAUA**A****G****U****G**.**C****A****G****A****A****C****U**G**U****C****A****C****A****A****A****C**G................UUUC........................................-**G****U****U****U****G****U****G****G**.AGAG**C****U****A****U****C****G**AGAGG | |
|  |  | NZ\_AAAC02000001.1/1354286-1354105  | CGGUG**A****G****G****U****A****G**AGGUU**G****C****A****G****U****C**.**A****U****U**AAG**A**G**U****A****U****C****A****U**.**U****U****C**U............**G****G****A****G****A****U****G****.****.****U****A****G****.****U****G****G**.**C****A****U****U**.GAU**G****A****A**....**G****G****A****A****U**.**G****A****G****A****A**AGG**A****A****U**.**G****A**.**U****U****G****C**CGA**A****G****U****A****A****G****U****U**.**G****U****G****U**....**C****C****A****C****C****A****U****.****.**.**G****C****A****C**..-**A****C****U**.**U****G**..**C****U****G****G****G****U****C****U****G**.**C****A****U****U**..UAAUA**A****G****U****G**.**C****A****G****A****A****C****U**G**U****C****A****C****A****A****A****C**G................UUUC........................................-**G****U****U****U****G****U****G****G**.AGAG**C****U****A****U****C****G**AGAGG | |
|  |  | NZ\_AAEN01000010.1/298140-298321  | CGGUG**A****G****G****U****A****G**AGGUU**G****C****A****G****U****C**.**A****U****U**AAG**A**G**U****A****U****C****A****U**.**U****U****C**U............**G****G****A****G****A****U****G****.****.****U****A****G****.****U****G****G**.**C****A****U****U**.GAU**G****A****A**....**G****G****A****A****U**.**G****A****G****A****A**AGG**A****A****U**.**G****A**.**U****U****G****C**CGA**A****G****U****A****A****G****U****U**.**G****U****G****U**....**C****C****A****C****C****A****U****.****.**.**G****C****A****C**..-**A****C****U**.**U****G**..**C****U****G****G****G****U****C****U****G**.**C****A****U****U**..UAAUA**A****G****U****G**.**C****A****G****A****A****C****U**G**U****C****A****C****A****A****A****C**G................UUUC........................................-**G****U****U****U****G****U****G****G**.AGAG**C****U****A****U****C****G**AGAGG | |
|  |  | NZ\_AAEO01000033.1/338562-338743  | CGGUG**A****G****G****U****A****G**AGGUU**G****C****A****G****U****C**.**A****U****U**AAG**A**G**U****A****U****C****A****U**.**U****U****C**U............**G****G****A****G****A****U****G****.****.****U****A****G****.****U****G****G**.**C****A****U****U**.GAU**G****A****A**....**G****G****A****A****U**.**G****A****G****A****A**AGG**A****A****U**.**G****A**.**U****U****G****C**CGA**A****G****U****A****A****G****U****U**.**G****U****G****U**....**C****C****A****C****C****A****U****.****.**.**G****C****A****C**..-**A****C****U**.**U****G**..**C****U****G****G****G****U****C****U****G**.**C****A****U****U**..UAAUA**A****G****U****G**.**C****A****G****A****A****C****U**G**U****C****A****C****A****A****A****C**G................UUUC........................................-**G****U****U****U****G****U****G****G**.AGAG**C****U****A****U****C****G**AGAGG | |
|  |  | NZ\_AAEP01000028.1/107232-107051  | CGGUG**A****G****G****U****A****G**AGGUU**G****C****A****G****U****C**.**A****U****U**AAG**A**G**U****A****U****C****A****U**.**U****U****C**U............**G****G****A****G****A****U****G****.****.****U****A****G****.****U****G****G**.**C****A****U****U**.GAU**G****A****A**....**G****G****A****A****U**.**G****A****G****A****A**AGG**A****A****U**.**G****A**.**U****U****G****C**CGA**A****G****U****A****A****G****U****U**.**G****U****G****U**....**C****C****A****C****C****A****U****.****.**.**G****C****A****C**..-**A****C****U**.**U****G**..**C****U****G****G****G****U****C****U****G**.**C****A****U****U**..UAAUA**A****G****U****G**.**C****A****G****A****A****C****U**G**U****C****A****C****A****A****A****C**G................UUUC........................................-**G****U****U****U****G****U****G****G**.AGAG**C****U****A****U****C****G**AGAGG | |
|  |  | NZ\_AAEQ01000022.1/109808-109627  | CGGUG**A****G****G****U****A****G**AGGUU**G****C****A****G****U****C**.**A****U****U**AAG**A**G**U****A****U****C****A****U**.**U****U****C**U............**G****G****A****G****A****U****G****.****.****U****A****G****.****U****G****G**.**C****A****U****U**.GAU**G****A****A**....**G****G****A****A****U**.**G****A****G****A****A**AGG**A****A****U**.**G****A**.**U****U****G****C**CGA**A****G****U****A****A****G****U****U**.**G****U****G****U**....**C****C****A****C****C****A****U****.****.**.**G****C****A****C**..-**A****C****U**.**U****G**..**C****U****G****G****G****U****C****U****G**.**C****A****U****U**..UAAUA**A****G****U****G**.**C****A****G****A****A****C****U**G**U****C****A****C****A****A****A****C**G................UUUC........................................-**G****U****U****U****G****U****G****G**.AGAG**C****U****A****U****C****G**AGAGG | |
|  |  | NZ\_AAER01000021.1/293874-294055  | CGGUG**A****G****G****U****A****G**AGGUU**G****C****A****G****U****C**.**A****U****U**AAG**A**G**U****A****U****C****A****U**.**U****U****C**U............**G****G****A****G****A****U****G****.****.****U****A****G****.****U****G****G**.**C****A****U****U**.GAU**G****A****A**....**G****G****A****A****U**.**G****A****G****A****A**AGG**A****A****U**.**G****A**.**U****U****G****C**CGA**A****G****U****A****A****G****U****U**.**G****U****G****U**....**C****C****A****C****C****A****U****.****.**.**G****C****A****C**..-**A****C****U**.**U****G**..**C****U****G****G****G****U****C****U****G**.**C****A****U****U**..UAAUA**A****G****U****G**.**C****A****G****A****A****C****U**G**U****C****A****C****A****A****A****C**G................UUUC........................................-**G****U****U****U****G****U****G****G**.AGAG**C****U****A****U****C****G**AGAGG | |
|  |  | NZ\_AAES01000027.1/16888-16707  | CGGUG**A****G****G****U****A****G**AGGUU**G****C****A****G****U****C**.**A****U****U**AAG**A**G**U****A****U****C****A****U**.**U****U****C**U............**G****G****A****G****A****U****G****.****.****U****A****G****.****U****G****G**.**C****A****U****U**.GAU**G****A****A**....**G****G****A****A****U**.**G****A****G****A****A**AGG**A****A****U**.**G****A**.**U****U****G****C**CGA**A****G****U****A****A****G****U****U**.**G****U****G****U**....**C****C****A****C****C****A****U****.****.**.**G****C****A****C**..-**A****C****U**.**U****G**..**C****U****G****G****G****U****C****U****G**.**C****A****U****U**..UAAUA**A****G****U****G**.**C****A****G****A****A****C****U**G**U****C****A****C****A****A****A****C**G................UUUC........................................-**G****U****U****U****G****U****G****G**.AGAG**C****U****A****U****C****G**AGAGG | |
|  |  | NC\_003366.1/1366236-1366062  | AACUG**A****G****A****U****A****G**AGGC.**G****C****G****A****U****G**.**A****U****U**AAU**A**G**U****A****U****C****U****U**.**U****G****C**.............**A****G****A****G****G****.****.****.****.****.****U****.****A****A****G****C**.**.****.****A****C**.AUU**G****A****A**....**G****C****A****A****A**.**G****U****G****A****A**AGG**A****U****G**.**A****A**.**U****C****G****C**CGA**A****A****C****C****A****U****U****A**.**G****A****A**-GAG.**G****C****U****U****U****A****A****.****.**.-**U****U****C**..**U****A****U****U**A**G****G**..**U****U****G****G****G****G****U****U****G**.**C****A****U****A**..GAAUA**U****A****U****G**.**U****A****A****C****A****C****U**G**U****C****A****C****A****A****A**-.................UUAU........................................--**U****U****U****G****U****G****G**.UGUG**C****U****A****U****C****A**UGAAA | |
|  |  | NC\_004722.1/842544-842363  | CGGUG**A****G****G****U****A****G**AGGUU**G****C****A****A****U****C**.**A****U****U**AAG**A**G**U****A****U****C****A****U**.**U****U****C**A............**G****G****A****G****A****U****G****.****.****.****U****A****G****U****G****G**.**C****A****U****U**.GAU**G****A****A**....**G****G****A****A****U**.**G****A****G****A****A**AGG**A****A****U**.**G****G**.**U****U****G****C**CGA**A****G****U****A****A****G****U****C**.**G****U****G****U**....**C****C****A****C****C****A****U****.****.**.**G****C****A****C**..-**G****C****U**.**U****G**..**C****U****G****G****G****U****C****U****G**.**C****A****U****U**..UAAUA**A****G****U****G**.**C****A****G****A****A****C****U**G**U****C****A****C****A****A****A****C**G................UUUC........................................-**G****U****U****U****G****U****G****G**.AGAG**C****U****A****U****C****G**AGAGG | |
|  |  | NZ\_AAEK01000002.1/121717-121536  | CGGUG**A****G****G****U****A****G**AGGUU**G****C****A****G****U****C**.**A****U****U**AAG**A**G**U****A****U****C****A****U**.**U****U****C**A............**G****G****A****G****A****U****G****.****.****.****U****A****G****U****G****G**.**C****A****U****U**.GAU**G****A****A**....**G****G****A****A****U**.**G****A****G****A****A**AGG**A****A****U**.**G****A**.**U****U****G****C**CGA**A****G****U****A****A****G****U****U**.**G****U****G****U**....**C****C****A****C****C****A****U****.****.**.**G****C****G****C**..-**A****C****U**.**U****G**..**C****U****G****G****G****U****C****U****G**.**C****A****U****U**..UAAUA**A****G****U****G**.**C****A****G****A****A****C****U**G**U****C****A****C****A****A****A****C**A................CUUC........................................-**G****U****U****U****G****U****G****G**.AGAG**C****U****A****U****C****G**AGAGG | |
|  |  | NC\_003997.3/1696832-1696646  | ACGUG**A****G****A****U****A****G**AGGUU**G****C****G****A****U****A**.**C****U****U**AUG**A**G**U****A****U****U****C****U**.**A****A****U**.............**G****G****A****G****A****C****.****.****.****A****C****A****G****A****G****A**.**G****G****U****C**.CAU**G****A****A**....**A****U****U****A****G**.**A****U****G****A****A**AGG**A****A****G**.**U****A**.**U****U****G****C**CGA**A****A****U****C****G****A****U****A**.**U****A****U**-...U**U****C****U****C****U****G****U****.****.**.-**A****U****G**..**U****A****U****C**.**G****A**..**U****U****G****G****G****G****C****U****G**.**U****U****U****U**.CGAAUA**G****A****A****A**.**C****A****G****A****A****C****U**G**U****C****A****U****A****U****G****U**ACA..............GACAUG......................................U**A****C****G****U****A****U****G****A**.AGAG**C****U****A****U****C****U**ACAAA | |
|  |  | NC\_005945.1/1696900-1696714  | ACGUG**A****G****A****U****A****G**AGGUU**G****C****G****A****U****A**.**C****U****U**AUG**A**G**U****A****U****U****C****U**.**A****A****U**.............**G****G****A****G****A****C****.****.****.****A****C****A****G****A****G****A**.**G****G****U****C**.CAU**G****A****A**....**A****U****U****A****G**.**A****U****G****A****A**AGG**A****A****G**.**U****A**.**U****U****G****C**CGA**A****A****U****C****G****A****U****A**.**U****A****U**-...U**U****C****U****C****U****G****U****.****.**.-**A****U****G**..**U****A****U****C**.**G****A**..**U****U****G****G****G****G****C****U****G**.**U****U****U****U**.CGAAUA**G****A****A****A**.**C****A****G****A****A****C****U**G**U****C****A****U****A****U****G****U**ACA..............GACAUG......................................U**A****C****G****U****A****U****G****A**.AGAG**C****U****A****U****C****U**ACAAA | |
|  |  | NC\_006274.1/1709214-1709028  | ACGUG**A****G****A****U****A****G**AGGUU**G****C****G****A****U****A**.**C****U****U**AUG**A**G**U****A****U****U****C****U**.**A****A****U**.............**G****G****A****G****A****C****.****.****.****A****C****A****G****A****G****A**.**G****G****U****C**.CAU**G****A****A**....**A****U****U****A****G**.**A****U****G****A****A**AGG**A****A****G**.**U****A**.**U****U****G****C**CGA**A****A****U****C****G****A****U****A**.**U****A****U**-...U**U****C****U****C****U****G****U****.****.**.-**A****U****G**..**U****A****U****C**.**G****A**..**U****U****G****G****G****G****C****U****G**.**U****U****U****U**.CGAAUA**G****A****A****A**.**C****A****G****A****A****C****U**G**U****C****A****U****A****U****G****U**ACA..............GACAUG......................................U**A****C****G****U****A****U****G****A**.AGAG**C****U****A****U****C****U**ACAAA | |
|  |  | NC\_007530.2/1696955-1696769  | ACGUG**A****G****A****U****A****G**AGGUU**G****C****G****A****U****A**.**C****U****U**AUG**A**G**U****A****U****U****C****U**.**A****A****U**.............**G****G****A****G****A****C****.****.****.****A****C****A****G****A****G****A**.**G****G****U****C**.CAU**G****A****A**....**A****U****U****A****G**.**A****U****G****A****A**AGG**A****A****G**.**U****A**.**U****U****G****C**CGA**A****A****U****C****G****A****U****A**.**U****A****U**-...U**U****C****U****C****U****G****U****.****.**.-**A****U****G**..**U****A****U****C**.**G****A**..**U****U****G****G****G****G****C****U****G**.**U****U****U****U**.CGAAUA**G****A****A****A**.**C****A****G****A****A****C****U**G**U****C****A****U****A****U****G****U**ACA..............GACAUG......................................U**A****C****G****U****A****U****G****A**.AGAG**C****U****A****U****C****U**ACAAA | |
|  |  | NZ\_AAAC02000001.1/2182146-2181960  | ACGUG**A****G****A****U****A****G**AGGUU**G****C****G****A****U****A**.**C****U****U**AUG**A**G**U****A****U****U****C****U**.**A****A****U**.............**G****G****A****G****A****C****.****.****.****A****C****A****G****A****G****A**.**G****G****U****C**.CAU**G****A****A**....**A****U****U****A****G**.**A****U****G****A****A**AGG**A****A****G**.**U****A**.**U****U****G****C**CGA**A****A****U****C****G****A****U****A**.**U****A****U**-...U**U****C****U****C****U****G****U****.****.**.-**A****U****G**..**U****A****U****C**.**G****A**..**U****U****G****G****G****G****C****U****G**.**U****U****U****U**.CGAAUA**G****A****A****A**.**C****A****G****A****A****C****U**G**U****C****A****U****A****U****G****U**ACA..............GACAUG......................................U**A****C****G****U****A****U****G****A**.AGAG**C****U****A****U****C****U**ACAAA | |
|  |  | NZ\_AAEN01000011.1/266388-266202  | ACGUG**A****G****A****U****A****G**AGGUU**G****C****G****A****U****A**.**C****U****U**AUG**A**G**U****A****U****U****C****U**.**A****A****U**.............**G****G****A****G****A****C****.****.****.****A****C****A****G****A****G****A**.**G****G****U****C**.CAU**G****A****A**....**A****U****U****A****G**.**A****U****G****A****A**AGG**A****A****G**.**U****A**.**U****U****G****C**CGA**A****A****U****C****G****A****U****A**.**U****A****U**-...U**U****C****U****C****U****G****U****.****.**.-**A****U****G**..**U****A****U****C**.**G****A**..**U****U****G****G****G****G****C****U****G**.**U****U****U****U**.CGAAUA**G****A****A****A**.**C****A****G****A****A****C****U**G**U****C****A****U****A****U****G****U**ACA..............GACAUG......................................U**A****C****G****U****A****U****G****A**.AGAG**C****U****A****U****C****U**ACAAA | |
|  |  | NZ\_AAEO01000025.1/264361-264175  | ACGUG**A****G****A****U****A****G**AGGUU**G****C****G****A****U****A**.**C****U****U**AUG**A**G**U****A****U****U****C****U**.**A****A****U**.............**G****G****A****G****A****C****.****.****.****A****C****A****G****A****G****A**.**G****G****U****C**.CAU**G****A****A**....**A****U****U****A****G**.**A****U****G****A****A**AGG**A****A****G**.**U****A**.**U****U****G****C**CGA**A****A****U****C****G****A****U****A**.**U****A****U**-...U**U****C****U****C****U****G****U****.****.**.-**A****U****G**..**U****A****U****C**.**G****A**..**U****U****G****G****G****G****C****U****G**.**U****U****U****U**.CGAAUA**G****A****A****A**.**C****A****G****A****A****C****U**G**U****C****A****U****A****U****G****U**ACA..............GACAUG......................................U**A****C****G****U****A****U****G****A**.AGAG**C****U****A****U****C****U**ACAAA | |
|  |  | NZ\_AAEP01000035.1/268616-268430  | ACGUG**A****G****A****U****A****G**AGGUU**G****C****G****A****U****A**.**C****U****U**AUG**A**G**U****A****U****U****C****U**.**A****A****U**.............**G****G****A****G****A****C****.****.****.****A****C****A****G****A****G****A**.**G****G****U****C**.CAU**G****A****A**....**A****U****U****A****G**.**A****U****G****A****A**AGG**A****A****G**.**U****A**.**U****U****G****C**CGA**A****A****U****C****G****A****U****A**.**U****A****U**-...U**U****C****U****C****U****G****U****.****.**.-**A****U****G**..**U****A****U****C**.**G****A**..**U****U****G****G****G****G****C****U****G**.**U****U****U****U**.CGAAUA**G****A****A****A**.**C****A****G****A****A****C****U**G**U****C****A****U****A****U****G****U**ACA..............GACAUG......................................U**A****C****G****U****A****U****G****A**.AGAG**C****U****A****U****C****U**ACAAA | |
|  |  | NZ\_AAEQ01000029.1/270089-269903  | ACGUG**A****G****A****U****A****G**AGGUU**G****C****G****A****U****A**.**C****U****U**AUG**A**G**U****A****U****U****C****U**.**A****A****U**.............**G****G****A****G****A****C****.****.****.****A****C****A****G****A****G****A**.**G****G****U****C**.CAU**G****A****A**....**A****U****U****A****G**.**A****U****G****A****A**AGG**A****A****G**.**U****A**.**U****U****G****C**CGA**A****A****U****C****G****A****U****A**.**U****A****U**-...U**U****C****U****C****U****G****U****.****.**.-**A****U****G**..**U****A****U****C**.**G****A**..**U****U****G****G****G****G****C****U****G**.**U****U****U****U**.CGAAUA**G****A****A****A**.**C****A****G****A****A****C****U**G**U****C****A****U****A****U****G****U**ACA..............GACAUG......................................U**A****C****G****U****A****U****G****A**.AGAG**C****U****A****U****C****U**ACAAA | |
|  |  | NZ\_AAER01000023.1/257440-257254  | ACGUG**A****G****A****U****A****G**AGGUU**G****C****G****A****U****A**.**C****U****U**AUG**A**G**U****A****U****U****C****U**.**A****A****U**.............**G****G****A****G****A****C****.****.****.****A****C****A****G****A****G****A**.**G****G****U****C**.CAU**G****A****A**....**A****U****U****A****G**.**A****U****G****A****A**AGG**A****A****G**.**U****A**.**U****U****G****C**CGA**A****A****U****C****G****A****U****A**.**U****A****U**-...U**U****C****U****C****U****G****U****.****.**.-**A****U****G**..**U****A****U****C**.**G****A**..**U****U****G****G****G****G****C****U****G**.**U****U****U****U**.CGAAUA**G****A****A****A**.**C****A****G****A****A****C****U**G**U****C****A****U****A****U****G****U**ACA..............GACAUG......................................U**A****C****G****U****A****U****G****A**.AGAG**C****U****A****U****C****U**ACAAA | |
|  |  | NZ\_AAES01000034.1/268385-268199  | ACGUG**A****G****A****U****A****G**AGGUU**G****C****G****A****U****A**.**C****U****U**AUG**A**G**U****A****U****U****C****U**.**A****A****U**.............**G****G****A****G****A****C****.****.****.****A****C****A****G****A****G****A**.**G****G****U****C**.CAU**G****A****A**....**A****U****U****A****G**.**A****U****G****A****A**AGG**A****A****G**.**U****A**.**U****U****G****C**CGA**A****A****U****C****G****A****U****A**.**U****A****U**-...U**U****C****U****C****U****G****U****.****.**.-**A****U****G**..**U****A****U****C**.**G****A**..**U****U****G****G****G****G****C****U****G**.**U****U****U****U**.CGAAUA**G****A****A****A**.**C****A****G****A****A****C****U**G**U****C****A****U****A****U****G****U**ACA..............GACAUG......................................U**A****C****G****U****A****U****G****A**.AGAG**C****U****A****U****C****U**ACAAA | |
|  |  | NC\_005957.1/1736329-1736143  | ACGUG**A****G****A****U****A****G**AGGUU**G****C****G****A****U****A**.**C****U****U**AUG**A**G**U****A****U****U****C****U**.**A****A****U**.............**G****G****A****G****A****C****.****.****.****A****C****A****G****A****G****A**.**G****G****U****C**.CAU**G****A****A**....**A****U****U****A****G**.**A****U****G****A****A**AGG**A****A****G**.**U****A**.**U****U****G****C**CGA**A****A****U****C****G****A****U****A**.**U****A****U**-...U**U****C****U****C****U****G****U****.****.**.-**A****U****G**..**U****A****U****C**.**G****A**..**U****U****G****G****G****G****C****U****G**.**U****U****U****U**.CGAAUA**G****A****A****A**.**C****A****G****A****A****C****U**G**U****C****A****U****A****U****G****U**ACAGAC...........GUGU........................................-**A****C****G****U****A****U****G****A**.AGAG**C****U****A****U****C****U**ACAAA | |
|  |  | NC\_003909.8/957672-957490  | CGGUG**A****G****G****U****A****G**AGGUU**G****C****A****G****U****C**.**A****U****U**AAG**A**G**U****A****U****C****A****U**.**U****U****C**A............**G****G****A****G****A****U****G****.****.****U****A****.****G****U****G****G**.**C****A****U****U**.GAU**G****A****A**....**C****G****A****A****U**.**G****A****G****A****A**AGG**A****A****U**.**G****A**.**U****U****G****C**CGA**A****G****U****A****A****G****C****C**C**G****U****G****.**...U**C****C****A****C****C****A****U****G****.**.**.****C****A****C**..-**G****C****U**.**U****G**..**C****U****G****G****G****U****C****U****G**.**C****A****U****U**..GAAUA**A****G****U****G**.**C****A****G****A****A****C****U**G**U****C****A****C****A****A****A****C**G................UUUC........................................-**G****U****U****U****G****U****G****G**.AGAG**C****U****A****U****C****G**AGAGG | |
|  |  | NC\_003909.8/1818638-1818452  | ACGUG**A****G****A****U****A****G**AGGUU**G****C****G****A****U****A**.**C****U****U**AUG**A**G**U****A****U****U****C****U**.**A****A****U**.............**G****G****A****G****A****C****.****.****.****A****C****A****G****A****G****A**G**.****G****U****C**.UAU**G****A****A**....**A****U****U****A****G**.**A****U****G****A****A**AGG**A****A****G**.**U****A**.**U****U****G****C**CGA**A****A****U****C****G****A****U****A**.**U****A****U**-...U**U****C****U****C****U****G****U****.****.**.-**A****U****G**..**U****A****U****C**.**G****A**..**U****U****G****G****G****G****C****U****G**.**U****U****U****U**.CGAAUA**G****A****A****A**.**C****A****G****A****A****C****U**G**U****C****A****U****A****U****G****U**ACA..............GACGCG......................................U**A****U****G****U****A****U****G****A**.AGAG**C****U****A****U****C****U**ACAAA | |
|  |  | NZ\_AAEK01000005.1/66139-66325  | ACGUG**A****G****A****U****A****G**AGGUU**G****C****G****A****U****A**.**C****U****U**AUG**A**G**U****A****U****U****C****U**.**A****A****U**.............**G****G****A****G****A****C****.****.****.****A****C****A****G****A****G****A**U**.****G****U****C**.UAU**G****A****A**....**A****U****U****A****G**.**A****U****G****A****A**AGG**A****A****G**.**U****A**.**U****U****G****C**CGA**A****A****U****C****G****A****U****A**.**U****A****U**-...U**U****C****U****C****U****G****U****.****.**.-**A****U****G**..**U****A****U****C**.**G****A**..**U****U****G****G****G****G****C****U****G**.**U****U****U****U**.CGAAUA**G****A****A****A**.**C****A****G****A****A****C****U**G**U****C****A****U****A****U****G****U**ACAGAC...........GUGU........................................-**A****C****G****U****A****U****G****A**.AGAG**C****U****A****U****C****U**ACAAA | |
|  |  | NC\_002570.2/3562930-3562757  | AGAUG**G****G****G****U****A****G**AGGA.**G****C****G****G****G****U**.**U****U****U**AAG**A**G**U****A****A****G****C****G**.**C****U****U**.............**G****G****A****G****G****.****.****.****.****.****A****U****G****A****C****A**.**.****.****A****C**.GAG**G****A****U**....**A****A****G****C****G**.**C****C****G****A****A**AGG**A****A****A**.**A****C**.**U****C****G****C**CGA**A****G****C**-**G****G****A****A**.**G****A****U****G**....**A****G****U****C****A****A****G****.****.**.**C****G****U****C**..**U****U****C****U**.**U****G**..**C****U****G****G****G****G****U****U****G**.**C****A****U****U**..GAAUA**A****A****U****G**.**U****A****A****C****A****C****U**G**U****C****A****C****A****G****C****A**.................GAU-........................................-**U****G****C****U****G****U****G****G**.AGAA**C****U****A****C****U****A**ACGUU | |
|  |  | NC\_002570.2/1819757-1819575  | AGUGA**G****G****A****U****A****G**AGGU.**G****C****A****A****A****A**.**A****C****C**AAG**A**G**U****A****C****A****C****A**.**A****U****U**.............**G****G****A****G****G****A****.****.****G****A****A****U****G****A****G****A**.**.****U****C****C**.GUU**G****A****G**....**A****A****U****U****G**.**U****G****G****A****A**AGG**G****G****A**.**A****U**.**U****U****G****C**CGA**A****G****C****U****G****G****A****A**.**G****A****A**-....**U****C****U****C****A****U****G****.****.**.-**U****U****C**..**U****G****A****A**.**G****G**..**C****U****G****G****U****U****C****U****G**.**U****A****U****U**..AAAUA**A****A****U****A**.**C****A****G****A****A****C****U**G**U****C****A****U****A****U****A****G**CGGAU............GUUG........................................-**C****U****A****U****A****U****G****G**.AGGG**C****U****A****U****C****U**CACGC | |
|  |  | NC\_004722.1/1699021-1698835  | ACGUG**A****G****A****U****A****G**AGGUU**G****C****G****A****U****A**.**C****U****U**AUG**A**G**U****A****U****U****C****U**.**A****A****U**.............**G****G****A****G****A****C****A****.****.****.****C****A****G****A****G****A**.**U****G****U****C**.UAU**G****A****A**....**C****U****U****A****G**.**A****U****G****A****A**AGG**A****A****G**.**U****A**.**U****U****G****C**CGA**A****A****U****U****G****A****U****A**.**A****A****U****U**....**U****C****U****C****U****G****.****.****.**.**C****A****U****U**..**U****A****U****C**.**A****A**..**U****U****G****G****G****G****C****U****G**.**U****U****U****U**.CGAAUA**G****A****A****A**.**C****A****G****A****A****C****U**G**U****C****A****U****A****U****G****U**ACAGAC...........GUGU........................................-**A****C****G****U****A****U****G****A**.AGAG**C****U****A****U****C****U**ACAAA | |
|  |  | NC\_002570.2/1619231-1619405  | GGAUG**A****G****G****U****A****G**AGGU.**G****C****A****A****U****G**.**C****G****A**AUC**A**G**U****A****C****C****C****A**.**C****U****U**.............**G****G****A****G****U****.****.****.****U****U****G****A****U****G****G****A**.**.****.****A****C**.UAG**G****A****A**....**G****A****G****U****G**.**G****G****G****A****A**AGG**U****C****A**.**A****U**.**U****U****G****C**CGA**A****G****U****G****A****A****U****G**.**U****A****U****G**....**U****C****C****A****U****C****C****.****.**.**C****A****U****A**..**C****G****U****U**.**U****G**..**C****U****G****G****G****U****C****G****U**.**U****U****U****U**..GAAUA**A****A****A****A**.**A****C****G****A****A****C****U**G**C****C****G****C****U****G****A**-.................CUG-........................................--**U****U****A****G****C****G****G**.AGAG**C****U****A****U****C****U**GCCAA | |
|  |  | NC\_003030.1/3651159-3650984  | AACUG**A****G****G****U****A****G**AGGC.**G****C****A****A****A****A**.**U****U****U**AAG**A**G**U****A****G****A****A****C**.**U****G****U**.............**G****G****A****G**--**.****.****.****A****C****A****A****G****C****A**.**.**--**C**.UAU**G****A****A**....**G****C****A****G****U**.**U****U****A****A****A**AGG**A****A****A**.**U****U**.**U****U****G****C**CGA**A****G****C****G****U****A****U****A**.**G****C****U****A**A...**U****G****C****U****U****U****A****.****.**A**U**-**G****C**..**U****G****U****A**C**U****G**..**C****U****G****G****G****U****U****U****G**.**U****A****U****A**..AAAUA**U****G****U****G**.**C****A****A****A****A****C****U**G**U****C****A****C****A****A**--AGU..............AUAA........................................---**U****U****G****U****G****G**.AGAA**C****U****A****U****C****A**UUGGU | |
|  |  | NC\_003997.3/1360863-1361044  | CUCAA**A****G****G****U****A****G**AGGCC**G****C****G****A****U****A**.**G****G****A**AAG**A**G**U****A****A****G****C**-.**U****A****U**G............**G****G****A****G****A****U****U****.****.****.****U****A****A****U****G****G**.**A****A****U****C**.UGU**G****A****U**...C**A****U****A****G****G**.**U****U****G****A****A**AGG**G****A****C**.**U****A**.**U****U****G****C**CGA**A****A****U****A****U****A****A****G**.**A****A****U****A**A...**C****C****A****U****C****.****.****.****.**U**U****A****U****U**..**C****A****U****A**.**U****A**..**U****U****G****G****G****A****C****U****G**.**C****A****U****U**..GAAUA**A****A****U****G**.**U****A****G****U****A****C****U**G**U****C****A****U****A****A****G****A**.................UUUA........................................-**U****U****U****U****A****U****G****G**.AGAG**C****U****A****U****U****U**GGAGA | |
|  |  | NC\_004722.1/1380246-1380427  | CUCAA**A****G****G****U****A****G**AGGCC**G****C****G****A****U****A**.**G****G****A**AAG**A**G**U****A****A****G****C**-.**U****A****U**G............**G****G****A****G****A****U****U****.****.****.****U****A****A****U****G****G**.**A****A****U****C**.UGU**G****A****U**...C**A****U****A****G****G**.**U****U****G****A****A**AGG**G****A****C**.**U****A**.**U****U****G****C**CGA**A****A****U****A****U****A****A****G**.**A****A****U****A**A...**C****C****A****U****C****.****.****.****.**U**U****A****U****U**..**C****A****U****A**.**U****A**..**U****U****G****G****G****A****C****U****G**.**C****A****U****U**..GAAUA**A****A****U****G**.**U****A****G****U****A****C****U**G**U****C****A****U****A****A****G****A**.................UUUA........................................-**U****U****U****U****A****U****G****G**.AGAG**C****U****A****U****U****U**GGAGA | |
|  |  | NC\_005945.1/1360932-1361113  | CUCAA**A****G****G****U****A****G**AGGCC**G****C****G****A****U****A**.**G****G****A**AAG**A**G**U****A****A****G****C**-.**U****A****U**G............**G****G****A****G****A****U****U****.****.****.****U****A****A****U****G****G**.**A****A****U****C**.UGU**G****A****U**...C**A****U****A****G****G**.**U****U****G****A****A**AGG**G****A****C**.**U****A**.**U****U****G****C**CGA**A****A****U****A****U****A****A****G**.**A****A****U****A**A...**C****C****A****U****C****.****.****.****.**U**U****A****U****U**..**C****A****U****A**.**U****A**..**U****U****G****G****G****A****C****U****G**.**C****A****U****U**..GAAUA**A****A****U****G**.**U****A****G****U****A****C****U**G**U****C****A****U****A****A****G****A**.................UUUA........................................-**U****U****U****U****A****U****G****G**.AGAG**C****U****A****U****U****U**GGAGA | |
|  |  | NC\_006274.1/1395586-1395767  | CUCAA**A****G****G****U****A****G**AGGCC**G****C****G****A****U****A**.**G****G****A**AAG**A**G**U****A****A****G****C**-.**U****A****U**G............**G****G****A****G****A****U****U****.****.****.****U****A****A****U****G****G**.**A****A****U****C**.UGU**G****A****U**...C**A****U****A****G****G**.**U****U****G****A****A**AGG**G****A****C**.**U****A**.**U****U****G****C**CGA**A****A****U****A****U****A****A****G**.**A****A****U****A**A...**C****C****A****U****C****.****.****.****.**U**U****A****U****U**..**C****A****U****A**.**U****A**..**U****U****G****G****G****A****C****U****G**.**C****A****U****U**..GAAUA**A****A****U****G**.**U****A****G****U****A****C****U**G**U****C****A****U****A****A****G****A**.................UUUA........................................-**U****U****U****U****A****U****G****G**.AGAG**C****U****A****U****U****U**GGAGA | |
|  |  | NC\_007530.2/1360986-1361167  | CUCAA**A****G****G****U****A****G**AGGCC**G****C****G****A****U****A**.**G****G****A**AAG**A**G**U****A****A****G****C**-.**U****A****U**G............**G****G****A****G****A****U****U****.****.****.****U****A****A****U****G****G**.**A****A****U****C**.UGU**G****A****U**...C**A****U****A****G****G**.**U****U****G****A****A**AGG**G****A****C**.**U****A**.**U****U****G****C**CGA**A****A****U****A****U****A****A****G**.**A****A****U****A**A...**C****C****A****U****C****.****.****.****.**U**U****A****U****U**..**C****A****U****A**.**U****A**..**U****U****G****G****G****A****C****U****G**.**C****A****U****U**..GAAUA**A****A****U****G**.**U****A****G****U****A****C****U**G**U****C****A****U****A****A****G****A**.................UUUA........................................-**U****U****U****U****A****U****G****G**.AGAG**C****U****A****U****U****U**GGAGA | |
|  |  | NZ\_AAAC02000001.1/1852960-1853141  | CUCAA**A****G****G****U****A****G**AGGCC**G****C****G****A****U****A**.**G****G****A**AAG**A**G**U****A****A****G****C**-.**U****A****U**G............**G****G****A****G****A****U****U****.****.****.****U****A****A****U****G****G**.**A****A****U****C**.UGU**G****A****U**...C**A****U****A****G****G**.**U****U****G****A****A**AGG**G****A****C**.**U****A**.**U****U****G****C**CGA**A****A****U****A****U****A****A****G**.**A****A****U****A**A...**C****C****A****U****C****.****.****.****.**U**U****A****U****U**..**C****A****U****A**.**U****A**..**U****U****G****G****G****A****C****U****G**.**C****A****U****U**..GAAUA**A****A****U****G**.**U****A****G****U****A****C****U**G**U****C****A****U****A****A****G****A**.................UUUA........................................-**U****U****U****U****A****U****G****G**.AGAG**C****U****A****U****U****U**GGAGA | |
|  |  | NZ\_AAEN01000008.1/63149-62968  | CUCAA**A****G****G****U****A****G**AGGCC**G****C****G****A****U****A**.**G****G****A**AAG**A**G**U****A****A****G****C**-.**U****A****U**G............**G****G****A****G****A****U****U****.****.****.****U****A****A****U****G****G**.**A****A****U****C**.UGU**G****A****U**...C**A****U****A****G****G**.**U****U****G****A****A**AGG**G****A****C**.**U****A**.**U****U****G****C**CGA**A****A****U****A****U****A****A****G**.**A****A****U****A**A...**C****C****A****U****C****.****.****.****.**U**U****A****U****U**..**C****A****U****A**.**U****A**..**U****U****G****G****G****A****C****U****G**.**C****A****U****U**..GAAUA**A****A****U****G**.**U****A****G****U****A****C****U**G**U****C****A****U****A****A****G****A**.................UUUA........................................-**U****U****U****U****A****U****G****G**.AGAG**C****U****A****U****U****U**GGAGA | |
|  |  | NZ\_AAEO01000037.1/12025-11844  | CUCAA**A****G****G****U****A****G**AGGCC**G****C****G****A****U****A**.**G****G****A**AAG**A**G**U****A****A****G****C**-.**U****A****U**G............**G****G****A****G****A****U****U****.****.****.****U****A****A****U****G****G**.**A****A****U****C**.UGU**G****A****U**...C**A****U****A****G****G**.**U****U****G****A****A**AGG**G****A****C**.**U****A**.**U****U****G****C**CGA**A****A****U****A****U****A****A****G**.**A****A****U****A**A...**C****C****A****U****C****.****.****.****.**U**U****A****U****U**..**C****A****U****A**.**U****A**..**U****U****G****G****G****A****C****U****G**.**C****A****U****U**..GAAUA**A****A****U****G**.**U****A****G****U****A****C****U**G**U****C****A****U****A****A****G****A**.................UUUA........................................-**U****U****U****U****A****U****G****G**.AGAG**C****U****A****U****U****U**GGAGA | |
|  |  | NZ\_AAEP01000030.1/166894-167075  | CUCAA**A****G****G****U****A****G**AGGCC**G****C****G****A****U****A**.**G****G****A**AAG**A**G**U****A****A****G****C**-.**U****A****U**G............**G****G****A****G****A****U****U****.****.****.****U****A****A****U****G****G**.**A****A****U****C**.UGU**G****A****U**...C**A****U****A****G****G**.**U****U****G****A****A**AGG**G****A****C**.**U****A**.**U****U****G****C**CGA**A****A****U****A****U****A****A****G**.**A****A****U****A**A...**C****C****A****U****C****.****.****.****.**U**U****A****U****U**..**C****A****U****A**.**U****A**..**U****U****G****G****G****A****C****U****G**.**C****A****U****U**..GAAUA**A****A****U****G**.**U****A****G****U****A****C****U**G**U****C****A****U****A****A****G****A**.................UUUA........................................-**U****U****U****U****A****U****G****G**.AGAG**C****U****A****U****U****U**GGAGA | |
|  |  | NZ\_AAEQ01000023.1/167154-167335  | CUCAA**A****G****G****U****A****G**AGGCC**G****C****G****A****U****A**.**G****G****A**AAG**A**G**U****A****A****G****C**-.**U****A****U**G............**G****G****A****G****A****U****U****.****.****.****U****A****A****U****G****G**.**A****A****U****C**.UGU**G****A****U**...C**A****U****A****G****G**.**U****U****G****A****A**AGG**G****A****C**.**U****A**.**U****U****G****C**CGA**A****A****U****A****U****A****A****G**.**A****A****U****A**A...**C****C****A****U****C****.****.****.****.**U**U****A****U****U**..**C****A****U****A**.**U****A**..**U****U****G****G****G****A****C****U****G**.**C****A****U****U**..GAAUA**A****A****U****G**.**U****A****G****U****A****C****U**G**U****C****A****U****A****A****G****A**.................UUUA........................................-**U****U****U****U****A****U****G****G**.AGAG**C****U****A****U****U****U**GGAGA | |
|  |  | NZ\_AAER01000019.1/62951-62770  | CUCAA**A****G****G****U****A****G**AGGCC**G****C****G****A****U****A**.**G****G****A**AAG**A**G**U****A****A****G****C**-.**U****A****U**G............**G****G****A****G****A****U****U****.****.****.****U****A****A****U****G****G**.**A****A****U****C**.UGU**G****A****U**...C**A****U****A****G****G**.**U****U****G****A****A**AGG**G****A****C**.**U****A**.**U****U****G****C**CGA**A****A****U****A****U****A****A****G**.**A****A****U****A**A...**C****C****A****U****C****.****.****.****.**U**U****A****U****U**..**C****A****U****A**.**U****A**..**U****U****G****G****G****A****C****U****G**.**C****A****U****U**..GAAUA**A****A****U****G**.**U****A****G****U****A****C****U**G**U****C****A****U****A****A****G****A**.................UUUA........................................-**U****U****U****U****A****U****G****G**.AGAG**C****U****A****U****U****U**GGAGA | |
|  |  | NZ\_AAES01000029.1/167124-167305  | CUCAA**A****G****G****U****A****G**AGGCC**G****C****G****A****U****A**.**G****G****A**AAG**A**G**U****A****A****G****C**-.**U****A****U**G............**G****G****A****G****A****U****U****.****.****.****U****A****A****U****G****G**.**A****A****U****C**.UGU**G****A****U**...C**A****U****A****G****G**.**U****U****G****A****A**AGG**G****A****C**.**U****A**.**U****U****G****C**CGA**A****A****U****A****U****A****A****G**.**A****A****U****A**A...**C****C****A****U****C****.****.****.****.**U**U****A****U****U**..**C****A****U****A**.**U****A**..**U****U****G****G****G****A****C****U****G**.**C****A****U****U**..GAAUA**A****A****U****G**.**U****A****G****U****A****C****U**G**U****C****A****U****A****A****G****A**.................UUUA........................................-**U****U****U****U****A****U****G****G**.AGAG**C****U****A****U****U****U**GGAGA | |
|  |  | NC\_000964.2/2910120-2909941  | GGUGA**A****G****A****U****A****G**AGGU.**G****C****G****A****A**-C**U****U****C**AAG**A**G**U****A****U****G****C****C**.**U****U****U**.............**G****G****A****G****A****A****.****.****.****.****A****G****A****U****G****G**.**A****U****U****C**.UGU**G****A****A**....**A****A****A****G****G**.**C****U****G****A****A**AGG**G****G****A**G**C****G**.**U****C****G****C**CGA**A****G****C****A****A****A****U****A**.**A****A****A****C**C...**C****C****A****U****C****.****.****.****.**G**G****U****A****U**..**U****A****U****U**.**U****G**..**C****U****G****G****C****C****G****U****G**.**C****A****U****U**..GAAUA**A****A****U****G**.**U****A****A****G****G****C****U**G**U****C****A****A****G****A****A****A**.................UCAU........................................-**U****U****U****C****U****U****G****G**.AGGG**C****U****A****U****C****U**CGUUG | |
|  |  | NC\_003909.8/1516721-1516902  | CUCAA**A****G****G****U****A****G**AGGCC**G****C****G****A****U****A**.**G****G****A**AAG**A**G**U****A****A****G****C**-.**U****A****U**G............**G****G****A****G****A****U****U****.****.****.****U****A****A****U****G****G**.**G****A****U****C**.UGU**G****A****U**...C**A****U****A****G****G**.**U****U****G****A****A**AGG**G****A****C**.**U****A**.**U****U****G****C**CGA**A****A****U****A****U****A****A****G**.**A****A****U****A**...A**C****C****A****U****C****U****.****.****.**.**U****A****U****U**..**C****A****U****A**.**U****A**..**U****U****G****G****G****A****C****U****G**.**C****A****U****U**..GAAUA**A****A****U****G**.**U****A****G****U****A****C****U**G**U****C****A****U****A****A****G****A**.................UUUA........................................-**U****U****U****U****A****U****G****G**.AGAG**C****U****A****U****U****U**GGAGA | |
|  |  | NZ\_AAEK01000009.1/12094-11913  | CUCAA**A****G****G****U****A****G**AGGCC**G****C****G****A****U****A**.**G****G****A**AAG**A**G**U****A****A****G****C**-.**U****A****U**G............**G****G****A****G****A****U****U****.****.****.****U****A****A****U****G****G**.**A****A****U****C**.UGU**G****A****U**...C**A****U****A****G****G**.**U****U****G****A****A**AGG**G****A****C**.**U****A**.**U****U****G****C**CGA**A****A****U****A****U****A****A****G**.**G****A****U****A**...A**C****C****A****U****C****U****.****.****.**.**U****A****U****U**..**C****A****U****A**.**U****A**..**U****U****G****G****G****A****C****U****G**.**C****A****U****U**..GAAUA**A****A****U****G**.**U****A****G****U****A****C****U**G**U****C****A****U****A****A****G****A**.................UUUA........................................-**U****U****U****U****A****U****G****G**.AGAG**C****U****A****U****U****U**GGAGA | |
|  |  | NC\_003366.1/2895497-2895328  | AAAAG**A****G****G****U****A****G**AGGC.**G****C****G****A****G****A**.**A****U****C**AAG**A**U**U****A****C****U****A****A**.**A****A****U**.............**G****G****A****G****U****.****.****.****.****.****U****A****A****G****U****A**.**.****.****G****C**.GUA**G****A****A**....**G****U****U****U****U**.**A****G****G****A****A**AGG**G****A****U**.**U****A**.**U****C****G****C**CGA**A****G****U****U****U****U****U****G**.**G****C****U****A**...A**U****A****C****U****U****U****A****.****.**.**A****G****G****C**..**U****A****A****A**.**U****G**..**C****U****G****G****G****G****U****U****G**.**U****A****U****A**..GAAUA**U****A****U****A**.**C****A****A****C****A****C****U**G**U****C****A****C****A**---.................AAA-........................................----**U****G****U****G****G**.AGAG**C****U****A****U****C****A**UCUUA | |
|  |  | NC\_005957.1/1382903-1383084  | CUCAA**A****G****G****U****A****G**AGGCC**G****C****G****A****U****A**.**G****G****A**AAG**A**G**U****A****A****G****C**-.**U****A****U**G............**G****G****A****G****A****U****U****.****.****.****U****A****A****U****G****G**.**A****A****U****C**.UGU**G****A****U**...C**A****U****A****G****G**.**U****U****G****A****A**AGG**G****A****C**.**U****G**.**U****U****G****C**CGA**A****A****U****A****U****A****A****G**.**A****A****U****A**...A**C****C****A****U****C****U****.****.****.**.**U****A****U****U**..**C****A****U****A**.**U****A**..**U****U****G****G****G****A****C****U****G**.**C****A****U****U**..GAAUA**A****A****U****G**.**U****A****G****U****A****C****U**G**U****C****A****U****A****A****G****A**.................UUUA........................................-**U****U****U****U****A****U****G****G**.AGAG**C****U****A****U****U****U**GGAGA | |
|  |  | NC\_006274.1/2909816-2910008  | CGAUG**A****G****G****U****A****G**AGGUU**G****C****A****A****C****U**.**U****U****U**AAG**A**G**U****A****A****A****A****C**.**G****G****A**.............**C****G****A****G****A****U****A****.****.****C****A****G****A****G****A****A**.**U****G****U****C**.UAA**A****A****C**....**U****C****C****G****U**.**U****U****G****A****A**AGG**A****A****A**.**A****G**.**U****U****G****C**CGA**A****G****U****U****U****A****U****A**.**U****U****U****C**...U**U****C****U****C****U****G****.****.****.**.**G****A****A****A**..**U****A****U****G**.**A****G**..**C****U****G****G****G****G****C****U****G**.**U****C****U****C**.CGAA.A**G****G****A****A**.**C****A****G****A****A****C****U**G**U****C****A****C****G****U****U****U**ACAAA............AUUACCGUG...................................U**A****A****A****C****G****U****G****G**.GGUG**C****U****A****U****C****U**UAACG | |
|  |  | NC\_004557.1/585855-586030  | UAGAA**A****G****G****U****A****G**AGGC.**G****C****G****G****U****A**.**U****U****U**AAU**A**G**U****A****U****C****U****G**.**U****A****C**.............**A****G****A****U****A****.****.****.****.****.****.****A****A****A****G****C**.**.****.****A****A**.GAU**G****A****U**....**G****U****A****C****A**.**G****U****G****A****A**AGG**A****A****A**.**U****A**.**U****C****G****C**CGA**A****G****C**-**A****U****G****C**.**A****G****U**-UAAA**G****C****U****U****U****G****A****.****.**U-**A****C****U**..**G****U****A****U**.**G****A**..**C****U****G****G****U****C****U****U****A**.**U****U****U****A**..AAAUA**U****G****A****A**.**U****A****A****G****A****U****U**G**U****C****A****C****A****A****A**-A................UGAA........................................--**U****U****U****G****U****G****G**.AGAG**C****U****A****U****C****A**UUCAA | |
|  |  | NC\_003997.3/2833645-2833837  | CGAUG**A****G****G****U****A****G**AGGUU**G****C****G****A****C****U**.**U****U****U**AAG**A**G**U****A****A****A****A****C**.**G****G****A**.............**C****G****A****G****A****U****A****.****.****C****A****G****A****G****A****A**.**U****G****U****C**.UAA**G****A****C**....**U****C****C****G****U**.**U****U****G****A****A**AGG**A****A****A**.**A****G**.**U****U****G****C**CGA**A****G****U****U****U****A****U****A**.**U****U****U****C**....**U****U****C****U****C****U****G****.****.**.**G****A****A****A**..**U****A****U****G**.**A****G**..**C****U****G****G****G****G****C****U****G**.**U****G****U****C**.UGAA.A**G****G****A****A**.**C****A****G****A****A****C****U**G**U****C****A****C****G****U****U****U**ACAAA............AUUACCGUG...................................U**A****A****A****C****G****U****G****G**.GGUG**C****U****A****U****C****U**UAACG | |
|  |  | NC\_005945.1/2834111-2834303  | CGAUG**A****G****G****U****A****G**AGGUU**G****C****G****A****C****U**.**U****U****U**AAG**A**G**U****A****A****A****A****C**.**G****G****A**.............**C****G****A****G****A****U****A****.****.****C****A****G****A****G****A****A**.**U****G****U****C**.UAA**G****A****C**....**U****C****C****G****U**.**U****U****G****A****A**AGG**A****A****A**.**A****G**.**U****U****G****C**CGA**A****G****U****U****U****A****U****A**.**U****U****U****C**....**U****U****C****U****C****U****G****.****.**.**G****A****A****A**..**U****A****U****G**.**A****G**..**C****U****G****G****G****G****C****U****G**.**U****G****U****C**.UGAA.A**G****G****A****A**.**C****A****G****A****A****C****U**G**U****C****A****C****G****U****U****U**ACAAA............AUUACCGUG...................................U**A****A****A****C****G****U****G****G**.GGUG**C****U****A****U****C****U**UAACG | |
|  |  | NC\_007530.2/2833773-2833965  | CGAUG**A****G****G****U****A****G**AGGUU**G****C****G****A****C****U**.**U****U****U**AAG**A**G**U****A****A****A****A****C**.**G****G****A**.............**C****G****A****G****A****U****A****.****.****C****A****G****A****G****A****A**.**U****G****U****C**.UAA**G****A****C**....**U****C****C****G****U**.**U****U****G****A****A**AGG**A****A****A**.**A****G**.**U****U****G****C**CGA**A****G****U****U****U****A****U****A**.**U****U****U****C**....**U****U****C****U****C****U****G****.****.**.**G****A****A****A**..**U****A****U****G**.**A****G**..**C****U****G****G****G****G****C****U****G**.**U****G****U****C**.UGAA.A**G****G****A****A**.**C****A****G****A****A****C****U**G**U****C****A****C****G****U****U****U**ACAAA............AUUACCGUG...................................U**A****A****A****C****G****U****G****G**.GGUG**C****U****A****U****C****U**UAACG | |
|  |  | NZ\_AAAC02000001.1/3301779-3301971  | CGAUG**A****G****G****U****A****G**AGGUU**G****C****G****A****C****U**.**U****U****U**AAG**A**G**U****A****A****A****A****C**.**G****G****A**.............**C****G****A****G****A****U****A****.****.****C****A****G****A****G****A****A**.**U****G****U****C**.UAA**G****A****C**....**U****C****C****G****U**.**U****U****G****A****A**AGG**A****A****A**.**A****G**.**U****U****G****C**CGA**A****G****U****U****U****A****U****A**.**U****U****U****C**....**U****U****C****U****C****U****G****.****.**.**G****A****A****A**..**U****A****U****G**.**A****G**..**C****U****G****G****G****G****C****U****G**.**U****G****U****C**.UGAA.A**G****G****A****A**.**C****A****G****A****A****C****U**G**U****C****A****C****G****U****U****U**ACAAA............AUUACCGUG...................................U**A****A****A****C****G****U****G****G**.GGUG**C****U****A****U****C****U**UAACG | |
|  |  | NZ\_AAEN01000017.1/69768-69960  | CGAUG**A****G****G****U****A****G**AGGUU**G****C****G****A****C****U**.**U****U****U**AAG**A**G**U****A****A****A****A****C**.**G****G****A**.............**C****G****A****G****A****U****A****.****.****C****A****G****A****G****A****A**.**U****G****U****C**.UAA**G****A****C**....**U****C****C****G****U**.**U****U****G****A****A**AGG**A****A****A**.**A****G**.**U****U****G****C**CGA**A****G****U****U****U****A****U****A**.**U****U****U****C**....**U****U****C****U****C****U****G****.****.**.**G****A****A****A**..**U****A****U****G**.**A****G**..**C****U****G****G****G****G****C****U****G**.**U****G****U****C**.UGAA.A**G****G****A****A**.**C****A****G****A****A****C****U**G**U****C****A****C****G****U****U****U**ACAAA............AUUACCGUG...................................U**A****A****A****C****G****U****G****G**.GGUG**C****U****A****U****C****U**UAACG | |
|  |  | NZ\_AAEO01000022.1/792595-792787  | CGAUG**A****G****G****U****A****G**AGGUU**G****C****G****A****C****U**.**U****U****U**AAG**A**G**U****A****A****A****A****C**.**G****G****A**.............**C****G****A****G****A****U****A****.****.****C****A****G****A****G****A****A**.**U****G****U****C**.UAA**G****A****C**....**U****C****C****G****U**.**U****U****G****A****A**AGG**A****A****A**.**A****G**.**U****U****G****C**CGA**A****G****U****U****U****A****U****A**.**U****U****U****C**....**U****U****C****U****C****U****G****.****.**.**G****A****A****A**..**U****A****U****G**.**A****G**..**C****U****G****G****G****G****C****U****G**.**U****G****U****C**.UGAA.A**G****G****A****A**.**C****A****G****A****A****C****U**G**U****C****A****C****G****U****U****U**ACAAA............AUUACCGUG...................................U**A****A****A****C****G****U****G****G**.GGUG**C****U****A****U****C****U**UAACG | |
|  |  | NZ\_AAEP01000026.1/119454-119646  | CGAUG**A****G****G****U****A****G**AGGUU**G****C****G****A****C****U**.**U****U****U**AAG**A**G**U****A****A****A****A****C**.**G****G****A**.............**C****G****A****G****A****U****A****.****.****C****A****G****A****G****A****A**.**U****G****U****C**.UAA**G****A****C**....**U****C****C****G****U**.**U****U****G****A****A**AGG**A****A****A**.**A****G**.**U****U****G****C**CGA**A****G****U****U****U****A****U****A**.**U****U****U****C**....**U****U****C****U****C****U****G****.****.**.**G****A****A****A**..**U****A****U****G**.**A****G**..**C****U****G****G****G****G****C****U****G**.**U****G****U****C**.UGAA.A**G****G****A****A**.**C****A****G****A****A****C****U**G**U****C****A****C****G****U****U****U**ACAAA............AUUACCGUG...................................U**A****A****A****C****G****U****G****G**.GGUG**C****U****A****U****C****U**UAACG | |
|  |  | NZ\_AAEQ01000035.1/399734-399542  | CGAUG**A****G****G****U****A****G**AGGUU**G****C****G****A****C****U**.**U****U****U**AAG**A**G**U****A****A****A****A****C**.**G****G****A**.............**C****G****A****G****A****U****A****.****.****C****A****G****A****G****A****A**.**U****G****U****C**.UAA**G****A****C**....**U****C****C****G****U**.**U****U****G****A****A**AGG**A****A****A**.**A****G**.**U****U****G****C**CGA**A****G****U****U****U****A****U****A**.**U****U****U****C**....**U****U****C****U****C****U****G****.****.**.**G****A****A****A**..**U****A****U****G**.**A****G**..**C****U****G****G****G****G****C****U****G**.**U****G****U****C**.UGAA.A**G****G****A****A**.**C****A****G****A****A****C****U**G**U****C****A****C****G****U****U****U**ACAAA............AUUACCGUG...................................U**A****A****A****C****G****U****G****G**.GGUG**C****U****A****U****C****U**UAACG | |
|  |  | NZ\_AAER01000040.1/564904-565096  | CGAUG**A****G****G****U****A****G**AGGUU**G****C****G****A****C****U**.**U****U****U**AAG**A**G**U****A****A****A****A****C**.**G****G****A**.............**C****G****A****G****A****U****A****.****.****C****A****G****A****G****A****A**.**U****G****U****C**.UAA**G****A****C**....**U****C****C****G****U**.**U****U****G****A****A**AGG**A****A****A**.**A****G**.**U****U****G****C**CGA**A****G****U****U****U****A****U****A**.**U****U****U****C**....**U****U****C****U****C****U****G****.****.**.**G****A****A****A**..**U****A****U****G**.**A****G**..**C****U****G****G****G****G****C****U****G**.**U****G****U****C**.UGAA.A**G****G****A****A**.**C****A****G****A****A****C****U**G**U****C****A****C****G****U****U****U**ACAAA............AUUACCGUG...................................U**A****A****A****C****G****U****G****G**.GGUG**C****U****A****U****C****U**UAACG | |
|  |  | NZ\_AAES01000035.1/734409-734601  | CGAUG**A****G****G****U****A****G**AGGUU**G****C****G****A****C****U**.**U****U****U**AAG**A**G**U****A****A****A****A****C**.**G****G****A**.............**C****G****A****G****A****U****A****.****.****C****A****G****A****G****A****A**.**U****G****U****C**.UAA**G****A****C**....**U****C****C****G****U**.**U****U****G****A****A**AGG**A****A****A**.**A****G**.**U****U****G****C**CGA**A****G****U****U****U****A****U****A**.**U****U****U****C**....**U****U****C****U****C****U****G****.****.**.**G****A****A****A**..**U****A****U****G**.**A****G**..**C****U****G****G****G****G****C****U****G**.**U****G****U****C**.UGAA.A**G****G****A****A**.**C****A****G****A****A****C****U**G**U****C****A****C****G****U****U****U**ACAAA............AUUACCGUG...................................U**A****A****A****C****G****U****G****G**.GGUG**C****U****A****U****C****U**UAACG | |
|  |  | NZ\_AABG04000023.1/31025-31202  | AUGCU**U****G****A****U****A****G**AGGC.**G****C****A****G****U****U**.**U****U****C**AAG**A**G**U****A****G****G****C****U**.**U****U****C**.............**U****G****A****A****A****A****G****.****.****.****U****U****U****G****A****G**.**C****U****U****U**.AAU**G****A****C**....**G****A****A****A****G**.**U****U****G****A****A**AGG**G****G****A**.**A****A**.**C****U****G****C**CGA**A****G****G****U****U****G****U****A**.**U****C****U****G**....**C****U****C****A****A****A**-**.****.**.**C****A****G****A**..**U****A****C****A**.**A****U**..**C****U****G****G****U****U****A****U****A**.**C****G****G****U**.UAAG.A**G****C****U****G**.**U****A****U****A****A****C****U**G**U****C****A****U****C****A****A**-U................UUAU........................................--**U****U****G****A****U****G****G**.AGCG**C****U****A****U****C****A**GUUCA | |
|  |  | NC\_000907.1/1655556-1655380  | UACAA**A****A****G****U****A****G**AGGC.**G****C****A****A****U****U**.**A****U****U**AUA**A**G**U****A****U****U****U****U**.**U****U****C**.............**A****G****A****G****U**-**.****.****.****.**-**G****G****A****U****A**.**.**-**A****C**.GAA**G****A****A**....**G****A****A****A****A**.**A****A****G****A****A**AGG**A****A****U**.**A****G**.**U****U****G****C**CGA**A****A****U****C****A****A****A****U**.**A****A****A****A**....**.****G****U****C****G**--**.****.**.**U****U****U****U**..**G****U****U****U**.**G****G**..**U****U****G****G****U****G****G****C****G**.**U****G****C****U**.CGAA.A**G****G****G****G**.**C****G****A****C****A****C****U**G**U****C****A****U****A****G****U****U**UUUCU............GAUU........................................-**A****A****C****U****A****U****G****G**.AGUG**C****U****A****C****G****G**UUGUU | |
|  |  | NC\_007146.1/1391584-1391760  | UACAA**A****A****G****U****A****G**AGGC.**G****C****A****A****U****U**.**A****U****U**AUA**A**G**U****A****U****U****U****U**.**U****U****C**.............**A****G****A****G****U**-**.****.****.****.**-**G****G****A****U****A**.**.**-**A****C**.GAA**G****A****A**....**G****A****A****A****A**.**A****A****G****A****A**AGG**A****A****U**.**A****G**.**U****U****G****C**CGA**A****A****U****C****A****A****A****U**.**A****A****A****A**....**.****G****U****C****G**--**.****.**.**U****U****U****U**..**G****U****U****U**.**G****G**..**U****U****G****G****U****G****G****C****G**.**U****G****C****U**.CGAA.A**G****G****G****G**.**C****G****A****C****A****C****U**G**U****C****A****U****A****G****U****U**UUUCU............GAUU........................................-**A****A****C****U****A****U****G****G**.AGUG**C****U****A****C****G****G**UUGUU | |
|  |  | NZ\_AADO01000003.1/156186-156010  | UACAA**A****A****G****U****A****G**AGGC.**G****C****A****A****U****U**.**A****U****U**AUA**A**G**U****A****U****U****U****U**.**U****U****C**.............**A****G****A****G****U**-**.****.****.****.****.****G****G****A****U****A**.**.**-**A****C**.GAA**G****A****A**....**G****A****A****A****A**.**A****A****G****A****A**AGG**A****A****U**.**A****G**.**U****U****G****C**CGA**A****A****U****C****A****A****A****U**.**A****A****A****A**....**.****G****U****C****G**--**.****.**.**U****U****U****U**..**G****U****U****U**.**G****G**..**U****U****G****G****U****G****G****C****G**.**U****G****C****U**.CGAA.A**G****G****G****G**.**C****G****A****C****A****C****U**G**U****C****A****U****A****G****U****U**UUUCU............GAUU........................................-**A****A****C****U****A****U****G****G**.AGUG**C****U****A****C****G****G**UUGUU | |
|  |  | NZ\_AADP01000001.1/760661-760837  | UACAA**A****A****G****U****A****G**AGGC.**G****C****A****A****U****U**.**A****U****U**AUA**A**G**U****A****U****U****U****U**.**U****U****C**.............**A****G****A****G****U**-**.****.****.****.**-**G****G****A****U****A**.**.**-**A****C**.GAA**G****A****A**....**G****A****A****A****A**.**A****A****G****A****A**AGG**A****A****U**.**A****G**.**U****U****G****C**CGA**A****A****U****C****A****A****A****U**.**A****A****A****A**....**.****G****U****C****G**--**.****.**.**U****U****U****U**..**G****U****U****U**.**G****G**..**U****U****G****G****U****G****G****C****G**.**U****G****C****U**.CGAA.A**G****G****G****G**.**C****G****A****C****A****C****U**G**U****C****A****U****A****G****U****U**UUUCU............GAUU........................................-**A****A****C****U****A****U****G****G**.AGUG**C****U****A****C****G****G**UUGUU | |
|  |  | NZ\_AAET01000127.1/3637-3813  | UACAA**A****A****G****U****A****G**AGGC.**G****C****A****A****U****U**.**A****U****U**AUA**A**G**U****A****U****U****U****U**.**U****U****C**.............**A****G****A****G****U**-**.****.****.****.**-**G****G****A****U****A**.**.**-**A****C**.GAA**G****A****A**....**G****A****A****A****A**.**A****A****G****A****A**AGG**A****A****U**.**A****G**.**U****U****G****C**CGA**A****A****U****C****A****A****A****U**.**A****A****A****A**....**.****G****U****C****G**--**.****.**.**U****U****U****U**..**G****U****U****U**.**G****G**..**U****U****G****G****U****G****G****C****G**.**U****G****C****U**.CGAA.A**G****G****G****G**.**C****G****A****C****A****C****U**G**U****C****A****U****A****G****U****U**UUUCU............GAUU........................................-**A****A****C****U****A****U****G****G**.AGUG**C****U****A****C****G****G**UUGUU | |
|  |  | NC\_005957.1/2899520-2899712  | CGAUG**A****G****G****U****A****G**AGGUU**G****C****G****A****C****U**.**U****U****U**AAG**A**G**U****A****A****A****A****C**.**G****G****A**.............**C****G****A****G****A****U****A****.****.****C****A****G****A****G****A****A**.**U****G****U****C**.UAA**G****A****C**....**U****C****C****G****U**.**U****U****G****A****A**AGG**A****A****A**.**A****G**.**U****U****G****C**CGA**A****G****U****U****U****A****U****A**.**U****U****U****C**....**U****U****C****U****C****U****G****.****.**.**G****A****A****A**..**U****A****U****G**.**A****G**..**C****U****G****G****G****G****C****U****G**.**U****C****U****C**.CGAA.A**G****G****A****A**.**C****A****G****A****A****C****U**G**U****C****A****C****G****U****U****U**ACAAA............AUUACCGUG...................................U**A****A****A****C****G****U****G****G**.GGUG**C****U****A****U****C****U**UAACG | |
|  |  | NC\_006270.2/3365772-3365596  | UAGUG**A****G****G****U****A****G**AGGUU**G****C****G****C****G****G**.**A****U****G**AUG**A**G**U****C****G****C****A****U**.**G****U****G**.............**U****G****A****G****G****C****.****.****.****U****G****.****A****U****G****G**G**.****G****C****C**.GAU**G****A****U**....**C****A****U****A****U**.**G****C****A****A****A**AGG**C****A****U**.**C****A**.**G****C****G****C**CGA**A****G****C****A****U****A****A****G**.**G****A****A**-...G**C****C****A****U****U****C****A****.****.**.-**U****U****C**..**U****U****U****A**.**U****G**..**C****U****G****G****G****U****C****U****G**.**C****A****U****U**..GAAUA**A****G****U****G**.**C****A****G****G****A****C****U**G**C****C****G****C****G****G****G**-.................UAUU........................................--**C****C****C****G****C****G****G**.AGGG**C****U****A****U****C****C**GGAGA | |
|  |  | NC\_006322.1/3365943-3365767  | UAGUG**A****G****G****U****A****G**AGGUU**G****C****G****C****G****G**.**A****U****G**AUG**A**G**U****C****G****C****A****U**.**G****U****G**.............**U****G****A****G****G****C****.****.****.****U****G****.****A****U****G****G**G**.****G****C****C**.GAU**G****A****U**....**C****A****U****A****U**.**G****C****A****A****A**AGG**C****A****U**.**C****A**.**G****C****G****C**CGA**A****G****C****A****U****A****A****G**.**G****A****A**-...G**C****C****A****U****U****C****A****.****.**.-**U****U****C**..**U****U****U****A**.**U****G**..**C****U****G****G****G****U****C****U****G**.**C****A****U****U**..GAAUA**A****G****U****G**.**C****A****G****G****A****C****U**G**C****C****G****C****G****G****G**-.................UAUU........................................--**C****C****C****G****C****G****G**.AGGG**C****U****A****U****C****C**GGAGA | |
|  |  | NZ\_AABO02000002.1/89339-89168  | UACAU**A****U****G****U****A****G**AGGU.**G****C****G****G****C****U**.**G****U****U**AUA**A**G**U****A****A****U****U****U**.**U****U**-.............**U****G****A****G****U**-**.****.****.****.**-**G****G****A****U****A**.**.**-**A****C**.GAU**G****A****A**....-**A****A****A****A**.**A****U****G****A****A**AGG**A****A****U**.**A****G**.**U****U****G****C**CGA**A****A****U****C****A****A****U****U**.**A****A****A****A**....**.****G****U****C****A**--**.****.**.**U****U****U****U**..**A****A****U****U**.**G****G**..**U****U****G****G****G****G****G****C****G**.**U****A****U****U**.CGAA.A**G****A****A****A**.**C****G****U****C****A****C****U**G**U****C****A****U****A****G****U**-A................UUUAUC......................................C-**A****C****U****A****U****G****G**.AGCG**C****U****A****C****U****G**GUUAG | |
|  |  | NZ\_AACJ01000060.1/394-565  | UACAU**A****U****G****U****A****G**AGGU.**G****C****G****G****C****U**.**G****U****U**AUA**A**G**U****A****A****U****U****U**.**U****U**-.............**U****G****A****G****U**-**.****.****.****.**-**G****G****A****U****A**.**.**-**A****C**.GAU**G****A****A**....-**A****A****A****A**.**A****U****G****A****A**AGG**A****A****U**.**A****G**.**U****U****G****C**CGA**A****A****U****C****A****A****U****U**.**A****A****A****A**....**.****G****U****C****A**--**.****.**.**U****U****U****U**..**A****A****U****U**.**G****G**..**U****U****G****G****G****G****G****C****G**.**U****A****U****U**.CGAA.A**G****A****A****A**.**C****G****U****C****A****C****U**G**U****C****A****U****A****G****U**-A................UUUAUC......................................C-**A****C****U****A****U****G****G**.AGCG**C****U****A****C****U****G**GUUAG | |
|  |  | NC\_003909.8/2906644-2906836  | CGAUG**A****G****G****U****A****G**AGGUU**G****C****G****A****C****U**.**U****U****U**AAU**A**G**U****A****A****A****A****C**.**G****G****A**.............**C****G****A****G****A****C****A****.****.****C****A****G****A****G****A****A**.**U****G****U****C**.UUA**G****A****C**....**U****C****C****G****U**.**U****U****G****A****A**AGG**A****A****A**.**A****G**.**U****U****G****C**CGA**A****G****U****U****U****A****U****A**.**U****U****U****C**....**U****U****C****U****C****U****G****.****.**.**G****A****A****A**..**U****A****U****G**.**A****G**..**C****U****G****G****G****G****C****U****G**.**U****C****U****C**.CGAA.A**G****G****A****A**.**C****A****G****A****A****C****U**G**U****C****A****C****G****U****U****U**ACAAA............AUUACCGUG...................................U**A****A****A****C****G****U****G****G**.GGUG**C****U****A****U****C****U**UAACG | |
|  |  | NC\_002663.1/1026545-1026371  | UACUU**G****U****G****U****A****G**AGGA.**G****C****G****A****U****C**.**A****C****U**AUA**A**G**U****A****U****U****U****U**.**U****U****C**.............**U****G****A****G****U**-**.****.****.****.**-**G****G****A****U****A**.**.**-**A****C**.GAA**G****A****G**....**G****A****A****A****A**.**A****G****G****A****A**AGG**A****G****U**.**G****A**.**C****C****G****C**CGA**A****A****U****C****A****A****U****U**.**G****A****A****A**....**.****G****U****C****A**--**.****.**.**U****U****U****U**..**G****A****U****U**.**G****G**..**U****U****G****G****U****G****G****C****G**.**U****A****U****U**.CGAA.A**G****G****A****A**.**C****G****U****C****A****U****U**G**U****C****A****U****A****G****U****C**UUU..............UUUA........................................-**A****A****C****U****A****U****G****G**.AGCG**C****U****A****C****U****G**GUUGG | |
|  |  | NC\_006582.1/3075743-3075570  | AGAUG**G****G****G****U****A****G**AGGC.**G****C****A****A****A****C**.**A****C****C**AUC**A**G**U****A****C****C****U****U**.**U****U****U**.............**U****G****A****G****C****G****.****.****.****.****A****A****G****G****C****C**.**.****C****G****C**.UUU**G****A****A**....**G****A****A****A****A**.**G****G****G****A****A**AGG**G****G****U**.**U****U**.**U****U****G****C**CGA**A****G****C**-**A****A****A****A**.**A****G****C****A**G...**G****G****C****C****U****G****.****.****.**C**U****G****C****U**..**U****U****U****U**.**A****G**..**C****U****G****G****G****C****U****G****G**.**C****A****U****U**..GAAGA**A****G****U****G**.**U****C****G****G****A****C****U**G**U****C****A****C****A****G**--.................GGAA........................................---**C****U****G****U****G****G**.AGAA**C****U****A****C****U****A**UGGAU | |
|  |  | NC\_004347.1/1041892-1042063  | CCUUU**A****A****G****U****A****G**AGGC.**G****C****G****C****U****G**.**C****C****U**AUG**A**C**U****A****C****U****U****G**.**U****G****C**.............**G****G****A****G****G**-**.****.****.****.****G****U****G****A****U****G**.**.**-**C****C**.GCA**G****A****U**....**G****U****A****C****A**.**A****G****G****A****A**AGG**A****G****U**.**C****A**.**G****C****G****C**CGA**A****G****U****A****G****C****C****A**.**G****G****U**-....**C****A****U****C****A****A**-**.****.**.-**A****C****C**..**G****A****G****C**.**U****G**..**C****U****G****G****U****U****U****U****G**.**C****A****U****C**..AAAUA**G****G****U****G**.**C****A****A****G****A****C****U**G**C****C****A****U****A****G****U**-C................AUCC........................................--**A****C****U****A****U****G****G**.AGCG**C****U****A****C****C****U**GAAGG | |
|  |  | NC\_003869.1/1930427-1930616  | AGGUG**A****G****G****U****A****G**AGGC.**G****C****G****G****G****U**C**A****U****C**AAG**A**G**U****A****A****C****A****U**.**G****C****C**.............**A****G****A****G****G****.****.****U****G****U****U****A****A****G****G****G**.**.****.****C****C**.GAU**G****A****A**....**G****G****U****G****U**.**G****U****G****A****A**AGG**G****G****U**G-**C**.**C****C****G****C**CGA**A****G****C**-**G****C****G****U**.**A****A****A****C**U...**U****C****C****U****U****A****A****.****.**G**G****U****U****U**..**A****C****G****C**.**A****G**..**C****U****G****G****G****C****C****U****A**.**U****G****C****C**..GAACA**G****G****U****A**.**U****A****G****G****A****C****U**G**U****C****A****C****U****G****A****A**GGCUC............CCCAGGC.....................................C**U****U****C****A****G****U****G****G**.AGAG**C****U****A****U****C****U**CGCUA | |
|  |  | NC\_002570.2/3211738-3211547  | AGUGA**U****G****G****U****A****G**AGGU.**G****C****G****A****A****A**.**A****C****C**AAG**A**G**U****A****C****A****C****A**.**G****U****C**.............**U****G****A****G****A****G****.****.****A****A****A****U****G****A****G****A**.**.****A****U****C**.GUU**G****A****C**....**G****A****C****U****G**U**U****G****G****A****A**AGG**G****G****G**.**A****U**.**U****C****G****C**CGA**A****G****U****G****C****A****G****A**.**U****C****G****G**G...**G****C****U****C****A****U****U****.****.**C**C****C****.****A**..**U****U****U****G**.**C****G**..**C****U****G****G****A****C****C****U****A**.**U****G****U****U**..GAAUA**A****G****C****A**.**U****A****G****G****G****C****U**G**U****C****A****C****A****A****C****A**CUAGCCCCAA.......CUAG........................................-**U****G****C****U****G****U****G****G**.AGAA**C****U****A****U****C****U**CACGU | |
|  |  | NC\_002506.1/232296-232124  | CCUUU**A****A****G****U****A****G**AGGC.**G****C****G****C****U****G**.**U****U****C**AUG**A**G**U****C****G****C****C****A**.**G****U****C**.............**G****U****A****G****G**-**.****.****.****.****U****U****G****A****C****C**.**.**-**C****C**.GAU**G****A****U**....**G****A****C****U****G**.**G****U****U****A****A**AGG**G****U****A**.**C****A**.**G****C****G****C**CGA**A****G****U****G****A****U****C****G**.**U****U****G**-....**C****G****U****C****A****U**-**.****.**.-**C****A****A**..**C****G****U****U**.**C****G**..**C****U****G****G****G****C****C****A****G**.**C****A****U****U**..GAACA**A****A****U****G**.**C****C****G****G****A****C****U**G**C****C****A****U****A****G****U**-GU...............GUUG........................................--**U****C****U****A****U****G****G**.AGCG**C****U****A****C****C****U**UGAAG | |
|  |  | NZ\_AAEK01000031.1/60689-60881  | CGAUG**A****G****G****U****A****G**AGGUU**G****C****G****A****C****U**.**U****U****U**AAG**A**G**U****A****A****A****A****C**.**G****G****A**.............**C****G****A****G****A****C****A****.****.****C****A****G****A****G****A****A**.**A****G****U****C**.ACC**G****A****C**....**U****C****C****G****U**.**U****U****G****A****A**AGG**A****A****A**.**A****G**.**U****U****G****C**CGA**A****G****U****U****U****A****U****A**.**U****U****U****C**....**U****U****C****U****C****U****G****.****.**.**G****A****A****A**..**U****A****U****G**.**A****G**..**C****U****G****G****G****G****C****U****G**.**U****C****U****C**.CGAA.A**G****G****A****A**.**C****A****G****A****A****C****U**G**U****C****A****C****G****U****U****U**ACAAA............AUUACCGUG...................................U**A****A****A****C****G****U****G****G**.GGUG**C****U****A****U****C****U**UAACG | |
|  |  | NC\_002745.2/1713917-1713742  | AAUUG**A****G****U****U****A****G**AGGUU**G****C****A****U****G****U**.**U****U****A**AUU**A**G**U****A****A****C****U****U**.**G****U**-C............**A****G****A****A****G****U****A****.****.****U****U****U****A****U****G****G**.**U****A****C****A**.UAA**G****U****U**...G**A****A****C****A****A**.**G****U****G****A****A**AGG**U****A****A**.**A****G**.**A****U****G****C**CGA**A****A****U****A****G****A****U****A**.**U****A****A****A**....**C****C****A****U****A****A****A****.****.**.-**U****U****A**..**U****A****U****C**.**U****A**..**U****U****G****G****G****A****C****A****G**.**U****U****U****U**.CGAAUA**G****G****A****A**.**C****U****G****U****A****C****U**G**U****C****A****C****A**---.................GAA-........................................----**U****G****U****G****A**.UGUG**C****U****A****C****C****U**UAUAU | |
|  |  | NC\_002758.2/1790310-1790135  | AAUUG**A****G****U****U****A****G**AGGUU**G****C****A****U****G****U**.**U****U****A**AUU**A**G**U****A****A****C****U****U**.**G****U**-C............**A****G****A****A****G****U****A****.****.****U****U****U****A****U****G****G**.**U****A****C****A**.UAA**G****U****U**...G**A****A****C****A****A**.**G****U****G****A****A**AGG**U****A****A**.**A****G**.**A****U****G****C**CGA**A****A****U****A****G****A****U****A**.**U****A****A****A**....**C****C****A****U****A****A****A****.****.**.-**U****U****A**..**U****A****U****C**.**U****A**..**U****U****G****G****G****A****C****A****G**.**U****U****U****U**.CGAAUA**G****G****A****A**.**C****U****G****U****A****C****U**G**U****C****A****C****A**---.................GAA-........................................----**U****G****U****G****A**.UGUG**C****U****A****C****C****U**UAUAU | |
|  |  | NC\_002951.2/1759610-1759435  | AAUUG**A****G****U****U****A****G**AGGUU**G****C****A****U****G****U**.**U****U****A**AUU**A**G**U****A****A****C****U****U**.**G****U**-C............**A****G****A****A****G****U****A****.****.****U****U****U****A****U****G****G**.**U****A****C****A**.UAA**G****U****U**...G**A****A****C****A****A**.**G****U****G****A****A**AGG**U****A****A**.**A****G**.**A****U****G****C**CGA**A****A****U****A****G****A****U****A**.**U****A****A****A**....**C****C****A****U****A****A****A****.****.**.-**U****U****A**..**U****A****U****C**.**U****A**..**U****U****G****G****G****A****C****A****G**.**U****U****U****U**.CGAAUA**G****G****A****A**.**C****U****G****U****A****C****U**G**U****C****A****C****A**---.................GAA-........................................----**U****G****U****G****A**.UGUG**C****U****A****C****C****U**UAUAU | |
|  |  | NC\_002952.2/1824557-1824382  | AAUUG**A****G****U****U****A****G**AGGUU**G****C****A****U****G****U**.**U****U****A**AUU**A**G**U****A****A****C****U****U**.**G****U**-C............**A****G****A****A****G****U****A****.****.****U****U****U****A****U****G****G**.**U****A****C****A**.UAA**G****U****U**...G**A****A****C****A****A**.**G****U****G****A****A**AGG**U****A****A**.**A****G**.**A****U****G****C**CGA**A****A****U****A****G****A****U****A**.**U****A****A****A**....**C****C****A****U****A****A****A****.****.**.-**U****U****A**..**U****A****U****C**.**U****A**..**U****U****G****G****G****A****C****A****G**.**U****U****U****U**.CGAAUA**G****G****A****A**.**C****U****G****U****A****C****U**G**U****C****A****C****A**---.................GAA-........................................----**U****G****U****G****A**.UGUG**C****U****A****C****C****U**UAUAU | |
|  |  | NC\_002953.3/1732590-1732415  | AAUUG**A****G****U****U****A****G**AGGUU**G****C****A****U****G****U**.**U****U****A**AUU**A**G**U****A****A****C****U****U**.**G****U**-C............**A****G****A****A****G****U****A****.****.****U****U****U****A****U****G****G**.**U****A****C****A**.UAA**G****U****U**...G**A****A****C****A****A**.**G****U****G****A****A**AGG**U****A****A**.**A****G**.**A****U****G****C**CGA**A****A****U****A****G****A****U****A**.**U****A****A****A**....**C****C****A****U****A****A****A****.****.**.-**U****U****A**..**U****A****U****C**.**U****A**..**U****U****G****G****G****A****C****A****G**.**U****U****U****U**.CGAAUA**G****G****A****A**.**C****U****G****U****A****C****U**G**U****C****A****C****A**---.................GAA-........................................----**U****G****U****G****A**.UGUG**C****U****A****C****C****U**UAUAU | |
|  |  | NC\_003923.1/1752938-1752763  | AAUUG**A****G****U****U****A****G**AGGUU**G****C****A****U****G****U**.**U****U****A**AUU**A**G**U****A****A****C****U****U**.**G****U**-C............**A****G****A****A****G****U****A****.****.****U****U****U****A****U****G****G**.**U****A****C****A**.UAA**G****U****U**...G**A****A****C****A****A**.**G****U****G****A****A**AGG**U****A****A**.**A****G**.**A****U****G****C**CGA**A****A****U****A****G****A****U****A**.**U****A****A****A**....**C****C****A****U****A****A****A****.****.**.-**U****U****A**..**U****A****U****C**.**U****A**..**U****U****G****G****G****A****C****A****G**.**U****U****U****U**.CGAAUA**G****G****A****A**.**C****U****G****U****A****C****U**G**U****C****A****C****A**---.................GAA-........................................----**U****G****U****G****A**.UGUG**C****U****A****C****C****U**UAUAU | |
|  |  | NC\_006370.1/1201127-1201310  | ACUAA**G****G****G****U****A****G**AGGC.**G****C****A****U****U****G**.**U****C****U**AUA**A**G**U****A****A****C****A****A**.**G****C****U**.............**G****G****A****G****G**-**.****.****.****.****G****U****G****A****U****U**.**.**-**C****C**.UAU**G****A****U**....**U****G****C****U****U**.**G****U****G****A****A**AGG**A****G****C**.**C****A**.**A****U****G****C**CGA**A****G****U****A****A****G****A****A**G**G****C****G****U**...U**A****A****U****C****G****A****.****.****.**.**A****C****G****.**..**U****U****C****U**.**U****G**..**C****U****G****G****G****G****U****U****G**.**U****A****U****C**..GAAUA**G****G****U****G**.**C****A****A****C****A****C****U**G**C****C****A****U****A****G****U****A**.................UAUAAUUACAU.................................U**A****A****C****U****A****U****G****G**.AGCG**C****U****A****C****U****G**UGAGG | |
|  |  | NC\_004459.1/2989398-2989219  | UAUCG**A****C****G****U****A****G**AGGC.**G****C****A****A****U****G**.**G****U****A**AAG**A**G**U****A****A****C****U****A**.**U****U****A**.............**U****U****G****G****G**-**.****.****.****.****G****U****G****A****U****G**.**.**-**C****C**.AAU**G****A****A**....**U****A****A****U****A**.**G****U****G****A****A**AGG**U****A****U**C**C****A**.**U****U****G****C**CGA**A****G****U****G****A****A****U****U**.**G****C****A****.**....**U****A****U****C****A****A****.****.****.**.**.****A****G****C**..**A****G****U****U**.**U****G**..**C****U****G****G****G****G****U****U****G**.**C****A****U****C**.CGAA.A**G****G****A****A**.**C****A****A****C****A****C****U**G**C****C****A****U****A****G****U****A**U................UUAAUGUA....................................U**A****A****C****U****A****U****G****G**.AGCG**C****U****A****C****U****G**UAGGU | |
|  |  | NC\_005139.1/1373591-1373770  | UAUCG**A****C****G****U****A****G**AGGC.**G****C****A****A****U****G**.**G****U****A**AAG**A**G**U****A****A****C****U****A**.**U****U****A**.............**U****U****G****G****G**-**.****.****.****.****G****U****G****A****U****G**.**.**-**C****C**.AAU**G****A****A**....**U****A****A****U****A**.**G****U****G****A****A**AGG**U****A****U**C**C****A**.**U****U****G****C**CGA**A****G****U****G****A****A****U****U**.**G****C****A****.**....**U****A****U****C****A****A****.****.****.**.**.****A****G****C**..**A****G****U****U**.**U****G**..**C****U****G****G****G****G****U****U****G**.**C****A****U****C**.CGAA.A**G****G****A****A**.**C****A****A****C****A****C****U**G**C****C****A****U****A****G****U****A**U................UUAAUGUA....................................U**A****A****C****U****A****U****G****G**.AGCG**C****U****A****C****U****G**UAGGU | |
|  |  | NC\_003366.1/742872-743043  | GACCA**A****A****G****U****A****G**AGGU.**G****C****C****G****U****A**.**A****U****U**AAG**A**G**U****A****G****U****C****A**.**U****A****A**.............**G****U****A****G****C**-**.****.****.****.****U****G****A****C****A****A**.**G****U****G****U**.-UU**U****A****U**....**G****U****A****U****G**.**A****U****G****A****A**AGG**G****A****U**.**U****A**.**U****G****G****C**CGA**A****G****A****G****A****U****A****U**.**U****A****A**-....**U****G****G****U****G****A**-**.****.**.-**U****U****A**..**A****U****A****U**.**U****U**..**C****U****G****G****G****U****A****U****A**.**U****G****U****A**..UAAUA**U****G****C****A**.**U****A****U****A****A****C****U**G**U****C****A****C****U****U****U**-.................GAAA........................................--**A****A****A****G****U****G****G**.AGUG**C****U****A****C****A****A**GGUAC | |
|  |  | NC\_006840.1/1836942-1836759  | UAAAA**G****G****G****U****A****G**AGGC.**G****C****A****C****C****G**.**C****C****U**AAG**A**G**U****A****A****A****U****U**.**G****U****A**.............**A****G****A****G****G**-**.****.****.****.****G****U****G****A****U****A**.**.**-**C****U**.UAU**G****A****C**....**G****A****C****A****A**.**U****U****G****A****A**AGG**A****G****U**.**C****G**.**G****U****G****C**CGA**A****G****U****U****A****A****U****U**.**A****C****G****U**U...**A****A****U****C****G****A****.****.****.**.**G****C****G****U**..-**A****U****U**.**A****G**..**C****U****G****G****G****G****U****U****A**.**U****U****U****C**..GAAGA**G****G****A****A**.**U****A****A****C****A****C****U**G**C****C****A****U****A****G****U****C**AAU..............UUUAUUUUU...................................U**A****A****C****U****A****U****G****G**.AGCG**C****U****A****C****U****G**UAGGG | |
|  |  | NC\_004603.1/1190723-1190903  | UGUUG**C****C****G****U****A****G**AGGC.**G****C****A****G****U****C**.**U****C****G**AAG**A**G**U****A****G****C****U****A**.**U****U****A**.............**U****U****G****G****G**-**.****.****.****.****G****U****G****A****U****G**.**.**-**C****C**.AAU**G****A****A**....**U****A****A****U****A**.**G****U****G****G****A**AGG**C****G****A**A**G****A**.**U****U****G****C**CGA**A****G****U****A****A****G****U****C**.**G****U****A****.**....**U****A****U****C****A****A****.****.****.**.**.****A****A****C**..**G****C****C****U**.**U****G**..**C****U****G****G****G****G****U****U****G**.**U****A****U****C**.UGAA.A**G****G****A****A**.**C****A****A****C****A****C****U**G**C****C****A****U****A****G****U****A**UA...............UUUACAUU....................................A**A****A****C****U****A****U****G****G**.AGCG**C****U****A****C****U****G**UAGGG | |
|  |  | NC\_004193.1/2421341-2421165  | CGGUG**A****G****G****U****A****G**AGGA.**G****C****A****U****A****C**.**A****A****C**AUU**A**G**U****A****A****U****C****G**.**A****C****A**.............**A****G****A****G****G****.****.****.****.****.****A****U****G****A****C****A**.**.****.****A****C**.GAU**G****A****U**....**A****G****U****U****G**.**G****U****G****G****A**AGG**G****U****U**.**G****U**.**U****U****G****C**CGA**A****G****C**-**A****U****A****A**.**U****A****A****G**....**G****G****U****C****A****G****A****.****.**.**C****U****U****A**..**U****U****A****U**.**U****G**..**C****U****G****G****U****A****C****A****U**.**C****U****U****U**..GAAUA**A****A****A****G**.**A****U****G****C****A****C****U**G**U****C****A****U****G****C****A**-AAAU.............UAAG........................................--**U****G****C****A****U****G****G**.AGAA**C****U****A****C****U****G**AUCGA | |
|  |  | NC\_000964.2/3420378-3420199  | CAGUG**A****G****G****U****A****G**AGGUU**G****C****G****C****G****G**.**A****U****G**AUG**A**G**U****C****A****C****A****C**.**A****U****G**.............**C****U****A****G****G****C****U****.****.****.****G****A****C****A****G****G**.**G****G****C****U**.GUU**A****A****A**....**C****A****U****G****U**.**G****U****A****A****A**AGG**C****A****U**.**C****A**.**G****C****G****C**CGA**A****G****U****G****U****G****G****A**.**G****A****A****A**G...**C****C****G****A****U****C****.****.****.**C**.****U****U****C**..**U****C****U****A**.**U****G**..**C****U****G****G****G****A****C****U****G**.**U****A****U****C**.UGAAUA**A****G****U****G**.**C****A****G****G****A****C****U**G**C****C****G****C****G****U****G**-.................CUUU........................................--**U****U****C****G****C****G****G**.AGGG**C****U****A****U****C****C**GGAGA | |
|  |  | NC\_004193.1/1752855-1753037  | GUUUU**G****G****A****U****A****G**AGGU.**G****C****G****G****A****G**.**A****C****C**AUC**A**G**U****A****U****A****U****A**.**C****G****C**.............**G****G****A****A****G****G****G****.****.****A****A****A****U****G****A****G**.**C****C****C****U**.AGU**G****A****A**....**G****C****G****U****A**.**U****G****G****A****A**AGG**G****G****A**.**A****U**.**C****U****G****C**CGA**A****G****C****G****A****G****U****G**.**A****A****A****U**...A**C****U****C****A****U****U****.****.****.**.**C****A****U****U**..**A****A****C****U**.**C****G**..**U****U****G****G****U****G****C****U****G**C**U****A****U****U**..GAACA**A****A****U****A**A**C****A****G****U****G****C****U**G**U****C****A****U****A****U****A****G**.................GAGA........................................-**C****U****A****U****A****U****G****G**.AGGG**C****U****A****U****C****G**AGCUG | |
|  |  | NC\_004722.1/3009682-3009874  | CGAUG**A****G****G****U****A****G**AGGUU**G****C****G****A****C****U**.**U****U****U**AAG**A**G**U****A****C****G****A****C**.**G****G****A**.............**C****G****A****G****A****C****A****.****.****C****A****G****A****G****A****A**.**U****G****U****C**.ACC**G****A****C**....**U****C****C****G****U**.**U****U****G****A****A**AGG**A****A****A**.**A****G**.**U****U****G****C**CGA**A****G****U****U****U****A****U****A**.**U****U****U****C**....**U****U****C****U****C****U****G****.****.**.**G****A****A****A**..**U****A****U****G**.**A****G**..**C****U****G****G****G****G****C****U****G**.**U****C****U****C**.CGAA.A**G****G****A****A**.**C****A****G****A****A****C****U**G**U****C****A****C****G****U****U****U**ACAAA............AUUACCGUG...................................U**A****A****A****C****G****U****G****G**.GGUG**C****U****A****U****C****U**UAACG | |
|  |  | NZ\_AACK01000021.1/33603-33432  | ACAAA**U****C****G****U****A****G**AGGU.**G****C****A****A****A****U**.**C****C****G**AUA**A**G**U****A****C****U****U****U**.**U****U****C**.............**U****G****A****G****U**-**.****.****.****.**-**G****G****A****G****A**.**.**-**A****C**.GAG**G****A****G**....**G****A****A****A****A**.**A****G****G****A****A**AGG**C****G****U**.**A****U**.**U****U****G****C**CGA**A****A****U****C****A****G****U****U**.**A****A****G**-....**C****G****U****C****A****U**-**.****.**.-**C****U****U**..**A****A****U****U**.**G****G**..**U****U****G****G****G****G****U****C****G**.**U****U****A****C**.CGAA.A**G****G****G****A**.**C****G****A****C****A****C****U**G**U****C****G****U****A****A****U**-CC...............UUGU........................................--**A****U****U****A****C****G****G**.AGUG**C****U****A****C****U****G**CUAGG | |
|  |  | NC\_002940.2/992934-993104  | ACAAA**U****U****G****U****A****G**AGGU.**G****C****A****A****A****U**.**C****C****G**AUA**A**G**U****A****U****U****U****C**.**U****U****C**.............**U****G****A****G****U**-**.****.****.****.**-**G****G****A****A****A**.**.**-**G****C**.GAU**G****A****A**....**G****G****G****G****A**.**A****G****G****A****A**AGG**C****G****U**.**A****U**.**U****U****G****C**CGA**A****A****U****C****A****A****U****U**.**A****A****G**-....**C****G****U****C****A****U**-**.****.**.-**C****U****U**..**A****G****U****U**.**G****G**..**U****U****G****G****G****G****U****C****G**.**U****U****G****C**.CGAA.A**G****G****G****A**.**C****G****A****C****A****C****U**G**U****C****G****U****A****A****U**-U................CAAU........................................--**A****U****U****A****C****G****G**.AGUG**C****U****A****C****U****A**UUAGG | |
|  |  | NC\_002976.3/1281544-1281369  | AAUAG**A****G****U****U****A****G**AGGUU**G****C****A****U****U****A**.**U****U****A**AUG**A**C**U****A****A****C****U****U**.**A****U**-C............**A****G****A****A****G****U****C****.****.****.****G****U****A****U****G****G**.**G****A****C****A**.UGU**G****U****U**...G**A****A****U****A****A**.**G****U****G****A****A**AGG**U****A****A**.**U****A**.**A****U****G****C**CGA**A****A****U****G****A****U****G****U**.**U****A****U****U**U...**C****C****A****U****A****A****.****.****.**.**A****U****U****A**..**G****C****A****U**.**U****G**..**U****U****G****G****G****A****C****A****A**.**C****U****U****U**.CGAAUA**G****A****A****G**.**U****U****G****U****A****C****U**G**U****C****A****C**----U................UUAU........................................-----**G****U****G****A**.UGUG**C****U****A****C****C****U**UAUAU | |
|  |  | NC\_004461.1/1386907-1386732  | AAUAG**A****G****U****U****A****G**AGGUU**G****C****A****U****U****A**.**U****U****A**AUG**A**C**U****A****A****C****U****U**.**A****U**-C............**A****G****A****A****G****U****C****.****.****.****G****U****A****U****G****G**.**G****A****C****A**.UGU**G****U****U**...G**A****A****U****A****A**.**G****U****G****A****A**AGG**U****A****A**.**U****A**.**A****U****G****C**CGA**A****A****U****G****A****U****G****U**.**U****A****U****U**U...**C****C****A****U****A****A****.****.****.**.**A****U****U****A**..**G****C****A****U**.**U****G**..**U****U****G****G****G****A****C****A****A**.**C****U****U****U**.CGAAUA**G****A****A****G**.**U****U****G****U****A****C****U**G**U****C****A****C**----U................UUAU........................................-----**G****U****G****A**.UGUG**C****U****A****C****C****U**UAUAU | |
|  |  | NC\_006270.2/2878019-2877840  | GGUGA**A****G****A****U****A****G**AGGU.**G****C****G****A****A**-C**U****U****C**AAG**A**G**U****A****G****G****C****U**.**U****G****A**.............**U****G****A****G****G****A****A****.****.****G****A****.****U****G****G****A**.**U****U****C****C**.GAU**G****A****A**....**G****A****A****A****G**.**C****C****G****A****A**AGG**G****G****A**G**C****G**.**U****C****G****C**CGA**A****G****C****G****G****G****G****A**.**A****A****A****A**....**U****C****C****A****C****U****C****.****.**.**G****U****U****U**..**U****U****C****C**.**U****G**..**C****U****G****G****C****U****U****U****A**.**C****A****U****U**..GAAUA**A****A****U****G**.**U****G****A****G****G****C****U**G**U****C****A****A****G****A****A****A**.................UCA-........................................-**U****U****U****C****U****U****G****G**.AGAG**C****U****A****U****C****U**CGUUG | |
|  |  | NC\_006322.1/2878230-2878051  | GGUGA**A****G****A****U****A****G**AGGU.**G****C****G****A****A**-C**U****U****C**AAG**A**G**U****A****G****G****C****U**.**U****G****A**.............**U****G****A****G****G****A****A****.****.****G****A****.****U****G****G****A**.**U****U****C****C**.GAU**G****A****A**....**G****A****A****A****G**.**C****C****G****A****A**AGG**G****G****A**G**C****G**.**U****C****G****C**CGA**A****G****C****G****G****G****G****A**.**A****A****A****A**....**U****C****C****A****C****U****C****.****.**.**G****U****U****U**..**U****U****C****C**.**U****G**..**C****U****G****G****C****U****U****U****A**.**C****A****U****U**..GAAUA**A****A****U****G**.**U****G****A****G****G****C****U**G**U****C****A****A****G****A****A****A**.................UCA-........................................-**U****U****U****C****U****U****G****G**.AGAG**C****U****A****U****C****U**CGUUG | |
|  |  | NC\_004557.1/2605606-2605439  | ACCCA**G****G****G****U****A****G**AGGA.**G****C****U****A****U****A**.**A****U****U**AAG**A**G**U****A****C****U****U****A**.**U****C****U**.............**U****A****A****A****C****.****.****.****.****.****U****G****C****C****A****A**.**.****.****G****U**.AAU**G****A****U**....**A****G****A****U****A**.**G****G****A****A****A**AGG**A****A****U**.**U****A**.**U****A****G****C**CGA**A****G****A****A****A****A****U****A**.**G****G****.****.**....**G****U****G****G****U****A****A****.****.**.**.****.****U****C**..**U****A****U****U**.**U****U**..**C****U****G****G****C****U****U****U****G**.**C****A****U****A**..AAAUA**U****G****U****G**.**U****A****A****A****G****C****U**G**U****C****A****C****U****U****U**-.................AA--........................................--**A****A****A****G****U****G****U**.UGAG**C****U****A****C****A****G**AGGUC | |
|  |  | NC\_002976.3/981298-981477  | AGAUU**U****U****G****A****U****G**AGGC.**G****C****A****U****C****A**.**A****U****C**AUG**A**G**U****A****A****A****C****U**.**U****U****A**G..........AU**A****A****U****U****U****G****U****.****.****.****C****U****G****C****U****A**.**A****C****A****A**....**.****U****U**A...**U****A****G****A****G**.**U****U****A****A****A**AGG**G****U****G**.**A****G**.**A****U****G****C**CGA**A****A****U****G****A****U****U****C**.**A****U****A****A**....**U****A****G****C****A****G**-**.****.**.**U****U****A****U**..**G****A****A****U**.**C****G**..**U****U****G****G****A****C****U****U****A**A**U****G****G****U**..UAAGA**G****C****U****A**.**U****A****A****G****U****U****U**G**U****C****A****U****U****A****U****U**.................AUUA........................................-**A****A****U****A****A****U****G****G**.AGUG**C****A****U****C****A****C**UUGUA | |
|  |  | NC\_004461.1/1086256-1086435  | AGAUU**U****U****G****A****U****G**AGGC.**G****C****A****U****C****A**.**A****U****C**AUG**A**G**U****A****A****A****C****U**.**U****U****A**G..........AU**A****A****U****U****U****G****U****.****.****.****C****U****G****C****U****A**.**A****C****A****A**....**.****U****U**A...**U****A****G****A****G**.**U****U****A****A****A**AGG**G****U****G**.**A****G**.**A****U****G****C**CGA**A****A****U****G****A****U****U****C**.**A****U****A****A**....**U****A****G****C****A****G**-**.****.**.**U****U****A****U**..**G****A****A****U**.**C****G**..**U****U****G****G****A****C****U****U****A**A**U****G****G****U**..UAAGA**G****C****U****A**.**U****A****A****G****U****U****U**G**U****C****A****U****U****A****U****U**.................AUUA........................................-**A****A****U****A****A****U****G****G**.AGUG**C****A****U****C****A****C**UUGUA | |
|  |  | NC\_002505.1/1198970-1199152  | UUUCG**C****C****G****U****A****G**AGGA.**G****C****G****G****U****U**.**A****C****G**AAA**A**G**U****A****U****C****C****A**.**C****A****G**.............**U****U****G****G****G**-**.****.****.****.****G****U****G****A****U****G**.**.**-**C****C**.AAU**G****A****A**....**U****U****G****U****G**.**G****A****A****A****A**AGG**C****G****U**.**U****G**.**C****C****G****C**CGA**A****G****U****C****A****A****C****U**.**U****G****C**-...C**C****A****U****C****A****A****C****.****.**.-**G****C****A**..-**G****U****U**.**G****G**..**C****U****G****G****G****G****U****U****A**.**C****A****U****U**..CAAUA**G****G****U****G**.**U****A****A****C****A****C****U**G**C****C****A****U****A****G****U****C**UAUAUUGUU........GUUA........................................-**A****A****C****U****A****U****G****G**.AGCG**C****U****A****C****U****G**UAGGG | |
|  |  | NC\_003454.1/361206-361027  | AUAAA**A****A****A****U****A****G**AGGU.**G****C****A****U****A****U**.**A****U****G**-UA**G**G**U****A****G****U****G****U**.**G****A****A**AAUGU........**U****A****A****G****G****U****.****.****.****.****A****U****A****A****G****C**C**.****A****C****C**.AAU**G**--...U**U****U****C****A****C**.**A****U****G****A****A**GGG**C****A****U**.**A****G**.**U****U****G****C**CGA**A****A****G****A****A****A****G****U**.**U****A****A****U**U...**G****C****U****U****A****U****.****.****.**G**A****U****U****A**..**A****U****U****U**.**U****C**..**U****U****G****G****U**-**C****A****A**.**U****G****U**-..CAACA**A****G****C****A**.**U****U****G**-**A****C****U**G**U****C****A****U****A****U****U****C**.................UUUU........................................-**G****A****A****U****A****U****G****G**.AGAG**C****U****A****U****U****U**AUAGU | |
|  |  | NC\_003030.1/3801637-3801809  | ACCUA**G****G****G****U****A****A**AGGU.**G****C****U****G****U****A**.**G****U****U**AUU**A**U**U****A****U****U****U****A**.**U****U****C**.............**U****U****A****G****C****.****.****.****.****.****U****G****G****C****A****A**.**.****.****G****C**.UUU**G****A****G**....**G****G****A****U****A**.**A****A****G****A****A**AGG**A****A****U**.**U****G**.**C****A****G****C**CGA**A****G****A****A****G****G****A****U**.**U****U****C**-....**C****G****G****C****A****G**-**.****.**.-**G****A****A**..**C****U****U****U**.**U****U**..**C****U****G****G****U****U****U****U****G**.**U****A****U****A**..AAAUA**U****A****U****G**.**C****A****G****A****A****C****U**G**U****C****A****C****U****A****U**-UC...............UUUU........................................--**A****U****A****G****U****G****G**.AGAG**C****U****A****C****A****A**GGUGC | |
|  |  | NZ\_AAEV01000005.1/55892-55712  | AAUUA**A****A****A****A****A****G**AGGUU**G****C****G****G****A****G**.**A****U****U**AAA**A**G**U****A****C****C****U****G**.**A****G****G**.............**A****U****A****G****A****A****U****.****.****.****.****G****A****A****G****G**.**A****U****U****C**.GAU**A****U****G**....**C****C****C****C****A**.**G****C****G****A****A**AGG**A****U****G**.**A****U**.**C****C****G****C**CGA**A****A****U****G****A****A****C****A**.**U****U****G****A**....**C****C****U****U****U****C**-**.****.**.**U****C****A****A**..**U****G****U****U**.**U****G**..**U****U****G****G****G****U****C****U****U**.**G****A****G****A**..UAACA**U****C****U****C**.**A****A****G****G****A****C****U**G**U****C****G****C****U****A****U****C**AU...............GACU........................................-**G****A****U****A****G****C****G****G**.GGUG**C****U****U****U****U****C**AAAUC | |
|  |  | NC\_006300.1/1325416-1325239  | UACAC**A****U****G****U****A****G**AGGU.**G****C****G****A****A****U**.**A****U****U**AUA**A**G**U****A****U****U****U****U**.**U****C****C**.............**A****G****A****G****U**-**.****.****.****.**-**G****G****A****U****A**.**.**-**A****C**.AAU**G****A****A**....**G****G****A****A****A**.**U****U****G****A****A**AGG**A****A****U**.**A****U**.**U****U****G****C**CGA**A****A****U****C****A****G****U****U**.**A****A****G**-....**C****G****U****C****A****U**-**.****.**.-**C****U****U**..**A****A****C****U**.**G****G**..**U****U****G****G****U****A****A****C****G**U**C****A****C****C**..GAA.A**G****G****A****A**.**C****G****U****U****A****C****U**G**C****C****A****U****A****G****U****C**A................UUUUUGAU....................................U**A****A****C****U****A****U****G****G**.AGCG**C****U****A****C****U****C**GUGGG | |
|  |  | NC\_000853.1/1519251-1519074  | UGACC**C****G****A****C****G****G**AGGC.**G****C****G****C****C****C**.**G****A****G**AUG**A**G**U****A****G****G****C****U**.**G****U****C**.............**C****C****A****U****C**-**.****.****.****A****G****G****.****G****G****A**.**G**-**G****A**.AUC**G****G****G**....**G****A****C****G****G**.**C****U****G****A****A**AGG**C****G****A**.**G****G**.**G****C****G****C**CGA**A****G****G**-**G****U****G****C**.**A****G****A****G**U...**U****C****C****U****C****C****C****.****.**G**C****U****C****U**..**G****C****A****U**.**G****C**..**C****U****G****G****G****G****G****U****A**.**U****G****G****G**..GAAUA**C****C****C****A**.**U****A****C****C****A****C****U**G**U****C****A****C****G****G****A****G**.................GUCU........................................-**C****U****C****C****G****U****G****G**.AGAG**C****C****G****A****U****C**GGGUC | |
|  |  | NZ\_AADW02000027.1/21929-21745  | CAGUG**G****G****A****U****G****G**AGGUU**G****C****A****G****U****U**.**C****U****U**AUA**A**G**U****A****C****A****G****U**.**C****A****C**.............**G****G****A****U****G****U****C****.****G****U****C****.****A****A****G****C**.**G****G****C****A**.GAU**G****A****G**....**G****U****G****A****U**.**U****G****A****A****A**AGG**A****A****G**.**A****A**.**U****U****G****C**CGA**A****A****U****C****A****A****U****C**.**A****U****C**-....**G****C****U****U****U****G****A****A****.**.-**G****A****U**..**G****A****U****C**.**G****A**..**U****U****G****G****G****A****U****U****A**.**G****A****U****C**..GAACA**G****G****U****C**.**U****A****A****U****A****C****U**G**U****C****A****C****A****G****G****U**CAGA.............UAUG........................................-**A****C****U****U****G****U****G****G**.GGAG**C****U****A****U****C****U**UUCAC | |
|  |  | NZ\_AAIO01000002.1/6335-6531  | CUUAG**A****A****G****U****A****G**AGGC.**G****C****G****C****C****A**.**A****A****U**AUU**A**G**U****A****G****U****G****A**.**U****C****A**.............**G****U****A****G****G**-**.****.****.****.****G****U****G****A****U****U**.**.**-**C****C**.GUU**G****A****U**....**C****G****A****U****U**.**G****C****G****A****A**AGG**A****U****U**.**U****G**.**G****U****G****C**CGA**A****G****U****G****G****U****U****C**.**G****A****U****U**....**U****A****U****C****A****C**-**.****.**.**G****A****U****C**..-**A****A****C**.**U****G**..**C****U****G****G****G****G****U****U****G**.**U****A****C****C**..UAAUA**G****G****U****G**.**C****A****A****C****A****C****U**G**C****C****A****U****A****G****U****A**.................UUUUCCGAUACGGAUAUUGGAAAUAG..................U**U****A****C****U****A****U****G****G**.AGCG**C****U****A****C****U****G**AUAGG | |
|  |  | NC\_004567.1/928916-929097  | AUCGA**A****A****G****A****A****G**AGGAU**G****C****G****G****U****U**.**A****A****C**AAU**A**G**U****A****G****C****C****G**.**G****C****U**.............**G****G****A****A****G****U****G****.****.****.****G****G****U****C****A****C**.**C****A****C****U**.UAU**G****A****A**....**G****G****U****C****A**.**G****U****G****A****A**CGG**G****G****C**.**A****A**.**C****C****G****C**CGA**A****A****U****C****G****A****U****G**.**G****A****U****C**...A**G****U****G****A****C****C**-**.****.**.**G****A****U****U**..**C****A****U****C**.**C****G**..**U****U****G****G****G****C****C****U****U**.**G****G****U****U**..GAAUA**A****A****U****C**.**A****U****G****G****A****C****U**G**U****C****G****C****A****G****C****U**A................GAAU........................................-**A****G****U****U****G****C****G****G**.GGCG**C****U****A****U****C****G**ACGAU | |
|  |  | NZ\_AAAW03000005.1/60351-60177  | GUGAA**U****G****A****U****A****G**AGGC.**G****C****A****G****U****G**U**A****U****U**AAG**A**G**U****A****A****U****U****A**.**U****C****C**.............**C****G****A****G****C**-**.****.****.****.****G****G****G****U****C****A**G**.**-**G****C**.UAG**G****A****C**....**G****G****A****U****A**.**A****G****G****A****A**AGG**A****A****U**.**C****G**.**C****U****G****C**CGA**A****G****C****A****A****A****G****G**.**C****C****C****A**....**G****G****A****C****A****A****.****.****.**.**A****G****G****G**..**U****C****U****U**.**A****G**..**C****U****G****G****U****C****U****U****C**.**C****A****U****U**..GAAAA**A****A****U****G**.**U****A****A****G****A****C****U**G**U****C****A****C****C****G****G**-.................GACU........................................--**C****C****G****G****U****G****G**.AGUG**C****U****A****U****C****U**CGUGA | |
|  |  | NZ\_AADW02000008.1/60264-60089  | CGAUG**A****G****G****U****A****G**AGGC.**G****C****G****G****U****C**.**U****U****G**AUU**A**G**U****A****U****U****U****G**.**G****U****C**.............**U****G****A****G****G****.****.****.****.****.****A****U****G****A****C****A**.**.****.****A****C**.AAA**G****A****C**....**G****A****U****C****G**.**A****A****G****A****A**AGG**U****G****A**.**G****A**.**C****C****G****C**CGA**A****G****C****A****U****C****G****U**.**C****A****U****G**....**U****G****U****C****A****G****A****.****.**.**C****A****U****G**..**A****C****G****A**.**U****G**..**C****U****G****G****U****C****G****A****A**C**C****G****G****G**..AAAGA**C****C****C****G**.**U****U****C****G****A****U****U**G**C****C****A****U****A****C****G**-C................GUAU........................................--**U****G****U****A****U****G****G**.AGCA**C****U****C****C****A****A**UUCGG | |
|  |  | NC\_006370.1/3172735-3172907  | UUAAU**C****A****G****U****A****G**AGGU.**G****C****G****C****U****G**.**C****C****U**AUA**A**G**U****A****A****C****C****G**.**U****G****C**.............**G****G****A****G****G**-**.****.****.****.****A****U****G****A****U****A**.**.**-**C****C**.GAC**G****A****U**....**G****C**-**C****G**.**G****A****G****A****A**AGG**A****G****U**.**C****A**.**G****U****G****C**CGA**A****G****U****A****A****C****U****G**.-**U****U****G**...U**U****A****U****C****A****.**-**.****.**.**C****A****C****U**..**C****A****G****U**.**U****G**..**C****U****G****G****G****U****C****U****G**.**C****G****U****C**..GAAUA**G****G****C****G**.**C****A****G****G****A****C****U**G**C****C****A****U****A****G**--.................UUUUUUU.....................................C--**C****U****A****U****G****G**.AGCG**C****U****A****C****C****U**GAAGG | |
|  |  | NC\_006510.1/984634-984455  | UAAUG**G****G****A****U****A****G**AGGUU**G****C****G****G****U****G**.**A****A****C**AAA**A**G**U****A****G****C****G****C**.**U****G****C**.............**C****G****A****G****G****C****U****.****.****.****C****G****G****A****G****C**.**G****G****U****C**.CAU**G****A****C**....**G****C****A****G****C**.**G****G****G****A****A**AGG**G****U****U**.**C****A**.**C****C****G****C**CGA**A****G****U****U****U****G****C****G**.**G****C****G****C**....**G****C****U****C****C****G**-**.****.**.**G****C****G****G**..**C****G****C****A**.**A****G**..**C****U****G****G****G****C****C****U****G**.**C****G****G****C**.CGAACA**G****G****U****G**.**C****A****G****G****A****C****U**G**C****C****A****U****C****G****C****C**.................UAC-........................................-**G****G****C****G****G****U****G****G**.AGCG**C****U****A****U****C****G**CCAUU | |
|  |  | NC\_006814.1/834695-834865  | GUAAA**U****C****A****U****A****G**AGGC.**G****C****A****A****C****U**.**G****A****C**AAG**A**G**U****A****A****C****A****A**.**U****U****A**.............**U****A****A****G****U****U****.****.****.****.****.****A****G****G****C****A**.**A****A****A****C**.GAU**G****A****U**....**U****A****A****U****U**.**G****U****G****A****A**AGG**G****G****A**.**G****G**.**U****U****G****C**CGA**A****G****C****U****U****A****.****A**.**C****C****A****.**....**C****G****C****C****A****A****.****.****.**.**.****A****G****G**..**U****A****U****A**.**G****G**..**C****U****G****G****G****U****C****G****C**.**A****G****U****U**..GAAUA**A****A****U****U**.**G****C****G****A****A****C****U**G**U****C****G****C****G****A****A**-.................AAU-........................................--**U****U****C****G****C****G****G**.AGCG**C****U****A****U****C****G**AAUAU | |
|  |  | NZ\_AAEV01000001.1/199067-198890  | AUCGA**A****A****G****A****A****G**AGGUU**G****C****C****U****C****A**.**A****U****U**AUU**A**G**U****A****G****A****U****C**.**C****G****G**.............**C****U****A****G****A****U****G****.****.****.****A****C****U****A****A****G**.**C****A****U****C**.AAU**G****A****C**....**C****C****G****A****A**.**U****U****G****A****A**AGG**A****A****A**.**A****G**.**A****G****G****C**CGA**A****A****U****G****A****C****U****A**.**C****A****U****G**....**C****U****U****A****U****U**-**.****.**.**C****A****U****G**..**U****A**--.**C****G**AU**U****U****G****G****G****C****U****C****U**.**G****G****U****U**..GAAUA**A****G****C****C**.**A****G****A****G****A****C****U**G**U****C****G****C****A****A****U**-.................GAAA........................................--**A****U****U****G****C****G****G**.AGCG**C****U****A****U****C****U**ACGAU | |
|  |  | NZ\_AADT03000018.1/60-231  | CAGUG**G****G****G****U****A****G**AGGU.**G****C****A****G****C****C**.**G****G****C**AAG**A**G**U****A****U****U****A****C**.**C****G****G**.............**U****G****A****G****G****.****.****.****.****.****G****G****G****G****C****C**.**.****.****C****C**.GGU**G****A****A**....**C****U****G****G****U**.**A****A****G****A****A**AGG**G****C****C**U**G****G**.**C****U****G****C**CGA**A****G****G****G****A****G****A****U**.**G****G****C****U**....**G****G****C****C****C****G**-**.****.**.**G****G****C****C**..**G****U****C****U**.**U****C**..**C****U****G****G****G****C****C****G****G**.**U****G****G****C**.UGAAUA**A****G****C****A**.**C****C****G****G****A****C****U**G**U****C****A****C****C****A**--.................UCA-........................................---**C****G****G****U****G****G**.AGCG**C****U****A****C****U****C**ACACG | |
|  |  | NC\_003212.1/817469-817272  | UGGUG**A****G****G****U****A****G**AGGUU**G****C****G****A****G****A**.**U****G****C**ACU**A**G**U****A****A****U****U****U**.**U****U****U**.............**C****G****A****G****G****C****G****A****A****A****C****A****A****A****G****A**.**C****G****C****C**.AAU**G****A****C**....**A****A****A****A****A**.**A****C****G****A****A**CAG**G****U****U**.**A****A**.**U****C****G****C**CGA**A****G****U****G****A****C****U****A**.**U****U****U****U**U...**U****C****U****U****U****G****U****A****U**C**G****A****A****A**..**U****A****G****U**.**U****G**..**U****U****G****G****G****A****C****A****G**.**U****U****U****C**.CUAA.A**G****G****A****G**.**C****U****G****G****A****C****U**G**C****U****A****U****A****A****G****A**A................UUUGUCGAAAU.................................U**U****C****U****U****A****U****A****G**GUGUG**C****U****A****U****C****U**GACAA | |
|  |  | NC\_003030.1/704200-704385  | ACCUU**U****U****G****U****A****G**AGGU.**G****C****U****U****U****A**A**G****U****C**AAG**A**G**U****A****A****C****C****G**.**U****U****U**.............**G****G****A****G****U****.****.****.****.****.****U****G****G****C****A****A**.**.****.****A****C**.UUA**G****A****U**....**G****A****A****C****G**.**G****U****A****A****A**AGG**G****G****C**U**U****U**.**U****A****G****C**CGA**A****G****C****A****U****U****U****A**.**G****A****.****.**....**U****U****G****G****C****A****G****A****.**.**.****.****U****U**..**U****A****U****U**.**U****G**..**C****U****G****G****C****U****U****U****U**.**C****A****U****A**..CAACA**U****A****U****G**.**A****A****U****G****G****C****U**G**U****C****A****C****U****U****U****A**UUA..............GUUAGUUAUUAG................................G**U**-**A****A****G****U****G****G**.AGCG**C****U****A****C****A****A**GGUAC | |
|  |  | NC\_004668.1/224792-224618  | AAAAG**A****G****G****U****A****G**AGGUC**G****C****G****G****U****U**.**U****U****U**A---U**U****A****C****G****C****U**.**U****G****U**.............**G****G****A****G****U****G****.****.****.****.****A****G****A****G****G****A**.**.****C****A****C**.UGG**G****A****A**....**G****C****A****A****G**.**U****U****U****A**--GG**A****U****C**.**A****A**.**U****C****G****C**CGA**A****A****U****G****C****A****U****A**.**A****C****G****G**A...**G****C****C****U****C****U****.****.****.**U**C****U****G****U**..**U****G****U****G**.**U****G**..**U****U****G****G****G****A****C****G****U**.**A****A****G****U**..UAAGA**G****C****U****U**.**G****C****G****G****A****C****U**G**U****C****U****U****A****G****U****A**.................GUGA........................................-**U****G****C****U****A****A****G****U**.UGUG**C****U****A****U****G****U**UUCGU | |
|  |  | NC\_002951.2/1440344-1440519  | AUAUU**U****U****G****A****U****G**AGGC.**G****C****A****U****C****A**.**A****U****C**AUG**A**G**U****A****A****A****G****U**.**U****U**-.............**A****G****A****U****U****A****.****.****.****C****U****G****U****C****U****G**.**C****U****A****A**.-CA**G****C****U**....-**A****A****A****U**.**U****U****G****A****A**AGG**G****U****G**.**C****G**.**A****U****G****C**CGA**A****G****C****A****A****U****U****A**.**U****A****A**-....**U****A****G****.****C****A****G****.****.**.-**U****U****A**..**U****A****A****U**U**U****G**..**U****U****G****G****A****C****U****U****U**U**U****G****G****U**..UAAGA**G****C****U****G**.**A****G****A****G****U****U****U**G**U****C****A****U****U****A****U****U**.................UAAA........................................-**A****A****U****A****A****U****G****G**.AGUG**C****A****U****C****A****C**UUGUA | |
|  |  | NC\_002952.2/1463346-1463521  | AUAUU**U****U****G****A****U****G**AGGC.**G****C****A****U****C****A**.**A****U****C**AUG**A**G**U****A****A****A****G****U**.**U****U**-.............**A****G****A****U****U****A****.****.****.****C****U****G****U****C****U****G**.**C****U****A****A**.-CA**G****C****U**....-**A****A****A****U**.**U****U****G****A****A**AGG**G****U****G**.**C****G**.**A****U****G****C**CGA**A****G****C****G****A****U****U****A**.**U****A****A**-....**U****A****G****.****C****A****G****.****.**.-**U****U****A**..**U****A****A****U**U**U****G**..**U****U****G****G****A****C****U****U****U**U**U****G****G****U**..UAAGA**G****C****U****G**.**A****G****A****G****U****U****U**G**U****C****A****U****U****A****U****U**.................UAAA........................................-**A****A****U****A****A****U****G****G**.AGUG**C****A****U****C****A****C**UUGUA | |
|  |  | NC\_002953.3/1429786-1429961  | AUAUU**U****U****G****A****U****G**AGGC.**G****C****A****U****C****A**.**A****U****C**AUG**A**G**U****A****A****A****G****U**.**U****U**-.............**A****G****A****U****U****A****.****.****.****C****U****G****U****C****U****G**.**C****U****A****A**.-CA**G****C****U**....-**A****A****A****U**.**U****U****G****A****A**AGG**G****U****G**.**C****G**.**A****U****G****C**CGA**A****G****C****G****A****U****U****A**.**U****A****A**-....**U****A****G****.****C****A****G****.****.**.-**U****U****A**..**U****A****A****U**U**U****G**..**U****U****G****G****A****C****U****U****U**U**U****G****G****U**..UAAGA**G****C****U****G**.**A****G****A****G****U****U****U**G**U****C****A****U****U****A****U****U**.................UAAA........................................-**A****A****U****A****A****U****G****G**.AGUG**C****A****U****C****A****C**UUGUA | |
|  |  | NC\_003923.1/1401232-1401407  | AUAUU**U****U****G****A****U****G**AGGC.**G****C****A****U****C****A**.**A****U****C**AUG**A**G**U****A****A****A****G****U**.**U****U**-.............**A****G****A****U****U****A****.****.****.****C****U****G****U****C****U****G**.**C****U****A****A**.-CA**G****C****U**....-**A****A****A****U**.**U****U****G****A****A**AGG**G****U****G**.**C****G**.**A****U****G****C**CGA**A****G****C****G****A****U****U****A**.**U****A****A**-....**U****A****G****.****C****A****G****.****.**.-**U****U****A**..**U****A****A****U**U**U****G**..**U****U****G****G****A****C****U****U****U**U**U****G****G****U**..UAAGA**G****C****U****G**.**A****G****A****G****U****U****U**G**U****C****A****U****U****A****U****U**.................UAAA........................................-**A****A****U****A****A****U****G****G**.AGUG**C****A****U****C****A****C**UUGUA | |
|  |  | NC\_002745.2/1399486-1399661  | AUAUU**U****U****G****A****U****G**AGGC.**G****C****A****U****C****A**.**A****U****C**AUG**A**G**U****A****A****A****G****U**.**U****U**-.............**A****G****A****U****U****A****.****.****.****C****U****G****U****C****U****G**.**C****U****A****A**.-CA**G****C****U**....-**G****A****A****U**.**U****U****G****A****A**AGG**G****U****G**.**C****G**.**A****U****G****C**CGA**A****G****C****G****A****U****U****A**.**U****A****A**-....**U****A****G****.****C****A****G****.****.**.-**U****U****A**..**U****A****A****U**U**U****G**..**U****U****G****G****A****C****U****U****U**U**U****G****G****U**..UAAGA**G****C****U****G**.**A****G****A****G****U****U****U**G**U****C****A****U****U****A****U****U**.................UAAA........................................-**A****A****U****A****A****U****G****G**.AGUG**C****A****U****C****A****C**UUGUA | |
|  |  | NC\_002758.2/1475815-1475990  | AUAUU**U****U****G****A****U****G**AGGC.**G****C****A****U****C****A**.**A****U****C**AUG**A**G**U****A****A****A****G****U**.**U****U**-.............**A****G****A****U****U****A****.****.****.****C****U****G****U****C****U****G**.**C****U****A****A**.-CA**G****C****U**....-**G****A****A****U**.**U****U****G****A****A**AGG**G****U****G**.**C****G**.**A****U****G****C**CGA**A****G****C****G****A****U****U****A**.**U****A****A**-....**U****A****G****.****C****A****G****.****.**.-**U****U****A**..**U****A****A****U**U**U****G**..**U****U****G****G****A****C****U****U****U**U**U****G****G****U**..UAAGA**G****C****U****G**.**A****G****A****G****U****U****U**G**U****C****A****U****U****A****U****U**.................UAAA........................................-**A****A****U****A****A****U****G****G**.AGUG**C****A****U****C****A****C**UUGUA | |
|  |  | NC\_002973.5/828183-827986  | UGGUG**A****G****G****U****A****G**AGGUU**G****C****G****A****G****A**.**U****G****C**ACU**A**G**U****A****A****U****U****U**.**U****U****U**.............**C****G****A****G****G****C****G****A****A****A****C****A****A****A****G****A**.**C****G****C****C**.GAC**G****A****C**....**A****A****A****G****A**.**A****U****G****A****A**CAG**G****U****U**.**G****A**.**U****C****G****C**CGA**A****G****U****G****A****C****U****A**.**U****U****U****U**C...**U****C****U****U****U****G****U****U****U**A**G****A****A****A**..**U****A****G****U**.**U****G**..**U****U****G****G****G****A****C****A****G**.**U****U****U****C**.CUAA.A**G****G****G****G**.**C****U****G****G****A****C****U**G**C****U****A****U****A****A****G****A**A................UUUGUCGAAAU.................................U**U****C****U****U****A****U****A****G**GUGUG**C****U****A****U****C****U**GACAA | |
|  |  | NC\_003210.1/826683-826486  | UGGUG**A****G****G****U****A****G**AGGUU**G****C****G****A****G****A**.**U****G****C**ACU**A**G**U****A****A****U****U****U**.**U****U****U**.............**C****G****A****G****G****C****G****A****A****A****C****A****A****A****G****A**.**C****G****C****C**.GAC**G****A****C**....**A****A****A****G****A**.**A****U****G****A****A**CAG**G****U****U**.**G****A**.**U****C****G****C**CGA**A****G****U****G****A****C****U****A**.**U****U****U****U**C...**U****C****U****U****U****G****U****U****U**A**G****A****A****A**..**U****A****G****U**.**U****G**..**U****U****G****G****G****A****C****A****G**.**U****U****U****C**.CUAA.A**G****G****G****G**.**C****U****G****G****A****C****U**G**C****U****A****U****A****A****G****A**A................UUUGUCGAAAU.................................U**U****C****U****U****A****U****A****G**GUGUG**C****U****A****U****C****U**GACAA | |
|  |  | NZ\_AADR01000092.1/1734-1537  | UGGUG**A****G****G****U****A****G**AGGUU**G****C****G****A****G****A**.**U****G****C**ACU**A**G**U****A****A****U****U****U**.**U****U****U**.............**C****G****A****G****G****C****G****A****A****A****C****A****A****A****G****A**.**C****G****C****C**.GAC**G****A****C**....**A****A****A****G****A**.**A****U****G****A****A**CAG**G****U****U**.**G****A**.**U****C****G****C**CGA**A****G****U****G****A****C****U****A**.**U****U****U****U**C...**U****C****U****U****U****G****U****U****U**A**G****A****A****A**..**U****A****G****U**.**U****G**..**U****U****G****G****G****A****C****A****G**.**U****U****U****C**.CUAA.A**G****G****G****G**.**C****U****G****G****A****C****U**G**C****U****A****U****A****A****G****A**A................UUUGUCGAAAU.................................U**U****C****U****U****A****U****A****G**GUGUG**C****U****A****U****C****U**GACAA | |
|  |  | NZ\_AABF02000218.1/184-5  | AUAAA**A****A****A****U****A****G**AGGU.**G****C****A****U****A****U**.**A****U****G**-UA**G**G**U****A****G****U****G****U**.**G****A****A**AAUGU........**U****A****A****G****G****U****.****.****.****.****A****U****A****A****G****U**.**C****A****C****C**.AAU**G**--...U**U****U****C****A****C**.**A****C****A****A****A**GGG**C****A****U**.**A****G**.**U****U****G****C**CGA**A****A****G****A****G****A****A****U**.**U****A****A****U**A...**G****C****U****U****A****U****.****.****.**G**A****U****U****A**..**A****U****U****U**.**U****C**..**U****U****G****G****U**-**C****A****A**.**U****G****U**-..CAACA**A****G****C****A**.**U****U****G**-**A****C****U**G**U****C****A****U****A****U****U****C**.................UCUU........................................-**G****A****A****U****A****U****G****G**.AGAG**C****U****A****U****U****A**UCUCG | |
|  |  | NZ\_AADW02000004.1/181244-181066  | CCGAC**A****G****A****U****A****G**AGGC.**G****C****G****G****A****U**.**G****A****C**AUC**A**G**U****A****G****C****A****A**.**C****A****C**UUAU.........**U****C****A****A****G****G****C****.****.****.****A****A****G****C****A****U**.**G****C****C****A**.------...A**G****U****G****A****U**.**G****U****G****A****A**AGG**G****G****A**A**A****U**.**C****C****G****C**CGA**A****G****U****G****A****A****C****A**.**A****G****A****A**A...**A****U****G****C****U****U****.****.****.**U**U****U****C****U**..**U****G****A****U**.**U****G**..**C****U****G****G****U****A****C****A****A**.**C****G****G****U**..UAAGA**U****C****C****G**.**U****U****G****U****A****C****U**G**C****C****G****A****A****A****C**-.................CCAC........................................--**G****U****U****U****C****G****G**.AGCG**C****U****A****U****C****U**UACGU | |
|  |  | NC\_002505.1/414433-414256  | UCUAG**C****A****G****A****A****G**AGGA.**G****C****A****C****U****G**.**C****C****C**A--**G**G**C****A****G****A****U****G**.**U****U****U**.............**U****G****U****G****G****A****G****.****.****.****C****C****U****C****A****A**.**C****U****C****C**.AAU**A****C****A**....**G****A****A****C****A**.**U****U****C****A****G**GGG**G****A****G**.**U****A**.**G****U****G****C**CGA**G****G****U****G****A****A****U****C**.**A****A****A****G**....**U****U****G****U****G****G**-**.****.**.**C****U****U****U**..**G****G****U****U**.**U****A**..**U****C****G****G****U****U****G****A****A**.**C****G****G****G**CUGAA.U**C****C****C**-.**U****U****C****A****A****C****U**G**U****C****A****U****C****A****G**-CUC..............GAAU........................................--**C****U****G****A****U****G****A**.AGAG**C****U****U****C****U****G**AGGGA | |
|  |  | NC\_004347.1/3280656-3280460  | CUUAG**A****A****G****U****A****G**AGAC.**G****C****A****C****C****A**.**A****A****U**AUC**A**G**U****A****G****U****G****A**.**U****C****A**.............**G****U****A****G****G**-**.****.****.****.****G****U****G****A****U****U**.**.**-**C****C**.AUU**G****A****U**....**C****G****A****U****C**.**A****C****G****A****A**AGG**A****U****U**.**U****G**.**G****U****A****C**CGA**A****G****U****G****G****U****U****U**.**G****A****U****U**....**U****A****U****C****A****C**-**.****.**.**G****A****U****C**..-**A****A****C**.**U****G**..**C****U****G****G****G****G****U****U****G**.**U****G****C****U**..GAAUA**G****G****U****A**.**C****A****A****U****A****C****U**G**C****C****G****U****A****G****U****A**UUUCCUGAUAUGGA...UAUUCGGAAUCG................................U**U****A****C****U****A****U****G****G**.AGUG**C****U****A****C****U****G**AUAGG | |
|  |  | NC\_006582.1/2146030-2146207  | UGUGA**G****A****G****U****A****G**AGGC.**G****C****G****U****A****A**.**A****C****G**AUC**A**G**U****A****C****G****G****C**.**U****G****C**.............**U****G****A****G****G****A****.****.****.****A****U****U****G****G****G****A**.**.****U****C****U**GUUU**G****A****A**....**C****C****A****G****C**.**C****G****G****A****A**AGG**G****G****U**.**U****U**.**A****U****G****C**CGA**A****A****U**-**G****G****G****C**.**G****G****U****U**U..U**U****C****C****C****A****A****.****.****.**A**G****G****C****C**..**G****U****A****C**.**C****A**..**U****U****G****G****G****G****C****U****G**.**U****U****U****C**.UGAAUA**A****G****G****G**.**C****A****G****C****A****C****U**G**U****C****A****U****A****C****G**-.................AA--........................................--**C****G****U****A****U****G****G**.GGGG**C****U****A****U****C****U**CAAAC | |
|  |  | NZ\_AAIO01000064.1/6936-6762  | AGGAA**C****A****G****A****A****G**AGGA.**G****C****G****U****U****A**.**A****C****U**A--**G**G**U****A****G****U****C****G**.**G****U****C**.............**A****G****A****G****G****A****G****.****.****C****A****.****C****A****A****A**.**C****U****C****C**.AGC**G****A****U**....**G****A****C****U****G**.**A****U****G****A****G**GGA**G****A****U**.**U****A**.**A****C****G****C**CGA**G****G****C****A****U****A****G****A**.**U****A****U****G**....**G****U****U****G****C****U****G****.****.**.**C****A****U****G**..**U****U****U****A**.**U****G**..**U****C****G****G****U****C****G****C****U**.**C****A****G****G**CUGAA.U**C****C****U**-.**G****A****C****G****A****U****U**G**U****C****A****C****C****U****G**-.................UAA-........................................--**U****U****G****G****U****G****G**.AGAG**C****U****U****C****U****G**GUGAC | |
|  |  | NC\_004459.1/1404838-1404660  | UUUUG**C****A****G****A****A****G**AGGA.**G****C****A****C****U****G**.**C****C****C**A--**G**G**C****A****G****A****U****G**.**U****U****U**.............**U****G****U****G****G****A****G****.****.****C****C****.****G****C****A****A**.**C****U****C****C**.AAC**A****C****A**....**G****A****A****C****A**.**U****U****C****A****G**GGG**G****A****G**.**U****A**.**G****U****G****C**CGA**G****G****U****A****G****A****U****C**.**A****A****A****A**....**U****U****G****C****A****G****G****.****.**.**A****U****U****U**..**G****A****U****C**.**U****G**..**U****C****G****G****U****U****G****A****C**U**U****G****G****G**UUGAG.U**C****C****C****A**.-**U****C****A****A****C****U**G**U****C****A****U****C****A****G**-.................CUCAG.......................................C-**C****U****G****A****U****G****A**.AGAG**C****U****U****C****U****G**AGAUG | |
|  |  | NC\_005139.1/3027782-3027960  | UUUUG**C****A****G****A****A****G**AGGA.**G****C****A****C****U****G**.**C****C****C**A--**G**G**C****A****G****A****U****G**.**U****U****U**.............**U****G****U****G****G****A****G****.****.****C****C****.****G****C****A****A**.**C****U****C****C**.AAC**A****C****A**....**G****A****A****C****A**.**U****U****C****A****G**GGG**G****A****G**.**U****A**.**G****U****G****C**CGA**G****G****U****A****G****A****U****C**.**A****A****A****A**....**U****U****G****C****A****G****G****.****.**.**A****U****U****U**..**G****A****U****C**.**U****G**..**U****C****G****G****U****U****G****A****C**U**U****G****G****G**UUGAG.U**C****C****C****A**.-**U****C****A****A****C****U**G**U****C****A****U****C****A****G**-.................CUCAG.......................................C-**C****U****G****A****U****G****A**.AGAG**C****U****U****C****U****G**AGAUG | |
|  |  | NC\_004347.1/4124733-4124559  | AGGAA**C****A****G****A****A****G**AGGA.**G****C****G****U****U****A**.**A****C****U**A--**G**G**U****A****G****U****C****A**.**A****U****C**.............**A****G****A****G****G****A****G****.****.****C****A****.****C****A****A****A**.**C****U****C****C**.AGC**G****A****U**....**G****A****U****U****G**.**A****U****G****A****G**GGA**G****A****U**.**U****A**.**G****C****G****C**CGA**G****G****C****A****U****A****G****A**.**U****G****U****G**....**G****U****U****G****C****U****G****.****.**.**C****A****U****G**..**U****U****U****A**.**U****G**..**U****C****G****G****U****C****G****C****U**.**U****A****G****G**CUGAA.U**C****C****U**-.**A****A****C****G****A****U****U**G**U****C****A****C****C****U****G**-.................UAA-........................................--**U****U****G****G****U****G****G**.AGAG**C****U****U****C****U****G**GUGAC | |
|  |  | NZ\_AAGR01000017.1/26461-26283  | CAAAC**A****G****A****U****A****G**AGGUU**G****C****A****A****C****C**.**A****A****C**AUG**A**G**U****A****G****C****U****U**.**G****A****U**.............**C****G****A****G****C****G****G****.****.****.****G****A****U****C****C****U**.**U****C****G****U**.GAU**G****A****C**....**A****U****C****A****A**.**G****C****C****A****A**AGG**G****G****C**.**G****G**.-**U****G****C**CGA**A****A****U****G****G****G****G****G**.**C****G****C****C**....**G****G****G****A****U****C**-**.****.**.**G****G****C****A**..**A****A****C****C**.**U****G**..**U****U****G****G****G****C****C****A****G**.**G****A****U****U**..GAAUA**A****G****U****C**.**C****U****G****U****A****C****U**G**U****C****G****C****U****U****A****G**.................AAAC........................................-**C****U****A****A****G****C****G****G**.GGCG**C****U****A****U****C****A**UUCAA | |
|  |  | NC\_006840.1/365527-365353  | AUAUA**A****A****G****A****A****G**AGGA.**G****C****A****U****U****A**.**C****C****U**A--**G**G**U****A****G****C****U****U**A**A****U****U**.............**U****G****U****G****G****A****G****.****.****.****.****C****C****C****A****A**A**C****U****C****C**.UAC**A****C****A**....**A****A****U****A****A**.**G****U****G****A****G**GGA**G****A****G**.**U****A**.**A****U****G****C**CGA**G****A****U****G****A****U****C****C**.**U****A**--....**U****U****G****G****G****A****G****.****.**.--**U****A**..**G****G****A****U**.**U****G**..**U****C****G****G****U****C****G****U****U**.**U****A****G****G**CUGAA.U**C****C****U**-.**A****A****C****G****A****U****U**G**U****C****A****C****U****U****A****A**.................UUUA........................................-**U****U****A****A****G****U****G****A**.AGAG**C****U****U****C****U****A**GCAAA | |
|  |  | NC\_004603.1/2873784-2873962  | UUAUG**U****A****G****A****A****G**AGGA.**G****C****A****C****U****G**.**C****C****C**A--**G**G**C****A****G****A****U****G**.**A****U****U**.............**U****G****U****G****G****A****A****.****.****C****C****.****G****C****A****A**.**U****U****C****C**.AAU**A****C****C**....**A****A****U****C****A**.**U****U****C****A****G**GGG**G****A****G**.**U****A**.**G****U****G****C**CGA**G****G****U****A****A****G****U****C**.**A****A****A****A**....**U****U****G****C****A****G****G****.****.**.**G****U****U****U**..**G****G****C****U**.**U****G**..**U****C****G****G****U****U****G****A****C**U**U****G****G****G**UUGAG.U**C****C****C****A**.-**U****C****A****A****C****U**G**U****C****A****U****C****A****G**-.................CACCG.......................................U-**C****U****G****A****U****G****A**.AGAG**C****U****U****C****U****G**AGGGU | |
|  |  | NC\_003869.1/719320-719498  | CGCAU**A****A****A****U****A****G**AGGA.**G****C****U****G****C****C**.**A****A****G**CAU-G**U****A****U****U****U****G**.**G****C****G**AGGUGU.......**U****A****A****G****G****A****G****.****.****.****A****A****G****A****A****C**.**C****U****C****C**.AAU**A****C**-...U**C****G****C****U****G**.**A****A****G****A****A**-GG**U****U****U**.**G****G**.**C****U****G****C**CGA**A****A****G****G****G****U****G****A**.**G****C****U****U**....**G****U****U****C****U****U**-**.****.**.**G****A****G****C**..**U****C****A****U**.**C****C**..**U****U****G****G****U****G****G****U****A**.**A****A****C**-.ACAA.A-**G****U****U**.**U****A****C****C****A****C****U**G**U****C****A****U****G****G****G**-.................ACCU........................................--**C****C****C****A****U****G****A**.AGCG**C****U****A****U****U****U**AUGCA | |
|  |  | NZ\_AAIU01000063.1/7664-7467  | CUUUA**U****G****G****U****A****G**AGGU.**G****C****G****C****U****A**.**A****U****U**AUA**A**G**U****A****G****U****G****A**.**U****C****A**.............**G****U****A****G****G**-**.****.****.****.****G****U****G****A****U****G**.**.**-**C****C**.UAA**G****A****U**....**C****G****U****U****C**.**A****C****G****A****A**AGG**A****G****U**.**U****A**.**G****U****G****C**CGA**A****G****U****U****G****A****G****C**.**U****A****U****C**....**C****A****U****C****A****U**-**.****.**.**G****A****U**-..-**C****U****A**C**U****G**..**C****U****G****G****U****G****U****U****G**C**U****G****U****U**..GAAAA**A****G****C****G**.**C****A****A****C****A****C****U**G**C****C****A****U****G****G****U**-GUUU.............UUUAUCGGGAAGAUAGAAGAUUUU....................A-**A****C****C****G****U****G****G**.AGCG**C****U****A****C****U****G**AUAGG | |
|  |  | NZ\_AAIV01000008.1/39504-39288  | AACAA**A****A****G****U****A****G**AGGU.**G****C****G****U****U****G**.**C****A****U**AAA**A**G**U****C****A****U****G****U**.**U****G****A**.............**A****U****U****G****G**-**.****.****.****.****G****U****G****A****U****G**.**.**-**C****C**.AAU**A****A****U**....**U****C****G****A****C**.**A****U****A****A****A**AGG**A****U****G**.**C****A**.**A****U****G****C**CGA**A****A****U****A****A****A****U****G**.**G****G****G****U**....**U****A****U****C****A****.****.****.****.**.**G****C****U****U**..**C****A****U****U**.**U****G**..**U****U****G****G****G****G****C****A****G**.**U****U****C****U**.CGAA.A**G****G****G****A**.**C****U****G****C****A****C****U**G**U****C****A****U****A****G****U****G**UU...............UUUAUCAACUUGAUAAAAAGAAUGAUAUUCAUAUUAAAUGGAUAU**U****G****C****U****G****U****G****G**.AGUG**C****U****A****C****U****A**GUUAA | |
|  |  | NZ\_AAIN01000006.1/15952-16127  | AGGCA**C****A****G****A****A****G**AGGA.**G****C****G****C****C****A**.**A****C****U**A--**G**G**U****A****G****C****A****G**.**G****C****U**.............**U****G****A****G****G****A****G****.****.****.****C****A****A****U****C****A**.**C****U****C****C**.AAA**G****A****C**....**G****A****C****C****U**.**G****U****G****A****G**GGA**G****A****U**.**U****G**.**G****C****G****C**CGA**G****G****C****A****A****A****A****C**.-**C****A****C**G...**U****G****C****U****U****G****.****.****.**A**G****U****G****C**..**G****U****U****U**.**U****G**..**U****C****G****G****U****C****G****U****U**.**C****A****G****G**CUGAA.U**C****C****U**-.**G****A****C****G****A****U****U**G**U****C****A****C****C****U****G**-.................UUAU........................................--**U****U****G****G****U****G****G**.AGAG**C****U****U****C****U****G**GUGAC | |
|  |  | NZ\_AAIN01000011.1/77337-77138  | GUCCU**G****A****G****U****A****G**AGGU.**G****C****G****C****C****A**.**A****G****C**AUC**A**G**U****A****A****C****A****G**.**U****C****A**.............**A****U****A****G****G**-**.****.****.****.****G****U****G****A****U****G**.**.**-**C****C**.GUU**G****A****C**....**G****G****G****C****U**.**G****U****G****A****A**AGG**G****U****A**U**U****G**.**G****C****G****C**CGA**A****G****U****G****G****A****U****G**.**U****G****U****G**....**U****A****U****C****A****C**-**.****.**.**C****G****C**-..**C****U****U****C**.**C****G**..**C****U****G****G****G****G****U****C****G**.**U****U****U****U**.CGAA.A**G****A****A****A**.**C****G****G****C****A****C****U**G**C****C****A****U****A****G****U****A**.................UUUUCUGUCGGGAUGACAGAUGUUGUAU................U**U****A****C****U****A****U****G****G**.AGCG**C****U****A****C****U****G**AUAGG | |
|  |  | NC\_002662.1/2276232-2276414  | CACAU**C****G****A****U****A****G**AGGUC**G****C****A****A****C****U**.**G****A****U**AU--G**A****A****U****C****U****A**.---CGC..........**C****G****A****G****U****U****G****.****.****.****.****G****A****G****C****A**.**C****A****A****C**.AAA**G****A****C**GCGU**A****U****U****U****A**.**G****A****G**-**G**AGG**A****G****A**.**G****G**.**U****U****G****C**CGA**A****A****G****A****A****U****U****U**.**U****G****U**-....**U****G****C****U****C****A**-**.****.**.-**G****C****A**..**A****G****G****U**.**U****C**..**U****U****G****G****G****C****U****A****G**.**U****G****A****G**..AAAAA**C****U****C****A**.**C****U****A****G****A****C****U**G**U****C****G****C****A****A****A****U**GG...............UUAAUAAC....................................C**A**--**U****G****C****G****G**.AGGG**C****U****A****U****U****C**GUUCA | |
|  |  | NZ\_AABH02000020.1/12426-12593  | UAUUG**U****C****A****A****A****G**AGGUU**G****C****A****G****C****C**.**A****A****C**AUG**A**G**U****C****A****C****A****U**.**G****A****A**.............**U****G****A****G****U****C****C****.****.****.****.****.****G****C****C****A**.**G****G****A****U**.GUU**G****A****A**....**A****U****C****A****U**.**G****U****A****A****A**AGG**G****A****A**.**G****G**.**U****U****G****C**CGA**A****A****G****G****G****U****U****A**.**A****C**--....**A****G****G****C****A****A**-**.****.**.--**G****A**..**U****A****A****C**.**C****U**..**U****U****G****G****G****C****U****A****G**.**U****G****A****U**..UAAGA**G****U****U****G**.**C****U****A****G****A****C****U**G**U****C****G****C****A**---.................GAAA........................................----**U****G****C****G****G**.UGCG**C****U****U****U****G****A**AAAGU | |
|  |  | NZ\_AABJ03000007.1/66428-66257  | AUCCG**U****A****A****U****A****G**AGGU.**G****C****A****A****A****U**.**G****A****C**AUC**A**U**U****A****G****U****U****U**.**A****U****U**.............**A****G****A****U****A****G****.****.****.****.****U****C****C****C****U****G**.**.****C****U****A**.GGA**U****C****U**....**A****A****U****A****A**.**A****U****G****A****A**AGG**G****G****A**.**U****U**.**U****U****G****C**CGA**A****A****U****C****G****A****U****U**.**G****U****A****U**....**G****A****G****C****A****G**-**.****.**.**A****U****A****C**UU-**A****U****C**.**G****A**..**U****U****G****A****G****U****C****G****U**.**G****G****U****U**..UAAUA**A****A****U****C**.**A****C****G****G****A****U****U**G**U****C****A****C****A**---.................GAGA........................................----**U****G****U****G****G**.AGUG**C****U****A****G****G****A**CGGAC | |
|  |  | NZ\_AABH02000013.1/9988-10156  | AUUUC**A****C****A****U****A****G**AGGUC**G****C****G****A****U****U**.**G****A****C**A---A**U****A****C****A****C****U**.**U****A****C**C............**G****G****A****G****U****U****G****.****.****.****C****U****G****U****A****G**.**C****A****A****U**.--U**G****A****U**...A**G****U****A****A****G**.**C****U****U****A**--GG**G****G****A**.**U****A**.**U****C****G****C**CGA**A****A****U****C****A****A****G****G**.**G****U****G****U**....**C****U****A****C****A****A****.****.****.**.**A****U****G****A**..**C****C****U****U**.**G****A**..**U****U****G****G****G****C****U****A****A**.**U****A****G****U**..UAAGA**G****C****U****A**.**U****U****A****G****A****C****U**G**U****C****G****C****A**---.................GAGA........................................----**U****G****C****G****G**.UGAG**C****U****A****U****G****G**AAACU | |
|  |  | NZ\_AABH02000106.1/368-200  | AUUUC**A****C****A****U****A****G**AGGUC**G****C****G****A****U****U**.**G****A****C**A---A**U****A****C****A****C****U**.**U****A****C**C............**G****G****A****G****U****U****G****.****.****.****C****U****G****U****A****G**.**C****A****A****U**.--U**G****A****U**...A**G****U****A****A****G**.**C****U****U****A**--GG**G****G****A**.**U****A**.**U****C****G****C**CGA**A****A****U****C****A****A****G****G**.**G****U****G****U**....**C****U****A****C****A****A****.****.****.**.**A****U****G****A**..**C****C****U****U**.**G****A**..**U****U****G****G****G****C****U****A****A**.**U****A****G****U**..UAAGA**G****C****U****A**.**U****U****A****G****A****C****U**G**U****C****G****C****A**---.................GAGA........................................----**U****G****C****G****G**.UGAG**C****U****A****U****G****G**AAACU | |
|  |  | NZ\_AABJ03000002.1/230549-230380  | CAAUA**A****A****A****U****A****G**AGGC.**G****C****G****A****U****C**.**A****U****C**A---C**A****A****A****A****A****C**.**U****G****C**.............**U****G****A****G****U****G****.****.****.****.****U****G****U****A****U****U**.**.****C****A****C**.GAU**G****A****A**....**G****C****A****G****U**.**C****C****U****A****A**-GG**G****U****U**.-**A**.**U****C****G****C**CGA**A****A****C****G****A****G****A****U**.**U****A****U****A**....**A****A****U****G****C****A**-**.****.**.**U****A****U****A**..**A****U****C****U**U**U****G**..**U****U****G****G****G****A****C****A****U**.**G****U****G****G**..AAAAA**C****C****G****C**.**A****U****G****G****A****C****U**G**U****C****U****C****G****G**--U................CAAU........................................---**C****C****G****G****G****G**.AGUG**C****U****A****U****U****A**AUUGG | |
|  |  | NC\_005362.1/111419-111246  | CAAUG**C****A****A****U****A****G**AGGUU**G****C****G****A****U****A**.**A****U****C**ACA----**G****A****A****U**.**C****U****A**CU...........**U****G****A****G****U****A****.****.****.****.****C****G****C****G****A****A**.**.****U****A****C**.AGU**G****A****G**..AG**U****A****U****A****U**.**U****U****U****A****G**---**G****A****U**.**U****A**.**C****C****G****C**CGA**A****A****U****A****A****A****U****U**.**U****U****C****U**....**U****U****C****G****U****G****A****.****.**.**A****G****U****U**..**A****A****U****U**.**U****G**..**U****U****G****G****U****A****U****G****U**.**A****A****G****A**..GAAUA**U****C****U****U**.**A****C****A****U****A****C****U**G**U****C****C****U****G****G**--UC...............GUCC........................................---**C****C****A****G****G****G**.AGCG**C****U****A****U****A****U**GAUUU | |
|  |  | NZ\_AAIU01000001.1/51300-51131  | AGGCA**C****A****G****A****A****G**AGGC.**G****C****G****U****U****A**.**A****C****U**A--**G**G**U****A****A****U****A****A**.**G****U****C**.............**G****G****A****G****A****A****.****.****.****.****U****A****C****C****C****C**A**.****U****U****C**.UUU**G****A****U**....**G****G****C****U****U**.**A****U****G****A****G**-GG**A****G****U**U**U****A**.**A****C****G****C**CGA**G****G****C****A****A****C**-**A**.**U****A****C**-....**G****U****G****G****U****G**-**.****.**.-**G****U****A**..**U****U****G****U**.**U****G**..**U****C****G****G****U****C****G****G****U**.**U****A****G****G**CUGAA.U**C****C****U****A**.**A****C**-**G****A****U****U**G**U****C****A****C****C****U****U**-.................UUU-........................................--**A****U****G****G****U****G****G**.AGAG**C****U****U****C****U****G**GUGAC | |
|  |  | NZ\_AAIV01000003.1/110542-110368  | AGGAA**C****A****G****A****A****G**AGGA.**G****C****G****U****U****A**.**A****C****U**A--**G**G**U****A****G****U****A****A**.**G****U****G**.............**A****G****A****G****G****A****.****.****.****.****U****G****C****C****A****A**.**.****U****C****C**.GAU**G****A****C**....**C****A****C****U****U**.**A****U****G****A****U**GGA**G****A****U**.**U****A**.**G****C****G****C**CGA**G****G****U****A****U****C****A****G**U**U****U****G****U**....**U****U****G****G****C****A****.****.****.**.**A****A****C****A**..**U****U****G****A**.**U****A**..**U****C****G****G****U****C****G****A****U**.**U****A****G****G**CUGAA.U**C****C****U****A**.**A****C**-**G****A****U****U**G**U****C****A****C****C****U****A**-U................UUUU........................................--**U****U****G****G****U****G****G**.AGAG**C****U****U****C****U****G**GUGAU | |
|  |  | NZ\_AABH02000010.1/23306-23474  | AAGAA**U****C****A****U****A****G**AGGUU**G****C****U****G****C****U**.**G****A****C**AUU**A**G**U****C****G****C****U****U**.**G****C**-.............**G****G****A****G****U****G****C****.****.****.****U****U****A****C****A****G**.**G****C****A****C**.AAU**G****A****A**....-**A****C****A****G**.**G****U****U****A****A**AGG**G****G****A**.**U****G**.**U****A****G****C**CGA**A****G****C**-**A**-**U****U**.**G****G****U****G**....**C****U****G****U****G****A**-**.****.**.**C****A****C****C**..**A****A****G****U**.**A****G**..**C****U****G****G****G****C****C****A****A**U**C****G****G****U**..GAAAA**A****U****C****G**.**U****U****G****G****A****C****U**G**U****C****G****C**----.................GUAA........................................-----**G****C****G****G**.GGUG**C****U****A****U****G****U**UAUAG | |
|  |  | NZ\_AAGR01000056.1/9188-9363  | AUAAC**G****A****U****U****A****G**AGGUU**G****C****A****U****C****U**.**G****U****C**AUG**A**A**U****C****C****U****A****A**.**C****C****G**.............**C****A****A****U****G****G****C****.****.****.****C****U****G****A****A****C**.**G****C****C****A**.GAU**G****C****G**....**C****G****G****U****U**.**G****C****A****A****A**AGG**G****G****U**.**G****G**.**A****U****G****C**CGA**A****A****U****G****C****C****G****G**.**A****U****U****U**....**G****U****U****C****A****G**-**.****.**.**A****A****A****U**..**C****C****G****A**.**C****A**..**U****U****G****G****G****C****C****A****G**U**U****G****G****A**..CAACA**U****C****C****G**.**C****U****G****G****A****C****U**G**U****C****G****C****G****A**--.................ACA-........................................---**U****C****G****C****G****G**.GGCG**C****U****A****A****U****G**AAUCG | |
|  |  | NC\_005303.1/669191-669021  | AAAUC**A****A****A****A****A****G**AGGUA**G****C****G****G****U****G**.**U****A****A**AAG**A**G**U****A****U****U****A****U**.**G****U**-.............**G****A****A****G****A****A****.****.****.****.**--**G****G****C****A**.**.****U****U****C**.UGU**G****A****A**....-**G****C****A****U**.**A****A****G****A****A**AGG**U****A****A**C**C****A**.**U****C****G****C**CGA**A****U****A****A****U****A****A****U**.**U****A****U**-....**G****G****C****A****A****U****A****.****.**.-**A****U**-..-**U****U****A**.**U****U**..**A****U****G****A****G****A****U****U****A**.**U****A****G****A**..AAAUA**U****C****U****A**.**U****A****A****U****A****U****U**C**U****U****G****C****U****G****U**-.................UUUA........................................--**A****C****A****G****U****A****A**AGGCG**C****U****U****U****A****A**UCAAG | |
|  |  | NZ\_AAAO02000018.1/33549-33722  | CAAUG**C****A****A****U****A****G**AGGUU**G****C****G****A****U****A**.**A****C****C**A---C**A****A****A****A****U****C**.**U****A****C**U............**U****G****A****G****U****A****.****.****.****.****C****G****C****G****A****A**.**.****U****A****C**.AUU**G****A****C**...A**G****U****A****G****U**.**U****U****U****U**--AG**G****A****U**.**U****A**.**U****C****G****C**CGA**A****A****C****A****A****G****C****U**.**U****A****C****U**....**U****U****C****G****C****G****A****.****.**.**A****U****U****A**..**A****G****C****U**.**U****G**..**U****U****G****G****G****A****U****G****U**.**G****A****G****A**..GCAUA**U****C****U****U**.**A****C****A****U****A****C****U**G**U****C****C****U****G****G**--.................UAGUC.......................................C--**C****C****A****G****G****G**.AGCG**C****U****A****U****A****U**GAUUU | |
|  |  | NC\_004431.1/4769195-4768999  | CAGGC**C****A****G****A****A****G**AGGC.**G****C****G****U****U****G**.**C****C****C**A--**A**G**U****A****A****C****G****G**.**U****G****U**U............**G****G****A****G****G****A****.****.****.****.**-**G****C****C****A****G**.**.****U****C****C**.UGU**G****A****U**...A**A****C****A****C****C**.-**U****G****A****G**GGG**G****U****G**.**C****A**.**U****C****G****C**CGA**G****G****U****G****A****U****U****G**.**A****A****C****G**G...**C****U****G****G****C****C****.****.****.**A**C****G****U****U**..**C****A**-**U**.**C****A**..**U****C****G****G****C****U****A****C****A**.**G****G****G****G**.CUAAAU**C****C****C****C**.**U****G**-**G****G****U****U**G**U****C****A****C****C****A****G****A**AGCGUUCGCAGUCGGGCGUUUCGC.....................................A**A****G****U****G****G****U****G****G**.AGCA**C****U****U****C****U****G**GGUGA | |
|  |  | NC\_006370.1/3733830-3734021  | UAAAA**C****G****G****A****A****G**AGGA.**G****C****G****U****U****A**.**C****C****C**A--**G**G**U****A****U****U****U****A**.**G****A****U**.............**U****G****C****A****G****G****.****.****.****G****C****C****C****C****A****C**.**.****C****C****U**.AAA**G****C****A**....**A****C****C****U****A**.**A****A****G****A****G**GGG**G****A****G**.**U****A**.**A****C****G****C**CGA**G****A****U****A****U****A****A****C**.**U****G****A**-....**U****U****G****G****G****G****C****.****.**.-**U****C****A**..**G****C****G****A**.**U****A**..**U****C****G****G****C****U****A****U****G**.**A****A****G****G**UUGAA.U**C****C****U****U**.**C****U**-**G****G****C****U**G**U****C****A****C****C****U****U****U**.................UUAUGCAUAAGUACCAUAUA........................A**A****A****A****G****G****U****G****G**.GGAG**C****U****U****C****U****G**GUUGU | |
|  |  | NC\_002662.1/1665920-1665745  | AACAA**A****A****U****U****A****G**AGGUU**G****C****G****A****A****G**.**A****C****G**A--**A**G**A****A****G****U****U****A**.**G****U**-.............**G****G****A****G****C****G****.****.****.****.**-**G****U****G****A****A**.**.****C****G****C**.ACU**G****A****A**....-**G****C****U****G**.**A****U****G**-**G**CGG**C****G****U**U**C****U**.**U****U****G****C**CGA**A****A****G****A****A****A****A****A**.**U****U**--....**G****U****C****A****C****A****A****.****.**.--**A****A**..**U****U****U****U**.**U****C**..**U****U****G****G****G****C****U****G****U**G**U****G****G****U**..UAAAU**C****C****U****A**.-**C****A****G****A****C****U**G**U****C****U****U****U****C****U****G**UCAG.............UACUG.......................................A**C****A****G****A****A****A****G****G**.GGAG**C****U****A****A****U****C**AAAAU | |
|  |  | NC\_006814.1/110177-110004  | CAAUG**C****A****A****U****A****G**AGGUU**G****C****G****A****C****A**.**A****U****C**ACA----**A****G****G****U**.**A****U****U**UU...........**G****G****A****G****U****C****.****.****.****.****C****G****C****G****A****A**.**.****G****A****U**.GAA**G****A****A**...G**A****A****U****A****U**.**C****U****C****A****G**---**G****G****U**.**U****G**.**C****C****G****C**CGA**A****A****U****G****G****A****U****U**C**A****A****U****A**....**U****U****C****G****C****G**-**.****.**.**U****A****U****U**..**A****A****U****C**.**U****G**..**U****U****G****G****G****C****U****A****C**.**A****G****G****A**..GCAUA**U****C****C****U**.**G****C****A****G****A****C****U**G**U****C****U****C****G****G**--.................UUAGUC......................................C--**C****C****G****A****G****G**.AGCG**C****U****A****U****A****A**GAUUU | |
|  |  | NZ\_AAGR01000046.1/13910-14082  | CCAAG**A****A****A****U****A****G**AGGUU**G****C****G****G****U****G**.**G****G****C**AAC-G**A****A****A****A****C****U**.**G****G**-.............**G****G****A****G****U****G****.****.****C****C****G****.****.****U****A****U**.**.****C****A****U**.GUU**G****A****A**....-**C****C****A****G**.**U****U****G****U****A**AGU**G****C****C**.-**A**.**C****C****G****C**CGA**A****A****U****G****U****U****G****C**.**A****C****G****.**....**A****U****A****U****U****C****G****G****.**A**U****C****G****U**..**G****C**-**G**.**C****A**..**U****U****G****G****G****C****C****A****U**.**G****G****G****U**..GCAUA**A****C****C****C**.**A****U****G****G****A****C****U**G**U****C****G****A****A****G****C**-.................ACUC........................................--**G****C****U****U****C****G****G**.GGCG**C****U****A****U****G****A**UUCUG | |
|  |  | NC\_000913.2/4231544-4231348  | CAGGC**C****A****G****A****A****G**AGGC.**G****C****G****U****U****G**.**C****C****C**A--**A**G**U****A****A****C****G****G**.**U****G****U**U............**G****G****A****G****G****A****.****.****.****.**-**G****C****C****A****G**.**.****U****C****C**.UGU**G****A****U**...A**A****C****A****C****C**.-**U****G****A****G**GGG**G****U****G**.**C****A**.**U****C****G****C**CGA**G****G****U****G****A****U****U****G**.**A****A****C****G**G...**C****U****G****G****C****C****.****.****.**A**C****G****U****U**..**C****A**-**U**.**C****A**..**U****C****G****G****C****U****A****C****A**.**G****G****G****G**CUGAA.U**C****C****C****C**.**U****G**-**G****G****U****U**G**U****C****A****C****C****A****G****A**AGCGUUCGCAGUCGGGCGUUUCGC.....................................A**A****G****U****G****G****U****G****G**.AGCA**C****U****U****C****U****G**GGUGA | |
|  |  | NC\_002655.2/5116767-5116571  | CAGGC**C****A****G****A****A****G**AGGC.**G****C****G****U****U****G**.**C****C****C**A--**A**G**U****A****A****C****G****G**.**U****G****U**U............**G****G****A****G****G****A****.****.****.****.**-**G****C****C****A****G**.**.****U****C****C**.UGU**G****A****U**...A**A****C****A****C****C**.-**U****G****A****G**GGG**G****U****G**.**C****A**.**U****C****G****C**CGA**G****G****U****G****A****U****U****G**.**A****A****C****G**G...**C****U****G****G****C****C****.****.****.**A**C****G****U****U**..**C****A**-**U**.**C****A**..**U****C****G****G****C****U****A****C****A**.**G****G****G****G**CUGAA.U**C****C****C****C**.**U****G**-**G****G****U****U**G**U****C****A****C****C****A****G****A**AGCGUUCGCAGUCGGGCGUUUCGC.....................................A**A****G****U****G****G****U****G****G**.AGCA**C****U****U****C****U****G**GGUGA | |
|  |  | NC\_002695.1/5086781-5086585  | CAGGC**C****A****G****A****A****G**AGGC.**G****C****G****U****U****G**.**C****C****C**A--**A**G**U****A****A****C****G****G**.**U****G****U**U............**G****G****A****G****G****A****.****.****.****.**-**G****C****C****A****G**.**.****U****C****C**.UGU**G****A****U**...A**A****C****A****C****C**.-**U****G****A****G**GGG**G****U****G**.**C****A**.**U****C****G****C**CGA**G****G****U****G****A****U****U****G**.**A****A****C****G**G...**C****U****G****G****C****C****.****.****.**A**C****G****U****U**..**C****A**-**U**.**C****A**..**U****C****G****G****C****U****A****C****A**.**G****G****G****G**CUGAA.U**C****C****C****C**.**U****G**-**G****G****U****U**G**U****C****A****C****C****A****G****A**AGCGUUCGCAGUCGGGCGUUUCGC.....................................A**A****G****U****G****G****U****G****G**.AGCA**C****U****U****C****U****G**GGUGA | |
|  |  | NC\_004337.1/4356133-4356329  | CAGGC**C****A****G****A****A****G**AGGC.**G****C****G****U****U****G**.**C****C****C**A--**A**G**U****A****A****C****G****G**.**U****G****U**U............**G****G****A****G****G****A****.****.****.****.**-**G****C****C****A****G**.**.****U****C****C**.UGU**G****A****U**...A**A****C****A****C****C**.-**U****G****A****G**GGG**G****U****G**.**C****A**.**U****C****G****C**CGA**G****G****U****G****A****U****U****G**.**A****A****C****G**G...**C****U****G****G****C****C****.****.****.**A**C****G****U****U**..**C****A**-**U**.**C****A**..**U****C****G****G****C****U****A****C****A**.**G****G****G****G**CUGAA.U**C****C****C****C**.**U****G**-**G****G****U****U**G**U****C****A****C****C****A****G****A**AGCGUUCGCAGUCGGGCGUUUCGC.....................................A**A****G****U****G****G****U****G****G**.AGCA**C****U****U****C****U****G**GGUGA | |
|  |  | NC\_004741.1/3415497-3415301  | CAGGC**C****A****G****A****A****G**AGGC.**G****C****G****U****U****G**.**C****C****C**A--**A**G**U****A****A****C****G****G**.**U****G****U**U............**G****G****A****G****G****A****.****.****.****.**-**G****C****C****A****G**.**.****U****C****C**.UGU**G****A****U**...A**A****C****A****C****C**.-**U****G****A****G**GGG**G****U****G**.**C****A**.**U****C****G****C**CGA**G****G****U****G****A****U****U****G**.**A****A****C****G**G...**C****U****G****G****C****C****.****.****.**A**C****G****U****U**..**C****A**-**U**.**C****A**..**U****C****G****G****C****U****A****C****A**.**G****G****G****G**CUGAA.U**C****C****C****C**.**U****G**-**G****G****U****U**G**U****C****A****C****C****A****G****A**AGCGUUCGCAGUCGGGCGUUUCGC.....................................A**A****G****U****G****G****U****G****G**.AGCA**C****U****U****C****U****G**GGUGA | |
|  |  | NZ\_AAGO01000092.1/9209-9034  | AACAA**A****A****U****U****A****G**AGGUU**G****C****A****A****A****G**.**A****C****G**A--**A**A**A****A****A****U****U****A**.**G****U**-.............**G****G****A****G****C****G****.****.****.****.**-**G****U****G****A****A**.**.****C****G****C**.AAU**G****A****A**....-**A****C****U****G**.**G****U****G**-**G**CGG**C****G****U**U**U****U**.**U****U****G****C**CGA**A****A****G****A****A****A****A****A**.**U****U**--....**G****U****C****A****C****A****G****.****.**.--**A****A**..**U****U****U****U**.**U****C**..**U****U****G****G****G****C****U****G****U**.**A****U****G****G**.UUAAAU**C****C****U**-.**A****C****A****G****A****C****U**G**U****C****U****U****U****C****U****G**UCAG.............UACUG.......................................A**C****A****G****A****A****G****G****G**.GGAG**C****U****A****A****U****C**AAAAU | |
|  |  | NZ\_AAGQ01000103.1/4368-4193  | CAAUA**C****A****A****U****A****G**AGGUC**G****C****G****A****U****C**G**U****U****C**A---C**A****A****A****A****G****C**.**A****A****G**G............**G****G****A****G****C****U****.****.****.****C****A****C****U****G****A****C**.**.****A****G****U**.GUU**G****A****G**...C**C****U****U****G****U**.**U****A****U****U****A**AGG**G****A****C**.-**A**.**U****C****G****C**CGA**A****A****U****C****A****G****C****A**.**G****U****G**-..GG**G****U****C****.****G****U****G****C****G**.-**C****G****C**..**U****G****U****U**.**G****G**..**U****U****G****G****G****C****U****G****C**.**A****G****G****A**..GAAAA**U****U****C****U**.**G****C****A****G****A****C****U**G**U****C****C****U****A****G**--.................UUUU........................................---**C****U****A****G****G****G**.AGCG**C****U****A****U****A****A**UUUUU | |
|  |  | NZ\_AACK01000044.1/2909-3092  | CAUUG**C****G****G****G****A****G**AGGC.**G****C****A****A**-**C**.**A****C****U**CAG-G**C****A****G****U****U****U**.**A****A****U**CCAA.........**G****G****C****A****A****C****.****.****.****.****U****A****G****G****G****C**.**C****G****U****U**.--U**G****A****G**...G**A****U****U****A****A**.**A****U****G****A****U**GGG**A****G****U**.**G****C**.**U****U****G****C**CGA**G****G****U****G****U****G****A****A**.**U****U****C**-.ACU**A****G****A****C****U****G****.****.****.**.-**G****A****A**..**U****G****A****A**.**C****A**..**U****C****G****G****C****U****G****A****G**U**C****A****G****G**UUGAA.U**C****C****U****A**.-**U****C****A****G****C****U**G**U****C****A****U****U****U****A****A**C................GCAAG.......................................U**U****U****A****A****A****U****G****G**.AGUG**C****U****C****U****G****A**UUAAU | |
|  |  | NC\_004547.2/4451556-4451748  | CAAGC**C****A****G****A****A****G**AGGC.**G****C****G****U****C****G**.**C****C****C**A--**G**G**U****A****A****G****A****U**.**A****U****C**.............**G****G****A****G****G****A****.****.****.****A****C****C****G****U****A****A**.**.****U****C****C**.GCU**G****A****U**....**G****A****U****A****U**.**C****C****G****A****G**GGG**G****A****G**.**C****G**.**A****C****G****C**CGA**G****A****U****G****C****G****G****U**.**G****A****A****A**....**U****U****.****C****G****G****C****.****.**.**U****U****U****C**..**A****C****C****C**.**U****A**..**U****C****G****A****C****U****A****C****A**.**G****A****G****G**CUGAA.U**C****C****U****C**.**U****G**-**G****G****U****U**G**U****C****A****C****C****G****G****A**UUCGUCCU.........GAUGGACGUCCAGC..............................A**A**--**G****G****U****G****G**.AGCG**C****U****U****C****U****G**GGUGU | |
|  |  | NC\_006512.1/1551517-1551340  | AGGCC**G****A****G****A****A****G**AGGU.**G****C****G****U****A****C**.**A****U****C**A--**G**G**G****A****C****U****U****U**.**U****G****A**GC...........**U****U****A****G****G****C****.****.****.****C****G****C****C****A****A****A**.**.****G****C****C**.AGA**G****U**-UAAG**U****C****G****A****A**.**A****G****A****G****A**GGG**G****A****U**.**G****U**C**A****C****G****C**CGA**G****G****U****A****A****U****A****U**.**U****G****U**-.CAU**U****U****U****G****G****C****.****.****.**.-**G****C****A**..**A****U****G****U**.**U****G**..**C****C****G****G****U****U****G****C****A**.**G****G****G****U**UUGAA.C**A****C****C****C**.-**G****C****A****A****C****U**G**U****C****A****C****C****U**--.................UUC-........................................---**A****G****G****U****G****G**.AGAG**C****U****U****C****U****G**GUGUU | |
|  |  | NC\_002940.2/1124335-1124144  | CAUUG**C****G****G****G****A****G**AGGC.**G****C****A****A**-**C**.**A****C****U**CAG-G**C****A****A****U****U****U**.**A****A****U**C............**U****U****A****G****G****C****.****.****.****A****A****C****U****A****G****G**.**.****G****C****C**.GUU**U****G****A**..GG**A****U****U****A****A**.**A****U****G****A****U**GGG**A****G****U**G**G****U**.**U****U****G****C**CGA**G****G****U****G****U****G****A****A**.**U****U****C**-....**A****C****U****A****G****A****C****U****G**.-**G****A****A**..**U****G****A****A**.**C****A**..**U****C****G****G****C****U****G****A****G**U**C****A****G****G**UUGAA.U**C****C****U****A**.-**U****C****A****G****C****U**G**U****C****A****U****U****U****A****A**AACGC............AUUAGCUG....................................U**U****U****A****A****A****U****G****G**.AGUG**C****U****C****U****G****A**UUAAU | |
|  |  | NZ\_AAIA01000001.1/376842-376648  | UUUCG**C****G****G****U****A****G**AGGU.**G****C****G****G****C****G**.**G****C****C**AUG**A**G**U****A****G****C****C****A**.**G****C****C**CGGAUUUUCCGAU**U****G****A****G****C****U****.****.****U****C****G****G****G****A****G****G**.**.****A****C****C**.GGC**G****A****A**.AGG**G****G****C**--.-**C****G****A****A**AGC**A****G****U**.**A****U**.**C****C****G****C**CGA**A****G****C****U****G****G****U****U**.**G****G****A**-..AA**C****C****U****C****A****A****G****.****.**.-**U****C****C**..**A****U****U****C**.**G****G**..**U****U****G****G****G**-**G****A****G**U**C****G****G****G**.CUAACA**C****C****U****G**.**C****U****C**-**A****C****U**G**U****C****A****C****C****C****G**-.................UUCACAG.....................................G-**C****G****G****G****U****G****G**.AGCG**C****U****A****U****C****G**GGAUU | |
|  |  | AACY01119379.1/30018-30189  | CCUUU**A****A****G****U****A****G**AGGC.**G****C****G****C****U****G**.**C****C****U**AUG**A**C**U****A****C****U****U****G**.**U****G****C**.............**G****G****A****G****G**-**.****.****.****.****G****U****G****A****U****G**.**.**-**C****C**.GCA**G****A****U**....**G****U****A****C****A**.**A****G****G****A****A**AGG**A****G****U**.**C****A**.**G****C****G****C**CGA**A****G****U****A****G****C****C****A**.**G****G****U**-....**C****A****U****C****A****A**-**.****.**.-**A****C****C**..**G****A****G****C**.**U****G**..**C****U****G****G****U****U****U****U****G**.**C****A****U****C**..AAAUA**G****G****U****G**.**C****A****A****G****A****C****U**G**C****C****A****U****A****G****U**-C................AUCC........................................--**A****C****U****A****U****G****G**.AGCG**C****U****A****C****C****U**GAAGG | |
|  |  | AACY01052642.1/1425-1596  | CCUUU**A****A****G****U****A****G**AGGC.**G****C****G****C****U****G**.**C****C****U**AUG**A**C**U****A****C****U****U****G**.**U****G****C**.............**G****G****A****G****G**-**.****.****.****.****G****U****G****A****U****G**.**.**-**C****C**.UGU**G****A****U**....**G****C****A****C****G**.**A****G****G****A****A**AGG**A****G****U**.**C****A**.**G****C****G****C**CGA**A****G****U****G****G****C****C****A**.**G****G**--...C**C****A****U****C****A****U****A****.****.**.--**C****C**..**G****G****G****C**.**U****G**..**C****U****G****G****U****U****C****U****G**.**C****A****U****C**..UAAUA**G****G****U****G**.**C****A****G****G****A****C****U**G**C****C****A****U****A****G****U**-C................AUCC........................................--**A****C****U****A****U****G****G**.AGCG**C****U****A****C****C****U**GAAGG | |
|  |  | AACY01000667.1/20871-20675  | CUUAG**A****A****G****U****A****G**AGGC.**G****C****A****C****C****A**.**A****A****U**AUC**A**G**U****A****G****U****G****A**.**U****C****A**.............**G****U****A****G****G**-**.****.****.****.****G****U****G****A****U****U**.**.**-**C****C**.GUU**G****A****U**....**C****G****U****U****C**.**A****C****G****A****A**AGG**A****U****U**.**U****G**.**G****U****G****C**CGA**A****G****U****A****G****U****U****U**.**G****A****U****U**....**U****A****U****C****A****C**-**.****.**.**G****A****U****C**..-**A****A****C**.**U****G**..**C****U****G****G****G****G****U****U****G**.**U****G****U****C**..UAAUA**G****G****U****A**.**C****A****A****C****A****C****U**G**C****C****A****U****A****G****U****A**UUUCCAGAUACGGA...UAUUAGGAAUAG................................U**U****A****C****U****A****U****G****G**.AGCG**C****U****A****C****U****G**AUAGG | |
|  |  | AACY01000707.1/27451-27647  | CUUAG**A****A****G****U****A****G**AGGC.**G****C****A****C****C****A**.**A****A****U**AUC**A**G**U****A****G****U****G****A**.**U****C****A**.............**G****U****A****G****G**-**.****.****.****.****G****U****G****A****U****U**.**.**-**C****C**.GUU**G****A****U**....**C****G****U****U****C**.**A****C****G****A****A**AGG**A****U****U**.**U****G**.**G****U****G****C**CGA**A****G****U****G****G****U****U****U**.**G****A****U****U**....**U****A****U****C****A****C**-**.****.**.**G****A****U****C**..-**A****A****C**.**U****G**..**C****U****G****G****G****G****U****U****G**.**U****G****C****C**..GAAUA**G****G****U****A**.**C****A****A****C****A****C****U**G**C****C****A****U****A****G****U****A**.................UUUUCUGAUACGGAUAUUAGGAAUAG..................U**U****A****C****U****A****U****G****G**.AGCG**C****U****A****C****U****G**AUAGG | |
|  |  | AACY01081919.1/740-566  | AGGAA**C****A****G****A****A****G**AGGA.**G****C****G****U****U****A**.**A****C****U**A--**G**G**U****A****G****U****C****A**.**G****U****C**.............**A****G****A****G****G****A****G****.****.****.****C****A****C****A****A****A**.**C****U****C****C**.AGC**G****A****U**....**G****A****U****U****G**.**A****U****G****A****G**GGA**G****A****U**.**U****A**.**A****C****G****C**CGA**G****G****C****A****U****A****G****A**.**U****G****U****G**...G**U****U****G****C****U****G**-**.****.**.**C****A****U****G**..**U****U****U****A**.**U****G**..**U****C****G****G****U****C****G****C****U**.**U****A****G****G**CUGAA.U**C****C****U**-.**A****A****C****G****A****U****U**G**U****C****A****C****C****U****G**-.................UAA-........................................--**U****U****G****G****U****G****G**.AGAG**C****U****U****C****U****G**GUGAC | |
|  |  | AACY01051883.1/29649-29823  | AGGAA**C****A****G****A****A****G**AGGA.**G****C****G****U****U****A**.**A****C****U**A--**G**G**U****A****G****U****C****A**.**G****U****C**.............**A****G****A****G****G****A****G****.****.****.****C****A****C****A****A****A**.**C****U****C****C**.GGC**G****A****U**....**G****A****U****U****G**.**A****U****G****A****G**GGA**G****A****U**.**U****A**.**A****C****G****C**CGA**G****G****C****A****U****A****G****A**.**U****G****U****G**...G**U****U****G****C****U****G**-**.****.**.**C****A****U****G**..**U****U****U****A**.**U****G**..**U****C****G****G****U****C****G****C****U**.**U****A****G****G**CUGAA.U**C****C****U**-.**A****A****C****G****A****U****U**G**U****C****A****C****C****U****G**-.................UAA-........................................--**U****U****G****G****U****G****G**.AGAG**C****U****U****C****U****G**GUGAC | |
|  |  | AACY01230106.1/64-241  | UAUGU**G****U****G****A****A****G**AGGU.**G****C****G****C****U****U**.**U****U****U**A--**A**G**C****A****U****A****U****G**.**A****G****C**.............**C****G****A****G****A****U****G****.****.****.****A****U****C****A****A****A**.**C****A****U****C**.UUU**G****A****A**....**A****C****U****C****A**.**U****A****G****A****G**-AG**A****A****U**.**A****A**.**G****C****G****C**CGA**A****A****U****A****A****G****G****C**.**A**-**U****A**...U**U****U****U****G****A****U****.****.****.**.**U****A****C****U**..**G****C****C****U**.**U****G**..**U****U****G****G****U****U****U****U****A**A**U****A****U****U**..GAA.A**G****A****U****A**C**U****A****A****A****A****C****U**G**U****C****G****U****G****U****A****A**U................UAAA........................................-**U****U****A****U****A****C****G****G**.AGCG**C****U****U****C****G****A**AAAUA | |
|  |  | AACY01043181.1/319-147  | ACAUU**C****A****G****A****A****G**AGGU.**G****C****G****A****U****A**.**C****U****U**A--**G**G**U****A****U****U****U****A**.**A****U****G**.............**G****U****A****G****G****U****.****.****.****.****G****A****C****G****A****A**.**C****G****C****C**.CAU**A****A****C**....**C****A****U****U****A**.**A****A****G****A****G**-GG**A****A****G**.**U****A**C**U****C****G****C**CGA**G****A****A****A****A****C****C****A**.**A****C****G****U**....**U****A****C****G****U****C****G****.****.**.**A****U****G****U**..**U****G****U****U**.**U****U**..**U****C****G****G****U****U****U****A****U**.**G****G****G****G**CUGAA.U**C****C****U****C**.**A**-**A****A****A****C****U**G**U****C****A****C****C****A****A****U**.................CU--........................................-**A**--**G****G****U****G****G**.AGAG**C****U****U****C****U****G**GCAGU | |
|  |  | AACY01688245.1/453-633  | UAGUU**A****G****G****A****A****G**AGGU.**G****C****A****U****G****C**.**A****C****U**A--**G**G**U****A****A****A****G****U**.**U****U****U**.............**U****G****A****A****G****A****C****.****.****.****G****C****C****A****A****C**.**G****U****C****U**.UUU**G****A****U**....**A****A****A****A****C**.**U****U****G****A****G**-GG**A****G****U**A**G****C**.**A****U****G****C**CGA**A****G****G****U****C****G****U****U**.**U****U****A****G**...A**G****U****U****G****G****U****U****.****.**.**C****U****A****A**..**A****A**-**G**.**A****U**..**C****U****G****G****U****U****U****C****U**.**A****A****G****G**CUGAG.U**C****C****U****U**.**A****U****U****A****A****C****U**G**U****C****A****U****C****A****A****G**.................CUCUG.......................................U**C****U****U****G****A****U****G****A**.AGCG**C****U****U****C****U****A**AGGCA | |
|  |  | AAGA01006314.1/568-765  | AGGGC**U****G****G****A****A****G**AGGU.**G****C****G****U****U**-A**U****U****C**A--**G**G**U****A****G****C****G****U**.**A****U****G**.............**U****U****A****G****G****A****G****.****.****.****C****A****C****A****A****A**.**C****U****C****C**.AUU**G****A****G**....**C****A****U****A****C**.**G****U****A****A****G**-GG**G****A****A**U**C****A**.**A****C****G****C**CGA**G****A****A****A****C****A****A****C**.**A****A****U**-...A**U****U****U****G****U****G****U****C****.**.-**A****U****U**..**G****U****U****G**.**U****U**..**U****C****G****G****G****C****G****A****C**.**U****A****G****G**UUGAA.U**C****C****U****A**.-**G****C****G****A****C****U**G**U****C****A****C****C****A****A**-UAGCUA...........UUUAAACGUAGCUUAUUAG.........................U-**U****U****G****G****U****G****G**.AGAG**C****U****U****C****U****G**GUCUU | |
|  |  | AACY01217159.1/270-443  | UAGAC**C****A****G****A****A****G**AGGA.**G****C****G****U****U****A**.**A****C****C**A--**G**G**U****A****G****A****A****C**.**A****U****C**.............**U****C****A****G****G****C****U****.****.****.****G****C****U****A****C****A**.**A****G****C****C**.------GCAG**G****A****U****G****U**.**U****U****G****A****G**GGG**G****A****U**.**U****A**.**A****C****G****C**CGA**G****G****A****U****C****A****G****A**.**U****G****C****.**....**U****G****U****A****G****C****.****.****.**.**G****G****C****A**..**G****A****U****G**.**A****U**..**U****C****G****G****U****U****G****U****U**.**A****A****G****G**CUGAA.U**C****C****U****U**.-**A****C****G****A****C****U**G**U****C****A****C****C****U****G**-.................UUUUU.......................................U-**G****U****G****G****U****G****G**.AGAG**C****U****U****C****U****G**GCCUU | |
|  |  | AACY01461732.1/433-254  | AACCC**G****U****G****A****A****G**AGGU.**G****C****A****U****C****U**.**G****G****C**CCG-U**U****A****A****G****C****U**.**U****U****A**.............**C****A****A****G****A****U****A****.****.****C****G****G****A****U****A****A**.**U****A****U****C**.AAU**G****C****G**....**U****A****A****A****G**.-**U****C****A****A**UCU**G****C****C**.**A****G**.**U****U****G****C**CGA**A****A****U****A****G****C****G****A**.**C****U****A****U**...C**U****U****A****U****C****C****A****A****.**.**A****U****A****G**..-**C****G****C**.**U****A**..**U****U****G****G****U****C****G****A****C**A**A****A****G****U**..GAA.A**A****C****U****U**C-**U****A****G****A****C****U**G**U****C****A****C****G****U****U****U**.................UUUU........................................-**A****A****A****C****G****U****G****G**.AGCG**C****U****U****C****A****G**GACAG | |
|  |  | SS\_cons |  | .....<<<<<<.....<<<<<<.<<<...[.[[<<<<.<<<.............<[[<<<<AAAAAAAAA.>>>>....]]>....>>>>>.>>]]]...>>>.>>.>>>>...<<<<<<<<.<<<<....aaaaaaaaa.>>>>..>>>>.>>..>><<<<<<<.<<<<.......>>>>.>>>>>>>.<<<<<<<<..............................................................>>>>>>>>.....>>>>>>..... |
|  |  | SS\_label |  | .....==P1==.....----P2----.......===P2a==.............=..-P2b..........P2b-......=....==P2a===......----P2-----...======P3=====..............=======P3========-----P4-----.......-----P4-----.===P5===..............................................................===P5===.....==P1==..... |
|  |  | RF |  | agaugaggUAGAGGu.GCgaua.aucAaGAGUAacuu.uuc.............gGAGgu....uaggaa..acC.gAUGAa....gaaaa.guGAAAGGgau.ua.ucGCCGAAguaAaua.aAag....uuccuaa...cuUu..uauU.ug..cUGGGccUg.uauu..gAAuAaaua.cAggACUGUCAcAauu.................uuua........................................uaauUgUGG.AGaGCUAccuuggga |
|  |  | SS\_align |  | :::::((((((,,,,.<<<<<<.<<<-------<<<<.<<<.............<<<<<<<...\_\_\_\_\_\_.>>>>.--->>>....>>>>>.>>------>>>.>>.>>>>,,,<<<<<<<<.<<<<....\_\_\_\_\_\_\_...>>>>..>>>>.>>..>><<<<<<<.<<<<..\_\_\_\_\_>>>>.>>>>>>>,<<<<<<<<.................\_\_\_\_........................................\_>>>>>>>>.,,,,))))))::::: |
