# Supplementary material for: The distributions, mechanisms, and structures of metabolite-binding riboswitches
Source: Genome Biol. 2007 Nov 12;8(11):R239. doi: 10.1186/gb-2007-8-11-r239 (PMC2258182; doi:10.1186/gb-2007-8-11-r239)
Supplement: Additional data file 2 — Sequence alignments of the riboswitch aptamer data sets annotated with new base-base interactions in HTML format. [file gb-2007-8-11-r239-S2.zip › HTML/PreQ1.html]

|  |  |  |  |  |
| --- | --- | --- | --- | --- |
|  |  | **Accession/Start-End** |  | **Sequence** |
|  |  | NC\_004193.1/467687-467621  | AAUGUGAUUUUAUCAC**C****G****U****G****G**UUCGUA-...ACCAUC**C****C****A****C****G**.....................CAAAAAAACUAGGAAGGAAAAUUAAAUAU | |
|  |  | NC\_006300.1/1848379-1848445  | AUGCACUCUUUUAAUU**C****G****U****G****G**UUCGAAA...ACC.UC**C****C****A****C****G**.....................CAAAAAAACUAAGGAAAACGAUGAAUAUU | |
|  |  | NC\_004722.1/1559900-1559966  | CUUAAAAAACGAAUAA**C****G****U****G****G**UUCGAA-...ACCAUC**C****C****A****C****G**.....................UAAAAAAACUAAGGAGAUUUUGUCAUGAA | |
|  |  | NC\_005957.1/1568652-1568718  | CUUAAAAAACGAAUAA**C****G****U****G****G**UUCGAA-...ACCAUC**C****C****A****C****G**.....................UAAAAAAACUAAGGAGAUUUUGUCAUGAA | |
|  |  | NC\_006274.1/1567270-1567336  | CUUAAAAAACGAAUAA**C****G****U****G****G**UUCGAA-...ACCAUC**C****C****A****C****G**.....................UAAAAAAACUAAGGAGAUUUUGUCAUGAA | |
|  |  | NZ\_AAEK01000008.1/51790-51724  | CUUAAAAAACGAAUAA**C****G****U****G****G**UUCGAA-...ACCAUC**C****C****A****C****G**.....................UAAAAAAACUAAGGAGAUUUUGUCAUGAA | |
|  |  | NZ\_AAEN01000011.1/111959-112025  | CUUAAAAAACGAAUAA**C****G****U****G****G**UUCGAA-...ACCAUC**C****C****A****C****G**.....................UAAAAAAACUAAGGAGAUUUUGUCAUGAA | |
|  |  | NZ\_AAEO01000025.1/111577-111643  | CUUAAAAAACGAAUAA**C****G****U****G****G**UUCGAA-...ACCAUC**C****C****A****C****G**.....................UAAAAAAACUAAGGAGAUUUUGUCAUGAA | |
|  |  | NZ\_AAEP01000035.1/114178-114244  | CUUAAAAAACGAAUAA**C****G****U****G****G**UUCGAA-...ACCAUC**C****C****A****C****G**.....................UAAAAAAACUAAGGAGAUUUUGUCAUGAA | |
|  |  | NZ\_AAER01000023.1/103001-103067  | CUUAAAAAACGAAUAA**C****G****U****G****G**UUCGAA-...ACCAUC**C****C****A****C****G**.....................UAAAAAAACUAAGGAGAUUUUGUCAUGAA | |
|  |  | NC\_003909.8/1668103-1668169  | CUAAAAAAACGAAUAA**C****G****U****G****G**UUCGAA-...ACCAUC**C****C****A****C****G**.....................UAAAAAAACUAAGGAGAUUUUGUCAUGAA | |
|  |  | NZ\_AABH02000024.1/1173-1240  | UCUGAGACUUAAUAAU**A****G****C****G****G**UUCAUCA...ACCAUC**C****C****G****C****U**.....................UAAAAAAACUAGGAGAUACUAUUUAUGCA | |
|  |  | NC\_003997.3/1542388-1542454  | CUUAAAAAACGAAUAA**C****G****U****G****G**UUCGAA-...ACCAUC**C****C****A****C****G**.....................UAAAAAAACUAAGGAGAUUUUGUCGUGAA | |
|  |  | NC\_005945.1/1542465-1542531  | CUUAAAAAACGAAUAA**C****G****U****G****G**UUCGAA-...ACCAUC**C****C****A****C****G**.....................UAAAAAAACUAAGGAGAUUUUGUCGUGAA | |
|  |  | NC\_007530.2/1542511-1542577  | CUUAAAAAACGAAUAA**C****G****U****G****G**UUCGAA-...ACCAUC**C****C****A****C****G**.....................UAAAAAAACUAAGGAGAUUUUGUCGUGAA | |
|  |  | NZ\_AAES01000034.1/113941-114007  | CUUAAAAAACGAAUAA**C****G****U****G****G**UUCGAA-...ACCAUC**C****C****A****C****G**.....................UAAAAAAACUAAGGAGAUUUUGUCGUGAA | |
|  |  | NC\_003909.8/1455246-1455311  | CCGUGUAAUGCACGGG**A****G****A****G****G**UUCGCGA...AC-.UC**C****C****U****C****U**.....................AUAAAAAACUAUGGAAACAACAAUAUCAU | |
|  |  | NC\_000907.1/1258314-1258248  | CCACUAGAAUACCCCC**C****G****U****A****G**UUCGCAA...ACC.UC**C****U****A****C****A**.....................AUAAAAAACUAGGUAAAAUAUGAAUAUUU | |
|  |  | NC\_007146.1/1290277-1290211  | CCACUAGAAUACCCCC**C****G****U****A****G**UUCGCAA...ACC.UC**C****U****A****C****A**.....................AUAAAAAACUAGGUAAAAUAUGAAUAUUU | |
|  |  | NZ\_AADO01000010.1/21811-21877  | CCACUAGAAUACCCCC**C****G****U****A****G**UUCGCAA...ACC.UC**C****U****A****C****A**.....................AUAAAAAACUAGGUAAAAUAUGAAUAUUU | |
|  |  | NZ\_AADP01000001.1/666704-666638  | CCACUAGAAUACCCCC**C****G****U****A****G**UUCGCAA...ACC.UC**C****U****A****C****A**.....................AUAAAAAACUAGGUAAAAUAUGAAUAUUU | |
|  |  | NZ\_AAET01000014.1/9613-9547  | CCACUAGAAUACCCCC**C****G****U****A****G**UUCGCAA...ACC.UC**C****U****A****C****A**.....................AUAAAAAACUAGGUAAAAUAUGAAUAUUU | |
|  |  | NC\_003997.3/1298760-1298825  | CCGUGCAAUGCACGGG**A****G****A****G****G**UUCGCGA...AC-.UC**C****C****U****C****U**.....................AUAAAAAACUAUGGAAACAACAAUAUCUU | |
|  |  | NC\_005945.1/1298829-1298894  | CCGUGCAAUGCACGGG**A****G****A****G****G**UUCGCGA...AC-.UC**C****C****U****C****U**.....................AUAAAAAACUAUGGAAACAACAAUAUCUU | |
|  |  | NC\_005957.1/1321418-1321483  | CCGUGCAAUGCACGGG**A****G****A****G****G**UUCGCGA...AC-.UC**C****C****U****C****U**.....................AUAAAAAACUAUGGAAACAACAAUAUCUU | |
|  |  | NC\_006274.1/1334225-1334290  | CCGUGCAAUGCACGGG**A****G****A****G****G**UUCGCGA...AC-.UC**C****C****U****C****U**.....................AUAAAAAACUAUGGAAACAACAAUAUCUU | |
|  |  | NC\_007530.2/1298883-1298948  | CCGUGCAAUGCACGGG**A****G****A****G****G**UUCGCGA...AC-.UC**C****C****U****C****U**.....................AUAAAAAACUAUGGAAACAACAAUAUCUU | |
|  |  | NZ\_AAAC02000001.1/1790824-1790889  | CCGUGCAAUGCACGGG**A****G****A****G****G**UUCGCGA...AC-.UC**C****C****U****C****U**.....................AUAAAAAACUAUGGAAACAACAAUAUCUU | |
|  |  | NZ\_AAEK01000009.1/73639-73574  | CCGUGCAAUGCACGGG**A****G****A****G****G**UUCGCGA...AC-.UC**C****C****U****C****U**.....................AUAAAAAACUAUGGAAACAACAAUAUCUU | |
|  |  | NZ\_AAEN01000008.1/125253-125188  | CCGUGCAAUGCACGGG**A****G****A****G****G**UUCGCGA...AC-.UC**C****C****U****C****U**.....................AUAAAAAACUAUGGAAACAACAAUAUCUU | |
|  |  | NZ\_AAEO01000037.1/74130-74065  | CCGUGCAAUGCACGGG**A****G****A****G****G**UUCGCGA...AC-.UC**C****C****U****C****U**.....................AUAAAAAACUAUGGAAACAACAAUAUCUU | |
|  |  | NZ\_AAEP01000030.1/105001-105066  | CCGUGCAAUGCACGGG**A****G****A****G****G**UUCGCGA...AC-.UC**C****C****U****C****U**.....................AUAAAAAACUAUGGAAACAACAAUAUCUU | |
|  |  | NZ\_AAEQ01000023.1/105050-105115  | CCGUGCAAUGCACGGG**A****G****A****G****G**UUCGCGA...AC-.UC**C****C****U****C****U**.....................AUAAAAAACUAUGGAAACAACAAUAUCUU | |
|  |  | NZ\_AAER01000019.1/125055-124990  | CCGUGCAAUGCACGGG**A****G****A****G****G**UUCGCGA...AC-.UC**C****C****U****C****U**.....................AUAAAAAACUAUGGAAACAACAAUAUCUU | |
|  |  | NZ\_AAES01000029.1/105020-105085  | CCGUGCAAUGCACGGG**A****G****A****G****G**UUCGCGA...AC-.UC**C****C****U****C****U**.....................AUAAAAAACUAUGGAAACAACAAUAUCUU | |
|  |  | NC\_003366.1/2295338-2295272  | UCCAAUGUAAUUUGGU**A****G****A****G****G**UUCGUA-...ACCAUC**C****C****U****C****U**.....................AUAAAAAACUAAGGGCUGUAACUGUAUUA | |
|  |  | NC\_004567.1/482217-482284  | UUUGUUUCAAUCAAUA**C****G****U****G****G**UUCGUA-...ACCAUC**C****C****A****C****G**....................UUAAAAAAACUAGGAGGAAUUAAGCGAAUG | |
|  |  | NC\_004557.1/2478227-2478296  | AUAAAAAAAUAAUAUG**G****A****C****A****G**UUCGUA-...ACCAUC**C****U****G****U****C**..................CCUAAAUAAAACUAUGGAGGUAUAUUAUGAUU | |
|  |  | NC\_004116.1/545608-545542  | AUCGAGUAUUUCUCGG**A****C****U****G****G**UUCGAAA...ACU.UC**C****C****A****G****A**.....................AUAAAAAACUAAGUGACUGAAAAUAUGAU | |
|  |  | NC\_004368.1/600207-600141  | AUCGAGUAUUUCUCGG**A****C****U****G****G**UUCGAAA...ACU.UC**C****C****A****G****A**.....................AUAAAAAACUAAGUGACUGAAAAUAUGAU | |
|  |  | NC\_003112.1/329231-329165  | UAUUACAAUCGCCGCC**C****G****U****G****G**UUCGAAA...ACC.UC**C****C****A****C****A**.....................UUAAAAAACUAAGGAAACCCUCAUGUCCC | |
|  |  | NC\_004668.1/2504798-2504864  | AUCGAGAAUUUCUCGG**A****C****U****G****G**UUCGGAA...ACU.UC**C****C****A****G****A**.....................AUAAAAAACUAAGUAUCUCUCAAUACUAA | |
|  |  | NC\_002570.2/2373353-2373288  | ACAGGUUUUCUGUGAG**A****G****A****G****G**UUCGCGA...AC-.UC**C****C****U****C****U**.....................AUAAAAAACUAAGGCAAGGCUGUAUCAAG | |
|  |  | NZ\_AAEQ01000029.1/115662-115727  | CUUAAAAAACGAAUAA**C****G****U****G****G**UUCGAA-...ACCAUC**C****C****A****C****G**.....................-UAAAAAACUAAGGAGAUUUUGUCAUGAA | |
|  |  | NC\_004193.1/2885887-2885818  | UCUGCACUACUGCAGG**A****G****A****G****G**UUCGCGAUUUAAA.UC**C****C****U****C****U**.....................AUAAAAAACUAAGGAGAUAAACAACACGC | |
|  |  | NC\_004722.1/1318948-1319013  | CCGUGCACCGCGCGGG**A****G****A****G****G**UUCGCGA...AC-.UC**C****C****U****C****U**.....................AUAAAAAACUAUGGAAACAACAAUAUCCU | |
|  |  | NZ\_AAAK03000099.1/1618-1684  | AAGACGCUCGUCUUGG**A****C****U****G****G**UUCGGAA...ACU.UC**C****C****A****G****A**.....................AUAAAAAACUAAGUAUCUCUAAAGAAAGG | |
|  |  | NC\_004668.1/2571400-2571333  | CUGUUGCAAUUGUAAC**C****A****A****G****G**UUCAUCA...ACCAUC**C****C****U****U****G**.....................UAAAAAAACUCGGAGAAAGAAGGAAAAAG | |
|  |  | NC\_003030.1/2531288-2531219  | AGUUUUCUUAAUUUAA**G****A****C****A****G**UUCGAA-...ACCAUC**C****U****G****U****C**..................UAUAAAUAAAACUAUGGAGGUAAAUUUACAUG | |
|  |  | NC\_002946.2/1637035-1637101  | AUUACAAUCGCCGCCC**C****G****U****G****G**UUCGAAA...ACC.UC**C****C****A****C****A**.....................CUAAAAAACUAAGGAAACCCUAUGUCCCG | |
|  |  | NC\_003116.1/2115289-2115355  | AUUACAAUCGCCGCCC**C****G****U****G****G**UUCGAAA...ACC.UC**C****C****A****C****A**.....................CUAAAAAACUAAGGAAACCCUAUGUCCCG | |
|  |  | NC\_002973.5/907964-908033  | UGGCAAAAUUGCCACA**C****G****U****G****G**UUCAUUCAU.ACCAUC**C****C****A****C****G**.....................UAAAAAAACUAGGAGGAAAAAUAAUGAAA | |
|  |  | NC\_003210.1/907915-907984  | UGGCAAAAUUGCCACA**C****G****U****G****G**UUCAUUCAU.ACCAUC**C****C****A****C****G**.....................UAAAAAAACUAGGAGGAAAAAUAAUGAAA | |
|  |  | NC\_003212.1/896842-896911  | UGGCAAAAUUGCCACA**C****G****U****G****G**UUCAUUCAU.ACCAUC**C****C****A****C****G**.....................UAAAAAAACUAGGAGGAAAAAUAAUGAAA | |
|  |  | NZ\_AADQ01000002.1/54240-54171  | UGGCAAAAUUGCCACA**C****G****U****G****G**UUCAUUCAU.ACCAUC**C****C****A****C****G**.....................UAAAAAAACUAGGAGGAAAAAUAAUGAAA | |
|  |  | NZ\_AADR01000005.1/51840-51909  | UGGCAAAAUUGCCACA**C****G****U****G****G**UUCAUUCAU.ACCAUC**C****C****A****C****G**.....................UAAAAAAACUAGGAGGAAAAAUAAUGAAA | |
|  |  | NC\_006582.1/2249581-2249516  | ACGAUAAUUAGAAAGA**A****G****A****G****G**UUCGCGA...AC-.UC**C****C****U****C****U**.....................AUAAAAAACUAACCAAUUGUUGGAGUAUC | |
|  |  | NC\_004193.1/2245816-2245748  | CAAACAUAAUUUUGUU**A****G****A****G****G**UUCUUAGCU.UCA.AC**C****C****U****C****U**.....................AUAAAAAACUAAGGACAAACAAUGAUUUA | |
|  |  | NC\_004567.1/2519571-2519505  | UAAUUAGGCGAGCUGG**C****C****U****G****G**UUCGUAA...ACU.UC**C****C****A****G****G**.....................AUAAAAAACCAAGAACUUCAGUUAUCGGA | |
|  |  | NC\_006270.2/1500838-1500904  | CGCUUGAAACUGCGGG**A****G****A****G****G**UUC-UAGC..AAA.AC**C****C****U****C****U**.....................AUAAAAAACUAAGGAAAGCUGUAUCCUUG | |
|  |  | NC\_006322.1/1501699-1501765  | CGCUUGAAACUGCGGG**A****G****A****G****G**UUC-UAGC..AAA.AC**C****C****U****C****U**.....................AUAAAAAACUAAGGAAAGCUGUAUCCUUG | |
|  |  | NZ\_AACK01000040.1/4344-4427  | AGACCGCUUAUUCUUU**C****G****U****G****G**UUCGCAA...ACC.UC**C****C****A****C****G**....CCAAAGAUUCUCUUUGAAAUCAAAACUAAGGAUACAAAAUGAAUUC | |
|  |  | NZ\_AADW02000018.1/25014-24948  | GUUGAGACUGUGUCUC**C****G****G****A****G**UUCGUA-...ACC.UC**C****U****C****C****G**....................UCACAAAAACUAGGAAUGGAAGUGUAUCCA | |
|  |  | NC\_002946.2/139426-139493  | CUUUACUGUCCAACUU**C****G****C****G****G**UUCGCAA...ACC.UC**C****C****G****C****G**....................UUACCAAAACUAGGAUUCGAUAUGUCAAAC | |
|  |  | NC\_003112.1/546205-546272  | CUUUACUGUCCAACUU**C****G****C****G****G**UUCGCAA...ACC.UC**C****C****G****C****G**....................UUACCAAAACUAGGAUUCGAUAUGUCAAAC | |
|  |  | NC\_003116.1/692411-692478  | CUUUACUGUCCAACUU**C****G****C****G****G**UUCGAAA...ACC.UC**C****C****G****C****G**....................UCACCAAAACUAGGAUUCGAUAUGUCAAAC | |
|  |  | NZ\_AABH02000005.1/34664-34729  | UCGGCAAUCGUGCCGG**A****C****U****G****G**UUCGGAA...AA-.UC**C****C****A****G****A**.....................AAAAAAAACCAAGUGUGAUUGUAUUGUAU | |
|  |  | NC\_002976.3/370935-370867  | CGUGCAAAAUAAUAAC**A****G****A****G****G**UUCCUAGCCGAA-.AC**C****C****U****C****U**.....................AUAAAAAACUAGACAUGAAAAUUUCAACA | |
|  |  | NC\_004461.1/477815-477747  | CGUGCAAAAUAAUAAC**A****G****A****G****G**UUCCUAGCCGAA-.AC**C****C****U****C****U**.....................AUAAAAAACUAGACAUGAAAAUUUCAACA | |
|  |  | NC\_002663.1/209176-209089  | UACUGUUUCUUUUCAU**A****G****U****G****G**UUCGUA-...ACCCUC**C****C****A****C****U**UGAACAACCAACAAUUGUUCGAAACAAAACUAGGAAAAUAGUAUGCAGUU | |
|  |  | NC\_002951.2/793698-793630  | UACAUAAAUGAAUAUC**A****G****A****G****G**UUCCUAGCUGAA-.AC**C****C****U****C****U**.....................AUAAAAAACUAGACAUUGAAAUUUCAAAC | |
|  |  | NC\_002952.2/800141-800073  | UACAUAAAUGAAUAUC**A****G****A****G****G**UUCCUAGCUGAA-.AC**C****C****U****C****U**.....................AUAAAAAACUAGACAUUGAAAUUUCAAAC | |
|  |  | NC\_002953.3/755309-755241  | UACAUAAAUGAAUAUC**A****G****A****G****G**UUCCUAGCUGAA-.AC**C****C****U****C****U**.....................AUAAAAAACUAGACAUUGAAAUUUCAAAC | |
|  |  | NC\_003923.1/756881-756813  | UACAUAAAUGAAUAUC**A****G****A****G****G**UUCCUAGCUGAA-.AC**C****C****U****C****U**.....................AUAAAAAACUAGACAUUGAAAUUUCAAAC | |
|  |  | NC\_000964.2/1438571-1438637  | CCGUGCGAUAUGCGGG**A****G****A****G****G**UUC-UAGCU.AC-.AC**C****C****U****C****U**.....................AUAAAAAACUAAGGACGAGCUGUAUCCUU | |
|  |  | NC\_006570.1/1231616-1231683  | UAGCAGAUUUUUAAUC**C****C****C****U****G**UUCGCA-...ACUAUC**C****A****G****G****G**....................UUAACAAAACUAUAGGAAAGCAAAUGACUA | |
|  |  | NC\_003366.1/1750878-1750809  | GGAAAAAUAAUAUAUA**G****A****C****A****G**UUCGAAA...GCC.UC**C****U****G****U****C**..................UUUAAAUAAAACUACGGUGUUUUAAGUAUCAU | |
|  |  | NC\_006510.1/1000346-1000413  | CGUACGCUUGUACGGG**A****G****A****G****G**UUUCUAGC..AAA.AC**C****C****U****C****U**.....................AUAAAAAACUAGGGACGGCUGUAUCCUAG | |
|  |  | NC\_006582.1/964307-964240  | UGUCCUACGGCAACAA**C****G****C****A****G**UUCGAG-...ACCUUC**C****U****G****C****G**....................UUAUCAAAACUAAGGAGAUUUUGCUAAAUG | |
|  |  | NC\_003030.1/3824123-3824193  | UGUUUUCUUGUAAUAA**G****A****C****A****G**UUCGCAA...UCCAUC**C****U****G****U****C**..................UAUAAACAAAACCAGGGCAAUAUCUACCGGGU | |
|  |  | NZ\_AAEV01000006.1/90356-90285  | UGAUACUAACACACAC**G****G****U****G****G**UUCGAAAUA.UCC.UC**C****C****G****C****C**..................UCAAACUAAAACUAGGAGAGUUACAUAUGCAC | |
|  |  | NC\_002951.2/1478097-1478164  | AGUUAGUUAUUUAUUG**A****G****C****G****G**UUUCUAA...AC-.AC**C****C****G****C****A**...................UGAUCAAAAUUUAGGAGGACAUUUUAAUAUG | |
|  |  | NC\_002745.2/1436217-1436284  | AGUAAGUUAUUUAUUG**A****G****C****G****G**UUUCUAA...AC-.AC**C****C****G****C****A**...................UGAUCAAAAUUUAGGAGGACAUUUUAAUAUG | |
|  |  | NC\_002758.2/1512628-1512695  | AGUAAGUUAUUUAUUG**A****G****C****G****G**UUUCUAA...AC-.AC**C****C****G****C****A**...................UGAUCAAAAUUUAGGAGGACAUUUUAAUAUG | |
|  |  | NC\_002953.3/1468326-1468393  | AGUAAGUUAUUUAUUG**A****G****C****G****G**UUUCUAA...AC-.AC**C****C****G****C****A**...................UGAUCAAAAUUUAGGAGGACAUUUUAAUAUG | |
|  |  | NC\_003923.1/1439771-1439838  | AGUAAGUUAUUUAUUG**A****G****C****G****G**UUUCUAA...AC-.AC**C****C****G****C****A**...................UGAUCAAAAUUUAGGAGGACAUUUUAAUAUG | |
|  |  | NC\_002745.2/761248-761180  | UACAUAAAUGAAUAUC**A****G****A****G****G**UUUCUAGCUGAA-.AC**C****C****U****C****U**.....................AUAAAAAACUAGACAUUGAAAUUUCAAAC | |
|  |  | NC\_002758.2/785497-785429  | UACAUAAAUGAAUAUC**A****G****A****G****G**UUUCUAGCUGAA-.AC**C****C****U****C****U**.....................AUAAAAAACUAGACAUUGAAAUUUCAAAC | |
|  |  | NC\_006449.1/767953-768021  | AAAGAUAAAUAAUUUA**C****G****A****U****G**UUUGGGAG..AC-.AC**C****A****U****C****G**...................CUAAAAAAAACCAGUAAUGGCGUUUUUUAGU | |
|  |  | NC\_002940.2/428646-428559  | UUAUUUUUCUUAAUCU**C****G****U****G****G**UUCGAAA...ACC.UC**C****C****A****U****G**CUUAAAAGCAUUGCUUUUCAAAAUCAAAACUAAGGAACACAAA------- | |
|  |  | NC\_002976.3/1021872-1021939  | UAGUAUAGUUAAUUUU**G****G****C****G****G**UUUCUAA...AC-.AC**C****C****G****C****U**...................UUAACAAAAUUUAGGAGGAAAGUUAUAUGUA | |
|  |  | NC\_004461.1/1133813-1133880  | UAGUAUAGUUAAUUUU**G****G****C****G****G**UUUCUAA...AC-.AC**C****C****G****C****U**...................UUAACAAAAUUUAGGAGGAAAGUUAUAUGUA | |
|  |  | NC\_006448.1/761530-761598  | AAAGAUAAAUAAUUUA**C****G****A****U****G**UUUGGGAG..AC-.AC**C****A****U****C****A**...................CUAAAAAAAACCAGUAAUGGCGUUUUUUAGU | |
|  |  | NC\_002952.2/1501417-1501484  | AGUAAGUUAUUUAUUG**A****G****C****G****G**UUUCUAA...AC-.AC**C****C****G****U****A**...................UGAUCAAAAUUUAGGAGGACAUUUUAAUAUG | |
|  |  | NC\_002163.1/162492-162416  | UGAACUUGAUAAACAA**A****U****C****A****G**UUCAAAAAUCAC-.UC**C****U****G****U****U**.............UUAAGAACACUAAAAACUAUGGAACAUAUCAUAGAAG | |
|  |  | NC\_004350.1/874174-874106  | UUGAAGAAUUACAUAA**G****G****A****U****G**UUUGGGAG..AA-.GC**C****A****U****C****U**...................AUAAAAAAAACCAGCCUAUUAUUUGGCAGCU | |
|  |  | NZ\_AABF02000039.1/8915-8981  | UUUCUUUAAAUUAAGU**A****G****A****U****G**UGC-UAGC..AAA.AC**C****A****U****C****U**.....................UUAAAAAACUAGACUUGGGGUGCAAGUCC | |
|  |  | NC\_003454.1/498141-498207  | AGAAAUUUUAAUAAGU**A****G****A****U****G**UGC-UAGC..AAA.AC**C****A****U****C****U**.....................UUAAAAAACUAGACUUGGGGUGCAAGUCC | |
|  |  | SS\_cons |  | ................<<<<<................>>>>>.................................................. |
|  |  | SS\_label |  | ................==P1=................=P1==.................................................. |
|  |  | RF |  | uguuaaauuuuauaugcGuGGUUCGuAA...ACC.UCCCaCg.....................AAAAAAAACUAgGgAaaaauauuuaaauu |
|  |  | SS\_align |  | ::::::::::::::::<<<<<\_\_\_\_\_\_\_...\_\_\_.\_\_>>>>>.....................::::::::::::::::::::::::::::: |
