# Supplementary material for: The distributions, mechanisms, and structures of metabolite-binding riboswitches
Source: Genome Biol. 2007 Nov 12;8(11):R239. doi: 10.1186/gb-2007-8-11-r239 (PMC2258182; doi:10.1186/gb-2007-8-11-r239)
Supplement: Additional data file 2 — Sequence alignments of the riboswitch aptamer data sets annotated with new base-base interactions in HTML format. [file gb-2007-8-11-r239-S2.zip › HTML/Purine.html]

|  |  |  |  |  |
| --- | --- | --- | --- | --- |
|  |  | **Accession/Start-End** |  | **Sequence** |
|  |  | NC\_003869.1/586365-586467  | AAAAAUUUAAUAAGA.AG.**C****A****C****U****C****A****U****A****U**AA**U****C****C****C****G****A****G**A.**A****U**.**A**U**G****G****C****U****C****G****G****G****A**.**G****U**CUC**U****A****C****C****G****A****A****C****A****A****C****C**..GU**A****A****A****U****U****G****U****U****C**.**G**.AC**U****A****U****G****A****G****U****G**AAAGU.GUACCUAGGG | |
|  |  | NC\_002570.2/1593074-1592972  | AUUUACAUUAAAAAA.AG.**C****A****C****U****C****G****U****A****U**AA**U****C****G****C****G****G****G**A.**A****U**.**A**G**G****G****C****C****C****G****C****A****A**.**G****U**UUC**U****A****C****C****A****G****G****C****U****G****C****C**..GU**A****A****A****C****A****G****C****C****U**.**G**.AC**U****A****C****G****A****G****U****G**AUACU.UUGACAUAGA | |
|  |  | NC\_002570.2/676475-676577  | CGUUCUUUAUAUAAA.GU.**A****C****C****U****C****A****U****A****U**AA**U****C****U****U****G****G****G**A.**A****U**.**A**U**G****G****C****C****C****A****A****A****A**.**G****U**UUC**U****A****C****C****U****G****C****U****G****A****C****C**..GU**A****A****A****U****C****G****G****C****G**.**G**.AC**U****A****U****G****G****G****G****A**AAGAU.UUUGGAUCUU | |
|  |  | NC\_000964.2/4004541-4004643  | CAUCUUAGAAAAAGA.CA.**U****U****C****U****U****G****U****A****U**AU**G****A****U****C****A****G****U**A.**A****U**.**A**U**G****G****U****C****U****G****A****U****U**.**G****U**UUC**U****A****C****C****U****A****G****U****A****A****C****C**..GU**A****A****A****A****A****A****C****U****A**.**G**.AC**U****A****C****A****A****G****A****A**AGUUU.GAAUAAAUUU | |
|  |  | NC\_003366.1/513088-512986  | UAAGUGUAUUAAAUU.UU.**A****A****C****U****C****G****U****A****U**AU**A****A****U****C****G****G****U**A.**A****U**.**A**U**G****G****U****C****C****G****A****A****A**.**G****U**UUC**U****A****C****C****U****G****C****U****A****A****C****C**..GU**A****A****A****A****U****A****G****C****A**.**G**.AC**U****A****C****G****A****G****G****A**GUUGU.ACUAUAAAUU | |
|  |  | NC\_000964.2/693775-693877  | AGAAAUCAAAUAAGA.UG.**A****A****U****U****C****G****U****A****U**AA**U****C****G****C****G****G****G**A.**A****U**.**A**U**G****G****C****U****C****G****C****A****A**.**G****U**CUC**U****A****C****C****A****A****G****C****U****A****C****C**..GU**A****A****A****U****G****G****C****U****U**.**G**.AC**U****A****C****G****U****A****A****A**CAUUU.CUUUCGUUUG | |
|  |  | NC\_002570.2/650309-650411  | AAUAAAUCGAAAACA.UC.**A****U****U****U****C****G****U****A****U**AA**U****G****G****C****A****G****G**A.**A****U**.**A**G**G****G****C****C****U****G****C****G****A**.**G****U**UUC**U****A****C****C****A****A****G****C****U****A****C****C**..GU**A****A****A****U****A****G****C****U****U**.**G**.AC**U****A****C****G****A****A****A****A**UAAUG.GGUUUUUUAC | |
|  |  | NC\_006270.2/2294931-2294831  | AAUUUGAUACAUUAU.AU.**C****A****C****U****C****A****U****A****U**AA**U****C****G****C****G****U****G**G.**A****U**.**A**U**G****G****C****A****C****G****C****A****A**.**G****U**UUC**U****A****C****C****G****G****G****C****A**-**C****C**..GU**A****A****A**-**U****G****U****C****C**.**G**.AC**U****A****U****G****A****G****U****G**GGCGA.UAAGAAAACG | |
|  |  | NC\_006322.1/2295788-2295688  | AAUUUGAUACAUUAU.AU.**C****A****C****U****C****A****U****A****U**AA**U****C****G****C****G****U****G**G.**A****U**.**A**U**G****G****C****A****C****G****C****A****A**.**G****U**UUC**U****A****C****C****G****G****G****C****A**-**C****C**..GU**A****A****A**-**U****G****U****C****C**.**G**.AC**U****A****U****G****A****G****U****G**GGCGA.UAAGAAAACG | |
|  |  | NC\_004193.1/1103943-1104045  | AAACCUUAUAUAUAG.UU.**U****U****U****U****C****A****U****A****U**AA**U****C****G****C****G****G****G**G.**A****U**.**A**U**G****G****C****C****U****G****C****A****A**.**G****U**UUC**U****A****C****C****G****G****U****U****U****A****C****C**..GU**A****A****A****U****G****A****A****C****C**.**G**.AC**U****A****U****G****G****A****A****A**AGCGG.AAAAUUCGAU | |
|  |  | NC\_003909.8/1650407-1650509  | AAAUAAAUAGUUAGC.UA.**C****A****C****U****C****A****U****A****U**AA**U****C****G****C****G****G****G**G.**A****U**.**A**U**G****G****C****C****U****G****C****A****A**.**G****U**UUC**U****A****C****C****G****A****A****G****U****A****C****C**..GU**A****A****A****U****A****C****U****U****U**.**G**.AC**U****A****U****G****A****G****U****G**AGGAC.GAAUAUAUUU | |
|  |  | NC\_003997.3/1497571-1497673  | AAAUAAAUAGUUAGC.UA.**C****A****C****U****C****A****U****A****U**AA**U****C****G****C****G****G****G**G.**A****U**.**A**U**G****G****C****C****U****G****C****A****A**.**G****U**UUC**U****A****C****C****G****A****A****G****U****A****C****C**..GU**A****A****A****U****A****C****U****U****U**.**G**.AC**U****A****U****G****A****G****U****G**AGGAC.GAAUAUAUUU | |
|  |  | NC\_005945.1/1497645-1497747  | AAAUAAAUAGUUAGC.UA.**C****A****C****U****C****A****U****A****U**AA**U****C****G****C****G****G****G**G.**A****U**.**A**U**G****G****C****C****U****G****C****A****A**.**G****U**UUC**U****A****C****C****G****A****A****G****U****A****C****C**..GU**A****A****A****U****A****C****U****U****U**.**G**.AC**U****A****U****G****A****G****U****G**AGGAC.GAAUAUAUUU | |
|  |  | NC\_005957.1/1521880-1521982  | AAAUAAAUAGUUAGC.UA.**C****A****C****U****C****A****U****A****U**AA**U****C****G****C****G****G****G**G.**A****U**.**A**U**G****G****C****C****U****G****C****A****A**.**G****U**UUC**U****A****C****C****G****A****A****G****U****A****C****C**..GU**A****A****A****U****A****C****U****U****U**.**G**.AC**U****A****U****G****A****G****U****G**AGGAC.GAAUAUAUUU | |
|  |  | NC\_006274.1/1532482-1532584  | AAAUAAAUAGUUAGC.UA.**C****A****C****U****C****A****U****A****U**AA**U****C****G****C****G****G****G**G.**A****U**.**A**U**G****G****C****C****U****G****C****A****A**.**G****U**UUC**U****A****C****C****G****A****A****G****U****A****C****C**..GU**A****A****A****U****A****C****U****U****U**.**G**.AC**U****A****U****G****A****G****U****G**AGGAC.GAAUAUAUUU | |
|  |  | NC\_007530.2/1497694-1497796  | AAAUAAAUAGUUAGC.UA.**C****A****C****U****C****A****U****A****U**AA**U****C****G****C****G****G****G**G.**A****U**.**A**U**G****G****C****C****U****G****C****A****A**.**G****U**UUC**U****A****C****C****G****A****A****G****U****A****C****C**..GU**A****A****A****U****A****C****U****U****U**.**G**.AC**U****A****U****G****A****G****U****G**AGGAC.GAAUAUAUUU | |
|  |  | NZ\_AAAC02000001.1/1985987-1986089  | AAAUAAAUAGUUAGC.UA.**C****A****C****U****C****A****U****A****U**AA**U****C****G****C****G****G****G**G.**A****U**.**A**U**G****G****C****C****U****G****C****A****A**.**G****U**UUC**U****A****C****C****G****A****A****G****U****A****C****C**..GU**A****A****A****U****A****C****U****U****U**.**G**.AC**U****A****U****G****A****G****U****G**AGGAC.GAAUAUAUUU | |
|  |  | NZ\_AAEK01000008.1/96466-96364  | AAAUAAAUAGUUAGC.UA.**C****A****C****U****C****A****U****A****U**AA**U****C****G****C****G****G****G**G.**A****U**.**A**U**G****G****C****C****U****G****C****A****A**.**G****U**UUC**U****A****C****C****G****A****A****G****U****A****C****C**..GU**A****A****A****U****A****C****U****U****U**.**G**.AC**U****A****U****G****A****G****U****G**AGGAC.GAAUAUAUUU | |
|  |  | NZ\_AAEN01000011.1/67143-67245  | AAAUAAAUAGUUAGC.UA.**C****A****C****U****C****A****U****A****U**AA**U****C****G****C****G****G****G**G.**A****U**.**A**U**G****G****C****C****U****G****C****A****A**.**G****U**UUC**U****A****C****C****G****A****A****G****U****A****C****C**..GU**A****A****A****U****A****C****U****U****U**.**G**.AC**U****A****U****G****A****G****U****G**AGGAC.GAAUAUAUUU | |
|  |  | NZ\_AAEO01000025.1/66855-66957  | AAAUAAAUAGUUAGC.UA.**C****A****C****U****C****A****U****A****U**AA**U****C****G****C****G****G****G**G.**A****U**.**A**U**G****G****C****C****U****G****C****A****A**.**G****U**UUC**U****A****C****C****G****A****A****G****U****A****C****C**..GU**A****A****A****U****A****C****U****U****U**.**G**.AC**U****A****U****G****A****G****U****G**AGGAC.GAAUAUAUUU | |
|  |  | NZ\_AAEP01000035.1/69365-69467  | AAAUAAAUAGUUAGC.UA.**C****A****C****U****C****A****U****A****U**AA**U****C****G****C****G****G****G**G.**A****U**.**A**U**G****G****C****C****U****G****C****A****A**.**G****U**UUC**U****A****C****C****G****A****A****G****U****A****C****C**..GU**A****A****A****U****A****C****U****U****U**.**G**.AC**U****A****U****G****A****G****U****G**AGGAC.GAAUAUAUUU | |
|  |  | NZ\_AAEQ01000029.1/70848-70950  | AAAUAAAUAGUUAGC.UA.**C****A****C****U****C****A****U****A****U**AA**U****C****G****C****G****G****G**G.**A****U**.**A**U**G****G****C****C****U****G****C****A****A**.**G****U**UUC**U****A****C****C****G****A****A****G****U****A****C****C**..GU**A****A****A****U****A****C****U****U****U**.**G**.AC**U****A****U****G****A****G****U****G**AGGAC.GAAUAUAUUU | |
|  |  | NZ\_AAER01000023.1/58182-58284  | AAAUAAAUAGUUAGC.UA.**C****A****C****U****C****A****U****A****U**AA**U****C****G****C****G****G****G**G.**A****U**.**A**U**G****G****C****C****U****G****C****A****A**.**G****U**UUC**U****A****C****C****G****A****A****G****U****A****C****C**..GU**A****A****A****U****A****C****U****U****U**.**G**.AC**U****A****U****G****A****G****U****G**AGGAC.GAAUAUAUUU | |
|  |  | NZ\_AAES01000034.1/69121-69223  | AAAUAAAUAGUUAGC.UA.**C****A****C****U****C****A****U****A****U**AA**U****C****G****C****G****G****G**G.**A****U**.**A**U**G****G****C****C****U****G****C****A****A**.**G****U**UUC**U****A****C****C****G****A****A****G****U****A****C****C**..GU**A****A****A****U****A****C****U****U****U**.**G**.AC**U****A****U****G****A****G****U****G**AGGAC.GAAUAUAUUU | |
|  |  | NC\_004722.1/1515239-1515341  | AAAUAAAUAGUUAGC.UA.**C****A****C****U****C****A****U****A****U**AA**U****C****G****C****G****G****G**G.**A****U**.**A**U**G****G****C****C****U****G****C****A****A**.**G****U**UUC**U****A****C****C****G****A****A****G****U****A****C****C**..GU**A****A****A****U****A****C****U****U****U**.**G**.AC**U****A****U****G****A****G****U****G**AGGAC.GAAUAUAAUU | |
|  |  | NC\_000964.2/697711-697813  | CAUGAAAUCAAAACA.CG.**A****C****C****U****C****A****U****A****U**AA**U****C****U****U****G****G****G**A.**A****U**.**A**U**G****G****C****C****C****A****U****A****A**.**G****U**UUC**U****A****C****C****C****G****G****C****A****A****C****C**..GU**A****A****A****U****U****G****C****C****G**.**G**.AC**U****A****U****G****C****A****G****G**AAAGU.GAUCGAUAAA | |
|  |  | NC\_002976.3/54816-54919  | CAUAAAAUAAUUUAU.AU.**G****A****C****U****C****A****U****A****U**AA**U****C****U****A****G****A****G**A.**A****U**.**A**U**G****G****C****U****U****U****A****G****A**A**G****U**UUC**U****A****C****C****G****U****G****U****C****G****C****C**..AU**A****A****A****C****G****A****C****A****C**.**G**.AC**U****A****U****G****A****G****U****A**ACAAU.CCAAUACAUU | |
|  |  | NC\_004461.1/2433047-2432944  | CAUAAAAUAAUUUAU.AU.**G****A****C****U****C****A****U****A****U**AA**U****C****U****A****G****A****G**A.**A****U**.**A**U**G****G****C****U****U****U****A****G****A**A**G****U**UUC**U****A****C****C****G****U****G****U****C****G****C****C**..AU**A****A****A****C****G****A****C****A****C**.**G**.AC**U****A****U****G****A****G****U****A**ACAAU.CCAAUACAUU | |
|  |  | NC\_003030.1/2824953-2824855  | AAUCGUUAAUAUAGU.UU.**A****A****C****U****C****A****U****A****U**AU-**U****U****C****C****U****G**A.**A****U**.**A**U**G****G****C****A****G****G****A****U**-.**G****U**UUC**U****A****C****A****A****G****G****A****A**-**C****C**..UU**A****A****A**-**U****U****U****C****U**.**U**.AC**U****A****U****G****A****G****U****G**AUUUG.UUUGUAUGCA | |
|  |  | NC\_003909.8/296465-296567  | GAAAAGUGAAUAUUA.UG.**C****C****G****U****C****G****U****A****U**AA**U****A****U****C****G****G****G**G.**A****U**.**A**U**G****G****C****C****C****G****A****A****A**.**G****U**UUC**U****A****C****C****U****A****G****C****U****A****C****C**..GU**A****A****A****U****G****G****C****U****U**.**G**.AC**U****A****C****G****A****G****G****C**GUUUU.UAUAAAGGUG | |
|  |  | NC\_003997.3/262601-262703  | GAAAAGUGAAUAUUA.UG.**C****C****G****U****C****G****U****A****U**AA**U****A****U****C****G****G****G**G.**A****U**.**A**U**G****G****C****C****C****G****A****A****A**.**G****U**UUC**U****A****C****C****U****A****G****C****U****A****C****C**..GU**A****A****A****U****G****G****C****U****U**.**G**.AC**U****A****C****G****A****G****G****C**GUUUU.UAUAAAGGUG | |
|  |  | NC\_004722.1/261558-261660  | GAAAAGUGAAUAUUA.UG.**C****C****G****U****C****G****U****A****U**AA**U****A****U****C****G****G****G**G.**A****U**.**A**U**G****G****C****C****C****G****A****A****A**.**G****U**UUC**U****A****C****C****U****A****G****C****U****A****C****C**..GU**A****A****A****U****G****G****C****U****U**.**G**.AC**U****A****C****G****A****G****G****C**GUUUU.UAUAAAGGUG | |
|  |  | NC\_005945.1/262614-262716  | GAAAAGUGAAUAUUA.UG.**C****C****G****U****C****G****U****A****U**AA**U****A****U****C****G****G****G**G.**A****U**.**A**U**G****G****C****C****C****G****A****A****A**.**G****U**UUC**U****A****C****C****U****A****G****C****U****A****C****C**..GU**A****A****A****U****G****G****C****U****U**.**G**.AC**U****A****C****G****A****G****G****C**GUUUU.UAUAAAGGUG | |
|  |  | NC\_005957.1/268537-268639  | GAAAAGUGAAUAUUA.UG.**C****C****G****U****C****G****U****A****U**AA**U****A****U****C****G****G****G**G.**A****U**.**A**U**G****G****C****C****C****G****A****A****A**.**G****U**UUC**U****A****C****C****U****A****G****C****U****A****C****C**..GU**A****A****A****U****G****G****C****U****U**.**G**.AC**U****A****C****G****A****G****G****C**GUUUU.UAUAAAGGUG | |
|  |  | NC\_006274.1/267835-267937  | GAAAAGUGAAUAUUA.UG.**C****C****G****U****C****G****U****A****U**AA**U****A****U****C****G****G****G**G.**A****U**.**A**U**G****G****C****C****C****G****A****A****A**.**G****U**UUC**U****A****C****C****U****A****G****C****U****A****C****C**..GU**A****A****A****U****G****G****C****U****U**.**G**.AC**U****A****C****G****A****G****G****C**GUUUU.UAUAAAGGUG | |
|  |  | NC\_007530.2/262601-262703  | GAAAAGUGAAUAUUA.UG.**C****C****G****U****C****G****U****A****U**AA**U****A****U****C****G****G****G**G.**A****U**.**A**U**G****G****C****C****C****G****A****A****A**.**G****U**UUC**U****A****C****C****U****A****G****C****U****A****C****C**..GU**A****A****A****U****G****G****C****U****U**.**G**.AC**U****A****C****G****A****G****G****C**GUUUU.UAUAAAGGUG | |
|  |  | NZ\_AAAC02000001.1/796036-796138  | GAAAAGUGAAUAUUA.UG.**C****C****G****U****C****G****U****A****U**AA**U****A****U****C****G****G****G**G.**A****U**.**A**U**G****G****C****C****C****G****A****A****A**.**G****U**UUC**U****A****C****C****U****A****G****C****U****A****C****C**..GU**A****A****A****U****G****G****C****U****U**.**G**.AC**U****A****C****G****A****G****G****C**GUUUU.UAUAAAGGUG | |
|  |  | NZ\_AAEK01000017.1/88397-88499  | GAAAAGUGAAUAUUA.UG.**C****C****G****U****C****G****U****A****U**AA**U****A****U****C****G****G****G**G.**A****U**.**A**U**G****G****C****C****C****G****A****A****A**.**G****U**UUC**U****A****C****C****U****A****G****C****U****A****C****C**..GU**A****A****A****U****G****G****C****U****U**.**G**.AC**U****A****C****G****A****G****G****C**GUUUU.UAUAAAGGUG | |
|  |  | NZ\_AAEN01000023.1/16980-17082  | GAAAAGUGAAUAUUA.UG.**C****C****G****U****C****G****U****A****U**AA**U****A****U****C****G****G****G**G.**A****U**.**A**U**G****G****C****C****C****G****A****A****A**.**G****U**UUC**U****A****C****C****U****A****G****C****U****A****C****C**..GU**A****A****A****U****G****G****C****U****U**.**G**.AC**U****A****C****G****A****G****G****C**GUUUU.UAUAAAGGUG | |
|  |  | NZ\_AAEO01000030.1/17090-17192  | GAAAAGUGAAUAUUA.UG.**C****C****G****U****C****G****U****A****U**AA**U****A****U****C****G****G****G**G.**A****U**.**A**U**G****G****C****C****C****G****A****A****A**.**G****U**UUC**U****A****C****C****U****A****G****C****U****A****C****C**..GU**A****A****A****U****G****G****C****U****U**.**G**.AC**U****A****C****G****A****G****G****C**GUUUU.UAUAAAGGUG | |
|  |  | NZ\_AAEP01000043.1/12962-13064  | GAAAAGUGAAUAUUA.UG.**C****C****G****U****C****G****U****A****U**AA**U****A****U****C****G****G****G**G.**A****U**.**A**U**G****G****C****C****C****G****A****A****A**.**G****U**UUC**U****A****C****C****U****A****G****C****U****A****C****C**..GU**A****A****A****U****G****G****C****U****U**.**G**.AC**U****A****C****G****A****G****G****C**GUUUU.UAUAAAGGUG | |
|  |  | NZ\_AAEQ01000040.1/4962-4860  | GAAAAGUGAAUAUUA.UG.**C****C****G****U****C****G****U****A****U**AA**U****A****U****C****G****G****G**G.**A****U**.**A**U**G****G****C****C****C****G****A****A****A**.**G****U**UUC**U****A****C****C****U****A****G****C****U****A****C****C**..GU**A****A****A****U****G****G****C****U****U**.**G**.AC**U****A****C****G****A****G****G****C**GUUUU.UAUAAAGGUG | |
|  |  | NZ\_AAER01000030.1/4374-4272  | GAAAAGUGAAUAUUA.UG.**C****C****G****U****C****G****U****A****U**AA**U****A****U****C****G****G****G**G.**A****U**.**A**U**G****G****C****C****C****G****A****A****A**.**G****U**UUC**U****A****C****C****U****A****G****C****U****A****C****C**..GU**A****A****A****U****G****G****C****U****U**.**G**.AC**U****A****C****G****A****G****G****C**GUUUU.UAUAAAGGUG | |
|  |  | NZ\_AAES01000040.1/14760-14862  | GAAAAGUGAAUAUUA.UG.**C****C****G****U****C****G****U****A****U**AA**U****A****U****C****G****G****G**G.**A****U**.**A**U**G****G****C****C****C****G****A****A****A**.**G****U**UUC**U****A****C****C****U****A****G****C****U****A****C****C**..GU**A****A****A****U****G****G****C****U****U**.**G**.AC**U****A****C****G****A****G****G****C**GUUUU.UAUAAAGGUG | |
|  |  | NC\_006270.2/693198-693300  | AGUAAUUUAAAAAAG.AC.**U****U****G****U****C****G****U****A****U**AA**U****C****A****U****G****G****G**G.**A****U**.**A**U**G****G****C****C****C****A****U****A****A**.**G****U**UUC**U****A****C****C****A****A****G****C****U****A****C****C**..GU**A****A****A****U****A****G****C****U****U**.**G**.AC**U****A****C****G****C****U****U****G**UAUAC.AAUAUUUUAU | |
|  |  | NC\_006322.1/692981-693083  | AGUAAUUUAAAAAAG.AC.**U****U****G****U****C****G****U****A****U**AA**U****C****A****U****G****G****G**G.**A****U**.**A**U**G****G****C****C****C****A****U****A****A**.**G****U**UUC**U****A****C****C****A****A****G****C****U****A****C****C**..GU**A****A****A****U****A****G****C****U****U**.**G**.AC**U****A****C****G****C****U****U****G**UAUAC.AAUAUUUUAU | |
|  |  | NC\_004722.1/259601-259703  | AGAUAAUAUAAAACG.AU.**C****C****U****U****C****A****U****A****U**AU**C****C****U****C****A****A****U**G.**A****U**.**A**U**G****G****U****U****U****G****A****G****A**.**G****U**CUC**U****A****C****C****G****G****G****U****U****A****C****C**..GU**A****A****A****C****A****A****C****C****U**.**G**.AC**U****A****U****G****A****A****G****G**CAGUG.UGUCUUAUAU | |
|  |  | NC\_000964.2/2319410-2319310  | UUACAAUAUAAUAGG.AA.**C****A****C****U****C****A****U****A****U**AA**U****C****G****C****G****U****G**G.**A****U**.**A**U**G****G****C****A****C****G****C****A****A**.**G****U**UUC**U****A****C****C****G****G****G****C****A**-**C****C**..GU**A****A****A**-**U****G****U****C****C**.**G**.AC**U****A****U****G****G****G****U****G**AGCAA.UGGAACCGCA | |
|  |  | NC\_003030.1/2905050-2904948  | GAAAAGUAAUAACAU.AU.**U****A****C****C****C****G****U****A****U**AU**G****C****U****U****A****G****A**A.**A****U**.**A**U**G****G****U****C****U****A****A****G****C**.**G****U**CUC**U****A****C****C****G****G****A****C****U****G****C****C**..GU**A****A****A****U****U****G****U****C****U**.**G**.AC**U****A****U****G****G****G****U****G**UUUAU.AAGUAUUUUA | |
|  |  | NC\_003210.1/611119-611017  | AAUCCGCUACAAUAA.UA.**U****A****G****U****C****G****U****A****U**AA**G****U****U****C****G****G****U**A.**A****U**.**A**U**G****G****A****C****C****G****U****U****C**.**G****U**UUC**U****A****C****C****A****G****G****C****A****A****C****C**..GU**A****A****A****A****U****G****C****C****A**.**G**.GC**U****A****C****G****A****G****C****U**AUUGU.AAAAUUUAAU | |
|  |  | NZ\_AADQ01000001.1/2431-2533  | AAUCCGCUACAAUAA.UA.**U****A****G****U****C****G****U****A****U**AA**G****U****U****C****G****G****U**A.**A****U**.**A**U**G****G****A****C****C****G****U****U****C**.**G****U**UUC**U****A****C****C****A****G****G****C****A****A****C****C**..GU**A****A****A****A****U****G****C****C****A**.**G**.GC**U****A****C****G****A****G****C****U**AUUGU.AAAAUUUAAU | |
|  |  | NC\_003212.1/610156-610054  | AAUCGUCUACAAUAA.UA.**A****A****G****U****C****G****U****A****U**AA**G****U****U****C****G****G****U**A.**A****U**.**A**U**G****G****A****C****C****G****U****U****C**.**G****U**UUC**U****A****C****C****A****G****G****C****A****A****C****C**..GU**A****A****A****A****U****G****C****C****A**.**G**.GC**U****A****C****G****A****G****C****U**AUUGU.AAAAUUUAAU | |
|  |  | NC\_003366.1/422820-422922  | UAUGUACUUAUAUAA.GU.**A****U****A****U****C****G****U****A****U**AU**G****C****U****C****G****A****C**G.**A****U**.**A**U**G****G****G****U****U****G****A****G****U**.**G****U**UUC**U****A****C****U****A****G****G****A****G****G****C****C**..GU**A****A****A****C****A****U****C****C****U**.**A**.AC**U****A****C****G****A****A****U****A**UAUAG.GUGAUUUCUA | |
|  |  | NC\_002973.5/617846-617744  | AAUCCGCUACAAUAA.UA.**A****A****G****U****C****G****U****A****U**AA**G****U****U****C****G****G****U**A.**A****U**.**A**U**G****G****A****C****C****G****U****U****C**.**G****U**UUC**U****A****C****C****A****G****G****C****A****A****C****C**..GU**A****A****A****A****U****G****C****C****A**.**G**.GC**U****A****C****G****A****G****C****U**AUUGU.AAAAUUUAAU | |
|  |  | NZ\_AADR01000111.1/1598-1496  | AAUCCGCUACAAUAA.UA.**A****A****G****U****C****G****U****A****U**AA**G****U****U****C****G****G****U**A.**A****U**.**A**U**G****G****A****C****C****G****U****U****C**.**G****U**UUC**U****A****C****C****A****G****G****C****A****A****C****C**..GU**A****A****A****A****U****G****C****C****A**.**G**.GC**U****A****C****G****A****G****C****U**AUUGU.AAAAUUUAAU | |
|  |  | NC\_003909.8/294509-294611  | AGAUAAUAUAAAACG.AU.**C****C****U****U****C****A****U****A****U**AU**C****C****U****C****A****A****A**G.**A****U**.**A**U**G****G****U****U****U****G****A****G****A**.**G****U**CUC**U****A****C****C****G****G****G****U****U****A****C****C**..GU**A****A****A****C****A****A****C****C****U**.**G**.AC**U****A****U****G****A****A****G****G**CAGUG.UGUCUUAUAU | |
|  |  | NC\_005957.1/266577-266679  | AGAUAAUAUAAAACG.AU.**C****C****U****U****C****A****U****A****U**AU**C****C****U****C****A****A****A**G.**A****U**.**A**U**G****G****U****U****U****G****A****G****A**.**G****U**CUC**U****A****C****C****G****G****G****U****U****A****C****C**..GU**A****A****A****C****A****A****C****C****U**.**G**.AC**U****A****U****G****A****A****G****G**CAGUG.UGUCUUAUAU | |
|  |  | NC\_006274.1/265875-265977  | AGAUAAUAUAAAACG.AU.**C****C****U****U****C****A****U****A****U**AU**C****C****U****C****A****A****A**G.**A****U**.**A**U**G****G****U****U****U****G****A****G****A**.**G****U**CUC**U****A****C****C****G****G****G****U****U****A****C****C**..GU**A****A****A****C****A****A****C****C****U**.**G**.AC**U****A****U****G****A****A****G****G**CAGUG.UGUCUUAUAU | |
|  |  | NC\_003366.1/2618421-2618323  | AAAACGGAAUAUAAA.CA.**A****A****C****U****C****G****U****A****U**AA-**G****C****U****U****U****G**A.**A****U**.**A**A**G****G****C****A****A****G****G****C**-.**G****U**UUC**U****A****C****C****G****G****A****A****A**-**C****C**..UU**A****A****A**-**U****U****U****C****C**.**G**.UC**U****A****U****G****A****G****U****G**AAUUU.GAUAUACUAU | |
|  |  | NC\_002973.5/1940027-1939926  | AUAACUUAAAACCGA.AA.**U****A****C****U****U****A****U****A****U**AA**U****A****G****U****U****G****C**G.**A****U**.-U**G****G****G****C****G****A****C****G****A**.**G****U**UUC**U****A****C****C****U****G****G****U****U****A****C****C**..GU**A****A****A****U****A****A****C****C****G**.**G**.AC**U****A****U****G****A****G****U****A**GUUUG.UAUAAAGAAG | |
|  |  | NC\_003212.1/2013345-2013244  | AUAACUUAAAACCGA.AA.**U****A****C****U****U****A****U****A****U**AA**U****A****G****U****U****G****C**G.**A****U**.-U**G****G****G****C****G****A****C****G****A**.**G****U**UUC**U****A****C****C****U****G****G****U****U****A****C****C**..GU**A****A****A****U****A****A****C****C****G**.**G**.AC**U****A****U****G****A****G****U****A**GUUUG.UAUAAAGAAG | |
|  |  | NZ\_AADQ01000095.1/1690-1791  | AUAACUUAAAACCGA.AA.**U****A****C****U****U****A****U****A****U**AA**U****A****G****U****U****G****C**G.**A****U**.-U**G****G****G****C****G****A****C****G****A**.**G****U**UUC**U****A****C****C****U****G****G****U****U****A****C****C**..GU**A****A****A****U****A****A****C****C****G**.**G**.AC**U****A****U****G****A****G****U****A**GUUUG.UAUAAAGAAG | |
|  |  | NZ\_AADR01000082.1/3415-3516  | AUAACUUAAAACCGA.AA.**U****A****C****U****U****A****U****A****U**AA**U****A****G****U****U****G****C**G.**A****U**.-U**G****G****G****C****G****A****C****G****A**.**G****U**UUC**U****A****C****C****U****G****G****U****U****A****C****C**..GU**A****A****A****U****A****A****C****C****G**.**G**.AC**U****A****U****G****A****G****U****A**GUUUG.UAUAAAGAAG | |
|  |  | NC\_003366.1/2871201-2871101  | AUAAAAAAAUAAAUU.UU.**G****C****U****U****C****G****U****A****U**AA**C****U****C****U****A****A****U**G.**A****U**.**A**U**G****G****A****U****U****A****G****A****G**.**G****U**CUC**U****A****C****C****A****A****G****A****A**-**C****C**..GA**G****A****A**-**U****U****C****U****U**.**G**.AU**U****A****C****G****A****A****G****A**AAGCU.UAUUUGCUUU | |
|  |  | NC\_002745.2/430797-430900  | GUUAAAUAAUUUACA.UA.**A****A****C****U****C****A****U****A****U**AA**U****C****U****A****A****A****G**A.**A****U**.**A**U**G****G****C****U****U****U****A****G****A**A**G****U**UUC**U****A****C****C****A****U****G****U****U****G****C****C**..UU**G****A****A****C****G****A****C****A****U**.**G**.AC**U****A****U****G****A****G****U****A**ACAAC.ACAAUACUAG | |
|  |  | NC\_002758.2/430754-430857  | GUUAAAUAAUUUACA.UA.**A****A****C****U****C****A****U****A****U**AA**U****C****U****A****A****A****G**A.**A****U**.**A**U**G****G****C****U****U****U****A****G****A**A**G****U**UUC**U****A****C****C****A****U****G****U****U****G****C****C**..UU**G****A****A****C****G****A****C****A****U**.**G**.AC**U****A****U****G****A****G****U****A**ACAAC.ACAAUACUAG | |
|  |  | NC\_002951.2/460059-460162  | GUUAAAUAAUUUACA.UA.**A****A****C****U****C****A****U****A****U**AA**U****C****U****A****A****A****G**A.**A****U**.**A**U**G****G****C****U****U****U****A****G****A**A**G****U**UUC**U****A****C****C****A****U****G****U****U****G****C****C**..UU**G****A****A****C****G****A****C****A****U**.**G**.AC**U****A****U****G****A****G****U****A**ACAAC.ACAAUACUAG | |
|  |  | NC\_002952.2/441050-441153  | GUUAAAUAAUUUACA.UA.**A****A****C****U****C****A****U****A****U**AA**U****C****U****A****A****A****G**A.**A****U**.**A**U**G****G****C****U****U****U****A****G****A**A**G****U**UUC**U****A****C****C****A****U****G****U****U****G****C****C**..UU**G****A****A****C****G****A****C****A****U**.**G**.AC**U****A****U****G****A****G****U****A**ACAAC.ACAAUACUAG | |
|  |  | NC\_002953.3/409191-409294  | GUUAAAUAAUUUACA.UA.**A****A****C****U****C****A****U****A****U**AA**U****C****U****A****A****A****G**A.**A****U**.**A**U**G****G****C****U****U****U****A****G****A**A**G****U**UUC**U****A****C****C****A****U****G****U****U****G****C****C**..UU**G****A****A****C****G****A****C****A****U**.**G**.AC**U****A****U****G****A****G****U****A**ACAAC.ACAAUACUAG | |
|  |  | NC\_003923.1/410546-410649  | GUUAAAUAAUUUACA.UA.**A****A****C****U****C****A****U****A****U**AA**U****C****U****A****A****A****G**A.**A****U**.**A**U**G****G****C****U****U****U****A****G****A**A**G****U**UUC**U****A****C****C****A****U****G****U****U****G****C****C**..UU**G****A****A****C****G****A****C****A****U**.**G**.AC**U****A****U****G****A****G****U****A**ACAAC.ACAAUACUAG | |
|  |  | NC\_006582.1/1115665-1115767  | AAGUUAAAAACGAAA.AC.**A****C****C****U****C****A****U****A****U**AU**A****C****U****C****G****G****G**A.**A****U**.**A**U**G****G****C****U****C****G****A****A****C**.**G****U**UUC**U****A****C****C****C****G****G****C****A****A****C****C**..GU**A****A****A****U****U****G****C****C****G**.**G**.AC**U****A****U****G****A****G****G****G**GAAGU.CAUUACGCGC | |
|  |  | NC\_006510.1/274257-274359  | AUGAAUAUUGUUGAA.UU.**C****C****G****U****C****G****U****A****U**AA**U****C****C****C****G****G****G**A.**A****U**.**A**U**G****G****C****U****C****G****G****G****A**.**G****U**UUC**U****A****C****C****A****A****G****C****U****A****C****C**..GU**A****A****A****U****A****G****C****U****U**.**G**.AC**U****A****C****G****A****G****G****G**AUGCG.GGAUCGGAGA | |
|  |  | NC\_003210.1/1958922-1958821  | AUAACUUAAAACCGA.AA.**U****A****C****U****U****G****U****A****U**AA**U****A****G****U****U****G****C**G.**A****U**.-U**G****G****G****C****G****A****C****G****A**.**G****U**UUC**U****A****C****C****U****G****G****U****U****A****C****C**..GU**A****A****A****U****A****A****C****C****G**.**G**.AC**U****A****U****G****A****G****U****A**GUUUG.UAUAAAGAAG | |
|  |  | NC\_006270.2/697054-697156  | CAUGACAUGAAAACA.CA.**U****C****C****U****C****A****U****A****U**AA**U****C****U****U****G****G****G**A.**A****U**.**A**U**G****G****C****C****C****A****U****A****A**.**G****U**CUC**U****A****C****C****C****G****A****U****G****A****C****C**..GU**A****A****A****U****C****A****U****C****G**.**G**.AC**U****A****U****G****C****A****G****G**AAAGU.GGACAAUAAA | |
|  |  | NC\_006322.1/696838-696940  | CAUGACAUGAAAACA.CA.**U****C****C****U****C****A****U****A****U**AA**U****C****U****U****G****G****G**A.**A****U**.**A**U**G****G****C****C****C****A****U****A****A**.**G****U**CUC**U****A****C****C****C****G****A****U****G****A****C****C**..GU**A****A****A****U****C****A****U****C****G**.**G**.AC**U****A****U****G****C****A****G****G**AAAGU.GGACAAUAAA | |
|  |  | NC\_006510.1/282580-282682  | AUAGUGUAUGAGAAG.AU.**C****C****C****U****C****A****U****A****U**AA**U****U****U****U****G****G****G**A.**A****U**.**A**U**G****G****C****C****C****A****A****A****A**.**G****U**UUC**U****A****C****C****C****A****A****U****C****A****C****C**..GU**A****A****A****U****G****A****U****U****G**.**G**.AC**U****A****U****G****A****G****G****G**AAAGG.AUCGGUUUUG | |
|  |  | NC\_003997.3/260641-260743  | AGAUAAUAUAAAACG.AU.**C****C****U****U****C****A****U****A****U**AU**C****C****U****C****A****A****A**G.**A****U**.**A**A**G****G****U****U****U****G****A****G****A**.**G****U**CUC**U****A****C****C****G****G****G****U****U****A****C****C**..GU**A****A****A****C****A****A****C****C****U**.**G**.AC**U****A****U****G****A****A****G****G**CAGUG.UGUCUUAUAU | |
|  |  | NC\_005945.1/260654-260756  | AGAUAAUAUAAAACG.AU.**C****C****U****U****C****A****U****A****U**AU**C****C****U****C****A****A****A**G.**A****U**.**A**A**G****G****U****U****U****G****A****G****A**.**G****U**CUC**U****A****C****C****G****G****G****U****U****A****C****C**..GU**A****A****A****C****A****A****C****C****U**.**G**.AC**U****A****U****G****A****A****G****G**CAGUG.UGUCUUAUAU | |
|  |  | NC\_007530.2/260641-260743  | AGAUAAUAUAAAACG.AU.**C****C****U****U****C****A****U****A****U**AU**C****C****U****C****A****A****A**G.**A****U**.**A**A**G****G****U****U****U****G****A****G****A**.**G****U**CUC**U****A****C****C****G****G****G****U****U****A****C****C**..GU**A****A****A****C****A****A****C****C****U**.**G**.AC**U****A****U****G****A****A****G****G**CAGUG.UGUCUUAUAU | |
|  |  | NZ\_AAEK01000017.1/86437-86539  | AGAUAAUAUAAAACG.AU.**C****C****U****U****C****A****U****A****U**AU**C****C****U****C****A****A****A**G.**A****U**.**A**A**G****G****U****U****U****G****A****G****A**.**G****U**CUC**U****A****C****C****G****G****G****U****U****A****C****C**..GU**A****A****A****C****A****A****C****C****U**.**G**.AC**U****A****U****G****A****A****G****G**CAGUG.UGUCUUAUAU | |
|  |  | NZ\_AAEN01000023.1/15020-15122  | AGAUAAUAUAAAACG.AU.**C****C****U****U****C****A****U****A****U**AU**C****C****U****C****A****A****A**G.**A****U**.**A**A**G****G****U****U****U****G****A****G****A**.**G****U**CUC**U****A****C****C****G****G****G****U****U****A****C****C**..GU**A****A****A****C****A****A****C****C****U**.**G**.AC**U****A****U****G****A****A****G****G**CAGUG.UGUCUUAUAU | |
|  |  | NZ\_AAEO01000030.1/15130-15232  | AGAUAAUAUAAAACG.AU.**C****C****U****U****C****A****U****A****U**AU**C****C****U****C****A****A****A**G.**A****U**.**A**A**G****G****U****U****U****G****A****G****A**.**G****U**CUC**U****A****C****C****G****G****G****U****U****A****C****C**..GU**A****A****A****C****A****A****C****C****U**.**G**.AC**U****A****U****G****A****A****G****G**CAGUG.UGUCUUAUAU | |
|  |  | NZ\_AAEP01000043.1/11002-11104  | AGAUAAUAUAAAACG.AU.**C****C****U****U****C****A****U****A****U**AU**C****C****U****C****A****A****A**G.**A****U**.**A**A**G****G****U****U****U****G****A****G****A**.**G****U**CUC**U****A****C****C****G****G****G****U****U****A****C****C**..GU**A****A****A****C****A****A****C****C****U**.**G**.AC**U****A****U****G****A****A****G****G**CAGUG.UGUCUUAUAU | |
|  |  | NZ\_AAEQ01000040.1/6922-6820  | AGAUAAUAUAAAACG.AU.**C****C****U****U****C****A****U****A****U**AU**C****C****U****C****A****A****A**G.**A****U**.**A**A**G****G****U****U****U****G****A****G****A**.**G****U**CUC**U****A****C****C****G****G****G****U****U****A****C****C**..GU**A****A****A****C****A****A****C****C****U**.**G**.AC**U****A****U****G****A****A****G****G**CAGUG.UGUCUUAUAU | |
|  |  | NZ\_AAER01000030.1/6334-6232  | AGAUAAUAUAAAACG.AU.**C****C****U****U****C****A****U****A****U**AU**C****C****U****C****A****A****A**G.**A****U**.**A**A**G****G****U****U****U****G****A****G****A**.**G****U**CUC**U****A****C****C****G****G****G****U****U****A****C****C**..GU**A****A****A****C****A****A****C****C****U**.**G**.AC**U****A****U****G****A****A****G****G**CAGUG.UGUCUUAUAU | |
|  |  | NZ\_AAES01000040.1/12800-12902  | AGAUAAUAUAAAACG.AU.**C****C****U****U****C****A****U****A****U**AU**C****C****U****C****A****A****A**G.**A****U**.**A**A**G****G****U****U****U****G****A****G****A**.**G****U**CUC**U****A****C****C****G****G****G****U****U****A****C****C**..GU**A****A****A****C****A****A****C****C****U**.**G**.AC**U****A****U****G****A****A****G****G**CAGUG.UGUCUUAUAU | |
|  |  | NC\_004193.1/760473-760575  | CAAUUUUUAUCCAAU.GC.**C****U****U****U****C****G****U****A****U**AU**C****C****U****C****G****A****U**A.**A****U**.**A**U**G****G****U****U****C****G****A****A****A**.**G****U**AUC**U****A****C****C****G****G****G****U****C****A****C****C**..GU**A****A****A****U****G****A****U****C****U**.**G**.AC**U****A****U****G****A****A****G****G**CAGAA.GCAGGUUCGG | |
|  |  | NC\_006270.2/4024210-4024312  | AAAUAAUAGAAGCCC.AC.**U****U****C****U****U****G****U****A****U**AU**A****A****U****C****A****G****U**A.**A****U**.**A**G**G****G****U****C****U****G****A****U****U**.**G****U**UUC**U****A****C****C****U****G****G****C****A****A****C****C**..GU**A****A****A****U****C****G****C****C****A**.**G**.AC**U****A****C****A****A****G****G****A**AGUUU.GAAUAGAUUU | |
|  |  | NC\_006322.1/4024324-4024426  | AAAUAAUAGAAGCCC.AC.**U****U****C****U****U****G****U****A****U**AU**A****A****U****C****A****G****U**A.**A****U**.**A**G**G****G****U****C****U****G****A****U****U**.**G****U**UUC**U****A****C****C****U****G****G****C****A****A****C****C**..GU**A****A****A****U****C****G****C****C****A**.**G**.AC**U****A****C****A****A****G****G****A**AGUUU.GAAUAGAUUU | |
|  |  | NC\_004193.1/786767-786868  | CCGACAAUUGAAAAU.GA.**A****C****C****U****C****A****U****A****U**AA**A****U****U****U****G****A****G**A.**A****U**.**A**U**G****G****C****U****C****A****G****A****A**.**G****U**UUC**U****A****C****C****C****A****G****C**-**A****C****C**..GU**A****A****A****U****G****G****C****U****G**.**G**.AC**U****A****U****G****A****G****G****G**AAGAU.GGAUCAUUUC | |
|  |  | NC\_002662.1/1159509-1159607  | UAGUCUAUAAUAGAA.CA.**A****U****C****U****U****A****U****U****U**AU-**A****C****C****U****A****G**G.**A****U**.**A**U**G****G****C****U****G****G****G****C**-.**G****U**UUC**U****A****C****C****U****C****G****U****A**-**C****C**..GU**A****A****A**-**U****G****C****G****A**.**G**.AC**A****A****U****A****A****G****G****A**AAUUC.GAUUUUUUAG | |
|  |  | NC\_000964.2/625993-625893  | AAUUAAAUAGCUAUU.AU.**C****A****C****U****U****G****U****A****U**AA**C****C****U****C****A****A****U**A.**A****U**.**A**U**G****G****U****U****U****G****A****G****G**.**G****U**GUC**U****A****C****C****A****G****G****A****A**-**C****C**..GU**A****A****A**-**A****U****C****C****U**.**G**.AU**U****A****C****A****A****A****A****U**UUGUU.UAUGACAUUU | |
|  |  | NC\_003909.8/382630-382528  | UUAAUACGGACGAUG.UU.**A****C****C****U****C****A****U****A****U**AU**A****C****U****C****G****A****U**A.**A****U**.**A**U**G****G****A****U****C****G****A****G****A**.**G****U**UUC**U****A****C****C****C****G****G****C****A****A****C****C**..UU**A****A****A****U****U****G****C****U****G**.**G**.AC**U****A****U****G****G****G****G****A**AAACU.AAUGAAUAUU | |
|  |  | NC\_003997.3/342356-342254  | UUAAUACGGACGAUG.UU.**A****C****C****U****C****A****U****A****U**AU**A****C****U****C****G****A****U**A.**A****U**.**A**U**G****G****A****U****C****G****A****G****A**.**G****U**UUC**U****A****C****C****C****G****G****C****A****A****C****C**..UU**A****A****A****U****U****G****C****U****G**.**G**.AC**U****A****U****G****G****G****G****A**AAACU.AAUGAAUAUU | |
|  |  | NC\_005945.1/342369-342267  | UUAAUACGGACGAUG.UU.**A****C****C****U****C****A****U****A****U**AU**A****C****U****C****G****A****U**A.**A****U**.**A**U**G****G****A****U****C****G****A****G****A**.**G****U**UUC**U****A****C****C****C****G****G****C****A****A****C****C**..UU**A****A****A****U****U****G****C****U****G**.**G**.AC**U****A****U****G****G****G****G****A**AAACU.AAUGAAUAUU | |
|  |  | NC\_005957.1/356354-356252  | UUAAUACGGACGAUG.UU.**A****C****C****U****C****A****U****A****U**AU**A****C****U****C****G****A****U**A.**A****U**.**A**U**G****G****A****U****C****G****A****G****A**.**G****U**UUC**U****A****C****C****C****G****G****C****A****A****C****C**..UU**A****A****A****U****U****G****C****U****G**.**G**.AC**U****A****U****G****G****G****G****A**AAACU.AAUGAAUAUU | |
|  |  | NC\_006274.1/357462-357360  | UUAAUACGGACGAUG.UU.**A****C****C****U****C****A****U****A****U**AU**A****C****U****C****G****A****U**A.**A****U**.**A**U**G****G****A****U****C****G****A****G****A**.**G****U**UUC**U****A****C****C****C****G****G****C****A****A****C****C**..UU**A****A****A****U****U****G****C****U****G**.**G**.AC**U****A****U****G****G****G****G****A**AAACU.AAUGAAUAUU | |
|  |  | NC\_007530.2/342356-342254  | UUAAUACGGACGAUG.UU.**A****C****C****U****C****A****U****A****U**AU**A****C****U****C****G****A****U**A.**A****U**.**A**U**G****G****A****U****C****G****A****G****A**.**G****U**UUC**U****A****C****C****C****G****G****C****A****A****C****C**..UU**A****A****A****U****U****G****C****U****G**.**G**.AC**U****A****U****G****G****G****G****A**AAACU.AAUGAAUAUU | |
|  |  | NZ\_AAAC02000001.1/859268-859166  | UUAAUACGGACGAUG.UU.**A****C****C****U****C****A****U****A****U**AU**A****C****U****C****G****A****U**A.**A****U**.**A**U**G****G****A****U****C****G****A****G****A**.**G****U**UUC**U****A****C****C****C****G****G****C****A****A****C****C**..UU**A****A****A****U****U****G****C****U****G**.**G**.AC**U****A****U****G****G****G****G****A**AAACU.AAUGAAUAUU | |
|  |  | NZ\_AAEK01000051.1/8150-8252  | UUAAUACGGACGAUG.UU.**A****C****C****U****C****A****U****A****U**AU**A****C****U****C****G****A****U**A.**A****U**.**A**U**G****G****A****U****C****G****A****G****A**.**G****U**UUC**U****A****C****C****C****G****G****C****A****A****C****C**..UU**A****A****A****U****U****G****C****U****G**.**G**.AC**U****A****U****G****G****G****G****A**AAACU.AAUGAAUAUU | |
|  |  | NZ\_AAEN01000023.1/83441-83339  | UUAAUACGGACGAUG.UU.**A****C****C****U****C****A****U****A****U**AU**A****C****U****C****G****A****U**A.**A****U**.**A**U**G****G****A****U****C****G****A****G****A**.**G****U**UUC**U****A****C****C****C****G****G****C****A****A****C****C**..UU**A****A****A****U****U****G****C****U****G**.**G**.AC**U****A****U****G****G****G****G****A**AAACU.AAUGAAUAUU | |
|  |  | NZ\_AAEO01000030.1/96886-96784  | UUAAUACGGACGAUG.UU.**A****C****C****U****C****A****U****A****U**AU**A****C****U****C****G****A****U**A.**A****U**.**A**U**G****G****A****U****C****G****A****G****A**.**G****U**UUC**U****A****C****C****C****G****G****C****A****A****C****C**..UU**A****A****A****U****U****G****C****U****G**.**G**.AC**U****A****U****G****G****G****G****A**AAACU.AAUGAAUAUU | |
|  |  | NZ\_AAEP01000046.1/48810-48708  | UUAAUACGGACGAUG.UU.**A****C****C****U****C****A****U****A****U**AU**A****C****U****C****G****A****U**A.**A****U**.**A**U**G****G****A****U****C****G****A****G****A**.**G****U**UUC**U****A****C****C****C****G****G****C****A****A****C****C**..UU**A****A****A****U****U****G****C****U****G**.**G**.AC**U****A****U****G****G****G****G****A**AAACU.AAUGAAUAUU | |
|  |  | NZ\_AAEQ01000034.1/49483-49381  | UUAAUACGGACGAUG.UU.**A****C****C****U****C****A****U****A****U**AU**A****C****U****C****G****A****U**A.**A****U**.**A**U**G****G****A****U****C****G****A****G****A**.**G****U**UUC**U****A****C****C****C****G****G****C****A****A****C****C**..UU**A****A****A****U****U****G****C****U****G**.**G**.AC**U****A****U****G****G****G****G****A**AAACU.AAUGAAUAUU | |
|  |  | NZ\_AAER01000042.1/191339-191441  | UUAAUACGGACGAUG.UU.**A****C****C****U****C****A****U****A****U**AU**A****C****U****C****G****A****U**A.**A****U**.**A**U**G****G****A****U****C****G****A****G****A**.**G****U**UUC**U****A****C****C****C****G****G****C****A****A****C****C**..UU**A****A****A****U****U****G****C****U****G**.**G**.AC**U****A****U****G****G****G****G****A**AAACU.AAUGAAUAUU | |
|  |  | NZ\_AAES01000043.1/48479-48377  | UUAAUACGGACGAUG.UU.**A****C****C****U****C****A****U****A****U**AU**A****C****U****C****G****A****U**A.**A****U**.**A**U**G****G****A****U****C****G****A****G****A**.**G****U**UUC**U****A****C****C****C****G****G****C****A****A****C****C**..UU**A****A****A****U****U****G****C****U****G**.**G**.AC**U****A****U****G****G****G****G****A**AAACU.AAUGAAUAUU | |
|  |  | NZ\_AAAC02000001.1/794076-794178  | AGAUAAUAUAAAACG.AU.**C****C****U****U****C****A****U****A****U**AU**C****C****U****C****A****A****A**G.**A****U**.**A**R**G****G****U****U****U****G****A****G****A**.**G****U**CUC**U****A****C****C****G****G****G****U****U****A****C****C**..GU**A****A****A****C****A****A****C****C****U**.**G**.AC**U****A****U****G****A****W****G****G**CAGUG.UGUCUUAUAU | |
|  |  | NC\_003030.1/1002176-1002275  | UAUAUAAAAAACUAA.AU.**U****U****C****U****C****G****U****A****U**AC-**A****C****C****G****G****U**A.**A****U**.**A**U**G****G****U****C****C****G****G****A****A**.**G****U**UUC**U****A****C****C****U****G****C****U****G**-**C****C**..AU**A****A****A**-**U****A****G****C****A**.**G**.AC**U****A****C****G****G****G****G****U**GUUAU.UGAUAAUAUA | |
|  |  | NC\_006582.1/1554717-1554819  | UAAACGAACAAAGCA.UC.**A****G****C****U****C****G****U****A****U**AA**U****A****G****C****G****G****U**A.**A****U**.**A**U**G****G****U****C****C****G****C****G****A**.**G****U**CUC**U****A****C****C****A****G****G****C****U****G****C****C**..GA**U****A****A****C****G****G****C****C****U**.**G**.AC**U****A****C****G****A****G****U****G**GUCUU.UUUCAGUUGU | |
|  |  | NC\_003909.8/336194-336296  | AAAGAAUAAUAUAUA.AG.**A****C****C****U****C****A****U****A****U**AA**U****C****G****C****G****G****G**G.**A****U**.**A**U**G****G****C****C****U****G****C****A****A**.**G****U**CUC**U****A****C****C****U****A****A****C****G****A****C****C**..GU**U****A****U****U****C****G****U****U****A**.**G**.AC**U****A****U****G****A****G****G****G**AAAGU.CACUCGGUAU | |
|  |  | NC\_003997.3/295331-295433  | AAAGAAUAAUAUAUA.AG.**A****C****C****U****C****A****U****A****U**AA**U****C****G****C****G****G****G**G.**A****U**.**A**U**G****G****C****C****U****G****C****A****A**.**G****U**CUC**U****A****C****C****U****A****A****C****G****A****C****C**..GU**U****A****U****U****C****G****U****U****A**.**G**.AC**U****A****U****G****A****G****G****G**AAAGU.CACUCGGUAU | |
|  |  | NC\_005945.1/295344-295446  | AAAGAAUAAUAUAUA.AG.**A****C****C****U****C****A****U****A****U**AA**U****C****G****C****G****G****G**G.**A****U**.**A**U**G****G****C****C****U****G****C****A****A**.**G****U**CUC**U****A****C****C****U****A****A****C****G****A****C****C**..GU**U****A****U****U****C****G****U****U****A**.**G**.AC**U****A****U****G****A****G****G****G**AAAGU.CACUCGGUAU | |
|  |  | NC\_005957.1/309524-309626  | AAAGAAUAAUAUAUA.AG.**A****C****C****U****C****A****U****A****U**AA**U****C****G****C****G****G****G**G.**A****U**.**A**U**G****G****C****C****U****G****C****A****A**.**G****U**CUC**U****A****C****C****U****A****A****C****G****A****C****C**..GU**U****A****U****U****C****G****U****U****A**.**G**.AC**U****A****U****G****A****G****G****G**AAAGU.CACUCGGUAU | |
|  |  | NC\_006274.1/309094-309196  | AAAGAAUAAUAUAUA.AG.**A****C****C****U****C****A****U****A****U**AA**U****C****G****C****G****G****G**G.**A****U**.**A**U**G****G****C****C****U****G****C****A****A**.**G****U**CUC**U****A****C****C****U****A****A****C****G****A****C****C**..GU**U****A****U****U****C****G****U****U****A**.**G**.AC**U****A****U****G****A****G****G****G**AAAGU.CACUCGGUAU | |
|  |  | NC\_007530.2/295331-295433  | AAAGAAUAAUAUAUA.AG.**A****C****C****U****C****A****U****A****U**AA**U****C****G****C****G****G****G**G.**A****U**.**A**U**G****G****C****C****U****G****C****A****A**.**G****U**CUC**U****A****C****C****U****A****A****C****G****A****C****C**..GU**U****A****U****U****C****G****U****U****A**.**G**.AC**U****A****U****G****A****G****G****G**AAAGU.CACUCGGUAU | |
|  |  | NZ\_AAAC02000001.1/812243-812345  | AAAGAAUAAUAUAUA.AG.**A****C****C****U****C****A****U****A****U**AA**U****C****G****C****G****G****G**G.**A****U**.**A**U**G****G****C****C****U****G****C****A****A**.**G****U**CUC**U****A****C****C****U****A****A****C****G****A****C****C**..GU**U****A****U****U****C****G****U****U****A**.**G**.AC**U****A****U****G****A****G****G****G**AAAGU.CACUCGGUAU | |
|  |  | NZ\_AAEK01000064.1/23153-23051  | AAAGAAUAAUAUAUA.AG.**A****C****C****U****C****A****U****A****U**AA**U****C****G****C****G****G****G**G.**A****U**.**A**U**G****G****C****C****U****G****C****A****A**.**G****U**CUC**U****A****C****C****U****A****A****C****G****A****C****C**..GU**U****A****U****U****C****G****U****U****A**.**G**.AC**U****A****U****G****A****G****G****G**AAAGU.CACUCGGUAU | |
|  |  | NZ\_AAEN01000023.1/36414-36516  | AAAGAAUAAUAUAUA.AG.**A****C****C****U****C****A****U****A****U**AA**U****C****G****C****G****G****G**G.**A****U**.**A**U**G****G****C****C****U****G****C****A****A**.**G****U**CUC**U****A****C****C****U****A****A****C****G****A****C****C**..GU**U****A****U****U****C****G****U****U****A**.**G**.AC**U****A****U****G****A****G****G****G**AAAGU.CACUCGGUAU | |
|  |  | NZ\_AAEO01000030.1/49824-49926  | AAAGAAUAAUAUAUA.AG.**A****C****C****U****C****A****U****A****U**AA**U****C****G****C****G****G****G**G.**A****U**.**A**U**G****G****C****C****U****G****C****A****A**.**G****U**CUC**U****A****C****C****U****A****A****C****G****A****C****C**..GU**U****A****U****U****C****G****U****U****A**.**G**.AC**U****A****U****G****A****G****G****G**AAAGU.CACUCGGUAU | |
|  |  | NZ\_AAEP01000046.1/1785-1887  | AAAGAAUAAUAUAUA.AG.**A****C****C****U****C****A****U****A****U**AA**U****C****G****C****G****G****G**G.**A****U**.**A**U**G****G****C****C****U****G****C****A****A**.**G****U**CUC**U****A****C****C****U****A****A****C****G****A****C****C**..GU**U****A****U****U****C****G****U****U****A**.**G**.AC**U****A****U****G****A****G****G****G**AAAGU.CACUCGGUAU | |
|  |  | NZ\_AAEQ01000034.1/2457-2559  | AAAGAAUAAUAUAUA.AG.**A****C****C****U****C****A****U****A****U**AA**U****C****G****C****G****G****G**G.**A****U**.**A**U**G****G****C****C****U****G****C****A****A**.**G****U**CUC**U****A****C****C****U****A****A****C****G****A****C****C**..GU**U****A****U****U****C****G****U****U****A**.**G**.AC**U****A****U****G****A****G****G****G**AAAGU.CACUCGGUAU | |
|  |  | NZ\_AAER01000042.1/238364-238262  | AAAGAAUAAUAUAUA.AG.**A****C****C****U****C****A****U****A****U**AA**U****C****G****C****G****G****G**G.**A****U**.**A**U**G****G****C****C****U****G****C****A****A**.**G****U**CUC**U****A****C****C****U****A****A****C****G****A****C****C**..GU**U****A****U****U****C****G****U****U****A**.**G**.AC**U****A****U****G****A****G****G****G**AAAGU.CACUCGGUAU | |
|  |  | NZ\_AAES01000043.1/1489-1591  | AAAGAAUAAUAUAUA.AG.**A****C****C****U****C****A****U****A****U**AA**U****C****G****C****G****G****G**G.**A****U**.**A**U**G****G****C****C****U****G****C****A****A**.**G****U**CUC**U****A****C****C****U****A****A****C****G****A****C****C**..GU**U****A****U****U****C****G****U****U****A**.**G**.AC**U****A****U****G****A****G****G****G**AAAGU.CACUCGGUAU | |
|  |  | NC\_004193.1/769686-769787  | UGAUGUAAUUGAAUA.GA.**A****A****U****G****C****G****U****A****U**AA**U****U****A****A****G****G****G**G.**A****U**.**A**U**G****G****C****C****C**-**A****C****A**.**G****U**UUC**U****A****C****C****A****G****A****C****C****A****C****C**..GU**A****A****A****U****G****G****U****U****U**.**G**.AC**U****A****C****G****C****A****G****U**AAUUA.UAUUUGUAUC | |
|  |  | NC\_004722.1/343847-343745  | UUAAUACGGACGAUG.UU.**A****C****C****U****C****A****U****A****U**AU**A****C****U****U****G****A****U**A.**A****U**.**A**U**G****G****A****U****C****G****A****G****A**.**G****U**UUC**U****A****C****C****C****G****G****C****A****A****C****C**..UU**A****A****A****U****U****G****C****U****G**.**G**.AC**U****A****U****G****G****G****G****A**AAACU.AAUGAAUAUU | |
|  |  | NZ\_AAAW03000042.1/15038-14936  | AAAAAAUUUAAAUAG.GG.**C****G****U****U****C****A****U****A****U**AA**U****C****G****C****G****G****A**G.**A****U**.**A**G**G****G****U****C****C****G****C****A****A**.**G****U**UUC**U****A****C****C****G****G****G****C****U****G****C****C**..GU**A****A****A****U****G****G****C****C****U**.**G**.AC**U****A****U****G****A****G****C****G**AAACU.GUGCCCAGGG | |
|  |  | NZ\_AADT03000005.1/34629-34731  | AAAAAUAAUUUAACC.CU.**G****C****U****U****C****G****U****A****U**AU**U****C****C****C****G****G****A**A.**A****U**.**G**C**G****G****U****C****C****G****G****G****A**.**G****U**UUC**U****A****C****C****A****G****G****C****A****A****C****C**..GU**A****A****A****U****U****G****C****C****C**.**G**.GC**U****A****C****G****A****A****G****G**UUAUU.CUUCGUCGUC | |
|  |  | NC\_002570.2/648442-648544  | ACAUGUAGAUAUCAU.CC.**C****U****U****U****C****G****U****A****U**AU**A****C****U****U****G****G****A**G.**A****U**.**A**A**G****G****U****C****C****A****G****G****A**.**G****U**UUC**U****A****C****C****A****G****A****U****C****A****C****C**..GU**A****A****A****U****G****A****U****C****U**.**G**.AC**U****A****U****G****A****A****G****G**UGGAA.UGGCUCGAUA | |
|  |  | NC\_004722.1/298774-298876  | AGAAACAAUAAUAUA.AG.**A****C****C****U****C****A****U****A****U**AA**U****C****G****C****G****G****G**G.**A****U**.**A**U**G****G****C****C****U****G****C****A****A**.**G****U**CUC**U****A****C****C****U****A****A****C****G****A****C****C**..GU**U****A****U****U****C****G****U****U****A**.**G**.AC**U****A****U****G****A****G****G****G**AAAGU.CACUCGGUAU | |
|  |  | NC\_006510.1/272473-272575  | UACGGAUAGACGAAA.GC.**C****C****U****U****C****A****U****A****U**AA**G****C****G****C****A****A****G**A.**A****U**.**A**U**G****G****C****U****U****G****C****G****C**.**G****U**CUC**U****A****C****C****G****G****G****C****C****G****C****C**..GU**A****A****A****C****G****G****C****C****C**.**G**.AC**U****A****U****G****A****A****G****G**CAGAA.GACGCUGCUA | |
|  |  | NZ\_AADW02000004.1/225764-225662  | GGAAGAUUGAAUAUA.AC.**A****C****C****U****C****G****U****A****U**AA**U****A****G****C****A****G****G**G.**A****U**.**A**U**G****G****C****U****U****G****C****A****A**.**G****U**UUC**U****A****C****C****C****G****A****C****G****A****C****C**..CU**A****A****A****U****C****G****U****U****G**.**G**.AC**U****A****U****G****G****G****G****U**AUAUG.GAUGUUCGUC | |
|  |  | NC\_006274.1/3685852-3685750  | CUUCGAAAAGAAUCA.CU.**G****C****C****U****C****A****U****A****U**AA**U****C****U****U****G****G****A**G.**A****U**.**A**A**G****G****U****C****C****A****U****A****A**.**G****U**UUC**U****A****C****C****U****G****G****C****A****A****C****C**..AU**G****A****A****U****U****G****C****U****A**.**G**.AC**U****A****U****G****A****G****G****G**AAAAA.GUGUGUAACA | |
|  |  | NZ\_AAGO01000011.1/2345-2247  | UUGAGCUGCUAUAAA.CA.**A****U****C****U****U****A****U****U****U**AU-**A****C****C****U****A****G**A.**A****U**.**A**U**G****G****C****U****G****G****G****C**-.**G****U**UUC**U****A****C****C****U****C****G****U****A**-**C****C**..GU**A****A****A**-**U****G****C****G****A**.**G**.AC**A****A****U****A****A****G****G****A**AAUUC.GAUUUUUCAA | |
|  |  | NZ\_AAAK03000113.1/5724-5822  | UAUUAGUUUUACAUA.CC.**U****A****C****U****U****A****U****A****U**AU-**C****G****U****C****A****U**A.**A****U**.**A**U**G****G****A****U****G****A****C****A**-.**G****U**UUC**U****A****G****C****C****A****G****U****A**-**C****C**..GU**A****A****A**-**U****G****C****U****G**.**G**.AC**U****A****U****A****A****G****U****A**AAAGA.UUGGCUAUUU | |
|  |  | NZ\_AADW02000005.1/186378-186480  | CUAAAAAACAAAAAA.UA.**A****U****G****C****C****G****U****A****U**AA**U****U****C****U****G****G****G**G.**A****U**.**A**U**G****G****C****C****C****G****G****A****A**.**G****U**CUC**U****A****C****A****G****G****A****A****C****A****C****C**..UU**A****A****A****G****G****U****U****C****C**.**U**.AC**U****A****C****G****G****C****G****U**GCACU.GAUUUCCGGU | |
|  |  | NC\_003909.8/3578068-3577966  | CUUCGAAAAGAAUCA.CU.**G****C****C****U****C****A****U****A****U**AA**U****C****U****U****G****G****A**G.**A****U**.**A**A**G****G****U****C****C****A****U****A****A**.**G****U**UUC**U****A****C****C****U****G****G****C****A****A****C****C**..AU**G****A****A****U****U****G****C****U****A**.**G**.AC**U****A****U****G****A****G****G****G**GAAAA.GUGUGUAACA | |
|  |  | NC\_003997.3/3605298-3605196  | CUUCGAAAAGAAUCA.CU.**G****C****C****U****C****A****U****A****U**AA**U****C****U****U****G****G****A**G.**A****U**.**A**A**G****G****U****C****C****A****U****A****A**.**G****U**UUC**U****A****C****C****U****G****G****C****A****A****C****C**..AU**G****A****A****U****U****G****C****U****A**.**G**.AC**U****A****U****G****A****G****G****G**GAAAA.GUGUGUAACA | |
|  |  | NC\_004722.1/3766254-3766152  | CUUCGAAAAGAAUCA.CU.**G****C****C****U****C****A****U****A****U**AA**U****C****U****U****G****G****A**G.**A****U**.**A**A**G****G****U****C****C****A****U****A****A**.**G****U**UUC**U****A****C****C****U****G****G****C****A****A****C****C**..AU**G****A****A****U****U****G****C****U****A**.**G**.AC**U****A****U****G****A****G****G****G**GAAAA.GUGUGUAACA | |
|  |  | NC\_005945.1/3605993-3605891  | CUUCGAAAAGAAUCA.CU.**G****C****C****U****C****A****U****A****U**AA**U****C****U****U****G****G****A**G.**A****U**.**A**A**G****G****U****C****C****A****U****A****A**.**G****U**UUC**U****A****C****C****U****G****G****C****A****A****C****C**..AU**G****A****A****U****U****G****C****U****A**.**G**.AC**U****A****U****G****A****G****G****G**GAAAA.GUGUGUAACA | |
|  |  | NC\_005957.1/3626969-3626867  | CUUCGAAAAGAAUCA.CU.**G****C****C****U****C****A****U****A****U**AA**U****C****U****U****G****G****A**G.**A****U**.**A**A**G****G****U****C****C****A****U****A****A**.**G****U**UUC**U****A****C****C****U****G****G****C****A****A****C****C**..AU**G****A****A****U****U****G****C****U****A**.**G**.AC**U****A****U****G****A****G****G****G**GAAAA.GUGUGUAACA | |
|  |  | NC\_007530.2/3605425-3605323  | CUUCGAAAAGAAUCA.CU.**G****C****C****U****C****A****U****A****U**AA**U****C****U****U****G****G****A**G.**A****U**.**A**A**G****G****U****C****C****A****U****A****A**.**G****U**UUC**U****A****C****C****U****G****G****C****A****A****C****C**..AU**G****A****A****U****U****G****C****U****A**.**G**.AC**U****A****U****G****A****G****G****G**GAAAA.GUGUGUAACA | |
|  |  | NZ\_AAAC02000001.1/4059572-4059470  | CUUCGAAAAGAAUCA.CU.**G****C****C****U****C****A****U****A****U**AA**U****C****U****U****G****G****A**G.**A****U**.**A**A**G****G****U****C****C****A****U****A****A**.**G****U**UUC**U****A****C****C****U****G****G****C****A****A****C****C**..AU**G****A****A****U****U****G****C****U****A**.**G**.AC**U****A****U****G****A****G****G****G**GAAAA.GUGUGUAACA | |
|  |  | NZ\_AAEN01000012.1/139994-140096  | CUUCGAAAAGAAUCA.CU.**G****C****C****U****C****A****U****A****U**AA**U****C****U****U****G****G****A**G.**A****U**.**A**A**G****G****U****C****C****A****U****A****A**.**G****U**UUC**U****A****C****C****U****G****G****C****A****A****C****C**..AU**G****A****A****U****U****G****C****U****A**.**G**.AC**U****A****U****G****A****G****G****G**GAAAA.GUGUGUAACA | |
|  |  | NZ\_AAEO01000020.1/51249-51351  | CUUCGAAAAGAAUCA.CU.**G****C****C****U****C****A****U****A****U**AA**U****C****U****U****G****G****A**G.**A****U**.**A**A**G****G****U****C****C****A****U****A****A**.**G****U**UUC**U****A****C****C****U****G****G****C****A****A****C****C**..AU**G****A****A****U****U****G****C****U****A**.**G**.AC**U****A****U****G****A****G****G****G**GAAAA.GUGUGUAACA | |
|  |  | NZ\_AAEP01000042.1/12489-12387  | CUUCGAAAAGAAUCA.CU.**G****C****C****U****C****A****U****A****U**AA**U****C****U****U****G****G****A**G.**A****U**.**A**A**G****G****U****C****C****A****U****A****A**.**G****U**UUC**U****A****C****C****U****G****G****C****A****A****C****C**..AU**G****A****A****U****U****G****C****U****A**.**G**.AC**U****A****U****G****A****G****G****G**GAAAA.GUGUGUAACA | |
|  |  | NZ\_AAEQ01000019.1/51159-51261  | CUUCGAAAAGAAUCA.CU.**G****C****C****U****C****A****U****A****U**AA**U****C****U****U****G****G****A**G.**A****U**.**A**A**G****G****U****C****C****A****U****A****A**.**G****U**UUC**U****A****C****C****U****G****G****C****A****A****C****C**..AU**G****A****A****U****U****G****C****U****A**.**G**.AC**U****A****U****G****A****G****G****G**GAAAA.GUGUGUAACA | |
|  |  | NZ\_AAER01000035.1/118314-118212  | CUUCGAAAAGAAUCA.CU.**G****C****C****U****C****A****U****A****U**AA**U****C****U****U****G****G****A**G.**A****U**.**A**A**G****G****U****C****C****A****U****A****A**.**G****U**UUC**U****A****C****C****U****G****G****C****A****A****C****C**..AU**G****A****A****U****U****G****C****U****A**.**G**.AC**U****A****U****G****A****G****G****G**GAAAA.GUGUGUAACA | |
|  |  | NZ\_AAES01000032.1/51320-51422  | CUUCGAAAAGAAUCA.CU.**G****C****C****U****C****A****U****A****U**AA**U****C****U****U****G****G****A**G.**A****U**.**A**A**G****G****U****C****C****A****U****A****A**.**G****U**UUC**U****A****C****C****U****G****G****C****A****A****C****C**..AU**G****A****A****U****U****G****C****U****A**.**G**.AC**U****A****U****G****A****G****G****G**GAAAA.GUGUGUAACA | |
|  |  | NC\_006371.1/1538896-1538796  | UAAUAAUGGGUAAAG.UU.**A****C****U****U****C****G****U****A****U**AA**C****C****C****C****A****C****U**U.**A****U**.**A**G**G****G****U****G****U****G****G****G****G**.**G****U**CUC**U****A****C****C****A****G****A****A****U**-**C****C**..GU**A****A****A**-**A****U****U****C****U**.**G**.AU**U****A****C****G****A****A****G****A**GUUGA.GUGAUUGAUC | |
|  |  | NC\_004460.1/504371-504471  | GACUUUCGGCGAUCA.AC.**G****C****U****U****C****A****U****A****U**AA**U****C****C****U****A****A****U**G.**A****U**.**A**U**G****G****U****U****U****G****G****G****A**.**G****U**UUC**U****A****C****C****A****A****G****A****G**-**C****C**..UU**A****A****A**-**C****U****C****U****U**.**G**.AU**U****A****U****G****A****A****G****U**CUGUC.GCUUUAUCCG | |
|  |  | NC\_005140.1/1130553-1130653  | GACUUUCGGCGAUCA.AC.**G****C****U****U****C****A****U****A****U**AA**U****C****C****U****A****A****U**G.**A****U**.**A**U**G****G****U****U****U****G****G****G****A**.**G****U**UUC**U****A****C****C****A****A****G****A****G**-**C****C**..UU**A****A****A**-**C****U****C****U****U**.**G**.AU**U****A****U****G****A****A****G****U**CUGUC.GCUUUAUCCG | |
|  |  | NC\_004116.1/1094305-1094209  | CAAUUAAAUAUAUGA.UU.**U****A****C****U****U****A****U****U****U**AU-**G****C****U****G****A****G**G.**A****U**.-U**G****G****C****U****U****A****G****C**-.**G****U**CUC**U****A****C****A****A****G****A****C****A**-**C****C**..GU-**A****A**-**U****G****U****C****U**.**A**.AC**A****A****U****A****A****G****U****A**AGCUA.AUAAAUAGCU | |
|  |  | NC\_004368.1/1163490-1163394  | CAAUUAAAUAUAUGA.UU.**U****A****C****U****U****A****U****U****U**AU-**G****C****U****G****A****G**G.**A****U**.-U**G****G****C****U****U****A****G****C**-.**G****U**CUC**U****A****C****A****A****G****A****C****A**-**C****C**..GU-**A****A**-**U****G****U****C****U**.**A**.AC**A****A****U****A****A****G****U****A**AGCUA.AUAAAUAGCU | |
|  |  | NZ\_AADT03000005.1/42245-42347  | CAAUAAAGCCAGUCU.UA.**A****C****U****U****C****G****U****A****U**AU**C****C****C****C****G****G****C**A.**A****U**.**A**G**G****G****A****C****C****G****G****G****G**.**G****U**UUC**U****A****C****C****A****G****G****C****A****A****C****C**..GC**A****A****A****U****U****G****C****C****C**.**G**.GC**U****A****C****G****A****A****G****G**UAUAU.CUUCGUUGCU | |
|  |  | NC\_004557.1/2551392-2551293  | CUCUAUAAUAAAUUA.UU.**G****A****C****U****C****A****U****A****U**AU-**C****C****C****C****U****U**A.**A****U**.**A**A**G****G****U****A****G****G****G****A**-.**G****U**AUC**U****A****C****C****A****G****A****A****G**-**C****C**..UU**A****A****A**-**C****U****U****C****U**.**G**.AC**U****A****U****G****A****G****U****G**AAUAAAGCAUUGUCAU | |
|  |  | NC\_003028.1/1754786-1754882  | AAAAUUGAAUAUCGU.UU.**U****A****C****U****U****G****U****U****U**AU-**G****U****C****G****U****G**A.**A****U**.-U**G****G****C****A****C****G****A****C**-.**G****U**UUC**U****A****C****A****A****G****G****U****G**-**C****C**..GG-**A****A**-**C****A****C****C****U**.**A**.AC**A****A****U****A****A****G****U****A**AGUCA.GCAGUGAGAU | |
|  |  | NC\_003098.1/1634825-1634921  | AAAAUUGAAUAUCGU.UU.**U****A****C****U****U****G****U****U****U**AU-**G****U****C****G****U****G**A.**A****U**.-U**G****G****C****A****C****G****A****C**-.**G****U**UUC**U****A****C****A****A****G****G****U****G**-**C****C**..GG-**A****A**-**C****A****C****C****U**.**A**.AC**A****A****U****A****A****G****U****A**AGUCA.GCAGUGAGAU | |
|  |  | NZ\_AAGY01000085.1/5640-5736  | AAAAUUGAAUAUCGU.UU.**U****A****C****U****U****G****U****U****U**AU-**G****U****C****G****U****G**A.**A****U**.-U**G****G****C****A****C****G****A****C**-.**G****U**UUC**U****A****C****A****A****G****G****U****G**-**C****C**..GG-**A****A**-**C****A****C****C****U**.**A**.AC**A****A****U****A****A****G****U****A**AGUCA.GCAGUGAGAU | |
|  |  | NZ\_AAEK01000001.1/183902-184006  | AUAAUUUUACACAUU.AU.**C****A****C****U****C****G****U****A****U**AU**A****C****U****C****G****G****U**A.**A****U**.**A**U**G****G****U****C****C****G****A****G****C**.**G****U**UUC**U****A****C****C****U****A****G****U****U****C****C****C**AAUG**A****A****A****G****A****A****C****U****G**.**G**.AC**U****A****C****G****G****G****U****U**AAAGU.AUUCGGUCGC | |
|  |  | NC\_003454.1/1645820-1645721  | UAAAUAAUUUUAAUA.AA.**A****A****U****U****C****G****U****A****U**AA-**G****C****C****U****A****A**U.**A****U**.**A**U**G****G****A****A****G****G****G****U**-.**G****U**CCC**U****A****C**-**G****G****U****U****A****A****C****C**..AU**A****A****A****U****U****A****A****C****C**.**A**.GC**U****A****C****G****A****A****A****A**AUGUU.UUACUGUGUU | |
|  |  | NZ\_AADW02000027.1/2980-2880  | GAAUAAACGUAUAGC.AA.**C****G****C****U****C****G****U****A****U**AA**U****A****G****U****G****G****G**G.**A****U**.-U**G****G****C****C****C****A****C****G****A**.**G****U**CUC**U****A****C****C****G****G****A****U****C****G****C****C**..GU-**A****A****C****G****A****U****C****C**.**G**.AC**U****A****C****G****G****G****U****G**GUGAG.UUACUGCUCU | |
|  |  | NZ\_AADW02000005.1/179959-180061  | AAAUCAUACAUGCAU.CU.**C****C****U****U****U****G****U****A****U**AU**A****C****U****C****G****C****G**A.**A****U**.**A**U**G****G****C****G****U****G****A****G****A**.**G****U**CUC**U****A****C****C****G****G****G****U****C****A****C****C**..UU**A****A****A****C****G****A****C****C****U**.**G**.AC**U****A****U****G****A****A****G****G**AGCAG.ACCCUUCGUA | |
|  |  | NC\_006582.1/1039390-1039492  | AAUGUCCAAUAGGAA.AA.**U****A****C****C****C****G****U****A****U**AA**U****U****G****C****A****G****G**A.**A****U**.**A**A**G****G****C****C****U****G****C****A****C**.**G****U**UUC**U****A****C****C****G****A****G****C****C****A****C****C**..GU**A****A****A****U****G****G****C****U****U**.**G**.AC**U****A****C****G****G****C****A****U**GAUAA.AUGGAGCGCA | |
|  |  | NC\_002737.1/930749-930845  | UGAAUUCAAUAAUGA.CA.**U****A****C****U****U****A****U****U****U**AU-**G****C****U****G****U****G**A.**A****U**.-U**G****G****C****G****C****A****G****C**-.**G****U**CUC**U****A****C****A****A****G****A****C****A**-**C****C**..UU-**A****A**-**U****G****U****C****U**.**A**.AC**A****A****U****A****A****G****U****A**AGCUU.UUAGGCUUGC | |
|  |  | NC\_003485.1/910599-910695  | UGAAUUCAAUAAUGA.CA.**U****A****C****U****U****A****U****U****U**AU-**G****C****U****G****U****G**A.**A****U**.-U**G****G****C****G****C****A****G****C**-.**G****U**CUC**U****A****C****A****A****G****A****C****A**-**C****C**..UU-**A****A**-**U****G****U****C****U**.**A**.AC**A****A****U****A****A****G****U****A**AGCUU.UUAGGCUUGC | |
|  |  | NC\_004070.1/846557-846653  | UGAAUUCAAUAAUGA.CA.**U****A****C****U****U****A****U****U****U**AU-**G****C****U****G****U****G**A.**A****U**.-U**G****G****C****G****C****A****G****C**-.**G****U**CUC**U****A****C****A****A****G****A****C****A**-**C****C**..UU-**A****A**-**U****G****U****C****U**.**A**.AC**A****A****U****A****A****G****U****A**AGCUU.UUAGGCUUGC | |
|  |  | NC\_004606.1/977077-977173  | UGAAUUCAAUAAUGA.CA.**U****A****C****U****U****A****U****U****U**AU-**G****C****U****G****U****G**A.**A****U**.-U**G****G****C****G****C****A****G****C**-.**G****U**CUC**U****A****C****A****A****G****A****C****A**-**C****C**..UU-**A****A**-**U****G****U****C****U**.**A**.AC**A****A****U****A****A****G****U****A**AGCUU.UUAGGCUUGC | |
|  |  | NC\_006086.1/857664-857760  | UGAAUUCAAUAAUGA.CA.**U****A****C****U****U****A****U****U****U**AU-**G****C****U****G****U****G**A.**A****U**.-U**G****G****C****G****C****A****G****C**-.**G****U**CUC**U****A****C****A****A****G****A****C****A**-**C****C**..UU-**A****A**-**U****G****U****C****U**.**A**.AC**A****A****U****A****A****G****U****A**AGCUU.UUAGGCUUGC | |
|  |  | NZ\_AAFV01000199.1/507-411  | UGAAUUCAAUAAUGA.CA.**U****A****C****U****U****A****U****U****U**AU-**G****C****U****G****U****G**A.**A****U**.-U**G****G****C****G****C****A****G****C**-.**G****U**CUC**U****A****C****A****A****G****A****C****A**-**C****C**..UU-**A****A**-**U****G****U****C****U**.**A**.AC**A****A****U****A****A****G****U****A**AGCUU.UUAGGCUUGC | |
|  |  | NC\_004605.1/1369721-1369821  | UAUAAUCGCAAGCGU.UU.**G****C****U****U****C****G****U****A****U**AA**C****C****C****C****A****A****U**G.**A****U**.**A**U**G****G****U****U****U****G****G****G****G**.**G****U**CUC**U****A****C****C****A****G****U****U****C**-**C****C**..GC**A****A****A**-**G****U****G****C****U**.**G**.AU**U****A****C****G****A****A****G****A**GUUGA.GAUCACUGUG | |
|  |  | NZ\_AAAW03000004.1/66862-66959  | UGAACUGAAUAAUAA.AA.**U****U****U****U****U****A****U****A****U**AA-**G****U****U****C****A****U**A.**A****U**.-G**G****G****U****U****G****A****A****C**-.**G****U**CUC**U****A****C****C****A****A****C****U****A**-**C****C**..GU**A****A****A**-**U****A****G****U****U**.**G**.AU**U****A****U****A****A****A****A****A**UUUCG.AACGGAAUGA | |
|  |  | NC\_004567.1/2968830-2968731  | UUAUCAAUACAACUA.AU.**U****G****C****C****U****A****U****A****U**AA**U**-**G****C****C****A****U**G.**A****U**.**A**U**G****G****A****U****G****G****C****G****A**.**G****U**UUC**U****A****C****C****C****A****G****U****G**-**C****C**..GU**A****A****A**-**C****A****C****U****G**.**G**.AC**U****A****U****A****A****G****C****G**AAUUG.AGUCGACGGG | |
|  |  | NZ\_AAEV01000003.1/13765-13667  | ACAAUCAAAUAAAAC.UU.**A****G****C****C****U****A****U****A****U**AA**U****G****U****C**-**U****U**A.**A****U**.**C**U**G****G****U**-**U****G****A****C****A**.**G****U**UUC**U****A****C****C****C****A****A****C****G**-**C****C**..GU**A****A****A**-**U****G****U****U****G**.**G**.AC**U****A****U****A****G****G****A****A**AACUA.ACUCUUAUGU | |
|  |  | NC\_002570.2/806873-806971  | UUAAUCGAGCUCAAC.AC.**U****C****U****U****C****G****U****A****U**AU**C****C****U****C**-**U****C**A.**A****U**.**A**U**G****G****G****A****U****G****A****G****G**.**G****U**CUC**U****A****C**-**A****G****G****U****A**-**C****C**..GU**A****A****A**-**U****A****C****C****U**.**A**.GC**U****A****C****G****A****A****A****A**GAAUG.CAGUUAAUGU | |
|  |  | NZ\_AAEK01000052.1/27552-27452  | AUUAAUUACAUAUGA.GA.**A****U****C****A****U****G****U****A****U**AA**C****U****C****C****A****A****G**A.**A****U**.**A**U**G****G****C****U****U****G****G****G****G**.**G****U**CUC**U****A****C****C****A****G****G****A****A**-**C****C**..AA**U****A****A**-**C****U****C****C****U**.**G**.AC**U****A****C****A****A****A****A****U**GCGUA.UUAUAGCGUU | |
|  |  | NC\_006814.1/237722-237626  | AUUAAGCCAAUACAA.AU.**A****U****C****U**-**A****U****A****U**AU-**C****G****U****C****G****A**A.**A****U**.**A**A**G****G****U****C****G****A****C****A**-.**G****U**UUC**U****A****C****C****C****A****C****U****A**-**C****C**..GU**A****A****A**-**U****G****G****U****G**.**G**.AC**U****A****U**-**A****G****G****U**AAACG.AAAUUAAGAA | |
|  |  | NC\_005363.1/3414604-3414703  | AAAUAACUUCAUAGUGUU.**U****C****C****C****C****G****U****A****U**AU-**G****U****U****G****C****G**A.**A****U**.**A**G**G****G****C****G****C****A****G****C**-.**G****U**UUC**U****A****C****C****A****G****G****C****A**-**C****C**..UC**A****A****A**-**U****G****C****C****U**.**G**.AC**U****A****U****G****G****A****G****G**UUCUU.UGUGAAGUUG | |
|  |  | NZ\_AADT03000021.1/9410-9513  | AAAAUAAAUAUGGCA.AU.**G****G****C****C****U****G****U****A****U**AA**U****U****G****G****G****G****G**A.**A****U**.**A**G**G****G****C****U****C****C****C****A****A**.**G****U**UUC**U****A****C****C****G****G****G****C****A****A****C****C**..GU**A****A****A****U****U****G****C****C****C**U**G**.GC**U****A****C****A****G****C****C****U**GAAAG.UGUACCUCAG | |
|  |  | NC\_004668.1/2288426-2288328  | UCCUAGAGAAACAUA.GA.**A****G****C****U****U****G****U****A****U**AA-**G****G****U****C****A****A**A.**A****U**.**A**C**G****G****U****U****G****A****C****C**-.**G****U**CUC**U****A****C****C****C****A****G****C****A**-**C****C**..GU**A****A****A**-**U****G****C****U****G**.**G**.UC**U****A****U****A****A****G****U****G**AAGAA.GAGCGGAUAG | |
|  |  | NC\_006449.1/1182949-1183044  | CAAACAAUGAGAACA.-U.**U****A****C****U****U****A****U****U****U**AU-**G****U****C****A****C****G**A.**A****U**.-G**G****G****C****G****U****G****A****C**-.**G****U**UUC**U****A****C****A****A****G****G****U****G**-**C****C**..GU-**A****A**-**C****A****C****C****U**.**A**.AC**A****A****U****A****A****G****U****A**AGCUA.AUUUAGUCAU | |
|  |  | NZ\_AAGS01000026.1/9921-10016  | CAAACAAUGAGAACA.-U.**U****A****C****U****U****A****U****U****U**AU-**G****U****C****A****C****G**A.**A****U**.-G**G****G****C****G****U****G****A****C**-.**G****U**UUC**U****A****C****A****A****G****G****U****G**-**C****C**..GU-**A****A**-**C****A****C****C****U**.**A**.AC**A****A****U****A****A****G****U****A**AGCUA.AUUUAGUCAU | |
|  |  | NZ\_AAGQ01000089.1/170-269  | UAACUUAACAAAUGU.UUC**U****G****C****U****U****A****U****A****U**AU-**C****G****C****U****G****C**G.**A****U**.**A**C**G****G****G****U****A****G****C****A**-.**G****U**CUC**U****A****C****C****C****G****G****A****G**-**C****C**..GU**A****A****A**-**C****U****C****C****G**.**G**.AC**U****A****U****A****G****G****U****A**AAGAA.GGGCCGGUAU | |
|  |  | NZ\_AABF02000293.1/1-98  | UAGAUAUUAAAUAAA.--.**A****A****U****U****C****G****U****A****U**AA-**G****C****C****U****A****A**U.**A****U**.**A**U**G****G****A****A****A****G****G****U**-.**G****U**CCC**U****A****C**-**G****G****U****U****A****A****C****C**..GU**A****A****A****U****U****A****A****C****C**.**A**.GC**U****A****C****G****A****A****A****A**AUGUU.UUUGCUGUAU | |
|  |  | NC\_006448.1/1185082-1185177  | CAAACAAUGAGAACA.-U.**U****A****C****U****U****A****U****U****U**AU-**G****U****C****A****C****G**A.**A****U**.-G**G****G****C****G****U****G****A****C**-.**G****U**UUC**U****A****C****A****A****G****G****U****G**-**C****C**..GU-**A****A**-**C****U****C****C****U**.**A**.AC**A****A****U****A****A****G****U****A**AGCUA.AUUUAGUCAU | |
|  |  | NC\_004567.1/2410478-2410577  | UUCAAAUAAGUGGUA.AU.**U****G****C****C****U****A****U****A****U**AA**U**-**G****U****C****A****U**G.**A****U**.**A**U**G****G****U****U****G****A****C****G****A**.**G****U**UUC**U****A****C****C****C****A****A****C****C**-**C****C**..GU**A****A****A**-**G****G****U****U****G**.**G**.AC**U****A****U****A****A****G****C****A**AACGA.GGUCAUCCCG | |
|  |  | NZ\_AABJ03000010.1/47704-47802  | AUUCAGAAUGUUGAA.AA.**A****G****C****U****U****A****U****A****U**AU**G****G****U****C****G****U**-A.**A****U**.**A**A**G****G**-**A****U****G****A****C****C**.**G****U**UUC**U****A****C****C****C****G****G****A****G**-**C****C**..AC**A****A****A**-**C****U****C****A****G**.**G**.AC**U****A****U****A****A****G****C****A**AUUAA.GUACUUGUGC | |
|  |  | NZ\_AADT03000002.1/89184-89083  | GAGUCUUCUUUUAGG.UU.**U****C****U****U****C****G****U****A****U**AG**U****C****C****C****G****G****A**G.**A****U**.-U**G****G****U****C****C****G****G****G****G**.**G****U**UUC**U****A****C****C****A****G****G****U****G****A****C****C**..GG-**A****A****U****C****A****C****C****U**U**G**.GC**U****A****C****G****A****A****G****G**GUUAU.UUCCUUUGUG | |
|  |  | NC\_006814.1/1971978-1972076  | AUACUUAACAAUCAA.GU.**U****A****U****C****U****A****U****A****U**AU-**C****G****U****C****G****A**A.**A****U**.**A**A**G****G****U****C****G****A****C****A**-.**G****U**AUC**U****A****C****C****C****U****G****A****G**-**C****C**..AU**A****A****A**-**U****U****C****A****G**.**G**.AC**U****A****U****A****G****G****U****A**UCAGA.CGUCAUAAUU | |
|  |  | NZ\_AAAO02000016.1/1579-1480  | CACUCUAGAUAUCAA.AA.**U****A****U****C****U****A****U****A****U**AU-**C****G****U****C****G****U**A.**A****U**.**A**A**G****G****U****C****G****A****C****A**-.**G****U**UUC**U****A****C****C****C****G****G****A****A**-**C****C**A.AU**U****A****A**-**U****U****C****U****G**.**G**.AC**U****A****U****A****G****G****U****A**AUCGA.UGUCAUAAGU | |
|  |  | NC\_005362.1/1949387-1949485  | AUACUUAACAAUCAA.GU.**U****A****U****C****U****A****U****A****U**AU-**C****G****U****C****G****A**A.**A****U**.**A**A**G****G****U****C****G****A****C****A**-.**G****U**AUC**U****A****C****C****C****U****G****A****G**-**C****C**..AU**A****A****A**-**U****U****C****A****G**.**G**.AC**U****A****U****A****G****G****U****A**UCAGA.CGUCAUAAAU | |
|  |  | NZ\_AAAO02000035.1/3299-3201  | AUACUUAACAAUCAA.GU.**U****A****U****C****U****A****U****A****U**AU-**C****G****U****C****G****A**A.**A****U**.**A**A**G****G****U****C****G****A****C****A**-.**G****U**AUC**U****A****C****C****C****U****G****A****G**-**C****C**..AU**A****A****A**-**U****U****C****A****G**.**G**.AC**U****A****U****A****G****G****U****A**UCAGA.CGUCAUAAAU | |
|  |  | NC\_005362.1/263146-263049  | CACUCUAGAUAUCAA.AU.**A****U****C****U**-**A****U****A****U**AU-**C****G****U****C****G****U**A.**A****U**.**A**A**G****G****U****C****G****A****C****A**-.**G****U**UUC**U****A****C****C****C****G****G****A****A**-**C****C**A.AU**U****A****A**-**U****U****C****U****G**.**G**.AC**U****A****U**-**A****G****G****U**AAUCG.AUGUCAUAAG | |
|  |  | NC\_005363.1/2004933-2004835  | ACUACUAACCGCGGU.UA.**C****A****C****A****A****A****U****A****U**AA-**G****U****C****G****G****A**G.**A****U**.**A**G**G****G****U****C****U****G****A****C**-.**G****U**UUC**U****A****C****C****U****G****C****C****A**-**C****C**..GU**A****A****A**-**G****G****G****C****A**.**G**.UC**U****A****U****U****U****G****G****A**GCGAA.UAUAUGUUGA | |
|  |  | NZ\_AADT03000002.1/98293-98192  | GACGAAUAUUACUAU.UA.**G****A****C****U****C****G****U****A****U**AA**C****C****C****C****G****G****C**G.**A****U**.-G**G****G****G****C****C****G****G****G****G**.**G****U**CUC**U****A****C****C****A****G****G****U****G****A****C****C**..GG-**A****A****U****C****A****C****C****U**C**G**.GC**U****A****C****G****A****G****G****G**UGAGC.GGCAGCUGGU | |
|  |  | NZ\_AABH02000036.1/17403-17500  | CAACUAAAGAAGUUA.UU.**U****G****C****A****G****A****U****A****U**AU-**C****G****U****U****G****G**A.**A****A**.**A**C**G****G****C****C****A****A****C****A**-.**G****U**UUC**U****A****C****C****A****C****G****C****C**-**C****C**..-A**A****A****A**-**G****U****C****G****U**.**G**.AC**U****A****U****C****C****G****C****A**AAUGU.UUUUGACGAU | |
|  |  | NC\_006055.1/484139-484044  | UAUAAAAUUAAUAUG.AA.**A****A****C****U****U****G****U****A****U**AA**U****C****C****U****U****C**--.**A****U**.**A**U**C****G****G****G****A****A****G****G****A**.**G****U**CUC**U****A****C****C****U****A****A****C****A**-**C****C**..---**A****A**-**U****G****U****U****A**.**G**.AU**U****A****U****G****A****G****U****U**UUAUG.GUUUUCGCUA | |
|  |  | NZ\_AABH02000272.1/211-308  | CAACUAAAGAAGUUA.UU.**U****G****C****A****G****A****U****A****U**AU-**C****G****U****U****G****G**A.**A****A**.**A**C**G****G****C****C****A****A****C****A**-.**G****U**UUC**U****A****C****C****A****C****G****C****C**-**C****C**..-A**A****A****A**-**G****U****C****G****U**.**G**.AC**U****A****U****C****C****G****C****A**AAUGU.UUCUGACGAU | |
|  |  | NZ\_AABJ03000003.1/47905-47810  | AAGAUAAAUAGCAAC.CA.**A****G****C****A****G****G****U****A****U**AU-**C****G****U****C****G****G**A.**U****A**.**A**U**G****G****C****U****G****A****C****A**-.**G****U**UUC**U****A****C****C****C****A****A****C****A**-**C****C**..---**A****A**-**U****G****U****U****G**.**G**.AC**U****A****U****C****U****G****U****G**GAUGU.CUUUUUGGCG | |
|  |  | NZ\_AABH02000038.1/17559-17463  | UAUUCUAUGUGAAAU.UU.**A****G****C****U****G****A****U****A****U**AG**U****A****U****C****G****A**--.**A****U**A**A**U**G****G**-**U****C****G****A****U****U**.**G****U**UUC**U****A****G****C****C****A****G****C****A**-**C****C**..---**C****A**-**U****G****C****U****G**.**G**AAC**U****A****U****C****A****U****A****A**ACAUG.UUAUUUAAUU | |
|  |  | NC\_006055.1/397027-397123  | AAUAAUUAAAUAUAA.AA.**A****A****C****U****U****A****U****A****C**AU-**G****A****C****A****A****C**AU**A****U**.-U**G****G****G****U****U****G****U****C**-.**G****A**C-C**U****G****C****C****U****C****U****G****G****A****C****C**..-U--**A****U****C****C****U****U****A**.**G**.AC**U****A****U****A****A****G****C****G**UGAGG.UUUUUUUACA | |
|  |  | SS\_cons |  | ...................<<<<<<<<B..<<<<<<<..ZZ.Z.AA>>>>>>>.<[...]b><<<<<<<aa....zzz>>>>>>.>...>>>>>>>>................ |
|  |  | SS\_label |  | ...................===P1===...---P2--.........--P2---.........---P3--.........---P3---...===P1===................ |
|  |  | RF |  | aAAauuaaAaAAAaA.au.aacUCgUAUAAucucgggA.AU.AUGGcccgaga.GUUUCUACCaggcaaCC..GUAAAuugccu.G.ACUAcGAguaAauuu.uauuUaUuuu |
|  |  | SS\_align |  | :::::::::::::::.::.((((((((,,,<<<<<<<\_.\_\_.\_\_\_\_>>>>>>>.,,,,,,,,<<<<<<<\_\_..\_\_\_\_\_>>>>>>.>.,,)))))))):::::.:::::::::: |
