# Supplementary material for: The distributions, mechanisms, and structures of metabolite-binding riboswitches
Source: Genome Biol. 2007 Nov 12;8(11):R239. doi: 10.1186/gb-2007-8-11-r239 (PMC2258182; doi:10.1186/gb-2007-8-11-r239)
Supplement: Additional data file 2 — Sequence alignments of the riboswitch aptamer data sets annotated with new base-base interactions in HTML format. [file gb-2007-8-11-r239-S2.zip › HTML/SAM-I.html]

|  |  |  |  |  |
| --- | --- | --- | --- | --- |
|  |  | **Accession/Start-End** |  | **Sequence** |
|  |  | NC\_002570.2/910199-910081  | UCAU**A****U****U****U****U****C****U****U**.**A****U****C**C.AGA**G**.**U****G****G****U**.**G****G****A****G****G****G**A...**C****U****G**.**G**.**C****C****C**UGU**G****A****A**.**G****C****C**-.**C****G****G****C**AA**C****C**.**U**.**C****U****U****U**-........................UUUU-....................................................-**A****A****A****G**.A...**A**..**G****G**U**G**..**C****C**AAU**U****C****C****A****G**...**C****A****G****A****A****C**....................AUGA..........U**G****U****U****C****U****G**.AA.**A**..**G****A**..**U****A****A****G****A****A****G****C****G**AACG | |
|  |  | NC\_003997.3/2953235-2953120  | AUGA**A****A****A****U****U****C****U****U**.**A****U****C**A.CGA**G**.**A****G****G****U**.**G****G****A****G****G****G**A...**C****U****G**.**G**.**C****C****C**UAU**G****A****A**.**A****C****C****U**.**C****G****G****C**AG**C****G**.**G**.**A****U****U****C**-........................GUUAU....................................................-**G****A****A****U**.A...**C**..**U****G**U**G**..**C****C**AAU**U****C****C****A****G**...**C****A****A****G**--....................GUAA...........--**C****U****U****G**.AA.**A**..**G****A**..**U****A****A****G****A****A****A****G****A**AGCU | |
|  |  | NC\_005945.1/2953927-2953812  | AUGA**A****A****A****U****U****C****U****U**.**A****U****C**A.CGA**G**.**A****G****G****U**.**G****G****A****G****G****G**A...**C****U****G**.**G**.**C****C****C**UAU**G****A****A**.**A****C****C****U**.**C****G****G****C**AG**C****G**.**G**.**A****U****U****C**-........................GUUAU....................................................-**G****A****A****U**.A...**C**..**U****G**U**G**..**C****C**AAU**U****C****C****A****G**...**C****A****A****G**--....................GUAA...........--**C****U****U****G**.AA.**A**..**G****A**..**U****A****A****G****A****A****A****G****A**AGCU | |
|  |  | NC\_007530.2/2953363-2953248  | AUGA**A****A****A****U****U****C****U****U**.**A****U****C**A.CGA**G**.**A****G****G****U**.**G****G****A****G****G****G**A...**C****U****G**.**G**.**C****C****C**UAU**G****A****A**.**A****C****C****U**.**C****G****G****C**AG**C****G**.**G**.**A****U****U****C**-........................GUUAU....................................................-**G****A****A****U**.A...**C**..**U****G**U**G**..**C****C**AAU**U****C****C****A****G**...**C****A****A****G**--....................GUAA...........--**C****U****U****G**.AA.**A**..**G****A**..**U****A****A****G****A****A****A****G****A**AGCU | |
|  |  | NZ\_AAAC02000001.1/3418914-3418799  | AUGA**A****A****A****U****U****C****U****U**.**A****U****C**A.CGA**G**.**A****G****G****U**.**G****G****A****G****G****G**A...**C****U****G**.**G**.**C****C****C**UAU**G****A****A**.**A****C****C****U**.**C****G****G****C**AG**C****G**.**G**.**A****U****U****C**-........................GUUAU....................................................-**G****A****A****U**.A...**C**..**U****G**U**G**..**C****C**AAU**U****C****C****A****G**...**C****A****A****G**--....................GUAA...........--**C****U****U****G**.AA.**A**..**G****A**..**U****A****A****G****A****A****A****G****A**AGCU | |
|  |  | NZ\_AAEN01000017.1/189376-189261  | AUGA**A****A****A****U****U****C****U****U**.**A****U****C**A.CGA**G**.**A****G****G****U**.**G****G****A****G****G****G**A...**C****U****G**.**G**.**C****C****C**UAU**G****A****A**.**A****C****C****U**.**C****G****G****C**AG**C****G**.**G**.**A****U****U****C**-........................GUUAU....................................................-**G****A****A****U**.A...**C**..**U****G**U**G**..**C****C**AAU**U****C****C****A****G**...**C****A****A****G**--....................GUAA...........--**C****U****U****G**.AA.**A**..**G****A**..**U****A****A****G****A****A****A****G****A**AGCU | |
|  |  | NZ\_AAEO01000022.1/912237-912122  | AUGA**A****A****A****U****U****C****U****U**.**A****U****C**A.CGA**G**.**A****G****G****U**.**G****G****A****G****G****G**A...**C****U****G**.**G**.**C****C****C**UAU**G****A****A**.**A****C****C****U**.**C****G****G****C**AG**C****G**.**G**.**A****U****U****C**-........................GUUAU....................................................-**G****A****A****U**.A...**C**..**U****G**U**G**..**C****C**AAU**U****C****C****A****G**...**C****A****A****G**--....................GUAA...........--**C****U****U****G**.AA.**A**..**G****A**..**U****A****A****G****A****A****A****G****A**AGCU | |
|  |  | NZ\_AAEP01000026.1/239046-238931  | AUGA**A****A****A****U****U****C****U****U**.**A****U****C**A.CGA**G**.**A****G****G****U**.**G****G****A****G****G****G**A...**C****U****G**.**G**.**C****C****C**UAU**G****A****A**.**A****C****C****U**.**C****G****G****C**AG**C****G**.**G**.**A****U****U****C**-........................GUUAU....................................................-**G****A****A****U**.A...**C**..**U****G**U**G**..**C****C**AAU**U****C****C****A****G**...**C****A****A****G**--....................GUAA...........--**C****U****U****G**.AA.**A**..**G****A**..**U****A****A****G****A****A****A****G****A**AGCU | |
|  |  | NZ\_AAEQ01000035.1/280128-280243  | AUGA**A****A****A****U****U****C****U****U**.**A****U****C**A.CGA**G**.**A****G****G****U**.**G****G****A****G****G****G**A...**C****U****G**.**G**.**C****C****C**UAU**G****A****A**.**A****C****C****U**.**C****G****G****C**AG**C****G**.**G**.**A****U****U****C**-........................GUUAU....................................................-**G****A****A****U**.A...**C**..**U****G**U**G**..**C****C**AAU**U****C****C****A****G**...**C****A****A****G**--....................GUAA...........--**C****U****U****G**.AA.**A**..**G****A**..**U****A****A****G****A****A****A****G****A**AGCU | |
|  |  | NZ\_AAER01000040.1/684532-684417  | AUGA**A****A****A****U****U****C****U****U**.**A****U****C**A.CGA**G**.**A****G****G****U**.**G****G****A****G****G****G**A...**C****U****G**.**G**.**C****C****C**UAU**G****A****A**.**A****C****C****U**.**C****G****G****C**AG**C****G**.**G**.**A****U****U****C**-........................GUUAU....................................................-**G****A****A****U**.A...**C**..**U****G**U**G**..**C****C**AAU**U****C****C****A****G**...**C****A****A****G**--....................GUAA...........--**C****U****U****G**.AA.**A**..**G****A**..**U****A****A****G****A****A****A****G****A**AGCU | |
|  |  | NZ\_AAES01000035.1/853998-853883  | AUGA**A****A****A****U****U****C****U****U**.**A****U****C**A.CGA**G**.**A****G****G****U**.**G****G****A****G****G****G**A...**C****U****G**.**G**.**C****C****C**UAU**G****A****A**.**A****C****C****U**.**C****G****G****C**AG**C****G**.**G**.**A****U****U****C**-........................GUUAU....................................................-**G****A****A****U**.A...**C**..**U****G**U**G**..**C****C**AAU**U****C****C****A****G**...**C****A****A****G**--....................GUAA...........--**C****U****U****G**.AA.**A**..**G****A**..**U****A****A****G****A****A****A****G****A**AGCU | |
|  |  | NC\_003869.1/1750376-1750263  | UUAA**A****A****U****C****U****C****U****U**.**A****U****C**A.AGA**G**.**A****G****G****U**.**G****G****A****G****G****G**A...**C****U****G**.**G**.**C****C****C**GAU**G****A****A**.**A****C****C**-.**C****G****G****C**AA**C****C**.**A**.**G****C****C**--........................UUAG-....................................................--**G****G****C**.A...**U**..**G****G**U**G**..**C****C**AAU**U****C****C****U****G**...**C****A****G****C****G**-....................GUUU...........-**C****G****C****U****G**.AA.**A**..**G****A**..**U****G****A****G****A****G****A****U****U**CUUG | |
|  |  | NC\_003909.8/3019934-3019819  | AUGA**A****A****A****U****U****C****U****U**.**A****U****C**A.CGA**G**.**A****G****G****U**.**G****G****A****G****G****G**A...**C****U****G**.**G**.**C****C****C**UAU**G****A****U**.**A****C****C****U**.**C****G****G****C**AG**C****G**.**G**.**A****U****U****C**-........................GUUAU....................................................-**G****A****A****U**.A...**C**..**U****G**U**G**..**C****C**AAU**U****C****C****A****G**...**C****A****A****G**--....................GUAA...........--**C****U****U****G**.AA.**A**..**G****A**..**U****A****A****G****A****A****A****G****A**AGCU | |
|  |  | NC\_004722.1/3147207-3147092  | AUGA**A****A****A****U****U****C****U****U**.**A****U****C**A.CGA**G**.**A****G****G****U**.**G****G****A****G****G****G**A...**C****U****G**.**G**.**C****C****C**UAU**G****A****U**.**A****C****C****U**.**C****G****G****C**AG**C****G**.**G**.**A****U****U****C**-........................GUUAU....................................................-**G****A****A****U**.A...**C**..**U****G**U**G**..**C****C**AAU**U****C****C****A****G**...**C****A****A****G**--....................GUAA...........--**C****U****U****G**.AA.**A**..**G****A**..**U****A****A****G****A****A****A****G****A**AGCU | |
|  |  | NC\_005957.1/3052794-3052679  | AUGA**A****A****A****U****U****C****U****U**.**A****U****C**A.CGA**G**.**A****G****G****U**.**G****G****A****G****G****G**A...**C****U****G**.**G**.**C****C****C**UAU**G****A****U**.**A****C****C****U**.**C****G****G****C**AG**C****G**.**G**.**A****U****U****C**-........................GUUAU....................................................-**G****A****A****U**.A...**C**..**U****G**U**G**..**C****C**AAU**U****C****C****A****G**...**C****A****A****G**--....................GUAA...........--**C****U****U****G**.AA.**A**..**G****A**..**U****A****A****G****A****A****A****G****A**AGCU | |
|  |  | NC\_006274.1/3037562-3037447  | AUGA**A****A****A****U****U****C****U****U**.**A****U****C**A.CGA**G**.**A****G****G****U**.**G****G****A****G****G****G**A...**C****U****G**.**G**.**C****C****C**UAU**G****A****U**.**A****C****C****U**.**C****G****G****C**AG**C****G**.**G**.**A****U****U****C**-........................GUUAU....................................................-**G****A****A****U**.A...**C**..**U****G**U**G**..**C****C**AAU**U****C****C****A****G**...**C****A****A****G**--....................GUAA...........--**C****U****U****G**.AA.**A**..**G****A**..**U****A****A****G****A****A****A****G****A**AGCU | |
|  |  | NC\_004722.1/174297-174169  | ACAC**A****U****A****C****U****C****U****U**.**A****U****C**A.AGA**G**.**U****G****G****C**.**G****G****A****G****G****G**A...**C****U****G**.**G**.**C****C****C**GAU**G****A****U**.**G****C****C**-.**C****G****G****C**AA**C****C**.**G**.**A****G****C****U****U**AUGAC...................GUAU-....................................................**A****A****G****C****U**.A...**A**..**G****G**U**G**..**C****U**AAU**U****C****C****U****G**...**C****A****A****A****A****C**GAG.................UUUU..........C**G****U****U****U****U****G**.GA.**A**..**G****A**..**U****A****A****G****A****G****A****G****G**AAUC | |
|  |  | NZ\_AAEK01000001.1/280075-280190  | AUGA**A****A****A****U****U****C****U****U**.**A****U****C**G.CGA**G**.**A****G****G****U**.**G****G****A****G****G****G**A...**C****U****G**.**G**.**C****C****C**UAU**G****A****U**.**A****C****C****U**.**C****G****G****C**AG**C****G**.**G**.**A****U****U****C**-........................GUUAU....................................................-**G****A****A****U**.A...**C**..**U****G**U**G**..**C****C**AAU**U****C****C****A****G**...**C****A****A****G**--....................GUAA...........--**C****U****U****G**.AA.**A**..**G****A**..**U****A****A****G****A****A****A****G****A**AGCU | |
|  |  | NC\_003909.8/205489-205361  | ACAC**A****U****A****C****U****C****U****U**.**A****U****C**A.AGA**G**.**U****G****G****C**.**G****G****A****G****G****G**A...**C****U****G**.**G**.**C****C****C**GAU**G****A****U**.**G****C****C**-.**C****G****G****C**AA**C****C**.**G**.**A****G****C****U****U**AUAAC...................GUAU-....................................................**A****A****G****C****U**.A...**A**..**G****G**U**G**..**C****U**AAU**U****C****C****U****G**...**C****A****A****A****A****C**G...................AGUU........CUC**G****U****U****U****U****G**.GA.**A**..**G****A**..**U****A****A****G****A****G****A****G****G**AAUC | |
|  |  | NC\_006274.1/177111-176983  | ACAC**A****U****A****C****U****C****U****U**.**A****U****C**A.AGA**G**.**U****G****G****C**.**G****G****A****G****G****G**A...**C****U****G**.**G**.**C****C****C**GAU**G****A****U**.**G****C****C**-.**C****G****G****C**AA**C****C**.**G**.**A****G****C****U****U**AUAAC...................GUAU-....................................................**A****A****G****C****U**.A...**A**..**G****G**U**G**..**C****U**AAU**U****C****C****U****G**...**C****A****A****A****A****C**GAG.................UUUU..........C**G****U****U****U****U****G**.GA.**A**..**G****A**..**U****A****A****G****A****G****A****G****G**AACC | |
|  |  | NC\_003909.8/4732575-4732453  | CUGA**U****U****U****C****U****C****U****U**.**A****U****C**A.AGA**G**.**A****G****G****U**.**G****G****A****G****G****G**A...**C****U****G**U**G**.**C****C****C**UGU**G****A****A**.**G****C****C**-.**C****G****G****C**AA**C****C**.**G**.-**U****C****A****A**C.......................UUUAU...................................................G**U****U****G****A****A**.A...**U**..**G****G**U**G**..**C****C**AAU**U****C****C****U****G**...**C****A****A****A****G****C**....................AAAU...........**G****C****U****U****U****G**.AG.**A**..**G****A**..**U****G****A****G****A****G****A****G****A**GGGA | |
|  |  | NC\_004557.1/1226207-1226090  | UUAA**A****A****U****A****U****C****U****U**.**A****U****C**A.AGA**G**.**C****G****G****U**.**U****G****A****G****G****G**A...**C****U****G**.**G**.**C****C****C**UAU**G****A****A**.**A****C****C**-.**C****A****G****C**AA**C****C**.**U**.-**A****U****A****C**........................ACAAA....................................................**G****U****A****U****A**.A...**A**..**G****G**U**G**..**C****U**AAC**U****C****C****A****G**...**C****A****G****G****A**-....................AAUU...........-**U****C****C****U****G**.AA.**A**..**G****A**..**U****A****A****G****A****A****A****A****G**UUUA | |
|  |  | NC\_006274.1/1397375-1397497  | AAUA**C****A****A****A****G****C****U****U**.**A****U****C**A.AGA**G**.**A****A****G****C**.**G****G****A****G****G****G**AA..**C****U****G**.**G**.**C****C****C**GGU**G****A****A**.**G****C****U**-.**C****G****G****C**AA**C****C**.**U**.**G****C****U****U**-........................AUAGA....................................................-**A****A****G****C**.A...**A**..**G****G**U**G**..**C****U**AAA**U****C****C****A****G**...**C****A****A****A****A****U**G...................GAAU.........CC**A****U****U****U****U****G**.AA.**A**..**G****A**..**U****A****A****G****G****U****A****A****A**AUAU | |
|  |  | NZ\_AAEK01000065.1/5093-5213  | AUUA**G****U****U****U****U****C****U****U**.**A****U****C**A.AGA**G**.**A****G****A****U**.**G****G****A****G****G****G**A...**C****U****G**.**G**.**C****C****C**GAU**G****A****A**.**A****U****C****U**.**C****A****G****C**AA**C****A**.**G**.**G****C****U**--........................ACACA....................................................--**A****G****U**.A...**C**..**U****G**U**G**..**C****U**AAU**U****C****C****A****G**...**C****A****A****A****C****G**UA..................UGAA..........G**C****G****U****U****U****G**.GA.**A**..**G****A**..**U****G****A****G****G****G****G****A****A**AUGG | |
|  |  | NC\_003997.3/4739984-4739863  | CUGA**U****U****U****C****U****C****U****U**.**A****U****C**A.AGA**G**.**A****G****G****U**.**G****G****A****G****G****G**A...**C****U****G**U**G**.**C****C****C**UGU**G****A****A**.**G****C****C**-.**C****G****G****C**AA**C****C**.**G**.-**U****C****A****A**C.......................UUAU-...................................................G**U****U****G****A****A**.A...**U**..**G****G**U**G**..**C****C**AAU**U****C****C****U****G**...**C****A****A****A****G****C**....................AAAU...........**G****C****U****U****U****G**.AG.**A**..**G****A**..**U****G****A****G****A****G****A****G****A**GGGA | |
|  |  | NC\_004722.1/4894715-4894594  | CUGA**U****U****U****C****U****C****U****U**.**A****U****C**A.AGA**G**.**A****G****G****U**.**G****G****A****G****G****G**A...**C****U****G**U**G**.**C****C****C**UGU**G****A****A**.**G****C****C**-.**C****G****G****C**AA**C****C**.**G**.-**U****C****A****A**C.......................UUAU-...................................................G**U****U****G****A****A**.A...**U**..**G****G**U**G**..**C****C**AAU**U****C****C****U****G**...**C****A****A****A****G****C**....................AAAU...........**G****C****U****U****U****G**.AG.**A**..**G****A**..**U****G****A****G****A****G****A****G****A**GGGA | |
|  |  | NC\_005945.1/4741319-4741198  | CUGA**U****U****U****C****U****C****U****U**.**A****U****C**A.AGA**G**.**A****G****G****U**.**G****G****A****G****G****G**A...**C****U****G**U**G**.**C****C****C**UGU**G****A****A**.**G****C****C**-.**C****G****G****C**AA**C****C**.**G**.-**U****C****A****A**C.......................UUAU-...................................................G**U****U****G****A****A**.A...**U**..**G****G**U**G**..**C****C**AAU**U****C****C****U****G**...**C****A****A****A****G****C**....................AAAU...........**G****C****U****U****U****G**.AG.**A**..**G****A**..**U****G****A****G****A****G****A****G****A**GGGA | |
|  |  | NC\_005957.1/4749683-4749562  | CUGA**U****U****U****C****U****C****U****U**.**A****U****C**A.AGA**G**.**A****G****G****U**.**G****G****A****G****G****G**A...**C****U****G**U**G**.**C****C****C**UGU**G****A****A**.**G****C****C**-.**C****G****G****C**AA**C****C**.**G**.-**U****C****A****A**C.......................UUAU-...................................................G**U****U****G****A****A**.A...**U**..**G****G**U**G**..**C****C**AAU**U****C****C****U****G**...**C****A****A****A****G****C**....................AAAU...........**G****C****U****U****U****G**.AG.**A**..**G****A**..**U****G****A****G****A****G****A****G****A**GGGA | |
|  |  | NC\_006274.1/4808856-4808735  | CUGA**U****U****U****C****U****C****U****U**.**A****U****C**A.AGA**G**.**A****G****G****U**.**G****G****A****G****G****G**A...**C****U****G**U**G**.**C****C****C**UGU**G****A****A**.**G****C****C**-.**C****G****G****C**AA**C****C**.**G**.-**U****C****A****A**C.......................UUAU-...................................................G**U****U****G****A****A**.A...**U**..**G****G**U**G**..**C****C**AAU**U****C****C****U****G**...**C****A****A****A****G****C**....................AAAU...........**G****C****U****U****U****G**.AG.**A**..**G****A**..**U****G****A****G****A****G****A****G****A**GGGA | |
|  |  | NC\_007530.2/4740110-4739989  | CUGA**U****U****U****C****U****C****U****U**.**A****U****C**A.AGA**G**.**A****G****G****U**.**G****G****A****G****G****G**A...**C****U****G**U**G**.**C****C****C**UGU**G****A****A**.**G****C****C**-.**C****G****G****C**AA**C****C**.**G**.-**U****C****A****A**C.......................UUAU-...................................................G**U****U****G****A****A**.A...**U**..**G****G**U**G**..**C****C**AAU**U****C****C****U****G**...**C****A****A****A****G****C**....................AAAU...........**G****C****U****U****U****G**.AG.**A**..**G****A**..**U****G****A****G****A****G****A****G****A**GGGA | |
|  |  | NZ\_AAAC02000001.1/83037-82916  | CUGA**U****U****U****C****U****C****U****U**.**A****U****C**A.AGA**G**.**A****G****G****U**.**G****G****A****G****G****G**A...**C****U****G**U**G**.**C****C****C**UGU**G****A****A**.**G****C****C**-.**C****G****G****C**AA**C****C**.**G**.-**U****C****A****A**C.......................UUAU-...................................................G**U****U****G****A****A**.A...**U**..**G****G**U**G**..**C****C**AAU**U****C****C****U****G**...**C****A****A****A****G****C**....................AAAU...........**G****C****U****U****U****G**.AG.**A**..**G****A**..**U****G****A****G****A****G****A****G****A**GGGA | |
|  |  | NZ\_AAEK01000012.1/81120-80999  | CUGA**U****U****U****C****U****C****U****U**.**A****U****C**A.AGA**G**.**A****G****G****U**.**G****G****A****G****G****G**A...**C****U****G**U**G**.**C****C****C**UGU**G****A****A**.**G****C****C**-.**C****G****G****C**AA**C****C**.**G**.-**U****C****A****A**C.......................UUAU-...................................................G**U****U****G****A****A**.A...**U**..**G****G**U**G**..**C****C**AAU**U****C****C****U****G**...**C****A****A****A****G****C**....................AAAU...........**G****C****U****U****U****G**.AG.**A**..**G****A**..**U****G****A****G****A****G****A****G****A**GGGA | |
|  |  | NZ\_AAEN01000026.1/85046-84925  | CUGA**U****U****U****C****U****C****U****U**.**A****U****C**A.AGA**G**.**A****G****G****U**.**G****G****A****G****G****G**A...**C****U****G**U**G**.**C****C****C**UGU**G****A****A**.**G****C****C**-.**C****G****G****C**AA**C****C**.**G**.-**U****C****A****A**C.......................UUAU-...................................................G**U****U****G****A****A**.A...**U**..**G****G**U**G**..**C****C**AAU**U****C****C****U****G**...**C****A****A****A****G****C**....................AAAU...........**G****C****U****U****U****G**.AG.**A**..**G****A**..**U****G****A****G****A****G****A****G****A**GGGA | |
|  |  | NZ\_AAEO01000032.1/86605-86484  | CUGA**U****U****U****C****U****C****U****U**.**A****U****C**A.AGA**G**.**A****G****G****U**.**G****G****A****G****G****G**A...**C****U****G**U**G**.**C****C****C**UGU**G****A****A**.**G****C****C**-.**C****G****G****C**AA**C****C**.**G**.-**U****C****A****A**C.......................UUAU-...................................................G**U****U****G****A****A**.A...**U**..**G****G**U**G**..**C****C**AAU**U****C****C****U****G**...**C****A****A****A****G****C**....................AAAU...........**G****C****U****U****U****G**.AG.**A**..**G****A**..**U****G****A****G****A****G****A****G****A**GGGA | |
|  |  | NZ\_AAEP01000027.1/83913-83792  | CUGA**U****U****U****C****U****C****U****U**.**A****U****C**A.AGA**G**.**A****G****G****U**.**G****G****A****G****G****G**A...**C****U****G**U**G**.**C****C****C**UGU**G****A****A**.**G****C****C**-.**C****G****G****C**AA**C****C**.**G**.-**U****C****A****A**C.......................UUAU-...................................................G**U****U****G****A****A**.A...**U**..**G****G**U**G**..**C****C**AAU**U****C****C****U****G**...**C****A****A****A****G****C**....................AAAU...........**G****C****U****U****U****G**.AG.**A**..**G****A**..**U****G****A****G****A****G****A****G****A**GGGA | |
|  |  | NZ\_AAEQ01000025.1/29364-29485  | CUGA**U****U****U****C****U****C****U****U**.**A****U****C**A.AGA**G**.**A****G****G****U**.**G****G****A****G****G****G**A...**C****U****G**U**G**.**C****C****C**UGU**G****A****A**.**G****C****C**-.**C****G****G****C**AA**C****C**.**G**.-**U****C****A****A**C.......................UUAU-...................................................G**U****U****G****A****A**.A...**U**..**G****G**U**G**..**C****C**AAU**U****C****C****U****G**...**C****A****A****A****G****C**....................AAAU...........**G****C****U****U****U****G**.AG.**A**..**G****A**..**U****G****A****G****A****G****A****G****A**GGGA | |
|  |  | NZ\_AAER01000029.1/29365-29486  | CUGA**U****U****U****C****U****C****U****U**.**A****U****C**A.AGA**G**.**A****G****G****U**.**G****G****A****G****G****G**A...**C****U****G**U**G**.**C****C****C**UGU**G****A****A**.**G****C****C**-.**C****G****G****C**AA**C****C**.**G**.-**U****C****A****A**C.......................UUAU-...................................................G**U****U****G****A****A**.A...**U**..**G****G**U**G**..**C****C**AAU**U****C****C****U****G**...**C****A****A****A****G****C**....................AAAU...........**G****C****U****U****U****G**.AG.**A**..**G****A**..**U****G****A****G****A****G****A****G****A**GGGA | |
|  |  | NZ\_AAES01000037.1/86176-86055  | CUGA**U****U****U****C****U****C****U****U**.**A****U****C**A.AGA**G**.**A****G****G****U**.**G****G****A****G****G****G**A...**C****U****G**U**G**.**C****C****C**UGU**G****A****A**.**G****C****C**-.**C****G****G****C**AA**C****C**.**G**.-**U****C****A****A**C.......................UUAU-...................................................G**U****U****G****A****A**.A...**U**..**G****G**U**G**..**C****C**AAU**U****C****C****U****G**...**C****A****A****A****G****C**....................AAAU...........**G****C****U****U****U****G**.AG.**A**..**G****A**..**U****G****A****G****A****G****A****G****A**GGGA | |
|  |  | NC\_002570.2/3590828-3590702  | AAGA**A****A****A****C****U****C****U****U**.**A****U****C**A.UGA**G**.**A****G****G****U**.**G****G****A****G****G****G**A...**C****U****G**.**G**.**C****C****C**GAU**G****A****A**.**G****C****C**-.**C****A****G****C**AA**C****C**.**G**.--**C****C****A**AGCAG...................CAAAU................................................CGCU**U****G****G****A****A**.A...**A**..**G****G**U**G**..**C****U**AAU**U****C****C****U****G**...**C****A****A****A****G****C**....................-GAU...........**G****C****U****U****U****G**.AG.**A**..**G****A**..**U****G****A****G****A****G****A****A****G**GGAA | |
|  |  | NC\_003997.3/177281-177153  | ACAC**A****U****A****C****U****C****U****U**.**A****U****C**A.AGA**G**.**U****G****G****C**.**G****G****A****G****G****G**A...**C****U****G**.**G**.**C****C****C**GAU**G****A****U**.**G****C****C**-.**C****G****G****C**AA**C****C**.**G**.**A****G****C****U****U**AUGAC...................GUAU-....................................................**A****A****G****C****U**.A...**A**..**G****G**U**G**..**C****U**AAU**U****C****C****U****G**...**C****A****A****A****A****U**GAG.................UUUU..........C**G****U****U****U****U****G**.GA.**A**..**G****A**..**U****A****A****G****A****G****A****G****G**AUCC | |
|  |  | NC\_005945.1/177282-177154  | ACAC**A****U****A****C****U****C****U****U**.**A****U****C**A.AGA**G**.**U****G****G****C**.**G****G****A****G****G****G**A...**C****U****G**.**G**.**C****C****C**GAU**G****A****U**.**G****C****C**-.**C****G****G****C**AA**C****C**.**G**.**A****G****C****U****U**AUGAC...................GUAU-....................................................**A****A****G****C****U**.A...**A**..**G****G**U**G**..**C****U**AAU**U****C****C****U****G**...**C****A****A****A****A****U**GAG.................UUUU..........C**G****U****U****U****U****G**.GA.**A**..**G****A**..**U****A****A****G****A****G****A****G****G**AUCC | |
|  |  | NC\_007530.2/177281-177153  | ACAC**A****U****A****C****U****C****U****U**.**A****U****C**A.AGA**G**.**U****G****G****C**.**G****G****A****G****G****G**A...**C****U****G**.**G**.**C****C****C**GAU**G****A****U**.**G****C****C**-.**C****G****G****C**AA**C****C**.**G**.**A****G****C****U****U**AUGAC...................GUAU-....................................................**A****A****G****C****U**.A...**A**..**G****G**U**G**..**C****U**AAU**U****C****C****U****G**...**C****A****A****A****A****U**GAG.................UUUU..........C**G****U****U****U****U****G**.GA.**A**..**G****A**..**U****A****A****G****A****G****A****G****G**AUCC | |
|  |  | NZ\_AAEN01000019.1/29097-28969  | ACAC**A****U****A****C****U****C****U****U**.**A****U****C**A.AGA**G**.**U****G****G****C**.**G****G****A****G****G****G**A...**C****U****G**.**G**.**C****C****C**GAU**G****A****U**.**G****C****C**-.**C****G****G****C**AA**C****C**.**G**.**A****G****C****U****U**AUGAC...................GUAU-....................................................**A****A****G****C****U**.A...**A**..**G****G**U**G**..**C****U**AAU**U****C****C****U****G**...**C****A****A****A****A****U**GAG.................UUUU..........C**G****U****U****U****U****G**.GA.**A**..**G****A**..**U****A****A****G****A****G****A****G****G**AUCC | |
|  |  | NZ\_AAEO01000023.1/68675-68803  | ACAC**A****U****A****C****U****C****U****U**.**A****U****C**A.AGA**G**.**U****G****G****C**.**G****G****A****G****G****G**A...**C****U****G**.**G**.**C****C****C**GAU**G****A****U**.**G****C****C**-.**C****G****G****C**AA**C****C**.**G**.**A****G****C****U****U**AUGAC...................GUAU-....................................................**A****A****G****C****U**.A...**A**..**G****G**U**G**..**C****U**AAU**U****C****C****U****G**...**C****A****A****A****A****U**GAG.................UUUU..........C**G****U****U****U****U****G**.GA.**A**..**G****A**..**U****A****A****G****A****G****A****G****G**AUCC | |
|  |  | NZ\_AAEP01000033.1/26678-26550  | ACAC**A****U****A****C****U****C****U****U**.**A****U****C**A.AGA**G**.**U****G****G****C**.**G****G****A****G****G****G**A...**C****U****G**.**G**.**C****C****C**GAU**G****A****U**.**G****C****C**-.**C****G****G****C**AA**C****C**.**G**.**A****G****C****U****U**AUGAC...................GUAU-....................................................**A****A****G****C****U**.A...**A**..**G****G**U**G**..**C****U**AAU**U****C****C****U****G**...**C****A****A****A****A****U**GAG.................UUUU..........C**G****U****U****U****U****G**.GA.**A**..**G****A**..**U****A****A****G****A****G****A****G****G**AUCC | |
|  |  | NZ\_AAEQ01000020.1/67580-67708  | ACAC**A****U****A****C****U****C****U****U**.**A****U****C**A.AGA**G**.**U****G****G****C**.**G****G****A****G****G****G**A...**C****U****G**.**G**.**C****C****C**GAU**G****A****U**.**G****C****C**-.**C****G****G****C**AA**C****C**.**G**.**A****G****C****U****U**AUGAC...................GUAU-....................................................**A****A****G****C****U**.A...**A**..**G****G**U**G**..**C****U**AAU**U****C****C****U****G**...**C****A****A****A****A****U**GAG.................UUUU..........C**G****U****U****U****U****G**.GA.**A**..**G****A**..**U****A****A****G****A****G****A****G****G**AUCC | |
|  |  | NZ\_AAER01000024.1/68677-68805  | ACAC**A****U****A****C****U****C****U****U**.**A****U****C**A.AGA**G**.**U****G****G****C**.**G****G****A****G****G****G**A...**C****U****G**.**G**.**C****C****C**GAU**G****A****U**.**G****C****C**-.**C****G****G****C**AA**C****C**.**G**.**A****G****C****U****U**AUGAC...................GUAU-....................................................**A****A****G****C****U**.A...**A**..**G****G**U**G**..**C****U**AAU**U****C****C****U****G**...**C****A****A****A****A****U**GAG.................UUUU..........C**G****U****U****U****U****G**.GA.**A**..**G****A**..**U****A****A****G****A****G****A****G****G**AUCC | |
|  |  | NZ\_AAES01000019.1/27683-27555  | ACAC**A****U****A****C****U****C****U****U**.**A****U****C**A.AGA**G**.**U****G****G****C**.**G****G****A****G****G****G**A...**C****U****G**.**G**.**C****C****C**GAU**G****A****U**.**G****C****C**-.**C****G****G****C**AA**C****C**.**G**.**A****G****C****U****U**AUGAC...................GUAU-....................................................**A****A****G****C****U**.A...**A**..**G****G**U**G**..**C****U**AAU**U****C****C****U****G**...**C****A****A****A****A****U**GAG.................UUUU..........C**G****U****U****U****U****G**.GA.**A**..**G****A**..**U****A****A****G****A****G****A****G****G**AUCC | |
|  |  | NC\_003909.8/4022849-4022972  | ACGA**A****C****A****U****U****C****U****U**.**A****U****C**U.AGA**G**.**A****G****G****U**.**A****G****A****G****G****G**A...**C****U****G**.**G**.**C****C****C**UAU**G****A****C**.**G****C****C****U**.**C****A****G****C**AA**C****C**.-.**A****U****U****A****A**C.......................AUUU-...................................................G**U****U****A****A****U**.A...**A**..**G****G**U**G**..**C****U**AAU**U****C****C****A****G**...**C****A****A****A****U****U**G...................UGAA..........A**G****A****U****U****U****G**.AC.**A**..**G****A**..**U****G****A****G****A****A****G****A****A**GACU | |
|  |  | NC\_005957.1/4062233-4062356  | ACGA**A****C****A****U****U****C****U****U**.**A****U****C**U.AGA**G**.**A****G****G****U**.**A****G****A****G****G****G**A...**C****U****G**.**G**.**C****C****C**UAU**G****A****C**.**G****C****C****U**.**C****A****G****C**AA**C****C**.-.**A****U****U****A****A**C.......................AUUU-...................................................G**U****U****A****A****U**.A...**A**..**G****G**U**G**..**C****U**AAU**U****C****C****A****G**...**C****A****A****A****U****U**G...................UGAA..........A**G****A****U****U****U****G**.AC.**A**..**G****A**..**U****G****A****G****A****A****G****A****A**GACU | |
|  |  | NC\_006274.1/4116460-4116583  | ACGA**A****C****A****U****U****C****U****U**.**A****U****C**U.AGA**G**.**A****G****G****U**.**A****G****A****G****G****G**A...**C****U****G**.**G**.**C****C****C**UAU**G****A****C**.**G****C****C****U**.**C****A****G****C**AA**C****C**.-.**A****U****U****A****A**C.......................AUUU-...................................................G**U****U****A****A****U**.A...**A**..**G****G**U**G**..**C****U**AAU**U****C****C****A****G**...**C****A****A****A****U****U**G...................UGAA..........A**G****A****U****U****U****G**.AC.**A**..**G****A**..**U****G****A****G****A****A****G****A****A**GACU | |
|  |  | NZ\_AAEK01000021.1/52446-52323  | ACGA**A****C****A****U****U****C****U****U**.**A****U****C**U.AGA**G**.**A****G****G****U**.**A****G****A****G****G****G**A...**C****U****G**.**G**.**C****C****C**UAU**G****A****C**.**G****C****C****U**.**C****A****G****C**AA**C****C**.-.**A****U****U****A****A**C.......................AUUU-...................................................G**U****U****A****A****U**.A...**A**..**G****G**U**G**..**C****U**AAU**U****C****C****A****G**...**C****A****A****A****U****U**G...................UGAA..........C**G****A****U****U****U****G**.AC.**A**..**G****A**..**U****G****A****G****A****A****G****A****A**GACU | |
|  |  | NC\_003997.3/320598-320718  | CGAU**A****C****A****U****U****C****U****U**.**A****U****C**C.AGA**G**.**A****G****G****U**.**G****G****A****G****G****G**A...**C****U****G**.**G**.**C****C****C**UAC**G****A****U**.**A****C****C****U**.**C****A****G****C**AA**C****G**.**G**.**G****U****U**--........................UUUUU....................................................--**A****A****U**.A...**C**..**C****G**U**G**..**C****U**AAC**U****C****C****A****G**...**C****A****A****G****C****C**AU..................AUAA..........A**G****G****C****U****U****G**.GA.**A**..**G****A**..**U****G****A****G****A****A****G****A****U**GUGA | |
|  |  | NC\_004722.1/324051-324171  | CGAU**A****C****A****U****U****C****U****U**.**A****U****C**C.AGA**G**.**A****G****G****U**.**G****G****A****G****G****G**A...**C****U****G**.**G**.**C****C****C**UAC**G****A****U**.**A****C****C****U**.**C****A****G****C**AA**C****G**.**G**.**G****U****U**--........................UUUUU....................................................--**A****A****U**.A...**C**..**C****G**U**G**..**C****U**AAC**U****C****C****A****G**...**C****A****A****G****C****C**AU..................AUAA..........A**G****G****C****U****U****G**.GA.**A**..**G****A**..**U****G****A****G****A****A****G****A****U**GUGA | |
|  |  | NC\_005945.1/320611-320731  | CGAU**A****C****A****U****U****C****U****U**.**A****U****C**C.AGA**G**.**A****G****G****U**.**G****G****A****G****G****G**A...**C****U****G**.**G**.**C****C****C**UAC**G****A****U**.**A****C****C****U**.**C****A****G****C**AA**C****G**.**G**.**G****U****U**--........................UUUUU....................................................--**A****A****U**.A...**C**..**C****G**U**G**..**C****U**AAC**U****C****C****A****G**...**C****A****A****G****C****C**AU..................AUAA..........A**G****G****C****U****U****G**.GA.**A**..**G****A**..**U****G****A****G****A****A****G****A****U**GUGA | |
|  |  | NC\_005957.1/334799-334919  | CGAU**A****C****A****U****U****C****U****U**.**A****U****C**C.AGA**G**.**A****G****G****U**.**G****G****A****G****G****G**A...**C****U****G**.**G**.**C****C****C**UAC**G****A****U**.**A****C****C****U**.**C****A****G****C**AA**C****G**.**G**.**G****U****U**--........................UUUUU....................................................--**A****A****U**.A...**C**..**C****G**U**G**..**C****U**AAC**U****C****C****A****G**...**C****A****A****G****C****C**AU..................AUAA..........A**G****G****C****U****U****G**.GA.**A**..**G****A**..**U****G****A****G****A****A****G****A****U**GUGA | |
|  |  | NC\_007530.2/320598-320718  | CGAU**A****C****A****U****U****C****U****U**.**A****U****C**C.AGA**G**.**A****G****G****U**.**G****G****A****G****G****G**A...**C****U****G**.**G**.**C****C****C**UAC**G****A****U**.**A****C****C****U**.**C****A****G****C**AA**C****G**.**G**.**G****U****U**--........................UUUUU....................................................--**A****A****U**.A...**C**..**C****G**U**G**..**C****U**AAC**U****C****C****A****G**...**C****A****A****G****C****C**AU..................AUAA..........A**G****G****C****U****U****G**.GA.**A**..**G****A**..**U****G****A****G****A****A****G****A****U**GUGA | |
|  |  | NZ\_AAAC02000001.1/837510-837630  | CGAU**A****C****A****U****U****C****U****U**.**A****U****C**C.AGA**G**.**A****G****G****U**.**G****G****A****G****G****G**A...**C****U****G**.**G**.**C****C****C**UAC**G****A****U**.**A****C****C****U**.**C****A****G****C**AA**C****G**.**G**.**G****U****U**--........................UUUUU....................................................--**A****A****U**.A...**C**..**C****G**U**G**..**C****U**AAC**U****C****C****A****G**...**C****A****A****G****C****C**AU..................AUAA..........A**G****G****C****U****U****G**.GA.**A**..**G****A**..**U****G****A****G****A****A****G****A****U**GUGA | |
|  |  | NZ\_AAEN01000023.1/61682-61802  | CGAU**A****C****A****U****U****C****U****U**.**A****U****C**C.AGA**G**.**A****G****G****U**.**G****G****A****G****G****G**A...**C****U****G**.**G**.**C****C****C**UAC**G****A****U**.**A****C****C****U**.**C****A****G****C**AA**C****G**.**G**.**G****U****U**--........................UUUUU....................................................--**A****A****U**.A...**C**..**C****G**U**G**..**C****U**AAC**U****C****C****A****G**...**C****A****A****G****C****C**AU..................AUAA..........A**G****G****C****U****U****G**.GA.**A**..**G****A**..**U****G****A****G****A****A****G****A****U**GUGA | |
|  |  | NZ\_AAEP01000046.1/27052-27172  | CGAU**A****C****A****U****U****C****U****U**.**A****U****C**C.AGA**G**.**A****G****G****U**.**G****G****A****G****G****G**A...**C****U****G**.**G**.**C****C****C**UAC**G****A****U**.**A****C****C****U**.**C****A****G****C**AA**C****G**.**G**.**G****U****U**--........................UUUUU....................................................--**A****A****U**.A...**C**..**C****G**U**G**..**C****U**AAC**U****C****C****A****G**...**C****A****A****G****C****C**AU..................AUAA..........A**G****G****C****U****U****G**.GA.**A**..**G****A**..**U****G****A****G****A****A****G****A****U**GUGA | |
|  |  | NZ\_AAEQ01000034.1/27725-27845  | CGAU**A****C****A****U****U****C****U****U**.**A****U****C**C.AGA**G**.**A****G****G****U**.**G****G****A****G****G****G**A...**C****U****G**.**G**.**C****C****C**UAC**G****A****U**.**A****C****C****U**.**C****A****G****C**AA**C****G**.**G**.**G****U****U**--........................UUUUU....................................................--**A****A****U**.A...**C**..**C****G**U**G**..**C****U**AAC**U****C****C****A****G**...**C****A****A****G****C****C**AU..................AUAA..........A**G****G****C****U****U****G**.GA.**A**..**G****A**..**U****G****A****G****A****A****G****A****U**GUGA | |
|  |  | NZ\_AAER01000042.1/213097-212977  | CGAU**A****C****A****U****U****C****U****U**.**A****U****C**C.AGA**G**.**A****G****G****U**.**G****G****A****G****G****G**A...**C****U****G**.**G**.**C****C****C**UAC**G****A****U**.**A****C****C****U**.**C****A****G****C**AA**C****G**.**G**.**G****U****U**--........................UUUUU....................................................--**A****A****U**.A...**C**..**C****G**U**G**..**C****U**AAC**U****C****C****A****G**...**C****A****A****G****C****C**AU..................AUAA..........A**G****G****C****U****U****G**.GA.**A**..**G****A**..**U****G****A****G****A****A****G****A****U**GUGA | |
|  |  | NZ\_AAES01000043.1/26756-26876  | CGAU**A****C****A****U****U****C****U****U**.**A****U****C**C.AGA**G**.**A****G****G****U**.**G****G****A****G****G****G**A...**C****U****G**.**G**.**C****C****C**UAC**G****A****U**.**A****C****C****U**.**C****A****G****C**AA**C****G**.**G**.**G****U****U**--........................UUUUU....................................................--**A****A****U**.A...**C**..**C****G**U**G**..**C****U**AAC**U****C****C****A****G**...**C****A****A****G****C****C**AU..................AUAA..........A**G****G****C****U****U****G**.GA.**A**..**G****A**..**U****G****A****G****A****A****G****A****U**GUGA | |
|  |  | NC\_003997.3/4074276-4074399  | ACGA**A****C****A****U****U****C****U****U**.**A****U****C**U.AGA**G**.**A****G****G****U**.**A****G****A****G****G****G**A...**C****U****G**.**G**.**C****C****C**UAU**G****A****C**.**G****C****C****U**.**C****A****G****C**AA**C****C**.-.**A****U****U****A****A**C.......................AUUU-...................................................G**U****U****A****A****U**.A...**A**..**G****G**U**G**..**C****U**AAU**U****C****C****A****G**...**C****A****A****A****U****U**G...................CGAA..........A**A****A****U****U****U****G**.AC.**A**..**G****A**..**U****G****A****G****A****A****G****A****A**GACU | |
|  |  | NC\_005945.1/4074776-4074899  | ACGA**A****C****A****U****U****C****U****U**.**A****U****C**U.AGA**G**.**A****G****G****U**.**A****G****A****G****G****G**A...**C****U****G**.**G**.**C****C****C**UAU**G****A****C**.**G****C****C****U**.**C****A****G****C**AA**C****C**.-.**A****U****U****A****A**C.......................AUUU-...................................................G**U****U****A****A****U**.A...**A**..**G****G**U**G**..**C****U**AAU**U****C****C****A****G**...**C****A****A****A****U****U**G...................CGAA..........A**A****A****U****U****U****G**.AC.**A**..**G****A**..**U****G****A****G****A****A****G****A****A**GACU | |
|  |  | NC\_007530.2/4074403-4074526  | ACGA**A****C****A****U****U****C****U****U**.**A****U****C**U.AGA**G**.**A****G****G****U**.**A****G****A****G****G****G**A...**C****U****G**.**G**.**C****C****C**UAU**G****A****C**.**G****C****C****U**.**C****A****G****C**AA**C****C**.-.**A****U****U****A****A**C.......................AUUU-...................................................G**U****U****A****A****U**.A...**A**..**G****G**U**G**..**C****U**AAU**U****C****C****A****G**...**C****A****A****A****U****U**G...................CGAA..........A**A****A****U****U****U****G**.AC.**A**..**G****A**..**U****G****A****G****A****A****G****A****A**GACU | |
|  |  | NZ\_AAAC02000001.1/4520037-4520160  | ACGA**A****C****A****U****U****C****U****U**.**A****U****C**U.AGA**G**.**A****G****G****U**.**A****G****A****G****G****G**A...**C****U****G**.**G**.**C****C****C**UAU**G****A****C**.**G****C****C****U**.**C****A****G****C**AA**C****C**.-.**A****U****U****A****A**C.......................AUUU-...................................................G**U****U****A****A****U**.A...**A**..**G****G**U**G**..**C****U**AAU**U****C****C****A****G**...**C****A****A****A****U****U**G...................CGAA..........A**A****A****U****U****U****G**.AC.**A**..**G****A**..**U****G****A****G****A****A****G****A****A**GACU | |
|  |  | NZ\_AAEN01000013.1/328785-328908  | ACGA**A****C****A****U****U****C****U****U**.**A****U****C**U.AGA**G**.**A****G****G****U**.**A****G****A****G****G****G**A...**C****U****G**.**G**.**C****C****C**UAU**G****A****C**.**G****C****C****U**.**C****A****G****C**AA**C****C**.-.**A****U****U****A****A**C.......................AUUU-...................................................G**U****U****A****A****U**.A...**A**..**G****G**U**G**..**C****U**AAU**U****C****C****A****G**...**C****A****A****A****U****U**G...................CGAA..........A**A****A****U****U****U****G**.AC.**A**..**G****A**..**U****G****A****G****A****A****G****A****A**GACU | |
|  |  | NZ\_AAEO01000019.1/415878-416001  | ACGA**A****C****A****U****U****C****U****U**.**A****U****C**U.AGA**G**.**A****G****G****U**.**A****G****A****G****G****G**A...**C****U****G**.**G**.**C****C****C**UAU**G****A****C**.**G****C****C****U**.**C****A****G****C**AA**C****C**.-.**A****U****U****A****A**C.......................AUUU-...................................................G**U****U****A****A****U**.A...**A**..**G****G**U**G**..**C****U**AAU**U****C****C****A****G**...**C****A****A****A****U****U**G...................CGAA..........A**A****A****U****U****U****G**.AC.**A**..**G****A**..**U****G****A****G****A****A****G****A****A**GACU | |
|  |  | NZ\_AAEP01000031.1/31192-31069  | ACGA**A****C****A****U****U****C****U****U**.**A****U****C**U.AGA**G**.**A****G****G****U**.**A****G****A****G****G****G**A...**C****U****G**.**G**.**C****C****C**UAU**G****A****C**.**G****C****C****U**.**C****A****G****C**AA**C****C**.-.**A****U****U****A****A**C.......................AUUU-...................................................G**U****U****A****A****U**.A...**A**..**G****G**U**G**..**C****U**AAU**U****C****C****A****G**...**C****A****A****A****U****U**G...................CGAA..........A**A****A****U****U****U****G**.AC.**A**..**G****A**..**U****G****A****G****A****A****G****A****A**GACU | |
|  |  | NZ\_AAEQ01000030.1/63312-63435  | ACGA**A****C****A****U****U****C****U****U**.**A****U****C**U.AGA**G**.**A****G****G****U**.**A****G****A****G****G****G**A...**C****U****G**.**G**.**C****C****C**UAU**G****A****C**.**G****C****C****U**.**C****A****G****C**AA**C****C**.-.**A****U****U****A****A**C.......................AUUU-...................................................G**U****U****A****A****U**.A...**A**..**G****G**U**G**..**C****U**AAU**U****C****C****A****G**...**C****A****A****A****U****U**G...................CGAA..........A**A****A****U****U****U****G**.AC.**A**..**G****A**..**U****G****A****G****A****A****G****A****A**GACU | |
|  |  | NZ\_AAER01000035.1/587122-587245  | ACGA**A****C****A****U****U****C****U****U**.**A****U****C**U.AGA**G**.**A****G****G****U**.**A****G****A****G****G****G**A...**C****U****G**.**G**.**C****C****C**UAU**G****A****C**.**G****C****C****U**.**C****A****G****C**AA**C****C**.-.**A****U****U****A****A**C.......................AUUU-...................................................G**U****U****A****A****U**.A...**A**..**G****G**U**G**..**C****U**AAU**U****C****C****A****G**...**C****A****A****A****U****U**G...................CGAA..........A**A****A****U****U****U****G**.AC.**A**..**G****A**..**U****G****A****G****A****A****G****A****A**GACU | |
|  |  | NZ\_AAES01000022.1/578982-578859  | ACGA**A****C****A****U****U****C****U****U**.**A****U****C**U.AGA**G**.**A****G****G****U**.**A****G****A****G****G****G**A...**C****U****G**.**G**.**C****C****C**UAU**G****A****C**.**G****C****C****U**.**C****A****G****C**AA**C****C**.-.**A****U****U****A****A**C.......................AUUU-...................................................G**U****U****A****A****U**.A...**A**..**G****G**U**G**..**C****U**AAU**U****C****C****A****G**...**C****A****A****A****U****U**G...................CGAA..........A**A****A****U****U****U****G**.AC.**A**..**G****A**..**U****G****A****G****A****A****G****A****A**GACU | |
|  |  | NC\_004722.1/4199707-4199831  | ACGA**A****C****A****U****U****C****U****U**.**A****U****C**U.AGA**G**.**A****G****G****U**.**A****G****A****G****G****G**A...**C****U****G**.**G**.**C****C****C**UGU**G****A****C**.**G****C****C****U**.**C****A****G****C**AA**C****C**.-.**A****U****U****A****A**C.......................AUUUU...................................................G**U****U****A****A****U**.A...**A**..**G****G**U**G**..**C****U**AAU**U****C****C****A****G**...**C****A****A****A****U****U**G...................UGAA..........A**G****A****U****U****U****G**.AC.**A**..**G****A**..**U****G****A****G****A****A****G****A****A**GACU | |
|  |  | NC\_003909.8/1518510-1518632  | AAUA**C****A****A****A****G****C****U****U**.**A****U****C**A.AGA**G**.**A****A****G****C**.**G****G****A****G****G****G**AA..**C****U****G**.**G**.**C****C****C**GGC**G****A****A**.**G****C****U**-.**C****G****G****C**AA**C****C**.**U**.**G****C****U****U**-........................AUAGA....................................................-**A****A****G****C**.A...**A**..**G****G**U**G**..**C****U**AAA**U****C****C****A****G**...**C****A****A****A****A****U**G...................GAAU.........CC**A****U****U****U****U****G**.AA.**A**..**G****A**..**U****A****A****G****G****U****A****A****A**AUAU | |
|  |  | NC\_003997.3/1362650-1362772  | AAUA**C****A****A****A****G****C****U****U**.**A****U****C**A.AGA**G**.**A****A****G****C**.**G****G****A****G****G****G**AA..**C****U****G**.**G**.**C****C****C**GGC**G****A****A**.**G****C****U**-.**C****G****G****C**AA**C****C**.**U**.**G****C****U****U**-........................AUAGA....................................................-**A****A****G****C**.A...**A**..**G****G**U**G**..**C****U**AAA**U****C****C****A****G**...**C****A****A****A****A****U**G...................GAAU.........CC**A****U****U****U****U****G**.AA.**A**..**G****A**..**U****A****A****G****G****U****A****A****A**AUAU | |
|  |  | NC\_005945.1/1362719-1362841  | AAUA**C****A****A****A****G****C****U****U**.**A****U****C**A.AGA**G**.**A****A****G****C**.**G****G****A****G****G****G**AA..**C****U****G**.**G**.**C****C****C**GGC**G****A****A**.**G****C****U**-.**C****G****G****C**AA**C****C**.**U**.**G****C****U****U**-........................AUAGA....................................................-**A****A****G****C**.A...**A**..**G****G**U**G**..**C****U**AAA**U****C****C****A****G**...**C****A****A****A****A****U**G...................GAAU.........CC**A****U****U****U****U****G**.AA.**A**..**G****A**..**U****A****A****G****G****U****A****A****A**AUAU | |
|  |  | NC\_005957.1/1384692-1384814  | AAUA**C****A****A****A****G****C****U****U**.**A****U****C**A.AGA**G**.**A****A****G****C**.**G****G****A****G****G****G**AA..**C****U****G**.**G**.**C****C****C**GGC**G****A****A**.**G****C****U**-.**C****G****G****C**AA**C****C**.**U**.**G****C****U****U**-........................AUAGA....................................................-**A****A****G****C**.A...**A**..**G****G**U**G**..**C****U**AAA**U****C****C****A****G**...**C****A****A****A****A****U**G...................GAAU.........CC**A****U****U****U****U****G**.AA.**A**..**G****A**..**U****A****A****G****G****U****A****A****A**AUAU | |
|  |  | NC\_007530.2/1362773-1362895  | AAUA**C****A****A****A****G****C****U****U**.**A****U****C**A.AGA**G**.**A****A****G****C**.**G****G****A****G****G****G**AA..**C****U****G**.**G**.**C****C****C**GGC**G****A****A**.**G****C****U**-.**C****G****G****C**AA**C****C**.**U**.**G****C****U****U**-........................AUAGA....................................................-**A****A****G****C**.A...**A**..**G****G**U**G**..**C****U**AAA**U****C****C****A****G**...**C****A****A****A****A****U**G...................GAAU.........CC**A****U****U****U****U****G**.AA.**A**..**G****A**..**U****A****A****G****G****U****A****A****A**AUAU | |
|  |  | NZ\_AAAC02000001.1/1854747-1854869  | AAUA**C****A****A****A****G****C****U****U**.**A****U****C**A.AGA**G**.**A****A****G****C**.**G****G****A****G****G****G**AA..**C****U****G**.**G**.**C****C****C**GGC**G****A****A**.**G****C****U**-.**C****G****G****C**AA**C****C**.**U**.**G****C****U****U**-........................AUAGA....................................................-**A****A****G****C**.A...**A**..**G****G**U**G**..**C****U**AAA**U****C****C****A****G**...**C****A****A****A****A****U**G...................GAAU.........CC**A****U****U****U****U****G**.AA.**A**..**G****A**..**U****A****A****G****G****U****A****A****A**AUAU | |
|  |  | NZ\_AAEK01000009.1/10305-10183  | AAUA**C****A****A****A****G****C****U****U**.**A****U****C**A.AGA**G**.**A****A****G****C**.**G****G****A****G****G****G**AA..**C****U****G**.**G**.**C****C****C**GGC**G****A****A**.**G****C****U**-.**C****G****G****C**AA**C****C**.**U**.**G****C****U****U**-........................AUAGA....................................................-**A****A****G****C**.A...**A**..**G****G**U**G**..**C****U**AAA**U****C****C****A****G**...**C****A****A****A****A****U**G...................GAAU.........CC**A****U****U****U****U****G**.AA.**A**..**G****A**..**U****A****A****G****G****U****A****A****A**AUAU | |
|  |  | NZ\_AAEN01000008.1/61362-61240  | AAUA**C****A****A****A****G****C****U****U**.**A****U****C**A.AGA**G**.**A****A****G****C**.**G****G****A****G****G****G**AA..**C****U****G**.**G**.**C****C****C**GGC**G****A****A**.**G****C****U**-.**C****G****G****C**AA**C****C**.**U**.**G****C****U****U**-........................AUAGA....................................................-**A****A****G****C**.A...**A**..**G****G**U**G**..**C****U**AAA**U****C****C****A****G**...**C****A****A****A****A****U**G...................GAAU.........CC**A****U****U****U****U****G**.AA.**A**..**G****A**..**U****A****A****G****G****U****A****A****A**AUAU | |
|  |  | NZ\_AAEO01000037.1/10237-10115  | AAUA**C****A****A****A****G****C****U****U**.**A****U****C**A.AGA**G**.**A****A****G****C**.**G****G****A****G****G****G**AA..**C****U****G**.**G**.**C****C****C**GGC**G****A****A**.**G****C****U**-.**C****G****G****C**AA**C****C**.**U**.**G****C****U****U**-........................AUAGA....................................................-**A****A****G****C**.A...**A**..**G****G**U**G**..**C****U**AAA**U****C****C****A****G**...**C****A****A****A****A****U**G...................GAAU.........CC**A****U****U****U****U****G**.AA.**A**..**G****A**..**U****A****A****G****G****U****A****A****A**AUAU | |
|  |  | NZ\_AAEP01000030.1/168681-168803  | AAUA**C****A****A****A****G****C****U****U**.**A****U****C**A.AGA**G**.**A****A****G****C**.**G****G****A****G****G****G**AA..**C****U****G**.**G**.**C****C****C**GGC**G****A****A**.**G****C****U**-.**C****G****G****C**AA**C****C**.**U**.**G****C****U****U**-........................AUAGA....................................................-**A****A****G****C**.A...**A**..**G****G**U**G**..**C****U**AAA**U****C****C****A****G**...**C****A****A****A****A****U**G...................GAAU.........CC**A****U****U****U****U****G**.AA.**A**..**G****A**..**U****A****A****G****G****U****A****A****A**AUAU | |
|  |  | NZ\_AAEQ01000023.1/168941-169063  | AAUA**C****A****A****A****G****C****U****U**.**A****U****C**A.AGA**G**.**A****A****G****C**.**G****G****A****G****G****G**AA..**C****U****G**.**G**.**C****C****C**GGC**G****A****A**.**G****C****U**-.**C****G****G****C**AA**C****C**.**U**.**G****C****U****U**-........................AUAGA....................................................-**A****A****G****C**.A...**A**..**G****G**U**G**..**C****U**AAA**U****C****C****A****G**...**C****A****A****A****A****U**G...................GAAU.........CC**A****U****U****U****U****G**.AA.**A**..**G****A**..**U****A****A****G****G****U****A****A****A**AUAU | |
|  |  | NZ\_AAER01000019.1/61164-61042  | AAUA**C****A****A****A****G****C****U****U**.**A****U****C**A.AGA**G**.**A****A****G****C**.**G****G****A****G****G****G**AA..**C****U****G**.**G**.**C****C****C**GGC**G****A****A**.**G****C****U**-.**C****G****G****C**AA**C****C**.**U**.**G****C****U****U**-........................AUAGA....................................................-**A****A****G****C**.A...**A**..**G****G**U**G**..**C****U**AAA**U****C****C****A****G**...**C****A****A****A****A****U**G...................GAAU.........CC**A****U****U****U****U****G**.AA.**A**..**G****A**..**U****A****A****G****G****U****A****A****A**AUAU | |
|  |  | NZ\_AAES01000029.1/168911-169033  | AAUA**C****A****A****A****G****C****U****U**.**A****U****C**A.AGA**G**.**A****A****G****C**.**G****G****A****G****G****G**AA..**C****U****G**.**G**.**C****C****C**GGC**G****A****A**.**G****C****U**-.**C****G****G****C**AA**C****C**.**U**.**G****C****U****U**-........................AUAGA....................................................-**A****A****G****C**.A...**A**..**G****G**U**G**..**C****U**AAA**U****C****C****A****G**...**C****A****A****A****A****U**G...................GAAU.........CC**A****U****U****U****U****G**.AA.**A**..**G****A**..**U****A****A****G****G****U****A****A****A**AUAU | |
|  |  | NC\_006274.1/334363-334481  | CGAU**A****C****A****U****U****C****U****U**.**A****U****C**C.AGA**G**.**A****G****G****U**.**G****G****A****G****G****G**A...**C****U****G**.**G**.**C****C****C**UAC**G****A****U**.**A****C****C****U**.**C****A****G****C**AA**C****G**.**G**.**G****U****U**--........................UUUU-....................................................--**A****A****U**.A...**C**..**C****G**U**G**..**C****U**AAC**U****C****C****A****G**...**C****A****A****G****C****C**U...................AUAA..........G**G****G****C****U****U****G**.GA.**A**..**G****A**..**U****G****A****G****A****A****G****A****U**GUGA | |
|  |  | NZ\_AAEK01000051.1/29982-29864  | CGAU**A****C****A****U****U****C****U****U**.**A****U****C**C.AGA**G**.**A****G****G****U**.**G****G****A****G****G****G**A...**C****U****G**.**G**.**C****C****C**UAC**G****A****U**.**A****C****C****U**.**C****A****G****C**AA**C****G**.**G**.**G****U****U**--........................UUUU-....................................................--**A****A****U**.A...**C**..**C****G**U**G**..**C****U**AAC**U****C****C****A****G**...**C****A****A****G****C****C**U...................AUAA..........G**G****G****C****U****U****G**.GA.**A**..**G****A**..**U****G****A****G****A****A****G****A****U**GUGA | |
|  |  | NC\_003030.1/2914848-2914726  | UGAU**A****A****G****G****U****C****U****U**.**A****U****C**A.AGA**G**.**A****G****G****U**.**G****G****A****G****G****G**A...**C****U****G**.**G**.**C****C****C**UAU**G****A****A**.**A****C****C**-.**C****A****A****C**AA**C****C**.**A**.**G****C****A****U****U**U.......................UUUAA.................................................UUA**G****A****U****G****U**.A...**U**..**G****G**U**G**..**U****U**AAU**U****C****C****U****G**...**C****A****A****A****G**-....................UUAA...........-**U****U****U****U****G**.AG.**A**..**G****A**..**U****A****A****G****A****G****G****A****U**UAUA | |
|  |  | NC\_003869.1/2076689-2076566  | CUCA**A****U****C****C****U****C****U****U**.**A****U****C**A.AGA**G**.**U****G****G****U**.**G****G****A****G****G****G**A...**C****U****G**.**G**.**C****C****C**GAU**G****A****A**.**A****C****C**-.**C****G****G****C**AA**C****C**.**G**.**G****C****A****C**-........................GUAA-....................................................-**G****U****G****C**.U...**U**..**G****G**U**G**..**C****C**AAU**U****C****C****U****G**...**C****A****G****G****U****U**GGG.................GUUA........CCC**A****G****C****C****U****G**.AG.**A**..**G****A**..**U****G****A****G****A****G****G****A****G**AGGC | |
|  |  | NC\_002973.5/325918-325785  | AUAU**U****U****U****C****U****C****U****U**.**A****U****C**G.AGA**G**.**U****G****G****C**.**A****G****A****G****G****G**A...**C****U****G**.**G**.**C****C****C**GAU**G****A****A**.**G****C****C**-.**C****G****G****C**AA**C****C**.**U**.**A****A****C****U****U**UA......................UUUAA...............................................GCGUA**A****A****G****U****G**.A...**A**..**G****G**U**G**..**C****U**AAU**U****C****C****A****G**...**C****A****A****A****A****U**GGU.................GUAU........UCC**A****U****U****U****U****G**.GU.**A**..**G****A**..**U****A****A****G****A****G****G****A****G**CUGG | |
|  |  | NC\_004722.1/1382033-1382155  | AAUA**C****A****A****A****G****C****U****U**.**A****U****C**A.AGA**G**.**A****A****G****C**.**G****G****A****G****G****G**AA..**C****U****G**.**G**.**C****C****C**GAA**G****A****A**.**G****C****U**-.**C****G****G****C**AA**C****C**.**U**.**G****C****U****U**-........................AUAGA....................................................-**A****A****G****C**.A...**A**..**G****G**U**G**..**C****U**AAA**U****C****C****A****G**...**C****A****A****A****A****U**G...................GGAU.........CC**A****U****U****U****U****G**.AA.**A**..**G****A**..**U****A****A****G****G****U****A****A****A**AUAU | |
|  |  | NC\_003909.8/361547-361667  | CGAU**A****C****A****U****U****C****U****U**.**A****U****C**C.AGA**G**.**A****G****G****U**.**G****G****A****G****G****G**A...**C****U****G**.**G**.**C****C****C**UAC**G****A****U**.**A****C****C****U**.**C****A****G****C**AA**C****G**.**G**.**G****U****U**--........................UUUUU....................................................--**A****A****U**.A...**C**..**C****G**U**G**..**C****U**AAC**U****C****C****A****G**...**C****A****A****G****C****C**UA..................UGAA..........A**G****G****C****U****U****G**.GA.**A**..**G****A**..**U****G****A****G****A****A****G****A****U**GUGA | |
|  |  | NC\_004193.1/1098106-1097986  | AUGA**C****A****A****U****U****C****U****U**.**A****U****C**C.AGA**G**.**A****G****G****U**.**G****G****A****G****G****G**A...**C****U****G**.**G**.**C****C****C**AAG**G****A****A**.**G****C****C****U**.**C****G****G****C**AA**C****A**.**G**.**A****C****U****U****A**U.......................UUGAU....................................................**U****A****A****G****U**.A...**C**..**U****G**U**G**..**C****C**AAU**U****C****C****A****G**...**U****A****G****C****G**-....................UAAU...........-**U****G****C****U****A**.GA.**A**..**G****A**..**U****G****A****G****A****A****G****A****G**UAUA | |
|  |  | NC\_003030.1/1073895-1073778  | UAAU**A****U****U****U****C****C****U****U**.**A****U****C**A.AGA**G**.**A****A****A****C**.**G****G****A****G****G****G**A...**C****U****G**.**G**.**C****C****C**AAU**G****A****U**.**G****U****U****U**.**C****A****G****C**AA**C****C**.**A**.**A****G****G****U**-........................UUUAU....................................................-**A****C****U****U**.A...**U**..**G****G**U**G**..**C****U**AAU**U****C****C****A****G**...**C****A****G****G****A**-....................UAUU...........-**U****U****C****U****G**.AA.**A**..**G****A**..**U****G****A****G****G****A****G****C****G**ACUA | |
|  |  | NC\_003909.8/225261-225140  | UUUA**C****U****C****A****U****U****G****U**.**A****U****C**A.AGA**G**.**A****G****G****U**.**G****G****A****G****G****G**A...**C****U****G**.**G**.**C****C****C**UUU**G****A****A**.**A****C****C****U**.**C****G****G****C**AG**C****A**.**G**.**G****U****U****C****A**........................UUUUU....................................................**U****G****A****A****U**.A...**C**..**U****G**U**G**..**C****C**ACU**U****C****C****U****G**...**C****A****A****G****C****U**....................UUAU...........**A****G****C****U****U****G**.AA.**A**..**G****A**..**U****A****G****A****A****U****G****A****G**GGAC | |
|  |  | NC\_003909.8/3299174-3299054  | AUUA**G****U****U****U****U****C****U****U**.**A****U****U**A.AGA**G**.**A****G****A****U**.**G****G****A****G****G****G**A...**C****U****G**.**G**.**C****C****C**GAU**G****A****A**.**A****U****C****U**.**C****A****G****C**AA**C****A**.**G**.**G****C****U**--........................AUAAA....................................................--**A****G****U**.A...**C**..**U****G**U**G**..**C****U**AAG**U****C****C****A****G**...**C****A****A****A****C****G**UA..................UGAA..........G**C****G****U****U****U****G**.GA.**A**..**G****A**..**U****G****A****G****G****G****G****A****A**AUGG | |
|  |  | NC\_003030.1/2991414-2991290  | AUGG**A****A****A****C****U****C****U****U**.**A****U****C**A.AGA**G**.**A****G****G****U**.**G****G****A****G****G****G**AA..**A****G****G**.**G**.**C****C****C**GUU**G****A****A**.**A****C****C**-.**C****G****G****C**AA**C****C**.**G**.-**A****U****G****U**AUUAA...................UUUAA..................................................GU**A****C****A****U****A**.A...**U**..**G****G**U**G**..**C****C**AAU**U****C****C****U****G**...**C****A****G****A****A**-....................-UUA...........-**U****U****C****U****G**.CA.**A**..**G****A**..**U****A****A****G****A****G****A****G****A**GAAU | |
|  |  | NZ\_AAAW03000200.1/1134-1020  | AUAA**A****C****A****C****U****C****U****U**.**A****U****C**C.AGA**G**U**A****G****G****U**.**G****G****A****G****G****G**A...**C****U****G**.**G**.**C****C****C**GAU**G****A****A**.**A****C****C**-.**C****G****G****C**AA**C****C**.**G**.**A****C****A****U**-........................GAAA-....................................................-**A****U****G****C**.A...**C**..**G****G**U**G**..**C****U**AAA**U****C****C****U****G**...**C****A****G****G**--....................AAAU...........--**A****C****U****G**.GG.**A**..**G****A**..**U****G****A****G****A****G****G****A****A**ACCC | |
|  |  | NZ\_AADR01000001.1/130761-130894  | AUAU**U****U****U****C****U****C****U****U**.**A****U****C**G.AGA**G**.**C****G****G****C**.**A****G****A****G****G****G**A...**C****U****G**.**G**.**C****C****C**GAU**G****A****A**.**G****C****C**-.**C****G****G****C**AA**C****C**.**U**.**A****A****C****U****U**UA......................UUUAA...............................................GCGUA**A****A****G****U****G**.A...**A**..**G****G**U**G**..**C****U**AAU**U****C****C****A****G**...**C****A****A****A****A****U**GGU.................GUAU........UCC**A****U****U****U****U****G**.GU.**A**..**G****A**..**U****A****A****G****A****G****G****A****G**CUGG | |
|  |  | NZ\_AADW02000002.1/258943-258826  | ACCG**C****A****A****C****A****C****U****U**.**A****U****C**C.AGA**G**.**A****G****G****U**.**G****G****A****G****G****G**A...**C****U****G**.**G**.**C****C****C**UAC**G****A****A**.**A****C****C****U**.**C****G****G****C**AA**C****A**.**G**.**A****C****U****C**-........................AUUAU....................................................-**G****A****G****C**.A...**C**..**U****G**U**G**..**C****C**AAU**U****C****C****A****U**...**C****A****G****A****C**-....................AAUU...........-**G****U****C****U****G**.AG.**A**..**G****A**..**U****G****A****G****U****C****G****A****G**UGGA | |
|  |  | NC\_004193.1/727019-727138  | AUAG**U****U****A****G****A****C****U****U**.**A****U****C**A.AGA**G**.**A****G****A****U**.**G****G****A****G****G****G**A...**U****U****G**.**G**.**C****C****C**GAU**G****A****A**.**G****U****C****U**.**C****A****G****C**AA**C****C**.**A**.**G****C****C****U**-........................AGAUA....................................................-**A****G****G****U**.A...**U**..**G****G**U**G**..**C****U**AAU**U****C****C****A****A**...**U****A****G****G****C****U**....................UACA...........**A****G****C****C****U****U**.AA.**A**..**G****A**..**U****A****A****G****A****A****G****A****G**CUAU | |
|  |  | NC\_003030.1/1976364-1976478  | AUAU**U****A****U****U****U****C****U****U**.**A****U****C**A.AGA-.**A****G****G****U**.**G****G****A****G****G****G**A...**C****U****G**.**G**.**C****C****C**UAU**G****A****A**.**G****C****C****U**.-**G****A****C**AA**C****C**.**G**.**G****C**---........................AAAU-....................................................---**G****U**.A...**C**..**G****G**U**G**..**U****U**AAU**U****C****C****U****G**...**C****A****A****A****A****C**U...................UAUU..........U**G****U****U****U****U****G**.AA.**A**..**G****A**..**U****A****A****G****A****A****A****A****C**AGCU | |
|  |  | NC\_003997.3/197194-197074  | UUUA**C****U****C****A****U****U****G****U**.**A****U****C**A.AGA**G**.**A****G****G****U**.**G****G****A****G****G****G**A...**C****U****G**.**G**.**C****C****C**UUU**G****A****A**.**A****C****C****U**.**C****G****G****C**AG**C****A**.**G**.**G****U****U****C****A**........................UUUU-....................................................**U****G****A****A****U**.A...**C**..**U****G**U**G**..**C****C**ACU**U****C****C****U****G**...**C****A****A****G****C****U**....................UUAU...........**A****G****C****U****U****G**.AA.**A**..**G****A**..**U****A****G****A****A****U****G****A****G**GGAC | |
|  |  | NC\_005945.1/197195-197075  | UUUA**C****U****C****A****U****U****G****U**.**A****U****C**A.AGA**G**.**A****G****G****U**.**G****G****A****G****G****G**A...**C****U****G**.**G**.**C****C****C**UUU**G****A****A**.**A****C****C****U**.**C****G****G****C**AG**C****A**.**G**.**G****U****U****C****A**........................UUUU-....................................................**U****G****A****A****U**.A...**C**..**U****G**U**G**..**C****C**ACU**U****C****C****U****G**...**C****A****A****G****C****U**....................UUAU...........**A****G****C****U****U****G**.AA.**A**..**G****A**..**U****A****G****A****A****U****G****A****G**GGAC | |
|  |  | NC\_005957.1/201148-201028  | UUUA**C****U****C****A****U****U****G****U**.**A****U****C**A.AGA**G**.**A****G****G****U**.**G****G****A****G****G****G**A...**C****U****G**.**G**.**C****C****C**UUU**G****A****A**.**A****C****C****U**.**C****G****G****C**AG**C****A**.**G**.**G****U****U****C****A**........................UUUU-....................................................**U****G****A****A****U**.A...**C**..**U****G**U**G**..**C****C**ACU**U****C****C****U****G**...**C****A****A****G****C****U**....................UUAU...........**A****G****C****U****U****G**.AA.**A**..**G****A**..**U****A****G****A****A****U****G****A****G**GGAC | |
|  |  | NC\_006274.1/198522-198402  | UUUA**C****U****C****A****U****U****G****U**.**A****U****C**A.AGA**G**.**A****G****G****U**.**G****G****A****G****G****G**A...**C****U****G**.**G**.**C****C****C**UUU**G****A****A**.**A****C****C****U**.**C****G****G****C**AG**C****A**.**G**.**G****U****U****C****A**........................UUUU-....................................................**U****G****A****A****U**.A...**C**..**U****G**U**G**..**C****C**ACU**U****C****C****U****G**...**C****A****A****G****C****U**....................UUAU...........**A****G****C****U****U****G**.AA.**A**..**G****A**..**U****A****G****A****A****U****G****A****G**GGAC | |
|  |  | NC\_007530.2/197194-197074  | UUUA**C****U****C****A****U****U****G****U**.**A****U****C**A.AGA**G**.**A****G****G****U**.**G****G****A****G****G****G**A...**C****U****G**.**G**.**C****C****C**UUU**G****A****A**.**A****C****C****U**.**C****G****G****C**AG**C****A**.**G**.**G****U****U****C****A**........................UUUU-....................................................**U****G****A****A****U**.A...**C**..**U****G**U**G**..**C****C**ACU**U****C****C****U****G**...**C****A****A****G****C****U**....................UUAU...........**A****G****C****U****U****G**.AA.**A**..**G****A**..**U****A****G****A****A****U****G****A****G**GGAC | |
|  |  | NZ\_AAAC02000001.1/736643-736523  | UUUA**C****U****C****A****U****U****G****U**.**A****U****C**A.AGA**G**.**A****G****G****U**.**G****G****A****G****G****G**A...**C****U****G**.**G**.**C****C****C**UUU**G****A****A**.**A****C****C****U**.**C****G****G****C**AG**C****A**.**G**.**G****U****U****C****A**........................UUUU-....................................................**U****G****A****A****U**.A...**C**..**U****G**U**G**..**C****C**ACU**U****C****C****U****G**...**C****A****A****G****C****U**....................UUAU...........**A****G****C****U****U****G**.AA.**A**..**G****A**..**U****A****G****A****A****U****G****A****G**GGAC | |
|  |  | NZ\_AAEN01000019.1/49010-48890  | UUUA**C****U****C****A****U****U****G****U**.**A****U****C**A.AGA**G**.**A****G****G****U**.**G****G****A****G****G****G**A...**C****U****G**.**G**.**C****C****C**UUU**G****A****A**.**A****C****C****U**.**C****G****G****C**AG**C****A**.**G**.**G****U****U****C****A**........................UUUU-....................................................**U****G****A****A****U**.A...**C**..**U****G**U**G**..**C****C**ACU**U****C****C****U****G**...**C****A****A****G****C****U**....................UUAU...........**A****G****C****U****U****G**.AA.**A**..**G****A**..**U****A****G****A****A****U****G****A****G**GGAC | |
|  |  | NZ\_AAEO01000023.1/48759-48879  | UUUA**C****U****C****A****U****U****G****U**.**A****U****C**A.AGA**G**.**A****G****G****U**.**G****G****A****G****G****G**A...**C****U****G**.**G**.**C****C****C**UUU**G****A****A**.**A****C****C****U**.**C****G****G****C**AG**C****A**.**G**.**G****U****U****C****A**........................UUUU-....................................................**U****G****A****A****U**.A...**C**..**U****G**U**G**..**C****C**ACU**U****C****C****U****G**...**C****A****A****G****C****U**....................UUAU...........**A****G****C****U****U****G**.AA.**A**..**G****A**..**U****A****G****A****A****U****G****A****G**GGAC | |
|  |  | NZ\_AAEP01000033.1/46590-46470  | UUUA**C****U****C****A****U****U****G****U**.**A****U****C**A.AGA**G**.**A****G****G****U**.**G****G****A****G****G****G**A...**C****U****G**.**G**.**C****C****C**UUU**G****A****A**.**A****C****C****U**.**C****G****G****C**AG**C****A**.**G**.**G****U****U****C****A**........................UUUU-....................................................**U****G****A****A****U**.A...**C**..**U****G**U**G**..**C****C**ACU**U****C****C****U****G**...**C****A****A****G****C****U**....................UUAU...........**A****G****C****U****U****G**.AA.**A**..**G****A**..**U****A****G****A****A****U****G****A****G**GGAC | |
|  |  | NZ\_AAEQ01000020.1/47667-47787  | UUUA**C****U****C****A****U****U****G****U**.**A****U****C**A.AGA**G**.**A****G****G****U**.**G****G****A****G****G****G**A...**C****U****G**.**G**.**C****C****C**UUU**G****A****A**.**A****C****C****U**.**C****G****G****C**AG**C****A**.**G**.**G****U****U****C****A**........................UUUU-....................................................**U****G****A****A****U**.A...**C**..**U****G**U**G**..**C****C**ACU**U****C****C****U****G**...**C****A****A****G****C****U**....................UUAU...........**A****G****C****U****U****G**.AA.**A**..**G****A**..**U****A****G****A****A****U****G****A****G**GGAC | |
|  |  | NZ\_AAER01000024.1/48765-48885  | UUUA**C****U****C****A****U****U****G****U**.**A****U****C**A.AGA**G**.**A****G****G****U**.**G****G****A****G****G****G**A...**C****U****G**.**G**.**C****C****C**UUU**G****A****A**.**A****C****C****U**.**C****G****G****C**AG**C****A**.**G**.**G****U****U****C****A**........................UUUU-....................................................**U****G****A****A****U**.A...**C**..**U****G**U**G**..**C****C**ACU**U****C****C****U****G**...**C****A****A****G****C****U**....................UUAU...........**A****G****C****U****U****G**.AA.**A**..**G****A**..**U****A****G****A****A****U****G****A****G**GGAC | |
|  |  | NZ\_AAES01000019.1/47595-47475  | UUUA**C****U****C****A****U****U****G****U**.**A****U****C**A.AGA**G**.**A****G****G****U**.**G****G****A****G****G****G**A...**C****U****G**.**G**.**C****C****C**UUU**G****A****A**.**A****C****C****U**.**C****G****G****C**AG**C****A**.**G**.**G****U****U****C****A**........................UUUU-....................................................**U****G****A****A****U**.A...**C**..**U****G**U**G**..**C****C**ACU**U****C****C****U****G**...**C****A****A****G****C****U**....................UUAU...........**A****G****C****U****U****G**.AA.**A**..**G****A**..**U****A****G****A****A****U****G****A****G**GGAC | |
|  |  | NC\_003210.1/309392-309259  | AUAU**U****U****U****C****U****C****U****U**.**A****U****C**G.AGA**G**.**C****G****G****C**.**A****G****A****G****G****G**A...**C****U****G**.**G**.**C****C****C**GAU**G****A****A**.**G****C****C**-.**C****G****G****C**AA**C****C**.**U**.**A****A****C****U****U**UA......................UUUAA...............................................GCAUA**A****A****G****U****G**.A...**A**..**G****G**U**G**..**C****U**AAU**U****C****C****A****G**...**C****A****A****A****A****U**GGU.................GUAU........UCC**G****U****U****U****U****G**.GU.**A**..**G****A**..**U****A****A****G****A****G****G****A****G**CUGG | |
|  |  | NC\_003212.1/327343-327210  | AUAU**U****U****U****C****U****C****U****U**.**A****U****C**G.AGA**G**.**C****G****G****C**.**A****G****A****G****G****G**A...**C****U****G**.**G**.**C****C****C**GAU**G****A****A**.**G****C****C**-.**C****G****G****C**AA**C****C**.**U**.**A****A****C****U****U**UA......................UUUAA...............................................GCGUA**A****A****G****U****G**.A...**A**..**G****G**U**G**..**C****U**AAU**U****C****C****A****G**...**C****A****A****A****A****U**GGU.................GUAU........UCC**G****U****U****U****U****G**.GU.**A**..**G****A**..**U****A****A****G****A****G****G****A****G**CUGG | |
|  |  | NZ\_AADQ01000021.1/21706-21839  | AUAU**U****U****U****C****U****C****U****U**.**A****U****C**G.AGA**G**.**C****G****G****C**.**A****G****A****G****G****G**A...**C****U****G**.**G**.**C****C****C**GAU**G****A****A**.**G****C****C**-.**C****G****G****C**AA**C****C**.**U**.**A****A****C****U****U**UA......................UUUAA...............................................GCGUA**A****A****G****U****G**.A...**A**..**G****G**U**G**..**C****U**AAU**U****C****C****A****G**...**C****A****A****A****A****U**GGU.................GUAU........UCC**G****U****U****U****U****G**.GU.**A**..**G****A**..**U****A****A****G****A****G****G****A****G**CUGG | |
|  |  | NC\_004193.1/2708652-2708532  | UACG**U****U****U****U****U****C****U****U**.**A****U****C**A.UGA**G**.**A****G****G****C**.**G****G****A****G****G****G**AA..**A****U****G**.**G**.**C****C****C**AAC**G****A****A**.**A****C****C****U**.**C****G****G****C**AA**C****A**.**G**.**G****U****U****C****U**........................UAUU-....................................................**A****G****A****A****U**.A...**C**..**U****G**U**G**..**C****C**AAU**U****C****C****A****U**...**C****A****A****G****C****A**....................-AAU...........**U****G****C****U****U****G**.AA.**A**..**G****A**..**U****A****A****G****A****G****U****A****G**AAUA | |
|  |  | NC\_004722.1/194629-194511  | UUUA**C****U****C****A****U****U****G****U**.**A****U****C**A.AGA**G**.**A****G****G****U**.**G****G****A****G****G****G**A...**C****U****G**.**G**.**C****C****C**UUU**G****A****A**.**A****C****C****U**.**C****G****G****C**AA**C****A**.**G**.**G****U****U****C**-........................AUUUU....................................................-**G****A****A****U**.A...**C**..**U****G**U**G**..**C****C**ACU**U****C****C****U****G**...**C****A****A****G****C**-....................UUUA..........U-**G****C****U****U****G**.AA.**A**..**G****A**..**U****A****G****A****A****U****G****A****G**GGAC | |
|  |  | NC\_004557.1/2676962-2676846  | AUAA**G****G****A****U****U****C****U****U**.**A****U****C**A.AGA**G**.**A****G****G****C**.**G****G****A****G****G****G**A...**C****U****G**.**G**.**C****C****C**UAU**G****A****A**.**A****C****C**-.**C****G****G****C**AA**C****C**.**A**.**A****A****A****A****U**........................AAUA-....................................................**A****U****U****U****U**.A...**A**..**G****G**U**G**..**C****C**AAU**U****C****C****A****G**...**C****A****G****G****U**-....................-GAA...........-**A****C****C****U****G**.AC.**A**..**G****A**..**U****A****A****G****A****C****G****U****A**GAGG | |
|  |  | NC\_004193.1/2437314-2437201  | AUGA**U****A****U****C****U****C****U****U**.**A****U****C**U.AGA**G**.**C****G****G****U**.**G****G****A****G****G****G**A...**C****U****G**.**G**.**C****C****C**UUU**G****A****A**.**A****C****C****G**.**C****G****G****C**AA**C****C**.**U**.**U****C****A****U****A**........................AUUA-....................................................**A****A****U****G****A**.A...**A**..**G****G**U**G**..**C****C**AAU**U****C****C****U****G**...**C****A****G**---....................-AAA...........---**A****U****G**.AA.**A**..**G****A**..**U****G****A****G****A****G****A****A****C**GUCA | |
|  |  | NC\_002973.5/1720640-1720520  | UAAA**U****U****A****C****U****C****U****U**.**A****U****U**A.UGA**G**.**U****G****G****U**.**A****G****A****G****G****G**A...**C****U****G**.**G**.**C****C****C**GUU**G****A****A**.**A****C****C**-.**C****A****G****C**AA**C****C**.**U**.**U****U****C****A****A**........................UUCG-....................................................**U****U****G****A****A**.A...**A**..**G****G**U**G**..**C****U**AAA**U****C****C****U****G**...**C****G****A****A****G****U**....................GUGA..........U**G****C****U****U****C****G**.AG.**A**..**G****A**..**U****A****A****G****A****G****A****G****A**CUUA | |
|  |  | NC\_003212.1/1790199-1790079  | UAAA**U****U****A****C****U****C****U****U**.**A****U****U**A.UGA**G**.**U****G****G****U**.**A****G****A****G****G****G**A...**C****U****G**.**G**.**C****C****C**GUU**G****A****A**.**A****C****C**-.**C****A****G****C**AA**C****C**.**U**.**U****U****C****A****A**........................UUCG-....................................................**U****U****G****A****A**.A...**A**..**G****G**U**G**..**C****U**AAA**U****C****C****U****G**...**C****G****A****A****G****U**....................GUGA..........U**G****C****U****U****C****G**.AG.**A**..**G****A**..**U****A****A****G****A****G****A****G****A**CUUA | |
|  |  | NZ\_AADR01000038.1/16228-16108  | UAAA**U****U****A****C****U****C****U****U**.**A****U****U**A.UGA**G**.**U****G****G****U**.**A****G****A****G****G****G**A...**C****U****G**.**G**.**C****C****C**GUU**G****A****A**.**A****C****C**-.**C****A****G****C**AA**C****C**.**U**.**U****U****C****A****A**........................UUCG-....................................................**U****U****G****A****A**.A...**A**..**G****G**U**G**..**C****U**AAA**U****C****C****U****G**...**C****G****A****A****G****U**....................GUGA..........U**G****C****U****U****C****G**.AG.**A**..**G****A**..**U****A****A****G****A****G****A****G****A**CUUA | |
|  |  | NZ\_AADT03000059.1/3706-3828  | CCGG**A****A****A****C****U****C****U****U**.**A****U****C**G.AGA**G**C**U****G****G****C**.**G****G****A****G****G****G**A...**C****U****G**.**G**.**C****C****C**GAU**G****A****A**.**G****C****C**-.**C****G****G****C**AA**C****C**.**G**.**G****C****C****U****C**U.......................UAGAA..................................................CC**C****A****G****G****U**.A...**C**..**G****G**U**G**..**C****C**AAU**U****C****C****U****G**...**C****A****G****G****A**-....................UUUC...........-**U****C****C****U****G**.GC.**A**..**G****A**..**U****G****A****G****A****G****U****U****G**AAAA | |
|  |  | NC\_003997.3/1375344-1375463  | UGAA**A****C****C****U****U****C****U****U**.**A****U****A**A.AGA**G**.**A****G****G****C**.**G****G****A****G****G****G**A...**C****U****G**.**G**.**C****C****C**UAC**G****A****U**.**G****C****C****U**.**C****G****G****C**AG**C****G**.**G**.**A****C****U****C****G**........................AUUUU....................................................**A****G****A****G****U**.G...**C**..**U****G**U**G**..**C****C**AAA**U****C****C****A****G**...**C****A****A****G****C**-....................AUGU...........-**G****C****U****U****G**.AA.**A**..**G****A**..**U****G****A****G****A****A****G****A****G**CGUU | |
|  |  | NC\_005945.1/1375412-1375531  | UGAA**A****C****C****U****U****C****U****U**.**A****U****A**A.AGA**G**.**A****G****G****C**.**G****G****A****G****G****G**A...**C****U****G**.**G**.**C****C****C**UAC**G****A****U**.**G****C****C****U**.**C****G****G****C**AG**C****G**.**G**.**A****C****U****C****G**........................AUUUU....................................................**A****G****A****G****U**.G...**C**..**U****G**U**G**..**C****C**AAA**U****C****C****A****G**...**C****A****A****G****C**-....................AUGU...........-**G****C****U****U****G**.AA.**A**..**G****A**..**U****G****A****G****A****A****G****A****G**CGUU | |
|  |  | NC\_005957.1/1398751-1398870  | UGAA**A****C****C****U****U****C****U****U**.**A****U****A**A.AGA**G**.**A****G****G****C**.**G****G****A****G****G****G**A...**C****U****G**.**G**.**C****C****C**UAC**G****A****U**.**G****C****C****U**.**C****G****G****C**AG**C****G**.**G**.**A****C****U****C****G**........................AUUUU....................................................**A****G****A****G****U**.G...**C**..**U****G**U**G**..**C****C**AAA**U****C****C****A****G**...**C****A****A****G****C**-....................AUGU...........-**G****C****U****U****G**.AA.**A**..**G****A**..**U****G****A****G****A****A****G****A****G**CGUU | |
|  |  | NC\_006274.1/1410129-1410248  | UGAA**A****C****C****U****U****C****U****U**.**A****U****A**A.AGA**G**.**A****G****G****C**.**G****G****A****G****G****G**A...**C****U****G**.**G**.**C****C****C**UAC**G****A****U**.**G****C****C****U**.**C****G****G****C**AG**C****G**.**G**.**A****C****U****C****G**........................AUUUU....................................................**A****G****A****G****U**.G...**C**..**U****G**U**G**..**C****C**AAA**U****C****C****A****G**...**C****A****A****G****C**-....................AUGU...........-**G****C****U****U****G**.AA.**A**..**G****A**..**U****G****A****G****A****A****G****A****G**CGUU | |
|  |  | NC\_007530.2/1375467-1375586  | UGAA**A****C****C****U****U****C****U****U**.**A****U****A**A.AGA**G**.**A****G****G****C**.**G****G****A****G****G****G**A...**C****U****G**.**G**.**C****C****C**UAC**G****A****U**.**G****C****C****U**.**C****G****G****C**AG**C****G**.**G**.**A****C****U****C****G**........................AUUUU....................................................**A****G****A****G****U**.G...**C**..**U****G**U**G**..**C****C**AAA**U****C****C****A****G**...**C****A****A****G****C**-....................AUGU...........-**G****C****U****U****G**.AA.**A**..**G****A**..**U****G****A****G****A****A****G****A****G**CGUU | |
|  |  | NZ\_AAAC02000001.1/1867309-1867428  | UGAA**A****C****C****U****U****C****U****U**.**A****U****A**A.AGA**G**.**A****G****G****C**.**G****G****A****G****G****G**A...**C****U****G**.**G**.**C****C****C**UAC**G****A****U**.**G****C****C****U**.**C****G****G****C**AG**C****G**.**G**.**A****C****U****C****G**........................AUUUU....................................................**A****G****A****G****U**.G...**C**..**U****G**U**G**..**C****C**AAA**U****C****C****A****G**...**C****A****A****G****C**-....................AUGU...........-**G****C****U****U****G**.AA.**A**..**G****A**..**U****G****A****G****A****A****G****A****G**CGUU | |
|  |  | NZ\_AAEN01000008.1/48659-48540  | UGAA**A****C****C****U****U****C****U****U**.**A****U****A**A.AGA**G**.**A****G****G****C**.**G****G****A****G****G****G**A...**C****U****G**.**G**.**C****C****C**UAC**G****A****U**.**G****C****C****U**.**C****G****G****C**AG**C****G**.**G**.**A****C****U****C****G**........................AUUUU....................................................**A****G****A****G****U**.G...**C**..**U****G**U**G**..**C****C**AAA**U****C****C****A****G**...**C****A****A****G****C**-....................AUGU...........-**G****C****U****U****G**.AA.**A**..**G****A**..**U****G****A****G****A****A****G****A****G**CGUU | |
|  |  | NZ\_AAEO01000017.1/2190-2309  | UGAA**A****C****C****U****U****C****U****U**.**A****U****A**A.AGA**G**.**A****G****G****C**.**G****G****A****G****G****G**A...**C****U****G**.**G**.**C****C****C**UAC**G****A****U**.**G****C****C****U**.**C****G****G****C**AG**C****G**.**G**.**A****C****U****C****G**........................AUUUU....................................................**A****G****A****G****U**.G...**C**..**U****G**U**G**..**C****C**AAA**U****C****C****A****G**...**C****A****A****G****C**-....................AUGU...........-**G****C****U****U****G**.AA.**A**..**G****A**..**U****G****A****G****A****A****G****A****G**CGUU | |
|  |  | NZ\_AAEP01000030.1/181375-181494  | UGAA**A****C****C****U****U****C****U****U**.**A****U****A**A.AGA**G**.**A****G****G****C**.**G****G****A****G****G****G**A...**C****U****G**.**G**.**C****C****C**UAC**G****A****U**.**G****C****C****U**.**C****G****G****C**AG**C****G**.**G**.**A****C****U****C****G**........................AUUUU....................................................**A****G****A****G****U**.G...**C**..**U****G**U**G**..**C****C**AAA**U****C****C****A****G**...**C****A****A****G****C**-....................AUGU...........-**G****C****U****U****G**.AA.**A**..**G****A**..**U****G****A****G****A****A****G****A****G**CGUU | |
|  |  | NZ\_AAEQ01000023.1/181644-181763  | UGAA**A****C****C****U****U****C****U****U**.**A****U****A**A.AGA**G**.**A****G****G****C**.**G****G****A****G****G****G**A...**C****U****G**.**G**.**C****C****C**UAC**G****A****U**.**G****C****C****U**.**C****G****G****C**AG**C****G**.**G**.**A****C****U****C****G**........................AUUUU....................................................**A****G****A****G****U**.G...**C**..**U****G**U**G**..**C****C**AAA**U****C****C****A****G**...**C****A****A****G****C**-....................AUGU...........-**G****C****U****U****G**.AA.**A**..**G****A**..**U****G****A****G****A****A****G****A****G**CGUU | |
|  |  | NZ\_AAER01000019.1/48467-48348  | UGAA**A****C****C****U****U****C****U****U**.**A****U****A**A.AGA**G**.**A****G****G****C**.**G****G****A****G****G****G**A...**C****U****G**.**G**.**C****C****C**UAC**G****A****U**.**G****C****C****U**.**C****G****G****C**AG**C****G**.**G**.**A****C****U****C****G**........................AUUUU....................................................**A****G****A****G****U**.G...**C**..**U****G**U**G**..**C****C**AAA**U****C****C****A****G**...**C****A****A****G****C**-....................AUGU...........-**G****C****U****U****G**.AA.**A**..**G****A**..**U****G****A****G****A****A****G****A****G**CGUU | |
|  |  | NZ\_AAES01000029.1/181603-181722  | UGAA**A****C****C****U****U****C****U****U**.**A****U****A**A.AGA**G**.**A****G****G****C**.**G****G****A****G****G****G**A...**C****U****G**.**G**.**C****C****C**UAC**G****A****U**.**G****C****C****U**.**C****G****G****C**AG**C****G**.**G**.**A****C****U****C****G**........................AUUUU....................................................**A****G****A****G****U**.G...**C**..**U****G**U**G**..**C****C**AAA**U****C****C****A****G**...**C****A****A****G****C**-....................AUGU...........-**G****C****U****U****G**.AA.**A**..**G****A**..**U****G****A****G****A****A****G****A****G**CGUU | |
|  |  | NC\_006582.1/3104700-3104581  | AUAU**U****C****A****U****U****C****U****U**.**A****U****C**G.AGA**G**.**A****G****G****U**.**G****G****A****G****G****G**A...**C****U****G**.**G**.**C****C****C**AAU**G****A****A**.**A****C****C**-.**C****G****G****C**AA**C****C**.**G**.**C****A****A****G**-........................UUCG-....................................................-**C****U****U****G**.A...**A**..**G****G**U**G**..**C****U**AAA**U****C****C****U****G**...**C****A****A****A****G****C**....................AUAU.........GG**G****C****U****U****U****G**.GG.**A**..**G****A**..**U****G****A****G****A****G****G****G****A**AGCA | |
|  |  | NC\_002570.2/1699950-1700073  | UCUC**G****U****A****U****U****C****U****U**.**A****U****C**C.AGA**G**.**A****G****G****U**.**G****G****A****G****G****G**A...**A****C****G**.**G**.**C****C****C**GAA**G****A****A**.**A****C****C****U**.**C****A****G****C**AA**C****C**.**A**.**G****C****C****A****C**G.......................AUCCU....................................................**G****U****G****G****U**.C...**A**..**G****G**U**G**..**C****U**AAU**U****C****C****U****G**...**C****A****A****G****C****A**....................UUAU..........U**U****G****C****U****U****G**.AG.**A**..**G****A**..**U****A****A****G****A****G****G****A****A**GCGA | |
|  |  | NZ\_AAEO01000030.1/75093-75213  | CGAU**A****C****A****U****U****C****U****U**.**A****U****C**C.AGA**G**.**A****G****G****U**.**G****G****A****G****G****G**A...**C****U****G**.**G**.**C****C****C**UAC**G****A****U**.**A****C****C****U**.**C****A****G****U**AA**C****G**.**G**.**G****U****U**--........................UUUUU....................................................--**A****A****U**.A...**C**..**C****G**U**G**..**C****U**AAC**U****C****C****A****G**...**C****A****A****G****C****C**AU..................AUAA..........A**G****G****C****U****U****G**.GA.**A**..**G****A**..**U****G****A****G****A****A****G****A****U**GUGA | |
|  |  | NZ\_AAEK01000030.1/59118-59236  | UUGC**A****U****A****G****U****C****U****U**.**A****U****C**A.AGA**A**A**A****G****G****U**.**G****G****A****G****G****G**A...**C****A****G**.**G**.**C****C****C**GAU**G****A****A**.**A****C****C****U**.**U****G****G****C**AA**C****A**.**G**.-**C****C****G**-........................UAUAA....................................................-**C****G****G****A**.A...**U**..**U****G**U**G**..**C****C**AAA**U****C****C****U****G**...**C****A****G****G**--UA..................GUAA..........U--**C****C****U****G**.AA.**A**..**G****A**..**U****A****A****G****A****A****A****G****A**GCCU | |
|  |  | NC\_005957.1/1833720-1833843  | UAAA**A****A****U****U****U****C****U****U**.**A****U****U**A.AGA**G**.**A****G****G****U**.**G****G****A****G****G****G**A...**C****U****G**.**G**.**C****C****C**UUC**G****A****A**.**G****C****C****U**.**C****A****G****C**AA**C****C**.**U**.**G****A****U****U****U**........................AUGUG....................................................**A****U****A****U****A**.A...**A**..**G****G**U**G**..**C****U**AAA**U****C****C****U****G**...**U****A****G****G****A****U**A...................UAAA..........A**G****U****C****C****U****A**.AU.**A**..**G****A**..**U****A****A****G****A****A****A****A****U**GGGU | |
|  |  | NZ\_AADW02000029.1/16706-16587  | CCGA**U****A****A****C****U****C****U****U**.**A****U****C**G.AGA**G**.**U****G****G****U**.**G****G****A****G****G****G**A...**C****U****G**.**G**.**C****C****C**GAU**G****A****A**.**A****C****C**-.**C****G****G****C**AA**C****C**.**G**.--**C****G****G**AA......................UUUAU...................................................U**C****C****G****A****A**.G...**U**..**G****G**U**G**..**C****U**AAU**U****C****C****A****G**...**C****A****G****A****C**-....................GAUU...........-**G****U****C****U****G**.CA.**A**..**G****A**..**U****G****A****G****A****G****C****A****A**AUGG | |
|  |  | NC\_003030.1/1131530-1131648  | AUUA**G****U****G****C****A****C****U****U**.**A****U****C**A.AGA**G**.**A****G****G****U**.**G****G****A****G****G****G**A...**C****C****G**.**G**.**C****C****C**UGU**G****A****A**.**G****C****C**-.**C****A****G****C**AA**C****C**.**U**.**G****U****A****U****A**UG......................UUAAU....................................................**U****A****U****A****C**.A...**A**..**G****G**U**G**..**C****U**AAU**U****C****C****U****G**...**C****A****G****C**--....................GCUA...........--**G****C****U****G**.AG.**A**..**G****A**..**U****G****A****G****A****A****U****A****U**AAAU | |
|  |  | NC\_003030.1/671363-671244  | UGUA**A****A****A****A****U****C****U****U**.**A****U****C**A.AGA**G**.**U****G****G****U**.**G****G****A****G****G****G**A...**C****U****G**.**G**.**C****C****C**UUU**G****A****A**.**A****C****C**-.**C****G****G****C**AA**C****C**.**A**.**G****U****A****U****A**UUU.....................UUUAA....................................................**U****A****U****A****U**.G...**U**..**G****G**U**G**..**C****U**AAA**U****C****C****U****G**...**C****A****G****C**--....................AAAC...........--**G****C****U****G**.AU.**A**..**G****A**..**U****G****A****G****A****A****U****A****A**UCGC | |
|  |  | NC\_004722.1/1394794-1394913  | UGAA**A****C****C****U****U****C****U****U**.**A****U****A**A.AGA**G**.**A****G****G****C**.**G****G****A****G****G****G**A...**C****U****G**.**G**.**C****C****C**UAC**G****A****U**.**G****C****C****U**.**C****G****G****C**AG**C****G**.**G**.**A****C****U****C****G**........................AUUUC....................................................**A****G****A****G****U**.G...**C**..**U****G**U**G**..**C****C**AAA**U****C****C****A****G**...**C****A****A****G****C**-....................GUGU...........-**G****C****U****U****G**.AA.**A**..**G****A**..**U****G****A****G****A****A****G****A****G**UGUU | |
|  |  | NC\_003909.8/5120384-5120265  | UUGC**A****U****A****G****U****C****U****U**.**A****U****C**A.AGA**A**A**A****G****G****U**.**G****G****A****G****G****G**A...**C****A****G**.**G**.**C****C****C**GAU**G****A****A**.**A****C****C****U**.**U****G****G****C**AA**C****A**.**G**.-**C****C****G**-........................UAUAA....................................................-**C****G****G****A**.A...**U**..**U****G**U**G**..**C****C**AAA**U****C****C****U****G**...**C****A****G****G****U****A**....................AUAA...........**A****C****C****C****U****G**.AG.**A**..**G****A**..**U****A****A****G****A****A****A****G****A**GCCU | |
|  |  | NC\_003997.3/5140331-5140212  | UUGC**A****U****A****G****U****C****U****U**.**A****U****C**A.AGA**A**A**A****G****G****U**.**G****G****A****G****G****G**A...**C****A****G**.**G**.**C****C****C**GAU**G****A****A**.**A****C****C****U**.**U****G****G****C**AA**C****A**.**G**.-**C****C****G**-........................UAUAA....................................................-**C****G****G****A**.A...**U**..**U****G**U**G**..**C****C**AAA**U****C****C****U****G**...**C****A****G****G****U****A**....................AUAA...........**A****U****C****C****U****G**.AG.**A**..**G****A**..**U****A****A****G****A****A****A****G****A**GCCU | |
|  |  | NC\_005945.1/5141702-5141583  | UUGC**A****U****A****G****U****C****U****U**.**A****U****C**A.AGA**A**A**A****G****G****U**.**G****G****A****G****G****G**A...**C****A****G**.**G**.**C****C****C**GAU**G****A****A**.**A****C****C****U**.**U****G****G****C**AA**C****A**.**G**.-**C****C****G**-........................UAUAA....................................................-**C****G****G****A**.A...**U**..**U****G**U**G**..**C****C**AAA**U****C****C****U****G**...**C****A****G****G****U****A**....................AUAA...........**A****U****C****C****U****G**.AG.**A**..**G****A**..**U****A****A****G****A****A****A****G****A**GCCU | |
|  |  | NC\_005957.1/5148712-5148593  | UUGC**A****U****A****G****U****C****U****U**.**A****U****C**A.AGA**A**A**A****G****G****U**.**G****G****A****G****G****G**A...**C****A****G**.**G**.**C****C****C**GAU**G****A****A**.**A****C****C****U**.**U****G****G****C**AA**C****A**.**G**.-**C****C****G**-........................UAUAA....................................................-**C****G****G****A**.A...**U**..**U****G**U**G**..**C****C**AAA**U****C****C****U****G**...**C****A****G****G****U****A**....................AUAA...........**A****U****C****C****U****G**.AG.**A**..**G****A**..**U****A****A****G****A****A****A****G****A**GCCU | |
|  |  | NC\_006274.1/5213250-5213131  | UUGC**A****U****A****G****U****C****U****U**.**A****U****C**A.AGA**A**A**A****G****G****U**.**G****G****A****G****G****G**A...**C****A****G**.**G**.**C****C****C**GAU**G****A****A**.**A****C****C****U**.**U****G****G****C**AA**C****A**.**G**.-**C****C****G**-........................UAUAA....................................................-**C****G****G****A**.A...**U**..**U****G**U**G**..**C****C**AAA**U****C****C****U****G**...**C****A****G****G****U****A**....................AUAA...........**A****U****C****C****U****G**.AG.**A**..**G****A**..**U****A****A****G****A****A****A****G****A**GCCU | |
|  |  | NC\_007530.2/5140457-5140338  | UUGC**A****U****A****G****U****C****U****U**.**A****U****C**A.AGA**A**A**A****G****G****U**.**G****G****A****G****G****G**A...**C****A****G**.**G**.**C****C****C**GAU**G****A****A**.**A****C****C****U**.**U****G****G****C**AA**C****A**.**G**.-**C****C****G**-........................UAUAA....................................................-**C****G****G****A**.A...**U**..**U****G**U**G**..**C****C**AAA**U****C****C****U****G**...**C****A****G****G****U****A**....................AUAA...........**A****U****C****C****U****G**.AG.**A**..**G****A**..**U****A****A****G****A****A****A****G****A**GCCU | |
|  |  | NZ\_AAAC02000001.1/481669-481550  | UUGC**A****U****A****G****U****C****U****U**.**A****U****C**A.AGA**A**A**A****G****G****U**.**G****G****A****G****G****G**A...**C****A****G**.**G**.**C****C****C**GAU**G****A****A**.**A****C****C****U**.**U****G****G****C**AA**C****A**.**G**.-**C****C****G**-........................UAUAA....................................................-**C****G****G****A**.A...**U**..**U****G**U**G**..**C****C**AAA**U****C****C****U****G**...**C****A****G****G****U****A**....................AUAA...........**A****U****C****C****U****G**.AG.**A**..**G****A**..**U****A****A****G****A****A****A****G****A**GCCU | |
|  |  | NZ\_AAEN01000025.1/97781-97900  | UUGC**A****U****A****G****U****C****U****U**.**A****U****C**A.AGA**A**A**A****G****G****U**.**G****G****A****G****G****G**A...**C****A****G**.**G**.**C****C****C**GAU**G****A****A**.**A****C****C****U**.**U****G****G****C**AA**C****A**.**G**.-**C****C****G**-........................UAUAA....................................................-**C****G****G****A**.A...**U**..**U****G**U**G**..**C****C**AAA**U****C****C****U****G**...**C****A****G****G****U****A**....................AUAA...........**A****U****C****C****U****G**.AG.**A**..**G****A**..**U****A****A****G****A****A****A****G****A**GCCU | |
|  |  | NZ\_AAEO01000026.1/48254-48135  | UUGC**A****U****A****G****U****C****U****U**.**A****U****C**A.AGA**A**A**A****G****G****U**.**G****G****A****G****G****G**A...**C****A****G**.**G**.**C****C****C**GAU**G****A****A**.**A****C****C****U**.**U****G****G****C**AA**C****A**.**G**.-**C****C****G**-........................UAUAA....................................................-**C****G****G****A**.A...**U**..**U****G**U**G**..**C****C**AAA**U****C****C****U****G**...**C****A****G****G****U****A**....................AUAA...........**A****U****C****C****U****G**.AG.**A**..**G****A**..**U****A****A****G****A****A****A****G****A**GCCU | |
|  |  | NZ\_AAEP01000040.1/89696-89577  | UUGC**A****U****A****G****U****C****U****U**.**A****U****C**A.AGA**A**A**A****G****G****U**.**G****G****A****G****G****G**A...**C****A****G**.**G**.**C****C****C**GAU**G****A****A**.**A****C****C****U**.**U****G****G****C**AA**C****A**.**G**.-**C****C****G**-........................UAUAA....................................................-**C****G****G****A**.A...**U**..**U****G**U**G**..**C****C**AAA**U****C****C****U****G**...**C****A****G****G****U****A**....................AUAA...........**A****U****C****C****U****G**.AG.**A**..**G****A**..**U****A****A****G****A****A****A****G****A**GCCU | |
|  |  | NZ\_AAEQ01000033.1/96995-97114  | UUGC**A****U****A****G****U****C****U****U**.**A****U****C**A.AGA**A**A**A****G****G****U**.**G****G****A****G****G****G**A...**C****A****G**.**G**.**C****C****C**GAU**G****A****A**.**A****C****C****U**.**U****G****G****C**AA**C****A**.**G**.-**C****C****G**-........................UAUAA....................................................-**C****G****G****A**.A...**U**..**U****G**U**G**..**C****C**AAA**U****C****C****U****G**...**C****A****G****G****U****A**....................AUAA...........**A****U****C****C****U****G**.AG.**A**..**G****A**..**U****A****A****G****A****A****A****G****A**GCCU | |
|  |  | NZ\_AAER01000033.1/17373-17254  | UUGC**A****U****A****G****U****C****U****U**.**A****U****C**A.AGA**A**A**A****G****G****U**.**G****G****A****G****G****G**A...**C****A****G**.**G**.**C****C****C**GAU**G****A****A**.**A****C****C****U**.**U****G****G****C**AA**C****A**.**G**.-**C****C****G**-........................UAUAA....................................................-**C****G****G****A**.A...**U**..**U****G**U**G**..**C****C**AAA**U****C****C****U****G**...**C****A****G****G****U****A**....................AUAA...........**A****U****C****C****U****G**.AG.**A**..**G****A**..**U****A****A****G****A****A****A****G****A**GCCU | |
|  |  | NZ\_AAES01000038.1/47306-47187  | UUGC**A****U****A****G****U****C****U****U**.**A****U****C**A.AGA**A**A**A****G****G****U**.**G****G****A****G****G****G**A...**C****A****G**.**G**.**C****C****C**GAU**G****A****A**.**A****C****C****U**.**U****G****G****C**AA**C****A**.**G**.-**C****C****G**-........................UAUAA....................................................-**C****G****G****A**.A...**U**..**U****G**U**G**..**C****C**AAA**U****C****C****U****G**...**C****A****G****G****U****A**....................AUAA...........**A****U****C****C****U****G**.AG.**A**..**G****A**..**U****A****A****G****A****A****A****G****A**GCCU | |
|  |  | NC\_006510.1/745492-745376  | ACCG**G****C****A****U****U****C****U****U**.**A****U****C**A.AGA**G**.**A****G****G****G**.**G****G****A****G****G****G**A...**C****U****G**.**G**.**C****C****C**GGU**G****A****A**.**C****C****C****U**.**C****A****G****C**AA**C****C**.**U**.**G****G****C****C**-........................-CGC-....................................................-**G****G****C****C**.A...**A**..**G****G**U**G**..**C****U**AAA**U****C****C****A****G**A..**C****A****G****G****C**-....................GGAA...........-**G****C****C****U****G**.GA.**A**..**G****A**..**U****A****A****G****A****A****G****A****A**GCGA | |
|  |  | NC\_003210.1/2491183-2491051  | UGUA**G****A****A****A****U****C****U****U**.**A****U****C**C.AGA**G**.**U****G****G****U**.**G****G****A****G****G****G**A...**A****A****U**.**G**.**C****C****C**UAU**G****A****A**.**G****C****C**-.**C****A****G****C**AA**C****C**.**U**.**A****A****A****C****A**AUAAUUC.................AUUAU...................................................G**U****G****U****U****U**.A...**A**..**G****G**U**G**..**C****U**AAG**U****C****A****U****G**...**C****A****G****A****A****C**AAC.................UAAU..........U**G****U****U****C****U****G**.AA.**A**..**G****A**..**U****G****A****G****A****A****G****G****A**AGUU | |
|  |  | NZ\_AADQ01000012.1/61193-61061  | UGUA**G****A****A****A****U****C****U****U**.**A****U****C**C.AGA**G**.**U****G****G****U**.**G****G****A****G****G****G**A...**A****A****U**.**G**.**C****C****C**UAU**G****A****A**.**G****C****C**-.**C****A****G****C**AA**C****C**.**U**.**A****A****A****C****A**AUAAUUC.................AUUAU...................................................G**U****G****U****U****U**.A...**A**..**G****G**U**G**..**C****U**AAG**U****C****A****U****G**...**C****A****G****A****A****C**AAC.................UAAU..........U**G****U****U****C****U****G**.AA.**A**..**G****A**..**U****G****A****G****A****A****G****G****A**AGUU | |
|  |  | NC\_006270.2/4014236-4014359  | AAGG**U****U****U****U****C****C****U****U**.**A****U****C**A.AGA**G**.**U****G****G****U**.**G****G****A****G****G****G**A...**C****U****G**.**G**.**C****C****C**UGU**G****A****A**.**A****C****C**-.**C****G****G****C**AA**C****C**.**G**.-**C****U****G****U**........................CUAUG....................................................**A****C****A****G****A**.A...**U**..**G****G**U**G**..**C****U**AAA**U****C****C****U****U**...**A****A****G****A****G****C**A...................UGUU........CGU**G****C****U****C****U****U**.GA.**A**..**G****A**..**U****A****A****G****G****A****G****G****A**GAUU | |
|  |  | NC\_006322.1/4014350-4014473  | AAGG**U****U****U****U****C****C****U****U**.**A****U****C**A.AGA**G**.**U****G****G****U**.**G****G****A****G****G****G**A...**C****U****G**.**G**.**C****C****C**UGU**G****A****A**.**A****C****C**-.**C****G****G****C**AA**C****C**.**G**.-**C****U****G****U**........................CUAUG....................................................**A****C****A****G****A**.A...**U**..**G****G**U**G**..**C****U**AAA**U****C****C****U****U**...**A****A****G****A****G****C**A...................UGUU........CGU**G****C****U****C****U****U**.GA.**A**..**G****A**..**U****A****A****G****G****A****G****G****A**GAUU | |
|  |  | NC\_004722.1/5321943-5321825  | UUGC**A****U****A****G****U****C****U****U**.**A****U****C**A.AGA**A**A**A****G****G****U**.**G****G****A****G****G****G**A...**C****A****G**.**G**.**C****C****C**GAU**G****A****A**.**A****C****C****U**.**U****G****G****C**AA**C****A**.**G**.-**C****C****G**-........................UAUAA....................................................-**C****G****G****A**.A...**U**..**U****G**U**G**..**C****C**AAA**U****C****C****U****G**...**C****A****G****G**--UA..................GUAA..........U--**C****C****U****G**.AG.**A**..**G****A**..**U****A****A****G****A****A****A****G****A**GCCU | |
|  |  | NC\_006274.1/3927244-3927089  | UAUA**U****A****A****C****U****C****U****U**.**A****U****C**A.AGA**G**C**A****G****G****U**.**G****G****A****G****G****G**AU..**U****U****G**.**G**.**C****C****C**GAU**G****A****A**.**G****C****C**-.**C****A****G****C**AA**C****C**.**G**.**A****C****C****G****U**AAUACCAUUGUGAAAUGGGGCG..UUUAU............................................GACGCCAA**A****A****G****G****C**.A...**C**..**G****G**U**G**..**C****U**AAU**U****C****C****A****G**...**C****A****G****A****A****A**G...................UAAA.........AC**U****U****U****C****U****G**.GC.**A**..**G****A**..**U****A****A****G****A****G****G****G****G**AGAA | |
|  |  | NC\_003997.3/3091685-3091573  | GAAU**A****U****U****U****U****C****U****U**.**A****U****C**C.AGA**G**.**A****G****G****U**.**G****G****A****G****G****G**A...**C****U****G**.**G**.**C****C****C**GAU**G****A****A**.**A****C****C**-.**C****A****G****C**AA**C****C**.-.**G****C**---........................-GAU-....................................................---**G****C**.A...-..**G****G**U**G**..**C****U**AAU**U****C****C****A****G**...**C****A****G****A****A****C**A...................AAUU..........U**G****U****U****C****U****G**.GG.**A**..**G****A**..**U****A****A****G****A****C****G****A****A**GAUA | |
|  |  | NC\_005945.1/3092379-3092267  | GAAU**A****U****U****U****U****C****U****U**.**A****U****C**C.AGA**G**.**A****G****G****U**.**G****G****A****G****G****G**A...**C****U****G**.**G**.**C****C****C**GAU**G****A****A**.**A****C****C**-.**C****A****G****C**AA**C****C**.-.**G****C**---........................-GAU-....................................................---**G****C**.A...-..**G****G**U**G**..**C****U**AAU**U****C****C****A****G**...**C****A****G****A****A****C**A...................AAUU..........U**G****U****U****C****U****G**.GG.**A**..**G****A**..**U****A****A****G****A****C****G****A****A**GAUA | |
|  |  | NC\_006274.1/3134611-3134499  | GAAU**A****U****U****U****U****C****U****U**.**A****U****C**C.AGA**G**.**A****G****G****U**.**G****G****A****G****G****G**A...**C****U****G**.**G**.**C****C****C**GAU**G****A****A**.**A****C****C**-.**C****A****G****C**AA**C****C**.-.**G****C**---........................-GAU-....................................................---**G****C**.A...-..**G****G**U**G**..**C****U**AAU**U****C****C****A****G**...**C****A****G****A****A****C**A...................AAUU..........U**G****U****U****C****U****G**.GG.**A**..**G****A**..**U****A****A****G****A****C****G****A****A**GAUA | |
|  |  | NC\_007530.2/3091813-3091701  | GAAU**A****U****U****U****U****C****U****U**.**A****U****C**C.AGA**G**.**A****G****G****U**.**G****G****A****G****G****G**A...**C****U****G**.**G**.**C****C****C**GAU**G****A****A**.**A****C****C**-.**C****A****G****C**AA**C****C**.-.**G****C**---........................-GAU-....................................................---**G****C**.A...-..**G****G**U**G**..**C****U**AAU**U****C****C****A****G**...**C****A****G****A****A****C**A...................AAUU..........U**G****U****U****C****U****G**.GG.**A**..**G****A**..**U****A****A****G****A****C****G****A****A**GAUA | |
|  |  | NZ\_AAAC02000001.1/3556137-3556025  | GAAU**A****U****U****U****U****C****U****U**.**A****U****C**C.AGA**G**.**A****G****G****U**.**G****G****A****G****G****G**A...**C****U****G**.**G**.**C****C****C**GAU**G****A****A**.**A****C****C**-.**C****A****G****C**AA**C****C**.-.**G****C**---........................-GAU-....................................................---**G****C**.A...-..**G****G**U**G**..**C****U**AAU**U****C****C****A****G**...**C****A****G****A****A****C**A...................AAUU..........U**G****U****U****C****U****G**.GG.**A**..**G****A**..**U****A****A****G****A****C****G****A****A**GAUA | |
|  |  | NZ\_AAEN01000017.1/327822-327710  | GAAU**A****U****U****U****U****C****U****U**.**A****U****C**C.AGA**G**.**A****G****G****U**.**G****G****A****G****G****G**A...**C****U****G**.**G**.**C****C****C**GAU**G****A****A**.**A****C****C**-.**C****A****G****C**AA**C****C**.-.**G****C**---........................-GAU-....................................................---**G****C**.A...-..**G****G**U**G**..**C****U**AAU**U****C****C****A****G**...**C****A****G****A****A****C**A...................AAUU..........U**G****U****U****C****U****G**.GG.**A**..**G****A**..**U****A****A****G****A****C****G****A****A**GAUA | |
|  |  | NZ\_AAEO01000022.1/1050709-1050597  | GAAU**A****U****U****U****U****C****U****U**.**A****U****C**C.AGA**G**.**A****G****G****U**.**G****G****A****G****G****G**A...**C****U****G**.**G**.**C****C****C**GAU**G****A****A**.**A****C****C**-.**C****A****G****C**AA**C****C**.-.**G****C**---........................-GAU-....................................................---**G****C**.A...-..**G****G**U**G**..**C****U**AAU**U****C****C****A****G**...**C****A****G****A****A****C**A...................AAUU..........U**G****U****U****C****U****G**.GG.**A**..**G****A**..**U****A****A****G****A****C****G****A****A**GAUA | |
|  |  | NZ\_AAEP01000026.1/377495-377383  | GAAU**A****U****U****U****U****C****U****U**.**A****U****C**C.AGA**G**.**A****G****G****U**.**G****G****A****G****G****G**A...**C****U****G**.**G**.**C****C****C**GAU**G****A****A**.**A****C****C**-.**C****A****G****C**AA**C****C**.-.**G****C**---........................-GAU-....................................................---**G****C**.A...-..**G****G**U**G**..**C****U**AAU**U****C****C****A****G**...**C****A****G****A****A****C**A...................AAUU..........U**G****U****U****C****U****G**.GG.**A**..**G****A**..**U****A****A****G****A****C****G****A****A**GAUA | |
|  |  | NZ\_AAEQ01000035.1/141676-141788  | GAAU**A****U****U****U****U****C****U****U**.**A****U****C**C.AGA**G**.**A****G****G****U**.**G****G****A****G****G****G**A...**C****U****G**.**G**.**C****C****C**GAU**G****A****A**.**A****C****C**-.**C****A****G****C**AA**C****C**.-.**G****C**---........................-GAU-....................................................---**G****C**.A...-..**G****G**U**G**..**C****U**AAU**U****C****C****A****G**...**C****A****G****A****A****C**A...................AAUU..........U**G****U****U****C****U****G**.GG.**A**..**G****A**..**U****A****A****G****A****C****G****A****A**GAUA | |
|  |  | NZ\_AAER01000040.1/822979-822867  | GAAU**A****U****U****U****U****C****U****U**.**A****U****C**C.AGA**G**.**A****G****G****U**.**G****G****A****G****G****G**A...**C****U****G**.**G**.**C****C****C**GAU**G****A****A**.**A****C****C**-.**C****A****G****C**AA**C****C**.-.**G****C**---........................-GAU-....................................................---**G****C**.A...-..**G****G**U**G**..**C****U**AAU**U****C****C****A****G**...**C****A****G****A****A****C**A...................AAUU..........U**G****U****U****C****U****G**.GG.**A**..**G****A**..**U****A****A****G****A****C****G****A****A**GAUA | |
|  |  | NZ\_AAES01000035.1/992448-992336  | GAAU**A****U****U****U****U****C****U****U**.**A****U****C**C.AGA**G**.**A****G****G****U**.**G****G****A****G****G****G**A...**C****U****G**.**G**.**C****C****C**GAU**G****A****A**.**A****C****C**-.**C****A****G****C**AA**C****C**.-.**G****C**---........................-GAU-....................................................---**G****C**.A...-..**G****G**U**G**..**C****U**AAU**U****C****C****A****G**...**C****A****G****A****A****C**A...................AAUU..........U**G****U****U****C****U****G**.GG.**A**..**G****A**..**U****A****A****G****A****C****G****A****A**GAUA | |
|  |  | NC\_003909.8/3108659-3108547  | GAAU**A****U****U****U****U****C****U****U**.**A****U****C**C.AGA**G**.**A****G****G****U**.**G****G****A****G****G****G**A...**C****U****G**.**G**.**C****C****C**GAU**G****A****A**.**A****C****C**-.**C****A****G****C**AA**C****C**.-.**G****C**---........................-GAU-....................................................---**G****C**.A...-..**G****G**U**G**..**C****U**AAU**U****C****C****A****G**...**C****A****G****A****A****C**A...................UAUU..........U**G****U****U****C****U****G**.GG.**A**..**G****A**..**U****A****A****G****A****C****G****A****A**GAUA | |
|  |  | NC\_005957.1/3170768-3170656  | GAAU**A****U****U****U****U****C****U****U**.**A****U****C**C.AGA**G**.**A****G****G****U**.**G****G****A****G****G****G**A...**C****U****G**.**G**.**C****C****C**GAU**G****A****A**.**A****C****C**-.**C****A****G****C**AA**C****C**.-.**G****C**---........................-GAU-....................................................---**G****C**.A...-..**G****G**U**G**..**C****U**AAU**U****C****C****A****G**...**C****A****G****A****A****C**A...................UAUU..........G**G****U****U****C****U****G**.GG.**A**..**G****A**..**U****A****A****G****A****C****G****A****A**GAUA | |
|  |  | NZ\_AAEK01000001.1/196687-196799  | GAAU**A****U****U****U****U****C****U****U**.**A****U****C**C.AGA**G**.**A****G****G****U**.**G****G****A****G****G****G**A...**C****U****G**.**G**.**C****C****C**GAU**G****A****A**.**A****C****C**-.**C****A****G****C**AA**C****C**.-.**G****C**---........................-GAU-....................................................---**G****C**.A...-..**G****G**U**G**..**C****U**AAU**U****C****C****A****G**...**C****A****G****A****A****C**A...................UAUU..........U**G****U****U****C****U****G**.GG.**A**..**G****A**..**U****A****A****G****A****C****G****A****A**GAUA | |
|  |  | NC\_005957.1/3870225-3870070  | UAUA**C****A****A****C****U****C****U****U**.**A****U****C**A.AGA**G**C**A****G****G****U**.**G****G****A****G****G****G**AU..**U****U****G**.**G**.**C****C****C**UAU**G****A****A**.**G****C****C**-.**C****A****G****C**AA**C****C**.**G**.**A****C****C****G****U**AAUACCAUUGUGAAAUGGGGCG..UUUAU............................................GACGCCAA**A****A****G****G****C**.A...**C**..**G****G**U**G**..**C****U**AAU**U****C****C****A****G**...**C****A****G****A****A****A**G...................UAAA.........AC**U****U****U****C****U****G**.GC.**A**..**G****A**..**U****A****A****G****A****G****G****G****G**AGAA | |
|  |  | NC\_002973.5/2444955-2444823  | UGUA**G****A****A****A****U****C****U****U**.**A****U****C**C.AGA**G**.**U****G****G****U**.**G****G****A****G****G****G**A...**A****A****U**.**G**.**C****C****C**UGU**G****A****A**.**A****C****C**-.**C****A****G****C**AA**C****C**.**U**.**A****A****A****C****A**AUAAUUC.................AUUAU...................................................G**U****G****U****U****U**.A...**A**..**G****G**U**G**..**C****U**AAG**U****C****A****U****G**...**C****A****G****A****A****C**AAC.................UAAU..........U**G****U****U****C****U****G**.AA.**A**..**G****A**..**U****G****A****G****A****A****G****G****A**AGUU | |
|  |  | NZ\_AADR01000018.1/36157-36025  | UGUA**G****A****A****A****U****C****U****U**.**A****U****C**C.AGA**G**.**U****G****G****U**.**G****G****A****G****G****G**A...**A****A****U**.**G**.**C****C****C**UGU**G****A****A**.**A****C****C**-.**C****A****G****C**AA**C****C**.**U**.**A****A****A****C****A**AUAAUUC.................AUUAU...................................................G**U****G****U****U****U**.A...**A**..**G****G**U**G**..**C****U**AAG**U****C****A****U****G**...**C****A****G****A****A****C**AAC.................UAAU..........U**G****U****U****C****U****G**.AA.**A**..**G****A**..**U****G****A****G****A****A****G****G****A**AGUU | |
|  |  | NC\_006270.2/1206885-1206760  | AUAG**C****U****G****U****U****C****U****U**.**A****U****C**A.AGA**G**.**A****G****G****C**.**A****G****A****G****G****G**A...**C****U****G**.**G**.**C****C****C**GAU**G****A****A**.**G****C****C****U**.**C****A****G****C**AA**C****C**.-.**G****G****U****G****A**AUG.....................AAUAU.................................................UCA**U****G****A****C****C**.A...**A**..**G****G**U**G**..**C****U**AAA**U****C****C****A****G**...**C****A****A****G****C****A**....................-GCC...........**U****G****C****U****U****G**.GA.**A**..**G****A**..**U****A****A****G****A****A****G****A****C**GGAC | |
|  |  | NC\_006322.1/1207740-1207615  | AUAG**C****U****G****U****U****C****U****U**.**A****U****C**A.AGA**G**.**A****G****G****C**.**A****G****A****G****G****G**A...**C****U****G**.**G**.**C****C****C**GAU**G****A****A**.**G****C****C****U**.**C****A****G****C**AA**C****C**.-.**G****G****U****G****A**AUG.....................AAUAU.................................................UCA**U****G****A****C****C**.A...**A**..**G****G**U**G**..**C****U**AAA**U****C****C****A****G**...**C****A****A****G****C****A**....................-GCC...........**U****G****C****U****U****G**.GA.**A**..**G****A**..**U****A****A****G****A****A****G****A****C**GGAC | |
|  |  | NC\_003997.3/3890745-3890590  | UAUA**C****A****A****C****U****C****U****U**.**A****U****C**A.AGA**G**C**A****G****G****U**.**G****G****A****G****G****G**AU..**U****U****G**.**G**.**C****C****C**GAU**G****A****A**.**G****C****C**-.**C****A****G****C**AA**C****C**.**G**.**A****C****C****G****U**AAUACCAUUGUGAAAUGGGGCG..UUUAU............................................GACGCCAA**A****A****G****G****C**.A...**C**..**G****G**U**G**..**C****U**AAU**U****C****C****A****G**...**C****A****G****A****A****A**G...................UAAA.........AC**U****U****U****C****U****G**.GC.**A**..**G****A**..**U****A****A****G****A****G****G****G****G**AGAA | |
|  |  | NC\_005945.1/3891245-3891090  | UAUA**C****A****A****C****U****C****U****U**.**A****U****C**A.AGA**G**C**A****G****G****U**.**G****G****A****G****G****G**AU..**U****U****G**.**G**.**C****C****C**GAU**G****A****A**.**G****C****C**-.**C****A****G****C**AA**C****C**.**G**.**A****C****C****G****U**AAUACCAUUGUGAAAUGGGGCG..UUUAU............................................GACGCCAA**A****A****G****G****C**.A...**C**..**G****G**U**G**..**C****U**AAU**U****C****C****A****G**...**C****A****G****A****A****A**G...................UAAA.........AC**U****U****U****C****U****G**.GC.**A**..**G****A**..**U****A****A****G****A****G****G****G****G**AGAA | |
|  |  | NC\_007530.2/3890872-3890717  | UAUA**C****A****A****C****U****C****U****U**.**A****U****C**A.AGA**G**C**A****G****G****U**.**G****G****A****G****G****G**AU..**U****U****G**.**G**.**C****C****C**GAU**G****A****A**.**G****C****C**-.**C****A****G****C**AA**C****C**.**G**.**A****C****C****G****U**AAUACCAUUGUGAAAUGGGGCG..UUUAU............................................GACGCCAA**A****A****G****G****C**.A...**C**..**G****G**U**G**..**C****U**AAU**U****C****C****A****G**...**C****A****G****A****A****A**G...................UAAA.........AC**U****U****U****C****U****G**.GC.**A**..**G****A**..**U****A****A****G****A****G****G****G****G**AGAA | |
|  |  | NZ\_AAAC02000001.1/4340399-4340244  | UAUA**C****A****A****C****U****C****U****U**.**A****U****C**A.AGA**G**C**A****G****G****U**.**G****G****A****G****G****G**AU..**U****U****G**.**G**.**C****C****C**GAU**G****A****A**.**G****C****C**-.**C****A****G****C**AA**C****C**.**G**.**A****C****C****G****U**AAUACCAUUGUGAAAUGGGGCG..UUUAU............................................GACGCCAA**A****A****G****G****C**.A...**C**..**G****G**U**G**..**C****U**AAU**U****C****C****A****G**...**C****A****G****A****A****A**G...................UAAA.........AC**U****U****U****C****U****G**.GC.**A**..**G****A**..**U****A****A****G****A****G****G****G****G**AGAA | |
|  |  | NZ\_AAEK01000007.1/34205-34360  | UAUA**C****A****A****C****U****C****U****U**.**A****U****C**A.AGA**G**C**A****G****G****U**.**G****G****A****G****G****G**AU..**U****U****G**.**G**.**C****C****C**GAU**G****A****A**.**G****C****C**-.**C****A****G****C**AA**C****C**.**G**.**A****C****C****G****U**AAUACCAUUGUGAAAUGGGGCG..UUUAU............................................GACGCCAA**A****A****G****G****C**.A...**C**..**G****G**U**G**..**C****U**AAU**U****C****C****A****G**...**C****A****G****A****A****A**G...................UAAA.........AC**U****U****U****C****U****G**.GC.**A**..**G****A**..**U****A****A****G****A****G****G****G****G**AGAA | |
|  |  | NZ\_AAEN01000013.1/145255-145100  | UAUA**C****A****A****C****U****C****U****U**.**A****U****C**A.AGA**G**C**A****G****G****U**.**G****G****A****G****G****G**AU..**U****U****G**.**G**.**C****C****C**GAU**G****A****A**.**G****C****C**-.**C****A****G****C**AA**C****C**.**G**.**A****C****C****G****U**AAUACCAUUGUGAAAUGGGGCG..UUUAU............................................GACGCCAA**A****A****G****G****C**.A...**C**..**G****G**U**G**..**C****U**AAU**U****C****C****A****G**...**C****A****G****A****A****A**G...................UAAA.........AC**U****U****U****C****U****G**.GC.**A**..**G****A**..**U****A****A****G****A****G****G****G****G**AGAA | |
|  |  | NZ\_AAEO01000019.1/232361-232206  | UAUA**C****A****A****C****U****C****U****U**.**A****U****C**A.AGA**G**C**A****G****G****U**.**G****G****A****G****G****G**AU..**U****U****G**.**G**.**C****C****C**GAU**G****A****A**.**G****C****C**-.**C****A****G****C**AA**C****C**.**G**.**A****C****C****G****U**AAUACCAUUGUGAAAUGGGGCG..UUUAU............................................GACGCCAA**A****A****G****G****C**.A...**C**..**G****G**U**G**..**C****U**AAU**U****C****C****A****G**...**C****A****G****A****A****A**G...................UAAA.........AC**U****U****U****C****U****G**.GC.**A**..**G****A**..**U****A****A****G****A****G****G****G****G**AGAA | |
|  |  | NZ\_AAEP01000031.1/214733-214888  | UAUA**C****A****A****C****U****C****U****U**.**A****U****C**A.AGA**G**C**A****G****G****U**.**G****G****A****G****G****G**AU..**U****U****G**.**G**.**C****C****C**GAU**G****A****A**.**G****C****C**-.**C****A****G****C**AA**C****C**.**G**.**A****C****C****G****U**AAUACCAUUGUGAAAUGGGGCG..UUUAU............................................GACGCCAA**A****A****G****G****C**.A...**C**..**G****G**U**G**..**C****U**AAU**U****C****C****A****G**...**C****A****G****A****A****A**G...................UAAA.........AC**U****U****U****C****U****G**.GC.**A**..**G****A**..**U****A****A****G****A****G****G****G****G**AGAA | |
|  |  | NZ\_AAEQ01000038.1/166640-166485  | UAUA**C****A****A****C****U****C****U****U**.**A****U****C**A.AGA**G**C**A****G****G****U**.**G****G****A****G****G****G**AU..**U****U****G**.**G**.**C****C****C**GAU**G****A****A**.**G****C****C**-.**C****A****G****C**AA**C****C**.**G**.**A****C****C****G****U**AAUACCAUUGUGAAAUGGGGCG..UUUAU............................................GACGCCAA**A****A****G****G****C**.A...**C**..**G****G**U**G**..**C****U**AAU**U****C****C****A****G**...**C****A****G****A****A****A**G...................UAAA.........AC**U****U****U****C****U****G**.GC.**A**..**G****A**..**U****A****A****G****A****G****G****G****G**AGAA | |
|  |  | NZ\_AAER01000035.1/403590-403435  | UAUA**C****A****A****C****U****C****U****U**.**A****U****C**A.AGA**G**C**A****G****G****U**.**G****G****A****G****G****G**AU..**U****U****G**.**G**.**C****C****C**GAU**G****A****A**.**G****C****C**-.**C****A****G****C**AA**C****C**.**G**.**A****C****C****G****U**AAUACCAUUGUGAAAUGGGGCG..UUUAU............................................GACGCCAA**A****A****G****G****C**.A...**C**..**G****G**U**G**..**C****U**AAU**U****C****C****A****G**...**C****A****G****A****A****A**G...................UAAA.........AC**U****U****U****C****U****G**.GC.**A**..**G****A**..**U****A****A****G****A****G****G****G****G**AGAA | |
|  |  | NZ\_AAES01000024.1/143258-143103  | UAUA**C****A****A****C****U****C****U****U**.**A****U****C**A.AGA**G**C**A****G****G****U**.**G****G****A****G****G****G**AU..**U****U****G**.**G**.**C****C****C**GAU**G****A****A**.**G****C****C**-.**C****A****G****C**AA**C****C**.**G**.**A****C****C****G****U**AAUACCAUUGUGAAAUGGGGCG..UUUAU............................................GACGCCAA**A****A****G****G****C**.A...**C**..**G****G**U**G**..**C****U**AAU**U****C****C****A****G**...**C****A****G****A****A****A**G...................UAAA.........AC**U****U****U****C****U****G**.GC.**A**..**G****A**..**U****A****A****G****A****G****G****G****G**AGAA | |
|  |  | NC\_000964.2/3998177-3998297  | AAGG**U****U****U****U****C****C****U****U**.**A****U****C**A.AGA**G**.**A****G****G****U**.**G****G****A****G****G****G**A...**C****U****G**.**G**.**C****C****C**UGC**G****A****U**.**A****C****C**-.**C****G****G****C**AA**C****C**.**G**.-**C****U****G**-........................UUUAA....................................................-**C****A****G****A**.A...**U**..**G****G**U**G**..**C****U**AAA**U****C****C****U****U**...**U****A****G****A****G****C**AA..................UGAU..........U**G****C****U****C****U****U**.GA.**A**..**G****A**..**U****A****A****G****G****U****U****G****A**GAUU | |
|  |  | NC\_003212.1/2538261-2538129  | UGUA**G****A****A****A****U****C****U****U**.**A****U****C**C.AGA**G**.**U****G****G****U**.**G****G****A****G****G****G**A...**A****A****U**.**G**.**C****C****C**UGU**G****A****A**.**A****C****C**-.**C****A****G****C**AA**C****C**.**U**.**A****A****A****C****A**AUAAUUC.................AUUAU...................................................G**U****G****U****U****U**.A...**A**..**G****G**U**G**..**C****U**AAG**U****C****A****U****G**...**C****A****G****A****A****C**AAC.................GAUU..........U**G****U****U****C****U****G**.AA.**A**..**G****A**..**U****G****A****G****A****A****G****G****A**AGUU | |
|  |  | NC\_004193.1/3294465-3294595  | ACGU**U****U****U****U****U****C****U****U**.**A****U****C**U.AGA**G**.**A****G****A****U**.**U****G****A****G****G****G**AU..**C****A****G**.**G**.**C****C****C**UAU**G****A****C**.**A****U****C****U**.**C****G****G****C**AG**C****G**.**G**.**A****U****U****C****U**UUAU....................AUUAA....................................................**A****G****A****A****U**.A...**C**..**U****G**U**G**..**C****C**AAU**U****C****C****U****G**...**C****A****A****A****U****G**C...................AAAC........GAG**C****A****U****U****U****G**.AA.**A**..**G****A**..**U****G****A****G****A****A****A****C****G**AUGG | |
|  |  | NC\_003909.8/3833860-3833704  | UAUA**C****A****A****C****U****C****U****U**.**A****U****C**A.AGA**G**C**A****G****G****U**.**G****G****A****G****G****G**AU..**U****U****G**.**G**.**C****C****C**GAU**G****A****A**.**G****C****C**-.**C****A****G****C**AA**C****C**.**G**.**A****C****C****G****U**AAUACCAUUGUGAAAUGGGGCG..UUUAU...........................................UUACGCCAA**A****A****G****G****C**.A...**C**..**G****G**U**G**..**C****U**AAU**U****C****C****A****G**...**C****A****G****A****A****A**G...................UAAA.........AC**U****U****U****C****U****G**.GC.**A**..**G****A**..**U****A****A****G****A****G****G****G****G**AGAA | |
|  |  | NC\_003366.1/2500090-2499971  | UUAU**A****U****A****C****U****C****U****U**.**A****U****C**C.AGA**G**.**A****G****G****U**.**G****G****A****G****G****G**AAA.**A****A****G**.**G**.**C****C****C**UAU**G****A****A**.**A****C****C**-.**C****G****G****C**AA**C****C**.-.**A****G****U****G****A**........................GAAA-....................................................**U****C****A****C****U**.A...**C**..**G****G**U**G**..**C****C**AAU**U****C****C****G****G**...**U****A****A****A****G****A**....................-AAU...........**U****C****U****U****U****A**.CA.**A**..**G****A**..**U****G****A****G****A****G****A****A****G**AUAA | |
|  |  | NC\_000964.2/1257609-1257743  | UCGA**U****A****U****U****U****C****U****U**.**A****U****C**G.UGA**G**.**A****G****G****U**.**G****G****A****G****G****G**A...**C****U****G**.**G**.**C****C****C**UUA**G****A****A**.**A****C****C****U**.**C****A****G****C**AA**C****C**.-.**G****G****C****U****U**G.......................UUUU-.........................................GCAUUUGCAAA**G****C****G****C****C**.A...**A**..**G****G**U**G**..**C****U**AAA**U****C****C****A****G**...**C****A****A****G****C****G**U...................UUUU.........UA**U****G****C****U****U****G**.GA.**A**..**G****A**..**U****A****A****G****A****A****G****A****A**GCGU | |
|  |  | NC\_002973.5/644652-644538  | UAGU**A****U****U****U****U****C****U****U**.**A****U****C**A.CGA**A**.**A****G****G****U**.**G****G****A****G****G****G**A...**C****U****G**.**G**.**C****C****C**UUU**G****A****A**.**G****C****C****U**.**U****A****G****C**AA**C****C**.**G**.-**G****A****A**-........................UUUAU....................................................-**U****U****U****C**.A...**C**..**G****G**U**G**..**C****U**AAU**U****C****C****A****G**...**C****A****G****U**--....................AUAU...........--**U****C****U****G**.AA.**A**..**G****A**..**U****A****A****G****U****C****G****G****A**AAUC | |
|  |  | NC\_003210.1/637933-637819  | UAGU**A****U****U****U****U****C****U****U**.**A****U****C**A.CGA**A**.**A****G****G****U**.**G****G****A****G****G****G**A...**C****U****G**.**G**.**C****C****C**UUU**G****A****A**.**G****C****C****U**.**U****A****G****C**AA**C****C**.**G**.-**G****A****A**-........................UUUAU....................................................-**U****U****U****C**.A...**C**..**G****G**U**G**..**C****U**AAU**U****C****C****A****G**...**C****A****G****U**--....................AUAU...........--**U****C****U****G**.AA.**A**..**G****A**..**U****A****A****G****U****C****G****G****A**AAUC | |
|  |  | NC\_003212.1/636921-636807  | UAGU**A****U****U****U****U****C****U****U**.**A****U****C**A.CGA**A**.**A****G****G****U**.**G****G****A****G****G****G**A...**C****U****G**.**G**.**C****C****C**UUU**G****A****A**.**G****C****C****U**.**U****A****G****C**AA**C****C**.**G**.-**G****A****A**-........................UUUAU....................................................-**U****U****U****C**.A...**C**..**G****G**U**G**..**C****U**AAU**U****C****C****A****G**...**C****A****G****U**--....................AUAU...........--**U****C****U****G**.AA.**A**..**G****A**..**U****A****A****G****U****C****G****G****A**AAUC | |
|  |  | NZ\_AADQ01000037.1/2075-2189  | UAGU**A****U****U****U****U****C****U****U**.**A****U****C**A.CGA**A**.**A****G****G****U**.**G****G****A****G****G****G**A...**C****U****G**.**G**.**C****C****C**UUU**G****A****A**.**G****C****C****U**.**U****A****G****C**AA**C****C**.**G**.-**G****A****A**-........................UUUAU....................................................-**U****U****U****C**.A...**C**..**G****G**U**G**..**C****U**AAU**U****C****C****A****G**...**C****A****G****U**--....................AUAU...........--**U****C****U****G**.AA.**A**..**G****A**..**U****A****A****G****U****C****G****G****A**AAUC | |
|  |  | NZ\_AADR01000029.1/1474-1588  | UAGU**A****U****U****U****U****C****U****U**.**A****U****C**A.CGA**A**.**A****G****G****U**.**G****G****A****G****G****G**A...**C****U****G**.**G**.**C****C****C**UUU**G****A****A**.**G****C****C****U**.**U****A****G****C**AA**C****C**.**G**.-**G****A****A**-........................UUUAU....................................................-**U****U****U****C**.A...**C**..**G****G**U**G**..**C****U**AAU**U****C****C****A****G**...**C****A****G****U**--....................AUAU...........--**U****C****U****G**.AA.**A**..**G****A**..**U****A****A****G****U****C****G****G****A**AAUC | |
|  |  | NC\_003210.1/1739604-1739484  | UAAA**U****U****G****C****U****C****U****U**.**A****U****A**A.UGA**G**.**U****G****G****U**.**A****G****A****G****G****G**A...**C****U****G**.**G**.**C****C****C**GUU**G****A****A**.**A****C****C**-.**C****G****G****C**AA**C****C**.**U**.**U****U****C****A****A**........................UACG-....................................................**U****U****G****A****A**.A...**A**..**G****G**U**G**..**C****U**AAA**U****C****C****U****G**...**C****G****A****A****G****U**....................GUGA..........U**G****C****U****U****C****G**.AG.**A**..**G****A**..**U****A****A****G****A****G****A****G****A**CUUA | |
|  |  | NZ\_AADQ01000032.1/6592-6712  | UAAA**U****U****G****C****U****C****U****U**.**A****U****A**A.UGA**G**.**U****G****G****U**.**A****G****A****G****G****G**A...**C****U****G**.**G**.**C****C****C**GUU**G****A****A**.**A****C****C**-.**C****G****G****C**AA**C****C**.**U**.**U****U****C****A****A**........................UACG-....................................................**U****U****G****A****A**.A...**A**..**G****G**U**G**..**C****U**AAA**U****C****C****U****G**...**C****G****A****A****G****U**....................GUGA..........U**G****C****U****U****C****G**.AG.**A**..**G****A**..**U****A****A****G****A****G****A****G****A**CUUA | |
|  |  | NC\_005957.1/768979-768860  | AUUC**G****A****U****G****U****C****U****U**.**A****U****C**A.AGA**G**C**A****G****G****U**.**G****G****A****G****G****G**A...**U****G****A**.**G**.**C****C****C**UAC**G****A****A**.**G****C****C**-.**C****G****G****C**AA**C****C**.**G**.**A****C****C****C****A**........................UUUA-....................................................**U****G****G****G****C**.A...**C**..**G****G**U**G**..**C****U**AAU**U****C****U****U****A**...**C****A****A****C****A****C**....................-AUU...........**G****U****G****U****U****G**.AA.**A**..**G****A**..**U****A****A****G****A****G****U****A****A**UAUG | |
|  |  | NC\_004722.1/755254-755135  | AUCG**G****A****U****G****U****C****U****U**.**A****U****C**A.AGA**G**C**A****G****G****U**.**G****G****A****G****G****G**A...**U****G****A**.**G**.**C****C****C**UAC**G****A****A**.**G****C****C**-.**C****G****G****C**AA**C****C**.**G**.**A****C****C****C****A**........................UUUA-....................................................**U****G****G****G****C**.A...**C**..**G****G**U**G**..**C****U**AAU**U****C****U****U****A**...**C****A****A****C****A****C**....................-AUU...........**G****U****G****U****U****G**.AA.**A**..**G****A**..**U****A****A****G****A****G****U****A****A**UAUG | |
|  |  | NZ\_AAEK01000017.1/12415-12297  | UUUA**C****U****C****A****U****U****G****U**.**A****U****C**A.AGA**G**.**A****G****G****U**.**G****G****A****G****G****G**A...**C****U****G**.**G**.**C****C****C**UUU**G****A****A**.**A****C****C****U**.**C****G****G****C**AA**C****A**.**G**.**G****U****U****C**-........................AUUUU....................................................-**G****A****A****U**.A...**C**..**U****G**U**G**..**C****C**ACC**U****C****C****U****G**...**C****A****A****G****C**-....................UUUG..........U-**G****C****U****U****G**.AA.**A**..**G****A**..**U****A****G****A****A****U****G****A****G**GGAC | |
|  |  | NC\_003869.1/500236-500359  | UAAC**A****C****G****C****U****C****U****U**.**A****U****C**A.AGA**G**.**A****G****G****U**.**G****G****A****G****G****G**AA..**A****G****A**.**G**.**C****C****C**GAU**G****A****A**.**A****C****C**-.**C****G****G****C**AA**C****C**.**U**.**G****U****C****C****U**........................UUUA-....................................................**A****G****G****A****U**.A...**A**..**G****G**U**G**..**C****C**AAU**U****C****U****C****U**...**C****A****G****A****A****G**A...................UUUU.........UU**C****U****U****C****U****G**.AA.**A**..**G****A**..**U****G****A****G****G****G****U****A****U**GCCU | |
|  |  | NC\_004193.1/1319034-1319148  | AUGA**A****A****A****U****A****C****U****U**.**A****U****C**A.AGA**G**.**A****G****G****U**.**G****G****A****G****G****G**A...**C****U****G**.**G**.**C****C****C**GCU**G****A****A**.**A****C****C****U**.**C****A****G****C**AA**C****A**.**G**A**A****C****G**--........................CAUC-....................................................--**U****G****U**.-...**C**..**U****G**U**G**..**C****U**AAA**U****C****C****U****G**...**C****A****A****G****C**-....................AAUA...........-**G****C****U****U****G**.AA.**A**..**G****A**..**U****A****A****G****U****U****G****A****G**GUUA | |
|  |  | NC\_003030.1/453556-453672  | UAAU**U****G****U****U****U****C****U****U**.**A****U****C**A.AGA**G**.**U****G****A****C**.**G****G****A****G****G****G**A...**U****A****G**.**G**.**C****C****C**UAU**G****A****A**.**G****U****C**-.**C****G****G****C**AA**C****A**.**U**.-**C****C****A****A**........................UUAUU....................................................**U****U****G****G****A**.G...**A**..**U****G**U**G**..**C****U**AAU**U****C****C****U****A**...**C****A****G****G****U**-....................-UUA...........-**U****C****C****U****G**.AG.**A**..**G****A**..**U****G****A****G****A****A****U****G****U**UUUU | |
|  |  | NC\_004193.1/3466527-3466394  | CUAA**U****A****U****C****U****C****U****U**.**A****U****U**G.AGA**G**.**U****G****G****C**.**U****G****A****G****G****G**A...**C****U****G**.**G**.**C****C****C**UGU**G****A****C**.**G****C****C**-.**C****G****G****C**AA**C****C**.**G**.**U****U****C****A****U**CG......................UAAUU..............................................CCAGUG**A****U****G****A****A**.U...**A**..**G****G**U**G**..**C****U**AAA**U****C****C****U****G**...**C****A****A****A****A****U**ACG.................GACA.........GU**A****U****U****U****U****G**.AG.**A**..**A****A**..**U****A****A****G****A****G****A****G****G**UGAU | |
|  |  | NC\_006582.1/1563575-1563693  | AAAA**A****C****A****C****U****C****U****U**.**A****U****A**A.CGA**G**.**A****A****G****C**.**G****G****A****G****G****G**A...**C****U****G**.**G**.**C****C****C**AAU**G****A****A**.**G****C****U****U**.**C****A****G****C**AA**C****C**.**A**.**U****U****C****A****U**........................UGCG-....................................................**A****U****G****A****A**.A...**A**..**G****G**U**G**..**C****U**AAA**U****C****C****A****G**...**C****A****A****A****G**-....................GGAA...........-**C****U****U****U****G**.GC.**A**..**G****A**..**U****A****A****G****G****G****G****A****U**UCAU | |
|  |  | NC\_000964.2/3996787-3996907  | AAGU**U****G****U****A****C****C****U****U**.**A****U****C**A.AGA**G**.**A****G****G****U**.**G****G****A****G****G****G**A...**C****U****G**.**G**.**C****C****C**UAU**G****A****U**.**A****C****C**-.**C****G****G****C**AA**C****C**.**G**.-**C****U****G****U**........................UUCAA....................................................**A****C****A****G****A**.A...**U**..**G****G**U**G**..**C****U**AAA**U****C****C****U****U**...**A****A****G****A****A****C**....................AUUG..........C**G****U****U****C****U****U**.GC.**A**..**G****A**..**U****G****A****G****G****C****G****G****A**GAUU | |
|  |  | NZ\_AADT03000017.1/30334-30203  | AAUG**A****A****A****C****U****C****U****U**.**A****U****C**G.AGA**G**.**U****G****G****U**.**G****G****A****G****G****G**A...**C****U****G**.**G**.**C****C****C**GAU**G****A****A**.**G****C****C**-.**C****G****G****C**AA**C****C**.-.-**C****G****C****U**CUCCUGG.................UUUCU...............................................CCGGG**G****G****C****G****A**.C...**A**..**G****G**U**G**..**C****C**AAU**U****C****C****U****G**...**C****A****G****G****G****C**....................AGAA..........G**G****C****C****C****U****G**.AG.**A**..**G****A**..**U****A****A****G****G****G****G****G****G**UAAG | |
|  |  | NC\_000964.2/1629408-1629527  | AUCU**A****A****A****A****A****C****U****U**.**A****U****C**A.AGA**G**.**C****G****G****C**.**U****G****A****G****G****G**A...**C****U****G**.**G**.**A****C****C**UAU**G****A****A**.**G****C****C**-.**C****G****G****C**AA**C****C**.**U**.**G****C****A**--........................UAGUU....................................................--**U****G****U**.A...**A**..**G****G**U**G**..**C****U**ACU**U****C****C****A****G**...**C****A****A****A****A****U**G...................AAUU.........CC**A****U****U****U****U****G**.AA.**A**..**G****A**..**U****A****A****G****G****G****C****U****G**CAUG | |
|  |  | NC\_002939.4/1014089-1014209  | ACGG**C****U****U****A****A****C****U****U**.**A****U****C**A.AGA**G**.**C****G****A****C**.**C****G****A****G****G****G**A...**C****A****G**.**G**.**C****C****C**GGU**G****A****C**.**G****U****C****G**.**C****G****G****C**AA**C****C**.**U**.**C****C****C****C**-........................AUGG-....................................................-**G****G****G****G**.A...**A**..**G****G**U**G**..**C****C**AAU**U****C****C****U****G**...**C****G****A****G****A****C**C...................GACA..........G**G****U****U****U****C****G**.GG.**A**..**G****A**..**U****A****A****G****G****A****A****G****A**GCGU | |
|  |  | NC\_003997.3/371136-371010  | GAAU**A****A****U****U****C****U****U****U**.**A****U****C**A.AGA**G**.**A****G****G****C**.**A****G****A****G****G****G**A...**C****C****G**.**G**.**C****C****C**UUU**G****A****A**.**G****C****C**-.**C****A****G****C**AA**C****C**.**U**.--**C****A****G**U.......................UUAUA................................................CAAA**C****U****G****A****A**.U...**A**..**G****G**U**G**..**C****U**AAU**U****C****C****U****G**...**C****A****A****A****A****U**G...................CAUU.........GC**A****U****U****U****U****G**.AA.**A**..**G****A**..**U****A****A****A****A****C****G****U****A**ACUA | |
|  |  | NC\_005945.1/371149-371023  | GAAU**A****A****U****U****C****U****U****U**.**A****U****C**A.AGA**G**.**A****G****G****C**.**A****G****A****G****G****G**A...**C****C****G**.**G**.**C****C****C**UUU**G****A****A**.**G****C****C**-.**C****A****G****C**AA**C****C**.**U**.--**C****A****G**U.......................UUAUA................................................CAAA**C****U****G****A****A**.U...**A**..**G****G**U**G**..**C****U**AAU**U****C****C****U****G**...**C****A****A****A****A****U**G...................CAUU.........GC**A****U****U****U****U****G**.AA.**A**..**G****A**..**U****A****A****A****A****C****G****U****A**ACUA | |
|  |  | NC\_005957.1/388370-388244  | GAAU**A****A****U****U****C****U****U****U**.**A****U****C**A.AGA**G**.**A****G****G****C**.**A****G****A****G****G****G**A...**C****C****G**.**G**.**C****C****C**UUU**G****A****A**.**G****C****C**-.**C****A****G****C**AA**C****C**.**U**.--**C****A****G**U.......................UUAUA................................................CAAA**C****U****G****A****A**.U...**A**..**G****G**U**G**..**C****U**AAU**U****C****C****U****G**...**C****A****A****A****A****U**G...................CAUU.........GC**A****U****U****U****U****G**.AA.**A**..**G****A**..**U****A****A****A****A****C****G****U****A**ACUA | |
|  |  | NC\_006274.1/387529-387403  | GAAU**A****A****U****U****C****U****U****U**.**A****U****C**A.AGA**G**.**A****G****G****C**.**A****G****A****G****G****G**A...**C****C****G**.**G**.**C****C****C**UUU**G****A****A**.**G****C****C**-.**C****A****G****C**AA**C****C**.**U**.--**C****A****G**U.......................UUAUA................................................CAAA**C****U****G****A****A**.U...**A**..**G****G**U**G**..**C****U**AAU**U****C****C****U****G**...**C****A****A****A****A****U**G...................CAUU.........GC**A****U****U****U****U****G**.AA.**A**..**G****A**..**U****A****A****A****A****C****G****U****A**ACUA | |
|  |  | NC\_007530.2/371136-371010  | GAAU**A****A****U****U****C****U****U****U**.**A****U****C**A.AGA**G**.**A****G****G****C**.**A****G****A****G****G****G**A...**C****C****G**.**G**.**C****C****C**UUU**G****A****A**.**G****C****C**-.**C****A****G****C**AA**C****C**.**U**.--**C****A****G**U.......................UUAUA................................................CAAA**C****U****G****A****A**.U...**A**..**G****G**U**G**..**C****U**AAU**U****C****C****U****G**...**C****A****A****A****A****U**G...................CAUU.........GC**A****U****U****U****U****G**.AA.**A**..**G****A**..**U****A****A****A****A****C****G****U****A**ACUA | |
|  |  | NZ\_AAAC02000001.1/887948-887822  | GAAU**A****A****U****U****C****U****U****U**.**A****U****C**A.AGA**G**.**A****G****G****C**.**A****G****A****G****G****G**A...**C****C****G**.**G**.**C****C****C**UUU**G****A****A**.**G****C****C**-.**C****A****G****C**AA**C****C**.**U**.--**C****A****G**U.......................UUAUA................................................CAAA**C****U****G****A****A**.U...**A**..**G****G**U**G**..**C****U**AAU**U****C****C****U****G**...**C****A****A****A****A****U**G...................CAUU.........GC**A****U****U****U****U****G**.AA.**A**..**G****A**..**U****A****A****A****A****C****G****U****A**ACUA | |
|  |  | NZ\_AAEN01000023.1/112222-112096  | GAAU**A****A****U****U****C****U****U****U**.**A****U****C**A.AGA**G**.**A****G****G****C**.**A****G****A****G****G****G**A...**C****C****G**.**G**.**C****C****C**UUU**G****A****A**.**G****C****C**-.**C****A****G****C**AA**C****C**.**U**.--**C****A****G**U.......................UUAUA................................................CAAA**C****U****G****A****A**.U...**A**..**G****G**U**G**..**C****U**AAU**U****C****C****U****G**...**C****A****A****A****A****U**G...................CAUU.........GC**A****U****U****U****U****G**.AA.**A**..**G****A**..**U****A****A****A****A****C****G****U****A**ACUA | |
|  |  | NZ\_AAEO01000030.1/125666-125540  | GAAU**A****A****U****U****C****U****U****U**.**A****U****C**A.AGA**G**.**A****G****G****C**.**A****G****A****G****G****G**A...**C****C****G**.**G**.**C****C****C**UUU**G****A****A**.**G****C****C**-.**C****A****G****C**AA**C****C**.**U**.--**C****A****G**U.......................UUAUA................................................CAAA**C****U****G****A****A**.U...**A**..**G****G**U**G**..**C****U**AAU**U****C****C****U****G**...**C****A****A****A****A****U**G...................CAUU.........GC**A****U****U****U****U****G**.AA.**A**..**G****A**..**U****A****A****A****A****C****G****U****A**ACUA | |
|  |  | NZ\_AAEP01000046.1/77590-77464  | GAAU**A****A****U****U****C****U****U****U**.**A****U****C**A.AGA**G**.**A****G****G****C**.**A****G****A****G****G****G**A...**C****C****G**.**G**.**C****C****C**UUU**G****A****A**.**G****C****C**-.**C****A****G****C**AA**C****C**.**U**.--**C****A****G**U.......................UUAUA................................................CAAA**C****U****G****A****A**.U...**A**..**G****G**U**G**..**C****U**AAU**U****C****C****U****G**...**C****A****A****A****A****U**G...................CAUU.........GC**A****U****U****U****U****G**.AA.**A**..**G****A**..**U****A****A****A****A****C****G****U****A**ACUA | |
|  |  | NZ\_AAEQ01000027.1/15302-15428  | GAAU**A****A****U****U****C****U****U****U**.**A****U****C**A.AGA**G**.**A****G****G****C**.**A****G****A****G****G****G**A...**C****C****G**.**G**.**C****C****C**UUU**G****A****A**.**G****C****C**-.**C****A****G****C**AA**C****C**.**U**.--**C****A****G**U.......................UUAUA................................................CAAA**C****U****G****A****A**.U...**A**..**G****G**U**G**..**C****U**AAU**U****C****C****U****G**...**C****A****A****A****A****U**G...................CAUU.........GC**A****U****U****U****U****G**.AA.**A**..**G****A**..**U****A****A****A****A****C****G****U****A**ACUA | |
|  |  | NZ\_AAER01000042.1/162558-162684  | GAAU**A****A****U****U****C****U****U****U**.**A****U****C**A.AGA**G**.**A****G****G****C**.**A****G****A****G****G****G**A...**C****C****G**.**G**.**C****C****C**UUU**G****A****A**.**G****C****C**-.**C****A****G****C**AA**C****C**.**U**.--**C****A****G**U.......................UUAUA................................................CAAA**C****U****G****A****A**.U...**A**..**G****G**U**G**..**C****U**AAU**U****C****C****U****G**...**C****A****A****A****A****U**G...................CAUU.........GC**A****U****U****U****U****G**.AA.**A**..**G****A**..**U****A****A****A****A****C****G****U****A**ACUA | |
|  |  | NZ\_AAES01000043.1/77259-77133  | GAAU**A****A****U****U****C****U****U****U**.**A****U****C**A.AGA**G**.**A****G****G****C**.**A****G****A****G****G****G**A...**C****C****G**.**G**.**C****C****C**UUU**G****A****A**.**G****C****C**-.**C****A****G****C**AA**C****C**.**U**.--**C****A****G**U.......................UUAUA................................................CAAA**C****U****G****A****A**.U...**A**..**G****G**U**G**..**C****U**AAU**U****C****C****U****G**...**C****A****A****A****A****U**G...................CAUU.........GC**A****U****U****U****U****G**.AA.**A**..**G****A**..**U****A****A****A****A****C****G****U****A**ACUA | |
|  |  | NC\_002570.2/3427474-3427348  | ACGG**A****U****A****C****U****C****U****U**.**A****U****C**C.AGA**G**U**U****G****G****U**.**G****G****A****G****G****G**A...**C****A****G**.**G**.**C****C****C**GAA**G****A****A**.**A****C****C****C**.**C****A****G****C**AA**C****C**.-.**A****A****C****A****C**CUG.....................UUAAA...............................................CAAAG**G****U****G****A****A**.A...**A**..**G****G**U**G**..**C****U**AA--**C****C****U****G**...**C****A****A****G****G****C**....................-GUU...........**G****C****C****U****U****G**.AA.**A**..**G****A**..**U****A****A****G****A****G****G****C****G**AAAG | |
|  |  | NC\_000964.2/1180114-1179983  | AUAU**C****C****G****U****U****C****U****U**.**A****U****C**A.AGA**G**.**A****A****G****C**.**A****G****A****G****G****G**A...**C****U****G**.**G**.**C****C****C**GAC**G****A****A**.**G****C****U****U**.**C****A****G****C**AA**C****C**.-.**G****G****U**--G.......................UAAU-......................................GGCGAUCAGCCAUG--**A****C****C**.A...**A**..**G****G**U**G**..**C****U**AAA**U****C****C****A****G**...**C****A****A****G****C****U**C...................-GAA..........C**A****G****C****U****U****G**.GA.**A**..**G****A**..**U****A****A****G****A****A****G****A****G**ACAA | |
|  |  | NC\_002570.2/1348809-1348936  | AUAA**A****A****A****G****A****C****U****U**.**A****U****C**G.AGA**G**.**A****G****G****C**.**A****G****A****G****G****G**A...**C****U****G**.**A**.**C****C****C**GAU**G****A****U**.**G****C****C**-.**C****G****G****C**AA**C****C**.-.**C****G****U****U****U**G.......................UUAGC...............................................CAAGC**A****A****A****C****G**.A...**A**..**G****G**U**G**..**C****U**AAU**U****U****C****A****G**...**C****A****G****A****A****U**G...................AUUU..........C**A****U****U****C****U****G**.GA.**A**..**G****A**..**U****A****A****G****C****G****A****A****G**GCGA | |
|  |  | NZ\_AAEK01000047.1/24547-24421  | UAAU**A****U****A****U****C****U****U****U**.**A****U****C**A.AGA**G**.**A****G****G****C**.**A****G****A****G****G****G**A...**C****C****G**.**G**.**C****C****C**UUU**G****A****A**.**G****C****C**-.**C****A****G****C**AA**C****C**.**U**.--**C****A****G**U.......................UUACA................................................CAAA**C****U****G****A****A**.U...**A**..**G****G**U**G**..**C****U**AAU**U****C****C****U****G**...**C****A****A****A****A****U**G...................CAUU.........GC**A****U****U****U****U****G**.AA.**A**..**G****A**..**U****A****A****A****A****C****A****U****A**ACUA | |
|  |  | NC\_003909.8/1084273-1084393  | CAAA**C****A****A****U****U****C****U****U**.**A****U****G**U.UGA**G**.**A****A****G****U**.**G****G****A****G****G****G**A...**C****G****G**.**G**.**C****C****C**UAU**G****A****A**.**A****C****U****U**.**C****G****G****C**AA**C****C**.**U**.-**C****G****U**-........................AUGAG....................................................-**A****C****G****A**.A...**A**..**G****G**U**G**..**C****C**AAA**U****C****C****U****G**...**C****A****G****G****U****G**A...................AGAA..........A**C****A****C****C****U****G**.AA.**A**..**G****A**..**U****A****A****G****A****G****C****G****G**UUCA | |
|  |  | NC\_003997.3/4074087-4073964  | AAGA**C****A****A****C****U****C****U****U**.**A****U****U**G.AGA**G**.**C****G****G****U**.**G****G****A****G****G****G**A...**A****A****G**.**G**.**C****C****C**UGU**G****A****A**.**A****C****C**-.**C****G****G****C**AA**C****C**.**U**.-**U****C****A****A**AC......................GAAAU..................................................GU**U****U****G****A****A**.A...**C**..**G****G**U**G**..**C****U**AAU**A****C****C****U****G**...**C****A****A****A****A****C**....................GAAU...........**G****U****U****U****U****G**.CA.**U**..**A****A**..**U****A****A****G****A****G****G****A****G**GAAC | |
|  |  | NC\_004722.1/4199517-4199394  | AAGA**C****A****A****C****U****C****U****U**.**A****U****U**G.AGA**G**.**C****G****G****U**.**G****G****A****G****G****G**A...**A****A****G**.**G**.**C****C****C**UGU**G****A****A**.**A****C****C**-.**C****G****G****C**AA**C****C**.**U**.-**U****C****A****A**AC......................GAAAU..................................................GU**U****U****G****A****A**.A...**C**..**G****G**U**G**..**C****U**AAU**A****C****C****U****G**...**C****A****A****A****A****C**....................GAAU...........**G****U****U****U****U****G**.CA.**U**..**A****A**..**U****A****A****G****A****G****G****A****G**GAAC | |
|  |  | NC\_005945.1/4074587-4074464  | AAGA**C****A****A****C****U****C****U****U**.**A****U****U**G.AGA**G**.**C****G****G****U**.**G****G****A****G****G****G**A...**A****A****G**.**G**.**C****C****C**UGU**G****A****A**.**A****C****C**-.**C****G****G****C**AA**C****C**.**U**.-**U****C****A****A**AC......................GAAAU..................................................GU**U****U****G****A****A**.A...**C**..**G****G**U**G**..**C****U**AAU**A****C****C****U****G**...**C****A****A****A****A****C**....................GAAU...........**G****U****U****U****U****G**.CA.**U**..**A****A**..**U****A****A****G****A****G****G****A****G**GAAC | |
|  |  | NC\_005957.1/4062044-4061921  | AAGA**C****A****A****C****U****C****U****U**.**A****U****U**G.AGA**G**.**C****G****G****U**.**G****G****A****G****G****G**A...**A****A****G**.**G**.**C****C****C**UGU**G****A****A**.**A****C****C**-.**C****G****G****C**AA**C****C**.**U**.-**U****C****A****A**AC......................GAAAU..................................................GU**U****U****G****A****A**.A...**C**..**G****G**U**G**..**C****U**AAU**A****C****C****U****G**...**C****A****A****A****A****C**....................GAAU...........**G****U****U****U****U****G**.CA.**U**..**A****A**..**U****A****A****G****A****G****G****A****G**GAAC | |
|  |  | NC\_006274.1/4116271-4116148  | AAGA**C****A****A****C****U****C****U****U**.**A****U****U**G.AGA**G**.**C****G****G****U**.**G****G****A****G****G****G**A...**A****A****G**.**G**.**C****C****C**UGU**G****A****A**.**A****C****C**-.**C****G****G****C**AA**C****C**.**U**.-**U****C****A****A**AC......................GAAAU..................................................GU**U****U****G****A****A**.A...**C**..**G****G**U**G**..**C****U**AAU**A****C****C****U****G**...**C****A****A****A****A****C**....................GAAU...........**G****U****U****U****U****G**.CA.**U**..**A****A**..**U****A****A****G****A****G****G****A****G**GAAC | |
|  |  | NC\_007530.2/4074214-4074091  | AAGA**C****A****A****C****U****C****U****U**.**A****U****U**G.AGA**G**.**C****G****G****U**.**G****G****A****G****G****G**A...**A****A****G**.**G**.**C****C****C**UGU**G****A****A**.**A****C****C**-.**C****G****G****C**AA**C****C**.**U**.-**U****C****A****A**AC......................GAAAU..................................................GU**U****U****G****A****A**.A...**C**..**G****G**U**G**..**C****U**AAU**A****C****C****U****G**...**C****A****A****A****A****C**....................GAAU...........**G****U****U****U****U****G**.CA.**U**..**A****A**..**U****A****A****G****A****G****G****A****G**GAAC | |
|  |  | NZ\_AAAC02000001.1/4519848-4519725  | AAGA**C****A****A****C****U****C****U****U**.**A****U****U**G.AGA**G**.**C****G****G****U**.**G****G****A****G****G****G**A...**A****A****G**.**G**.**C****C****C**UGU**G****A****A**.**A****C****C**-.**C****G****G****C**AA**C****C**.**U**.-**U****C****A****A**AC......................GAAAU..................................................GU**U****U****G****A****A**.A...**C**..**G****G**U**G**..**C****U**AAU**A****C****C****U****G**...**C****A****A****A****A****C**....................GAAU...........**G****U****U****U****U****G**.CA.**U**..**A****A**..**U****A****A****G****A****G****G****A****G**GAAC | |
|  |  | NZ\_AAEN01000013.1/328596-328473  | AAGA**C****A****A****C****U****C****U****U**.**A****U****U**G.AGA**G**.**C****G****G****U**.**G****G****A****G****G****G**A...**A****A****G**.**G**.**C****C****C**UGU**G****A****A**.**A****C****C**-.**C****G****G****C**AA**C****C**.**U**.-**U****C****A****A**AC......................GAAAU..................................................GU**U****U****G****A****A**.A...**C**..**G****G**U**G**..**C****U**AAU**A****C****C****U****G**...**C****A****A****A****A****C**....................GAAU...........**G****U****U****U****U****G**.CA.**U**..**A****A**..**U****A****A****G****A****G****G****A****G**GAAC | |
|  |  | NZ\_AAEO01000019.1/415689-415566  | AAGA**C****A****A****C****U****C****U****U**.**A****U****U**G.AGA**G**.**C****G****G****U**.**G****G****A****G****G****G**A...**A****A****G**.**G**.**C****C****C**UGU**G****A****A**.**A****C****C**-.**C****G****G****C**AA**C****C**.**U**.-**U****C****A****A**AC......................GAAAU..................................................GU**U****U****G****A****A**.A...**C**..**G****G**U**G**..**C****U**AAU**A****C****C****U****G**...**C****A****A****A****A****C**....................GAAU...........**G****U****U****U****U****G**.CA.**U**..**A****A**..**U****A****A****G****A****G****G****A****G**GAAC | |
|  |  | NZ\_AAEP01000031.1/31381-31504  | AAGA**C****A****A****C****U****C****U****U**.**A****U****U**G.AGA**G**.**C****G****G****U**.**G****G****A****G****G****G**A...**A****A****G**.**G**.**C****C****C**UGU**G****A****A**.**A****C****C**-.**C****G****G****C**AA**C****C**.**U**.-**U****C****A****A**AC......................GAAAU..................................................GU**U****U****G****A****A**.A...**C**..**G****G**U**G**..**C****U**AAU**A****C****C****U****G**...**C****A****A****A****A****C**....................GAAU...........**G****U****U****U****U****G**.CA.**U**..**A****A**..**U****A****A****G****A****G****G****A****G**GAAC | |
|  |  | NZ\_AAEQ01000030.1/63123-63000  | AAGA**C****A****A****C****U****C****U****U**.**A****U****U**G.AGA**G**.**C****G****G****U**.**G****G****A****G****G****G**A...**A****A****G**.**G**.**C****C****C**UGU**G****A****A**.**A****C****C**-.**C****G****G****C**AA**C****C**.**U**.-**U****C****A****A**AC......................GAAAU..................................................GU**U****U****G****A****A**.A...**C**..**G****G**U**G**..**C****U**AAU**A****C****C****U****G**...**C****A****A****A****A****C**....................GAAU...........**G****U****U****U****U****G**.CA.**U**..**A****A**..**U****A****A****G****A****G****G****A****G**GAAC | |
|  |  | NZ\_AAER01000035.1/586933-586810  | AAGA**C****A****A****C****U****C****U****U**.**A****U****U**G.AGA**G**.**C****G****G****U**.**G****G****A****G****G****G**A...**A****A****G**.**G**.**C****C****C**UGU**G****A****A**.**A****C****C**-.**C****G****G****C**AA**C****C**.**U**.-**U****C****A****A**AC......................GAAAU..................................................GU**U****U****G****A****A**.A...**C**..**G****G**U**G**..**C****U**AAU**A****C****C****U****G**...**C****A****A****A****A****C**....................GAAU...........**G****U****U****U****U****G**.CA.**U**..**A****A**..**U****A****A****G****A****G****G****A****G**GAAC | |
|  |  | NZ\_AAES01000022.1/579171-579294  | AAGA**C****A****A****C****U****C****U****U**.**A****U****U**G.AGA**G**.**C****G****G****U**.**G****G****A****G****G****G**A...**A****A****G**.**G**.**C****C****C**UGU**G****A****A**.**A****C****C**-.**C****G****G****C**AA**C****C**.**U**.-**U****C****A****A**AC......................GAAAU..................................................GU**U****U****G****A****A**.A...**C**..**G****G**U**G**..**C****U**AAU**A****C****C****U****G**...**C****A****A****A****A****C**....................GAAU...........**G****U****U****U****U****G**.CA.**U**..**A****A**..**U****A****A****G****A****G****G****A****G**GAAC | |
|  |  | NC\_006270.2/1291279-1291409  | GAAG**A****G****A****U****U****C****U****U**.**A****U****C**A.CGA**G**.**A****G****G****U**.**G****G****A****G****G****G**A...**C****U****G**.**G**.**C****C****C**UUU**G****A****A**.**A****C****C****U**.**C****A****G****C**AA**C****C**.-.**G****G****U****C****U**GCACUGACGAC.............GUCA-..................................................GU**G****C****A****C****C**.A...**A**..**G****G**U**G**..**C****U**AAA**U****C****C****A****G**...**C****A****A****G****C**-....................GGAU...........-**G****C****U****U****G**.GA.**A**..**G****A**..**U****A****A****G****A****A****G****A****A**GCGA | |
|  |  | NC\_006322.1/1292141-1292271  | GAAG**A****G****A****U****U****C****U****U**.**A****U****C**A.CGA**G**.**A****G****G****U**.**G****G****A****G****G****G**A...**C****U****G**.**G**.**C****C****C**UUU**G****A****A**.**A****C****C****U**.**C****A****G****C**AA**C****C**.-.**G****G****U****C****U**GCACUGACGAC.............GUCA-..................................................GU**G****C****A****C****C**.A...**A**..**G****G**U**G**..**C****U**AAA**U****C****C****A****G**...**C****A****A****G****C**-....................GGAU...........-**G****C****U****U****G**.GA.**A**..**G****A**..**U****A****A****G****A****A****G****A****A**GCGA | |
|  |  | NC\_003909.8/212933-213052  | AGCA**A****U****U****U****A****C****U****U**.**A****U****C**C.AGA**G**.**A****G****G****U**.**A****G****A****G****G****G**A...**C****U****G**.**G**.**C****C****C**UAU**G****A****C**.**A****C****C****U**.**C****A****G****C**AG**C****G**.**G**.**G****U****U****C****U**G.......................UAAUA....................................................**G****G****A****A****C**.A...**C**..**C****G**U**G**..**C****U**AAU**U****C****C****A****G**...**C****A****A****G****C**-....................-AAG...........-**U****C****U****U****G**.AA.**A**..**G****A**..**U****A****A****G****U****G****A****U****G**GGCC | |
|  |  | NC\_003997.3/185577-185696  | AGCA**A****U****U****U****A****C****U****U**.**A****U****C**C.AGA**G**.**A****G****G****U**.**A****G****A****G****G****G**A...**C****U****G**.**G**.**C****C****C**UAU**G****A****C**.**A****C****C****U**.**C****A****G****C**AG**C****G**.**G**.**G****U****U****C****U**G.......................UAAUA....................................................**G****G****A****A****C**.A...**C**..**C****G**U**G**..**C****U**AAU**U****C****C****A****G**...**C****A****A****G****C**-....................-AAG...........-**U****C****U****U****G**.AA.**A**..**G****A**..**U****A****A****G****U****G****A****U****G**GGCC | |
|  |  | NC\_004722.1/183057-183176  | AGCA**A****U****U****U****A****C****U****U**.**A****U****C**C.AGA**G**.**A****G****G****U**.**A****G****A****G****G****G**A...**C****U****G**.**G**.**C****C****C**UAU**G****A****C**.**A****C****C****U**.**C****A****G****C**AG**C****G**.**G**.**G****U****U****C****U**G.......................UAAUA....................................................**G****G****A****A****C**.A...**C**..**C****G**U**G**..**C****U**AAU**U****C****C****A****G**...**C****A****A****G****C**-....................-AAG...........-**U****C****U****U****G**.AA.**A**..**G****A**..**U****A****A****G****U****G****A****U****G**GGCC | |
|  |  | NC\_005945.1/185578-185697  | AGCA**A****U****U****U****A****C****U****U**.**A****U****C**C.AGA**G**.**A****G****G****U**.**A****G****A****G****G****G**A...**C****U****G**.**G**.**C****C****C**UAU**G****A****C**.**A****C****C****U**.**C****A****G****C**AG**C****G**.**G**.**G****U****U****C****U**G.......................UAAUA....................................................**G****G****A****A****C**.A...**C**..**C****G**U**G**..**C****U**AAU**U****C****C****A****G**...**C****A****A****G****C**-....................-AAG...........-**U****C****U****U****G**.AA.**A**..**G****A**..**U****A****A****G****U****G****A****U****G**GGCC | |
|  |  | NC\_006274.1/185411-185530  | AGCA**A****U****U****U****A****C****U****U**.**A****U****C**C.AGA**G**.**A****G****G****U**.**A****G****A****G****G****G**A...**C****U****G**.**G**.**C****C****C**UAU**G****A****C**.**A****C****C****U**.**C****A****G****C**AG**C****G**.**G**.**G****U****U****C****U**G.......................UAAUA....................................................**G****G****A****A****C**.A...**C**..**C****G**U**G**..**C****U**AAU**U****C****C****A****G**...**C****A****A****G****C**-....................-AAG...........-**U****C****U****U****G**.AA.**A**..**G****A**..**U****A****A****G****U****G****A****U****G**GGCC | |
|  |  | NC\_007530.2/185577-185696  | AGCA**A****U****U****U****A****C****U****U**.**A****U****C**C.AGA**G**.**A****G****G****U**.**A****G****A****G****G****G**A...**C****U****G**.**G**.**C****C****C**UAU**G****A****C**.**A****C****C****U**.**C****A****G****C**AG**C****G**.**G**.**G****U****U****C****U**G.......................UAAUA....................................................**G****G****A****A****C**.A...**C**..**C****G**U**G**..**C****U**AAU**U****C****C****A****G**...**C****A****A****G****C**-....................-AAG...........-**U****C****U****U****G**.AA.**A**..**G****A**..**U****A****A****G****U****G****A****U****G**GGCC | |
|  |  | NZ\_AAAC02000001.1/725026-725145  | AGCA**A****U****U****U****A****C****U****U**.**A****U****C**C.AGA**G**.**A****G****G****U**.**A****G****A****G****G****G**A...**C****U****G**.**G**.**C****C****C**UAU**G****A****C**.**A****C****C****U**.**C****A****G****C**AG**C****G**.**G**.**G****U****U****C****U**G.......................UAAUA....................................................**G****G****A****A****C**.A...**C**..**C****G**U**G**..**C****U**AAU**U****C****C****A****G**...**C****A****A****G****C**-....................-AAG...........-**U****C****U****U****G**.AA.**A**..**G****A**..**U****A****A****G****U****G****A****U****G**GGCC | |
|  |  | NZ\_AAEK01000017.1/1885-2004  | AGCA**A****U****U****U****A****C****U****U**.**A****U****C**C.AGA**G**.**A****G****G****U**.**A****G****A****G****G****G**A...**C****U****G**.**G**.**C****C****C**UAU**G****A****C**.**A****C****C****U**.**C****A****G****C**AG**C****G**.**G**.**G****U****U****C****U**G.......................UAAUA....................................................**G****G****A****A****C**.A...**C**..**C****G**U**G**..**C****U**AAU**U****C****C****A****G**...**C****A****A****G****C**-....................-AAG...........-**U****C****U****U****G**.AA.**A**..**G****A**..**U****A****A****G****U****G****A****U****G**GGCC | |
|  |  | NZ\_AAEN01000019.1/37393-37512  | AGCA**A****U****U****U****A****C****U****U**.**A****U****C**C.AGA**G**.**A****G****G****U**.**A****G****A****G****G****G**A...**C****U****G**.**G**.**C****C****C**UAU**G****A****C**.**A****C****C****U**.**C****A****G****C**AG**C****G**.**G**.**G****U****U****C****U**G.......................UAAUA....................................................**G****G****A****A****C**.A...**C**..**C****G**U**G**..**C****U**AAU**U****C****C****A****G**...**C****A****A****G****C**-....................-AAG...........-**U****C****U****U****G**.AA.**A**..**G****A**..**U****A****A****G****U****G****A****U****G**GGCC | |
|  |  | NZ\_AAEO01000023.1/60376-60257  | AGCA**A****U****U****U****A****C****U****U**.**A****U****C**C.AGA**G**.**A****G****G****U**.**A****G****A****G****G****G**A...**C****U****G**.**G**.**C****C****C**UAU**G****A****C**.**A****C****C****U**.**C****A****G****C**AG**C****G**.**G**.**G****U****U****C****U**G.......................UAAUA....................................................**G****G****A****A****C**.A...**C**..**C****G**U**G**..**C****U**AAU**U****C****C****A****G**...**C****A****A****G****C**-....................-AAG...........-**U****C****U****U****G**.AA.**A**..**G****A**..**U****A****A****G****U****G****A****U****G**GGCC | |
|  |  | NZ\_AAEP01000033.1/34974-35093  | AGCA**A****U****U****U****A****C****U****U**.**A****U****C**C.AGA**G**.**A****G****G****U**.**A****G****A****G****G****G**A...**C****U****G**.**G**.**C****C****C**UAU**G****A****C**.**A****C****C****U**.**C****A****G****C**AG**C****G**.**G**.**G****U****U****C****U**G.......................UAAUA....................................................**G****G****A****A****C**.A...**C**..**C****G**U**G**..**C****U**AAU**U****C****C****A****G**...**C****A****A****G****C**-....................-AAG...........-**U****C****U****U****G**.AA.**A**..**G****A**..**U****A****A****G****U****G****A****U****G**GGCC | |
|  |  | NZ\_AAEQ01000020.1/59284-59165  | AGCA**A****U****U****U****A****C****U****U**.**A****U****C**C.AGA**G**.**A****G****G****U**.**A****G****A****G****G****G**A...**C****U****G**.**G**.**C****C****C**UAU**G****A****C**.**A****C****C****U**.**C****A****G****C**AG**C****G**.**G**.**G****U****U****C****U**G.......................UAAUA....................................................**G****G****A****A****C**.A...**C**..**C****G**U**G**..**C****U**AAU**U****C****C****A****G**...**C****A****A****G****C**-....................-AAG...........-**U****C****U****U****G**.AA.**A**..**G****A**..**U****A****A****G****U****G****A****U****G**GGCC | |
|  |  | NZ\_AAER01000024.1/60381-60262  | AGCA**A****U****U****U****A****C****U****U**.**A****U****C**C.AGA**G**.**A****G****G****U**.**A****G****A****G****G****G**A...**C****U****G**.**G**.**C****C****C**UAU**G****A****C**.**A****C****C****U**.**C****A****G****C**AG**C****G**.**G**.**G****U****U****C****U**G.......................UAAUA....................................................**G****G****A****A****C**.A...**C**..**C****G**U**G**..**C****U**AAU**U****C****C****A****G**...**C****A****A****G****C**-....................-AAG...........-**U****C****U****U****G**.AA.**A**..**G****A**..**U****A****A****G****U****G****A****U****G**GGCC | |
|  |  | NZ\_AAES01000019.1/35978-36097  | AGCA**A****U****U****U****A****C****U****U**.**A****U****C**C.AGA**G**.**A****G****G****U**.**A****G****A****G****G****G**A...**C****U****G**.**G**.**C****C****C**UAU**G****A****C**.**A****C****C****U**.**C****A****G****C**AG**C****G**.**G**.**G****U****U****C****U**G.......................UAAUA....................................................**G****G****A****A****C**.A...**C**..**C****G**U**G**..**C****U**AAU**U****C****C****A****G**...**C****A****A****G****C**-....................-AAG...........-**U****C****U****U****G**.AA.**A**..**G****A**..**U****A****A****G****U****G****A****U****G**GGCC | |
|  |  | NC\_003909.8/4022659-4022536  | AGAC**A****A****A****C****U****C****U****U**.**A****U****U**G.AGA**G**.**C****G****G****U**.**G****G****A****G****G****G**A...**A****A****G**.**G**.**C****C****C**UGU**G****A****A**.**A****C****C**-.**C****G****G****C**AA**C****C**.**U**.-**U****C****A****A**AC......................GAAAU..................................................GU**U****U****G****A****A**.A...**C**..**G****G**U**G**..**C****U**AAU**A****C****C****U****G**...**C****A****A****A****A****C**....................GAAU...........**G****U****U****U****U****G**.CA.**U**..**A****A**..**U****A****A****G****A****G****G****A****G**GAUC | |
|  |  | NZ\_AAEK01000021.1/52636-52759  | AGAC**A****A****A****C****U****C****U****U**.**A****U****U**G.AGA**G**.**C****G****G****U**.**G****G****A****G****G****G**A...**A****A****G**.**G**.**C****C****C**UGU**G****A****A**.**A****C****C**-.**C****G****G****C**AA**C****C**.**U**.-**U****C****A****A**AC......................GAAAU..................................................GU**U****U****G****A****A**.A...**C**..**G****G**U**G**..**C****U**AAU**A****C****C****U****G**...**C****A****A****A****A****C**....................GAAU...........**G****U****U****U****U****G**.CA.**U**..**A****A**..**U****A****A****G****A****G****G****A****G**GAUC | |
|  |  | NC\_002745.2/875399-875516  | GCGU**A****A****A****C****U****C****U****U**.**A****U****C**G.AGA**G**.**U****G****G****U**.**G****G****A****G****G****G**A...**U****G****U**.**G**.**C****C****C**UAC**G****A****A**.**G****C****C**-.**C****G****G****C**AA**C****C**.**G**.-**U****C****U**-........................UAUAU....................................................-**A****G****A****A**.A...**U**..**G****G**U**G**..**C****C**AAU**U****C****A****C****A**...**U****A****A****A****G****U**....................UUUA...........**A****C****U****U****U****U**.GA.**A**..**G****A**..**U****G****A****G****A****G****A****A****A**CAAU | |
|  |  | NC\_002758.2/907203-907320  | GCGU**A****A****A****C****U****C****U****U**.**A****U****C**G.AGA**G**.**U****G****G****U**.**G****G****A****G****G****G**A...**U****G****U**.**G**.**C****C****C**UAC**G****A****A**.**G****C****C**-.**C****G****G****C**AA**C****C**.**G**.-**U****C****U**-........................UAUAU....................................................-**A****G****A****A**.A...**U**..**G****G**U**G**..**C****C**AAU**U****C****A****C****A**...**U****A****A****A****G****U**....................UUUA...........**A****C****U****U****U****U**.GA.**A**..**G****A**..**U****G****A****G****A****G****A****A****A**CAAU | |
|  |  | NC\_002951.2/900597-900714  | GCGU**A****A****A****C****U****C****U****U**.**A****U****C**G.AGA**G**.**U****G****G****U**.**G****G****A****G****G****G**A...**U****G****U**.**G**.**C****C****C**UAC**G****A****A**.**G****C****C**-.**C****G****G****C**AA**C****C**.**G**.-**U****C****U**-........................UAUAU....................................................-**A****G****A****A**.A...**U**..**G****G**U**G**..**C****C**AAU**U****C****A****C****A**...**U****A****A****A****G****U**....................UUUA...........**A****C****U****U****U****U**.GA.**A**..**G****A**..**U****G****A****G****A****G****A****A****A**CAAU | |
|  |  | NC\_002952.2/908398-908515  | GCGU**A****A****A****C****U****C****U****U**.**A****U****C**G.AGA**G**.**U****G****G****U**.**G****G****A****G****G****G**A...**U****G****U**.**G**.**C****C****C**UAC**G****A****A**.**G****C****C**-.**C****G****G****C**AA**C****C**.**G**.-**U****C****U**-........................UAUAU....................................................-**A****G****A****A**.A...**U**..**G****G**U**G**..**C****C**AAU**U****C****A****C****A**...**U****A****A****A****G****U**....................UUUA...........**A****C****U****U****U****U**.GA.**A**..**G****A**..**U****G****A****G****A****G****A****A****A**CAAU | |
|  |  | NC\_002953.3/861012-861129  | GCGU**A****A****A****C****U****C****U****U**.**A****U****C**G.AGA**G**.**U****G****G****U**.**G****G****A****G****G****G**A...**U****G****U**.**G**.**C****C****C**UAC**G****A****A**.**G****C****C**-.**C****G****G****C**AA**C****C**.**G**.-**U****C****U**-........................UAUAU....................................................-**A****G****A****A**.A...**U**..**G****G**U**G**..**C****C**AAU**U****C****A****C****A**...**U****A****A****A****G****U**....................UUUA...........**A****C****U****U****U****U**.GA.**A**..**G****A**..**U****G****A****G****A****G****A****A****A**CAAU | |
|  |  | NC\_003923.1/877043-877160  | GCGU**A****A****A****C****U****C****U****U**.**A****U****C**G.AGA**G**.**U****G****G****U**.**G****G****A****G****G****G**A...**U****G****U**.**G**.**C****C****C**UAC**G****A****A**.**G****C****C**-.**C****G****G****C**AA**C****C**.**G**.-**U****C****U**-........................UAUAU....................................................-**A****G****A****A**.A...**U**..**G****G**U**G**..**C****C**AAU**U****C****A****C****A**...**U****A****A****A****G****U**....................UUUA...........**A****C****U****U****U****U**.GA.**A**..**G****A**..**U****G****A****G****A****G****A****A****A**CAAU | |
|  |  | NC\_006582.1/604078-603961  | AUAC**G****A****A****U****U****C****U****U**.**A****U****U**A.AGA**G**.**G****A****G****C**.**A****G****A****G****G****G**A...**C****U****G**.**G**.**C****C****C**AAU**G****A****U**.**G****C****U****U**.**C****A****G****C**AA**C****C**.-.**C****C****G****C**-........................CAUGA....................................................-**G****C****G****G**.A...**A**..**G****G**U**G**..**C****U**AAU**U****C****C****A****G**...**C****A****G****G****A****C**....................-UAC...........**G****U****C****C****U****G**.GG.**A**..**G****A**..**U****A****A****G****A****G****A****U****U**GAAU | |
|  |  | NZ\_AAEK01000052.1/2958-3087  | AAAU**U****A****A****U****A****C****U****U**.**A****U****C**C.AGA**G**.**A****G****G****U**.**G****G****A****G****G****G**A...**C****C****G**.**G**.**C****C****C**UAU**G****A****A**.**A****C****C****U**.**C****A****G****C**AA**C****C**.-.**C****C****U****A****U**G.......................UAAAU..................................................GU**A****U****A****G****G**.A...**A**..**G****G**U**G**..**C****U**AAU**U****C****C**-**G**...**C****A****G****A****G****A**ACACG...............AUGU.........GU**U****U****U****U****U****G**.GA.**A**..**G****A**..**U****A****A****G****A****G****G****A****U**UCUU | |
|  |  | NC\_000964.2/2024468-2024363  | UCAA**U****A****U****U****U****U****C****U**.**A****U****C**C.AGA**G**.**A****G****G****U**.**G****G****A****G****G****G**A...**C****U****G**.**G**.**C****C****C**UAU**G****A****A**.**A****C****C****U**.**C****G****G****C**AA**C****A**.-.-----........................UUAU-....................................................-----.-...-..**U****G**U**G**..**C****C**AAU**U****C****C****A****G**...**C****A****A****G****C**-....................GCUA...........-**G****C****U****U****G**.AA.**A**..**G****A**..**U****A****G****G****A****A****A****G****C**AAGG | |
|  |  | NC\_004557.1/303331-303450  | UAAA**A****A****G****C****U****C****U****U**.**A****U****C**G.AGA**G**.**A****G****G****U**.**G****G****A****G****G****G**AA..**A****G****G**.**G**.**C****C****C**UAU**G****A****A**.**A****C****C**-.**C****G****G****C**AA**C****C**.-.**A****A****U****A****U**U.......................UUUA-.................................................GAA**G****U****A****U****U**.A...**A**..**G****G**U**G**..**C****C**AAU**U****C****C****U****G**...**C****A****G****A**--....................AAGU...........--**U****C****U****G**.CA.**A**..**G****A**..**U****A****A****G****A****G****G****G****C**UGGC | |
|  |  | NC\_004193.1/3200645-3200523  | AUGA**A****A****U****A****U****C****U****U**.**A****U****C**C.UGA**G**.**A****G****G****U**.**G****G****A****G****G****G**AA..**A****U****G**.**G**.**C****C****C**AAA**G****A****A**.**G****C****C****U**.**C****G****G****C**AA**C****A**.**G**.**G****U****U****C**-........................UAGCU....................................................**U****G****A****A****U**.A...**C**..**U****G**U**G**..**C****C**AAA**U****C****C****A****U**...**C****A****A****G****U****A**....................UUCU..........A**U****G****C****U****U****G**.GU.**A**..**G****A**..**U****A****A****G****A****G****A****A****G**UCGG | |
|  |  | NC\_006270.2/1734721-1734841  | AAAA**G****C****A****A****A****C****U****U**.**A****U****C**A.AGA**G**.**C****G****G****U**.**G****G****A****G****G****G**A...**C****U****G**.**G**.**U****C****C**GAU**G****A****A**.**A****C****C**-.**C****G****G****C**AA**C****C**.**U**.**G****C****G****U**-........................GUGAA....................................................-**G****C****G****U**.A...**A**..**G****G**U**G**..**C****U**ACU**U****C****C****A****G**...**C****A****A****A****A****U**G...................CCCU..........C**A****U****U****U****U****G**.AG.**A**..**G****A**..**U****A****A****G****G****A****A****U****A**GCGG | |
|  |  | NC\_006322.1/1735567-1735687  | AAAA**G****C****A****A****A****C****U****U**.**A****U****C**A.AGA**G**.**C****G****G****U**.**G****G****A****G****G****G**A...**C****U****G**.**G**.**U****C****C**GAU**G****A****A**.**A****C****C**-.**C****G****G****C**AA**C****C**.**U**.**G****C****G****U**-........................GUGAA....................................................-**G****C****G****U**.A...**A**..**G****G**U**G**..**C****U**ACU**U****C****C****A****G**...**C****A****A****A****A****U**G...................CCCU..........C**A****U****U****U****U****G**.AG.**A**..**G****A**..**U****A****A****G****G****A****A****U****A**GCGG | |
|  |  | NZ\_AABG04000106.1/1107-1226  | ACAG**G****U****A****A****C****C****U****U**.**A****U****C**A.AGA**G**.**A****G****G****C**.**G****G****A****G****G****G**A...**A****U****G**.**G**G**C****C****C**UAU**G****A****A**.**A****C****C**-.**C****G****G****C**AA**C****C**.**G**.-**G****C****A**-........................GAAU-....................................................-**U****G****U****A**.U...**C**..**G****G**U**G**..**C****C**AAU**U****C****C****U****A**...**C****A****G****G****A****U**G...................UAAA..........A**G****U****C****C****U****G**.AC.**A**..**G****A**..**U****G****A****G****G****A****U****A****A**AAAA | |
|  |  | NC\_006582.1/1705486-1705370  | AUGU**U****U****U****U****U****C****U****U**.**A****U****C**C.AGA**G**.**A****G****A****U**.**G****G****A****G****G****G**AU..**U****U****G**.**G**.**C****C****C**UUU**G****A****A**.**G****U****C****U**.**C****A****G****C**AA**C****C**.**G**.-**G****C****C**-........................-UUU-....................................................-**G****G****C****A**.A...**U**..**G****G**U**G**..**C****U**AAU**U****C****C****A****A**...**U****A****G****G****U****A**....................-AUG...........**U****A****C****C****U****A**.GG.**A**..**G****A**..**U****A****A****G****A****A****G****U****U**CGUU | |
|  |  | NC\_004722.1/374997-374871  | GAAU**A****C****U****U****C****U****U****U**.**A****U****C**A.AGA**G**.**A****G****G****C**.**A****G****A****G****G****G**A...**C****C****G**.**G**.**C****C****C**UUU**G****A****A**.**G****C****C**-.**C****A****G****C**AA**C****C**.**U**.--**C****A****G**U.......................UUAUA................................................CAAA**C****U****G****A****A**.U...**A**..**G****G**U**G**..**C****U**AAU**U****C****C****U****G**...**C****A****A****A****A****U**G...................CAAU.........GC**A****U****U****U****U****G**.GA.**A**..**G****A**..**U****A****A****A****A****C****U****C****A**ACUA | |
|  |  | NC\_003909.8/2536316-2536445  | AAAU**U****A****A****U****A****C****U****U**.**A****U****C**C.AGA**G**.**A****G****G****U**.**G****G****A****G****G****G**A...**A****C****G**.**G**.**C****C****C**UAU**G****A****A**.**A****C****C****U**.**C****A****G****C**AA**C****C**.-.**C****C****U****A****U**A.......................UAUAU..................................................UU**A****U****A****G****G**.A...**A**..**G****G**U**G**..**C****U**AAU**U****C****C****G****.**...**C****A****G****A****G****A**ACACG...............AUGU.........GU**U****U****U****U****U****G**.GA.**A**..**G****A**..**U****A****A****G****A****G****G****A****U**UCUU | |
|  |  | NC\_005957.1/2515869-2515998  | AAAU**U****A****A****U****A****C****U****U**.**A****U****C**C.AGA**G**.**A****G****G****U**.**G****G****A****G****G****G**A...**A****C****G**.**G**.**C****C****C**UAU**G****A****A**.**A****C****C****U**.**C****A****G****C**AA**C****C**.-.**C****C****U****A****U**G.......................UAAAU..................................................GC**A****U****A****G****G**.A...**A**..**G****G**U**G**..**C****U**AAU**U****C****C****G****.**...**C****A****G****A****G****A**ACACG...............UUGU.........GU**U****U****U****U****U****G**.GA.**A**..**G****A**..**U****A****A****G****A****G****G****A****U**UCUU | |
|  |  | NC\_006274.1/2520375-2520504  | AAAU**U****A****A****U****A****C****U****U**.**A****U****C**C.AGA**G**.**A****G****G****U**.**G****G****A****G****G****G**A...**A****C****G**.**G**.**C****C****C**UAU**G****A****A**.**A****C****C****U**.**C****A****G****C**AA**C****C**.-.**C****C****U****A****U**G.......................UAAAU..................................................GC**A****U****A****G****G**.A...**A**..**G****G**U**G**..**C****U**AAU**U****C****C****G****.**...**C****A****G****A****G****A**ACACG...............UUGU.........GU**U****U****U****U****U****G**.GA.**A**..**G****A**..**U****A****A****G****A****G****G****A****U**UCUU | |
|  |  | NC\_006270.2/969425-969552  | ACUA**U****A****C****U****U****C****U****U**.**A****U****U**C.AGA**G**.**A****G****G****C**.**G****G****A****G****G****G**AA..**U****U****G**.**G**.**C****C****C**UGU**G****A****A**.**A****C****C****U**.**C****G****G****C**AG**C****G**.**G**.**G****U****U****C****U**GC......................AUACA..................................................GC**A****G****A****A****U**.A...**C**..**U****G**U**G**..**C****C**ACA**U****C****C****A****A**...**C****A****A****G****C****C**....................GUAC..........G**G****G****C****U****U****G**.GA.**A**..**G****A**..**U****A****A****G****A****A****G****A****G**AGCG | |
|  |  | NC\_006322.1/969719-969846  | ACUA**U****A****C****U****U****C****U****U**.**A****U****U**C.AGA**G**.**A****G****G****C**.**G****G****A****G****G****G**AA..**U****U****G**.**G**.**C****C****C**UGU**G****A****A**.**A****C****C****U**.**C****G****G****C**AG**C****G**.**G**.**G****U****U****C****U**GC......................AUACA..................................................GC**A****G****A****A****U**.A...**C**..**U****G**U**G**..**C****C**ACA**U****C****C****A****A**...**C****A****A****G****C****C**....................GUAC..........G**G****G****C****U****U****G**.GA.**A**..**G****A**..**U****A****A****G****A****A****G****A****G**AGCG | |
|  |  | NC\_003909.8/471132-471006  | GAAU**A****U****A****C****C****U****U****U**.**A****U****C**A.AGA**G**.**A****G****G****C**.**A****G****A****G****G****G**A...**C****C****G**.**G**.**C****C****C**UUU**G****A****A**.**G****C****C**-.**C****A****G****C**AA**C****C**.**U**.--**C****A****G**U.......................UUAUA................................................CAAA**C****U****G****A****A**.U...**A**..**G****G**U**G**..**C****U**AAU**U****C****C****U****G**...**C****A****A****A****A****U**G...................CAUU.........GC**A****U****U****U****U****G**.AA.**A**..**G****A**..**U****A****A****A****A****C****G****U****A**ACUA | |
|  |  | NC\_006270.2/3302453-3302332  | UAUG**U****U****U****C****U****C****U****U**.**A****U****C**C.AGA**G**.**A****G****G****U**.**G****G****A****G****G****G**A...**A****G****U**.**G**.**C****C****C**UAU**G****A****A**.**A****C****C**-.**C****G****G****C**AA**C****C**.**A**.-**U****C****A****A**C.......................ACGU-...................................................G**U****U****G****A****A**.A...**U**..**G****G**U**G**..**C****C**AAU**U****C****A****C****G**...**C****G****A****A****G****C**....................GUUA..........U**G****C****U****U****U****G**.AA.**A**..**G****A**..**U****G****A****G****A****G****A****A****A**GGCC | |
|  |  | NC\_006322.1/3302623-3302502  | UAUG**U****U****U****C****U****C****U****U**.**A****U****C**C.AGA**G**.**A****G****G****U**.**G****G****A****G****G****G**A...**A****G****U**.**G**.**C****C****C**UAU**G****A****A**.**A****C****C**-.**C****G****G****C**AA**C****C**.**A**.-**U****C****A****A**C.......................ACGU-...................................................G**U****U****G****A****A**.A...**U**..**G****G**U**G**..**C****C**AAU**U****C****A****C****G**...**C****G****A****A****G****C**....................GUUA..........U**G****C****U****U****U****G**.AA.**A**..**G****A**..**U****G****A****G****A****G****A****A****A**GGCC | |
|  |  | NC\_000964.2/3363537-3363417  | UAUA**U****U****U****C****U****C****U****U**.**A****U****C**A.AGA**G**.**A****G****G****U**.**G****G****A****G****G****G**A...**A****G****U**.**G**.**C****C****C**UAU**G****A****A**.**G****C****C**-.**C****G****G****C**AA**C****C**.**A**.-**U****C****A****A**........................CACUG....................................................**U****U****G****A****A**.A...**U**..**G****G**U**G**..**C****C**AAU**U****C****A****C****A**...**C****G****A****A****G****C**....................GUUC..........A**G****C****U****U****U****G**.AA.**A**..**G****A**..**U****G****A****G****A****G****A****A****A**GGCA | |
|  |  | NC\_000964.2/1426177-1426291  | CUAU**A****U****U****U****U****C****U****U**.**A****U****C**A.AGA**G**C**A****G****G****C**.**A****G****A****G****G****G**A...**C****G****A**.**G**.**C****C****C**GAU**G****A****A**.**G****C****C**-.**C****G****G****C**AA**C****C**.**G**.**A****C****U**--........................UAUAA....................................................--**A****G****C**.A...**C**..**G****G**U**G**..**C****U**AAU**U****C****U****U****G**...**C****A****G****C****U**-....................-AGC...........-**G****G****C****U****G**.AG.**A**..**G****A**..**U****A****A****G****A****U****U****C****G**GACG | |
|  |  | NC\_005957.1/188037-188156  | AGCA**A****U****U****U****A****C****U****U**.**A****U****C**C.AGA**G**.**A****G****G****U**.**A****G****A****G****G****G**A...**C****U****G**.**G**.**C****C****C**UAU**G****A****C**.**A****C****C****U**.**C****A****G****C**AG**C****G**.**G**.**G****U****U****C****U**G.......................CAAGA....................................................**U****G****A****A****C**.A...**C**..**C****G**U**G**..**C****U**AAU**U****C****C****A****G**...**C****A****A****G****C**-....................-AAG...........-**U****C****U****U****G**.AA.**A**..**G****A**..**U****A****A****G****U****G****A****U****G**GGCC | |
|  |  | NC\_006177.1/138150-138272  | CCGA**A****U****A****C****U****C****U****U**.**A****U****C**A.AGA**G**.**A****A****G****C**.**G****G****A****G****G****G**AC..**C****U****G**.**G**.**C****C****C**GAU**G****A****A**.**G****C****U****U**.**C****G****G****C**AA**C****C**.**A**.**G****C****C****U****G**C.......................GUCAC....................................................**C****A****G****G****C**.A...**A**..**G****G**U**G**..**C****C**AAG**U****C****C****A****G**...**C****C****G****G****G****C**....................-AUA...........**C****U****A****C****C****G**.GG.**A**..**G****A**..**U****A****A****G****A****G****G****G****A**AGAG | |
|  |  | NC\_004193.1/2856872-2856750  | UUAU**U****U****U****U****C****C****U****U**.**A****U****C**A.AGA**G**U**C****G****G****G**.**G****G****A****G****G****A**AU..**C****U****G**.**G**.**U****C****C**AUU**G****A****U**.**C****C****C****G**.**C****A****G****C**AA**C****C**.**A**.**G****U****U****A****C**A.......................AUGAA....................................................**G****U****A****A****C**.A...**U**..**G****G**U**G**..**C****U**CAU**U****C****C****A****G**...**C****A****A****G****C**-....................GUAG...........-**G****C****U****U****G**.AU.**A**..**G****A**..**U****G****A****G****A****A****A****A****G**UGUU | |
|  |  | NC\_003997.3/2459353-2459482  | AAAU**U****A****A****U****A****C****U****U**.**A****U****C**C.AGA**G**.**A****G****G****U**.**G****G****A****G****G****G**A...**A****C****G**.**G**.**C****C****C**UAU**G****A****A**.**A****C****C****U**.**C****A****G****C**AA**C****C**.-.**C****C****U****A****U**G.......................UAAAU..................................................GC**A****U****A****G****G**.A...**A**..**G****G**U**G**..**C****U**AAU**U****C****C****G****.**...**C****A****G****A****G****A**ACACG...............UUGU.........GU**U****U****U****U****U****G**.GA.**A**..**G****A**..**U****G****A****G****A****G****G****A****U**UCUU | |
|  |  | NC\_005945.1/2459405-2459534  | AAAU**U****A****A****U****A****C****U****U**.**A****U****C**C.AGA**G**.**A****G****G****U**.**G****G****A****G****G****G**A...**A****C****G**.**G**.**C****C****C**UAU**G****A****A**.**A****C****C****U**.**C****A****G****C**AA**C****C**.-.**C****C****U****A****U**G.......................UAAAU..................................................GC**A****U****A****G****G**.A...**A**..**G****G**U**G**..**C****U**AAU**U****C****C****G****.**...**C****A****G****A****G****A**ACACG...............UUGU.........GU**U****U****U****U****U****G**.GA.**A**..**G****A**..**U****G****A****G****A****G****G****A****U**UCUU | |
|  |  | NC\_007530.2/2459477-2459606  | AAAU**U****A****A****U****A****C****U****U**.**A****U****C**C.AGA**G**.**A****G****G****U**.**G****G****A****G****G****G**A...**A****C****G**.**G**.**C****C****C**UAU**G****A****A**.**A****C****C****U**.**C****A****G****C**AA**C****C**.-.**C****C****U****A****U**G.......................UAAAU..................................................GC**A****U****A****G****G**.A...**A**..**G****G**U**G**..**C****U**AAU**U****C****C****G****.**...**C****A****G****A****G****A**ACACG...............UUGU.........GU**U****U****U****U****U****G**.GA.**A**..**G****A**..**U****G****A****G****A****G****G****A****U**UCUU | |
|  |  | NZ\_AAAC02000001.1/2934219-2934348  | AAAU**U****A****A****U****A****C****U****U**.**A****U****C**C.AGA**G**.**A****G****G****U**.**G****G****A****G****G****G**A...**A****C****G**.**G**.**C****C****C**UAU**G****A****A**.**A****C****C****U**.**C****A****G****C**AA**C****C**.-.**C****C****U****A****U**G.......................UAAAU..................................................GC**A****U****A****G****G**.A...**A**..**G****G**U**G**..**C****U**AAU**U****C****C****G****.**...**C****A****G****A****G****A**ACACG...............UUGU.........GU**U****U****U****U****U****G**.GA.**A**..**G****A**..**U****G****A****G****A****G****G****A****U**UCUU | |
|  |  | NZ\_AAEN01000016.1/757907-758036  | AAAU**U****A****A****U****A****C****U****U**.**A****U****C**C.AGA**G**.**A****G****G****U**.**G****G****A****G****G****G**A...**A****C****G**.**G**.**C****C****C**UAU**G****A****A**.**A****C****C****U**.**C****A****G****C**AA**C****C**.-.**C****C****U****A****U**G.......................UAAAU..................................................GC**A****U****A****G****G**.A...**A**..**G****G**U**G**..**C****U**AAU**U****C****C****G****.**...**C****A****G****A****G****A**ACACG...............UUGU.........GU**U****U****U****U****U****G**.GA.**A**..**G****A**..**U****G****A****G****A****G****G****A****U**UCUU | |
|  |  | NZ\_AAEO01000022.1/417585-417714  | AAAU**U****A****A****U****A****C****U****U**.**A****U****C**C.AGA**G**.**A****G****G****U**.**G****G****A****G****G****G**A...**A****C****G**.**G**.**C****C****C**UAU**G****A****A**.**A****C****C****U**.**C****A****G****C**AA**C****C**.-.**C****C****U****A****U**G.......................UAAAU..................................................GC**A****U****A****G****G**.A...**A**..**G****G**U**G**..**C****U**AAU**U****C****C****G****.**...**C****A****G****A****G****A**ACACG...............UUGU.........GU**U****U****U****U****U****G**.GA.**A**..**G****A**..**U****G****A****G****A****G****G****A****U**UCUU | |
|  |  | NZ\_AAEP01000025.1/557513-557642  | AAAU**U****A****A****U****A****C****U****U**.**A****U****C**C.AGA**G**.**A****G****G****U**.**G****G****A****G****G****G**A...**A****C****G**.**G**.**C****C****C**UAU**G****A****A**.**A****C****C****U**.**C****A****G****C**AA**C****C**.-.**C****C****U****A****U**G.......................UAAAU..................................................GC**A****U****A****G****G**.A...**A**..**G****G**U**G**..**C****U**AAU**U****C****C****G****.**...**C****A****G****A****G****A**ACACG...............UUGU.........GU**U****U****U****U****U****G**.GA.**A**..**G****A**..**U****G****A****G****A****G****G****A****U**UCUU | |
|  |  | NZ\_AAEQ01000035.1/774145-774016  | AAAU**U****A****A****U****A****C****U****U**.**A****U****C**C.AGA**G**.**A****G****G****U**.**G****G****A****G****G****G**A...**A****C****G**.**G**.**C****C****C**UAU**G****A****A**.**A****C****C****U**.**C****A****G****C**AA**C****C**.-.**C****C****U****A****U**G.......................UAAAU..................................................GC**A****U****A****G****G**.A...**A**..**G****G**U**G**..**C****U**AAU**U****C****C****G****.**...**C****A****G****A****G****A**ACACG...............UUGU.........GU**U****U****U****U****U****G**.GA.**A**..**G****A**..**U****G****A****G****A****G****G****A****U**UCUU | |
|  |  | NZ\_AAER01000040.1/190221-190350  | AAAU**U****A****A****U****A****C****U****U**.**A****U****C**C.AGA**G**.**A****G****G****U**.**G****G****A****G****G****G**A...**A****C****G**.**G**.**C****C****C**UAU**G****A****A**.**A****C****C****U**.**C****A****G****C**AA**C****C**.-.**C****C****U****A****U**G.......................UAAAU..................................................GC**A****U****A****G****G**.A...**A**..**G****G**U**G**..**C****U**AAU**U****C****C****G****.**...**C****A****G****A****G****A**ACACG...............UUGU.........GU**U****U****U****U****U****G**.GA.**A**..**G****A**..**U****G****A****G****A****G****G****A****U**UCUU | |
|  |  | NZ\_AAES01000035.1/359808-359937  | AAAU**U****A****A****U****A****C****U****U**.**A****U****C**C.AGA**G**.**A****G****G****U**.**G****G****A****G****G****G**A...**A****C****G**.**G**.**C****C****C**UAU**G****A****A**.**A****C****C****U**.**C****A****G****C**AA**C****C**.-.**C****C****U****A****U**G.......................UAAAU..................................................GC**A****U****A****G****G**.A...**A**..**G****G**U**G**..**C****U**AAU**U****C****C****G****.**...**C****A****G****A****G****A**ACACG...............UUGU.........GU**U****U****U****U****U****G**.GA.**A**..**G****A**..**U****G****A****G****A****G****G****A****U**UCUU | |
|  |  | NZ\_AAAS03000001.1/385327-385446  | GAUU**C****A****U****U****U****C****U****U**.**A****U****C**A.CGA**G**.**C****G****A****C**.**C****G****A****G****G****G**A...**C****U****G**.**G**.**C****C****C**UAU**G****A****C**.**G****U****C****G**.**C****G****G****C**AA**C****C**.-.**C****C****C****C**-........................GCAA-....................................................-**G****G****G****G**.A...**C**..**G****G**U**G**..**C****C**AAA**U****C****C****U****G**...**C****G****G****A****A****C**G...................GCAA..........C**G****U****U****C****C****G**.GG.**A**..**G****A**..**U****A****A****G****G****A****A****G****A**GCGU | |
|  |  | NC\_006582.1/951399-951519  | AAAA**A****A****G****G****A****C****U****U**.**A****U****C**A.AGA**G**.**C****G****A****C**.**U****G****A****G****G****G**AU..**U****A****G**.**G**.**C****C****C**AAU**G****A****C**.**G****U****C**-.**C****A****G****C**AA**C****C**.-.**U****C****C****C**-........................GUUAC....................................................-**G****G****G****G**.A...**A**..**G****G**U**G**..**C****C**AAU**U****C****C****U****G**...**C****A****G****A****A****U**G...................GGAU..........C**A****U****U****C****U****G**.AA.**A**..**G****A**..**U****A****A****G****C****C****G****G****A**AAAA | |
|  |  | NC\_003997.3/3892924-3893045  | UAAA**U****A****C****U****U****C****U****U**.**A****U****C**A.AGA**G**C**A****G****G****U**.**G****G****A****G****G****G**A...**C****G****A**.**G**.**C****C****C**GAC**G****A****A**.**A****C****C**-.**C****G****G****C**AA**C****C**.**G**.**A****U****C****U****A**C.......................AUAAU..................................................UG**U****A****G****A****C**.A...**C**..**G****G**U**G**..**C****U**AAU**U****C****U****C****G**...**C****A****G****C****A**-....................-UUA...........-**C****G****C****U****G**.AC.**A**..**G****A**..**U****A****A****G****G****A****G****C****U**GGUU | |
|  |  | NC\_005945.1/3893424-3893545  | UAAA**U****A****C****U****U****C****U****U**.**A****U****C**A.AGA**G**C**A****G****G****U**.**G****G****A****G****G****G**A...**C****G****A**.**G**.**C****C****C**GAC**G****A****A**.**A****C****C**-.**C****G****G****C**AA**C****C**.**G**.**A****U****C****U****A**C.......................AUAAU..................................................UG**U****A****G****A****C**.A...**C**..**G****G**U**G**..**C****U**AAU**U****C****U****C****G**...**C****A****G****C****A**-....................-UUA...........-**C****G****C****U****G**.AC.**A**..**G****A**..**U****A****A****G****G****A****G****C****U**GGUU | |
|  |  | NC\_005957.1/3872406-3872527  | UAAA**U****A****C****U****U****C****U****U**.**A****U****C**A.AGA**G**C**A****G****G****U**.**G****G****A****G****G****G**A...**C****G****A**.**G**.**C****C****C**GAC**G****A****A**.**A****C****C**-.**C****G****G****C**AA**C****C**.**G**.**A****U****C****U****A**C.......................AUAAU..................................................UG**U****A****G****A****C**.A...**C**..**G****G**U**G**..**C****U**AAU**U****C****U****C****G**...**C****A****G****C****A**-....................-UUA...........-**C****G****C****U****G**.AC.**A**..**G****A**..**U****A****A****G****G****A****G****C****U**GGUU | |
|  |  | NC\_006274.1/3929424-3929545  | UAAA**U****A****C****U****U****C****U****U**.**A****U****C**A.AGA**G**C**A****G****G****U**.**G****G****A****G****G****G**A...**C****G****A**.**G**.**C****C****C**GAC**G****A****A**.**A****C****C**-.**C****G****G****C**AA**C****C**.**G**.**A****U****C****U****A**C.......................AUAAU..................................................UG**U****A****G****A****C**.A...**C**..**G****G**U**G**..**C****U**AAU**U****C****U****C****G**...**C****A****G****C****A**-....................-UUA...........-**C****G****C****U****G**.AC.**A**..**G****A**..**U****A****A****G****G****A****G****C****U**GGUU | |
|  |  | NC\_007530.2/3893051-3893172  | UAAA**U****A****C****U****U****C****U****U**.**A****U****C**A.AGA**G**C**A****G****G****U**.**G****G****A****G****G****G**A...**C****G****A**.**G**.**C****C****C**GAC**G****A****A**.**A****C****C**-.**C****G****G****C**AA**C****C**.**G**.**A****U****C****U****A**C.......................AUAAU..................................................UG**U****A****G****A****C**.A...**C**..**G****G**U**G**..**C****U**AAU**U****C****U****C****G**...**C****A****G****C****A**-....................-UUA...........-**C****G****C****U****G**.AC.**A**..**G****A**..**U****A****A****G****G****A****G****C****U**GGUU | |
|  |  | NZ\_AAAC02000001.1/4342578-4342699  | UAAA**U****A****C****U****U****C****U****U**.**A****U****C**A.AGA**G**C**A****G****G****U**.**G****G****A****G****G****G**A...**C****G****A**.**G**.**C****C****C**GAC**G****A****A**.**A****C****C**-.**C****G****G****C**AA**C****C**.**G**.**A****U****C****U****A**C.......................AUAAU..................................................UG**U****A****G****A****C**.A...**C**..**G****G**U**G**..**C****U**AAU**U****C****U****C****G**...**C****A****G****C****A**-....................-UUA...........-**C****G****C****U****G**.AC.**A**..**G****A**..**U****A****A****G****G****A****G****C****U**GGUU | |
|  |  | NZ\_AAEN01000013.1/147434-147555  | UAAA**U****A****C****U****U****C****U****U**.**A****U****C**A.AGA**G**C**A****G****G****U**.**G****G****A****G****G****G**A...**C****G****A**.**G**.**C****C****C**GAC**G****A****A**.**A****C****C**-.**C****G****G****C**AA**C****C**.**G**.**A****U****C****U****A**C.......................AUAAU..................................................UG**U****A****G****A****C**.A...**C**..**G****G**U**G**..**C****U**AAU**U****C****U****C****G**...**C****A****G****C****A**-....................-UUA...........-**C****G****C****U****G**.AC.**A**..**G****A**..**U****A****A****G****G****A****G****C****U**GGUU | |
|  |  | NZ\_AAEO01000019.1/234540-234661  | UAAA**U****A****C****U****U****C****U****U**.**A****U****C**A.AGA**G**C**A****G****G****U**.**G****G****A****G****G****G**A...**C****G****A**.**G**.**C****C****C**GAC**G****A****A**.**A****C****C**-.**C****G****G****C**AA**C****C**.**G**.**A****U****C****U****A**C.......................AUAAU..................................................UG**U****A****G****A****C**.A...**C**..**G****G**U**G**..**C****U**AAU**U****C****U****C****G**...**C****A****G****C****A**-....................-UUA...........-**C****G****C****U****G**.AC.**A**..**G****A**..**U****A****A****G****G****A****G****C****U**GGUU | |
|  |  | NZ\_AAEP01000031.1/212555-212434  | UAAA**U****A****C****U****U****C****U****U**.**A****U****C**A.AGA**G**C**A****G****G****U**.**G****G****A****G****G****G**A...**C****G****A**.**G**.**C****C****C**GAC**G****A****A**.**A****C****C**-.**C****G****G****C**AA**C****C**.**G**.**A****U****C****U****A**C.......................AUAAU..................................................UG**U****A****G****A****C**.A...**C**..**G****G**U**G**..**C****U**AAU**U****C****U****C****G**...**C****A****G****C****A**-....................-UUA...........-**C****G****C****U****G**.AC.**A**..**G****A**..**U****A****A****G****G****A****G****C****U**GGUU | |
|  |  | NZ\_AAEQ01000038.1/168819-168940  | UAAA**U****A****C****U****U****C****U****U**.**A****U****C**A.AGA**G**C**A****G****G****U**.**G****G****A****G****G****G**A...**C****G****A**.**G**.**C****C****C**GAC**G****A****A**.**A****C****C**-.**C****G****G****C**AA**C****C**.**G**.**A****U****C****U****A**C.......................AUAAU..................................................UG**U****A****G****A****C**.A...**C**..**G****G**U**G**..**C****U**AAU**U****C****U****C****G**...**C****A****G****C****A**-....................-UUA...........-**C****G****C****U****G**.AC.**A**..**G****A**..**U****A****A****G****G****A****G****C****U**GGUU | |
|  |  | NZ\_AAER01000035.1/405769-405890  | UAAA**U****A****C****U****U****C****U****U**.**A****U****C**A.AGA**G**C**A****G****G****U**.**G****G****A****G****G****G**A...**C****G****A**.**G**.**C****C****C**GAC**G****A****A**.**A****C****C**-.**C****G****G****C**AA**C****C**.**G**.**A****U****C****U****A**C.......................AUAAU..................................................UG**U****A****G****A****C**.A...**C**..**G****G**U**G**..**C****U**AAU**U****C****U****C****G**...**C****A****G****C****A**-....................-UUA...........-**C****G****C****U****G**.AC.**A**..**G****A**..**U****A****A****G****G****A****G****C****U**GGUU | |
|  |  | NZ\_AAES01000024.1/145437-145558  | UAAA**U****A****C****U****U****C****U****U**.**A****U****C**A.AGA**G**C**A****G****G****U**.**G****G****A****G****G****G**A...**C****G****A**.**G**.**C****C****C**GAC**G****A****A**.**A****C****C**-.**C****G****G****C**AA**C****C**.**G**.**A****U****C****U****A**C.......................AUAAU..................................................UG**U****A****G****A****C**.A...**C**..**G****G**U**G**..**C****U**AAU**U****C****U****C****G**...**C****A****G****C****A**-....................-UUA...........-**C****G****C****U****G**.AC.**A**..**G****A**..**U****A****A****G****G****A****G****C****U**GGUU | |
|  |  | NC\_002976.3/480760-480876  | GCUU**U****A****A****C****U****C****U****U**.**A****U****C**G.AGA**G**.**A****G****G****U**.**G****G****A****G****G****G**A...**U****G****U**.**G**.**C****C****C**UAA**G****A****A**.**G****C****C**-.**C****G****G****C**AA**C****C**.**G**.-**U****C****U**-........................AAAAU....................................................-**A****G****A****A**.A...**U**..**G****G**U**G**..**C****C**AAU**U****C****A****C****A**...**U****A****A****A****G****U**....................-AUA...........**A****C****U****U****U****A**.GA.**A**..**G****A**..**U****G****A****G****A****G****A****A****A**GAAC | |
|  |  | NC\_004461.1/588088-588204  | GCUU**U****A****A****C****U****C****U****U**.**A****U****C**G.AGA**G**.**A****G****G****U**.**G****G****A****G****G****G**A...**U****G****U**.**G**.**C****C****C**UAA**G****A****A**.**G****C****C**-.**C****G****G****C**AA**C****C**.**G**.-**U****C****U**-........................AAAAU....................................................-**A****G****A****A**.A...**U**..**G****G**U**G**..**C****C**AAU**U****C****A****C****A**...**U****A****A****A****G****U**....................-AUA...........**A****C****U****U****U****A**.GA.**A**..**G****A**..**U****G****A****G****A****G****A****A****A**GAAC | |
|  |  | NC\_006270.2/3063546-3063425  | AAUA**U****G****C****A****G****C****U****U**.**A****U****A**A.AGA**G**.**A****G****A****U**.**G****G****A****G****G****G**A...**C****U****G**.**G**.**C****C****C**GGU**G****A****A**.**A****U****C****U**.**C****A****G****C**AA**C****C**.**U**.**G****C****A**--........................GCAG-....................................................--**U****G****C**.A...**A**..**G****G**U**G**..**C****U**AAA**U****C****C****A****G**...**C****A****G****A****A****U**G...................UCAG.......GCGC**A****U****U****C****U****G**.AA.**A**..**G****A**..**U****A****A****G****U****U****G****A****A**CAAC | |
|  |  | NC\_006322.1/3063723-3063602  | AAUA**U****G****C****A****G****C****U****U**.**A****U****A**A.AGA**G**.**A****G****A****U**.**G****G****A****G****G****G**A...**C****U****G**.**G**.**C****C****C**GGU**G****A****A**.**A****U****C****U**.**C****A****G****C**AA**C****C**.**U**.**G****C****A**--........................GCAG-....................................................--**U****G****C**.A...**A**..**G****G**U**G**..**C****U**AAA**U****C****C****A****G**...**C****A****G****A****A****U**G...................UCAG.......GCGC**A****U****U****C****U****G**.AA.**A**..**G****A**..**U****A****A****G****U****U****G****A****A**CAAC | |
|  |  | NC\_004193.1/3162066-3162189  | AGCA**A****A****U****C****U****C****U****U**.**A****U****C**A.AGA**G**.**U****G****G****U**.**G****G****A****G****G****G**AA..**U****A****G**.**G**.**C****C****C**UGC**G****A****A**.**G****C****C**-.**C****G****G****C**AA**C****C**.**U**.---**G****U**AGC.....................AAUU-.................................................GCU**A****U****U****G****A**.A...**A**..**G****G**U**G**..**C****U**AAA**U****C****C****U****A**...**C****A****G****A****C****U**....................UCAU...........**C****G****U****C****U****G**.GA.**A**..**G****A**..**U****A****A****G****A****G****G****A****G**GUUC | |
|  |  | NC\_003212.1/1772469-1772347  | AAUU**U****A****U****C****U****C****U****U**.**A****U****C**C.AGA**G**.**C****G****G****U**.**A****G****A****G****G****G**A...**C****U****G**.**A**.**C****C****C**UUU**G****A****A**.**G****C****C**-.**C****A****G****C**AA**C****C**.**U**.**A****C****A****C****A**........................UAUA-....................................................**A****G****U****G****A**.A...**A**..**G****G**U**G**..**C****U**AA--**U****C****U****G**UUG**C****A****G****G****A****G**U...................AAUA..........U**C****U****C****C****U****G**.AA.**C**..**G****A**..**U****G****A****G****A****G****C****A****A**AGGU | |
|  |  | NC\_006510.1/974629-974751  | GCAG**G****C****U****U****U****C****U****U**.**A****U****C**A.AGA**G**C**A****G****G****C**.**G****G****A****G****G****G**A...**C****G****A**.**G**.**C****C****C**AAU**G****A****A**.**G****C****C**-.**C****G****G****C**AA**C****C**.**G**.**G****C****U****U****G**G.......................CGCGC..................................................GC**C****A****A****G****C**.A...**C**..**G****G**U**G**..**C****U**AAU**U****C****U****U****G**...**C****A****G****C****G**-....................GAAA...........-**C****G****C****U****G**.AG.**A**..**G****A**..**U****A****A****G****A****A****G****A****G**CGCC | |
|  |  | NC\_006510.1/883125-883249  | CGUC**C****C****A****U****U****C****U****U**.**A****U****C**A.AGA**G**.**A****A****G****C**.**G****G****A****G****G****G**AA..**C****U****G**.**G**.**C****C****C**AAU**G****A****A**.**G****C****U****U**.**C****A****G****C**AA**C****C**.**A**.**G****C****C****G****C**........................-CCG-....................................................**G****C****G****G****C**.C...**A**..**G****G**U**G**..**C****U**AAA**U****C****C****A****G**...**C****G****A****A****U****U**GAG.................CAUU..........C**A****A****U****U****C****G**.GC.**A**..**G****A**..**U****A****A****G****A****A****G****A****A**GCAU | |
|  |  | NC\_004722.1/4007990-4007836  | UAUA**C****A****A****C****U****C****U****U**.**A****U****C**A.AGA**G**C**A****G****G****U**.**G****G****A****G****G****G**AU..**U****U****G**.**G**.**C****C****C**GAU**G****A****A**.**G****C****C**-.**C****A**-**C**AA**C****C**.**G**.**A****C****C****G****U**AAUACCAUUGUGAAAUGGGGCG..UUUAU............................................UACGCCAA**A****A****G****G****C**.A...**C**..**G****G**U**G**..**C****U**AAU**U****C****C****A****G**...**C****A****G****A****A****A**G...................UAAA.........AC**U****U****U****C****U****G**.GC.**A**..**G****A**..**U****A****A****G****A****G****G****G****G**AGAA | |
|  |  | NC\_003909.8/3836040-3836159  | UAAA**U****A****C****U****U****C****U****U**.**A****U****C**A.AGA**G**C**A****G****G****U**.**G****G****A****G****G****G**A...**C****G****A**.**G**.**C****C****C**GAC**G****A****A**.**A****C****C**-.**C****G****G****C**AA**C****C**.**G**.**A****U****C****U****A**C.......................AAUU-...................................................G**U****A****G****A****C**.A...**C**..**G****G**U**G**..**C****U**AAU**U****C****U****C****G**...**C****A****G****C****A**-....................-UUA...........-**C****G****C****U****G**.AC.**A**..**G****A**..**U****A****A****G****G****A****G****C****U**GGUU | |
|  |  | NC\_004722.1/4010169-4010288  | UAAA**U****A****C****U****U****C****U****U**.**A****U****C**A.AGA**G**C**A****G****G****U**.**G****G****A****G****G****G**A...**C****G****A**.**G**.**C****C****C**GAC**G****A****A**.**A****C****C**-.**C****G****G****C**AA**C****C**.**G**.**A****U****C****U****A**C.......................AAUU-...................................................G**U****A****G****A****C**.A...**C**..**G****G**U**G**..**C****U**AAU**U****C****U****C****G**...**C****A****G****C****A**-....................-UUA...........-**C****G****C****U****G**.AC.**A**..**G****A**..**U****A****A****G****G****A****G****C****U**GGUU | |
|  |  | NZ\_AAEK01000007.1/32025-31906  | UAAA**U****A****C****U****U****C****U****U**.**A****U****C**A.AGA**G**C**A****G****G****U**.**G****G****A****G****G****G**A...**C****G****A**.**G**.**C****C****C**GAC**G****A****A**.**A****C****C**-.**C****G****G****C**AA**C****C**.**G**.**A****U****C****U****A**C.......................AAUU-...................................................G**U****A****G****A****C**.A...**C**..**G****G**U**G**..**C****U**AAU**U****C****U****C****G**...**C****A****G****C****A**-....................-UUA...........-**C****G****C****U****G**.AC.**A**..**G****A**..**U****A****A****G****G****A****G****C****U**GGUU | |
|  |  | NC\_006270.2/1488837-1488950  | AUCA**A****U****A****U****U****C****U****U**.**A****U****C**A.AGA**G**C**A****G****G****C**.**A****G****A****G****G****G**A...**C****A****A**.**G**.**C****C****C**GAU**G****A****A**.**G****C****C**-.**C****G****G****C**AA**C****C**.**G**.**A****C****U**--........................UUUUA....................................................--**A****G****C**.A...**C**..**G****G**U**G**..**C****U**AAU**U****C****U****U****G**...**C****A****G****C**--....................UGAC...........--**G****C****U****G**.AG.**A**..**G****A**..**U****A****A****G****G****A****U****U****C**GAAC | |
|  |  | NC\_006322.1/1489698-1489811  | AUCA**A****U****A****U****U****C****U****U**.**A****U****C**A.AGA**G**C**A****G****G****C**.**A****G****A****G****G****G**A...**C****A****A**.**G**.**C****C****C**GAU**G****A****A**.**G****C****C**-.**C****G****G****C**AA**C****C**.**G**.**A****C****U**--........................UUUUA....................................................--**A****G****C**.A...**C**..**G****G**U**G**..**C****U**AAU**U****C****U****U****G**...**C****A****G****C**--....................UGAC...........--**G****C****U****G**.AG.**A**..**G****A**..**U****A****A****G****G****A****U****U****C**GAAC | |
|  |  | NC\_004193.1/2134373-2134257  | AUUG**A****A****U****A****A****C****U****U**.**A****U****C**C.AGA**G**.**U****G****A****C**.**G****G****A****G****G****G**AA..**C****A****G**.**G**.**A****C****C**UAC**G****A****U**.**G****U****C****A**.**C****A****G****C**AA**C****C**.**U**.**A****C****C**--........................UUUAC....................................................--**G****G****A**.G...**U**..**G****G**U**G**..**C****U**UCU**U****C****C****U****G**...**C****A****G****A****A**-....................UUUU...........-**U****U****C****U****G**.AA.**A**..**G****A**..**U****A****A****G****G****U****A****A****U**GAUA | |
|  |  | NC\_004193.1/3200757-3200878  | UUAA**U****A****C****U****U****C****U****U**.**A****U****C**G.AGA**G**.**A****A****G****C**.**U****A****A****G****G****G**AC..**C****U****G**.**G**.**C****C****U**GUU**G****A****C**.**G****C****U****U**.**C****A****G****C**AA**C****C**.**U**.-**C****U****A****U**........................CUCCA....................................................**U****U****A****G****A**.A...**A**..**G****G**U**G**..**C****U**ACC**U****C****C****A****G**...**C****A****A****G****A****U**....................GUAU...........**G****U****C****U****U****G**.AA.**A**..**G****A**..**U****A****A****G****A****G****U****C****C**AGAU | |
|  |  | NC\_006582.1/1983205-1983318  | GAAU**A****G****G****U****U****C****U****U**.**A****U****C**A.AGA**G**.**A****A****G****U**.**G****G****A****G****G****G**A...**A****U****G**.**G**.**C****C****C**AAU**G****A****A**.**G****C****U****U**.**C****A****G****C**AA**C****C**.**A**.**G****C****C****A**-........................ACCA-....................................................-**U****G****G****U**.C...**A**..**G****G**U**G**..**C****U**AAA**U****C****C****A****G**...**C****A****G****U**--....................-UUA...........--**U****C****U****G**.CA.**A**..**G****A**..**U****A****A****G****A****G****A****A****G**CAUG | |
|  |  | NC\_003366.1/2665238-2665061  | UUAA**U****A****A****A****U****C****U****U**.**A****U****C**A.AGA**G**.**A****G****G****U**.**G****G****A****G****G****G**A...**C****U****G**.**G**.**C****C****C**UGU**G****A****A**.**A****C****C**-.**C****A****G****C**AA**C****C**.**G**.-**G****U****A****A**UUCUUUGCGG..............UUAAAACAAUGCUGAUUUUAAAAUAAAAAAAUCAGUAGUAAUUUCCUAUGCAAAGAU**U****U****A****U****A**.G...**C**..**G****G**U**G**..**C****U**AAA**U****C****C****U****G**...**C****G****G****U**--....................AGAA...........--**A****C****U****G**.AG.**A**..**G****A**..**U****A****A****G****A****A****A****G****A**GAGU | |
|  |  | NC\_002745.2/2381652-2381543  | UAAG**C****A****U****C****A****C****U****U**.**A****U****C**U.AGA**G**.**A****G****G****U**.**G****G****A****G****G****G**A...**C****U****G**.**G**.**C****C****C**UAU**G****A****A**.**G****C****C****U**.**C****G****G****C**AA**C****A**.**U**.-----........................CUCGA....................................................-----.-...**A**..**U****G**U**G**..**C****C**AAU**U****C****C****A****G**...**U****A****A****C****C****G**....................-UAA...........**U****G****G****U****U****U**.GA.**A**..**G****A**..**U****A****A****G****C****A****G****G****U**AAAG | |
|  |  | NC\_002758.2/2451152-2451043  | UAAG**C****A****U****C****A****C****U****U**.**A****U****C**U.AGA**G**.**A****G****G****U**.**G****G****A****G****G****G**A...**C****U****G**.**G**.**C****C****C**UAU**G****A****A**.**G****C****C****U**.**C****G****G****C**AA**C****A**.**U**.-----........................CUCGA....................................................-----.-...**A**..**U****G**U**G**..**C****C**AAU**U****C****C****A****G**...**U****A****A****C****C****G**....................-UAA...........**U****G****G****U****U****U**.GA.**A**..**G****A**..**U****A****A****G****C****A****G****G****U**AAAG | |
|  |  | NC\_002951.2/2381009-2380900  | UAAG**C****A****U****C****A****C****U****U**.**A****U****C**U.AGA**G**.**A****G****G****U**.**G****G****A****G****G****G**A...**C****U****G**.**G**.**C****C****C**UAU**G****A****A**.**G****C****C****U**.**C****G****G****C**AA**C****A**.**U**.-----........................CUCGA....................................................-----.-...**A**..**U****G**U**G**..**C****C**AAU**U****C****C****A****G**...**U****A****A****C****C****G**....................-UAA...........**U****G****G****U****U****U**.GA.**A**..**G****A**..**U****A****A****G****C****A****G****G****U**AAAG | |
|  |  | NC\_002952.2/2480784-2480675  | UAAG**C****A****U****C****A****C****U****U**.**A****U****C**U.AGA**G**.**A****G****G****U**.**G****G****A****G****G****G**A...**C****U****G**.**G**.**C****C****C**UAU**G****A****A**.**G****C****C****U**.**C****G****G****C**AA**C****A**.**U**.-----........................CUCGA....................................................-----.-...**A**..**U****G**U**G**..**C****C**AAU**U****C****C****A****G**...**U****A****A****C****C****G**....................-UAA...........**U****G****G****U****U****U**.GA.**A**..**G****A**..**U****A****A****G****C****A****G****G****U**AAAG | |
|  |  | NC\_002953.3/2372971-2372862  | UAAG**C****A****U****C****A****C****U****U**.**A****U****C**U.AGA**G**.**A****G****G****U**.**G****G****A****G****G****G**A...**C****U****G**.**G**.**C****C****C**UAU**G****A****A**.**G****C****C****U**.**C****G****G****C**AA**C****A**.**U**.-----........................CUCGA....................................................-----.-...**A**..**U****G**U**G**..**C****C**AAU**U****C****C****A****G**...**U****A****A****C****C****G**....................-UAA...........**U****G****G****U****U****U**.GA.**A**..**G****A**..**U****A****A****G****C****A****G****G****U**AAAG | |
|  |  | NC\_003923.1/2393866-2393757  | UAAG**C****A****U****C****A****C****U****U**.**A****U****C**U.AGA**G**.**A****G****G****U**.**G****G****A****G****G****G**A...**C****U****G**.**G**.**C****C****C**UAU**G****A****A**.**G****C****C****U**.**C****G****G****C**AA**C****A**.**U**.-----........................CUCGA....................................................-----.-...**A**..**U****G**U**G**..**C****C**AAU**U****C****C****A****G**...**U****A****A****C****C****G**....................-UAA...........**U****G****G****U****U****U**.GA.**A**..**G****A**..**U****A****A****G****C****A****G****G****U**AAAG | |
|  |  | NZ\_AADW02000012.1/72076-71959  | UGAC**U****U****C****U****U****U****U****U**.**A****U****C**G.CGA**G**.**A****G****A****C**.**G****G****A****G****G****G**A...**C****U****G**.**G**.**C****C****C**GAU**G****A****U**.**G****U****U****U**.**C****G****G****C**AG**C****G**.**G**.**A****C****G****A**-........................UUAAA....................................................-**U****C****G****U**.A...**C**..**U****G**U**G**..**C****C**AAA**U****C****C****A****G**...**C****A****A****G****C**-....................UGCG...........-**G****C****U****U****G**.AG.**A**..**G****A**..**U****A****A****G****A****A****G****A****G**CGUC | |
|  |  | NZ\_AADW02000008.1/46455-46570  | AAUU**A****C****A****U****U****C****U****U**.**A****U****C**C.AGA**G**.**A****G****G****U**.**G****G****A****G****G****G**A...**C****U****G**.**G**.**C****C****C**UUU**G****A****A**.**G****C****C****U**.**C****A****G****C**AA**C****A**.**G**.**G****U****C**--........................GAAA-....................................................--**G****A****C**.A...**C**..**U****G**U**G**..**C****U**AAU**U****C****C****U****G**...**C****G****G****G****U****G**....................-UUG...........**U****A****C****C****C****G**.AU.**C**..**G****A**..**U****A****A****G****C****U****U****C****C**UUUG | |
|  |  | NC\_004557.1/1456777-1456655  | UAAA**A****A****A****A****G****C****U****U**.**A****U****U**A.AGA**G**.**C****G****G****U**.**G****G****A****G****G****G**A...**C****U****G**.**G**.**C****C****C**UAU**G****A****A**.**G****C****C**-.**C****G****G****C**AA**C****C**.**U**.**G****U****A**-**U**AUG.....................UGUUU................................................GCAU**A****U****U****A****U**.A...**A**..**G****G**U**G**..**C****U**AAA**U****C****C****U****G**...**C****G****G****U**--....................GUAA...........--**A****C****C****G**.AG.**A**..**G****A**..**U****G****A****G****G****A****U****U****U**GAAG | |
|  |  | NC\_002973.5/1697696-1697574  | AAUU**U****A****U****C****U****C****U****U**.**A****U****C**C.AGA**G**.**C****G****G****U**.**A****G****A****G****G****G**A...**C****U****G**.**A**.**C****C****C**UUU**G****A****A**.**G****C****C**-.**C****A****G****C**AA**C****C**.**U**.**A****C****A****C****A**........................UAUA-....................................................**A****G****U****G****A**.A...**A**..**G****G**U**G**..**C****U**AA--**U****C****U****G**UUG**C****A****G****G****A****G**U...................AUUA..........U**C****U****U****C****U****G**.AA.**C**..**G****A**..**U****G****A****G****A****G****C****A****A**AGGU | |
|  |  | NC\_003210.1/1716658-1716536  | AAUU**U****A****U****C****U****C****U****U**.**A****U****C**C.AGA**G**.**C****G****G****U**.**A****G****A****G****G****G**A...**C****U****G**.**A**.**C****C****C**UUU**G****A****A**.**G****C****C**-.**C****A****G****C**AA**C****C**.**U**.**A****C****A****C****A**........................UAUA-....................................................**A****G****U****G****A**.A...**A**..**G****G**U**G**..**C****U**AA--**U****C****U****G**UUG**C****A****G****G****A****G**U...................AUUA..........U**C****U****U****C****U****G**.AA.**C**..**G****A**..**U****G****A****G****A****G****C****A****A**AGGU | |
|  |  | NZ\_AADQ01000032.1/29537-29659  | AAUU**U****A****U****C****U****C****U****U**.**A****U****C**C.AGA**G**.**C****G****G****U**.**A****G****A****G****G****G**A...**C****U****G**.**A**.**C****C****C**UUU**G****A****A**.**G****C****C**-.**C****A****G****C**AA**C****C**.**U**.**A****C****A****C****A**........................UAUA-....................................................**A****G****U****G****A**.A...**A**..**G****G**U**G**..**C****U**AA--**U****C****U****G**UUG**C****A****G****G****A****G**U...................AUUA..........U**C****U****U****C****U****G**.AA.**C**..**G****A**..**U****G****A****G****A****G****C****A****A**AGGU | |
|  |  | NZ\_AADR01000096.1/1559-1437  | AAUU**U****A****U****C****U****C****U****U**.**A****U****C**C.AGA**G**.**C****G****G****U**.**A****G****A****G****G****G**A...**C****U****G**.**A**.**C****C****C**UUU**G****A****A**.**G****C****C**-.**C****A****G****C**AA**C****C**.**U**.**A****C****A****C****A**........................UAUA-....................................................**A****G****U****G****A**.A...**A**..**G****G**U**G**..**C****U**AA--**U****C****U****G**UUG**C****A****G****G****A****G**U...................AUUA..........U**C****U****U****C****U****G**.AA.**C**..**G****A**..**U****G****A****G****A****G****C****A****A**AGGU | |
|  |  | NC\_006582.1/1701642-1701766  | GAAU**A****A****G****A****A****C****U****U**.**A****U****C**A.AGA**G**.**U****G****G****C**.**G****G****A****G****G****G**AC..**C****U****G**.**A**.**C****C****C**AAU**G****A****A**.**G****C****C**-.**C****G****G****C**AA**C****C**.-.**A****G****U****U****U**A.......................UUUGU....................................................**A****A****A****C****C**.A...**C**..**G****G**U**G**..**C****U**ACA**U****U****C****A****G**...**C****A****G****A****G****C**AG..................CUUU..........U**G****U****U****C****U****G**.AA.**A**..**G****A**..**U****A****A****G****U****G****A****G****G**CGAA | |
|  |  | NC\_006582.1/3008456-3008335  | CAUA**A****C****G****C****U****U****U****U**.**A****U****C**C.AGA**G**A**U****G****G****C**.**G****G****A****G****G****G**A...**C****A****G**.**G**.**C****C****C**GAA**G****A****A**.**G****C****C**-.**C****A****G****C**AA**C****C**.-.**A****A****C****A****C**........................GUAAC....................................................**G****U****G****U****A**.A...**A**..**G****G**U**G**..**C****U**AA--**C****C****U****G**...**C****A****G****A****A****U**G...................CUCG.........GC**G****U****U****C****U****G**.GA.**A**..**G****A**..**U****A****A****G****A****G****G****C****G**AAAG | |
|  |  | NC\_003212.1/871741-871851  | ACAU**A****G****U****A****A****C****U****U**.**A****U****C**A.AGA**A**.**A****G****G****U**.**G****G****A****G****G****G**UU..**C****U****G**.**G**.**C****C****C**AGU**G****A****A**.**G****C****C****U**.**U****G****G****C**AA**C****C**.**G**.**G****A**---........................CUUU-....................................................---**U****C**.A...**C**..**G****G**U**G**..**C****C**AAA**U****C****C****A****G**...**C****A****G****U**--....................-AUC...........--**A****C****U****G**.AC.**A**..**G****A**..**U****A****A****G****G****C****A****C****G**CGAA | |
|  |  | NC\_004722.1/2587946-2588075  | AAAU**U****A****A****U****A****C****U****U**.**A****U****C**C.AGA**G**.**A****G****G****U**.**G****G****A****G****G****G**A...**A****C****G**.**G**.**C****C****C**UAA**A****A****A**.**A****C****C****U**.**C****A****G****C**AA**C****C**.-.**C****C****U****A****U**GCA.....................AUUUU....................................................**A****U****A****G****G**.A...**A**..**G****G**U**G**..**C****U**AAU**U****C****C**-**G**...**C****A****G****A****G****G**ACACG...............AUGU.........GU**U****U****U****U****U****G**.AA.**A**..**G****A**..**U****A****A****G****A****G****G****A****U**UCUU | |
|  |  | NZ\_AAAH01000942.2/4682-4807  | ACAC**A****C****G****C****A****C****U****C**.**A****U****C**C.AGA**G**.**C****G****G****U**.**G****G****A****G****G****G**A...**C****C****G**.**G**.**C****C****C**GUU**G****A****A**.**A****C****C****G**.**C****A****G****C**AA**C****C**.**C**.**U****C****G****U****A**UGC.....................GUCGC................................................CGCA**U****A****C****G****A**.A...**C**..**G****G**U**G**..**C****U**AAG**U****C****C****G****G**...**C****A****G****A****G**-....................-GUG...........-**U****U****C****U****G**.GA.**A**..**G****A**..**U****G****A****G****C****G****A****C****G**UGGA | |
|  |  | NZ\_AADW02000005.1/169417-169533  | UAUA**U****G****A****C****U****C****U****U**.**A****U****C**G.UGA**G**U**U****G****G****C**.**A****G****A****G****G****G**AA..**U****U****G**.**G**.**C****C****C**GAA**G****A****C**.**G****C****C****G**.**C****A****G****C**AA**C****C**.**G**.**A****C****C**--........................AUCC-....................................................--**G****G****C**.A...**C**..**G****G**U**G**..**C****U**ACA**U****C****C****A****A**...**C****A****G****A****C**-....................AUUC...........-**G****U****C****U****G**.AG.**A**..**G****A**..**U****A****A****G****A****G****G****A****C**GGAA | |
|  |  | NZ\_AADW02000008.1/73933-74052  | CCAA**U****A****A****U****U****C****U****U**.**A****U****C**A.AGA**G**.**A****A****G****U**.**C****G****A****G****G****G**A...**A****C****G**.**G**.**C****C****C**GAA**G****A****C**.**A****C****U****U**.**C****A****G****C**AA**C****C**.-.**C****C****G****C**-........................GCGUA....................................................-**G****C****G****G**.A...**A**..**G****G**U**G**..**C****U**ACU**U****C****C****U****A**...**A****A****A****G****G****A**....................AUUU..........U**U****C****C****U****U****U**.GA.**A**..**G****A**..**U****A****A****G****A****G****C****A****G**ACAU | |
|  |  | NC\_002745.2/1844635-1844510  | ACGG**A****U****U****C****U****C****U****U**.**A****U****C**C.UGA**G**.**U****G****G****U**.**G****G****A****G****G****G**AC..**A****U****G**.**G**A**C****C****C**AAU**G****A****A**.**A****C****C**-.**C****A****G****C**AA**C****C**.**U**.-**C****U****U****U**U.......................UUUAU...................................................A**A****A****A****G****A**.A...**A**..**G****G**U**G**..**C****C**AAA-**C****C****G****U**UUG**C****A****G****A****C****A**....................AAUA...........**G****G****U****C****U****G**.AA.**C**..**G****A**..**U****A****A****G****A****G****C****G****A**AUGG | |
|  |  | NC\_002758.2/1922428-1922303  | ACGG**A****U****U****C****U****C****U****U**.**A****U****C**C.UGA**G**.**U****G****G****U**.**G****G****A****G****G****G**AC..**A****U****G**.**G**A**C****C****C**AAU**G****A****A**.**A****C****C**-.**C****A****G****C**AA**C****C**.**U**.-**C****U****U****U**U.......................UUUAU...................................................A**A****A****A****G****A**.A...**A**..**G****G**U**G**..**C****C**AAA-**C****C****G****U**UUG**C****A****G****A****C****A**....................AAUA...........**G****G****U****C****U****G**.AA.**C**..**G****A**..**U****A****A****G****A****G****C****G****A**AUGG | |
|  |  | NC\_002952.2/1959055-1958930  | ACGG**A****U****U****C****U****C****U****U**.**A****U****C**C.UGA**G**.**U****G****G****U**.**G****G****A****G****G****G**AC..**A****U****G**.**G**A**C****C****C**AAU**G****A****A**.**A****C****C**-.**C****A****G****C**AA**C****C**.**U**.-**C****U****U****U**U.......................UUUAU...................................................A**A****A****A****G****A**.A...**A**..**G****G**U**G**..**C****C**AAA-**C****C****G****U**UUG**C****A****G****A****C****A**....................AAUA...........**G****G****U****C****U****G**.AA.**C**..**G****A**..**U****A****A****G****A****G****C****G****A**AUGG | |
|  |  | NC\_002953.3/1860163-1860038  | ACGG**A****U****U****C****U****C****U****U**.**A****U****C**C.UGA**G**.**U****G****G****U**.**G****G****A****G****G****G**AC..**A****U****G**.**G**A**C****C****C**AAU**G****A****A**.**A****C****C**-.**C****A****G****C**AA**C****C**.**U**.-**C****U****U****U**U.......................UUUAU...................................................A**A****A****A****G****A**.A...**A**..**G****G**U**G**..**C****C**AAA-**C****C****G****U**UUG**C****A****G****A****C****A**....................AAUA...........**G****G****U****C****U****G**.AA.**C**..**G****A**..**U****A****A****G****A****G****C****G****A**AUGG | |
|  |  | NC\_003923.1/1880828-1880703  | ACGG**A****U****U****C****U****C****U****U**.**A****U****C**C.UGA**G**.**U****G****G****U**.**G****G****A****G****G****G**AC..**A****U****G**.**G**A**C****C****C**AAU**G****A****A**.**A****C****C**-.**C****A****G****C**AA**C****C**.**U**.-**C****U****U****U**U.......................UUUAU...................................................A**A****A****A****G****A**.A...**A**..**G****G**U**G**..**C****C**AAA-**C****C****G****U**UUG**C****A****G****A****C****A**....................AAUA...........**G****G****U****C****U****G**.AA.**C**..**G****A**..**U****A****A****G****A****G****C****G****A**AUGG | |
|  |  | NC\_002951.2/1892983-1892859  | ACGG**A****U****U****C****U****C****U****U**.**A****U****C**C.UGA**G**.**U****G****G****U**.**G****G****A****G****G****G**AC..**A****U****G**.**G**A**C****C****C**AAU**G****A****A**.**A****C****C**-.**C****A****G****C**AA**C****C**.**U**.-**C****U****U****U**U.......................UUAUA....................................................**A****A****A****G****A**.A...**A**..**G****G**U**G**..**C****C**AAA-**C****C****G****U**UUG**C****A****G****A****C****A**....................AAUA...........**G****G****U****C****U****G**.AA.**C**..**G****A**..**U****A****A****G****A****G****C****G****A**AUGG | |
|  |  | NZ\_AAIK01000012.1/37289-37166  | UUUC**A****A****G****C****U****A****U****C**.**A****U****C**C.AGA**A**.**A****G****G****U**.**G****G****A****G****G****G**A...**C****U****G**.**G**.**C****C****C**UGA**G****A****A**.**G****C****C****U**.**U****G****G****C**AA**C****C**.**G**.**U****C****A****U****U**........................GCUCU....................................................**A****A****U****G****A**.G...**C**..**G****G**U**G**..**C****C**AAU**U****C****C****A****U**...**C****C****C****G****G****C**A...................UAAA..........G**A****C****C****G****G****G**.AA.**U**..**G****A**..**U****G****A****U****G****G****U****A****U**GCAU | |
|  |  | NC\_006510.1/3025519-3025396  | UGUU**G****C****G****C****U****C****U****U**.**A****U****C**A.AGA**G**.**A****G****G****U**.**G****G****A****G****G****G**A...**U****G****U**.**G**.**C****C****C**AAU**G****A****A**.**G****C****C**-.**C****G****G****C**AA**C****C**.**G**.-**U****C****A****G**CG......................CAUGU...................................................G**C****U****G****A****A**.A...**U**..**G****G**U**G**..**C****C**AAU**U****C****A****C****A**...**C****A****A****A****G****C**....................GGCC..........U**G****C****U****U****U****G**.AG.**A**..**G****A**..**U****A****A****G****A****G****A****C****G**GAAU | |
|  |  | NC\_003210.1/882763-882874  | ACAU**A****G****U****A****A****C****U****U**.**A****U****C**A.AGA**A**.**A****G****G****U**.**G****G****A****G****G****G**UU..**C****U****G**.**G**.**C****C****C**CGU**G****A****A**.**G****C****C****U**.**U****G****G****C**AA**C****C**.**G**.**G****A**---........................UUUU-....................................................---**U****C**.A...**C**..**G****G**U**G**..**C****C**AAA**U****C****C****A****G**...**C****A****G****G**--....................UAAC...........--**A****C****U****G**.AC.**A**..**G****A**..**U****A****A****G****G****C****A****C****G**CGAA | |
|  |  | NZ\_AADQ01000002.1/79331-79220  | ACAU**A****G****U****A****A****C****U****U**.**A****U****C**A.AGA**A**.**A****G****G****U**.**G****G****A****G****G****G**UU..**C****U****G**.**G**.**C****C****C**CGU**G****A****A**.**G****C****C****U**.**U****G****G****C**AA**C****C**.**G**.**G****A**---........................UUUU-....................................................---**U****C**.A...**C**..**G****G**U**G**..**C****C**AAA**U****C****C****A****G**...**C****A****G****G**--....................UAAC...........--**A****C****U****G**.AC.**A**..**G****A**..**U****A****A****G****G****C****A****C****G**CGAA | |
|  |  | NC\_006510.1/2876477-2876349  | ACGG**A****U****A****C****U****C****U****U**.**A****U****C**C.CGA**G**C**C****G****G****U**.**G****G****A****G****G****G**A...**C****A****G**.**G**.**C****C****C**GAU**G****A****A**.**G****C****C**-.**C****A****G****C**AA**C****C**.**G**.-**U****C****A****C**AACUG...................UACAU..................................................GU**G****U****G****A****A**.A...**U**..**G****G**U**G**..**C****U**AA--**C****C****U****G**UGG**C****A****A****G****G****C**....................GCAG...........**U****C****C****U****U****G**.AA.**C**..**G****A**..**U****A****A****G****A****G****U****G****A**AAGG | |
|  |  | NC\_002976.3/1409744-1409621  | ACGG**A****U****U****C****U****C****U****U**.**A****U****C**C.UGA**G**.**U****G****G****U**.**G****G****A****G****G****G**AC..**A****U****G**.**G**A**C****C****C**AAU**G****A****A**.**A****C****C**-.**C****A****G****C**AA**C****C**.**U**.-**C****U****U****U**........................AUUU-....................................................**A****A****A****G****A**.A...**A**..**G****G**U**G**..**C****C**AAA-**C****C****G****U**UUG**C****A****G****A****C**-AA..................AUAU..........G-**G****U****C****U****G**.AA.**C**..**G****A**..**U****A****A****G****A****G****C****G****A**AUGG | |
|  |  | NC\_004461.1/1508629-1508506  | ACGG**A****U****U****C****U****C****U****U**.**A****U****C**C.UGA**G**.**U****G****G****U**.**G****G****A****G****G****G**AC..**A****U****G**.**G**A**C****C****C**AAU**G****A****A**.**A****C****C**-.**C****A****G****C**AA**C****C**.**U**.-**C****U****U****U**........................AUUU-....................................................**A****A****A****G****A**.A...**A**..**G****G**U**G**..**C****C**AAA-**C****C****G****U**UUG**C****A****G****A****C**-AA..................AUAU..........G-**G****U****C****U****G**.AA.**C**..**G****A**..**U****A****A****G****A****G****C****G****A**AUGG | |
|  |  | NC\_004193.1/2365520-2365396  | ACGG**A****U****A****C****U****C****U****U**.**A****U****U**C.AGA**G**U**U****G****G****U**.**G****G****A****G****G****G**A...**C****A****G**.**A**.**C****C****C**GAU**G****A****A**.**G****C****C**-.**C****A****G****C**AA**C****C**.-.**A****U****C****A****C**........................UACUG....................................................**G****U****G****A****A**.A...**A**..**G****G**U**G**..**C****U**AA--**U****C****U****G**AUG**C****A****A****G****G****A**U...................AAUA.........GU**U****C****C****U****U****G**.AA.**C**..**A****A**..**U****A****A****G****A****G****C****G****A**AAGG | |
|  |  | NC\_002939.4/2700111-2700227  | GUAG**A****C****C****U****U****C****U****U**.**A****U****C**A.AGA**G**.**U****G****G****U**.**G****G****A****G****G****G**A...**A****A****G**.**G**.**C****C****C**UGU**G****A****A**.**A****C****C****A**.**C****A****G****C**AA**C****C**.**G**.**G****U****C****C****G**........................GUAG-....................................................**C****G****G****A****C**.G...**C**CA**G****G**U**G**..**C****U**AAA**U****C****C****U****G**...**C****C****C**---....................GAAA...........---**G****G****G**.AG.**C**..**G****A**..**U****G****A****G****A****G****G****G****A**GCUU | |
|  |  | NC\_002745.2/15949-16061  | UUCA**U****A****U****U****U****C****U****U**.**A****U****U**G.UGA**G**.**A****A****G****U**.**U****G****A****G****G****G**AC..**U****U****G**.**G**.**C****C****C**UGU**G****A****U**.**A****C****U****U**.**C****A****G****C**AA**C****C**.**G**.**A****C****U**--........................UUAU-....................................................--**A****G****C**.A...**C**..**G****G**U**G**..**C****U**AAA**A****C****C****A****A**...**C****G****A****G**--....................-UUA...........--**C****U****C****G**.AA.**U**..**G****A**..**U****A****A****G****U****A****U****A****A**AGAC | |
|  |  | NC\_002758.2/15949-16061  | UUCA**U****A****U****U****U****C****U****U**.**A****U****U**G.UGA**G**.**A****A****G****U**.**U****G****A****G****G****G**AC..**U****U****G**.**G**.**C****C****C**UGU**G****A****U**.**A****C****U****U**.**C****A****G****C**AA**C****C**.**G**.**A****C****U**--........................UUAU-....................................................--**A****G****C**.A...**C**..**G****G**U**G**..**C****U**AAA**A****C****C****A****A**...**C****G****A****G**--....................-UUA...........--**C****U****C****G**.AA.**U**..**G****A**..**U****A****A****G****U****A****U****A****A**AGAC | |
|  |  | NC\_002951.2/15968-16080  | UUCA**U****A****U****U****U****C****U****U**.**A****U****U**G.UGA**G**.**A****A****G****U**.**U****G****A****G****G****G**AC..**U****U****G**.**G**.**C****C****C**UGU**G****A****U**.**A****C****U****U**.**C****A****G****C**AA**C****C**.**G**.**A****C****U**--........................UUAU-....................................................--**A****G****C**.A...**C**..**G****G**U**G**..**C****U**AAA**A****C****C****A****A**...**C****G****A****G**--....................-UUA...........--**C****U****C****G**.AA.**U**..**G****A**..**U****A****A****G****U****A****U****A****A**AGAC | |
|  |  | NC\_002952.2/15932-16044  | UUCA**U****A****U****U****U****C****U****U**.**A****U****U**G.UGA**G**.**A****A****G****U**.**U****G****A****G****G****G**AC..**U****U****G**.**G**.**C****C****C**UGU**G****A****U**.**A****C****U****U**.**C****A****G****C**AA**C****C**.**G**.**A****C****U**--........................UUAU-....................................................--**A****G****C**.A...**C**..**G****G**U**G**..**C****U**AAA**A****C****C****A****A**...**C****G****A****G**--....................-UUA...........--**C****U****C****G**.AA.**U**..**G****A**..**U****A****A****G****U****A****U****A****A**AGAC | |
|  |  | NC\_002953.3/15942-16054  | UUCA**U****A****U****U****U****C****U****U**.**A****U****U**G.UGA**G**.**A****A****G****U**.**U****G****A****G****G****G**AC..**U****U****G**.**G**.**C****C****C**UGU**G****A****U**.**A****C****U****U**.**C****A****G****C**AA**C****C**.**G**.**A****C****U**--........................UUAU-....................................................--**A****G****C**.A...**C**..**G****G**U**G**..**C****U**AAA**A****C****C****A****A**...**C****G****A****G**--....................-UUA...........--**C****U****C****G**.AA.**U**..**G****A**..**U****A****A****G****U****A****U****A****A**AGAC | |
|  |  | NC\_003923.1/15942-16054  | UUCA**U****A****U****U****U****C****U****U**.**A****U****U**G.UGA**G**.**A****A****G****U**.**U****G****A****G****G****G**AC..**U****U****G**.**G**.**C****C****C**UGU**G****A****U**.**A****C****U****U**.**C****A****G****C**AA**C****C**.**G**.**A****C****U**--........................UUAU-....................................................--**A****G****C**.A...**C**..**G****G**U**G**..**C****U**AAA**A****C****C****A****A**...**C****G****A****G**--....................-UUA...........--**C****U****C****G**.AA.**U**..**G****A**..**U****A****A****G****U****A****U****A****A**AGAC | |
|  |  | NC\_006177.1/278265-278396  | CCGU**U****G****A****C****U****C****U****U**.**A****U****C**C.AGA**G**U**A****G****G****C**.**U****G****A****G****G****G**A...**C****U****G**.**G**.**C****C****C**GAU**G****A****C**.**G****C****C****A**.**C****G****G****C**AA**C****C**.**G**.-**G****C****A****C**G.......................UCUCC..............................................CCAGGC**G****U****G****C****A**.G...**C**..**G****G**U**G**..**C****C**AAU**U****C****C****U****G**...**G****G****G****A****G****G**G...................CUCC.........GC**C****C****U****C****C****C**.GA.**A**..**G****A**..**U****G****A****G****A****G****G****C****A**GCCG | |
|  |  | NZ\_AAEB02000051.1/4037-3902  | GCGC**G****A****A****C****C****C****G****C**.**A****U****C**A.AGA**G**.**C****G****G****U**.**G****G****A****G****G****G**AA..**C****U****G**.**G**.**C****C****C**AGC**G****A****A**.**G****C****C****G**.**C****G****G****C**AA**C****C**.**G**.**G****C****G****G****G**GGGCC...................CUUAC...................................................C**C****C****C****G****U**.G...**A**..**G****G**U**G**..**C****C**AAU**U****C****C****A****G**...**C****A****G****G****U****C**UCCC................CGAG........GGG**G****G****C****C****U****G**.AA.**A**..**G****A**..**U****G****U****G****G****G****G****G****A**GAGC | |
|  |  | NC\_003997.3/4553691-4553556  | GGAU**A****C****U****C****U****C****U****U**.**A****U****C**C.CGA**G**C**U****G****G****C**.**G****G****A****G****G****G**A...**C****A****G**.**G**.**C****C****C**GAU**G****A****A**.**G****C****C**-.**C****A****G****C**AA**C****C**.**U**.--**C****A****C**UUGUAGUGG...............UAAAU................................................ACAG**G****U****G****A****A**.U...**A**..**G****G**U**G**..**C****U**AAA**A****C****C****U****G**UG.**C****G****A****G****G****C**....................UACA..........G**G****U****C****U****C****G**.AA.**C**..**G****A**..**U****A****A****G****A****G****C****G****A**AGGG | |
|  |  | NC\_004722.1/4695817-4695682  | GGAU**A****C****U****C****U****C****U****U**.**A****U****C**C.CGA**G**C**U****G****G****C**.**G****G****A****G****G****G**A...**C****A****G**.**G**.**C****C****C**GAU**G****A****A**.**G****C****C**-.**C****A****G****C**AA**C****C**.**U**.--**C****A****C**UUGUAGUGG...............UAAAU................................................ACAG**G****U****G****A****A**.U...**A**..**G****G**U**G**..**C****U**AAA**A****C****C****U****G**UG.**C****G****A****G****G****C**....................UACA..........G**G****U****C****U****C****G**.AA.**C**..**G****A**..**U****A****A****G****A****G****C****G****A**AGGG | |
|  |  | NC\_005945.1/4554911-4554776  | GGAU**A****C****U****C****U****C****U****U**.**A****U****C**C.CGA**G**C**U****G****G****C**.**G****G****A****G****G****G**A...**C****A****G**.**G**.**C****C****C**GAU**G****A****A**.**G****C****C**-.**C****A****G****C**AA**C****C**.**U**.--**C****A****C**UUGUAGUGG...............UAAAU................................................ACAG**G****U****G****A****A**.U...**A**..**G****G**U**G**..**C****U**AAA**A****C****C****U****G**UG.**C****G****A****G****G****C**....................UACA..........G**G****U****C****U****C****G**.AA.**C**..**G****A**..**U****A****A****G****A****G****C****G****A**AGGG | |
|  |  | NC\_006274.1/4612836-4612701  | GGAU**A****C****U****C****U****C****U****U**.**A****U****C**C.CGA**G**C**U****G****G****C**.**G****G****A****G****G****G**A...**C****A****G**.**G**.**C****C****C**GAU**G****A****A**.**G****C****C**-.**C****A****G****C**AA**C****C**.**U**.--**C****A****C**UUGUAGUGG...............UAAAU................................................ACAG**G****U****G****A****A**.U...**A**..**G****G**U**G**..**C****U**AAA**A****C****C****U****G**UG.**C****G****A****G****G****C**....................UACA..........G**G****U****C****U****C****G**.AA.**C**..**G****A**..**U****A****A****G****A****G****C****G****A**AGGG | |
|  |  | NC\_007530.2/4553818-4553683  | GGAU**A****C****U****C****U****C****U****U**.**A****U****C**C.CGA**G**C**U****G****G****C**.**G****G****A****G****G****G**A...**C****A****G**.**G**.**C****C****C**GAU**G****A****A**.**G****C****C**-.**C****A****G****C**AA**C****C**.**U**.--**C****A****C**UUGUAGUGG...............UAAAU................................................ACAG**G****U****G****A****A**.U...**A**..**G****G**U**G**..**C****U**AAA**A****C****C****U****G**UG.**C****G****A****G****G****C**....................UACA..........G**G****U****C****U****C****G**.AA.**C**..**G****A**..**U****A****A****G****A****G****C****G****A**AGGG | |
|  |  | NZ\_AAAC02000001.1/4997429-4997294  | GGAU**A****C****U****C****U****C****U****U**.**A****U****C**C.CGA**G**C**U****G****G****C**.**G****G****A****G****G****G**A...**C****A****G**.**G**.**C****C****C**GAU**G****A****A**.**G****C****C**-.**C****A****G****C**AA**C****C**.**U**.--**C****A****C**UUGUAGUGG...............UAAAU................................................ACAG**G****U****G****A****A**.U...**A**..**G****G**U**G**..**C****U**AAA**A****C****C****U****G**UG.**C****G****A****G****G****C**....................UACA..........G**G****U****C****U****C****G**.AA.**C**..**G****A**..**U****A****A****G****A****G****C****G****A**AGGG | |
|  |  | NZ\_AAEK01000003.1/182682-182547  | GGAU**A****C****U****C****U****C****U****U**.**A****U****C**C.CGA**G**C**U****G****G****C**.**G****G****A****G****G****G**A...**C****A****G**.**G**.**C****C****C**GAU**G****A****A**.**G****C****C**-.**C****A****G****C**AA**C****C**.**U**.--**C****A****C**UUGUAGUGG...............UAAAU................................................ACAG**G****U****G****A****A**.U...**A**..**G****G**U**G**..**C****U**AAA**A****C****C****U****G**UG.**C****G****A****G****G****C**....................UACA..........G**G****U****C****U****C****G**.AA.**C**..**G****A**..**U****A****A****G****A****G****C****G****A**AGGG | |
|  |  | NZ\_AAEN01000027.1/165370-165235  | GGAU**A****C****U****C****U****C****U****U**.**A****U****C**C.CGA**G**C**U****G****G****C**.**G****G****A****G****G****G**A...**C****A****G**.**G**.**C****C****C**GAU**G****A****A**.**G****C****C**-.**C****A****G****C**AA**C****C**.**U**.--**C****A****C**UUGUAGUGG...............UAAAU................................................ACAG**G****U****G****A****A**.U...**A**..**G****G**U**G**..**C****U**AAA**A****C****C****U****G**UG.**C****G****A****G****G****C**....................UACA..........G**G****U****C****U****C****G**.AA.**C**..**G****A**..**U****A****A****G****A****G****C****G****A**AGGG | |
|  |  | NZ\_AAEO01000031.1/165015-164880  | GGAU**A****C****U****C****U****C****U****U**.**A****U****C**C.CGA**G**C**U****G****G****C**.**G****G****A****G****G****G**A...**C****A****G**.**G**.**C****C****C**GAU**G****A****A**.**G****C****C**-.**C****A****G****C**AA**C****C**.**U**.--**C****A****C**UUGUAGUGG...............UAAAU................................................ACAG**G****U****G****A****A**.U...**A**..**G****G**U**G**..**C****U**AAA**A****C****C****U****G**UG.**C****G****A****G****G****C**....................UACA..........G**G****U****C****U****C****G**.AA.**C**..**G****A**..**U****A****A****G****A****G****C****G****A**AGGG | |
|  |  | NZ\_AAEP01000032.1/98186-98321  | GGAU**A****C****U****C****U****C****U****U**.**A****U****C**C.CGA**G**C**U****G****G****C**.**G****G****A****G****G****G**A...**C****A****G**.**G**.**C****C****C**GAU**G****A****A**.**G****C****C**-.**C****A****G****C**AA**C****C**.**U**.--**C****A****C**UUGUAGUGG...............UAAAU................................................ACAG**G****U****G****A****A**.U...**A**..**G****G**U**G**..**C****U**AAA**A****C****C****U****G**UG.**C****G****A****G****G****C**....................UACA..........G**G****U****C****U****C****G**.AA.**C**..**G****A**..**U****A****A****G****A****G****C****G****A**AGGG | |
|  |  | NZ\_AAEQ01000031.1/164836-164701  | GGAU**A****C****U****C****U****C****U****U**.**A****U****C**C.CGA**G**C**U****G****G****C**.**G****G****A****G****G****G**A...**C****A****G**.**G**.**C****C****C**GAU**G****A****A**.**G****C****C**-.**C****A****G****C**AA**C****C**.**U**.--**C****A****C**UUGUAGUGG...............UAAAU................................................ACAG**G****U****G****A****A**.U...**A**..**G****G**U**G**..**C****U**AAA**A****C****C****U****G**UG.**C****G****A****G****G****C**....................UACA..........G**G****U****C****U****C****G**.AA.**C**..**G****A**..**U****A****A****G****A****G****C****G****A**AGGG | |
|  |  | NZ\_AAER01000035.1/1067862-1067727  | GGAU**A****C****U****C****U****C****U****U**.**A****U****C**C.CGA**G**C**U****G****G****C**.**G****G****A****G****G****G**A...**C****A****G**.**G**.**C****C****C**GAU**G****A****A**.**G****C****C**-.**C****A****G****C**AA**C****C**.**U**.--**C****A****C**UUGUAGUGG...............UAAAU................................................ACAG**G****U****G****A****A**.U...**A**..**G****G**U**G**..**C****U**AAA**A****C****C****U****G**UG.**C****G****A****G****G****C**....................UACA..........G**G****U****C****U****C****G**.AA.**C**..**G****A**..**U****A****A****G****A****G****C****G****A**AGGG | |
|  |  | NZ\_AAES01000022.1/98965-99100  | GGAU**A****C****U****C****U****C****U****U**.**A****U****C**C.CGA**G**C**U****G****G****C**.**G****G****A****G****G****G**A...**C****A****G**.**G**.**C****C****C**GAU**G****A****A**.**G****C****C**-.**C****A****G****C**AA**C****C**.**U**.--**C****A****C**UUGUAGUGG...............UAAAU................................................ACAG**G****U****G****A****A**.U...**A**..**G****G**U**G**..**C****U**AAA**A****C****C****U****G**UG.**C****G****A****G****G****C**....................UACA..........G**G****U****C****U****C****G**.AA.**C**..**G****A**..**U****A****A****G****A****G****C****G****A**AGGG | |
|  |  | NC\_003909.8/4541309-4541174  | GGAU**A****C****U****C****U****C****U****U**.**A****U****C**C.CGA**G**C**U****G****G****C**.**G****G****A****G****G****G**A...**C****A****G**.**G**.**C****C****C**GAU**G****A****A**.**G****C****C**-.**C****A****G****C**AA**C****C**.-.**U****C****A****C****U**UGUAUUG.................GUAAA................................................CACA**A****G****U****G****A**.A..U**A**..**G****G**U**G**..**C****U**AAA**A****C****C****U****G**UG.**C****G****A****G****G****C**....................UACA..........G**G****U****C****U****C****G**.AA.**C**..**G****A**..**U****A****A****G****A****G****C****G****A**AGGG | |
|  |  | NC\_005957.1/4553311-4553176  | GGAU**A****C****U****C****U****C****U****U**.**A****U****C**C.CGA**G**C**U****G****G****C**.**G****G****A****G****G****G**A...**C****A****G**.**G**.**C****C****C**GAU**G****A****A**.**G****C****C**-.**C****A****G****C**AA**C****C**.-.**U****C****A****C****U**UGUAUUG.................GUAAA................................................CACA**A****G****U****G****A**.A..U**A**..**G****G**U**G**..**C****U**AAA**A****C****C****U****G**UG.**C****G****A****G****G****C**....................UACA..........G**G****U****C****U****C****G**.AA.**C**..**G****A**..**U****A****A****G****A****G****C****G****A**AGGG | |
|  |  | NC\_006270.2/1486652-1486495  | UUGA**U****U****U****C****U****C****U****U**.**A****U****C**G.AGA**G**U**U****G****G****G**.**U****G****A****A****G****G**A...**C****U****G**.**G**.**C****C****U**AAU**G****A****U**.**C****C****C****A**A**C****A****G****C**AA**C****C**.**G**.**A****C****C****G****U**AAUACC..................AUUGU........................GAAAUGGGGCGCGAAUCUCUGCGCCGCU**A****G****G****G****C**.A...**C**..**G****G**U**G**..**C****U**AAU**U****C****C****A****U**...**C****A****G****A****C****U**....................UUGA...........**A****U****U****C****U****G**.AG.**A**..**G****A**..**U****A****A****G****A****G****A****G****G**CGUA | |
|  |  | NC\_006322.1/1487513-1487356  | UUGA**U****U****U****C****U****C****U****U**.**A****U****C**G.AGA**G**U**U****G****G****G**.**U****G****A****A****G****G**A...**C****U****G**.**G**.**C****C****U**AAU**G****A****U**.**C****C****C****A**A**C****A****G****C**AA**C****C**.**G**.**A****C****C****G****U**AAUACC..................AUUGU........................GAAAUGGGGCGCGAAUCUCUGCGCCGCU**A****G****G****G****C**.A...**C**..**G****G**U**G**..**C****U**AAU**U****C****C****A****U**...**C****A****G****A****C****U**....................UUGA...........**A****U****U****C****U****G**.AG.**A**..**G****A**..**U****A****A****G****A****G****A****G****G**CGUA | |
|  |  | NZ\_AAIB01000001.1/59869-59747  | UUUU**A****C****G****C****U****G****U****C**.**A****U****C**G.AGA**A**.**A****G****G****U**.**G****G****A****G****G****G**A...**C****U****G**.**G**.**C****C****C**UGA**G****A****A**.**A****C****C****U**.**U****G****G****C**AA**C****C**.**G**.**U****C****A****U**-........................UGCAA....................................................**U****A****U****G****A**.U...**U**..**G****G**U**G**..**C****C**AAU**U****C****C****A****U**...**C****C****C****G****G****A**U...................UACU..........G**G****C****C****G****G****G**.AG.**A**..**G****A**..**U****G****A****U****G****G****U****A****U**GCAU | |
|  |  | NC\_002976.3/1935508-1935398  | AUUC**A****A****U****A****A****C****U****U**.**A****U****C**A.AGA**G**.**A****A****G****U**.**G****G****A****G****G****G**A...**C****U****G**.**G**.**C****C****C**AAA**G****A****A**.**G****C****U****U**.**C****G****G****C**AA**C****A**.**U**.**U**----........................GUAUC....................................................-----.-...**A**..**U****G**U**G**..**C****C**AAU**U****C****C****A****G**...**U****A****A****C****C****G**....................-AGA...........**A****G****G****U****U****A**.GA.**A**..**G****A**..**U****A****A****G****G****U****U****A****A**ACAC | |
|  |  | NC\_004461.1/1940505-1940395  | AUUC**A****A****U****A****A****C****U****U**.**A****U****C**A.AGA**G**.**A****A****G****U**.**G****G****A****G****G****G**A...**C****U****G**.**G**.**C****C****C**AAA**G****A****A**.**G****C****U****U**.**C****G****G****C**AA**C****A**.**U**.**U**----........................GUAUC....................................................-----.-...**A**..**U****G**U**G**..**C****C**AAU**U****C****C****A****G**...**U****A****A****C****C****G**....................-AGA...........**A****G****G****U****U****A**.GA.**A**..**G****A**..**U****A****A****G****G****U****U****A****A**ACAC | |
|  |  | NC\_006270.2/3080583-3080432  | GCGG**A****U****A****C****U****C****U****U**.**A****U****C**C.CGA**G**C**U****G****G****U**.**G****G****A****G****G****G**A...**C****A****G**.**G**.**C****C****C**AAU**G****A****A**.**A****C****C**-.**C****A****G****C**AA**C****C**.**G**.**G****U****U****U****C**UCUUA...................UUAAU...............................GGAAAAAAACAGUUUCUGAGA**C****A****A****C****U**.A...**C**..**G****G**U**G**..**C****U**AA--**C****C****U****G**AUG**C****A****A****G****G****U**GU..................UCAA..........U**A****C****C****U****U****G**.AG.**C**..**G****A**..**U****A****A****G****A****G****U****G****A**AAGG | |
|  |  | NC\_006322.1/3080760-3080609  | GCGG**A****U****A****C****U****C****U****U**.**A****U****C**C.CGA**G**C**U****G****G****U**.**G****G****A****G****G****G**A...**C****A****G**.**G**.**C****C****C**AAU**G****A****A**.**A****C****C**-.**C****A****G****C**AA**C****C**.**G**.**G****U****U****U****C**UCUUA...................UUAAU...............................GGAAAAAAACAGUUUCUGAGA**C****A****A****C****U**.A...**C**..**G****G**U**G**..**C****U**AA--**C****C****U****G**AUG**C****A****A****G****G****U**GU..................UCAA..........U**A****C****C****U****U****G**.AG.**C**..**G****A**..**U****A****A****G****A****G****U****G****A**AAGG | |
|  |  | NC\_002932.3/606182-606302  | UUUC**G****A****G****C****U****A****U****C**.**A****U****C**C.AGA**A**.**A****G****G****C**.**G****G****A****G****G****G**A...**C****U****G**.**G**.**C****C****C**UGC**G****A****A**.**G****C****C****U**.**U****G****G****C**AA**C****C**.**U**.**U****C****A****U**-........................UCCAC....................................................-**A****U****G****A**.G...**C**..**G****G**U**G**..**C****C**AAA**U****C****C****A****U**...**C****C****C****G****G****A**....................GGAA..........A**U****C****C****G****G****G**.AA.**A**..**G****A**..**U****G****A****U****G****U****A****U****G**CAUU | |
|  |  | NZ\_AADW02000002.1/57116-57233  | GCAU**A****U****C****U****A****C****U****U**.**A****U****C**G.AGA**G**.**C****G****A****C**.**C****G****A****G****G****G**AU..**U****A****G**.**G**.**C****C****C**AAC**G****A****C**.**G****U****C**-.**C****G****G****C**AA**C****C**.-.**A****C****C**--........................UUUAA....................................................--**G****G****A**.A...**C**..**G****G**U**G**..**C****C**ACA**U****C****C****U****A**...**C****A****G****A****A****U**....................GUUU..........C**A****U****U****C****U****G**.AG.**A**..**G****A**..**U****A****A****G****U****U****A****C****G**AAAU | |
|  |  | NC\_006510.1/972366-972184  | AUCG**G****U****A****C****U****C****U****U**.**A****U****C**A.AGA**G**U**U****G****G****C**.**U****G****A****G****G****G**AA..**U****U****G**.**G**.**C****C****C**AAU**G****A****A**.**G****C****C**-.**C****A****G****C**AA**C****C**.**G**.**A****C****C****G****U**AA......................UACUAUCGUGAGAUAGGGCGCACGCCAAGGGCGGCGCCGGAAGCGUCAUGCUUCCGC**A****G****G****G****C**.A...**C**..**G****G**U**G**..**C****U**AAG**U****C****C****A****A**...**C****A****G****A****A****A**GACCG...............AUGU..........C**U****U****U****C****U****G**.AA.**A**..**G****A**..**U****A****A****G****A****G****G****C****G**CGAA | |
|  |  | NZ\_AAIC01000627.1/953-830  | AACA**A****U****U****C****A****U****U****U**.**A****U****C**A.AGA**A**.**A****G****G****U**.**G****G****A****G****G****G**U...**A****A****G**.**G**.**C****C****C**GUU**G****A****A**.**A****C****C****U**.**U****A****G****C**AA**C****C**.-.**C****U****G****A****U**GG......................UAAUA...................................................C**A****U****C****A****G**.U...**A**..**G****G**U**G**..**C****U**AAU**U****C****C****U****G**...**C****U****U****C****A****U**....................UUUC...........**A****U****G****A****A****G**.AA.**A**..**G****A**..**U****A****A****A****U****C****A****C****A**CUGC | |
|  |  | NC\_002973.5/882830-882941  | ACAU**A****G****C****A****A****C****U****U**.**A****U****C**A.AGA**A**.**A****G****G****U**.**G****G****A****G****G****G**UU..**C****U****G**.**G**.**C****C****C**CGU**G****A****A**.**G****C****C****U**.**U****G****G****C**AA**C****C**.**G**.**G****A**---........................AGUU-....................................................---**U****C**.A...**C**..**G****G**U**G**..**C****C**AAA**U****C****C****A****G**...**C****A****G****G**--....................UAAC...........--**A****C****U****G**.AC.**A**..**G****A**..**U****A****A****G****G****C****A****C****G**CGAA | |
|  |  | NZ\_AADR01000005.1/26707-26818  | ACAU**A****G****C****A****A****C****U****U**.**A****U****C**A.AGA**A**.**A****G****G****U**.**G****G****A****G****G****G**UU..**C****U****G**.**G**.**C****C****C**CGU**G****A****A**.**G****C****C****U**.**U****G****G****C**AA**C****C**.**G**.**G****A**---........................AGUU-....................................................---**U****C**.A...**C**..**G****G**U**G**..**C****C**AAA**U****C****C****A****G**...**C****A****G****G**--....................UAAC...........--**A****C****U****G**.AC.**A**..**G****A**..**U****A****A****G****G****C****A****C****G**CGAA | |
|  |  | NZ\_AAHJ01000010.1/33494-33373  | UUUA**A****C****G****C****U****A****U****C**.**A****U****C**G.AGA**A**.**A****G****G****U**.**G****G****A****G****G****G**A...**C****U****G**.**G**.**C****C****C**UGA**G****A****A**.**G****C****C****U**.**U****G****G****C**AA**C****C**.**G**.**U****C****A****C**-........................GCACA....................................................-**G****U****G****A**.U...**U**..**G****G**U**G**..**C****C**AAU**U****C****C****A****U**...**C****C****C****G****G****A**C...................UGCG..........A**G****C****C****G****G****G**.AG.**A**..**G****A**..**U****G****A****U****G****G****U****A****U**GUCU | |
|  |  | NC\_000964.2/3128389-3128237  | GCGG**A****U****A****C****U****C****U****U**.**A****U****C**C.CGA**G**C**U****G****G****C**.**G****G****A****G****G****G**A...**C****A****G**.**G**.**C****C****C**UAU**G****A****A**.**G****C****C**-.**C****A****G****C**AA**C****C**.-.**G****G****U****U****U**CUCUGUUA................UUUAU.................................UAUGUUCAACUGAGUGAGA**C****A****A****C****C**.A...**A**..**G****G**U**G**..**C****U**AA--**C****C****U****G**UUG**C****A****A****G****G****U**U...................GUAU........GAU**U****C****C****U****U****G**.AG.**C**..**G****A**..**U****A****A****G****A****G****U****G****A**AAGG | |
|  |  | NZ\_AADW02000005.1/169223-169108  | CAUC**C****A****A****U****U****C****U****U**.**A****U****C**A.AGA**G**C**A****G****A****C**.**G****G****A****G****G****G**A...**C****G****A**.**G**.**C****C****C**UAC**G****A****U**.**G****U****C****G**.**C****A****G****C**AA**C****C**.**G**.**A****C****C**--........................ACUU-....................................................--**G****G****C**.A...**C**..**G****G**U**G**..**C****U**AAU**U****C****U****U****G**...**C****A****G****C****U**-....................AUUA...........-**C****G****C****U****G**.AC.**A**..**G****A**..**U****A****A****G****A****G****U****C****C**ACGC | |
|  |  | NC\_000964.2/1385204-1385035  | ACAU**U****U****U****C****U****C****U****U**.**A****U****C**G.AGA**G**U**U****G****G****G**.**C****G****A****G****G****G**A...**U****U****G**.**G**.**C****C****U**UUU**G****A****C**.**C****C****C****A**A**C****A****G****C**AA**C****C**.**G**.**A****C****C****G****U**AAUACC..................AUUGU.........GAAAUGGGGCGCACUGCUUUUCGCGCCGAGACUGAUGUCUCAU**A****A****G****G****C**.A...**C**..**G****G**U**G**..**C****U**AAU**U****C****C****A****U**...**C****A****G****A****U**-....................-UGU...........-**G****U****C****U****G**.AG.**A**..**G****A**..**U****G****A****G****A****G****A****G****G**CAGU | |
|  |  | NC\_006177.1/1830599-1830728  | CGGC**G****G****G****C****U****C****U****U**.**A****U****C**C.AGA**G**.**A****G****G****U**.**G****G****A****G****G****G**AA..**C****U****G**.**G**.**C****C****C**GAU**G****A****A**.**A****C****C**-.**C****G****G****C**AA**C****C**.**C**.**A****G****G****A****C**G.......................CUGC-...............................................GGGGC**G****U****C****C****G**.A...**A**..**G****G**U**G**..**C****C**AAC**U****C****C****A****C**...**C****G****G****C****G****G**C...................CUCA.........CG**C****C****G****C****C****G**.GA.**A**..**G****A**..**U****A****A****G****A****G****A****G****C**GGAC | |
|  |  | NZ\_AAIJ01000018.1/24135-24016  | UUUU**A****A****G****C****U****G****U****C**.**A****U****C**C.AGA**A**.**A****G****G****U**.**G****G****A****G****G****G**A...**C****U****G**.**G**.**C****C****C**UGA**G****A****A**.**G****C****C****U**.**U****G****G****C**AA**C****C**.**G**.**U****C****A**--........................UUCG-....................................................--**U****G****A**.U...**U**..**G****G**U**G**..**C****C**AAU**U****C****C****A****A**...**C****C****C****G****G****A**G...................AGCU..........G**U****C****C****G****G****G**AAA.**A**..**G****A**..**U****G****A****U****G****G****U****A****U**GUGC | |
|  |  | NC\_002976.3/2600726-2600612  | UUAC**C****U****A****A****C****C****U****U**.**A****U****U**U.UGA**G**.**A****A****G****C**.**A****G****A****G****G****G**AU..**U****U****G**.**G**.**C****C****C**GUA**G****A****A**.**G****C****U****U**.**C****A****G****C**AA**C****C**.**G**.**A****C****U**--........................UUAAA....................................................-**U****A****G****C**.A...**C**..**G****G**U**G**..**C****U**AAU**A****C****C****A****A**...**C****G****A****G**--....................-CAA...........--**C****U****C****G**.AA.**U**..**G****A**..**U****A****A****G****U****A****C****G****A**UAAG | |
|  |  | NC\_004461.1/15964-16078  | UUAC**C****U****A****A****C****C****U****U**.**A****U****U**U.UGA**G**.**A****A****G****C**.**A****G****A****G****G****G**AU..**U****U****G**.**G**.**C****C****C**GUA**G****A****A**.**G****C****U****U**.**C****A****G****C**AA**C****C**.**G**.**A****C****U**--........................UUAAA....................................................-**U****A****G****C**.A...**C**..**G****G**U**G**..**C****U**AAU**A****C****C****A****A**...**C****G****A****G**--....................-CAA...........--**C****U****C****G**.AA.**U**..**G****A**..**U****A****A****G****U****A****C****G****A**UAAG | |
|  |  | NC\_003212.1/172391-172507  | UUAC**A****A****U****U****U****C****U****U**.**A****U****C**C.AGA**G**.**U****G****G****U**.**G****G****A****G****G****G**AA..**U****C****G**.**G**.**C****C****C**AGU**G****A****A**.**A****C****C**-.**C****G****G****C**AG**C****G**.**G**.**A****G****C**--........................GCAA-....................................................--**G****U****U**.-...**C**..**U****A**U**G**..**C****U**AAU**U****C****C****G****A**U..**C****A****G****A****A****G**....................UAAU...........**A****U****U****C****U****G**.GC.**A**..**G****A**..**U****A****A****G****U****A****G****U****A**GCUU | |
|  |  | NC\_003210.1/137126-137242  | UUAC**G****U****U****U****U****C****U****U**.**A****U****C**A.AGA**G**.**U****G****G****U**.**G****G****A****G****G****G**AA..**U****C****G**.**G**.**C****C****C**AGU**G****A****A**.**A****C****C**-.**C****A****G****C**AG**C****G**.**G**.**A****G****C**--........................GCAA-....................................................--**G****U****U**.-...**C**..**U****A**U**G**..**C****U**AAU**U****C****C****G****A**U..**C****A****G****A****A****G**....................UAAU...........**A****U****U****C****U****G**.GC.**A**..**G****A**..**U****A****A****G****U****A****G****U****A**GCUU | |
|  |  | NZ\_AAIC01000009.1/45881-46000  | UUUC**A****A****G****C****U****A****U****C**.**A****U****C**C.AGA**A**.**A****G****G****U**.**G****G****A****G****G****G**A...**G****U****G**.**G**.**C****C****C**UGA**G****A****A**.**G****C****C****U**.**U****G****G****C**AA**C****C**.**G**.**U****C****A**--........................GUAG-....................................................--**U****G****A**.U...**U**..**G****G**U**G**..**C****C**AAU**U****C****C****A****A**...**C****C****C****G****G****A**CA..................AGCA..........G**U****G****C****G****G****G**.GA.**A**..**G****A**..**U****G****A****U****U****G****U****A****U**AUGC | |
|  |  | NC\_002973.5/148864-148980  | UUAC**G****U****U****U****U****C****U****U**.**A****U****C**A.AGA**G**.**C****G****G****U**.**G****G****A****G****G****G**AA..**U****C****G**.**G**.**C****C****C**AGU**G****A****A**.**G****C****C**-.**C****A****G****C**AG**C****G**.**G**.**A****G****C**--........................GCAA-....................................................--**G****U****U**.-...**C**..**U****A**U**G**..**C****U**AAU**U****C****C****G****A**U..**C****A****G****A****A****G**....................UAAU...........**A****U****U****C****U****G**.GC.**A**..**G****A**..**U****A****A****G****U****A****G****U****A**GCUU | |
|  |  | NC\_006510.1/1382529-1382647  | AGCA**U****A****C****G****U****C****U****U**.**A****U****C**A.AGA**G**.**U****G****G****G**.**C****G****A****G****A****G**AA..**C****G****G**.**G**.**C****U****U**GAU**G****A****C**.**C****C****C****A**.**C****A****G****C**AA**C****C**.**U**.**G****C****C**--........................-GCA-....................................................--**G****G****C**.A...**A**..**G****G**U**G**..**C****U**AAC**A****C****C****C****G**...**C****A****A****A****G****C**G...................GUUU..........C**G****C****U****U****U****G**.GA.**U**..**G****A**..**U****A****A****G****A****A****C****G****G**CUCA | |
|  |  | NC\_005125.1/2730833-2730707  | GUCU**C****U****U****G****A****C****U****U**.**A****U****C**C.AGA**G**C**A****G****G****C**.**G****U****A****G****G****G**AA..**C****A****G**.**G**.**C****C****C**GGU**G****A****C**.**G****C****C****A**.**C****G****G****C**AA**C****C**.**G**.--**C****C****C**AC......................UAGCA..................................................AC**G****G****G****A****A**.A...**A**..**G****G**U**G**..**C****C**AAU**U****C****C****U****G**...**C****G****G****U****U****C**C...................-UCA..........C**G****A****A****C****C****G**.GA.**A**..**G****A**..**U****A****A****G****U****C****A****G****G**CAUC | |
|  |  | NZ\_AADW02000019.1/7529-7669  | ACGG**A****U****A****C****U****C****U****U**.**A****U****U**C.UGA**G**C**A****G****G****U**.**G****G****A****G****G****G**AAC.**A****A****G**.**G**.**C****C****C**GAA**G****A****A**.**A****C****C**-.**C****G****G****C**AA**C****C**.**G**.-**U****C****U****U**AUA.....................UUAAU......................................UUCAUCAUUAAUGU**A****G****G****A****A**.A...**A**..**G****G**U**G**..**C****U**--C**U****C****C****U****G**AAG**C****G****A****A****G****U**....................AAAC...........**A****C****U****U****C****G**.AA.**C**..**G****A**..**U****A****A****G****A****G****G****G****U**AAAG | |
|  |  | NC\_003888.3/4708429-4708590  | UUCA**U****A****C****C****G****C****U****C**.**A****U****C**C.AGA**G**.**G****G****G****C**.**A****G****A****G****G****G**AU..**A****C****G**.**G**.**C****C****C**GAU**G****A****A**.**G****C****C****C**.**C****G****G****C**AA**C****C**.**C**.**U****C****C**-**A**GUCGG...................UUCUU........................GUCACACGGACGUGGCGAGGCUCCCGGC**U****A****G****G****G**.A...**A**..**G****G**U**G**..**C****C**AAA**U****C****C****G****U**...**C****U****C****A****C****G**GCG.................AGAU.......GCGU**C****G****U****G****A****G**.GA.**A**..**G****A**..**U****G****A****G****G****A****G****A****A**AGGG | |
|  |  | NZ\_AAAQ02000005.1/25541-25695  | UUGA**G****A****U****U****G****C****U****C**.**A****U****C**C.AGA**G**.**G****G****G****U**.**G****G****A****G****G****G**AC..**A****C****G**.**G**.**C****C****C**UGU**G****A****A**.**G****C****C****C**.**C****G****G****C**AA**C****C**.**A**.**U****C****C****C****G**GUGGACG.................UUGCU..................................CGUCAGGUGCGAGGCGGC**C****G****G****G****A**.C...**A**..**G****G**U**G**..**C****C**AAC**U****C****C****G****U**...**C****C****C****A****C****U**G...................UCAA.....GGUGGC**A****G****U****G****G****G**.GA.**A**..**G****A**..**U****G****A****G****G****G****G****A****G**AACG | |
|  |  | NC\_006177.1/2688761-2688642  | CCCG**C****C****C****G****G****U****U****C**.**A****U****C**G.AGA**G**.**U****G****G****C**.**G****G****A****G****G****G**A...**C****U****G**.**G**.**C****C****C**CAU**G****A****U**.**G****C****C****A**.**C****G****G****C**AA**C****C**.**U**.-**C****U****C****C**........................CGCGG....................................................**G****G****A****G****A**.A...**C**..**G****G**U**G**..**C****C**AAA**U****C****C****A****G**...**C****G****G****A****C**-....................ACUC..........G-**G****U****C****C****G**.AG.**A**..**G****A**..**U****G****A****A****G****C****G****U****G**CGCA | |
|  |  | NC\_003454.1/1317660-1317560  | AAAU**A****A****A****U****A****A****C****C**.**A****U****C**C.AGA**G**.**A****A****A****C**.**G****G****A****G****G****G**A...**C****U****G**.**G**.**C****C****C**AAU**G****A****U**.**G****U****U****U**.**C****A****G****C**AA**C****C**.**U**.**A****C****U**--........................UAAA-....................................................--**U****G****U**.G...**U**..**G****G**U**G**..**C****U**AAU**U****C****C****A****G**...------....................----...........------.AG.**A**..**G****A**..**U****G****G****A****G****A****G****G****A**AAAU | |
|  |  | NC\_000964.2/1423998-1423828  | AUAU**A****U****U****C****U****C****U****U**.**A****U****C**G.AGA**G**U**U****G****G****G**.**C****G****A****G****G****G**AU..**U****U****G**.**G**.**C****C****U**UUU**G****A****C**.**C****C****C****A**A**A****A****G****C**AA**C****C**.**G**.**A****C****C****G****U**AAUUCCAUUGUGAAAUGGGGCGCAUUUUU............................UUCGCGCCGAGACGCUGGUCUCUU**A****A****G****G****C**.A...**C**..**G****G**U**G**..**C****U**AAU**U****C****C****A****U**U..**C****A****G****A****U**-....................-CUG...........-**A****U****C****U****G**.AG.**A**..**G****A**..**U****A****A****G****A****G****A****G****G**CGGA | |
|  |  | NZ\_AAEB02000044.1/15380-15505  | UUCC**G****G****G****C****G****C****U****C**.**A****U****C**G.AGA**G**.**C****G****G****U**.**G****G****A****G****G****G**A...**C****G****G**.**G**.**C****C****C**UGC**G****A****A**.**G****C****C****G**.**C****G****G****C**AA**C****C**.**G**.**G****C****G****G****G**CGGCG...................GACGC...................................................C**C****G****C****G****C**.C...**A**..**G****G**U**G**..**C****C**AAU**U****C****C****C****G**...**C****G****G****A****G**-....................GAGA...........-**C****U****C****C****G**.AG.**A**..**G****A**..**U****G****A****G****C****C****G****G****C**AGCC | |
|  |  | NC\_003155.3/4858633-4858473  | CGAA**U****A****C****C****G****C****U****C**.**A****U****C**C.AGA**G**.**G****G****G****C**.**A****G****A****G****G****G**AU..**A****C****G**.**G**.**C****C****C**GAU**G****A****A**.**G****C****C****C**.**C****G****G****C**AA**C****C**.**C**.**U****C****C****A****G**CCGGUCUUGUCACG..........UUGAU...................................GUGGCGAGGCUCCCGGC**U****C****G****G****G**.A...**A**..**G****G**U**G**..**C****C**AAA**U****C****C****G****U**...**C****U****C****A****C****G**G...................CGAA.....GUGCGU**C****G****U****G****A****G**.GA.**A**..**G****A**..**U****G****A****G****G****A****G****A****A**AGGG | |
|  |  | NZ\_AAAC02000001.1/716908-716780  | NNNN**N****N****N****N****N****N****N****N**.**N****N****N**N.NNA**G**.**U****G****G****C**.**G****G****A****G****G****G**A...**C****U****G**.**G**.**C****C****C**UCU**G****A****U**.**G****C****C**-.**C****G****G****C**AA**C****C**.**G**.**A****G****C****U****U**AUGAC...................GUAU-....................................................**A****A****G****C****U**.A...**A**..**G****G**U**G**..**C****U**AAU**U****C****C****U****G**...**C****A****A****A****A****U**GAG.................UUUU..........C**G****U****U****U****U****G**.GA.**A**..**G****A**..**U****A****A****G****A****G****A****G****G**AUCC | |
|  |  | NZ\_AABF02000063.1/3018-3118  | AAAU**A****A****A****U****A****A****C****C**.**A****U****C**C.AGA**G**.**A****A****A****C**.**U****G****A****G****G****G**A...**C****U****G**.**G**.**C****C****C**UAU**G****A****U**.**G****U****U****U**.**C****A****G****C**AA**C****C**.**U**.**A****C****U**--........................UAAA-....................................................--**U****G****U**.G...**U**..**G****G**U**G**..**C****U**AAU**U****C****C****A****G**...------....................----...........------.AG.**A**..**G****A**..**U****G****G****A****A****A****G****G****U**CAAU | |
|  |  | NZ\_AAEW01000066.1/3421-3300  | GACA**A****C****C****G****U****G****U****U**.**A****U****C**A.CGA**G**.**U****G****G****U**.**G****G****A****G****G****G**A...**A****U****G**.**G**.**C****C****C**UUU**G****A****A**.**A****C****C****A**.**C****A****G****C**AA**C****C**.**G**.**G****U****C****C****U**GACG....................UUCA-..................................................GG**A****U****G****A****C**.-...**A**..**G****G**U**G**..**C****U**AAU**U****C****C****A****C**...**C****C****C****C**--....................GCAA...........--**G****G****G****G**.AC.**A**..**G****A**..**U****G****A****G****A****C****G****G****C**GUCU | |
|  |  | NZ\_AADT03000039.1/9873-9756  | AUCC**C****A****C****G****G****C****U****U**.**A****U****C**G.CGA**G**.**A****G****G****U**.**G****G****A****G****G****G**-...**A****G****G**.**G**C**C****C****C**GAU**G****A****A**.**A****C****C**-.**C****G****G****C**AA**C****C**.-.**C****C****G****C****U**........................AUUUA....................................................**G****C****C****G****G**.A...**A**..**G****G**U**G**..**C****C**AAG**U****G****C****C****G**...**C****A****G****G****A**-....................AACC...........-**U****C****C****U****G**.GA.**A**..**G****A**..**U****A****A****G****U****U****G****A****C**UCUU | |
|  |  | NC\_003454.1/987493-987392  | UGGA**A****A****U****A****A****A****C****C**.**A****U****C**A.AGA**G**.**A****G****A****U**.**U****G****A****G****G****G**A...**C****A****G**.**G**.**C****C****C**GUU**G****A****G**.**A****U****C****U**.**C****A****G****C**AA**C****C**.**U**.**A****C****G**--........................UAAAA....................................................--**U****G****U**.G...**U**..**G****G**U**G**..**C****U**AAU**U****C****C****U****G**...------....................----...........------.AU.**A**..**G****A**..**U****G****G****A****A****A****A****G****A**UUAU | |
|  |  | NC\_005835.1/397567-397417  | GCCC**G****U****U****C****U****C****U****U**.**A****U****C**C.AGA**G**.**C****G****G****U**.**G****G****A****G****G****G**U...**A****C****G**.**G**.**C****C****C**UGU**G****A****A**.**G****C****C****G**.**C****G****G****C**AA**C****C**.**U**.-**C****C****C****G**CCCCUUCCG...............UUCCA......................................UGGCCGGAUGCGGG**C****G****G****G****G**.C...**U**..**G****G**U**G**..**C****C**AAC**G****C****C****G****G**...**C****C****C****G****G****G**CGGGG...............GAAA.........CG**C****C****C****G****G****G**.GA.**C**..**G****A**..**U****A****A****G****A****G****A****G****G**GGGG | |
|  |  | NC\_006461.1/728132-727982  | GCCC**G****U****U****C****U****C****U****U**.**A****U****C**C.AGA**G**.**C****G****G****U**.**G****G****A****G****G****G**U...**A****C****G**.**G**.**C****C****C**UGU**G****A****A**.**G****C****C****G**.**C****G****G****C**AA**C****C**.**U**.-**C****C****C****G**CCCCUUCCG...............UUCCA......................................UGGCCGGAUGCGGG**C****G****G****G****G**.C...**U**..**G****G**U**G**..**C****C**AAC**G****C****C****G****G**...**C****C****C****G****G****G**CGGGG...............GAAA.........CG**C****C****C****G****G****G**.GA.**C**..**G****A**..**U****A****A****G****A****G****A****G****G**GGGG | |
|  |  | NZ\_AABF02000062.1/1992-1888  | UAGA**A****A****U****G****A****A****C****C**.**A****U****C**A.AGA**G**.**A****G****A****U**.**U****G****A****G****G****G**A...**C****A****G**.**G**.**C****C****C**GUU**G****A****G**.**A****U****C****U**.**C****A****G****C**AA**C****C**.**U**.**A****C****A****U****U**........................AAUU-....................................................**A****U****U****G****U**.G...**U**..**G****G**U**G**..**C****U**AAU**U****C****C****U****G**...------....................----...........------.AU.**A**..**G****A**..**U****G****G****A****A****A****A****G****G**UUAU | |
|  |  | NZ\_AAII01000013.1/63749-63882  | UUCA**U****C****U****G****G****C****U****C**.**A****U****C**C.AGA**G**.**G****G****G****C**.**A****G****A****G****G****G**A...**A****C****G**.**G**.**C****C****C**AGC**G****A****A**.**G****C****C****C**.**C****G****G****C**AA**C****C**.-.**A****C****C****G****U**CGC.....................AUCCA.................................................UGC**G****C****G****G****C**.A...-..**G****G**U**G**..**C****U**AAU**U****C****C****G****A**...**C****C****C****G****G****G**AC..................GCAG.....CCGGCU**C****C****C****G****G****G**.AA.**A**..**G****A**..**U****G****A****G****G****A****G****U****U**CCGC | |
|  |  | NZ\_AAAS03000004.1/72829-72686  | AAAC**G****C****A****U****U****C****U****U**.**A****U****C**A.AGA**G**.**U****G****G****U**.**G****G****A****G****G****G**A...**A****A****G**.**G**.**C****C****C**UGC**G****A****A**.**G****C****C****A**.**C****A****G****C**AA**C****C**.**G**.**G****U****C****U****U**CCGGGUCGCA..............UUCGA................................GCACGAAGGCGAACCGGAAG**G****U****G****A****C**.-...**A**..**G****G**U**G**..**C****U**AAA**U****C****C****U**-...--**G****C****C**-....................GGGA...........-**G****G****C**--.AA.**A**..**G****A**..**U****G****A****G****A****A****C****G****G**CGCU | |
|  |  | NZ\_AAIE01000003.1/136778-136908  | UUCA**U****C****U****G****G****C****U****C**.**A****U****C**C.AGA**G**.**G****G****G****C**.**A****G****A****G****G****G**A...**A****C****G**.**G**.**C****C****C**AGC**G****A****A**.**G****C****C****C**.**C****G****G****C**AA**C****C**.-.**A****C****C**--CGC.....................AUCCC..............................................GUGCGC--**G****G****C**.A...-..**G****G**U**G**..**C****U**AAU**U****C****C****G****G**...**C****C****U****G****G****U**GGCA................GACU.........GC**C****C****C****G****G****G**.AA.**A**..**G****A**..**U****G****A****G****G****A****G****C****U**UCUU | |
|  |  | NC\_003919.1/3558028-3557894  | CCUA**G****C****C****U****C****A****C****C**.**A****U****C**G.AGA**C**.**C****G****G****C**.**G****G****A****G****G****G**A...**C****A****G**.**G**.**C****C****C**UUU**G****A****U**.**G****C****C****G**.**G****G****G****C**AG**C****C**.**A**.**G****C****G**-**G**AGCGCG..................CAAGC.................................................GUC**C****G****C****G****U**.U...**U**..**G****G**U**G**..**C****C**AAA**U****C****C****U****G**...**C****G****G****G****G****A**C...................CUCC.......GCGU**C****C****G****C****C****G**.AA.**A**..**G****A**..**U****G****G****U****U****C****G****A****A**UCGU | |
|  |  | NZ\_AADW02000005.1/146206-146089  | CAUA**U****A****A****C****A****C****U****U**.**A****U****C**A.AGA**G**U**G****G****A****C**.**C****G****A****G****A****G**AU..**C****U****G**.**G**.**A****U****C**GAU**G****A****C**.**G****U****C****C**.**C****A****G****C**AA**C****C**.**U**.**G****C**---........................GCACA....................................................---**G****C**.A...**A**..**G****G**U**G**..**C****U**ACG**A****C****C****A****G**...**C****A****A****A****G****C**....................GAAA...........**G****C****U****U****U****G**.GU.**U**..**G****A**..**U****A****A****A****A****A****G****C****G**GCGA | |
|  |  | NC\_003902.1/3379779-3379645  | CGUA**G****C****C****U****C****A****C****C**.**A****U****C**G.AGA**C**.**C****G****G****C**.**G****G****A****G****G****G**A...**C****A****G**.**G**.**C****C****C**UUU**G****A****U**.**G****C****C****G**.**G****G****G****C**AG**C****C**.**A**.**G****C****G**-**G**AGCGCG..................CAAGC.................................................GCC**C****G****C****G****U**.U...**U**..**G****G**U**G**..**C****C**AAA**U****C****C****U****G**...**C****G****G****G****G****A**C...................CUCC.......GCGU**C****C****G****C****C****G**.AA.**A**..**G****A**..**U****G****G****U****U****C****G****A****A**UCGU | |
|  |  | NC\_007086.1/1531032-1531166  | CGUA**G****C****C****U****C****A****C****C**.**A****U****C**G.AGA**C**.**C****G****G****C**.**G****G****A****G****G****G**A...**C****A****G**.**G**.**C****C****C**UUU**G****A****U**.**G****C****C****G**.**G****G****G****C**AG**C****C**.**A**.**G****C****G**-**G**AGCGCG..................CAAGC.................................................GCC**C****G****C****G****U**.U...**U**..**G****G**U**G**..**C****C**AAA**U****C****C****U****G**...**C****G****G****G****G****A**C...................CUCC.......GCGU**C****C****G****C****C****G**.AA.**A**..**G****A**..**U****G****G****U****U****C****G****A****A**UCGU | |
|  |  | NZ\_AADW02000002.1/259178-259295  | CCUA**U****A****C****G****A****U****A****U**.**A****U****C**A.AGA**G**.**U****G****G****A**.**C****G****A****G****A****G**AC..**C****U****G**.**G**.**C****U****C**UAG**G****A****C**.**U****C****C****A**.**C****G****G****C**AA**C****C**.**U**.**G****C****C**--........................GAUC-....................................................--**G****G****C**.A...**A**..**G****G**U**G**..**C****U**CCG**A****C****C****A****G**...**C****A****A****A****G****C**....................AGUU...........**G****C****U****U****U****G**.GA.**U**..**G****A**..**U****A****C****A****C****A****C****G****A**GAAA | |
|  |  | NC\_006834.1/1911391-1911524  | CUUA**G****C****C****U****C****A****C****C**.**A****U****C**G.AGA**C**.**C****G****G****C**.**G****G****A****G****G****G**A...**C****A****G**.**G**.**C****C****C**UUU**G****A****U**.**G****C****C****G**.**G****G****G****C**AG**C****C**.**A**.**G****C****G**-**G**AGCGCG..................CAAGC.................................................GUC**C****G****C****G****U**.U...**U**..**G****G**U**G**..**C****C**AAA**U****C****C****U****G**...**C****G****G****G****G****A**CCC.................CGCG..........U**C****C****G****C****C****G**.AA.**A**..**G****A**..**U****G****G****U****U****C****G****A****C**UCGU | |
|  |  | NZ\_AADQ01000086.1/1-112  | UUUU-----**C****U****U**.**A****U****C**A.AGA**G**.**C****G****G****U**.**G****G****A****G****G****G**AA..**U****C****G**.**G**.**C****C****C**AGU**G****A****A**.**G****C****C**-.**C****A****G****C**AG**C****G**.**G**.**A****G****C**--........................GCAA-....................................................--**G****U****U**.-...**C**..**U****A**U**G**..**C****U**AAU**U****C****C****G****A**U..**C****A****G****A****A****G**....................UAAU...........**A****U****U****C****U****G**.GC.**A**..**G****A**..**U****A****A****G****U****A****G****U****A**GCUU | |
|  |  | NZ\_AABD03000014.1/41139-41016  | AGUA**G****U****U****G****A****U****U****U**.**A****U****A**A.AGA**A**.**G****A****G****G**.**G****G****A****G****A****G**AA..**C****G****G**.**G**.**C****U****C**UUU**G****A****A**.**C****C****U****C**.**U****A****G****C**AA**C****C**.**U**.-**C****C****G****G**GAG.....................CAAAU....................................................**C****C****A****G****A**.A...**A**..**G****G**U**G**..**C****U**AAU**U****C****C****U****G**A..**C****C****U****G****A**-....................AUAA...........-**A****C****A****G****G**.GC.**A**..**U****A**..**U****A****A****A****U****U****A****A****U**UGCA | |
|  |  | NZ\_AABN02000002.1/621382-621261  | AAAA**G****A****C****A****A****C****U****C**.**A****U****G**A.AGA**G**.**G****A****C****G**.**A****G****A****G****G****G**GU..**C****U****G**.**G**.**C****C****C**GAU**G****A****C**.**C****G****U****C**.**C****G****G****C**AA**C****C**.**U**.**G****C****C**--........................CGCAA....................................................--**G****G****C**.A...**A**..**G****G**U**G**..**C****C**AAA**G****C****C****A****G**...**C****C****A****C****G****G**UG..................AGAU..........G**C****C****G****C****G****G**.UG.**C**..**C****A**..**U****G****A****G****G****U****C****G****A**CAGC | |
|  |  | NC\_001263.1/1363053-1363199  | AGGG**U****C****A****C****C****U****U****U**.**A****U****C**C.AGA**G**U**C****G****G****C**.**G****C****A****G****G****G**AC..**C****U****G**.**G**.**C****C****C**CAU**G****A****C**C**G****C****C****G**.**C****A****G****C**AA**C****C**.**G**.-**G****C****C****C**UC......................AUCA-....................................................**C****G****G****C****A**.G...**C**..**G****G**U**G**..**C****U**--U**U****C****C****A****G**A..**C****C****C****G****C****G**CGAGCAGCGCCCGA......CGAU..GGGCGGCGC**C****G****C****G****G****G**.AA.**C**..**G****A**..**U****A****A****A****G****G****A****A****G**GCGG | |
|  |  | NZ\_AAEB02000006.1/49815-49938  | UCUG**G****C****C****C****G****U****A****C**.**A****U****C**G.AGA**G**.**A****G****G****C**G**C****G****A****G****G****G**AA..**C****U****G**.**G**.**C****C****C**AAA**G****A****C**C**G****C****C**-.**C****G****G****C**AA**C****C**.**U**.**G****G****C****U****U**........................CUCCG....................................................**G****A****G****C****C**.A...**A**..**G****G**U**G**..**C****C**AAU**G****C****C****A****G**...**C****C****C****G****C****C**....................GAGA...........**G****A****C****G****G****G**.GA.**C**..**G****A**..**U****G****U****A****G****C****U****C****G**AAAC | |
|  |  | NZ\_AAIC01000108.1/4619-4742  | AUGU**A****G****U****G****A****U****U****U**.**A****U****A**A.AGA**A**.**G****G****G****C**.**G****G****A****G****A****G**AU..**U****A****G**.**G**.**C****U****C**AAG**G****A****A**.**A****C****C****C**.**U****G****G****C**AA**C****C**.**U**.--**G****C**-........................UGCAA....................................................-**G****G****A****A**.A...**A**..**G****G**U**G**..**C****C**AAA**A****C****C****U****A**A..**C****C****U****G****C****U**CA..................AUAU..........G**A****G****C****C****G****G**.AA.**A**.U**G****A**..**U****A****A****A****U****U****A****A****U**UGAU | |
|  |  | NZ\_AAHE01000003.1/65030-64890  | UGGA**G****C****G****A****C****C****U****C**.**A****U****C**C.AGA**G**.**C****G****C****C**.**C****G****A****G****A****G**AU..**C****U****G**.**G**.**C****U****C**GUU**G****A****C**.**G****G****C****G**.**C****G****G****C**AA**C****C**.**G**.**G****A****C****C****U**........................CAUC-....................................................**A****C****G****U****C**.A...**C**..**G****G**U**G**..**C****C**AAG**G****C****C****A****G**...**C****C****C****A****G****C**GCAAGAGG............UCAACGAUGGCCGCG**U****G****U****G****G****G**.AA.**C**..**G****A**..**U****C****A****G****G****G****A****A****G**GUCA | |
|  |  | NZ\_AABD03000001.1/130314-130212  | CGCA**C****A****G****C****A****U****U****U**.**A****U****A**C.AGA**A**.**U****A****G****G**.**G****A****A****G****A****G**AG..**C****A****G**.**G**.**C****U****C**AAU**G****A****U**.**C****C****U****A**.**U****A****G****C**AA**C****C**.**G**.**A****C**---........................UUAAA....................................................---**G****A**.A...**C**..**G****G**U**G**..**C****U**AAA-**C****C****U****G**...------....................-CCC...........------.AA.**A**..**A****A**..**U****A****A****A****A****G****G****G****A**ACUA | |
|  |  | NC\_002937.3/1264327-1264192  | CCUG**C****G****U****C****A****C****U****C**.**A****U****G**A.CGA**G**.**G****G****U****G**.**C****G****A****G****G****G**AC..**U****U****G**.**G**.**C****C****C**GAU**G****A****C**.**C****A****C****C**.**C****G****G****C**AA**C****C**.**U**.-**G****U****G****C**AGGCGAGACGCC............GUCA-................................................GCCA**G****C****A****C****A**.A...**C**..**G****G**U**G**..**C****C**AA--**C****C****A****G**...**C****C****G****C****G**-AC..................GGGA..........A-**C****G****C****G****G**.GA.**A**..**C****A**..**U****G****A****G****G****C****C****A****G**GCUU | |
|  |  | NZ\_AAIA01000001.1/71466-71350  | AGCU**U****C****A****A****A****A****A****C**.**A****U****U**A.AGA**G**.**U****G****G****C**G**U****G****A****G****G****G**UU..**C****U****G**.**A**.**C****C****C**GAU**G****A****C**C**G****C****C**-.**C****G****G****C**AA**C****C**.**U**.**G****G****C****G**-........................AAGC-....................................................-**C****G****C****C**.A...**A**..**G****G**U**G**..**C****C**AAG**G****U****C****A****G**...**C****C****C****G**--....................GAAA...........--**C****G****G****G**.GA.**C**..**G****A**..**U****G****U****G****G****C****G****A****A**CGAC | |
|  |  | NZ\_AABD03000005.1/216932-217045  | GGGU**C****G****U****G****U****A****U****U**.**A****U****A**C.AGA**A**C**G****A****A****G**.**G****G****A****G****A****G**AU..**U****A****G**.**G**.**C****U****C**UAU**G****A****A**.**C****U****U****C**.**U****G****G****C**AA**C****C**.**U**.**G****C****C****C**-........................ACCA-....................................................-**A****A****G****C**.A...**A**..**G****G**U**G**..**C****U**AAU**U****C****C****U****A**...**C****C****C**---....................-AUG...........---**U****G****G**.AA.**C**..**U****A**..**U****A****A****A****A****A****G****U****A**CGAC | |
|  |  | NC\_006510.1/460747-460629  | UUGC**A****U****A****C****G****U****U****G**.**C****U****C**G.GGA**G**.**U****G****A****G**.**G****G****A****G****G****A**A...**U****U****G**.**G**.**C****U****C**AAU**G****A****A**.**C****U****C**-.**C****G****U****C**AA**C****C**.**U**.**G****C****C****G**-........................CCUA-....................................................-**U****A****G****C**.A...**A**..**A****G**U**G**..**C****C**AAU**U****C****C****A****G**...**C****U****A****A****A****G**....................AAGA..........U**C****U****U****U****A****G**.UA.**A**..**A****A**..**U****A****A****G****G****G****G****C****A**CCCU | |
|  |  | NZ\_AAEF02000160.1/414-313  | CCGG**C****A****A****C****A****C**-**C**.**A****U****C**C.AGA**G**.**U****G****G****C**.**C****G****A****G****A****G**AC..**C****U****G**.**G**.**C****U****C**GAC**G****A****C**.**G****C****C****A**.**C****A****G****C**AA**C****C**.-.**A****C****C****G****C**........................-GCA-....................................................**G****C****G****G****C**.A...-..**G****G**U**G**..**C****U**CCC**G****C****C****A****G**G..**.****.****.****.****.****.**...................................**.****.****.****.****.****.**.GG.**C**..**G****A**..**U****G**-**G****A****G****G****A****A**CGCA | |
|  |  | NC\_005364.1/557978-558087  | AAAU**A****A****U****A****U****C****U****U**.**A****U****U**A.AGA**A**.**C****C****A****U**.**G****G****A****G****A****G**AU..**A****A****A**.**A**.**C****U****U**AAU**G****A****A**.**G****U****G****G**.**U****A****G****C**AA**C****C**.**U**.**A**----........................UUAGA....................................................----**U**.U...**A**..**G****G**U**G**..**C****U**UAA**A****U****A****A****G**...**G****G****C****U**--....................-UUA...........--**A****G****C****U**.AA.**C**..**A****A**..**U****G****A****G****A****U****U****G****U**CUUA | |
|  |  | NZ\_AAIK01000053.1/11876-11737  | UCCA**U****U****G****C****U****C****A****U**C**A****U****C**A.AAA**A**.**A****G****G****C**.**U****G****A****G****G****G**AA..**U****A****G**.**A**.**C****C****C**GGU**G****A****A**.**G****C****C****U**.**U****G****A****C**AA**C****C**.**G**.**A****C****U****C****C**G.......................UUCAA................................................AGAC**G****G****A****A****C**.A...**C**..**G****G**U**G**..**A****C**ACA**U****U****C****U****U**...**U****U****A****A****A****C**GCGC................UAUU......CAUGA**G****U****U****U****U****C**.AG.**A**CU**G****A**..**U****A****C****G****A****U****U****C****A**CGCC | |
|  |  | NZ\_AAEF02000010.1/52121-52019  | ACUU**G****A****C****U****C****G****C****C**.**A****U****C**C.AGA**G**.**C****G****G****C**.**C****G****A****G****A****G**AC..**C****C****G**.**G**.**C****U****C**GUC**G****A****C**.**G****C****C****G**.**C****A****G****C**AA**C****C**.**U**.**C****C****C**--........................CCCG-....................................................--**G****G****G**.C...**A**..**G****G**U**G**..**C****U**CCC**G****C****C****G****G**G..**.****.****.****.****.****.**...................................**.****.****.****.****.****.**.AC.**C**..**G****A**..**U****G****G****G****A****A****G****G****G**GACG | |
|  |  | NZ\_AAEF02000050.1/6416-6521  | UCGA**A****C****C****A****C****A****C****C**.**A****U****C**C.AGA**G**.**C****G****G****C**.**C****G****A****G****A****G**UC..**C****U****G**.**G**.**C****U****C**GAC**G****A****C**.**G****C****C****G**.**C****A****G****C**AA**C****C**.-.**C****C****C****C****G**........................AGCCC....................................................**G****G****G****C****G**.A...**G**..**G****G**U**G**..**C****U**AAA**G****C****C****A****G**...------....................----...........------.GC.**C**..**G****A**..**U****G****G****A****G****U****U****C****G**UGAU | |
|  |  | NZ\_AAEF02000035.1/11882-11779  | CUGG**A****C****C****C****A****C**-**C**.**A****U****C**C.AGA**G**.**C****G****G****C**.**C****G****A****G****A****G**AC..**C****U****G**.**G**.**C****U****C**GUC**G****A****C**.**G****C****C****G**.**C****A****G****C**AA**C****C**.**G**.**C****C****C****C****C**........................-UCG-....................................................**G****G****G****G****U**.G...**U**..**G****G**U**G**..**C****U**CAC**G****C****C****A****G**G..**.****.****.****.****.****.**...................................**.****.****.****.****.****.**.AC.**C**..**G****A**..**U****G**-**G****A****G****G****A****C**GACC | |
|  |  | NC\_001263.1/886190-886055  | AGCC**A****C****C****C****A****C****U****U**.---C.AGA**G**.**C****G****C****C**.**C****G****A****G****A****G**AC..**C****U****G**.**G**.**C****U****C**GAC**G****A****C**.**G****G****C****G**.**C****G****G****C**AA**C****C**.**G**.**G****A****C**--CCC.....................AUCAC....................................................--**G****U****C**.A...**C**..**G****G**U**G**..**C****C**AAG**G****C****C****A****G**...**C****C****C****C****C****U**GGGCGUG.............UCAACGAUGAGCCGC**C****G****G****G****C****G**.GG.**A**..-**C**..**C****A****A****G****C****G****G****G****A**AGGC | |
|  |  | NZ\_AAEF02000040.1/10779-10892  | GCUG**G****C****C****C****G****A****C****C**.**A****U****C**C.AGA**G**.**C****G****G****C**.**C****G****A****G****A****G**AC..**C****U****G**.**G**.**C****U****C**GUC**G****A****C**.**G****C****C****G**.**C****A****G****C**AA**C****C**.**A**.**U****C****C****C****C**CGC.....................CGCCC.................................................CGG**C****G****G****G****G**.A...**G**..**G****G**U**G**..**C****U**CAC**G****C****C****A****G**G..**.****.****.****.****.****.**...................................**.****.****.****.****.****.**.AC.**C**..**G****A**..**U****G****G****G****A****G****G****A****A**GAAG | |
|  |  | NZ\_AABJ03000007.1/4799-4696  | CAGU**U****A****A****A****A****C****C****U**.**A****U****U**C.AGA**G**C**G****U****A****A**.**U****G****A****G****G****G**AU..**A****C****C**.**A**.**C****C****C**GAU**G****A****G**.**C****U****A****C**.**C****G****G****C**AA**C****C**.**U**.**G****C****U**--........................UUUU-....................................................--**U****G****C**.A...**A**..**G****G**U**G**..**C****C**CCA**G****U****G****G****U**A..**.****.****.****.****.****.**...................................------.AG.**C**..**G****A**..**U****G****G****G****U****C****U****A****A**GUCU | |
|  |  | NZ\_AAHE01000002.1/179586-179456  | CUCG**G****C****A****C****G****G****U****C**.**A****U****C**C.UGA**G**.**C****G****C****C**.**C****G****A****G****G****G**AAUU**C****C****A**.**G**A**C****C****C**ACU**G****A****C**.**G****G****C****G**.**C****G****G****C**AA**C****C**.**G**.**G****C****C****C****U**........................CAUC-....................................................**A****C****G****G****C**.A...**C**..**G****G**U**G**..**C****C**CCG**U****C****U****G****G**...**A****A****G****G****C****C**CCGCGC..............GCAA.........GA**C****G****C****C**--.GA.**C**..**G****A**..**U****G****G****C****G****U****G****A****G**AGCG | |
|  |  | NZ\_AABH02000008.1/48171-48031  | UUUA**A****A****A****C****A****G****C****U**.**A****U****C**A.AGA**G**.**U****A****G****G**.**C****G****A****G****U****G**AU..**A****C****A**.**G**.**C****A****C**AAU**G****A****C**.**C****C****U****G**.**C****A****C****C**AA**C****C**.**G**.**G**-**C****A****A**A.......................AUAAC..................GAAAAGCAGCUACGAGAGUGUAUUUUCAUCAGUU**U****U****G****A****C**.A...**U**..**G****G**U**G**..**G****U**CCA**U****C****U****G****U**...**.****.****.****.****.****.**...................................------.AC.**A**..**G****A**..**U****A****G****U****C****G****U****C****A**CGUU | |
|  |  | NZ\_AAHG01000001.1/499488-499600  | UAAG**A****G****U****G****A****C****C****C**.**A****U****C**C.CGA**G**.**C****G****G****C**.**C****G****A****G****A****G**AC..**C****U****G**.**G**.**A****U****C**GUU**G****A****C**.**G****C****C****G**.**C****A****G****C**AA**C****C**.**C**.**U****G****G**--........................AAACC....................................................--**U****C****G**.G...**U**..**G****G**U**G**GA**C****G**UAG**U****C****C****A****G**...**C**-----....................GGCA...........-----**G**.AA.**G**..**G****A**CG**U****G****G****G****C****A****U****C****G**ACAU | |
|  |  | NZ\_AAAP01003574.1/1026-908  | UCCG**A****G****C****C****A****A****C****C**.**A****U****C**C.GGA**G**.**C****G****G****C**.**C****G****A****G****U****G**AC..**G****U****G**.**G**.**C****A****C**GUC**G****A****C**.**G****C****C****G**.**C****A****G****C**AA**C****C**.-.-**C****C****C****C**GGACCGC.................CUCG-.............................................GCGGCCG**G****G****C****G****C**.A...**G**..**G****G**U**G**..**C****U**ACU**G****C****C****A****C**...**.****.****.****.****.****.**...................................-----**G**.AU.**C**..**G****A**..**U****G****G****A****G****G****A****G****G**ACUC | |
|  |  | NC\_001263.1/980694-980823  | CCGU**G****C****G****C****G****G****U****C**.**A****U****C**C.AGA**G**U**C****G****C****C**.**C****C****A****G****G****G**UGUU**U****C****C**.**U**G**C****C****C**GCC**U****A****C**.**G****G****C****G**.**C****A****G****C**AA**C****C**.**G**.-**G****C****C**-........................UUCAU..................................................CA**C****G****G****U****C**.A...**C**..**G****G**U**G**..**C****U**-UU**C****A****G****G****A**A..**A****G****G****G****C****C**G...................UUUA.........GG**U****G****C****G****C****C**.GA.**C**..**G****A**..**U****G****G****C****G****C****G****A****G**CGGC | |
|  |  | AAGA01012172.1/587-471  | AUUG**A****A****A****C****U****G****U****U**.**A****U****A**A.AGA**A**.**A****G****G****C**.**A****G****A****G****G****G**AU..**U****A****G**.**A**.**C****C****C**GAU**G****A****A**.**G****C****C****U**.**U****A****G****C**AA**C****C**.-.**C****U****U****U****A**........................CUAU-....................................................**U****A****A****A****G**.A...**A**..**G****G**U**G**..**C****U**AAA**U****U****C****U****A**...**C****C****A****A**--....................UUUU...........--**U****U****G****G**.AU.**A**..**G****A**..**U****A****A****C****A****A****A****A****A**UGAA | |
|  |  | AAFX01049186.1/415-546  | AUCA**A****C****G****G****U****C****U****C**.**A****U****U**C.AGA**G**.**U****G****G****C**.**A****G****A****G****G****G**A...**C****U****G**.**G**.**C****C****C**GAU**G****A****A**.**G****C****C****A**.**C****G****G****C**AA**C****C**.**G**.**G****C****C****U****C**G.......................AUAAU.................................................UUC**G****A****G****G****U**.GACC**A**..**G****G**U**G**..**C****C**AAU**U****C****C****A****G**...**C****U****C****G****A****G**C...................GAAA..........G**U****U****C****G****G****G**AAA.**A**..**A****A**..**U****G****A****G****A****A****G****A****G**CAGU | |
|  |  | AAGA01004112.1/794-937  | UCAU**G****C****A****U****U****C****U****C**.**A****U****C**A.AGA**A**.**A****G****G****U**.**G****G****A****G****G****G**AU..**C****A****G**.**G**.**U****C****C**UUU**G****A****A**.**G****C****C****U**.**U****G****G****C**AA**C****C**.**A**.**U****C****U****A****G**UAC.....................UUAAU..............................................UAAGUG**C****U****A****G****A**.A...**A**..**G****G**U**G**..**C****U**ACA**U****C****C****U****G**...**C****U****U****A****G****U**AC..................UUAA.AAACUUAUGU**G****C****U****G****A****G**.AA.**A**..**G****A**..**U****G****A****G****U****G****C****G****A**AGAG | |
|  |  | AAFX01048923.1/825-710  | CUGC**A****G****A****C****G****C****U****U**.**A****U****C**C.AGA**G**.**U****G****G****C**.**U****G****A****G****G****G**AA..**C****A****G**.**G**.**C****C****C**GUC**G****A****A**.**G****C****C****A**.**C****A****G****C**AA**C****C**.**A**.**G****G****C**--........................GCAA-....................................................--**G****C****C**.C...**A**..**G****G**U**G**..**C****U**ACU**U****C****C****U****G**...**C****C****C****G****A**-....................UAAA...........-**C****C****G****G****G**.AG.**A**..**G****A**..**U****A****A****G****A****G****C****C****U**CGGC | |
|  |  | AACY01426272.1/132-241  | AGUU**U****A****U****U****A****C****U****U**.**A****U****C**A.AGA**A**.**A****G****G****U**.**G****G****A****G****G****G**A...**C****A****G**.**G**.**C****C****C**UGU**G****A****A**.**G****C****C****U**.**U****G****G****C**AA**C****C**.**U**.-----........................UUGAC....................................................-----.-...**U**..**G****G**U**G**..**C****C**AAU**U****C****C****U****G**AU.**U****U****C****C****A**-....................-AUG...........-**U****G****G****A****A**.UA.**A**..**G****A**..**U****A****A****G****U****U****A****A****A**AGAG | |
|  |  | AAFX01098755.1/483-341  | UACC**G****U****U****U****A****C****U****U**.**A****U****C**A.AGA**A**.**A****G****G****C**.**U****G****A****G****G****G**UA..**A****U****G**.**G**.**C****C****C**UGC**G****A****A**.**G****C****C****U**.**U****G****A****C**AA**C****C**.**U**.-**C****C****U****U**C.......................UACAA...................................CUCCUGGCGGACUUGGG**A****A****G****G****A**.A...**A**..**G****G**U**G**..**C****C**AAA**U****C****C****A****U**...**C****C****U****U****G****C**CG..................CGUU..........U**G****C****G****G****G****G**.AA.**A**..**G****A**..**U****A****A****G****U****C****A****G****A**UCAC | |
|  |  | AAFX01054820.1/1101-968  | CGGG**A****C****G****C****U****C****U****U**.**A****U****C**G.AGA**G**.**U****G****G****U**.**G****G****A****G****G****G**A...**C****U****G**.**G**.**C****C****C**GAC**G****A****A**.**G****C****C**-.**C****G****G****C**AA**C****C**.**C**.**G****C****C****G****A**ACC.....................GUCAA................................................CGGU**U****C****G****G****G**.C...**G**..**G****G**U**G**..**C****C**AAG**U****C****C****G****A**...**C****C****U****C****A****U**UCC.................GCAG........CGG**A****U****G****G****G****G**.AG.**A**..**G****A**..**U****G****A****G****A****G****U****U****C**GAAA | |
|  |  | AACY01080826.1/330-455  | UUGC**A****U****A****A****A****C****U****U**.**A****U****G**A.AGA**A**.**A****G****G****U**.**G****G****A****G****G****G**A...**U****U****G**.**G**.**C****C****C**UUU**G****A****A**.**A****C****C****U**.**U****G****G****C**AA**C****C**.-.**C****U****U****A****G**........................CUAAU....................................................**A****U****A****A****A**.A.GU**A**..**G****G**U**G**..**C****U**AAA**U****C****C****A****A**...**U****U****U****C****G****A**UU..................UUUA..........A**U****U****G****A****A****A**.UA.**A**..**G****A**..**U****A****A****G****U****G****U****G****G**UUUU | |
|  |  | AAFY01008477.1/792-654  | GACU**C****C****G****U****G****C****U****U**.**A****U****C**A.AGA**A**.**A****G****G****U**.**G****G****A****G****G****G**AA..**U****A****G**.**G**.**C****C****C**UGC**G****A****A**.**A****C****C****U**.**U****G****G****C**AA**C****C**.-.**A****A****C****U****A**UAAGUCCCG...............AAUGU.............................................UCGGGAU**U****G****G****A****A**.A...**A**UC**G****G**U**G**..**C****U**AAA**U****C****C****U****A**...**U****U****C****G****C****U**....................-UUA...........**A****G****C****G****G****A**.AA.**A**..**G****A**..**U****A****A****G****U****A****G****A****A**AAAU | |
|  |  | AAFX01081827.1/80-234  | UGAA**U****A****C****C****G****C****U****C**.**A****U****C**C.AGA**G**.**G****G****G****U**.**G****G****A****G****G****G**A...**C****C****G**.**G**.**C****C****C**UGC**G****A****A**.**G****C****C****C**.**C****G****G****C**AA**C****C**.**A**.-**C****C****C****G**GCUGUGGCGCGUCGCG........AUCCU.........................................GCGAGGCCGGC**C****G****G****G****A**GA...**U**..**G****G**U**G**..**C****C**AAC**U****C****C****G****G**...**C****C****U****G****C****G**A...................CCAA......GGUGG**C****G****C****A****G****G**.GA.**A**..**G****A**..**U****G****G****G****G****A****G****A****A**AGGC | |
|  |  | AAFZ01018340.1/187-305  | UUGA**G****A****A****U****C****G****A****U**.**A****U****C**G.AGA**G**.**C****G****A****C**G**U****G****A****G****G****G**UU..**C****U****G**.**G**.**C****C****C**AGU**G****A****A**C**G****U****C**-.**C****G****G****C**AA**C****C**.**U**.**G****C****C****C**-........................CAUG-....................................................-**G****G****G****C**.A...**A**..**G****G**U**G**..**C****C**AAU**G****C****C****A****G**...**C****C****C****G****G**-....................GAAA...........-**C****C****G****G****G**.AA.**C**..**G****A**..**U****G****U****G****G****U****C****C****A**CAUG | |
|  |  | AAGA01023434.1/7-144  | UACU**G****A****A****A****U****G****U****U**.**A****U****C**A.AGA**A**.**A****G****G****U**.**G****G****A****G****G****G**AU..**U****A****G**.**A**.**C****C****C**AUU**G****A****A**.**G****C****C****U**.**U****A****G****C**AA**C****C**.-.**C****U****U****A****G**........................ACUUA....................................................**C****U****A****A****G**.A...**A**..**G****G**U**G**..**C****U**AAA**U****U****C****U****A**...**C****U****U****A****A****U**UUUCGAUUCA..........UUAA....UCGAUAC**A****U****U**-**G****G**.AU.**A**..**G****A**..**U****A****A****C****U****A****A****A****A**ACUA | |
|  |  | AAFX01122557.1/18-159  | CCCU**G****G****C****U****G****C****U****C**.**A****U****C**C.AGA**G**.**A****G****G****U**.**G****G****A****G****G****G**A...**C****C****G**.**G**.**C****C****C**UGA**G****A****A**.**A****C****C****U**.**C****G****G****C**AA**C****C**.**G**.**C****G****A****A**-........................GCGCA....................................................-**G****U****C****G**.C...**U**..**G****G**U**G**..**C****C**AAA**U****C****C****G****G**...**C****A****G****A****A****G**UUCUCCGGCAGGGUGGCCGGGAGA.........CA**U****U****U****C****U****G**.GA.**A**..**G****A**..**U****G****A****G****G****A****A****A****U**UCGC | |
|  |  | AAFX01071693.1/989-870  | GGGC**G****A****G****C****A****C****U****U**.**A****U****C**CCAGA**G**.**U****G****G****C**.**U****G****A****G****G****G**AA..**C****A****G**.**G**G**C****C****C**GCU**G****A****A**.**G****C****C****A**.**C****A****G****C**AA**C****C**A**G**.**G****C****G****C**-........................GAGA-....................................................-**G****C****C****C**.C...**C**..**G****G**U**G**..**C****U**ACU**U****C****C****U****G**...**C****C****C****G****A**-....................-UCA...........-**A****C****G****G****G**.AG.**A**..**G****A**..**U****A****A****G****A****G****C****C****U**CGGU | |
|  |  | AAFX01104703.1/201-32  | CCGC**G****C****G****C****U****U****U****C**.**A****U****C**A.AGA**G**.**U****G****A****C**.**U****G****A****G****G****G**A...**C****U****G**.**G**.**C****C****C**GUU**G****A****A**.**G****U****C****A**.**C****G****G****C**AA**C****C**.-.**G****C****U****C****A**AAGGUUGGUCGACCGCG.......AAAUA...............................GCGCGGCAGAUCAGUCGAUGG**G****G****A****C****C**.A...-..**G****G**U**G**..**C****C**AAA**U****C****C****A****G**...**C****U****U****C****G****G**UGU.................UGUU..CCGACAACA**U****C****G****A****G****G**.GA.**A**..**G****A**..**U****G****A****G****A****G****G****A****U**CCGU | |
|  |  | AACY01585427.1/646-519  | ACGU**U****U****A****U****U****U****G****U**.**A****U****C**G.AGA**U**G**A****U****A****C**.**U****G****A****G****G****G**AU..**C****U****G**.**G**.**C****C****C**UUU**G****A****A**.**G****U****G****U**C**G****A****G****C**AG**C****C**.**U**.**U****U****U**--........................CAGC-....................................................--**A****U****A**.A...**A**..**G****G**U**G**..**C****U**AAU**G****C****C****A****G**...**C****C****C****A****A****C**CGGC................AAAU........GUC**G****G****U****G****G****G**.AGU**C**..**G****A**..**U****G****C****G****A****C****U****U****G**AUUG | |
|  |  | AACY01775498.1/574-468  | UCCA**U****U****G****U****G****U****U****C**.**A****U****C**A.AGA**G**.**A****G****U****G**.**U****G****A****G****G****G**UU..**C****U****G**.**G**.**C****C****C**UAU**G****A****C**.**C****A****U****U**.**C****A****C****C**AA**C****C**.**G**.**G****C****C****C****U**........................UUCG-....................................................**G****G****G****G****C**.A...**C**..**G****G**U**G**..**G****U**AAC**G****C****C****A****G**...------....................AUUC...........------.--.-..**G****A**..**U****G****A****G****G****A****G****A****A**ACCA | |
|  |  | AACY01520571.1/680-806  | UACG**A****A****A****U****U****G****G****U**.**A****U****C**G.AGA**U**G**A****C****A****C**.**U****G****A****G****G****G**AU..**C****U****G**.**G**.**C****C****C**UUU**G****A****A**.**G****U****G****A**C**G****A****G****C**AG**C****C**.**U**.**U****U****U**--........................UCAGC....................................................--**A****U****A**.A...**A**..**G****G**U**G**..**C****U**AAU**G****C****C****A****G**...**C****C****C****A****A****C**CGGA................AUAU..........A**G****G****U****G****G****G**.AGU**C**..**G****A**..**U****G****C****G****A****C****U****U****G**AUUG | |
|  |  | AACY01438816.1/560-676  | UCAG**G****G****G****C****G****U****U****C**.**A****U****C**A.AGA**G**.**A****G****U****G**.**U****G****A****G****G****G**UU..**C****U****G**.**G**.**C****C****C**UAU**G****A****C**.**C****A****U****U**.**C****A****C****C**AA**C****C**.**G**.**G****C****C****C****C**........................UUCG-....................................................**G****G****G****G****C**.A...**C**..**G****G**U**G**..**G****U**AAC**G****C****C****A****G**...**A****U****U****C**--....................-GAU...........--**G****A****G****G**.AG.**A**..**A****A**..**U****C****A****U****G****G****U****C****A**UGUC | |
|  |  | AACY01675808.1/610-483  | ACUA**U****U****A****U****A****U****G****U**.**A****U****C**A.CGA**U**G**A****C****A****C**.**U****G****A****G****G****G**AU..**C****U****G**.**G**.**C****C****C**UUU**G****A****A**.**G****U****G****U**C**G****A****G****C**AU**C****C**.**U**.**U****U****U**--........................CAGC-....................................................--**A****U****A**.A...**A**..**G****G**U**G**..**C****U**AAU**G****C****C****A****G**...**C****C****C****A****U****C**GGGU................AGUU........GAC**G****G****U****G****G****G**.AGU**C**..**G****A**..**U****G****C****G****A****C****U****C****G**AUUC | |
|  |  | AACY01111288.1/617-741  | AAAA**G****U****U****A****A****U****G****C**.**A****U****C**G.AGA**U**G**A****C****A****C**.**G****G****A****G****G****G**AU..**C****U****G**.**G**.**C****C****C**UUU**G****A****U**.**G****U****G****U**C**A****A****G****C**AG**C****C**.**U**.**U****U****U**--........................CAGC-....................................................--**C****U****G**.A...**A**..**G****G**U**G**..**C****U**AAA**G****C****C****A****G**...**U****C****U****C****U****U**C...................CUAA.......GUGG**G****A****G****G****G****A**.AU.**C**..**G****A**..**U****G****C****G****A****C****U****C****G**AAGG | |
|  |  | AACY01470187.1/42-170  | GCAC**U****A****U****U****A****U****G****U**.**A****U****C**A.AGA**U**G**A****C****A****C**.**U****G****A****G****G****G**AU..**C****U****G**.**G**.**C****C****C**UUU**G****A****A**.**G****U****G****U**C**G****A****G****C**AG**C****C**.**U**.**U****U****U**--........................CAGC-....................................................--**G****U****A**.U...**A**..**G****G**U**G**..**C****U**AAU**G****C****C****A****G**...**C****C****C****A****A****U**CGGC................UAAU.......UGUC**G****G****U****G****G****G**.AGU**C**..**G****A**..**U****G****C****G****A****C****U****C****G**AUUC | |
|  |  | AACY01379654.1/207-340  | UUUG**C****C****U****U****G****C****U****U**.**A****U****C**A.AGA**A**.**A****G****A****C**.**C****G****A****G****G****G**A...**U****U****G**.**G**G**C****C****C**GCU**G****A****C**.**G****U****C****U**.**U****A****G****C**AA**C****C**.-.**U****U****C****U****G**GUAGG...................AUUUC.............................CGAGCAAAGCUCGGCAACCUCCC**C****G****G****U****U**.U...**C**..**G****G**U**G**..**C****U**ACC**U****C****C****C****A**...------....................----...........------.CC.**A**..**G****A**..**U****G****A****G****C****U****A****A****U**CUCU | |
|  |  | AAFZ01007634.1/875-1007  | UCAU**G****C****A****U****U****C****U****C**.**C****A****U**C.AGA**A**.**C****G****G****U**.**G****G****A****G****G****C**AU..**C****A****G**.**G**U**C****C****U**UUU**G****A****A**.**G****C****C****U**.**U****G****G****C**AA**A****C**.-.--**A****U****C**UGGUACUU................AUUAA..................................................GU**G****C****U****A****A**.A...**A**..**G****G**U**G**..-**U**ACA**U****C****C****U****G**...**C****U****G****G****U****A**CA..................AUAU..........U**U****G****U****C****U****G**.AA.**A**..**A****G**..**U****G****A****U****U****C****A****A****G**AGUU | |
|  |  | SS\_cons |  | ....<<<<<<<<.<<<.....<.<<<<.[[[<<<....AAA.A.>>>...]]].>>>>.><<<..<<.<.<<<<<.................................................................................>>>>>.....>..>>.>..>>...<aaaa...<<<<<<...................................>>>>>>....>..>>..>>>>>>>>>.... |
|  |  | SS\_label |  | ....=====P1=====.....--P2--....P2a....=P2b=.P2a.......--P2--=======P3======.................................................................................=========P3==========....P2b=...--P4--...................................--P4--.......======P1=====.... |
|  |  | predicted\_pair\_1 |  | ............................[.......................].............................................................................................................................................................................................................. |
|  |  | predicted\_pair\_2 |  | ....................................................................................................................................................................................<..........................................................>................... |
|  |  | RF |  | auaaaaucUCUU.AUCa.AGAG.AGGu.GGAGGGA...CUG.G.CCCuAUGAA.aCCU.CaGCAACC.g.gccua........................uUuaa....................................................uaggu.A...c..GGUG..CuAAUUCCuG...CAaaac....................auaa...........guuuUG.AA.A..GA..UAAGAgaaggauu |
|  |  | SS\_align |  | ::::((((((((.(((,.,,,<.<<<<.---<<<\_...\_\_\_.\_.>>>------.>>>>.><<<<-<<.<.<<<<<........................\_\_\_\_\_....................................................>>>>>.-...>..>>>>..>>,,,,,,,,...<<<<<<....................\_\_\_\_...........>>>>>>.,,.,..))..))))))))):::: |
