# Supplementary material for: The distributions, mechanisms, and structures of metabolite-binding riboswitches
Source: Genome Biol. 2007 Nov 12;8(11):R239. doi: 10.1186/gb-2007-8-11-r239 (PMC2258182; doi:10.1186/gb-2007-8-11-r239)
Supplement: Additional data file 2 — Sequence alignments of the riboswitch aptamer data sets annotated with new base-base interactions in HTML format. [file gb-2007-8-11-r239-S2.zip › HTML/SAM-II.html]

|  |  |  |  |  |
| --- | --- | --- | --- | --- |
|  |  | **Accession/Start-End** |  | **Sequence** |
|  |  | NC\_004463.1/1219779-1219703  | UCACUUAUCC**C****G****U****G****G****U**...GAUUU**G**AGCC........**G****G****C****C**.G**G**CUU**G****C****.****A****G****C****C****A****C****G**..UUAAAUAAG**U****C**.**G****C**UAAACA..**G****G****C****C**GGGGACCUCU | |
|  |  | NC\_003047.1/580190-580115  | AGCUUUAUCC**C****G****U****G****G****U**...GAUUU**G**GCC.........**G****G****U****C**.G**G**CUU**G****C****.****A****G****C****C****A****C****G**..UUAAACAAG**U****A**.**G****C**UAAAAA..**G****G****C****C**GGGUGUCAAA | |
|  |  | NC\_003062.1/429234-429159  | AAGCUUAUCC**C****G****U****G****G****U**...GAUUU**G**GCC.........**G****G****U****C**.G**G**CUU**G****C****.****A****G****C****C****A****C****G**..UUAAACAAG**U****G**.**G****C**UAAAAA..**G****A****C****C**GGGUGCUAGG | |
|  |  | NC\_003304.1/429434-429359  | AAGCUUAUCC**C****G****U****G****G****U**...GAUUU**G**GCC.........**G****G****U****C**.G**G**CUU**G****C****.****A****G****C****C****A****C****G**..UUAAACAAG**U****G**.**G****C**UAAAAA..**G****A****C****C**GGGUGCUAGG | |
|  |  | NZ\_AAED02000008.1/69660-69735  | ACUUUAUCCC**C****G****U****G****G****U**...GAUUU**G**GCC.........**G****G****U****C**.G**G**CUU**G****C****.****A****G****C****C****A****C****G**..UUAAAGAAG**U****C**.**G****C**UAAAAA..**G****G****C****C**GUGCUCGAAG | |
|  |  | NC\_003047.1/3461765-3461839  | UCAAAUGAUC**C****G****U****G****G****U**...GAUUU**G**GCC.........**G****G****C****C**.G**G**CUU**G****C****.****A****G****C****C****A****C****G**..UUAAAGAAG**U****C**.**G****C**UAAAG...**G****G****C****C**GAGGGAACAA | |
|  |  | NZ\_AAIS01000001.1/1100106-1100182  | UGACCUGUCC**C****G****U****G****G****U**...GAUUU**G**AGCC........**G****G****C****C**.G**G**CUU**G****C****.****A****G****C****C****A****C****G**..UUAAAUAAG**U****U**.**G****C**UAAACA..**G****G****C****C**GGGGACAAUG | |
|  |  | NC\_005296.1/5380768-5380844  | CGACUUAUCC**C****G****U****G****G****U**...GAUUU**G**AGCC........**G****G****C****C**.G**G**CUU**G****C****.****A****G****C****C****A****C****G**..UUAAACAAG**U****C**.**G****C**UAAACA..**G****G****C****C**GGGGACGUUG | |
|  |  | NC\_003317.1/1664139-1664215  | AAACCUGUUC**C****G****U****G****G****U**...GAUUU**G**GCC.........**G****G****C****C**.G**G**CUU**G****C****.****A****G****C****C****A****C****G**..UUAAAGAAU**U****C**.**G****C**UAAAUAA.**G****G****C****C**GCGGUUCGUU | |
|  |  | NC\_004310.3/318161-318085  | AAACCUGUUC**C****G****U****G****G****U**...GAUUU**G**GCC.........**G****G****C****C**.G**G**CUU**G****C****.****A****G****C****C****A****C****G**..UUAAAGAAU**U****C**.**G****C**UAAAUAA.**G****G****C****C**GCGGUUCGUU | |
|  |  | NC\_006932.1/340054-339978  | AAACCUGUUC**C****G****U****G****G****U**...GAUUU**G**GCC.........**G****G****C****C**.G**G**CUU**G****C****.****A****G****C****C****A****C****G**..UUAAAGAAU**U****C**.**G****C**UAAAUAA.**G****G****C****C**GCGGUUCGUU | |
|  |  | NC\_003062.1/2703249-2703322  | GAAACGCCUC**A****G****U****G****G****U**...GAUUU**G**CC..........**G****A****C****C**.G**G**CUU**G****C****.****A****G****C****C****A****C****U**..UUAAAGAAG**U****C**.**G****C**UAAAG...**G****G****U****C**GAGGAAAAGG | |
|  |  | NC\_003304.1/2703360-2703433  | GAAACGCCUC**A****G****U****G****G****U**...GAUUU**G**CC..........**G****A****C****C**.G**G**CUU**G****C****.****A****G****C****C****A****C****U**..UUAAAGAAG**U****C**.**G****C**UAAAG...**G****G****U****C**GAGGAAAAGG | |
|  |  | NC\_004463.1/1515208-1515284  | UUGCCUGUUC**C****G****U****G****G****U**...CAUUU**G**AGCC........**G****G****C****C**.G**G**CUU**G****C****.****A****G****C****C****A****C****G**..UUAAAAAAC**U****C**.**G****C**UAAACA..**G****G****C****C**GGGGACGCUU | |
|  |  | NZ\_AAED02000001.1/195270-195344  | CAGGCGAACC**C****G****U****G****G****U**...GAUUU**G**GCC.........**G****G****U****C**.G**G**CUU**G****C****.****A****G****C****C****A****C****G**..UAAAAGAAG**U****C**.**G****C**UAAAA...**G****G****C****C**GUGGAUACCA | |
|  |  | NZ\_AAAG02000001.1/970178-970105  | GCCCCAGAUU**C****G****U****G****G****G**...GAUUU**G**A...........**G****A****C****C**.G**G**CUU**G****C****.****G****C****C****C****A****C****G**..UUAAACAAA**C****A**.**G****C**UAAAAG..**G****G****U****C**GUGUGACGAA | |
|  |  | NZ\_AAAP01003864.1/43187-43112  | UGUCUUGGUC**C****G****U****G****G****U**...GAUUU**G**UCC.........**G****A****C****C**.G**G**AUU**G****C****.****G****G****C****C****A****C****G**..UUAAACAUC**C****C**.**G****C**UAAAGA..**G****G****U****C**CGAGCGCUCC | |
|  |  | NC\_005296.1/5009666-5009590  | AACCAGAACU**C****G****U****G****G****U**...CAUUU**G**AGCC........**G****G****C****C**.G**G**CUU**G****C****.****A****G****C****C****A****C****G**..UUAAACAAC**U****C**.**G****C**UAAACA..**G****G****C****C**GGGGACAGUU | |
|  |  | NC\_002678.2/4543370-4543296  | GUCGUUAUCC**C****G****U****G****G****U**...GAUUU**G**GCC.........**G****G****U****C**.G**G**CUU**G****C****.****A****G****C****C****A****C****G**..UUAAACAAG**U****C**.**G****C**UAAAG...**G****A****C****C**GUUGGCCGCG | |
|  |  | NC\_002678.2/2853662-2853736  | GCAACAGUUC**C****G****U****G****G****U**...GAUUU**G**GCC.........**G****G****U****C**.G**G**CUU**G****C****.****A****G****C****C****A****C****G**..UUAAACAAU**U****C**.**G****C**UAAAG...**G****G****C****C**GUUUGCGUGA | |
|  |  | NZ\_AAIS01000002.1/212898-212822  | CCGAUCAUUC**C****G****U****G****G****U**...CAUUU**G**AGCC........**G****G****C****C**.G**G**CUU**G****C****.****A****G****C****C****A****C****G**..UUAAACAAC**U****C**.**G****C**UAAACA..**G****G****C****C**GGGGACAGCA | |
|  |  | NZ\_AAFG02000012.1/39305-39230  | AUCAAGACCC**C****G****U****G****G****C**...GAUUU**G**UUU.........**.****A****C****C**.G**G**AUU**G****C****G****G****G****C****C****A****C****G**..UAAAACAAG**C****C**.**G****C**UAAAGA..**G****G****U****C**AGGACGACGG | |
|  |  | NZ\_AAIG01000004.1/111515-111590  | AGCCAAGGGC**C****G****U****G****G****C**...GAUUU**G**UGA.........**.****A****C****C**.G**G**AUU**G****C****G****G****G****C****C****A****C****G**..UUAAACAUA**C****C**.**G****C**UAAAGA..**G****G****U****C**GAGGCCGCUG | |
|  |  | NC\_002927.3/4852882-4852805  | CACAUUGUUC**G****G****C****G****C****C**...GAUUU**G**CCU.........**G****A****U****C**.C**G**CUU**G****C****.****G****G****G****C****G****C****C**UCUUAUAAAUC**C****A**.**G****C**UAAAGA..**G****G****U****C**UGAAUGACCA | |
|  |  | NC\_002929.2/48077-48154  | CACAUUGUUC**G****G****C****G****C****C**...GAUUU**G**CCU.........**G****A****U****C**.C**G**CUU**G****C****.****G****G****G****C****G****C****C**UCUUAUAAAUC**C****A**.**G****C**UAAAGA..**G****G****U****C**UGAAUGACCA | |
|  |  | NC\_002928.3/4417695-4417618  | CACAUUGUUC**G****G****C****G****C****C**...GAUUU**G**CCU.........**G****A****U****C**.C**G**CUU**G****C****.****G****G****G****C****G****C****C**UCUUAUAAAUC**C****A**.**G****C**UAAAGA..**G****G****U****C**UGAAUGACCA | |
|  |  | NZ\_AAAE01000150.1/27828-27755  | GGGCAAAAGC**C****G****U****G****G****U**...GCUUU**G**U...........**.****G****C****C**.G**G**AUU**G****C****G****G****G****C****C****A****C****G**..UUAAAGAAA**C****C**.**G****C**UAAAGA..**G****G****C****G**AGGGCCCGCC | |
|  |  | NC\_004463.1/7516215-7516140  | GCGUUCGUCC**A****G****C****G****C****C**...GAUUU**G**AUC.........**.****C****U****C**.A**G**CUU**G****C****.****G****G****G****C****G****C****U**.UCUAAAAAUG**C****A**.**G****C**UAAAGA..**G****A****C****C**CGCUAUCCCU | |
|  |  | NC\_002971.2/1955256-1955185  | AUAUUAAAAA**C****A****U****G****C****C**...GAUUU**G**AUA.........**.****A****U****C**.A**G**CUU**G****C****.****G****G****G****C****A****U****.**...UAAAA.AA**C****A**.**G****C**UAAAGC..**G****G****U****G**CACUUAGUUA | |
|  |  | NC\_002971.2/99863-99938  | UGUAAGAUUU**U****G****C****G****C****C**...GAUUU**G**AGC.........**.****C****U****C**.A**G**CUU**G****C****.****G****G****G****C****G****C****A**.UCAAUAAAUA**C****A**.**G****C**UAAAGC..**G****G****G****A**GUGCAGAUCU | |
|  |  | NC\_006677.1/1829617-1829541  | CGUGCCUGAC**C****C****U****C****G****U**...GAUUU**G**AGC.........**G****G****C****C**.G**G**CUU**G****C****.****G****G****C****G****A****G****G**CUAAAGAACUG**C****.**.**G****C**UAAAGA..**G****G****C****C**CUGCGUCGUU | |
|  |  | NC\_003911.11/1410075-1410149  | UCAGGAACCA**C****G****U****G****G****C**...GAUUU**G**UU..........**.****A****C****C**.G**G**AUU**G****C****G****G****G****C****C****A****C****G**.CUAAACAUCA**C****C**.**G****C**UAAAA...**G****G****U****C**AGGAUGAAAG | |
|  |  | NZ\_AAFQ02000001.1/189270-189342  | UACUUGCCGC**A****G****C****G****C****C**...GAUUU**G**............**.****C****U****C**.A**G**CUU**G****C****.****G****G****G****C****G****C****U**..UAAAAAGUA**C****C**.**G****C**UAAAGAC.**G****A****A****C**AUCCAUGAAC | |
|  |  | NZ\_AAEM01000014.1/96194-96122  | UUCCGCCCAC**A****G****C****G****C****C**...GAUUU**G**A...........**.****U****C****C**.G**G**AUU**G****C****.****G****G****G****C****G****C****U**..CAACAAAUG**C****A**.**G****C**UAAAGA..**G****G****G****G**CCCUCGACCG | |
|  |  | NZ\_AADF01000001.1/1218407-1218335  | GCACUUCAGC**G****G****C****G****C****C**...GAUUU**G**A...........**G****C****U****C**.U**G**AUU**G****C****.****G****G****G****C****G****C****.**..UUAUAAAUA**C****A**.**G****C**UAAAAC..**G****G****G****A**ACCACCCAGU | |
|  |  | NZ\_AAFH01000003.1/273404-273480  | UUCGGCCGGA**C****G****C****G****C****C**...GAUUU**G**AA..........**U****C****C****C**.A**G**CUU**G****C****.****G****G****G****C****G****C****.**.UUGAAAAAUA**C****G**.**G****C**UAAAGCGA**G****G****C****G**ACGGAAACCC | |
|  |  | NZ\_AADX02000016.1/28968-29052  | UAUCAAUCCA**U****G****C****G****C****C**...GAUUU**G**AGGAGAAGUAGC**A****C****U****C**.A**G**CUU**G****C****.****G****G****G****C****G****C****A**..AUAUAAAUA**C****U**.**G****C**UAAAGC..**G****A****G****U**UGAUAUUGAU | |
|  |  | NC\_006513.1/1668391-1668465  | UUCGCCUCCA**C****G****C****G****C****C**...GAUUU**G**AA..........**C****C****A****C**.A**G**CUU**G****C****.****G****G****G****C****G****C****.**.UCAAUAAAUC**C****G**.**G****C**UAAAGC..**G****A****G****G**GCGACCCAGU | |
|  |  | NC\_005085.1/1718551-1718478  | CUAAUGUUCA**U****G****C****G****C****C**...GAUUU**G**A...........**.****C****A****C**.A**G**CUU**G****C****.****G****G****G****C****G****C****.**.UCGAUAAAUG**C****C**A**G****C**UAAAGC..**G****U****A****U**CCGGAUCAAC | |
|  |  | NZ\_AAAG02000001.1/82642-82568  | GGUCACACUA**C****G****C****G****C****C**...GAUUU**G**CU..........**C****C****U****C**.A**G**CUU**G****C****.****G****G****G****C****G****C****G**..CAACAAAUG**C****G**.**G****C**UAAAGC..**G****G****G****G**GGCGUCGGGU | |
|  |  | NC\_004663.1/4108971-4109044  | GAAAUGAAAG**U****G****U****G****G****.**ACAGAUUU**G**............**.****A****G****C**.A**G**CUU**G****C****.****A****A****C****C****A****C****G**..GAAAA.AAA**U****.**.**G****C**UAAAACAC**G****U****C****U**UGUUGACGCC | |
|  |  | NC\_003228.3/69588-69660  | UGAAAUGAAA**U****G****U****G****G****.**AAAGAUUU**G**............**.****A****G****U**.A**G**CUU**G****C****.****A****A****C****C****A****C****A**..GAAAA.AAA**U****.**.**G****C**UAAAACA.**A****C****A****U**UCCCUUCUGU | |
|  |  | NC\_006347.1/64055-64127  | UGAAAUGAAA**U****G****U****G****G****.**AAAGAUUU**G**............**.****A****G****U**.A**G**CUU**G****C****.****A****A****C****C****A****C****A**..GAAAA.AAA**U****.**.**G****C**UAAAACA.**A****C****A****U**UCCCUUCUGU | |
|  |  | NC\_002950.2/1994573-1994644  | AACCAAUCAG**C****G****U****G****G****.**AAGGAUUU**G**U...........**.****A****A****U**..**G**CUU**G****C****.****A****A****C****C****A****C****G**..AGAAA.AAA**U****.**.**G****C**UAAAAC..**A****U****U****U**CUUCUUAGAA | |
|  |  | NC\_006570.1/167445-167367  | UAAUAGCGCA**A****G****U****C****G****C**UG.UAUUU**A**AC..........**.****A****A****A**CA**A**CUU**G****C****U****U****G****C****G****A****C****A**..UUAAAUAAU**A****A**.**G****C**UAAAGC.A**U****U****U****C**UACCAAAUUU | |
|  |  | NZ\_AADF01000001.1/1281606-1281530  | AUAUUAUCCG**U****U****C****G****C****C**...GAGUU**A**AAC.........**A****G****A****C**.A**A**CUU**G****C****G****G****G****G****C****G****A****C**..UAAUAAAUU**C****U**.**G****C**UAAAGC..**G****U****C****A**CCUGCUCGUG | |
|  |  | NZ\_AAFQ02000002.1/263441-263518  | UAAUGCAAGG**G****U****C****G****C****C**...GAGUU**A**CU..........**G****A****A****C**.A**A**CUU**G****C****A****G****G****G****C****G****A****C**AUUAAACCAAA**C****U**.**G****C**UAAAGC..**G****U****U****C**GCCGAGGCAC | |
|  |  | AACY01634724.1/748-819  | AAAAUUUUUG**U****G****C****G****C****U**...GAUUU**A**............**.****A****C****U**.G**A**AUU**G****C****.****G****A****G****C****G****C****.**.UUUAAAAAAA**C****C**.**G****C**UAAAAA..**A****G****U****U**UUUUUCUCAC | |
|  |  | AACY01020306.1/1313-1384  | AAAUAAUAUU**U****G****C****G****C****U**...GAUUU**A**............**.****A****C****U**.G**A**AUU**G****C****.****G****A****G****C****G****C****.**.UAUAAAAAAA**C****C**.**G****C**UAAAAA..**A****G****U****U**UUUUUUACCU | |
|  |  | AACY01690877.1/295-223  | AAACAUUAUU**U****G****C****G****C****U**...GAUUU**A**............**A****A****C****U**.G**A**AUU**G****C****.****G****A****G****C****G****C****.**.UUAAAAAAAA**C****C**.**G****C**UAAAUA..**A****G****U****U**UUUUUUCUCC | |
|  |  | AACY01230350.1/532-461  | ACAAAAAUUA**U****G****C****G****C****U**...GAUUU**A**............**.****A****C****U**.G**A**AUU**G****C****.****G****A****G****C****G****C****.**.UUUAAAAAAA**C****C**.**G****C**UAAAAA..**A****G****U****U**UUUUUUCUCA | |
|  |  | AACY01250056.1/665-736  | ACAAAAUUUG**U****G****C****G****C****U**...GAUUU**A**............**.****A****C****U**.G**A**AUU**G****C****.****G****A****G****C****G****C****.**.UUUAAAAAAA**C****C**.**G****C**UAAAAA..**A****G****U****U**UUUUUUCUCA | |
|  |  | AACY01016228.1/577-649  | AAAUAUUACU**U****G****C****G****C****U**...GAUUU**A**............**A****A****C****U**.G**A**AUU**G****C****.****G****A****G****C****G****C****.**.UUAAAAAAAA**C****C**.**G****C**UAAAUA..**A****G****U****U**UUUUUUCUCC | |
|  |  | AACY01253705.1/33-104  | ACAAAAUUUA**U****G****C****G****C****U**...GAUUU**A**............**.****A****C****U**.G**A**AUU**G****C****.****G****A****G****C****G****C****.**.UUUAAAAAAA**C****C**.**G****C**UAAAGA..**A****G****U****U**UUUUUUCUCA | |
|  |  | AACY01751567.1/295-224  | AAAUAAUUUA**U****G****C****G****C****U**...GAUUU**A**............**.****A****C****U**.G**A**AUU**G****C****.****G****A****G****C****G****C****.**.UUUAAAAAAA**C****C**.**G****C**UAAAUA..**A****G****U****U**UUUUUACCUC | |
|  |  | AACY01278945.1/193-122  | ACAUAAAAAG**A****G****C****G****C****U**...GAUUU**A**............**.****A****C****U**.G**A**AUU**G****C****.****G****A****G****C****G****C****U**..UUAAAAAAA**C****C**.**G****C**UAAAUA..**A****G****U****U**UUUUUACCUC | |
|  |  | AACY01457158.1/852-781  | ACAUAAAAAG**A****G****C****G****C****U**...GAUUU**A**............**.****A****C****U**.G**A**AUU**G****C****.****G****A****G****C****G****C****U**..UUAAAAAAA**C****C**.**G****C**UAAAUA..**A****G****U****U**UUUUUACCUC | |
|  |  | AACY01569318.1/164-93  | ACAUAAAAAG**A****G****C****G****C****U**...GAUUU**A**............**.****A****C****U**.G**A**AUU**G****C****.****G****A****G****C****G****C****U**..UUAAAAAAA**C****C**.**G****C**UAAAUA..**A****G****U****U**UUUUUACCUC | |
|  |  | AACY01600729.1/120-191  | ACAUAAAAAG**A****G****C****G****C****U**...GAUUU**A**............**.****A****C****U**.G**A**AUU**G****C****.****G****A****G****C****G****C****U**..UUAAAAAAA**C****C**.**G****C**UAAAUA..**A****G****U****U**UUUUUACCUC | |
|  |  | AACY01634414.1/716-645  | ACAUAAAAAG**A****G****C****G****C****U**...GAUUU**A**............**.****A****C****U**.G**A**AUU**G****C****.****G****A****G****C****G****C****U**..UUAAAAAAA**C****C**.**G****C**UAAAUA..**A****G****U****U**UUUUUACCUC | |
|  |  | AACY01303516.1/372-301  | CAAAACUCAG**U****G****C****G****C****U**...GAUUU**A**............**.****A****C****U**.G**A**AUU**G****C****.****G****A****G****C****G****C****.**.UUUAAAAAAA**C****C**.**G****C**UAAAAA..**A****G****U****U**UUUUUCUCAC | |
|  |  | AACY01321748.1/123-51  | AAAAGCUUUU**U****G****C****G****C****U**...GAUUU**A**............**A****A****C****U**.G**A**AUU**G****C****.****G****A****G****C****G****C****.**.UUUAAAAAAC**C****U**.**G****C**UAAAAA..**A****G****U****U**UUUUACCUCA | |
|  |  | AACY01043753.1/215-286  | ACAAAAUUUA**U****G****C****G****C****U**...GAUUU**A**............**.****C****C****U**.G**A**AUU**G****C****.****G****A****G****C****G****C****.**.UUUAAAAAAA**C****C**.**G****C**UAAAAA..**A****G****U****U**UUUUUUCUCA | |
|  |  | AACY01049918.1/968-897  | ACAAAAUUUA**U****G****C****G****C****U**...GAUUU**A**............**.****C****C****U**.G**A**AUU**G****C****.****G****A****G****C****G****C****.**.UUUAAAAAAA**C****C**.**G****C**UAAAAA..**A****G****U****U**UUUUUUCUCA | |
|  |  | AACY01101458.1/5923-5852  | ACAAAAUUUA**U****G****C****G****C****U**...GAUUU**A**............**.****C****C****U**.G**A**AUU**G****C****.****G****A****G****C****G****C****.**.UUUAAAAAAA**C****C**.**G****C**UAAAAA..**A****G****U****U**UUUUUUCUCA | |
|  |  | AACY01128340.1/608-537  | ACAAAAUUUA**U****G****C****G****C****U**...GAUUU**A**............**.****C****C****U**.G**A**AUU**G****C****.****G****A****G****C****G****C****.**.UUUAAAAAAA**C****C**.**G****C**UAAAAA..**A****G****U****U**UUUUUUCUCA | |
|  |  | AACY01411038.1/144-215  | ACAAAAUUUA**U****G****C****G****C****U**...GAUUU**A**............**.****C****C****U**.G**A**AUU**G****C****.****G****A****G****C****G****C****.**.UUUAAAAAAA**C****C**.**G****C**UAAAAA..**A****G****U****U**UUUUUUCUCA | |
|  |  | AACY01525434.1/163-234  | ACAAAAUUUA**U****G****C****G****C****U**...GAUUU**A**............**.****C****C****U**.G**A**AUU**G****C****.****G****A****G****C****G****C****.**.UUUAAAAAAA**C****C**.**G****C**UAAAAA..**A****G****U****U**UUUUUUCUCA | |
|  |  | AACY01547195.1/159-230  | ACAAAAUUUA**U****G****C****G****C****U**...GAUUU**A**............**.****C****C****U**.G**A**AUU**G****C****.****G****A****G****C****G****C****.**.UUUAAAAAAA**C****C**.**G****C**UAAAAA..**A****G****U****U**UUUUUUCUCA | |
|  |  | AACY01549185.1/26-97  | ACAAAAUUUA**U****G****C****G****C****U**...GAUUU**A**............**.****C****C****U**.G**A**AUU**G****C****.****G****A****G****C****G****C****.**.UUUAAAAAAA**C****C**.**G****C**UAAAAA..**A****G****U****U**UUUUUUCUCA | |
|  |  | AACY01267576.1/737-808  | UACAAAAUUC**U****G****C****G****C****U**...GAUUU**A**............**.****C****C****U**.G**A**AUU**G****C****.****G****A****G****C****G****C****U**..UUAAAAAAA**C****C**.**G****C**UAAAAA..**A****G****U****U**UUUUAUCUCA | |
|  |  | AACY01000458.1/1109-1038  | AAACAAUAUU**U****G****C****G****C****U**...GAUUU**A**............**A****A****C****U**.G**A**AUU**G****C****.****G****A****G****C****G****C****.**..UUAAAAAAA**C****C**.**G****C**UAAACA..**A****G****U****U**UUUUUCCUCC | |
|  |  | AACY01357550.1/480-552  | AAAUACUACU**U****G****C****G****C****U**...GAUUU**A**............**A****A****C****U**.G**A**AUU**G****C****.****G****A****G****C****G****C****.**.UUAAAAAAAA**C****C**.**G****C**UAAAUA..**A****G****U****U**UUUUUUCUCC | |
|  |  | AACY01789429.1/402-473  | ACAAAAUUUA**U****G****C****G****C****U**...GAUUU**A**............**.****C****C****U**.G**A**AUU**G****C****.****G****A****G****C****G****C****.**.UUUAAAAAAA**C****C**.**G****C**UAAAAA..**A****G****U****U**UUUUUUUCUC | |
|  |  | AACY01453671.1/551-480  | ACAAAAUUUG**U****G****C****G****C****U**...GAUUU**A**............**.****A****C****U**.G**A**AUU**G****C****.****G****A****G****C****G****C****.**.UUUAAAAAAA**C****A**.**G****C**UAAAAA..**A****G****U****U**UUUUUUCCUC | |
|  |  | AACY01619100.1/399-470  | AAAAAAAAUA**U****G****C****G****C****U**...GAUUU**A**............**.****A****C****U**.G**A**AUU**G****C****.****G****A****G****C****G****C****.**.UUAAAAAAAA**C****C**.**G****C**UAAACA..**A****G****U****U**UUUUUCUCAC | |
|  |  | AACY01372079.1/297-368  | ACAAAAAUUC**U****G****C****G****C****U**...GAUUU**A**............**.****A****C****U**.G**A**AUU**G****C****.****G****A****G****C****G****C****.**.UUUAAAAAAA**C****C**.**G****C**UAAAUA..**A****G****U****U**UUUUUUCUCA | |
|  |  | AACY01023421.1/538-467  | AAAUAAUUUA**U****G****C****G****C****U**...GAUUU**A**............**.****A****C****U**.G**A**AUU**G****C****.****G****A****G****C****G****C****.**.UAUAAAAAAA**C****C**.**G****C**UAAAUA..**A****G****U****U**UUUUUUACCU | |
|  |  | AACY01098502.1/378-449  | ACAAAAAUUA**U****G****C****G****C****U**...GAUUU**A**............**.****A****C****U**.G**A**AUU**G****C****.****G****A****G****C****G****C****.**.UUUAAAAAAA**C****A**.**G****C**UAAAAA..**A****G****U****U**UUUUUUUCUC | |
|  |  | AACY01309385.1/706-635  | ACAAAAUUUA**U****G****C****G****C****U**...GAUUU**A**............**.****A****C****U**.G**A**AUU**G****C****.****G****A****G****C****G****C****.**.UUUAAAAAAA**C****C**.**G****C**UAAAAA..**U****G****U****U**UUUUUUCUCA | |
|  |  | AACY01329816.1/133-62  | AAAUAAUUUA**U****G****C****G****C****U**...GAUUU**A**............**.****A****C****U**.G**A**AUU**G****C****.****G****A****G****C****G****C****.**.UAUAAAAAAA**C****C**.**G****C**UAAACA..**A****G****U****U**UUUUUACCUC | |
|  |  | AACY01556347.1/767-696  | AAACAUUUCU**U****G****C****G****C****U**...GAUUU**A**............**.****A****C****U**.G**A**AUU**G****C****.****G****A****G****C****G****C****.**.UAUAAAAAAA**C****C**.**G****C**UAAAUA..**A****G****U****U**UUUUUACCUC | |
|  |  | AACY01803609.1/388-459  | AAACAUUUCU**U****G****C****G****C****U**...GAUUU**A**............**.****A****C****U**.G**A**AUU**G****C****.****G****A****G****C****G****C****.**.UAUAAAAAAA**C****C**.**G****C**UAAAUA..**A****G****U****U**UUUUUACCUC | |
|  |  | AACY01035837.1/1123-1194  | AAAUAAUUUA**U****G****C****G****C****U**...GAUUU**A**............**.****A****C****U**.G**A**AUU**G****C****.****G****A****G****C****G****C****.**.UAUAAAAAAA**C****C**.**G****C**UAAAUA..**A****G****U****U**UUUUUACCUC | |
|  |  | AACY01090575.1/963-892  | AAAUAAUUUA**U****G****C****G****C****U**...GAUUU**A**............**.****A****C****U**.G**A**AUU**G****C****.****G****A****G****C****G****C****.**.UAUAAAAAAA**C****C**.**G****C**UAAAUA..**A****G****U****U**UUUUUACCUC | |
|  |  | AACY01187448.1/143-72  | AAAUAAUUUA**U****G****C****G****C****U**...GAUUU**A**............**.****A****C****U**.G**A**AUU**G****C****.****G****A****G****C****G****C****.**.UAUAAAAAAA**C****C**.**G****C**UAAAUA..**A****G****U****U**UUUUUACCUC | |
|  |  | AACY01401139.1/473-402  | AAAAGAUUUU**U****G****C****G****C****U**...GAUUU**A**............**.****A****C****U**.G**A**AUU**G****C****.****G****A****G****C****G****C****.**.UUUAAAAAAA**C****C**.**G****C**UAAAUA..**A****G****U****U**UUUUUUCCUC | |
|  |  | AACY01403391.1/151-80  | AAAUAAUUUA**U****G****C****G****C****U**...GAUUU**A**............**.****A****C****U**.G**A**AUU**G****C****.****G****A****G****C****G****C****.**.UAUAAAAAAA**C****C**.**G****C**UAAAUA..**A****G****U****U**UUUUUACCUC | |
|  |  | AACY01216447.1/336-264  | AAAAGUUUCC**U****G****C****G****C****U**...GAUUU**A**............**A****A****C****U**.G**A**AUU**G****C****.****G****A****G****C****G****C****.**.UUUAAAAAAC**C****U**.**G****C**UAAACA..**A****G****U****U**UUUUUACCUC | |
|  |  | AACY01078968.1/463-391  | AGACAUAACU**U****G****C****G****C****U**...GAUUU**A**............**A****A****C****U**.G**A**AUU**G****C****.****G****A****G****C****G****C****.**.UUAAAAAAAA**C****C**.**G****C**UAAAUA..**A****G****U****U**UUUUUUCUCC | |
|  |  | AACY01103224.1/794-723  | AAACAUUUAU**U****G****C****G****C****U**...GAUUU**A**............**.****A****C****U**.G**A**AUU**G****C****.****G****A****G****C****G****C****.**.UAUAAAAAAA**C****C**.**G****C**UAAAUA..**A****G****U****U**UUUUUUCCUC | |
|  |  | AACY01352090.1/439-368  | AAAUGAUUUA**U****G****C****G****C****U**...GAUUU**A**............**.****A****C****U**.G**A**AUU**G****C****.****G****A****G****C****G****C****.**.UAUAAAAAAA**C****C**.**G****C**UAAAAA..**A****G****U****U**UUUUUUCCUC | |
|  |  | AACY01006340.1/302-231  | ACAAAAAAAA**C****G****C****G****C****U**...GAUUU**A**............**.****A****C****U**.G**A**AUU**G****C****.****G****A****G****C****G****C****.**.UUAAAAAAAA**C****C**.**G****C**UAAAUA..**A****G****U****U**UUUUUCUCAC | |
|  |  | AACY01552650.1/556-627  | AAAAAAUUAA**U****G****C****G****C****U**...GAUUU**A**............**.****U****C****U**.G**A**AUU**G****C****.****G****A****G****C****G****C****.**.UUUAAAAAAA**C****C**.**G****C**UAAAUA..**A****G****U****U**UUUUUACCUC | |
|  |  | AACY01557027.1/302-373  | AAAUAAUUUA**U****G****C****G****C****U**...GAUUU**A**............**.****A****C****U**.G**A**AUU**G****C****.****G****A****G****C****G****C****.**.UAUAAAAAAA**C****C**.**G****C**UAAACA..**A****G****U****U**UUUUUGCCUC | |
|  |  | AACY01771905.1/714-786  | AAAAUGUUUA**U****G****C****G****C****U**...GAUUU**A**............**A****A****C****U**.G**A**AUU**G****C****.****G****A****G****C****G****C****U**..UUAAAAAAC**C****U**.**G****C**UAAAUA..**A****G****U****U**UUUUGCCUCA | |
|  |  | AACY01696123.1/51-123  | AAAAGUUUUC**U****G****C****G****C****U**...GAUUU**A**............**A****A****C****U**.G**A**AUU**G****C****.****G****A****G****C****G****C****.**.UAUAAAAAAC**C****U**.**G****C**UAAACA..**A****G****U****U**UUUUUACCUC | |
|  |  | AACY01152763.1/713-784  | AAAUAAUUUA**U****G****C****G****C****U**...GAUUU**A**............**.****A****C****U**.G**A**AUU**G****C****.****G****A****G****C****G****C****.**.UAUAAAAAAA**C****C**.**G****C**UAAAUA..**A****G****U****U**UUUUUUAUCU | |
|  |  | AACY01024941.1/302-231  | AAACAAUAUU**U****G****C****G****C****U**...GAUUU**A**............**.****A****C****U**.G**A**AUU**G****C****.****G****A****G****C****G****C****.**.UAUAAAAAAA**C****C**.**G****C**UAAAAU..**A****G****U****U**UUUUUACCUC | |
|  |  | AACY01019662.1/983-1055  | AAAACGUUUA**U****G****C****G****C****U**...GAUUU**A**............**A****A****C****U**.G**A**AUU**G****C****.****G****A****G****C****G****C****U**..UUAAAAAAC**C****U**.**G****C**UAAACA..**A****G****U****U**UUUUACCUCA | |
|  |  | AACY01634508.1/636-565  | AAAUAAUUUA**U****G****C****G****C****U**...GAUUU**A**............**.****A****C****U**.G**A**AUU**G****C****.****G****A****G****C****G****C****.**.UAUAAAAAAA**C****C**.**G****C**UAAAUA..**A****G****U****U**UUUUUGCCUC | |
|  |  | AACY01759351.1/647-576  | AAAAAAUUUA**U****G****C****G****C****U**...GAUUU**A**............**.****A****C****U**.G**A**AUU**G****C****.****G****A****G****C****G****C****.**.UUAAAAAAAA**C****C**.**G****C**UAAAAU..**A****G****U****U**UUUUUUCUCA | |
|  |  | AACY01151208.1/524-595  | AAAAAAAAUA**U****G****C****G****C****U**...GAUUU**A**............**.****A****C****U**.G**A**AUU**G****C****.****G****A****G****C****G****C****.**.UUAAAAAAAA**C****C**.**G****C**UAAAAU..**A****G****U****U**UUUUUUCUCA | |
|  |  | AACY01287505.1/389-318  | AAAAAAAAUA**U****G****C****G****C****U**...GAUUU**A**............**.****A****C****U**.G**A**AUU**G****C****.****G****A****G****C****G****C****.**.UUAAAAAAAA**C****C**.**G****C**UAAAAU..**A****G****U****U**UUUUUUCUCA | |
|  |  | AACY01710010.1/269-340  | ACAAAAUUUC**U****G****C****G****C****U**...GAUUU**A**............**.****U****C****U**.G**A**AUU**G****C****.****G****A****G****C****G****C****.**.UAUAAAAAAA**C****C**.**G****C**UAAAAA..**A****G****U****U**UUUUUCUCAC | |
|  |  | AACY01038093.1/570-641  | ACAAAAUUUA**C****G****C****G****C****U**...GAUUU**A**............**.****A****C****U**.G**A**AUU**G****C****.****G****A****G****C****G****C****.**.UUAAAAAAAA**C****C**.**G****C**UAAAAU..**A****G****U****U**UUUUUUCUCA | |
|  |  | AACY01409674.1/452-523  | ACAAAAAAAG**C****G****C****G****C****U**...GAUUU**A**............**.****A****C****U**.G**A**AUU**G****C****.****G****A****G****C****G****C****.**.UUAAAAAAAA**C****C**.**G****C**UAAAUA..**A****G****U****U**UUUUUCCUCA | |
|  |  | AACY01103076.1/1426-1355  | AAAAGAAUUC**U****G****C****G****C****U**...GAUUU**A**............**.****A****C****U**.G**A**AUU**G****C****.****G****A****G****C****G****C****.**.UAUAAAAAAA**C****C**.**G****C**UAAAUA..**A****G****U****U**UUUUUACCUC | |
|  |  | AACY01288676.1/93-164  | AAAAAAAUUA**U****G****C****G****C****U**...GAUUU**A**............**.****U****C****U**.G**A**AUU**G****C****.****G****A****G****C****G****C****A**..UAAAAAAAA**C****C**.**G****C**UAAAAU..**A****G****U****U**UUUUUUCUCA | |
|  |  | AACY01612046.1/164-92  | AAAAGUUUUU**U****G****C****G****C****U**...GAUUU**A**............**A****A****C****U**.G**A**AUU**G****C****.****G****A****G****C****G****C****.**.UAUAAAAAAC**C****U**.**G****C**UAAACA..**A****G****U****U**UUUUUCCUCG | |
|  |  | AACY01595931.1/61-133  | ACAAAAUUUA**U****G****C****G****C****U**...GAUUU**A**............**.****C****C****U**.G**A**AUU**G****C****.****G****A****G****C****G****C****.**.UUUAAAAAAA**C****C**.**G****C**UAAAAAA.**A****G****U****U**UUUUUUCUCA | |
|  |  | AACY01095680.1/2003-1931  | AAAACCUUUU**U****G****C****G****C****U**...GAUUU**A**............**A****A****C****U**.G**A**AUU**G****C****.****G****A****G****C****G****C****.**.UAUAAAAAAC**C****U**.**G****C**UAAACA..**A****G****U****U**UUUUGCCUCA | |
|  |  | AACY01347653.1/528-600  | AAAACCUUUU**U****G****C****G****C****U**...GAUUU**A**............**A****A****C****U**.G**A**AUU**G****C****.****G****A****G****C****G****C****.**.UAUAAAAAAC**C****U**.**G****C**UAAACA..**A****G****U****U**UUUUGCCUCA | |
|  |  | AACY01135886.1/289-217  | AAGAACAACU**U****G****C****G****C****U**...GAUUU**A**............**A****A****C****U**.G**A**AUU**G****C****.****G****A****G****C****G****C****.**.UUAAAAAAAA**C****C**.**G****C**UAAAUA..**A****G****U****U**UUUUUUCUCC | |
|  |  | AACY01015025.1/1610-1539  | AAAAAAAUUA**U****G****C****G****C****U**...GAUUU**A**............**.****A****C****U**.G**A**AUU**G****C****.****G****A****G****C****G****C****.**.UUAAAAAAAA**C****C**.**G****C**UAAAAU..**A****G****U****U**UUUUUUCUCA | |
|  |  | AACY01139406.1/681-610  | AAAAAAAUUA**U****G****C****G****C****U**...GAUUU**A**............**.****A****C****U**.G**A**AUU**G****C****.****G****A****G****C****G****C****.**.UUAAAAAAAA**C****C**.**G****C**UAAAAU..**A****G****U****U**UUUUUUCUCA | |
|  |  | AACY01186057.1/452-381  | AAAAAAAUUA**U****G****C****G****C****U**...GAUUU**A**............**.****A****C****U**.G**A**AUU**G****C****.****G****A****G****C****G****C****.**.UUAAAAAAAA**C****C**.**G****C**UAAAAU..**A****G****U****U**UUUUUUCUCA | |
|  |  | AACY01222687.1/727-656  | AAAAAAAUUA**U****G****C****G****C****U**...GAUUU**A**............**.****A****C****U**.G**A**AUU**G****C****.****G****A****G****C****G****C****.**.UUAAAAAAAA**C****C**.**G****C**UAAAAU..**A****G****U****U**UUUUUUCUCA | |
|  |  | AACY01252690.1/347-418  | AAAAAAAUUA**U****G****C****G****C****U**...GAUUU**A**............**.****A****C****U**.G**A**AUU**G****C****.****G****A****G****C****G****C****.**.UUAAAAAAAA**C****C**.**G****C**UAAAAU..**A****G****U****U**UUUUUUCUCA | |
|  |  | AACY01261492.1/258-329  | AAAAAAAUUA**U****G****C****G****C****U**...GAUUU**A**............**.****A****C****U**.G**A**AUU**G****C****.****G****A****G****C****G****C****.**.UUAAAAAAAA**C****C**.**G****C**UAAAAU..**A****G****U****U**UUUUUUCUCA | |
|  |  | AACY01307878.1/393-464  | AAAAAAAUUA**U****G****C****G****C****U**...GAUUU**A**............**.****A****C****U**.G**A**AUU**G****C****.****G****A****G****C****G****C****.**.UUAAAAAAAA**C****C**.**G****C**UAAAAU..**A****G****U****U**UUUUUUCUCA | |
|  |  | AACY01311740.1/446-375  | AAAAAAAUUA**U****G****C****G****C****U**...GAUUU**A**............**.****A****C****U**.G**A**AUU**G****C****.****G****A****G****C****G****C****.**.UUAAAAAAAA**C****C**.**G****C**UAAAAU..**A****G****U****U**UUUUUUCUCA | |
|  |  | AACY01325303.1/446-375  | AAAAAAAUUA**U****G****C****G****C****U**...GAUUU**A**............**.****A****C****U**.G**A**AUU**G****C****.****G****A****G****C****G****C****.**.UUAAAAAAAA**C****C**.**G****C**UAAAAU..**A****G****U****U**UUUUUUCUCA | |
|  |  | AACY01381678.1/518-447  | AAAAAAAUUA**U****G****C****G****C****U**...GAUUU**A**............**.****A****C****U**.G**A**AUU**G****C****.****G****A****G****C****G****C****.**.UUAAAAAAAA**C****C**.**G****C**UAAAAU..**A****G****U****U**UUUUUUCUCA | |
|  |  | AACY01431937.1/505-434  | AAAAAAAUUA**U****G****C****G****C****U**...GAUUU**A**............**.****A****C****U**.G**A**AUU**G****C****.****G****A****G****C****G****C****.**.UUAAAAAAAA**C****C**.**G****C**UAAAAU..**A****G****U****U**UUUUUUCUCA | |
|  |  | AACY01656529.1/297-226  | AAAAAAAUUA**U****G****C****G****C****U**...GAUUU**A**............**.****A****C****U**.G**A**AUU**G****C****.****G****A****G****C****G****C****.**.UUAAAAAAAA**C****C**.**G****C**UAAAAU..**A****G****U****U**UUUUUUCUCA | |
|  |  | AACY01674707.1/58-129  | AAAAAAAUUA**U****G****C****G****C****U**...GAUUU**A**............**.****A****C****U**.G**A**AUU**G****C****.****G****A****G****C****G****C****.**.UUAAAAAAAA**C****C**.**G****C**UAAAAU..**A****G****U****U**UUUUUUCUCA | |
|  |  | AACY01751701.1/424-353  | AAAAAAAUUA**U****G****C****G****C****U**...GAUUU**A**............**.****A****C****U**.G**A**AUU**G****C****.****G****A****G****C****G****C****.**.UUAAAAAAAA**C****C**.**G****C**UAAAAU..**A****G****U****U**UUUUUUCUCA | |
|  |  | AACY01764037.1/482-411  | AAAAAAAUUA**U****G****C****G****C****U**...GAUUU**A**............**.****A****C****U**.G**A**AUU**G****C****.****G****A****G****C****G****C****.**.UUAAAAAAAA**C****C**.**G****C**UAAAAU..**A****G****U****U**UUUUUUCUCA | |
|  |  | AACY01803750.1/456-385  | AAAAAAAUUA**U****G****C****G****C****U**...GAUUU**A**............**.****A****C****U**.G**A**AUU**G****C****.****G****A****G****C****G****C****.**.UUAAAAAAAA**C****C**.**G****C**UAAAAU..**A****G****U****U**UUUUUUCUCA | |
|  |  | AACY01647873.1/746-818  | AAAAGCUUCU**U****G****C****G****C****U**...GAUUU**A**............**A****A****C****U**.G**A**AUU**G****C****.****G****A****G****C****G****C****.**.UAUAAAAAAA**C****U**.**G****C**UAAACA..**A****G****U****U**UUUUACCUCA | |
|  |  | AACY01806452.1/87-159  | CUAUUUAAAU**A****G****C****G****C****U**...GAUUU**A**A...........**.****G****U****C**.G**A**AUU**G****C****.****G****G****G****C****G****C****U**..UAAAAAAAC**C****U**.**G****C**UAAAUA..**G****A****U****G**UUUCUAGUGA | |
|  |  | AACY01017409.1/3149-3078  | AAAAGAAUUC**U****G****C****G****C****U**...GAUUU**A**............**.****A****C****U**.G**A**AUU**G****C****.****G****A****G****C****G****C****.**.UAUAAAAAAA**C****C**.**G****C**UAAAUA..**A****G****U****U**UUUUUUCCUC | |
|  |  | AACY01693958.1/435-507  | AUUAUUUACA**A****G****C****G****C****U**...GAUUU**A**A...........**.****A****U****C**.G**A**AUU**G****C****A****.****G****G****C****G****C****U**..AUAAAAAAC**C****U**.**G****C**UAAAUA..**G****A****U****G**UUUCUUGUGA | |
|  |  | AACY01069488.1/656-584  | ACAAAAAUUA**U****G****C****G****C****U**...GAUUU**A**............**.****A****C****U**.G**A**AUU**G****C****.****G****A****G****C****G****C****.**.UUAAAAAAAA**C****C**.**G****C**UAAAAAU.**A****G****U****U**UUUUUUCUCA | |
|  |  | AACY01750731.1/534-606  | GUUAUUUACA**A****G****C****G****C****U**...GAUUU**A**A...........**.****G****U****C**.A**A**AUU**G****C****.****G****G****G****C****G****C****U**..UUAAAAAAC**C****U**.**G****C**UAAAUA..**G****A****U****G**UUUCCUAUGA | |
|  |  | AACY01759550.1/203-132  | AAAAGCUUUU**U****G****C****G****C****U**...GAUUU**A**............**.****A****C****U**.G**A**AUU**G****C****.****G****A****G****C****G****C****.**.UUUAAAAAAC**C****U**.**G****C**UAAAUA..**A****G****U****U**UUUUACCUCA | |
|  |  | AACY01093006.1/89-160  | ACAAAAUUUA**U****G****C****G****C****U**...GAUUU**A**............**.****A****C****U**.G**A**AUU**G****C****.****G****A****G****C****G****C****.**.UUAAAAAAAA**C****C**.**G****C**UAAAAU..**A****G****U****U**UUUUUUCUCA | |
|  |  | AACY01475878.1/463-534  | AAGUAAUUUA**U****G****C****G****C****U**...GAUUU**A**............**.****A****C****U**.G**A**AUU**G****C****.****G****A****G****C****G****C****U**..AUAAAAAAA**C****C**.**G****C**UAAAUA..**A****G****U****U**UUUUUACCUC | |
|  |  | AACY01026421.1/107-178  | ACAAAAUUUA**U****G****C****G****C****U**...GAUUU**A**............**.****C****C****U**.G**A**AUU**G****C****.****G****A****G****C****G****C****.**.UCUAAAAAAA**C****C**.**G****C**UAAAAA..**A****G****U****U**UUUUUUCUCA | |
|  |  | AACY01076684.1/1377-1448  | ACAAAAAAUA**U****G****C****G****C****U**...GAUUU**A**............**.****A****C****U**.G**A**AUU**G****C****.****G****A****G****C****G****C****.**.UUAAAAAAAA**C****C**.**G****C**UAAAAU..**A****G****U****U**UUUUUUCUCA | |
|  |  | AACY01121792.1/126-55  | ACAAAAAAUA**U****G****C****G****C****U**...GAUUU**A**............**.****A****C****U**.G**A**AUU**G****C****.****G****A****G****C****G****C****.**.UUAAAAAAAA**C****C**.**G****C**UAAAAU..**A****G****U****U**UUUUUUCUCA | |
|  |  | AACY01270769.1/600-529  | ACAAAAAAUA**U****G****C****G****C****U**...GAUUU**A**............**.****A****C****U**.G**A**AUU**G****C****.****G****A****G****C****G****C****.**.UUAAAAAAAA**C****C**.**G****C**UAAAAU..**A****G****U****U**UUUUUUCUCA | |
|  |  | AACY01544803.1/247-318  | AAAAAAAAUA**U****G****C****G****C****U**...GAUUU**A**............**.****U****C****U**.G**A**AUU**G****C****.****G****A****G****C****G****C****.**.UUAAAAAAAA**C****C**.**G****C**UAAAUA..**A****G****U****U**UUUUUCUCAC | |
|  |  | AACY01133972.1/673-600  | AAAAGUUCAA**U****G****C****G****C****U**...GAUUU**A**............**A****A****C****U**.G**A**AUU**G****C****.****G****A****G****C****G****C****.**.UAUAAAAAAA**C****C**U**G****C**UAAACA..**A****G****U****U**UUUUGCCUCA | |
|  |  | AACY01356025.1/786-714  | GUUAUUUACA**A****G****C****G****C****U**...GAUUU**A**A...........**.****A****U****C**.G**A**AUU**G****C****.****G****G****G****C****G****C****U**..AUAAAAAAC**C****U**.**G****C**UAAAUA..**G****A****U****G**UUUCUAGUGA | |
|  |  | AACY01299846.1/161-90  | AAAAAAAUUA**U****G****C****G****C****U**...GAUUU**A**............**.****A****C****U**.G**A**AUU**G****C****.****G****A****G****C****G****C****.**.UUAAAAAAAA**C****C**.**G****C**UAAAAU..**A****G****U****U**UUUUUUUCUC | |
|  |  | AACY01233132.1/462-391  | AAAAAAAUUA**U****G****C****G****C****U**...GAUUU**A**............**.****A****C****U**.G**A**AUU**G****C****.****G****A****G****C****G****C****.**.UUAAAAAAAA**C****C**.**G****C**UAAAAU..**A****G****U****U**UUUUUUCUCU | |
|  |  | AACY01052136.1/1852-1781  | AAAAGCUUUU**U****G****C****G****C****U**...GAUUU**A**............**.****A****C****U**.G**A**AUU**G****C****.****G****A****G****C****G****C****.**.UUUAAAAAAC**C****U**.**G****C**UAAAUA..**A****G****U****U**UUUUUUCUCA | |
|  |  | AACY01057936.1/2947-2876  | AAAAGCUUUU**U****G****C****G****C****U**...GAUUU**A**............**.****A****C****U**.G**A**AUU**G****C****.****G****A****G****C****G****C****.**.UUUAAAAAAC**C****U**.**G****C**UAAAUA..**A****G****U****U**UUUUUUCUCA | |
|  |  | AACY01057937.1/461-390  | AAAAGCUUUU**U****G****C****G****C****U**...GAUUU**A**............**.****A****C****U**.G**A**AUU**G****C****.****G****A****G****C****G****C****.**.UUUAAAAAAC**C****U**.**G****C**UAAAUA..**A****G****U****U**UUUUUUCUCA | |
|  |  | AACY01411248.1/316-245  | AAAAGCUUUU**U****G****C****G****C****U**...GAUUU**A**............**.****A****C****U**.G**A**AUU**G****C****.****G****A****G****C****G****C****.**.UUUAAAAAAC**C****U**.**G****C**UAAAUA..**A****G****U****U**UUUUUUCUCA | |
|  |  | AACY01588110.1/38-109  | AAAAGCUUUU**U****G****C****G****C****U**...GAUUU**A**............**.****A****C****U**.G**A**AUU**G****C****.****G****A****G****C****G****C****.**.UUUAAAAAAC**C****U**.**G****C**UAAAUA..**A****G****U****U**UUUUUUCUCA | |
|  |  | AACY01473560.1/452-523  | AAAAGCUUUU**U****G****C****G****C****U**...GAUUU**A**............**.****A****C****U**.G**A**AUU**G****C****.****G****A****G****C****G****C****.**.UUUAAAAAAC**C****U**.**G****C**UAAAUA..**A****G****U****U**UUUUUCUCAC | |
|  |  | AACY01440642.1/413-342  | AAAUAUAAUU**U****G****C****G****C****U**...GAUUU**A**............**.****A****C****C**.A**U**AUU**G****C****.****A****A****G****C****G****C****A**..UAAUAAAUA**U****A**.**G****C**UAAAAA..**G****G****G****G**AUGCCCAGUU | |
|  |  | AACY01041938.1/982-1053  | AUUAAACGAA**U****G****C****G****C****U**...GAUUU**A**............**.****A****C****U**.G**A**AUU**G****C****.****G****A****G****C****G****C****.**.UAUAAAAAAA**C****C**.**G****C**UAAAUA..**A****G****U****U**UUUUUACCUC | |
|  |  | AACY01628642.1/707-778  | AUUAAACGAA**U****G****C****G****C****U**...GAUUU**A**............**.****A****C****U**.G**A**AUU**G****C****.****G****A****G****C****G****C****.**.UAUAAAAAAA**C****C**.**G****C**UAAAUA..**A****G****U****U**UUUUUACCUC | |
|  |  | AACY01076967.1/2589-2515  | AUACAUGAAU**U****G****C****G****C****C**...GAUUU**G**AG..........**.****C****A****C**.A**G**CUU**G****C****.****G****G****G****C****G****C****A**..AUAAAAAUG**C****A**.**G****C**UAAAGAG.**G****U****A****G**UUGAAACCGC | |
|  |  | AACY01142769.1/543-472  | ACAAAAUUUA**U****G****C****G****C****U**...GAUUU**A**............**.****A****C****U**.G**A**AUU**G****C****.****G****A****G****C****G****C****.**.UUAAAAAAAA**C****C**.**G****C**UAAAAU..**A****G****U****U**UUUUUUCUCU | |
|  |  | AACY01048433.1/2436-2364  | GUUAUUUACA**A****G****C****G****C****U**...GAUUU**A**A...........**.****G****U****C**.G**A**AUU**G****C****.****G****G****G****C****G****C****U**..AUAAAAAAC**C****U**.**G****C**UAAAUA..**G****A****U****G**UUUCAUGUGA | |
|  |  | AACY01070137.1/415-487  | GUUAUUUACA**A****G****C****G****C****U**...GAUUU**A**A...........**.****G****U****C**.G**A**AUU**G****C****.****G****G****G****C****G****C****U**..AUAAAAAAC**C****U**.**G****C**UAAAUA..**G****A****U****G**UUUCAUGUGA | |
|  |  | AACY01103401.1/2299-2228  | ACAAAAAUUA**U****G****C****G****C****U**...GAUUU**A**............**.****A****C****U**.G**A**AUU**G****C****.****G****A****G****C****G****C****.**.UUAAAAAAAA**C****C**.**G****C**UAAAAU..**A****G****U****U**UUUUUUCUCA | |
|  |  | AACY01195436.1/662-591  | ACAAAAAUUA**U****G****C****G****C****U**...GAUUU**A**............**.****A****C****U**.G**A**AUU**G****C****.****G****A****G****C****G****C****.**.UUAAAAAAAA**C****C**.**G****C**UAAAAU..**A****G****U****U**UUUUUUCUCA | |
|  |  | AACY01498054.1/304-232  | AUUAUUUACA**A****G****C****G****C****U**...GAUUU**A**A...........**.****G****U****C**.G**A**AUU**G****C****.****G****G****G****C****G****C****U**..CUAAAAAAC**C****U**.**G****C**UAAAUA..**G****A****U****G**UUUCAUGAGA | |
|  |  | AACY01493270.1/405-334  | AGAAGCUUAU**U****G****C****G****C****U**...GAUUU**A**............**.****A****C****U**.G**A**AUU**G****C****.****G****A****G****C****G****C****.**.UAUAAAAAAA**C****C**.**G****C**UAAAUA..**A****G****U****U**UUUUUUCCUC | |
|  |  | AACY01348042.1/437-509  | ACAAGAAAAC**C****G****C****G****C****U**...GAUUU**A**............**A****C****U****U**.A**A**AUU**G****C****.****G****A****G****C****G****C****.**.AUUAAAAAAA**C****A**.**G****C**UAAAAA..**A****G****G****U**UUUUGCCCAG | |
|  |  | AACY01445262.1/234-162  | ACAAGAAAAA**C****G****C****G****C****U**...GAUUU**A**............**A****C****U****U**.A**A**AUU**G****C****.****G****A****G****C****G****C****.**.UUUAAAAAAA**C****A**.**G****C**UAAAAA..**A****G****G****U**UUUUUGCCCA | |
|  |  | AACY01086877.1/617-545  | ACAAGAAAAU**C****G****C****G****C****U**...GAUUU**A**............**A****C****U****U**.A**A**AUU**G****C****.****G****A****G****C****G****C****.**.AUUAAAAAAA**C****A**.**G****C**UAAAAA..**A****G****G****U**UUUUGCCCAG | |
|  |  | AACY01178195.1/758-830  | ACAAGAAAAA**C****G****C****G****C****U**...GAUUU**A**............**A****C****U****U**.A**A**AUU**G****C****.****G****A****G****C****G****C****.**.AUUAAAAAAA**C****A**.**G****C**UAAAAA..**A****G****G****U**UUUUGCCCAG | |
|  |  | AACY01108759.1/4017-3940  | UUUUACGGAA**U****G****U****G****G****U**...GAUUU**G**AGCC........**A****G****U****C**.G**G**CUU**G****C****.****G****G****C****C****A****C****A**..UAAAAAAAG**U****C**.**G****C**UAAAAAA.**G****G****C****U**GGGAGAGACC | |
|  |  | AACY01570886.1/484-561  | UUUUACGGAA**U****G****U****G****G****U**...GAUUU**G**AGCC........**A****G****U****C**.G**G**CUU**G****C****.****G****G****C****C****A****C****A**..UAAAAAAAG**U****C**.**G****C**UAAAAAA.**G****G****C****U**GGGAGAGACC | |
|  |  | AACY01585882.1/131-54  | UUUUACGGAA**U****G****U****G****G****U**...GAUUU**G**AGCC........**A****G****U****C**.G**G**CUU**G****C****.****G****G****C****C****A****C****A**..UAAAAAAAG**U****C**.**G****C**UAAAAAA.**G****G****C****U**GGGAGAGACC | |
|  |  | AACY01619791.1/218-141  | UUUUACGGAA**U****G****U****G****G****U**...GAUUU**G**AGCC........**A****G****U****C**.G**G**CUU**G****C****.****G****G****C****C****A****C****A**..UAAAAAAAG**U****C**.**G****C**UAAAAAA.**G****G****C****U**GGGAGAGACC | |
|  |  | AACY01644470.1/665-737  | GUUAUUUACA**A****G****C****G****C****U**...GAUUU**A**A...........**.****G****U****C**.G**A**AUU**G****C****.****G****G****G****C****G****C****U**..AUAAAAAAC**C****U**.**G****C**UAAAUA..**G****A****U****G**UUUCUUGUGA | |
|  |  | AACY01534516.1/549-620  | ACAAAAAAAA**C****G****C****G****C****U**...GAUUU**A**............**.****U****C****U**.G**A**AUU**G****C****.****G****A****G****C****G****C****.**.UUAAAAAAAA**C****C**.**G****C**UAAAUA..**A****G****U****U**UUUUUCUCUC | |
|  |  | AACY01804136.1/192-119  | UAAUUUCUUC**U****G****U****C****G****U**...GAUUU**G**C...........**.****A****C****C**.A**U**AUU**G****C****.****C****G****C****G****A****C****A**.AUAAAUAAAA**G****C**.**G****C**UAAAAC..**G****G****A****A**UUUAAACAAG | |
|  |  | AACY01120549.1/935-863  | AAAAGGUUUA**U****G****C****G****C****U**...GAUUU**A**A...........**.****A****C****U**.G**A**AUU**G****C****.****G****A****G****C****G****C****.**.UUUAAAAAAC**C****U**.**G****C**UAAACA..**A****G****U****U**UUUUGCCUCA | |
|  |  | AACY01029331.1/580-509  | AAAACGUUUU**U****G****C****G****C****U**...GAUUU**A**............**.****A****C****U**.G**A**AUU**G****C****.****G****A****G****C****G****C****U**..AUAAAAAAC**C****U**.**G****C**UAAAUA..**A****G****U****U**UUUUUUCUCA | |
|  |  | AACY01064827.1/1110-1039  | AAAACGUUUU**U****G****C****G****C****U**...GAUUU**A**............**.****A****C****U**.G**A**AUU**G****C****.****G****A****G****C****G****C****U**..AUAAAAAAC**C****U**.**G****C**UAAAUA..**A****G****U****U**UUUUUUCUCA | |
|  |  | AACY01076673.1/120-191  | AAACAUUAAU**C****G****C****G****C****U**...GAUUU**A**............**.****A****C****C**.G**U**AUU**G****C****.****A****A****G****C****G****C****G**..UGAUAAAUG**U****A**.**G****C**UAAAAA..**G****G****G****G**AUGCCCAGUU | |
|  |  | AACY01381865.1/20-91  | AAAACGUUUU**U****G****C****G****C****U**...GAUUU**A**............**.****A****C****U**.G**A**AUU**G****C****.****G****A****G****C****G****C****U**..AUAAAAAAC**C****U**.**G****C**UAAAUA..**A****G****U****U**UUUUUUCUCA | |
|  |  | AACY01643985.1/166-95  | ACAAAAAAAG**C****G****C****G****C****U**...GAUUU**A**............**.****U****C****U**.G**A**AUU**G****C****.****G****A****G****C****G****C****.**.UUAAAAAAAA**C****C**.**G****C**UAAAUA..**A****G****U****U**UUUUUCUCAC | |
|  |  | AACY01265380.1/385-313  | ACAAGAAAAG**U****G****C****G****C****U**...GAUUU**A**A...........**.****C****U****U**.A**A**AUU**G****C****.****G****A****G****C****G****C****A**..UUAAAAAAA**C****A**.**G****C**UAAAAA..**A****G****G****U**UUUUGCCCGG | |
|  |  | AACY01121489.1/446-523  | UUUUACAGAA**U****G****U****G****G****U**...GAUUU**G**AGCC........**A****G****U****C**.G**G**CUU**G****C****.****G****G****C****C****A****C****A**..UAAAAGAAA**U****C**.**G****C**UAAAAAA.**G****G****C****U**GGGAGAGACC | |
|  |  | AACY01441579.1/355-284  | AAACAUUAUU**C****G****C****G****C****U**...GAUUU**A**............**.****A****C****C**.A**U**AUU**G****C****.****A****A****G****C****G****C****.**.AUAAUAAAUG**U****A**.**G****C**UAAAAA..**G****G****G****G**AUGCCCAGUU | |
|  |  | AACY01073927.1/447-376  | AGUAAUUUUG**G****G****C****G****C****U**...GAUUU**A**............**.****U****C****U**.G**A**AUU**G****C****.****G****A****G****C****G****C****.**.AAUAAAAAAA**C****C**.**G****C**UAAAAA..**A****G****U****U**UUUUUUCUCA | |
|  |  | AACY01439621.1/376-447  | ACAAAAUUUA**U****G****C****G****C****U**...GAUUU**A**............**.****C****A****U**.G**A**AUU**G****C****.****G****A****G****C****G****C****.**.UCUAAAAAAA**C****C**.**G****C**UAAAAA..**G****U****U****U**UUUUCUCCCA | |
|  |  | AACY01433405.1/386-314  | AUUGAUACAU**A****G****C****G****C****U**...GAUUU**G**A...........**.****A****U****C**.A**G**AUU**G****C****.****G****G****G****C****G****C****U**..UUAAAAAUC**C****U**.**G****C**UAAAGC..**G****A****C****A**UUUUUAUGCC | |
|  |  | AACY01044026.1/185-257  | UUUCAAAAUU**A****G****C****G****C****U**...GAUUU**A**A...........**.****G****A****C**.A**A**AUU**G****C****.****G****A****G****C****G****C****U**..UUAAAAAAU**C****C**.**G****C**UAAAGC..**G****U****U****U**AAUCUUUCGA | |
|  |  | AACY01718172.1/291-221  | ACAUAAAAAA**C****G****C****G****C****U**...GAUUU**A**............**.****U****C****U**.G**A**AUU**G****C****.****G****A****G****C****G****C****.**..UUAAAAAAA**C****C**.**G****C**UAAAUA..**A****G****U****U**UUUUUCUCAC | |
|  |  | AACY01116235.1/1088-1017  | AAACAUUACU**C****G****C****G****C****U**...GAUUU**A**............**.****A****C****C**.A**U**AUU**G****C****.****A****A****G****C****G****C****.**.AUAAUAAAUG**U****A**.**G****C**UAAAAA..**G****G****G****G**AUGCCCAGUU | |
|  |  | AACY01785307.1/562-633  | AAACAUUACU**C****G****C****G****C****U**...GAUUU**A**............**.****A****C****C**.A**U**AUU**G****C****.****A****A****G****C****G****C****.**.AUAAUAAAUG**U****A**.**G****C**UAAAAA..**G****G****G****G**AUGCCCAGUU | |
|  |  | AACY01075589.1/2317-2394  | AUUUACGGAA**U****G****U****G****G****U**...GAUUU**G**AGCC........**A****G****U****C**.G**G**CUU**G****C****.****G****G****C****C****A****C****.**.AUAAAAGAAA**U****C**.**G****C**UAAAAAA.**G****G****C****U**GGGAGAGACC | |
|  |  | AACY01293033.1/916-845  | AAACAUAAUU**C****G****C****G****C****U**...GAUUU**A**............**.****A****C****C**.A**U**AUU**G****C****.****A****A****G****C****G****C****.**.AUAAUAAAUG**U****A**.**G****C**UAAAAA..**G****G****G****G**AUGCCCAGUU | |
|  |  | AACY01181281.1/450-378  | AAAAGCUGCA**U****G****C****G****C****U**...GAUUU**A**U...........**.****.****A****C**UG**A**AUU**G****C****.****G****A****G****C****G****C****.**.UAUAAAAAAA**C****C**.**G****C**UAAAAC..**G****U****U****U**UUUUUCUCAC | |
|  |  | AACY01431623.1/281-353  | UUUCAGAAUU**A****G****C****G****C****U**...GAUUU**A**A...........**.****G****A****C**.A**A**AUU**G****C****.****G****A****G****C****G****C****U**..UUAAAAAAU**C****C**.**G****C**UAAAGC..**G****U****U****U**AAUCUUUCGA | |
|  |  | AACY01773448.1/601-529  | ACAAGAAAAG**C****G****C****G****C****U**...GAUUU**A**A...........**.****C****U****U**.A**A**AUU**G****C****.****G****A****G****C****G****C****.**.AUUAAAAAAA**C****A**.**G****C**UAAAAA..**A****G****G****U**UUUUCGCCCG | |
|  |  | AACY01013609.1/1545-1622  | UUUUACGGAA**U****G****U****G****G****U**...GAUUU**G**AGCC........**A****G****U****C**.G**G**CUU**G****C****.****G****G****C****C****A****C****A**..UAAAAGAAG**U****C**.**G****C**UAAAAAA.**G****G****C****U**GGGAGAGACC | |
|  |  | AACY01078288.1/1112-1189  | UUUUACGGAA**U****G****U****G****G****U**...GAUUU**G**AGCC........**A****G****U****C**.G**G**CUU**G****C****.****G****G****C****C****A****C****A**..UAAAAGAAG**U****C**.**G****C**UAAAAAA.**G****G****C****U**GGGAGAGACC | |
|  |  | AAFY01004711.1/584-660  | AUGCGCUGCU**C****G****U****G****G****U**...GAUUU**G**GCC.........**G****G****C****C**.G**G**CUU**G****C****.****A****G****C****C****A****C****.**.GUAAAACAAG**U****C**.**G****C**UAAAAAG.**G****G****C****C**GCGCGCAGGA | |
|  |  | AACY01241674.1/566-637  | AGUAAUUUCA**G****G****C****G****C****U**...GAUUU**A**............**.****U****C****U**.G**A**AUU**G****C****.****G****A****G****C****G****C****A**..AUAAAAAAA**C****C**.**G****C**UAAAUA..**A****G****U****U**UUUUUUCUCA | |
|  |  | AACY01331632.1/615-543  | UUUUAAAAUU**A****G****C****G****C****U**...GAUUU**A**A...........**.****G****A****C**.A**A**AUU**G****C****.****G****A****G****C****G****C****U**..UUAAAAAAU**C****C**.**G****C**UAAAGC..**G****U****U****A**AAUCUUUCGA | |
|  |  | AACY01185668.1/368-292  | AUUUUCUAAU**U****G****U****G****G****U**...GAUUU**G**AGCC........**G****A****C****C**.G**G**CUU**G****C****.****G****G****C****C****A****C****A**..GAAAAUAUG**C****A**.**G****C**UUAAAA..**G****G****U****C**GGAUGCUCAU | |
|  |  | AACY01775319.1/125-53  | ACUAAUUUCU**G****G****C****G****C****U**...GAUUU**A**............**.****U****C****U**.G**A**AUU**G****C****.****G****A****G****C****G****C****A**..AUAAAAAAA**C****C**.**G****C**UAAAAAA.**A****G****U****U**UUUUUACUCA | |
|  |  | AACY01565142.1/308-380  | ACUAAUUUUU**G****G****C****G****C****U**...GAUUU**A**............**.****U****C****U**.G**A**AUU**G****C****.****G****A****G****C****G****C****A**..AUAAAAAAA**C****C**.**G****C**UAAAAAA.**A****G****U****U**UUUUUUCUCA | |
|  |  | AACY01536804.1/771-698  | UUAUAAAACA**C****G****U****C****G****U**...GAUUU**G**C...........**.****A****C****C**.C**U**AUU**G****C****.****C****G****C****G****A****C****.**AAUAAAUAAAA**G****U**.**G****C**UAAAAA..**G****G****A****U**UUGACCCUAA | |
|  |  | AACY01209727.1/708-780  | AUUAGUACAU**A****G****C****G****C****C**...GAUUU**G**A...........**.****A****U****C**.A**G**AUU**G****C****.****G****G****G****C****G****C****U**..UUAAAAAUC**C****A**.**G****C**UAAAGC..**G****A****C****A**UGUCUUGACC | |
|  |  | AACY01035686.1/4473-4545  | AAAAGCUGCA**U****G****C****G****C****U**...GAUUU**A**............**.****U****A****C**UG**A**AUU**G****C****.****G****A****G****C****G****C****.**.UAUAAAAAAA**C****C**.**G****C**UAAAAU..**G****U****U****U**UUUUUCUCAC | |
|  |  | AACY01077171.1/822-750  | AAAAGCUGCA**U****G****C****G****C****U**...GAUUU**A**............**.****U****A****C**UG**A**AUU**G****C****.****G****A****G****C****G****C****.**.UAUAAAAAAA**C****C**.**G****C**UAAAAU..**G****U****U****U**UUUUUCUCAC | |
|  |  | AACY01361486.1/365-437  | AAAAGCUGCA**U****G****C****G****C****U**...GAUUU**A**............**.****U****A****C**UG**A**AUU**G****C****.****G****A****G****C****G****C****.**.UAUAAAAAAA**C****C**.**G****C**UAAAAU..**G****U****U****U**UUUUUCUCAC | |
|  |  | AACY01007478.1/2896-2825  | AAAUAGAUAU**C****G****C****G****C****U**...GAUUU**A**............**.****A****C****C**.A**U**AUU**G****C****.****A****A****G****C****G****C****A**..UAAUAAAUG**U****A**.**G****C**UAAAAA..**G****G****G****A**GAUGCCCAGU | |
|  |  | AACY01748608.1/641-569  | AAUUAUACAU**A****G****C****G****C****U**...GAGUU**G**A...........**.****A****U****C**.A**G**AUU**G****C****.****G****G****G****C****G****C****U**..UUAAAAAUC**C****A**.**G****C**UAAAGC..**G****A****C****A**GUUCUACACC | |
|  |  | AACY01273405.1/367-439  | AAAAGCUGCA**U****G****C****G****C****U**...GAUUU**A**............**.****U****A****C**UG**A**AUU**G****C****.****G****A****G****C****G****C****.**.UAUAAAAAAA**C****C**.**G****C**UAAAAU..**G****U****U****U**UUUUCCUCAC | |
|  |  | AACY01080761.1/1072-998  | AACUUAAGUA**A****G****C****G****C****U**...GAUUU**G**AGC.........**.****U****C****C**.A**G**CUU**G****C****.****G****A****G****C****G****C****U**..AUAUAAAUA**C****U**.**G****C**UAAAGA..**G****G****U****G**GUUAGGAAAC | |
|  |  | AACY01304150.1/463-390  | AAUCCUUUGA**A****G****U****C****G****U**...GAUUU**G**............**.****G****A****C**AG**U**AUU**G****C****.****A****G****C****G****A****C****A**..UUAAAAAAA**U****U**.**G****C**UAAAAUG.**G****U****U****G**AAAACUAGAA | |
|  |  | AACY01363007.1/302-231  | ACUAAUUUGA**A****G****C****G****C****U**...GAUUU**A**............**.****A****C****C**.A**A**AUU**G****C****.****A****A****G****C****G****C****U**..AAAUAAAUU**U****U**.**G****C**UAAAAA..**G****G****G****U**AUAUGCCCAG | |
|  |  | AACY01705186.1/762-834  | AAACAUAUCU**C****G****C****G****C****U**...GAUUU**A**............**.****A****C****C**.A**U**AUU**G****C****.****A****A****G****C****G****C****A**..UAAUAAAUG**U****A**.**G****C**UAAAAAA.**G****G****G****G**AUGCCCAGUU | |
|  |  | AACY01048873.1/705-779  | AUACAGUGAC**U****G****C****G****C****C**...GAUUU**G**AG..........**.****C****A****C**.A**G**CUU**G****C****.****G****G****G****C****G****C****.**.UGUAAAAAUG**C****A**.**G****C**UAAAGAG.**G****U****A****U**AUAAACCACC | |
|  |  | AACY01078841.1/1470-1539  | ACAAAACUUC**U****G****C****G****C****U**...GAUUU**A**............**.****A****C****U**.G**A**AUU**G****C****.****G****A****G****C****G****C****U**..UAAAAAAAC**C****C**.**G****C**UAAAU...**A****G****U****U**UUUUUUCUC. | |
|  |  | AACY01503107.1/95-168  | AUUAAUCUAC**A****G****U****C****G****U**...GAUUU**G**A...........**.****A****C****C**.C**U**AUU**G****C****.****C****G****C****G****A****C****A**..UAAAAUAAU**G****G**U**G****C**UAAAAA..**G****G****U****C**AAUAAGCCCG | |
|  |  | AACY01406742.1/517-588  | AAAAGAAAGC**A****G****C****G****C****U**...GAUUU**A**............**.****A****C****U**.U**A**AUU**G****C****.****G****A****G****C****G****C****U**..UAAAAAAAA**C****A**.**G****C**UAAAAA..**A****G****G****U**UUUGGCCCGG | |
|  |  | AACY01257470.1/768-696  | GAUAUUAAUU**A****G****C****G****C****C**...GAUUU**A**A...........**.****G****U****U**.A**A**AUU**G****C****.****G****G****G****C****G****C****U**..UAAAAAAUC**C****A**.**G****C**UAAAGC..**G****A****C****A**UAUCAACCAC | |
|  |  | AACY01012229.1/926-854  | GUUAUUUACA**A****G****C****G****C****U**...GAUUU**A**A...........**.****G****U****C**.G**A**AUU**G****C****.****G****G****G****C****G****C****U**..UUACAAAAC**C****U**.**G****C**UAAAUA..**G****A****U****G**UUUCUUAUGA | |
|  |  | AACY01374712.1/241-312  | AUUUCUUCAA**A****G****C****G****C****C**...GAUUU**A**............**.****A****C****C**.A**A**AUU**G****C****.****A****G****G****C****G****C****U**..UUAUAAAUG**U****A**.**G****C**UAAACA..**G****G****A****G**AUAGCCCAGU | |
|  |  | AACY01268978.1/672-743  | AAAACGUUUU**U****G****C****G****C****U**...GAUUU**A**............**.****A****C****U**.G**A**AUU**G****C****.****G****A****G****C****G****C****U**..UUAAAAAAC**C****C**U**G****C**UAAUA...**A****G****U****U**UUUUUUCUCA | |
|  |  | AACY01404705.1/733-805  | ACAAGAAAAG**C****G****C****G****C****U**...GAUUU**A**............**.****A****C****U**.A**A**AUU**G****C****.****G****A****G****C****G****C****.**.AUUAAAAAAA**C****A**.**G****C**UAAAGUA.**G****G****U****U**UUUGCCCAGC | |
|  |  | AACY01206430.1/327-401  | AUUUAAAUAA**A****G****C****G****C****U**...GAUUU**G**AGC.........**.****U****C****C**.A**G**CUU**G****C****.****G****A****G****C****G****C****U**..AUAUAAAUA**U****U**.**G****C**UAAAGA..**G****G****G****U**GGUAAGGAAC | |
|  |  | AACY01299030.1/820-892  | UUAUUUCAUU**A****G****C****A****C****C**...GAUUU**G**A...........**.****A****C****C**.A**G**CUU**G****C****.****G****G****G****U****G****C****U**..UUAUAAAUA**C****U**.**G****C**UAAAGA..**G****G****A****U**UCUUCUGAAA | |
|  |  | AACY01637175.1/180-251  | AUUAAUUAAA**G****G****C****G****C****C**...GAUUU**A**............**.****A****C****C**.A**A**AUU**G****C****.****G****G****G****C****G****C****.**.UUUAUAAAUG**U****A**.**G****C**UAAACA..**G****G****A****G**AUAGCCCAGU | |
|  |  | AACY01485766.1/35-108  | UAAUUUUGCA**U****G****U****C****G****U**...GAUUU**A**C...........**.****U****C****C**.G**U**AUU**G****C****.****U****G****C****G****A****C****A**..AUAAAUAAA**A****G**U**G****C**UAAAAA..**G****G****A****U**UAAUACCCCG | |
|  |  | AACY01593967.1/296-369  | AAUUAUCUAC**A****G****U****C****G****U**...GAUUU**G**A...........**.****A****C****C**.C**U**AUU**G****C****.****C****G****C****G****A****C****A**..AAAAAUAAU**G****G**U**G****C**UAAAAA..**G****G****U****U**AAUAAGCCCG | |
|  |  | AACY01344345.1/903-829  | GAUAAUGUGU**G****U****C****G****C****U**...GAUUU**G**ACA.........**.****G****U****C**.A**G**AUU**G****C****.****G****A****G****C****G****A****U**..UUAUAAAUA**C****U**.**G****C**UAAAGA..**G****G****U****G**UAUCAUAAAA | |
|  |  | AACY01001152.1/1307-1234  | AUAAUAAUCG**U****G****U****C****G****U**...GUUUU**G**U...........**.****G****C****C**.G**U**AUU**G****C****.****C****G****C****G****A****C****A**..AAAAAUAAA**G****U**U**G****C**UAAAAA..**G****G****U****U**UAUAACCCCG | |
|  |  | AACY01001150.1/214-141  | ACAAUAAUCG**U****G****U****C****G****U**...GUUUU**G**U...........**.****G****C****C**.G**U**AUU**G****C****.****C****G****C****G****A****C****A**..AAAAAUAAA**G****U**U**G****C**UAAAAA..**G****G****U****U**UGUAACCCCG | |
|  |  | AACY01607341.1/463-536  | AAUUUAAUUA**U****G****U****C****G****U**...GAUUU**A**G...........**.****A****C****C**.A**A**AUU**G****C****.****C****G****C****G****A****C****G**.UUAAACUAGA**G****U**.**G****C**UAAAAA..**G****G****A****G**UAGGCCCGAU | |
|  |  | AACY01029422.1/740-811  | AUUUCCCUAA**A****G****C****G****C****C**...GAUUU**A**............**.****A****C****C**.A**A**AUU**G****C****.****A****G****G****C****G****C****U**..UUAUAAAUG**U****A**.**G****C**UAAACA..**G****G****A****G**AUAGCCCAGU | |
|  |  | AACY01369203.1/279-351  | GAUAUUGGUC**A****G****C****G****C****C**...GAUUU**A**A...........**.****G****U****U**.A**A**AUU**G****C****.****G****G****G****C****G****C****U**..UAAAAAAUC**C****A**.**G****C**UAAAGC..**G****A****C****A**UAUCAACCAC | |
|  |  | AACY01143447.1/807-879  | AAAUGCCCUA**A****G****U****C****G****U**...GAUUU**G**G...........**.****A****U****A**.G**U**AUU**G****C****.****A****G****C****G****A****C****U**..UAAAAUAAA**U****U**.**G****C**UAAAAU..**G****G****U****A**AAUUAUAUGA | |
|  |  | AACY01208105.1/656-583  | UUAUAUUUAU**A****G****C****G****C****U**...GAUUU**A**AA..........**.****U****A****C**.A**A**AUU**G****C****.****G****A****G****C****G****C****U**..CUAAAAAAU**C****C**.**G****C**UAAAGC..**G****U****U****C**AAUCUUUCGA | |
|  |  | AACY01015393.1/1526-1453  | AUUUAAUAAC**A****G****U****C****G****U**...GUUUU**G**C...........**.****A****C****C**.A**U**AUU**G****C****.****C****G****C****G****A****C****.**GUAAAAAAAAU**G****U**.**G****C**UAAAAA..**G****G****U****A**AAUAGUAAUA | |
|  |  | AACY01063684.1/832-903  | AAACAUUAAG**G****G****C****G****C****U**...GAUUU**A**............**.****A****C****C**.A**U**AUU**G****C****.****A****A****G****C****G****C****.**.AUAAUAAAUU**U****A**.**G****C**UAAAAA..**G****G****G****G**UUCGCCCAGU | |
|  |  | AACY01096380.1/1858-1786  | CAUAUUAGUU**A****G****C****G****C****C**...GAUUU**A**A...........**.****G****U****U**.A**A**AUU**G****C****.****G****G****G****C****G****C****U**..UUAAAAAUC**C****A**.**G****C**UAAAGC..**G****A****C****U**CUUCAACCAC | |
|  |  | AACY01348412.1/728-657  | AAACACUAAU**C****G****C****G****C****U**...GAUUU**A**............**.****A****C****C**.A**U**AUU**G****C****.****A****A****G****C****G****C****.**.AUAAUAAAUG**U****A**.**G****C**UAAAAA..**G****G****G****C**ACGCCCAGUU | |
|  |  | AACY01731488.1/627-700  | UGUAUUUACA**A****G****C****G****C****U**...GAUUU**A**A...........**.****G****U****C**.A**A**AUU**G****C****G****G****G****G****C****G****C****U**..UUAAAAAAC**C****U**.**G****C**UAAAUA..**G****A****U****G**CUUCUUGUGA | |
|  |  | AACY01060962.1/1608-1534  | UUAUUUAUAU**A****A****C****G****C****C**...GAUUU**G**AAA.........**.****A****A****C**.A**G**AUU**G****C****.****G****G****G****C****G****U****U**..UUAAAAGUG**C****A**.**G****C**UAAAGA..**G****U****U****G**GGAUCGCCCC | |
|  |  | AACY01093264.1/245-318  | UUAGACUAAA**U****G****U****C****G****U**...GAUUU**G**C...........**.****A****C****C**.C**U**AUU**G****C****.****C****G****C****G****A****C****.**ACAAAAUAAAA**G****U**.**G****C**UAAAAA..**G****G****A****U**UUGACCCUAA | |
|  |  | AACY01137881.1/882-812  | UCGAUUCCAU**A****G****C****G****U****U**...GAUUU**A**............**.****A****C****C**.G**A**CUU**G****C****.****U****G****A****C****G****C****U**..UUAAAAAUU**A****.**.**G****C**UAAACA..**G****G****A****C**UUUUUAAACC | |
|  |  | AACY01733748.1/700-628  | CAUAUUAGUU**A****G****C****G****C****C**...GAUUU**A**A...........**.****G****U****U**.A**A**AUU**G****C****.****G****G****G****C****G****C****U**..UAAAAAAUC**C****A**.**G****C**UAAAGC..**G****A****C****A**CUUCAACCAC | |
|  |  | AACY01717922.1/636-564  | UUUCAUUCAU**A****G****C****G****C****U**...GAUUU**A**A...........**.****A****A****C**.A**A**AUU**G****C****.****G****A****G****C****G****C****U**..UUAUAAAAC**C****C**.**G****C**UAAAGC..**G****U****A****A**AAUCUUCGUC | |
|  |  | AACY01728866.1/235-164  | AAACAUUAGG**G****G****C****G****C****U**...GAUUU**A**............**.****A****C****C**.A**U**AUU**G****C****.****A****A****G****C****G****C****.**.AUAAUAAAUU**U****G**.**G****C**UAAAAA..**G****G****G****G**UUCGCCCAGU | |
|  |  | AACY01083180.1/106-178  | CAUAUUAGUU**A****G****C****G****C****C**...GAUUU**A**A...........**.****G****U****U**.A**A**AUU**G****C****.****G****G****G****C****G****C****U**..UAAAAAAUC**C****A**.**G****C**UAAAGC..**G****A****C****G**CUUCAACCAC | |
|  |  | AAFX01060896.1/134-59  | AUCAAUCAGU**A****G****C****G****C****C**...GAUUU**G**AGCG........**.****A****U****C**.A**G**CUU**G****C****.****G****G****G****C****G****C****U**..UAAUAAAUG**C****A**.**G****C**UAAAGC..**G****U****U****G**GCGCCCAUCA | |
|  |  | AACY01803676.1/412-340  | AUUUCUAAAU**A****G****C****G****C****U**...GAUUU**A**A...........**.****U****A****C**.A**A**AUU**G****C****.****G****A****G****C****G****C****U**..UUAUAAAAU**C****C**.**G****C**UAAAGC..**G****U****U****A**AAUCUUUCGA | |
|  |  | AACY01003120.1/597-670  | AAGAAAAUAA**U****G****U****C****G****U**...GUUUU**G**C...........**.****A****C****C**.A**U**AUU**G****C****.****C****G****C****G****A****C****A**..AAAAAUAAU**G****U**U**G****C**UAAAAA..**G****G****U****A**UACAUACCCC | |
|  |  | AACY01021379.1/450-378  | UUAUUCAUAA**A****G****C****A****C****C**...GAUUU**G**A...........**.****A****C****C**.A**G**AUU**G****C****.****G****G****G****U****G****C****U**..UUAUAAAUA**C****U**.**G****C**UAAAUA..**G****G****A****C**ACUCAAAGAC | |
|  |  | AACY01387396.1/186-115  | AUUUCCUUAA**G****G****C****G****C****C**...GAUUU**A**............**.****A****C****C**.A**A**AUU**G****C****.****A****G****G****C****G****C****.**.UUUAUAAAUG**U****A**.**G****C**UAAACA..**G****G****A****G**GUAGCCCAGU | |
|  |  | AACY01064046.1/354-425  | AUCAACGUAA**G****G****C****G****C****C**...GAUUU**A**............**.****A****C****C**.A**A**AUU**G****C****.****A****G****G****C****G****C****U**..UUAUAAAUA**U****A**.**G****C**UAAAUA..**G****G****A****G**AUAGCCCAGU | |
|  |  | AACY01205026.1/629-558  | AUAUAUGCGA**A****G****C****G****C****U**...GAUUU**A**............**.****A****C****C**.G**A**AUU**G****C****.****G****A****G****C****G****C****U**..AAGUAAAUG**C****A**.**G****C**UAAACA..**G****G****U****U**UUUGCCUGGU | |
|  |  | AACY01555644.1/376-305  | AUUUCCUCAA**G****G****C****G****C****C**...GAUUU**A**............**.****A****C****C**.A**A**AUU**G****C****.****A****G****G****C****G****C****U**..UUAUAAAUG**U****A**.**G****C**UAAACA..**G****G****A****G**GUAGCCCAGU | |
|  |  | AACY01112290.1/822-751  | UUUCAACAUU**A****G****C****G****C****U**...GAUUU**A**A...........**.****A****A****C**.A**A**AUU**G****C****.****G****A****G****C****G****C****U**..UUAAAAAUC**C****.**.**G****C**UAAAGC..**G****U****U****A**AAUCUUUCGA | |
|  |  | AACY01619688.1/590-517  | UAAAGUGCUC**A****U****C****G****C****U**...GAUUU**A**A...........**.****U****A****C**.A**A**CUU**G****C****.****G****G****G****C****G****A****U**..UAAUAAAAA**C****A**.**G****C**UAAAGC.G**G****U****C****G**UAGUCGUCUC | |
|  |  | AACY01135370.1/405-334  | AUUCUCGAAA**G****G****C****G****C****C**...GAUUU**A**............**.****A****C****C**.A**A**AUU**G****C****.****A****G****G****C****G****C****.**.UUUAUAAAUG**U****A**.**G****C**UAAACA..**G****G****A****G**GUAGCCCAGU | |
|  |  | AACY01657037.1/580-506  | UAAGUUUGUC**A****G****C****G****C****C**...GAUUU**G**AAG.........**.****C****A****C**.A**G**AUU**G****C****.****G****G****G****C****G****C****U**..UAAUAAAUG**C****A**.**G****C**UAAAGC..**G****C****A****G**GGGUCACCCA | |
|  |  | AACY01767229.1/536-607  | UAAUUAAUUU**A****A****U****C****G****U**...GAUUU**G**............**.****A****U****C**.C**U**AUU**G****C****.****C****G****C****G****A****U****U**..UAAAAGAAG**G****C**.**G****C**UAAAAC..**G****G****A****A**AUUUUUCCCA | |
|  |  | AACY01232127.1/830-758  | AUUCAUAAAU**A****G****C****G****C****U**...GAGUU**G**A...........**.****A****U****C**.A**G**AUU**G****C****.****G****G****G****C****G****C****U**..UUAUAAAUC**C****A**.**G****C**UAAAGC..**G****A****C****A**UUUCGUAACC | |
|  |  | AACY01015394.1/1301-1228  | AUUUAUUUGU**G****G****U****C****G****U**...GCUUU**G**C...........**.****A****C****C**.A**U**AUU**G****C****.****C****G****C****G****A****C****G**..UAAAAAAAA**U****G**U**G****C**UAAAAA..**G****G****U****A**AAUAGAAAUA | |
|  |  | AACY01805754.1/590-661  | AUUUCCCUAA**G****G****C****G****C****C**...GAUUU**A**............**.****A****C****C**.A**A**AUU**G****C****.****A****G****G****C****G****C****U**..UUAUAAAUG**U****A**.**G****C**UAAACA..**G****G****A****G**GUAGCCCAGU | |
|  |  | AACY01262165.1/92-20  | UAUUUAAUUA**A****G****C****G****C****U**...GAUUU**A**A...........**.****U****U****C**.A**A**AUU**G****C****.****G****A****G****C****G****C****U**..UUAUAAAUC**C****U**.**G****C**UAAAGC..**G****A****U****A**CUCUUAAUAG | |
|  |  | AAFZ01015352.1/161-235  | AUAUGGGUUC**A****G****C****G****C****C**...GAUUU**G**AUU.........**.****U****U****C**.A**G**CUU**G****C****.****G****G****G****C****G****C****U**..CUAAAAAUA**C****A**.**G****C**UAAAGC..**G****G****C****G**GCAAUUUCAA | |
|  |  | AACY01443998.1/251-180  | UAAUAUACUU**A****A****U****C****G****U**...GAUUU**G**A...........**.****U****C****C**..**U**AUU**G****C****.****U****G****C****G****A****U****U**..UAAAAGAAA**G****U**.**G****C**UAAAAC..**G****G****A****A**UUAUUCCCAG | |
|  |  | AACY01810462.1/352-423  | AAGUUAAUUA**A****G****C****A****C****U**...GAUUU**G**A...........**.****A****C****C**..**U**AUU**G****C****.****G****A****G****U****G****C****U**..AUAUAAAUG**C****A**.**G****C**UAAAAC..**G****G****U****G**AAUAUUUAAU | |
|  |  | AACY01274729.1/277-349  | UAAAUGAAUU**U****G****C****G****C****C**...GAUUU**A**A...........**.****G****U****U**.A**A**AUU**G****C****.****G****G****G****C****G****C****U**..UUAUAAAUC**C****A**.**G****C**UAAAGC..**G****A****C****A**AUUCGACCAC | |
|  |  | AACY01805250.1/232-160  | UAGUUAAUUG**A****G****C****G****C****U**...GAUUU**A**A...........**.****U****U****C**.A**A**AUU**G****C****.****G****A****G****C****G****C****U**..UUAUAAAUC**C****U**.**G****C**UAAAGC..**G****A****U****A**AUCUUAAUAG | |
|  |  | AACY01328616.1/715-643  | UUUUACUUGU**A****G****C****G****C****U**...GAUUU**A**A...........**.****U****A****C**.A**A**AUU**G****C****.****G****A****G****C****G****C****U**..UUAUAAAAU**C****C**.**G****C**UAAAGC..**G****U****U****A**AAUCUUUCGA | |
|  |  | AACY01618212.1/708-780  | UAAAAUUUGA**A****G****C****G****C****U**...AAUUU**G**AA..........**A****C****U****C**.A**G**CUU**G****C****.****G****A****G****C****G****C****A**..UUAAAAAAG**.****.**.**G****C**UAAAGC..**G****G****G****U**CGAUUCCUGU | |
|  |  | AACY01071221.1/1003-932  | UAAUAUAUUU**A****A****U****C****G****U**...GAUUU**G**............**.****A****U****C**.C**U**AUU**G****C****.****U****A****C****G****A****U****U**..UAAAAGAAA**G****U**.**G****C**UAAAAC..**G****G****A****A**UUAUUCCCAG | |
|  |  | AACY01411598.1/527-597  | UAAUAAAUUA**A****U****U****C****G****U**...GAUUU**G**............**.****G****U****C**.C**U**AUU**G****C****.****A****G****C****G****A****A****U**..UAAAAGAAU**U****.**.**G****C**UAAAAC..**G****G****A****A**UAUUUUCCCA | |
|  |  | AAFY01022252.1/320-391  | UGUCAUGUUU**C****G****U****G****G****.**...GAUUU**G**UAG.........**.****A****C****C**.G**G**CUU**G****C****.****G****G****C****C****A****C****G**..UUAAAA.AC**C****.**.**G****C**UAAAGA..**G****G****A****C**AGGAUGGCAU | |
|  |  | AACY01739744.1/798-726  | UAUAUUUACA**A****G****C****G****C****U**...GAUUU**A**A...........**.****U****U****C**.A**A**AUU**G****C****.****G****A****G****C****G****C****U**..UUACAAAUC**C****A**.**G****C**UAAAGC..**G****A****A****U**UAUCAUAGAU | |
|  |  | AACY01336805.1/605-675  | AUUUGUACAU**A****G****C****G****C****U**...GAUUU**A**A...........**.****U****A****C**.A**A**AUU**G****C****.****G****A****G****C****G****C****U**..UUAUAAAAC**C****C**.**G****C**UAAAGC..**G****A****U****U**AAUCUUUU.. | |
|  |  | AACY01577166.1/700-774  | AUCUCAACAU**A****G****C****G****C****U**...GAUUU**A**A...........**.****G****U****C**.A**A**AUU**G****C****U****G****A****G****C****G****C****U**..UUAAAAAAU**C****C**A**G****C**UAAAGC..**G****A****U****C**AAGUCUUAAG | |
|  |  | AACY01711975.1/649-723  | CGAUUGGGCU**G****A****C****G****C****C**...GAUUU**G**AA..........**A****A****A****C**.A**G**AUU**G****C****.****G****G****G****C****G****U****U**..UUAAAAAUG**C****A**.**G****C**UAAAGA..**G****U****U****G**GGACCGCCCC | |
|  |  | AACY01450288.1/942-870  | UUUCACUCAU**A****G****C****G****C****U**...GAUUU**A**A...........**.****A****A****C**.A**A**AUU**G****C****.****G****A****G****C****G****C****U**..UUAUAAAAC**C****C**.**G****C**UAAAGC..**G****C****A****A**AAUCUUCGUC | |
|  |  | AACY01796596.1/410-338  | CUAAGUUAAU**A****G****C****G****C****C**...AUUUU**G**A...........**.****A****U****C**.A**G**AUU**G****C****.****G****G****G****C****G****C****.**.UUUAAAAAUC**U****A**.**G****C**UAAAGC..**G****A****C****C**UUUGUAGACC | |
|  |  | AACY01048420.1/1231-1159  | UUUUGAUUGU**A****G****C****G****C****U**...GAUUU**A**A...........**.****U****A****C**.A**A**AUU**G****C****.****G****A****G****C****G****C****U**..UUAUAAAAU**C****C**.**G****C**UAAAGC..**G****U****U****A**AAUCUUUCGA | |
|  |  | AACY01795422.1/192-263  | UACAUCCUCU**A****G****C****G****C****U**...GAUUU**A**............**.****A****C****C**.A**A**AUU**G****C****.****G****A****G****C****G****C****U**..GAGUAAAUG**C****A**.**G****C**UAAACA..**G****G****G****A**UUUUUUGCCC | |
|  |  | AACY01563135.1/653-579  | UCUUUUCAUU**A****G****C****G****C****U**...GAUUU**A**A...........**.****G****U****C**.G**A**AUU**G****C****G****G****G****G****C****G****C****U**..AUAUAAAAU**C****C**U**G****C**UAAAUA..**G****A****U****G**CUUCUAUGUG | |
|  |  | AACY01494096.1/177-249  | UGGCCCCCGA**A****G****U****G****G****U**...GAUUU**G**C...........**.****G****G****C**.A**U**AUU**G****C****.****A****G****C****C****A****C****U**..CUAAAAAAC**G****C**.**G****C**UAAAAA..**G****C****C****C**UGCUCCUCCU | |
|  |  | AACY01343213.1/325-253  | UAAUUUGUUU**A****G****C****G****C****U**...GAGUU**A**A...........**.****G****A****C**.A**A**AUU**G****C****.****G****G****G****C****G****C****U**..UAAUAAAUC**C****A**.**G****C**UAAAGC..**G****U****U****U**AAGUCUAAUG | |
|  |  | AACY01742267.1/298-227  | AUUCUCAAAA**G****G****C****G****C****C**...GAUUU**A**............**.****A****C****C**.A**A**AUU**G****C****.****A****G****G****C****G****C****.**.UUGAUAAAUG**U****A**.**G****C**UAAACA..**G****G****A****G**GUAGCCCAGU | |
|  |  | AACY01009558.1/369-440  | UUAAUUGUUA**C****A****U****C****G****U**...GAUUU**G**............**.****A****U****C**.C**U**AUU**G****C****.****C****G****C****G****A****U****G**..CUAAAGAAA**G****U**.**G****C**UAAAAC..**G****G****A****A**UUUUUUUCCC | |
|  |  | AACY01035068.1/423-495  | UUUCACUUGU**A****G****C****G****C****U**...GAUUU**A**A...........**.****U****A****C**.A**A**AUU**G****C****.****G****A****G****C****G****C****U**..UUAUAAAAU**C****C**.**G****C**UAAAGC..**G****U****C****A**AAUCUUUCGA | |
|  |  | AACY01005012.1/3996-3922  | AAUGAAACAU**A****U****C****G****C****U**...AAUUU**G**AUA.........**.****G****U****C**.A**G**AUU**G****C****.****G****G****G****C****G****A****U**..UUAUAAAUA**C****A**.**G****C**UAAAGA..**G****A****U****A**UACCUAAAAA | |
|  |  | AACY01138226.1/394-468  | AGCUUUUUAA**C****G****C****G****C****U**...GAUUU**A**AU..........**.****A****U****C**.A**A**CUU**G****C****A****G****A****G****C****G****C****.**..UUAAAAAAU**C****C**U**G****C**UAAAGC..**G****G****A****A**UAUAAACACA | |
|  |  | AACY01065007.1/3819-3890  | ACAUUCGCAU**U****G****C****G****C****U**...GAUUU**A**............**.****A****C****C**.A**A**AUU**G****C****.****G****A****G****C****G****C****A**..GAGUAAAUG**C****A**.**G****C**UAAACA..**G****G****G****U**UUUUGCCCAG | |
|  |  | AACY01810614.1/469-395  | AUUUGUAUUA**U****U****C****G****C****C**...GAUUU**G**AUA.........**.****G****U****C**.A**G**AUU**G****C****.****G****G****G****C****G****A****.**.UUAAUAAAUA**C****U**.**G****C**UAAAGA..**G****A****U****G**GCAUUUCCAA | |
|  |  | AACY01641010.1/498-569  | ACAUCCGCUU**A****G****C****G****C****U**...GAUUU**A**............**.****A****C****C**.G**A**AUU**G****C****.****G****A****G****C****G****C****U**..ACGUAAAUG**C****A**.**G****C**UAAACA..**G****G****G****U**UUUUUUGCCC | |
|  |  | AACY01420045.1/260-332  | CUUAAGUAAU**A****G****C****G****C****C**...GAGUU**G**A...........**.****A****U****C**.A**G**AUU**G****C****.****G****G****G****C****G****C****U**..UUAUAAAUC**C****A**.**G****C**UAAAGC..**G****A****C****A**UUUCUAGACC | |
|  |  | AACY01079367.1/220-149  | AUAUUCGCAU**U****G****C****G****C****U**...GAUUU**A**............**.****A****C****C**.G**A**AUU**G****C****.****G****A****G****C****G****C****.**.UGAGUAAAUG**C****A**.**G****C**UAAACA..**G****G****G****U**UUUUGCCCAG | |
|  |  | AACY01144579.1/372-300  | AAUCAUAAUU**A****G****C****G****C****U**...GAGUU**A**............**A****G****A****C**.A**A**CUU**G****C****.****G****G****G****C****G****C****U**..AUAUAAAUC**C****A**.**G****C**UAAAGC..**G****U****U****U**AAGUCAUAAG | |
|  |  | AAFY01000804.1/399-325  | UCUUAUCGAA**C****G****C****G****C****C**...GAUUU**G**CUC.........**.****C****U****C**.A**G**CUU**G****C****.****G****G****G****C****G****C****G**..AUAAAAAUG**C****G**.**G****C**UAAAGC..**G****A****G****A**GGGCGUGCAC | |
|  |  | AACY01114116.1/334-263  | UUAAUACAAG**A****G****U****C****G****U**...GAUUU**G**A...........**.****U****C****C**..**U**AUU**G****C****.****A****A****C****G****A****C****U**.UUUAAAUAAA**U****.**.**G****C**UAAAAC..**G****G****A****A**AAGGAAAUUC | |
|  |  | AACY01598367.1/520-591  | UUAAUACAAG**A****G****U****C****G****U**...GAUUU**G**A...........**.****U****C****C**..**U**AUU**G****C****.****A****A****C****G****A****C****U**.UUUAAAUAAA**U****.**.**G****C**UAAAAC..**G****G****A****A**AAGGAAAUUC | |
|  |  | AAFX01003096.1/692-764  | CGAGUCUGUU**C****G****U****G****G****U**...GAGUU**A**A...........**.****U****G****C**.G**A**CUU**G****C****.****G****A****C****C****A****C****.**.GUUAAAUAAA**C****U**.**G****C**UAAACA..**G****C****G****U**UAGGACAAUU | |
|  |  | AACY01363695.1/458-530  | GAUAUUGAUU**A****G****C****G****U****C**...GAUUU**A**A...........**.****G****U****U**.A**A**AUU**G****C****.****G****G****G****C****G****C****U**..UAAUAAAUC**C****A**.**G****C**UAAAGC..**G****A****C****A**UAUCAACCAC | |
|  |  | AACY01344986.1/476-404  | UUUGAUUUUU**A****G****C****G****C****U**...GAUUU**A**A...........**.****U****A****C**.A**A**AUU**G****C****.****G****A****G****C****G****C****U**..UUACAAAAU**C****C**.**G****C**UAAAGC..**G****A****A****U**CUUUCGAGAC | |
|  |  | AACY01439837.1/368-440  | UAAUAAAUAC**A****G****C****G****C****C**...GAUUU**G**UU..........**.****U****U****C**.A**G**CUU**G****C****.****G****G****G****C****G****C****A**..AAAGAAAUU**C****A**.**G****C**UAAAA...**G****G****U****U**UAGUCCGCCC | |
|  |  | AACY01110626.1/1268-1196  | UUUGUUUAAU**A****G****C****G****C****U**...GAUUU**A**A...........**.****U****A****C**.A**A**AUU**G****C****.****G****A****G****C****G****C****U**..UUACAAAAU**C****C**.**G****C**UAAAGC..**G****A****C****A**AUCUUUCGAG | |
|  |  | AACY01694218.1/380-452  | UUUGUUCUUU**A****G****C****G****C****U**...GAUUU**A**A...........**.****U****A****C**.A**A**AUU**G****C****.****G****A****G****C****G****C****U**..UUACAAAAU**C****C**.**G****C**UAAAGC..**G****A****C****A**AUCUUUCGAG | |
|  |  | AACY01743318.1/80-8  | UAUUUAAAAC**A****G****C****G****C****U**...GAGUU**A**A...........**.****A****G****C**.A**A**CUU**G****C****.****G****G****G****C****G****C****U**..UUAUAAAUC**C****U**.**G****C**UAAAGC..**G****C****A****U**AAGUCAUUCU | |
|  |  | AACY01068451.1/1141-1212  | AUAUUCGCCU**A****G****C****G****C****U**...GAUUU**A**............**.****A****C****C**.A**A**AUU**G****C****.****G****A****G****C****G****C****U**..GAGUAAAUG**C****A**.**G****C**UAAACA..**G****G****G****A**UUUUUUUUAC | |
|  |  | AACY01036105.1/707-634  | UCACUCCUAU**A****G****C****G****C****U**...GAUUU**A**A...........**.****G****U****C**.A**A**AUU**G****C****U****G****A****G****C****G****C****U**..UUAAAAAUC**C****A**.**G****C**UAAAGC..**G****A****U****U**AAGUCUUUAG | |
|  |  | AACY01037785.1/1399-1326  | UUCUUGCCUC**A****U****C****G****C****C**...GAUUU**A**AC..........**G****A****U****C**.A**A**CUU**G****C****.****G****G****G****C****G****A****U**..UAAAAA.UA**C****A**.**G****C**UAAAGC..**G****C****U****C**AAGGCCAGCA | |
|  |  | AACY01017218.1/767-696  | ACAUUCGCAU**U****G****C****G****C****U**...GAUUU**A**............**.****A****C****C**.G**A**AUU**G****C****.****G****A****G****C****G****C****.**.UGAGUAAAUG**C****A**.**G****C**UAAACA..**G****G****G****U**UUUUGCCCAG | |
|  |  | AACY01020820.1/548-477  | AUAAUUGAUU**C****G****U****C****G****U**...CAUUU**A**A...........**.****C****U****C**.U**A**CUU**G****C****.****A****G****C****G****A****C****.**.CUUAAAUAAC**U****.**.**G****C**UAAACA..**G****A****G****C**AAAGACAUAC | |
|  |  | AACY01186534.1/705-632  | UAAAAAAGAA**A****U****C****G****C****U**...GAUUU**A**AG..........**.****A****G****C**.A**A**AUU**G****C****.****G****G****G****C****G****A****U**..UAAUAAAUA**C****A**.**G****C**UAAAGC..**G****U****U****G**GCAGUCGUUU | |
|  |  | AACY01295864.1/258-330  | UUUGAACUUU**A****G****C****G****C****U**...GAUUU**A**A...........**.****U****A****C**.A**A**AUU**G****C****.****G****A****G****C****G****C****U**..UUACAAAAU**C****C**.**G****C**UAAAGC..**G****A****A****U**CUUUCGAGAC | |
|  |  | AACY01054701.1/930-1002  | AUAUUUAAUU**A****G****C****G****C****U**...GAGUU**A**U...........**.****A****A****C**.A**A**CUU**G****C****.****G****G****G****C****G****C****U**..UAAUAAAUC**C****A**.**G****C**UAAAGC..**G****U****U****A**AGUCUCAGAC | |
|  |  | AACY01086722.1/474-400  | GUUCGUACAU**A****G****C****G****C****U**...GAUUU**A**A...........**.****A****U****C**.A**A**AUU**G****C****G****G****A****G****C****G****C****U**..UAAAAAAUU**C****C**U**G****C**UAAAGC..**G****A****C****U**AGAUCAUAUG | |
|  |  | AACY01162985.1/784-713  | UUAUUAUCAA**A****A****C****G****C****C**...GAUUU**G**............**.****U****C****U**.G**G**CUU**G****C****.****G****G****G****C****G****U****U**..UAAUAAAUU**C****A**.**G****C**UAAAUA..**A****G****G****U**CCCAAUCUUA | |
|  |  | AACY01746239.1/641-569  | UAUUUUUUAA**A****G****C****G****C****U**...GAGUU**A**A...........**.****G****A****C**.A**A**CUU**G****C****.****G****G****G****C****G****C****U**..AUACAAAUC**C****A**.**G****C**UAAAGC..**G****U****U****U**AAGUCAAAUG | |
|  |  | AACY01398489.1/88-16  | UAAAUUCACU**A****G****C****G****C****U**...GAGUU**A**A...........**.****A****G****C**.A**A**CUU**G****C****.****G****G****G****C****G****C****U**..UAAUAAAUC**C****U**.**G****C**UAAAGC..**G****C****A****A**UAGUCAUUCA | |
|  |  | AACY01759909.1/903-831  | ACAUCCGCGU**A****G****C****G****C****U**...GAUUU**A**............**.****A****C****C**.G**A**AUU**G****C****.****G****A****G****C****G****C****U**..AAGUAAAUG**C****A**.**G****C**UAAACAU.**G****G****A****U**UUUUUGCCCA | |
|  |  | AACY01046906.1/484-410  | AUUAUCCCAU**U****G****C****G****C****C**...GAUUU**G**AG..........**C****U****U****C**.A**G**CUU**G****C****.****G****G****G****C****G****C****A**..UAAAAAGUG**C****C**.**G****C**UAAAGC..**G****U****G****G**GAGGUUAUCC | |
|  |  | AACY01178840.1/179-107  | AAGAUUAAUC**A****G****C****G****C****C**...GAGUU**A**A...........**.****G****A****C**.A**A**CUU**G****C****.****G****G****G****C****G****C****U**..UAAUAAAUC**C****U**.**G****C**UAAAGC..**G****U****A****U**AAGUCAUUUA | |
|  |  | AACY01607831.1/443-515  | UAAAUCCCUC**A****G****C****G****C****U**...GAGUU**A**A...........**.****G****G****C**.A**A**CUU**G****C****.****G****G****G****C****G****C****U**..UUAUAAAUC**C****U**.**G****C**UAAAGC..**G****C****A****U**AAGUCAUCUA | |
|  |  | AACY01012558.1/1352-1280  | AUAUUUAUUU**A****G****C****G****C****U**...GAGUU**A**U...........**.****A****A****C**.A**A**CUU**G****C****.****G****G****G****C****G****C****U**..UAAUAAAUC**C****A**.**G****C**UAAAGC..**G****U****U****A**AGUCUUAGAC | |
|  |  | AACY01046191.1/615-688  | AUCACUAUAU**A****G****C****G****C****U**...GAUUU**A**A...........**.****G****U****C**.A**A**AUU**G****C****U****G****A****G****C****G****C****U**..UAAAAAAUC**C****A**.**G****C**UAAAGC..**G****A****A****U**AAGUCUUUAG | |
|  |  | AACY01609258.1/665-738  | GUUCAAAUUG**A****G****C****G****C****C**...GAUUU**A**G...........**.****A****U****C**.A**A**CUU**G****C****G****G****G****G****C****G****C****U**..UAAUAAAUC**C****U**.**G****C**UAAAAA..**G****A****U****G**CUUCUUAUGA | |
|  |  | AACY01717520.1/773-837  | ACAAAAUUUA**U****G****C****G****C****U**...GAUUU**A**............**.****A****C****U**.G**A**AUU**G****C****.****G****A****G****C****G****C****.**.UUAAAAAAAA**C****C**.**G****C**UAAAAU..**A****G****U****U**UUU....... | |
|  |  | AADL01000262.1/155-228  | CAAUCGACCC**G****G****U****G****G****.**AGUGAUUU**G**A...........**.****G****A****C**..**G**AUU**G****C****.****A****A****C****C****A****C****C**UUAAAAAAAAG**U****.**.**G****C**UAAAA...**G****U****C****C**AGGAUGCGAG | |
|  |  | AACY01147536.1/376-305  | UUAAUGACUA**C****G****U****C****G****U**...GAUUU**G**............**.****A****U****C**.C**U**AUU**G****C****.****U****A****C****G****A****C****U**..UUAAAAUAA**G****A**.**G****C**UAAAAC..**G****G****A****A**AAUGAUUUUC | |
|  |  | AACY01083158.1/801-872  | AAUUCAUAGA**A****G****U****C****G****U**...GAUUU**A**A...........**.****C****U****C**.U**A**AUU**G****C****.****A****G****C****G****A****C****U**.UUAAAUUAAC**U****.**.**G****C**UAAAC...**G****G****A****G**AAAAGACUUA | |
|  |  | AACY01210929.1/147-75  | AUUAGUACAU**A****G****C****G****C****C**...GAGUU**G**A...........**.****A****U****C**.A**G**AUU**G****C****.****G****G****G****C****G****C****U**..UUAGAAAUC**C****A**.**G****C**UAAAGC..**G****A****C****A**UUAUAGACCA | |
|  |  | AACY01546156.1/191-118  | UUUUGGGAAA**U****G****C****G****C****C**...GAUUU**G**AA..........**.****A****U****C**.A**G**CUU**G****C****.****G****G****G****C****G****C****A**..AUAGAAAUA**C****C**.**G****C**UAAAGA..**G****A****C****A**ACCUGCUUUA | |
|  |  | AACY01398829.1/645-717  | AUAUUCGCCU**A****G****C****G****C****U**...GAUUU**A**............**.****A****C****C**AA**A**AUU**G****C****.****G****A****G****C****G****C****U**..AAGUAAAUG**C****A**.**G****C**UAAACA..**G****G****G****A**UUUUUUUGCC | |
|  |  | AAFZ01007814.1/393-464  | CUGCGCACUU**C****G****U****G****G****.**...GAUUU**G**UCG.........**.****A****C****C**.G**G**CUU**G****C****G****A****.****C****C****A****C****.**.G.UAAAA.AA**U****C**.**G****C**UAAAGA..**G****G****G****G**AUACAUGGCU | |
|  |  | AAFX01086593.1/823-750  | AAACUCAGAU**C****G****U****G****G****U**...GAUUU**A**C...........**.****G****G****C**.A**A**CUU**G****C****G****G****G****C****C****A****C****G**..CUAAACAAA**C****U**.**G****C**UAAAGC..**G****C****C****G**GCCCGGCGCC | |
|  |  | AACY01518342.1/652-581  | AAAUUUAGAU**C****G****C****A****C****C**...GAUUU**G**A...........**.****U****C****C**.A**G**CUU**G****C****.****G****G****G****U****G****C****.**.UCUAUAAAUA**C****U**.**G****C**UAAAC...**G****G****U****G**CUCUUAUAGA | |
|  |  | AACY01257615.1/242-166  | UUAAUUAAUG**A****G****C****A****C****C**...GAUUU**A**CUA.........**.****C****U****C**AA**U**AUU**G****C****.****G****G****G****U****G****C****U**..UUAUAAAUG**C****A**.**G****C**UAAAACG.**G****A****G****A**UAUUAACUUC | |
|  |  | AACY01133850.1/178-250  | AUAAGCACUU**A****G****C****G****C****C**...GAGUU**A**A...........**.****G****A****C**.A**A**CUU**G****C****.****G****G****G****C****G****C****U**..UAAUAAAUC**C****A**.**G****C**UAAAGC..**G****U****U****C**UAGUCACAUG | |
|  |  | AACY01382442.1/386-458  | AUUAUUUUAU**A****G****C****G****C****C**...GAGUU**A**A...........**.****A****A****C**.A**A**AUU**G****C****.****G****G****G****C****G****C****U**..UAACAAAUC**C****A**.**G****C**UAAAGC..**G****U****C****A**AGUCUCAGAC | |
|  |  | AAFX01013962.1/23-97  | CAUCGCCCUC**A****G****U****G****G****U**...GAUUU**G**GCC.........**G****G****U****C**.U**G**GUU**G****C****.****A****G****C****C****A****C****U**..UUAAAUAAA**U****G**.**G****C**UAAGA...**G****A****C****C**GUUCAACCGG | |
|  |  | AACY01686919.1/581-653  | UAAAUCUCUA**A****G****C****G****C****C**...GAGUU**A**A...........**.****G****G****C**.A**A**CUU**G****C****.****G****G****G****C****G****C****U**..UUAUAAAUC**C****U**.**G****C**UAAAGC..**G****C****A****U**UAGUCAUUUA | |
|  |  | AAFY01015686.1/315-244  | UUAUUAUUUC**U****G****U****G****G****.**AAGGAUUU**G**............**.****A****U****U**..**G**AUU**G****C****.****A****G****C****C****A****C****A**..CUAAACAAA**U****.**.**G****C**UAAAAC..**A****A****U****A**UUUAGCCAGC | |
|  |  | AACY01485965.1/521-447  | AUGAUGUAAU**U****G****U****C****C****U**...GAUUU**A**GAU.........**A****C****U****C**.U**A**AUU**G****C****.****U****G****G****G****A****C****A**.UUAAACAAGU**A****.**.**G****C**UAAAG...**G****A****G****A**AAACAUGCCU | |
|  |  | AACY01339997.1/454-380  | UCGUCAAAUC**A****U****C****G****C****C**...GAUUU**A**AC..........**G****A****U****C**.A**A**CUU**G****C****.****G****G****G****C****G****A****U**..CAAGAAAUA**C****A**.**G****C**UAAAGC..**G****C****U****C**AAGGCCAGCA | |
|  |  | AACY01303559.1/517-441  | AUUAUUAACC**U****G****U****C****C****U**...GAUUU**A**GAAAUA......**.****C****U****C**.U**A**CUU**G****C****.****U****G****G****G****A****C****G**..UUAAAUACA**A****C**.**G****C**UAAAG...**G****A****G****G**CAAUAUGGAC | |
|  |  | AACY01040684.1/56-126  | AUAAUUAAAU**U****G****U****C****G****U**...CAUUU**A**A...........**.****C****U****C**.U**A**CUU**G****C****.****A****A****C****G****A****C****.**.UUAAAAUAAA**U****.**.**G****C**UAAAC...**G****G****A****G**UUAAGAUUUU | |
|  |  | AACY01324379.1/719-793  | AAAUUAUAAA**U****G****U****C****C****C**...GAUUU**A**GACA........**.****C****U****C**.U**A**AUU**G****C****.****U****A****G****G****A****C****A**..AUAAAUAAA**A****A**.**G****C**UAAAG...**G****A****G****C**UAAAAUGACC | |
|  |  | AADL01000001.1/82341-82269  | AAAAAGGCAG**G****G****U****G****G****.**AUUGAUUU**G**A...........**.****G****G****U**.A**U**AUU**G****C****.****A****A****C****C****A****C****C**..UAAAAAAAG**U****.**.**G****C**UAAAA...**G****U****C****C**AGGUCAUUUA | |
|  |  | AADL01000760.1/79-7  | AAAAAGGCAG**G****G****U****G****G****.**AUUGAUUU**G**A...........**.****G****G****U**.A**U**AUU**G****C****.****A****A****C****C****A****C****C**..UAAAAAAAG**U****.**.**G****C**UAAAA...**G****U****C****C**AGGUCAUUUA | |
|  |  | AACY01716874.1/390-462  | UAAAUUUUUA**A****G****C****G****C****C**...GAGUU**A**A...........**.****G****A****C**.A**A**CUU**G****C****.****G****G****G****C****G****C****U**..AUACAAAUC**C****A**.**G****C**UAAAGC..**G****C****A****A**AGUCAAUCUG | |
|  |  | AACY01568908.1/196-123  | AUAAGAAAAU**U****G****U****C****C****U**...GAUUU**A**GAUA........**.****C****U****C**.U**A**AUU**G****C****.****U****G****G****G****A****C****.**.AUUAAACAAG**U****A**.**G****C**UAAA....**G****G****A****G**AGAAAAUGUC | |
|  |  | AACY01708361.1/406-485  | UAAUGUUACU**A****G****U****C****C****U**...GAUUU**A**GAACCUA.....**.****A****C****U**CU**A**CUU**G****C****.****U****A****G****G****A****C****U**..UUAAACAAA**A****C**.**G****C**UAAAGG..**A****G****G****C**AUAUGAAACU | |
|  |  | AACY01194872.1/433-508  | AAAAUAAUAU**U****G****U****C****C****U**...GAUUU**A**GAU.........**.****A****C****U**CU**A**AUU**G****C****.****U****G****G****G****A****C****U**..CAAAACAAA**A****C**.**G****C**UAAAGG..**A****G****A****U**AUUAUGUUCA | |
|  |  | AAFX01007833.1/535-461  | GGCGGCACCA**U****G****C****G****C****C**...GAUUU**G**AG..........**C****A****C****C**.A**G**CUU**G****C****.****G****G****G****C****G****C****.**.GUAACAAAUG**C****A**.**G****C**UAAAGC..**G****G****C****G**GCGAGAAUCG | |
|  |  | AACY01629239.1/834-761  | CUCUCUAAGA**C****G****C****G****C****C**...GAUUU**G**AC..........**.****U****A****C**.A**G**CUU**G****C****.****G****G****G****C****G****C****.**.AAAACAAAUG**C****A**.**G****C**UAAAGC..**G****U****U****G**GGACCGUCCC | |
|  |  | AACY01726413.1/603-677  | GGAUUCCCUU**A****G****C****G****C****C**...GAUUU**G**AAC.........**.****A****U****C**.A**G**CUU**G****C****.****G****G****G****C****G****C****U**..UUACAAGUA**C****G**.**G****C**UAAAGC..**G****A****G****G**UGCAAGUGAA | |
|  |  | AACY01804053.1/537-465  | AUUAAUUUAU**A****G****C****G****C****C**...GAGUU**A**A...........**.****U****A****C**.A**A**AUU**G****C****.****G****G****G****C****G****C****U**..UAACAAAUC**C****A**.**G****C**UAAAGC..**G****U****U****A**AGUCUCAGAC | |
|  |  | AACY01640652.1/678-752  | AAAAUAAUAU**C****G****U****C****C****U**...GAUUU**A**GAUA........**.****C****U****C**.U**A**AUU**G****C****.****U****G****G****G****A****C****U**..CAAAACAAA**A****C**.**G****C**UAAAG...**G****A****G****A**UAUAAUGAUC | |
|  |  | AACY01059804.1/1-72  | .AAAUCCCUC**A****G****C****G****C****U**...GAGUU**A**A...........**.****G****G****C**.A**A**CUU**G****C****.****G****G****G****C****G****C****.**.UUUAUAAAUC**C****U**.**G****C**UAAAGC..**G****C****A****U**AAGUCAUCUA | |
|  |  | AACY01030883.1/1285-1207  | UAAAUUUAUU**A****G****U****C****C****U**...GAUUU**A**GAACUUAG....**.****C****U****C**.U**A**CUU**G****C****.****U****G****G****G****A****C****U**..UUAAACACA**A****C**.**G****C**UAAAG...**G****A****G****A**AAACAUGGCA | |
|  |  | AACY01407375.1/161-83  | UAUUUAAAUU**A****G****U****C****C****U**...GAUUU**A**GAACUUAA....**.****C****U****C**.U**A**CUU**G****C****.****U****G****G****G****A****C****U**..UUAAACACA**A****C**.**G****C**UAAAG...**G****A****G****G**CAAAUGACUA | |
|  |  | AACY01052066.1/732-656  | UUAUUAAAUU**A****G****U****C****C****U**...GAUUU**A**AAAAAA......**.****C****U****U**.U**A**CUU**G****C****.****U****A****G****G****A****C****U**..AUAAACAAA**A****C**.**G****C**UAAAG...**G****A****G****A**AAAAUGAAAC | |
|  |  | AACY01104951.1/3509-3438  | AUAAUCUAUA**U****G****U****C****G****U**...CAUUU**A**A...........**.****C****U****C**.U**A**AUU**G****C****.****A****G****C****G****A****C****.**..UCAAAAUAA**U****U**.**G****C**UAAAUA..**G****A****G****C**AAGGACAUUG | |
|  |  | AACY01471089.1/280-210  | AAAUGGCCUA**G****U****U****C****G****U**...GAUUU**G**............**.****A****U****C**.C**U**AUU**G****C****.****U****G****C****G****A****A****U**..UAAAAGAAU**U****.**.**G****C**UAAAAC..**G****G****A****A**UAUUUUCCCA | |
|  |  | AACY01152099.1/841-780  | ..........**U****G****C****G****C****U**...GAUUU**A**............**.****A****C****U**.G**A**AUU**G****C****.****G****A****G****C****G****C****.**.UUAAAAAAAA**C****C**.**G****C**UAAAAU..**A****G****U****U**UUUUUUCUCA | |
|  |  | AACY01412809.1/636-558  | AUAAUUAUUU**A****G****U****C****C****U**...GAUUU**A**GAAAUUAA....**.****C****U****C**.U**A**CUU**G****C****.****U****G****G****G****A****C****U**..UUAAACACA**A****C**.**G****C**UAAAG...**G****A****G****G**CAAAUGAAAC | |
|  |  | AACY01204343.1/389-316  | UGAUUCUUCA**U****G****C****G****C****C**...GAUUU**G**C...........**.****U****U****C**.U**G**CUU**G****C****.****G****G****G****C****G****C****.**.UUUAUAAAUC**C****A**.**G****C**UAAAGAC.**G****A****C****A**CCAGAGAACC | |
|  |  | AACY01751094.1/701-623  | UAAUCUAAAU**A****G****U****C****C****U**...GAUUU**A**GAAAAAAA....**.****C****U****C**.U**A**CUU**G****C****.****U****G****G****G****A****C****U**..UUAAACACA**A****C**.**G****C**UAAAG...**G****A****G****G**CAAAUGAAAA | |
|  |  | AACY01010400.1/730-652  | AAGAUUAAUU**A****G****U****C****C****U**...GAUUU**A**GAACAUAA....**.****C****U****C**.U**A**CUU**G****C****.****U****A****G****G****A****C****U**..UAAAAUAAA**A****C**.**G****C**UAAAG...**G****A****G****A**AACAUGAAAA | |
|  |  | AACY01211012.1/340-412  | UAAGCCAUCC**A****G****C****G****C****C**...GAGUU**A**A...........**.****G****G****C**.A**A**CUU**G****C****.****G****G****G****C****G****C****U**..UAAUAAAUC**C****A**.**G****C**UAAAGC..**G****C****A****A**AAGUCUUCAG | |
|  |  | AACY01447866.1/139-67  | AUAAGGUCUU**A****G****C****G****C****C**...GAGUU**A**A...........**.****G****G****C**.A**A**CUU**G****C****.****G****G****G****C****G****C****U**..UAAUAAAUC**C****U**.**G****C**UAAAGC..**G****U****C****A**AAGUCUUAUG | |
|  |  | AACY01740961.1/860-789  | UAAGAAUAAA**C****G****A****G****G****.**AUCGAUUU**G**............**.****A****C****U**..**G**AUU**G****C****.****A****A****C****C****U****C****.**.UUUAAAAAAG**U****.**.**G****C**UAAAGC..**A****G****A****U**GCUUUCCCAC | |
|  |  | AACY01228944.1/817-741  | AUAAUUAAUU**A****G****U****C****C****U**...GAUUU**A**AAAAAA......**.****C****U****U**.U**A**CUU**G****C****.****U****G****G****G****A****C****U**..UUAAACACA**A****C**.**G****C**UAAAG...**G****A****G****A**AAAAUGAGAC | |
|  |  | AACY01330004.1/585-646  | AUGUAACUCC**U****G****C****G****C****U**...GAUUU**A**............**.****A****C****U**.G**A**AUU**G****C****.****G****A****G****C****G****C****U**..UAAAAAAAA**C****C**.**G****C**UAAAUA..**A****G****U****U**.......... | |
|  |  | AACY01468151.1/8-78  | AAAUAUGCGC**U****A****U****C****G****U**...GCUUU**G**............**.****A****U****C**.C**U**AUU**G****C****.****A****G****C****G****A****U****G**..UAAAAAAAC**G****.**.**G****C**UAAAUC..**G****G****A****A**AGUGUUUUAA | |
|  |  | AACY01472192.1/508-583  | GUGCCCAAAC**A****U****C****G****C****U**...GAUUU**A**AGU.........**.****A****A****C**.A**A**CUU**G****C****A****G****A****G****C****G****A****U**..UCAUAAAUC**C****U**.**G****C**UAAAGC..**G****U****U****G**GUUUCUCAAU | |
|  |  | AACY01803857.1/95-173  | UAAAAUUAAU**A****G****U****C****C****U**...GAUUU**A**GAACUUAA....**.****C****U****C**.U**A**CUU**G****C****.****U****G****G****G****A****C****U**..UUAAACACA**A****C**.**G****C**UAAAG...**G****A****G****A**AAACAUGGCA | |
|  |  | AACY01280263.1/756-678  | UAAUUAAAUA**A****G****U****C****C****U**...GAUUU**A**GAAAAUAA....**.****C****U****C**.U**A**CUU**G****C****.****U****G****G****G****A****C****U**..UUAAAAACA**A****C**.**G****C**UAAAG...**G****A****G****G**AAUAUGGUUA | |
|  |  | AACY01606755.1/379-455  | GUAUUAAAUU**A****G****U****C****C****U**...GAUUU**A**AAAAAA......**.****C****U****U**.U**A**CUU**G****C****.****U****A****G****G****A****C****U**..AUAAACAAA**A****C**.**G****C**UAAAG...**G****A****G****A**AAAAUGAAAC | |
|  |  | AACY01108391.1/1205-1276  | UGUUUUAAAC**A****G****A****G****G****.**AAGGAUUU**G**............**.****A****C****U**..**G**AUU**G****C****A****A****.****C****C****U****C****U**..UAAAAAAAU**U****.**.**G****C**UAAAGC..**A****G****U****A**AUUCUUUUAC | |
|  |  | AACY01566954.1/260-332  | AUACACCGUU**A****G****C****G****C****C**...GAGUU**A**A...........**.****G****G****C**.A**A**CUU**G****C****.****G****G****G****C****G****C****.**.UUAAUAAAUC**C****U**.**G****C**UAAAGC..**G****U****C****U**AAGUCUUAUG | |
|  |  | AACY01322558.1/490-563  | AAGUGUUAUU**A****G****C****G****C****U**...GAUUU**A**A...........**.****U****U****C**.A**A**AUU**G****C****U****G****A****G****C****G****C****U**..UAAUAAAUC**C****A**.**G****C**UAAAGC..**G****A****C****A**AAGUCUAAUG | |
|  |  | AACY01169741.1/775-697  | AAUUAAAAAC**A****G****U****C****C****U**...GAUUU**A**GAAAUCAA....**.****C****U****C**.U**A**CUU**G****C****.****U****G****G****G****A****C****U**..AUAAAAACA**A****C**.**G****C**UAAAG...**G****A****G****G**AAAAUGACCA | |
|  |  | AACY01030532.1/300-371  | UGUCUUAAAC**A****G****A****G****G****.**AAGGAUUU**G**............**.****A****C****U**..**G**AUU**G****C****A****A****.****C****C****U****C****U**..UAAAAAAAU**U****.**.**G****C**UAAAGC..**A****G****U****A**AUUCUUUUAC | |
|  |  | AACY01532354.1/406-333  | AAGUGUUUAU**A****G****C****G****C****U**...GAUUU**A**............**A****U****U****C**.A**A**AUU**G****C****U****G****A****G****C****G****C****U**..UAACAAAUC**C****A**.**G****C**UAAAGC..**G****A****U****U**AAGUCUCAUG | |
|  |  | AACY01407622.1/759-824  | GUAAAAAAGA**U****A****U****G****C****C**...GAUUU**G**............**A****U****C****C**..**U**AUU**G****C****.****G****G****G****C****A****U****.**......AAAAC**U****.**.**G****C**UAAAAA..**G****G****A****U**UUUUCUAAAA | |
|  |  | AAFZ01001716.1/526-599  | AUGCAGCUUG**C****G****U****G****G****C**...GAUUU**G**UA..........**.****A****C****C**.G**G**AUU**G****U****G****G****G****C****C****A****C****G**..UUAAUCUUA**C****C**.**G****C**UAAAA...**G****G****U****C**GAGGGCAGAG | |
|  |  | AAFZ01015569.1/388-461  | AGACAGCGCG**C****G****U****G****G****C**...GAUUU**G**UU..........**.****G****C****C**.G**G**AUU**G****C****G****G****G****C****C****A****C****A**..GGAAAGAUA**C****C**.**G****C**UAAAA...**G****G****C****C**AGUCGUCGGG | |
|  |  | AACY01128039.1/113-187  | UACUUGUGUU**G****G****C****U****C****C**...GAUUU**G**A...........**.****U****C****C**.A**G**AUU**G****C****G****G****G****G****A****G****C****U**..UUAUAAAUA**C****C**A**G****C**UAAACC..**G****G****A****C**AGACUUCUGA | |
|  |  | AACY01524439.1/369-440  | ACAAAUUAAA**C****G****A****G****G****.**AUCGAUUU**G**............**.****U****C****U**..**G**AUU**G****C****.****A****A****C****C****U****C****.**.UAUAAAAAAG**U****.**.**G****C**UAAAGC..**A****G****U****U**GCUUUCACUA | |
|  |  | AACY01299425.1/796-723  | AAAAGAGAGU**A****G****U****C****C****U**...GAUUU**A**GAUA........**.****C****U****C**.U**A**AUU**G****C****.****U****G****G****G****A****C****.**.AUUAAAC.AA**A****C**.**G****C**UAAAG...**G****A****G****A**UAAAAUGCCU | |
|  |  | AACY01138517.1/127-198  | UUUUACCCUC**A****G****A****G****G****.**AACGAUUU**G**............**.****A****C****U**..**G**AUU**G****C****.****A****A****C****C****U****C****U**..UUAAAAAAG**U****.**.**G****C**UAAAGC..**A****G****U****A**GCUUUCACUU | |
|  |  | AACY01350058.1/153-224  | CAAACUGUUA**A****G****A****G****G****.**AAGGAUUU**G**............**.****A****C****U**..**G**AUU**G****C****.****A****A****C****C****U****C****.**.AUAAAAAAAA**U****.**.**G****C**UAAAGC..**A****G****U****G**UUGCUUUCCA | |
|  |  | AACY01302267.1/417-495  | AAGAUUAGUA**A****G****U****C****C****U**...GAUUU**A**GAACAUAA....**.****C****U****C**.U**A**CUU**G****C****.****U****A****G****G****A****C****U**..UAAAAUAAA**A****C**.**G****C**UAAAG...**G****A****G****A**AACAUGAAAA | |
|  |  | AACY01111178.1/1961-2029  | UAUCAAUAAG**A****A****C****A****C****C**...GAUUU**A**A...........**.****G****C****C**.G**A**CUU**G****C****.****A****G****G****U****G****U****.**....AUAAAAA**U****.**.**G****C**UAAAUA..**G****G****C****A**GUUAGAAAUA | |
|  |  | AACY01026002.1/1144-1066  | UAUUUAAUUC**A****G****U****C****C****U**...GAUUU**A**GAAAUUAA....**.****C****U****C**.U**A**CUU**G****C****.****U****G****G****G****A****C****U**..AUAAAAACA**A****C**.**G****C**UAAAG...**G****A****G****G**AAUAUGGUUA | |
|  |  | AACY01114769.1/340-262  | UAAUUAAAUU**A****G****U****C****C****U**...GAUUU**A**GAAAAUAA....**.****C****U****C**.U**A**CUU**G****C****.****U****G****G****G****A****C****U**..AUAAAAACA**A****C**.**G****C**UAAAG...**G****A****G****G**AAUAUGGUUA | |
|  |  | AACY01781441.1/330-408  | AUAAAUUAUU**A****G****U****C****C****U**...GAUUU**A**GAAAAAAA....**.****C****U****C**.U**A**CUU**G****C****.****U****G****G****G****A****C****.**.UUUAAACACA**A****C**.**G****C**UAAAG...**G****A****G****C**AAAAUGAAAA | |
|  |  | AACY01116221.1/1249-1171  | UAAUUAAAAA**A****G****U****C****C****U**...GAUUU**A**GAAAUCAA....**.****C****U****C**.U**A**CUU**G****C****.****U****G****G****G****A****C****U**..AUAAAAACA**A****C**.**G****C**UAAAG...**G****A****G****G**AAUAUGGUUA | |
|  |  | AACY01238576.1/409-482  | AAGUGUUUAU**A****G****C****G****C****U**...GAUUU**A**A...........**.****U****U****C**.A**A**AUU**G****C****U****G****A****G****C****G****C****U**..UAACAAAUC**C****A**.**G****C**UAAAGC..**G****A****U****U**AAGUCUUAUG | |
|  |  | AACY01195056.1/317-393  | ACAAUUGACU**U****G****U****C****C****U**...GAUUU**A**GAUUAA......**.****C****U****C**.U**A**CUU**G****C****.****U****G****G****G****A****C****.**.UUUAAACAUA**A****C**.**G****C**UAAAG...**G****A****G****A**AAAAUGAAAC | |
|  |  | AACY01703360.1/373-298  | GUGCCCGAAA**A****U****C****G****C****U**...GAUUU**A**AG..........**U****A****A****C**.A**A**CUU**G****C****A****G****A****G****C****G****A****U**..UCAUAAAUC**C****U**.**G****C**UAAAGC..**G****U****U****G**GUUUCUCAAU | |
|  |  | AACY01592380.1/740-672  | UAUGUAUAAG**A****A****C****A****C****C**...GAUUU**A**A...........**.****G****C****C**.G**A**CUU**G****C****.****A****G****G****U****G****U****.**....AUAAAAA**U****.**.**G****C**UAAAUA..**G****G****C****A**GUUAGAAAUA | |
|  |  | AACY01059543.1/138-216  | UUAUACAUUU**A****G****U****C****C****U**...GAUUU**A**GAAAUUAA....**.****C****U****C**.U**A**CUU**G****C****.****U****G****G****G****A****C****U**..UUAAACACA**A****C**.**G****C**UAAAG...**G****A****G****G**CAAAUGAAAC | |
|  |  | AACY01587812.1/691-765  | AAUCCUUACC**U****G****C****U****C****C**...GAUUU**G**A...........**.****U****C****C**.A**G**AUU**G****C****G****G****G****G****A****G****C****.**.UCUAUAAAUA**C****C**A**G****C**UAAACC..**G****G****A****C**AGACUUCUGA | |
|  |  | AACY01015163.1/1170-1092  | ACUAUUUAUU**A****G****U****C****C****U**...GAUUU**A**GAACAUAA....**.****C****U****C**.U**A**CUU**G****C****.****U****A****G****G****A****C****U**..AUAAACAAA**A****C**.**G****C**UAAAG...**G****A****G****A**AACAUGAAAA | |
|  |  | AACY01239580.1/17-95  | UAAAUUAAUC**A****G****U****C****U****U**...GAUUU**A**UAACAUAA....**.****C****U****C**.U**A**CUU**G****C****.****U****A****G****G****A****C****U**..AUAAAAAAA**A****C**.**G****C**UAAAG...**G****A****G****A**AACAUGAAAA | |
|  |  | AACY01753623.1/390-312  | UAUAAAAAGC**A****G****U****C****C****U**...GAUUU**A**GAAAUCAA....**.****C****U****C**.U**A**CUU**G****C****.****U****G****G****G****A****C****U**..UUAAACACA**A****C**.**G****C**UAAAG...**G****A****G****G**AAAAUGACUA | |
|  |  | AACY01043176.1/165-87  | AAAGAAAAAU**A****G****U****C****C****U**...GAUUU**A**GAACUAAA....**.****C****U****C**.U**A**CUU**G****C****.****U****G****G****G****A****C****U**..UAAAAAACA**A****C**.**G****C**UAAAG...**G****A****G****A**CAAAUGAAAA | |
|  |  | AACY01179457.1/26-103  | UUAUCAAUUU**A****G****U****C****U****U**...GAUUU**A**GAAAAAA.....**.****C****U****C**.U**A**CUU**G****C****.****U****A****G****G****A****C****U**..UUAAACAUA**A****C**.**G****C**UAAAG...**G****A****G****G**CAUAUGAAAC | |
|  |  | AACY01723843.1/78-6  | AUUAUUUUAU**A****G****C****G****C****C**...GAGUU**A**A...........**.****U****A****C**.A**A**AUU**G****C****.****G****G****G****C****G****C****U**..CAACAAAUC**C****U**.**G****C**UAAAGC..**G****U****C****A**AAUCUUUACU | |
|  |  | AAFY01010936.1/515-586  | GUCGAGUCGA**U****G****U****G****G****.**AAAGAUUU**G**............**.****A****C****U**..**G**AUU**G****C****.****A****A****C****C****A****C****G**..GUAAAAAAA**U****.**.**G****C**UAAAAC..**A****G****G****C**GCUUAUCGAC | |
|  |  | AACY01262154.1/487-414  | AAGUAUGGGU**A****G****C****G****C****U**...GAUUU**A**A...........**.****U****U****C**.A**A**AUU**G****C****U****G****A****G****C****G****C****U**..UAACAAAUC**C****A**.**G****C**UAAAGC..**G****A****C****U**AAGUCUAAUG | |
|  |  | AACY01076595.1/1125-1054  | UUGAUGCAAC**A****G****A****G****G****.**AAGGAUUU**G**............**.****A****C****U**..**G**AUU**G****C****.****A****A****C****C****U****C****A**..UUAAAAAAU**U****.**.**G****C**UAAAGC..**A****G****U****A**UUUUUUUUGC | |
|  |  | AACY01323302.1/378-455  | AAGAAAUAAU**A****G****U****C****C****U**...GAUUU**A**GAACUAAA....**.****C****U****C**.U**A**CUU**G****C****.****U****G****G****G****A****C****U**..UUAAAAACA**A****C**.**G****C**UAAAG...**G****A****G****A**CAAAUGAAA. | |
|  |  | AACY01577149.1/58-136  | AUUUUAUUUU**A****G****U****C****C****U**...GAUUU**A**GAAAAUAA....**.****C****U****C**.U**A**CUU**G****C****.****U****G****G****G****A****C****U**..UUAAACACA**A****U**.**G****C**UAAAG...**G****A****G****G**CAAAUGAAAA | |
|  |  | AACY01441539.1/121-199  | UAGUUUAAAU**A****G****U****C****C****U**...GAUUU**A**GAAAUUA.....**U****C****U****C**.U**A**CUU**G****C****.****U****G****G****G****A****C****U**..AUAAAAACA**A****C**.**G****C**UAAAG...**G****A****G****G**AAUAUGGUUA | |
|  |  | AACY01119923.1/2403-2336  | GAUUAAAAGU**A****A****C****A****C****C**...GAUUU**A**A...........**.****A****C****C**.A**A**CUU**G****C****.****A****G****G****U****G****U****.**....AUA.AAA**A****C**.**G****C**UAAAA...**G****G****G****A**GUUAAAAAAU | |
|  |  | AACY01618939.1/62-1  | AAAACGUUAU**U****G****C****G****C****U**...GAUUU**A**A...........**.****.****A****C**AA**A**AUU**G****C****.****G****A****G****C****G****C****U**..AUAAAAAAA**C****U**.**G****C**UAAACAA.**G****U****.****.**.......... | |
|  |  | AACY01071586.1/1538-1459  | AAAUAAAACU**A****G****U****C****C****U**...GAUUU**A**GAAAAUUA....**U****C****U****C**.U**A**CUU**G****C****.****U****A****G****G****A****C****U**..AUAAAAAUA**A****C**.**G****C**UAAAG...**G****A****G****G**AAUAUGGUUA | |
|  |  | AACY01295782.1/28-104  | AUAAUAAAAA**U****G****U****C****U****U**...GAUUU**A**GAAAUU......**A****C****U****C**.U**A**CUU**G****C****.****U****G****G****G****A****C****A**..UUAAA.CAA**A****C**.**G****C**UAAAG...**G****A****G****A**AUAUAAUGAA | |
|  |  | AACY01357055.1/846-768  | CAAUUUAAUU**A****G****U****C****C****U**...GAUUU**A**GAAAAUAA....**.****C****U****C**.U**A**CUU**G****C****.****U****G****G****G****A****C****U**..UUAAACUUA**A****C**.**G****C**UAAAG...**G****A****G****G**AACAUGUCUU | |
|  |  | AACY01802493.1/257-335  | AUAUUAAGUC**U****G****U****C****C****U**...GAUUU**A**GAAAAUAA....**.****C****U****C**.U**A**CUU**G****C****.****U****G****G****G****A****C****.**.UUUAAACACA**A****C**.**G****C**UAAAG...**G****A****G****G**CAAAUGAAAA | |
|  |  | AACY01083726.1/233-311  | AUAUUAAGUC**A****G****U****C****C****U**...GAUUU**A**GAAAUUAA....**.****C****U****C**.U**A**CUU**G****C****.****U****G****G****G****A****C****U**..AUAAAAACA**A****C**.**G****C**UAAAG...**G****A****G****G**AAUAUGGUUA | |
|  |  | AACY01703360.1/474-401  | GACUUACAAA**A****A****C****G****C****C**...GAUUU**G**A...........**.****C****U****C**.A**G**CUU**U****C****A****G****G****G****C****G****U****U**..UCACAAAUA**C****U**.**G****C**UAAAGA..**G****A****G****G**GUAACCUGCU | |
|  |  | AACY01218646.1/619-554  | UAAAUUUAAU**U****U****U****G****C****C**...GAUUU**G**A...........**.****U****C****C**..**U**AUU**G****C****.****G****G****G****C****A****U****.**.......AUAA**C****U**.**G****C**UAAACA..**G****G****A****A**UUUUCAUAAC | |
|  |  | AACY01601258.1/406-484  | AUAAUCAAAU**U****G****U****C****C****U**...GAUUU**A**GAAAAUAA....**.****C****U****C**.U**A**CUU**G****C****.****U****G****G****G****A****C****.**.UUAAAACAUA**A****C**.**G****C**UAAAG...**G****A****G****A**AAAAUGAAAC | |
|  |  | AACY01647250.1/391-469  | AUAUUAAAUU**A****G****U****C****C****U**...GAUUU**A**GAAAAUAA....**.****C****U****C**.U**A**CUU**G****C****.****U****G****G****G****A****C****U**..UAAAACACA**A****C**.**G****C**UAAAG...**G****A****G****A**AAAAUGAAAC | |
|  |  | AACY01663926.1/254-333  | AAAUAAAUUU**A****G****U****C****C****U**...GAUUU**A**GAAAAUUA....**U****C****U****C**.U**A**CUU**G****C****.****U****G****G****G****A****C****U**..AUAAAAACA**A****C**.**G****C**UAAAG...**G****A****G****G**AAUAUGGUUA | |
|  |  | AACY01013355.1/513-591  | AUAAUAAACC**U****G****U****C****C****U**...GAUUU**A**GAAAAUAA....**.****C****U****C**.U**A**CUU**G****C****.****U****G****G****G****A****C****.**.UUUAAACACA**A****C**.**G****C**UAAAG...**G****A****G****A**AAAAUGAAAC | |
|  |  | AACY01173581.1/866-788  | AUGAUUAACU**A****G****U****C****U****U**...GAUUU**A**GAACAUGC....**.****C****U****C**.U**A**CUU**G****C****.****U****A****A****G****A****C****U**..UUAAAUAAA**A****C**.**G****C**UAAAG...**G****A****G****A**AACAUGAAAA | |
|  |  | AAFY01001997.1/156-229  | GUACCAGUUA**A****G****U****G****G****A**.UAAAUUU**G**A...........**G****A****U****U**..**G**AUU**G****C****.****A****A****C****C****A****C****.**.AUAAAAAAAA**U****.**.**G****C**UAAAGC..**A****A****U****C**GCUGCAUGCA | |
|  |  | AACY01193535.1/762-685  | AUAAUUAAAU**U****G****U****C****C****U**...GAUUU**A**GAAUUAC.....**.****C****U****C**.U**A**CUU**G****C****.****U****G****G****G****A****C****.**.UUUAAACACA**A****C**.**G****C**UAAAG...**G****A****G****A**AAAAUGAAAC | |
|  |  | AACY01505952.1/544-622  | AAUAUCAAUU**A****G****U****C****C****U**...GAUUU**A**GAAAUUAA....**.****C****U****C**.U**A**CUU**G****C****.****U****G****G****G****A****C****U**..UAAAACACA**A****C**.**G****C**UAAAG...**G****A****G****A**AACAUGAAAC | |
|  |  | AACY01472192.1/408-481  | GACUUAUAAA**A****A****C****G****C****C**...GAUUU**G**A...........**.****C****U****C**.A**G**CUU**U****C****A****G****G****G****C****G****U****U**..AAACAAAUA**C****U**.**G****C**UAAAGA..**G****A****G****G**GCGACCUGCU | |
|  |  | AACY01387303.1/645-569  | AUAAUUAUAU**A****G****U****C****C****U**...GAUUU**A**GAAAAA......**.****C****U****C**.U**A**CUU**G****C****.****U****U****G****G****A****C****U**..UAAAACACA**A****C**.**G****C**UAAAG...**G****A****G****A**AAAAUGAAAC | |
|  |  | AACY01512031.1/547-469  | AUUAUAAAUU**U****G****U****C****C****U**...GAUUU**A**GAAAAUAA....**.****C****U****C**.U**A**CUU**G****C****.****U****G****G****G****A****C****.**.UUUAAACACA**A****C**.**G****C**UAAAG...**G****A****G****A**AAAAUGAAGC | |
|  |  | AACY01718476.1/775-839  | UAAAUUUUAA**A****U****U****G****C****C**...GAUUU**G**............**.****A****U****C**.C**U**AUU**G****C****.****G****G****G****C****A****A****.**........AAA**C****U**.**G****C**UAAACA..**G****G****A****A**UUUUUCAUUA | |
|  |  | AACY01546227.1/632-554  | AACUGAAAUU**A****G****U****C****C****U**...GAUUU**A**GAAAUCAA....**.****C****U****C**.U**A**CUU**G****C****.****U****G****G****G****A****C****U**..AUAAAAACA**A****C**.**G****C**UAAAG...**G****A****G****G**AAUAUGGUUA | |
|  |  | AACY01014734.1/627-706  | UAACGAGAUC**A****G****U****C****C****U**...GAUUU**A**GAAAAUUA....**U****C****U****C**.U**A**CUU**G****C****.****U****A****G****G****A****C****U**..AUAAAAAUA**A****C**.**G****C**UAAAG...**G****A****G****G**AAUAUGGUUA | |
|  |  | AACY01105960.1/959-1023  | AUAUAUUUAA**C****C****U****G****C****C**...GAUUU**G**A...........**.****U****C****C**..**U**AUU**G****C****.****G****G****G****C****A****U****.**........AAA**C****U**.**G****C**UAAAAC..**G****G****A****A**UUCCCAUAAU | |
|  |  | AACY01555600.1/333-259  | UAAUUGUUAC**U****U****C****G****C****C**...GAUUU**G**A...........**.****G****A****C**.A**G**CUU**G****C****.****G****G****G****C****G****U****U**UAAUUGAAAUA**C****A**.**G****C**UAAUGC..**A****U****U****G**AAUUAAACCU | |
|  |  | AACY01089416.1/2291-2358  | UCAUAUAGAU**A****U****C****G****C****C**...GAUUU**G**AU..........**.****U****U****C**.A**G**CUU**G****C****.****C****G****G****C****G****U****.**.....AAAAUA**.****.**.**G****C**UAAAGC..**G****A****U****C**GGUAAACCAC | |
|  |  | AACY01091224.1/333-410  | AAUUUAAAUA**A****G****U****C****C****U**...GAUUU**A**GAAAUU......**A****C****U****C**.U**A**CUU**G****C****.****U****U****G****G****A****C****U**..UUAAACACA**A****C**.**G****C**UAAAG...**G****A****G****A**AAUAUGAAAA | |
|  |  | AACY01301409.1/171-106  | UAUUUAUCUA**A****U****U****G****C****C**...GAUUU**G**A...........**.****U****C****C**..**U**AUU**G****C****.****G****G****G****C****A****C****U**........AAA**C****U**.**G****C**UAAAAA..**G****G****A****G**AUUUCAUUGA | |
|  |  | AACY01006291.1/1818-1744  | UAAUUAUAAA**U****U****C****G****C****C**...GAUUU**G**A...........**.****G****A****C**.A**G**CUU**G****C****.****G****G****G****C****G****U****U**UAAUUGAAAUA**C****A**.**G****C**UAAUGC..**A****U****U****G**AAUUAAACCU | |
|  |  | AACY01038517.1/807-878  | AAUAAGCAUC**U****A****C****A****C****C**...GAUUU**G**A...........**.****A****A****C**.A**G**CUU**G****C****.****G****G****G****U****G****U****G**...UAUCGAAA**C****A**.**G****C**UAAAGC..**G****U****G****A**UACUCCCCCC | |
|  |  | AACY01139746.1/133-69  | ACAACAUGCA**A****G****U****G****C****C**...GAUUU**A**............**.****G****C****C**.A**A**AUU**G****C****.****A****G****G****C****A****U****.**........AAA**C****U**.**G****C**UAAAAA..**G****G****A****A**AUCGUACAAA | |
|  |  | AACY01048443.1/2035-1964  | UGUUGAUAAC**A****G****A****G****G****.**AAGGAUUU**G**............**.****A****C****U**..**G**AUU**G****C****.****A****A****C****C****U****C****.**..UCAAAAAAA**U****U**.**G****C**UAAAGC..**A****G****U****A**AUUCUUUUAC | |
|  |  | AACY01565532.1/733-665  | AAUACAUAAA**U****A****C****A****C****C**...GAUUU**G**AG..........**.****A****U****C**.A**G**CUU**G****C****.****G****G****G****U****G****U****.**......UAAAA**C****A**.**G****C**UAAAGC..**G****A****A****U**UUUAUGUUUU | |
|  |  | AACY01273759.1/177-248  | AAUAGCUCUC**U****A****C****A****C****C**...GAUUU**G**A...........**.****A****A****C**.A**G**CUU**G****C****.****G****G****G****U****G****U****.**.G.UAUCGAAA**C****A**.**G****C**UAAAGC..**G****U****G****A**UACUCCACAC | |
|  |  | AACY01530672.1/841-918  | UAUAAAUAGU**U****G****U****C****C****U**...GAUUU**A**GAAAAAAA....**C****U****C****U**..**A**CUU**G****C****.****U****G****G****G****A****C****.**.UUUAAAACAC**A****C**.**G****C**UAAA....**G****G****A****G**AAACAUGAAA | |
|  |  | AACY01124935.1/63-128  | CUAGAAAUCU**C****A****U****G****C****C**...GAUUU**G**A...........**.****U****C****C**..**U**AUU**G****C****.****G****G****G****C****G****U****.**.......AAAA**C****U**.**G****C**UAAAAA..**G****G****A****A**UUUUCUAUAA | |
|  |  | AACY01190081.1/659-724  | CUAAAAAUUA**U****A****A****G****C****C**...GAUUU**G**A...........**.****U****C****C**..**U**AUU**G****C****.****G****G****G****C****U****U****.**.......AUAA**C****U**.**G****C**UAAAAA..**G****G****A****A**GUUUCAUAAA | |
|  |  | AACY01519817.1/635-567  | GUAAUAACGG**U****A****C****G****C****C**...GUUUU**G**AAA.........**.****U****U****C**.A**G**CUU**G****C****.****C****G****G****C****G****U****.**.....UAAAUA**.****.**.**G****C**UAAAGC..**G****A****U****C**GGUAAACCAC | |
|  |  | SS\_cons |  | ..........<<<<<<........[............BBBB..]...AAAA>>>>>>...........aa.aa........bbbb.......... |
|  |  | SS\_label |  | ..........==P1==.....................-P2-..........==P1==........................-P2-.......... |
|  |  | pair\_prediction\_1 |  | ........................[..................]................................................... |
|  |  | pair\_prediction\_2 |  | ..................................................<.................>.......................... |
|  |  | RF |  | uAaauuaucccGUGgu...GAUUUGA...........GACC.GGCUUGC.ggcCACg..UUAAAAAAauc.GCUAAAAA..GGUcuuuuuaccac |
|  |  | SS\_align |  | ..........<<<<<<...................................>>>>>>...................................... |
