# Supplementary material for: The distributions, mechanisms, and structures of metabolite-binding riboswitches
Source: Genome Biol. 2007 Nov 12;8(11):R239. doi: 10.1186/gb-2007-8-11-r239 (PMC2258182; doi:10.1186/gb-2007-8-11-r239)
Supplement: Additional data file 2 — Sequence alignments of the riboswitch aptamer data sets annotated with new base-base interactions in HTML format. [file gb-2007-8-11-r239-S2.zip › HTML/TPP.html]

|  |  |  |  |  |
| --- | --- | --- | --- | --- |
|  |  | **Accession/Start-End** |  | **Sequence** |
|  |  | NC\_006510.1/643262-643362  | **A****U****C****A****U****C****C****A****C****U****A**..**G**.**G****G****G**.**G****G****C****C****U****U****U**AG-..........................................................................................................................................................................-**A****A****A****G****G****C****U**GAG**A**.**U****C****A****A****A**GUGU...............................................................................GCC**U****U****U**.**G****A**...........**G****A****C****C****C****U**UA.........................GC**A**.**C****C****U****G**A.U.**C****U****G****G**.GUAAU...............................G.**C****C****A****G**C**G**.U**A****G****G**.**G**A....**A****G****U****G****G****A****G****G****A****G** | |
|  |  | NC\_004193.1/678921-678825  | **A****A****A****A****U****C****C****A****C****U****A**..**G**.**G****G****G**.**U****G****C****C****A****U**----..........................................................................................................................................................................-**U****U****U****G****G****C****U**GAG**A**.**U****A****A****G****A**UGCA................................................................................UU**U****C****U**.**U****G**...........**A****U****C****C****C****U**UU.........................GA**A**.**C****C****U****G**A.A.**C****U****G****G**.UUAAU...............................A.**C****C****A****G**C**G**.U**A****G****G**.**A**A....**A****G****U****G****G****A****G****U****U****G** | |
|  |  | NC\_006510.1/411989-412095  | **C****U****U****A****U****C****U****G****C****U****A**..**G**.**G****G****G**.**A****G****U****C****C****G****A**GA-..........................................................................................................................................................................G**C****C****G****G****G****C****U**GAG**A**.**A****A****A****G****A**ACGC..........................................................................GAUGAAGU**U****C****U**.**U****U**...........**G****A****C****C****C****U**UU.........................GA**A**.**C****C****U****G**A.U.**C****U****G****G**.GUCAU...............................G.**C****C****A****G**C**G**.U**A****G****G**.**G**A....**A****G****C****A****G****G****C****A****G****C** | |
|  |  | NZ\_AAEK01000033.1/47712-47810  | **U****U****U****A****U****C****C****A****C****U****A**..**G**.**G****G****G**.**G****G****C****C****U****A****U**U--..........................................................................................................................................................................-**A****U****A****G****G****C****U**GAG**A**.**U****C****A****A****A**UGGG................................................................................AA**U****U****U**.**G****A**...........**G****A****C****U****C****U**UA.........................GU**A**.**C****C****U****G**A.U.**C****U****G****G**.UUAAU...............................G.**C****C****A****G**C**G**.U**A****G****G**.**G**A....**A****G****U****G****G****A****A****A****A****G** | |
|  |  | NC\_003909.8/2504738-2504836  | **U****U****U****A****U****C****C****A****C****U****A**..**G**.**G****G****G**.**G****G****C****C****U****A****U**U--..........................................................................................................................................................................-**A****U****A****G****G****C****U**GAG**A**.**U****C****A****A****A**UUGG................................................................................AA**U****U****U**.**G****A**...........**G****A****C****U****C****U**UA.........................GU**A**.**C****C****U****G**A.U.**C****U****G****G**.UUAAU...............................G.**C****C****A****G**C**G**.U**A****G****G**.**G**A....**A****G****U****G****G****G****A****A****A****G** | |
|  |  | NC\_003997.3/2433591-2433689  | **U****U****U****A****U****C****C****A****C****U****A**..**G**.**G****G****G**.**G****G****C****C****U****A****U**U--..........................................................................................................................................................................-**A****U****A****G****G****C****U**GAG**A**.**U****C****A****A****A**UUGG................................................................................AA**U****U****U**.**G****A**...........**G****A****C****U****C****U**UA.........................GU**A**.**C****C****U****G**A.U.**C****U****G****G**.UUAAU...............................G.**C****C****A****G**C**G**.U**A****G****G**.**G**A....**A****G****U****G****G****G****A****A****A****G** | |
|  |  | NC\_005945.1/2433643-2433741  | **U****U****U****A****U****C****C****A****C****U****A**..**G**.**G****G****G**.**G****G****C****C****U****A****U**U--..........................................................................................................................................................................-**A****U****A****G****G****C****U**GAG**A**.**U****C****A****A****A**UUGG................................................................................AA**U****U****U**.**G****A**...........**G****A****C****U****C****U**UA.........................GU**A**.**C****C****U****G**A.U.**C****U****G****G**.UUAAU...............................G.**C****C****A****G**C**G**.U**A****G****G**.**G**A....**A****G****U****G****G****G****A****A****A****G** | |
|  |  | NC\_006274.1/2490752-2490850  | **U****U****U****A****U****C****C****A****C****U****A**..**G**.**G****G****G**.**G****G****C****C****U****A****U**U--..........................................................................................................................................................................-**A****U****A****G****G****C****U**GAG**A**.**U****C****A****A****A**UUGG................................................................................AA**U****U****U**.**G****A**...........**G****A****C****U****C****U**UA.........................GU**A**.**C****C****U****G**A.U.**C****U****G****G**.UUAAU...............................G.**C****C****A****G**C**G**.U**A****G****G**.**G**A....**A****G****U****G****G****G****A****A****A****G** | |
|  |  | NC\_007530.2/2433715-2433813  | **U****U****U****A****U****C****C****A****C****U****A**..**G**.**G****G****G**.**G****G****C****C****U****A****U**U--..........................................................................................................................................................................-**A****U****A****G****G****C****U**GAG**A**.**U****C****A****A****A**UUGG................................................................................AA**U****U****U**.**G****A**...........**G****A****C****U****C****U**UA.........................GU**A**.**C****C****U****G**A.U.**C****U****G****G**.UUAAU...............................G.**C****C****A****G**C**G**.U**A****G****G**.**G**A....**A****G****U****G****G****G****A****A****A****G** | |
|  |  | NZ\_AAAC02000001.1/2908503-2908601  | **U****U****U****A****U****C****C****A****C****U****A**..**G**.**G****G****G**.**G****G****C****C****U****A****U**U--..........................................................................................................................................................................-**A****U****A****G****G****C****U**GAG**A**.**U****C****A****A****A**UUGG................................................................................AA**U****U****U**.**G****A**...........**G****A****C****U****C****U**UA.........................GU**A**.**C****C****U****G**A.U.**C****U****G****G**.UUAAU...............................G.**C****C****A****G**C**G**.U**A****G****G**.**G**A....**A****G****U****G****G****G****A****A****A****G** | |
|  |  | NZ\_AAEN01000016.1/732146-732244  | **U****U****U****A****U****C****C****A****C****U****A**..**G**.**G****G****G**.**G****G****C****C****U****A****U**U--..........................................................................................................................................................................-**A****U****A****G****G****C****U**GAG**A**.**U****C****A****A****A**UUGG................................................................................AA**U****U****U**.**G****A**...........**G****A****C****U****C****U**UA.........................GU**A**.**C****C****U****G**A.U.**C****U****G****G**.UUAAU...............................G.**C****C****A****G**C**G**.U**A****G****G**.**G**A....**A****G****U****G****G****G****A****A****A****G** | |
|  |  | NZ\_AAEP01000025.1/531751-531849  | **U****U****U****A****U****C****C****A****C****U****A**..**G**.**G****G****G**.**G****G****C****C****U****A****U**U--..........................................................................................................................................................................-**A****U****A****G****G****C****U**GAG**A**.**U****C****A****A****A**UUGG................................................................................AA**U****U****U**.**G****A**...........**G****A****C****U****C****U**UA.........................GU**A**.**C****C****U****G**A.U.**C****U****G****G**.UUAAU...............................G.**C****C****A****G**C**G**.U**A****G****G**.**G**A....**A****G****U****G****G****G****A****A****A****G** | |
|  |  | NZ\_AAEQ01000035.1/799909-799811  | **U****U****U****A****U****C****C****A****C****U****A**..**G**.**G****G****G**.**G****G****C****C****U****A****U**U--..........................................................................................................................................................................-**A****U****A****G****G****C****U**GAG**A**.**U****C****A****A****A**UUGG................................................................................AA**U****U****U**.**G****A**...........**G****A****C****U****C****U**UA.........................GU**A**.**C****C****U****G**A.U.**C****U****G****G**.UUAAU...............................G.**C****C****A****G**C**G**.U**A****G****G**.**G**A....**A****G****U****G****G****G****A****A****A****G** | |
|  |  | NZ\_AAER01000040.1/164459-164557  | **U****U****U****A****U****C****C****A****C****U****A**..**G**.**G****G****G**.**G****G****C****C****U****A****U**U--..........................................................................................................................................................................-**A****U****A****G****G****C****U**GAG**A**.**U****C****A****A****A**UUGG................................................................................AA**U****U****U**.**G****A**...........**G****A****C****U****C****U**UA.........................GU**A**.**C****C****U****G**A.U.**C****U****G****G**.UUAAU...............................G.**C****C****A****G**C**G**.U**A****G****G**.**G**A....**A****G****U****G****G****G****A****A****A****G** | |
|  |  | NZ\_AAES01000035.1/334046-334144  | **U****U****U****A****U****C****C****A****C****U****A**..**G**.**G****G****G**.**G****G****C****C****U****A****U**U--..........................................................................................................................................................................-**A****U****A****G****G****C****U**GAG**A**.**U****C****A****A****A**UUGG................................................................................AA**U****U****U**.**G****A**...........**G****A****C****U****C****U**UA.........................GU**A**.**C****C****U****G**A.U.**C****U****G****G**.UUAAU...............................G.**C****C****A****G**C**G**.U**A****G****G**.**G**A....**A****G****U****G****G****G****A****A****A****G** | |
|  |  | NZ\_AAEO01000022.1/391822-391920  | **U****U****U****A****U****C****C****A****C****U****A**..**G**.**G****G****G**.**G****G****C****C****U****A****U**U--..........................................................................................................................................................................-**A****U****A****G****G****C****U**GAG**A**.**U****C****A****A****A**UUGG................................................................................AA**U****U****U**.**G****A**...........**G****A****C****U****C****U**UA.........................GC**A**.**C****C****U****G**A.U.**C****U****G****G**.UUAAU...............................G.**C****C****A****G**C**G**.U**A****G****G**.**G**A....**A****G****U****G****G****G****A****A****A****G** | |
|  |  | NC\_005957.1/2481747-2481845  | **U****U****U****A****U****C****C****A****C****U****A**..**G**.**G****G****G**.**G****G****C****C****U****A****U**U--..........................................................................................................................................................................-**A****U****A****G****G****C****U**GAG**A**.**U****C****A****A****A**UGGG................................................................................AA**U****U****U**.**G****A**...........**G****A****C****U****C****U**UA.........................GU**A**.**C****C****U****G**A.U.**C****U****G****G**.UUAAU...............................G.**C****C****A****G**C**G**.U**A****G****G**.**G**A....**A****G****U****G****G****G****A****A****A****G** | |
|  |  | NC\_003366.1/1581046-1580944  | **A****A****A****A****A****U****A****G****C****U****A**..**G**.**G****G****G**.**G****G****C****C****A****G****U**AGU..........................................................................................................................................................................G**U****C****U****G****G****C****U**GAG**A**U**U****A****G****A****A**AUGA...............................................................................AAU**U****U****C**.**U****U**...........**G****A****C****C****C****U**U-.........................UA**A**.**C****C****U****G**A.U.**C****U****G****G**.UUAAU...............................A.**C****C****A****G**C**G**.U**A****G****G**.**G**A....**A****G****C****C****C****A****A****G****A****G** | |
|  |  | NC\_006582.1/829775-829683  | **A****A****G****A****A****C****C****A****C****U****A**..**G**.**G****G****G**.**A****G****C****C****A****U**----..........................................................................................................................................................................--**C****U****G****G****C****U**GAG**A**.**A****A****A****G****C**GCU-..................................................................................**G****C****U**.**U****U**...........**G****A****C****C****C****U**UU.........................GA**A**.**C****C****U****G**A.U.**C****U****G****G**.CUUGU...............................A.**C****C****A****G**C**G**.U**A****G****G**.**G**A....**A****G****U****G****G****A****G****A****A****C** | |
|  |  | NC\_005835.1/302202-302304  | **G****G****G****G****C****A****G****G****C****U****A**..**G**.**G****G****G**.**U****G****C****C****C****G****A**AUG..........................................................................................................................................................................G**A****A****G****G****G****C****U**GAG**A**.**G****C****U****G****G**GUUU...............................................................................CUC**C****C****A**.**G****C**...........**A****A****C****C****C****U**UG.........................GA**A**.**C****C****U****G**A.U.**C****C****G****G**.GUAAU...............................G.**C****C****G****G**C**G**.G**A****G****G**.**G**A....**A****G****C****C****U****A****U****G****C****G** | |
|  |  | NC\_006461.1/640042-640144  | **G****G****G****G****C****A****G****G****C****U****A**..**G**.**G****G****G**.**U****G****C****C****C****G****A**AUG..........................................................................................................................................................................G**A****A****G****G****G****C****U**GAG**A**.**G****C****U****G****G**GUUU...............................................................................CUC**C****C****A**.**G****C**...........**A****A****C****C****C****U**UG.........................GA**A**.**C****C****U****G**A.U.**C****C****G****G**.GUAAU...............................G.**C****C****G****G**C**G**.G**A****G****G**.**G**A....**A****G****C****C****U****A****U****G****C****G** | |
|  |  | NC\_002689.2/1310823-1310722  | **C****A****G****U****U****C****A****U****A****A****A**..**G**.**G****G****G**.**A****G****C****U****U****C****G**UA-..........................................................................................................................................................................-**G****G****A****G****G****C****U**GAG**A**G**G****A****U****C****C**GAGG.................................................................................A**G****G****A**.**U****C**...........**G****A****C****C****C****U**UU.........................GA**A**.**C****C****U****G**A.U.**C****C****G****G**.GUAAU...............................G.**C****C****G****G**C**G**.G**A****G****G**.**G**A..AA**U****U****A****U****G****U****C****G****G****A** | |
|  |  | NZ\_AAAP01003877.1/60656-60551  | **A****U****C****U****U****U****C****C****G****A****A**..**G**.**G****G****G**.**A****G****C****C****C****C****G**AUC..........................................................................................................................................................................G**A****G****G****G****G****C****U**GAG**A**G**G****C****C****G****G**UCAU.............................................................................CAGAG**C****C****G**.**G****C**...........**G****A****C****C****C****U**UG.........................GA**A**.**C****C****U****G**A.U.**C****C****G****G**.GUCAU...............................U.**C****C****G****G**C**G**.A**A****G****G**.**G**A....**U****C****G****G****A****A****C****C****A****A** | |
|  |  | NC\_006270.2/4036920-4036823  | **G****A****A****G****U****C****C****A****C****U****A**..**G**.**G****G****G**.**A****G****C****C****G****U****A**A--..........................................................................................................................................................................-**G****C****C****G****G****C****U**GAG**A**.**C****A****A****G****A**AGCG.................................................................................A**U****C****U**.**U****G**...........**G****A****C****C****C****U**UU.........................GA**A**.**C****C****U****G**A.U.**C****U****A****G**.UUCAU...............................A.**C****U****A****G**C**G**.U**A****G****G**.**G**A....**A****G****U****G****G****A****G****C****C****G** | |
|  |  | NC\_006322.1/4037034-4036937  | **G****A****A****G****U****C****C****A****C****U****A**..**G**.**G****G****G**.**A****G****C****C****G****U****A**A--..........................................................................................................................................................................-**G****C****C****G****G****C****U**GAG**A**.**C****A****A****G****A**AGCG.................................................................................A**U****C****U**.**U****G**...........**G****A****C****C****C****U**UU.........................GA**A**.**C****C****U****G**A.U.**C****U****A****G**.UUCAU...............................A.**C****U****A****G**C**G**.U**A****G****G**.**G**A....**A****G****U****G****G****A****G****C****C****G** | |
|  |  | NC\_004722.1/2525752-2525850  | **U****U****C****A****U****C****C****A****C****U****A**..**G**.**G****G****G**.**G****G****C****C****U****U****U**U--..........................................................................................................................................................................-**A****U****A****G****G****C****U**GAG**A**.**U****C****A****A****A**UGUG................................................................................AU**U****U****U**.**G****A**...........**G****A****C****U****C****U**UA.........................GU**A**.**C****C****U****G**A.U.**C****U****G****G**.UUAAU...............................G.**C****C****A****G**C**G**.U**A****G****G**.**G**A....**A****G****U****G****G****G****A****A****A****G** | |
|  |  | NZ\_AAIT01000006.1/234799-234703  | **C****U****C****A****U****A****C****A****C****U****U**..**G**.**G****G****G**.**A****G****C****C****U****C****G**CA-..........................................................................................................................................................................-**C****G****A****G****G****C****U**GAG**A**.**G****G****U****G****C**CGG-..................................................................................**G****C****A**.**C****C**...........**G****A****C****C****C****A**UC.........................GA**A**.**C****C****U****G**A.U.**C****C****G****G**.GUCAU...............................G.**C****C****G****G**C**G**.U**A****G****G**.**G**A....**A****G****G****G****U****A****G****A****G****U** | |
|  |  | NC\_003869.1/2140374-2140279  | **U****C****A****A****A****G****U****G****C****U****A**..**G**.**G****G****G**.**A****G****C****C****A****G****A**---..........................................................................................................................................................................-**A****A****U****G****G****C****U**GAG**A**G**G****G****G****A****U**UCGU..................................................................................-**U****C**.**C****C**...........**G****A****C****C****C****U**UA.........................GA**A**.**C****C****U****G**A.C.**C****A****G****G**.GUAAU...............................G.**C****C****U****G**C**G**.A**A****G****G**.**G**A....**A****G****C****A****C****G****U****U****U****U** | |
|  |  | NC\_004310.3/228052-227953  | **C****A****A****A****A****U****U****C****G****U****U**..**G**.**G****G****G**.**U****G****C****C****U****U****G**AA-..........................................................................................................................................................................-**A****G****A****G****G****C****U**GAG**A**.**G****A****C****G****C**AUUC................................................................................UC**G****C****G**.**U****C**...........**A****A****C****C****C****A**UU.........................GA**A**.**C****C****U****G**A.U.**C****C****G****G**.GUAAU...............................A.**C****C****U****G**C**G**.U**A****G****G**.**G**A....**A****C****G****G****A****G****U****U****U****G** | |
|  |  | NC\_006932.1/229609-229510  | **C****A****A****A****A****U****U****C****G****U****U**..**G**.**G****G****G**.**U****G****C****C****U****U****G**AA-..........................................................................................................................................................................-**A****G****A****G****G****C****U**GAG**A**.**G****A****C****G****C**AUUC................................................................................UC**G****C****G**.**U****C**...........**A****A****C****C****C****A**UU.........................GA**A**.**C****C****U****G**A.U.**C****C****G****G**.GUAAU...............................A.**C****C****U****G**C**G**.U**A****G****G**.**G**A....**A****C****G****G****A****G****U****U****U****G** | |
|  |  | NC\_004193.1/505048-504940  | **A****G****C****A****A****C****U****A****C****U****A**..**G**.**G****G****G**.**U****G****C****C****C****A****G**UACUAUG......................................................................................................................................................................U**G****U****G****G****G****C****U**GAG**A**.**G****G****A****A****A**GCAC..............................................................................CUAC**U****U****U**.**C****C**...........**G****A****C****U****C****U**UAU........................GG**A**.**C****C****U****G**A.U.**C****U****G****G**.UUAAU...............................A.**C****C****A****G**C**G**.U**A****G****G**.**G**A....**A****G****U****A****G****U****C****A****A****U** | |
|  |  | NC\_002947.3/5596334-5596235  | **A****U****G****U****U****C****U****U****G****U****C**..**G**.**G****G****G**.**U****G****C****C****U****U****G**AAG..........................................................................................................................................................................C**A****A****A****G****G****C****U**GAG**A**.**U****C****G****C****A**GAGG..................................................................................**U****G****C**.**G****G**...........**A****U****C****C****C****G**UU.........................GA**A**.**C****C****U****G**A.U.**C****A****G****G**.UUAGC...............................G.**C****C****U****G**C**G**.U**A****G****G**.**G**A....**A****C****A****A****G****A****U****U****G****C** | |
|  |  | NC\_002976.3/1744175-1744084  | **A****U****U****U****G****C****U****A****C****U****A**..**G**.**G****G****G**.**A****G****C****C****A****A**----..........................................................................................................................................................................--**A****A****G****G****C****U**GAG**A**.**U****G****A****A****U**GUA-..................................................................................-**U****U**.**C****A**...........**G****A****C****C****C****U**UA.........................UA**A**.**C****C****U****G**A.U.**U****U****G****G**.UUAAU...............................A.**C****C****A****A**C**G**.U**A****G****G**.**A**A....**A****G****U****A****G****U****U****A****U****U** | |
|  |  | NC\_004461.1/1749151-1749060  | **A****U****U****U****G****C****U****A****C****U****A**..**G**.**G****G****G**.**A****G****C****C****A****A**----..........................................................................................................................................................................--**A****A****G****G****C****U**GAG**A**.**U****G****A****A****U**GUA-..................................................................................-**U****U**.**C****A**...........**G****A****C****C****C****U**UA.........................UA**A**.**C****C****U****G**A.U.**U****U****G****G**.UUAAU...............................A.**C****C****A****A**C**G**.U**A****G****G**.**A**A....**A****G****U****A****G****U****U****A****U****U** | |
|  |  | NZ\_AABG04000011.1/50728-50630  | **U****C****A****U****C****A****C****G****C****U****A**..**G**.**G****G****G**.**U****G****C****C****A****U**----..........................................................................................................................................................................-**U****G****U****G****G****C****U**GAG**A**.**A****A****G****A****G**ACUU..............................................................................AAGU**C****U****C**.**U****U**...........**A****A****C****C****C****U**UU.........................GA**A**.**C****C****U****G**A.U.**G****C****G****G**.UUAGU...............................A.**C****C****G****C**C**G**.G**A****G****G**.**G**A....**A****G****C****G****G****A****A****A****A****U** | |
|  |  | NC\_004557.1/877068-877169  | **A****A****A****U****A****A****A****U****U****U****A**..**G**.**G****G****G**.**A****G****C****U****U****A****U**AAA..........................................................................................................................................................................A**A****U****A****G****G****C****U**GAG**A**A**A****G****A****G****A**UGUU.................................................................................A**U****C****U**.**C****U**...........**G****A****C****C****C****U**CA.........................UA**A**.**C****C****U****G**A.U.**U****U****G****G**.CUAAU...............................A.**C****C****A****A**C**G**.U**A****G****G**.**G**A....**A****C****A****U****U****G****U****G****U****A** | |
|  |  | NC\_000853.1/809942-810044  | **A****C****C****U****U****C****C****C****C****A****G**..**G**.**G****G****G**.**A****G****C****U****C****C****U**AUU..........................................................................................................................................................................C**C****G****G****G****G****C****U**GAG**A**G**G****A****G****G****A**CGGA................................................................................AG**U****C****C**.**U****C**...........**G****A****C****C****C****C**AA.........................GA**A**.**C****C****U****G**A.U.**C****C****G****G**.GUAAU...............................G.**C****C****G****G**C**G**.G**A****G****G**.**G**A....**U****C****G****G****G****G****A****A****G****G** | |
|  |  | NC\_003869.1/1524796-1524701  | **C****U****C****A****A****G****U****G****C****U****A**..**G**.**G****G****G**.**A****G****C****C****A****A****A**---..........................................................................................................................................................................-**A****A****U****G****G****C****U**GAG**A**G**G****G****G****A****U**UCGU..................................................................................-**U****C**.**C****C**...........**G****A****C****C****C****U**UA.........................GA**A**.**C****C****U****G**A.C.**C****A****G****G**.GUAAU...............................G.**C****C****U****G**C**G**.A**A****G****G**.**G**A....**A****G****C****A****C****G****U****U****U****U** | |
|  |  | NC\_003869.1/1681524-1681429  | **C****U****C****A****A****G****U****G****C****U****A**..**G**.**G****G****G**.**A****G****C****C****A****G****A**---..........................................................................................................................................................................-**A****A****U****G****G****C****U**GAG**A**G**G****G****G****A****U**UCGU..................................................................................-**U****C**.**C****C**...........**G****A****C****C****C****U**UA.........................GA**A**.**C****C****U****G**A.C.**C****A****G****G**.GUAAU...............................G.**C****C****U****G**C**G**.A**A****G****G**.**G**A....**A****G****C****A****C****G****U****U****U****U** | |
|  |  | NC\_003366.1/2917472-2917377  | **C****U****U****U****A****U****A****G****C****U****A**..**G**.**G****G****G**.**U****G****C****C****U**-----..........................................................................................................................................................................--**U****U****G****G****C****U**GAG**A**.**G****A****U****A****A**CAUU...............................................................................UCG**U****U****A**.**U****U**...........**A****A****C****C****C****U**UA.........................AA**A**.**C****C****U****G**A.U.**C****U****G****G**.AUAAU...............................A.**C****C****A****G**C**G**.U**A****G****G**.**A**A....**A****G****C****C****U****G****U****A****G****A** | |
|  |  | NC\_006177.1/179294-179392  | **C****C****G****A****U****C****C****A****C****U****A**..**G**.**G****G****G**.**A****G****C****C****U****U**----..........................................................................................................................................................................--**C****G****G****G****C****U**GAG**A**A**C****A****G****G****U**CUGC..............................................................................CGAG**A****C****C**.**U****G**...........**G****A****C****C****C****U**UA.........................GA**A**.**C****C****U****G**A.A.**C****U****G****G**.GUAAU...............................A.**C****C****A****G**C**G**.G**A****G****G**.**G**A....**A****G****U****G****G****A****C****G****G****C** | |
|  |  | NC\_002947.3/3613932-3614039  | **A****G****C****G****C****C****A****C****C****A****A**..**G**.**G****G****G**.**A****G****C****C****C****G****G**CA-..........................................................................................................................................................................-**A****U****G****G****G****C****U**GAG**A**A**A****C****C****G****C**UGAA.........................................................................CGCUACAGC**G****C****G**.**G****U**...........**G****A****C****C****C****U**UC.........................GA**A**.**C****C****U****G**A.U.**C****C****G****G**.AUCAU...............................G.**C****C****G****G**C**G**.A**A****G****G**.**G**A....**U****G****G****G****G****C****U****U****G****U** | |
|  |  | NZ\_AADW02000001.1/280183-280091  | **C****A****C****A****U****C****C****A****C****U****A**..**G**.**G****G****G**.**A****G****C****C****C****G**----..........................................................................................................................................................................-**C****U****C****G****G****C****U**GAG**A**.**C****G****A****U****U**UACU..................................................................................--**U**.**C****G**...........**G****A****C****C****C****U**UU.........................GA**A**.**C****C****U****G**A.U.**C****U****A****G**.UUCAU...............................A.**C****U****A****G**C**G**.U**A****G****G**.**A**A....**A****G****U****G****G****A****G****C****G****G** | |
|  |  | NC\_003909.8/4930919-4930811  | **G****U****G****A****U****A****A****A****C****U****A**..**G**.**G****G****G**.**U****G****C****C****U****A****A**CGUAUG.......................................................................................................................................................................C**G****U****A****G****G****C****U**GAG**A**.**G****A****G****A****A**GCGC............................................................................GUGAAC**U****U****C**.**U****U**...........**A****A****C****C****C****U**UU.........................GG**A**.**C****C****U****G**A.U.**C****U****G****G**.CUCGU...............................A.**C****C****A****G**C**G**.U**A****G****G**.**G**A....**A****G****U****U****A****A****C****G****G****C** | |
|  |  | NC\_003997.3/4953502-4953394  | **G****U****G****A****U****A****A****A****C****U****A**..**G**.**G****G****G**.**U****G****C****C****U****A****A**CGUAUG.......................................................................................................................................................................C**G****U****A****G****G****C****U**GAG**A**.**G****A****G****A****A**GCGC............................................................................GUAAAC**U****U****C**.**U****U**...........**A****A****C****C****C****U**UU.........................GG**A**.**C****C****U****G**A.U.**C****U****G****G**.CUCGU...............................A.**C****C****A****G**C**G**.U**A****G****G**.**G**A....**A****G****U****U****A****A****C****G****G****C** | |
|  |  | NC\_004722.1/5132191-5132083  | **G****U****G****A****U****A****A****A****C****U****A**..**G**.**G****G****G**.**U****G****C****C****U****A****A**CGUAUG.......................................................................................................................................................................C**G****U****A****G****G****C****U**GAG**A**.**G****A****G****A****A**GCGC............................................................................GUGAAC**U****U****C**.**U****U**...........**A****A****C****C****C****U**UU.........................GG**A**.**C****C****U****G**A.U.**C****U****G****G**.CUCGU...............................A.**C****C****A****G**C**G**.U**A****G****G**.**G**A....**A****G****U****U****A****A****C****G****G****C** | |
|  |  | NC\_005945.1/4954839-4954731  | **G****U****G****A****U****A****A****A****C****U****A**..**G**.**G****G****G**.**U****G****C****C****U****A****A**CGUAUG.......................................................................................................................................................................C**G****U****A****G****G****C****U**GAG**A**.**G****A****G****A****A**GCGC............................................................................GUAAAC**U****U****C**.**U****U**...........**A****A****C****C****C****U**UU.........................GG**A**.**C****C****U****G**A.U.**C****U****G****G**.CUCGU...............................A.**C****C****A****G**C**G**.U**A****G****G**.**G**A....**A****G****U****U****A****A****C****G****G****C** | |
|  |  | NC\_005957.1/4959383-4959275  | **G****U****G****A****U****A****A****A****C****U****A**..**G**.**G****G****G**.**U****G****C****C****U****A****A**CGUAUG.......................................................................................................................................................................C**G****U****A****G****G****C****U**GAG**A**.**G****A****G****A****A**GCGC............................................................................GUGAAC**U****U****C**.**U****U**...........**A****A****C****C****C****U**UU.........................GG**A**.**C****C****U****G**A.U.**C****U****G****G**.CUCGU...............................A.**C****C****A****G**C**G**.U**A****G****G**.**G**A....**A****G****U****U****A****A****C****G****G****C** | |
|  |  | NC\_006274.1/5012926-5012818  | **G****U****G****A****U****A****A****A****C****U****A**..**G**.**G****G****G**.**U****G****C****C****U****A****A**CGUAUG.......................................................................................................................................................................C**G****U****A****G****G****C****U**GAG**A**.**G****A****G****A****A**GCGC............................................................................GUGAAC**U****U****C**.**U****U**...........**A****A****C****C****C****U**UU.........................GG**A**.**C****C****U****G**A.U.**C****U****G****G**.CUCGU...............................A.**C****C****A****G**C**G**.U**A****G****G**.**G**A....**A****G****U****U****A****A****C****G****G****C** | |
|  |  | NC\_007530.2/4953628-4953520  | **G****U****G****A****U****A****A****A****C****U****A**..**G**.**G****G****G**.**U****G****C****C****U****A****A**CGUAUG.......................................................................................................................................................................C**G****U****A****G****G****C****U**GAG**A**.**G****A****G****A****A**GCGC............................................................................GUAAAC**U****U****C**.**U****U**...........**A****A****C****C****C****U**UU.........................GG**A**.**C****C****U****G**A.U.**C****U****G****G**.CUCGU...............................A.**C****C****A****G**C**G**.U**A****G****G**.**G**A....**A****G****U****U****A****A****C****G****G****C** | |
|  |  | NZ\_AAAC02000001.1/294704-294596  | **G****U****G****A****U****A****A****A****C****U****A**..**G**.**G****G****G**.**U****G****C****C****U****A****A**CGUAUG.......................................................................................................................................................................C**G****U****A****G****G****C****U**GAG**A**.**G****A****G****A****A**GCGC............................................................................GUAAAC**U****U****C**.**U****U**...........**A****A****C****C****C****U**UU.........................GG**A**.**C****C****U****G**A.U.**C****U****G****G**.CUCGU...............................A.**C****C****A****G**C**G**.U**A****G****G**.**G**A....**A****G****U****U****A****A****C****G****G****C** | |
|  |  | NZ\_AAEK01000055.1/27008-26900  | **G****U****G****A****U****A****A****A****C****U****A**..**G**.**G****G****G**.**U****G****C****C****U****A****A**CGUAUG.......................................................................................................................................................................C**G****U****A****G****G****C****U**GAG**A**.**G****A****G****A****A**GCGC............................................................................GUGAAC**U****U****C**.**U****U**...........**A****A****C****C****C****U**UU.........................GG**A**.**C****C****U****G**A.U.**C****U****G****G**.CUCGU...............................A.**C****C****A****G**C**G**.U**A****G****G**.**G**A....**A****G****U****U****A****A****C****G****G****C** | |
|  |  | NZ\_AAEN01000018.1/105729-105837  | **G****U****G****A****U****A****A****A****C****U****A**..**G**.**G****G****G**.**U****G****C****C****U****A****A**CGUAUG.......................................................................................................................................................................C**G****U****A****G****G****C****U**GAG**A**.**G****A****G****A****A**GCGC............................................................................GUAAAC**U****U****C**.**U****U**...........**A****A****C****C****C****U**UU.........................GG**A**.**C****C****U****G**A.U.**C****U****G****G**.CUCGU...............................A.**C****C****A****G**C**G**.U**A****G****G**.**G**A....**A****G****U****U****A****A****C****G****G****C** | |
|  |  | NZ\_AAEO01000027.1/105777-105885  | **G****U****G****A****U****A****A****A****C****U****A**..**G**.**G****G****G**.**U****G****C****C****U****A****A**CGUAUG.......................................................................................................................................................................C**G****U****A****G****G****C****U**GAG**A**.**G****A****G****A****A**GCGC............................................................................GUGAAC**U****U****C**.**U****U**...........**A****A****C****C****C****U**UU.........................GG**A**.**C****C****U****G**A.U.**C****U****G****G**.CUCGU...............................A.**C****C****A****G**C**G**.U**A****G****G**.**G**A....**A****G****U****U****A****A****C****G****G****C** | |
|  |  | NZ\_AAEP01000037.1/118492-118384  | **G****U****G****A****U****A****A****A****C****U****A**..**G**.**G****G****G**.**U****G****C****C****U****A****A**CGUAUG.......................................................................................................................................................................C**G****U****A****G****G****C****U**GAG**A**.**G****A****G****A****A**GCGC............................................................................GUAAAC**U****U****C**.**U****U**...........**A****A****C****C****C****U**UU.........................GG**A**.**C****C****U****G**A.U.**C****U****G****G**.CUCGU...............................A.**C****C****A****G**C**G**.U**A****G****G**.**G**A....**A****G****U****U****A****A****C****G****G****C** | |
|  |  | NZ\_AAEQ01000042.1/148927-148819  | **G****U****G****A****U****A****A****A****C****U****A**..**G**.**G****G****G**.**U****G****C****C****U****A****A**CGUAUG.......................................................................................................................................................................C**G****U****A****G****G****C****U**GAG**A**.**G****A****G****A****A**GCGC............................................................................GUAAAC**U****U****C**.**U****U**...........**A****A****C****C****C****U**UU.........................GG**A**.**C****C****U****G**A.U.**C****U****G****G**.CUCGU...............................A.**C****C****A****G**C**G**.U**A****G****G**.**G**A....**A****G****U****U****A****A****C****G****G****C** | |
|  |  | NZ\_AAER01000038.1/21678-21570  | **G****U****G****A****U****A****A****A****C****U****A**..**G**.**G****G****G**.**U****G****C****C****U****A****A**CGUAUG.......................................................................................................................................................................C**G****U****A****G****G****C****U**GAG**A**.**G****A****G****A****A**GCGC............................................................................GUAAAC**U****U****C**.**U****U**...........**A****A****C****C****C****U**UU.........................GG**A**.**C****C****U****G**A.U.**C****U****G****G**.CUCGU...............................A.**C****C****A****G**C**G**.U**A****G****G**.**G**A....**A****G****U****U****A****A****C****G****G****C** | |
|  |  | NZ\_AAES01000020.1/147582-147474  | **G****U****G****A****U****A****A****A****C****U****A**..**G**.**G****G****G**.**U****G****C****C****U****A****A**CGUAUG.......................................................................................................................................................................C**G****U****A****G****G****C****U**GAG**A**.**G****A****G****A****A**GCGC............................................................................GUAAAC**U****U****C**.**U****U**...........**A****A****C****C****C****U**UU.........................GG**A**.**C****C****U****G**A.U.**C****U****G****G**.CUCGU...............................A.**C****C****A****G**C**G**.U**A****G****G**.**G**A....**A****G****U****U****A****A****C****G****G****C** | |
|  |  | NZ\_AAED02000003.1/319670-319780  | **A****A****C****C****A****U****C****A****C****C****A**..**G**.**G****G****G**.**G****G****C****C****C****G****G**AC-..........................................................................................................................................................................A**A****C****G****G****G****C****U**GAG**A**U**A****C****C****G****C**UGGC........................................................................UUCAAGCAGC**G****C****G**.**G****U**...........**G****A****C****C****C****U**UU.........................GA**A**.**C****C****U****G**A.U.**C****C****A****G**.UUCAU...............................A.**C****U****G****G**C**G**.U**A****G****G**.**G**A...U**G****G****U****G****C****G****A****C****G****G** | |
|  |  | NC\_006270.2/941742-941843  | **U****A****G****C****A****U****U****A****C****U****A**..**G**.**G****G****G**.**U****G****C****C****C****G****A**AU-..........................................................................................................................................................................-**G****C****G****G****G****C****U**GAG**A**.**G****A****G****A****G**ACAU..............................................................................GCGU**U****U****C**.**U****U**...........**A****A****C****C****C****U**UU.........................GG**A**.**C****C****U****G**A.U.**C****U****G****G**.UUCGU...............................A.**C****C****A****G**C**G**.U**G****G****G**.**G**A....**A****G****U****A****A****A****A****A****G****G** | |
|  |  | NC\_006322.1/942035-942136  | **U****A****G****C****A****U****U****A****C****U****A**..**G**.**G****G****G**.**U****G****C****C****C****G****A**AU-..........................................................................................................................................................................-**G****C****G****G****G****C****U**GAG**A**.**G****A****G****A****G**ACAU..............................................................................GCGU**U****U****C**.**U****U**...........**A****A****C****C****C****U**UU.........................GG**A**.**C****C****U****G**A.U.**C****U****G****G**.UUCGU...............................A.**C****C****A****G**C**G**.U**G****G****G**.**G**A....**A****G****U****A****A****A****A****A****G****G** | |
|  |  | NC\_000964.2/954991-955091  | **A****A****U****A****G****U****U****A****C****U****G**..**G**.**G****G****G**.**U****G****C****C****C****G****C**UU-..........................................................................................................................................................................-**U****C****G****G****G****C****U**GAG**A**.**G****A****G****A****A**GGCA...............................................................................AGC**U****U****C**.**U****U**...........**A****A****C****C****C****U**UU.........................GG**A**.**C****C****U****G**A.U.**C****U****G****G**.UUCGU...............................A.**C****C****A****G**C**G**.U**G****G****G**.**G**A....**A****G****U****A****G****A****G****G****A****A** | |
|  |  | NZ\_AABH02000002.1/41536-41631  | **G****C****U****G****U****C****C****A****C****U****A**..**G**.**G****G****G**.**U****G****C****C****U****U**----..........................................................................................................................................................................-**C****U****U****G****G****C****U**GAG**A**.**U****G****A****U****A**UUAC.................................................................................U**U****A****U**.**C****A**...........**A****U****C****C****C****U**UC.........................GA**A**.**C****C****U****G**A.U.**C****U****A****G**.UCAAU...............................A.**C****U****A****G**C**G**.U**A****G****G**.**A**A....**A****G****U****G****G****U****A****U****U****C** | |
|  |  | NC\_003030.1/3058129-3058029  | **A****U****A****U****A****G****U****U****A****A****C**..**G**.**G****G****G**.**A****G****C****C****U****G****U**AG-..........................................................................................................................................................................-**A****C****A****G****G****C****U**GAG**A**G**U****G****G****A****A**UGUG.................................................................................A**U****U****C**.**C****A**...........**G****A****C****C****C****U**CA.........................UA**A**.**C****C****U****G**A.U.**U****U****G****G**.AUAAU...............................G.**C****C****A****A**C**G**.U**A****G****G**.**G**A...G**U****U****A****A****U****G****C****A****U****C** | |
|  |  | NZ\_AAFG02000005.1/77783-77879  | **A****G****C****G****A****A****C****C****G****U****U**..**G**.**G****G****G**.**U****G****C****C****U****C****C**U--..........................................................................................................................................................................-**G****G****A****G****G****C****U**GAG**A**.**G****G****C****G****U**GCAU..................................................................................**G****C****G**.**C****U**...........**A****A****C****C****C****A**UC.........................GA**A**.**C****C****U****G**A.U.**C****C****G****G**.GCAAU...............................A.**C****C****G****G**C**G**.U**A****G****G**.**G**A....**A****C****G****G****U****C****U****A****C****U** | |
|  |  | NC\_003078.1/1192148-1192053  | **G****C****G****C****A****U****U****C****C****G****A**..**G**.**G****G****G**.**A****G****C****A****C****C****A**GA-..........................................................................................................................................................................-**C****G****G****U****G****C****U**GAG**A**.**U****G****G****C****G**UUGA..................................................................................-**G****C**.**C****G**...........**G****A****C****C****C****U**U-.........................GA**A**.**C****C****U****G**A.U.**C****C****G****G**.GUCAU...............................G.**C****C****G****G**C**G**.U**A****G****G**.**A**A....**C****G****G****A****A****A****G****A****U****G** | |
|  |  | NC\_003062.1/2541734-2541630  | **G****C****C****A****U****U****C****A****C****C****A**..**G**.**G****G****G**.**U****G****U****C****C****C****G**UC-..........................................................................................................................................................................A**A****G****G****G****G****C****U**GAG**A**U**U****C****U****G****C**UUUC..............................................................................AUGC**G****C****A**.**G****U**...........**G****A****C****C****C****G**UU.........................GA**A**.**C****C****U****G**A.U.**C****C****A****G**.UUCAU...............................A.**C****U****G****G**C**G**.U**A****G****G**.**G**A...C**G****G****U****G****C****A****A****A****G****A** | |
|  |  | NC\_003304.1/2541847-2541743  | **G****C****C****A****U****U****C****A****C****C****A**..**G**.**G****G****G**.**U****G****U****C****C****C****G**UC-..........................................................................................................................................................................A**A****G****G****G****G****C****U**GAG**A**U**U****C****U****G****C**UUUC..............................................................................AUGC**G****C****A**.**G****U**...........**G****A****C****C****C****G**UU.........................GA**A**.**C****C****U****G**A.U.**C****C****A****G**.UUCAU...............................A.**C****U****G****G**C**G**.U**A****G****G**.**G**A...C**G****G****U****G****C****A****A****A****G****A** | |
|  |  | NC\_002570.2/902230-902322  | **U****A****U****A****A****C****C****A****C****U****A**..**G**.**G****G****G**.**U****G****U****C****G****A**----..........................................................................................................................................................................--**A****A****G****A****C****U**GAG**A**.**G****A****G****G****C**GAAU..................................................................................-**C****C**.**U****C**...........**A****A****C****U****C****U**UG.........................GA**A**.**C****C****U****G**A.U.**C****U****A****G**.UUCAU...............................A.**C****U****A****G**C**G**.A**A****G****G**.**G**A....**A****G****U****G****G****C****G****C****A****U** | |
|  |  | NC\_002689.2/1109576-1109677  | **G****A****G****G****A****C****G****A****U****A****A**..**G**.**G****G****G**.**A****G****C****U****U****C****G**UA-..........................................................................................................................................................................-**A****G****A****G****G****C****U**GAG**A**G**G****A****U****C****C**GAGA.................................................................................A**G****G****A**.**U****C**...........**G****A****C****C****C****U**UU.........................GA**A**.**C****C****U****G**A.U.**C****C****G****G**.GUAAU...............................G.**C****C****G****G**C**G**.G**A****G****G**.**G**A..AG**U****A****U****G****U****U****G****U****U****U** | |
|  |  | NC\_002952.2/2254059-2253964  | **A****G****G****A****A****C****U****A****C****U****A**..**G**.**G****G****G**.**A****G****C****C****U****A****A**UG-..........................................................................................................................................................................A**U****A****U****G****G****C****U**GAG**A**.**U****G****A****A****C**U---..................................................................................**G****U****U**.**C****A**...........**G****A****C****C****C****U**UA.........................UG**A**.**C****C****U****G**A.U.**U****U****G****G**.UUAGU...............................A.**C****C****A****A**C**G**.U**A****G****G**.**A**A....**A****G****U****A****G****U****U****A****U****U** | |
|  |  | NC\_002505.1/59789-59880  | **U****U****C****C****A****C****U****U****G****U****C**..**G**.**G****A****G**.**U****G****C****C****A****U**----..........................................................................................................................................................................--**U****G****G****G****C****U**GAG**A**.**C****C****G****U****U**UAUU..................................................................................--**C**.**G****G**...........**G****A****U****C****C****G**UU.........................GA**A**.**C****C****U****G**A.U.**C****A****G****G**.UUAAU...............................A.**C****C****U****G**C**G**.A**A****G****G**.**G**A....**A****C****A****A****G****A****G****A****A****G** | |
|  |  | NZ\_AAAU03000010.1/6051-6148  | **C****G****G****U****A****C****U****U****G****U****C**..**G**.**G****G****G**.**U****G****C****C****C****C****U**GU-..........................................................................................................................................................................-**G****G****G****G****G****C****U**GAG**A**.**U****C****G****G****C**CGAG..................................................................................**G****C****C**.**G****G**...........**G****U****C****C****C****G**UU.........................GA**A**.**C****C****U****G**A.U.**C****A****G****G**.GUGAC...............................G.**C****C****U****G**C**G**.U**A****G****G**.**G**A....**A****C****A****A****G****A****C****G****U****C** | |
|  |  | NC\_003317.1/1774842-1774941  | **C****A****A****A****A****U****U****C****G****U****U**..**G**.**G****G****G**.**U****G****C****C****U****U****G**AA-..........................................................................................................................................................................-**A****G****A****G****G****C****U**GAG**A**.**G****A****C****G****C**AUUC................................................................................UC**A****C****G**.**U****C**...........**A****A****C****C****C****A**UU.........................GA**A**.**C****C****U****G**A.U.**C****C****G****G**.GUAAU...............................A.**C****C****U****G**C**G**.U**A****G****G**.**G**A....**A****C****G****G****A****G****U****U****U****G** | |
|  |  | NC\_004193.1/861147-861053  | **C****G****U****A****C****G****C****A****C****U****A**..**G**.**G****G****G**.**A****G****C****U****A****U****A**AC-..........................................................................................................................................................................-**U****A****U****G****G****C****U**GAG**A**.**A****G****G****C****A**A---..................................................................................**U****G****C**.**C****U**...........**G****A****C****C****C****U**UA.........................UA**A**.**C****C****C****G**A.A.**C****U****A****G**.AUAAU...............................A.**C****U****A****G**C**G**.U**G****G****G**.**G**A....**A****G****U****G****U****A****G****U****U****C** | |
|  |  | NC\_006177.1/3180655-3180556  | **G****A****G****A****G****C****C****G****C****U****A**..**G**.**G****G****G**.**A****G****C****C****G****U****U**GC-..........................................................................................................................................................................-**A****A****C****G****G****C****U**GAG**A**A**C****C****G****G****G**GUGA................................................................................AC**C****C****C**.**G****G**...........**G****A****C****C****C****U**G-.........................UC**A**.**C****C****U****G**A.U.**C****C****G****G**.GUAAU...............................G.**C****C****G****G**C**G**.U**A****G****G**.**G**A....**A****G****C****G****A****G****C****C****U****C** | |
|  |  | NC\_003063.1/1761990-1761895  | **A****G****C****U****C****U****U****C****C****G****A**..**G**.**G****G****G**.**A****G****C****A****C****C****A**GA-..........................................................................................................................................................................-**C****G****G****U****G****C****U**GAG**A**.**U****G****G****U****G**GUGA..................................................................................-**A****C**.**C****G**...........**G****A****C****C****C****U**U-.........................GA**A**.**C****C****U****G**A.U.**C****C****G****G**.GUCAU...............................G.**C****C****G****G**C**G**.U**A****G****G**.**A**A....**C****G****G****A****A****C****U****G****U****C** | |
|  |  | NC\_003305.1/312766-312861  | **A****G****C****U****C****U****U****C****C****G****A**..**G**.**G****G****G**.**A****G****C****A****C****C****A**GA-..........................................................................................................................................................................-**C****G****G****U****G****C****U**GAG**A**.**U****G****G****U****G**GUGA..................................................................................-**A****C**.**C****G**...........**G****A****C****C****C****U**U-.........................GA**A**.**C****C****U****G**A.U.**C****C****G****G**.GUCAU...............................G.**C****C****G****G**C**G**.U**A****G****G**.**A**A....**C****G****G****A****A****C****U****G****U****C** | |
|  |  | NC\_005877.1/117694-117789  | **A****A****A****A****A****G****C****U****A****C****C**..**G**.**G****G****G**.**A****G****C****C****A****U****U**---..........................................................................................................................................................................-**A****A****U****G****G****C****U**GAG**A**G**G****A****U****C****C**AAG-..................................................................................**G****G****A**.**U****C**...........**G****A****C****C****C****G**U-.........................GA**A**.**C****C****U****G**A.U.**C****C****G****G**.GUAAU...............................A.**C****C****G****G**C**G**.G**A****G****G**.**G**A...G**A****U****A****G****C****C****A****A****U****G** | |
|  |  | NC\_006512.1/821519-821630  | **G****G****A****A****U****C****U****U****G****U****C**..**G**.**G****A****G**.**U****G****C****C****G****G****U**AGCUUUCUUAAUGAAGU............................................................................................................................................................U**A****C****C****G****G****C****U**GAG**A**.**C****C****G****C****A**AU--..................................................................................**U****G****C**.**G****G**...........**G****A****U****C****C****G**UU.........................GA**A**.**C****C****U****G**A.U.**C****A****G****G**.CUAAU...............................A.**C****C****U****G**C**G**.A**A****G****G**.**G**A....**A****C****A****A****G****A****G****A****G****C** | |
|  |  | NC\_002976.3/2096235-2096337  | **U****U****U****A****A****C****C****A****C****U****G**..**G**.**A****A****G**.**U****G****C****C****U****U****U**GU-..........................................................................................................................................................................U**U****A****A****G****G****C****U**GAG**A**.**G****U****A****A****A**GAAC..............................................................................UGAC**U****U****U**.**A****U**...........**A****A****U****U****C****C**UG.........................GA**A**.**C****C****U****G**A.U.**C****C****A****G**.UUCAU...............................A.**C****U****G****G**C**G**.U**A****G****G**.**G**A....**A****G****U****G****G****C****G****A****C****G** | |
|  |  | NC\_004461.1/2101479-2101581  | **U****U****U****A****A****C****C****A****C****U****G**..**G**.**A****A****G**.**U****G****C****C****U****U****U**GU-..........................................................................................................................................................................U**U****A****A****G****G****C****U**GAG**A**.**G****U****A****A****A**GAAC..............................................................................UGAC**U****U****U**.**A****U**...........**A****A****U****U****C****C**UG.........................GA**A**.**C****C****U****G**A.U.**C****C****A****G**.UUCAU...............................A.**C****U****G****G**C**G**.U**A****G****G**.**G**A....**A****G****U****G****G****C****G****A****C****G** | |
|  |  | NZ\_AABG04000052.1/21103-21003  | **U****A****U****G****U****C****G****C****A****U****A**..**G**.**G****G****G**.**U****G****C****U****G****G****U**GAA..........................................................................................................................................................................U**G****C****C****G****G****C****U**GAG**A**G**C****A****G****G****G**AGUU................................................................................UU**C****C****C**.**U****C**...........**A****A****C****C****C****U**--.........................UA**A**.**C****C****U****G**A.U.**C****U****G****G**.GUAAU...............................G.**C****C****A****G**C**G**.U**A****G****G**.**G**A....**A****U****G****U****G****U****A****A****A****U** | |
|  |  | NC\_002745.2/2150153-2150058  | **A****G****G****A****A****C****U****A****C****U****A**..**G**.**G****G****G**.**A****G****C****C****U****A****A**UG-..........................................................................................................................................................................A**U****A****U****G****G****C****U**GAG**A**.**U****G****A****A****U**UG--..................................................................................-**U****U**.**C****A**...........**G****A****C****C****C****U**UA.........................UG**A**.**C****C****U****G**A.U.**U****U****G****G**.UUAGU...............................A.**C****C****A****A**C**G**.U**A****G****G**.**A**A....**A****G****U****A****G****U****U****A****U****U** | |
|  |  | NC\_002758.2/2226585-2226490  | **A****G****G****A****A****C****U****A****C****U****A**..**G**.**G****G****G**.**A****G****C****C****U****A****A**UG-..........................................................................................................................................................................A**U****A****U****G****G****C****U**GAG**A**.**U****G****A****A****U**UG--..................................................................................-**U****U**.**C****A**...........**G****A****C****C****C****U**UA.........................UG**A**.**C****C****U****G**A.U.**U****U****G****G**.UUAGU...............................A.**C****C****A****A**C**G**.U**A****G****G**.**A**A....**A****G****U****A****G****U****U****A****U****U** | |
|  |  | NC\_002951.2/2151694-2151599  | **A****G****G****A****A****C****U****A****C****U****A**..**G**.**G****G****G**.**A****G****C****C****U****A****A**UG-..........................................................................................................................................................................A**U****A****U****G****G****C****U**GAG**A**.**U****G****A****A****U**UG--..................................................................................-**U****U**.**C****A**...........**G****A****C****C****C****U**UA.........................UG**A**.**C****C****U****G**A.U.**U****U****G****G**.UUAGU...............................A.**C****C****A****A**C**G**.U**A****G****G**.**A**A....**A****G****U****A****G****U****U****A****U****U** | |
|  |  | NC\_002953.3/2155766-2155671  | **A****G****G****A****A****C****U****A****C****U****A**..**G**.**G****G****G**.**A****G****C****C****U****A****A**UG-..........................................................................................................................................................................A**U****A****U****G****G****C****U**GAG**A**.**U****G****A****A****U**UG--..................................................................................-**U****U**.**C****A**...........**G****A****C****C****C****U**UA.........................UG**A**.**C****C****U****G**A.U.**U****U****G****G**.UUAGU...............................A.**C****C****A****A**C**G**.U**A****G****G**.**A**A....**A****G****U****A****G****U****U****A****U****U** | |
|  |  | NC\_003923.1/2176661-2176566  | **A****G****G****A****A****C****U****A****C****U****A**..**G**.**G****G****G**.**A****G****C****C****U****A****A**UG-..........................................................................................................................................................................A**U****A****U****G****G****C****U**GAG**A**.**U****G****A****A****U**UG--..................................................................................-**U****U**.**C****A**...........**G****A****C****C****C****U**UA.........................UG**A**.**C****C****U****G**A.U.**U****U****G****G**.UUAGU...............................A.**C****C****A****A**C**G**.U**A****G****G**.**A**A....**A****G****U****A****G****U****U****A****U****U** | |
|  |  | NZ\_AAAT03000001.1/215607-215705  | **G****G****G****U****U****C****U****U****G****U****C**..**G**.**G****G****G**.**U****G****C****C****U****U****G**CU-..........................................................................................................................................................................A**U****G****A****G****G****C****U**GAG**A**.**U****C****G****A****A**UAAU..................................................................................**U****U****C**.**G****G**...........**A****U****C****C****C****G**UU.........................GA**A**.**C****C****U****G**A.U.**C****A****G****G**.UUAGC...............................G.**C****C****U****G**C**G**.U**A****G****G**.**G**A....**A****C****A****A****G****A****U****U****U****C** | |
|  |  | NZ\_AADT03000012.1/69323-69421  | **U****A****G****U****U****A****C****C****C****C****G**..**G**.**G****G****G**.**U****G****C****C****C****U****U**---..........................................................................................................................................................................-**A****G****G****G****G****C****U**GAG**A**.**A****G****U****G****C**CGCA..................................................................................**G****C****A**.**C****U**...........**A****A****C****C****C****U**UG.........................GA**A**.**C****C****U****G**A.U.**C****U****G****G**.GUCAU...............................A.**C****C****A****G**C**G**.U**A****G****G**.**G**A.AGU**G****G****G****G****A****C****C****G****A****A** | |
|  |  | NC\_006582.1/1860935-1861039  | **U****A****G****A****A****A****G****A****C****U****A**..**G**.**G****G****G**.**U****G****C****C****U****A****A**UGAA.........................................................................................................................................................................G**U****U****A****G****G****C****U**GAG**A**A**U****A****A****G****A**ACGU...............................................................................AUU**U****C****U**.**U****U**...........**U****A****C****C****C****U**AA.........................UA**A**.**C****C****U****G**A.U.**C****U****G****G**.GUGAU...............................G.**C****C****A****G**C**G**.G**A****G****G**.**G**A....**A****G****U****C****G****G****U****U****U****G** | |
|  |  | NC\_003911.11/45973-46070  | **C****G****C****C****A****A****C****C****G****U****U**..**G**.**G****G****G**.**U****G****C****C****C****U****G**UG-..........................................................................................................................................................................-**U****G****G****G****G****C****U**GAG**A**.**G****G****C****G****C**GGGU..................................................................................**G****C****G**.**U****C**...........**A****A****C****C****C****A**UC.........................GA**A**.**C****C****U****G**A.U.**C****C****G****G**.GCAAU...............................A.**C****C****G****G**C**G**.U**A****G****G**.**G**A....**A****C****G****G****U****C****U****A****U****C** | |
|  |  | NC\_006270.2/1270341-1270442  | **G****U****U****U****U****C****C****A****C****U****A**..**G**.**G****G****G**.**A****G****U****C****C****U****U**GA-..........................................................................................................................................................................U**A****A****G****G****G****C****U**GAG**A**.**U****A****A****A****A**GUUU...............................................................................GAC**U****U****U**.**U****A**...........**G****A****C****C****C****U**CA.........................UA**A**.**C****C****U****G**A.A.**C****A****G****G**.UUCAA...............................A.**C****C****U****G**C**G**.U**A****G****G**.**G**A....**A****G****U****G****G****C****A****C****G****G** | |
|  |  | NC\_006322.1/1271200-1271301  | **G****U****U****U****U****C****C****A****C****U****A**..**G**.**G****G****G**.**A****G****U****C****C****U****U**GA-..........................................................................................................................................................................U**A****A****G****G****G****C****U**GAG**A**.**U****A****A****A****A**GUUU...............................................................................GAC**U****U****U**.**U****A**...........**G****A****C****C****C****U**CA.........................UA**A**.**C****C****U****G**A.A.**C****A****G****G**.UUCAA...............................A.**C****C****U****G**C**G**.U**A****G****G**.**G**A....**A****G****U****G****G****C****A****C****G****G** | |
|  |  | NZ\_AADT03000042.1/1588-1492  | **G****A****G****C****G****A****C****C****G****U****G**..**G**.**G****G****G**.**A****G****C****U****U****U****A**AU-..........................................................................................................................................................................-**U****U****A****A****G****C****U**GAG**A**.**U****G****G****G****G**UAAG..................................................................................**C****C****C**.**C****A**...........**A****A****C****C****C****C**G-.........................GA**A**.**C****C****U****G**A.U.**G****U****G****G**.GUAAU...............................G.**C****C****A****C**C**G**.U**A****G****G**.**G**A....**A****C****G****G****U****C****G****C****U****G** | |
|  |  | NC\_006370.1/106620-106710  | **U****G****A****A****A****C****U****U****G****U****C**..**G**.**G****A****G**.**U****G****C****C****A****C**----..........................................................................................................................................................................--**A****U****G****G****C****U**GAG**A**.**C****C****G****U****A**U---..................................................................................**U****A****C**.**G****G**...........**G****A****U****C****C****G**UU.........................GA**A**.**C****C****U****G**A.U.**C****A****G****G**.CUAAU...............................A.**C****C****U****G**C**G**.A**A****G****G**.**G**A....**A****C****A****A****G****A****G****A****A****A** | |
|  |  | NZ\_AADT03000011.1/45261-45169  | **A****A****A****C****A****U****A****A****G****C****A**..**G**.**G****G****G**.**U****G****C****C****G****C**----..........................................................................................................................................................................--**A****A****G****G****C****U**GAG**A**.**G****G****G****C****U**AAAC..................................................................................-**G****C**.**C****U**...........**A****A****C****C****C****U**UU.........................GA**A**.**C****C****U****G**A.U.**G****U****G****G**.GUAAU...............................G.**C****C****A****C**C**G**.U**A****G****G**.**G**A....**A****G****U****A****A****U****G****A****U****G** | |
|  |  | NC\_002678.2/4674022-4673913  | **U****G****C****A****U****U****C****A****C****C****A**..**G**.**G****G****G**.**A****G****U****C****C****C****G**GC-..........................................................................................................................................................................A**A****G****G****G****G****C****U**GAG**A**U**A****C****U****G****C**UGGC.........................................................................UUUCGCGGC**G****C****A**.**G****U**...........**G****A****C****C****C****G**UU.........................GA**A**.**C****C****U****G**A.U.**C****C****A****G**.UUCAU...............................A.**C****U****G****G**C**G**.U**A****G****G**.**G**A...C**G****G****U****G****C****A****A****G****C****G** | |
|  |  | NC\_002976.3/212280-212188  | **U****A****U****A****U****G****C****A****C****U****A**..**G**.**G****G****G**.**U****G****U****U****U****U**----..........................................................................................................................................................................--**U****U****A****A****C****U**GAG**A**.**U****G****G****A****U**AUCA..................................................................................-**U****C**.**C****A**...........**A****A****C****C****C****U**UU.........................GA**A**.**C****C****U****G**A.A.**C****U****A****G**.CUUAU...............................A.**C****U****A****G**C**G**.G**A****G****G**.**A**A....**A****G****U****G****U****U****G****A****A****U** | |
|  |  | NC\_004461.1/328039-327947  | **U****A****U****A****U****G****C****A****C****U****A**..**G**.**G****G****G**.**U****G****U****U****U****U**----..........................................................................................................................................................................--**U****U****A****A****C****U**GAG**A**.**U****G****G****A****U**AUCA..................................................................................-**U****C**.**C****A**...........**A****A****C****C****C****U**UU.........................GA**A**.**C****C****U****G**A.A.**C****U****A****G**.CUUAU...............................A.**C****U****A****G**C**G**.G**A****G****G**.**A**A....**A****G****U****G****U****U****G****A****A****U** | |
|  |  | NC\_002570.2/2025553-2025660  | **A****G****A****A****A****U****A****A****C****U****A**..**G**.**G****G****G**.**A****G****U****C****C****A****A**UGA..........................................................................................................................................................................G**C****U****G****G****G****C****U**GAG**A**.**A****A****A****A****A**ACGC...........................................................................GAUGAAG**U****U****U**.**U****U**..........A**A****A****C****C****C****U**CG.........................GG**A**.**C****C****U****G**A.U.**C****U****G****G**.AUCAU...............................A.**C****C****A****G**C**G**.U**G****G****G**.**G**A....**A****G****U****U****A****U****C****A****U****G** | |
|  |  | NC\_004578.1/5648734-5648637  | **G****C****G****U****U****C****U****U****G****U****C**..**G**.**G****G****G**.**U****G****C****C****U****U****G**CU-..........................................................................................................................................................................A**U****G****A****G****G****C****U**GAG**A**.**U****C****G****G****U**UAAA..................................................................................-**C****C**.**G****G**...........**A****U****C****C****C****G**UU.........................GA**A**.**C****C****U****G**A.U.**C****A****G****G**.UUAGC...............................G.**C****C****U****G**C**G**.U**A****G****G**.**G**A....**A****C****A****A****G****A****U****U****U****C** | |
|  |  | NC\_007005.1/614914-615011  | **G****C****G****U****U****C****U****U****G****U****C**..**G**.**G****G****G**.**U****G****C****C****U****U****G**CU-..........................................................................................................................................................................A**U****G****A****G****G****C****U**GAG**A**.**U****C****G****G****U**UAAA..................................................................................-**C****C**.**G****G**...........**A****U****C****C****C****G**UU.........................GA**A**.**C****C****U****G**A.U.**C****A****G****G**.UUAGC...............................G.**C****C****U****G**C**G**.U**A****G****G**.**G**A....**A****C****A****A****G****A****U****U****U****C** | |
|  |  | NC\_004347.1/2834965-2834863  | **A****G****C****G****C****C****G****C****C****A****A**..**G**.**G****G****G**.**U****G****U****U****U****G****C**CAAAAUUC.....................................................................................................................................................................G**G****C****A****G****A****C****U**GAG**A**.**U****G****U****C****A**UCA-..................................................................................-**G****A**.**C****G**...........**A****A****C****C****C****U**UA.........................GA**A**.**C****C****U****G**A.U.**C****C****G****G**.CUAAU...............................A.**C****C****G****G**C**G**.U**A****G****G**.**A**A....**U****G****G****G****C****C****A****A****U****C** | |
|  |  | NC\_004722.1/729288-729384  | **U****G****A****A****A****C****C****A****C****U****A**..**G**.**G****G****G**.**U****G****C****U****U****G**----..........................................................................................................................................................................-**A****C****U****U****G****C****U**GAG**A**.**G****A****G****G****A**AUAA..................................................................................**U****C****C**.**U****U**...........**A****A****C****C****C****U**UACA.......................AC**A**.**C****C****U****G**A.U.**C****U****A****G**.GUAAU...............................A.**C****U****A****G**C**G**.A**A****G****G**.**G**A....**A****G****U****G****G****A****A****C****A****U** | |
|  |  | NC\_002578.1/1238734-1238634  | **A****A****C****U****A****G****G****C****G****A****G**..**G**.**G****G****G**.**A****G****C****U****C****C****A**UA-..........................................................................................................................................................................A**G****G****G****G****G****C****U**GAG**A**G**G****A****U****C****C**GGAU..................................................................................**G****G****A**.**U****C**...........**G****A****C****C****C****C**UG.........................GA**A**.**C****C****U****G**A.U.**C****C****G****G**.GUAAU...............................A.**C****C****G****G**C**G**.G**A****G****G**.**G**A...A**U****C****G****U****A****U****G****A****U****G** | |
|  |  | NZ\_AADW02000004.1/123130-123027  | **A****C****G****A****G****C****A****A****C****U****A**..**G**.**G****G****G**.**A****G****C****U****G****G****A**GGA..........................................................................................................................................................................G**U****C****C****G****G****C****U**GAG**A**G**G****A****A****A****A**CCAC..............................................................................UCUG**U****U****U**.**U****C**...........**G****A****C****C****C****U**G-.........................UC**A**.**C****C****U****G**A.A.**C****U****G****G**.AUAAU...............................A.**C****C****A****G**C**G**.U**A****G****G**.**A**A....**A****G****U****U****G****A****G****A****U****C** | |
|  |  | NC\_002939.4/640928-640834  | **A****U****A****G****U****C****U****G****C****U****G**..**G**.**G****G****G**.**A****G****U****U****C****U****U**---..........................................................................................................................................................................-**G****G****G****A****A****C****U**GAG**A**.**C****G****G****G****C**AAC-..................................................................................**G****C****C**.**C****G**...........**A****A****C****C****C****U**UU.........................GA**A**.**C****C****U****G**A.U.**C****C****G****G**.UUUAU...............................A.**C****C****G****G**C**G**.U**A****G****G**.**G**A....**A****G****C****G****G****C****C****A****G****A** | |
|  |  | NC\_002696.2/2234291-2234193  | **G****U****C****U****G****U****U****C****A****C****C**..**G**.**G****G****G**.**G****G****C****C****G****C****U**CG-..........................................................................................................................................................................-**C****G****C****G****G****C****U**GAG**A**U**U****G****G****G****C**UCAC..................................................................................**G****C****C**.**C****U**...........**G****A****C****C****C****G**UA.........................GA**A**.**C****C****U****G**A.U.**C****C****G****G**.GUCAU...............................G.**C****C****G****G**C**G**.A**A****G****G**.**G**A....**G****G****G****A****A****C****G****C****G****G** | |
|  |  | NC\_003909.8/828950-829045  | **U****G****A****A****A****C****C****A****C****U****A**..**G**.**G****G****G**.**U****G****C****U****U****G**----..........................................................................................................................................................................-**U****U****G****U****G****C****U**GAG**A**.**G****A****G****G****A**AUAA..................................................................................**U****C****C**.**U****U**...........**A****A****C****C****C****U**UAC........................AG**A**.**C****C****U****G**A.U.**C****U****A****G**.GUAAU...............................A.**C****U****A****G**C**G**.A**A****G****G**.**G**A....**A****G****U****G****G****A****G****C****A****A** | |
|  |  | NC\_002678.2/3104122-3104015  | **A****A****C****G****C****U****C****U****A****A****C**..**G**.**G****G****G**.**U****G****C****C****G****G****A**CGCGAUCUUCGCG................................................................................................................................................................G**A****C****C****G****G****C****U**GAG**A**.**G****G****C****A****G**UCUC..................................................................................--**G**.**C****C**...........**A****A****C****C****C****G**CU.........................GA**A**.**C****C****U****G**A.U.**C****C****G****G**.UUUGU...............................A.**C****C****G****G**C**G**.G**A****G****G**.**G**A....**U****U****A****G****A****C****G****U****U****U** | |
|  |  | NC\_003869.1/2399659-2399563  | **U****A****U****G****U****C****C****G****C****U****G**..**G**.**G****G****G**.**A****G****C****C****U****G****A**---..........................................................................................................................................................................-**A****A****A****G****G****C****U**GAG**A**.**C****G****G****C****A**GAAA..................................................................................**U****G****C**.**C****G**...........**A****A****C****C****C****U**UG.........................GA**A**.**C****C****U****G**A.U.**G****A****G****G**.GUAAU...............................G.**C****C****U****C**C**G**.U**A****G****G**.**G**A...A**U****G****C****G****G****G****C****U****C****A** | |
|  |  | NC\_003030.1/3060579-3060672  | **U****A****U****A****U****G****U****G****C****U****A**..**G**.**G****G****G**.**U****G****C****C****U****U**----..........................................................................................................................................................................--**U****A****G****G****C****U**GAG**A**.**A****A****C****A****G**UUUG...............................................................................UCA**C**-**G**.**U****U**...........**A****A****C****C****C****U**--.........................UA**A**.**C****C****U****G**A.U.**C****U****G****G**.AUAAU...............................A.**C****C****A****G**C**G**.U**A****G****G**.**G**A....**A****G****C****A****G****U****U****U****G****A** | |
|  |  | NC\_004603.1/3232562-3232471  | **C****U****U****C****A****C****U****U****G****U****C**..**G**.**G****A****G**.**U****G****C****C****A****U**----..........................................................................................................................................................................--**A****A****G****G****C****U**GAG**A**.**C****C****G****U****U**AAUU..................................................................................--**C**.**G****G**...........**G****A****U****C****C****G**UU.........................GA**A**.**C****C****U****G**A.U.**C****A****G****A**.UUAAU...............................A.**U****C****U****G**C**G**.A**A****G****G**.**G**A....**A****C****A****A****G****A****G****A****A****G** | |
|  |  | NC\_004460.1/1577318-1577227  | **C****C****A****A****C****A****U****A****G****U****C**..**G**.**G****G****G**.**A****G****C****C****U****U**----..........................................................................................................................................................................-**A****U****C****G****G****C****U**GAG**A**.**U****C****G****C****C**AC--..................................................................................-**G****C**.**G****A**...........**G****A****C****C****C****G**UU.........................GA**A**.**C****C****U****G**A.U.**U****C****A****G**.UUAAU...............................A.**C****U****G****A**C**G**.U**A****G****G**.**G**A....**A****C****U****A****U****U****A****C****G****C** | |
|  |  | NC\_005140.1/303695-303604  | **C****C****A****A****C****A****U****A****G****U****C**..**G**.**G****G****G**.**A****G****C****C****U****U**----..........................................................................................................................................................................-**A****U****C****G****G****C****U**GAG**A**.**U****C****G****C****C**AC--..................................................................................-**G****C**.**G****A**...........**G****A****C****C****C****G**UU.........................GA**A**.**C****C****U****G**A.U.**U****C****A****G**.UUAAU...............................A.**C****U****G****A**C**G**.U**A****G****G**.**G**A....**A****C****U****A****U****U****A****C****G****C** | |
|  |  | NZ\_AAEF02000181.1/1367-1270  | **G****C****A****C****A****A****C****G****A****C****A**..**G**.**G****G****G**.**A****G****C****G****C****C****G**UG-..........................................................................................................................................................................-**G****G****G****C****G****C****U**GAG**A**G**U****G****C****G****G**GUUU..................................................................................**C****C****G**.**C****A**...........**G****A****C****C****C****U**C-.........................GA**A**.**C****C****U****G**A.U.**C****C****G****G**.UUCAG...............................A.**C****C****G****G**C**G**.U**A****G****G**.**G**A....**G****U****C****G****G****G****A****C****G****U** | |
|  |  | NC\_003366.1/843540-843634  | **C****G****U****A****A****G****U****G****C****U****A**..**G**.**G****G****G**.**U****G****C****A****U****U****U**UU-..........................................................................................................................................................................-**A****U****A****U****G****C****U**GAG**A**.**G****G****A****U****A**A---..................................................................................**U****A****U**.**C****U**...........**A****A****C****C****C****U**UA.........................AA**A**.**C****C****U****G**A.U.**G****U****A****G**.UUAGU...............................A.**C****U****A****C**C**G**.U**A****G****G**.**G**A....**A****G****C****A****A****A****A****G****C****U** | |
|  |  | NZ\_AAAW03000127.1/1179-1284  | **C****A****U****A****A****U****C****A****C****U****A**..**G**.**G****G****G**.**G****G****C****C****G****A****A**UAA..........................................................................................................................................................................G**G****U****C****G****G****C****U**GAG**A**U**A****A****A****G****G**ACCC............................................................................AAGAAU**C****C****U**.**U****U**...........**G****A****C****C****C****U**--.........................UA**A**.**C****C****U****G**A.U.**C****U****G****G**.GUAAU...............................G.**C****C****A****G**C**G**.U**A****G****G**.**G**A...A**G****G****U****G****G****A****U****A****A****U** | |
|  |  | NC\_006582.1/730609-730710  | **U****A****A****A****A****C****C****G****C****U****A**..**G**.**G****G****G**.**U****G****C****U****U****C****G**AAUGA........................................................................................................................................................................U**G****G****A****A****G****C****U**GAG**A**.**G****A****G****G****C**AAAC..................................................................................**G****C****C**.**U****U**...........**A****A****C****C****C****U**UA.........................UC**A**.**C****C****U****G**A.U.**C****U****G****G**.UUCGG...............................A.**C****C****A****G**C**G**.G**A****G****G**.**G**A....**A****G****C****G****G****G****C****A****C****G** | |
|  |  | NZ\_AABH02000001.1/81245-81153  | **A****A****U****A****A****U****C****A****C****U****A**..**G**.**G****G****G**.**C****G****U****C****G****C**----..........................................................................................................................................................................-**A****A****A****G****A****C****U**GAG**A**.**U****G****A****U****G**UAUA..................................................................................--**U**.**C****G**...........**G****U****C****C****C****U**UU.........................GA**A**.**C****C****U****G**A.U.**C****U****A****G**.UUAAU...............................A.**C****U****A****G**C**G**.U**A****G****G**.**A**A....**A****G****U****G****A****C****C****A****A****U** | |
|  |  | NC\_000964.2/1606664-1606758  | **G****A****U****U****C****A****U****C****C****U****A**..**G**.**G****G****G**.**U****G****C****U****U****U****G**---..........................................................................................................................................................................-**C****G****A****A****G****C****U**GAG**A**.**G****A****G****A****C**UUU-..................................................................................**G****U****C**.**U****C**...........**A****A****C****C****C****U**UU.........................UG**A**.**C****C****U****G**A.U.**C****U****G****G**.AUCAU...............................G.**C****C****A****G**C**G**.G**A****G****G**.**G**A....**A****G****C****G****G****U****G****A****A****A** | |
|  |  | NC\_004557.1/1866897-1866795  | **A****U****A****A****A****G****U****U****U****C****A**..**G**.**G****G****G**.**A****G****C****U****G****G****U**UUA..........................................................................................................................................................................U**G****U****C****A****G****C****U**GAG**A**G**U****A****A****G****A**ACUA...............................................................................AAU**U****C****U**.**U****A**...........**G****A****C****C****C****U**U-.........................UA**A**.**C****C****U****G**A.U.**C****U****G****G**.AUAAU...............................G.**C****C****A****G**C**G**.U**A****G****G**.**G**A....**G****A****U****G****G****U****U****U****A****C** | |
|  |  | NC\_003997.3/752271-752367  | **U****G****A****A****A****C****C****A****C****U****A**..**G**.**G****G****G**.**U****G****C****U****U****G**----..........................................................................................................................................................................-**U****U****G****U****G****C****U**GAG**A**.**G****A****G****G****A**AUAA..................................................................................**U****C****C**.**U****U**...........**A****A****C****C****C****U**UAUA.......................AC**A**.**C****C****U****G**A.U.**C****U****A****G**.GUAAU...............................A.**C****U****A****G**C**G**.A**A****G****G**.**G**A....**A****G****U****G****G****A****A****C****A****A** | |
|  |  | NC\_005945.1/752167-752263  | **U****G****A****A****A****C****C****A****C****U****A**..**G**.**G****G****G**.**U****G****C****U****U****G**----..........................................................................................................................................................................-**U****U****G****U****G****C****U**GAG**A**.**G****A****G****G****A**AUAA..................................................................................**U****C****C**.**U****U**...........**A****A****C****C****C****U**UAUA.......................AC**A**.**C****C****U****G**A.U.**C****U****A****G**.GUAAU...............................A.**C****U****A****G**C**G**.A**A****G****G**.**G**A....**A****G****U****G****G****A****A****C****A****A** | |
|  |  | NC\_005957.1/743219-743315  | **U****G****A****A****A****C****C****A****C****U****A**..**G**.**G****G****G**.**U****G****C****U****U****G**----..........................................................................................................................................................................-**U****U****G****U****G****C****U**GAG**A**.**G****A****G****G****A**AUAA..................................................................................**U****C****C**.**U****U**...........**A****A****C****C****C****U**UAUA.......................AC**A**.**C****C****U****G**A.U.**C****U****A****G**.GUAAU...............................A.**C****U****A****G**C**G**.A**A****G****G**.**G**A....**A****G****U****G****G****A****A****C****A****A** | |
|  |  | NC\_006274.1/742233-742329  | **U****G****A****A****A****C****C****A****C****U****A**..**G**.**G****G****G**.**U****G****C****U****U****G**----..........................................................................................................................................................................-**U****U****G****U****G****C****U**GAG**A**.**G****A****G****G****A**AUAA..................................................................................**U****C****C**.**U****U**...........**A****A****C****C****C****U**UAUA.......................AC**A**.**C****C****U****G**A.U.**C****U****A****G**.GUAAU...............................A.**C****U****A****G**C**G**.A**A****G****G**.**G**A....**A****G****U****G****G****A****A****C****A****A** | |
|  |  | NC\_007530.2/752272-752368  | **U****G****A****A****A****C****C****A****C****U****A**..**G**.**G****G****G**.**U****G****C****U****U****G**----..........................................................................................................................................................................-**U****U****G****U****G****C****U**GAG**A**.**G****A****G****G****A**AUAA..................................................................................**U****C****C**.**U****U**...........**A****A****C****C****C****U**UAUA.......................AC**A**.**C****C****U****G**A.U.**C****U****A****G**.GUAAU...............................A.**C****U****A****G**C**G**.A**A****G****G**.**G**A....**A****G****U****G****G****A****A****C****A****A** | |
|  |  | NZ\_AAAC02000001.1/1254262-1254358  | **U****G****A****A****A****C****C****A****C****U****A**..**G**.**G****G****G**.**U****G****C****U****U****G**----..........................................................................................................................................................................-**U****U****G****U****G****C****U**GAG**A**.**G****A****G****G****A**AUAA..................................................................................**U****C****C**.**U****U**...........**A****A****C****C****C****U**UAUA.......................AC**A**.**C****C****U****G**A.U.**C****U****A****G**.GUAAU...............................A.**C****U****A****G**C**G**.A**A****G****G**.**G**A....**A****G****U****G****G****A****A****C****A****A** | |
|  |  | NZ\_AAEN01000010.1/401318-401222  | **U****G****A****A****A****C****C****A****C****U****A**..**G**.**G****G****G**.**U****G****C****U****U****G**----..........................................................................................................................................................................-**U****U****G****U****G****C****U**GAG**A**.**G****A****G****G****A**AUAA..................................................................................**U****C****C**.**U****U**...........**A****A****C****C****C****U**UAUA.......................AC**A**.**C****C****U****G**A.U.**C****U****A****G**.GUAAU...............................A.**C****U****A****G**C**G**.A**A****G****G**.**G**A....**A****G****U****G****G****A****A****C****A****A** | |
|  |  | NZ\_AAEO01000033.1/441744-441648  | **U****G****A****A****A****C****C****A****C****U****A**..**G**.**G****G****G**.**U****G****C****U****U****G**----..........................................................................................................................................................................-**U****U****G****U****G****C****U**GAG**A**.**G****A****G****G****A**AUAA..................................................................................**U****C****C**.**U****U**...........**A****A****C****C****C****U**UAUA.......................AC**A**.**C****C****U****G**A.U.**C****U****A****G**.GUAAU...............................A.**C****U****A****G**C**G**.A**A****G****G**.**G**A....**A****G****U****G****G****A****A****C****A****A** | |
|  |  | NZ\_AAEP01000028.1/4061-4157  | **U****G****A****A****A****C****C****A****C****U****A**..**G**.**G****G****G**.**U****G****C****U****U****G**----..........................................................................................................................................................................-**U****U****G****U****G****C****U**GAG**A**.**G****A****G****G****A**AUAA..................................................................................**U****C****C**.**U****U**...........**A****A****C****C****C****U**UAUA.......................AC**A**.**C****C****U****G**A.U.**C****U****A****G**.GUAAU...............................A.**C****U****A****G**C**G**.A**A****G****G**.**G**A....**A****G****U****G****G****A****A****C****A****A** | |
|  |  | NZ\_AAEQ01000022.1/6623-6719  | **U****G****A****A****A****C****C****A****C****U****A**..**G**.**G****G****G**.**U****G****C****U****U****G**----..........................................................................................................................................................................-**U****U****G****U****G****C****U**GAG**A**.**G****A****G****G****A**AUAA..................................................................................**U****C****C**.**U****U**...........**A****A****C****C****C****U**UAUA.......................AC**A**.**C****C****U****G**A.U.**C****U****A****G**.GUAAU...............................A.**C****U****A****G**C**G**.A**A****G****G**.**G**A....**A****G****U****G****G****A****A****C****A****A** | |
|  |  | NZ\_AAER01000025.1/6128-6224  | **U****G****A****A****A****C****C****A****C****U****A**..**G**.**G****G****G**.**U****G****C****U****U****G**----..........................................................................................................................................................................-**U****U****G****U****G****C****U**GAG**A**.**G****A****G****G****A**AUAA..................................................................................**U****C****C**.**U****U**...........**A****A****C****C****C****U**UAUA.......................AC**A**.**C****C****U****G**A.U.**C****U****A****G**.GUAAU...............................A.**C****U****A****G**C**G**.A**A****G****G**.**G**A....**A****G****U****G****G****A****A****C****A****A** | |
|  |  | NZ\_AAES01000021.1/6137-6233  | **U****G****A****A****A****C****C****A****C****U****A**..**G**.**G****G****G**.**U****G****C****U****U****G**----..........................................................................................................................................................................-**U****U****G****U****G****C****U**GAG**A**.**G****A****G****G****A**AUAA..................................................................................**U****C****C**.**U****U**...........**A****A****C****C****C****U**UAUA.......................AC**A**.**C****C****U****G**A.U.**C****U****A****G**.GUAAU...............................A.**C****U****A****G**C**G**.A**A****G****G**.**G**A....**A****G****U****G****G****A****A****C****A****A** | |
|  |  | NZ\_AAAS03000003.1/34417-34515  | **C****C****A****A****C****U****C****G****C****U****A**..**G**.**G****G****G**.**A****G****U****U**------..........................................................................................................................................................................--**U****C****G****A****C****U**GAG**A**.**G****U****C****C****G**ACGC...........................................................................GCUGCUA**C****G****G**.**A****C**...........**A****A****C****C****C****U**UU.........................GA**A**.**C****C****U****G**A.U.**C****C****G****G**.AUAAU...............................G.**C****C****G****G**C**G**.U**A****G****G**.**G**A....**A****G****C****G****U****U****C****C****A****C** | |
|  |  | NC\_006461.1/1687922-1687836  | **G****G****C****C****G****U****C****A****C****C****G**..**G**.**G****G****G**.**U****G****C****C****C****C****A**A--..........................................................................................................................................................................-**A****G****G****G****G****C****U**GAG**A**.---------..................................................................................---.--........GCA**U****A****C****C****C****U**UG.........................GA**A**.**C****C****U****G**A.U.**C****C****G****G**.GUCAU...............................G.**C****C****G****G**C**G**.U**A****G****G**.**G**A...A**G****G****U****G****A****C****G****G****C****C** | |
|  |  | NC\_000964.2/1241574-1241675  | **U****U****U****A****A****C****C****A****C****U****A**..**G**.**G****G****G**.**U****G****U****C****C****U****U**CA-..........................................................................................................................................................................U**A****A****G****G****G****C****U**GAG**A**.**U****A****A****A****A**GUGU...............................................................................GAC**U****U****U**.**U****A**...........**G****A****C****C****C****U**CA.........................UA**A**.**C****U****U****G**A.A.**C****A****G****G**.UUCAG...............................A.**C****C****U****G**C**G**.U**A****G****G**.**G**A....**A****G****U****G****G****A****G****C****G****G** | |
|  |  | NC\_003366.1/1876370-1876274  | **A****G****A****U****A****U****U****G****C****U****A**..**G**.**G****G****G**.**U****G****C****U****G****U****A**---..........................................................................................................................................................................-**A****A****A****G****G****C****U**GAG**A**.**G****G****G****A****U**AAUA.................................................................................A**G****U****C**.**C****U**...........**A****A****C****U****C****U**AA.........................UA**A**.**C****C****U****G**A.U.**U****U****G****G**.UUAAU...............................A.**C****C****A****G**C**G**.U**A****G****G**.**G**A....**A****G****U****A****U****A****U****U****U****U** | |
|  |  | NC\_004459.1/971218-971127  | **U****U****U****C****A****C****U****U****G****U****C**..**G**.**G****A****G**.**U****G****C****C****U****U**----..........................................................................................................................................................................--**A****G****G****G****C****U**GAG**A**.**C****C****G****U****U**UAUU..................................................................................--**C**.**G****G**...........**G****A****U****C****C****G**UU.........................GA**A**.**C****C****U****G**A.U.**C****A****G****G**.UUAGA...............................A.**C****C****U****G**C**G**.A**A****G****G**.**G**A....**A****C****A****A****G****A****G****A****A****G** | |
|  |  | NC\_002745.2/1056072-1055979  | **C****G****C****A****C****A****C****A****C****U****A**..**G**.**G****G****G**.**U****G****U****U****U**-----..........................................................................................................................................................................--**U****A****U****A****C****U**GAG**A**.**U****G****A****G****G**CUUG.................................................................................C**C****C****U**.**C****A**...........**A****A****C****C****C****U**UU.........................GA**A**.**C****C****U****G**A.U.**C****U****A****G**.CUUGA...............................A.**C****U****A****G**C**G**.U**A****G****G**.**A**A....**A****G****U****G****U****U****A****C****U****A** | |
|  |  | NC\_002758.2/1132400-1132307  | **C****G****C****A****C****A****C****A****C****U****A**..**G**.**G****G****G**.**U****G****U****U****U**-----..........................................................................................................................................................................--**U****A****U****A****C****U**GAG**A**.**U****G****A****G****G**CUUG.................................................................................C**C****C****U**.**C****A**...........**A****A****C****C****C****U**UU.........................GA**A**.**C****C****U****G**A.U.**C****U****A****G**.CUUGA...............................A.**C****U****A****G**C**G**.U**A****G****G**.**A**A....**A****G****U****G****U****U****A****C****U****A** | |
|  |  | NC\_002951.2/1097387-1097294  | **C****G****C****A****C****A****C****A****C****U****A**..**G**.**G****G****G**.**U****G****U****U****U**-----..........................................................................................................................................................................--**U****A****U****A****C****U**GAG**A**.**U****G****A****G****G**CUUG.................................................................................C**C****C****U**.**C****A**...........**A****A****C****C****C****U**UU.........................GA**A**.**C****C****U****G**A.U.**C****U****A****G**.CUUGA...............................A.**C****U****A****G**C**G**.U**A****G****G**.**A**A....**A****G****U****G****U****U****A****C****U****A** | |
|  |  | NC\_002952.2/1098290-1098197  | **C****G****C****A****C****A****C****A****C****U****A**..**G**.**G****G****G**.**U****G****U****U****U**-----..........................................................................................................................................................................--**U****A****U****A****C****U**GAG**A**.**U****G****A****G****G**CUUG.................................................................................C**C****C****U**.**C****A**...........**A****A****C****C****C****U**UU.........................GA**A**.**C****C****U****G**A.U.**C****U****A****G**.CUUGA...............................A.**C****U****A****G**C**G**.U**A****G****G**.**A**A....**A****G****U****G****U****U****A****C****U****A** | |
|  |  | NC\_002953.3/1086434-1086341  | **C****G****C****A****C****A****C****A****C****U****A**..**G**.**G****G****G**.**U****G****U****U****U**-----..........................................................................................................................................................................--**U****A****U****A****C****U**GAG**A**.**U****G****A****G****G**CUUG.................................................................................C**C****C****U**.**C****A**...........**A****A****C****C****C****U**UU.........................GA**A**.**C****C****U****G**A.U.**C****U****A****G**.CUUGA...............................A.**C****U****A****G**C**G**.U**A****G****G**.**A**A....**A****G****U****G****U****U****A****C****U****A** | |
|  |  | NC\_003923.1/1057711-1057618  | **C****G****C****A****C****A****C****A****C****U****A**..**G**.**G****G****G**.**U****G****U****U****U**-----..........................................................................................................................................................................--**U****A****U****A****C****U**GAG**A**.**U****G****A****G****G**CUUG.................................................................................C**C****C****U**.**C****A**...........**A****A****C****C****C****U**UU.........................GA**A**.**C****C****U****G**A.U.**C****U****A****G**.CUUGA...............................A.**C****U****A****G**C**G**.U**A****G****G**.**A**A....**A****G****U****G****U****U****A****C****U****A** | |
|  |  | NC\_006371.1/809805-809715  | **G****C****U****U****G****U****U****A****G****U****C**..**G**.**G****G****G**.**A****G****C****C****U**-----..........................................................................................................................................................................--**U****U****G****G****C****U**GAG**A**.**C****C****A****C****U**UCG-..................................................................................-**G****U**.**G****G**...........**G****A****C****C****C****G**UU.........................GA**A**.**C****C****U****G**A.U.**C****C****A****A**.UUAAC...............................A.**U****U****G****G**C**G**.G**A****G****G**.**G**A....**A****C****U****A****A****U****A****G****U****A** | |
|  |  | NC\_000913.2/4194303-4194210  | **A****A****U****U****U****C****U****U****G****U****C**..**G**.**G****A****G**.**U****G****C****C****U****U****A**---..........................................................................................................................................................................-**A****C****U****G****G****C****U**GAG**A**.**C****C****G****U****U**UAUU..................................................................................--**C**.**G****G**...........**G****A****U****C****C****G**CG.........................GA**A**.**C****C****U****G**A.U.**C****A****G****G**.CUAAU...............................A.**C****C****U****G**C**G**.A**A****G****G**.**G**A....**A****C****A****A****G****A****G****U****U****A** | |
|  |  | NC\_002655.2/5073851-5073758  | **A****A****U****U****U****C****U****U****G****U****C**..**G**.**G****A****G**.**U****G****C****C****U****U****A**---..........................................................................................................................................................................-**A****C****U****G****G****C****U**GAG**A**.**C****C****G****U****U**UAUU..................................................................................--**C**.**G****G**...........**G****A****U****C****C****G**CG.........................GA**A**.**C****C****U****G**A.U.**C****A****G****G**.CUAAU...............................A.**C****C****U****G**C**G**.A**A****G****G**.**G**A....**A****C****A****A****G****A****G****U****U****A** | |
|  |  | NC\_002695.1/5005105-5005012  | **A****A****U****U****U****C****U****U****G****U****C**..**G**.**G****A****G**.**U****G****C****C****U****U****A**---..........................................................................................................................................................................-**A****C****U****G****G****C****U**GAG**A**.**C****C****G****U****U**UAUU..................................................................................--**C**.**G****G**...........**G****A****U****C****C****G**CG.........................GA**A**.**C****C****U****G**A.U.**C****A****G****G**.CUAAU...............................A.**C****C****U****G**C**G**.A**A****G****G**.**G**A....**A****C****A****A****G****A****G****U****U****A** | |
|  |  | NC\_004337.1/4215114-4215021  | **A****A****U****U****U****C****U****U****G****U****C**..**G**.**G****A****G**.**U****G****C****C****U****U****A**---..........................................................................................................................................................................-**A****C****U****G****G****C****U**GAG**A**.**C****C****G****U****U**UAUU..................................................................................--**C**.**G****G**...........**G****A****U****C****C****G**CG.........................GA**A**.**C****C****U****G**A.U.**C****A****G****G**.CUAAU...............................A.**C****C****U****G**C**G**.A**A****G****G**.**G**A....**A****C****A****A****G****A****G****U****U****A** | |
|  |  | NC\_004431.1/4727487-4727394  | **A****A****U****U****U****C****U****U****G****U****C**..**G**.**G****A****G**.**U****G****C****C****U****U****A**---..........................................................................................................................................................................-**A****C****U****G****G****C****U**GAG**A**.**C****C****G****U****U**UAUU..................................................................................--**C**.**G****G**...........**G****A****U****C****C****G**CG.........................GA**A**.**C****C****U****G**A.U.**C****A****G****G**.CUAAU...............................A.**C****C****U****G**C**G**.A**A****G****G**.**G**A....**A****C****A****A****G****A****G****U****U****A** | |
|  |  | NC\_004741.1/3558106-3558199  | **A****A****U****U****U****C****U****U****G****U****C**..**G**.**G****A****G**.**U****G****C****C****U****U****A**---..........................................................................................................................................................................-**A****C****U****G****G****C****U**GAG**A**.**C****C****G****U****U**UAUU..................................................................................--**C**.**G****G**...........**G****A****U****C****C****G**CG.........................GA**A**.**C****C****U****G**A.U.**C****A****G****G**.CUAAU...............................A.**C****C****U****G**C**G**.A**A****G****G**.**G**A....**A****C****A****A****G****A****G****U****U****A** | |
|  |  | NC\_006582.1/1760187-1760096  | **A****G****A****U****U****C****C****A****C****U****A**..**G**.**G****G****G**.**U****G****C****U****U**-----..........................................................................................................................................................................--**C****G****U****G****C****U**GAG**A**.**A****A****A****G****G**UUCG..................................................................................**C****C****U**.**U****U**...........**A****A****C****U****C****U**U-.........................CA**A**.**C****C****U****G**A.U.**C****U****A****G**.UUCAU...............................A.**C****U****A****G**C**G**.U**A****G****G**.**G**A....**A****G****U****G****G****A****A****A****C****G** | |
|  |  | NZ\_AACK01000018.1/30420-30505  | **A****A****U****U****U****U****U****A****G****U****C**..**G**.**G****G****G**.**U****G****C****U****U****C****U**UA-..........................................................................................................................................................................-**A****G****A****A****G****C****U**GAG**A**.---------..................................................................................---.--........UUA**A****A****C****C****C****G**U-.........................GA**A**.**C****C****U****G**A.U.**A****C****A****G**.UUAGU...............................A.**C****U****G****A**C**G**.U**A****G****G**.**A**A....**A****C****U****A****A****U****A****U****G****C** | |
|  |  | NC\_003078.1/1633734-1633838  | **G****C****C****A****U****U****C****A****C****C****A**..**G**.**G****G****G**.**G****G****U****C****C****C****G**GC-..........................................................................................................................................................................A**A****G****G****G****G****C****U**GAG**A**U**A****C****U****G****C**UAGA..............................................................................GCGC**G****C****A**.**G****U**...........**G****A****C****C****C****G**UU.........................GA**A**.**C****C****U****G**A.U.**C****C****A****G**.UUCAC...............................A.**C****U****G****G**C**G**.U**A****G****G**.**G**A...C**G****G****U****G****C****A****G****A****C****G** | |
|  |  | NC\_006841.1/350580-350670  | **C****G****C****C****A****U****U****A****G****U****C**..**G**.**G****G****G**.**A****G****C****C****A****A**----..........................................................................................................................................................................--**U****A****G****G****C****U**GAG**A**.-**U****C****G****C**AAUA..................................................................................**G****C****G**.--...........**A****A****C****C****C****G**UU.........................GA**A**.**C****C****U****G**A.U.**U****C****A****G**.UUAGU...............................A.**C****U****G****A**C**G**.U**A****G****G**.**G**A....**A****C****U****A****A****U****G****G****U****C** | |
|  |  | NC\_006274.1/5014662-5014554  | **G****U****G****A****U****A****A****A****C****U****A**..**G**.**G****G****A**.**U****G****C****C****U****A****A**CGUAUG.......................................................................................................................................................................C**C****U****A****G****G****C****U**GAG**A**.**G****A****G****A****A**GCGC............................................................................GUGAAC**U****U****C**.**U****U**...........**A****A****C****C****C****U**UU.........................GG**A**.**C****C****U****G**A.U.**C****U****G****G**.CUCGU...............................A.**C****C****A****G**C**G**.U**A****G****G**.**G**A....**A****G****U****U****A****A****C****G****G****U** | |
|  |  | NC\_004463.1/7330682-7330803  | **C****A****C****C****G****U****U****C****C****G****A**..**G**.**G****G****G**.**U****G****C****U****C****C****G**AG-..........................................................................................................................................................................G**A****G****G****A****G****C****U**GAG**A**U**A****C****C****G****C**UAAA.............................................................UGGGCAAAUCCGCCCAGGACC**G****C****G**.**G****U**...........**G****A****C****C****C****U**UU.........................GA**A**.**C****C****U****G**A.U.**C****C****G****G**.GUCAU...............................G.**C****C****G****G**C**G**.A**A****G****G**.**G**A...C**A****G****G****G****A****U****G****U****U****A** | |
|  |  | NC\_006582.1/2048185-2048277  | **U****A****C****A****A****C****C****A****C****U****A**..**G**.**G****G****G**.**U****G****C****G****U****U**----..........................................................................................................................................................................--**U****A****U****G****C****U**GAG**A**.**A****A****G****G****C**GUUU.................................................................................A**G****C****C**.**U****U**...........**A****A****C****C****C****U**--.........................UU**A**.**C****C****U****G**A.U.**C****U****A****G**.GUAAU...............................G.**C****U****A****G**C**G**.A**A****G****G**.**G**A....**A****G****U****G****C****G****G****U****G****U** | |
|  |  | NZ\_AAIU01000039.1/3212-3305  | **A****A****C****C****U****C****U****U****G****U****C**..**G**.**G****A****G**.**U****G****C****C****A****U****U**---..........................................................................................................................................................................-**U****A****U****G****G****C****U**GAG**A**.**C****C****G****U****U**UAUU..................................................................................--**C**.**G****G**...........**G****A****U****C****C****G**UU.........................GA**A**.**C****C****U****G**A.U.**C****A****G****G**.CUAAC...............................A.**C****C****U****G**C**G**.A**A****G****G**.**A**A....**A****C****A****A****G****C****A****U****G****A** | |
|  |  | NZ\_AAAP01001729.1/337-237  | **A****A****U****U****G****U****U****C****C****G****A**..**G**.**G****G****G**.**G****G****C****C****C****C****A**UG-..........................................................................................................................................................................C**C****G****G****G****G****C****U**GAG**A**G**U****G****G****G****C**AAGC................................................................................CA**G****C****C**.**C****U**...........**G****A****C****C****C****U**U-.........................GA**A**.**C****C****U****G**A.U.**C****C****G****G**.CUCGU...............................A.**C****C****G****G**C**G**.G**A****G****G**.**G**A....**C****G****G****G****A****A****C****C****U****G** | |
|  |  | NZ\_AAEB02000012.1/19501-19604  | **G****A****A****G****A****A****G****A****A****C****C**..**G**.**G****G****G**.**A****G****C****U****C****C****C**GGC..........................................................................................................................................................................A**G****G****G****A****G****C****U**GAG**A**G**G****G****C****G****C**UGGG...............................................................................GCC**G****C****G**.**C****C**...........**G****A****C****C****C****G**AG.........................GA**A**.**C****C****U****G**A.U.**C****C****G****G**.GUAAU...............................G.**C****C****G****G**C**G**.G**A****G****G**.**G**A....**G****A****G****C****U****U****U****G****A****G** | |
|  |  | NC\_005139.1/3296358-3296267  | **U****U****U****C****A****C****U****U****G****U****C**..**G**.**G****A****G**.**U****G****C****C****U****U**----..........................................................................................................................................................................--**U****G****G****G****C****U**GAG**A**.**C****C****G****U****U**UAUU..................................................................................--**C**.**G****G**...........**G****A****U****C****C****G**UU.........................GA**A**.**C****C****U****G**A.U.**C****A****G****G**.UUAGA...............................A.**C****C****U****G**C**G**.A**A****G****G**.**G**A....**A****C****A****A****G****A****G****A****A****G** | |
|  |  | NZ\_AAEB02000025.1/11366-11280  | **A****A****G****C****A****G****C****G****C****U****G**..**G**.**G****G****G**.**U****G****C****C****U****C****G**GU-..........................................................................................................................................................................-**A****G****G****G****G****C****U**GAG**A**.---------..................................................................................---.--........GCA**G****A****C****C****C****U**UG.........................GA**A**.**C****C****U****G**A.A.**C****C****G****G**.UUAGC...............................A.**C****C****G****G**C**G**.U**A****G****G**.**G**A....**A****G****C****G****C****G****G****A****U****A** | |
|  |  | NZ\_AAAH01000406.2/303-410  | **A****C****G****C****G****C****U****G****C****C****A**..**G**.**G****G****G**.**A****G****C****C****G****G****G**UAA..........................................................................................................................................................................G**C****C****C****G****G****C****U**GAG**A**G**U****G****C****G****G**CCCG...........................................................................AUUGAUG**C****C****G**.**C****U**...........**G****A****C****C****C****U**UU.........................GA**A**.**C****C****U****G**A.U.**C****U****G****G**.GUUAU...............................G.**C****C****A****G**C**G**.G**A****G****G**.**G**A...A**G****G****C**-**G****U****A****U****U****G** | |
|  |  | NZ\_AAEK01000002.1/10137-10233  | **U****G****A****A****A****C****C****A****C****U****A**..**G**.**G****G****G**.**U****G****C****U****U****G**----..........................................................................................................................................................................-**A****U****U****U****G****C****U**GAG**A**.**G****A****G****G****A**AUAA..................................................................................**U****C****C**.**U****U**...........**A****A****C****C****C****U**UACA.......................AC**A**.**C****C****U****G**A.U.**C****U****A****G**.GUAAU...............................A.**C****U****A****G**C**G**.A**A****G****G**.**G**A....**A****G****U****G****G****A****A****C****A****U** | |
|  |  | NC\_004350.1/666170-666077  | **A****A****A****U****A****U****C****A****C****A****A**..**G**.**G****A****G**.**U****G****C****U****A****A****C**---..........................................................................................................................................................................-**C****U****U****A****G****C****U**GAG**A**.**U****U****G****C****A**UU--..................................................................................**U****G****C**.**A****A**...........**A****A****U****C****C****U**AU.........................GG**A**.**C****C****U****G**A.U.**C****U****A****G**.UUAAU...............................A.**C****U****A****G**C**G**.U**G****G****G**.**A**A....**U****G****U****G****A****U****U****C****G****U** | |
|  |  | NZ\_AAIO01000035.1/17703-17821  | **G****G****C****G****U****C****G****C****C****A****A**..**G**.**G****G****G**.**U****G****U****U****U****G****C**AGCGAAUUAAUUUACGUUGAUUAC.....................................................................................................................................................U**G****C****A****A****A****C****U**GAG**A**.**U****G****U****C****A**GU--..................................................................................**U****G****A**.**C****G**...........**A****A****C****C****C****U**UA.........................GA**A**.**C****C****U****G**A.U.**C****C****G****G**.CUAAU...............................A.**C****C****G****G**C**G**.U**A****G****G**.**A**A....**U****G****G****G****C****C****A****A****U****C** | |
|  |  | NZ\_AADW02000007.1/27974-27879  | **U****A****U****C****U****A****C****A****C****U****A**..**G**.**G****G****G**.**A****G****U****C****U****C****G**CA-..........................................................................................................................................................................-**G****C****A****G****A****C****U**GAG**A**.**C****G****G****A****A**GUA-..................................................................................-**U****C**.**C****G**...........**G****A****C****C****C****U**UU.........................GA**A**.**C****C****U****G**A.U.**C****U****A****G**.UUCGU...............................A.**C****U****A****G**C**G**.G**A****G****G**.**G**A....**A****G****U****G****G****A****U****U****C****C** | |
|  |  | NC\_004547.2/4296609-4296698  | **C****C****G****U****U****C****U****C****A****A****C**..**G**.**G****G****G**.**U****G****C****G****G****A****A**AAU..........................................................................................................................................................................U**U****U****U****C****G****C****U**GAG**A**.---------..................................................................................---.--........AGA**U****A****C****C****C****G**UC.........................GA**A**.**C****C****U****G**A.U.**C****C****G****G**.UUAAU...............................A.**C****C****G****G**C**G**.A**A****G****G**.**G**A...U**U****U****G****A****G****A****G****U****G****C** | |
|  |  | NZ\_AAED02000001.1/28783-28688  | **C****U****C****G****C****U****C****U****A****A****C**..**G**.**G****G****G**.**U****G****C****C****G****U****C**GAU..........................................................................................................................................................................U**G****A****C****G****G****C****U**GAG**A**.**G****G****C****A****A**AG--..................................................................................--**G**.**C****C**...........**A****A****C****C****C****G**CG.........................GA**A**.**C****C****U****G**A.U.**C****C****G****G**.CUCAU...............................A.**C****C****G****G**C**G**.G**A****G****G**.**G**A....**U****U****A****G****A****C****G****C****U****G** | |
|  |  | NC\_003030.1/1541773-1541868  | **U****U****U****U****A****G****U****G****C****U****A**..**G**.**G****G****G**.**U****G****C****C****U****U**----..........................................................................................................................................................................--**U****U****G****G****C****U**GAG**A**.**G****A****A****G****U**UAAA................................................................................AA**A****C****U**.**U****U**...........**A****A****C****C****C****U**UU.........................GA**A**.**C****C****U****G**A.U.**U****C****G****G**.UUAAU...............................U.**C****U****G****A**C**G**.A**A****G****G**.**A**A....**A****G****C****U****U****U****U****U****A****A** | |
|  |  | NZ\_AABN02000006.1/6172-6268  | **U****C****A****A****A****C****A****G****C****U****A**..**G**.**G****G****G**.**U****G****C****C****C****G****U**---..........................................................................................................................................................................-**A****C****G****G****G****C****U**GAG**A**U**U****G****A****A****A**CAGG................................................................................UG**U****U****U**.**C****U**...........**G****A****C****C****C****U**UG.........................GA**A**.**C****C****U****G**A.U.**G****C****G****G**.GUA--...............................A.**C****C****G****C**C**G**.U**A****G****G**.**G**A....**A****G****C****U****G****G****C****A****G****A** | |
|  |  | NC\_006840.1/37315-37420  | **A****G****A****A****A****C****U****U****G****U****C**..**G**.**G****A****G**.**U****G****C****U****U****A****G**UGCUUUUUAAAG.................................................................................................................................................................U**A****C****G****A****G****C****U**GAG**A**.**C****C****G****C****A**G---..................................................................................**U****G****C**.**G****G**...........**G****A****U****C****C****G**UA.........................GA**A**.**C****C****U****G**A.U.**C****A****G****G**.CUAAU...............................A.**C****C****U****G**C**G**.A**A****G****G**.**G**A....**A****C****A****A****G****A****G****A****C****A** | |
|  |  | NC\_004347.1/2560962-2560871  | **G****U****C****A****U****C****U****U****G****U****C**..**G**.**G****A****G**.**U****G****C****C****U****U**----..........................................................................................................................................................................--**U****U****G****G****C****U**GAG**A**.**C****C****G****U****U**UAUU..................................................................................--**C**.**G****G**...........**G****A****U****C****C****G**UU.........................GA**A**.**C****C****U****G**A.U.**C****A****G****G**.UUAAA...............................A.**C****C****U****G**C**G**.A**A****G****G**.**A**A....**A****C****A****A****G****C****A****U****A****A** | |
|  |  | NC\_002570.2/1519534-1519635  | **A****C****A****G****G****A****G****A****C****U****A**..**G**.**G****G****G**.**U****G****U****C****U****G****C**UUA..........................................................................................................................................................................A**G****U****G****G****A****C****U**GAG**A**A**A****A****A****G****G**UGUG................................................................................UU**C****C****U**.**U****U**...........**U****A****C****C****C****U**C-.........................AU**A**.**C****C****U****G**A.U.**C****U****G****G**.AUCAU...............................G.**C****C****A****G**C**G**.U**A****G****G**.**G**A....**A****G****U****C****G****A****C****U****G****C** | |
|  |  | NZ\_AADV02000002.1/103942-103854  | **A****C****U****C****A****U****A****G****C****U****A**..**G**.**G****G****G**.**U****G****U****C****U****G****U**GAA..........................................................................................................................................................................G**C****C****A****G****G****C****U**GAG**A**.---------..................................................................................---.--.........AA**G****A****C****C****C****U**UA.........................GA**A**.**C****C****U****G**AGA.**C****U****G****G**.GUAAU...............................A.**C****C****A****G**C**G**.U**A****G****G**.**G**A....**A****G****C****U****G****U****U****U****A****U** | |
|  |  | NC\_000964.2/1391158-1391053  | **G****G****A****A****A****G****C****A****C****U****A**..**G**.**G****G****G**.**U****G****C****U****G****U**----..........................................................................................................................................................................-**U****U****U****G****G****C****U**GAG**A**.**U****A****A****A****G**CGCG.......................................................................GAAGAAACGCG**C****U****U**.**U****G**...........**A****U****C****C****C****U**UA.........................UG**A**.**C****C****C****G**A.U.**C****U****G****G**.AUAAU...............................A.**C****C****A****G**C**G**.U**G****G****G**.**G**A....**A****G****U****G****C****A****G****G****U****U** | |
|  |  | NC\_003062.1/2700139-2700036  | **C****U****U****G****A****U****C****U****A****A****C**..**G**.**G****G****G**.**U****G****U****C****U****U****C**UGUGCUUUUGUGC................................................................................................................................................................A**G****A****A****G****G****C****U**GAG**A**.**G****G****C****U****U**----..................................................................................--**G**.**C****C**...........**A****A****C****C****C****G**AA.........................GA**A**.**C****C****U****G**A.U.**C****C****G****G**.UUCAU...............................A.**C****C****G****G**C**G**.G**A****G****G**.**G**A....**U****U****A****G****A****C****G****G****C****U** | |
|  |  | NC\_003304.1/2700250-2700147  | **C****U****U****G****A****U****C****U****A****A****C**..**G**.**G****G****G**.**U****G****U****C****U****U****C**UGUGCUUUUGUGC................................................................................................................................................................A**G****A****A****G****G****C****U**GAG**A**.**G****G****C****U****U**----..................................................................................--**G**.**C****C**...........**A****A****C****C****C****G**AA.........................GA**A**.**C****C****U****G**A.U.**C****C****G****G**.UUCAU...............................A.**C****C****G****G**C**G**.G**A****G****G**.**G**A....**U****U****A****G****A****C****G****G****C****U** | |
|  |  | NC\_004193.1/1325497-1325599  | **U****U****U****U****C****A****C****A****C****U****A**..**G**.**G****G****G**.**A****G****C****U****A****U****G**GU-..........................................................................................................................................................................A**A****A****U****A****G****C****U**GAG**A**.**U****G****A****A****C**AUCG..............................................................................GACC**G****U****U**.**U****A**...........**G****A****C****C****C****U**UU.........................GU**A**.**C****U****C****G**A.A.**C****A****G****G**.AUAAU...............................G.**C****C****U****G**C**G**.U**G****A****G**.**G**A....**A****G****U****G****U****G****G****U****C****A** | |
|  |  | NZ\_AAIP01000001.1/41886-41990  | **C****C****U****C****C****A****U****C****C****C****C**..**G**.**G****G****G**.**A****G****C****C****U****G****U**CGCGCAAA.....................................................................................................................................................................A**G****C****A****G****G****U****U**GAG**A**.**G****C****G****G****A**AUAA..................................................................................**A****C****C**.**G****C**...........**G****A****C****C****C****G**UU.........................GA**A**.**C****C****U****G**A.U.**C****C****G****G**.GUAAU...............................A.**C****C****G****G**C**G**.G**A****G****G**.**G**A....**G****G****G****U****U****G****G****C****U****U** | |
|  |  | NC\_004369.1/1190813-1190915  | **U****C****G****A****U****A****U****U****G****C****A**..**C**.**G****G****G**.**U****G****U****C****C****G****G**UGAAAG.......................................................................................................................................................................U**C****C****G****G****G****C****U**GAG**A**G**G****U****G****G****C**ACA-..................................................................................**G****C****C**.**A****C**...........**A****A****C****C****G****U**U-.........................GA**A**.**C****C****U****G**A.U.**C****C****G****G**.GUAAU...............................G.**C****C****G****G**C**G**AU**A****G****G**.**G**A....**G****G****A****A****A****C****A****U****G****A** | |
|  |  | NC\_005835.1/1376155-1376069  | **G****G****C****C****G****U****C****A****C****C****G**..**G**.**G****G****G**.**U****G****C****C****C****C****A**A--..........................................................................................................................................................................-**A****A****G****G****G****C****U**GAG**A**.---------..................................................................................---.--........GCA**U****A****C****C****C****U**UG.........................GA**A**.**C****C****U****G**A.U.**C****C****G****G**.GUCAU...............................G.**C****C****G****G**C**G**.U**A****G****G**.**G**A...A**G****G****U****G****A****C****G****G****C****C** | |
|  |  | NC\_003366.1/1623314-1623411  | **U****U****C****A****U****G****U****G****C****U****A**..**G**.**G****G****G**.**U****G****C****C****U****U**----..........................................................................................................................................................................--**U****A****G****G****C****U**GAG**A**.**G****A****U****G****A**UUAU..............................................................................UUUA**U****C****A**.**U****U**...........**A****A****C****C****C****U**CA.........................AC**A**.**C****C****U****G**A.U.**C****U****G****G**.AAAAU...............................U.**C****C****A****G**C**G**.U**A****G****G**.**G**A....**A****G****C****G****U****U****U****A****G****G** | |
|  |  | NC\_006351.1/1490584-1490486  | **C****C****G****G****C****C****A****U****C****A****G**..**G**.**G****G****G**.**A****G****C****C****G****A****C**G--..........................................................................................................................................................................-**A****U****C****G****G****C****U**GAG**A**G**G****U****U****C****C**GCGC................................................................................GC**G****G****A**.**A****C**...........**G****A****C****C****C****C**U-.........................AA**A**.**C****C****U****G**A.U.**C****C****G****G**.GUAAU...............................G.**C****C****G****G**C**G**.U**A****G****G**.**A**A....**U****G****A****G****G****U****C****C****G****C** | |
|  |  | NZ\_AAEB02000005.1/53165-53264  | **G****A****G****G****C****A****A****G****A****C****C**..**G**.**G****G****G**.**A****G****C****C****G****C**----..........................................................................................................................................................................--**G****A****G****G****C****U**GAG**A**G**G****G****C****G****C**CGCG...............................................................................CGG**G****C****G**.**C****C**...........**G****A****C****C****C****G**UU.........................GA**A**.**C****C****U****G**A.U.**C****C****G****G**.GUAAU...............................G.**C****C****G****G**C**G**.C**A****G****G**.**G**A..GA**U****U****C****U****C****G****G****A****U****G** | |
|  |  | NZ\_AAHS01000090.1/13480-13578  | **C****C****G****G****C****C****A****U****C****A****G**..**G**.**G****G****G**.**A****G****C****C****G****A****C**G--..........................................................................................................................................................................-**A****U****C****G****G****C****U**GAG**A**G**G****U****U****C****C**GCGC................................................................................GC**G****G****A**.**A****C**...........**G****A****C****C****C****C**U-.........................AA**A**.**C****C****U****G**A.U.**C****C****G****G**.GUAAU...............................G.**C****C****G****G**C**G**.U**A****G****G**.**A**A....**U****G****A****G****G****U****C****C****G****C** | |
|  |  | NZ\_AAHT01000002.1/93633-93535  | **C****C****G****G****C****C****A****U****C****A****G**..**G**.**G****G****G**.**A****G****C****C****G****A****C**G--..........................................................................................................................................................................-**A****U****C****G****G****C****U**GAG**A**G**G****U****U****C****C**GCGC................................................................................GC**G****G****A**.**A****C**...........**G****A****C****C****C****C**U-.........................AA**A**.**C****C****U****G**A.U.**C****C****G****G**.GUAAU...............................G.**C****C****G****G**C**G**.U**A****G****G**.**A**A....**U****G****A****G****G****U****C****C****G****C** | |
|  |  | NZ\_AAHU01000006.1/169192-169290  | **C****C****G****G****C****C****A****U****C****A****G**..**G**.**G****G****G**.**A****G****C****C****G****A****C**G--..........................................................................................................................................................................-**A****U****C****G****G****C****U**GAG**A**G**G****U****U****C****C**GCGC................................................................................GC**G****G****A**.**A****C**...........**G****A****C****C****C****C**U-.........................AA**A**.**C****C****U****G**A.U.**C****C****G****G**.GUAAU...............................G.**C****C****G****G**C**G**.U**A****G****G**.**A**A....**U****G****A****G****G****U****C****C****G****C** | |
|  |  | NZ\_AAHV01000011.1/10644-10546  | **C****C****G****G****C****C****A****U****C****A****G**..**G**.**G****G****G**.**A****G****C****C****G****A****C**G--..........................................................................................................................................................................-**A****U****C****G****G****C****U**GAG**A**G**G****U****U****C****C**GCGC................................................................................GC**G****G****A**.**A****C**...........**G****A****C****C****C****C**U-.........................AA**A**.**C****C****U****G**A.U.**C****C****G****G**.GUAAU...............................G.**C****C****G****G**C**G**.U**A****G****G**.**A**A....**U****G****A****G****G****U****C****C****G****C** | |
|  |  | NZ\_AAHW01000054.1/13454-13552  | **C****C****G****G****C****C****A****U****C****A****G**..**G**.**G****G****G**.**A****G****C****C****G****A****C**G--..........................................................................................................................................................................-**A****U****C****G****G****C****U**GAG**A**G**G****U****U****C****C**GCGC................................................................................GC**G****G****A**.**A****C**...........**G****A****C****C****C****C**U-.........................AA**A**.**C****C****U****G**A.U.**C****C****G****G**.GUAAU...............................G.**C****C****G****G**C**G**.U**A****G****G**.**A**A....**U****G****A****G****G****U****C****C****G****C** | |
|  |  | NC\_006526.1/155239-155341  | **G****A****G****G****C****A****U****C****C****C****C**..**G**.**G****G****G**.**G****G****C****C****G****U****A**UAA..........................................................................................................................................................................A**U****A****C****G****G****C****U**GAG**A**A**U****G****A****G****C**UGAU.................................................................................U**G****C****U**.**C****U**...........**A****A****C****C****C****G**UC.........................GA**A**.**C****C****U****G**A.U.**C****C****G****G**CUUAAC...............................A.**C****C****G****G**C**G**.U**A****G****G**.**G**A....**G****G****G****A****A****G****G****C****A****U** | |
|  |  | NC\_002976.3/658547-658452  | **U****G****A****A****C****G****C****A****C****U****A**..**G**.**G****G****G**.**U****G****U****A****U****U**----..........................................................................................................................................................................-**C****U****U****U****A****C****U**GAG**A**.**U****G****A****G****G**CCAA..................................................................................**C****C****U**.**C****A**...........**A****A****C****C****C****U**UC.........................GA**A**.**C****C****U****G**A.U.**C****U****A****G**.CUAGUU..............................A.**C****U****A****G**C**G**.U**A****G****G**.**A**A....**A****G****U****G****U****U****G****U****U****A** | |
|  |  | NC\_004461.1/766031-765936  | **U****G****A****A****C****G****C****A****C****U****A**..**G**.**G****G****G**.**U****G****U****A****U****U**----..........................................................................................................................................................................-**C****U****U****U****A****C****U**GAG**A**.**U****G****A****G****G**CCAA..................................................................................**C****C****U**.**C****A**...........**A****A****C****C****C****U**UC.........................GA**A**.**C****C****U****G**A.U.**C****U****A****G**.CUAGUU..............................A.**C****U****A****G**C**G**.U**A****G****G**.**A**A....**A****G****U****G****U****U****G****U****U****A** | |
|  |  | NZ\_AAAQ02000002.1/19834-19932  | **A****C****C****A****A****A****C****G****A****C****A**..**G**.**G****G****G**.**A****G****C****G****C****C****U**C--..........................................................................................................................................................................-**U****A****G****C****G****C****U**GAG**A**G**U****G****C****G****G**CACA.................................................................................G**C****C****G**.**C****A**...........**G****A****C****C****C****U**UA.........................CU**A**.**C****C****U****G**A.U.**C****U****G****G**.GUAAU...............................G.**C****C****A****G**C**G**.A**A****G****G**.**A**A....**G****U****C****G****U****G****G****G****A****A** | |
|  |  | NZ\_AAFQ02000001.1/130106-130002  | **C****A****A****A****G****C****U****U****G****U****C**..**G**.**G****G****G**.**U****G****C****C****A****A****A**GAUGAUUCAU...................................................................................................................................................................C**U****G****A****G****G****C****U**GAG**A**.**U****C****G****C****A**GA--..................................................................................**U****G****C**.**G****A**...........**G****A****C****C****C****G**CC.........................GA**A**.**C****C****U****G**A.U.**C****G****G****G**.AUCAU...............................G.**C****C****C****G**C**G**.U**A****G****G**.**G**A....**A****C****A****A****G****G****C****A****C****U** | |
|  |  | NZ\_AAIS01000001.1/575165-575037  | **G****G****G****U****G****U****U****C****C****G****A**..**G**.**G****G****G**.**U****G****C****U****C****C****G**UC-..........................................................................................................................................................................A**G****G****G****A****G****C****U**GAG**A**U**A****C****C****G****C**AAGC......................................................UCGCAAUCCGAAGACGGAUUGCAGGACC**G****C****G**.**G****U**...........**G****A****C****C****C****U**UU.........................GA**A**.**C****C****U****G**A.U.**C****C****G****G**.GUCAU...............................G.**C****C****G****G**C**G**.A**A****G****G**.**G**A...C**A****G****G****G****A****U****G****U****U****U** | |
|  |  | NC\_004557.1/222874-222767  | **A****U****G****U****A****G****U****G****C****U****A**..**G**.**G****G****G**.**U****G****C****U****A****U****A**U--..........................................................................................................................................................................-**A****A****G****A****G****C****U**GAG**A**.**A****A****U****G****A**AUUA......................................................................UUUAUGAUCAAU**U****C****A**.**U****U**...........**A****A****C****C****C****U**U-.........................GU**A**.**C****C****U****G**A.A.**C****U****A****G**.AUAAU...............................U.**C****U****A****G**C**G**.U**A****G****G**.**G**A....**A****G****C****C****U****U****U****U****A****U** | |
|  |  | NC\_006512.1/2529689-2529598  | **C****G****C****U****U****U****A****C****C****A****A**..**G**.**G****G****G**.**U****G****C****C****G****G****C**---..........................................................................................................................................................................-**A****A****C****G****G****C****U**GAG**A**.**U****G****C****A****U**UA--..................................................................................--**G**.**C****G**...........**A****A****C****C****C****U**UA.........................AA**A**.**C****C****U****G**A.U.**C****C****G****G**.UUAAU...............................A.**C****U****G****G**C**G**.U**A****G****G**.**G**A....**U****G****G****U****A****G****A****A****A****A** | |
|  |  | NZ\_AAFG02000004.1/195897-195800  | **C****G****C****C****C****C****A****C****C****U****C**..**G**.**G****G****G**.**U****G****C****G****C****G****A**GCG..........................................................................................................................................................................G**U****C****G****U****G****C****U**GAG**A**.**U****G****C****C****U**AUG-..................................................................................-**G****G**.**C****G**...........**A****A****C****C****C****G**CU.........................GA**A**.**C****C****U****G**A.A.**C****C****G****G**.AUUAU...............................A.**C****C****G****G**C**G**.U**A****G****G**.**G**A....**A****G****G****U****U****U****G****G****U****A** | |
|  |  | NC\_003155.3/7332955-7332849  | **U****A****A****A****G****C****A****C****U****C****G**..**C**.**G****G****G**.**A****G****C****C****C****G****G**ACGC.........................................................................................................................................................................A**C****C****G****G****G****C****U**GAG**A**G**G****G****A****G****G**CUGG.............................................................................GACGG**C****C****U**.**C****C**...........**G****A****C****C****G****U**AC.........................GA**A**.**C****C****U****G**A.U.**C****C****G****G**.GUCAU...............................G.**C****C****G****G**C**G**.A**A****G****G**.**G**A....**G****G****G****G****C****U****G****G****A****C** | |
|  |  | NZ\_AAHE01000005.1/33477-33587  | **G****G****C****A****G****U****C****A****C****C****A**..**G**.**G****G****G**.**U****G****C****C****U****U****U**CCGCGCGAGGAGC................................................................................................................................................................G**G****A****G****G****G****C****U**GAG**A**.**G****G****G****C****A**AGCG..................................................................................**U****G****C**.**C****C**...........**A****A****C****C****C****U**AG.........................GA**A**.**C****C****U****G**A.U.**C****U****G****G**.UUCAG...............................A.**C****C****A****G**C**G**.G**A****G****G**.**G**A...G**C****G****U****G****A****C****G****G****G****U** | |
|  |  | NZ\_AAHE01000109.1/458-348  | **G****G****C****A****G****U****C****A****C****C****A**..**G**.**G****G****G**.**U****G****C****C****U****U****U**CCGCGCGAGGAGC................................................................................................................................................................G**G****A****G****G****G****C****U**GAG**A**.**G****G****G****C****A**AGCG..................................................................................**U****G****C**.**C****C**...........**A****A****C****C****C****U**AG.........................GA**A**.**C****C****U****G**A.U.**C****U****G****G**.UUCAG...............................A.**C****C****A****G**C**G**.G**A****G****G**.**G**A...G**C****G****U****G****A****C****G****G****G****U** | |
|  |  | NZ\_AAIV01000034.1/37602-37510  | **G****A****C****A****U****C****U****U****G****U****C**..**G**.**G****A****G**.**U****G****C****C****A****U**----..........................................................................................................................................................................-**U****A****U****G****G****C****U**GAG**A**.**C****C****G****U****U**UAUU..................................................................................--**C**.**G****G**...........**G****A****U****C****C****G**UU.........................GA**A**.**C****C****U****G**A.U.**C****A****G****G**.CUAGA...............................A.**C****C****U****G**C**G**.A**A****G****G**.**A**A....**A****C****A****A****G****C****G****U****A****A** | |
|  |  | NC\_004547.2/273692-273562  | **C****A****A****A****U****C****U****U****G****U****C**..**G**.**G****A****G**.**U****G****C****C****U****A****G**CGUGCUUGUGUUUCGUUUUUACGGAACCUAUGAGCG.........................................................................................................................................C**A****C****A****G****G****C****U**GAG**A**.**C****C****G****U****U**AAUU..................................................................................--**C**.**G****G**...........**G****A****U****C****C****G**CG.........................GA**A**.**C****C****U****G**A.U.**C****G****G****G**.UUAAU...............................A.**C****C****U****G**C**G**.A**A****G****G**.**G**A....**A****C****A****A****G****A****G****U****A****A** | |
|  |  | NZ\_AAAP01000319.1/234-137  | **G****G****U****G****A****A****C****G****A****C****A**..**G**.**G****G****G**.**A****G****C****U****C****C****U**GG-..........................................................................................................................................................................-**A****G****G****A****G****C****U**GAG**A**G**U****G****C****G****G**ACCA..................................................................................**C****C****G**.**C****A**...........**G****A****C****C****C****U**C-.........................GA**A**.**C****C****U****G**A.U.**C****C****G****G**.UUAGC...............................A.**C****C****G****G**U**G**.G**A****G****G**.**A**A....**G****U****C****G****G****G****A****A****U****C** | |
|  |  | NC\_005070.1/134905-135006  | **A****A****A****C****A****C****C****A****C****U****A**..**G**.**G****G****G**.**U****G****C****C****U****C****G**AUCGGUUUUCUGGUCG.............................................................................................................................................................A**C****G****G****G****G****C****U**GAG**A**.---------..................................................................................---.--........UCA**C****A****C****C****C****U**CU.........................GA**A**.**C****C****U****G**A.C.**C****C****G****G**.GUCAU...............................G.**C****C****G****G**C**G**.A**A****G****G**.**G**A....**A****G****U****G****A****C****C****A****G****C** | |
|  |  | NC\_003112.1/2190524-2190620  | **A****G****C****U****C****C****U****U****G****U****C**..**G**.**G****A****G**.**U****G****C****C****G****C****C**GCC..........................................................................................................................................................................G**G****G****C****G****G****C****U**GAG**A**.**U****U****G****C****G**AAA-..................................................................................-**G****C**.**A****G**...........**A****A****U****C****C****G**UA.........................GA**A**.**C****C****U****G**-.U.**C****G****G****G**.GUAAU...............................G.**C****C****U****G**C**G**.U**A****G****G**.**A**A....**A****C****A****A****A****C****C****G****U****C** | |
|  |  | NC\_005126.1/527145-527027  | **A****C****U****U****U****C****U****U****G****U****C**..**G**.**G****A****G**.**U****G****C****C****U****A****G**CGUAAGCUGUUUUAUCGCAGCAUA.....................................................................................................................................................C**A****C****A****G****G****C****U**GAG**A**.**C****C****G****U****U**AAUU..................................................................................--**C**.**G****G**...........**G****A****U****C****C****G**CG.........................GA**A**.**C****C****U****G**A.U.**C****G****G****G**.UUAAU...............................A.**C****C****C****G**C**G**.A**A****G****G**.**G**A....**A****C****A****A****G****A****G****U****A****A** | |
|  |  | NZ\_AAIV01000019.1/28422-28522  | **G****U****G****G****C****C****G****C****C****A****A**..**G**.**G****G****G**.**U****G****U****U****C****A****G**UAAAA........................................................................................................................................................................C**C****U****G****A****A****C****U**GAG**A**.**U****G****U****C****U**GUAU..................................................................................-**G****A**.**C****G**...........**A****A****C****C****C****U**UA.........................GA**A**.**C****C****U****G**A.U.**C****C****G****G**.CUUAU...............................A.**C****C****G****G**C**G**.U**A****G****G**.**A**A....**U****G****G****G****C****C****A****C****A****C** | |
|  |  | NZ\_AABK03000021.1/78082-78171  | **C****C****A****C****U****A****A****G****C****U****A**..**G**.**G****G****G**.**U****G****C****C****U****A****A**AAUA.........................................................................................................................................................................U**U****U****A****G****G****C****U**GAG**A**.---------..................................................................................---.--.........AA**U****A****C****C****C****U**UA.........................GA**A**.**C****C****U****G**AGA.**C****U****G****G**.UUAAU...............................A.**C****C****A****G**C**G**.G**A****G****G**.**G**A....**A****G****C****U****G****U****U****U****A****U** | |
|  |  | NC\_004193.1/1419474-1419570  | **A****A****A****G****C****C****U****G****C****A****A**..**G**.**G****G****G**.**A****G****C****C****A****U**----..........................................................................................................................................................................--**A****U****G****G****C****U**GAG**A**G**U****G****G****A****C**GUAU...............................................................................AAU**G****U****U**.**C****U**...........**G****A****C****C****C****U**UU.........................GA**A**.**C****C****U****G**-.U.-**U****A****G**.UUAGU...............................A.**C****U****A****G**C**G**.U**A****G****G**.**G**A...U**U****G****U****A****G****U****C****U****U****U** | |
|  |  | NC\_001264.1/178388-178282  | **A****U****C****G****U****C****A**-**A****C****A**..**G**.**G****G****G**.**U****G****C****C****U****C****C**GCAUAUGGGC...................................................................................................................................................................C**G****G****A****G****G****C****U**GAG**A**.**G****G****G****C****A**ACUC.................................................................................G**G****G****C**.**C****U**...........**A****A****C****C****C****U**AU.........................GA**A**.**C****C****U****G**A.A.**C****U****G****G**.UUAGC...............................A.**C****C****A****G**C**G**.G**A****G****G**.**G**A....**G****U****G****U****G****A****C****G****G****G** | |
|  |  | NZ\_AAHZ01000002.1/307891-308018  | **G****A****U****A****G****C****U****U****G****A****C**..**G**.**G****G****G**.**U****G****C****C****G****G****U**GG-..........................................................................................................................................................................A**A****C****C****G****G****C****U**GAG**A**.**U****C****G****U****C**CUGC.....................................................GCCGCGACUCAUACGAAGCGCGGCGCCAC**G****A****C**.**G****G**...........**A****U****C****C****C****G**AC.........................GA**A**.**C****C****U****G**A.U.**C****C****G****G**.CUAGU...............................A.**C****C****G****G**C**G**.U**A****G****G**.**G**A....**U****C****A****A****G****C****G****G****A****G** | |
|  |  | NC\_003888.3/2264719-2264823  | **C****A****A****G****G****C****A****C****U****C****G**..**C**.**G****G****G**.**A****G****C****C****C****G****G**ACGC.........................................................................................................................................................................A**C****C****G****G****G****C****U**GAG**A**G**G****G****A****G****G**CUGG...............................................................................CGG**C****C****U**.**C****C**...........**G****A****C****C****G****U**AC.........................GA**A**.**C****C****U****G**A.U.**C****C****G****G**.GUCAU...............................G.**C****C****G****G**C**G**.A**A****G****G**.**G**A....**G****G****G****G****C****U****G****G****A****C** | |
|  |  | NC\_003454.1/862422-862326  | **A****U****A****U****A****U****G****U****A****C****U**..**G**.**G****G****G**.**A****G****C****U****U**-----..........................................................................................................................................................................--**U****G****U****G****C****U**GAG**A**.**U****U****A****G****A**ACCU..............................................................................UUUU**U****C****U**.**U****A**...........**G****A****C****C****C****A**UA.........................GU**A**.**C****C****U****G**A.U.**U****U****G****G**.AUAAU...............................G.**C****C****A****A**C**G**.A**A****G****G**.**G**A....**G****U****A****C****C****A****U****C****U****U** | |
|  |  | NZ\_AAEV01000019.1/5894-5796  | **U****A****U****C****U****G****C****A****C****U****A**..**G**.**G****G****G**.**U****G****U****U****C****U****U**AG-..........................................................................................................................................................................-**A****A****G****A****A****C****U**GAG**A**.**U****A****C****G****C**UUGU...............................................................................UCA**G****C****G**.**U****G**...........**A****U****C****C****C****U**UU.........................GA**A**.**C****C****U****G**-.U.-**A****A****G**.UUAGU...............................A.**C****U****U****G**C**G**.A**A****G****G**.**A**A....**A****G****U****G****U****U****U****A****A****U** | |
|  |  | NZ\_AAIU01000009.1/25457-25355  | **C****C****U****C****G****C****G****C****C****A****A**..**G**.**G****G****G**.**U****G****U****U****A****G****G**GCUAGUUG.....................................................................................................................................................................C**U****C****U****G****A****C****U**GAG**A**.**U****A****U****C****G**UUU-..................................................................................-**G****A**.**U****A**...........**A****A****C****C****C****U**UA.........................GA**A**.**C****C****U****G**A.U.**C****C****G****G**.CUAAU...............................A.**C****C****G****G**C**G**.U**A****G****G**.**A**A....**U****G****G****G****C****U****U****A****U****U** | |
|  |  | NC\_003450.3/2150221-2150328  | **G****A****C****U****U****A****C****C****C****C****A**..**C**.**G****G****G**.**U****G****C****C****C****A****A**UGC..........................................................................................................................................................................A**U****U****G****G****G****C****U**GAG**A**U**U****G****C****G****C**GCUG.............................................................................UUGCU**G****C****G**.**C****G**..........G**G****A****C****C****G****U**UC.........................GA**A**.**C****C****U****G**-.U.**C****U****G****G**.UUAAC...............................A.**C****C****A****G**C**G**.A**A****G****G**.**A**A..GC**G****A****G****G****A****U****U****G****A****U** | |
|  |  | NC\_006958.1/2120271-2120378  | **G****A****C****U****U****A****C****C****C****C****A**..**C**.**G****G****G**.**U****G****C****C****C****A****A**UGC..........................................................................................................................................................................A**U****U****G****G****G****C****U**GAG**A**U**U****G****C****G****C**GCUG.............................................................................UUGCU**G****C****G**.**C****G**..........G**G****A****C****C****G****U**UC.........................GA**A**.**C****C****U****G**-.U.**C****U****G****G**.UUAAC...............................A.**C****C****A****G**C**G**.A**A****G****G**.**A**A..GC**G****A****G****G****A****U****U****G****A****U** | |
|  |  | NC\_002976.3/2366921-2367015  | **U****A****G****U****A****U****C****A****C****U****A**..**G**.**G****G****G**.**U****G****C****A****A****U****U**CA-..........................................................................................................................................................................-**A****U****U****U****G****C****U**GAG**A**.**G****A****A****A****G**UCUA..................................................................................**C****U****U**.**U****C**...........**A****A****C****C****C****U**U-.........................GA**A**.**C****C****U****G**-.U.-**U****G****G**.UUAGC...............................A.**C****C****G****G**C**G**.U**A****G****G**.**A**A....**A****G****U****G****A****G****C****A****A****U** | |
|  |  | NC\_004461.1/250277-250183  | **U****A****G****U****A****U****C****A****C****U****A**..**G**.**G****G****G**.**U****G****C****A****A****U****U**CA-..........................................................................................................................................................................-**A****U****U****U****G****C****U**GAG**A**.**G****A****A****A****G**UCUA..................................................................................**C****U****U**.**U****C**...........**A****A****C****C****C****U**U-.........................GA**A**.**C****C****U****G**-.U.-**U****G****G**.UUAGC...............................A.**C****C****G****G**C**G**.U**A****G****G**.**A**A....**A****G****U****G****A****G****C****A****A****U** | |
|  |  | NZ\_AAIN01000009.1/67996-68094  | **G****G****C****G****G****C****G****C****C****A****A**..**G**.**G****G****G**.**U****G****U****C****U****G****C**CGCA.........................................................................................................................................................................U**G****C****A****G****A****C****U**GAG**A**.**U****G****U****C****U**CAG-..................................................................................-**G****A**.**C****G**...........**A****A****C****C****C****U**UA.........................GA**A**.**C****C****U****G**A.U.**C****C****G****G**.CUGAU...............................A.**C****C****G****G**C**G**.U**A****G****G**.**A**A....**U****G****G****G****C****U****G****A****U****A** | |
|  |  | NZ\_AAAG02000003.1/373081-373206  | **A****A****C****C****U****G****U****U****C****C****A**..**G**.**G****G****G**.**G****G****C****C****C****C****G**GC-..........................................................................................................................................................................A**A****G****G****G****G****C****U**GAG**A**U**A****C****C****G****A**UGGC.........................................................UGAAGGGGGCUUUCCCCGAGGGCGC**G****C****G**.**G****U**...........**G****A****C****C****C****U**UC.........................GA**A**.**C****C****U****G**A.U.**C****C****G****G**.GUCAU...............................G.**C****C****G****G**C**G**.A**A****G****G**.**G**A...C**G****G****A****A****C****G****C****G****G****G** | |
|  |  | NZ\_AAIN01000016.1/37471-37380  | **C****A****G****C****A****C****U****U****G****U****C**..**G**.**G****A****G**.**U****G****C****C****U****U**----..........................................................................................................................................................................--**A****G****G****G****C****U**GAG**A**.**C****C****G****U****U**UAUU..................................................................................--**C**.**G****G**...........**G****A****U****C****C****G**UU.........................GA**A**.**C****C****U****G**A.U.**C****A****G****G**.UUAGA...............................A.**C****C****U****G**C**G**.A**A****G****G**.**A**A....**A****C****A****A****G****C****A****U****C****A** | |
|  |  | NC\_003116.1/339009-338913  | **G****C****U****U****U****C****U****U****G****U****C**..**G**.**G****A****G**.**U****G****C****C****G****C****C**GCC..........................................................................................................................................................................G**G****G****C****G****G****C****U**GAG**A**.**U****U****G****C****G**AAA-..................................................................................-**G****C**.**A****G**...........**A****A****U****C****C****G**UA.........................GA**A**.**C****C****U****G**-.U.**C****G****G****G**.GUAAU...............................G.**C****C****U****G**C**G**.U**A****G****G**.**A**A....**A****C****A****A****A****C****C****G****U****C** | |
|  |  | NZ\_AAHG01000006.1/50490-50589  | **U****A****C****G****A****C**-**C****A****C****A**..**C**.**G****G****G**.**U****G****C****C****C****U****U**GC-..........................................................................................................................................................................-**A****A****G****G****G****C****U**GAG**A**U**C****G****G****G****C**UGAC...............................................................................GCG**G****C****C**.**U****G**..........C**G****A****C****C****G****U**U-.........................GA**A**.**C****C****U****G**-.U.**C****C****G****G**.GUAAU...............................G.**C****C****G****G**C**G**.A**A****G****G**.**A**A....**G****U****G****A****G****U****A****U****U****C** | |
|  |  | NZ\_AAAS03000003.1/7626-7721  | **G****C****A****G****C****C****U****G****C****U****G**..**G**.**G****G****G**.**A****G****U****U****C****U****U**C--..........................................................................................................................................................................-**G****A****G****A****A****C****U**GAG**A**.**C****G****G****G****C**ACC-..................................................................................**G****C****C**.**C****G**...........**A****A****C****C****C****U**UA.........................CC**A**.**C****C****U****G**A.U.**C****C****G****G**.GUAAU...............................G.**C****C****G****G**C**G**.U**A****G****G**.**G**A....**A****G****C****G****G****C****C****A****G****A** | |
|  |  | NC\_002978.6/463803-463708  | **U****A****C****A****C****G****C****A****U****U****A**..**G**.**G****G****G**.**U****G****C****U****C****U****A**G--..........................................................................................................................................................................-**A****A****A****A****G****C****U**GAG**A**.**G****U****A****C****A**UAAA..................................................................................-**G****U**.**A****C**...........**A****A****C****C****C****U**UU.........................GA**A**.**C****C****U****G**A.U.**A****U****U****G**.UUAAA...............................G.**C****A****A****G**C**G**.U**A****G****G**.**G**A....**A****A****U****A****U****A****U****G****U****A** | |
|  |  | NC\_006085.1/186523-186619  | **G****A****G****C****A****A****C****G****A****C****A**..**G**.**G****G****G**.**A****G****C****A****U****C****G**UC-..........................................................................................................................................................................-**G****G****A****U****G****C****U**GAG**A**G**U****G****G****G****C**ACC-..................................................................................**G****C****C**.**C****A**...........**G****A****C****C****C****U**C-.........................GA**A**.**C****C****U****G**A.A.**C****C****G****G**.UUAGG...............................A.**C****C****G****G**C**G**.U**A****G****G**.**G**A....**G****U****C****G****G****G****C****U****C****U** | |
|  |  | NC\_002663.1/444121-444206  | **G****A****C****U****C****U****U****A****G****U****C**..**G**.**G****G****G**.**U****G****C****U****A****U****U**GC-..........................................................................................................................................................................-**G****U****U****A****G****C****U**GAG**A**.---------..................................................................................---.--........UGA**U****A****C****C****C****G**U-.........................GA**A**.**C****C****U****G**A.U.**G****C****A****G**.UUAAU...............................A.**C****U****G****A**C**G**.U**A****G****G**.**A**A....**A****C****U****A****G****C****A****G****U****C** | |
|  |  | NC\_003228.3/2983140-2983225  | **G****U****A****A****A****G****A****C****A****A****A**..**G**.**G****G****G**.**U****G****C****C****A****C****C**C--..........................................................................................................................................................................-**G****G****U****G****G****C****U**GAG**A**.---------..................................................................................---.--........UUA**U****A****C****C****C****U**AA.........................GA**A**.**C****C****U****G**A.U.**G****C****A****G**.UUAGU...............................A.**C****U****G****C**C**G**.A**A****G****G**.**G**A....**U****U****G****U****G****U****A****U****U****U** | |
|  |  | NC\_006347.1/2909526-2909611  | **G****U****A****A****A****G****A****C****A****A****A**..**G**.**G****G****G**.**U****G****C****C****A****C****C**C--..........................................................................................................................................................................-**G****G****U****G****G****C****U**GAG**A**.---------..................................................................................---.--........UUA**U****A****C****C****C****U**AA.........................GA**A**.**C****C****U****G**A.U.**G****C****A****G**.UUAGU...............................A.**C****U****G****C**C**G**.A**A****G****G**.**G**A....**U****U****G****U****G****U****A****U****U****U** | |
|  |  | NC\_006155.1/350736-350618  | **A****G****A****C****U****C****U****U****G****U****C**..**G**.**G****A****G**.**U****G****C****C****U****A****G**CACCUGCUUUUUUAGGAAAGCAAA.....................................................................................................................................................C**G****C****A****G****G****C****U**GAG**A**.**C****C****G****U****U**AAUU..................................................................................--**C**.**G****G**...........**G****A****U****C****C****G**CG.........................GA**A**.**C****C****U****G**A.U.**C****G****G****G**.UUAAU...............................A.**C****C****C****G**C**G**.A**A****G****G**.**G**A....**A****C****A****A****G****A****G****U****A****A** | |
|  |  | NC\_000911.1/2999078-2999166  | **U****G****C****C****A****U****A****G****C****U****A**..**G**.**G****G****G**.**U****G****U****C****U****A****G**AAA..........................................................................................................................................................................G**C****U****A****G****G****C****U**GAG**A**.---------..................................................................................---.--.........AA**A****A****C****C****C****U**UA.........................GA**A**.**C****C****U****G**AGA.**C****U****G****G**.GUAAU...............................A.**C****C****A****G**C**G**.G**A****G****G**.**G**A....**A****G****C****U****C****A****C****C****A****U** | |
|  |  | NC\_002937.3/2184870-2184777  | **U****C****A****G****U****C****A****G****C****U****A**..**G**.**G****G****G**.**A****G****C****C****U****U**----..........................................................................................................................................................................--**C****G****G****G****C****U**GAG**A**G**U****G****G****G****C**ACGU..................................................................................-**C****C**.**C****A**...........**G****A****C****C****C****U**GU.........................GA**A**.**C****C****U****G**A.C.**G****C****A****G**.UUCAC...............................A.**C****U****G****C**C**G**.U**A****G****G**.**G**A....**A****G****C****U****G****A****G****C****G****C** | |
|  |  | NC\_003143.1/4190343-4190461  | **A****G****G****C****U****C****U****U****G****U****C**..**G**.**G****A****G**.**U****G****C****C****U****A****G**CACCUGCUUUUUUAGGAAAGCAAA.....................................................................................................................................................C**G****C****A****G****G****C****U**GAG**A**.**C****C****G****U****U**AAUU..................................................................................--**C**.**G****G**...........**G****A****U****C****C****G**CG.........................GA**A**.**C****C****U****G**A.U.**C****G****G****G**.UUAAU...............................A.**C****C****C****G**C**G**.A**A****G****G**.**G**A....**A****C****A****A****G****A****G****U****A****A** | |
|  |  | NC\_004088.1/555372-555254  | **A****G****G****C****U****C****U****U****G****U****C**..**G**.**G****A****G**.**U****G****C****C****U****A****G**CACCUGCUUUUUUAGGAAAGCAAA.....................................................................................................................................................C**G****C****A****G****G****C****U**GAG**A**.**C****C****G****U****U**AAUU..................................................................................--**C**.**G****G**...........**G****A****U****C****C****G**CG.........................GA**A**.**C****C****U****G**A.U.**C****G****G****G**.UUAAU...............................A.**C****C****C****G**C**G**.A**A****G****G**.**G**A....**A****C****A****A****G****A****G****U****A****A** | |
|  |  | NC\_005810.1/3451026-3451144  | **A****G****G****C****U****C****U****U****G****U****C**..**G**.**G****A****G**.**U****G****C****C****U****A****G**CACCUGCUUUUUUAGGAAAGCAAA.....................................................................................................................................................C**G****C****A****G****G****C****U**GAG**A**.**C****C****G****U****U**AAUU..................................................................................--**C**.**G****G**...........**G****A****U****C****C****G**CG.........................GA**A**.**C****C****U****G**A.U.**C****G****G****G**.UUAAU...............................A.**C****C****C****G**C**G**.A**A****G****G**.**G**A....**A****C****A****A****G****A****G****U****A****A** | |
|  |  | NC\_004431.1/2476104-2476015  | **C****C****A****A****A****C****G****A****C****U****C**..**G**.**G****G****G**.**U****G****C****C****C****U****U**CUUUG........................................................................................................................................................................U**G****A****A****G****G****C****U**GAG**A**.---------..................................................................................---.--.........AA**U****A****C****C****C****G**UA.........................UC**A**.**C****C****U****G**A.U.**C****U****G****G**.AUAAU...............................G.**C****C****A****G**C**G**.U**A****G****G**.**G**A....**A****G****U****C****A****C****G****G****A****C** | |
|  |  | NZ\_AAIT01000010.1/164659-164535  | **G****G****C****G****C****C****A****C****C****A****A**..**G**.**G****G****G**.**A****G****C****C****C****C****G**GC-..........................................................................................................................................................................A**A****G****G****G****G****C****U**GAG**A**A**A****C****C****G****C**UGGC.........................................................CUCGAUCGUCCCGGCGACGGGCGGC**G****C****G**.**G****U**...........**G****A****C****C****C****U**UU.........................GA**A**.**C****C****U****G**A.C.**C****C****G****G**.AUCAU...............................G.**C****C****G****G**C**G**.G**A****G****G**.**A**A....**U****G****G****G****A****C****A****G****A****U** | |
|  |  | NC\_006087.1/1470316-1470213  | **G****C****U****U****C****U****G****A****A****C****A**..**C**.**G****G****G**.**A****G****U****C****C****G****G**UGA..........................................................................................................................................................................G**C****C****G****G****G****C****U**GAG**A**G**G****A****A****G****C**UUAU..............................................................................CCAA**G****C****U**.**U****C**...........**G****A****C****C****G****U**C-.........................GA**A**.**C****C****U****G**A.U.**C****U****G****G**.GUCAU...............................G.**C****C****A****G**C**G**.C**A****G****G**.**G**A....**G****G****C****U****A****C****U****C****G****C** | |
|  |  | NC\_002737.1/482956-482867  | **U****A****U****U****U****C****A****C****A****A****A**..**G**.**G****A****G**.**U****G****C****U**------..........................................................................................................................................................................--**U****U****G****G****C****U**GAG**A**.**U****C****G****C****A**AU--..................................................................................**U****G****C**.**G****A**...........**A****A****U****C****C****U**GA.........................GG**A**.**C****C****U****G**A.U.**C****U****U****G**.UUAGU...............................A.**C****A****A****G**C**G**.U**A****G****G**.**G**A....**U****U****G****U****G****A****C****C****A****A** | |
|  |  | NC\_003485.1/531498-531409  | **U****A****U****U****U****C****A****C****A****A****A**..**G**.**G****A****G**.**U****G****C****U**------..........................................................................................................................................................................--**U****U****G****G****C****U**GAG**A**.**U****C****G****C****A**AU--..................................................................................**U****G****C**.**G****A**...........**A****A****U****C****C****U**GA.........................GG**A**.**C****C****U****G**A.U.**C****U****U****G**.UUAGU...............................A.**C****A****A****G**C**G**.U**A****G****G**.**G**A....**U****U****G****U****G****A****C****C****A****A** | |
|  |  | NC\_004070.1/464992-464903  | **U****A****U****U****U****C****A****C****A****A****A**..**G**.**G****A****G**.**U****G****C****U**------..........................................................................................................................................................................--**U****U****G****G****C****U**GAG**A**.**U****C****G****C****A**AU--..................................................................................**U****G****C**.**G****A**...........**A****A****U****C****C****U**GA.........................GG**A**.**C****C****U****G**A.U.**C****U****U****G**.UUAGU...............................A.**C****A****A****G**C**G**.U**A****G****G**.**G**A....**U****U****G****U****G****A****C****C****A****A** | |
|  |  | NC\_004606.1/1431750-1431839  | **U****A****U****U****U****C****A****C****A****A****A**..**G**.**G****A****G**.**U****G****C****U**------..........................................................................................................................................................................--**U****U****G****G****C****U**GAG**A**.**U****C****G****C****A**AU--..................................................................................**U****G****C**.**G****A**...........**A****A****U****C****C****U**GA.........................GG**A**.**C****C****U****G**A.U.**C****U****U****G**.UUAGU...............................A.**C****A****A****G**C**G**.U**A****G****G**.**G**A....**U****U****G****U****G****A****C****C****A****A** | |
|  |  | NC\_006086.1/510790-510701  | **U****A****U****U****U****C****A****C****A****A****A**..**G**.**G****A****G**.**U****G****C****U**------..........................................................................................................................................................................--**U****U****G****G****C****U**GAG**A**.**U****C****G****C****A**AU--..................................................................................**U****G****C**.**G****A**...........**A****A****U****C****C****U**GA.........................GG**A**.**C****C****U****G**A.U.**C****U****U****G**.UUAGU...............................A.**C****A****A****G**C**G**.U**A****G****G**.**G**A....**U****U****G****U****G****A****C****C****A****A** | |
|  |  | NZ\_AAFV01000105.1/879-968  | **U****A****U****U****U****C****A****C****A****A****A**..**G**.**G****A****G**.**U****G****C****U**------..........................................................................................................................................................................--**U****U****G****G****C****U**GAG**A**.**U****C****G****C****A**AU--..................................................................................**U****G****C**.**G****A**...........**A****A****U****C****C****U**GA.........................GG**A**.**C****C****U****G**A.U.**C****U****U****G**.UUAGU...............................A.**C****A****A****G**C**G**.U**A****G****G**.**G**A....**U****U****G****U****G****A****C****C****A****A** | |
|  |  | NC\_004113.1/322901-322992  | **A****C****G****A****C****A****U****G****C****U****A**..**G**.**G****G****G**.**U****G****U****C****U****G****C**GGUCAC.......................................................................................................................................................................A**G****C****A****G****G****C****U**GAG**A**.---------..................................................................................---.--........GCA**A****A****C****C****C****U**UA.........................GA**A**.**C****C****U****G**A.A.**C****C****A****G**.AUCAU...............................G.**C****U****G****G**C**G**.C**A****G****G**.**G**A....**A****G****C****U****G****U****C****U****A****G** | |
|  |  | NC\_005085.1/3448483-3448396  | **C****C****C****U****G****U****C****A****C****C****G**..**G**.**G****G****G**.**U****G****C****C****C****G****C**CA-..........................................................................................................................................................................U**G****C****G****G****G****C****U**GAG**A**.---------..................................................................................---.--.........GA**C****A****C****C****C****U**GA.........................GA**A**.**C****C****U****G**A.U.**C****U****G****G**.AUCAU...............................G.**C****C****A****G**C**G**.G**A****G****G**.**G**A...G**C****G****U****G****A****U****G****A****A****G** | |
|  |  | NC\_003030.1/3156266-3156125  | **U****A****U****U****U****U****A****G****C****U****A**..**G**.**G****G****G**.**U****G****C****C****U****U****U**UAAGGCUUUUAUAGUUGAUAUCAUUAAAAAUAUUUAACUAAUAAAAGAC............................................................................................................................U**U****U****A****G****G****C****U**GAG**A**.**G****G****A****G****A**AA--..................................................................................--**U**.**C****C**...........**A****A****C****C****C****U**UU.........................GA**A**.**C****U****U****G**A.U.**G****U****A****G**.UUAAU...............................A.**C****U****A****C**C**G**.U**A****G****G**.**G**A....**A****G****C****A****G****U****G****C****A****U** | |
|  |  | NC\_000913.2/2183454-2183365  | **C****C****A****A****A****C****G****A****C****U****C**..**G**.**G****G****G**.**U****G****C****C****C****U****U**CUGCG........................................................................................................................................................................U**G****A****A****G****G****C****U**GAG**A**.---------..................................................................................---.--.........AA**U****A****C****C****C****G**UA.........................UC**A**.**C****C****U****G**A.U.**C****U****G****G**.AUAAU...............................G.**C****C****A****G**C**G**.U**A****G****G**.**G**A....**A****G****U****C****A****C****G****G****A****C** | |
|  |  | NC\_002655.2/2929184-2929095  | **C****C****A****A****A****C****G****A****C****U****C**..**G**.**G****G****G**.**U****G****C****C****C****U****U**CUGCG........................................................................................................................................................................U**G****A****A****G****G****C****U**GAG**A**.---------..................................................................................---.--.........AA**U****A****C****C****C****G**UA.........................UC**A**.**C****C****U****G**A.U.**C****U****G****G**.AUAAU...............................G.**C****C****A****G**C**G**.U**A****G****G**.**G**A....**A****G****U****C****A****C****G****G****A****C** | |
|  |  | NC\_002695.1/2858965-2858876  | **C****C****A****A****A****C****G****A****C****U****C**..**G**.**G****G****G**.**U****G****C****C****C****U****U**CUGCG........................................................................................................................................................................U**G****A****A****G****G****C****U**GAG**A**.---------..................................................................................---.--.........AA**U****A****C****C****C****G**UA.........................UC**A**.**C****C****U****G**A.U.**C****U****G****G**.AUAAU...............................G.**C****C****A****G**C**G**.U**A****G****G**.**G**A....**A****G****U****C****A****C****G****G****A****C** | |
|  |  | NC\_004337.1/2190955-2190866  | **C****C****A****A****A****C****G****A****C****U****C**..**G**.**G****G****G**.**U****G****C****C****C****U****U**CUGCG........................................................................................................................................................................U**G****A****A****G****G****C****U**GAG**A**.---------..................................................................................---.--.........AA**U****A****C****C****C****G**UA.........................UC**A**.**C****C****U****G**A.U.**C****U****G****G**.AUAAU...............................G.**C****C****A****G**C**G**.U**A****G****G**.**G**A....**A****G****U****C****A****C****G****G****A****C** | |
|  |  | NC\_004741.1/2172479-2172390  | **C****C****A****A****A****C****G****A****C****U****C**..**G**.**G****G****G**.**U****G****C****C****C****U****U**CUGCG........................................................................................................................................................................U**G****A****A****G****G****C****U**GAG**A**.---------..................................................................................---.--.........AA**U****A****C****C****C****G**UA.........................UC**A**.**C****C****U****G**A.U.**C****U****G****G**.AUAAU...............................G.**C****C****A****G**C**G**.U**A****G****G**.**G**A....**A****G****U****C****A****C****G****G****A****C** | |
|  |  | NZ\_AABF02000046.1/7226-7130  | **A****U****A****U****A****U****G****U****A****C****U**..**G**.**G****G****G**.**A****G****C****U****A**-----..........................................................................................................................................................................--**U****A****U****G****C****U**GAG**A**.**U****U****A****G****A**ACCU..............................................................................UUUU**U****C****U**.**U****A**...........**G****A****C****C****C****A**UA.........................GU**A**.**C****C****U****G**A.U.**U****U****G****G**.AUAAU...............................G.**C****C****A****A**C**G**.A**A****G****G**.**G**A....**G****U****A****C****C****A****C****C****U****U** | |
|  |  | NC\_004605.1/128021-127921  | **G****C****G****A****A****A****U****A****G****U****C**..**G**.**G****G****G**.**G****G****C****C****A****C****G**UGAUAAG......................................................................................................................................................................C**G****U****U****G****G****C****U**GAG**A**.**U****C****G****A****A**A---..................................................................................**U****U****C**.**G****A**...........**G****A****C****C****C****G**UU.........................GA**A**.**C****C****U****G**A.U.**U****C****A****G**.UUAGC...............................A.**C****U****G****A**C**G**.U**A****G****G**.**G**A....**A****C****U****A****U****G****C****G****C****G** | |
|  |  | NC\_004445.1/16666-16554  | **A****G****U****C****C****C****U****G****U****C****A**..**G**.**G****G****G**.**A****G****U****C****U****C****G**CCA..........................................................................................................................................................................G**A****G****A****G****A****C****U**GAG**A**G**G****C****U****A****A**UAGC......................................................................GAUUUUCGCGGC**U****U****A**.**G****C**...........**G****A****C****C****C****U**UA.........................GA**A**.**C****C****U****G**A.C.**C****C****A****G**.CUGAU...............................A.**C****U****G****G**C**G**.U**A****G****G**.**A**A....**G****A****C****U****C****G****U****A****U****C** | |
|  |  | NC\_004834.1/16639-16527  | **A****G****U****C****C****C****U****G****U****C****A**..**G**.**G****G****G**.**A****G****U****C****U****C****G**CCA..........................................................................................................................................................................G**A****G****A****G****A****C****U**GAG**A**G**G****C****U****A****A**UAGC......................................................................GAUUUUCGCGGC**U****U****A**.**G****C**...........**G****A****C****C****C****U**UA.........................GA**A**.**C****C****U****G**A.C.**C****C****A****G**.CUGAU...............................A.**C****U****G****G**C**G**.U**A****G****G**.**A**A....**G****A****C****U****C****G****U****A****U****C** | |
|  |  | NZ\_AAGP01000030.1/25584-25483  | **U****U****C****U****C****A****C****G****A****C****A**..**G**.**G****G****G**.**A****G****C****G****C****C****G**AUA..........................................................................................................................................................................G**G****G****G****C****G****C****U**GAG**A**G**U****G****C****A****G**AUGA................................................................................AG**C****U****G**.**C****A**...........**G****A****C****C****C****U**C-.........................GA**A**.**C****C****U****G**A.U.**G****C****G****G**.CUAGC...............................A.**C****C****G****C**C**G**.A**A****G****G**.**A**A....**G****U****C****G****A****G****A****C****U****C** | |
|  |  | NZ\_AAGR01000021.1/43-133  | **A****C****C****U****U****C****A****C****A****A****A**..**G**.**G****G****G**.**A****G****C****C****A**-----..........................................................................................................................................................................--**U****U****G****G****C****U**GAG**A**A**C****G****G****G****G**AAA-..................................................................................-**C****C**.**C****G**...........**G****A****C****C****C****U**UC.........................GA**A**.**C****C****U****G**-.U.-**U****C****G**.UUAAU...............................G.**C****G****A****G**C**G**.U**A****G****G**.**G**A...U**U****U****G****U****G****A****A****U****G****G** | |
|  |  | NC\_003028.1/684648-684729  | **U****A****A****A****G****A****C****A****U****U****U**..**G**.**G****G****G**.**U****G****C****U****U**-----..........................................................................................................................................................................--**U****A****G****G****C****U**GAG**A**.---------..................................................................................---.--........UGA**U****A****C****C****C****A**UU.........................GA**A**.**C****C****U****G**A.U.**A****C****A****G**.UUAAG...............................A.**C****U****G****G**C**G**.A**A****G****G**.**G**A....**A****A****U****G****U****G****A****A****A****U** | |
|  |  | NC\_003098.1/639235-639316  | **U****A****A****A****G****A****C****A****U****U****U**..**G**.**G****G****G**.**U****G****C****U****U**-----..........................................................................................................................................................................--**U****A****G****G****C****U**GAG**A**.---------..................................................................................---.--........UGA**U****A****C****C****C****A**UU.........................GA**A**.**C****C****U****G**A.U.**A****C****A****G**.UUAAG...............................A.**C****U****G****G**C**G**.A**A****G****G**.**G**A....**A****A****U****G****U****G****A****A****A****U** | |
|  |  | NZ\_AAGY01000076.1/7650-7569  | **U****A****A****A****G****A****C****A****U****U****U**..**G**.**G****G****G**.**U****G****C****U****U**-----..........................................................................................................................................................................--**U****A****G****G****C****U**GAG**A**.---------..................................................................................---.--........UGA**U****A****C****C****C****A**UU.........................GA**A**.**C****C****U****G**A.U.**A****C****A****G**.UUAAG...............................A.**C****U****G****G**C**G**.A**A****G****G**.**G**A....**A****A****U****G****U****G****A****A****A****U** | |
|  |  | NZ\_AABO02000004.1/138142-138225  | **C****C****G****A****C****U****U****A****G****U****C**..**G**.**G****G****G**.**U****G****C****U****G****A****U**---..........................................................................................................................................................................-**A****A****C****A****G****C****U**GAG**A**.---------..................................................................................---.--........UAA**U****A****C****C****C****G**U-.........................GA**A**.**C****C****U****G**A.U.**A****C****A****G**.UUAAU...............................A.**C****U****G****A**C**G**.U**A****G****G**.**A**A....**A****C****U****A****A****U****G****G****U****C** | |
|  |  | NZ\_AACJ01000007.1/47296-47213  | **C****C****G****A****C****U****U****A****G****U****C**..**G**.**G****G****G**.**U****G****C****U****G****A****U**---..........................................................................................................................................................................-**A****A****C****A****G****C****U**GAG**A**.---------..................................................................................---.--........UAA**U****A****C****C****C****G**U-.........................GA**A**.**C****C****U****G**A.U.**A****C****A****G**.UUAAU...............................A.**C****U****G****A**C**G**.U**A****G****G**.**A**A....**A****C****U****A****A****U****G****G****U****C** | |
|  |  | NC\_002946.2/1981304-1981207  | **G****C****C****U****U****C****U****U****G****U****C**..**G**.**G****A****G**.**U****G****C****C****G****C****C**CGCC.........................................................................................................................................................................G**G****G****C****G****G****C****U**GAG**A**.**U****U****G****C****G**AAA-..................................................................................-**G****C**.**A****A**...........**A****A****U****C****C****G**UA.........................GA**A**.**C****C****U****G**-.U.**C****G****G****G**.GUAAU...............................G.**C****C****U****G**C**G**.U**A****G****G**.**A**A....**A****C****A****A****A****A****C****C****U****C** | |
|  |  | NC\_006905.1/120814-120722  | **C****C****G****A****A****C****U****C****A****A****C**..**G**.**G****G****G**.**U****G****C**-**C****G****C**GCUCAUG......................................................................................................................................................................C**G****C****G****C****G****C****U**GAG**A**.---------..................................................................................---.--........AAA**U****A****C****C****C****G**UC.........................GA**A**.**C****C****U****G**A.U.**C****C****G****G**.AUAAU...............................G.**C****C****G****G**C**G**.A**A****G****G**.**G**A...U**U****U****G****A****G****G****U****U****A****C** | |
|  |  | NC\_005966.1/278008-277886  | **G****C****A****U****G****C****U****U****G****A****C**..**G**.**G****A****G**.**C****G****C****G****A****G****U**AAC..........................................................................................................................................................................A**A****C****U****C****G****C****U**GAG**A**.**U****U****G****G****G**UAAA...........................................................UCUGUUUUUUGAACUGAUUUGAU**C****C****C**.**A****A**...........**G****U****A****C****C****G**UU.........................GA**A**.**C****C****U****G**A.U.**C****A****G****G**.UUAAU...............................A.**C****C****U****G**C**G**.U**A****G****G**.**A**A....**U****C****A****A****G****U****C****A****U****C** | |
|  |  | NZ\_AAFA01000059.1/1464-1552  | **A****U****A****U****A****C****A****C****A****A****A**..**G**.**G****A****G**.**U****G****C****U**------..........................................................................................................................................................................--**U****C****G****G****C****U**GAG**A**.**U****C****G****C****A**AC--..................................................................................**U****G****C**.**G****A**...........**A****A****U****C****C****U**C-.........................GA**A**.**C****C****U****G**A.U.**C****U****A****G**.UUAAA...............................A.**C****U****A****G**C**G**.U**A****G****G**.**A**A....**U****U****G****U****G****U****G****C****U****U** | |
|  |  | NC\_005126.1/705796-705699  | **G****C****C****A****U****C****U****C****A****A****C**..**G**.**G****G****G**.**U****G****C****U****G****G****C**UGUUUAAAACA..................................................................................................................................................................G**C****C****U****U****G****C****U**GAG**A**.---------..................................................................................---.--........GUA**A****A****C****C****C****G**CC.........................GA**A**.**C****C****U****G**A.U.**C****C****G****G**.CUAAU...............................A.**C****C****G****G**C**G**.U**A****G****G**.**G**A...U**U****U****G****A****G****C****U****G****C****U** | |
|  |  | NC\_004547.2/3578493-3578404  | **G****U****A****C****A****U****G****A****C****U****C**..**G**.**G****G****G**.**U****G****C****C****C****U****U**CUUUG........................................................................................................................................................................U**G****A****A****G****G****C****U**GAG**A**.---------..................................................................................---.--.........AA**U****A****C****C****C****G**UA.........................CC**A**.**C****C****U****G**A.U.**C****U****G****G**.AUAAU...............................G.**C****C****A****G**C**G**.U**A****G****G**.**G**A....**A****G****U****C****A****C****G****G****U****A** | |
|  |  | NZ\_AAEH02000055.1/48245-48360  | **A****C****G****A****C****G****A****A****A****C****A**..**G**.**G****G****G**.**U****G****C****U****U****C****G**AGUGCGGCCAUGGGUAGUUCCGGGCAACGCU..............................................................................................................................................G**C****G****A****G****G****C****U**GAG**A**.---------..................................................................................---.--.........AA**G****A****C****C****C****U**UC.........................GC**A**.**C****C****C****G**A.U.**C****C****G****G**.GUAAU...............................A.**C****C****G****G**C**G**.A**G****G****G**.**A**A....**G****U****U****U****C****U****G****A****U****C** | |
|  |  | NC\_002516.1/5583870-5583741  | **G****G****G****U****U****C****U****U****G****U****C**..**G**.**G****G****G**.**U****G****C****C****C****U****A**UAC..........................................................................................................................................................................G**A****G****G****G****G****C****U**GAG**A**.**U****C****G****G****A**UAGU..................................................................................**U****C****C**.**G****G**...........**A****U****C****C****C****G**UU.........................GA**A**.**C****C****U****G**A.U.**C****G****G****G**.CUAGC.GUCCGGUUCCGCCUCGCGCGGACCGCAGCAA.**C****C****C****G**C**G**.U**A****G****G**.**G**A....**A****C****A****A****G****A****U****G****U****C** | |
|  |  | NZ\_AABQ07000004.1/314259-314130  | **G****G****G****U****U****C****U****U****G****U****C**..**G**.**G****G****G**.**U****G****C****C****C****U****A**UAC..........................................................................................................................................................................G**A****G****G****G****G****C****U**GAG**A**.**U****C****G****G****A**UAGU..................................................................................**U****C****C**.**G****G**...........**A****U****C****C****C****G**UU.........................GA**A**.**C****C****U****G**A.U.**C****G****G****G**.CUAGC.GUCCGGUUCCGCCUCGCGCGGACCGCAGCAA.**C****C****C****G**C**G**.U**A****G****G**.**G**A....**A****C****A****A****G****A****U****G****U****C** | |
|  |  | NC\_003047.1/3532865-3532971  | **G****C****U****G****C****U****C****U****A****A****C**..**G**.**G****G****G**.**U****G****C****C****C****U****G**GCCGGCUUUGCGA................................................................................................................................................................C**C****A****U****G****G****C****U**GAG**A**.**G****G****C****U****U**CG--..................................................................................-**A****G**.**C****C**...........**A****A****C****C****C****G**CG.........................GA**A**.**C****C****U****G**A.U.**C****C****G****G**.CUCAU...............................A.**C****C****G****G**C**G**.G**A****G****G**.**G**A....**U****U****A****G****A****A****G****C****G****A** | |
|  |  | NC\_004722.1/413885-413980  | **A****A****U****U****A****A****G****G****A****C****C**..**G**.**G****G****G**.**A****G****C****C****A****A**----..........................................................................................................................................................................--**U****U****G****G****C****U**GAG**A**G**G****A****U****G****U**AAGU................................................................................AA**A****C****A**.**U****C**...........**G****A****C****C****C****U**C-.........................-A**A**.**C****C****U****G**A.U.**C****U****G****G**.AUAAU...............................G.**C****C****A****G**C**G**.U**A****G****G**.**G**A...G**U****U****A****C****U****U****A****A****A****G** | |
|  |  | NZ\_AAHI01000026.1/52217-52333  | **A****C****G****A****C****G****A****A****A****C****A**..**G**.**G****G****G**.**U****G****C****U****U****C****G**UGGCGCGGCCGGCAGAUGUUCCGGGCAGCGCG.............................................................................................................................................G**C****G****A****G****G****C****U**GAG**A**.---------..................................................................................---.--.........AA**G****A****C****C****C****U**UC.........................GC**A**.**C****C****C****G**A.U.**C****C****G****G**.GUAAU...............................A.**C****C****G****G**C**G**.A**G****G****G**.**A**A....**G****U****U****U****C****U****G****A****U****C** | |
|  |  | NZ\_AAHL01000010.1/79414-79298  | **A****C****G****A****C****G****A****A****A****C****A**..**G**.**G****G****G**.**U****G****C****U****U****C****G**UGGCGCGGCCGGCAGAUGUUCCGGGCAGCGCG.............................................................................................................................................G**C****G****A****G****G****C****U**GAG**A**.---------..................................................................................---.--.........AA**G****A****C****C****C****U**UC.........................GC**A**.**C****C****C****G**A.U.**C****C****G****G**.GUAAU...............................A.**C****C****G****G**C**G**.A**G****G****G**.**A**A....**G****U****U****U****C****U****G****A****U****C** | |
|  |  | NC\_005042.1/1627739-1627644  | **A****A****A****U****A****U****C****A****C****U****A**..**G**.**G****G****G**.**U****G****C****C****U****A****C**AAGCUAUUGC...................................................................................................................................................................U**U****U****U****G****G****C****U**GAG**A**.---------..................................................................................---.--........UCA**C****A****C****C****C****U**CU.........................GA**A**.**C****C****U****G**A.U.**U****C****G****G**.UUUAU...............................A.**C****C****G****U**C**G**.A**A****G****G**.**A**A....**A****G****U****G****A****A****A****G****A****G** | |
|  |  | NZ\_AADX02000003.1/7710-7806  | **G****A****C****C****U****A****A****C****C****U****A**..**G**.**G****G****G**.**A****G****C****C****G****C****U**---..........................................................................................................................................................................-**U****G****C****G****G****C****U**GAG**A**A**A****U****G****A****C**AACC..................................................................................**G****U****C**.**A****U**...........**G****A****C****C****C****U**UA.........................AU**A**.**C****U****U****G**A.U.**C****C****A****G**.ACCAU...............................G.**C****U****G****G**C**G**.A**A****A****G**.**G**A....**A****G****G****A****A****A****C****A****A****C** | |
|  |  | NC\_002663.1/1459568-1459472  | **G****C****A****C****U****U****U****A****G****U****C**..**G**.**G****G****G**.**U****G****C****U****U****U****G**UAACCAAAUUGU.................................................................................................................................................................A**C****A****A****A****G****C****U**GAG**A**.---------..................................................................................---.--........UGA**U****A****C****C****C****G**U-.........................GA**A**.**C****C****U****G**A.U.**A****C****A****G**.CUAAC...............................A.**C****U****G****A**C**G**.U**A****G****G**.**A**A....**A****C****U****A****A****U****U****A****U****G** | |
|  |  | NC\_003450.3/741181-741081  | **A****C****U****A****G****U****G****A****C****A****C**..**G**.**G****G****G**.**U****G****C****A****A****A****A**GCACUUUAAAAAAG...............................................................................................................................................................C**U****U****U****C****G****C****U**GAG**A**.---------..................................................................................---.--........UUA**C****A****C****C****C****G**UC.........................GA**A**.**C****C****U****G**A.U.**C****C****A****G**.UUAGU...............................A.**C****U****G****G**C**G**.A**A****G****G**.**G**A...C**U****G****U****C****G****C****A****U****U****G** | |
|  |  | NC\_006958.1/742654-742554  | **A****C****U****A****G****U****G****A****C****A****C**..**G**.**G****G****G**.**U****G****C****A****A****A****A**GCACUUUAAAAAAG...............................................................................................................................................................C**U****U****U****C****G****C****U**GAG**A**.---------..................................................................................---.--........UUA**C****A****C****C****C****G**UC.........................GA**A**.**C****C****U****G**A.U.**C****C****A****G**.UUAGU...............................A.**C****U****G****G**C**G**.A**A****G****G**.**G**A...C**U****G****U****C****G****C****A****U****U****G** | |
|  |  | NC\_002570.2/2798924-2799023  | **G****G****A****A****U****C****C****A****C****U****A**..**G**.**G****G****G**.**U****G****C****A****A**-----..........................................................................................................................................................................--**A****C****C****G****C****U**GAG**A**.**G****A****G****A****U**GUUU............................................................................UUUAGC**A****U****C**.**U****U**...........**A****A****C****C****C****U**CAU........................UC**A**.**C****C****U****G**A.U.**C****U****A****G**.GUAAU...............................A.**C****U****A****G**C**G**.A**A****G****G**.**G**A....**A****G****U****G****G****C****C****A****U****C** | |
|  |  | NC\_003909.8/506312-506407  | **A****A****U****U****A****A****G****G****A****C****C**..**G**.**G****G****G**.**A****G****C****C****A****A**----..........................................................................................................................................................................--**U****U****G****G****C****U**GAG**A**G**G****A****U****G****U**GAGU................................................................................AA**A****C****A**.**U****C**...........**G****A****C****C****C****U**C-.........................-A**A**.**C****C****U****G**A.U.**C****U****G****G**.AUAAU...............................G.**C****C****A****G**C**G**.U**A****G****G**.**G**A...G**U****U****A****C****U****U****A****A****A****G** | |
|  |  | NZ\_AAEK01000015.1/88953-88858  | **A****A****U****U****A****A****G****G****A****C****C**..**G**.**G****G****G**.**A****G****C****C****A****A**----..........................................................................................................................................................................--**U****U****G****G****C****U**GAG**A**G**G****A****U****G****U**AAAC................................................................................AA**A****C****A**.**U****C**...........**G****A****C****C****C****U**C-.........................-A**A**.**C****C****U****G**A.U.**C****U****G****G**.AUAAU...............................G.**C****C****A****G**C**G**.U**A****G****G**.**G**A...G**U****U****A****C****U****U****A****A****A****G** | |
|  |  | NZ\_AAHG01000017.1/15030-14894  | **U****U****G****A****U****A****C****G****A****C****A**..**G**.**G****G****G**.**A****G****C****G****U****C****G**CCGUCGGGCAUCACUGGAACAUUUCCAGGGAACCGGAGG......................................................................................................................................C**G****G****G****C****G****C****U**GAG**A**G**U****G****C****G****G**ACAG..................................................................................**C****C****G**.**C****A**...........**G****A****C****C****C****U**C-.........................GA**A**.**C****C****U****G**A.U.**C****C****G****G**.UUAGU...............................A.**C****C****G****G**C**G**CA**A****G****G**.**G**A....**G****U****C****G****A****G****U****U****C****U** | |
|  |  | NC\_002578.1/470047-469948  | **C****U****G****G****U****G****U****G****G****U****G**..**G**.**G****G****G**.**A****G****C****U****C****C****A**UA-..........................................................................................................................................................................-**A****G****G****G****G****C****U**GAG**A**G**G****A****U****C****C**GGAU..................................................................................**G****G****A**.**U****C**...........**G****A****U****C****C****C**UG.........................GA**A**.**C****C****U****G**A.U.**C****C****G****G**.GUAAU...............................A.**C****C****G****G**C**G**.G**A****G****G**.**G**A...A**A****U****U****A****U****G****G****A****A****A** | |
|  |  | NC\_002977.5/2895493-2895583  | **C****C****G****U****A****A****G****G****C****U****G**..**G**.**G****G****G**.**U****G****C****C****U****G****G**CGAAUG.......................................................................................................................................................................G**C****C****G****G****G****C****U**GAG**A**.---------..................................................................................---.--.........GA**C****A****C****C****C****U**UC.........................GA**A**.**C****C****U****G**A.U.**C****C****G****G**.CUGAU...............................A.**C****C****G****G**C**G**.U**A****G****G**.**G**A....**A****G****C****U****G****A****G****C****U****U** | |
|  |  | NC\_003997.3/399319-399414  | **A****A****U****U****A****A****G****G****A****C****C**..**G**.**G****G****G**.**A****G****C****C****A****A**----..........................................................................................................................................................................--**U****U****G****G****C****U**GAG**A**G**G****A****U****G****U**GAGC................................................................................AA**A****C****A**.**U****C**...........**G****A****C****C****C****U**C-.........................-A**A**.**C****C****U****G**A.U.**C****U****G****G**.AUAAU...............................G.**C****C****A****G**C**G**.U**A****G****G**.**G**A...G**U****U****A****C****U****U****A****A****A****G** | |
|  |  | NC\_005945.1/399332-399427  | **A****A****U****U****A****A****G****G****A****C****C**..**G**.**G****G****G**.**A****G****C****C****A****A**----..........................................................................................................................................................................--**U****U****G****G****C****U**GAG**A**G**G****A****U****G****U**GAGC................................................................................AA**A****C****A**.**U****C**...........**G****A****C****C****C****U**C-.........................-A**A**.**C****C****U****G**A.U.**C****U****G****G**.AUAAU...............................G.**C****C****A****G**C**G**.U**A****G****G**.**G**A...G**U****U****A****C****U****U****A****A****A****G** | |
|  |  | NC\_005957.1/423300-423395  | **A****A****U****U****A****A****G****G****A****C****C**..**G**.**G****G****G**.**A****G****C****C****A****A**----..........................................................................................................................................................................--**U****U****G****G****C****U**GAG**A**G**G****A****U****G****U**GAGC................................................................................AA**A****C****A**.**U****C**...........**G****A****C****C****C****U**C-.........................-A**A**.**C****C****U****G**A.U.**C****U****G****G**.AUAAU...............................G.**C****C****A****G**C**G**.U**A****G****G**.**G**A...G**U****U****A****C****U****U****A****A****A****G** | |
|  |  | NC\_006274.1/416692-416787  | **A****A****U****U****A****A****G****G****A****C****C**..**G**.**G****G****G**.**A****G****C****C****A****A**----..........................................................................................................................................................................--**U****U****G****G****C****U**GAG**A**G**G****A****U****G****U**GAGC................................................................................AA**A****C****A**.**U****C**...........**G****A****C****C****C****U**C-.........................-A**A**.**C****C****U****G**A.U.**C****U****G****G**.AUAAU...............................G.**C****C****A****G**C**G**.U**A****G****G**.**G**A...G**U****U****A****C****U****U****A****A****A****G** | |
|  |  | NC\_007530.2/399319-399414  | **A****A****U****U****A****A****G****G****A****C****C**..**G**.**G****G****G**.**A****G****C****C****A****A**----..........................................................................................................................................................................--**U****U****G****G****C****U**GAG**A**G**G****A****U****G****U**GAGC................................................................................AA**A****C****A**.**U****C**...........**G****A****C****C****C****U**C-.........................-A**A**.**C****C****U****G**A.U.**C****U****G****G**.AUAAU...............................G.**C****C****A****G**C**G**.U**A****G****G**.**G**A...G**U****U****A****C****U****U****A****A****A****G** | |
|  |  | NZ\_AAAC02000001.1/916207-916302  | **A****A****U****U****A****A****G****G****A****C****C**..**G**.**G****G****G**.**A****G****C****C****A****A**----..........................................................................................................................................................................--**U****U****G****G****C****U**GAG**A**G**G****A****U****G****U**GAGC................................................................................AA**A****C****A**.**U****C**...........**G****A****C****C****C****U**C-.........................-A**A**.**C****C****U****G**A.U.**C****U****G****G**.AUAAU...............................G.**C****C****A****G**C**G**.U**A****G****G**.**G**A...G**U****U****A****C****U****U****A****A****A****G** | |
|  |  | NZ\_AAEN01000023.1/140442-140537  | **A****A****U****U****A****A****G****G****A****C****C**..**G**.**G****G****G**.**A****G****C****C****A****A**----..........................................................................................................................................................................--**U****U****G****G****C****U**GAG**A**G**G****A****U****G****U**GAGC................................................................................AA**A****C****A**.**U****C**...........**G****A****C****C****C****U**C-.........................-A**A**.**C****C****U****G**A.U.**C****U****G****G**.AUAAU...............................G.**C****C****A****G**C**G**.U**A****G****G**.**G**A...G**U****U****A****C****U****U****A****A****A****G** | |
|  |  | NZ\_AAEO01000030.1/153881-153976  | **A****A****U****U****A****A****G****G****A****C****C**..**G**.**G****G****G**.**A****G****C****C****A****A**----..........................................................................................................................................................................--**U****U****G****G****C****U**GAG**A**G**G****A****U****G****U**GAGC................................................................................AA**A****C****A**.**U****C**...........**G****A****C****C****C****U**C-.........................-A**A**.**C****C****U****G**A.U.**C****U****G****G**.AUAAU...............................G.**C****C****A****G**C**G**.U**A****G****G**.**G**A...G**U****U****A****C****U****U****A****A****A****G** | |
|  |  | NZ\_AAEP01000046.1/105771-105866  | **A****A****U****U****A****A****G****G****A****C****C**..**G**.**G****G****G**.**A****G****C****C****A****A**----..........................................................................................................................................................................--**U****U****G****G****C****U**GAG**A**G**G****A****U****G****U**GAGC................................................................................AA**A****C****A**.**U****C**...........**G****A****C****C****C****U**C-.........................-A**A**.**C****C****U****G**A.U.**C****U****G****G**.AUAAU...............................G.**C****C****A****G**C**G**.U**A****G****G**.**G**A...G**U****U****A****C****U****U****A****A****A****G** | |
|  |  | NZ\_AAEQ01000043.1/88646-88551  | **A****A****U****U****A****A****G****G****A****C****C**..**G**.**G****G****G**.**A****G****C****C****A****A**----..........................................................................................................................................................................--**U****U****G****G****C****U**GAG**A**G**G****A****U****G****U**GAGC................................................................................AA**A****C****A**.**U****C**...........**G****A****C****C****C****U**C-.........................-A**A**.**C****C****U****G**A.U.**C****U****G****G**.AUAAU...............................G.**C****C****A****G**C**G**.U**A****G****G**.**G**A...G**U****U****A****C****U****U****A****A****A****G** | |
|  |  | NZ\_AAER01000042.1/134375-134280  | **A****A****U****U****A****A****G****G****A****C****C**..**G**.**G****G****G**.**A****G****C****C****A****A**----..........................................................................................................................................................................--**U****U****G****G****C****U**GAG**A**G**G****A****U****G****U**GAGC................................................................................AA**A****C****A**.**U****C**...........**G****A****C****C****C****U**C-.........................-A**A**.**C****C****U****G**A.U.**C****U****G****G**.AUAAU...............................G.**C****C****A****G**C**G**.U**A****G****G**.**G**A...G**U****U****A****C****U****U****A****A****A****G** | |
|  |  | NZ\_AAES01000043.1/105441-105536  | **A****A****U****U****A****A****G****G****A****C****C**..**G**.**G****G****G**.**A****G****C****C****A****A**----..........................................................................................................................................................................--**U****U****G****G****C****U**GAG**A**G**G****A****U****G****U**GAGC................................................................................AA**A****C****A**.**U****C**...........**G****A****C****C****C****U**C-.........................-A**A**.**C****C****U****G**A.U.**C****U****G****G**.AUAAU...............................G.**C****C****A****G**C**G**.U**A****G****G**.**G**A...G**U****U****A****C****U****U****A****A****A****G** | |
|  |  | NZ\_AABJ03000005.1/70020-69938  | **C****A****U****C****A****A****A****A****U****C****U**..**G**.**G****G****G**.**U****G****C****C****C****A**----..........................................................................................................................................................................--**A****G****G****G****C****U**GAG**A**.---------..................................................................................---.--........CUA**U****A****C****C****C****A**CA.........................UA**A**.**C****C****U****G**A.U.**C****U****A****G**.UUCGU...............................A.**C****U****A****G**C**G**.U**A****G****G**.**G**A....**G****A****U****U****A****C****U****U****G****A** | |
|  |  | NC\_002677.1/377385-377489  | **A****A****A****A****A****A****C****C****A****C****G**..**C**.**G****G****G**.**A****G****C****A****C****A****C**ACCA.........................................................................................................................................................................A**G****U****G****C****G****C****U**GAG**A**G**G****A****C****G****G**AUCG...............................................................................GGG**C****C****G**.**U****C**...........**G****A****C****C****G****U**AU.........................GA**A**.**C****C****U****G**A.-.**C****C****G****G**.GUAAU...............................G.**C****C****G****G**C**G**.U**A****G****G**.**G**A...G**A****U****G****A****A****U****A****A****U****G** | |
|  |  | NZ\_AABJ03000002.1/60299-60213  | **U****A****C****U****U****C****U****A****U****C****U**..**G**.**G****G****G**.**U****G****C****C****U****U****U**ACUU.........................................................................................................................................................................U**U****A****A****G****G****C****U**GAG**A**.---------..................................................................................---.--.........UU**U****A****C****C****C****A**UC.........................GA**A**.**C****C****U****G**-.U.-**A****G****G**.UUAAU...............................A.**C****C****U****G**C**G**.U**A****G****G**.**G**A....**G****A****U****A****G****C****G****A****A****A** | |
|  |  | NC\_003197.1/126801-126709  | **C****C****G****A****A****C****U****C****A****A****C**..**G**.**G****G****G**.**U****G****C**-**C****G****C**GCUCAUG......................................................................................................................................................................C**G****C****G****C****G****C****U**GAG**A**.---------..................................................................................---.--........AAA**U****A****C****C****C****G**UC.........................GA**A**.**C****C****U****G**A.U.**C****C****G****G**.AUAAU...............................G.**C****C****G****G**C**G**.A**A****G****G**.**G**A...U**U****U****G****A****G****G****C****U****A****C** | |
|  |  | NC\_003198.1/128216-128124  | **C****C****G****A****A****C****U****C****A****A****C**..**G**.**G****G****G**.**U****G****C**-**C****G****C**GCUCAUG......................................................................................................................................................................C**G****C****G****C****G****C****U**GAG**A**.---------..................................................................................---.--........AAA**U****A****C****C****C****G**UC.........................GA**A**.**C****C****U****G**A.U.**C****C****G****G**.AUAAU...............................G.**C****C****G****G**C**G**.A**A****G****G**.**G**A...U**U****U****G****A****G****G****C****U****A****C** | |
|  |  | NC\_004631.1/128207-128115  | **C****C****G****A****A****C****U****C****A****A****C**..**G**.**G****G****G**.**U****G****C**-**C****G****C**GCUCAUG......................................................................................................................................................................C**G****C****G****C****G****C****U**GAG**A**.---------..................................................................................---.--........AAA**U****A****C****C****C****G**UC.........................GA**A**.**C****C****U****G**A.U.**C****C****G****G**.AUAAU...............................G.**C****C****G****G**C**G**.A**A****G****G**.**G**A...U**U****U****G****A****G****G****C****U****A****C** | |
|  |  | NC\_006511.1/128305-128213  | **C****C****G****A****A****C****U****C****A****A****C**..**G**.**G****G****G**.**U****G****C**-**C****G****C**GCUCAUG......................................................................................................................................................................C**G****C****G****C****G****C****U**GAG**A**.---------..................................................................................---.--........AAA**U****A****C****C****C****G**UC.........................GA**A**.**C****C****U****G**A.U.**C****C****G****G**.AUAAU...............................G.**C****C****G****G**C**G**.A**A****G****G**.**G**A...U**U****U****G****A****G****G****C****U****A****C** | |
|  |  | NC\_003143.1/3197151-3197054  | **C****A****U****U****C****U****G****A****C****U****C**..**G**.**G****G****G**.**U****G****C****C****U****G****C**CACAAUGCGGUG.................................................................................................................................................................G**U****A****A****G****G****C****U**GAG**A**.---------..................................................................................---.--........GAU**G****A****C****C****C****G**UA.........................UU**A**.**C****C****U****G**A.U.**C****U****G****G**.AUUAU...............................G.**C****C****A****G**C**G**.U**A****G****G**.**G**A....**A****G****U****C****U****C****G****G****C****A** | |
|  |  | NC\_004088.1/1522876-1522973  | **C****A****U****U****C****U****G****A****C****U****C**..**G**.**G****G****G**.**U****G****C****C****U****G****C**CACAAUGCGGUG.................................................................................................................................................................G**U****A****A****G****G****C****U**GAG**A**.---------..................................................................................---.--........GAU**G****A****C****C****C****G**UA.........................UU**A**.**C****C****U****G**A.U.**C****U****G****G**.AUUAU...............................G.**C****C****A****G**C**G**.U**A****G****G**.**G**A....**A****G****U****C****U****C****G****G****C****A** | |
|  |  | NC\_005810.1/3027837-3027740  | **C****A****U****U****C****U****G****A****C****U****C**..**G**.**G****G****G**.**U****G****C****C****U****G****C**CACAAUGCGGUG.................................................................................................................................................................G**U****A****A****G****G****C****U**GAG**A**.---------..................................................................................---.--........GAU**G****A****C****C****C****G**UA.........................UU**A**.**C****C****U****G**A.U.**C****U****G****G**.AUUAU...............................G.**C****C****A****G**C**G**.U**A****G****G**.**G**A....**A****G****U****C****U****C****G****G****C****A** | |
|  |  | NC\_006155.1/3340719-3340622  | **C****A****U****U****C****U****G****A****C****U****C**..**G**.**G****G****G**.**U****G****C****C****U****G****C**CACAAUGCGGUG.................................................................................................................................................................G**U****A****A****G****G****C****U**GAG**A**.---------..................................................................................---.--........GAU**G****A****C****C****C****G**UA.........................UU**A**.**C****C****U****G**A.U.**C****U****G****G**.AUUAU...............................G.**C****C****A****G**C**G**.U**A****G****G**.**G**A....**A****G****U****C****U****C****G****G****C****A** | |
|  |  | NZ\_AAHZ01000012.1/105220-105319  | **G****U****C****U****C****U****G****G****A****U****A**..**G**.**G****G****G**.**U****G****C****C****C****G****C**CGACCGU......................................................................................................................................................................G**G****C****G****G****G****C****U**GAG**A**.**G****C****G****C****C**C---..................................................................................**G****G****C**.**G****C**...........**A****A****C****C****C****U**G-.........................GA**A**.**C****C****U****G**A.U.**C****C****G****G**.UUAAC...............................A.**C****C****G****G**C**G**.G**A****G****G**.**A**A....**A****C****G****C****C****A****G****A****G****C** | |
|  |  | NC\_003112.1/2161704-2161612  | **A****C****A****U****U****G****A****A****A****C****A**..**G**.**G****G****G**.**U****G****C****U****G****C****C**UGAUGUUU.....................................................................................................................................................................A**G****G****C****G****G****C****U**GAG**A**.---------..................................................................................---.--.........AA**U****A****C****C****C****U**UU.........................AC**A**.**C****C****C****G**A.U.**C****G****G****G**.AUAAU...............................A.**C****C****U****G**C**G**.U**G****G****G**.**G**A....**G****U****U****U****U****C****A****C****G****G** | |
|  |  | NC\_004369.1/1474220-1474117  | **G****G****C****A****G****U****C****C****C****C****A**..**C**.**G****G****G**.**C****G****C****C****C****G****A**GC-..........................................................................................................................................................................-**A****C****G****G****G****C****U**GAG**A**.**U****C****G****C****G**CUGA............................................................................UUGCUG**C****G****C**.**G****A**..........G**C****A****C****C****G****U**UC.........................GA**A**.**C****C****U****G**-.U.**C****C****G****G**.UUAGU...............................A.**C****C****G****G**C**G**.A**A****G****G**.**A**A....**G****A****G****A****G****G****A****A****U****G** | |
|  |  | NZ\_AADW02000009.1/20239-20140  | **G****A****A****G****A****C****C****G****C****A****A**..**G**.**G****G****G**.**U****G****U****C****G****C****G**UC-..........................................................................................................................................................................A**A****G****C****G****G****C****U**GAG**A**.**G****A****A****G****U**GAUU................................................................................GG**A****C****U**.**U****U**...........**A****A****C****C****C****U**UC.........................GA**A**.**C****C****U****G**-.U.**U****C****C****G**.UUCAG...............................A.**C****G****G****A**C**G**.U**A****G****G**.**G**A....**U****G****U****G****G****C****G****C****A****C** | |
|  |  | NC\_002946.2/2011274-2011366  | **A****C****A****U****C****G****A****A****A****C****A**..**G**.**G****G****G**.**U****G****C****U****G****C****C**UGAUGUUC.....................................................................................................................................................................A**G****G****C****G****G****C****U**GAG**A**.---------..................................................................................---.--.........AA**U****A****C****C****C****U**UU.........................AC**A**.**C****C****C****G**A.U.**C****G****G****G**.AUAAU...............................A.**C****C****U****G**C**G**.U**G****G****G**.**G**A....**G****U****U****U****U****C****A****C****G****G** | |
|  |  | NC\_003116.1/365583-365675  | **A****C****A****U****C****G****A****A****A****C****A**..**G**.**G****G****G**.**U****G****C****U****G****C****C**UGAUGUUC.....................................................................................................................................................................A**G****G****C****G****G****C****U**GAG**A**.---------..................................................................................---.--.........AA**U****A****C****C****C****U**UU.........................AC**A**.**C****C****C****G**A.U.**C****G****G****G**.AUAAU...............................A.**C****C****U****G**C**G**.U**G****G****G**.**G**A....**G****U****U****U****U****C****A****C****G****G** | |
|  |  | NZ\_AAGP01000036.1/47955-48057  | **C****A****C****G****A****C****C****C****C****A****C**..**G**.**G**-**G**.**A****G****C****C****U****U****C**UG-..........................................................................................................................................................................A**A****G****A****G****G****C****U**GAG**A**.**U****C****A****G****G**CUGA.............................................................................AACCG**C****C****U**.**G****A**...........**G****A**-**C****C****G**UA.........................GA**A**.**C****C****U****G**A.U.**C****C****G****G**.UUGAU...............................A.**C****C****G****G**C**G**.U**A****G****G**.**A**A...G**U****G****A****G****G****A****C****U****G****C** | |
|  |  | NC\_005072.1/1543261-1543170  | **A****A****A****U****A****U****A****A****C****U****A**..**G**.**G****G****G**.**U****G****C****U****A****A****G**UCAAUU.......................................................................................................................................................................A**C****U****U****A****G****C****U**GAG**A**.---------..................................................................................---.--........UCA**U****A****C****C****C****U**UC.........................GA**A**.**C****C****U****G**A.A.**A****C****A****G**.UUAAA...............................A.**C****U****G****A**C**G**.C**A****G****G**.**A**A....**A****G****U****U****U****C****A****A****U****U** | |
|  |  | NC\_004459.1/721886-721768  | **A****A****C****C****A****C****U****C****A****U****C**..**G**.**G****G****G**.**C****G****C****U****U****A****A**CUGUGGUGUUUUAUUGCAUCG........................................................................................................................................................C**A****G****U****G****G****C****U**GAG**A**.**C****U****A****G****G**CAAA................................................................................GG**C****C****U**.**A****G**...........**G****A****C****C****C****G**U-.........................AA**A**.**C****C****U****G**A.A.**C****C****A****G**.AUAAU...............................G.**C****U****G****G**C**G**.U**A****G****G**.**A**A....**U****U****G****A****G****U****C****G****G****G** | |
|  |  | NC\_005139.1/427793-427911  | **A****A****C****C****A****C****U****C****A****U****C**..**G**.**G****G****G**.**C****G****C****U****U****A****A**CUGUGGUGUUUUAUUGCAUCG........................................................................................................................................................C**A****G****U****G****G****C****U**GAG**A**.**C****U****A****G****G**CAAA................................................................................GG**C****C****U**.**A****G**...........**G****A****C****C****C****G**U-.........................AA**A**.**C****C****U****G**A.A.**C****C****A****G**.AUAAU...............................G.**C****U****G****G**C**G**.U**A****G****G**.**A**A....**U****U****G****A****G****U****C****G****G****G** | |
|  |  | NC\_002678.2/1469030-1468924  | **G****C****C****C****A****U****C****C****A****C****A**..**G**.**G****G****G**.**U****G****C****U****C****C****G**UACGG........................................................................................................................................................................U**C****G****G****G****G****C****U**GAG**A**C**G****G****G****G****G**CGGC...............................................................................AAG**C****C****C**.**A****C**..........A**G****A****C****C****C****U**A-.........................GA**A**.**G****C****U****G**A.U.**C****U****G****G**.GUAAU...............................A.**C****C****A****G**C**G**.G**A****G****C**.**G**A...G**G****C****G****G****G****C****G****A****U****G** | |
|  |  | NZ\_AABH02000001.1/36465-36384  | **G****U****A****A****A****U****A****A****C****U****A**..**G**.**G****G****G**.**C****G****C****C****A****A**----..........................................................................................................................................................................--**U****U****G****G****C****U**GAG**A**.---------..................................................................................---.--.........AG**A****A****C****C****C****U**UU.........................GA**A**.**C****C****U****G**A.G.**A****U****G****G**.AUAAU...............................G.**C****C****A****G**C**G**.G**A****G****G**.**A**A....**A****G****U****G****A****A****U****U****G****U** | |
|  |  | NZ\_AAAQ02000006.1/124647-124559  | **G****C****U****G****U****G****G****A****C****G****C**..**G**.**G****G****G**.**U****G****C****C****A****C****C**CCCA.........................................................................................................................................................................G**G****G****U****G****G****C****U**GAG**A**.---------..................................................................................---.--........GGA**C****A****C****C****C****G**U-.........................GA**A**.**C****C****U****G**A.U.**C****U****A****G**.GUAGU...............................A.**C****U****A****G**C**G**.G**A****G****G**.**G**A....**C****G****U****C****C****A****A****U****U****G** | |
|  |  | NZ\_AABN02000006.1/6333-6432  | **C****G****C****C****A****G****U****G****C****U****G**..**G**.**G****G****G**.**G****G****C****C****U****U**----..........................................................................................................................................................................G**U****C****A****G****G****C****U**GAG**A**U**U****G****A****A****G**CGCA...............................................................................AAG**C****U****U**.**C****U**...........**G****A****C****C****C****C**UU.........................GA**A**.**C****C****U****G**A.U.**G****C****A****G**.UUAAC...............................A.**C****U****G****C**C**G**.A**A****G****G**.**G**A....**A****G****C****C****A****U****G****A****U****A** | |
|  |  | NC\_003997.3/752098-752203  | **A****U****A****G****U****U****U****G****C****U****A**..**G**.**G****A****G**.**A****G****C****U****G****G****U**GU-..........................................................................................................................................................................U**G****C****C****A****G****C****U**GAG**A**G**U****A****A****G****A**CCUU...............................................................................AAG**U****C****U**.**U****U**...........**G****A****U****C****C****U**UUUUA......................UU**A**.**C****C****U****G**A.U.**C****U****A****G**.AUUAU...............................G.**C****U****A****G**C**G**.U**A****G****G**.**G**A....**A****G****C****A****A****U****U****C****G****G** | |
|  |  | NC\_005945.1/751994-752099  | **A****U****A****G****U****U****U****G****C****U****A**..**G**.**G****A****G**.**A****G****C****U****G****G****U**GU-..........................................................................................................................................................................U**G****C****C****A****G****C****U**GAG**A**G**U****A****A****G****A**CCUU...............................................................................AAG**U****C****U**.**U****U**...........**G****A****U****C****C****U**UUUUA......................UU**A**.**C****C****U****G**A.U.**C****U****A****G**.AUUAU...............................G.**C****U****A****G**C**G**.U**A****G****G**.**G**A....**A****G****C****A****A****U****U****C****G****G** | |
|  |  | NC\_006274.1/742060-742165  | **A****U****A****G****U****U****U****G****C****U****A**..**G**.**G****A****G**.**A****G****C****U****G****G****U**GU-..........................................................................................................................................................................U**G****C****C****A****G****C****U**GAG**A**G**U****A****A****G****A**CCUU...............................................................................AAG**U****C****U**.**U****U**...........**G****A****U****C****C****U**UUUUA......................UU**A**.**C****C****U****G**A.U.**C****U****A****G**.AUUAU...............................G.**C****U****A****G**C**G**.U**A****G****G**.**G**A....**A****G****C****A****A****U****U****C****G****G** | |
|  |  | NC\_007530.2/752099-752204  | **A****U****A****G****U****U****U****G****C****U****A**..**G**.**G****A****G**.**A****G****C****U****G****G****U**GU-..........................................................................................................................................................................U**G****C****C****A****G****C****U**GAG**A**G**U****A****A****G****A**CCUU...............................................................................AAG**U****C****U**.**U****U**...........**G****A****U****C****C****U**UUUUA......................UU**A**.**C****C****U****G**A.U.**C****U****A****G**.AUUAU...............................G.**C****U****A****G**C**G**.U**A****G****G**.**G**A....**A****G****C****A****A****U****U****C****G****G** | |
|  |  | NZ\_AAAC02000001.1/1254089-1254194  | **A****U****A****G****U****U****U****G****C****U****A**..**G**.**G****A****G**.**A****G****C****U****G****G****U**GU-..........................................................................................................................................................................U**G****C****C****A****G****C****U**GAG**A**G**U****A****A****G****A**CCUU...............................................................................AAG**U****C****U**.**U****U**...........**G****A****U****C****C****U**UUUUA......................UU**A**.**C****C****U****G**A.U.**C****U****A****G**.AUUAU...............................G.**C****U****A****G**C**G**.U**A****G****G**.**G**A....**A****G****C****A****A****U****U****C****G****G** | |
|  |  | NZ\_AAEK01000002.1/9964-10069  | **A****U****A****G****U****U****U****G****C****U****A**..**G**.**G****A****G**.**A****G****C****U****G****G****U**GU-..........................................................................................................................................................................U**G****C****C****A****G****C****U**GAG**A**G**U****A****A****G****A**CCUU...............................................................................AAG**U****C****U**.**U****U**...........**G****A****U****C****C****U**UUUUA......................UU**A**.**C****C****U****G**A.U.**C****U****A****G**.AUUAU...............................G.**C****U****A****G**C**G**.U**A****G****G**.**G**A....**A****G****C****A****A****U****U****C****G****G** | |
|  |  | NZ\_AAEN01000010.1/401491-401386  | **A****U****A****G****U****U****U****G****C****U****A**..**G**.**G****A****G**.**A****G****C****U****G****G****U**GU-..........................................................................................................................................................................U**G****C****C****A****G****C****U**GAG**A**G**U****A****A****G****A**CCUU...............................................................................AAG**U****C****U**.**U****U**...........**G****A****U****C****C****U**UUUUA......................UU**A**.**C****C****U****G**A.U.**C****U****A****G**.AUUAU...............................G.**C****U****A****G**C**G**.U**A****G****G**.**G**A....**A****G****C****A****A****U****U****C****G****G** | |
|  |  | NZ\_AAEP01000028.1/3888-3993  | **A****U****A****G****U****U****U****G****C****U****A**..**G**.**G****A****G**.**A****G****C****U****G****G****U**GU-..........................................................................................................................................................................U**G****C****C****A****G****C****U**GAG**A**G**U****A****A****G****A**CCUU...............................................................................AAG**U****C****U**.**U****U**...........**G****A****U****C****C****U**UUUUA......................UU**A**.**C****C****U****G**A.U.**C****U****A****G**.AUUAU...............................G.**C****U****A****G**C**G**.U**A****G****G**.**G**A....**A****G****C****A****A****U****U****C****G****G** | |
|  |  | NZ\_AAEQ01000022.1/6450-6555  | **A****U****A****G****U****U****U****G****C****U****A**..**G**.**G****A****G**.**A****G****C****U****G****G****U**GU-..........................................................................................................................................................................U**G****C****C****A****G****C****U**GAG**A**G**U****A****A****G****A**CCUU...............................................................................AAG**U****C****U**.**U****U**...........**G****A****U****C****C****U**UUUUA......................UU**A**.**C****C****U****G**A.U.**C****U****A****G**.AUUAU...............................G.**C****U****A****G**C**G**.U**A****G****G**.**G**A....**A****G****C****A****A****U****U****C****G****G** | |
|  |  | NZ\_AAER01000025.1/5955-6060  | **A****U****A****G****U****U****U****G****C****U****A**..**G**.**G****A****G**.**A****G****C****U****G****G****U**GU-..........................................................................................................................................................................U**G****C****C****A****G****C****U**GAG**A**G**U****A****A****G****A**CCUU...............................................................................AAG**U****C****U**.**U****U**...........**G****A****U****C****C****U**UUUUA......................UU**A**.**C****C****U****G**A.U.**C****U****A****G**.AUUAU...............................G.**C****U****A****G**C**G**.U**A****G****G**.**G**A....**A****G****C****A****A****U****U****C****G****G** | |
|  |  | NZ\_AAES01000021.1/5964-6069  | **A****U****A****G****U****U****U****G****C****U****A**..**G**.**G****A****G**.**A****G****C****U****G****G****U**GU-..........................................................................................................................................................................U**G****C****C****A****G****C****U**GAG**A**G**U****A****A****G****A**CCUU...............................................................................AAG**U****C****U**.**U****U**...........**G****A****U****C****C****U**UUUUA......................UU**A**.**C****C****U****G**A.U.**C****U****A****G**.AUUAU...............................G.**C****U****A****G**C**G**.U**A****G****G**.**G**A....**A****G****C****A****A****U****U****C****G****G** | |
|  |  | NZ\_AABI03000001.1/462754-462853  | **C****G****C****G****C****U****C****C****U****U****A**..**G**.**G****G****G**.**A****G****G****C****U****G****C**---..........................................................................................................................................................................-**G****A****A****G****C****C****U**GAG**A**C**U****A****G****U****A**CUUA................................................................................UG**U****G****C**.**U****U**..........A**U****A****C****C****C****U**UC.........................GA**A**.**C****C****U****G**A.U.**C****C****G****G**.UUAGU...............................A.**C****C****G****G**C**G**.U**A****G****G**.**A**A....**U****A****G****G****A****U****A****A****A****A** | |
|  |  | NZ\_AAAI03000001.1/1128627-1128721  | **U****G****G****A****U****G****A****A****A****C****A**..**G**.**G****G****G**.**U****G****C****C****G****U****U**CGGAUGGGCC...................................................................................................................................................................G**G****G****C****G****G****C****U**GAG**A**.---------..................................................................................---.--.........GA**G****U****C****C****C****U**UC.........................GA**A**.**C****C****C****G**A.U.**C****C****G****G**.CUAGU...............................A.**C****C****G****G**C**G**.U**G****G****G**.**A**A....**G****U****U****U****C****A****A****A****U****A** | |
|  |  | NC\_003909.8/500911-501015  | **U****A****C****U****A****A****A****C****A****C****A**..**U**.**G****G****G**.**A****G****U****U****U****G****U**GGA..........................................................................................................................................................................U**A****C****A****A****A****C****U**GAG**A**G**U****A****U****G****A**CUAG..............................................................................UCCG**U****C****A**.**U****U**...........**G****A****C****C****A****U**UU.........................GA**A**.**C****C****U****G**-.U.-**U****G****G**.AUAAU...............................G.**C****C****A****G**C**G**.U**A****G****G**.**G**A..GA**G****U****G****U****A****A****A****A****G****C** | |
|  |  | NC\_003454.1/254989-254891  | **A****A****C****U****A****G****U****U****A****C****A**..**A**.**G****G****G**.**A****G****U****U****A****A****U**AA-..........................................................................................................................................................................-**A****U****U****G****A****C****U**GAG**A**A**A****A****G****G****A**UGUG.................................................................................A**G****C****C**.**U****U**...........**G****A****C****C****U****U**U-.........................UG**A**.**C****C****U****G**A.U.**U****U****G****G**.AUAAU...............................G.**C****C****A****A**C**G**.U**A****G****G**.**A**A....**G****U****A****A****A****A****G****A****G****U** | |
|  |  | NZ\_AABF02000072.1/5040-5138  | **A****A****C****U****A****G****U****U****A****C****A**..**A**.**G****G****G**.**A****G****U****U****A****A****U**AA-..........................................................................................................................................................................-**A****U****U****G****A****C****U**GAG**A**A**A****A****G****G****A**UGUG.................................................................................A**G****C****C**.**U****U**...........**G****A****C****C****U****U**U-.........................UG**A**.**C****C****U****G**A.U.**U****U****G****G**.AUAAU...............................G.**C****C****A****A**C**G**.U**A****G****G**.**A**A....**G****U****A****A****A****A****G****A****G****U** | |
|  |  | NC\_006570.1/1057441-1057347  | **A****A****A****A****G****A****C****A****A****C****A**..**G**.**G****G****G**.**U****G****C****U****G****U****U**UUUAUUAA.....................................................................................................................................................................A**A****A****U****G****G****C****U**GAG**A**.---------..................................................................................---.--........AGU**G****A****C****C****C****U**UU.........................GU**A**.**C****C****U****G**A.U.**C****U****A****A**.GUAAU...............................A.**U****U****A****G**C**G**.U**A****G****G**.**G**A...G**U****U****U****G****U****U****U****G****G****U** | |
|  |  | NC\_005706.1/14680-14568  | **G****G****U****U****C****C****U****G****U****C****A**..**G**.**G****G****G**.**A****G****U****C****U****C****G**CCA..........................................................................................................................................................................G**A****G****A****G****A****C****U**GAG**A**G**G****C****U****G****A**UAGC......................................................................GAUUUUCGCGGC**U****U****A**.**G****C**...........**G****A****C****C****C****U**UA.........................GA**A**.**C****C****U****G**A.C.**C****C****A****G**.CUGAU...............................A.**C****U****G****G**C**G**.U**A****G****G**.**A**A....**G****A****C****U****C****G****U****A****U****C** | |
|  |  | NZ\_AAEW01000047.1/10879-10783  | **G****A****U****G****G****C****U****G****C****U****G**..**G**.**G****G****G**.**A****G****U****G****A**-----..........................................................................................................................................................................--**U****U****C****A****C****U**GAG**A**.**G****C****U****C****U**GUUA..............................................................................GAUU**G****G****A**.**G****C**...........**G****A****C****C****C****U**UG.........................GA**A**.**C****C****U****G**A.U.**C****C****G****G**.AUUAU...............................A.**C****C****G****G**C**G**.U**A****G****G**.**G**A....**A****G****C****G****A****C****C****U****G****A** | |
|  |  | NC\_005957.1/743046-743151  | **A****U****A****G****C****U****U****G****C****U****A**..**G**.**G****A****G**.**A****G****C****U****G****G****U**GU-..........................................................................................................................................................................U**G****C****C****A****G****C****U**GAG**A**G**U****A****A****G****A**CCUU...............................................................................AAG**U****C****U**.**U****U**...........**G****A****U****C****C****U**UUUUA......................UU**A**.**C****C****U****G**A.U.**C****U****A****G**.AUUAU...............................G.**C****U****A****G**C**G**.U**A****G****G**.**G**A....**A****G****C****A****A****U****U****C****G****G** | |
|  |  | NC\_005071.1/178356-178452  | **G****C****A****A****A****A****C****A****C****U****A**..**G**.**G****G****G**.**U****G****C****C****A****U****U**CAGCAAUCGCU..................................................................................................................................................................G**A****G****U****G****G****C****U**GAG**A**.---------..................................................................................---.--........UCA**C****A****C****C****C****U**CC.........................GA**A**.**C****C****U****G**A.U.**A****C****G****G**.GUUGU...............................G.**C****C****G****G**C**G**.C**A****G****G**.**G**A....**A****G****U****G****A****A****A****U****G****A** | |
|  |  | NC\_003098.1/636353-636434  | **U****A****G****A****G****A****C****A****U****U****U**..**G**.**G****G****G**.**U****G****C****U****U**-----..........................................................................................................................................................................--**U****A****A****G****C****U**GAG**A**.---------..................................................................................---.--........UGA**U****A****C****C****C****A**UU.........................GA**A**.**C****C****U****G**A.U.**A****C****A****G**.UUAAG...............................A.**C****U****G****G**C**G**.A**A****G****G**.**G**A....**A****A****U****G****U****G****A****A****A****C** | |
|  |  | NZ\_AABO02000010.1/6026-5934  | **C****G****C****A****C****U****U****A****G****U****C**..**G**.**G****G****G**.**U****G****C****U****G****U****U**UGAUUUAU.....................................................................................................................................................................A**G****A****C****A****G****C****U**GAG**A**.---------..................................................................................---.--........UGA**U****A****C****C****C****G**U-.........................GA**A**.**C****C****U****G**A.U.**A****C****A****G**.CUAAU...............................A.**C****U****G****A**C**G**.U**A****G****G**.**A**A....**A****C****U****A****A****A****U****U****A****U** | |
|  |  | NC\_006300.1/631611-631525  | **C****C****U****C****A****U****U****A****G****U****C**..**G**.**G****G****G**.**U****G****C****U****G****C****A**UU-..........................................................................................................................................................................U**U****G****C****G****G****C****U**GAG**A**.---------..................................................................................---.--........UAA**U****A****C****C****C****G**U-.........................GA**A**.**C****C****U****G**A.A.**A****C****A****G**.CUAAU...............................A.**C****U****G****G**C**G**.U**A****G****G**.**G**A....**A****C****U****A****A****C****C****G****C****A** | |
|  |  | NZ\_AAAJ03000018.1/64881-64997  | **U****C****G****A****C****G****A****A****A****C****A**..**G**.**G****G****G**.**U****G****C****U****U****C****G**CGCACGGGCGGCAUGGUUUUCAUGCCGCGGCG.............................................................................................................................................G**C****G****A****G****G****C****U**GAG**A**.---------..................................................................................---.--.........GA**G****A****C****C****C****U**UU.........................GC**A**.**C****C****C****G**A.U.**C****C****G****G**.GUAAU...............................A.**C****C****G****G**C**G**.C**G****G****G**.**A**A....**G****U****U****U****C****C****G****G****A****A** | |
|  |  | NZ\_AAAE01000114.1/14764-14856  | **U****G****U****C****C****C****A****C****C****U****C**..**G**.**G****G****G**.**U****G****C****C****C****G****C**---..........................................................................................................................................................................-**A****A****G****G****G****C****U**GAG**A**.-**C****G****C****U**GCGC..................................................................................-**G****C**.**G**-...........**A****A****C****C****C****G**UU.........................GA**A**.**C****C****U****G**A.C.**C****C****G****G**.UUAGG...............................A.**C****C****G****G**C**G**.G**A****G****G**.**G**A....**A****G****G****U****G****C****A****U****G****G** | |
|  |  | NZ\_AAEW01000027.1/7802-7895  | **G****A****A****A****A****G****C****C****C****U****G**..**G**.**G****G****G**.**A****G****U****U****U**-----..........................................................................................................................................................................--**A****U****U****A****C****U**GAG**A**.**G****U****C****C****U**GUUG.................................................................................C**G****G****G**.**A****C**...........**G****A****C****C****C****U**UA.........................GA**A**.**C****C****U****G**A.U.**C****C****G****G**.GUUAU...............................A.**C****C****G****G**C**G**.U**A****G****G**.**G**A....**A****G****C****G****G****C****A****C****U****U** | |
|  |  | NZ\_AAAH01000537.2/1730-1638  | **C****G****U****G****U****U****C****C****G****U****A**..**G**.**G****G****G**.**C****G****C****U****U****U****G**CGACAAAC.....................................................................................................................................................................G**C****G****A****G****G****C****U**GAG**A**.---------..................................................................................---.--.........GG**A****A****C****C****C****U**U-.........................GA**A**.**C****C****U****G**A.U.**C****C****G****G**.GUAAU...............................A.**C****C****G****G**C**G**.U**A****G****G**.**A**A...A**U****C****G****G****A****A****C****C****G****G** | |
|  |  | NZ\_AADW02000010.1/84489-84391  | **A****G****C****G****A****C****C****A****C****U****A**..**G**.**G****G****G**.**A****G****U****C****A****G****U**AG-..........................................................................................................................................................................-**G****C****U****G****A****C****U**GAG**A**.**C****G****A****C****G**GAGA..................................................................................**C****G****U**G**C****G**...........**G****A****C****C****C****U**UU.........................GA**A**.**C****C****U****G**A.U.**U****A****A****G**.UUUGG...............................A.**C****U****U****A**C**G**.G**A****G****G**.**G**A....**A****G****U****G****G****C****G****U****G****A** | |
|  |  | NC\_003028.1/681818-681899  | **U****A****G****A****G****A****C****A****U****U****U**..**G**.**G****G****G**.**U****G****C****U****U**-----..........................................................................................................................................................................--**U****A****G****G****C****U**GAG**A**.---------..................................................................................---.--........UAA**U****A****C****C****C****A**UU.........................GA**A**.**C****C****U****G**A.U.**A****C****A****G**.UUAAG...............................A.**C****U****G****G**C**G**.A**A****G****G**.**G**A....**A****A****U****G****U****G****A****A****C****A** | |
|  |  | NZ\_AAGY01000143.1/77-158  | **U****A****G****A****G****A****C****A****U****U****U**..**G**.**G****G****G**.**U****G****C****U****U**-----..........................................................................................................................................................................--**U****A****G****G****C****U**GAG**A**.---------..................................................................................---.--........UAA**U****A****C****C****C****A**UU.........................GA**A**.**C****C****U****G**A.U.**A****C****A****G**.UUAAG...............................A.**C****U****G****G**C**G**.A**A****G****G**.**G**A....**A****A****U****G****U****G****A****A****C****A** | |
|  |  | NZ\_AAAH01000604.2/794-688  | **C****A****G****C****G****U****G****U****A****U****G**..**A**.**G****G****G**.**A****G****A****U****C****G****G**GGA..........................................................................................................................................................................G**C****C****G****G****G****C****U**GAG**A**G**G****A****U****G****G**UCGC............................................................................UAUCCA**C****C****A**.**U****C**...........**G****A****C****C****U****C**GU.........................GA**A**.**C****C****U****G**C.U.**C****U****G****G**.GUAAU...............................G.**C****C****A****G**C**G**.A**A****G****G**.**G**A....**A****U****A****C****A****C****G****G****U****G** | |
|  |  | NC\_004722.1/729116-729220  | **A****U****A****G****U****U****U****G****C****U****A**..**G**.**G****A****G**.**A****G****C****U****G****G****U**GU-..........................................................................................................................................................................U**G****C****C****A****G****C****U**GAG**A**.**G****U****A****G****G**CCGU...............................................................................AAG**C****C****U**.**U****U**...........**G****A****U****C****C****U**UUUUA......................UU**A**.**C****C****U****G**A.U.**C****U****A****G**.AUUAU...............................G.**C****U****A****G**C**G**.U**A****G****G**.**G**A....**A****G****C****A****A****U****U****C****G****G** | |
|  |  | NC\_006300.1/1517793-1517707  | **C****U****G****U****U****C****U****C****A****A****C**..**G**.**G****G****G**.**U****G****C****U****U****C****U**UC-..........................................................................................................................................................................-**G****G****A****G****G****C****U**GAG**A**.---------..................................................................................---.--.........GA**A****A****C****U****C****G**UA.........................GA**A**.**C****C****U****G**A.U.**C****U****G****A**.UUAAU...............................U.**A****C****A****G**C**G**.U**A****G****G**.**G**A...U**U****U****G****A****G****A****A****A****G****G** | |
|  |  | NC\_006300.1/1375924-1376007  | **C****G****C****C****G****U****U****A****G****U****C**..**G**.**G****G****G**.**U****G****C****U****U****A****A**---..........................................................................................................................................................................-**A****U****U****A****G****C****U**GAG**A**.---------..................................................................................---.--........AAA**U****A****C****C****C****G**U-.........................GA**A**.**C****C****U****G**A.U.**A****C****A****G**.UUAAC...............................A.**C****U****G****G**C**G**.U**A****G****G**.**A**A....**A****C****U****A****A****U****C****A****A****U** | |
|  |  | NZ\_AAAQ02000002.1/568740-568635  | **G****C****G****A****A****A****A****C****C****C****G**..**C**.**G****G****G**.**A****G****C****C****G****G****G**GCGGA........................................................................................................................................................................A**C****C****C****G****G****C****U**GAG**A**G**G****G****A****G****G**CUGU...............................................................................CCG**C****C****U**.**C****C**...........**G****A****C****C****G****C**A-.........................GA**A**.**C****C****U****G**A.U.**C****C****G****G**.GUCAU...............................A.**C****C****G****G**C**G**.A**A****G****G**.**G**A...G**U****G****G****C****G****C****G****C****C****G** | |
|  |  | NC\_000962.2/510326-510223  | **G****G****G****U****A****C****C****C****A****C****G**..**C**.**G****G****G**.**A****G****C****G****C****A****C**GCCG.........................................................................................................................................................................A**G****U****G****C****G****C****U**GAG**A**G**G****A****C****G****G**CUCG...............................................................................GGG**C****C****G**.**U****C**...........**G****A****C****C****G****U**AC.........................GA**A**.**C****C****U****G**A.-.**C****C****G****G**.GUAAU...............................G.**C****C****G****G**C**G**.U**A****G****G**.**G**A....**G****U****U****G****C****A****A****A****U****G** | |
|  |  | NC\_002755.2/511775-511672  | **G****G****G****U****A****C****C****C****A****C****G**..**C**.**G****G****G**.**A****G****C****G****C****A****C**GCCG.........................................................................................................................................................................A**G****U****G****C****G****C****U**GAG**A**G**G****A****C****G****G**CUCG...............................................................................GGG**C****C****G**.**U****C**...........**G****A****C****C****G****U**AC.........................GA**A**.**C****C****U****G**A.-.**C****C****G****G**.GUAAU...............................G.**C****C****G****G**C**G**.U**A****G****G**.**G**A....**G****U****U****G****C****A****A****A****U****G** | |
|  |  | NC\_002945.3/511345-511242  | **G****G****G****U****A****C****C****C****A****C****G**..**C**.**G****G****G**.**A****G****C****G****C****A****C**GCCG.........................................................................................................................................................................A**G****U****G****C****G****C****U**GAG**A**G**G****A****C****G****G**CUCG...............................................................................GGG**C****C****G**.**U****C**...........**G****A****C****C****G****U**AC.........................GA**A**.**C****C****U****G**A.-.**C****C****G****G**.GUAAU...............................G.**C****C****G****G**C**G**.U**A****G****G**.**G**A....**G****U****U****G****C****A****A****A****U****G** | |
|  |  | NC\_003450.3/1371746-1371643  | **G****G****C****A****G****U****C****C****C****C****A**..**C**.**G****G****G**.**C****G****C****C****C****G****A**GC-..........................................................................................................................................................................-**A****C****G****G****G****C****U**GAG**A**.**U****C****G****C****G**CUGA............................................................................UUGCUG**C****G****C**.**G****A**..........G**C****A****C****C****G****U**UU.........................GA**A**.**C****C****U****G**-.U.**C****C****G****G**.UUAGC...............................A.**C****C****G****G**C**G**.A**A****G****G**.**A**A....**G****A****G****A****G****G****A****A****U****G** | |
|  |  | NC\_006958.1/1373213-1373110  | **G****G****C****A****G****U****C****C****C****C****A**..**C**.**G****G****G**.**C****G****C****C****C****G****A**GC-..........................................................................................................................................................................-**A****C****G****G****G****C****U**GAG**A**.**U****C****G****C****G**CUGA............................................................................UUGCUG**C****G****C**.**G****A**..........G**C****A****C****C****G****U**UU.........................GA**A**.**C****C****U****G**-.U.**C****C****G****G**.UUAGC...............................A.**C****C****G****G**C**G**.A**A****G****G**.**A**A....**G****A****G****A****G****G****A****A****U****G** | |
|  |  | NC\_006513.1/996439-996533  | **G****C****A****G****C****U****C****G****U****U****G**..**G**.**G****G****G**.**U****G****C****C****C****G****U**GCGGCUUUCG...................................................................................................................................................................C**A****C****G****C****G****C****U**GAG**A**.---------..................................................................................---.--.........AA**A****A****C****C****C****U**UU.........................GA**A**.**C****C****U****G**A.U.**C****C****G****G**.AUCGC...............................A.**C****C****G****G**C**G**.U**A****G****G**.**G**A....**A****A****C****G****C****U****U****U****C****A** | |
|  |  | NC\_003155.3/8583516-8583429  | **G****U****A****C****C****G****G****A****C****A****C**..**G**.**G****G****G**.**U****G****C****C****C****C****A**UC-..........................................................................................................................................................................C**G****A****G****G****G****C****U**GAG**A**.---------..................................................................................---.--........UCA**C****A****C****C****C****G**UC.........................GA**A**.**C****C****U****G**A.A.**C****C****A****G**.UUCGU...............................A.**C****U****G****G**C**G**.G**A****G****G**.**G**A....**U****G****U****C****U****U****C****C****A****U** | |
|  |  | NC\_007146.1/1130519-1130599  | **C****A****A****G****C****C****U****A****G****U****C**..**G**.**G****G****G**.**U****G****C****A****A**-----..........................................................................................................................................................................--**U****A****C****G****C****U**GAG**A**.---------..................................................................................---.--........UCA**U****A****C****C****C****G**U-.........................GA**A**.**C****C****U****G**A.A.**A****C****A****G**.UUAAU...............................A.**C****U****G****A**C**G**.U**A****G****G**.**A**A....**A****C****U****A****G****G****A****A****U****A** | |
|  |  | NZ\_AADO01000001.1/138090-138010  | **C****A****A****G****C****C****U****A****G****U****C**..**G**.**G****G****G**.**U****G****C****A****A**-----..........................................................................................................................................................................--**U****A****C****G****C****U**GAG**A**.---------..................................................................................---.--........UCA**U****A****C****C****C****G**U-.........................GA**A**.**C****C****U****G**A.A.**A****C****A****G**.UUAAU...............................A.**C****U****G****A**C**G**.U**A****G****G**.**A**A....**A****C****U****A****G****G****A****A****U****A** | |
|  |  | NC\_000907.1/1082633-1082713  | **A****A****A****G****C****C****U****A****G****U****C**..**G**.**G****G****G**.**U****G****C****A****A**-----..........................................................................................................................................................................--**U****A****C****G****C****U**GAG**A**.---------..................................................................................---.--........UCA**U****A****C****C****C****G**U-.........................GA**A**.**C****C****U****G**A.A.**A****C****A****G**.UUAAU...............................A.**C****U****G****A**C**G**.U**A****G****G**.**A**A....**A****C****U****A****G****G****A****A****U****A** | |
|  |  | NZ\_AADP01000001.1/500097-500177  | **A****A****A****G****C****C****U****A****G****U****C**..**G**.**G****G****G**.**U****G****C****A****A**-----..........................................................................................................................................................................--**U****A****C****G****C****U**GAG**A**.---------..................................................................................---.--........UCA**U****A****C****C****C****G**U-.........................GA**A**.**C****C****U****G**A.A.**A****C****A****G**.UUAAU...............................A.**C****U****G****A**C**G**.U**A****G****G**.**A**A....**A****C****U****A****G****G****A****A****U****A** | |
|  |  | NC\_003997.3/393915-394017  | **A****A****U****A****A****G****C****A****C**-**A**..**U**.**G****G****G**.**A****G****U****U****U****G****U**GGA..........................................................................................................................................................................U**G****C****A****A****A****C****U**GAG**A**G**U****A****U****G****A**CUAU..............................................................................UCCG**U****C****A**.**U****U**...........**G****A****C****C****A****U**UU.........................GA**A**.**C****C****U****G**-.U.-**U****G****G**.AUAAU...............................G.**C****C****A****G**C**G**.U**A****G****G**.**G**A...G**A****G****U****G****U****A****A****A****A****G** | |
|  |  | NC\_005945.1/393928-394030  | **A****A****U****A****A****G****C****A****C**-**A**..**U**.**G****G****G**.**A****G****U****U****U****G****U**GGA..........................................................................................................................................................................U**G****C****A****A****A****C****U**GAG**A**G**U****A****U****G****A**CUAU..............................................................................UCCG**U****C****A**.**U****U**...........**G****A****C****C****A****U**UU.........................GA**A**.**C****C****U****G**-.U.-**U****G****G**.AUAAU...............................G.**C****C****A****G**C**G**.U**A****G****G**.**G**A...G**A****G****U****G****U****A****A****A****A****G** | |
|  |  | NC\_005957.1/417897-417999  | **A****A****U****A****A****G****C****A****C**-**A**..**U**.**G****G****G**.**A****G****U****U****U****G****U**GGA..........................................................................................................................................................................U**G****C****A****A****A****C****U**GAG**A**G**U****A****U****G****A**CUAU..............................................................................UCCG**U****C****A**.**U****U**...........**G****A****C****C****A****U**UU.........................GA**A**.**C****C****U****G**-.U.-**U****G****G**.AUAAU...............................G.**C****C****A****G**C**G**.U**A****G****G**.**G**A...G**A****G****U****G****U****A****A****A****A****G** | |
|  |  | NC\_006274.1/410331-410433  | **A****A****U****A****A****G****C****A****C**-**A**..**U**.**G****G****G**.**A****G****U****U****U****G****U**GGA..........................................................................................................................................................................U**G****C****A****A****A****C****U**GAG**A**G**U****A****U****G****A**CUAU..............................................................................UCCG**U****C****A**.**U****U**...........**G****A****C****C****A****U**UU.........................GA**A**.**C****C****U****G**-.U.-**U****G****G**.AUAAU...............................G.**C****C****A****G**C**G**.U**A****G****G**.**G**A...G**A****G****U****G****U****A****A****A****A****G** | |
|  |  | NC\_007530.2/393915-394017  | **A****A****U****A****A****G****C****A****C**-**A**..**U**.**G****G****G**.**A****G****U****U****U****G****U**GGA..........................................................................................................................................................................U**G****C****A****A****A****C****U**GAG**A**G**U****A****U****G****A**CUAU..............................................................................UCCG**U****C****A**.**U****U**...........**G****A****C****C****A****U**UU.........................GA**A**.**C****C****U****G**-.U.-**U****G****G**.AUAAU...............................G.**C****C****A****G**C**G**.U**A****G****G**.**G**A...G**A****G****U****G****U****A****A****A****A****G** | |
|  |  | NZ\_AAAC02000001.1/910803-910905  | **A****A****U****A****A****G****C****A****C**-**A**..**U**.**G****G****G**.**A****G****U****U****U****G****U**GGA..........................................................................................................................................................................U**G****C****A****A****A****C****U**GAG**A**G**U****A****U****G****A**CUAU..............................................................................UCCG**U****C****A**.**U****U**...........**G****A****C****C****A****U**UU.........................GA**A**.**C****C****U****G**-.U.-**U****G****G**.AUAAU...............................G.**C****C****A****G**C**G**.U**A****G****G**.**G**A...G**A****G****U****G****U****A****A****A****A****G** | |
|  |  | NZ\_AAEN01000023.1/135034-135136  | **A****A****U****A****A****G****C****A****C**-**A**..**U**.**G****G****G**.**A****G****U****U****U****G****U**GGA..........................................................................................................................................................................U**G****C****A****A****A****C****U**GAG**A**G**U****A****U****G****A**CUAU..............................................................................UCCG**U****C****A**.**U****U**...........**G****A****C****C****A****U**UU.........................GA**A**.**C****C****U****G**-.U.-**U****G****G**.AUAAU...............................G.**C****C****A****G**C**G**.U**A****G****G**.**G**A...G**A****G****U****G****U****A****A****A****A****G** | |
|  |  | NZ\_AAEO01000030.1/148478-148580  | **A****A****U****A****A****G****C****A****C**-**A**..**U**.**G****G****G**.**A****G****U****U****U****G****U**GGA..........................................................................................................................................................................U**G****C****A****A****A****C****U**GAG**A**G**U****A****U****G****A**CUAU..............................................................................UCCG**U****C****A**.**U****U**...........**G****A****C****C****A****U**UU.........................GA**A**.**C****C****U****G**-.U.-**U****G****G**.AUAAU...............................G.**C****C****A****G**C**G**.U**A****G****G**.**G**A...G**A****G****U****G****U****A****A****A****A****G** | |
|  |  | NZ\_AAEP01000046.1/100367-100469  | **A****A****U****A****A****G****C****A****C**-**A**..**U**.**G****G****G**.**A****G****U****U****U****G****U**GGA..........................................................................................................................................................................U**G****C****A****A****A****C****U**GAG**A**G**U****A****U****G****A**CUAU..............................................................................UCCG**U****C****A**.**U****U**...........**G****A****C****C****A****U**UU.........................GA**A**.**C****C****U****G**-.U.-**U****G****G**.AUAAU...............................G.**C****C****A****G**C**G**.U**A****G****G**.**G**A...G**A****G****U****G****U****A****A****A****A****G** | |
|  |  | NZ\_AAEQ01000043.1/94049-93947  | **A****A****U****A****A****G****C****A****C**-**A**..**U**.**G****G****G**.**A****G****U****U****U****G****U**GGA..........................................................................................................................................................................U**G****C****A****A****A****C****U**GAG**A**G**U****A****U****G****A**CUAU..............................................................................UCCG**U****C****A**.**U****U**...........**G****A****C****C****A****U**UU.........................GA**A**.**C****C****U****G**-.U.-**U****G****G**.AUAAU...............................G.**C****C****A****G**C**G**.U**A****G****G**.**G**A...G**A****G****U****G****U****A****A****A****A****G** | |
|  |  | NZ\_AAER01000042.1/139779-139677  | **A****A****U****A****A****G****C****A****C**-**A**..**U**.**G****G****G**.**A****G****U****U****U****G****U**GGA..........................................................................................................................................................................U**G****C****A****A****A****C****U**GAG**A**G**U****A****U****G****A**CUAU..............................................................................UCCG**U****C****A**.**U****U**...........**G****A****C****C****A****U**UU.........................GA**A**.**C****C****U****G**-.U.-**U****G****G**.AUAAU...............................G.**C****C****A****G**C**G**.U**A****G****G**.**G**A...G**A****G****U****G****U****A****A****A****A****G** | |
|  |  | NZ\_AAES01000043.1/100037-100139  | **A****A****U****A****A****G****C****A****C**-**A**..**U**.**G****G****G**.**A****G****U****U****U****G****U**GGA..........................................................................................................................................................................U**G****C****A****A****A****C****U**GAG**A**G**U****A****U****G****A**CUAU..............................................................................UCCG**U****C****A**.**U****U**...........**G****A****C****C****A****U**UU.........................GA**A**.**C****C****U****G**-.U.-**U****G****G**.AUAAU...............................G.**C****C****A****G**C**G**.U**A****G****G**.**G**A...G**A****G****U****G****U****A****A****A****A****G** | |
|  |  | NC\_006270.2/3124875-3124967  | **G****A****U****G****A****C****C****A****C****A****A**..**G**.**G****G****G**.**A****G****C****C****U****C**----..........................................................................................................................................................................--**C****G****G****G****C****U**GAG**A**G**U****G****A****A****C**GACC..................................................................................**G****U****U**.**C****U**...........**G****A****C****C****C****U**UC.........................GA**A**.**C****C****U****G**-.U.-**U****A****G**.UUAAU...............................G.**C****U****G****G**C**G**.U**A****G****G**.**A**A....**U****G****U****G****G****C****A****A****A****G** | |
|  |  | NC\_006322.1/3125049-3125141  | **G****A****U****G****A****C****C****A****C****A****A**..**G**.**G****G****G**.**A****G****C****C****U****C**----..........................................................................................................................................................................--**C****G****G****G****C****U**GAG**A**G**U****G****A****A****C**GACC..................................................................................**G****U****U**.**C****U**...........**G****A****C****C****C****U**UC.........................GA**A**.**C****C****U****G**-.U.-**U****A****G**.UUAAU...............................G.**C****U****G****G**C**G**.U**A****G****G**.**A**A....**U****G****U****G****G****C****A****A****A****G** | |
|  |  | NC\_004668.1/2676348-2676265  | **A****A****A****A****A****A****C****A****U****U****U**..**G**.**G****G****G**.**U****G****C****U****G****U**----..........................................................................................................................................................................-**U****A****U****G****G****C****U**GAG**A**.---------..................................................................................---.--........UGA**U****A****C****C****C****A**UU.........................GA**A**.**C****C****U****G**A.U.**G****C****A****G**.UUAGU...............................A.**C****U****G****U**C**G**.C**A****G****G**.**G**A....**A****A****U****G****C****C****G****A****U****U** | |
|  |  | NZ\_AAEK01000015.1/94340-94237  | **A****A****U****A****A****G****C****A****C**-**A**..**U**.**G****G****G**.**A****G****U****U****U****G****U**GGA..........................................................................................................................................................................U**A****C****A****A****A****C****U**GAG**A**G**U****A****U****G****A**CUAA..............................................................................UCCG**U****C****A**.**U****U**...........**G****A****C****C****A****U**UU.........................GA**A**.**C****C****U****G**-.U.-**U****G****G**.AUAAU...............................G.**C****C****A****G**C**G**.U**A****G****G**.**G**A..GA**A****G****U****G****U****A****A****A****A****G** | |
|  |  | NC\_006370.1/420522-420400  | **C****C****G****U****U****C****U****C****A****U****U**..**G**.**G****G****G**.**A****G****C****C****C****G****U**AUUUAUACAGAUGCACGUAGCAACUAGAG................................................................................................................................................U**A****C****A****G****G****C****U**GAG**A**.**U****U****G****C****A**UC--..................................................................................-**G****C**.**A****G**...........**A****A****C****C****C****A**CA.........................GA**A**.**C****C****U****G**A.A.**C****C****A****G**.GUCAU...............................G.**C****U****G****G**C**G**.U**A****G****G**.**A**A....**U****U****G****A****G****A****A****A****A****G** | |
|  |  | NC\_004663.1/2977734-2977823  | **U****U****C****A****C****U****A****A****G****U****A**..**G**.**G****G****G**.**U****G****C****C****U****U****A**ACAU.........................................................................................................................................................................G**A****C****G****G****G****C****U**GAG**A**.---------..................................................................................---.--........ACA**U****A****C****C****C****A**UA.........................GA**A**.**U****C****U****G**A.A.**C****C****G****G**.GUAAU...............................G.**C****C****G****G**C**G**.U**A****G****A**.**G**A....**A****C****U****A****A****U****U****A****A****U** | |
|  |  | NC\_003197.1/4382698-4382480  | **G****U****U****A****U****C****U****U****G****U****C**..**G**.**G****A****G**.**U****G****C****U****A****A****U**UUUCCACAAAAGCGUUCGUGAUGCGUCAAGGCGGCAAGUCGGUGAAUCUCCAGGAGCUUACAUAAGUAAGUGACUGGAGUGAGCGGACGAAGCCAACGAAGAGGCAGCGCGAAGGAUGAAGUGG.................................................A**A****A****A****G****G****C****U**GAG**A**.**C****C****G****U****U**AAUU..................................................................................--**C**.**G****G**...........**G****A****U****C****C****G**CG.........................GA**A**.**C****C****U****G**A.U.**C****A****G****G**.UUAAU...............................A.**C****C****U****G**C**G**.A**A****G****G**.**G**A....**A****C****A****A****G****A****G****U****A****A** | |
|  |  | NC\_003198.1/3569836-3570054  | **G****U****U****A****U****C****U****U****G****U****C**..**G**.**G****A****G**.**U****G****C****U****A****A****U**UUUCCACAAAAGCGUUCGUGAUGCGUCAAGGCGGCAAGUCGGUGAAUCUCCAGGAGCUUACAUAAGUAAGUGACUGGAGUGAGCGGACGAAGCCAACGAAGAGGCAGCGCGAAGGAUGAAGUGG.................................................A**A****A****A****G****G****C****U**GAG**A**.**C****C****G****U****U**AAUU..................................................................................--**C**.**G****G**...........**G****A****U****C****C****G**CG.........................GA**A**.**C****C****U****G**A.U.**C****A****G****G**.UUAAU...............................A.**C****C****U****G**C**G**.A**A****G****G**.**G**A....**A****C****A****A****G****A****G****U****A****A** | |
|  |  | NC\_004631.1/3555495-3555713  | **G****U****U****A****U****C****U****U****G****U****C**..**G**.**G****A****G**.**U****G****C****U****A****A****U**UUUCCACAAAAGCGUUCGUGAUGCGUCAAGGCGGCAAGUCGGUGAAUCUCCAGGAGCUUACAUAAGUAAGUGACUGGAGUGAGCGGACGAAGCCAACGAAGAGGCAGCGCGAAGGAUGAAGUGG.................................................A**A****A****A****G****G****C****U**GAG**A**.**C****C****G****U****U**AAUU..................................................................................--**C**.**G****G**...........**G****A****U****C****C****G**CG.........................GA**A**.**C****C****U****G**A.U.**C****A****G****G**.UUAAU...............................A.**C****C****U****G**C**G**.A**A****G****G**.**G**A....**A****C****A****A****G****A****G****U****A****A** | |
|  |  | NC\_006511.1/4155004-4154786  | **G****U****U****A****U****C****U****U****G****U****C**..**G**.**G****A****G**.**U****G****C****U****A****A****U**UUUCCACAAAAGCGUUCGUGAUGCGUCAAGGCGGCAAGUCGGUGAAUCUCCAGGAGCUUACAUAAGUAAGUGACUGGAGUGAGCGGACGAAGCCAACGAAGAGGCAGCGCGAAGGAUGAAGUGG.................................................A**A****A****A****G****G****C****U**GAG**A**.**C****C****G****U****U**AAUU..................................................................................--**C**.**G****G**...........**G****A****U****C****C****G**CG.........................GA**A**.**C****C****U****G**A.U.**C****A****G****G**.UUAAU...............................A.**C****C****U****G**C**G**.A**A****G****G**.**G**A....**A****C****A****A****G****A****G****U****A****A** | |
|  |  | NC\_006905.1/4316273-4316055  | **G****U****U****A****U****C****U****U****G****U****C**..**G**.**G****A****G**.**U****G****C****U****A****A****U**UUUCCACAAAAGCGUUCGUGAUGCGUCAAGGCGGCAAGUCGGUGAAUCUCCAGGAGCUUACAUAAGUAAGUGACUGGAGUGAGCGGACGAAGCCAACGAAGAGGCAGCGCGAAGGAUGAAGUGG.................................................A**A****A****A****G****G****C****U**GAG**A**.**C****C****G****U****U**AAUU..................................................................................--**C**.**G****G**...........**G****A****U****C****C****G**CG.........................GA**A**.**C****C****U****G**A.U.**C****A****G****G**.UUAAU...............................A.**C****C****U****G**C**G**.A**A****G****G**.**G**A....**A****C****A****A****G****A****G****U****A****A** | |
|  |  | NC\_005296.1/4039380-4039511  | **G****C****C****C****G****U****U****C****C****G****A**..**G**.**G****G****G**.**G****G****C****U****C****C****G**AC-..........................................................................................................................................................................G**A****G****G****A****G****C****U**GAG**A**U**A****C****C****G****C**ACGC...................................................UCGCCGCUUCGCUCCGCGAGGCACAGAGACC**G****C****G**.**G****U**...........**G****A****C****C****C****U**UU.........................GA**A**.**C****C****U****G**A.U.**C****C****G****G**.GUCAU...............................G.**C****C****G****G**C**G**.A**A****G****G**.**G**A...C**A****G****G****G****A****U****G****C****A****U** | |
|  |  | NZ\_AAIE01000001.1/426279-426180  | **A****A****G****U****U****G****A****U**-**C****G**..**C**.**G****G****G**.**A****G****C****U****C****C****G**CC-..........................................................................................................................................................................A**A****G****G****G****G****C****U**GAG**A**G**G****G****C****G****G**CUGG................................................................................GG**C****C****G**.**C****C**...........**G****A****C****C****G****C**AG.........................GA**A**.**C****C****U****G**-.U.**C****C****G****G**.GUAAU...............................G.**C****C****G****G**C**G**.U**A****G****G**.**G**A....**G****U****C****U****U****A****C****A****U****G** | |
|  |  | NZ\_AABI03000001.1/653146-653038  | **U****C****A****U****U****C****U****U****G****U****C**..**G**.**G****G****G**.**U****G****C****U****A****G****U**UUUUGUGUGCUUUAUUUGCGUGCAA....................................................................................................................................................A**A****C****A****G****G****C****U**GAG**A**.---------..................................................................................---.--.........AA**A****A****C****C****C****G**U-.........................UG**A**.**C****C****U****G**A.U.**C****C****G****G**.CUAGU...............................A.**C****C****G****G**C**G**.U**A****G****G**.**G**A....**A****C****G****A****G****A****A****G****A****G** | |
|  |  | NC\_006348.1/2819412-2819288  | **C****C****G****A****C****G****A****A****A****C****A**..**G**.**G****G****G**.**U****G****C****U****U****C****G**CGCGUCCCCGGCCUGCCGAGUCGGCACGGCCGGAUGCGCG.....................................................................................................................................A**C****G****A****G****G****C****U**GAG**A**.---------..................................................................................---.--.........GA**G****A****C****C****C****U**UC.........................GC**A**.**C****C****C****G**A.U.**C****C****G****G**.GUAAU...............................A.**C****C****G****G**C**G**.C**G****G****G**.**A**A....**G****U****U****U****C****C****G****A****U****C** | |
|  |  | NC\_006350.1/3753517-3753393  | **C****C****G****A****C****G****A****A****A****C****A**..**G**.**G****G****G**.**U****G****C****U****U****C****G**CGCGUCCCCGGCCUGCCGAGUCGGCACGGCCGGAUGCGCG.....................................................................................................................................A**C****G****A****G****G****C****U**GAG**A**.---------..................................................................................---.--.........GA**G****A****C****C****C****U**UC.........................GC**A**.**C****C****C****G**A.U.**C****C****G****G**.GUAAU...............................A.**C****C****G****G**C**G**.C**G****G****G**.**A**A....**G****U****U****U****C****C****G****A****U****C** | |
|  |  | NZ\_AAHM01000003.1/36830-36954  | **C****C****G****A****C****G****A****A****A****C****A**..**G**.**G****G****G**.**U****G****C****U****U****C****G**CGCGUCCCCGGCCUGCCGAGUCGGCACGGCCGGAUGCGCG.....................................................................................................................................A**C****G****A****G****G****C****U**GAG**A**.---------..................................................................................---.--.........GA**G****A****C****C****C****U**UC.........................GC**A**.**C****C****C****G**A.U.**C****C****G****G**.GUAAU...............................A.**C****C****G****G**C**G**.C**G****G****G**.**A**A....**G****U****U****U****C****C****G****A****U****C** | |
|  |  | NZ\_AAHN01000018.1/56991-57115  | **C****C****G****A****C****G****A****A****A****C****A**..**G**.**G****G****G**.**U****G****C****U****U****C****G**CGCGUCCCCGGCCUGCCGAGUCGGCACGGCCGGAUGCGCG.....................................................................................................................................A**C****G****A****G****G****C****U**GAG**A**.---------..................................................................................---.--.........GA**G****A****C****C****C****U**UC.........................GC**A**.**C****C****C****G**A.U.**C****C****G****G**.GUAAU...............................A.**C****C****G****G**C**G**.C**G****G****G**.**A**A....**G****U****U****U****C****C****G****A****U****C** | |
|  |  | NZ\_AAHO01000029.1/57210-57086  | **C****C****G****A****C****G****A****A****A****C****A**..**G**.**G****G****G**.**U****G****C****U****U****C****G**CGCGUCCCCGGCCUGCCGAGUCGGCACGGCCGGAUGCGCG.....................................................................................................................................A**C****G****A****G****G****C****U**GAG**A**.---------..................................................................................---.--.........GA**G****A****C****C****C****U**UC.........................GC**A**.**C****C****C****G**A.U.**C****C****G****G**.GUAAU...............................A.**C****C****G****G**C**G**.C**G****G****G**.**A**A....**G****U****U****U****C****C****G****A****U****C** | |
|  |  | NZ\_AAHP01000027.1/54004-53880  | **C****C****G****A****C****G****A****A****A****C****A**..**G**.**G****G****G**.**U****G****C****U****U****C****G**CGCGUCCCCGGCCUGCCGAGUCGGCACGGCCGGAUGCGCG.....................................................................................................................................A**C****G****A****G****G****C****U**GAG**A**.---------..................................................................................---.--.........GA**G****A****C****C****C****U**UC.........................GC**A**.**C****C****C****G**A.U.**C****C****G****G**.GUAAU...............................A.**C****C****G****G**C**G**.C**G****G****G**.**A**A....**G****U****U****U****C****C****G****A****U****C** | |
|  |  | NZ\_AAHQ01000008.1/301091-300967  | **C****C****G****A****C****G****A****A****A****C****A**..**G**.**G****G****G**.**U****G****C****U****U****C****G**CGCGUCCCCGGCCUGCCGAGUCGGCACGGCCGGAUGCGCG.....................................................................................................................................A**C****G****A****G****G****C****U**GAG**A**.---------..................................................................................---.--.........GA**G****A****C****C****C****U**UC.........................GC**A**.**C****C****C****G**A.U.**C****C****G****G**.GUAAU...............................A.**C****C****G****G**C**G**.C**G****G****G**.**A**A....**G****U****U****U****C****C****G****A****U****C** | |
|  |  | NZ\_AAHR01000026.1/57397-57521  | **C****C****G****A****C****G****A****A****A****C****A**..**G**.**G****G****G**.**U****G****C****U****U****C****G**CGCGUCCCCGGCCUGCCGAGUCGGCACGGCCGGAUGCGCG.....................................................................................................................................A**C****G****A****G****G****C****U**GAG**A**.---------..................................................................................---.--.........GA**G****A****C****C****C****U**UC.........................GC**A**.**C****C****C****G**A.U.**C****C****G****G**.GUAAU...............................A.**C****C****G****G**C**G**.C**G****G****G**.**A**A....**G****U****U****U****C****C****G****A****U****C** | |
|  |  | NZ\_AAHS01000013.1/78189-78065  | **C****C****G****A****C****G****A****A****A****C****A**..**G**.**G****G****G**.**U****G****C****U****U****C****G**CGCGUCCCCGGCCUGCCGAGUCGGCACGGCCGGAUGCGCG.....................................................................................................................................A**C****G****A****G****G****C****U**GAG**A**.---------..................................................................................---.--.........GA**G****A****C****C****C****U**UC.........................GC**A**.**C****C****C****G**A.U.**C****C****G****G**.GUAAU...............................A.**C****C****G****G**C**G**.C**G****G****G**.**A**A....**G****U****U****U****C****C****G****A****U****C** | |
|  |  | NZ\_AAHT01000001.1/4046928-4046804  | **C****C****G****A****C****G****A****A****A****C****A**..**G**.**G****G****G**.**U****G****C****U****U****C****G**CGCGUCCCCGGCCUGCCGAGUCGGCACGGCCGGAUGCGCG.....................................................................................................................................A**C****G****A****G****G****C****U**GAG**A**.---------..................................................................................---.--.........GA**G****A****C****C****C****U**UC.........................GC**A**.**C****C****C****G**A.U.**C****C****G****G**.GUAAU...............................A.**C****C****G****G**C**G**.C**G****G****G**.**A**A....**G****U****U****U****C****C****G****A****U****C** | |
|  |  | NZ\_AAHU01000011.1/55834-55958  | **C****C****G****A****C****G****A****A****A****C****A**..**G**.**G****G****G**.**U****G****C****U****U****C****G**CGCGUCCCCGGCCUGCCGAGUCGGCACGGCCGGAUGCGCG.....................................................................................................................................A**C****G****A****G****G****C****U**GAG**A**.---------..................................................................................---.--.........GA**G****A****C****C****C****U**UC.........................GC**A**.**C****C****C****G**A.U.**C****C****G****G**.GUAAU...............................A.**C****C****G****G**C**G**.C**G****G****G**.**A**A....**G****U****U****U****C****C****G****A****U****C** | |
|  |  | NZ\_AAHV01000012.1/55322-55446  | **C****C****G****A****C****G****A****A****A****C****A**..**G**.**G****G****G**.**U****G****C****U****U****C****G**CGCGUCCCCGGCCUGCCGAGUCGGCACGGCCGGAUGCGCG.....................................................................................................................................A**C****G****A****G****G****C****U**GAG**A**.---------..................................................................................---.--.........GA**G****A****C****C****C****U**UC.........................GC**A**.**C****C****C****G**A.U.**C****C****G****G**.GUAAU...............................A.**C****C****G****G**C**G**.C**G****G****G**.**A**A....**G****U****U****U****C****C****G****A****U****C** | |
|  |  | NZ\_AAHW01000028.1/42908-42784  | **C****C****G****A****C****G****A****A****A****C****A**..**G**.**G****G****G**.**U****G****C****U****U****C****G**CGCGUCCCCGGCCUGCCGAGUCGGCACGGCCGGAUGCGCG.....................................................................................................................................A**C****G****A****G****G****C****U**GAG**A**.---------..................................................................................---.--.........GA**G****A****C****C****C****U**UC.........................GC**A**.**C****C****C****G**A.U.**C****C****G****G**.GUAAU...............................A.**C****C****G****G**C**G**.C**G****G****G**.**A**A....**G****U****U****U****C****C****G****A****U****C** | |
|  |  | NZ\_AAIQ01000725.1/1168-1044  | **C****C****G****A****C****G****A****A****A****C****A**..**G**.**G****G****G**.**U****G****C****U****U****C****G**CGCGUCCCCGGCCUGCCGAGUCGGCACGGCCGGAUGCGCG.....................................................................................................................................A**C****G****A****G****G****C****U**GAG**A**.---------..................................................................................---.--.........GA**G****A****C****C****C****U**UC.........................GC**A**.**C****C****C****G**A.U.**C****C****G****G**.GUAAU...............................A.**C****C****G****G**C**G**.C**G****G****G**.**A**A....**G****U****U****U****C****C****G****A****U****C** | |
|  |  | NC\_006677.1/1475626-1475504  | **C****G****C****U****U****C****A****C****C****G****U**..**G**.**G****G****G**.**G****G****C****U****C****C****G**GC-..........................................................................................................................................................................A**U****G****G****G****G****C****U**GAG**A**U**U****C****U****G****C**UGGU...........................................................GUGACCCUUAAAAGUCAUGCGGU**G****C****A**.**G****U**...........**G****A****C****C****C****A**UU.........................GA**A**.**C****C****U****G**A.U.**C****C****A****G**.UUCAU...............................A.**C****U****G****G**C**G**.G**A****G****G**.**G**A....**U****G****G****U****G****G****U****C****G****C** | |
|  |  | NC\_003197.1/2241973-2241884  | **C****U****U****G****A****C****G****A****C****U****C**..**G**.**G****G****G**.**U****G****C****C****C****U****U**CUUUG........................................................................................................................................................................U**G****A****A****G****G****C****U**GAG**A**.---------..................................................................................---.--.........AA**U****A****C****C****C****G**UA.........................CC**A**.**C****C****U****G**A.U.**C****U****G****G**.AUAAU...............................G.**C****C****A****G**C**G**.U**A****G****G**.**G**A....**A****G****U****C****U****G****A****C****A****C** | |
|  |  | NC\_003198.1/2207556-2207467  | **C****U****U****G****A****C****G****A****C****U****C**..**G**.**G****G****G**.**U****G****C****C****C****U****U**CUUUG........................................................................................................................................................................U**G****A****A****G****G****C****U**GAG**A**.---------..................................................................................---.--.........AA**U****A****C****C****C****G**UA.........................CC**A**.**C****C****U****G**A.U.**C****U****G****G**.AUAAU...............................G.**C****C****A****G**C**G**.U**A****G****G**.**G**A....**A****G****U****C****U****G****A****C****A****C** | |
|  |  | NC\_004631.1/796380-796469  | **C****U****U****G****A****C****G****A****C****U****C**..**G**.**G****G****G**.**U****G****C****C****C****U****U**CUUUG........................................................................................................................................................................U**G****A****A****G****G****C****U**GAG**A**.---------..................................................................................---.--.........AA**U****A****C****C****C****G**UA.........................CC**A**.**C****C****U****G**A.U.**C****U****G****G**.AUAAU...............................G.**C****C****A****G**C**G**.U**A****G****G**.**G**A....**A****G****U****C****U****G****A****C****A****C** | |
|  |  | NC\_005363.1/1319798-1319709  | **A****C****C****U****G****A****C****G****C****U****A**..**G**.**G****G****G**.**U****G****U****U****G****G****U**GAAUU........................................................................................................................................................................C**A****C****C****G****A****C****U**GAG**A**.---------..................................................................................---.--.........AU**A****A****C****C****C****U**UU.........................GA**A**.**C****C****U****G**A.U.**A****G****A****G**.AUAAU...............................G.**C****U****C****G**C**G**.C**A****G****G**.**G**A....**A****G****C****A****A****G****A****A****U****A** | |
|  |  | NC\_006511.1/789113-789202  | **C****U****U****G****A****C****G****A****C****U****C**..**G**.**G****G****G**.**U****G****C****C****C****U****U**CUUUG........................................................................................................................................................................U**G****A****A****G****G****C****U**GAG**A**.---------..................................................................................---.--.........AA**U****A****C****C****C****G**UA.........................CC**A**.**C****C****U****G**A.U.**C****U****G****G**.AUAAU...............................G.**C****C****A****G**C**G**.U**A****G****G**.**G**A....**A****G****U****C****U****G****A****C****A****C** | |
|  |  | NC\_006905.1/2264491-2264402  | **C****U****U****G****A****C****G****A****C****U****C**..**G**.**G****G****G**.**U****G****C****C****C****U****U**CUUUG........................................................................................................................................................................U**G****A****A****G****G****C****U**GAG**A**.---------..................................................................................---.--.........AA**U****A****C****C****C****G**UA.........................CC**A**.**C****C****U****G**A.U.**C****U****G****G**.AUAAU...............................G.**C****C****A****G**C**G**.U**A****G****G**.**G**A....**A****G****U****C****U****G****A****C****A****C** | |
|  |  | NC\_006840.1/270635-270740  | **G****A****C****U****U****C****U****C****A****U****C**..**G**.**G****G****G**.**A****G****C****U**------..........................................................................................................................................................................--**U****U****G****G****C****U**GAG**A**.**U****U****A****A****U**GAAG....................................................................AUUGGAUAAUGUUC**A****U****U**.**U****A**...........**A****A****C****C****C****G**UA.........................UC**A**.**C****C****U****G**A.A.**C****C****A****G**.AUAAU...............................G.**C****U****G****G**C**G**.U**A****G****G**.**A**A....**U****U****G****A****G****A****U****G****A****C** | |
|  |  | NZ\_AAEI01000033.1/52065-52181  | **A****C****G****A****C****G****A****A****A****C****A**..**G**.**G****G****G**.**U****G****C****U****U****C****G**UGGCGCGGCCGGCGGAUGUUCCGGGCAGCGCG.............................................................................................................................................A**U****G****A****G****G****C****U**GAG**A**.---------..................................................................................---.--.........AA**A****A****C****C****C****U**UU.........................GC**A**.**C****C****C****G**A.U.**C****C****G****G**.GUAAU...............................A.**C****C****G****G**C**G**.A**G****G****G**.**A**A....**G****U****U****U****C****U****G****A****A****C** | |
|  |  | NC\_004116.1/815866-815775  | **A****A****A****U****A****C****A****C****A****A****G**..**G**.**G****A****G**.**U****G****C****C****U**-----..........................................................................................................................................................................--**U****G****A****G****C****U**GAG**A**.**U****U****G****C****A**GAUA..................................................................................**U****G****C**.**A****A**...........**A****A****U****C****C****U**C-.........................UA**A**.**C****C****U****G**A.U.**C****U****C****G**.UUAGG...............................A.**C****G****A****G**C**G**.U**A****G****G**.**A**A....**U****U****G****U****G****G****U****U****U****G** | |
|  |  | NC\_004337.1/73452-73360  | **C****C****G****U****U****C****U****C****A****A****C**..**G**.**G****G****G**.**U****G****C**-**C****A****C**GCAAACG......................................................................................................................................................................C**G****U****G****C****G****C****U**GAG**A**.---------..................................................................................---.--........AAA**U****A****C****C****C****G**UC.........................GA**A**.**C****C****U****G**A.U.**C****C****G****G**.AUAAC...............................G.**C****C****G****G**C**G**.A**A****G****G**.**G**A...U**U****U****G****A****G****G****C****U****C****C** | |
|  |  | NC\_004368.1/860304-860213  | **A****A****A****U****A****C****A****C****A****A****G**..**G**.**G****A****G**.**U****G****C****C****U**-----..........................................................................................................................................................................--**U****G****A****G****C****U**GAG**A**.**U****U****G****C****A**GAUA..................................................................................**U****G****C**.**A****A**...........**A****A****U****C****C****U**C-.........................UA**A**.**C****C****U****G**A.U.**C****U****C****G**.UUAGG...............................A.**C****G****A****G**C**G**.U**A****G****G**.**A**A....**U****U****G****U****G****G****U****U****U****G** | |
|  |  | NC\_004431.1/79951-79859  | **C****C****G****U****U****C****U****C****A****A****C**..**G**.**G****G****G**.**U****G****C**-**C****A****C**GCAGCCG......................................................................................................................................................................C**G****U****G****C****G****C****U**GAG**A**.---------..................................................................................---.--........AAA**U****A****C****C****C****G**UC.........................GA**A**.**C****C****U****G**A.U.**C****C****G****G**.AUAAC...............................G.**C****C****G****G**C**G**.A**A****G****G**.**G**A...U**U****U****G****A****G****G****C****U****C****C** | |
|  |  | NC\_004741.1/72938-72846  | **C****C****G****U****U****C****U****C****A****A****C**..**G**.**G****G****G**.**U****G****C**-**C****A****C**GCAAACG......................................................................................................................................................................C**G****U****G****C****G****C****U**GAG**A**.---------..................................................................................---.--........AAA**U****A****C****C****C****G**UC.........................GA**A**.**C****C****U****G**A.U.**C****C****G****G**.AUAAC...............................G.**C****C****G****G**C**G**.A**A****G****G**.**G**A...U**U****U****G****A****G****G****C****U****C****C** | |
|  |  | NC\_003450.3/1126306-1126408  | **U****U****U****C****A****U****A****G****A****C****A**..**C**.**G****G****G**.**U****G****C****U****C****G****G**UGAAAA.......................................................................................................................................................................U**C****C****G****G****G****C****U**GAG**A**U**C****U****G****G****C**AUA-..................................................................................**G****C****C**.**A****C**...........**G****A****C****C****G****U**C-.........................GA**A**.**C****C****U****G**A.U.**C****C****G****G**.AUAAU...............................G.**C****C****G****G**C**G**AU**A****G****G**.**G**A....**G****G****A****A****A****A****A****U****A****U** | |
|  |  | NC\_004369.1/2042066-2042172  | **C****C****C****A****A****C****C****C****C****C****A**..**C**.**G****G****G**.**C****G****C****C****C****G****G**GGC..........................................................................................................................................................................A**C****C****G****G****G****C****U**GAG**A**.**U****U****G****C****G**CUGC...........................................................................CUUGCCG**C****G****C**.**A****A**..........G**G****A****C****C****G****U**AU.........................GA**A**.**C****C****U****G**-.U.**C****U****G****G**.UUAGC...............................A.**C****C****A****G**C**G**.A**A****G****G**.**A**A....**G****A****G****A****G****G****C****C****U****A** | |
|  |  | NC\_006958.1/1127774-1127876  | **U****U****U****C****A****U****A****G****A****C****A**..**C**.**G****G****G**.**U****G****C****U****C****G****G**UGAAAA.......................................................................................................................................................................U**C****C****G****G****G****C****U**GAG**A**U**C****U****G****G****C**AUA-..................................................................................**G****C****C**.**A****C**...........**G****A****C****C****G****U**C-.........................GA**A**.**C****C****U****G**A.U.**C****C****G****G**.AUAAU...............................G.**C****C****G****G**C**G**AU**A****G****G**.**G**A....**G****G****A****A****A****A****A****U****A****U** | |
|  |  | NC\_006085.1/133010-133097  | **C****C****C****A****U****G****G****A****C****A****C**..**G**.**G****G****G**.**U****G****C****G****C****C****U**CG-..........................................................................................................................................................................G**U****G****G****C****G****C****U**GAG**A**.---------..................................................................................---.--........UCA**C****A****C****C****C****G**UG.........................GA**A**.**C****C****U****G**A.U.**C****U****A****G**.UUCGU...............................A.**C****U****G****G**C**G**.A**A****G****G**.**G**A....**U****G****U****C****C****G****A****G****A****U** | |
|  |  | NC\_007146.1/447771-447852  | **A****A****U****U****U****U****U****A****G****U****C**..**G**.**G****G****G**.**U****G****C****C****G****A**----..........................................................................................................................................................................--**A****A****G****G****C****U**GAG**A**.---------..................................................................................---.--........UGA**U****A****C****C****C****G**U-.........................GA**A**.**C****C****U****G**A.A.**A****C****A****G**.UUAGC...............................A.**C****U****G****A**C**G**.U**A****G****G**.**A**A....**A****C****U****A****A****U****A****U****G****C** | |
|  |  | NC\_007146.1/504664-504745  | **A****A****U****U****U****U****U****A****G****U****C**..**G**.**G****G****G**.**U****G****C****C****G****A**----..........................................................................................................................................................................--**A****A****G****G****C****U**GAG**A**.---------..................................................................................---.--........UGA**U****A****C****C****C****G**U-.........................GA**A**.**C****C****U****G**A.A.**A****C****A****G**.UUAGC...............................A.**C****U****G****A**C**G**.U**A****G****G**.**A**A....**A****C****U****A****A****U****A****U****G****C** | |
|  |  | NZ\_AADO01000002.1/152193-152274  | **A****A****U****U****U****U****U****A****G****U****C**..**G**.**G****G****G**.**U****G****C****C****G****A**----..........................................................................................................................................................................--**A****A****G****G****C****U**GAG**A**.---------..................................................................................---.--........UGA**U****A****C****C****C****G**U-.........................GA**A**.**C****C****U****G**A.A.**A****C****A****G**.UUAGC...............................A.**C****U****G****A**C**G**.U**A****G****G**.**A**A....**A****C****U****A****A****U****A****U****G****C** | |
|  |  | NZ\_AADO01000002.1/209232-209313  | **A****A****U****U****U****U****U****A****G****U****C**..**G**.**G****G****G**.**U****G****C****C****G****A**----..........................................................................................................................................................................--**A****A****G****G****C****U**GAG**A**.---------..................................................................................---.--........UGA**U****A****C****C****C****G**U-.........................GA**A**.**C****C****U****G**A.A.**A****C****A****G**.UUAGC...............................A.**C****U****G****A**C**G**.U**A****G****G**.**A**A....**A****C****U****A****A****U****A****U****G****C** | |
|  |  | NZ\_AADP01000002.1/290569-290650  | **A****A****U****U****U****U****U****A****G****U****C**..**G**.**G****G****G**.**U****G****C****C****G****A**----..........................................................................................................................................................................--**A****A****G****G****C****U**GAG**A**.---------..................................................................................---.--........UGA**U****A****C****C****C****G**U-.........................GA**A**.**C****C****U****G**A.A.**A****C****A****G**.UUAGC...............................A.**C****U****G****A**C**G**.U**A****G****G**.**A**A....**A****C****U****A****A****U****A****U****G****C** | |
|  |  | NZ\_AAET01000009.1/11922-12003  | **A****A****U****U****U****U****U****A****G****U****C**..**G**.**G****G****G**.**U****G****C****C****G****A**----..........................................................................................................................................................................--**A****A****G****G****C****U**GAG**A**.---------..................................................................................---.--........UGA**U****A****C****C****C****G**U-.........................GA**A**.**C****C****U****G**A.A.**A****C****A****G**.UUAGC...............................A.**C****U****G****A**C**G**.U**A****G****G**.**A**A....**A****C****U****A****A****U****A****U****G****C** | |
|  |  | NZ\_AAET01000129.1/2519-2438  | **A****A****U****U****U****U****U****A****G****U****C**..**G**.**G****G****G**.**U****G****C****C****G****A**----..........................................................................................................................................................................--**A****A****G****G****C****U**GAG**A**.---------..................................................................................---.--........UGA**U****A****C****C****C****G**U-.........................GA**A**.**C****C****U****G**A.A.**A****C****A****G**.UUAGC...............................A.**C****U****G****A**C**G**.U**A****G****G**.**A**A....**A****C****U****A****A****U****A****U****G****C** | |
|  |  | NZ\_AADP01000002.1/232211-232292  | **A****A****C****U****U****U****U****A****G****U****C**..**G**.**G****G****G**.**U****G****C****C****G****A**----..........................................................................................................................................................................--**A****A****G****G****C****U**GAG**A**.---------..................................................................................---.--........UGA**U****A****C****C****C****G**U-.........................GA**A**.**C****C****U****G**A.A.**A****C****A****G**.UUAGC...............................A.**C****U****G****A**C**G**.U**A****G****G**.**A**A....**A****C****U****A****A****U****A****U****G****C** | |
|  |  | NC\_000964.2/3178144-3178238  | **G****A****U****G****A****C****C****A****C****A****A**..**G**.**G****G****G**.**A****G****C****A****U****U**----..........................................................................................................................................................................--**A****A****A****G****C****U**GAG**A**G**U****G****A****G****C**GGUU................................................................................UC**G****U****U**.**C****U**...........**G****A****C****C****C****U**UU.........................GA**A**.**C****C****U****G**-.U.-**U****A****G**.UUAAC...............................G.**C****U****G****G**C**G**.U**A****G****G**.**G**A....**U****G****U****G****G****C****A****A****A****G** | |
|  |  | NZ\_AAIJ01000033.1/7510-7598  | **A****A****A****U****C****A****U****C****U****U****A**..**G**.**G****G****G**.**U****G****C****U****C****C****G**CAC..........................................................................................................................................................................A**A****G****G****G****G****C****U**GAG**A**.---------..................................................................................---.--........UUA**A****A****C****C****C****U**UG.........................AC**A**.**C****U****U****G**A.U.**G****C****A****G**.GUAAU...............................G.**C****U****G****A**C**G**.C**A****A****G**.**G**A....**A****A****G****A****U****G****A****A****G****C** | |
|  |  | NZ\_AAGG01000003.1/256001-256102  | **U****G****C****C****C****A****U****C****C****C****C**..**G**.**G****G****G**.**A****G****C****C****A****G**----..........................................................................................................................................................................-**A****C****C****G****G****C****U**GAG**A**G**G****G****G****U****G**GGAU............................................................................AGUCUC**C****A****C**.**C****C**...........**G****A****C****C****C****G**UU.........................GA**A**.**C****C****U****G**A.U.**C****C****C****G**.UUAGC...............................G.**C****G****G****G**C**G**.G**A****G****G**.**G**A....**A****G****G****G****U****G****G****U****C****G** | |
|  |  | NC\_005877.1/1024953-1025050  | **A****U****A****U****G****U****C****G****A****C****U**..**G**.**G****G****G**.**A****G****C****C****G****A**----..........................................................................................................................................................................-**A****A****A****G****G****C****U**GAG**A**G**G****A****U****C****U**AAAA..................................................................................**A****G****A**.**U****C**...........**G****A****C****C****C****U**UU.........................GA**A**.**C****C****U****G**A.U.**C****C****G****G**.GUAAU...............................A.**C****C****G****G**C**G**.G**A****G****G**.**G**A..UU**U****U****A****G****U****G****G****A****A****U** | |
|  |  | NC\_004567.1/99249-99347  | **U****U****U****A****A****A****C****A****C****U****A**..**G**.**G****G****G**.**U****G****U****C****C****A****A**AA-..........................................................................................................................................................................-**A****U****G****G****G****C****U**GAG**A**.**U****G****G****U****G**CUGU...............................................................................AAG**U****A****C**.**C****G**...........**A****U****C****C****C****U**UU.........................GA**A**.**C****C****U****G**-.U.-**A****A****G**.CUCAA...............................A.**C****U****U****G**C**G**.U**A****G****G**.**A**A....**A****G****U****G****U****C****A****C****A****G** | |
|  |  | NZ\_AABH02000033.1/15589-15689  | **U****U****A****A****C****A****U****A****C****U****A**..**G**.**G****G****G**.**U****G****C****U****U****U****U**AA-..........................................................................................................................................................................-**A****A****A****A****G****C****U**GAG**A**.**G****A****G****A****A**GAGA................................................................................UA**A****U****C**.**U****U**.........UG**A****A****C****C****C****U**UU.........................GA**A**.**C****C****U****G**A.U.**C****U****G**-.-UAGC...............................AA**G****C****A****G**C**G**.U**A****G****G**.**A**A....**A****G****U****A****U****G****A****A****U****A** | |
|  |  | NC\_005085.1/243048-243188  | **C****G****G****C****A****U****C****G****C****U****A**..**G**.**G****G****G**.**U****C****C****U****G****C****G**CCUGGCGUUCGGCGCAGAUGGCUAGCAAAGUGUAGGCCAUGGCGGCGGAUGACGGA.....................................................................................................................G**C****G****C****G****G****G****U**GAG**A**.---------..................................................................................---.--.........AA**U****A****C****C****C****U**UG.........................GA**A**.**C****C****U****G**A.C.**C****C****G****G**.AUAAU...............................A.**C****C****G****G**C**G**.U**A****G****G**.**G**A....**A****G****C****G****U****C****A****C****C****U** | |
|  |  | NC\_003909.8/828777-828882  | **A****U****A****G****U****U****U****G****C****U****A**..**G**.**G****A****G**.**A****G****C****U****G****G****U**GU-..........................................................................................................................................................................U**G****C****C****A****G****C****U**GAG**A**G**U****A****A****G****G**CCUU...............................................................................AAG**U****C****U**.**U****U**...........**G****A****U****C****C****U**UUUUA......................UU**A**.**C****C****U****G**A.U.**C****U****A****G**.AUUAU...............................G.**C****U****A****G**C**G**.U**A****G****G**.**G**A....**A****G****C****A****A****U****U****C****G****G** | |
|  |  | NZ\_AABH02000005.1/43062-42980  | **C****A****C****C****A****A****A****A****U****C****U**..**G**.**G****G****G**.**U****G****C**-**C****G**----..........................................................................................................................................................................-**U****C****G****C****G****C****U**GAG**A**.---------..................................................................................---.--........UAA**A****A****C****C****C****A**UC.........................AC**A**.**C****C****U****G**A.U.**C****U****A****G**.UUAAU...............................A.**C****U****A****G**C**G**.U**A****G****G**.**G**A....**G****A****U****U****U****A****C****U****U****G** | |
|  |  | NZ\_AAAV02000002.1/122459-122360  | **U****C****U****C****A****U****A****C****C****C****C**..**G**.**G****G****G**.**A****G****C****C****A****G**----..........................................................................................................................................................................-**U****G****U****G****G****C****U**GAG**A**G**G****G****G****C****U**GGUC..............................................................................ACGC**A****G****C**.**C****C**...........**G****A****C****C****C****G**UU.........................GA**A**.**C****C****U****G**A.A.**C****C****C****G**.UUAGC...............................A.**C****G****G****G**C**G**.G**A****G****G**.**G**A....**A****G****G****G****C****A****G****U****G****C** | |
|  |  | NZ\_AAEV01000013.1/25-129  | **A****U****U****U****U****G****U****A****A****C****A**..**G**.**G****G****G**.**U****G****C****U****G****G****G**CUUU.........................................................................................................................................................................A**C****C****U****G****G****C****U**GAG**A**U**U****A****A****A****A**CUAA..............................................................................AUGG**U****U****U**.**U****U**...........**G****A****C****C****C****U**C-.........................GA**A**.**C****C****U****G**A.U.**A****U****G****G**.AUAAU...............................G.**C****C****A****G**C**G**.C**A****G****G**.**A**A....**G****A****C****U****C****A****A****U****U****G** | |
|  |  | NC\_006085.1/962710-962816  | **A****G****A****C****A****A****C****G****A****C****G**..**C**.**G****G****G**.**A****G****C****C****C****A****C**UGGUCGC......................................................................................................................................................................U**G****G****G****G****G****C****U**GAG**A**G**G****G****C****G****C**CACG...............................................................................AAG**G****U****G**.**C****C**...........**G****A****C****C****G****C**CG.........................GA**A**.**C****C****U****G**-.U.**C****C****G****G**.GUCAU...............................G.**C****C****G****G**C**G**.A**A****G****G**.**G**A....**G****U****G****G****U****U****G****A****C****A** | |
|  |  | NC\_006814.1/563420-563503  | **A****U****A****A****G****U****C****A****C****U****A**..**G**.**G****G****G**.**U****G****C****U****G****A**----..........................................................................................................................................................................--**A****A****A****G****C****U**GAG**A**.---------..................................................................................---.--.........UA**U****A****C****C****C****U**UC.........................GA**A**.**C****U****U****G**A.A.**C****A****A****G**.GUAAU...............................G.**C****U****U****G**C**G**AU**A****A****G**.**G**A...A**A****G****U****G****C****U****A****A****U****U** | |
|  |  | NC\_002936.3/713568-713480  | **U****A****A****A****C****U****C****G****C****U****A**..**G**.**G****G****G**.**U****G****C****U****C**-----..........................................................................................................................................................................--**C****A****G****G****C****U**GAG**A**.-**G****G****C****A**CAUA..................................................................................-**G****C**.**C**-...........**A****A****C****C****C****U**A-.........................AA**A**.**C****C****U****G**A.A.**C****U****C****G**.GUAAU...............................G.**C****G****A****G**C**G**.U**A****G****G**.**G**A....**A****G****C****G****A****A****C****U****U****U** | |
|  |  | NC\_000907.1/380481-380562  | **A****G****U****U****U****U****U****A****G****U****C**..**G**.**G****G****G**.**U****G****C****C****G****A**----..........................................................................................................................................................................--**A****A****G****G****C****U**GAG**A**.---------..................................................................................---.--........UGA**U****A****C****C****C****G**U-.........................GA**A**.**C****C****U****G**A.A.**A****C****A****G**.UUAGC...............................A.**C****U****G****A**C**G**.U**A****G****G**.**A**A....**A****C****U****A****A****U****A****U****G****C** | |
|  |  | NC\_000913.2/75610-75518  | **C****C****G****U****U****C****U****C****A****A****C**..**G**.**G****G****G**.**U****G****C**-**C****A****C**GCGUACG......................................................................................................................................................................C**G****U****G****C****G****C****U**GAG**A**.---------..................................................................................---.--........AAA**U****A****C****C****C****G**UC.........................GA**A**.**C****C****U****G**A.U.**C****C****G****G**.AUAAC...............................G.**C****C****G****G**C**G**.A**A****G****G**.**G**A...U**U****U****G****A****G****G****C****U****C****C** | |
|  |  | NC\_002655.2/80430-80338  | **C****C****G****U****U****C****U****C****A****A****C**..**G**.**G****G****G**.**U****G****C**-**C****A****C**GCGGACG......................................................................................................................................................................C**G****U****G****C****G****C****U**GAG**A**.---------..................................................................................---.--........AAA**U****A****C****C****C****G**UC.........................GA**A**.**C****C****U****G**A.U.**C****C****G****G**.AUAAC...............................G.**C****C****G****G**C**G**.A**A****G****G**.**G**A...U**U****U****G****A****G****G****C****U****C****C** | |
|  |  | NC\_002695.1/80429-80337  | **C****C****G****U****U****C****U****C****A****A****C**..**G**.**G****G****G**.**U****G****C**-**C****A****C**GCGGACG......................................................................................................................................................................C**G****U****G****C****G****C****U**GAG**A**.---------..................................................................................---.--........AAA**U****A****C****C****C****G**UC.........................GA**A**.**C****C****U****G**A.U.**C****C****G****G**.AUAAC...............................G.**C****C****G****G**C**G**.A**A****G****G**.**G**A...U**U****U****G****A****G****G****C****U****C****C** | |
|  |  | NC\_002935.2/922838-922962  | **U****U****U****A****U****A****A****A****U****C****A**..**C**.**G****G****G**.**U****G****C****U****G****G****A**CGGCAUACGUUUGCC..............................................................................................................................................................A**C****A****A****A****G****C****U**GAG**A**.**C****A****G****G****G**CGAG.....................................................................AAGACGUGCACGU**C****C****C**.**U****G**...........**A****A****C****C****G****U**U-.........................GA**A**.**C****C****U****G**A.U.**C****C****G****G**.GUAAU...............................A.**C****C****G****G**C**G**AU**A****G****G**.**A**A....**G****A****A****U****A****A****U****G****A****A** | |
|  |  | NC\_002940.2/462086-462171  | **U****U****G****U****U****C****U****C****A****U****U**..**G**.**G****G****G**.**U****G****C****U****G****A****A**A--..........................................................................................................................................................................-**A****U****A****A****G****C****U**GAG**A**.---------..................................................................................---.--.........AA**U****A****C****C****C****A**UA.........................GA**A**.**C****C****U****G**A.U.**C****U****G****A**.UUCGU...............................A.**U****C****A****G**C**G**.U**A****G****G**.**G**A...U**U****U****G****A****G****G****C****C****A****U** | |
|  |  | NC\_002973.5/353205-353303  | **A****A****G****A****A****A****A****A****C****U****A**..**G**.**G****G****G**.**G****G****C****C****G****A****U**---..........................................................................................................................................................................-**U****C****U****G****G****C****U**GAG**A**U**A****G****G****A****A**GGUA..............................................................................AUGC**U****U****U**.**C****U**...........**G****A****C****C****C****U**UU.........................GA**A**.**C****C****U****G**-.U.-**U****U****G**.UUAGU...............................G.**C****A****A****G**C**G**.U**A****G****G**.**G**A....**A****G****U****G****A****A****U****G****U****G** | |
|  |  | NC\_003210.1/340086-340184  | **A****A****G****A****A****A****A****A****C****U****A**..**G**.**G****G****G**.**G****G****C****C****G****A****U**---..........................................................................................................................................................................-**U****C****U****G****G****C****U**GAG**A**U**A****G****G****A****A**GGUA..............................................................................AUGC**U****U****U**.**C****U**...........**G****A****C****C****C****U**UU.........................GA**A**.**C****C****U****G**-.U.-**U****U****G**.UUAGU...............................G.**C****A****A****G**C**G**.U**A****G****G**.**G**A....**A****G****U****G****A****A****U****G****U****G** | |
|  |  | NC\_003212.1/354876-354974  | **A****A****G****A****A****A****A****A****C****U****A**..**G**.**G****G****G**.**G****G****C****C****G****A****U**---..........................................................................................................................................................................-**U****C****U****G****G****C****U**GAG**A**U**A****G****G****A****A**GGUA..............................................................................AUGC**U****U****U**.**C****U**...........**G****A****C****C****C****U**UU.........................GA**A**.**C****C****U****G**-.U.-**U****U****G**.UUAGU...............................G.**C****A****A****G**C**G**.U**A****G****G**.**G**A....**A****G****U****G****A****A****U****G****U****G** | |
|  |  | NZ\_AAAK03000012.1/14294-14389  | **A****A****A****C****U****C****A****C****A****A****A**..**G**.**G****G****G**.**A****G****U****C****C****A****A**---..........................................................................................................................................................................-**U****U****G****G****G****C****U**GAG**A**.**U****U****G****A****A**UCUA.................................................................................U**U****U****C**.**U****A**...........**A****A****C****C****C****U**UC.........................GU**A**.**C****C****U****G**-.U.**A****U****C****G**.GUUAU...............................G.**C****G****A****G**C**G**.U**A****G****G**.**A**A....**U****U****G****U****G****A****A****U****A****A** | |
|  |  | NZ\_AADQ01000022.1/1288-1386  | **A****A****G****A****A****A****A****A****C****U****A**..**G**.**G****G****G**.**G****G****C****C****G****A****U**---..........................................................................................................................................................................-**U****C****U****G****G****C****U**GAG**A**U**A****G****G****A****A**GGUA..............................................................................AUGC**U****U****U**.**C****U**...........**G****A****C****C****C****U**UU.........................GA**A**.**C****C****U****G**-.U.-**U****U****G**.UUAGU...............................G.**C****A****A****G**C**G**.U**A****G****G**.**G**A....**A****G****U****G****A****A****U****G****U****G** | |
|  |  | NZ\_AADR01000001.1/100039-99941  | **A****A****G****A****A****A****A****A****C****U****A**..**G**.**G****G****G**.**G****G****C****C****G****A****U**---..........................................................................................................................................................................-**U****C****U****G****G****C****U**GAG**A**U**A****G****G****A****A**GGUA..............................................................................AUGC**U****U****U**.**C****U**...........**G****A****C****C****C****U**UU.........................GA**A**.**C****C****U****G**-.U.-**U****U****G**.UUAGU...............................G.**C****A****A****G**C**G**.U**A****G****G**.**G**A....**A****G****U****G****A****A****U****G****U****G** | |
|  |  | NC\_000907.1/437548-437629  | **G****A****C****U****U****U****U****A****G****U****C**..**G**.**G****G****G**.**U****G****C****C****G****A**----..........................................................................................................................................................................--**A****A****G****G****C****U**GAG**A**.---------..................................................................................---.--........UGA**U****A****C****C****C****G**U-.........................GA**A**.**C****C****U****G**A.A.**A****C****A****G**.UUAGC...............................A.**C****U****G****A**C**G**.U**A****G****G**.**A**A....**A****C****U****A****A****U****A****U****G****C** | |
|  |  | NZ\_AAAJ03000001.1/436731-436842  | **C****U****G****C****U****A****A****C****G****C****G**..**G**.**G****G****G**.**U****C****C****U****G****C****G**UCGCACGGAGGUUGGAGGUCCGUGUU...................................................................................................................................................G**C****G****U****G****G****G****U**GAG**A**.---------..................................................................................---.--.........AA**U****A****C****C****C****U**UU.........................GA**A**.**C****C****U****G**A.U.**C****U****G****G**.AUAAU...............................G.**C****C****A****G**C**G**.C**A****G****G**.**G**A...A**G****C****G****U****A****C****G****G****A****U** | |
|  |  | NZ\_AAAW03000022.1/37348-37249  | **A****G****A****A****U****A****G****U****A****U****U**..**G**.**G****G****G**.**A****G****C****U****G****G****A**A--..........................................................................................................................................................................-**A****A****C****G****G****C****U**GAG**A**G**G****G****A****A****A**CGAG................................................................................UG**U****U****U**.**C****C**...........**G****A****C****C****C****A**U-.........................UG**A**.**C****C****U****G**A.U.**C****U****A****G**.GUAAU...............................G.**C****U****A****G**C**G**.G**A****G****G**.**G**A...C**U****U****A****G****G****A****U****A****C****A** | |
|  |  | NC\_002935.2/27456-27558  | **A****G****U****G****U****C****C****C****C****C****G**..**C**.**G****G****G**.**C****G****C****C****C****G****A**GC-..........................................................................................................................................................................-**A****C****G****G****G****C****U**GAG**A**.**U****U****G****C****G**CUGA.............................................................................UGCUG**U****G****C**.**A****A**..........G**C****A****C****C****G****U**UU.........................GA**A**.**C****C****U****G**-.U.**C****U****G****G**.UUAAC...............................A.**C****C****A****G**C**G**.A**A****G****G**.**A**A....**G****A****G****A****G****G****A****G****C****G** | |
|  |  | NZ\_AADF01000001.1/790769-790680  | **C****C****C****A****C****U****C****G****U****U****A**..**G**.**G****G****G**.**U****G****C****C****A****A****C**CGCAAA.......................................................................................................................................................................A**C****U****U****G****G****C****U**GAG**A**.---------..................................................................................---.--.........AU**U****A****C****C****C****U**UC.........................GA**A**.**C****C****U****G**A.C.-**C****G****G**.GUCAU...............................G.**C****C****G****U**C**G**.U**A****G****G**.**G**A....**A****A****C****G****A****A****C****U****C****U** | |
|  |  | NC\_006085.1/574374-574270  | **A****A****A****G****G****U****U****U****C****C****G**..**C**.**G****G****G**.**A****G****C****C****G****C****A**CAGAACAGG....................................................................................................................................................................A**U****G****C****G****G****C****U**GAG**A**G**G****G****C****A****U**AUUC..................................................................................--**G**.**C****C**...........**G****A****C****C****G****C**UU.........................UC**A**.**C****C****U****G**A.U.**G****C****G****G**.GUCAU...............................G.**C****C****G****C**C**G**.G**A****G****G**.**G**A....**G****G****A****C****A****U****G****C****U****G** | |
|  |  | NZ\_AAHI01000013.1/143883-143774  | **U****U****G****C****U****A****A****C****G****C****G**..**G**.**G****G****G**.**U****C****C****U****G****C****A**AUGCGUUCGGUCAAACAACCGGCA.....................................................................................................................................................U**U****G****C****G****G****G****U**GAG**A**.---------..................................................................................---.--.........AA**U****A****C****C****C****U**UU.........................GA**A**.**C****C****U****G**A.U.**C****U****G****G**.AUAAU...............................G.**C****C****A****G**C**G**.C**A****G****G**.**G**A...A**G****C****G****U****A****C****G****G****A****U** | |
|  |  | NZ\_AAHL01000040.1/51293-51402  | **U****U****G****C****U****A****A****C****G****C****G**..**G**.**G****G****G**.**U****C****C****U****G****C****A**AUGCGUUCGGUCAAACAACCGGCA.....................................................................................................................................................U**U****G****C****G****G****G****U**GAG**A**.---------..................................................................................---.--.........AA**U****A****C****C****C****U**UU.........................GA**A**.**C****C****U****G**A.U.**C****U****G****G**.AUAAU...............................G.**C****C****A****G**C**G**.C**A****G****G**.**G**A...A**G****C****G****U****A****C****G****G****A****U** | |
|  |  | NC\_003295.1/132458-132364  | **U****G****G****A****U****G****A****A****A****C****A**..**G**.**G****G****G**.**U****G****C****C****G****U****G**CGGAUGGGCC...................................................................................................................................................................G**C****G****C****G****G****C****U**GAG**A**.---------..................................................................................---.--.........GA**G****U****C****C****C****U**UC.........................GC**A**.**C****C****C****G**A.U.**C****C****G****G**.UUCGU...............................A.**C****C****G****G**C**G**.U**G****G****G**.**A**A....**G****U****U****U****C****U****U****C****A****G** | |
|  |  | NC\_003909.8/4579444-4579538  | **C****G****U****U****A****U****C****A****C****A****G**..**G**.**G****G****G**.**A****G****C****C****A****U**----..........................................................................................................................................................................--**G****C****C****G****C****U**GAG**A**G**G****G****A****A****C**ACUU.................................................................................C**G****U****U**.**C****C**...........**G****A****C****C****C****U**UC.........................GA**A**.**C****C****U****G**-.-.**U****U****A****G**.UUAAU...............................G.**C****U****A****A**C**G**.C**A****G****G**.**G**A...U**U****G****U****G****C****A****A****A****C****G** | |
|  |  | NC\_003997.3/4589425-4589519  | **C****G****U****U****A****U****C****A****C****A****G**..**G**.**G****G****G**.**A****G****C****C****A****U**----..........................................................................................................................................................................--**G****C****C****G****C****U**GAG**A**G**G****G****A****A****C**ACUU.................................................................................C**G****U****U**.**C****C**...........**G****A****C****C****C****U**UC.........................GA**A**.**C****C****U****G**-.-.**U****U****A****G**.UUAAU...............................G.**C****U****A****A**C**G**.C**A****G****G**.**G**A...U**U****G****U****G****C****A****A****A****C****G** | |
|  |  | NC\_005945.1/4590648-4590742  | **C****G****U****U****A****U****C****A****C****A****G**..**G**.**G****G****G**.**A****G****C****C****A****U**----..........................................................................................................................................................................--**G****C****C****G****C****U**GAG**A**G**G****G****A****A****C**ACUU.................................................................................C**G****U****U**.**C****C**...........**G****A****C****C****C****U**UC.........................GA**A**.**C****C****U****G**-.-.**U****U****A****G**.UUAAU...............................G.**C****U****A****A**C**G**.C**A****G****G**.**G**A...U**U****G****U****G****C****A****A****A****C****G** | |
|  |  | NC\_005957.1/4590278-4590372  | **C****G****U****U****A****U****C****A****C****A****G**..**G**.**G****G****G**.**A****G****C****C****A****U**----..........................................................................................................................................................................--**G****C****C****G****C****U**GAG**A**G**G****G****A****A****C**ACUU.................................................................................C**G****U****U**.**C****C**...........**G****A****C****C****C****U**UC.........................GA**A**.**C****C****U****G**-.-.**U****U****A****G**.UUAAU...............................G.**C****U****A****A**C**G**.C**A****G****G**.**G**A...U**U****G****U****G****C****A****A****A****C****G** | |
|  |  | NC\_007530.2/4589552-4589646  | **C****G****U****U****A****U****C****A****C****A****G**..**G**.**G****G****G**.**A****G****C****C****A****U**----..........................................................................................................................................................................--**G****C****C****G****C****U**GAG**A**G**G****G****A****A****C**ACUU.................................................................................C**G****U****U**.**C****C**...........**G****A****C****C****C****U**UC.........................GA**A**.**C****C****U****G**-.-.**U****U****A****G**.UUAAU...............................G.**C****U****A****A**C**G**.C**A****G****G**.**G**A...U**U****G****U****G****C****A****A****A****C****G** | |
|  |  | NZ\_AAAC02000001.1/5032772-5032866  | **C****G****U****U****A****U****C****A****C****A****G**..**G**.**G****G****G**.**A****G****C****C****A****U**----..........................................................................................................................................................................--**G****C****C****G****C****U**GAG**A**G**G****G****A****A****C**ACUU.................................................................................C**G****U****U**.**C****C**...........**G****A****C****C****C****U**UC.........................GA**A**.**C****C****U****G**-.-.**U****U****A****G**.UUAAU...............................G.**C****U****A****A**C**G**.C**A****G****G**.**G**A...U**U****G****U****G****C****A****A****A****C****G** | |
|  |  | NZ\_AAEN01000027.1/201119-201213  | **C****G****U****U****A****U****C****A****C****A****G**..**G**.**G****G****G**.**A****G****C****C****A****U**----..........................................................................................................................................................................--**G****C****C****G****C****U**GAG**A**G**G****G****A****A****C**ACUU.................................................................................C**G****U****U**.**C****C**...........**G****A****C****C****C****U**UC.........................GA**A**.**C****C****U****G**-.-.**U****U****A****G**.UUAAU...............................G.**C****U****A****A**C**G**.C**A****G****G**.**G**A...U**U****G****U****G****C****A****A****A****C****G** | |
|  |  | NZ\_AAEO01000031.1/200753-200847  | **C****G****U****U****A****U****C****A****C****A****G**..**G**.**G****G****G**.**A****G****C****C****A****U**----..........................................................................................................................................................................--**G****C****C****G****C****U**GAG**A**G**G****G****A****A****C**ACUU.................................................................................C**G****U****U**.**C****C**...........**G****A****C****C****C****U**UC.........................GA**A**.**C****C****U****G**-.-.**U****U****A****G**.UUAAU...............................G.**C****U****A****A**C**G**.C**A****G****G**.**G**A...U**U****G****U****G****C****A****A****A****C****G** | |
|  |  | NZ\_AAEP01000032.1/62456-62362  | **C****G****U****U****A****U****C****A****C****A****G**..**G**.**G****G****G**.**A****G****C****C****A****U**----..........................................................................................................................................................................--**G****C****C****G****C****U**GAG**A**G**G****G****A****A****C**ACUU.................................................................................C**G****U****U**.**C****C**...........**G****A****C****C****C****U**UC.........................GA**A**.**C****C****U****G**-.-.**U****U****A****G**.UUAAU...............................G.**C****U****A****A**C**G**.C**A****G****G**.**G**A...U**U****G****U****G****C****A****A****A****C****G** | |
|  |  | NZ\_AAEQ01000031.1/200575-200669  | **C****G****U****U****A****U****C****A****C****A****G**..**G**.**G****G****G**.**A****G****C****C****A****U**----..........................................................................................................................................................................--**G****C****C****G****C****U**GAG**A**G**G****G****A****A****C**ACUU.................................................................................C**G****U****U**.**C****C**...........**G****A****C****C****C****U**UC.........................GA**A**.**C****C****U****G**-.-.**U****U****A****G**.UUAAU...............................G.**C****U****A****A**C**G**.C**A****G****G**.**G**A...U**U****G****U****G****C****A****A****A****C****G** | |
|  |  | NZ\_AAER01000035.1/1103594-1103688  | **C****G****U****U****A****U****C****A****C****A****G**..**G**.**G****G****G**.**A****G****C****C****A****U**----..........................................................................................................................................................................--**G****C****C****G****C****U**GAG**A**G**G****G****A****A****C**ACUU.................................................................................C**G****U****U**.**C****C**...........**G****A****C****C****C****U**UC.........................GA**A**.**C****C****U****G**-.-.**U****U****A****G**.UUAAU...............................G.**C****U****A****A**C**G**.C**A****G****G**.**G**A...U**U****G****U****G****C****A****A****A****C****G** | |
|  |  | NC\_002937.3/2184704-2184611  | **G****A****C****C****G****G****A****G****C****U****A**..**G**.**G****G****G**.**A****G****C****C****U****U**----..........................................................................................................................................................................--**C****G****G****G****C****U**GAG**A**G**U****G****G****G****U**UCGU..................................................................................-**C****C**.**C****A**...........**G****A****C****C****C****U**GU.........................GA**A**.**C****C****U****G**A.C.**G****C****A****G**.UUAGC...............................A.**C****U****G****C**C**G**.U**A****G****G**.**G**A....**A****G****C****C****G****C****G****C****A****C** | |
|  |  | NZ\_AAEH02000046.1/12413-12522  | **C****U****G****C****U****A****A****C****G****C****G**..**G**.**G****G****G**.**U****C****C****U****G****C****A**AUGCGCCCGGUCAAACAACCGGCA.....................................................................................................................................................U**U****G****C****G****G****G****U**GAG**A**.---------..................................................................................---.--.........AA**U****A****C****C****C****U**UU.........................GA**A**.**C****C****U****G**A.U.**C****U****G****G**.AUAAU...............................G.**C****C****A****G**C**G**.C**A****G****G**.**G**A...A**G****C****G****U****A****C****G****G****A****U** | |
|  |  | NZ\_AAEI01000002.1/246834-246943  | **C****U****G****C****U****A****A****C****G****C****G**..**G**.**G****G****G**.**U****C****C****U****G****C****A**AUGCGUUCGGUCAAACAACCGGCA.....................................................................................................................................................U**U****G****C****G****G****G****U**GAG**A**.---------..................................................................................---.--.........AA**U****A****C****C****C****U**UU.........................GA**A**.**C****C****U****G**A.U.**C****U****G****G**.AUAAU...............................G.**C****C****A****G**C**G**.C**A****G****G**.**G**A...A**G****C****G****U****A****C****G****G****A****U** | |
|  |  | NZ\_AAEK01000023.1/6859-6953  | **C****G****U****U****A****U****C****A****C****U****G**..**G**.**G****G****G**.**A****G****C****C****A****U**----..........................................................................................................................................................................--**G****C****C****G****C****U**GAG**A**G**G****G****A****A****C**ACUU.................................................................................C**G****U****U**.**C****C**...........**G****A****C****C****C****U**UC.........................GA**A**.**C****C****U****G**-.-.**U****U****A****G**.UUAAU...............................A.**C****U****A****A**C**G**.C**A****G****G**.**G**A...U**U****G****U****G****C****A****A****A****C****G** | |
|  |  | NC\_004307.2/141008-140907  | **A****C****U****G****U****A****A****U****G****C****A**..**G**.**G****G****G**.**A****A****C****U****C****G****C**AUGUGGUU.....................................................................................................................................................................C**G****C****G****A****G****U****U**GAG**A**.**A****A****G****G****C**CUGA..................................................................................**G****C****C**.**U**-...........**G****A****C****C****C****U**UA.........................GA**A**.**C****C****U****G**-.U.-**U****G****G**.UUAAG...............................A.**C****C****A****U**C**G**.U**A****G****G**.**G**A....**G****C****A****G****U****A****A****A****U****G** | |
|  |  | NZ\_AABM02000032.1/6293-6394  | **A****C****U****G****U****A****A****U****G****C****A**..**G**.**G****G****G**.**A****A****C****U****C****G****C**AUGUGGUU.....................................................................................................................................................................C**G****C****G****A****G****U****U**GAG**A**.**A****A****G****G****C**CUGA..................................................................................**G****C****C**.**U**-...........**G****A****C****C****C****U**UA.........................GA**A**.**C****C****U****G**-.U.-**U****G****G**.UUAAG...............................A.**C****C****A****U**C**G**.U**A****G****G**.**G**A....**G****C****A****G****U****A****A****A****U****G** | |
|  |  | NC\_003212.1/1469336-1469233  | **C****G****U****U****A****C****C****A****C****A****G**..**G**.**G****G****G**.**G****G****C****U****U**-----..........................................................................................................................................................................C-**U****U****A****G****C****U**GAG**A**U**U****G****A****A****U**CCGC........................................................................GUGUUUUUGG**A****U****U**.**C****U**...........**G****A****C****C****C****U**UU.........................GA**A**.**C****C****U****G**-.U.-**U****C****G**.UUAAU...............................A.**C****G****A****G**C**G**.U**A****G****G**.**G**A...U**U****G****U****G****G****C****G****A****U****U** | |
|  |  | NC\_006369.1/2751384-2751288  | **G****U****C****C****A****G****C****A****U****U****A**..**G**.**G****G****G**.**U****G****C****C****A****A****U**UGAUA........................................................................................................................................................................A**G****U****U****G****G****C****U**GAG**A**.--**G****G****A**UCAC..................................................................................-**C****C**.--...........**A****A****C****C****C****U**UU.........................GA**A**.**C****C****U****G**A.U.**C****U****C****A**.UUAAU...............................C.**U****G****A****G**C**G**.G**A****G****G**.**A**A....**A****A****U****G****U****G****U****C****A****A** | |
|  |  | NC\_002950.2/1999857-1999751  | **A****A****U****U****G****G****G****A****G****A****A**..**G**.**G****G****G**.**U****G****C****U****U****C****C**UGUAGCAAUCGGAAUGCGU..........................................................................................................................................................G**G****A****U****G****G****C****U**GAG**A**.---------..................................................................................---.--........ACA**A****A****C****C****C****U**CA.........................UC**A**.**C****C****U****G**A.A.**C****C****G****G**.AUAAU...............................A.**C****C****G****G**C**G**.U**A****G****G**.**A**A..AC**U****C****U****C****C****G****U****C****U****G** | |
|  |  | NZ\_AABH02000002.1/41403-41321  | **A****U****A****U****U****U****U****A****U****C****U**..**G**.**G****G****G**.**U****A****C****C****G****U**----..........................................................................................................................................................................--**A****A****G****G****U****U**GAG**A**.---------..................................................................................---.--........UAA**U****A****C****C****C****A**UU.........................GA**A**.**C****C****U****G**-.U.**C****A****G****G**.UUAAU...............................A.**C****C****U****G**C**G**.A**A****G****G**.**A**A...G**A****A****U****A****A****G****U****U****A****G** | |
|  |  | NC\_006348.1/3183744-3183854  | **U****U****G****C****U****A****A****C****G****C****G**..**G**.**G****G****G**.**U****C****C****U****G****C****A**AUGCGCUCGGUCAAACGAUCGUGCG....................................................................................................................................................C**A****G****C****G****G****G****U**GAG**A**.---------..................................................................................---.--.........AA**U****A****C****C****C****U**UU.........................GA**A**.**C****C****U****G**A.U.**C****U****G****G**.AUAAU...............................G.**C****C****A****G**C**G**.C**A****G****G**.**G**A...A**G****C****G****U****C****A****G****G****G****U** | |
|  |  | NC\_006350.1/1504234-1504124  | **U****U****G****C****U****A****A****C****G****C****G**..**G**.**G****G****G**.**U****C****C****U****G****C****A**AUGCGCUCGGUCAAACGAUCGUGCG....................................................................................................................................................C**A****G****C****G****G****G****U**GAG**A**.---------..................................................................................---.--.........AA**U****A****C****C****C****U**UU.........................GA**A**.**C****C****U****G**A.U.**C****U****G****G**.AUAAU...............................G.**C****C****A****G**C**G**.C**A****G****G**.**G**A...A**G****C****G****U****C****A****G****G****G****U** | |
|  |  | NZ\_AAHM01000002.1/231434-231324  | **U****U****G****C****U****A****A****C****G****C****G**..**G**.**G****G****G**.**U****C****C****U****G****C****A**AUGCGCUCGGUCAAACGAUCGUGCG....................................................................................................................................................C**A****G****C****G****G****G****U**GAG**A**.---------..................................................................................---.--.........AA**U****A****C****C****C****U**UU.........................GA**A**.**C****C****U****G**A.U.**C****U****G****G**.AUAAU...............................G.**C****C****A****G**C**G**.C**A****G****G**.**G**A...A**G****C****G****U****C****A****G****G****G****U** | |
|  |  | NZ\_AAHN01000013.1/82526-82416  | **U****U****G****C****U****A****A****C****G****C****G**..**G**.**G****G****G**.**U****C****C****U****G****C****A**AUGCGCUCGGUCAAACGAUCGUGCG....................................................................................................................................................C**A****G****C****G****G****G****U**GAG**A**.---------..................................................................................---.--.........AA**U****A****C****C****C****U**UU.........................GA**A**.**C****C****U****G**A.U.**C****U****G****G**.AUAAU...............................G.**C****C****A****G**C**G**.C**A****G****G**.**G**A...A**G****C****G****U****C****A****G****G****G****U** | |
|  |  | NZ\_AAHO01000004.1/47444-47554  | **U****U****G****C****U****A****A****C****G****C****G**..**G**.**G****G****G**.**U****C****C****U****G****C****A**AUGCGCUCGGUCAAACGAUCGUGCG....................................................................................................................................................C**A****G****C****G****G****G****U**GAG**A**.---------..................................................................................---.--.........AA**U****A****C****C****C****U**UU.........................GA**A**.**C****C****U****G**A.U.**C****U****G****G**.AUAAU...............................G.**C****C****A****G**C**G**.C**A****G****G**.**G**A...A**G****C****G****U****C****A****G****G****G****U** | |
|  |  | NZ\_AAHP01000041.1/2137-2027  | **U****U****G****C****U****A****A****C****G****C****G**..**G**.**G****G****G**.**U****C****C****U****G****C****A**AUGCGCUCGGUCAAACGAUCGUGCG....................................................................................................................................................C**A****G****C****G****G****G****U**GAG**A**.---------..................................................................................---.--.........AA**U****A****C****C****C****U**UU.........................GA**A**.**C****C****U****G**A.U.**C****U****G****G**.AUAAU...............................G.**C****C****A****G**C**G**.C**A****G****G**.**G**A...A**G****C****G****U****C****A****G****G****G****U** | |
|  |  | NZ\_AAHQ01000006.1/333585-333695  | **U****U****G****C****U****A****A****C****G****C****G**..**G**.**G****G****G**.**U****C****C****U****G****C****A**AUGCGCUCGGUCAAACGAUCGUGCG....................................................................................................................................................C**A****G****C****G****G****G****U**GAG**A**.---------..................................................................................---.--.........AA**U****A****C****C****C****U**UU.........................GA**A**.**C****C****U****G**A.U.**C****U****G****G**.AUAAU...............................G.**C****C****A****G**C**G**.C**A****G****G**.**G**A...A**G****C****G****U****C****A****G****G****G****U** | |
|  |  | NZ\_AAHR01000046.1/47623-47733  | **U****U****G****C****U****A****A****C****G****C****G**..**G**.**G****G****G**.**U****C****C****U****G****C****A**AUGCGCUCGGUCAAACGAUCGUGCG....................................................................................................................................................C**A****G****C****G****G****G****U**GAG**A**.---------..................................................................................---.--.........AA**U****A****C****C****C****U**UU.........................GA**A**.**C****C****U****G**A.U.**C****U****G****G**.AUAAU...............................G.**C****C****A****G**C**G**.C**A****G****G**.**G**A...A**G****C****G****U****C****A****G****G****G****U** | |
|  |  | NZ\_AAHS01000042.1/25865-25755  | **U****U****G****C****U****A****A****C****G****C****G**..**G**.**G****G****G**.**U****C****C****U****G****C****A**AUGCGCUCGGUCAAACGAUCGUGCG....................................................................................................................................................C**A****G****C****G****G****G****U**GAG**A**.---------..................................................................................---.--.........AA**U****A****C****C****C****U**UU.........................GA**A**.**C****C****U****G**A.U.**C****U****G****G**.AUAAU...............................G.**C****C****A****G**C**G**.C**A****G****G**.**G**A...A**G****C****G****U****C****A****G****G****G****U** | |
|  |  | NZ\_AAHT01000001.1/1617092-1616982  | **U****U****G****C****U****A****A****C****G****C****G**..**G**.**G****G****G**.**U****C****C****U****G****C****A**AUGCGCUCGGUCAAACGAUCGUGCG....................................................................................................................................................C**A****G****C****G****G****G****U**GAG**A**.---------..................................................................................---.--.........AA**U****A****C****C****C****U**UU.........................GA**A**.**C****C****U****G**A.U.**C****U****G****G**.AUAAU...............................G.**C****C****A****G**C**G**.C**A****G****G**.**G**A...A**G****C****G****U****C****A****G****G****G****U** | |
|  |  | NZ\_AAHU01000022.1/46157-46267  | **U****U****G****C****U****A****A****C****G****C****G**..**G**.**G****G****G**.**U****C****C****U****G****C****A**AUGCGCUCGGUCAAACGAUCGUGCG....................................................................................................................................................C**A****G****C****G****G****G****U**GAG**A**.---------..................................................................................---.--.........AA**U****A****C****C****C****U**UU.........................GA**A**.**C****C****U****G**A.U.**C****U****G****G**.AUAAU...............................G.**C****C****A****G**C**G**.C**A****G****G**.**G**A...A**G****C****G****U****C****A****G****G****G****U** | |
|  |  | NZ\_AAHV01000021.1/67575-67465  | **U****U****G****C****U****A****A****C****G****C****G**..**G**.**G****G****G**.**U****C****C****U****G****C****A**AUGCGCUCGGUCAAACGAUCGUGCG....................................................................................................................................................C**A****G****C****G****G****G****U**GAG**A**.---------..................................................................................---.--.........AA**U****A****C****C****C****U**UU.........................GA**A**.**C****C****U****G**A.U.**C****U****G****G**.AUAAU...............................G.**C****C****A****G**C**G**.C**A****G****G**.**G**A...A**G****C****G****U****C****A****G****G****G****U** | |
|  |  | NZ\_AAHW01000017.1/67486-67376  | **U****U****G****C****U****A****A****C****G****C****G**..**G**.**G****G****G**.**U****C****C****U****G****C****A**AUGCGCUCGGUCAAACGAUCGUGCG....................................................................................................................................................C**A****G****C****G****G****G****U**GAG**A**.---------..................................................................................---.--.........AA**U****A****C****C****C****U**UU.........................GA**A**.**C****C****U****G**A.U.**C****U****G****G**.AUAAU...............................G.**C****C****A****G**C**G**.C**A****G****G**.**G**A...A**G****C****G****U****C****A****G****G****G****U** | |
|  |  | NZ\_AAIQ01000205.1/870-980  | **U****U****G****C****U****A****A****C****G****C****G**..**G**.**G****G****G**.**U****C****C****U****G****C****A**AUGCGCUCGGUCAAACGAUCGUGCG....................................................................................................................................................C**A****G****C****G****G****G****U**GAG**A**.---------..................................................................................---.--.........AA**U****A****C****C****C****U**UU.........................GA**A**.**C****C****U****G**A.U.**C****U****G****G**.AUAAU...............................G.**C****C****A****G**C**G**.C**A****G****G**.**G**A...A**G****C****G****U****C****A****G****G****G****U** | |
|  |  | NC\_000962.2/501047-501150  | **C****C****C****U****G****U****A****G****A****C****A**..**C**.**G****G****G**.**A****G****U****C****C****C****G**GGAG.........................................................................................................................................................................C**G****G****G****G****U****C****U**GAG**A**G**U****G****G****G****C**GCGC................................................................................CU**G****C****C**.**C****U**...........**U****A****C****C****G****U**C-.........................AC**A**.**C****C****U****G**A.U.**C****C****G****G**.AUCAU...............................G.**C****C****G****G**C**G**.A**A****G****G**.**G**A...G**G****U****C****A****A****G****G****A****U****G** | |
|  |  | NC\_002755.2/502496-502599  | **C****C****C****U****G****U****A****G****A****C****A**..**C**.**G****G****G**.**A****G****U****C****C****C****G**GGAG.........................................................................................................................................................................C**G****G****G****G****U****C****U**GAG**A**G**U****G****G****G****C**GCGC................................................................................CU**G****C****C**.**C****U**...........**U****A****C****C****G****U**C-.........................AC**A**.**C****C****U****G**A.U.**C****C****G****G**.AUCAU...............................G.**C****C****G****G**C**G**.A**A****G****G**.**G**A...G**G****U****C****A****A****G****G****A****U****G** | |
|  |  | NC\_002945.3/502066-502169  | **C****C****C****U****G****U****A****G****A****C****A**..**C**.**G****G****G**.**A****G****U****C****C****C****G**GGAG.........................................................................................................................................................................C**G****G****G****G****U****C****U**GAG**A**G**U****G****G****G****C**GCGC................................................................................CU**G****C****C**.**C****U**...........**U****A****C****C****G****U**C-.........................AC**A**.**C****C****U****G**A.U.**C****C****G****G**.AUCAU...............................G.**C****C****G****G**C**G**.A**A****G****G**.**G**A...G**G****U****C****A****A****G****G****A****U****G** | |
|  |  | NC\_002932.3/1352467-1352566  | **A****A****A****U****C****A****U****C****U****U****G**..**G**.**G****G****G**.**U****G****C****U****U****C****G**CCAUGUUCCAUGAA...............................................................................................................................................................C**C****G****A****A****G****C****U**GAG**A**.---------..................................................................................---.--........UCA**C****A****C****C****C****C**UA.........................UA**A**.**C****U****U****G**A.U.**G****C****A****G**.GUAAU...............................G.**C****U****G****A**C**G**.C**A****A****G**.**G**A....**A****A****G****A****U****G****A****A****G****C** | |
|  |  | NC\_004722.1/404776-404881  | **A****A****G****A****A****A****C****A****C**-**A**..**U**.**G****G****G**.**A****G****U****U****U****G****U**GGA..........................................................................................................................................................................U**A****C****A****A****A****C****U**GAG**A**G**U****A****U****G****G**CUAC...........................................................................UAGUCCG**U****C****A**.**U****U**...........**G****A****C****C****A****U**UU.........................GA**A**.**C****C****U****G**-.U.-**U****G****G**.AUAAU...............................G.**C****C****A****G**C**G**.U**A****G****G**.**G**A...G**A****G****U****G****U****A****A****A****A****G** | |
|  |  | NZ\_AAEO01000033.1/441917-441812  | **A****U****A****G****U****U****U****G****C****U****A**..**G**.**G****A****G**.**A****G****C****U****G****G****U**GU-..........................................................................................................................................................................U**G****C****C****A****G****C****U**GAG**A**G**U****A****A****G****A**CCUU...............................................................................AAG**U****C****U**.**U****U**...........**G****A****U****C****C****U**UUUUA......................UU**A**.**C****C****U****G**A.U.**C****U****A****G**.ACUAU...............................G.**C****U****A****G**C**G**.U**A****G****G**.**G**A....**A****G****C****A****A****U****U****C****G****G** | |
|  |  | NC\_004432.1/850847-850757  | **U****A****U****G****U****U****A****U****C****A****G**..**G**.**C****G****G**.**U****G****C****C****A****U****U**AAA..........................................................................................................................................................................A**U****A****U****G****G****C****U**GAG**A**.---------..................................................................................---.--........AAA**U****A****C****G****C****U**UA.........................UG**A**.**C****C****U****G**A.U.**C****U****A****G**.UUAGU...............................A.**C****U****A****G**C**G**.G**A****G****G**.**C**A..AG**U****G****A****U****A****A****U****A****U****A** | |
|  |  | NC\_002505.1/1374773-1375014  | **G****U****C****C****U****G****U****A****G****U****C**..**G**.**G****G****G**.**A****G****C****C****U****G****A**GAGCGUAUAUACCCUUCUUACUUGAAGCUGCAGCGGUGUUGGCUACGUUCGUUCACCCCAAUCACAUAGUUUAUCUAUGCUCAUGGGGAUGAACUCACUUGUCGCCUACCUGCAACUCCAAGUAGUUUGGGUAUAACAACGUGCGAAU.........................U**A****A****A****G****G****C****U**GAG**A**.**U****C****G****C****G**UA--..................................................................................-**G****C**.**G****A**...........**G****A****C****C****C****G**UU.........................GA**A**.**C****C****U****G**A.U.**U****C****A****G**.UUAGG...............................A.**C****U****G****A**C**G**.U**A****G****G**.**G**A....**A****C****U****A****U****C****C****U****C****A** | |
|  |  | NZ\_AABC04000004.1/17978-18070  | **U****A****A****A****U****A****U****C****C****A****G**..**G**.**G****G****G**.**A****G****C****U****A****U**----..........................................................................................................................................................................-**U****A****U****A****G****C****U**GAG**A**.**G****G****A****U****A**GGGA..................................................................................--**U**.**C**-...........**G****A****C****C****C****C**GG.........................GA**A**.**C****C****U****G**A.U.**C****C****G****G**.CCAGU...............................A.**C****C****G****G**C**G**.G**A****G****G**.**G**A...U**U****G****G****A****U****G****A****A****A****G** | |
|  |  | NZ\_AAAY02000005.1/107734-107823  | **C****A****U****C****C****A****U****G****C****U****A**..**G**.**G****G****G**.**U****G****C****C****U****A****C**AUA..........................................................................................................................................................................A**C****C****A****G****G****C****U**GAG**A**.---------..................................................................................---.--........UCA**C****A****C****C****C****U**UA.........................AC**A**.**C****C****U****G**AGU.**C****U****G****G**.GUAAU...............................A.**C****C****A****G**C**G**.G**A****G****G**.**G**A....**A****G****C****U****G****U****U****U****A****U** | |
|  |  | NZ\_AAII01000108.1/2417-2534  | **C****G****U****C****C****C****A****G****A****U****G**..**C**.**G****G****G**.**A****G****C****U****U****C****C**ACCCU........................................................................................................................................................................G**G****G****A****G****G****C****U**GAG**A**G**G****G****C****G****G**CCGG.....................................................................GCAACUGCCGGUG**C****C****G**.**C****C**...........**G****A****C****C****G****C**CU.........................GA**A**.**C****C****U****G**-.U.**C****C****G****G**.GUAAU...............................U.**C****C****G****G**C**G**.U**A****G****G**.**G**A.GUG**A****U****C****A****A****G****G****U****G****G** | |
[truncated: 288,141 more chars]
